# Pathogenic Effects and Potential Regulatory Mechanisms of Tea Polyphenols on Obesity

**DOI:** 10.1155/2019/2579734

**Published:** 2019-06-11

**Authors:** Zhandong Li, Yue Liu, Weihao Zhao, Xiuhua Wu, Xiujuan Jiang, Lili Yang, Chaoqun Xing, Lixin You, Jiwei Song, Hao Li

**Affiliations:** ^1^College of Food Engineering, Jilin Engineering Normal University, Changchun, China; ^2^Measurement Biotechnique Research Center, Jilin Engineering Normal University, Changchun, China; ^3^Key Laboratory for Molecular Enzymology and Engineering of the Ministry of Education, College of Life Science, Jilin University, Changchun, China; ^4^College of Biology and Food Engineering, Changchun Science Technology University, Changchun, China

## Abstract

Overweight and obesity are major threats to human health. Tea polyphenols exert multiple beneficial effects on human health and may play a positive regulatory role in fat assumption. However, how tea polyphenols contribute to the regulation of fat metabolism remains unclear to date. Small RNA expression profile can be regulated by tea polyphenols in adipocytes. Therefore, tea polyphenols may regulate fat metabolism by controlling small RNA-associated biological processes. In this study, we developed a systematic research platform based on mouse models and performed small RNA sequencing to identify the specific role of small RNAs in the regulatory effect of tea polyphenols on fat metabolism. We compared the expression levels of different small RNA subtypes, including piRNAs and miRNAs, and identified a group of differentially expressed small RNAs in the experimental and control groups. Most of these small RNAs participate in lipid metabolism, suggesting that small RNAs play a significant role in tea polyphenol-associated obesity and related pathogenesis. Furthermore, gene ontology and KEGG pathway enrichment indicated that small RNAs influence the regulatory effects of tea polyphenols on obesity, revealing the potential pathogenic mechanisms for such nutritional disease.

## 1. Introduction

With the improvement of people's living standards in recent years, the excessive intake of high fat foods and poor living habits have led to an increase in the number of overweight and obese people. The major pathogenesis of obesity is the excessive accumulation of adipose tissue, which occurs as the fat cell volume increases and the cell number increases at the single cell level [1]. The imbalance in energy metabolism caused by obesity causes related chronic diseases, such as type II diabetes, hypertension, and cardiovascular and cerebrovascular diseases [2]. Therefore, obesity has become a major threaten to human health. Recent publications have attributed obesity to various endogenous and exogenous factors. Polyphenols from tea reportedly interfere with fat accumulation and are functionally related to the pathogenesis of obesity.

Tea polyphenols, which refer to the phenolic content in tea, provide potential beneficial effects on human health. Tea polyphenols have four major subtypes, namely, catechins, theaflavins, tannins, and flavonoids [3, 4], which are derived from different tea subtypes and have diverse beneficial contributions to human health. Catechins account for the largest proportion of dry tea weight. Recent publications [3, 4] have reported that catechins play potential beneficial regulatory role in cardiovascular health and an effective role in anti-inflammatory actions, reflecting the potential health beneficial role of tea polyphenols [5–7]. Clinical application experience and related phenotypic experiments confirmed that polyphenols interfere with fat accumulation, but the detailed regulatory mechanisms remain unknown to date.

Independent studies have confirmed that polyphenols can interfere with the small RNA spectrum of adipose tissues. Therefore, we selected small RNAs as the research object in the present study to reveal the potential relationships between tea polyphenols and obesity. Small RNAs are noncoding RNA molecules usually with less than 200 nucleotides [8, 9]. Different from messenger RNAs, which usually have encoding potentials, small RNAs mainly participate in regulatory processes at the posttranscription levels [9]. RNA silencing, the generic term for negative regulatory effects on gene expression mostly induced by noncoding RNAs, is a significant biological process involving small RNAs and is the major mechanism of small RNA-mediated posttranscriptional regulation [10]. MiRNAs [11], piRNAs [12], and siRNAs are the major subgroups of the small RNA family and are functionally related to multiple physical and pathological conditions. MiRNAs, generally 22 nucleotides long, are widely distributed regulatory noncoding RNA molecules that function in RNA silencing and posttranscriptional gene expression regulation [11]. piRNAs share similar RNA silencing approaches to miRNAs and are mediated by the RNA-induced silencing complex; they also specifically interact with piwi proteins and mainly participate in the silencing of transposons [12]. In the present study, we also focused on small interfering RNAs (siRNAs), another subtype of small RNAs similar to microRNAs at the structural and functional levels. Different from some microRNAs that are derived from stem loop RNA products and function by translational repression, siRNAs specifically contribute to the cleaving of transcribed but not translated mRNAs. Therefore, small RNAs are functional regulatory molecules for human physical biological processes.

In this study, we applied small RNA sequencing to mouse fat tissue samples and attempted to reveal the expression profiles of different subtypes of small RNAs in each experimental mouse group. Based on the differential expression profile of each group and their respective obesity-associated phenotype, we screened a group of functional biological processes that may reveal the significant and potential pathogenic mechanisms of tea polyphenols for nutritional obesity at the small RNA regulatory level. We identified the specific fat accumulation regulatory mechanisms of polyphenols and confirmed for the first time that microRNAs may play an important role during such pathogenesis.

## 2. Materials and Methods

### 2.1. Mouse Model Grouping and Establishment

Thirty mice (BALB/c) were divided into three randomized groups: high dose (0.4 mL tea polyphenols/10 g), low dose (0.2 mL tea polyphenols/10 g), and blank control (0.1 mL tea polyphenols/10 g). Feeding standards differ according to the growth and development characteristics of different stages of experimental BALB/c mice. Obesity model mice in their first 2 weeks were fed daily with 100 g of base feed, 10 g of animal fat (lard), 10 g of milk powder, one egg, and bean sprouts. The basic feed was replaced in the next 6 weeks. The blank group was fed with the basic feed, and the feed was added according to the principle of a small number of times. The amount of feed added was basically the same as the amount of feed added last time. The mice were fed a full-price nutrient pellet feed, which should contain a certain proportion of crude fiber. Each group of mice was orally administered a daily dose of 0.1 mL. The mice in the low-dose group were intragastrically administered 0.2 mL of the reagent, and the mice in the high-dose group were intragastrically administered 0.4 mL of the reagent per day. During the test, the mice were free to eat and drink, and the breeding environment should be quiet, ventilated, and dry. The temperature and humidity were preferably controlled at 18–22°C and 50%–60%, respectively. The body weight was weighed every 3 days, the food intake of each group of mice was recorded, and the mice were observed for abnormal performance.

### 2.2. Potential Variable Index Monitoring

To evaluate the metabolic contributions of tea polyphenol intake on mouse models and explore the potential contribution of miRNAs to such biological or pathological processes, we performed four physical and genetic inspection processes one by one.

*(1) Evaluation of Body Weight Alteration.* The experimental animals used were placed in an electronic balance for live weighing, and records were made. The increase in body weight of the experimental animals was calculated from the changes in body weight of the mice in the administration and control groups.

In this process, we presented the absolute mouse weight as a variable index and recorded the absolute mouse weight alteration.

*(2) Evaluation of Body Fat.* The experimental animal mice were weighed in vivo and dissected after sacrifice, and their whole body fat was peeled off. The percentage of body fat was then calculated. Measuring only the fat weight around the genitals of the animals (mice) was possible. In this process, we presented a novel parameter, namely, body fat, as the reference index for the evaluation of fat accumulation in each mouse group.

*(3) Evaluation of the Volume and Number of Fat Cells.* At the end of the observation course, the experimental and control animals (white mice) were sacrificed. Each piece of adipose tissue was taken and fixed with formaldehyde calcium, and then frozen sections were prepared and stained with Sudan III dye solution. The stained parts on the sections were adipose tissue. The number of fat cells was counted under a microscope, and the size of fat cells was measured with a micrometer.

### 2.3. Evaluation and Functional Annotation of Differentially Expressed Small RNAs

**Figure 1:**
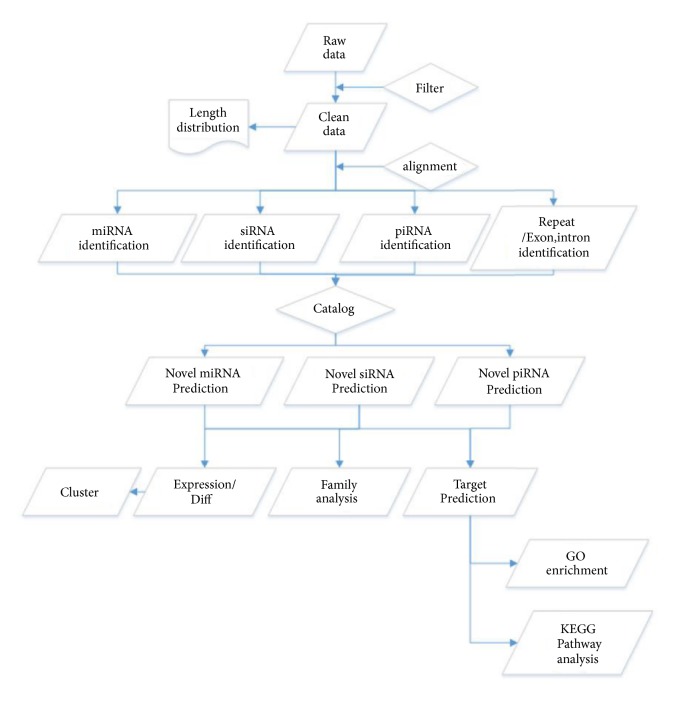
*General workflow for small RNA sequencing.* Following such workflow, we firstly filtered the raw sequencing data for clean data and identified respective length distribution. Based on clean data, we recognized multiple small RNAs subtypes and predicted the possible regulatory target for each small RNA subtype. For further analysis, we summarized the functional annotation of differential expressed small RNAs and annotated their respective biological target by GO and KEGG enrichment.

To identify the functional contribution of small regulatory RNAs to tea polyphenol-associated fat accumulation and adipose cell proliferation, we performed a small RNA sequencing and related bioinformatics analysis on the fat tissue of mice from the experimental and control groups. Using the BGISEQ-500 technology, we focused on three major subtypes of small RNAs, miRNA, piRNA, and siRNA, in three provided samples, XIAO1 (high-dose group), DUI4 (control), and DA3 (low-dose group). The detailed analysis pipeline can be seen in Figure 1.

#### 2.3.1. Screening DESs

Referring to “the significance of digital gene expression profiles,” we developed a strict algorithm to identify differentially expressed genes between two samples. Correction on P-value corresponding to differential gene expression test was performed using the Bonferroni method. Considering that DES analysis generates a large amount of multiplicity problems in which thousands of hypotheses (e.g., is gene x differentially expressed between the two groups?) are tested simultaneously, we performed the correction for false positive (type I) and false negative (type II) errors by using the FDR method. We assumed that we selected R differentially expressed genes in which S generally shows differential expression and the other V genes are false positive. If the error ratio “Q = V / R” must stay below a cutoff (e.g., 5%), we should preset the FDR to a number no larger than 0.05. We used “FDR ≤ 0.001 and the absolute value of log2 ratio ≤ 1” as the default threshold to judge the significance of gene expression difference. More stringent criteria with smaller FDR and larger fold-change value can be used to identify DEGs.

(1) $$\begin{array}{c} P=1-\overset{m-1}{\underset{i=0}{\sum }}\frac{\left(\begin{array}{c} M\\ i \end{array}\right)\left(\begin{array}{c} N-M\\ n-i \end{array}\right)}{\left(\begin{array}{c} N\\ n \end{array}\right)} \end{array}$$

#### 2.3.2. Clustering Analysis

We performed hierarchical clustering for differentially expressed miRNAs using pheatmap, a function of R. For clustering more than two groups, we performed the intersection and union DESs between them.

#### 2.3.3. Functional Enrichment Analysis

Gene ontology (GO) analysis, an international standard gene functional classification system, offers a dynamic-updated controlled vocabulary and a strictly defined concept to comprehensively describe the properties of genes and their products in any organism. GO has three ontologies: molecular function, cellular component, and biological process. The basic unit of GO is GO-term. Every GO-term belongs to a type of ontology. GO enrichment analysis provides all GO terms significantly enriched in a list of genes, compared to a genome background, and filters the genes that correspond to specific biological functions. This method firstly maps all genes to GO terms in the database (http://www.geneontology.org/), calculates gene numbers for every term, and then uses a hypergeometric test to find significantly enriched GO terms in the input gene list. Based on “GO Term Finder” (http://www.yeastgenome.org/goTermFinder), we screened significantly enriched GO terms with corrected p-value ≤ 0.05.

KEGG pathway analysis: Pathway-based analysis helps to further understand DES target gene biological functions. KEGG (the major public pathway-related database) is used to perform pathway enrichment analysis. This analysis identifies significantly enriched metabolic pathways or signal transduction pathways in DES target genes compared with the whole genome background. We also screened out significantly enriched KEGG pathways with corrected p-value ≤ 0.05.

## 3. Results

### 3.1. Results for Body Weight Alteration Evaluation

The experimental animals used were placed on an electronic balance to record live weight. The increase in body weight of the experimental animals was calculated from the changes in body weight of the mice in the administration and control groups. The body weight of the mice is shown in Table 1.

From the body weight changes shown in Tables 1, 2, and 3, we can summarize some characteristics of body weight induced by tea polyphenols of different doses.

(1) The blank group had the highest body weight of 53.5 g. The lowest was 42.8 g, the average was 48.8 g, and the obesity was 9.6%–12.3%. Relatively average weight, the life habits, and diet of mice may be similar.

(2) The low-dose group had the highest body weight of 50.2 g. The lowest was 31.6 g, the average was 43.5 g, and the obesity was 15.4%–27.4%. Among them, mice Nos. 01 and 07 had significantly lower body weights, which may be caused by excessive intragastric administration. The weight of the mice in 08 was significantly higher than that in the other groups. This result may be attributed to the intragastric administration of the reagent to the mice during gavage. The esophagus is shallow, causing the mice to vomit the agent.

(3) The high-dose group had the highest body weight of 47.2 g. The lowest was 23.4g, the average was 35.7 g, and the obesity was 32.2%–34.5%. Among them, mice Nos. 01 and 06 had significantly lower body weights than the other mice, which may be caused by injury to the mice during the intragastric administration or the selection of test animals. The weight of mouse No. 02 is significantly higher than the weight of the others possibly due to the intragastric administration. The mice vomited the reagents, resulting in a small dose remaining in the stomach.

(4) The average body weight of the mice in the blank group was 48.8 g, the average body weight of the mice in the low-dose group was 43.5 g, and the average weight of the mice in the high-dose group was 35.7 g. The average body weight of the mice was slightly lower in the low-dose group than in the blank group; the average body weight of the mice was significantly lower in the high-dose group than in the blank group.

### 3.2. Results for Body Fat Evaluation

We obtained and calculated the absolute and relative fat weight of our experimental mouse models. The experimental animal mice were weighed in vivo and dissected after sacrifice. The whole body fat was peeled off, and the percentage of body fat was calculated. Measuring only the fat weight around the genitals of the animals (mice) was possible. The percentage of body fat was calculated as follows.

(2) $$\begin{array}{c} Body\;\;fat=\frac{Body\;\;fat\;\;total\;\;weight}{living\;\;body\;\;weight}\times 100 \end{array}$$

#### 3.2.1. Results for Absolute Fat Weight

Table 2 shows the specific distribution pattern of absolute fat weight of the experimental and control groups. To avoid the sex-induced differences, we selected the male mice for summarization of characteristics. The characteristics can be summarized as follows:

(1) The net fat weight of the blank group was 1.71 g, the lowest was 0.78 g, and the average value was 1.22 g. Among them, mice Nos. 01 and 02 had low fat net weight, which may be due to incomplete anatomy. The fat weight of mice Nos. 03 and 07 was higher than that of the control group.

(2) The low-dose second group of mice had a net fat weight of 1.60 g, a minimum of 0.47 g, and an average of 1.04 g. The fat weight of mice Nos. 01 and 07 was low. Mouse No. 01 may have incomplete anatomy. Mouse No. 07 had significantly lower fat weight than other mice in such group. The fat weight of mouse No. 05 was obviously high probably due to the physical influence of the small rat.

(3) The net fat weight of the high-dose group was 2.10 g, the lowest was 0.46 g, and the average was 1.19 g. The fat weight of mouse No. 04 was significantly low, which may be due to incomplete anatomy. The fat weight of mice Nos. 05 and 08 was significantly high, and mouse No. 05 may be affected by the body physique of mouse No. 08 because of their high body weight.

(4) The average fat net weights of the blank, low-dose, and high-dose groups were 1.22, 1.04, and 1.19g, respectively. The average fat net weights of the low-dose and high-dose groups were lower than that of the blank group.

#### 3.2.2. Results for Relative Fat Weight

Apart from absolute fat weight, we also calculated and summarized the relative fat weight for each mouse group and obtained some distributed features (Table 3). The characteristics can be summarized as follows:

(1) The body fat of the blank group was 3.64%, the lowest was 1.57%, and the average was 2.50%. Mice Nos. 01, 02, and 04 had low body fat. Due to the high body weight of the mice, the net fat weight was low, resulting in low body fat. Mice Nos. 03, 07, and 08 had high body fat. Mice Nos. 03 and 08 had low body weight and high fat body weight, resulting in high body fat. Mouse No. 07 had relatively high body weight and fat weight.

(2) The low-dose mice had a body fat of 4.48%, a minimum of 1.02%, and an average of 2.39%. The body fat of mouse No. 01 was significantly low. Due to the high body weight of the mice, the fat net weight was significantly low, resulting in low body fat in the mice. The body fat of mouse No. 05 was significantly high because of its low body weight and high fat weight.

(3) The high-dose group had the highest body fat of 4.68%, the lowest was 1.46%, and the average was 3.33%. Mice Nos. 02 and 04 had low body fat. Due to the high body weight of mouse No. 02, the fat weight of mouse No. 04 was significantly low, resulting in low body fat. The body fat of mouse No. 08 was high. The fat weight of mice Nos. 05 and 08 was significantly high, whereas that of mouse No. 06 was significantly low.

(4) The average body fat percentages of the blank, low-dose, and high-dose groups were 2.50%, 2.39%, and 3.33%, respectively. The average body fat of the mice in the low-dose and high-dose groups was slightly lower than that of the mice in the blank group.

### 3.3. Alteration in the Volume and Number of Fat Cells

#### 3.3.1. Microscopic Examination Result of Mouse Fat Cells

**Figure 2:**
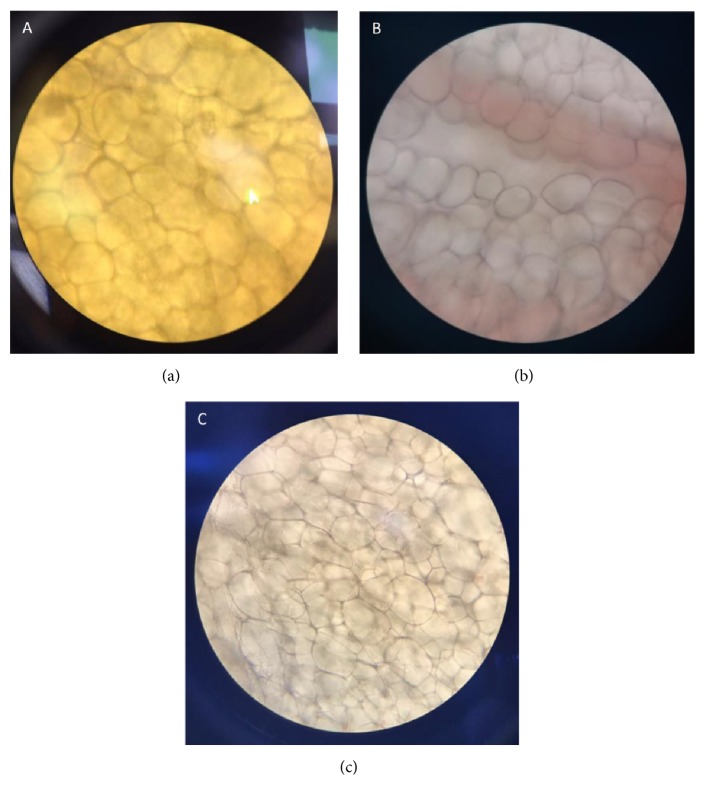
*Microscopic examination result of mouse fat cells.* We isolated the adipose tissue from mice in different groups. The stained parts on the sections were adipose tissue. The number and proportion of fats were counted and calculated according to microscope images measured by a micrometer.

Micrographs of the mouse fat cells from the blank control, low-dose, and high-dose groups are shown in Figure 2.

#### 3.3.2. Comparison of Microscopic Examination Results from Different Experimental Groups

Under the same magnification field of view, the number of fat cells in the blank group was 136. Overall, the volume of fat cells was large. The number of fat cells in the low-dose group was 142, which was lower than that in the blank group. The number of fat cells increased and the volume of fat cells decreased in the low-dose group compared with the blank group. The number of fat cells in the high-dose group was 118, which was lower than that in the blank group. The volume of adipocytes in the blank group increased.

### 3.4. Small RNA Sequencing

In this study, we sequenced our samples using BGISEQ-500 technology. Raw data were filtered, and the dirty tags were removed. The average percentage of clean tags exceeded 70%, reflecting the high sequencing quality of our study. Using the computational approach we mentioned above, we applied novel small RNA prediction, small RNA expression analysis, and small RNA target prediction analysis dependent on our sequencing approach.

#### 3.4.1. Comparison of Different Target Prediction Software

As mentioned in the Materials and Methods, we used various software tools (RNA hybrid and miRanda) to predict the target gene of our annotated miRNAs. The two algorithms shared a large proportion of the predicted targets and had their respective individual prediction targets. RNA hybrid identified 30,097 targets, whereas miRanda identified 29,704 targets. Considering the MFE of potential microRNAs and their respective targets, we filtered the optimal targets by using the optimal computational approach described in the Materials and Methods.

#### 3.4.2. Differentially Expressed Small RNAs

**Figure 3:**
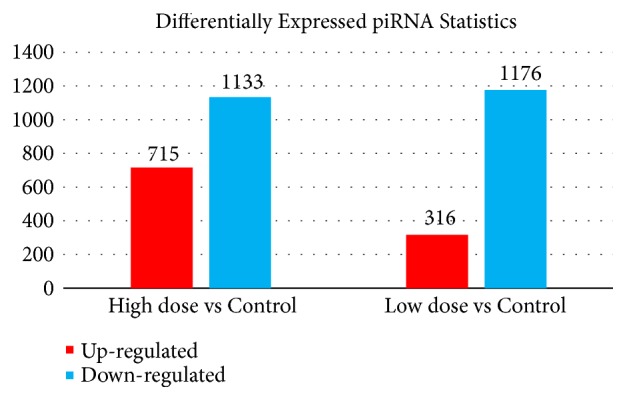
*Differential piRNA expression level in the tea polyphenols treated and control groups*. Compared to the control group, both experimental groups (low dose and high dose) turn out to have more downregulated genes than high-regulated piRNAs. And the higher dose such group applied, the less upregulated piRNAs it may have with similar downregulated piRNA kinds.

**Figure 4:**
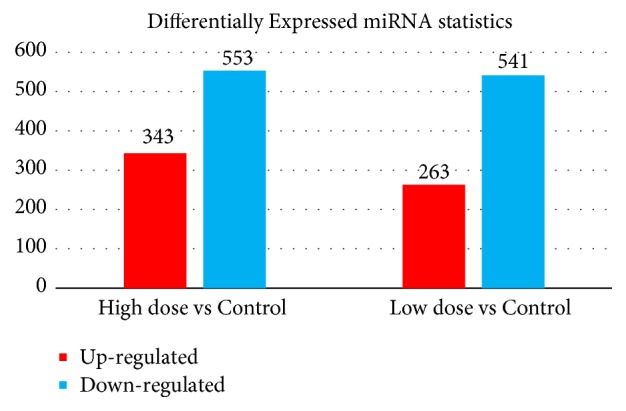
*Differential miRNA expression level in the tea polyphenols treated and control groups.* Compared to the control group, both experimental groups (low dose and high dose) turn out to have more downregulated genes than high-regulated miRNAs. And the higher dose such group applied, the less upregulated miRNAs it may have with similar downregulated miRNA kinds.

As described in the Materials and Methods, we used ExpDiff to identify the differentially expressed small RNAs (piRNAs and miRNAs) in different cells. The detailed annotation of each differentially expressed small RNA is provided in the Supplementary Materials. The differential expression profile between our target cell lines is presented as a hierarchical cluster in Figures 3 and 4.

#### 3.4.3. Differentially Expressed Small RNA Target Prediction

As described in the Materials and Methods, we used a few well-applied software tools to predict the potential targets for each differentially expressed small RNA. The prediction results are provided in the Supplementary Materials.

#### 3.4.4. Functional Enrichment Analysis of Differentially Expressed Small RNAs

**Figure 5:**
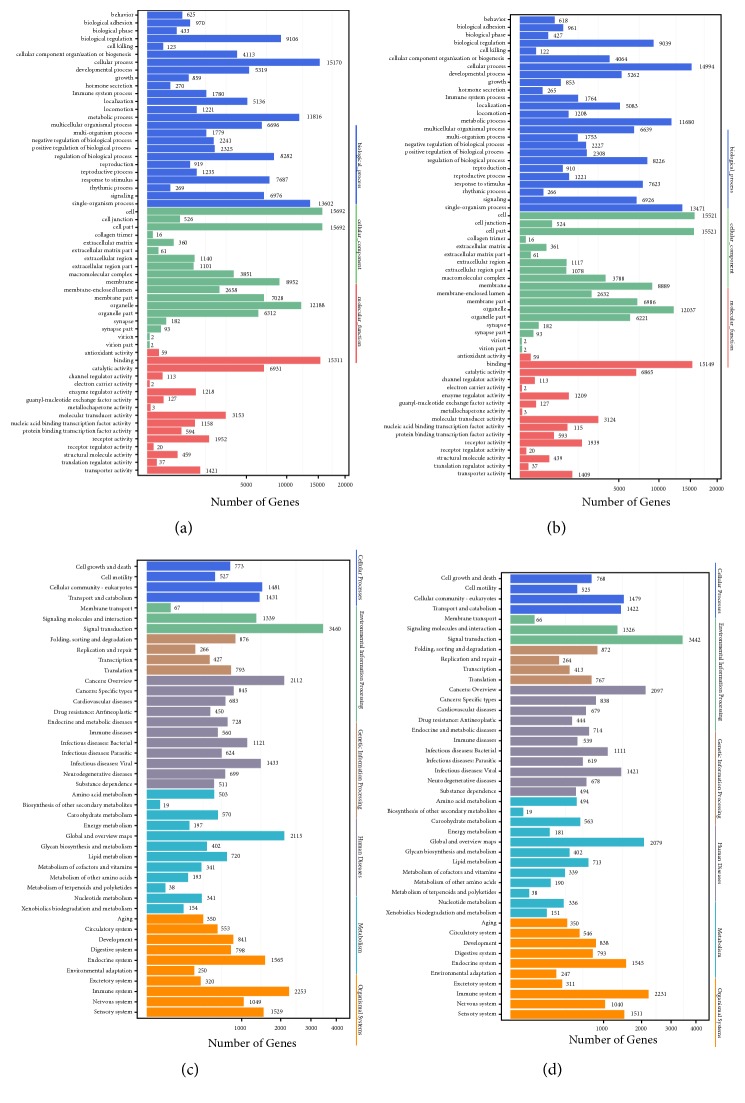
*Functional enrichment for differentially expressed small RNAs.* Based on the target genes of our recognized small RNAs, we summarized the functional enrichment pattern of such genes and annotated the enrichment by GO or KEGG terms: (a) functional enrichment of differentially expressed miRNAs in the control and low-dose group; (b) functional enrichment of differentially expressed miRNAs in the control and high-dose group; (c) functional enrichment of differentially expressed piRNAs in the control and low-dose group; (d) functional enrichment of differentially expressed piRNAs in the control and high-dose group.

We applied gene ontology enrichment and KEGG pathway enrichment analysis by using the specific approach described in the Materials and Methods. The detailed gene ontology functional classification pattern, gene ontology functional enrichment results, KEGG pathway classification pattern, and KEGG enrichment pattern of the microRNAs are illustrated in Figure 5.

## 4. Discussion

In this study, we set up a novel experimental model to study the tea polyphenol-associated pathogenesis of obesity and the potential contribution of small RNAs to such processes. Based on the experimental and computational results mentioned above, we validated the contribution of tea polyphenols to fat metabolism and obesity pathogenesis and revealed the potential small RNA expression and functional enrichment patterns that may be pathologically related to tea polyphenol-associated obesity. The detailed analysis for each result can be found below.

### 4.1. Phenotypic Differences between Different Experimental Groups

We compared the body weight, absolute fat weight, and relative fat weight of mice with different tea polyphenol treatment patterns. Although the body weight of the mice in the high-dose group was averagely lower than those of the mice in the control and low-dose groups, the fat net weight was similar between the high-dose and blank control groups. The low-dose group had a similar relative fat weight to the control group, but the high-dose group had higher fat relative weight, indicating the positive correlation between intake tea polyphenols and relative fat weight. Therefore, at the level of body weight and related fat weight parameters, we can summarize that tea polyphenols may positively contribute to the accumulation of fat. The higher the dose, the more effective the tea polyphenols.

Fat accumulation in nutritional obesity has two major irreplaceable pathogenic processes: escalation of volume and number of fat cells. Microscopic examination of Sudan III-dye mesenteric cells showed that the volume of fat cells gradually increased and the number of fat cells in one field of view increased with increasing dose. Therefore, tea polyphenol-induced obesity may be attributed to the two mechanisms for the accumulation of pathogenic fat. Our research results indicate that tea polyphenols can induce fat accumulation by promoting fat cell proliferation and extending fat cell volumes, leading to nutritional obesity.

### 4.2. Discussion for Genetic (Small RNA) Differences between Different Experimental Groups

We obtained the differentially expressed small RNAs in three experimental samples to reveal the regulatory mechanisms for tea polyphenol-related obesity. As shown in the Results, we analyzed the differentially expressed genes in the different samples. Some of the identified differentially expressed genes and transcripts are functionally related to fat accumulation and polyphenol-associated biological processes, implying the accuracy of our identified differentially expressed genes between groups and their potential irreplaceable role in tea polyphenol-associated obesity.

#### 4.2.1. Differentially Expressed piRNAs

As mentioned in the Results, we obtained a group of piRNAs differentially expressed in the mice of the high-dose and blank groups, implying their potential contributions to tea polyphenol metabolism and related obesity pathogenesis. Limited by the length of the article, we selected two upregulated piRNAs, mmu_piR_000802 and mmu_piR_002435, together with one downregulated piRNA mmu_piR_000691 for detailed discussion. Both mmu_piR_000802 and mmu_piR_002435 can be specifically identified to be expressed in the male germ cells and contribute to the regulation of cell stemness maintenance and cell differentiation [13]. Previous studies on the contribution of tea polyphenols to obesity [14, 15] reported that the inhibition of stem cell differentiation can be functionally related to polyphenol metabolism and may further trigger fat accumulation and adipocyte proliferation. Apart from such two upregulated piRNAs, a specific piRNA named mmu_piR_000691 was also differentially expressed in the adipocytes of the control and high-dose groups. Early identified in germ cells (oocytes not sperms), such piRNA is downregulated in polyphenol-treated adipocytes. Given its specific positive regulatory role in cell differentiation [16], such gene may be identified as a downregulated gene in the treated fat cells. We also identified some differentially expressed genes in the low-dose group compared with the control group. A specific piRNA, mmu_piR_002643, also contributes to the regulation of cell differentiation similarly to mmu_piR_000802 and mmu_piR_002435, which corresponds to our functional hypothesis of tea polyphenols effect on obesity-associated biological and pathological processes.

#### 4.2.2. Differentially Expressed miRNAs

Apart from the piRNAs we analyzed above, we also identified a group of microRNAs that were differentially expressed in the experimental and control groups. We identified multiple differentially expressed microRNAs in the high-dose group compared with the controls. MiR-138, a famous regulator microRNA that contributes to the regulation of cell proliferation and differentiation, was upregulated in the high-dose group, corresponding to previous reports on its ability to promote cell proliferation [17, 18]. In this case, it may promote the proliferation of adipocytes, corresponding with our experiment results. MiR-7115, which is downregulated in highly proliferative and dedifferentiated adipocytes, is a novel identified polyphenol-associated gene that participates in the pathogenesis of obesity. Similarly, the low-dose group also had differential microRNA expression profile compared with the control. Functional microRNAs such as mmu-miR-700-3p and mmu-miR-342-5p contribute to polyphenol-associated obesity [19], reflecting the specific role of tea polyphenols in nutritional obesity.

#### 4.2.3. Target Gene Distribution for Differentially Expressed sRNAs

Based on the effective software mentioned in the Materials and Methods, we also predicted some potential targets for identified small RNAs to reveal the complicated regulatory mechanisms of polyphenol-associated obesity. One of the functional differentially expressed small RNAs named mmu-let-7a-1-3p targets a functional gene named IBA57 (NM_173785). Considering that IBA57 is functionally related to polyphenol- and sunburn-associated biological processes, we identified such gene to be differentially expressed in the experimental and control groups. Therefore, the target of the identified differentially expressed small RNA may also be functionally related to biological processes associated with tea polyphenol-activated obesity [20].

### 4.3. Functional Enrichment on Differentially Expressed sRNAs

Using the target annotation and prediction approach mentioned above, we applied gene ontology enrichment and KEGG pathway enrichment analysis by using the specific approach described in the Materials and Methods. Comparing the expression patterns of the normal control and high-dose groups, we enriched the differentially expressed genes into functional clusters described by gene ontology or KEGG pathways. Response to stimulus and signaling are two major biological processes that differentially expressed genes are enriched in, indicating their differences in such two biological processes. Recent publications have reported that tea polyphenols affect the sensitivity of individual response to stimulus by interfering small RNAs [21], corresponding with such enrichment result.

Similarly, response to stimulus and rhythmic processes were enriched by differentially expressed genes after comparing the low-dose and control groups. Apart from the specific biological processes, response to stimulus, which was analyzed above and showed an internal relationship with polyphenols, and rhythmic process as a typical immune abnormality may be functionally related to polyphenol-associated biological processes, especially obesity. Differential treatments with different doses of polyphenols may lead to distinctive expression pattern with distinctive functional enrichment, which corresponds to our enrichment result and validates the specific role of polyphenol-associated biological processes for obesity.

We established a series of mouse model experiments to explore the potential relationship between polyphenols and obesity together with their specific small RNA regulators. Based on related experiments, we concluded that fat cells become more activated with increasing polyphenol dose and that polyphenols are definitely obesity-associated exogenous regulators. Analysis on gene ontology and KEGG pathway revealed the potential regulatory biological processes for polyphenol-associated obesity. In summary, tea polyphenols functionally contribute to the regulation of obesity-associated processes and microRNAs. These substances may be potential and effective regulators for tea polyphenol-associated obesity, revealing the potential pathogenic mechanisms for such nutritional disease.

## 5. Conclusion

This study investigated the functional role of small RNA subgroups in tea polyphenol-regulated obesity pathogenesis. Various small RNAs and functional enrichment patterns of such identified small RNAs were screened out and experimentally related to tea polyphenol-induced fat metabolism alteration. Depending on mouse model-based controlled experiments, we identified a group of differentially expressed genes that may contribute to the weight alteration and fat metabolism induced by tea polyphenols. We determined the detailed role of small RNAs in the regulatory effects of tea polyphenols on obesity, combined with functional enrichment, revealing the potential pathogenic mechanisms for such nutritional disease.

## Figures and Tables

**Table 1:** Mouse body weight.

mouse ID	dose		
	blank control mouse weight (g)	Low dose (0.2ml) mouse weight (g)	High dose (0.4ml) mouse weight (g)
01	49.7	45.9	26.5
02	53.5	46.1	47.2
03	47.0	44.4	35.3
04	50.6	46.5	31.4
05	48.3	35.7	36.5
06	47.7	46.5	23.4
07	52.4	31.6	40.4
08	42.8	50.2	43.2
09	43.6	45.1	37.4
10	51.9	43.4	
Average	48.8	43.5	35.7

**Table 2:** Mouse fat net weight.

mouse ID	dose		
	Blank control mouse weight (g)	Low dose (0.2ml) mouse weight (g)	High dose (0.4ml) mouse weight (g)
01	0.78	0.47	0.97
02	0.87	0.85	1.17
03	1.71	1.07	1.16
04	0.96	1.42	0.46
05	1.14	1.60	1.71
06	1.18	1.40	0.98
07	1.63	0.56	1.09
08	1.47	1.06	2.10
09	1.01	0.81	1.08
10	1.49	1.16	
Average	1.22	1.04	1.19

**Table 3:** Mouse relative fat weight.

mouse ID	dose		
	Blank control mouse weight (%)	Low dose (0.2ml) mouse weight (%)	High dose (0.4ml) mouse weight (%)
01	1.57	1.02	3.66
02	1.63	1.84	2.48
03	3.64	2.41	3.29
04	1.90	3.05	1.46
05	2.36	4.48	4.68
06	2.47	3.01	4.19
07	3.11	1.77	2.70
08	3.43	2.11	4.86
09	2.32	1.80	2.89
10	2.87	2.67	
Average	2.50	2.39	3.33

## Data Availability

The raw sequencing data and related raw analytic results used to support the findings of this study are available from the corresponding author upon request.

## Supplementary Materials

Supplementary 1 Supplementary Material S1. Differentially expressed microRNAs in the control group vs. low-dose group.

Supplementary 2 Supplementary Material S2. Differentially expressed microRNAs in the control group vs. high-dose group.

Supplementary 3 Supplementary Material S3. Differentially expressed piRNAs in the control group vs. low-dose group.

Supplementary 4 Supplementary Material S4. Differential expressed piRNAs in the control group vs. high-dose group.

Supplementary 5 Supplementary Material S5. Small RNA target prediction result.
