# Supplementary material for: Pathogenic Effects and Potential Regulatory Mechanisms of Tea Polyphenols on Obesity
Source: Biomed Res Int. 2019 Jun 11;2019:2579734. doi: 10.1155/2019/2579734 (PMC6595166; doi:10.1155/2019/2579734)
Supplement: Supplementary 1 — Supplementary Material S1. Differentially expressed microRNAs in the control group vs. low-dose group. [file 2579734.f1.pdf]

miRNA id	Count (DUI4)	Count (DA3)	TPM (DUI4)	TPM (DA3)	log2 Ratio(DA3/DUI4)	Up-Down-Regulation (DA3/DUI4)	P-value	FDR
novel_mir626	0	2987	0.001	148.11	17.17630953	Up	0	0
novel_mir18	0	514	0.001	25.49	14.63764375	Up	2.15E-201	1.30E-200
mmu-miR-138-2-3p	0	455	0.001	22.56	14.46147945	Up	2.27E-178	1.29E-177
novel_mir22	0	414	0.001	20.53	14.32544601	Up	2.26E-162	1.24E-161
mmu-miR-3473e	0	390	0.001	19.34	14.23930017	Up	5.25E-153	2.80E-152
mmu-miR-5114	0	348	0.001	17.26	14.07514484	Up	1.29E-136	6.58E-136
novel_mir750	0	202	0.001	10.02	13.29059489	Up	1.21E-79	3.98E-79
novel_mir426	0	192	0.001	9.52	13.21674586	Up	9.69E-76	2.98E-75
mmu-miR-138-1-3p	0	179	0.001	8.88	13.11634396	Up	1.15E-70	3.31E-70
novel_mir557	0	178	0.001	8.83	13.10819772	Up	2.82E-70	8.09E-70
novel_mir364	0	157	0.001	7.78	12.92555444	Up	4.41E-62	1.14E-61
novel_mir622	0	154	0.001	7.64	12.89935692	Up	6.53E-61	1.67E-60
novel_mir683	0	151	0.001	7.49	12.87075	Up	9.68E-60	2.44E-59
novel_mir248	0	147	0.001	7.29	12.8317031	Up	3.52E-58	8.65E-58
novel_mir133	0	133	0.001	6.59	12.68606275	Up	1.02E-52	2.34E-52
novel_mir529	0	132	0.001	6.55	12.67727919	Up	2.51E-52	5.73E-52
novel_mir54	0	132	0.001	6.55	12.67727919	Up	2.51E-52	5.72E-52
novel_mir632	0	124	0.001	6.15	12.5863707	Up	3.33E-49	7.32E-49
mmu-miR-7065-5p	0	122	0.001	6.05	12.56271943	Up	2.01E-48	4.39E-48
novel_mir543	0	122	0.001	6.05	12.56271943	Up	2.01E-48	4.38E-48
novel_mir742	0	121	0.001	6	12.55074679	Up	4.93E-48	1.06E-47
novel_mir519	0	118	0.001	5.85	12.51422091	Up	7.30E-47	1.56E-46
novel_mir629	0	116	0.001	5.75	12.48934624	Up	4.40E-46	9.35E-46
novel_mir760	0	115	0.001	5.7	12.4767462	Up	1.08E-45	2.28E-45
novel_mir424	0	112	0.001	5.55	12.43827206	Up	1.60E-44	3.34E-44
novel_mir66	0	112	0.001	5.55	12.43827206	Up	1.60E-44	3.34E-44
novel_mir153	0	108	0.001	5.36	12.38801729	Up	5.83E-43	1.20E-42
mmu-miR-700-3p	0	108	0.001	5.36	12.38801729	Up	5.83E-43	1.20E-42
novel_mir496	0	106	0.001	5.26	12.36084708	Up	3.52E-42	7.21E-42
novel_mir381	0	104	0.001	5.16	12.33315535	Up	2.12E-41	4.32E-41
novel_mir643	0	102	0.001	5.06	12.30492167	Up	1.28E-40	2.58E-40
novel_mir185	0	98	0.001	4.86	12.2467406	Up	4.66E-39	9.25E-39
novel_mir336	0	98	0.001	4.86	12.2467406	Up	4.66E-39	9.23E-39

novel_mir720	0	92	0.001	4.56	12.15481811	Up	1.02E-36	2.00E-36
novel_mir292	0	92	0.001	4.56	12.15481811	Up	1.02E-36	1.99E-36
novel_mir548	0	87	0.001	4.31	12.07347215	Up	9.13E-35	1.75E-34
novel_mir587	0	86	0.001	4.26	12.05663772	Up	2.24E-34	4.30E-34
novel_mir653	0	85	0.001	4.21	12.03960452	Up	5.51E-34	1.05E-33
novel_mir635	0	85	0.001	4.21	12.03960452	Up	5.51E-34	1.05E-33
novel_mir673	0	85	0.001	4.21	12.03960452	Up	5.51E-34	1.05E-33
novel_mir392	0	81	0.001	4.02	11.97297979	Up	2.00E-32	3.76E-32
mmu-miR-3086-5p	0	73	0.001	3.62	11.82177398	Up	2.65E-29	4.91E-29
novel_mir347	0	72	0.001	3.57	11.80170836	Up	6.52E-29	1.19E-28
novel_mir676	0	72	0.001	3.57	11.80170836	Up	6.52E-29	1.19E-28
novel_mir269	0	72	0.001	3.57	11.80170836	Up	6.52E-29	1.19E-28
novel_mir397	0	69	0.001	3.42	11.73978061	Up	9.66E-28	1.75E-27
mmu-miR-141-5p	0	69	0.001	3.42	11.73978061	Up	9.66E-28	1.74E-27
novel_mir211	0	68	0.001	3.37	11.71853288	Up	2.37E-27	4.26E-27
novel_mir461	0	67	0.001	3.32	11.69696753	Up	5.82E-27	1.04E-26
novel_mir112	0	64	0.001	3.17	11.63026713	Up	8.63E-26	1.53E-25
novel_mir525	0	64	0.001	3.17	11.63026713	Up	8.63E-26	1.53E-25
novel_mir152	0	64	0.001	3.17	11.63026713	Up	8.63E-26	1.53E-25
mmu-miR-8114	0	62	0.001	3.07	11.58402294	Up	5.21E-25	9.17E-25
novel_mir442	0	61	0.001	3.02	11.56033283	Up	1.28E-24	2.24E-24
novel_mir119	0	61	0.001	3.02	11.56033283	Up	1.28E-24	2.24E-24
novel_mir146	0	60	0.001	2.98	11.54109662	Up	3.14E-24	5.49E-24
novel_mir449	0	59	0.001	2.93	11.51668495	Up	7.71E-24	1.34E-23
novel_mir706	0	59	0.001	2.93	11.51668495	Up	7.71E-24	1.34E-23
mmu-miR-6912-5p	0	59	0.001	2.93	11.51668495	Up	7.71E-24	1.34E-23
novel_mir711	0	59	0.001	2.93	11.51668495	Up	7.71E-24	1.33E-23
novel_mir404	0	58	0.001	2.88	11.4918531	Up	1.89E-23	3.27E-23
novel_mir286	0	57	0.001	2.83	11.46658634	Up	4.65E-23	7.98E-23
novel_mir301	0	57	0.001	2.83	11.46658634	Up	4.65E-23	7.97E-23
mmu-miR-7024-5p	0	56	0.001	2.78	11.44086917	Up	1.14E-22	1.95E-22
mmu-miR-5119	0	56	0.001	2.78	11.44086917	Up	1.14E-22	1.95E-22
novel_mir470	0	54	0.001	2.68	11.38801729	Up	6.89E-22	1.17E-21
novel_mir764	0	54	0.001	2.68	11.38801729	Up	6.89E-22	1.17E-21
novel_mir120	0	52	0.001	2.58	11.33315535	Up	4.16E-21	7.01E-21
novel_mir500	0	51	0.001	2.53	11.30492167	Up	1.02E-20	1.71E-20

novel_mir652	0	50	0.001	2.48	11.27612441	Up	2.51E-20	4.19E-20
novel_mir232	0	50	0.001	2.48	11.27612441	Up	2.51E-20	4.18E-20
mmu-miR-673-5p	0	50	0.001	2.48	11.27612441	Up	2.51E-20	4.18E-20
novel_mir691	0	50	0.001	2.48	11.27612441	Up	2.51E-20	4.17E-20
novel_mir719	0	49	0.001	2.43	11.2467406	Up	6.16E-20	1.02E-19
novel_mir491	0	49	0.001	2.43	11.2467406	Up	6.16E-20	1.02E-19
novel_mir624	0	49	0.001	2.43	11.2467406	Up	6.16E-20	1.02E-19
novel_mir250	0	48	0.001	2.38	11.21674586	Up	1.51E-19	2.49E-19
novel_mir95	0	47	0.001	2.33	11.18611424	Up	3.71E-19	6.09E-19
novel_mir36	0	47	0.001	2.33	11.18611424	Up	3.71E-19	6.08E-19
novel_mir305	0	46	0.001	2.28	11.15481811	Up	9.12E-19	1.49E-18
novel_mir521	0	46	0.001	2.28	11.15481811	Up	9.12E-19	1.49E-18
novel_mir327	0	45	0.001	2.23	11.12282799	Up	2.24E-18	3.64E-18
novel_mir473	0	44	0.001	2.18	11.09011242	Up	5.50E-18	8.92E-18
novel_mir334	0	44	0.001	2.18	11.09011242	Up	5.50E-18	8.91E-18
novel_mir163	0	43	0.001	2.13	11.05663772	Up	1.35E-17	2.18E-17
novel_mir335	0	43	0.001	2.13	11.05663772	Up	1.35E-17	2.17E-17
novel_mir254	0	42	0.001	2.08	11.02236781	Up	3.32E-17	5.33E-17
novel_mir373	0	41	0.001	2.03	10.98726401	Up	8.15E-17	1.30E-16
novel_mir567	0	41	0.001	2.03	10.98726401	Up	8.15E-17	1.30E-16
novel_mir478	0	40	0.001	1.98	10.95128471	Up	2.00E-16	3.18E-16
novel_mir179	0	40	0.001	1.98	10.95128471	Up	2.00E-16	3.17E-16
novel_mir729	0	40	0.001	1.98	10.95128471	Up	2.00E-16	3.17E-16
novel_mir47	0	39	0.001	1.93	10.91438513	Up	4.92E-16	7.76E-16
novel_mir252	0	39	0.001	1.93	10.91438513	Up	4.92E-16	7.75E-16
mmu-miR-5103	0	38	0.001	1.88	10.87651695	Up	1.21E-15	1.90E-15
novel_mir440	0	38	0.001	1.88	10.87651695	Up	1.21E-15	1.90E-15
mmu-miR-6961-5p	0	36	0.001	1.79	10.80574387	Up	7.29E-15	1.14E-14
novel_mir744	0	36	0.001	1.79	10.80574387	Up	7.29E-15	1.14E-14
novel_mir479	0	36	0.001	1.79	10.80574387	Up	7.29E-15	1.14E-14
novel_mir733	0	35	0.001	1.74	10.76487159	Up	1.79E-14	2.78E-14
novel_mir79	0	33	0.001	1.64	10.6794801	Up	1.08E-13	1.67E-13
novel_mir180	0	33	0.001	1.64	10.6794801	Up	1.08E-13	1.67E-13
novel_mir688	0	32	0.001	1.59	10.63481105	Up	2.65E-13	4.09E-13
novel_mir513	0	31	0.001	1.54	10.58871464	Up	6.51E-13	1.00E-12
novel_mir25	0	31	0.001	1.54	10.58871464	Up	6.51E-13	9.98E-13

novel_mir51	0	31	0.001	1.54	10.58871464	Up	6.51E-13	9.97E-13
mmu-miR-6979-3p	0	31	0.001	1.54	10.58871464	Up	6.51E-13	9.96E-13
novel_mir644	0	31	0.001	1.54	10.58871464	Up	6.51E-13	9.94E-13
mmu-miR-1930-5p	0	31	0.001	1.54	10.58871464	Up	6.51E-13	9.93E-13
novel_mir684	0	30	0.001	1.49	10.54109662	Up	1.60E-12	2.43E-12
novel_mir466	0	30	0.001	1.49	10.54109662	Up	1.60E-12	2.43E-12
novel_mir388	0	30	0.001	1.49	10.54109662	Up	1.60E-12	2.42E-12
novel_mir723	0	29	0.001	1.44	10.4918531	Up	3.93E-12	5.93E-12
mmu-miR-3076-5p	0	29	0.001	1.44	10.4918531	Up	3.93E-12	5.92E-12
novel_mir155	0	29	0.001	1.44	10.4918531	Up	3.93E-12	5.91E-12
novel_mir135	0	28	0.001	1.39	10.44086917	Up	9.65E-12	1.45E-11
novel_mir716	0	28	0.001	1.39	10.44086917	Up	9.65E-12	1.45E-11
novel_mir531	0	27	0.001	1.34	10.38801729	Up	2.37E-11	3.54E-11
novel_mir765	0	27	0.001	1.34	10.38801729	Up	2.37E-11	3.54E-11
novel_mir666	0	27	0.001	1.34	10.38801729	Up	2.37E-11	3.53E-11
novel_mir753	0	27	0.001	1.34	10.38801729	Up	2.37E-11	3.53E-11
mmu-miR-125b-2-3p	0	27	0.001	1.34	10.38801729	Up	2.37E-11	3.52E-11
novel_mir12	0	27	0.001	1.34	10.38801729	Up	2.37E-11	3.52E-11
mmu-miR-136-5p	0	27	0.001	1.34	10.38801729	Up	2.37E-11	3.51E-11
novel_mir416	0	26	0.001	1.29	10.33315535	Up	5.82E-11	8.62E-11
mmu-miR-1199-5p	0	26	0.001	1.29	10.33315535	Up	5.82E-11	8.60E-11
novel_mir171	0	25	0.001	1.24	10.27612441	Up	1.43E-10	2.11E-10
mmu-miR-5107-5p	0	25	0.001	1.24	10.27612441	Up	1.43E-10	2.10E-10
novel_mir195	0	25	0.001	1.24	10.27612441	Up	1.43E-10	2.10E-10
novel_mir10	0	25	0.001	1.24	10.27612441	Up	1.43E-10	2.10E-10
novel_mir175	0	25	0.001	1.24	10.27612441	Up	1.43E-10	2.10E-10
novel_mir24	0	25	0.001	1.24	10.27612441	Up	1.43E-10	2.09E-10
novel_mir5	0	25	0.001	1.24	10.27612441	Up	1.43E-10	2.09E-10
mmu-miR-496a-3p	0	25	0.001	1.24	10.27612441	Up	1.43E-10	2.09E-10
mmu-miR-7015-3p	0	24	0.001	1.19	10.21674586	Up	3.51E-10	5.11E-10
mmu-miR-7058-5p	0	24	0.001	1.19	10.21674586	Up	3.51E-10	5.10E-10
mmu-miR-669e-5p	0	23	0.001	1.14	10.15481811	Up	8.62E-10	1.25E-09
mmu-miR-129-5p	0	23	0.001	1.14	10.15481811	Up	8.62E-10	1.25E-09
mmu-miR-671-5p	0	23	0.001	1.14	10.15481811	Up	8.62E-10	1.25E-09
mmu-miR-5120	0	23	0.001	1.14	10.15481811	Up	8.62E-10	1.24E-09
novel_mir83	0	23	0.001	1.14	10.15481811	Up	8.62E-10	1.24E-09

novel_mir707	0	23	0.001	1.14	10.15481811	Up	8.62E-10	1.24E-09
novel_mir34	0	23	0.001	1.14	10.15481811	Up	8.62E-10	1.24E-09
novel_mir410	0	23	0.001	1.14	10.15481811	Up	8.62E-10	1.24E-09
novel_mir417	0	22	0.001	1.09	10.09011242	Up	2.12E-09	3.03E-09
novel_mir297	0	22	0.001	1.09	10.09011242	Up	2.12E-09	3.03E-09
novel_mir566	0	22	0.001	1.09	10.09011242	Up	2.12E-09	3.02E-09
novel_mir393	0	22	0.001	1.09	10.09011242	Up	2.12E-09	3.02E-09
novel_mir574	0	22	0.001	1.09	10.09011242	Up	2.12E-09	3.01E-09
mmu-miR-672-5p	0	22	0.001	1.09	10.09011242	Up	2.12E-09	3.01E-09
mmu-miR-3091-3p	0	21	0.001	1.04	10.02236781	Up	5.20E-09	7.36E-09
novel_mir391	0	21	0.001	1.04	10.02236781	Up	5.20E-09	7.35E-09
novel_mir321	0	20	0.001	0.99	9.951284715	Up	1.28E-08	1.80E-08
novel_mir372	0	20	0.001	0.99	9.951284715	Up	1.28E-08	1.80E-08
novel_mir612	0	20	0.001	0.99	9.951284715	Up	1.28E-08	1.79E-08
mmu-miR-879-3p	0	19	0.001	0.94	9.876516947	Up	3.14E-08	4.38E-08
mmu-miR-490-5p	0	19	0.001	0.94	9.876516947	Up	3.14E-08	4.38E-08
novel_mir746	0	19	0.001	0.94	9.876516947	Up	3.14E-08	4.37E-08
novel_mir598	0	19	0.001	0.94	9.876516947	Up	3.14E-08	4.37E-08
mmu-miR-664-5p	0	19	0.001	0.94	9.876516947	Up	3.14E-08	4.36E-08
novel_mir294	0	19	0.001	0.94	9.876516947	Up	3.14E-08	4.36E-08
mmu-miR-6904-5p	0	19	0.001	0.94	9.876516947	Up	3.14E-08	4.35E-08
mmu-miR-185-3p	0	19	0.001	0.94	9.876516947	Up	3.14E-08	4.35E-08
mmu-miR-202-3p	1	545	0.03	27.02	9.814849459	Up	5.57E-211	3.45E-210
novel_mir31	0	18	0.001	0.89	9.797661526	Up	7.70E-08	1.06E-07
mmu-miR-6985-3p	0	18	0.001	0.89	9.797661526	Up	7.70E-08	1.06E-07
mmu-miR-377-3p	0	18	0.001	0.89	9.797661526	Up	7.70E-08	1.06E-07
mmu-miR-3572-3p	0	18	0.001	0.89	9.797661526	Up	7.70E-08	1.06E-07
novel_mir583	0	18	0.001	0.89	9.797661526	Up	7.70E-08	1.06E-07
novel_mir406	0	18	0.001	0.89	9.797661526	Up	7.70E-08	1.06E-07
novel_mir770	0	18	0.001	0.89	9.797661526	Up	7.70E-08	1.06E-07
mmu-miR-500-3p	1	524	0.03	25.98	9.758223215	Up	8.39E-203	5.11E-202
mmu-miR-672-3p	0	17	0.001	0.84	9.714245518	Up	1.89E-07	2.59E-07
mmu-miR-3058-5p	0	17	0.001	0.84	9.714245518	Up	1.89E-07	2.58E-07
novel_mir288	0	17	0.001	0.84	9.714245518	Up	1.89E-07	2.58E-07
novel_mir665	0	17	0.001	0.84	9.714245518	Up	1.89E-07	2.58E-07
novel_mir561	0	17	0.001	0.84	9.714245518	Up	1.89E-07	2.57E-07

mmu-miR-195a-3p	0	17	0.001	0.84	9.714245518	Up	1.89E-07	2.57E-07
novel_mir484	0	17	0.001	0.84	9.714245518	Up	1.89E-07	2.57E-07
novel_mir628	0	16	0.001	0.79	9.625708843	Up	4.65E-07	6.28E-07
novel_mir115	0	16	0.001	0.79	9.625708843	Up	4.65E-07	6.27E-07
novel_mir604	0	16	0.001	0.79	9.625708843	Up	4.65E-07	6.26E-07
novel_mir534	0	16	0.001	0.79	9.625708843	Up	4.65E-07	6.25E-07
mmu-miR-3079-3p	0	16	0.001	0.79	9.625708843	Up	4.65E-07	6.25E-07
novel_mir428	0	16	0.001	0.79	9.625708843	Up	4.65E-07	6.24E-07
novel_mir317	0	16	0.001	0.79	9.625708843	Up	4.65E-07	6.23E-07
novel_mir739	0	16	0.001	0.79	9.625708843	Up	4.65E-07	6.22E-07
novel_mir399	0	16	0.001	0.79	9.625708843	Up	4.65E-07	6.21E-07
novel_mir278	0	16	0.001	0.79	9.625708843	Up	4.65E-07	6.21E-07
novel_mir608	0	16	0.001	0.79	9.625708843	Up	4.65E-07	6.20E-07
novel_mir692	0	16	0.001	0.79	9.625708843	Up	4.65E-07	6.19E-07
novel_mir29	0	16	0.001	0.79	9.625708843	Up	4.65E-07	6.18E-07
novel_mir138	0	16	0.001	0.79	9.625708843	Up	4.65E-07	6.18E-07
novel_mir759	0	16	0.001	0.79	9.625708843	Up	4.65E-07	6.17E-07
novel_mir563	0	16	0.001	0.79	9.625708843	Up	4.65E-07	6.16E-07
mmu-miR-505-3p	0	15	0.001	0.74	9.531381461	Up	1.14E-06	1.51E-06
novel_mir730	0	15	0.001	0.74	9.531381461	Up	1.14E-06	1.51E-06
novel_mir728	0	15	0.001	0.74	9.531381461	Up	1.14E-06	1.51E-06
mmu-miR-6983-5p	0	15	0.001	0.74	9.531381461	Up	1.14E-06	1.50E-06
novel_mir623	0	15	0.001	0.74	9.531381461	Up	1.14E-06	1.50E-06
novel_mir489	0	15	0.001	0.74	9.531381461	Up	1.14E-06	1.50E-06
novel_mir414	0	15	0.001	0.74	9.531381461	Up	1.14E-06	1.50E-06
mmu-miR-744-3p	0	15	0.001	0.74	9.531381461	Up	1.14E-06	1.50E-06
novel_mir433	0	15	0.001	0.74	9.531381461	Up	1.14E-06	1.49E-06
novel_mir565	0	15	0.001	0.74	9.531381461	Up	1.14E-06	1.49E-06
mmu-miR-671-3p	0	15	0.001	0.74	9.531381461	Up	1.14E-06	1.49E-06
mmu-miR-188-5p	1	431	0.03	21.37	9.476408692	Up	1.35E-166	7.55E-166
mmu-miR-7051-5p	0	14	0.001	0.69	9.430452552	Up	2.80E-06	3.64E-06
novel_mir4	0	14	0.001	0.69	9.430452552	Up	2.80E-06	3.64E-06
novel_mir264	0	14	0.001	0.69	9.430452552	Up	2.80E-06	3.64E-06
novel_mir577	0	14	0.001	0.69	9.430452552	Up	2.80E-06	3.63E-06
novel_mir409	0	14	0.001	0.69	9.430452552	Up	2.80E-06	3.63E-06
mmu-miR-6928-3p	0	14	0.001	0.69	9.430452552	Up	2.80E-06	3.62E-06

novel_mir299	0	14	0.001	0.69	9.430452552	Up	2.80E-06	3.62E-06
novel_mir356	0	14	0.001	0.69	9.430452552	Up	2.80E-06	3.61E-06
novel_mir132	0	14	0.001	0.69	9.430452552	Up	2.80E-06	3.61E-06
novel_mir429	0	14	0.001	0.69	9.430452552	Up	2.80E-06	3.61E-06
novel_mir655	0	13	0.001	0.64	9.321928095	Up	6.88E-06	8.81E-06
mmu-miR-6935-3p	0	13	0.001	0.64	9.321928095	Up	6.88E-06	8.80E-06
novel_mir475	0	13	0.001	0.64	9.321928095	Up	6.88E-06	8.79E-06
novel_mir487	0	13	0.001	0.64	9.321928095	Up	6.88E-06	8.78E-06
novel_mir390	0	13	0.001	0.64	9.321928095	Up	6.88E-06	8.77E-06
novel_mir43	0	13	0.001	0.64	9.321928095	Up	6.88E-06	8.76E-06
novel_mir6	0	13	0.001	0.64	9.321928095	Up	6.88E-06	8.75E-06
novel_mir762	0	13	0.001	0.64	9.321928095	Up	6.88E-06	8.74E-06
mmu-miR-674-3p	0	13	0.001	0.64	9.321928095	Up	6.88E-06	8.73E-06
novel_mir215	0	13	0.001	0.64	9.321928095	Up	6.88E-06	8.72E-06
mmu-miR-3082-3p	0	13	0.001	0.64	9.321928095	Up	6.88E-06	8.71E-06
novel_mir351	0	13	0.001	0.64	9.321928095	Up	6.88E-06	8.70E-06
novel_mir724	0	13	0.001	0.64	9.321928095	Up	6.88E-06	8.69E-06
novel_mir735	0	13	0.001	0.64	9.321928095	Up	6.88E-06	8.68E-06
mmu-miR-7052-3p	0	12	0.001	0.6	9.22881869	Up	1.69E-05	2.12E-05
novel_mir76	0	12	0.001	0.6	9.22881869	Up	1.69E-05	2.12E-05
novel_mir494	0	12	0.001	0.6	9.22881869	Up	1.69E-05	2.12E-05
novel_mir569	0	12	0.001	0.6	9.22881869	Up	1.69E-05	2.12E-05
novel_mir549	0	12	0.001	0.6	9.22881869	Up	1.69E-05	2.11E-05
mmu-miR-7091-3p	0	12	0.001	0.6	9.22881869	Up	1.69E-05	2.11E-05
mmu-miR-1943-3p	0	12	0.001	0.6	9.22881869	Up	1.69E-05	2.11E-05
novel_mir467	0	12	0.001	0.6	9.22881869	Up	1.69E-05	2.11E-05
novel_mir105	0	12	0.001	0.6	9.22881869	Up	1.69E-05	2.10E-05
novel_mir726	0	12	0.001	0.6	9.22881869	Up	1.69E-05	2.10E-05
novel_mir654	0	12	0.001	0.6	9.22881869	Up	1.69E-05	2.10E-05
mmu-miR-1938	0	12	0.001	0.6	9.22881869	Up	1.69E-05	2.10E-05
novel_mir128	0	12	0.001	0.6	9.22881869	Up	1.69E-05	2.09E-05
novel_mir144	0	11	0.001	0.55	9.103287808	Up	4.15E-05	5.13E-05
novel_mir108	0	11	0.001	0.55	9.103287808	Up	4.15E-05	5.12E-05
novel_mir33	0	11	0.001	0.55	9.103287808	Up	4.15E-05	5.12E-05
novel_mir758	0	11	0.001	0.55	9.103287808	Up	4.15E-05	5.11E-05
novel_mir734	0	11	0.001	0.55	9.103287808	Up	4.15E-05	5.10E-05

novel_mir116	0	11	0.001	0.55	9.103287808	Up	4.15E-05	5.10E-05
novel_mir253	0	11	0.001	0.55	9.103287808	Up	4.15E-05	5.09E-05
mmu-let-7g-3p	0	11	0.001	0.55	9.103287808	Up	4.15E-05	5.09E-05
novel_mir184	0	11	0.001	0.55	9.103287808	Up	4.15E-05	5.08E-05
novel_mir100	0	11	0.001	0.55	9.103287808	Up	4.15E-05	5.07E-05
novel_mir272	0	11	0.001	0.55	9.103287808	Up	4.15E-05	5.07E-05
novel_mir68	0	11	0.001	0.55	9.103287808	Up	4.15E-05	5.06E-05
novel_mir718	0	11	0.001	0.55	9.103287808	Up	4.15E-05	5.06E-05
novel_mir455	0	10	0.001	0.5	8.965784285	Up	0.000101972	0.000123791
mmu-miR-6954-3p	0	10	0.001	0.5	8.965784285	Up	0.000101972	0.000123652
mmu-miR-5620-5p	0	10	0.001	0.5	8.965784285	Up	0.000101972	0.000123513
novel_mir668	0	10	0.001	0.5	8.965784285	Up	0.000101972	0.000123374
novel_mir547	0	10	0.001	0.5	8.965784285	Up	0.000101972	0.000123236
novel_mir285	0	10	0.001	0.5	8.965784285	Up	0.000101972	0.000123098
novel_mir512	0	10	0.001	0.5	8.965784285	Up	0.000101972	0.00012296
mmu-miR-344-3p	0	10	0.001	0.5	8.965784285	Up	0.000101972	0.000122823
mmu-miR-3154	0	10	0.001	0.5	8.965784285	Up	0.000101972	0.000122686
mmu-miR-18b-5p	0	9	0.001	0.45	8.813781191	Up	0.000250446	0.000299979
mmu-miR-7679-5p	0	9	0.001	0.45	8.813781191	Up	0.000250446	0.000299646
mmu-miR-666-5p	0	9	0.001	0.45	8.813781191	Up	0.000250446	0.000299314
mmu-miR-6967-5p	0	9	0.001	0.45	8.813781191	Up	0.000250446	0.000298982
mmu-miR-8099	0	9	0.001	0.45	8.813781191	Up	0.000250446	0.000298651
mmu-miR-1839-3p	0	9	0.001	0.45	8.813781191	Up	0.000250446	0.000298321
mmu-miR-351-3p	0	9	0.001	0.45	8.813781191	Up	0.000250446	0.000297992
mmu-miR-665-3p	0	9	0.001	0.45	8.813781191	Up	0.000250446	0.000297663
mmu-miR-181c-3p	0	9	0.001	0.45	8.813781191	Up	0.000250446	0.000297336
mmu-miR-6240	0	9	0.001	0.45	8.813781191	Up	0.000250446	0.000297009
mmu-miR-3569-5p	0	9	0.001	0.45	8.813781191	Up	0.000250446	0.000296682
mmu-miR-129b-3p	0	9	0.001	0.45	8.813781191	Up	0.000250446	0.000296357
mmu-miR-376b-5p	0	9	0.001	0.45	8.813781191	Up	0.000250446	0.000296032
mmu-miR-5627-5p	0	9	0.001	0.45	8.813781191	Up	0.000250446	0.000295707
mmu-miR-6911-3p	0	9	0.001	0.45	8.813781191	Up	0.000250446	0.000295384
mmu-miR-362-3p	0	9	0.001	0.45	8.813781191	Up	0.000250446	0.000295061
mmu-miR-184-5p	0	8	0.001	0.4	8.64385619	Up	0.0006151	0.000723095
mmu-miR-344d-3p	0	8	0.001	0.4	8.64385619	Up	0.0006151	0.000722307
mmu-miR-7053-5p	0	8	0.001	0.4	8.64385619	Up	0.0006151	0.000721521

mmu-miR-8103	0	8	0.001	0.4	8.64385619	Up	0.0006151	0.000720737
mmu-miR-8098	0	8	0.001	0.4	8.64385619	Up	0.0006151	0.000719954
mmu-miR-3072-3p	0	8	0.001	0.4	8.64385619	Up	0.0006151	0.000719173
mmu-miR-6913-5p	0	8	0.001	0.4	8.64385619	Up	0.0006151	0.000718394
mmu-miR-6976-5p	0	8	0.001	0.4	8.64385619	Up	0.0006151	0.000717617
mmu-miR-324-3p	2	547	0.07	27.12	8.597786541	Up	1.51E-209	9.27E-209
mmu-miR-200b-5p	1	202	0.03	10.02	8.383704292	Up	1.47E-77	4.68E-77
mmu-miR-193a-5p	3	410	0.1	20.33	7.667466405	Up	2.03E-154	1.09E-153
mmu-miR-200a-5p	1	79	0.03	3.92	7.029747343	Up	5.86E-30	1.09E-29
mmu-miR-541-5p	1	73	0.03	3.62	6.914883386	Up	1.19E-27	2.14E-27
mmu-miR-2137	2	163	0.07	8.08	6.850856561	Up	9.75E-61	2.49E-60
mmu-miR-378d	2	162	0.07	8.03	6.841901255	Up	2.37E-60	6.00E-60
mmu-miR-25-5p	1	58	0.03	2.88	6.584962501	Up	6.81E-22	1.16E-21
mmu-miR-1943-5p	1	53	0.03	2.63	6.453956489	Up	5.59E-20	9.28E-20
mmu-miR-700-5p	2	89	0.07	4.41	5.977279923	Up	2.26E-32	4.24E-32
mmu-miR-6691-5p	1	35	0.03	1.74	5.857980995	Up	4.00E-13	6.15E-13
mmu-miR-3474	1	29	0.03	1.44	5.584962501	Up	7.38E-11	1.09E-10
mmu-miR-8096	1	27	0.03	1.34	5.48112669	Up	4.17E-10	6.05E-10
mmu-miR-20b-5p	2	62	0.07	3.07	5.454739923	Up	3.89E-22	6.62E-22
mmu-miR-3099-3p	1	26	0.03	1.29	5.426264755	Up	9.89E-10	1.42E-09
mmu-miR-99b-3p	9	252	0.31	12.5	5.333516069	Up	4.83E-85	1.67E-84
mmu-miR-33-5p	3	81	0.1	4.02	5.329123596	Up	4.23E-28	7.66E-28
mmu-miR-874-3p	2	56	0.07	2.78	5.311586151	Up	7.03E-20	1.16E-19
mmu-miR-380-3p	1	22	0.03	1.09	5.183221824	Up	3.10E-08	4.34E-08
mmu-miR-181a-1-3p	1	19	0.03	0.94	4.969626351	Up	4.03E-07	5.46E-07
mmu-miR-1947-5p	2	44	0.07	2.18	4.960829403	Up	2.15E-15	3.38E-15
mmu-miR-1188-5p	13	259	0.44	12.84	4.866997868	Up	4.51E-83	1.53E-82
mmu-miR-1945	1	16	0.03	0.79	4.718818247	Up	5.15E-06	6.61E-06
mmu-miR-384-5p	1	15	0.03	0.74	4.624490865	Up	1.20E-05	1.51E-05
mmu-miR-3057-5p	1	13	0.03	0.64	4.415037499	Up	6.40E-05	7.78E-05
mmu-miR-322-3p	2	30	0.07	1.49	4.411813598	Up	3.10E-10	4.51E-10
novel_mir679	322	3758	10.97	186.34	4.086301968	Up	0	0
mmu-miR-3065-3p	5	50	0.17	2.48	3.866733469	Up	7.50E-15	1.17E-14
mmu-miR-30d-3p	3	29	0.1	1.44	3.847996907	Up	4.77E-09	6.76E-09
mmu-miR-7115-5p	3	28	0.1	1.39	3.797012978	Up	1.07E-08	1.51E-08
mmu-miR-1934-3p	2	19	0.07	0.94	3.74723393	Up	2.72E-06	3.54E-06

mmu-miR-501-3p	213	1688	7.25	83.7	3.529174723	Up	0	0
mmu-miR-669a-3p	3	23	0.1	1.14	3.510961919	Up	5.71E-07	7.56E-07
mmu-miR-132-3p	2	14	0.07	0.69	3.301169535	Up	0.000145922	0.000175171
mmu-miR-145a-3p	3	17	0.1	0.84	3.070389328	Up	5.85E-05	7.12E-05
mmu-miR-378c	20	110	0.68	5.45	3.002649578	Up	6.25E-25	1.10E-24
mmu-miR-29c-5p	3	16	0.1	0.79	2.981852653	Up	0.000123935	0.000148943
novel_mir1	3055	14537	104.04	720.82	2.792500737	Up	0	0
mmu-miR-378b	1358	6140	46.25	304.45	2.718680042	Up	0	0
mmu-miR-449a-5p	6	25	0.2	1.24	2.632268215	Up	6.60E-06	8.45E-06
mmu-miR-29b-3p	6	23	0.2	1.14	2.510961919	Up	2.64E-05	3.27E-05
mmu-miR-142a-3p	9	34	0.31	1.69	2.446683126	Up	3.38E-07	4.59E-07
mmu-miR-378a-3p	32847	115383	1118.61	5721.28	2.354630819	Up	0	0
mmu-miR-5126	1718	5337	58.51	264.64	2.177276016	Up	0	0
mmu-miR-134-5p	350	1046	11.92	51.87	2.121516134	Up	1.96E-148	1.03E-147
novel_mir441	7068	18459	240.7	915.29	1.926992777	Up	0	0
mmu-miR-34c-3p	285	732	9.71	36.3	1.902426347	Up	1.83E-90	6.82E-90
mmu-miR-1981-5p	5585	11985	190.2	594.28	1.643625583	Up	0	0
mmu-miR-140-3p	487	964	16.58	47.8	1.527566611	Up	9.06E-87	3.18E-86
mmu-miR-532-3p	868	1669	29.56	82.76	1.485287376	Up	9.21E-143	4.77E-142
novel_mir498	95	181	3.24	8.97	1.469114172	Up	8.92E-17	1.42E-16
mmu-miR-877-5p	2487	4330	84.7	214.7	1.341888317	Up	0	0
mmu-miR-330-5p	175	293	5.96	14.53	1.285650467	Up	1.57E-21	2.66E-21
novel_mir507	1396	0	47.54	0.001	-15.53685428	Down	0	0
novel_mir453	1020	0	34.74	0.001	-15.08431013	Down	2.94E-232	1.86E-231
novel_mir727	849	0	28.91	0.001	-14.81928099	Down	1.98E-193	1.17E-192
mmu-miR-7115-3p	800	0	27.24	0.001	-14.73343908	Down	2.64E-182	1.52E-181
novel_mir589	731	0	24.89	0.001	-14.60327861	Down	1.23E-166	6.90E-166
mmu-miR-297c-5p	728	0	24.79	0.001	-14.59747065	Down	5.90E-166	3.28E-165
novel_mir361	663	0	22.58	0.001	-14.46275787	Down	3.39E-151	1.80E-150
novel_mir201	627	0	21.35	0.001	-14.38194845	Down	5.06E-143	2.64E-142
novel_mir714	555	0	18.9	0.001	-14.20609861	Down	1.13E-126	5.49E-126
mmu-miR-3470b	535	0	18.22	0.001	-14.15323534	Down	3.93E-122	1.87E-121
novel_mir439	517	0	17.61	0.001	-14.10410729	Down	4.80E-118	2.26E-117
novel_mir147	515	0	17.54	0.001	-14.09836113	Down	1.37E-117	6.40E-117
novel_mir774	510	0	17.37	0.001	-14.08431013	Down	1.87E-116	8.67E-116
novel_mir154	509	0	17.33	0.001	-14.08098403	Down	3.15E-116	1.46E-115

novel_mir57	508	0	17.3	0.001	-14.07848442	Down	5.31E-116	2.45E-115
mmu-miR-7031-5p	502	0	17.1	0.001	-14.0617087	Down	1.22E-114	5.56E-114
novel_mir485	498	0	16.96	0.001	-14.04984855	Down	9.90E-114	4.45E-113
novel_mir596	492	0	16.76	0.001	-14.03273453	Down	2.28E-112	1.01E-111
novel_mir121	485	0	16.52	0.001	-14.01192607	Down	8.86E-111	3.88E-110
novel_mir30	472	0	16.07	0.001	-13.97208231	Down	7.93E-108	3.45E-107
novel_mir555	467	0	15.9	0.001	-13.95673915	Down	1.08E-106	4.67E-106
novel_mir518	463	0	15.77	0.001	-13.94489504	Down	8.77E-106	3.77E-105
mmu-miR-6924-5p	462	0	15.73	0.001	-13.94123105	Down	1.48E-105	6.33E-105
novel_mir741	462	0	15.73	0.001	-13.94123105	Down	1.48E-105	6.30E-105
novel_mir345	461	0	15.7	0.001	-13.93847694	Down	2.50E-105	1.06E-104
novel_mir755	456	0	15.53	0.001	-13.92277021	Down	3.41E-104	1.43E-103
novel_mir194	456	0	15.53	0.001	-13.92277021	Down	3.41E-104	1.42E-103
novel_mir508	455	0	15.5	0.001	-13.9199806	Down	5.75E-104	2.39E-103
novel_mir686	451	0	15.36	0.001	-13.9068906	Down	4.65E-103	1.93E-102
novel_mir183	442	0	15.05	0.001	-13.87747587	Down	5.14E-101	2.11E-100
novel_mir401	439	0	14.95	0.001	-13.86785786	Down	2.47E-100	1.01E-99
novel_mir709	438	0	14.92	0.001	-13.86495992	Down	4.16E-100	1.69E-99
novel_mir273	438	0	14.92	0.001	-13.86495992	Down	4.16E-100	1.69E-99
novel_mir593	436	0	14.85	0.001	-13.85817531	Down	1.18E-99	4.77E-99
mmu-miR-6919-3p	431	0	14.68	0.001	-13.84156435	Down	1.62E-98	6.46E-98
novel_mir315	426	0	14.51	0.001	-13.8247599	Down	2.21E-97	8.79E-97
novel_mir23	425	0	14.47	0.001	-13.8207773	Down	3.73E-97	1.48E-96
novel_mir44	424	0	14.44	0.001	-13.81778312	Down	6.29E-97	2.48E-96
novel_mir600	421	0	14.34	0.001	-13.8077574	Down	3.02E-96	1.19E-95
novel_mir443	417	0	14.2	0.001	-13.79360331	Down	2.44E-95	9.58E-95
novel_mir516	414	0	14.1	0.001	-13.78340754	Down	1.17E-94	4.58E-94
novel_mir324	412	0	14.03	0.001	-13.77622739	Down	3.34E-94	1.30E-93
novel_mir435	411	0	14	0.001	-13.77313921	Down	5.63E-94	2.18E-93
novel_mir326	411	0	14	0.001	-13.77313921	Down	5.63E-94	2.17E-93
novel_mir533	409	0	13.93	0.001	-13.76590764	Down	1.60E-93	6.16E-93
novel_mir751	403	0	13.72	0.001	-13.74399286	Down	3.69E-92	1.41E-91
novel_mir368	403	0	13.72	0.001	-13.74399286	Down	3.69E-92	1.41E-91
novel_mir208	402	0	13.69	0.001	-13.74083483	Down	6.22E-92	2.36E-91
novel_mir411	400	0	13.62	0.001	-13.73343908	Down	1.77E-91	6.70E-91
novel_mir592	397	0	13.52	0.001	-13.72280753	Down	8.50E-91	3.19E-90

novel_mir693	397	0	13.52	0.001	-13.72280753	Down	8.50E-91	3.18E-90
novel_mir552	395	0	13.45	0.001	-13.71531855	Down	2.42E-90	8.99E-90
novel_mir472	393	0	13.38	0.001	-13.7077905	Down	6.88E-90	2.55E-89
novel_mir469	392	0	13.35	0.001	-13.70455212	Down	1.16E-89	4.28E-89
mmu-miR-7002-5p	391	0	13.32	0.001	-13.70130646	Down	1.96E-89	7.20E-89
novel_mir49	389	0	13.25	0.001	-13.69370474	Down	5.57E-89	2.03E-88
novel_mir432	387	0	13.18	0.001	-13.68606275	Down	1.58E-88	5.75E-88
novel_mir448	386	0	13.15	0.001	-13.68277518	Down	2.67E-88	9.67E-88
novel_mir204	384	0	13.08	0.001	-13.67507492	Down	7.60E-88	2.73E-87
novel_mir680	383	0	13.04	0.001	-13.67065625	Down	1.28E-87	4.59E-87
novel_mir638	381	0	12.98	0.001	-13.66400276	Down	3.65E-87	1.30E-86
novel_mir551	380	0	12.94	0.001	-13.65955	Down	6.16E-87	2.18E-86
novel_mir490	380	0	12.94	0.001	-13.65955	Down	6.16E-87	2.17E-86
mmu-miR-467b-5p	377	0	12.84	0.001	-13.64835758	Down	2.95E-86	1.03E-85
novel_mir386	376	0	12.8	0.001	-13.64385619	Down	4.98E-86	1.73E-85
novel_mir69	376	0	12.8	0.001	-13.64385619	Down	4.98E-86	1.73E-85
mmu-miR-299b-3p	366	0	12.46	0.001	-13.60501645	Down	9.30E-84	3.18E-83
novel_mir617	365	0	12.43	0.001	-13.60153868	Down	1.57E-83	5.35E-83
novel_mir275	362	0	12.33	0.001	-13.58988518	Down	7.53E-83	2.55E-82
novel_mir274	362	0	12.33	0.001	-13.58988518	Down	7.53E-83	2.54E-82
novel_mir241	360	0	12.26	0.001	-13.58167136	Down	2.14E-82	7.19E-82
novel_mir260	359	0	12.23	0.001	-13.57813678	Down	3.61E-82	1.21E-81
mmu-miR-411-3p	357	0	12.16	0.001	-13.56985561	Down	1.03E-81	3.43E-81
novel_mir213	351	0	11.95	0.001	-13.544723	Down	2.37E-80	7.88E-80
novel_mir293	351	0	11.95	0.001	-13.544723	Down	2.37E-80	7.85E-80
novel_mir418	349	0	11.89	0.001	-13.53746109	Down	6.74E-80	2.23E-79
novel_mir554	348	0	11.85	0.001	-13.53259944	Down	1.14E-79	3.75E-79
novel_mir481	348	0	11.85	0.001	-13.53259944	Down	1.14E-79	3.73E-79
novel_mir350	347	0	11.82	0.001	-13.52894242	Down	1.92E-79	6.26E-79
novel_mir476	345	0	11.75	0.001	-13.52037314	Down	5.45E-79	1.78E-78
novel_mir235	345	0	11.75	0.001	-13.52037314	Down	5.45E-79	1.77E-78
novel_mir344	343	0	11.68	0.001	-13.51175265	Down	1.55E-78	5.02E-78
novel_mir176	342	0	11.65	0.001	-13.50804233	Down	2.62E-78	8.45E-78
novel_mir607	342	0	11.65	0.001	-13.50804233	Down	2.62E-78	8.42E-78
novel_mir573	341	0	11.61	0.001	-13.50308035	Down	4.41E-78	1.42E-77
mmu-miR-770-5p	339	0	11.54	0.001	-13.4943556	Down	1.26E-77	4.01E-77

novel_mir349	337	0	11.48	0.001	-13.48683502	Down	3.57E-77	1.13E-76
novel_mir497	337	0	11.48	0.001	-13.48683502	Down	3.57E-77	1.13E-76
novel_mir190	336	0	11.44	0.001	-13.48179943	Down	6.03E-77	1.89E-76
novel_mir164	336	0	11.44	0.001	-13.48179943	Down	6.03E-77	1.88E-76
novel_mir320	333	0	11.34	0.001	-13.46913302	Down	2.89E-76	8.99E-76
novel_mir694	333	0	11.34	0.001	-13.46913302	Down	2.89E-76	8.96E-76
novel_mir98	330	0	11.24	0.001	-13.45635442	Down	1.39E-75	4.26E-75
novel_mir228	329	0	11.2	0.001	-13.45121111	Down	2.34E-75	7.17E-75
novel_mir640	329	0	11.2	0.001	-13.45121111	Down	2.34E-75	7.15E-75
novel_mir738	326	0	11.1	0.001	-13.43827206	Down	1.12E-74	3.42E-74
novel_mir599	325	0	11.07	0.001	-13.4343676	Down	1.90E-74	5.76E-74
novel_mir486	324	0	11.03	0.001	-13.42914517	Down	3.20E-74	9.66E-74
novel_mir282	324	0	11.03	0.001	-13.42914517	Down	3.20E-74	9.63E-74
novel_mir316	323	0	11	0.001	-13.4252159	Down	5.40E-74	1.62E-73
novel_mir772	322	0	10.97	0.001	-13.42127591	Down	9.10E-74	2.73E-73
novel_mir606	317	0	10.8	0.001	-13.39874369	Down	1.24E-72	3.70E-72
novel_mir558	317	0	10.8	0.001	-13.39874369	Down	1.24E-72	3.69E-72
novel_mir304	316	0	10.76	0.001	-13.39339046	Down	2.10E-72	6.21E-72
novel_mir698	316	0	10.76	0.001	-13.39339046	Down	2.10E-72	6.19E-72
novel_mir247	314	0	10.69	0.001	-13.38397423	Down	5.97E-72	1.76E-71
novel_mir177	314	0	10.69	0.001	-13.38397423	Down	5.97E-72	1.75E-71
mmu-miR-6952-3p	313	0	10.66	0.001	-13.37991982	Down	1.01E-71	2.95E-71
novel_mir75	312	0	10.63	0.001	-13.37585398	Down	1.70E-71	4.96E-71
novel_mir226	312	0	10.63	0.001	-13.37585398	Down	1.70E-71	4.95E-71
novel_mir64	307	0	10.45	0.001	-13.35121532	Down	2.32E-70	6.68E-70
novel_mir181	306	0	10.42	0.001	-13.34706766	Down	3.91E-70	1.12E-69
novel_mir140	306	0	10.42	0.001	-13.34706766	Down	3.91E-70	1.12E-69
novel_mir182	306	0	10.42	0.001	-13.34706766	Down	3.91E-70	1.11E-69
novel_mir341	303	0	10.32	0.001	-13.33315535	Down	1.88E-69	5.32E-69
novel_mir419	301	0	10.25	0.001	-13.32333629	Down	5.34E-69	1.50E-68
novel_mir84	300	0	10.22	0.001	-13.31910758	Down	9.01E-69	2.53E-68
mmu-miR-6936-5p	300	0	10.22	0.001	-13.31910758	Down	9.01E-69	2.52E-68
novel_mir740	297	0	10.11	0.001	-13.30349538	Down	4.32E-68	1.20E-67
novel_mir662	297	0	10.11	0.001	-13.30349538	Down	4.32E-68	1.20E-67
novel_mir407	297	0	10.11	0.001	-13.30349538	Down	4.32E-68	1.20E-67
novel_mir743	295	0	10.05	0.001	-13.29490788	Down	1.23E-67	3.39E-67

novel_mir218	293	0	9.98	0.001	-13.2848241	Down	3.50E-67	9.60E-67
novel_mir701	293	0	9.98	0.001	-13.2848241	Down	3.50E-67	9.57E-67
mmu-miR-182-3p	292	0	9.94	0.001	-13.27903014	Down	5.90E-67	1.61E-66
mmu-miR-363-5p	290	0	9.88	0.001	-13.27029533	Down	1.68E-66	4.56E-66
novel_mir690	290	0	9.88	0.001	-13.27029533	Down	1.68E-66	4.55E-66
novel_mir595	288	0	9.81	0.001	-13.26003742	Down	4.78E-66	1.29E-65
novel_mir289	288	0	9.81	0.001	-13.26003742	Down	4.78E-66	1.28E-65
novel_mir588	287	0	9.77	0.001	-13.25414285	Down	8.06E-66	2.15E-65
novel_mir149	284	0	9.67	0.001	-13.23930017	Down	3.87E-65	1.03E-64
novel_mir586	283	0	9.64	0.001	-13.23481743	Down	6.53E-65	1.73E-64
novel_mir636	278	0	9.47	0.001	-13.20914871	Down	8.91E-64	2.35E-63
novel_mir394	278	0	9.47	0.001	-13.20914871	Down	8.91E-64	2.34E-63
novel_mir198	277	0	9.43	0.001	-13.20304206	Down	1.50E-63	3.94E-63
novel_mir237	271	0	9.23	0.001	-13.17211493	Down	3.46E-62	8.97E-62
novel_mir771	270	0	9.19	0.001	-13.16584915	Down	5.84E-62	1.51E-61
novel_mir483	268	0	9.13	0.001	-13.15639914	Down	1.66E-61	4.28E-61
novel_mir90	267	0	9.09	0.001	-13.15006458	Down	2.80E-61	7.20E-61
novel_mir78	264	0	8.99	0.001	-13.1341054	Down	1.35E-60	3.43E-60
novel_mir187	263	0	8.96	0.001	-13.12928302	Down	2.27E-60	5.77E-60
novel_mir302	262	0	8.92	0.001	-13.12282799	Down	3.83E-60	9.69E-60
novel_mir58	262	0	8.92	0.001	-13.12282799	Down	3.83E-60	9.66E-60
novel_mir427	259	0	8.82	0.001	-13.10656294	Down	1.84E-59	4.62E-59
novel_mir109	259	0	8.82	0.001	-13.10656294	Down	1.84E-59	4.61E-59
novel_mir131	257	0	8.75	0.001	-13.0950673	Down	5.23E-59	1.31E-58
novel_mir55	256	0	8.72	0.001	-13.09011242	Down	8.82E-59	2.20E-58
novel_mir560	256	0	8.72	0.001	-13.09011242	Down	8.82E-59	2.20E-58
novel_mir77	255	0	8.68	0.001	-13.08347933	Down	1.49E-58	3.69E-58
novel_mir712	254	0	8.65	0.001	-13.07848442	Down	2.51E-58	6.20E-58
novel_mir150	254	0	8.65	0.001	-13.07848442	Down	2.51E-58	6.19E-58
novel_mir348	254	0	8.65	0.001	-13.07848442	Down	2.51E-58	6.18E-58
novel_mir114	253	0	8.62	0.001	-13.07347215	Down	4.23E-58	1.04E-57
novel_mir172	253	0	8.62	0.001	-13.07347215	Down	4.23E-58	1.03E-57
novel_mir630	251	0	8.55	0.001	-13.0617087	Down	1.20E-57	2.93E-57
mmu-miR-6994-3p	251	0	8.55	0.001	-13.0617087	Down	1.20E-57	2.92E-57
novel_mir38	249	0	8.48	0.001	-13.04984855	Down	3.43E-57	8.30E-57
novel_mir167	248	0	8.45	0.001	-13.04473563	Down	5.78E-57	1.40E-56

novel_mir60	245	0	8.34	0.001	-13.02583167	Down	2.77E-56	6.69E-56
mmu-miR-3071-3p	243	0	8.28	0.001	-13.01541505	Down	7.89E-56	1.89E-55
novel_mir225	242	0	8.24	0.001	-13.00842862	Down	1.33E-55	3.19E-55
novel_mir37	240	0	8.17	0.001	-12.99612036	Down	3.79E-55	9.03E-55
novel_mir562	239	0	8.14	0.001	-12.99081308	Down	6.39E-55	1.52E-54
novel_mir621	238	0	8.11	0.001	-12.9854862	Down	1.08E-54	2.55E-54
mmu-miR-292b-5p	238	0	8.11	0.001	-12.9854862	Down	1.08E-54	2.55E-54
novel_mir352	238	0	8.11	0.001	-12.9854862	Down	1.08E-54	2.54E-54
novel_mir249	237	0	8.07	0.001	-12.97835296	Down	1.82E-54	4.27E-54
novel_mir717	236	0	8.04	0.001	-12.97297979	Down	3.07E-54	7.19E-54
novel_mir145	236	0	8.04	0.001	-12.97297979	Down	3.07E-54	7.17E-54
novel_mir268	235	0	8	0.001	-12.96578428	Down	5.17E-54	1.20E-53
novel_mir205	235	0	8	0.001	-12.96578428	Down	5.17E-54	1.20E-53
novel_mir757	235	0	8	0.001	-12.96578428	Down	5.17E-54	1.20E-53
novel_mir343	234	0	7.97	0.001	-12.96036401	Down	8.73E-54	2.02E-53
novel_mir332	229	0	7.8	0.001	-12.92925841	Down	1.19E-52	2.72E-52
novel_mir166	227	0	7.73	0.001	-12.9162527	Down	3.39E-52	7.69E-52
novel_mir689	226	0	7.7	0.001	-12.91064273	Down	5.72E-52	1.30E-51
mmu-miR-6953-3p	226	0	7.7	0.001	-12.91064273	Down	5.72E-52	1.29E-51
novel_mir360	221	0	7.53	0.001	-12.87843415	Down	7.81E-51	1.75E-50
novel_mir261	220	0	7.49	0.001	-12.87075	Down	1.32E-50	2.94E-50
novel_mir510	218	0	7.42	0.001	-12.85720347	Down	3.75E-50	8.35E-50
mmu-miR-7681-5p	215	0	7.32	0.001	-12.83762793	Down	1.80E-49	3.99E-49
novel_mir143	215	0	7.32	0.001	-12.83762793	Down	1.80E-49	3.98E-49
novel_mir745	215	0	7.32	0.001	-12.83762793	Down	1.80E-49	3.97E-49
novel_mir27	213	0	7.25	0.001	-12.82376528	Down	5.12E-49	1.12E-48
novel_mir511	210	0	7.15	0.001	-12.80372753	Down	2.46E-48	5.35E-48
novel_mir87	209	0	7.12	0.001	-12.79766153	Down	4.14E-48	8.97E-48
novel_mir323	207	0	7.05	0.001	-12.78340754	Down	1.18E-47	2.54E-47
novel_mir436	206	0	7.02	0.001	-12.77725532	Down	1.99E-47	4.28E-47
novel_mir134	206	0	7.02	0.001	-12.77725532	Down	1.99E-47	4.27E-47
mmu-miR-764-5p	205	0	6.98	0.001	-12.76901132	Down	3.35E-47	7.19E-47
mmu-miR-154-3p	204	0	6.95	0.001	-12.76279726	Down	5.66E-47	1.21E-46
novel_mir223	201	0	6.85	0.001	-12.74188827	Down	2.72E-46	5.78E-46
novel_mir210	200	0	6.81	0.001	-12.73343908	Down	4.58E-46	9.70E-46
novel_mir532	199	0	6.78	0.001	-12.72706956	Down	7.73E-46	1.63E-45

novel_mir382	196	0	6.67	0.001	-12.70347105	Down	3.71E-45	7.78E-45
novel_mir463	193	0	6.57	0.001	-12.68167766	Down	1.78E-44	3.70E-44
novel_mir81	189	0	6.44	0.001	-12.65284497	Down	1.44E-43	2.98E-43
novel_mir165	189	0	6.44	0.001	-12.65284497	Down	1.44E-43	2.98E-43
novel_mir59	181	0	6.16	0.001	-12.58871464	Down	9.45E-42	1.93E-41
novel_mir438	179	0	6.1	0.001	-12.57459353	Down	2.69E-41	5.47E-41
novel_mir564	179	0	6.1	0.001	-12.57459353	Down	2.69E-41	5.46E-41
novel_mir85	179	0	6.1	0.001	-12.57459353	Down	2.69E-41	5.45E-41
novel_mir207	175	0	5.96	0.001	-12.54109662	Down	2.18E-40	4.38E-40
novel_mir627	175	0	5.96	0.001	-12.54109662	Down	2.18E-40	4.38E-40
novel_mir412	173	0	5.89	0.001	-12.52405192	Down	6.19E-40	1.24E-39
novel_mir220	173	0	5.89	0.001	-12.52405192	Down	6.19E-40	1.24E-39
novel_mir505	173	0	5.89	0.001	-12.52405192	Down	6.19E-40	1.23E-39
novel_mir732	165	0	5.62	0.001	-12.45635442	Down	4.06E-38	8.01E-38
novel_mir667	164	0	5.59	0.001	-12.44863257	Down	6.84E-38	1.35E-37
novel_mir203	161	0	5.48	0.001	-12.41996018	Down	3.28E-37	6.43E-37
novel_mir535	157	0	5.35	0.001	-12.38532318	Down	2.66E-36	5.17E-36
novel_mir318	156	0	5.31	0.001	-12.37449615	Down	4.49E-36	8.70E-36
novel_mir61	156	0	5.31	0.001	-12.37449615	Down	4.49E-36	8.68E-36
novel_mir538	154	0	5.24	0.001	-12.3553511	Down	1.28E-35	2.47E-35
novel_mir127	144	0	4.9	0.001	-12.25856603	Down	2.38E-33	4.49E-33
novel_mir625	143	0	4.87	0.001	-12.24970606	Down	4.02E-33	7.57E-33
novel_mir159	141	0	4.8	0.001	-12.22881869	Down	1.14E-32	2.15E-32
novel_mir222	139	0	4.73	0.001	-12.20762447	Down	3.25E-32	6.07E-32
mmu-miR-7034-3p	138	0	4.7	0.001	-12.19844504	Down	5.48E-32	1.02E-31
novel_mir267	135	0	4.6	0.001	-12.16741815	Down	2.63E-31	4.89E-31
novel_mir527	125	0	4.26	0.001	-12.05663772	Down	4.91E-29	9.01E-29
novel_mir212	123	0	4.19	0.001	-12.03273453	Down	1.40E-28	2.54E-28
novel_mir447	123	0	4.19	0.001	-12.03273453	Down	1.40E-28	2.54E-28
novel_mir216	122	0	4.15	0.001	-12.01889562	Down	2.36E-28	4.27E-28
novel_mir313	116	0	3.95	0.001	-11.94763694	Down	5.43E-27	9.72E-27
novel_mir53	115	0	3.92	0.001	-11.93663794	Down	9.15E-27	1.63E-26
novel_mir610	108	0	3.68	0.001	-11.84549005	Down	3.56E-25	6.27E-25
novel_mir695	103	0	3.51	0.001	-11.77725532	Down	4.86E-24	8.47E-24
novel_mir329	99	0	3.37	0.001	-11.71853288	Down	3.93E-23	6.76E-23
mmu-miR-7058-3p	95	0	3.24	0.001	-11.6617781	Down	3.18E-22	5.43E-22

novel_mir754	94	0	3.2	0.001	-11.64385619	Down	5.37E-22	9.13E-22
novel_mir283	90	0	3.06	0.001	-11.57931594	Down	4.35E-21	7.32E-21
novel_mir659	88	0	3	0.001	-11.55074679	Down	1.24E-20	2.07E-20
novel_mir647	84	0	2.86	0.001	-11.48179943	Down	1.00E-19	1.65E-19
novel_mir420	79	0	2.69	0.001	-11.39339046	Down	1.37E-18	2.23E-18
novel_mir658	76	0	2.59	0.001	-11.33873638	Down	6.56E-18	1.06E-17
mmu-miR-6901-3p	55	0	1.87	0.001	-10.86882255	Down	3.85E-13	5.93E-13
novel_mir231	50	0	1.7	0.001	-10.73131903	Down	5.26E-12	7.90E-12
novel_mir91	38	0	1.29	0.001	-10.33315535	Down	2.79E-09	3.96E-09
mmu-miR-335-3p	1821	1	62.01	0.05	-10.27635708	Down	0	0
mmu-miR-1249-3p	1709	1	58.2	0.05	-10.18487534	Down	0	0
novel_mir200	34	0	1.16	0.001	-10.17990909	Down	2.26E-08	3.17E-08
novel_mir265	24	0	0.82	0.001	-9.6794801	Down	4.21E-06	5.41E-06
novel_mir73	23	0	0.78	0.001	-9.607330314	Down	7.10E-06	8.95E-06
mmu-miR-363-3p	562	1	19.14	0.05	-8.58044702	Down	6.69E-126	3.22E-125
mmu-miR-98-3p	321	1	10.93	0.05	-7.772149591	Down	2.03E-71	5.89E-71
mmu-miR-214-3p	3450	13	117.49	0.64	-7.520250349	Down	0	0
mmu-miR-485-3p	1118	5	38.07	0.25	-7.250582665	Down	2.74E-243	1.75E-242
mmu-miR-466d-3p	619	3	21.08	0.15	-7.134768556	Down	9.05E-135	4.60E-134
mmu-let-7b-3p	4824	25	164.28	1.24	-7.049672922	Down	0	0
mmu-miR-7658-5p	386	2	13.15	0.1	-7.038918989	Down	3.37E-84	1.16E-83
mmu-miR-466k	531	3	18.08	0.15	-6.913288367	Down	5.49E-115	2.51E-114
mmu-miR-10a-3p	497	3	16.93	0.15	-6.818475662	Down	2.37E-107	1.03E-106
mmu-miR-8091	249	2	8.48	0.1	-6.40599236	Down	1.82E-53	4.20E-53
mmu-miR-365-3p	2080	17	70.84	0.84	-6.398031074	Down	0	0
mmu-miR-7030-3p	247	2	8.41	0.1	-6.394033895	Down	5.09E-53	1.17E-52
mmu-miR-342-5p	703	6	23.94	0.3	-6.318316841	Down	2.25E-148	1.18E-147
mmu-miR-16-2-3p	819	8	27.89	0.4	-6.123604124	Down	5.20E-171	2.94E-170
mmu-miR-664-3p	7023	73	239.17	3.62	-6.045902931	Down	0	0
mmu-miR-211-5p	969	11	33	0.55	-5.906890596	Down	1.11E-199	6.65E-199
mmu-miR-296-5p	352	4	11.99	0.2	-5.905687849	Down	2.61E-73	7.79E-73
mmu-miR-7658-3p	958	11	32.62	0.55	-5.890181353	Down	3.09E-197	1.84E-196
mmu-miR-136-3p	254	3	8.65	0.15	-5.849665727	Down	4.87E-53	1.12E-52
mmu-miR-669c-5p	2122	26	72.27	1.29	-5.807953924	Down	0	0
mmu-miR-122-3p	1028	13	35.01	0.64	-5.773551346	Down	9.81E-210	6.04E-209
mmu-miR-29b-1-5p	618	8	21.05	0.4	-5.717676423	Down	2.44E-126	1.18E-125

mmu-miR-871-5p	306	4	10.42	0.2	-5.703211467	Down	4.19E-63	1.10E-62
mmu-miR-1981-3p	1371	19	46.69	0.94	-5.634309022	Down	8.85E-277	6.12E-276
mmu-miR-3473g	429	6	14.61	0.3	-5.605849867	Down	1.97E-87	7.04E-87
mmu-miR-495-3p	1555	22	52.96	1.09	-5.602503082	Down	0	0
mmu-miR-18a-5p	2295	33	78.16	1.64	-5.574662747	Down	0	0
mmu-miR-1b-5p	1922	28	65.45	1.39	-5.557236404	Down	0	0
mmu-miR-466h-3p	6225	91	211.99	4.51	-5.554724968	Down	0	0
mmu-miR-6960-5p	951	14	32.39	0.69	-5.552808296	Down	3.09E-191	1.80E-190
mmu-miR-125b-5p	28523	419	971.36	20.78	-5.546738519	Down	0	0
mmu-miR-101b-3p	2311	34	78.7	1.69	-5.541268484	Down	0	0
mmu-miR-770-3p	665	10	22.65	0.5	-5.501439145	Down	7.83E-134	3.94E-133
mmu-miR-669o-3p	522	8	17.78	0.4	-5.474111514	Down	4.04E-105	1.71E-104
mmu-let-7c-5p	693066	10730	23602.57	532.05	-5.471238317	Down	0	0
mmu-miR-6955-3p	127	2	4.33	0.1	-5.43629512	Down	2.45E-26	4.37E-26
mmu-miR-23b-5p	2841	47	96.75	2.33	-5.375859801	Down	0	0
novel_mir41	962	17	32.76	0.84	-5.285402219	Down	1.80E-189	1.04E-188
mmu-miR-26b-3p	832	15	28.33	0.74	-5.258661521	Down	1.17E-163	6.49E-163
mmu-miR-1982-3p	166	3	5.65	0.15	-5.235216462	Down	1.34E-33	2.54E-33
mmu-miR-574-3p	57274	1046	1950.48	51.87	-5.232785025	Down	0	0
mmu-miR-450b-5p	385	7	13.11	0.35	-5.227168953	Down	2.34E-76	7.29E-76
mmu-miR-10b-5p	56638	1110	1928.82	55.04	-5.131094235	Down	0	0
mmu-miR-450a-5p	2033	40	69.23	1.98	-5.127825013	Down	0	0
mmu-let-7f-1-3p	8696	178	296.14	8.83	-5.067722122	Down	0	0
mmu-miR-125a-5p	47302	985	1610.88	48.84	-5.043642016	Down	0	0
mmu-miR-30c-5p	4172	87	142.08	4.31	-5.042871807	Down	0	0
novel_mir580	529	11	18.02	0.55	-5.034023582	Down	1.25E-102	5.16E-102
mmu-miR-7021-5p	470	10	16.01	0.5	-5.000901403	Down	4.85E-91	1.83E-90
mmu-let-7b-5p	450255	9887	15333.57	490.25	-4.967032187	Down	0	0
novel_mir141	836	19	28.47	0.94	-4.920637926	Down	2.47E-159	1.34E-158
mmu-miR-190b-5p	522	12	17.78	0.6	-4.889149013	Down	7.66E-100	3.09E-99
mmu-miR-98-5p	20797	487	708.25	24.15	-4.874163602	Down	0	0
mmu-let-7f-5p	150494	3545	5125.12	175.78	-4.865742947	Down	0	0
mmu-let-7g-5p	583994	14179	19888.09	703.07	-4.822092584	Down	0	0
mmu-miR-181d-3p	285	7	9.71	0.35	-4.794044468	Down	1.51E-54	3.55E-54
mmu-miR-6899-3p	197	5	6.71	0.25	-4.746312766	Down	6.98E-38	1.37E-37
mmu-miR-10a-5p	172002	4430	5857.58	219.66	-4.736960668	Down	0	0

mmu-miR-669f-3p	234	6	7.97	0.3	-4.731545318	Down	1.05E-44	2.21E-44
mmu-miR-92b-3p	13554	351	461.59	17.4	-4.729452758	Down	0	0
mmu-miR-329-3p	1079	28	36.75	1.39	-4.724587462	Down	5.95E-201	3.59E-200
mmu-let-7a-5p	212506	5626	7236.96	278.97	-4.697201907	Down	0	0
mmu-miR-106b-3p	1160	31	39.5	1.54	-4.680850397	Down	7.54E-215	4.73E-214
mmu-miR-543-3p	1410	40	48.02	1.98	-4.600063068	Down	4.38E-258	2.89E-257
mmu-miR-19b-3p	17318	498	589.77	24.69	-4.578153697	Down	0	0
mmu-miR-1843a-5p	3545	103	120.73	5.11	-4.562317112	Down	0	0
mmu-miR-199b-3p	419760	12600	14295.05	624.77	-4.516046676	Down	0	0
mmu-miR-124-3p	1460	44	49.72	2.18	-4.511426256	Down	3.31E-264	2.22E-263
mmu-let-7d-3p	829655	25169	28254.15	1248.01	-4.500761388	Down	0	0
mmu-miR-219a-1-3p	655	20	22.31	0.99	-4.494118178	Down	4.48E-119	2.12E-118
mmu-miR-410-3p	2887	89	98.32	4.41	-4.478634355	Down	0	0
mmu-miR-1843a-3p	3360	105	114.43	5.21	-4.457038149	Down	0	0
mmu-miR-466i-5p	3069	96	104.52	4.76	-4.456673646	Down	0	0
mmu-miR-1a-3p	3691	118	125.7	5.85	-4.425404215	Down	0	0
mmu-miR-18a-3p	1002	32	34.12	1.59	-4.423518976	Down	8.63E-180	4.92E-179
mmu-miR-148b-3p	669	22	22.78	1.09	-4.385367707	Down	7.74E-120	3.68E-119
mmu-miR-199a-3p	5439	191	185.23	9.47	-4.289809542	Down	0	0
mmu-let-7d-5p	88588	3114	3016.89	154.41	-4.288224001	Down	0	0
mmu-miR-877-3p	1440	51	49.04	2.53	-4.276749689	Down	3.30E-252	2.15E-251
novel_mir3	504	18	17.16	0.89	-4.269100407	Down	4.27E-89	1.57E-88
mmu-miR-126a-5p	2565	92	87.35	4.56	-4.259701974	Down	0	0
mmu-miR-381-3p	222	8	7.56	0.4	-4.240314329	Down	6.56E-40	1.30E-39
mmu-miR-142a-5p	1952	71	66.48	3.52	-4.239273048	Down	0	0
mmu-miR-134-3p	375	14	12.77	0.69	-4.210018353	Down	5.22E-66	1.40E-65
mmu-miR-1306-5p	3075	115	104.72	5.7	-4.199431273	Down	0	0
mmu-miR-29a-5p	357	14	12.16	0.69	-4.139403057	Down	3.26E-62	8.47E-62
mmu-miR-9-5p	205	8	6.98	0.4	-4.125155131	Down	2.56E-36	4.99E-36
mmu-miR-222-3p	42054	1686	1432.16	83.6	-4.098545926	Down	0	0
mmu-miR-144-3p	9264	374	315.49	18.54	-4.088881128	Down	0	0
mmu-miR-3058-3p	393	16	13.38	0.79	-4.082081652	Down	9.45E-68	2.61E-67
mmu-miR-26b-5p	12288	505	418.47	25.04	-4.062817733	Down	0	0
mmu-miR-199a-5p	294	12	10.01	0.6	-4.060335663	Down	5.38E-51	1.21E-50
mmu-miR-204-5p	40468	1684	1378.15	83.5	-4.044812914	Down	0	0
novel_mir354	406	17	13.83	0.84	-4.041268018	Down	1.79E-69	5.08E-69

mmu-miR-467a-5p	2002	85	68.18	4.21	-4.017456461	Down	0	0
mmu-miR-210-3p	1358	58	46.25	2.88	-4.005312649	Down	1.02E-227	6.44E-227
mmu-miR-6948-3p	514	22	17.5	1.09	-4.004954882	Down	4.21E-87	1.49E-86
mmu-miR-3077-3p	281	12	9.57	0.6	-3.995484519	Down	2.85E-48	6.19E-48
mmu-miR-30b-5p	5056	235	172.18	11.65	-3.885515713	Down	0	0
mmu-miR-574-5p	8937	416	304.35	20.63	-3.882915639	Down	0	0
mmu-miR-467e-5p	173	8	5.89	0.4	-3.880195729	Down	1.28E-29	2.37E-29
mmu-miR-450b-3p	274	13	9.33	0.64	-3.865733271	Down	7.49E-46	1.58E-45
mmu-miR-335-5p	308	15	10.49	0.74	-3.825345597	Down	5.19E-51	1.16E-50
mmu-miR-7043-3p	682	33	23.23	1.64	-3.824221434	Down	2.08E-111	9.15E-111
mmu-miR-466f-3p	692	34	23.57	1.69	-3.801856607	Down	1.55E-112	6.91E-112
mmu-miR-434-3p	6211	306	211.52	15.17	-3.801501091	Down	0	0
novel_mir773	465	23	15.84	1.14	-3.796466606	Down	5.74E-76	1.77E-75
mmu-miR-19a-3p	322	16	10.97	0.79	-3.795567062	Down	5.68E-53	1.30E-52
mmu-miR-6956-3p	301	15	10.25	0.74	-3.791954829	Down	1.44E-49	3.21E-49
mmu-miR-467d-5p	357	18	12.16	0.89	-3.772194082	Down	2.44E-58	6.05E-58
mmu-miR-484	326990	16685	11135.74	827.33	-3.750590725	Down	0	0
mmu-miR-1306-3p	2633	136	89.67	6.74	-3.733804901	Down	0	0
novel_mir642	195	10	6.64	0.5	-3.731183242	Down	2.57E-32	4.81E-32
mmu-miR-152-3p	385	20	13.11	0.99	-3.72709535	Down	2.89E-62	7.53E-62
mmu-miR-466g	345	18	11.75	0.89	-3.72271161	Down	7.15E-56	1.72E-55
mmu-miR-200b-3p	722	38	24.59	1.88	-3.709267168	Down	5.07E-115	2.33E-114
mmu-miR-615-3p	894	48	30.45	2.38	-3.677408749	Down	3.44E-141	1.77E-140
mmu-miR-345-5p	206	11	7.02	0.55	-3.673967507	Down	1.12E-33	2.13E-33
mmu-let-7i-5p	632649	34070	21545.05	1689.37	-3.672799205	Down	0	0
mmu-miR-127-3p	91586	5018	3118.99	248.82	-3.647904569	Down	0	0
novel_mir590	345	19	11.75	0.94	-3.64385619	Down	5.62E-55	1.34E-54
mmu-miR-3102-5p.2-5p	470	26	16.01	1.29	-3.633530337	Down	2.85E-74	8.62E-74
mmu-miR-22-3p	649510	36222	22119.26	1796.07	-3.622387637	Down	0	0
mmu-miR-29c-3p	1699	95	57.86	4.71	-3.618767358	Down	1.90E-263	1.26E-262
mmu-miR-215-5p	9705	547	330.51	27.12	-3.607264839	Down	0	0
mmu-miR-297a-5p	1216	69	41.41	3.42	-3.597910972	Down	3.73E-188	2.15E-187
novel_mir619	529	30	18.02	1.49	-3.596214775	Down	9.24E-83	3.11E-82
mmu-miR-421-3p	493	28	16.79	1.39	-3.594445442	Down	3.41E-77	1.08E-76
mmu-miR-100-5p	10567	605	359.86	30	-3.584401344	Down	0	0
mmu-miR-433-3p	741	43	25.23	2.13	-3.566214871	Down	1.85E-114	8.37E-114

mmu-miR-186-5p	978	57	33.31	2.83	-3.557081396	Down	2.43E-150	1.29E-149
mmu-miR-382-5p	7802	467	265.7	23.16	-3.520091069	Down	0	0
mmu-miR-1191a	2116	127	72.06	6.3	-3.515774918	Down	0	0
mmu-miR-300-3p	247	15	8.41	0.74	-3.506508625	Down	1.52E-38	3.00E-38
mmu-miR-126a-3p	16986	1049	578.46	52.01	-3.475356259	Down	0	0
mmu-miR-369-5p	13132	815	447.21	40.41	-3.468168192	Down	0	0
mmu-miR-350-3p	6146	384	209.3	19.04	-3.458466928	Down	0	0
mmu-miR-193b-5p	334	21	11.37	1.04	-3.450576821	Down	4.92E-51	1.11E-50
mmu-miR-361-5p	3892	246	132.54	12.2	-3.441474772	Down	0	0
novel_mir240	188	12	6.4	0.6	-3.415037499	Down	3.61E-29	6.65E-29
mmu-miR-30e-3p	1726	112	58.78	5.55	-3.404765682	Down	2.17E-254	1.43E-253
novel_mir103	416	27	14.17	1.34	-3.402534852	Down	1.63E-62	4.27E-62
mmu-miR-6943-3p	467	31	15.9	1.54	-3.368024509	Down	1.97E-69	5.57E-69
mmu-miR-5100	329	22	11.2	1.09	-3.361098692	Down	3.24E-49	7.15E-49
novel_mir722	445	30	15.15	1.49	-3.345933558	Down	7.36E-66	1.97E-65
mmu-miR-1843b-5p	691	47	23.53	2.33	-3.336099461	Down	4.37E-101	1.80E-100
mmu-miR-8107	484	33	16.48	1.64	-3.328948523	Down	3.34E-71	9.68E-71
mmu-miR-183-5p	6526	448	222.24	22.21	-3.322837206	Down	0	0
mmu-miR-669b-5p	2447	170	83.33	8.43	-3.305231444	Down	0	0
mmu-miR-106b-5p	1730	122	58.92	6.05	-3.283750383	Down	4.05E-247	2.63E-246
mmu-miR-34b-3p	1882	134	64.09	6.64	-3.270844123	Down	1.16E-267	7.79E-267
mmu-miR-122-5p	219616	16164	7479.09	801.49	-3.222106319	Down	0	0
mmu-miR-340-5p	622	46	21.18	2.28	-3.21559686	Down	3.02E-88	1.09E-87
mmu-miR-15b-5p	7680	580	261.54	28.76	-3.184896029	Down	0	0
mmu-miR-15b-3p	3374	255	114.9	12.64	-3.184310429	Down	0	0
mmu-miR-30e-5p	659	50	22.44	2.48	-3.17766065	Down	1.95E-92	7.48E-92
mmu-miR-337-5p	340	26	11.58	1.29	-3.166192283	Down	2.87E-48	6.23E-48
mmu-miR-339-3p	1973	151	67.19	7.49	-3.165208906	Down	2.12E-272	1.45E-271
mmu-miR-143-3p	42639	3312	1452.08	164.23	-3.144331344	Down	0	0
mmu-miR-32-5p	976	76	33.24	3.77	-3.140283954	Down	1.20E-134	6.05E-134
mmu-miR-103-3p	467	37	15.9	1.83	-3.119111212	Down	8.81E-65	2.33E-64
mmu-miR-434-5p	1009	80	34.36	3.97	-3.113519124	Down	4.45E-138	2.29E-137
novel_mir355	416	33	14.17	1.64	-3.111072038	Down	7.68E-58	1.87E-57
mmu-miR-7687-3p	400	32	13.62	1.59	-3.098628033	Down	1.78E-55	4.26E-55
mmu-miR-196a-5p	1985	160	67.6	7.93	-3.091630475	Down	5.18E-268	3.51E-267
mmu-miR-181c-5p	10624	860	361.8	42.64	-3.084913065	Down	0	0

novel_mir39	196	16	6.67	0.79	-3.077762203	Down	1.15E-27	2.08E-27
mmu-let-7f-2-3p	4574	372	155.77	18.45	-3.077724688	Down	0	0
mmu-miR-23b-3p	3461	283	117.87	14.03	-3.07060966	Down	0	0
mmu-miR-8112	2833	234	96.48	11.6	-3.056105102	Down	0	0
mmu-miR-24-2-5p	2114	175	71.99	8.68	-3.052029571	Down	6.04E-282	4.20E-281
mmu-miR-3074-5p	826812	68615	28157.33	3402.28	-3.048936756	Down	0	0
mmu-miR-1964-3p	971	81	33.07	4.02	-3.040255639	Down	5.29E-130	2.63E-129
mmu-let-7e-3p	2271	190	77.34	9.42	-3.037415799	Down	2.69E-301	1.89E-300
mmu-miR-1231-3p	24	2	0.82	0.1	-3.03562391	Down	0.000273976	0.00032243
mmu-miR-376a-3p	572	48	19.48	2.38	-3.032960199	Down	4.82E-77	1.51E-76
novel_mir387	642	54	21.86	2.68	-3.027988495	Down	3.45E-86	1.20E-85
mmu-miR-17-3p	404	35	13.76	1.74	-2.983321259	Down	4.40E-54	1.03E-53
mmu-miR-487b-3p	977	85	33.27	4.21	-2.982329728	Down	2.67E-128	1.32E-127
mmu-miR-224-5p	2979	261	101.45	12.94	-2.970859343	Down	0	0
mmu-miR-5113	262	23	8.92	1.14	-2.968009886	Down	2.27E-35	4.36E-35
mmu-miR-486b-3p	41684	3704	1419.56	183.66	-2.950334472	Down	0	0
mmu-miR-485-5p	11334	1009	385.98	50.03	-2.947660737	Down	0	0
mmu-miR-130b-5p	899	80	30.62	3.97	-2.94726337	Down	6.13E-117	2.86E-116
mmu-miR-215-3p	1648	148	56.12	7.34	-2.934663041	Down	5.48E-212	3.42E-211
mmu-miR-196b-5p	203113	18457	6917.07	915.19	-2.918017863	Down	0	0
mmu-miR-203-3p	1240	113	42.23	5.6	-2.914769515	Down	8.22E-159	4.45E-158
mmu-miR-223-3p	43758	4015	1490.19	199.08	-2.90407609	Down	0	0
mmu-miR-451a	1416703	131155	48246.25	6503.34	-2.891164055	Down	0	0
mmu-miR-99a-5p	95499	8924	3252.25	442.5	-2.877688801	Down	0	0
mmu-miR-6395	32	3	1.09	0.15	-2.861293729	Down	3.53E-05	4.36E-05
mmu-miR-467c-5p	273	26	9.3	1.29	-2.849859651	Down	2.08E-35	4.01E-35
mmu-miR-3084-3p	313	30	10.66	1.49	-2.838823202	Down	2.94E-40	5.89E-40
mmu-miR-27b-3p	301568	29054	10269.99	1440.65	-2.833642991	Down	0	0
mmu-miR-155-5p	207	20	7.05	0.99	-2.832122827	Down	5.39E-27	9.66E-27
mmu-miR-3087-3p	328	32	11.17	1.59	-2.812530515	Down	9.55E-42	1.95E-41
mmu-miR-221-3p	3918	386	133.43	19.14	-2.801420339	Down	0	0
novel_mir2	856	86	29.15	4.26	-2.774570548	Down	7.98E-105	3.36E-104
mmu-miR-7a-5p	10399	1045	354.14	51.82	-2.772738884	Down	0	0
mmu-miR-7075-3p	338	34	11.51	1.69	-2.767792682	Down	2.63E-42	5.41E-42
mmu-miR-222-5p	1087	110	37.02	5.45	-2.76397676	Down	3.84E-132	1.93E-131
mmu-miR-328-3p	615598	63078	20964.38	3127.73	-2.744752282	Down	0	0

mmu-miR-132-5p	642	66	21.86	3.27	-2.74093086	Down	4.61E-78	1.47E-77
mmu-miR-148a-3p	4641	486	158.05	24.1	-2.713275984	Down	0	0
mmu-miR-15a-5p	3532	370	120.28	18.35	-2.712544805	Down	0	0
mmu-miR-423-3p	96384	10155	3282.39	503.54	-2.704568372	Down	0	0
mmu-let-7a-1-3p	4541	479	154.65	23.75	-2.703007415	Down	0	0
mmu-miR-374b-5p	960	102	32.69	5.06	-2.691640087	Down	8.12E-114	3.66E-113
novel_mir308	276	30	9.4	1.49	-2.657348426	Down	2.11E-33	4.00E-33
mmu-miR-130a-3p	6553	717	223.16	35.55	-2.650156992	Down	0	0
mmu-miR-7070-3p	45	5	1.53	0.25	-2.613531653	Down	2.22E-06	2.89E-06
novel_mir339	979	110	33.34	5.45	-2.612925969	Down	2.36E-112	1.04E-111
novel_mir8	204	23	6.95	1.14	-2.607979153	Down	1.50E-24	2.62E-24
mmu-miR-598-3p	213	24	7.25	1.19	-2.607019422	Down	1.39E-25	2.46E-25
mmu-miR-149-5p	15203	1723	517.74	85.44	-2.599244231	Down	0	0
mmu-miR-125b-1-3p	376	43	12.8	2.13	-2.587218475	Down	1.12E-43	2.31E-43
mmu-miR-92a-3p	1542701	177498	52537.15	8801.26	-2.577555958	Down	0	0
mmu-miR-409-3p	4007	462	136.46	22.91	-2.574428758	Down	0	0
mmu-miR-193b-3p	5550	641	189.01	31.78	-2.572271536	Down	0	0
mmu-miR-26a-5p	32901	3869	1120.45	191.84	-2.546102801	Down	0	0
mmu-miR-30d-5p	59416	7035	2023.43	348.83	-2.536206915	Down	0	0
mmu-miR-101a-3p	601	72	20.47	3.57	-2.519515123	Down	5.78E-67	1.58E-66
mmu-miR-499-5p	1176	143	40.05	7.09	-2.49794471	Down	3.28E-128	1.61E-127
mmu-miR-29a-3p	43946	5354	1496.59	265.48	-2.495003956	Down	0	0
mmu-miR-128-3p	90942	11119	3097.06	551.34	-2.489885154	Down	0	0
mmu-miR-218-5p	2785	343	94.84	17.01	-2.479112522	Down	1.24E-298	8.69E-298
mmu-miR-28a-3p	6689	826	227.8	40.96	-2.475480127	Down	0	0
mmu-miR-1247-5p	151	19	5.14	0.94	-2.451035698	Down	2.23E-17	3.59E-17
mmu-miR-27a-5p	262	33	8.92	1.64	-2.443347895	Down	3.99E-29	7.34E-29
mmu-miR-27a-3p	898139	114286	30586.4	5666.89	-2.432261209	Down	0	0
mmu-miR-150-5p	141786	18076	4828.57	896.3	-2.429542391	Down	0	0
mmu-miR-1934-5p	453	58	15.43	2.88	-2.421597345	Down	9.50E-49	2.08E-48
mmu-miR-16-5p	117675	15122	4007.46	749.83	-2.418052668	Down	0	0
mmu-miR-1839-5p	6981	902	237.74	44.73	-2.410069995	Down	0	0
mmu-miR-323-3p	376	49	12.8	2.43	-2.397115591	Down	2.99E-40	6.00E-40
mmu-miR-341-3p	7007	920	238.63	45.62	-2.387037075	Down	0	0
mmu-miR-326-3p	7949	1046	270.71	51.87	-2.383775907	Down	0	0
mmu-miR-30a-3p	7899	1053	269	52.21	-2.365208109	Down	0	0

mmu-miR-204-3p	836	114	28.47	5.65	-2.33311972	Down	7.91E-85	2.72E-84
mmu-miR-125a-3p	191	26	6.5	1.29	-2.333068652	Down	1.49E-20	2.49E-20
mmu-let-7i-3p	3059	418	104.18	20.73	-2.329286321	Down	2.00E-305	1.42E-304
mmu-miR-379-5p	10898	1492	371.13	73.98	-2.326717419	Down	0	0
mmu-miR-872-3p	5362	738	182.6	36.59	-2.319165445	Down	0	0
mmu-miR-191-5p	701318	96872	23883.6	4803.41	-2.31388945	Down	0	0
mmu-miR-192-5p	59308	8248	2019.75	408.98	-2.304074531	Down	0	0
mmu-miR-7a-1-3p	5153	732	175.49	36.3	-2.27334737	Down	0	0
mmu-miR-3074-2-3p	1162	167	39.57	8.28	-2.256704392	Down	8.63E-113	3.86E-112
novel_mir50	6062	878	206.44	43.54	-2.245309223	Down	0	0
mmu-miR-140-5p	3461	506	117.87	25.09	-2.232012198	Down	0	0
mmu-miR-128-1-5p	54	8	1.84	0.4	-2.201633861	Down	2.38E-06	3.10E-06
mmu-miR-28a-5p	235	35	8	1.74	-2.200912694	Down	2.01E-23	3.46E-23
mmu-miR-1198-3p	391	59	13.32	2.93	-2.184621513	Down	1.27E-37	2.50E-37
mmu-miR-676-3p	12346	1897	420.45	94.06	-2.160281008	Down	0	0
novel_mir168	7485	1156	254.9	57.32	-2.152820859	Down	0	0
novel_mir330	483	76	16.45	3.77	-2.125451155	Down	1.34E-44	2.79E-44
mmu-miR-22-5p	1438	228	48.97	11.31	-2.114299267	Down	1.14E-128	5.64E-128
mmu-miR-425-3p	14377	2291	489.61	113.6	-2.10767019	Down	0	0
novel_mir705	570	91	19.41	4.51	-2.105600779	Down	9.98E-52	2.25E-51
mmu-miR-96-5p	733	117	24.96	5.8	-2.105493129	Down	4.60E-66	1.24E-65
mmu-miR-99b-5p	22541	3599	767.64	178.46	-2.104829144	Down	0	0
mmu-miR-21a-5p	199248	32041	6785.45	1588.76	-2.09454329	Down	0	0
mmu-miR-8116	5084	823	173.14	40.81	-2.084944449	Down	0	0
mmu-let-7j	148	24	5.04	1.19	-2.08246216	Down	2.57E-14	3.99E-14
mmu-miR-20a-5p	740	121	25.2	6	-2.070389328	Down	2.47E-65	6.57E-65
mmu-miR-185-5p	6895	1141	234.81	56.58	-2.053129769	Down	0	0
mmu-miR-652-3p	79718	13339	2714.82	661.42	-2.037217971	Down	0	0
mmu-miR-486a-3p	10018	1755	341.17	87.02	-1.97107187	Down	0	0
mmu-miR-200a-3p	871	159	29.66	7.88	-1.912251063	Down	2.76E-69	7.78E-69
mmu-miR-411-5p	2483	458	84.56	22.71	-1.896647671	Down	3.18E-192	1.86E-191
novel_mir579	223	42	7.59	2.08	-1.867516357	Down	2.50E-18	4.05E-18
mmu-let-7k	497155	94392	16930.76	4680.44	-1.854930669	Down	0	0
mmu-miR-214-5p	193	37	6.57	1.83	-1.844049722	Down	8.74E-16	1.38E-15
mmu-miR-301a-3p	606	118	20.64	5.85	-1.818934441	Down	1.10E-45	2.30E-45
mmu-miR-187-3p	1259	256	42.88	12.69	-1.756612837	Down	7.12E-89	2.59E-88

mmu-miR-182-5p	496	102	16.89	5.06	-1.738960038	Down	1.41E-35	2.71E-35
mmu-miR-24-3p	1965736	406005	66943.73	20131.81	-1.733472051	Down	0	0
mmu-miR-191-3p	1938	425	66	21.07	-1.64727571	Down	1.97E-124	9.43E-124
mmu-miR-375-3p	107106	24305	3647.53	1205.17	-1.597683178	Down	0	0
mmu-miR-23a-3p	590879	135159	20122.56	6701.88	-1.586176097	Down	0	0
mmu-miR-139-5p	81212	18953	2765.7	939.79	-1.557234352	Down	0	0
mmu-miR-130b-3p	304	71	10.35	3.52	-1.555983434	Down	2.28E-19	3.73E-19
novel_mir340	418	98	14.24	4.86	-1.550920927	Down	5.95E-26	1.06E-25
mmu-miR-378a-5p	264	62	8.99	3.07	-1.55008246	Down	6.77E-17	1.08E-16
mmu-miR-151-5p	15744	3755	536.17	186.19	-1.525914909	Down	0	0
mmu-miR-7a-2-3p	384	92	13.08	4.56	-1.520256811	Down	2.58E-23	4.43E-23
mmu-miR-532-5p	1178	285	40.12	14.13	-1.50556014	Down	2.46E-67	6.75E-67
mmu-miR-154-5p	6444	1560	219.45	77.35	-1.504419073	Down	0	0
mmu-miR-223-5p	3003	744	102.27	36.89	-1.471081311	Down	2.59E-163	1.42E-162
novel_mir415	278	70	9.47	3.47	-1.448428763	Down	3.31E-16	5.23E-16
mmu-miR-322-5p	10984	2768	374.06	137.25	-1.446463551	Down	0	0
mmu-miR-429-3p	49649	12590	1690.81	624.28	-1.437449398	Down	0	0
mmu-miR-425-5p	10249	2611	349.03	129.47	-1.430733201	Down	0	0
mmu-miR-339-5p	2081	532	70.87	26.38	-1.425730484	Down	1.03E-108	4.49E-108
mmu-miR-669o-5p	176	45	5.99	2.23	-1.425512293	Down	1.49E-10	2.17E-10
mmu-miR-181d-5p	7179	1876	244.48	93.02	-1.394103604	Down	0	0
mmu-miR-431-5p	1363	361	46.42	17.9	-1.374786935	Down	4.06E-68	1.14E-67
mmu-miR-543-5p	585	159	19.92	7.88	-1.337950113	Down	5.12E-29	9.38E-29
mmu-miR-1198-5p	252063	68565	8584.08	3399.8	-1.336213642	Down	0	0
mmu-miR-338-5p	418	115	14.24	5.7	-1.320915322	Down	8.81E-21	1.48E-20
mmu-miR-34a-5p	5305	1459	180.66	72.34	-1.320411611	Down	1.70E-244	1.10E-243
mmu-let-7c-2-3p	245	69	8.34	3.42	-1.286051059	Down	2.47E-12	3.73E-12
mmu-miR-30a-5p	2990	851	101.83	42.2	-1.27084775	Down	6.57E-131	3.28E-130
mmu-miR-146a-5p	159456	45988	5430.32	2280.32	-1.251800923	Down	0	0
mmu-miR-150-3p	907	271	30.89	13.44	-1.200606733	Down	1.25E-37	2.46E-37
mmu-miR-205-5p	705	212	24.01	10.51	-1.191872734	Down	2.86E-29	5.28E-29
mmu-miR-25-3p	109617	33471	3733.04	1659.66	-1.169463248	Down	0	0
mmu-miR-202-5p	425	134	14.47	6.64	-1.123809775	Down	9.20E-17	1.46E-16
mmu-miR-423-5p	3566	1130	121.44	56.03	-1.115972298	Down	1.99E-127	9.71E-127
mmu-miR-138-5p	510	174	17.37	8.63	-1.00916529	Down	6.27E-17	1.00E-16
