# Supplementary material for: Pathogenic Effects and Potential Regulatory Mechanisms of Tea Polyphenols on Obesity
Source: Biomed Res Int. 2019 Jun 11;2019:2579734. doi: 10.1155/2019/2579734 (PMC6595166; doi:10.1155/2019/2579734)
Supplement: Supplementary 2 — Supplementary Material S2. Differentially expressed microRNAs in the control group vs. high-dose group. [file 2579734.f2.pdf]

miRNA id	Count (DUI4)	Count (XIA01)	TPM (DUI4)	TPM (XIA01)	log2 Ratio(XIA01/DUI4)	Up-Down-Regulation (XIA01/DUI4)	P-value	FDR
mmu-miR-700-3p	0	351	0.001	8.79	13.10164745	Up	1.16E-84	2.87E-84
novel_mir626	0	294	0.001	7.36	12.84549005	Up	5.05E-71	1.16E-70
mmu-miR-669e-5p	0	172	0.001	4.31	12.07347215	Up	7.94E-42	1.62E-41
mmu-miR-6961-5p	0	162	0.001	4.06	11.98726401	Up	1.96E-39	3.96E-39
mmu-miR-5114	0	156	0.001	3.91	11.93295289	Up	5.36E-38	1.08E-37
mmu-miR-1930-5p	0	127	0.001	3.18	11.63481105	Up	4.67E-31	9.09E-31
mmu-miR-376b-5p	0	110	0.001	2.75	11.4252159	Up	5.46E-27	1.05E-26
mmu-miR-7058-5p	0	106	0.001	2.65	11.37177664	Up	4.95E-26	9.48E-26
mmu-miR-490-5p	0	93	0.001	2.33	11.18611424	Up	6.39E-23	1.20E-22
mmu-miR-7b-5p	0	92	0.001	2.3	11.16741815	Up	1.11E-22	2.08E-22
novel_mir248	0	91	0.001	2.28	11.15481811	Up	1.92E-22	3.60E-22
mmu-miR-129-5p	0	91	0.001	2.28	11.15481811	Up	1.92E-22	3.59E-22
novel_mir18	0	80	0.001	2	10.96578428	Up	8.25E-20	1.53E-19
mmu-miR-744-3p	0	80	0.001	2	10.96578428	Up	8.25E-20	1.53E-19
mmu-miR-671-5p	0	76	0.001	1.9	10.8917837	Up	7.48E-19	1.38E-18
mmu-miR-5134-3p	0	76	0.001	1.9	10.8917837	Up	7.48E-19	1.38E-18
novel_mir479	0	75	0.001	1.88	10.87651695	Up	1.30E-18	2.38E-18
novel_mir426	0	72	0.001	1.8	10.81378119	Up	6.78E-18	1.24E-17
novel_mir397	0	67	0.001	1.68	10.71424552	Up	1.07E-16	1.93E-16
mmu-miR-133b-3p	0	67	0.001	1.68	10.71424552	Up	1.07E-16	1.93E-16
mmu-miR-181c-3p	0	63	0.001	1.58	10.62570884	Up	9.66E-16	1.74E-15
mmu-miR-466h-5p	0	63	0.001	1.58	10.62570884	Up	9.66E-16	1.73E-15
mmu-miR-674-5p	0	60	0.001	1.5	10.55074679	Up	5.05E-15	9.00E-15
mmu-miR-328-5p	0	57	0.001	1.43	10.48179943	Up	2.64E-14	4.68E-14
mmu-miR-672-5p	0	55	0.001	1.38	10.43045255	Up	7.93E-14	1.40E-13
mmu-miR-496a-3p	0	48	0.001	1.2	10.22881869	Up	3.76E-12	6.59E-12
novel_mir22	0	46	0.001	1.15	10.16741815	Up	1.13E-11	1.98E-11
mmu-miR-7024-5p	0	45	0.001	1.13	10.14210706	Up	1.96E-11	3.42E-11
mmu-miR-483-5p	0	43	0.001	1.08	10.0768156	Up	5.91E-11	1.03E-10
mmu-miR-195a-3p	0	43	0.001	1.08	10.0768156	Up	5.91E-11	1.02E-10
mmu-miR-6905-5p	0	41	0.001	1.03	10.00842862	Up	1.78E-10	3.08E-10
novel_mir157	0	39	0.001	0.98	9.936637939	Up	5.35E-10	9.21E-10
mmu-miR-7091-5p	0	39	0.001	0.98	9.936637939	Up	5.35E-10	9.19E-10

novel_mir506	0	38	0.001	0.95	9.891783703	Up	9.29E-10	1.59E-09
mmu-miR-709	0	37	0.001	0.93	9.861086906	Up	1.61E-09	2.75E-09
mmu-miR-9-3p	0	37	0.001	0.93	9.861086906	Up	1.61E-09	2.75E-09
novel_mir540	0	37	0.001	0.93	9.861086906	Up	1.61E-09	2.75E-09
novel_mir287	0	37	0.001	0.93	9.861086906	Up	1.61E-09	2.74E-09
novel_mir703	0	36	0.001	0.9	9.813781191	Up	2.80E-09	4.73E-09
novel_mir221	0	36	0.001	0.9	9.813781191	Up	2.80E-09	4.72E-09
novel_mir707	0	36	0.001	0.9	9.813781191	Up	2.80E-09	4.71E-09
mmu-miR-125b-2-3p	0	35	0.001	0.88	9.781359714	Up	4.85E-09	8.17E-09
mmu-miR-664-5p	0	34	0.001	0.85	9.731319031	Up	8.41E-09	1.41E-08
mmu-miR-99a-3p	0	34	0.001	0.85	9.731319031	Up	8.41E-09	1.41E-08
novel_mir161	0	33	0.001	0.83	9.696967526	Up	1.46E-08	2.44E-08
novel_mir446	0	32	0.001	0.8	9.64385619	Up	2.53E-08	4.22E-08
mmu-miR-6546-3p	0	32	0.001	0.8	9.64385619	Up	2.53E-08	4.21E-08
mmu-miR-669d-5p	0	31	0.001	0.78	9.607330314	Up	4.40E-08	7.30E-08
mmu-miR-3068-5p	0	30	0.001	0.75	9.550746785	Up	7.63E-08	1.26E-07
novel_mir11	0	30	0.001	0.75	9.550746785	Up	7.63E-08	1.26E-07
mmu-miR-351-3p	0	30	0.001	0.75	9.550746785	Up	7.63E-08	1.26E-07
novel_mir715	0	30	0.001	0.75	9.550746785	Up	7.63E-08	1.26E-07
mmu-miR-669h-5p	0	29	0.001	0.73	9.511752654	Up	1.32E-07	2.17E-07
mmu-miR-6900-3p	0	28	0.001	0.7	9.451211112	Up	2.30E-07	3.75E-07
mmu-miR-344-3p	0	28	0.001	0.7	9.451211112	Up	2.30E-07	3.74E-07
mmu-miR-7655-3p	0	27	0.001	0.68	9.409390936	Up	3.98E-07	6.47E-07
mmu-miR-340-3p	0	27	0.001	0.68	9.409390936	Up	3.98E-07	6.46E-07
novel_mir517	0	27	0.001	0.68	9.409390936	Up	3.98E-07	6.45E-07
novel_mir389	0	27	0.001	0.68	9.409390936	Up	3.98E-07	6.44E-07
mmu-miR-6952-5p	0	27	0.001	0.68	9.409390936	Up	3.98E-07	6.43E-07
novel_mir334	0	27	0.001	0.68	9.409390936	Up	3.98E-07	6.43E-07
mmu-miR-210-5p	0	27	0.001	0.68	9.409390936	Up	3.98E-07	6.42E-07
novel_mir536	0	26	0.001	0.65	9.344295908	Up	6.91E-07	1.11E-06
novel_mir92	0	26	0.001	0.65	9.344295908	Up	6.91E-07	1.11E-06
novel_mir451	0	26	0.001	0.65	9.344295908	Up	6.91E-07	1.11E-06
novel_mir357	0	25	0.001	0.63	9.299208018	Up	1.20E-06	1.91E-06
mmu-miR-6904-5p	0	25	0.001	0.63	9.299208018	Up	1.20E-06	1.91E-06
novel_mir756	0	25	0.001	0.63	9.299208018	Up	1.20E-06	1.91E-06
mmu-miR-23a-5p	0	24	0.001	0.6	9.22881869	Up	2.08E-06	3.30E-06

mmu-miR-6915-5p	0	24	0.001	0.6	9.22881869	Up	2.08E-06	3.30E-06
mmu-miR-5107-5p	0	24	0.001	0.6	9.22881869	Up	2.08E-06	3.29E-06
mmu-miR-671-3p	0	24	0.001	0.6	9.22881869	Up	2.08E-06	3.29E-06
mmu-miR-3080-5p	0	23	0.001	0.58	9.17990909	Up	3.61E-06	5.67E-06
novel_mir646	0	23	0.001	0.58	9.17990909	Up	3.61E-06	5.67E-06
novel_mir14	0	23	0.001	0.58	9.17990909	Up	3.61E-06	5.66E-06
novel_mir63	0	23	0.001	0.58	9.17990909	Up	3.61E-06	5.65E-06
mmu-miR-6984-5p	0	22	0.001	0.55	9.103287808	Up	6.26E-06	9.78E-06
mmu-miR-7687-5p	0	22	0.001	0.55	9.103287808	Up	6.26E-06	9.76E-06
mmu-miR-18b-5p	0	22	0.001	0.55	9.103287808	Up	6.26E-06	9.75E-06
novel_mir174	0	22	0.001	0.55	9.103287808	Up	6.26E-06	9.74E-06
mmu-miR-1943-3p	0	22	0.001	0.55	9.103287808	Up	6.26E-06	9.72E-06
mmu-miR-6991-3p	0	22	0.001	0.55	9.103287808	Up	6.26E-06	9.71E-06
novel_mir16	0	21	0.001	0.53	9.049848549	Up	1.09E-05	1.68E-05
novel_mir513	0	21	0.001	0.53	9.049848549	Up	1.09E-05	1.68E-05
novel_mir28	0	21	0.001	0.53	9.049848549	Up	1.09E-05	1.67E-05
mmu-miR-201-5p	0	21	0.001	0.53	9.049848549	Up	1.09E-05	1.67E-05
mmu-miR-7689-3p	0	21	0.001	0.53	9.049848549	Up	1.09E-05	1.67E-05
mmu-miR-5103	0	21	0.001	0.53	9.049848549	Up	1.09E-05	1.67E-05
novel_mir17	0	21	0.001	0.53	9.049848549	Up	1.09E-05	1.66E-05
mmu-miR-6933-5p	0	21	0.001	0.53	9.049848549	Up	1.09E-05	1.66E-05
mmu-miR-7672-5p	0	21	0.001	0.53	9.049848549	Up	1.09E-05	1.66E-05
novel_mir170	0	20	0.001	0.5	8.965784285	Up	1.89E-05	2.86E-05
mmu-miR-669f-5p	0	20	0.001	0.5	8.965784285	Up	1.89E-05	2.86E-05
mmu-miR-8114	0	20	0.001	0.5	8.965784285	Up	1.89E-05	2.86E-05
novel_mir70	0	20	0.001	0.5	8.965784285	Up	1.89E-05	2.85E-05
mmu-miR-3102-5p	0	20	0.001	0.5	8.965784285	Up	1.89E-05	2.85E-05
mmu-miR-3572-5p	0	20	0.001	0.5	8.965784285	Up	1.89E-05	2.84E-05
novel_mir478	0	20	0.001	0.5	8.965784285	Up	1.89E-05	2.84E-05
mmu-miR-7667-5p	0	20	0.001	0.5	8.965784285	Up	1.89E-05	2.84E-05
novel_mir456	0	20	0.001	0.5	8.965784285	Up	1.89E-05	2.83E-05
novel_mir94	0	20	0.001	0.5	8.965784285	Up	1.89E-05	2.83E-05
mmu-miR-203-5p	0	19	0.001	0.48	8.906890596	Up	3.27E-05	4.89E-05
mmu-miR-7003-5p	0	19	0.001	0.48	8.906890596	Up	3.27E-05	4.89E-05
mmu-miR-219a-5p	0	19	0.001	0.48	8.906890596	Up	3.27E-05	4.88E-05
novel_mir537	0	19	0.001	0.48	8.906890596	Up	3.27E-05	4.87E-05

mmu-miR-7679-3p	0	19	0.001	0.48	8.906890596	Up	3.27E-05	4.87E-05
mmu-miR-219b-5p	0	19	0.001	0.48	8.906890596	Up	3.27E-05	4.86E-05
novel_mir524	0	19	0.001	0.48	8.906890596	Up	3.27E-05	4.85E-05
novel_mir465	0	19	0.001	0.48	8.906890596	Up	3.27E-05	4.85E-05
novel_mir338	0	19	0.001	0.48	8.906890596	Up	3.27E-05	4.84E-05
novel_mir65	0	19	0.001	0.48	8.906890596	Up	3.27E-05	4.84E-05
novel_mir656	0	19	0.001	0.48	8.906890596	Up	3.27E-05	4.83E-05
novel_mir635	0	19	0.001	0.48	8.906890596	Up	3.27E-05	4.82E-05
mmu-miR-7669-3p	0	19	0.001	0.48	8.906890596	Up	3.27E-05	4.82E-05
mmu-miR-6993-5p	0	19	0.001	0.48	8.906890596	Up	3.27E-05	4.81E-05
novel_mir572	0	18	0.001	0.45	8.813781191	Up	5.68E-05	8.32E-05
novel_mir570	0	18	0.001	0.45	8.813781191	Up	5.68E-05	8.31E-05
mmu-miR-138-2-3p	0	18	0.001	0.45	8.813781191	Up	5.68E-05	8.30E-05
novel_mir444	0	18	0.001	0.45	8.813781191	Up	5.68E-05	8.29E-05
novel_mir421	0	18	0.001	0.45	8.813781191	Up	5.68E-05	8.28E-05
mmu-miR-142b	0	18	0.001	0.45	8.813781191	Up	5.68E-05	8.27E-05
novel_mir460	0	18	0.001	0.45	8.813781191	Up	5.68E-05	8.26E-05
mmu-miR-361-3p	0	18	0.001	0.45	8.813781191	Up	5.68E-05	8.25E-05
novel_mir530	0	18	0.001	0.45	8.813781191	Up	5.68E-05	8.24E-05
mmu-miR-3473a	0	18	0.001	0.45	8.813781191	Up	5.68E-05	8.23E-05
novel_mir768	0	17	0.001	0.43	8.74819285	Up	9.85E-05	0.00014236
mmu-miR-1191b-3p	0	17	0.001	0.43	8.74819285	Up	9.85E-05	0.000142175
mmu-miR-7091-3p	0	17	0.001	0.43	8.74819285	Up	9.85E-05	0.000141991
novel_mir769	0	17	0.001	0.43	8.74819285	Up	9.85E-05	0.000141807
mmu-miR-1968-5p	0	17	0.001	0.43	8.74819285	Up	9.85E-05	0.000141624
mmu-miR-674-3p	0	17	0.001	0.43	8.74819285	Up	9.85E-05	0.000141441
novel_mir238	0	17	0.001	0.43	8.74819285	Up	9.85E-05	0.000141259
novel_mir119	0	17	0.001	0.43	8.74819285	Up	9.85E-05	0.000141077
novel_mir162	0	17	0.001	0.43	8.74819285	Up	9.85E-05	0.000140896
novel_mir571	0	17	0.001	0.43	8.74819285	Up	9.85E-05	0.000140715
mmu-miR-7240-5p	0	16	0.001	0.4	8.64385619	Up	0.000170882	0.000243212
novel_mir42	0	16	0.001	0.4	8.64385619	Up	0.000170882	0.000242901
novel_mir72	0	16	0.001	0.4	8.64385619	Up	0.000170882	0.000242591
mmu-miR-3079-5p	0	16	0.001	0.4	8.64385619	Up	0.000170882	0.000242282
novel_mir767	0	16	0.001	0.4	8.64385619	Up	0.000170882	0.000241974
mmu-miR-30b-3p	0	16	0.001	0.4	8.64385619	Up	0.000170882	0.000241666

mmu-miR-7672-3p	0	16	0.001	0.4	8.64385619	Up	0.000170882	0.00024136
novel_mir668	0	16	0.001	0.4	8.64385619	Up	0.000170882	0.000241054
novel_mir495	0	16	0.001	0.4	8.64385619	Up	0.000170882	0.000240749
mmu-miR-7042-5p	0	16	0.001	0.4	8.64385619	Up	0.000170882	0.000240444
novel_mir6	0	16	0.001	0.4	8.64385619	Up	0.000170882	0.000240141
novel_mir56	0	16	0.001	0.4	8.64385619	Up	0.000170882	0.000239838
mmu-miR-3064-5p	0	16	0.001	0.4	8.64385619	Up	0.000170882	0.000239536
mmu-miR-129b-3p	0	16	0.001	0.4	8.64385619	Up	0.000170882	0.000239235
novel_mir169	0	16	0.001	0.4	8.64385619	Up	0.000170882	0.000238934
novel_mir234	0	16	0.001	0.4	8.64385619	Up	0.000170882	0.000238634
novel_mir189	0	16	0.001	0.4	8.64385619	Up	0.000170882	0.000238335
mmu-miR-7679-5p	0	15	0.001	0.38	8.569855608	Up	0.00029649	0.000412492
novel_mir20	0	15	0.001	0.38	8.569855608	Up	0.00029649	0.000411977
novel_mir124	0	15	0.001	0.38	8.569855608	Up	0.00029649	0.000411463
novel_mir602	0	15	0.001	0.38	8.569855608	Up	0.00029649	0.000410951
mmu-miR-1839-3p	0	15	0.001	0.38	8.569855608	Up	0.00029649	0.00041044
novel_mir468	0	15	0.001	0.38	8.569855608	Up	0.00029649	0.00040993
mmu-miR-5116	0	15	0.001	0.38	8.569855608	Up	0.00029649	0.000409421
novel_mir137	0	15	0.001	0.38	8.569855608	Up	0.00029649	0.000408914
novel_mir575	0	15	0.001	0.38	8.569855608	Up	0.00029649	0.000408408
novel_mir650	0	15	0.001	0.38	8.569855608	Up	0.00029649	0.000407903
mmu-miR-1951	0	15	0.001	0.38	8.569855608	Up	0.00029649	0.000407399
novel_mir130	0	15	0.001	0.38	8.569855608	Up	0.00029649	0.000406897
mmu-miR-488-3p	0	15	0.001	0.38	8.569855608	Up	0.00029649	0.000406396
novel_mir464	0	15	0.001	0.38	8.569855608	Up	0.00029649	0.000405896
novel_mir365	0	15	0.001	0.38	8.569855608	Up	0.00029649	0.000405397
mmu-miR-698-5p	0	15	0.001	0.38	8.569855608	Up	0.00029649	0.0004049
novel_mir403	0	15	0.001	0.38	8.569855608	Up	0.00029649	0.000404404
novel_mir400	0	15	0.001	0.38	8.569855608	Up	0.00029649	0.000403909
novel_mir224	0	15	0.001	0.38	8.569855608	Up	0.00029649	0.000403415
novel_mir681	0	14	0.001	0.35	8.451211112	Up	0.000514426	0.000695694
mmu-miR-466c-5p	0	14	0.001	0.35	8.451211112	Up	0.000514426	0.00069485
novel_mir158	0	14	0.001	0.35	8.451211112	Up	0.000514426	0.000694007
novel_mir542	0	14	0.001	0.35	8.451211112	Up	0.000514426	0.000693167
novel_mir522	0	14	0.001	0.35	8.451211112	Up	0.000514426	0.000692329
mmu-miR-879-3p	0	14	0.001	0.35	8.451211112	Up	0.000514426	0.000691493

novel_mir199	0	14	0.001	0.35	8.451211112	Up	0.000514426	0.000690659
mmu-miR-494-5p	0	14	0.001	0.35	8.451211112	Up	0.000514426	0.000689827
novel_mir663	0	14	0.001	0.35	8.451211112	Up	0.000514426	0.000688997
novel_mir671	0	14	0.001	0.35	8.451211112	Up	0.000514426	0.000688168
novel_mir328	0	14	0.001	0.35	8.451211112	Up	0.000514426	0.000687342
novel_mir45	0	14	0.001	0.35	8.451211112	Up	0.000514426	0.000686518
novel_mir553	0	14	0.001	0.35	8.451211112	Up	0.000514426	0.000685696
novel_mir556	0	14	0.001	0.35	8.451211112	Up	0.000514426	0.000684876
mmu-miR-6957-3p	0	14	0.001	0.35	8.451211112	Up	0.000514426	0.000684058
novel_mir682	0	14	0.001	0.35	8.451211112	Up	0.000514426	0.000683241
novel_mir747	0	14	0.001	0.35	8.451211112	Up	0.000514426	0.000682427
mmu-miR-3572-3p	0	14	0.001	0.35	8.451211112	Up	0.000514426	0.000681614
mmu-miR-103-2-5p	0	14	0.001	0.35	8.451211112	Up	0.000514426	0.000680804
novel_mir648	0	14	0.001	0.35	8.451211112	Up	0.000514426	0.000679995
novel_mir664	0	14	0.001	0.35	8.451211112	Up	0.000514426	0.000679189
mmu-miR-3097-3p	0	14	0.001	0.35	8.451211112	Up	0.000514426	0.000678384
novel_mir402	0	14	0.001	0.35	8.451211112	Up	0.000514426	0.000677581
novel_mir488	0	14	0.001	0.35	8.451211112	Up	0.000514426	0.00067678
mmu-miR-183-3p	0	14	0.001	0.35	8.451211112	Up	0.000514426	0.000675981
novel_mir398	0	14	0.001	0.35	8.451211112	Up	0.000514426	0.000675184
mmu-miR-3057-5p	1	188	0.03	4.71	7.294620749	Up	9.54E-44	1.97E-43
mmu-miR-33-5p	3	608	0.1	15.22	7.249824549	Up	1.05E-139	4.09E-139
mmu-miR-541-5p	1	160	0.03	4.01	7.062495926	Up	4.09E-37	8.16E-37
mmu-miR-7010-5p	1	151	0.03	3.78	6.977279923	Up	5.51E-35	1.09E-34
mmu-miR-7115-5p	3	465	0.1	11.64	6.862947248	Up	7.91E-106	2.32E-105
mmu-miR-202-3p	1	123	0.03	3.08	6.68182404	Up	2.26E-28	4.38E-28
mmu-miR-5622-3p	1	114	0.03	2.85	6.569855608	Up	3.00E-26	5.75E-26
mmu-miR-6691-5p	1	113	0.03	2.83	6.559695742	Up	5.15E-26	9.85E-26
mmu-miR-1943-5p	1	110	0.03	2.75	6.518325308	Up	2.62E-25	4.98E-25
mmu-miR-7651-5p	1	99	0.03	2.48	6.36923381	Up	1.02E-22	1.91E-22
mmu-miR-500-3p	1	98	0.03	2.45	6.351675438	Up	1.75E-22	3.27E-22
mmu-miR-25-5p	1	93	0.03	2.33	6.279223644	Up	2.61E-21	4.86E-21
mmu-miR-1947-5p	2	198	0.07	4.96	6.146841388	Up	1.74E-44	3.60E-44
mmu-miR-200b-5p	1	79	0.03	1.98	6.044394119	Up	5.00E-18	9.15E-18
mmu-miR-700-5p	2	180	0.07	4.51	6.009628701	Up	2.93E-40	5.96E-40
mmu-miR-181a-1-3p	1	69	0.03	1.73	5.849665727	Up	1.09E-15	1.95E-15

mmu-miR-380-3p	1	66	0.03	1.65	5.781359714	Up	5.43E-15	9.68E-15
mmu-miR-1945	1	64	0.03	1.6	5.736965594	Up	1.59E-14	2.82E-14
mmu-miR-3474	1	63	0.03	1.58	5.718818247	Up	2.72E-14	4.81E-14
mmu-miR-99b-3p	9	596	0.31	14.92	5.58883551	Up	3.46E-127	1.23E-126
mmu-miR-30d-3p	3	186	0.1	4.66	5.54225805	Up	3.10E-40	6.28E-40
mmu-miR-3074-1-3p	1	52	0.03	1.3	5.437405312	Up	9.72E-12	1.70E-11
mmu-miR-874-3p	2	119	0.07	2.98	5.411813598	Up	5.19E-26	9.91E-26
mmu-miR-29c-5p	3	156	0.1	3.91	5.289096702	Up	2.80E-33	5.50E-33
mmu-miR-675-3p	1	46	0.03	1.15	5.26052755	Up	2.36E-10	4.08E-10
mmu-miR-449a-5p	6	279	0.2	6.98	5.125155131	Up	8.43E-58	1.81E-57
mmu-miR-8097	1	39	0.03	0.98	5.029747343	Up	9.60E-09	1.61E-08
mmu-miR-8096	1	38	0.03	0.95	4.984893108	Up	1.63E-08	2.71E-08
mmu-miR-145a-3p	3	123	0.1	3.08	4.944858446	Up	1.11E-25	2.11E-25
mmu-miR-3473d	2	85	0.07	2.13	4.927354698	Up	3.72E-18	6.82E-18
mmu-miR-505-5p	4	164	0.14	4.11	4.875639662	Up	7.12E-34	1.40E-33
mmu-miR-20b-5p	2	81	0.07	2.03	4.857980995	Up	3.08E-17	5.59E-17
mmu-miR-93-3p	2	73	0.07	1.83	4.708344916	Up	2.07E-15	3.71E-15
mmu-miR-3099-3p	1	30	0.03	0.75	4.64385619	Up	1.08E-06	1.73E-06
mmu-miR-3084-5p	4	139	0.14	3.48	4.635588574	Up	3.61E-28	6.97E-28
mmu-miR-592-5p	1	28	0.03	0.7	4.544320516	Up	3.05E-06	4.81E-06
mmu-miR-3095-3p	2	56	0.07	1.4	4.321928095	Up	1.47E-11	2.57E-11
mmu-miR-128-2-5p	2	55	0.07	1.38	4.301169535	Up	2.47E-11	4.30E-11
mmu-miR-26a-1-3p	1	23	0.03	0.58	4.273018494	Up	4.03E-05	5.92E-05
mmu-miR-324-3p	2	54	0.07	1.35	4.269460675	Up	4.14E-11	7.20E-11
mmu-miR-369-3p	1	21	0.03	0.53	4.142957954	Up	0.000112155	0.000160037
mmu-miR-132-3p	2	46	0.07	1.15	4.038135129	Up	2.53E-09	4.28E-09
mmu-miR-455-5p	4	92	0.14	2.3	4.038135129	Up	1.31E-17	2.39E-17
mmu-miR-7212-5p	1	19	0.03	0.48	4	Up	0.000309914	0.000421165
mmu-miR-200a-5p	1	19	0.03	0.48	4	Up	0.000309914	0.000420652
mmu-miR-491-5p	1	19	0.03	0.48	4	Up	0.000309914	0.000420139
mmu-miR-1934-3p	2	39	0.07	0.98	3.807354922	Up	8.84E-08	1.46E-07
mmu-miR-2137	2	34	0.07	0.85	3.602036014	Up	1.08E-06	1.73E-06
mmu-miR-31-3p	2	32	0.07	0.8	3.514573173	Up	2.93E-06	4.63E-06
mmu-miR-193a-5p	3	42	0.1	1.05	3.392317423	Up	1.30E-07	2.14E-07
mmu-miR-142a-3p	9	106	0.31	2.65	3.095652239	Up	1.87E-16	3.36E-16
mmu-miR-324-5p	2	23	0.07	0.58	3.050626073	Up	0.000234688	0.000326918

mmu-miR-29b-3p	6	66	0.2	1.65	3.044394119	Up	2.06E-10	3.56E-10
mmu-miR-146b-5p	60	558	2.04	13.97	2.775690963	Up	3.24E-72	7.47E-72
mmu-miR-532-5p	1178	7657	40.12	191.67	2.256231034	Up	0	0
mmu-let-7j	148	799	5.04	20	1.988504361	Up	4.66E-70	1.06E-69
mmu-miR-199a-3p	5439	27813	185.23	696.23	1.910246202	Up	0	0
mmu-miR-330-5p	175	855	5.96	21.4	1.844226561	Up	5.97E-68	1.34E-67
novel_mir441	7068	33011	240.7	826.35	1.779516822	Up	0	0
mmu-miR-378c	20	88	0.68	2.2	1.693896872	Up	1.78E-07	2.91E-07
mmu-miR-1188-5p	13	53	0.44	1.33	1.595850817	Up	0.000124177	0.000176964
mmu-miR-184-3p	6266	21891	213.39	547.99	1.360656997	Up	0	0
mmu-miR-219a-2-3p	205	647	6.98	16.2	1.214694872	Up	6.29E-29	1.22E-28
mmu-miR-138-5p	510	1507	17.37	37.72	1.118731922	Up	1.61E-57	3.46E-57
mmu-miR-145a-5p	687	1914	23.4	47.91	1.033818284	Up	3.94E-64	8.66E-64
mmu-miR-320-3p	11186	30999	380.94	775.99	1.026474277	Up	0	0
mmu-miR-3074-5p	826812	28	28157.33	0.7	-15.29579609	Down	0	0
novel_mir2	856	0	29.15	0.001	-14.83120826	Down	0	0
novel_mir727	849	0	28.91	0.001	-14.81928099	Down	0	0
novel_mir589	731	0	24.89	0.001	-14.60327861	Down	1.86E-273	1.17E-272
mmu-miR-342-5p	703	0	23.94	0.001	-14.54713553	Down	5.18E-263	3.17E-262
novel_mir361	663	0	22.58	0.001	-14.46275787	Down	4.30E-248	2.59E-247
novel_mir387	642	0	21.86	0.001	-14.41600578	Down	2.93E-240	1.74E-239
novel_mir201	627	0	21.35	0.001	-14.38194845	Down	1.15E-234	6.79E-234
novel_mir705	570	0	19.41	0.001	-14.2445125	Down	2.10E-213	1.17E-212
novel_mir714	555	0	18.9	0.001	-14.20609861	Down	8.26E-208	4.55E-207
novel_mir580	529	0	18.02	0.001	-14.13731139	Down	4.12E-198	2.24E-197
novel_mir619	529	0	18.02	0.001	-14.13731139	Down	4.12E-198	2.23E-197
novel_mir439	517	0	17.61	0.001	-14.10410729	Down	1.23E-193	6.63E-193
novel_mir147	515	0	17.54	0.001	-14.09836113	Down	6.87E-193	3.67E-192
novel_mir774	510	0	17.37	0.001	-14.08431013	Down	5.03E-191	2.67E-190
novel_mir154	509	0	17.33	0.001	-14.08098403	Down	1.19E-190	6.27E-190
novel_mir57	508	0	17.3	0.001	-14.07848442	Down	2.80E-190	1.47E-189
novel_mir485	498	0	16.96	0.001	-14.04984855	Down	1.51E-186	7.76E-186
novel_mir596	492	0	16.76	0.001	-14.03273453	Down	2.60E-184	1.33E-183
novel_mir121	485	0	16.52	0.001	-14.01192607	Down	1.06E-181	5.38E-181
novel_mir330	483	0	16.45	0.001	-14.00579996	Down	5.92E-181	2.98E-180
novel_mir30	472	0	16.07	0.001	-13.97208231	Down	7.51E-177	3.68E-176

novel_mir518	463	0	15.77	0.001	-13.94489504	Down	1.71E-173	8.26E-173
novel_mir741	462	0	15.73	0.001	-13.94123105	Down	4.03E-173	1.94E-172
novel_mir345	461	0	15.7	0.001	-13.93847694	Down	9.51E-173	4.55E-172
novel_mir755	456	0	15.53	0.001	-13.92277021	Down	6.97E-171	3.30E-170
novel_mir194	456	0	15.53	0.001	-13.92277021	Down	6.97E-171	3.29E-170
novel_mir508	455	0	15.5	0.001	-13.9199806	Down	1.65E-170	7.73E-170
novel_mir686	451	0	15.36	0.001	-13.9068906	Down	5.11E-169	2.38E-168
novel_mir183	442	0	15.05	0.001	-13.87747587	Down	1.16E-165	5.37E-165
novel_mir401	439	0	14.95	0.001	-13.86785786	Down	1.53E-164	7.03E-164
novel_mir709	438	0	14.92	0.001	-13.86495992	Down	3.61E-164	1.65E-163
novel_mir273	438	0	14.92	0.001	-13.86495992	Down	3.61E-164	1.65E-163
novel_mir593	436	0	14.85	0.001	-13.85817531	Down	2.01E-163	9.09E-163
mmu-miR-6919-3p	431	0	14.68	0.001	-13.84156435	Down	1.47E-161	6.61E-161
novel_mir315	426	0	14.51	0.001	-13.8247599	Down	1.08E-159	4.79E-159
novel_mir44	424	0	14.44	0.001	-13.81778312	Down	6.01E-159	2.66E-158
novel_mir600	421	0	14.34	0.001	-13.8077574	Down	7.91E-158	3.48E-157
novel_mir443	417	0	14.2	0.001	-13.79360331	Down	2.46E-156	1.08E-155
novel_mir103	416	0	14.17	0.001	-13.79055214	Down	5.80E-156	2.53E-155
novel_mir516	414	0	14.1	0.001	-13.78340754	Down	3.23E-155	1.40E-154
novel_mir324	412	0	14.03	0.001	-13.77622739	Down	1.80E-154	7.79E-154
novel_mir435	411	0	14	0.001	-13.77313921	Down	4.25E-154	1.83E-153
novel_mir326	411	0	14	0.001	-13.77313921	Down	4.25E-154	1.82E-153
novel_mir533	409	0	13.93	0.001	-13.76590764	Down	2.37E-153	1.01E-152
novel_mir354	406	0	13.83	0.001	-13.75551354	Down	3.11E-152	1.32E-151
novel_mir751	403	0	13.72	0.001	-13.74399286	Down	4.09E-151	1.73E-150
novel_mir368	403	0	13.72	0.001	-13.74399286	Down	4.09E-151	1.72E-150
novel_mir208	402	0	13.69	0.001	-13.74083483	Down	9.66E-151	4.04E-150
novel_mir411	400	0	13.62	0.001	-13.73343908	Down	5.38E-150	2.24E-149
novel_mir592	397	0	13.52	0.001	-13.72280753	Down	7.08E-149	2.94E-148
novel_mir693	397	0	13.52	0.001	-13.72280753	Down	7.08E-149	2.93E-148
novel_mir552	395	0	13.45	0.001	-13.71531855	Down	3.94E-148	1.63E-147
novel_mir472	393	0	13.38	0.001	-13.7077905	Down	2.20E-147	9.02E-147
novel_mir469	392	0	13.35	0.001	-13.70455212	Down	5.19E-147	2.12E-146
mmu-miR-7002-5p	391	0	13.32	0.001	-13.70130646	Down	1.22E-146	4.99E-146
novel_mir432	387	0	13.18	0.001	-13.68606275	Down	3.80E-145	1.54E-144
novel_mir448	386	0	13.15	0.001	-13.68277518	Down	8.97E-145	3.63E-144

novel_mir204	384	0	13.08	0.001	-13.67507492	Down	5.00E-144	2.02E-143
novel_mir680	383	0	13.04	0.001	-13.67065625	Down	1.18E-143	4.74E-143
novel_mir638	381	0	12.98	0.001	-13.66400276	Down	6.57E-143	2.63E-142
novel_mir551	380	0	12.94	0.001	-13.65955	Down	1.55E-142	6.19E-142
novel_mir490	380	0	12.94	0.001	-13.65955	Down	1.55E-142	6.17E-142
novel_mir386	376	0	12.8	0.001	-13.64385619	Down	4.82E-141	1.88E-140
novel_mir69	376	0	12.8	0.001	-13.64385619	Down	4.82E-141	1.87E-140
novel_mir617	365	0	12.43	0.001	-13.60153868	Down	6.11E-137	2.35E-136
novel_mir275	362	0	12.33	0.001	-13.58988518	Down	8.03E-136	3.07E-135
novel_mir274	362	0	12.33	0.001	-13.58988518	Down	8.03E-136	3.06E-135
novel_mir241	360	0	12.26	0.001	-13.58167136	Down	4.47E-135	1.70E-134
novel_mir260	359	0	12.23	0.001	-13.57813678	Down	1.06E-134	4.00E-134
mmu-miR-411-3p	357	0	12.16	0.001	-13.56985561	Down	5.88E-134	2.21E-133
novel_mir213	351	0	11.95	0.001	-13.544723	Down	1.02E-131	3.80E-131
novel_mir293	351	0	11.95	0.001	-13.544723	Down	1.02E-131	3.79E-131
novel_mir418	349	0	11.89	0.001	-13.53746109	Down	5.67E-131	2.10E-130
novel_mir554	348	0	11.85	0.001	-13.53259944	Down	1.34E-130	4.92E-130
novel_mir481	348	0	11.85	0.001	-13.53259944	Down	1.34E-130	4.90E-130
novel_mir350	347	0	11.82	0.001	-13.52894242	Down	3.16E-130	1.15E-129
novel_mir590	345	0	11.75	0.001	-13.52037314	Down	1.76E-129	6.40E-129
novel_mir476	345	0	11.75	0.001	-13.52037314	Down	1.76E-129	6.38E-129
novel_mir235	345	0	11.75	0.001	-13.52037314	Down	1.76E-129	6.36E-129
novel_mir344	343	0	11.68	0.001	-13.51175265	Down	9.81E-129	3.53E-128
novel_mir176	342	0	11.65	0.001	-13.50804233	Down	2.31E-128	8.31E-128
novel_mir573	341	0	11.61	0.001	-13.50308035	Down	5.46E-128	1.96E-127
novel_mir349	337	0	11.48	0.001	-13.48683502	Down	1.70E-126	6.01E-126
novel_mir497	337	0	11.48	0.001	-13.48683502	Down	1.70E-126	5.99E-126
novel_mir190	336	0	11.44	0.001	-13.48179943	Down	4.00E-126	1.41E-125
novel_mir164	336	0	11.44	0.001	-13.48179943	Down	4.00E-126	1.41E-125
novel_mir320	333	0	11.34	0.001	-13.46913302	Down	5.27E-125	1.83E-124
novel_mir694	333	0	11.34	0.001	-13.46913302	Down	5.27E-125	1.82E-124
novel_mir98	330	0	11.24	0.001	-13.45635442	Down	6.93E-124	2.39E-123
mmu-miR-5100	329	0	11.2	0.001	-13.45121111	Down	1.63E-123	5.60E-123
novel_mir228	329	0	11.2	0.001	-13.45121111	Down	1.63E-123	5.58E-123
novel_mir640	329	0	11.2	0.001	-13.45121111	Down	1.63E-123	5.56E-123
novel_mir738	326	0	11.1	0.001	-13.43827206	Down	2.15E-122	7.25E-122

novel_mir599	325	0	11.07	0.001	-13.4343676	Down	5.07E-122	1.71E-121
novel_mir486	324	0	11.03	0.001	-13.42914517	Down	1.20E-121	4.00E-121
novel_mir282	324	0	11.03	0.001	-13.42914517	Down	1.20E-121	3.99E-121
novel_mir316	323	0	11	0.001	-13.4252159	Down	2.83E-121	9.37E-121
novel_mir772	322	0	10.97	0.001	-13.42127591	Down	6.67E-121	2.20E-120
novel_mir606	317	0	10.8	0.001	-13.39874369	Down	4.89E-119	1.61E-118
novel_mir558	317	0	10.8	0.001	-13.39874369	Down	4.89E-119	1.60E-118
novel_mir304	316	0	10.76	0.001	-13.39339046	Down	1.15E-118	3.77E-118
novel_mir698	316	0	10.76	0.001	-13.39339046	Down	1.15E-118	3.76E-118
novel_mir247	314	0	10.69	0.001	-13.38397423	Down	6.43E-118	2.09E-117
novel_mir177	314	0	10.69	0.001	-13.38397423	Down	6.43E-118	2.08E-117
mmu-miR-6952-3p	313	0	10.66	0.001	-13.37991982	Down	1.52E-117	4.90E-117
novel_mir75	312	0	10.63	0.001	-13.37585398	Down	3.58E-117	1.15E-116
novel_mir226	312	0	10.63	0.001	-13.37585398	Down	3.58E-117	1.15E-116
novel_mir64	307	0	10.45	0.001	-13.35121532	Down	2.63E-115	8.33E-115
novel_mir181	306	0	10.42	0.001	-13.34706766	Down	6.20E-115	1.96E-114
novel_mir140	306	0	10.42	0.001	-13.34706766	Down	6.20E-115	1.95E-114
novel_mir182	306	0	10.42	0.001	-13.34706766	Down	6.20E-115	1.95E-114
novel_mir341	303	0	10.32	0.001	-13.33315535	Down	8.15E-114	2.55E-113
novel_mir419	301	0	10.25	0.001	-13.32333629	Down	4.54E-113	1.42E-112
novel_mir740	297	0	10.11	0.001	-13.30349538	Down	1.41E-111	4.35E-111
novel_mir662	297	0	10.11	0.001	-13.30349538	Down	1.41E-111	4.34E-111
novel_mir407	297	0	10.11	0.001	-13.30349538	Down	1.41E-111	4.32E-111
novel_mir743	295	0	10.05	0.001	-13.29490788	Down	7.86E-111	2.40E-110
novel_mir218	293	0	9.98	0.001	-13.2848241	Down	4.38E-110	1.33E-109
novel_mir701	293	0	9.98	0.001	-13.2848241	Down	4.38E-110	1.33E-109
novel_mir690	290	0	9.88	0.001	-13.27029533	Down	5.76E-109	1.74E-108
novel_mir595	288	0	9.81	0.001	-13.26003742	Down	3.21E-108	9.65E-108
novel_mir289	288	0	9.81	0.001	-13.26003742	Down	3.21E-108	9.62E-108
novel_mir588	287	0	9.77	0.001	-13.25414285	Down	7.57E-108	2.25E-107
novel_mir149	284	0	9.67	0.001	-13.23930017	Down	9.96E-107	2.95E-106
novel_mir586	283	0	9.64	0.001	-13.23481743	Down	2.35E-106	6.92E-106
novel_mir636	278	0	9.47	0.001	-13.20914871	Down	1.72E-104	5.02E-104
novel_mir415	278	0	9.47	0.001	-13.20914871	Down	1.72E-104	5.00E-104
novel_mir394	278	0	9.47	0.001	-13.20914871	Down	1.72E-104	4.99E-104
novel_mir198	277	0	9.43	0.001	-13.20304206	Down	4.07E-104	1.18E-103

novel_mir308	276	0	9.4	0.001	-13.19844504	Down	9.60E-104	2.76E-103
novel_mir237	271	0	9.23	0.001	-13.17211493	Down	7.03E-102	2.01E-101
novel_mir771	270	0	9.19	0.001	-13.16584915	Down	1.66E-101	4.72E-101
novel_mir483	268	0	9.13	0.001	-13.15639914	Down	9.25E-101	2.62E-100
novel_mir90	267	0	9.09	0.001	-13.15006458	Down	2.18E-100	6.17E-100
novel_mir78	264	0	8.99	0.001	-13.1341054	Down	2.87E-99	8.09E-99
novel_mir187	263	0	8.96	0.001	-13.12928302	Down	6.78E-99	1.90E-98
novel_mir302	262	0	8.92	0.001	-13.12282799	Down	1.60E-98	4.48E-98
novel_mir58	262	0	8.92	0.001	-13.12282799	Down	1.60E-98	4.47E-98
novel_mir427	259	0	8.82	0.001	-13.10656294	Down	2.10E-97	5.87E-97
novel_mir109	259	0	8.82	0.001	-13.10656294	Down	2.10E-97	5.85E-97
novel_mir131	257	0	8.75	0.001	-13.0950673	Down	1.17E-96	3.24E-96
novel_mir55	256	0	8.72	0.001	-13.09011242	Down	2.77E-96	7.64E-96
novel_mir560	256	0	8.72	0.001	-13.09011242	Down	2.77E-96	7.62E-96
novel_mir77	255	0	8.68	0.001	-13.08347933	Down	6.53E-96	1.79E-95
novel_mir712	254	0	8.65	0.001	-13.07848442	Down	1.54E-95	4.23E-95
novel_mir150	254	0	8.65	0.001	-13.07848442	Down	1.54E-95	4.21E-95
novel_mir348	254	0	8.65	0.001	-13.07848442	Down	1.54E-95	4.20E-95
novel_mir114	253	0	8.62	0.001	-13.07347215	Down	3.64E-95	9.90E-95
novel_mir172	253	0	8.62	0.001	-13.07347215	Down	3.64E-95	9.88E-95
novel_mir630	251	0	8.55	0.001	-13.0617087	Down	2.03E-94	5.49E-94
mmu-miR-6994-3p	251	0	8.55	0.001	-13.0617087	Down	2.03E-94	5.48E-94
novel_mir38	249	0	8.48	0.001	-13.04984855	Down	1.13E-93	3.04E-93
novel_mir167	248	0	8.45	0.001	-13.04473563	Down	2.67E-93	7.15E-93
mmu-miR-7030-3p	247	0	8.41	0.001	-13.03789009	Down	6.29E-93	1.68E-92
novel_mir60	245	0	8.34	0.001	-13.02583167	Down	3.51E-92	9.31E-92
mmu-miR-3071-3p	243	0	8.28	0.001	-13.01541505	Down	1.95E-91	5.18E-91
novel_mir225	242	0	8.24	0.001	-13.00842862	Down	4.61E-91	1.22E-90
novel_mir37	240	0	8.17	0.001	-12.99612036	Down	2.57E-90	6.74E-90
novel_mir562	239	0	8.14	0.001	-12.99081308	Down	6.06E-90	1.58E-89
novel_mir621	238	0	8.11	0.001	-12.9854862	Down	1.43E-89	3.72E-89
novel_mir352	238	0	8.11	0.001	-12.9854862	Down	1.43E-89	3.71E-89
novel_mir249	237	0	8.07	0.001	-12.97835296	Down	3.38E-89	8.75E-89
novel_mir717	236	0	8.04	0.001	-12.97297979	Down	7.98E-89	2.05E-88
novel_mir145	236	0	8.04	0.001	-12.97297979	Down	7.98E-89	2.05E-88
novel_mir268	235	0	8	0.001	-12.96578428	Down	1.88E-88	4.82E-88

novel_mir205	235	0	8	0.001	-12.96578428	Down	1.88E-88	4.81E-88
novel_mir757	235	0	8	0.001	-12.96578428	Down	1.88E-88	4.79E-88
novel_mir343	234	0	7.97	0.001	-12.96036401	Down	4.44E-88	1.13E-87
novel_mir332	229	0	7.8	0.001	-12.92925841	Down	3.26E-86	8.20E-86
novel_mir166	227	0	7.73	0.001	-12.9162527	Down	1.81E-85	4.54E-85
novel_mir689	226	0	7.7	0.001	-12.91064273	Down	4.28E-85	1.07E-84
mmu-miR-6953-3p	226	0	7.7	0.001	-12.91064273	Down	4.28E-85	1.07E-84
novel_mir360	221	0	7.53	0.001	-12.87843415	Down	3.14E-83	7.76E-83
novel_mir261	220	0	7.49	0.001	-12.87075	Down	7.41E-83	1.82E-82
novel_mir510	218	0	7.42	0.001	-12.85720347	Down	4.13E-82	1.01E-81
novel_mir143	215	0	7.32	0.001	-12.83762793	Down	5.43E-81	1.32E-80
novel_mir745	215	0	7.32	0.001	-12.83762793	Down	5.43E-81	1.32E-80
novel_mir27	213	0	7.25	0.001	-12.82376528	Down	3.02E-80	7.33E-80
novel_mir511	210	0	7.15	0.001	-12.80372753	Down	3.98E-79	9.58E-79
novel_mir87	209	0	7.12	0.001	-12.79766153	Down	9.39E-79	2.26E-78
novel_mir323	207	0	7.05	0.001	-12.78340754	Down	5.23E-78	1.25E-77
novel_mir436	206	0	7.02	0.001	-12.77725532	Down	1.23E-77	2.96E-77
novel_mir134	206	0	7.02	0.001	-12.77725532	Down	1.23E-77	2.95E-77
mmu-miR-764-5p	205	0	6.98	0.001	-12.76901132	Down	2.91E-77	6.95E-77
mmu-miR-154-3p	204	0	6.95	0.001	-12.76279726	Down	6.88E-77	1.63E-76
novel_mir223	201	0	6.85	0.001	-12.74188827	Down	9.05E-76	2.12E-75
novel_mir210	200	0	6.81	0.001	-12.73343908	Down	2.14E-75	5.00E-75
novel_mir532	199	0	6.78	0.001	-12.72706956	Down	5.04E-75	1.18E-74
novel_mir382	196	0	6.67	0.001	-12.70347105	Down	6.63E-74	1.54E-73
novel_mir642	195	0	6.64	0.001	-12.69696753	Down	1.56E-73	3.64E-73
novel_mir463	193	0	6.57	0.001	-12.68167766	Down	8.72E-73	2.02E-72
novel_mir81	189	0	6.44	0.001	-12.65284497	Down	2.71E-71	6.22E-71
novel_mir165	189	0	6.44	0.001	-12.65284497	Down	2.71E-71	6.21E-71
novel_mir240	188	0	6.4	0.001	-12.64385619	Down	6.39E-71	1.46E-70
novel_mir59	181	0	6.16	0.001	-12.58871464	Down	2.61E-68	5.88E-68
novel_mir438	179	0	6.1	0.001	-12.57459353	Down	1.45E-67	3.25E-67
novel_mir564	179	0	6.1	0.001	-12.57459353	Down	1.45E-67	3.25E-67
novel_mir85	179	0	6.1	0.001	-12.57459353	Down	1.45E-67	3.24E-67
novel_mir207	175	0	5.96	0.001	-12.54109662	Down	4.51E-66	1.00E-65
novel_mir627	175	0	5.96	0.001	-12.54109662	Down	4.51E-66	1.00E-65
novel_mir412	173	0	5.89	0.001	-12.52405192	Down	2.51E-65	5.56E-65

novel_mir220	173	0	5.89	0.001	-12.52405192	Down	2.51E-65	5.55E-65
novel_mir505	173	0	5.89	0.001	-12.52405192	Down	2.51E-65	5.54E-65
mmu-miR-1982-3p	166	0	5.65	0.001	-12.46403515	Down	1.03E-62	2.25E-62
novel_mir732	165	0	5.62	0.001	-12.45635442	Down	2.42E-62	5.30E-62
novel_mir667	164	0	5.59	0.001	-12.44863257	Down	5.72E-62	1.25E-61
novel_mir203	161	0	5.48	0.001	-12.41996018	Down	7.52E-61	1.63E-60
novel_mir535	157	0	5.35	0.001	-12.38532318	Down	2.33E-59	5.06E-59
novel_mir318	156	0	5.31	0.001	-12.37449615	Down	5.51E-59	1.19E-58
novel_mir61	156	0	5.31	0.001	-12.37449615	Down	5.51E-59	1.19E-58
novel_mir538	154	0	5.24	0.001	-12.3553511	Down	3.07E-58	6.61E-58
novel_mir127	144	0	4.9	0.001	-12.25856603	Down	1.65E-54	3.51E-54
novel_mir625	143	0	4.87	0.001	-12.24970606	Down	3.89E-54	8.27E-54
novel_mir159	141	0	4.8	0.001	-12.22881869	Down	2.17E-53	4.59E-53
novel_mir222	139	0	4.73	0.001	-12.20762447	Down	1.21E-52	2.55E-52
mmu-miR-7034-3p	138	0	4.7	0.001	-12.19844504	Down	2.85E-52	6.01E-52
novel_mir267	135	0	4.6	0.001	-12.16741815	Down	3.75E-51	7.89E-51
mmu-miR-6955-3p	127	0	4.33	0.001	-12.08015131	Down	3.61E-48	7.59E-48
novel_mir527	125	0	4.26	0.001	-12.05663772	Down	2.01E-47	4.21E-47
novel_mir212	123	0	4.19	0.001	-12.03273453	Down	1.12E-46	2.34E-46
novel_mir447	123	0	4.19	0.001	-12.03273453	Down	1.12E-46	2.34E-46
novel_mir216	122	0	4.15	0.001	-12.01889562	Down	2.65E-46	5.51E-46
novel_mir313	116	0	3.95	0.001	-11.94763694	Down	4.58E-44	9.46E-44
novel_mir53	115	0	3.92	0.001	-11.93663794	Down	1.08E-43	2.22E-43
novel_mir610	108	0	3.68	0.001	-11.84549005	Down	4.41E-41	9.01E-41
novel_mir695	103	0	3.51	0.001	-11.77725532	Down	3.23E-39	6.52E-39
novel_mir329	99	0	3.37	0.001	-11.71853288	Down	1.00E-37	2.01E-37
mmu-miR-7058-3p	95	0	3.24	0.001	-11.6617781	Down	3.12E-36	6.21E-36
novel_mir754	94	0	3.2	0.001	-11.64385619	Down	7.36E-36	1.46E-35
novel_mir283	90	0	3.06	0.001	-11.57931594	Down	2.28E-34	4.52E-34
novel_mir659	88	0	3	0.001	-11.55074679	Down	1.27E-33	2.51E-33
novel_mir647	84	0	2.86	0.001	-11.48179943	Down	3.95E-32	7.71E-32
novel_mir420	79	0	2.69	0.001	-11.39339046	Down	2.89E-30	5.63E-30
novel_mir658	76	0	2.59	0.001	-11.33873638	Down	3.81E-29	7.39E-29
mmu-miR-6901-3p	55	0	1.87	0.001	-10.86882255	Down	2.59E-21	4.83E-21
novel_mir231	50	0	1.7	0.001	-10.73131903	Down	1.90E-19	3.50E-19
mmu-miR-7070-3p	45	0	1.53	0.001	-10.57931594	Down	1.39E-17	2.53E-17

novel_mir200	34	0	1.16	0.001	-10.17990909	Down	1.76E-13	3.11E-13
mmu-miR-532-3p	868	1	29.56	0.03	-9.944468053	Down	0	0
novel_mir265	24	0	0.82	0.001	-9.6794801	Down	9.47E-10	1.62E-09
novel_mir73	23	0	0.78	0.001	-9.607330314	Down	2.23E-09	3.80E-09
mmu-miR-199b-3p	419760	746	14295.05	18.67	-9.580578023	Down	0	0
mmu-miR-485-3p	1118	3	38.07	0.08	-8.894438855	Down	0	0
novel_mir408	13	0	0.44	0.001	-8.781359714	Down	1.20E-05	1.83E-05
novel_mir129	13	0	0.44	0.001	-8.781359714	Down	1.20E-05	1.83E-05
mmu-miR-7021-5p	470	2	16.01	0.05	-8.322829498	Down	1.56E-171	7.40E-171
mmu-miR-466d-3p	619	3	21.08	0.08	-8.041659152	Down	8.56E-225	4.88E-224
mmu-miR-98-3p	321	2	10.93	0.05	-7.772149591	Down	2.75E-116	8.77E-116
mmu-miR-3077-3p	281	2	9.57	0.05	-7.58044702	Down	1.76E-101	4.99E-101
novel_mir507	1396	11	47.54	0.28	-7.407571266	Down	0	0
mmu-miR-299b-3p	366	3	12.46	0.08	-7.283088353	Down	4.17E-131	1.55E-130
mmu-miR-329-3p	1079	10	36.75	0.25	-7.199672345	Down	0	0
mmu-miR-7681-5p	215	2	7.32	0.05	-7.193771743	Down	4.29E-77	1.02E-76
mmu-miR-7115-3p	800	8	27.24	0.2	-7.089582893	Down	1.84E-282	1.19E-281
mmu-miR-1249-3p	1709	17	58.2	0.43	-7.080538683	Down	0	0
mmu-miR-495-3p	1555	17	52.96	0.43	-6.944422652	Down	0	0
mmu-miR-148b-3p	669	9	22.78	0.23	-6.629990076	Down	1.41E-232	8.28E-232
mmu-miR-335-3p	1821	25	62.01	0.63	-6.621005251	Down	0	0
mmu-miR-669c-5p	2122	30	72.27	0.75	-6.590362488	Down	0	0
mmu-miR-466h-3p	6225	88	211.99	2.2	-6.590348878	Down	0	0
mmu-let-7b-3p	4824	69	164.28	1.73	-6.569241004	Down	0	0
mmu-miR-24-3p	1965736	30462	66943.73	762.54	-6.45599211	Down	0	0
mmu-miR-122-3p	1028	16	35.01	0.4	-6.451623252	Down	0	0
mmu-miR-136-3p	254	4	8.65	0.1	-6.434628228	Down	3.15E-88	8.01E-88
mmu-miR-7031-5p	502	8	17.1	0.2	-6.417852515	Down	6.52E-173	3.13E-172
mmu-miR-486b-5p	76698	1230	2611.97	30.79	-6.406532649	Down	0	0
mmu-miR-574-3p	57274	941	1950.48	23.56	-6.371345856	Down	0	0
mmu-miR-335-5p	308	5	10.49	0.13	-6.334359244	Down	1.76E-106	5.21E-106
mmu-miR-669o-3p	522	9	17.78	0.23	-6.272477653	Down	1.05E-178	5.20E-178
mmu-miR-1843a-3p	3360	71	114.43	1.78	-6.00644428	Down	0	0
mmu-miR-204-3p	836	18	28.47	0.45	-5.983373681	Down	4.97E-280	3.16E-279
mmu-miR-211-5p	969	21	33	0.53	-5.960329855	Down	0	0
mmu-miR-6538	3948	91	134.45	2.28	-5.881892121	Down	0	0

novel_mir23	425	10	14.47	0.25	-5.854993017	Down	6.46E-142	2.54E-141
mmu-miR-214-3p	3450	83	117.49	2.08	-5.81981063	Down	0	0
mmu-miR-339-5p	2081	51	70.87	1.28	-5.790959334	Down	0	0
mmu-miR-363-5p	290	7	9.88	0.18	-5.77844223	Down	4.77E-97	1.33E-96
mmu-miR-484	326990	8379	11135.74	209.75	-5.730382811	Down	0	0
mmu-miR-6924-5p	462	12	15.73	0.3	-5.71241236	Down	1.32E-152	5.61E-152
mmu-miR-664-3p	7023	184	239.17	4.61	-5.697125877	Down	0	0
mmu-miR-466f-3p	692	19	23.57	0.48	-5.617773543	Down	1.93E-226	1.11E-225
mmu-miR-1306-5p	3075	87	104.72	2.18	-5.586065057	Down	0	0
mmu-miR-669f-3p	234	7	7.97	0.18	-5.468510913	Down	8.48E-77	2.00E-76
mmu-miR-7658-5p	386	12	13.15	0.3	-5.453956489	Down	3.55E-125	1.23E-124
mmu-miR-1981-3p	1371	45	46.69	1.13	-5.368718911	Down	0	0
novel_mir141	836	28	28.47	0.7	-5.345943761	Down	9.48E-267	5.86E-266
mmu-miR-142a-5p	1952	66	66.48	1.65	-5.332382453	Down	0	0
mmu-miR-337-5p	340	12	11.58	0.3	-5.270528942	Down	1.15E-108	3.47E-108
mmu-miR-7658-3p	958	34	32.62	0.85	-5.262150131	Down	4.51E-303	2.97E-302
mmu-miR-467a-5p	2002	73	68.18	1.83	-5.219433046	Down	0	0
mmu-miR-8091	249	9	8.48	0.23	-5.204358499	Down	1.02E-79	2.47E-79
mmu-miR-6948-3p	514	19	17.5	0.48	-5.188176706	Down	1.87E-162	8.43E-162
novel_mir607	342	13	11.65	0.33	-5.14172012	Down	3.50E-108	1.05E-107
mmu-let-7d-3p	829655	33041	28254.15	827.1	-5.094257209	Down	0	0
mmu-let-7f-1-3p	8696	348	296.14	8.71	-5.087462841	Down	0	0
mmu-miR-1964-3p	971	40	33.07	1	-5.04745114	Down	1.41E-300	9.23E-300
mmu-miR-126a-5p	2565	107	87.35	2.68	-5.026502797	Down	0	0
mmu-miR-34b-3p	1882	80	64.09	2	-5.002027365	Down	0	0
mmu-miR-202-5p	425	19	14.47	0.48	-4.913886706	Down	8.67E-131	3.20E-130
mmu-miR-144-3p	9264	426	315.49	10.66	-4.887314934	Down	0	0
mmu-miR-124-3p	1460	67	49.72	1.68	-4.887293158	Down	0	0
mmu-miR-543-3p	1410	65	48.02	1.63	-4.880691534	Down	0	0
mmu-miR-434-5p	1009	47	34.36	1.18	-4.863871272	Down	4.00E-306	2.65E-305
novel_mir555	467	22	15.9	0.55	-4.853451337	Down	2.61E-142	1.03E-141
mmu-miR-6943-3p	467	23	15.9	0.58	-4.776830055	Down	3.22E-141	1.26E-140
mmu-miR-101a-3p	601	30	20.47	0.75	-4.770476697	Down	7.77E-181	3.89E-180
mmu-miR-467d-5p	357	18	12.16	0.45	-4.756074417	Down	7.01E-108	2.09E-107
mmu-miR-6956-3p	301	15	10.25	0.38	-4.753480681	Down	2.17E-91	5.74E-91
mmu-miR-877-3p	1440	74	49.04	1.85	-4.728361803	Down	0	0

mmu-miR-199a-5p	294	15	10.01	0.38	-4.719298745	Down	6.30E-89	1.62E-88
mmu-miR-3470b	535	28	18.22	0.7	-4.702024227	Down	8.89E-160	3.96E-159
mmu-miR-204-5p	40468	2116	1378.15	52.97	-4.701413603	Down	0	0
mmu-let-7f-2-3p	4574	254	155.77	6.36	-4.614246833	Down	0	0
mmu-miR-23b-5p	2841	160	96.75	4.01	-4.592587519	Down	0	0
mmu-miR-16-2-3p	819	47	27.89	1.18	-4.56288917	Down	2.19E-239	1.30E-238
mmu-miR-26b-3p	832	48	28.33	1.2	-4.561224291	Down	6.74E-243	4.03E-242
mmu-miR-106b-5p	1730	100	58.92	2.5	-4.55875743	Down	0	0
novel_mir453	1020	59	34.74	1.48	-4.552928673	Down	3.19E-297	2.08E-296
mmu-miR-127-3p	91586	5503	3118.99	137.75	-4.500954703	Down	0	0
mmu-miR-152-3p	385	23	13.11	0.58	-4.498470975	Down	1.68E-112	5.21E-112
novel_mir340	418	25	14.24	0.63	-4.498453507	Down	5.60E-122	1.88E-121
novel_mir355	416	25	14.17	0.63	-4.491344119	Down	2.78E-121	9.24E-121
mmu-miR-29b-1-5p	618	38	21.05	0.95	-4.46974891	Down	1.66E-178	8.17E-178
mmu-miR-350-3p	6146	382	209.3	9.56	-4.452417883	Down	0	0
mmu-miR-3473g	429	27	14.61	0.68	-4.425277622	Down	8.20E-124	2.82E-123
mmu-miR-292b-5p	238	15	8.11	0.38	-4.415630591	Down	2.29E-69	5.20E-69
mmu-miR-134-3p	375	24	12.77	0.6	-4.411652214	Down	4.83E-108	1.44E-107
mmu-miR-10a-3p	497	32	16.93	0.8	-4.403438163	Down	1.80E-142	7.13E-142
novel_mir41	962	62	32.76	1.55	-4.401595236	Down	4.38E-274	2.76E-273
novel_mir49	389	25	13.25	0.63	-4.394496721	Down	6.49E-112	2.01E-111
mmu-miR-7687-3p	400	26	13.62	0.65	-4.389143175	Down	9.51E-115	2.98E-114
mmu-miR-871-5p	306	20	10.42	0.5	-4.381283373	Down	4.73E-88	1.20E-87
mmu-miR-770-3p	665	44	22.65	1.1	-4.363935621	Down	6.60E-189	3.43E-188
mmu-miR-3058-3p	393	26	13.38	0.65	-4.363494588	Down	2.49E-112	7.73E-112
mmu-miR-125b-5p	28523	1928	971.36	48.26	-4.331106258	Down	0	0
mmu-miR-182-3p	292	20	9.94	0.5	-4.313245852	Down	3.20E-83	7.91E-83
mmu-miR-486b-3p	41684	2859	1419.56	71.57	-4.309945038	Down	0	0
mmu-miR-92b-3p	13554	930	461.59	23.28	-4.309449006	Down	0	0
mmu-let-7c-2-3p	245	18	8.34	0.45	-4.212050477	Down	6.11E-69	1.39E-68
mmu-miR-130a-3p	6553	504	223.16	12.62	-4.144294641	Down	0	0
novel_mir773	465	36	15.84	0.9	-4.137503524	Down	9.84E-128	3.51E-127
mmu-miR-467b-5p	377	29	12.84	0.73	-4.136604928	Down	4.82E-104	1.39E-103
mmu-miR-300-3p	247	19	8.41	0.48	-4.13099949	Down	1.02E-68	2.31E-68
mmu-miR-125a-5p	47302	3701	1610.88	92.65	-4.11991424	Down	0	0
mmu-miR-363-3p	562	45	19.14	1.13	-4.082196152	Down	1.09E-152	4.63E-152

mmu-miR-7075-3p	338	27	11.51	0.68	-4.081209277	Down	1.48E-92	3.96E-92
mmu-miR-151-3p	38861	3158	1323.42	79.05	-4.065361715	Down	0	0
mmu-miR-8112	2833	234	96.48	5.86	-4.041257337	Down	0	0
mmu-miR-365-3p	2080	173	70.84	4.33	-4.032125282	Down	0	0
mmu-miR-450b-5p	385	33	13.11	0.83	-3.981412539	Down	3.08E-103	8.84E-103
mmu-miR-181d-3p	285	25	9.71	0.63	-3.946047562	Down	2.34E-76	5.51E-76
mmu-miR-770-5p	339	30	11.54	0.75	-3.943608818	Down	2.70E-90	7.06E-90
mmu-miR-1247-5p	151	14	5.14	0.35	-3.876341532	Down	1.52E-40	3.09E-40
mmu-miR-7043-3p	682	65	23.23	1.63	-3.833045285	Down	4.64E-176	2.26E-175
mmu-miR-433-3p	741	72	25.23	1.8	-3.809071395	Down	5.18E-190	2.71E-189
mmu-miR-186-5p	978	95	33.31	2.38	-3.806921875	Down	3.23E-250	1.96E-249
mmu-miR-193b-5p	334	33	11.37	0.83	-3.775977108	Down	3.76E-86	9.45E-86
mmu-miR-485-5p	11334	1162	385.98	29.09	-3.729930893	Down	0	0
mmu-miR-130b-5p	899	95	30.62	2.38	-3.685440804	Down	4.71E-224	2.68E-223
mmu-miR-434-3p	6211	663	211.52	16.6	-3.671538935	Down	0	0
mmu-miR-30e-5p	659	71	22.44	1.78	-3.65612353	Down	1.13E-163	5.13E-163
mmu-miR-101b-3p	2311	253	78.7	6.33	-3.636086231	Down	0	0
mmu-miR-297c-5p	728	80	24.79	2	-3.631686366	Down	2.03E-179	1.01E-178
novel_mir84	300	33	10.22	0.83	-3.62214005	Down	6.37E-75	1.49E-74
mmu-let-7i-3p	3059	343	104.18	8.59	-3.600276401	Down	0	0
mmu-miR-382-5p	7802	877	265.7	21.95	-3.597505383	Down	0	0
mmu-miR-210-3p	1358	153	46.25	3.83	-3.594037068	Down	0	0
mmu-miR-27b-3p	301568	34155	10269.99	854.99	-3.58638342	Down	0	0
mmu-miR-183-5p	6526	745	222.24	18.65	-3.574870969	Down	0	0
mmu-miR-27a-3p	898139	102583	30586.4	2567.92	-3.574218149	Down	0	0
mmu-miR-139-3p	2866	341	97.6	8.54	-3.514573173	Down	0	0
mmu-miR-323-3p	376	45	12.8	1.13	-3.501749132	Down	9.08E-91	2.39E-90
novel_mir50	6062	730	206.44	18.27	-3.498173997	Down	0	0
mmu-miR-19a-3p	322	40	10.97	1	-3.455491621	Down	6.44E-77	1.53E-76
mmu-miR-1306-3p	2633	328	89.67	8.21	-3.449171271	Down	0	0
mmu-miR-196b-5p	203113	25423	6917.07	636.4	-3.442155316	Down	0	0
mmu-miR-375-3p	107106	13760	3647.53	344.45	-3.404553361	Down	0	0
mmu-miR-6960-5p	951	124	32.39	3.1	-3.385208348	Down	1.39E-219	7.84E-219
mmu-miR-466k	531	70	18.08	1.75	-3.368967851	Down	6.93E-123	2.35E-122
mmu-miR-224-5p	2979	398	101.45	9.96	-3.348479313	Down	0	0
mmu-miR-3087-3p	328	44	11.17	1.1	-3.344053757	Down	4.84E-76	1.14E-75

mmu-miR-32-5p	976	132	33.24	3.3	-3.332382453	Down	4.98E-222	2.81E-221
mmu-miR-130b-3p	304	41	10.35	1.03	-3.328914525	Down	1.89E-70	4.32E-70
mmu-miR-369-5p	13132	1778	447.21	44.51	-3.328751043	Down	0	0
mmu-miR-17-5p	512	70	17.44	1.75	-3.316973213	Down	7.81E-117	2.50E-116
mmu-miR-361-5p	3892	532	132.54	13.32	-3.314761837	Down	0	0
mmu-miR-431-5p	1363	188	46.42	4.71	-3.300947558	Down	8.00E-307	5.33E-306
mmu-miR-3102-5p.2-5p	470	66	16.01	1.65	-3.278435378	Down	3.21E-106	9.42E-106
mmu-miR-148a-3p	4641	666	158.05	16.67	-3.245055026	Down	0	0
mmu-miR-7a-2-3p	384	55	13.08	1.38	-3.244622369	Down	2.19E-86	5.53E-86
mmu-let-7a-1-3p	4541	659	154.65	16.5	-3.228468904	Down	0	0
mmu-miR-450b-3p	274	41	9.33	1.03	-3.179232744	Down	5.63E-61	1.23E-60
mmu-miR-10b-5p	56638	8555	1928.82	214.15	-3.171024933	Down	0	0
mmu-miR-19b-3p	17318	2642	589.77	66.14	-3.156557488	Down	0	0
mmu-miR-8116	5084	783	173.14	19.6	-3.143013505	Down	0	0
mmu-miR-379-5p	10898	1682	371.13	42.1	-3.140032486	Down	0	0
mmu-miR-487b-3p	977	151	33.27	3.78	-3.137763727	Down	7.71E-210	4.27E-209
mmu-miR-615-3p	894	139	30.45	3.48	-3.129283017	Down	1.19E-191	6.32E-191
mmu-miR-322-5p	10984	1711	374.06	42.83	-3.126576118	Down	0	0
novel_mir107	859	134	29.25	3.35	-3.126203624	Down	6.05E-184	3.08E-183
mmu-miR-150-5p	141786	22235	4828.57	556.6	-3.116883179	Down	0	0
novel_mir168	7485	1197	254.9	29.96	-3.088821844	Down	0	0
mmu-miR-10a-5p	172002	27598	5857.58	690.85	-3.083860347	Down	0	0
mmu-miR-410-3p	2887	473	98.32	11.84	-3.053815835	Down	0	0
mmu-miR-196a-5p	1985	328	67.6	8.21	-3.041569119	Down	0	0
novel_mir8	204	34	6.95	0.85	-3.031478231	Down	1.21E-43	2.48E-43
mmu-let-7c-5p	693066	116097	23602.57	2906.21	-3.021733098	Down	0	0
mmu-miR-193b-3p	5550	949	189.01	23.76	-2.991855824	Down	0	0
mmu-let-7a-5p	212506	36409	7236.96	911.41	-2.989211693	Down	0	0
mmu-miR-7a-1-3p	5153	889	175.49	22.25	-2.979511582	Down	0	0
mmu-miR-18a-3p	1002	173	34.12	4.33	-2.978178717	Down	4.73E-204	2.60E-203
mmu-miR-18a-5p	2295	397	78.16	9.94	-2.97511271	Down	0	0
mmu-miR-30c-5p	4172	755	142.08	18.9	-2.910245347	Down	0	0
mmu-let-7f-5p	150494	27422	5125.12	686.44	-2.900380256	Down	0	0
mmu-let-7b-5p	450255	82319	15333.57	2060.66	-2.895515237	Down	0	0
mmu-miR-215-3p	1648	302	56.12	7.56	-2.892056869	Down	0	0
mmu-miR-301a-3p	606	112	20.64	2.8	-2.881944238	Down	7.03E-120	2.32E-119

mmu-miR-466i-5p	3069	568	104.52	14.22	-2.87778566	Down	0	0
mmu-let-7k	497155	92808	16930.76	2323.22	-2.865449052	Down	0	0
mmu-miR-24-2-5p	2114	398	71.99	9.96	-2.853578871	Down	0	0
mmu-miR-6899-3p	197	37	6.71	0.93	-2.851010145	Down	8.87E-40	1.80E-39
mmu-miR-6936-5p	300	57	10.22	1.43	-2.837308144	Down	2.35E-59	5.08E-59
mmu-miR-342-3p	14666	2798	499.46	70.04	-2.834118153	Down	0	0
mmu-miR-222-3p	42054	8090	1432.16	202.51	-2.822127623	Down	0	0
mmu-miR-215-5p	9705	1879	330.51	47.04	-2.812733958	Down	0	0
mmu-miR-125a-3p	191	37	6.5	0.93	-2.805137097	Down	5.45E-38	1.10E-37
mmu-miR-28a-5p	235	47	8	1.18	-2.76121314	Down	1.44E-45	2.98E-45
mmu-miR-30d-5p	59416	12082	2023.43	302.44	-2.742082076	Down	0	0
mmu-miR-1191a	2116	432	72.06	10.81	-2.736832129	Down	0	0
mmu-miR-5126	1718	358	58.51	8.96	-2.707112581	Down	0	0
mmu-miR-30a-5p	2990	628	101.83	15.72	-2.695489532	Down	0	0
mmu-miR-96-5p	733	154	24.96	3.86	-2.692945182	Down	1.52E-134	5.74E-134
mmu-miR-1843b-5p	691	147	23.53	3.68	-2.676723649	Down	5.38E-126	1.88E-125
mmu-miR-341-3p	7007	1495	238.63	37.42	-2.672893963	Down	0	0
mmu-miR-467e-5p	173	37	5.89	0.93	-2.662965013	Down	1.05E-32	2.06E-32
mmu-miR-15b-5p	7680	1650	261.54	41.3	-2.662817923	Down	0	0
mmu-miR-182-5p	496	109	16.89	2.73	-2.629196472	Down	3.54E-89	9.15E-89
mmu-miR-543-5p	585	129	19.92	3.23	-2.624611577	Down	1.25E-104	3.64E-104
mmu-miR-339-3p	1973	437	67.19	10.94	-2.618633792	Down	0	0
mmu-let-7d-5p	88588	19755	3016.89	494.52	-2.60896132	Down	0	0
mmu-miR-1934-5p	453	101	15.43	2.53	-2.608528772	Down	7.14E-81	1.73E-80
mmu-miR-151-5p	15744	3523	536.17	88.19	-2.604003519	Down	0	0
mmu-miR-8107	484	109	16.48	2.73	-2.593743386	Down	9.62E-86	2.41E-85
novel_mir339	979	223	33.34	5.58	-2.578917077	Down	2.74E-170	1.28E-169
mmu-miR-667-3p	473	108	16.11	2.7	-2.576925182	Down	4.18E-83	1.03E-82
mmu-miR-328-3p	615598	140738	20964.38	3523.04	-2.573047404	Down	0	0
mmu-miR-222-5p	1087	253	37.02	6.33	-2.54802749	Down	3.20E-186	1.64E-185
mmu-miR-219a-1-3p	655	157	22.31	3.93	-2.505089296	Down	8.65E-111	2.64E-110
mmu-let-7g-5p	583994	142493	19888.09	3566.97	-2.479133743	Down	0	0
mmu-miR-149-5p	15203	3742	517.74	93.67	-2.466568813	Down	0	0
mmu-miR-326-3p	7949	1977	270.71	49.49	-2.451539234	Down	0	0
mmu-miR-34c-3p	285	71	9.71	1.78	-2.447594054	Down	6.11E-48	1.28E-47
mmu-miR-30e-3p	1726	431	58.78	10.79	-2.445630494	Down	1.09E-280	6.98E-280

novel_mir3	504	126	17.16	3.15	-2.445625819	Down	3.04E-83	7.52E-83
mmu-miR-106b-3p	1160	291	39.5	7.28	-2.439842298	Down	1.08E-188	5.60E-188
mmu-miR-22-3p	649510	166068	22119.26	4157.11	-2.411650293	Down	0	0
novel_mir722	445	114	15.15	2.85	-2.410283969	Down	1.87E-72	4.31E-72
mmu-miR-128-1-5p	54	14	1.84	0.35	-2.394278939	Down	5.15E-10	8.87E-10
mmu-miR-330-3p	768	199	26.15	4.98	-2.392593299	Down	1.04E-122	3.52E-122
mmu-miR-1198-3p	391	102	13.32	2.55	-2.38502493	Down	4.70E-63	1.03E-62
mmu-miR-98-5p	20797	5421	708.25	135.7	-2.383837976	Down	0	0
mmu-let-7e-3p	2271	609	77.34	15.24	-2.343351862	Down	0	0
mmu-miR-540-3p	525	141	17.88	3.53	-2.340606648	Down	2.82E-82	6.90E-82
mmu-miR-92a-3p	1542701	417999	52537.15	10463.6	-2.327958644	Down	0	0
mmu-miR-218-5p	2785	767	94.84	19.2	-2.304389352	Down	0	0
mmu-miR-23b-3p	3461	965	117.87	24.16	-2.286504214	Down	0	0
mmu-miR-1843a-5p	3545	992	120.73	24.83	-2.281628047	Down	0	0
mmu-miR-99b-5p	22541	6370	767.64	159.46	-2.267235315	Down	0	0
mmu-miR-154-5p	6444	1848	219.45	46.26	-2.246055099	Down	0	0
mmu-miR-30b-5p	5056	1460	172.18	36.55	-2.235974261	Down	0	0
mmu-miR-103-3p	467	136	15.9	3.4	-2.225420114	Down	6.26E-69	1.42E-68
mmu-miR-374b-5p	960	287	32.69	7.18	-2.186793628	Down	9.43E-137	3.62E-136
mmu-miR-26a-5p	32901	9948	1120.45	249.02	-2.169744748	Down	0	0
mmu-miR-125b-1-3p	376	114	12.8	2.85	-2.167109986	Down	4.92E-54	1.04E-53
mmu-miR-450a-5p	2033	637	69.23	15.95	-2.117840924	Down	6.11E-276	3.86E-275
novel_mir91	38	12	1.29	0.3	-2.10433666	Down	1.54E-06	2.45E-06
mmu-miR-192-5p	59308	18846	2019.75	471.76	-2.098051726	Down	0	0
mmu-miR-26b-5p	12288	3907	418.47	97.8	-2.09721783	Down	0	0
mmu-miR-139-5p	81212	26032	2765.7	651.65	-2.085475465	Down	0	0
mmu-miR-181c-5p	10624	3453	361.8	86.44	-2.065421431	Down	0	0
mmu-miR-3084-3p	313	102	10.66	2.55	-2.063638286	Down	1.50E-42	3.07E-42
mmu-miR-423-3p	96384	31418	3282.39	786.47	-2.061283024	Down	0	0
mmu-miR-122-5p	219616	72330	7479.09	1810.61	-2.046386917	Down	0	0
mmu-miR-191-3p	1938	645	66	16.15	-2.03093186	Down	4.73E-249	2.86E-248
mmu-miR-223-3p	43758	14731	1490.19	368.76	-2.014742209	Down	0	0
mmu-miR-486a-3p	10018	3389	341.17	84.84	-2.007674266	Down	0	0
mmu-miR-15b-3p	3374	1172	114.9	29.34	-1.969438022	Down	0	0
mmu-miR-6395	32	11	1.09	0.28	-1.960829403	Down	2.28E-05	3.42E-05
mmu-miR-191-5p	701318	245090	23883.6	6135.24	-1.960828626	Down	0	0

mmu-miR-212-3p	1429	501	48.67	12.54	-1.956495426	Down	3.69E-175	1.79E-174
mmu-miR-27a-5p	262	93	8.92	2.33	-1.936713755	Down	3.61E-33	7.10E-33
mmu-miR-598-3p	213	76	7.25	1.9	-1.931981577	Down	3.92E-27	7.57E-27
novel_mir498	95	34	3.24	0.85	-1.930459067	Down	6.74E-13	1.19E-12
mmu-miR-409-3p	4007	1457	136.46	36.47	-1.903696017	Down	0	0
mmu-miR-15a-5p	3532	1292	120.28	32.34	-1.895005189	Down	0	0
mmu-miR-340-5p	622	231	21.18	5.78	-1.873561191	Down	5.95E-73	1.38E-72
mmu-miR-93-5p	68728	25759	2340.55	644.81	-1.859901562	Down	0	0
mmu-miR-3074-2-3p	1162	436	39.57	10.91	-1.858755964	Down	3.37E-133	1.26E-132
mmu-miR-345-5p	206	79	7.02	1.98	-1.8259706	Down	1.78E-24	3.38E-24
mmu-miR-411-5p	2483	964	84.56	24.13	-1.809147461	Down	5.49E-272	3.41E-271
mmu-miR-421-3p	493	192	16.79	4.81	-1.803493431	Down	2.47E-55	5.30E-55
mmu-miR-7662-3p	56	22	1.91	0.55	-1.796069115	Down	1.61E-07	2.64E-07
mmu-miR-381-3p	222	88	7.56	2.2	-1.780882711	Down	2.36E-25	4.50E-25
mmu-miR-194-5p	11756	4723	400.35	118.23	-1.759665651	Down	0	0
mmu-miR-338-5p	418	168	14.24	4.21	-1.758057008	Down	1.43E-45	2.97E-45
mmu-miR-466g	345	140	11.75	3.5	-1.74723393	Down	1.67E-37	3.34E-37
mmu-miR-297a-5p	1216	501	41.41	12.54	-1.723441855	Down	2.03E-125	7.08E-125
mmu-miR-574-5p	8937	3719	304.35	93.1	-1.708878293	Down	0	0
novel_mir39	196	82	6.67	2.05	-1.702062852	Down	2.84E-21	5.29E-21
mmu-miR-296-5p	352	152	11.99	3.8	-1.657760335	Down	1.47E-35	2.92E-35
mmu-miR-6988-3p	227	98	7.73	2.45	-1.657686665	Down	1.62E-23	3.07E-23
mmu-miR-503-3p	1431	621	48.73	15.55	-1.647895642	Down	3.35E-138	1.29E-137
mmu-miR-29c-3p	1699	748	57.86	18.72	-1.627985888	Down	9.73E-161	4.35E-160
mmu-miR-1198-5p	252063	111234	8584.08	2784.48	-1.624255591	Down	0	0
mmu-miR-669b-5p	2447	1089	83.33	27.26	-1.612050418	Down	2.11E-227	1.22E-226
mmu-miR-128-3p	90942	40809	3097.06	1021.56	-1.600125392	Down	0	0
mmu-miR-17-3p	404	187	13.76	4.68	-1.555900035	Down	3.75E-37	7.49E-37
mmu-miR-140-5p	3461	1644	117.87	41.15	-1.518232238	Down	1.58E-293	1.03E-292
mmu-miR-132-5p	642	308	21.86	7.71	-1.503490636	Down	3.04E-55	6.50E-55
mmu-miR-22-5p	1438	714	48.97	17.87	-1.454358562	Down	1.61E-115	5.11E-115
mmu-miR-425-3p	14377	7187	489.61	179.91	-1.444357646	Down	0	0
mmu-miR-100-5p	10567	5309	359.86	132.9	-1.437094645	Down	0	0
mmu-miR-190b-5p	522	269	17.78	6.73	-1.401576914	Down	8.75E-41	1.78E-40
mmu-miR-150-3p	907	475	30.89	11.89	-1.377391156	Down	7.93E-68	1.78E-67
mmu-miR-877-5p	2487	1312	84.7	32.84	-1.366907842	Down	1.72E-180	8.57E-180

mmu-miR-223-5p	3003	1587	102.27	39.73	-1.364082307	Down	6.86E-217	3.84E-216
mmu-miR-203-3p	1240	660	42.23	16.52	-1.35405456	Down	1.11E-89	2.88E-89
mmu-miR-30a-3p	7899	4216	269	105.54	-1.349816284	Down	0	0
novel_mir579	223	120	7.59	3	-1.339137385	Down	3.31E-17	6.01E-17
mmu-miR-872-3p	5362	2993	182.6	74.92	-1.285263961	Down	0	0
mmu-miR-23a-3p	590879	333546	20122.56	8349.52	-1.26904869	Down	0	0
mmu-miR-187-3p	1259	724	42.88	18.12	-1.24272195	Down	9.53E-80	2.31E-79
mmu-miR-1a-3p	3691	2129	125.7	53.29	-1.238047912	Down	1.05E-228	6.11E-228
mmu-miR-29a-5p	357	206	12.16	5.16	-1.236700258	Down	1.06E-23	2.00E-23
mmu-miR-126a-3p	16986	10088	578.46	252.53	-1.195762415	Down	0	0
mmu-miR-28a-3p	6689	4138	227.8	103.58	-1.137022283	Down	0	0
mmu-miR-21a-5p	199248	125910	6785.45	3151.85	-1.106245619	Down	0	0
mmu-let-7i-5p	632649	415207	21545.05	10393.71	-1.051645735	Down	0	0
mmu-miR-451a	1416703	935689	48246.25	23422.72	-1.042508191	Down	0	0
mmu-miR-669o-5p	176	119	5.99	2.98	-1.007243672	Down	2.44E-09	4.13E-09
