# Supplementary material for: Pathogenic Effects and Potential Regulatory Mechanisms of Tea Polyphenols on Obesity
Source: Biomed Res Int. 2019 Jun 11;2019:2579734. doi: 10.1155/2019/2579734 (PMC6595166; doi:10.1155/2019/2579734)
Supplement: Supplementary 3 — Supplementary Material S3. Differentially expressed piRNAs in the control group vs. low-dose group. [file 2579734.f3.pdf]

miRNA id	Count (DUI4)	Count (DA3)	TPM (DUI4)	TPM (DA3)	log2 Ratio (DA3/DUI4)	Up-Down-Regulation (DA3/DUI4)	P-value	FDR
novel_pir2296	0	5189	0.001	257.3	17.97309193	Up	0	0
novel_pir2295	0	3061	0.001	151.78	17.21162217	Up	0	0
novel_pir1968	0	337	0.001	16.71	14.02842411	Up	2.53E-132	8.01E-131
novel_pir373	0	296	0.001	14.68	13.84156435	Up	2.52E-116	6.78E-115
novel_pir1535	0	260	0.001	12.89	13.65396464	Up	2.82E-102	5.14E-101
novel_pir1634	0	257	0.001	12.74	13.63707766	Up	4.18E-101	7.34E-100
novel_pir2444	0	242	0.001	12	13.55074679	Up	2.98E-95	4.27E-94
novel_pir1636	0	234	0.001	11.6	13.50183718	Up	3.95E-92	5.02E-91
novel_pir1633	0	231	0.001	11.45	13.48305998	Up	5.85E-91	7.16E-90
novel_pir1632	0	227	0.001	11.26	13.45891921	Up	2.13E-89	2.41E-88
novel_pir86	0	221	0.001	10.96	13.41996018	Up	4.67E-87	4.68E-86
mmu_piR_000802	0	215	0.001	10.66	13.37991982	Up	1.03E-84	9.44E-84
novel_pir1635	0	210	0.001	10.41	13.34568245	Up	9.17E-83	7.77E-82
novel_pir245	0	208	0.001	10.31	13.33175671	Up	5.53E-82	4.51E-81
novel_pir222	0	206	0.001	10.21	13.31769525	Up	3.34E-81	2.64E-80
novel_pir411	0	203	0.001	10.07	13.29777606	Up	4.94E-80	3.73E-79
mmu_piR_002435	0	193	0.001	9.57	13.22430321	Up	3.95E-76	2.49E-75
novel_pir1040	0	192	0.001	9.52	13.21674586	Up	9.69E-76	6.08E-75
novel_pir2198	0	179	0.001	8.88	13.11634396	Up	1.15E-70	6.27E-70
mmu_piR_001529	0	175	0.001	8.68	13.08347933	Up	4.17E-69	2.18E-68
novel_pir555	0	162	0.001	8.03	12.97118427	Up	4.93E-64	2.20E-63
novel_pir2427	0	159	0.001	7.88	12.94397991	Up	7.31E-63	3.14E-62
novel_pir1285	0	150	0.001	7.44	12.86108691	Up	2.38E-59	9.37E-59
novel_pir427	0	147	0.001	7.29	12.8317031	Up	3.52E-58	1.36E-57
novel_pir342	0	143	0.001	7.09	12.79156991	Up	1.28E-56	4.72E-56
novel_pir435	0	138	0.001	6.84	12.73978061	Up	1.15E-54	4.03E-54
novel_pir1411	0	136	0.001	6.74	12.71853288	Up	6.91E-54	2.40E-53
novel_pir1375	0	134	0.001	6.64	12.69696753	Up	4.17E-53	1.42E-52
novel_pir1941	0	133	0.001	6.59	12.68606275	Up	1.02E-52	3.46E-52
novel_pir2062	0	132	0.001	6.55	12.67727919	Up	2.51E-52	8.34E-52
novel_pir2387	0	132	0.001	6.55	12.67727919	Up	2.51E-52	8.33E-52
novel_pir2012	0	130	0.001	6.45	12.65508345	Up	1.52E-51	4.96E-51
novel_pir2336	0	128	0.001	6.35	12.63254088	Up	9.14E-51	2.92E-50

novel_pir1398	0	128	0.001	6.35	12.63254088	Up	9.14E-51	2.92E-50
novel_pir1679	0	126	0.001	6.25	12.60964047	Up	5.52E-50	1.72E-49
novel_pir328	0	126	0.001	6.25	12.60964047	Up	5.52E-50	1.72E-49
novel_pir1264	0	125	0.001	6.2	12.5980525	Up	1.35E-49	4.17E-49
novel_pir2005	0	124	0.001	6.15	12.5863707	Up	3.33E-49	1.02E-48
novel_pir1510	0	124	0.001	6.15	12.5863707	Up	3.33E-49	1.01E-48
novel_pir2396	0	117	0.001	5.8	12.50183718	Up	1.79E-46	5.28E-46
novel_pir975	0	115	0.001	5.7	12.4767462	Up	1.08E-45	3.14E-45
novel_pir1565	0	115	0.001	5.7	12.4767462	Up	1.08E-45	3.13E-45
novel_pir1626	0	114	0.001	5.65	12.46403515	Up	2.66E-45	7.64E-45
novel_pir1008	0	113	0.001	5.6	12.45121111	Up	6.53E-45	1.86E-44
novel_pir1876	0	112	0.001	5.55	12.43827206	Up	1.60E-44	4.55E-44
novel_pir1595	0	110	0.001	5.45	12.41204051	Up	9.67E-44	2.70E-43
novel_pir2435	0	108	0.001	5.36	12.38801729	Up	5.83E-43	1.60E-42
novel_pir1162	0	107	0.001	5.31	12.37449615	Up	1.43E-42	3.91E-42
novel_pir343	0	104	0.001	5.16	12.33315535	Up	2.12E-41	5.70E-41
novel_pir59	0	102	0.001	5.06	12.30492167	Up	1.28E-40	3.38E-40
novel_pir1045	0	102	0.001	5.06	12.30492167	Up	1.28E-40	3.38E-40
novel_pir295	0	102	0.001	5.06	12.30492167	Up	1.28E-40	3.37E-40
novel_pir1850	0	99	0.001	4.91	12.26150731	Up	1.90E-39	4.89E-39
novel_pir2129	0	98	0.001	4.86	12.2467406	Up	4.66E-39	1.19E-38
novel_pir852	0	96	0.001	4.76	12.21674586	Up	2.81E-38	7.14E-38
mmu_piR_038994	0	93	0.001	4.61	12.17055104	Up	4.16E-37	1.04E-36
novel_pir1331	0	93	0.001	4.61	12.17055104	Up	4.16E-37	1.04E-36
novel_pir1316	0	93	0.001	4.61	12.17055104	Up	4.16E-37	1.04E-36
novel_pir1252	0	92	0.001	4.56	12.15481811	Up	1.02E-36	2.55E-36
novel_pir648	0	89	0.001	4.41	12.10656294	Up	1.51E-35	3.72E-35
novel_pir1113	0	89	0.001	4.41	12.10656294	Up	1.51E-35	3.71E-35
novel_pir173	0	88	0.001	4.36	12.09011242	Up	3.72E-35	9.07E-35
novel_pir196	0	88	0.001	4.36	12.09011242	Up	3.72E-35	9.06E-35
novel_pir816	0	85	0.001	4.21	12.03960452	Up	5.51E-34	1.32E-33
novel_pir845	0	85	0.001	4.21	12.03960452	Up	5.51E-34	1.32E-33
novel_pir1477	0	85	0.001	4.21	12.03960452	Up	5.51E-34	1.32E-33
novel_pir2367	0	85	0.001	4.21	12.03960452	Up	5.51E-34	1.32E-33
novel_pir1363	0	85	0.001	4.21	12.03960452	Up	5.51E-34	1.31E-33
novel_pir1597	0	84	0.001	4.17	12.02583167	Up	1.35E-33	3.22E-33

novel_pir90	0	84	0.001	4.17	12.02583167	Up	1.35E-33	3.22E-33
novel_pir1540	0	83	0.001	4.12	12.00842862	Up	3.32E-33	7.84E-33
mmu_piR_038351	0	83	0.001	4.12	12.00842862	Up	3.32E-33	7.83E-33
novel_pir235	0	81	0.001	4.02	11.97297979	Up	2.00E-32	4.64E-32
novel_pir519	0	81	0.001	4.02	11.97297979	Up	2.00E-32	4.64E-32
novel_pir951	0	80	0.001	3.97	11.95492329	Up	4.92E-32	1.14E-31
novel_pir1817	0	80	0.001	3.97	11.95492329	Up	4.92E-32	1.14E-31
novel_pir2391	0	79	0.001	3.92	11.93663794	Up	1.21E-31	2.76E-31
novel_pir1137	0	79	0.001	3.92	11.93663794	Up	1.21E-31	2.76E-31
novel_pir1084	0	78	0.001	3.87	11.91811785	Up	2.97E-31	6.73E-31
novel_pir143	0	77	0.001	3.82	11.89935692	Up	7.29E-31	1.65E-30
novel_pir2207	0	76	0.001	3.77	11.88034881	Up	1.79E-30	4.01E-30
novel_pir632	0	75	0.001	3.72	11.86108691	Up	4.40E-30	9.77E-30
novel_pir199	0	75	0.001	3.72	11.86108691	Up	4.40E-30	9.76E-30
novel_pir1522	0	74	0.001	3.67	11.84156435	Up	1.08E-29	2.39E-29
novel_pir84	0	74	0.001	3.67	11.84156435	Up	1.08E-29	2.39E-29
novel_pir2179	0	74	0.001	3.67	11.84156435	Up	1.08E-29	2.38E-29
novel_pir473	0	74	0.001	3.67	11.84156435	Up	1.08E-29	2.38E-29
novel_pir887	0	73	0.001	3.62	11.82177398	Up	2.65E-29	5.83E-29
novel_pir1694	0	73	0.001	3.62	11.82177398	Up	2.65E-29	5.82E-29
novel_pir676	0	72	0.001	3.57	11.80170836	Up	6.52E-29	1.42E-28
novel_pir1587	0	72	0.001	3.57	11.80170836	Up	6.52E-29	1.42E-28
novel_pir33	0	72	0.001	3.57	11.80170836	Up	6.52E-29	1.42E-28
novel_pir486	0	70	0.001	3.47	11.76071995	Up	3.93E-28	8.46E-28
novel_pir2096	0	70	0.001	3.47	11.76071995	Up	3.93E-28	8.45E-28
novel_pir1737	0	70	0.001	3.47	11.76071995	Up	3.93E-28	8.44E-28
novel_pir253	0	69	0.001	3.42	11.73978061	Up	9.66E-28	2.06E-27
novel_pir539	0	69	0.001	3.42	11.73978061	Up	9.66E-28	2.06E-27
novel_pir402	0	69	0.001	3.42	11.73978061	Up	9.66E-28	2.06E-27
novel_pir2364	0	68	0.001	3.37	11.71853288	Up	2.37E-27	5.01E-27
novel_pir1351	0	68	0.001	3.37	11.71853288	Up	2.37E-27	5.00E-27
novel_pir755	0	67	0.001	3.32	11.69696753	Up	5.82E-27	1.22E-26
novel_pir1638	0	66	0.001	3.27	11.67507492	Up	1.43E-26	2.99E-26
novel_pir2031	0	65	0.001	3.22	11.65284497	Up	3.51E-26	7.29E-26
mmu_piR_024234	0	64	0.001	3.17	11.63026713	Up	8.63E-26	1.78E-25
novel_pir1223	0	64	0.001	3.17	11.63026713	Up	8.63E-26	1.78E-25

novel_pir1433	0	63	0.001	3.12	11.60733031	Up	2.12E-25	4.35E-25
novel_pir824	0	63	0.001	3.12	11.60733031	Up	2.12E-25	4.34E-25
novel_pir1327	0	63	0.001	3.12	11.60733031	Up	2.12E-25	4.34E-25
novel_pir1495	0	63	0.001	3.12	11.60733031	Up	2.12E-25	4.33E-25
novel_pir319	0	63	0.001	3.12	11.60733031	Up	2.12E-25	4.33E-25
novel_pir2448	0	63	0.001	3.12	11.60733031	Up	2.12E-25	4.32E-25
novel_pir431	0	63	0.001	3.12	11.60733031	Up	2.12E-25	4.32E-25
novel_pir1907	0	61	0.001	3.02	11.56033283	Up	1.28E-24	2.59E-24
novel_pir1726	0	61	0.001	3.02	11.56033283	Up	1.28E-24	2.59E-24
novel_pir2199	0	61	0.001	3.02	11.56033283	Up	1.28E-24	2.58E-24
novel_pir2327	0	60	0.001	2.98	11.54109662	Up	3.14E-24	6.31E-24
novel_pir1394	0	59	0.001	2.93	11.51668495	Up	7.71E-24	1.55E-23
novel_pir1157	0	59	0.001	2.93	11.51668495	Up	7.71E-24	1.54E-23
novel_pir1772	0	59	0.001	2.93	11.51668495	Up	7.71E-24	1.54E-23
novel_pir2076	0	59	0.001	2.93	11.51668495	Up	7.71E-24	1.54E-23
novel_pir772	0	58	0.001	2.88	11.4918531	Up	1.89E-23	3.76E-23
novel_pir1987	0	57	0.001	2.83	11.46658634	Up	4.65E-23	9.21E-23
novel_pir89	0	57	0.001	2.83	11.46658634	Up	4.65E-23	9.20E-23
novel_pir1290	0	57	0.001	2.83	11.46658634	Up	4.65E-23	9.19E-23
novel_pir1451	0	56	0.001	2.78	11.44086917	Up	1.14E-22	2.25E-22
novel_pir1955	0	56	0.001	2.78	11.44086917	Up	1.14E-22	2.24E-22
novel_pir1407	0	56	0.001	2.78	11.44086917	Up	1.14E-22	2.24E-22
novel_pir2277	0	55	0.001	2.73	11.41468524	Up	2.81E-22	5.50E-22
novel_pir487	0	55	0.001	2.73	11.41468524	Up	2.81E-22	5.49E-22
novel_pir1181	0	54	0.001	2.68	11.38801729	Up	6.89E-22	1.34E-21
novel_pir2201	0	54	0.001	2.68	11.38801729	Up	6.89E-22	1.34E-21
novel_pir1307	0	54	0.001	2.68	11.38801729	Up	6.89E-22	1.34E-21
novel_pir1568	0	54	0.001	2.68	11.38801729	Up	6.89E-22	1.34E-21
novel_pir1052	0	54	0.001	2.68	11.38801729	Up	6.89E-22	1.34E-21
novel_pir1497	0	53	0.001	2.63	11.36084708	Up	1.69E-21	3.26E-21
novel_pir740	0	53	0.001	2.63	11.36084708	Up	1.69E-21	3.26E-21
novel_pir1748	0	53	0.001	2.63	11.36084708	Up	1.69E-21	3.25E-21
novel_pir358	0	53	0.001	2.63	11.36084708	Up	1.69E-21	3.25E-21
novel_pir1567	0	52	0.001	2.58	11.33315535	Up	4.16E-21	7.97E-21
novel_pir1924	0	52	0.001	2.58	11.33315535	Up	4.16E-21	7.96E-21
novel_pir2100	0	51	0.001	2.53	11.30492167	Up	1.02E-20	1.95E-20

novel_pir1983	0	50	0.001	2.48	11.27612441	Up	2.51E-20	4.74E-20
novel_pir1435	0	50	0.001	2.48	11.27612441	Up	2.51E-20	4.74E-20
novel_pir1503	0	50	0.001	2.48	11.27612441	Up	2.51E-20	4.74E-20
novel_pir1973	0	49	0.001	2.43	11.2467406	Up	6.16E-20	1.16E-19
novel_pir265	0	49	0.001	2.43	11.2467406	Up	6.16E-20	1.16E-19
novel_pir2461	0	47	0.001	2.33	11.18611424	Up	3.71E-19	6.91E-19
novel_pir1246	0	47	0.001	2.33	11.18611424	Up	3.71E-19	6.91E-19
novel_pir1851	0	46	0.001	2.28	11.15481811	Up	9.12E-19	1.69E-18
novel_pir4	0	46	0.001	2.28	11.15481811	Up	9.12E-19	1.69E-18
novel_pir457	0	46	0.001	2.28	11.15481811	Up	9.12E-19	1.69E-18
novel_pir2027	0	46	0.001	2.28	11.15481811	Up	9.12E-19	1.69E-18
novel_pir2046	0	46	0.001	2.28	11.15481811	Up	9.12E-19	1.69E-18
novel_pir19	0	46	0.001	2.28	11.15481811	Up	9.12E-19	1.69E-18
novel_pir60	0	45	0.001	2.23	11.12282799	Up	2.24E-18	4.13E-18
novel_pir1485	0	45	0.001	2.23	11.12282799	Up	2.24E-18	4.12E-18
novel_pir1616	0	45	0.001	2.23	11.12282799	Up	2.24E-18	4.12E-18
novel_pir662	0	44	0.001	2.18	11.09011242	Up	5.50E-18	1.00E-17
novel_pir2392	0	44	0.001	2.18	11.09011242	Up	5.50E-18	1.00E-17
novel_pir2067	0	44	0.001	2.18	11.09011242	Up	5.50E-18	1.00E-17
novel_pir2369	0	44	0.001	2.18	11.09011242	Up	5.50E-18	1.00E-17
novel_pir1436	0	43	0.001	2.13	11.05663772	Up	1.35E-17	2.45E-17
novel_pir2044	0	43	0.001	2.13	11.05663772	Up	1.35E-17	2.45E-17
novel_pir671	0	42	0.001	2.08	11.02236781	Up	3.32E-17	5.98E-17
novel_pir264	0	42	0.001	2.08	11.02236781	Up	3.32E-17	5.98E-17
novel_pir185	0	42	0.001	2.08	11.02236781	Up	3.32E-17	5.97E-17
novel_pir1763	0	42	0.001	2.08	11.02236781	Up	3.32E-17	5.97E-17
novel_pir87	0	42	0.001	2.08	11.02236781	Up	3.32E-17	5.96E-17
novel_pir1546	0	41	0.001	2.03	10.98726401	Up	8.15E-17	1.46E-16
novel_pir1025	0	41	0.001	2.03	10.98726401	Up	8.15E-17	1.46E-16
novel_pir1469	0	41	0.001	2.03	10.98726401	Up	8.15E-17	1.46E-16
novel_pir1997	0	40	0.001	1.98	10.95128471	Up	2.00E-16	3.55E-16
novel_pir17	0	40	0.001	1.98	10.95128471	Up	2.00E-16	3.55E-16
novel_pir1461	0	40	0.001	1.98	10.95128471	Up	2.00E-16	3.55E-16
novel_pir421	0	40	0.001	1.98	10.95128471	Up	2.00E-16	3.54E-16
novel_pir1864	0	39	0.001	1.93	10.91438513	Up	4.92E-16	8.65E-16
novel_pir550	0	39	0.001	1.93	10.91438513	Up	4.92E-16	8.64E-16

novel_pir1856	0	39	0.001	1.93	10.91438513	Up	4.92E-16	8.63E-16
novel_pir752	0	39	0.001	1.93	10.91438513	Up	4.92E-16	8.62E-16
novel_pir165	0	38	0.001	1.88	10.87651695	Up	1.21E-15	2.11E-15
novel_pir537	0	38	0.001	1.88	10.87651695	Up	1.21E-15	2.11E-15
novel_pir2452	0	38	0.001	1.88	10.87651695	Up	1.21E-15	2.11E-15
novel_pir1771	0	38	0.001	1.88	10.87651695	Up	1.21E-15	2.11E-15
novel_pir2053	0	38	0.001	1.88	10.87651695	Up	1.21E-15	2.10E-15
novel_pir520	0	38	0.001	1.88	10.87651695	Up	1.21E-15	2.10E-15
novel_pir1704	0	38	0.001	1.88	10.87651695	Up	1.21E-15	2.10E-15
novel_pir1646	0	38	0.001	1.88	10.87651695	Up	1.21E-15	2.10E-15
novel_pir616	0	38	0.001	1.88	10.87651695	Up	1.21E-15	2.10E-15
novel_pir2462	0	38	0.001	1.88	10.87651695	Up	1.21E-15	2.09E-15
novel_pir118	0	38	0.001	1.88	10.87651695	Up	1.21E-15	2.09E-15
novel_pir991	0	37	0.001	1.83	10.83762793	Up	2.97E-15	5.12E-15
novel_pir512	0	37	0.001	1.83	10.83762793	Up	2.97E-15	5.11E-15
novel_pir177	0	37	0.001	1.83	10.83762793	Up	2.97E-15	5.11E-15
novel_pir1559	0	37	0.001	1.83	10.83762793	Up	2.97E-15	5.10E-15
novel_pir685	0	37	0.001	1.83	10.83762793	Up	2.97E-15	5.10E-15
novel_pir1265	0	37	0.001	1.83	10.83762793	Up	2.97E-15	5.09E-15
novel_pir1166	0	36	0.001	1.79	10.80574387	Up	7.29E-15	1.24E-14
novel_pir2450	0	36	0.001	1.79	10.80574387	Up	7.29E-15	1.24E-14
novel_pir1456	0	36	0.001	1.79	10.80574387	Up	7.29E-15	1.24E-14
novel_pir1532	0	35	0.001	1.74	10.76487159	Up	1.79E-14	3.04E-14
novel_pir2349	0	35	0.001	1.74	10.76487159	Up	1.79E-14	3.04E-14
novel_pir1747	0	35	0.001	1.74	10.76487159	Up	1.79E-14	3.04E-14
novel_pir2058	0	35	0.001	1.74	10.76487159	Up	1.79E-14	3.04E-14
novel_pir1474	0	35	0.001	1.74	10.76487159	Up	1.79E-14	3.03E-14
novel_pir1733	0	35	0.001	1.74	10.76487159	Up	1.79E-14	3.03E-14
novel_pir212	0	35	0.001	1.74	10.76487159	Up	1.79E-14	3.03E-14
novel_pir1895	0	35	0.001	1.74	10.76487159	Up	1.79E-14	3.02E-14
novel_pir1885	0	34	0.001	1.69	10.72280753	Up	4.39E-14	7.40E-14
novel_pir1478	0	34	0.001	1.69	10.72280753	Up	4.39E-14	7.39E-14
novel_pir2047	0	34	0.001	1.69	10.72280753	Up	4.39E-14	7.38E-14
novel_pir1665	0	34	0.001	1.69	10.72280753	Up	4.39E-14	7.38E-14
novel_pir888	0	34	0.001	1.69	10.72280753	Up	4.39E-14	7.37E-14
novel_pir1195	0	34	0.001	1.69	10.72280753	Up	4.39E-14	7.36E-14

novel_pir376	0	34	0.001	1.69	10.72280753	Up	4.39E-14	7.36E-14
novel_pir834	0	34	0.001	1.69	10.72280753	Up	4.39E-14	7.35E-14
novel_pir197	0	34	0.001	1.69	10.72280753	Up	4.39E-14	7.34E-14
novel_pir1111	0	34	0.001	1.69	10.72280753	Up	4.39E-14	7.34E-14
novel_pir898	0	34	0.001	1.69	10.72280753	Up	4.39E-14	7.33E-14
novel_pir276	0	34	0.001	1.69	10.72280753	Up	4.39E-14	7.33E-14
novel_pir1370	0	33	0.001	1.64	10.6794801	Up	1.08E-13	1.79E-13
mmu_piR_038258	0	33	0.001	1.64	10.6794801	Up	1.08E-13	1.79E-13
novel_pir154	0	33	0.001	1.64	10.6794801	Up	1.08E-13	1.78E-13
novel_pir345	0	33	0.001	1.64	10.6794801	Up	1.08E-13	1.78E-13
novel_pir768	0	33	0.001	1.64	10.6794801	Up	1.08E-13	1.78E-13
novel_pir388	0	33	0.001	1.64	10.6794801	Up	1.08E-13	1.78E-13
novel_pir445	0	32	0.001	1.59	10.63481105	Up	2.65E-13	4.34E-13
novel_pir2303	0	32	0.001	1.59	10.63481105	Up	2.65E-13	4.34E-13
novel_pir1682	0	32	0.001	1.59	10.63481105	Up	2.65E-13	4.34E-13
novel_pir2451	0	32	0.001	1.59	10.63481105	Up	2.65E-13	4.33E-13
novel_pir2088	0	32	0.001	1.59	10.63481105	Up	2.65E-13	4.33E-13
novel_pir2338	0	32	0.001	1.59	10.63481105	Up	2.65E-13	4.33E-13
novel_pir1541	0	32	0.001	1.59	10.63481105	Up	2.65E-13	4.32E-13
novel_pir1015	0	31	0.001	1.54	10.58871464	Up	6.51E-13	1.05E-12
novel_pir2121	0	31	0.001	1.54	10.58871464	Up	6.51E-13	1.05E-12
novel_pir1016	0	31	0.001	1.54	10.58871464	Up	6.51E-13	1.05E-12
novel_pir2376	0	31	0.001	1.54	10.58871464	Up	6.51E-13	1.05E-12
novel_pir2036	0	31	0.001	1.54	10.58871464	Up	6.51E-13	1.05E-12
novel_pir1693	0	31	0.001	1.54	10.58871464	Up	6.51E-13	1.05E-12
novel_pir1337	0	31	0.001	1.54	10.58871464	Up	6.51E-13	1.05E-12
novel_pir1957	0	31	0.001	1.54	10.58871464	Up	6.51E-13	1.05E-12
novel_pir921	0	30	0.001	1.49	10.54109662	Up	1.60E-12	2.57E-12
novel_pir1596	0	30	0.001	1.49	10.54109662	Up	1.60E-12	2.56E-12
novel_pir725	0	30	0.001	1.49	10.54109662	Up	1.60E-12	2.56E-12
novel_pir28	0	30	0.001	1.49	10.54109662	Up	1.60E-12	2.56E-12
novel_pir695	0	29	0.001	1.44	10.4918531	Up	3.93E-12	6.24E-12
novel_pir2356	0	29	0.001	1.44	10.4918531	Up	3.93E-12	6.23E-12
novel_pir2446	0	29	0.001	1.44	10.4918531	Up	3.93E-12	6.23E-12
novel_pir2235	0	29	0.001	1.44	10.4918531	Up	3.93E-12	6.22E-12
novel_pir1438	0	29	0.001	1.44	10.4918531	Up	3.93E-12	6.22E-12

novel_pir1058	0	29	0.001	1.44	10.4918531	Up	3.93E-12	6.21E-12
novel_pir524	0	29	0.001	1.44	10.4918531	Up	3.93E-12	6.21E-12
novel_pir192	0	29	0.001	1.44	10.4918531	Up	3.93E-12	6.20E-12
novel_pir446	0	29	0.001	1.44	10.4918531	Up	3.93E-12	6.20E-12
novel_pir703	0	29	0.001	1.44	10.4918531	Up	3.93E-12	6.19E-12
novel_pir665	0	29	0.001	1.44	10.4918531	Up	3.93E-12	6.19E-12
novel_pir2041	0	29	0.001	1.44	10.4918531	Up	3.93E-12	6.18E-12
novel_pir1383	0	28	0.001	1.39	10.44086917	Up	9.65E-12	1.51E-11
novel_pir741	0	28	0.001	1.39	10.44086917	Up	9.65E-12	1.51E-11
novel_pir1434	0	28	0.001	1.39	10.44086917	Up	9.65E-12	1.51E-11
novel_pir1653	0	28	0.001	1.39	10.44086917	Up	9.65E-12	1.51E-11
novel_pir1282	0	28	0.001	1.39	10.44086917	Up	9.65E-12	1.51E-11
novel_pir2000	0	28	0.001	1.39	10.44086917	Up	9.65E-12	1.50E-11
novel_pir598	0	28	0.001	1.39	10.44086917	Up	9.65E-12	1.50E-11
novel_pir1205	0	28	0.001	1.39	10.44086917	Up	9.65E-12	1.50E-11
novel_pir1827	0	28	0.001	1.39	10.44086917	Up	9.65E-12	1.50E-11
novel_pir32	0	28	0.001	1.39	10.44086917	Up	9.65E-12	1.50E-11
novel_pir2004	0	28	0.001	1.39	10.44086917	Up	9.65E-12	1.50E-11
novel_pir982	0	28	0.001	1.39	10.44086917	Up	9.65E-12	1.50E-11
novel_pir1301	0	28	0.001	1.39	10.44086917	Up	9.65E-12	1.50E-11
novel_pir215	0	27	0.001	1.34	10.38801729	Up	2.37E-11	3.66E-11
novel_pir1902	0	27	0.001	1.34	10.38801729	Up	2.37E-11	3.66E-11
novel_pir91	0	27	0.001	1.34	10.38801729	Up	2.37E-11	3.66E-11
novel_pir1364	0	27	0.001	1.34	10.38801729	Up	2.37E-11	3.66E-11
novel_pir291	0	27	0.001	1.34	10.38801729	Up	2.37E-11	3.65E-11
novel_pir2092	0	27	0.001	1.34	10.38801729	Up	2.37E-11	3.65E-11
novel_pir220	0	27	0.001	1.34	10.38801729	Up	2.37E-11	3.65E-11
novel_pir1077	0	26	0.001	1.29	10.33315535	Up	5.82E-11	8.90E-11
novel_pir1594	0	26	0.001	1.29	10.33315535	Up	5.82E-11	8.89E-11
novel_pir15	0	26	0.001	1.29	10.33315535	Up	5.82E-11	8.89E-11
novel_pir708	0	26	0.001	1.29	10.33315535	Up	5.82E-11	8.88E-11
novel_pir1538	0	26	0.001	1.29	10.33315535	Up	5.82E-11	8.87E-11
novel_pir490	0	26	0.001	1.29	10.33315535	Up	5.82E-11	8.86E-11
novel_pir1818	0	26	0.001	1.29	10.33315535	Up	5.82E-11	8.86E-11
novel_pir1878	0	26	0.001	1.29	10.33315535	Up	5.82E-11	8.85E-11
novel_pir1896	0	26	0.001	1.29	10.33315535	Up	5.82E-11	8.84E-11

novel_pir1545	0	26	0.001	1.29	10.33315535	Up	5.82E-11	8.84E-11
novel_pir793	0	26	0.001	1.29	10.33315535	Up	5.82E-11	8.83E-11
novel_pir85	0	26	0.001	1.29	10.33315535	Up	5.82E-11	8.82E-11
novel_pir523	0	26	0.001	1.29	10.33315535	Up	5.82E-11	8.82E-11
novel_pir24	0	26	0.001	1.29	10.33315535	Up	5.82E-11	8.81E-11
novel_pir2250	0	25	0.001	1.24	10.27612441	Up	1.43E-10	2.15E-10
novel_pir2200	0	25	0.001	1.24	10.27612441	Up	1.43E-10	2.14E-10
novel_pir588	0	25	0.001	1.24	10.27612441	Up	1.43E-10	2.14E-10
novel_pir1623	0	25	0.001	1.24	10.27612441	Up	1.43E-10	2.14E-10
novel_pir890	0	25	0.001	1.24	10.27612441	Up	1.43E-10	2.14E-10
novel_pir2281	0	25	0.001	1.24	10.27612441	Up	1.43E-10	2.14E-10
novel_pir1807	0	25	0.001	1.24	10.27612441	Up	1.43E-10	2.14E-10
novel_pir1062	0	25	0.001	1.24	10.27612441	Up	1.43E-10	2.13E-10
novel_pir2293	0	25	0.001	1.24	10.27612441	Up	1.43E-10	2.13E-10
novel_pir437	0	25	0.001	1.24	10.27612441	Up	1.43E-10	2.13E-10
novel_pir494	0	25	0.001	1.24	10.27612441	Up	1.43E-10	2.13E-10
novel_pir517	0	25	0.001	1.24	10.27612441	Up	1.43E-10	2.13E-10
novel_pir1030	0	25	0.001	1.24	10.27612441	Up	1.43E-10	2.13E-10
novel_pir1964	0	25	0.001	1.24	10.27612441	Up	1.43E-10	2.12E-10
novel_pir854	0	25	0.001	1.24	10.27612441	Up	1.43E-10	2.12E-10
novel_pir1945	0	24	0.001	1.19	10.21674586	Up	3.51E-10	5.17E-10
novel_pir819	0	24	0.001	1.19	10.21674586	Up	3.51E-10	5.16E-10
novel_pir1586	0	24	0.001	1.19	10.21674586	Up	3.51E-10	5.16E-10
novel_pir2287	0	24	0.001	1.19	10.21674586	Up	3.51E-10	5.16E-10
novel_pir2318	0	24	0.001	1.19	10.21674586	Up	3.51E-10	5.15E-10
novel_pir730	0	24	0.001	1.19	10.21674586	Up	3.51E-10	5.15E-10
novel_pir2014	0	24	0.001	1.19	10.21674586	Up	3.51E-10	5.14E-10
novel_pir839	0	24	0.001	1.19	10.21674586	Up	3.51E-10	5.14E-10
novel_pir2006	0	24	0.001	1.19	10.21674586	Up	3.51E-10	5.14E-10
novel_pir18	0	24	0.001	1.19	10.21674586	Up	3.51E-10	5.13E-10
novel_pir75	0	24	0.001	1.19	10.21674586	Up	3.51E-10	5.13E-10
novel_pir601	0	24	0.001	1.19	10.21674586	Up	3.51E-10	5.12E-10
novel_pir193	0	24	0.001	1.19	10.21674586	Up	3.51E-10	5.12E-10
novel_pir1024	0	24	0.001	1.19	10.21674586	Up	3.51E-10	5.12E-10
novel_pir674	0	24	0.001	1.19	10.21674586	Up	3.51E-10	5.11E-10
novel_pir686	0	24	0.001	1.19	10.21674586	Up	3.51E-10	5.11E-10

novel_pir1759	0	24	0.001	1.19	10.21674586	Up	3.51E-10	5.11E-10
novel_pir2068	0	23	0.001	1.14	10.15481811	Up	8.62E-10	1.25E-09
novel_pir1828	0	23	0.001	1.14	10.15481811	Up	8.62E-10	1.25E-09
novel_pir2380	0	23	0.001	1.14	10.15481811	Up	8.62E-10	1.25E-09
novel_pir1731	0	23	0.001	1.14	10.15481811	Up	8.62E-10	1.25E-09
novel_pir569	0	23	0.001	1.14	10.15481811	Up	8.62E-10	1.24E-09
novel_pir1792	0	23	0.001	1.14	10.15481811	Up	8.62E-10	1.24E-09
novel_pir2022	0	23	0.001	1.14	10.15481811	Up	8.62E-10	1.24E-09
novel_pir159	0	23	0.001	1.14	10.15481811	Up	8.62E-10	1.24E-09
novel_pir1893	0	23	0.001	1.14	10.15481811	Up	8.62E-10	1.24E-09
novel_pir2382	0	23	0.001	1.14	10.15481811	Up	8.62E-10	1.24E-09
novel_pir468	0	23	0.001	1.14	10.15481811	Up	8.62E-10	1.24E-09
novel_pir474	0	23	0.001	1.14	10.15481811	Up	8.62E-10	1.24E-09
novel_pir2421	0	23	0.001	1.14	10.15481811	Up	8.62E-10	1.24E-09
novel_pir233	0	23	0.001	1.14	10.15481811	Up	8.62E-10	1.24E-09
novel_pir659	0	22	0.001	1.09	10.09011242	Up	2.12E-09	3.01E-09
novel_pir1492	0	22	0.001	1.09	10.09011242	Up	2.12E-09	3.01E-09
novel_pir353	0	22	0.001	1.09	10.09011242	Up	2.12E-09	3.01E-09
novel_pir1107	0	22	0.001	1.09	10.09011242	Up	2.12E-09	3.01E-09
novel_pir2278	0	22	0.001	1.09	10.09011242	Up	2.12E-09	3.00E-09
novel_pir428	0	22	0.001	1.09	10.09011242	Up	2.12E-09	3.00E-09
novel_pir1284	0	22	0.001	1.09	10.09011242	Up	2.12E-09	3.00E-09
novel_pir2135	0	22	0.001	1.09	10.09011242	Up	2.12E-09	3.00E-09
novel_pir1766	0	22	0.001	1.09	10.09011242	Up	2.12E-09	2.99E-09
novel_pir217	0	22	0.001	1.09	10.09011242	Up	2.12E-09	2.99E-09
novel_pir1314	0	22	0.001	1.09	10.09011242	Up	2.12E-09	2.99E-09
mmu_piR_027027	0	21	0.001	1.04	10.02236781	Up	5.20E-09	7.32E-09
novel_pir1926	0	21	0.001	1.04	10.02236781	Up	5.20E-09	7.31E-09
mmu_piR_018376	0	21	0.001	1.04	10.02236781	Up	5.20E-09	7.31E-09
novel_pir2247	0	21	0.001	1.04	10.02236781	Up	5.20E-09	7.30E-09
novel_pir792	0	21	0.001	1.04	10.02236781	Up	5.20E-09	7.29E-09
novel_pir1354	0	21	0.001	1.04	10.02236781	Up	5.20E-09	7.29E-09
mmu_piR_022918	0	21	0.001	1.04	10.02236781	Up	5.20E-09	7.28E-09
novel_pir1199	0	21	0.001	1.04	10.02236781	Up	5.20E-09	7.28E-09
novel_pir202	0	21	0.001	1.04	10.02236781	Up	5.20E-09	7.27E-09
novel_pir463	0	21	0.001	1.04	10.02236781	Up	5.20E-09	7.27E-09

novel_pir1608	0	20	0.001	0.99	9.951284715	Up	1.28E-08	1.78E-08
novel_pir946	0	20	0.001	0.99	9.951284715	Up	1.28E-08	1.78E-08
novel_pir2136	0	20	0.001	0.99	9.951284715	Up	1.28E-08	1.77E-08
novel_pir1507	0	20	0.001	0.99	9.951284715	Up	1.28E-08	1.77E-08
novel_pir1511	0	20	0.001	0.99	9.951284715	Up	1.28E-08	1.77E-08
novel_pir1021	0	20	0.001	0.99	9.951284715	Up	1.28E-08	1.77E-08
novel_pir1131	0	20	0.001	0.99	9.951284715	Up	1.28E-08	1.77E-08
novel_pir2048	0	20	0.001	0.99	9.951284715	Up	1.28E-08	1.77E-08
novel_pir1097	0	20	0.001	0.99	9.951284715	Up	1.28E-08	1.77E-08
mmu_piR_024221	0	20	0.001	0.99	9.951284715	Up	1.28E-08	1.77E-08
novel_pir433	0	20	0.001	0.99	9.951284715	Up	1.28E-08	1.76E-08
novel_pir958	0	20	0.001	0.99	9.951284715	Up	1.28E-08	1.76E-08
novel_pir1005	0	20	0.001	0.99	9.951284715	Up	1.28E-08	1.76E-08
novel_pir673	0	20	0.001	0.99	9.951284715	Up	1.28E-08	1.76E-08
novel_pir1700	0	20	0.001	0.99	9.951284715	Up	1.28E-08	1.76E-08
novel_pir964	0	20	0.001	0.99	9.951284715	Up	1.28E-08	1.76E-08
novel_pir2223	0	20	0.001	0.99	9.951284715	Up	1.28E-08	1.76E-08
novel_pir906	0	19	0.001	0.94	9.876516947	Up	3.14E-08	4.30E-08
novel_pir2301	0	19	0.001	0.94	9.876516947	Up	3.14E-08	4.29E-08
novel_pir757	0	19	0.001	0.94	9.876516947	Up	3.14E-08	4.29E-08
novel_pir88	0	19	0.001	0.94	9.876516947	Up	3.14E-08	4.29E-08
novel_pir1803	0	19	0.001	0.94	9.876516947	Up	3.14E-08	4.29E-08
novel_pir1824	0	19	0.001	0.94	9.876516947	Up	3.14E-08	4.28E-08
mmu_piR_004567	0	19	0.001	0.94	9.876516947	Up	3.14E-08	4.28E-08
novel_pir1963	0	19	0.001	0.94	9.876516947	Up	3.14E-08	4.28E-08
novel_pir1115	0	19	0.001	0.94	9.876516947	Up	3.14E-08	4.27E-08
novel_pir2159	0	19	0.001	0.94	9.876516947	Up	3.14E-08	4.27E-08
novel_pir1031	0	19	0.001	0.94	9.876516947	Up	3.14E-08	4.27E-08
novel_pir1256	0	19	0.001	0.94	9.876516947	Up	3.14E-08	4.26E-08
novel_pir1043	0	19	0.001	0.94	9.876516947	Up	3.14E-08	4.26E-08
novel_pir731	0	19	0.001	0.94	9.876516947	Up	3.14E-08	4.26E-08
novel_pir1226	0	19	0.001	0.94	9.876516947	Up	3.14E-08	4.25E-08
novel_pir503	0	19	0.001	0.94	9.876516947	Up	3.14E-08	4.25E-08
novel_pir219	0	19	0.001	0.94	9.876516947	Up	3.14E-08	4.25E-08
novel_pir1057	0	19	0.001	0.94	9.876516947	Up	3.14E-08	4.25E-08
novel_pir352	0	19	0.001	0.94	9.876516947	Up	3.14E-08	4.24E-08

novel_pir1610	0	18	0.001	0.89	9.797661526	Up	7.70E-08	1.04E-07
novel_pir797	0	18	0.001	0.89	9.797661526	Up	7.70E-08	1.03E-07
novel_pir93	0	18	0.001	0.89	9.797661526	Up	7.70E-08	1.03E-07
novel_pir826	0	18	0.001	0.89	9.797661526	Up	7.70E-08	1.03E-07
novel_pir1267	0	18	0.001	0.89	9.797661526	Up	7.70E-08	1.03E-07
novel_pir2400	0	18	0.001	0.89	9.797661526	Up	7.70E-08	1.03E-07
novel_pir218	0	18	0.001	0.89	9.797661526	Up	7.70E-08	1.03E-07
novel_pir2335	0	18	0.001	0.89	9.797661526	Up	7.70E-08	1.03E-07
novel_pir2453	0	18	0.001	0.89	9.797661526	Up	7.70E-08	1.03E-07
novel_pir2388	0	18	0.001	0.89	9.797661526	Up	7.70E-08	1.03E-07
novel_pir1927	0	18	0.001	0.89	9.797661526	Up	7.70E-08	1.03E-07
novel_pir1671	0	18	0.001	0.89	9.797661526	Up	7.70E-08	1.03E-07
novel_pir2240	0	18	0.001	0.89	9.797661526	Up	7.70E-08	1.03E-07
novel_pir321	0	18	0.001	0.89	9.797661526	Up	7.70E-08	1.03E-07
novel_pir1543	0	18	0.001	0.89	9.797661526	Up	7.70E-08	1.03E-07
novel_pir1624	0	18	0.001	0.89	9.797661526	Up	7.70E-08	1.02E-07
novel_pir668	0	18	0.001	0.89	9.797661526	Up	7.70E-08	1.02E-07
novel_pir1583	0	18	0.001	0.89	9.797661526	Up	7.70E-08	1.02E-07
novel_pir2312	0	18	0.001	0.89	9.797661526	Up	7.70E-08	1.02E-07
novel_pir815	0	18	0.001	0.89	9.797661526	Up	7.70E-08	1.02E-07
novel_pir2467	0	17	0.001	0.84	9.714245518	Up	1.89E-07	2.50E-07
novel_pir1288	0	17	0.001	0.84	9.714245518	Up	1.89E-07	2.50E-07
novel_pir570	0	17	0.001	0.84	9.714245518	Up	1.89E-07	2.50E-07
novel_pir1360	0	17	0.001	0.84	9.714245518	Up	1.89E-07	2.50E-07
novel_pir791	0	17	0.001	0.84	9.714245518	Up	1.89E-07	2.49E-07
novel_pir963	0	17	0.001	0.84	9.714245518	Up	1.89E-07	2.49E-07
novel_pir1326	0	17	0.001	0.84	9.714245518	Up	1.89E-07	2.49E-07
novel_pir1358	0	17	0.001	0.84	9.714245518	Up	1.89E-07	2.49E-07
novel_pir1141	0	17	0.001	0.84	9.714245518	Up	1.89E-07	2.49E-07
novel_pir1324	0	17	0.001	0.84	9.714245518	Up	1.89E-07	2.49E-07
novel_pir2381	0	17	0.001	0.84	9.714245518	Up	1.89E-07	2.48E-07
novel_pir2415	0	17	0.001	0.84	9.714245518	Up	1.89E-07	2.48E-07
novel_pir2333	0	17	0.001	0.84	9.714245518	Up	1.89E-07	2.48E-07
novel_pir1004	0	17	0.001	0.84	9.714245518	Up	1.89E-07	2.48E-07
novel_pir1333	0	17	0.001	0.84	9.714245518	Up	1.89E-07	2.48E-07
novel_pir2304	0	17	0.001	0.84	9.714245518	Up	1.89E-07	2.48E-07

novel_pir2204	0	17	0.001	0.84	9.714245518	Up	1.89E-07	2.47E-07
novel_pir1237	0	17	0.001	0.84	9.714245518	Up	1.89E-07	2.47E-07
novel_pir1439	0	17	0.001	0.84	9.714245518	Up	1.89E-07	2.47E-07
novel_pir2090	0	17	0.001	0.84	9.714245518	Up	1.89E-07	2.47E-07
novel_pir640	0	17	0.001	0.84	9.714245518	Up	1.89E-07	2.47E-07
novel_pir78	0	17	0.001	0.84	9.714245518	Up	1.89E-07	2.47E-07
novel_pir920	0	16	0.001	0.79	9.625708843	Up	4.65E-07	6.04E-07
novel_pir2424	0	16	0.001	0.79	9.625708843	Up	4.65E-07	6.03E-07
novel_pir1376	0	16	0.001	0.79	9.625708843	Up	4.65E-07	6.03E-07
novel_pir156	0	16	0.001	0.79	9.625708843	Up	4.65E-07	6.02E-07
novel_pir101	0	16	0.001	0.79	9.625708843	Up	4.65E-07	6.02E-07
novel_pir1573	0	16	0.001	0.79	9.625708843	Up	4.65E-07	6.02E-07
novel_pir378	0	16	0.001	0.79	9.625708843	Up	4.65E-07	6.01E-07
novel_pir790	0	16	0.001	0.79	9.625708843	Up	4.65E-07	6.01E-07
novel_pir475	0	16	0.001	0.79	9.625708843	Up	4.65E-07	6.00E-07
novel_pir1343	0	16	0.001	0.79	9.625708843	Up	4.65E-07	6.00E-07
novel_pir544	0	16	0.001	0.79	9.625708843	Up	4.65E-07	6.00E-07
novel_pir1489	0	16	0.001	0.79	9.625708843	Up	4.65E-07	5.99E-07
novel_pir1177	0	16	0.001	0.79	9.625708843	Up	4.65E-07	5.99E-07
novel_pir1154	0	16	0.001	0.79	9.625708843	Up	4.65E-07	5.98E-07
novel_pir833	0	16	0.001	0.79	9.625708843	Up	4.65E-07	5.98E-07
novel_pir2379	0	16	0.001	0.79	9.625708843	Up	4.65E-07	5.98E-07
novel_pir174	0	16	0.001	0.79	9.625708843	Up	4.65E-07	5.97E-07
novel_pir1823	0	16	0.001	0.79	9.625708843	Up	4.65E-07	5.97E-07
novel_pir356	0	16	0.001	0.79	9.625708843	Up	4.65E-07	5.96E-07
novel_pir2071	0	16	0.001	0.79	9.625708843	Up	4.65E-07	5.96E-07
novel_pir856	0	16	0.001	0.79	9.625708843	Up	4.65E-07	5.96E-07
novel_pir670	0	16	0.001	0.79	9.625708843	Up	4.65E-07	5.95E-07
novel_pir1039	0	16	0.001	0.79	9.625708843	Up	4.65E-07	5.95E-07
novel_pir1842	0	15	0.001	0.74	9.531381461	Up	1.14E-06	1.45E-06
novel_pir2429	0	15	0.001	0.74	9.531381461	Up	1.14E-06	1.45E-06
novel_pir1899	0	15	0.001	0.74	9.531381461	Up	1.14E-06	1.45E-06
novel_pir151	0	15	0.001	0.74	9.531381461	Up	1.14E-06	1.45E-06
novel_pir413	0	15	0.001	0.74	9.531381461	Up	1.14E-06	1.45E-06
novel_pir1729	0	15	0.001	0.74	9.531381461	Up	1.14E-06	1.45E-06
novel_pir653	0	15	0.001	0.74	9.531381461	Up	1.14E-06	1.45E-06

novel_pir1867	0	15	0.001	0.74	9.531381461	Up	1.14E-06	1.44E-06
novel_pir1915	0	15	0.001	0.74	9.531381461	Up	1.14E-06	1.44E-06
novel_pir2334	0	15	0.001	0.74	9.531381461	Up	1.14E-06	1.44E-06
novel_pir1751	0	15	0.001	0.74	9.531381461	Up	1.14E-06	1.44E-06
novel_pir429	0	15	0.001	0.74	9.531381461	Up	1.14E-06	1.44E-06
novel_pir1365	0	15	0.001	0.74	9.531381461	Up	1.14E-06	1.44E-06
novel_pir214	0	15	0.001	0.74	9.531381461	Up	1.14E-06	1.44E-06
novel_pir1090	0	15	0.001	0.74	9.531381461	Up	1.14E-06	1.44E-06
novel_pir1099	0	15	0.001	0.74	9.531381461	Up	1.14E-06	1.44E-06
novel_pir1210	0	15	0.001	0.74	9.531381461	Up	1.14E-06	1.44E-06
novel_pir823	0	15	0.001	0.74	9.531381461	Up	1.14E-06	1.44E-06
novel_pir837	0	15	0.001	0.74	9.531381461	Up	1.14E-06	1.43E-06
novel_pir1825	0	15	0.001	0.74	9.531381461	Up	1.14E-06	1.43E-06
novel_pir1174	0	14	0.001	0.69	9.430452552	Up	2.80E-06	3.49E-06
novel_pir2282	0	14	0.001	0.69	9.430452552	Up	2.80E-06	3.49E-06
novel_pir1116	0	14	0.001	0.69	9.430452552	Up	2.80E-06	3.48E-06
novel_pir292	0	14	0.001	0.69	9.430452552	Up	2.80E-06	3.48E-06
novel_pir902	0	14	0.001	0.69	9.430452552	Up	2.80E-06	3.48E-06
novel_pir1038	0	14	0.001	0.69	9.430452552	Up	2.80E-06	3.48E-06
novel_pir1381	0	14	0.001	0.69	9.430452552	Up	2.80E-06	3.48E-06
novel_pir956	0	14	0.001	0.69	9.430452552	Up	2.80E-06	3.47E-06
novel_pir1020	0	14	0.001	0.69	9.430452552	Up	2.80E-06	3.47E-06
novel_pir511	0	14	0.001	0.69	9.430452552	Up	2.80E-06	3.47E-06
novel_pir1313	0	14	0.001	0.69	9.430452552	Up	2.80E-06	3.47E-06
novel_pir1962	0	14	0.001	0.69	9.430452552	Up	2.80E-06	3.46E-06
novel_pir160	0	14	0.001	0.69	9.430452552	Up	2.80E-06	3.46E-06
novel_pir1951	0	14	0.001	0.69	9.430452552	Up	2.80E-06	3.46E-06
novel_pir466	0	14	0.001	0.69	9.430452552	Up	2.80E-06	3.46E-06
novel_pir1551	0	14	0.001	0.69	9.430452552	Up	2.80E-06	3.46E-06
novel_pir2447	0	14	0.001	0.69	9.430452552	Up	2.80E-06	3.45E-06
novel_pir1917	0	14	0.001	0.69	9.430452552	Up	2.80E-06	3.45E-06
novel_pir2353	0	14	0.001	0.69	9.430452552	Up	2.80E-06	3.45E-06
novel_pir2113	0	14	0.001	0.69	9.430452552	Up	2.80E-06	3.45E-06
novel_pir370	0	14	0.001	0.69	9.430452552	Up	2.80E-06	3.44E-06
novel_pir1956	0	14	0.001	0.69	9.430452552	Up	2.80E-06	3.44E-06
novel_pir1292	0	14	0.001	0.69	9.430452552	Up	2.80E-06	3.44E-06

novel_pir1847	0	14	0.001	0.69	9.430452552	Up	2.80E-06	3.44E-06
novel_pir268	0	14	0.001	0.69	9.430452552	Up	2.80E-06	3.44E-06
novel_pir1075	0	14	0.001	0.69	9.430452552	Up	2.80E-06	3.43E-06
novel_pir1308	0	13	0.001	0.64	9.321928095	Up	6.88E-06	8.38E-06
novel_pir1858	0	13	0.001	0.64	9.321928095	Up	6.88E-06	8.37E-06
novel_pir2023	0	13	0.001	0.64	9.321928095	Up	6.88E-06	8.37E-06
novel_pir1909	0	13	0.001	0.64	9.321928095	Up	6.88E-06	8.36E-06
novel_pir2329	0	13	0.001	0.64	9.321928095	Up	6.88E-06	8.36E-06
novel_pir737	0	13	0.001	0.64	9.321928095	Up	6.88E-06	8.35E-06
novel_pir163	0	13	0.001	0.64	9.321928095	Up	6.88E-06	8.35E-06
novel_pir773	0	13	0.001	0.64	9.321928095	Up	6.88E-06	8.34E-06
novel_pir2245	0	13	0.001	0.64	9.321928095	Up	6.88E-06	8.34E-06
novel_pir881	0	13	0.001	0.64	9.321928095	Up	6.88E-06	8.33E-06
novel_pir1765	0	13	0.001	0.64	9.321928095	Up	6.88E-06	8.33E-06
mmu_piR_037320	0	13	0.001	0.64	9.321928095	Up	6.88E-06	8.32E-06
novel_pir586	0	13	0.001	0.64	9.321928095	Up	6.88E-06	8.32E-06
novel_pir1673	0	13	0.001	0.64	9.321928095	Up	6.88E-06	8.31E-06
novel_pir271	0	13	0.001	0.64	9.321928095	Up	6.88E-06	8.31E-06
novel_pir698	0	13	0.001	0.64	9.321928095	Up	6.88E-06	8.30E-06
novel_pir2074	0	13	0.001	0.64	9.321928095	Up	6.88E-06	8.30E-06
novel_pir1121	0	13	0.001	0.64	9.321928095	Up	6.88E-06	8.29E-06
novel_pir1255	0	13	0.001	0.64	9.321928095	Up	6.88E-06	8.29E-06
novel_pir1898	0	13	0.001	0.64	9.321928095	Up	6.88E-06	8.28E-06
novel_pir1127	0	13	0.001	0.64	9.321928095	Up	6.88E-06	8.28E-06
novel_pir1158	0	13	0.001	0.64	9.321928095	Up	6.88E-06	8.27E-06
novel_pir2211	0	13	0.001	0.64	9.321928095	Up	6.88E-06	8.26E-06
novel_pir269	0	13	0.001	0.64	9.321928095	Up	6.88E-06	8.26E-06
novel_pir216	0	13	0.001	0.64	9.321928095	Up	6.88E-06	8.25E-06
novel_pir2003	0	13	0.001	0.64	9.321928095	Up	6.88E-06	8.25E-06
novel_pir1562	0	13	0.001	0.64	9.321928095	Up	6.88E-06	8.24E-06
novel_pir208	0	13	0.001	0.64	9.321928095	Up	6.88E-06	8.24E-06
novel_pir633	0	13	0.001	0.64	9.321928095	Up	6.88E-06	8.23E-06
novel_pir536	0	13	0.001	0.64	9.321928095	Up	6.88E-06	8.23E-06
novel_pir1227	0	13	0.001	0.64	9.321928095	Up	6.88E-06	8.22E-06
novel_pir2057	0	13	0.001	0.64	9.321928095	Up	6.88E-06	8.22E-06
novel_pir316	0	13	0.001	0.64	9.321928095	Up	6.88E-06	8.21E-06

novel_pir1701	0	13	0.001	0.64	9.321928095	Up	6.88E-06	8.21E-06
novel_pir2368	0	13	0.001	0.64	9.321928095	Up	6.88E-06	8.20E-06
novel_pir999	0	13	0.001	0.64	9.321928095	Up	6.88E-06	8.20E-06
novel_pir1144	0	13	0.001	0.64	9.321928095	Up	6.88E-06	8.19E-06
novel_pir1548	0	13	0.001	0.64	9.321928095	Up	6.88E-06	8.19E-06
novel_pir2309	0	13	0.001	0.64	9.321928095	Up	6.88E-06	8.18E-06
novel_pir1341	0	13	0.001	0.64	9.321928095	Up	6.88E-06	8.18E-06
novel_pir572	0	12	0.001	0.6	9.22881869	Up	1.69E-05	1.99E-05
novel_pir415	0	12	0.001	0.6	9.22881869	Up	1.69E-05	1.99E-05
novel_pir2438	0	12	0.001	0.6	9.22881869	Up	1.69E-05	1.99E-05
novel_pir1460	0	12	0.001	0.6	9.22881869	Up	1.69E-05	1.99E-05
novel_pir338	0	12	0.001	0.6	9.22881869	Up	1.69E-05	1.99E-05
novel_pir787	0	12	0.001	0.6	9.22881869	Up	1.69E-05	1.99E-05
novel_pir1515	0	12	0.001	0.6	9.22881869	Up	1.69E-05	1.99E-05
novel_pir609	0	12	0.001	0.6	9.22881869	Up	1.69E-05	1.98E-05
novel_pir851	0	12	0.001	0.6	9.22881869	Up	1.69E-05	1.98E-05
novel_pir184	0	12	0.001	0.6	9.22881869	Up	1.69E-05	1.98E-05
novel_pir1183	0	12	0.001	0.6	9.22881869	Up	1.69E-05	1.98E-05
novel_pir1476	0	12	0.001	0.6	9.22881869	Up	1.69E-05	1.98E-05
novel_pir2268	0	12	0.001	0.6	9.22881869	Up	1.69E-05	1.98E-05
novel_pir2244	0	12	0.001	0.6	9.22881869	Up	1.69E-05	1.98E-05
novel_pir1620	0	12	0.001	0.6	9.22881869	Up	1.69E-05	1.98E-05
novel_pir1735	0	12	0.001	0.6	9.22881869	Up	1.69E-05	1.97E-05
novel_pir178	0	12	0.001	0.6	9.22881869	Up	1.69E-05	1.97E-05
novel_pir1838	0	12	0.001	0.6	9.22881869	Up	1.69E-05	1.97E-05
mmu_piR_000362	0	12	0.001	0.6	9.22881869	Up	1.69E-05	1.97E-05
novel_pir1421	0	12	0.001	0.6	9.22881869	Up	1.69E-05	1.97E-05
novel_pir788	0	12	0.001	0.6	9.22881869	Up	1.69E-05	1.97E-05
novel_pir254	0	12	0.001	0.6	9.22881869	Up	1.69E-05	1.97E-05
novel_pir362	0	12	0.001	0.6	9.22881869	Up	1.69E-05	1.97E-05
novel_pir1590	0	12	0.001	0.6	9.22881869	Up	1.69E-05	1.97E-05
novel_pir418	0	12	0.001	0.6	9.22881869	Up	1.69E-05	1.96E-05
novel_pir581	0	12	0.001	0.6	9.22881869	Up	1.69E-05	1.96E-05
novel_pir1480	0	12	0.001	0.6	9.22881869	Up	1.69E-05	1.96E-05
novel_pir2419	0	12	0.001	0.6	9.22881869	Up	1.69E-05	1.96E-05
novel_pir645	0	12	0.001	0.6	9.22881869	Up	1.69E-05	1.96E-05

novel_pir871	0	12	0.001	0.6	9.22881869	Up	1.69E-05	1.96E-05
novel_pir1578	0	12	0.001	0.6	9.22881869	Up	1.69E-05	1.96E-05
novel_pir2286	0	12	0.001	0.6	9.22881869	Up	1.69E-05	1.96E-05
novel_pir1830	0	12	0.001	0.6	9.22881869	Up	1.69E-05	1.95E-05
novel_pir621	0	12	0.001	0.6	9.22881869	Up	1.69E-05	1.95E-05
novel_pir2256	0	12	0.001	0.6	9.22881869	Up	1.69E-05	1.95E-05
novel_pir2087	0	12	0.001	0.6	9.22881869	Up	1.69E-05	1.95E-05
novel_pir1427	0	12	0.001	0.6	9.22881869	Up	1.69E-05	1.95E-05
novel_pir969	0	11	0.001	0.55	9.103287808	Up	4.15E-05	4.76E-05
novel_pir2142	0	11	0.001	0.55	9.103287808	Up	4.15E-05	4.76E-05
novel_pir1912	0	11	0.001	0.55	9.103287808	Up	4.15E-05	4.75E-05
novel_pir1160	0	11	0.001	0.55	9.103287808	Up	4.15E-05	4.75E-05
novel_pir2054	0	11	0.001	0.55	9.103287808	Up	4.15E-05	4.75E-05
novel_pir285	0	11	0.001	0.55	9.103287808	Up	4.15E-05	4.75E-05
novel_pir700	0	11	0.001	0.55	9.103287808	Up	4.15E-05	4.74E-05
novel_pir1621	0	11	0.001	0.55	9.103287808	Up	4.15E-05	4.74E-05
novel_pir944	0	11	0.001	0.55	9.103287808	Up	4.15E-05	4.74E-05
novel_pir1044	0	11	0.001	0.55	9.103287808	Up	4.15E-05	4.74E-05
novel_pir2024	0	11	0.001	0.55	9.103287808	Up	4.15E-05	4.73E-05
novel_pir2171	0	11	0.001	0.55	9.103287808	Up	4.15E-05	4.73E-05
novel_pir1769	0	11	0.001	0.55	9.103287808	Up	4.15E-05	4.73E-05
novel_pir257	0	11	0.001	0.55	9.103287808	Up	4.15E-05	4.72E-05
novel_pir1816	0	11	0.001	0.55	9.103287808	Up	4.15E-05	4.72E-05
novel_pir764	0	11	0.001	0.55	9.103287808	Up	4.15E-05	4.72E-05
novel_pir1582	0	11	0.001	0.55	9.103287808	Up	4.15E-05	4.72E-05
novel_pir521	0	11	0.001	0.55	9.103287808	Up	4.15E-05	4.71E-05
novel_pir1943	0	11	0.001	0.55	9.103287808	Up	4.15E-05	4.71E-05
novel_pir542	0	11	0.001	0.55	9.103287808	Up	4.15E-05	4.71E-05
novel_pir1185	0	11	0.001	0.55	9.103287808	Up	4.15E-05	4.70E-05
novel_pir904	0	11	0.001	0.55	9.103287808	Up	4.15E-05	4.70E-05
novel_pir423	0	11	0.001	0.55	9.103287808	Up	4.15E-05	4.70E-05
novel_pir304	0	11	0.001	0.55	9.103287808	Up	4.15E-05	4.70E-05
novel_pir1815	0	11	0.001	0.55	9.103287808	Up	4.15E-05	4.69E-05
novel_pir799	0	11	0.001	0.55	9.103287808	Up	4.15E-05	4.69E-05
novel_pir406	0	11	0.001	0.55	9.103287808	Up	4.15E-05	4.69E-05
novel_pir2072	0	11	0.001	0.55	9.103287808	Up	4.15E-05	4.69E-05

novel_pir808	0	11	0.001	0.55	9.103287808	Up	4.15E-05	4.68E-05
novel_pir2206	0	11	0.001	0.55	9.103287808	Up	4.15E-05	4.68E-05
novel_pir746	0	11	0.001	0.55	9.103287808	Up	4.15E-05	4.68E-05
novel_pir1908	0	11	0.001	0.55	9.103287808	Up	4.15E-05	4.67E-05
novel_pir1690	0	11	0.001	0.55	9.103287808	Up	4.15E-05	4.67E-05
novel_pir1245	0	11	0.001	0.55	9.103287808	Up	4.15E-05	4.67E-05
novel_pir927	0	11	0.001	0.55	9.103287808	Up	4.15E-05	4.67E-05
novel_pir1615	0	11	0.001	0.55	9.103287808	Up	4.15E-05	4.66E-05
novel_pir2374	0	11	0.001	0.55	9.103287808	Up	4.15E-05	4.66E-05
novel_pir849	0	11	0.001	0.55	9.103287808	Up	4.15E-05	4.66E-05
novel_pir2185	0	11	0.001	0.55	9.103287808	Up	4.15E-05	4.66E-05
novel_pir796	0	11	0.001	0.55	9.103287808	Up	4.15E-05	4.65E-05
novel_pir2348	0	11	0.001	0.55	9.103287808	Up	4.15E-05	4.65E-05
novel_pir913	0	11	0.001	0.55	9.103287808	Up	4.15E-05	4.65E-05
novel_pir1813	0	11	0.001	0.55	9.103287808	Up	4.15E-05	4.64E-05
novel_pir2110	0	11	0.001	0.55	9.103287808	Up	4.15E-05	4.64E-05
novel_pir804	0	11	0.001	0.55	9.103287808	Up	4.15E-05	4.64E-05
novel_pir895	0	11	0.001	0.55	9.103287808	Up	4.15E-05	4.64E-05
novel_pir1919	0	11	0.001	0.55	9.103287808	Up	4.15E-05	4.63E-05
novel_pir2470	0	11	0.001	0.55	9.103287808	Up	4.15E-05	4.63E-05
novel_pir25	0	11	0.001	0.55	9.103287808	Up	4.15E-05	4.63E-05
novel_pir1628	0	10	0.001	0.5	8.965784285	Up	0.000101972	0.000112835
novel_pir1112	0	10	0.001	0.5	8.965784285	Up	0.000101972	0.000112771
novel_pir769	0	10	0.001	0.5	8.965784285	Up	0.000101972	0.000112706
novel_pir1338	0	10	0.001	0.5	8.965784285	Up	0.000101972	0.000112642
novel_pir1042	0	10	0.001	0.5	8.965784285	Up	0.000101972	0.000112578
novel_pir317	0	10	0.001	0.5	8.965784285	Up	0.000101972	0.000112513
novel_pir2060	0	10	0.001	0.5	8.965784285	Up	0.000101972	0.000112449
novel_pir407	0	10	0.001	0.5	8.965784285	Up	0.000101972	0.000112385
novel_pir1196	0	10	0.001	0.5	8.965784285	Up	0.000101972	0.000112321
novel_pir2156	0	10	0.001	0.5	8.965784285	Up	0.000101972	0.000112257
novel_pir1488	0	10	0.001	0.5	8.965784285	Up	0.000101972	0.000112193
novel_pir2160	0	10	0.001	0.5	8.965784285	Up	0.000101972	0.000112129
novel_pir1821	0	10	0.001	0.5	8.965784285	Up	0.000101972	0.000112065
novel_pir357	0	10	0.001	0.5	8.965784285	Up	0.000101972	0.000112002
novel_pir1059	0	10	0.001	0.5	8.965784285	Up	0.000101972	0.000111938

novel_pir2432	0	10	0.001	0.5	8.965784285	Up	0.000101972	0.000111874
novel_pir1657	0	10	0.001	0.5	8.965784285	Up	0.000101972	0.000111811
novel_pir506	0	10	0.001	0.5	8.965784285	Up	0.000101972	0.000111747
novel_pir1347	0	10	0.001	0.5	8.965784285	Up	0.000101972	0.000111684
novel_pir638	0	10	0.001	0.5	8.965784285	Up	0.000101972	0.000111621
mmu_piR_002945	0	10	0.001	0.5	8.965784285	Up	0.000101972	0.000111558
novel_pir1868	0	10	0.001	0.5	8.965784285	Up	0.000101972	0.000111494
novel_pir1585	0	10	0.001	0.5	8.965784285	Up	0.000101972	0.000111431
novel_pir641	0	10	0.001	0.5	8.965784285	Up	0.000101972	0.000111368
mmu_piR_011141	0	10	0.001	0.5	8.965784285	Up	0.000101972	0.000111305
novel_pir736	0	10	0.001	0.5	8.965784285	Up	0.000101972	0.000111243
novel_pir225	0	10	0.001	0.5	8.965784285	Up	0.000101972	0.00011118
novel_pir450	0	10	0.001	0.5	8.965784285	Up	0.000101972	0.000111117
novel_pir462	0	10	0.001	0.5	8.965784285	Up	0.000101972	0.000111054
novel_pir1033	0	10	0.001	0.5	8.965784285	Up	0.000101972	0.000110992
novel_pir36	0	10	0.001	0.5	8.965784285	Up	0.000101972	0.000110929
novel_pir162	0	10	0.001	0.5	8.965784285	Up	0.000101972	0.000110867
novel_pir1865	0	10	0.001	0.5	8.965784285	Up	0.000101972	0.000110805
novel_pir1990	0	10	0.001	0.5	8.965784285	Up	0.000101972	0.000110742
novel_pir169	0	10	0.001	0.5	8.965784285	Up	0.000101972	0.00011068
novel_pir937	0	10	0.001	0.5	8.965784285	Up	0.000101972	0.000110618
novel_pir704	0	10	0.001	0.5	8.965784285	Up	0.000101972	0.000110556
novel_pir564	0	10	0.001	0.5	8.965784285	Up	0.000101972	0.000110494
novel_pir1032	0	10	0.001	0.5	8.965784285	Up	0.000101972	0.000110432
novel_pir985	0	10	0.001	0.5	8.965784285	Up	0.000101972	0.00011037
novel_pir1056	0	10	0.001	0.5	8.965784285	Up	0.000101972	0.000110308
novel_pir811	0	10	0.001	0.5	8.965784285	Up	0.000101972	0.000110247
novel_pir1404	0	10	0.001	0.5	8.965784285	Up	0.000101972	0.000110185
novel_pir141	0	10	0.001	0.5	8.965784285	Up	0.000101972	0.000110123
novel_pir479	0	10	0.001	0.5	8.965784285	Up	0.000101972	0.000110062
novel_pir1206	0	10	0.001	0.5	8.965784285	Up	0.000101972	0.00011
novel_pir2373	0	10	0.001	0.5	8.965784285	Up	0.000101972	0.000109939
novel_pir2276	0	10	0.001	0.5	8.965784285	Up	0.000101972	0.000109878
novel_pir1412	0	10	0.001	0.5	8.965784285	Up	0.000101972	0.000109816
novel_pir1229	0	10	0.001	0.5	8.965784285	Up	0.000101972	0.000109755
novel_pir1833	0	10	0.001	0.5	8.965784285	Up	0.000101972	0.000109694

novel_pir1808	0	10	0.001	0.5	8.965784285	Up	0.000101972	0.000109633
novel_pir344	0	10	0.001	0.5	8.965784285	Up	0.000101972	0.000109572
novel_pir120	0	10	0.001	0.5	8.965784285	Up	0.000101972	0.000109511
novel_pir1103	0	10	0.001	0.5	8.965784285	Up	0.000101972	0.00010945
novel_pir73	0	10	0.001	0.5	8.965784285	Up	0.000101972	0.00010939
novel_pir1780	0	10	0.001	0.5	8.965784285	Up	0.000101972	0.000109329
novel_pir1013	0	10	0.001	0.5	8.965784285	Up	0.000101972	0.000109268
novel_pir311	0	10	0.001	0.5	8.965784285	Up	0.000101972	0.000109208
novel_pir1001	0	10	0.001	0.5	8.965784285	Up	0.000101972	0.000109147
novel_pir2439	0	10	0.001	0.5	8.965784285	Up	0.000101972	0.000109087
novel_pir861	0	10	0.001	0.5	8.965784285	Up	0.000101972	0.000109026
novel_pir501	0	10	0.001	0.5	8.965784285	Up	0.000101972	0.000108966
novel_pir189	0	10	0.001	0.5	8.965784285	Up	0.000101972	0.000108906
novel_pir288	0	10	0.001	0.5	8.965784285	Up	0.000101972	0.000108846
novel_pir758	0	10	0.001	0.5	8.965784285	Up	0.000101972	0.000108786
novel_pir749	0	10	0.001	0.5	8.965784285	Up	0.000101972	0.000108726
novel_pir2241	0	10	0.001	0.5	8.965784285	Up	0.000101972	0.000108666
novel_pir2098	0	10	0.001	0.5	8.965784285	Up	0.000101972	0.000108606
novel_pir2365	0	10	0.001	0.5	8.965784285	Up	0.000101972	0.000108546
novel_pir426	0	10	0.001	0.5	8.965784285	Up	0.000101972	0.000108486
mmu_piR_000442	0	8	0.001	0.4	8.64385619	Up	0.0006151	0.000644454
mmu_piR_012500	0	8	0.001	0.4	8.64385619	Up	0.0006151	0.000644105
mmu_piR_028051	1	81	0.03	4.02	7.06608919	Up	9.95E-31	2.24E-30
mmu_piR_022097	32	1478	1.09	73.29	6.071216324	Up	0	0
mmu_piR_000870	1	39	0.03	1.93	6.007494537	Up	1.22E-14	2.07E-14
mmu_piR_000159	3	93	0.1	4.61	5.526694846	Up	1.31E-32	3.04E-32
mmu_piR_000958	1	24	0.03	1.19	5.309855263	Up	5.55E-09	7.76E-09
mmu_piR_000935	2838	52145	96.65	2585.62	4.74159672	Up	0	0
mmu_piR_038322	1	16	0.03	0.79	4.718818247	Up	5.15E-06	6.27E-06
mmu_piR_038312	1	15	0.03	0.74	4.624490865	Up	1.20E-05	1.42E-05
mmu_piR_033077	2	23	0.07	1.14	4.025535092	Up	1.04E-07	1.38E-07
novel_pir2034	1123	7145	38.24	354.29	3.211776317	Up	0	0
novel_pir2037	247	464	8.41	23.01	1.452083278	Up	1.13E-39	2.94E-39
novel_pir150	21	38	0.72	1.88	1.38466385	Up	0.00025209	0.000266432
novel_pir125	4195	0	142.86	0.001	-17.1242425	Down	0	0
novel_pir13	1438	0	48.97	0.001	-15.57961058	Down	0	0

novel_pir230	1224	0	41.68	0.001	-15.34706766	Down	1.40E-278	1.18E-276
novel_pir14	1223	0	41.65	0.001	-15.34602888	Down	2.36E-278	1.90E-276
novel_pir615	991	0	33.75	0.001	-15.04259988	Down	1.13E-225	8.08E-224
novel_pir229	857	0	29.19	0.001	-14.83318659	Down	3.02E-195	1.94E-193
novel_pir340	856	0	29.15	0.001	-14.83120826	Down	5.09E-195	3.07E-193
novel_pir123	853	0	29.05	0.001	-14.82625054	Down	2.44E-194	1.43E-192
novel_pir130	837	0	28.5	0.001	-14.7986743	Down	1.05E-190	5.96E-189
novel_pir131	786	0	26.77	0.001	-14.70832952	Down	3.99E-179	2.20E-177
novel_pir863	778	0	26.5	0.001	-14.69370474	Down	2.62E-177	1.40E-175
novel_pir728	764	0	26.02	0.001	-14.66733334	Down	3.95E-174	2.06E-172
novel_pir273	745	0	25.37	0.001	-14.6308359	Down	8.14E-170	4.03E-168
novel_pir961	743	0	25.3	0.001	-14.62684976	Down	2.32E-169	1.12E-167
novel_pir136	720	0	24.52	0.001	-14.58167136	Down	3.87E-164	1.82E-162
novel_pir1524	686	0	23.36	0.001	-14.51175265	Down	2.03E-156	9.12E-155
novel_pir611	675	0	22.99	0.001	-14.48871885	Down	6.39E-154	2.80E-152
novel_pir2175	670	0	22.82	0.001	-14.47801117	Down	8.72E-153	3.74E-151
novel_pir1517	663	0	22.58	0.001	-14.46275787	Down	3.39E-151	1.42E-149
novel_pir778	661	0	22.51	0.001	-14.45827844	Down	9.64E-151	3.96E-149
novel_pir1581	637	0	21.69	0.001	-14.40474243	Down	2.71E-145	1.07E-143
novel_pir967	627	0	21.35	0.001	-14.38194845	Down	5.06E-143	1.96E-141
novel_pir2332	625	0	21.28	0.001	-14.37721053	Down	1.44E-142	5.46E-141
novel_pir711	617	0	21.01	0.001	-14.35878854	Down	9.44E-141	3.44E-139
novel_pir1994	616	0	20.98	0.001	-14.35672706	Down	1.59E-140	5.70E-139
novel_pir2237	612	0	20.84	0.001	-14.34706766	Down	1.29E-139	4.53E-138
novel_pir79	599	0	20.4	0.001	-14.31628153	Down	1.15E-136	3.91E-135
novel_pir780	597	0	20.33	0.001	-14.31132259	Down	3.28E-136	1.09E-134
novel_pir10	588	0	20.02	0.001	-14.28915435	Down	3.63E-134	1.19E-132
novel_pir47	580	0	19.75	0.001	-14.26956503	Down	2.38E-132	7.66E-131
novel_pir2321	568	0	19.34	0.001	-14.23930017	Down	1.26E-129	3.93E-128
novel_pir1707	560	0	19.07	0.001	-14.21901722	Down	8.27E-128	2.54E-126
novel_pir1262	556	0	18.93	0.001	-14.20838679	Down	6.70E-127	2.02E-125
novel_pir1368	550	0	18.73	0.001	-14.19306328	Down	1.54E-125	4.59E-124
novel_pir1334	532	0	18.12	0.001	-14.14529533	Down	1.89E-121	5.52E-120
novel_pir1179	519	0	17.67	0.001	-14.10901442	Down	1.69E-118	4.80E-117
novel_pir166	517	0	17.61	0.001	-14.10410729	Down	4.80E-118	1.34E-116
novel_pir766	512	0	17.44	0.001	-14.09011242	Down	6.56E-117	1.81E-115

novel_pir228	510	0	17.37	0.001	-14.08431013	Down	1.87E-116	5.08E-115
novel_pir551	508	0	17.3	0.001	-14.07848442	Down	5.31E-116	1.41E-114
novel_pir2184	507	0	17.27	0.001	-14.07598046	Down	8.96E-116	2.34E-114
novel_pir126	502	0	17.1	0.001	-14.0617087	Down	1.22E-114	3.15E-113
novel_pir1382	500	0	17.03	0.001	-14.05579081	Down	3.48E-114	8.85E-113
novel_pir1207	495	0	16.86	0.001	-14.04131692	Down	4.75E-113	1.19E-111
novel_pir2257	491	0	16.72	0.001	-14.02928723	Down	3.85E-112	9.53E-111
novel_pir2099	484	0	16.48	0.001	-14.00842862	Down	1.49E-110	3.66E-109
novel_pir67	484	0	16.48	0.001	-14.00842862	Down	1.49E-110	3.61E-109
novel_pir1734	484	0	16.48	0.001	-14.00842862	Down	1.49E-110	3.57E-109
novel_pir2083	483	0	16.45	0.001	-14.00579996	Down	2.52E-110	5.94E-109
novel_pir3	480	0	16.35	0.001	-13.99700302	Down	1.21E-109	2.82E-108
novel_pir617	476	0	16.21	0.001	-13.98459647	Down	9.80E-109	2.25E-107
novel_pir2049	476	0	16.21	0.001	-13.98459647	Down	9.80E-109	2.23E-107
novel_pir6	476	0	16.21	0.001	-13.98459647	Down	9.80E-109	2.20E-107
novel_pir628	472	0	16.07	0.001	-13.97208231	Down	7.93E-108	1.74E-106
novel_pir364	471	0	16.04	0.001	-13.96938652	Down	1.34E-107	2.90E-106
novel_pir329	469	0	15.97	0.001	-13.96307669	Down	3.81E-107	8.17E-106
novel_pir573	468	0	15.94	0.001	-13.96036401	Down	6.42E-107	1.36E-105
novel_pir2086	466	0	15.87	0.001	-13.95401451	Down	1.83E-106	3.84E-105
novel_pir226	464	0	15.8	0.001	-13.94763694	Down	5.20E-106	1.08E-104
novel_pir1655	462	0	15.73	0.001	-13.94123105	Down	1.48E-105	3.04E-104
novel_pir872	461	0	15.7	0.001	-13.93847694	Down	2.50E-105	5.07E-104
novel_pir1406	460	0	15.67	0.001	-13.93571756	Down	4.21E-105	8.47E-104
novel_pir111	459	0	15.63	0.001	-13.93203016	Down	7.10E-105	1.41E-103
novel_pir2214	458	0	15.6	0.001	-13.92925841	Down	1.20E-104	2.36E-103
novel_pir195	458	0	15.6	0.001	-13.92925841	Down	1.20E-104	2.34E-103
novel_pir2306	458	0	15.6	0.001	-13.92925841	Down	1.20E-104	2.31E-103
novel_pir68	458	0	15.6	0.001	-13.92925841	Down	1.20E-104	2.29E-103
novel_pir547	456	0	15.53	0.001	-13.92277021	Down	3.41E-104	6.45E-103
novel_pir2093	455	0	15.5	0.001	-13.9199806	Down	5.75E-104	1.08E-102
novel_pir1148	450	0	15.32	0.001	-13.90312868	Down	7.85E-103	1.46E-101
novel_pir1215	449	0	15.29	0.001	-13.90030079	Down	1.32E-102	2.44E-101
novel_pir1161	447	0	15.22	0.001	-13.89368074	Down	3.77E-102	6.80E-101
novel_pir2026	445	0	15.15	0.001	-13.88703017	Down	1.07E-101	1.92E-100
novel_pir2271	443	0	15.09	0.001	-13.88130519	Down	3.05E-101	5.41E-100

novel_pir348	441	0	15.02	0.001	-13.87459719	Down	8.68E-101	1.51E-99
novel_pir469	438	0	14.92	0.001	-13.86495992	Down	4.16E-100	7.18E-99
novel_pir1705	438	0	14.92	0.001	-13.86495992	Down	4.16E-100	7.12E-99
novel_pir380	436	0	14.85	0.001	-13.85817531	Down	1.18E-99	2.01E-98
novel_pir2263	436	0	14.85	0.001	-13.85817531	Down	1.18E-99	1.99E-98
novel_pir575	433	0	14.75	0.001	-13.84842733	Down	5.69E-99	9.47E-98
novel_pir1258	433	0	14.75	0.001	-13.84842733	Down	5.69E-99	9.39E-98
novel_pir347	432	0	14.71	0.001	-13.84450963	Down	9.59E-99	1.57E-97
novel_pir400	432	0	14.71	0.001	-13.84450963	Down	9.59E-99	1.56E-97
novel_pir1937	432	0	14.71	0.001	-13.84450963	Down	9.59E-99	1.54E-97
novel_pir1689	430	0	14.64	0.001	-13.83762793	Down	2.73E-98	4.36E-97
novel_pir1716	430	0	14.64	0.001	-13.83762793	Down	2.73E-98	4.32E-97
novel_pir809	429	0	14.61	0.001	-13.83466856	Down	4.60E-98	7.23E-97
novel_pir1073	429	0	14.61	0.001	-13.83466856	Down	4.60E-98	7.17E-97
novel_pir2139	429	0	14.61	0.001	-13.83466856	Down	4.60E-98	7.12E-97
novel_pir1218	429	0	14.61	0.001	-13.83466856	Down	4.60E-98	7.06E-97
novel_pir2095	427	0	14.54	0.001	-13.82773965	Down	1.31E-97	1.99E-96
novel_pir1881	427	0	14.54	0.001	-13.82773965	Down	1.31E-97	1.98E-96
novel_pir1055	426	0	14.51	0.001	-13.8247599	Down	2.21E-97	3.31E-96
novel_pir2059	422	0	14.37	0.001	-13.81077244	Down	1.79E-96	2.66E-95
novel_pir651	420	0	14.3	0.001	-13.80372753	Down	5.09E-96	7.51E-95
novel_pir820	417	0	14.2	0.001	-13.79360331	Down	2.44E-95	3.52E-94
novel_pir2226	416	0	14.17	0.001	-13.79055214	Down	4.12E-95	5.85E-94
novel_pir204	416	0	14.17	0.001	-13.79055214	Down	4.12E-95	5.81E-94
novel_pir1065	413	0	14.06	0.001	-13.77930897	Down	1.98E-94	2.77E-93
novel_pir119	412	0	14.03	0.001	-13.77622739	Down	3.34E-94	4.64E-93
novel_pir2018	412	0	14.03	0.001	-13.77622739	Down	3.34E-94	4.60E-93
novel_pir1395	412	0	14.03	0.001	-13.77622739	Down	3.34E-94	4.57E-93
novel_pir1684	412	0	14.03	0.001	-13.77622739	Down	3.34E-94	4.54E-93
novel_pir1441	411	0	14	0.001	-13.77313921	Down	5.63E-94	7.60E-93
novel_pir1159	408	0	13.89	0.001	-13.76175898	Down	2.70E-93	3.62E-92
novel_pir495	407	0	13.86	0.001	-13.75863964	Down	4.56E-93	6.07E-92
novel_pir1100	405	0	13.79	0.001	-13.75133484	Down	1.30E-92	1.72E-91
novel_pir22	405	0	13.79	0.001	-13.75133484	Down	1.30E-92	1.70E-91
novel_pir1775	404	0	13.76	0.001	-13.74819285	Down	2.19E-92	2.85E-91
novel_pir2105	403	0	13.72	0.001	-13.74399286	Down	3.69E-92	4.78E-91

novel_pir2395	403	0	13.72	0.001	-13.74399286	Down	3.69E-92	4.75E-91
novel_pir1900	403	0	13.72	0.001	-13.74399286	Down	3.69E-92	4.72E-91
novel_pir783	401	0	13.66	0.001	-13.73766986	Down	1.05E-91	1.33E-90
novel_pir115	401	0	13.66	0.001	-13.73766986	Down	1.05E-91	1.32E-90
novel_pir1465	400	0	13.62	0.001	-13.73343908	Down	1.77E-91	2.21E-90
novel_pir396	399	0	13.59	0.001	-13.73025784	Down	2.99E-91	3.70E-90
novel_pir2193	398	0	13.55	0.001	-13.72600523	Down	5.04E-91	6.20E-90
novel_pir917	397	0	13.52	0.001	-13.72280753	Down	8.50E-91	1.03E-89
novel_pir1643	397	0	13.52	0.001	-13.72280753	Down	8.50E-91	1.03E-89
novel_pir2423	397	0	13.52	0.001	-13.72280753	Down	8.50E-91	1.02E-89
novel_pir315	397	0	13.52	0.001	-13.72280753	Down	8.50E-91	1.01E-89
novel_pir612	397	0	13.52	0.001	-13.72280753	Down	8.50E-91	1.01E-89
novel_pir1114	395	0	13.45	0.001	-13.71531855	Down	2.42E-90	2.85E-89
novel_pir1028	394	0	13.42	0.001	-13.71209705	Down	4.08E-90	4.77E-89
novel_pir2354	394	0	13.42	0.001	-13.71209705	Down	4.08E-90	4.75E-89
novel_pir1659	393	0	13.38	0.001	-13.7077905	Down	6.88E-90	7.91E-89
novel_pir516	393	0	13.38	0.001	-13.7077905	Down	6.88E-90	7.86E-89
novel_pir1359	391	0	13.32	0.001	-13.70130646	Down	1.96E-89	2.22E-88
novel_pir876	388	0	13.21	0.001	-13.68934285	Down	9.39E-89	1.05E-87
novel_pir751	388	0	13.21	0.001	-13.68934285	Down	9.39E-89	1.04E-87
novel_pir1088	388	0	13.21	0.001	-13.68934285	Down	9.39E-89	1.04E-87
novel_pir1862	388	0	13.21	0.001	-13.68934285	Down	9.39E-89	1.03E-87
novel_pir2411	387	0	13.18	0.001	-13.68606275	Down	1.58E-88	1.73E-87
novel_pir1340	387	0	13.18	0.001	-13.68606275	Down	1.58E-88	1.72E-87
novel_pir23	387	0	13.18	0.001	-13.68606275	Down	1.58E-88	1.71E-87
novel_pir571	386	0	13.15	0.001	-13.68277518	Down	2.67E-88	2.87E-87
novel_pir1241	386	0	13.15	0.001	-13.68277518	Down	2.67E-88	2.85E-87
novel_pir314	386	0	13.15	0.001	-13.68277518	Down	2.67E-88	2.84E-87
novel_pir346	386	0	13.15	0.001	-13.68277518	Down	2.67E-88	2.82E-87
novel_pir2192	385	0	13.11	0.001	-13.67838007	Down	4.51E-88	4.73E-87
novel_pir1481	384	0	13.08	0.001	-13.67507492	Down	7.60E-88	7.94E-87
novel_pir1670	382	0	13.01	0.001	-13.66733334	Down	2.16E-87	2.25E-86
novel_pir713	381	0	12.98	0.001	-13.66400276	Down	3.65E-87	3.77E-86
novel_pir540	381	0	12.98	0.001	-13.66400276	Down	3.65E-87	3.75E-86
novel_pir481	381	0	12.98	0.001	-13.66400276	Down	3.65E-87	3.73E-86
novel_pir282	381	0	12.98	0.001	-13.66400276	Down	3.65E-87	3.71E-86

novel_pir974	381	0	12.98	0.001	-13.66400276	Down	3.65E-87	3.69E-86
novel_pir959	381	0	12.98	0.001	-13.66400276	Down	3.65E-87	3.67E-86
novel_pir548	380	0	12.94	0.001	-13.65955	Down	6.16E-87	6.13E-86
novel_pir669	380	0	12.94	0.001	-13.65955	Down	6.16E-87	6.10E-86
novel_pir1740	379	0	12.91	0.001	-13.65620138	Down	1.04E-86	1.02E-85
novel_pir1727	379	0	12.91	0.001	-13.65620138	Down	1.04E-86	1.02E-85
novel_pir2254	378	0	12.87	0.001	-13.65172443	Down	1.75E-86	1.71E-85
novel_pir545	378	0	12.87	0.001	-13.65172443	Down	1.75E-86	1.70E-85
novel_pir697	377	0	12.84	0.001	-13.64835758	Down	2.95E-86	2.85E-85
novel_pir2289	376	0	12.8	0.001	-13.64385619	Down	4.98E-86	4.79E-85
novel_pir2101	376	0	12.8	0.001	-13.64385619	Down	4.98E-86	4.77E-85
novel_pir2375	375	0	12.77	0.001	-13.6404709	Down	8.41E-86	8.00E-85
novel_pir1985	374	0	12.74	0.001	-13.63707766	Down	1.42E-85	1.34E-84
novel_pir1721	373	0	12.7	0.001	-13.63254088	Down	2.39E-85	2.25E-84
novel_pir1034	373	0	12.7	0.001	-13.63254088	Down	2.39E-85	2.24E-84
novel_pir1294	372	0	12.67	0.001	-13.6291289	Down	4.04E-85	3.77E-84
novel_pir1750	371	0	12.63	0.001	-13.62456702	Down	6.81E-85	6.32E-84
novel_pir2176	371	0	12.63	0.001	-13.62456702	Down	6.81E-85	6.29E-84
novel_pir688	370	0	12.6	0.001	-13.62113611	Down	1.15E-84	1.05E-83
novel_pir278	370	0	12.6	0.001	-13.62113611	Down	1.15E-84	1.05E-83
novel_pir1388	370	0	12.6	0.001	-13.62113611	Down	1.15E-84	1.04E-83
novel_pir1984	369	0	12.57	0.001	-13.61769703	Down	1.94E-84	1.75E-83
novel_pir1942	368	0	12.53	0.001	-13.61309879	Down	3.27E-84	2.94E-83
novel_pir1269	367	0	12.5	0.001	-13.60964047	Down	5.51E-84	4.93E-83
novel_pir634	367	0	12.5	0.001	-13.60964047	Down	5.51E-84	4.91E-83
novel_pir205	366	0	12.46	0.001	-13.60501645	Down	9.30E-84	8.24E-83
novel_pir124	366	0	12.46	0.001	-13.60501645	Down	9.30E-84	8.20E-83
novel_pir2265	364	0	12.4	0.001	-13.5980525	Down	2.64E-83	2.32E-82
novel_pir774	364	0	12.4	0.001	-13.5980525	Down	2.64E-83	2.31E-82
novel_pir2471	364	0	12.4	0.001	-13.5980525	Down	2.64E-83	2.30E-82
novel_pir1379	364	0	12.4	0.001	-13.5980525	Down	2.64E-83	2.29E-82
novel_pir954	364	0	12.4	0.001	-13.5980525	Down	2.64E-83	2.28E-82
novel_pir448	363	0	12.36	0.001	-13.59339112	Down	4.46E-83	3.83E-82
novel_pir374	362	0	12.33	0.001	-13.58988518	Down	7.53E-83	6.43E-82
novel_pir1676	362	0	12.33	0.001	-13.58988518	Down	7.53E-83	6.40E-82
novel_pir2378	361	0	12.29	0.001	-13.5851973	Down	1.27E-82	1.07E-81

novel_pir1804	361	0	12.29	0.001	-13.5851973	Down	1.27E-82	1.07E-81
novel_pir1232	361	0	12.29	0.001	-13.5851973	Down	1.27E-82	1.06E-81
novel_pir775	360	0	12.26	0.001	-13.58167136	Down	2.14E-82	1.78E-81
novel_pir556	360	0	12.26	0.001	-13.58167136	Down	2.14E-82	1.78E-81
novel_pir682	360	0	12.26	0.001	-13.58167136	Down	2.14E-82	1.77E-81
novel_pir416	359	0	12.23	0.001	-13.57813678	Down	3.61E-82	2.97E-81
novel_pir377	359	0	12.23	0.001	-13.57813678	Down	3.61E-82	2.96E-81
novel_pir2314	358	0	12.19	0.001	-13.57341051	Down	6.09E-82	4.95E-81
novel_pir743	358	0	12.19	0.001	-13.57341051	Down	6.09E-82	4.92E-81
novel_pir132	358	0	12.19	0.001	-13.57341051	Down	6.09E-82	4.90E-81
novel_pir2097	356	0	12.12	0.001	-13.56510208	Down	1.73E-81	1.39E-80
novel_pir667	356	0	12.12	0.001	-13.56510208	Down	1.73E-81	1.38E-80
mmu_piR_022820	355	0	12.09	0.001	-13.56152662	Down	2.92E-81	2.32E-80
novel_pir1260	354	0	12.06	0.001	-13.55794229	Down	4.93E-81	3.89E-80
novel_pir591	354	0	12.06	0.001	-13.55794229	Down	4.93E-81	3.87E-80
novel_pir748	354	0	12.06	0.001	-13.55794229	Down	4.93E-81	3.86E-80
novel_pir1627	354	0	12.06	0.001	-13.55794229	Down	4.93E-81	3.84E-80
novel_pir1647	353	0	12.02	0.001	-13.55314928	Down	8.32E-81	6.46E-80
novel_pir1814	352	0	11.99	0.001	-13.54954404	Down	1.40E-80	1.08E-79
novel_pir1791	352	0	11.99	0.001	-13.54954404	Down	1.40E-80	1.08E-79
novel_pir1523	352	0	11.99	0.001	-13.54954404	Down	1.40E-80	1.08E-79
novel_pir710	352	0	11.99	0.001	-13.54954404	Down	1.40E-80	1.07E-79
novel_pir313	351	0	11.95	0.001	-13.544723	Down	2.37E-80	1.80E-79
novel_pir1493	350	0	11.92	0.001	-13.54109662	Down	3.99E-80	3.03E-79
novel_pir369	349	0	11.89	0.001	-13.53746109	Down	6.74E-80	5.06E-79
novel_pir7	349	0	11.89	0.001	-13.53746109	Down	6.74E-80	5.04E-79
novel_pir1319	349	0	11.89	0.001	-13.53746109	Down	6.74E-80	5.02E-79
novel_pir664	349	0	11.89	0.001	-13.53746109	Down	6.74E-80	5.00E-79
novel_pir862	349	0	11.89	0.001	-13.53746109	Down	6.74E-80	4.99E-79
novel_pir1415	348	0	11.85	0.001	-13.53259944	Down	1.14E-79	8.38E-79
novel_pir1012	348	0	11.85	0.001	-13.53259944	Down	1.14E-79	8.35E-79
novel_pir186	348	0	11.85	0.001	-13.53259944	Down	1.14E-79	8.31E-79
novel_pir206	347	0	11.82	0.001	-13.52894242	Down	1.92E-79	1.40E-78
novel_pir767	347	0	11.82	0.001	-13.52894242	Down	1.92E-79	1.39E-78
novel_pir242	347	0	11.82	0.001	-13.52894242	Down	1.92E-79	1.39E-78
novel_pir724	346	0	11.78	0.001	-13.52405192	Down	3.23E-79	2.33E-78

novel_pir1203	346	0	11.78	0.001	-13.52405192	Down	3.23E-79	2.32E-78
novel_pir243	346	0	11.78	0.001	-13.52405192	Down	3.23E-79	2.31E-78
novel_pir2107	345	0	11.75	0.001	-13.52037314	Down	5.45E-79	3.89E-78
novel_pir2168	344	0	11.72	0.001	-13.51668495	Down	9.20E-79	6.53E-78
novel_pir656	344	0	11.72	0.001	-13.51668495	Down	9.20E-79	6.51E-78
novel_pir1135	343	0	11.68	0.001	-13.51175265	Down	1.55E-78	1.09E-77
novel_pir283	343	0	11.68	0.001	-13.51175265	Down	1.55E-78	1.09E-77
novel_pir102	342	0	11.65	0.001	-13.50804233	Down	2.62E-78	1.83E-77
novel_pir910	342	0	11.65	0.001	-13.50804233	Down	2.62E-78	1.82E-77
novel_pir1190	341	0	11.61	0.001	-13.50308035	Down	4.41E-78	3.06E-77
novel_pir655	341	0	11.61	0.001	-13.50308035	Down	4.41E-78	3.05E-77
novel_pir1644	341	0	11.61	0.001	-13.50308035	Down	4.41E-78	3.04E-77
novel_pir203	340	0	11.58	0.001	-13.49934763	Down	7.45E-78	5.10E-77
novel_pir493	340	0	11.58	0.001	-13.49934763	Down	7.45E-78	5.08E-77
novel_pir2476	340	0	11.58	0.001	-13.49934763	Down	7.45E-78	5.07E-77
novel_pir2	340	0	11.58	0.001	-13.49934763	Down	7.45E-78	5.05E-77
novel_pir489	340	0	11.58	0.001	-13.49934763	Down	7.45E-78	5.03E-77
novel_pir2080	339	0	11.54	0.001	-13.4943556	Down	1.26E-77	8.46E-77
novel_pir66	339	0	11.54	0.001	-13.4943556	Down	1.26E-77	8.43E-77
novel_pir1296	339	0	11.54	0.001	-13.4943556	Down	1.26E-77	8.40E-77
novel_pir567	339	0	11.54	0.001	-13.4943556	Down	1.26E-77	8.37E-77
novel_pir2219	339	0	11.54	0.001	-13.4943556	Down	1.26E-77	8.34E-77
novel_pir1066	338	0	11.51	0.001	-13.49060021	Down	2.12E-77	1.40E-76
novel_pir995	338	0	11.51	0.001	-13.49060021	Down	2.12E-77	1.40E-76
novel_pir1080	338	0	11.51	0.001	-13.49060021	Down	2.12E-77	1.39E-76
novel_pir260	337	0	11.48	0.001	-13.48683502	Down	3.57E-77	2.34E-76
novel_pir949	336	0	11.44	0.001	-13.48179943	Down	6.03E-77	3.93E-76
novel_pir596	336	0	11.44	0.001	-13.48179943	Down	6.03E-77	3.92E-76
novel_pir940	336	0	11.44	0.001	-13.48179943	Down	6.03E-77	3.91E-76
novel_pir1140	335	0	11.41	0.001	-13.47801117	Down	1.02E-76	6.57E-76
novel_pir2103	335	0	11.41	0.001	-13.47801117	Down	1.02E-76	6.55E-76
novel_pir1629	335	0	11.41	0.001	-13.47801117	Down	1.02E-76	6.53E-76
novel_pir485	334	0	11.37	0.001	-13.47294463	Down	1.72E-76	1.10E-75
novel_pir1432	334	0	11.37	0.001	-13.47294463	Down	1.72E-76	1.09E-75
novel_pir1611	333	0	11.34	0.001	-13.46913302	Down	2.89E-76	1.84E-75
novel_pir1730	333	0	11.34	0.001	-13.46913302	Down	2.89E-76	1.83E-75

novel_pir2195	331	0	11.27	0.001	-13.4601999	Down	8.23E-76	5.18E-75
novel_pir1231	330	0	11.24	0.001	-13.45635442	Down	1.39E-75	8.68E-75
novel_pir1079	330	0	11.24	0.001	-13.45635442	Down	1.39E-75	8.65E-75
novel_pir1874	330	0	11.24	0.001	-13.45635442	Down	1.39E-75	8.63E-75
novel_pir1385	329	0	11.2	0.001	-13.45121111	Down	2.34E-75	1.45E-74
novel_pir74	328	0	11.17	0.001	-13.44734157	Down	3.95E-75	2.44E-74
novel_pir2224	328	0	11.17	0.001	-13.44734157	Down	3.95E-75	2.43E-74
novel_pir1958	328	0	11.17	0.001	-13.44734157	Down	3.95E-75	2.42E-74
novel_pir1459	328	0	11.17	0.001	-13.44734157	Down	3.95E-75	2.42E-74
novel_pir51	327	0	11.14	0.001	-13.44346161	Down	6.66E-75	4.06E-74
novel_pir2227	326	0	11.1	0.001	-13.43827206	Down	1.12E-74	6.83E-74
novel_pir1979	326	0	11.1	0.001	-13.43827206	Down	1.12E-74	6.81E-74
novel_pir2266	325	0	11.07	0.001	-13.4343676	Down	1.90E-74	1.14E-73
novel_pir69	325	0	11.07	0.001	-13.4343676	Down	1.90E-74	1.14E-73
novel_pir211	325	0	11.07	0.001	-13.4343676	Down	1.90E-74	1.13E-73
novel_pir180	325	0	11.07	0.001	-13.4343676	Down	1.90E-74	1.13E-73
novel_pir2220	325	0	11.07	0.001	-13.4343676	Down	1.90E-74	1.13E-73
novel_pir1323	323	0	11	0.001	-13.4252159	Down	5.40E-74	3.20E-73
novel_pir973	323	0	11	0.001	-13.4252159	Down	5.40E-74	3.19E-73
novel_pir2260	323	0	11	0.001	-13.4252159	Down	5.40E-74	3.18E-73
novel_pir1794	322	0	10.97	0.001	-13.42127591	Down	9.10E-74	5.34E-73
novel_pir1993	321	0	10.93	0.001	-13.41600578	Down	1.54E-73	8.99E-73
novel_pir577	319	0	10.86	0.001	-13.40673648	Down	4.37E-73	2.55E-72
novel_pir1389	318	0	10.83	0.001	-13.40274562	Down	7.37E-73	4.29E-72
novel_pir1904	316	0	10.76	0.001	-13.39339046	Down	2.10E-72	1.22E-71
novel_pir1373	316	0	10.76	0.001	-13.39339046	Down	2.10E-72	1.21E-71
novel_pir2292	315	0	10.73	0.001	-13.38936246	Down	3.54E-72	2.04E-71
novel_pir389	315	0	10.73	0.001	-13.38936246	Down	3.54E-72	2.03E-71
novel_pir1443	315	0	10.73	0.001	-13.38936246	Down	3.54E-72	2.03E-71
novel_pir720	315	0	10.73	0.001	-13.38936246	Down	3.54E-72	2.02E-71
novel_pir1277	315	0	10.73	0.001	-13.38936246	Down	3.54E-72	2.02E-71
novel_pir175	315	0	10.73	0.001	-13.38936246	Down	3.54E-72	2.01E-71
novel_pir2273	314	0	10.69	0.001	-13.38397423	Down	5.97E-72	3.38E-71
novel_pir1490	313	0	10.66	0.001	-13.37991982	Down	1.01E-71	5.68E-71
novel_pir2357	313	0	10.66	0.001	-13.37991982	Down	1.01E-71	5.67E-71
novel_pir1918	312	0	10.63	0.001	-13.37585398	Down	1.70E-71	9.53E-71

novel_pir721	311	0	10.59	0.001	-13.37041497	Down	2.86E-71	1.60E-70
novel_pir1151	310	0	10.56	0.001	-13.36632221	Down	4.83E-71	2.70E-70
novel_pir535	309	0	10.52	0.001	-13.36084708	Down	8.15E-71	4.54E-70
novel_pir579	309	0	10.52	0.001	-13.36084708	Down	8.15E-71	4.52E-70
novel_pir627	309	0	10.52	0.001	-13.36084708	Down	8.15E-71	4.51E-70
novel_pir1440	309	0	10.52	0.001	-13.36084708	Down	8.15E-71	4.50E-70
novel_pir1392	309	0	10.52	0.001	-13.36084708	Down	8.15E-71	4.48E-70
novel_pir525	309	0	10.52	0.001	-13.36084708	Down	8.15E-71	4.47E-70
novel_pir1526	307	0	10.45	0.001	-13.35121532	Down	2.32E-70	1.26E-69
novel_pir1974	307	0	10.45	0.001	-13.35121532	Down	2.32E-70	1.26E-69
novel_pir1366	306	0	10.42	0.001	-13.34706766	Down	3.91E-70	2.12E-69
novel_pir635	306	0	10.42	0.001	-13.34706766	Down	3.91E-70	2.12E-69
novel_pir1718	306	0	10.42	0.001	-13.34706766	Down	3.91E-70	2.11E-69
novel_pir1564	305	0	10.39	0.001	-13.34290803	Down	6.59E-70	3.55E-69
novel_pir950	305	0	10.39	0.001	-13.34290803	Down	6.59E-70	3.54E-69
novel_pir1739	305	0	10.39	0.001	-13.34290803	Down	6.59E-70	3.53E-69
novel_pir1029	304	0	10.35	0.001	-13.33734315	Down	1.11E-69	5.94E-69
novel_pir2233	304	0	10.35	0.001	-13.33734315	Down	1.11E-69	5.92E-69
novel_pir1335	304	0	10.35	0.001	-13.33734315	Down	1.11E-69	5.90E-69
novel_pir1445	303	0	10.32	0.001	-13.33315535	Down	1.88E-69	9.93E-69
novel_pir140	303	0	10.32	0.001	-13.33315535	Down	1.88E-69	9.91E-69
novel_pir1950	303	0	10.32	0.001	-13.33315535	Down	1.88E-69	9.88E-69
novel_pir2043	302	0	10.28	0.001	-13.32755264	Down	3.17E-69	1.66E-68
novel_pir38	302	0	10.28	0.001	-13.32755264	Down	3.17E-69	1.66E-68
novel_pir563	301	0	10.25	0.001	-13.32333629	Down	5.34E-69	2.78E-68
novel_pir303	301	0	10.25	0.001	-13.32333629	Down	5.34E-69	2.77E-68
novel_pir2181	301	0	10.25	0.001	-13.32333629	Down	5.34E-69	2.77E-68
novel_pir875	301	0	10.25	0.001	-13.32333629	Down	5.34E-69	2.76E-68
novel_pir882	301	0	10.25	0.001	-13.32333629	Down	5.34E-69	2.75E-68
novel_pir784	301	0	10.25	0.001	-13.32333629	Down	5.34E-69	2.74E-68
novel_pir1006	300	0	10.22	0.001	-13.31910758	Down	9.01E-69	4.62E-68
novel_pir1002	300	0	10.22	0.001	-13.31910758	Down	9.01E-69	4.60E-68
novel_pir1152	299	0	10.18	0.001	-13.31344994	Down	1.52E-68	7.74E-68
novel_pir1910	299	0	10.18	0.001	-13.31344994	Down	1.52E-68	7.72E-68
novel_pir546	298	0	10.15	0.001	-13.30919211	Down	2.56E-68	1.30E-67
novel_pir207	298	0	10.15	0.001	-13.30919211	Down	2.56E-68	1.30E-67

novel_pir121	298	0	10.15	0.001	-13.30919211	Down	2.56E-68	1.29E-67
novel_pir1932	296	0	10.08	0.001	-13.29920802	Down	7.29E-68	3.67E-67
novel_pir1234	296	0	10.08	0.001	-13.29920802	Down	7.29E-68	3.66E-67
novel_pir76	295	0	10.05	0.001	-13.29490788	Down	1.23E-67	6.16E-67
novel_pir1605	295	0	10.05	0.001	-13.29490788	Down	1.23E-67	6.14E-67
novel_pir860	294	0	10.01	0.001	-13.28915435	Down	2.07E-67	1.03E-66
novel_pir1826	294	0	10.01	0.001	-13.28915435	Down	2.07E-67	1.03E-66
novel_pir2474	294	0	10.01	0.001	-13.28915435	Down	2.07E-67	1.03E-66
novel_pir387	293	0	9.98	0.001	-13.2848241	Down	3.50E-67	1.72E-66
novel_pir694	293	0	9.98	0.001	-13.2848241	Down	3.50E-67	1.72E-66
novel_pir832	292	0	9.94	0.001	-13.27903014	Down	5.90E-67	2.89E-66
novel_pir1408	292	0	9.94	0.001	-13.27903014	Down	5.90E-67	2.89E-66
novel_pir2210	292	0	9.94	0.001	-13.27903014	Down	5.90E-67	2.88E-66
novel_pir1072	292	0	9.94	0.001	-13.27903014	Down	5.90E-67	2.87E-66
novel_pir1922	291	0	9.91	0.001	-13.27466934	Down	9.96E-67	4.83E-66
novel_pir1603	291	0	9.91	0.001	-13.27466934	Down	9.96E-67	4.82E-66
novel_pir190	291	0	9.91	0.001	-13.27466934	Down	9.96E-67	4.81E-66
novel_pir1894	290	0	9.88	0.001	-13.27029533	Down	1.68E-66	8.09E-66
novel_pir368	289	0	9.84	0.001	-13.2644426	Down	2.83E-66	1.36E-65
novel_pir1037	288	0	9.81	0.001	-13.26003742	Down	4.78E-66	2.29E-65
novel_pir978	288	0	9.81	0.001	-13.26003742	Down	4.78E-66	2.29E-65
novel_pir1502	288	0	9.81	0.001	-13.26003742	Down	4.78E-66	2.28E-65
novel_pir2390	287	0	9.77	0.001	-13.25414285	Down	8.06E-66	3.84E-65
novel_pir483	287	0	9.77	0.001	-13.25414285	Down	8.06E-66	3.83E-65
novel_pir2153	287	0	9.77	0.001	-13.25414285	Down	8.06E-66	3.82E-65
novel_pir779	287	0	9.77	0.001	-13.25414285	Down	8.06E-66	3.81E-65
novel_pir541	286	0	9.74	0.001	-13.24970606	Down	1.36E-65	6.41E-65
novel_pir2197	285	0	9.71	0.001	-13.24525558	Down	2.29E-65	1.08E-64
novel_pir1853	285	0	9.71	0.001	-13.24525558	Down	2.29E-65	1.08E-64
novel_pir2050	285	0	9.71	0.001	-13.24525558	Down	2.29E-65	1.07E-64
novel_pir2441	284	0	9.67	0.001	-13.23930017	Down	3.87E-65	1.81E-64
novel_pir1965	284	0	9.67	0.001	-13.23930017	Down	3.87E-65	1.80E-64
novel_pir1173	284	0	9.67	0.001	-13.23930017	Down	3.87E-65	1.80E-64
novel_pir557	284	0	9.67	0.001	-13.23930017	Down	3.87E-65	1.79E-64
novel_pir630	284	0	9.67	0.001	-13.23930017	Down	3.87E-65	1.79E-64
novel_pir281	284	0	9.67	0.001	-13.23930017	Down	3.87E-65	1.78E-64

novel_pir1745	283	0	9.64	0.001	-13.23481743	Down	6.53E-65	2.99E-64
novel_pir805	283	0	9.64	0.001	-13.23481743	Down	6.53E-65	2.99E-64
novel_pir1855	283	0	9.64	0.001	-13.23481743	Down	6.53E-65	2.98E-64
novel_pir460	282	0	9.6	0.001	-13.22881869	Down	1.10E-64	5.00E-64
novel_pir702	281	0	9.57	0.001	-13.22430321	Down	1.86E-64	8.42E-64
novel_pir1971	281	0	9.57	0.001	-13.22430321	Down	1.86E-64	8.40E-64
novel_pir1225	281	0	9.57	0.001	-13.22430321	Down	1.86E-64	8.38E-64
novel_pir497	281	0	9.57	0.001	-13.22430321	Down	1.86E-64	8.36E-64
novel_pir1508	280	0	9.54	0.001	-13.21977355	Down	3.13E-64	1.40E-63
novel_pir349	280	0	9.54	0.001	-13.21977355	Down	3.13E-64	1.40E-63
novel_pir183	280	0	9.54	0.001	-13.21977355	Down	3.13E-64	1.40E-63
novel_pir722	279	0	9.5	0.001	-13.2137118	Down	5.28E-64	2.35E-63
novel_pir1129	279	0	9.5	0.001	-13.2137118	Down	5.28E-64	2.34E-63
novel_pir1194	279	0	9.5	0.001	-13.2137118	Down	5.28E-64	2.34E-63
novel_pir866	278	0	9.47	0.001	-13.20914871	Down	8.91E-64	3.93E-63
novel_pir580	278	0	9.47	0.001	-13.20914871	Down	8.91E-64	3.92E-63
novel_pir1400	277	0	9.43	0.001	-13.20304206	Down	1.50E-63	6.60E-63
novel_pir1086	277	0	9.43	0.001	-13.20304206	Down	1.50E-63	6.59E-63
novel_pir1986	277	0	9.43	0.001	-13.20304206	Down	1.50E-63	6.57E-63
novel_pir1786	276	0	9.4	0.001	-13.19844504	Down	2.54E-63	1.11E-62
novel_pir1377	276	0	9.4	0.001	-13.19844504	Down	2.54E-63	1.10E-62
novel_pir187	275	0	9.37	0.001	-13.19383333	Down	4.28E-63	1.86E-62
novel_pir990	275	0	9.37	0.001	-13.19383333	Down	4.28E-63	1.85E-62
novel_pir1238	275	0	9.37	0.001	-13.19383333	Down	4.28E-63	1.85E-62
novel_pir2383	274	0	9.33	0.001	-13.18766137	Down	7.21E-63	3.11E-62
novel_pir97	274	0	9.33	0.001	-13.18766137	Down	7.21E-63	3.10E-62
novel_pir2363	273	0	9.3	0.001	-13.183015	Down	1.22E-62	5.21E-62
novel_pir1749	273	0	9.3	0.001	-13.183015	Down	1.22E-62	5.20E-62
novel_pir2137	272	0	9.26	0.001	-13.17679648	Down	2.05E-62	8.75E-62
novel_pir502	271	0	9.23	0.001	-13.17211493	Down	3.46E-62	1.47E-61
novel_pir98	271	0	9.23	0.001	-13.17211493	Down	3.46E-62	1.47E-61
novel_pir1336	270	0	9.19	0.001	-13.16584915	Down	5.84E-62	2.47E-61
novel_pir337	269	0	9.16	0.001	-13.16113188	Down	9.85E-62	4.17E-61
novel_pir1156	269	0	9.16	0.001	-13.16113188	Down	9.85E-62	4.16E-61
novel_pir1302	268	0	9.13	0.001	-13.15639914	Down	1.66E-61	7.00E-61
novel_pir754	268	0	9.13	0.001	-13.15639914	Down	1.66E-61	6.98E-61

novel_pir1715	268	0	9.13	0.001	-13.15639914	Down	1.66E-61	6.96E-61
novel_pir981	267	0	9.09	0.001	-13.15006458	Down	2.80E-61	1.17E-60
novel_pir1890	267	0	9.09	0.001	-13.15006458	Down	2.80E-61	1.17E-60
novel_pir1500	267	0	9.09	0.001	-13.15006458	Down	2.80E-61	1.17E-60
novel_pir1787	266	0	9.06	0.001	-13.14529533	Down	4.73E-61	1.96E-60
novel_pir48	266	0	9.06	0.001	-13.14529533	Down	4.73E-61	1.96E-60
novel_pir408	265	0	9.02	0.001	-13.13891172	Down	7.98E-61	3.30E-60
novel_pir1883	264	0	8.99	0.001	-13.1341054	Down	1.35E-60	5.54E-60
novel_pir1429	264	0	8.99	0.001	-13.1341054	Down	1.35E-60	5.53E-60
novel_pir9	263	0	8.96	0.001	-13.12928302	Down	2.27E-60	9.31E-60
novel_pir1681	263	0	8.96	0.001	-13.12928302	Down	2.27E-60	9.29E-60
novel_pir1992	262	0	8.92	0.001	-13.12282799	Down	3.83E-60	1.56E-59
novel_pir2243	262	0	8.92	0.001	-13.12282799	Down	3.83E-60	1.56E-59
novel_pir1153	261	0	8.89	0.001	-13.1179677	Down	6.46E-60	2.62E-59
novel_pir2216	261	0	8.89	0.001	-13.1179677	Down	6.46E-60	2.62E-59
novel_pir1848	261	0	8.89	0.001	-13.1179677	Down	6.46E-60	2.61E-59
novel_pir971	261	0	8.89	0.001	-13.1179677	Down	6.46E-60	2.60E-59
novel_pir1829	261	0	8.89	0.001	-13.1179677	Down	6.46E-60	2.60E-59
novel_pir2078	261	0	8.89	0.001	-13.1179677	Down	6.46E-60	2.59E-59
novel_pir1845	261	0	8.89	0.001	-13.1179677	Down	6.46E-60	2.59E-59
novel_pir293	260	0	8.85	0.001	-13.11146174	Down	1.09E-59	4.36E-59
novel_pir1391	259	0	8.82	0.001	-13.10656294	Down	1.84E-59	7.33E-59
novel_pir2305	259	0	8.82	0.001	-13.10656294	Down	1.84E-59	7.32E-59
novel_pir1105	259	0	8.82	0.001	-13.10656294	Down	1.84E-59	7.30E-59
novel_pir1425	259	0	8.82	0.001	-13.10656294	Down	1.84E-59	7.29E-59
novel_pir801	259	0	8.82	0.001	-13.10656294	Down	1.84E-59	7.27E-59
novel_pir2302	259	0	8.82	0.001	-13.10656294	Down	1.84E-59	7.26E-59
novel_pir1009	258	0	8.79	0.001	-13.10164745	Down	3.10E-59	1.22E-58
novel_pir1209	257	0	8.75	0.001	-13.0950673	Down	5.23E-59	2.05E-58
novel_pir892	257	0	8.75	0.001	-13.0950673	Down	5.23E-59	2.05E-58
novel_pir1310	257	0	8.75	0.001	-13.0950673	Down	5.23E-59	2.04E-58
novel_pir1041	257	0	8.75	0.001	-13.0950673	Down	5.23E-59	2.04E-58
novel_pir1348	256	0	8.72	0.001	-13.09011242	Down	8.82E-59	3.43E-58
novel_pir1822	256	0	8.72	0.001	-13.09011242	Down	8.82E-59	3.43E-58
novel_pir1339	256	0	8.72	0.001	-13.09011242	Down	8.82E-59	3.42E-58
novel_pir1753	255	0	8.68	0.001	-13.08347933	Down	1.49E-58	5.76E-58

novel_pir2212	255	0	8.68	0.001	-13.08347933	Down	1.49E-58	5.75E-58
novel_pir1126	254	0	8.65	0.001	-13.07848442	Down	2.51E-58	9.68E-58
novel_pir1882	253	0	8.62	0.001	-13.07347215	Down	4.23E-58	1.63E-57
novel_pir947	253	0	8.62	0.001	-13.07347215	Down	4.23E-58	1.62E-57
novel_pir1420	252	0	8.58	0.001	-13.06676193	Down	7.14E-58	2.73E-57
novel_pir1096	251	0	8.55	0.001	-13.0617087	Down	1.20E-57	4.60E-57
novel_pir122	251	0	8.55	0.001	-13.0617087	Down	1.20E-57	4.59E-57
novel_pir8	251	0	8.55	0.001	-13.0617087	Down	1.20E-57	4.58E-57
novel_pir1035	250	0	8.51	0.001	-13.05494342	Down	2.03E-57	7.71E-57
novel_pir1534	250	0	8.51	0.001	-13.05494342	Down	2.03E-57	7.69E-57
novel_pir584	250	0	8.51	0.001	-13.05494342	Down	2.03E-57	7.68E-57
novel_pir2253	249	0	8.48	0.001	-13.04984855	Down	3.43E-57	1.29E-56
novel_pir2229	249	0	8.48	0.001	-13.04984855	Down	3.43E-57	1.29E-56
novel_pir1248	249	0	8.48	0.001	-13.04984855	Down	3.43E-57	1.29E-56
novel_pir855	249	0	8.48	0.001	-13.04984855	Down	3.43E-57	1.29E-56
novel_pir2118	249	0	8.48	0.001	-13.04984855	Down	3.43E-57	1.28E-56
novel_pir1095	248	0	8.45	0.001	-13.04473563	Down	5.78E-57	2.16E-56
novel_pir135	248	0	8.45	0.001	-13.04473563	Down	5.78E-57	2.16E-56
novel_pir1128	248	0	8.45	0.001	-13.04473563	Down	5.78E-57	2.15E-56
novel_pir1800	248	0	8.45	0.001	-13.04473563	Down	5.78E-57	2.15E-56
novel_pir2372	247	0	8.41	0.001	-13.03789009	Down	9.75E-57	3.62E-56
novel_pir2111	247	0	8.41	0.001	-13.03789009	Down	9.75E-57	3.61E-56
novel_pir534	247	0	8.41	0.001	-13.03789009	Down	9.75E-57	3.60E-56
novel_pir1514	246	0	8.38	0.001	-13.03273453	Down	1.64E-56	6.05E-56
novel_pir1384	246	0	8.38	0.001	-13.03273453	Down	1.64E-56	6.04E-56
novel_pir1108	246	0	8.38	0.001	-13.03273453	Down	1.64E-56	6.03E-56
novel_pir444	244	0	8.31	0.001	-13.02063276	Down	4.68E-56	1.71E-55
novel_pir1098	243	0	8.28	0.001	-13.01541505	Down	7.89E-56	2.88E-55
novel_pir467	243	0	8.28	0.001	-13.01541505	Down	7.89E-56	2.87E-55
novel_pir1519	243	0	8.28	0.001	-13.01541505	Down	7.89E-56	2.87E-55
novel_pir12	243	0	8.28	0.001	-13.01541505	Down	7.89E-56	2.86E-55
novel_pir2165	243	0	8.28	0.001	-13.01541505	Down	7.89E-56	2.86E-55
novel_pir1092	242	0	8.24	0.001	-13.00842862	Down	1.33E-55	4.81E-55
novel_pir1662	241	0	8.21	0.001	-13.00316651	Down	2.25E-55	8.09E-55
novel_pir1416	241	0	8.21	0.001	-13.00316651	Down	2.25E-55	8.08E-55
novel_pir65	240	0	8.17	0.001	-12.99612036	Down	3.79E-55	1.36E-54

novel_pir1660	239	0	8.14	0.001	-12.99081308	Down	6.39E-55	2.29E-54
novel_pir1393	239	0	8.14	0.001	-12.99081308	Down	6.39E-55	2.28E-54
novel_pir1592	239	0	8.14	0.001	-12.99081308	Down	6.39E-55	2.28E-54
novel_pir1706	238	0	8.11	0.001	-12.9854862	Down	1.08E-54	3.83E-54
novel_pir590	238	0	8.11	0.001	-12.9854862	Down	1.08E-54	3.82E-54
novel_pir1770	238	0	8.11	0.001	-12.9854862	Down	1.08E-54	3.81E-54
novel_pir1462	238	0	8.11	0.001	-12.9854862	Down	1.08E-54	3.81E-54
novel_pir1650	238	0	8.11	0.001	-12.9854862	Down	1.08E-54	3.80E-54
novel_pir1723	237	0	8.07	0.001	-12.97835296	Down	1.82E-54	6.39E-54
novel_pir817	237	0	8.07	0.001	-12.97835296	Down	1.82E-54	6.37E-54
novel_pir821	237	0	8.07	0.001	-12.97835296	Down	1.82E-54	6.36E-54
novel_pir559	236	0	8.04	0.001	-12.97297979	Down	3.07E-54	1.07E-53
novel_pir2084	235	0	8	0.001	-12.96578428	Down	5.17E-54	1.80E-53
novel_pir464	235	0	8	0.001	-12.96578428	Down	5.17E-54	1.80E-53
novel_pir1430	235	0	8	0.001	-12.96578428	Down	5.17E-54	1.80E-53
novel_pir1019	234	0	7.97	0.001	-12.96036401	Down	8.73E-54	3.02E-53
novel_pir1978	233	0	7.93	0.001	-12.95310515	Down	1.47E-53	5.09E-53
novel_pir639	233	0	7.93	0.001	-12.95310515	Down	1.47E-53	5.08E-53
novel_pir658	233	0	7.93	0.001	-12.95310515	Down	1.47E-53	5.07E-53
novel_pir2236	233	0	7.93	0.001	-12.95310515	Down	1.47E-53	5.06E-53
novel_pir246	232	0	7.9	0.001	-12.94763694	Down	2.48E-53	8.50E-53
novel_pir2283	232	0	7.9	0.001	-12.94763694	Down	2.48E-53	8.49E-53
novel_pir576	232	0	7.9	0.001	-12.94763694	Down	2.48E-53	8.47E-53
novel_pir915	231	0	7.87	0.001	-12.94214792	Down	4.19E-53	1.42E-52
novel_pir859	231	0	7.87	0.001	-12.94214792	Down	4.19E-53	1.42E-52
novel_pir1533	230	0	7.83	0.001	-12.93479659	Down	7.06E-53	2.39E-52
novel_pir549	230	0	7.83	0.001	-12.93479659	Down	7.06E-53	2.39E-52
novel_pir249	229	0	7.8	0.001	-12.92925841	Down	1.19E-52	4.02E-52
novel_pir250	229	0	7.8	0.001	-12.92925841	Down	1.19E-52	4.01E-52
novel_pir822	229	0	7.8	0.001	-12.92925841	Down	1.19E-52	4.00E-52
novel_pir109	229	0	7.8	0.001	-12.92925841	Down	1.19E-52	4.00E-52
novel_pir496	229	0	7.8	0.001	-12.92925841	Down	1.19E-52	3.99E-52
novel_pir629	228	0	7.76	0.001	-12.92184094	Down	2.01E-52	6.72E-52
novel_pir327	228	0	7.76	0.001	-12.92184094	Down	2.01E-52	6.71E-52
novel_pir1870	228	0	7.76	0.001	-12.92184094	Down	2.01E-52	6.70E-52
novel_pir1060	228	0	7.76	0.001	-12.92184094	Down	2.01E-52	6.68E-52

novel_pir1593	227	0	7.73	0.001	-12.9162527	Down	3.39E-52	1.12E-51
novel_pir607	227	0	7.73	0.001	-12.9162527	Down	3.39E-52	1.12E-51
novel_pir1558	226	0	7.7	0.001	-12.91064273	Down	5.72E-52	1.89E-51
novel_pir46	226	0	7.7	0.001	-12.91064273	Down	5.72E-52	1.88E-51
novel_pir55	225	0	7.66	0.001	-12.90312868	Down	9.65E-52	3.17E-51
novel_pir835	225	0	7.66	0.001	-12.90312868	Down	9.65E-52	3.16E-51
novel_pir1793	225	0	7.66	0.001	-12.90312868	Down	9.65E-52	3.16E-51
novel_pir2144	224	0	7.63	0.001	-12.89746734	Down	1.63E-51	5.31E-51
novel_pir729	224	0	7.63	0.001	-12.89746734	Down	1.63E-51	5.30E-51
novel_pir35	224	0	7.63	0.001	-12.89746734	Down	1.63E-51	5.29E-51
novel_pir1312	223	0	7.59	0.001	-12.88988417	Down	2.74E-51	8.91E-51
novel_pir1278	222	0	7.56	0.001	-12.88417052	Down	4.63E-51	1.50E-50
novel_pir1259	222	0	7.56	0.001	-12.88417052	Down	4.63E-51	1.50E-50
novel_pir941	222	0	7.56	0.001	-12.88417052	Down	4.63E-51	1.50E-50
novel_pir2469	222	0	7.56	0.001	-12.88417052	Down	4.63E-51	1.49E-50
novel_pir1468	221	0	7.53	0.001	-12.87843415	Down	7.81E-51	2.51E-50
novel_pir1293	221	0	7.53	0.001	-12.87843415	Down	7.81E-51	2.51E-50
novel_pir2239	221	0	7.53	0.001	-12.87843415	Down	7.81E-51	2.51E-50
novel_pir919	221	0	7.53	0.001	-12.87843415	Down	7.81E-51	2.50E-50
novel_pir818	221	0	7.53	0.001	-12.87843415	Down	7.81E-51	2.50E-50
novel_pir11	220	0	7.49	0.001	-12.87075	Down	1.32E-50	4.19E-50
novel_pir488	220	0	7.49	0.001	-12.87075	Down	1.32E-50	4.19E-50
novel_pir689	220	0	7.49	0.001	-12.87075	Down	1.32E-50	4.18E-50
novel_pir2126	219	0	7.46	0.001	-12.86495992	Down	2.22E-50	7.03E-50
novel_pir1722	219	0	7.46	0.001	-12.86495992	Down	2.22E-50	7.02E-50
novel_pir2225	219	0	7.46	0.001	-12.86495992	Down	2.22E-50	7.00E-50
novel_pir1418	218	0	7.42	0.001	-12.85720347	Down	3.75E-50	1.18E-49
novel_pir1146	218	0	7.42	0.001	-12.85720347	Down	3.75E-50	1.18E-49
novel_pir213	217	0	7.39	0.001	-12.85135865	Down	6.32E-50	1.97E-49
novel_pir100	217	0	7.39	0.001	-12.85135865	Down	6.32E-50	1.97E-49
novel_pir477	216	0	7.36	0.001	-12.84549005	Down	1.07E-49	3.31E-49
novel_pir1239	216	0	7.36	0.001	-12.84549005	Down	1.07E-49	3.31E-49
novel_pir2081	216	0	7.36	0.001	-12.84549005	Down	1.07E-49	3.30E-49
novel_pir465	216	0	7.36	0.001	-12.84549005	Down	1.07E-49	3.30E-49
novel_pir1328	216	0	7.36	0.001	-12.84549005	Down	1.07E-49	3.29E-49
novel_pir2347	215	0	7.32	0.001	-12.83762793	Down	1.80E-49	5.53E-49

novel_pir1047	215	0	7.32	0.001	-12.83762793	Down	1.80E-49	5.53E-49
novel_pir404	215	0	7.32	0.001	-12.83762793	Down	1.80E-49	5.52E-49
novel_pir1618	214	0	7.29	0.001	-12.8317031	Down	3.03E-49	9.29E-49
novel_pir1930	214	0	7.29	0.001	-12.8317031	Down	3.03E-49	9.28E-49
novel_pir139	213	0	7.25	0.001	-12.82376528	Down	5.12E-49	1.56E-48
novel_pir2148	213	0	7.25	0.001	-12.82376528	Down	5.12E-49	1.55E-48
novel_pir2298	212	0	7.22	0.001	-12.81778312	Down	8.63E-49	2.61E-48
novel_pir735	212	0	7.22	0.001	-12.81778312	Down	8.63E-49	2.61E-48
novel_pir1710	210	0	7.15	0.001	-12.80372753	Down	2.46E-48	7.42E-48
novel_pir104	209	0	7.12	0.001	-12.79766153	Down	4.14E-48	1.25E-47
novel_pir1423	209	0	7.12	0.001	-12.79766153	Down	4.14E-48	1.25E-47
novel_pir2183	208	0	7.08	0.001	-12.78953364	Down	6.99E-48	2.10E-47
novel_pir589	207	0	7.05	0.001	-12.78340754	Down	1.18E-47	3.54E-47
novel_pir2222	207	0	7.05	0.001	-12.78340754	Down	1.18E-47	3.53E-47
novel_pir1901	206	0	7.02	0.001	-12.77725532	Down	1.99E-47	5.95E-47
novel_pir2161	206	0	7.02	0.001	-12.77725532	Down	1.99E-47	5.94E-47
novel_pir1754	204	0	6.95	0.001	-12.76279726	Down	5.66E-47	1.69E-46
novel_pir1295	203	0	6.91	0.001	-12.75447	Down	9.55E-47	2.84E-46
novel_pir683	203	0	6.91	0.001	-12.75447	Down	9.55E-47	2.84E-46
novel_pir1094	203	0	6.91	0.001	-12.75447	Down	9.55E-47	2.83E-46
novel_pir1674	202	0	6.88	0.001	-12.74819285	Down	1.61E-46	4.77E-46
novel_pir723	202	0	6.88	0.001	-12.74819285	Down	1.61E-46	4.76E-46
novel_pir509	202	0	6.88	0.001	-12.74819285	Down	1.61E-46	4.76E-46
novel_pir455	202	0	6.88	0.001	-12.74819285	Down	1.61E-46	4.75E-46
novel_pir54	201	0	6.85	0.001	-12.74188827	Down	2.72E-46	7.99E-46
novel_pir1243	201	0	6.85	0.001	-12.74188827	Down	2.72E-46	7.97E-46
novel_pir948	200	0	6.81	0.001	-12.73343908	Down	4.58E-46	1.34E-45
novel_pir2114	200	0	6.81	0.001	-12.73343908	Down	4.58E-46	1.34E-45
novel_pir1187	200	0	6.81	0.001	-12.73343908	Down	4.58E-46	1.34E-45
novel_pir1897	199	0	6.78	0.001	-12.72706956	Down	7.73E-46	2.25E-45
novel_pir1251	199	0	6.78	0.001	-12.72706956	Down	7.73E-46	2.25E-45
novel_pir2352	199	0	6.78	0.001	-12.72706956	Down	7.73E-46	2.25E-45
novel_pir672	198	0	6.74	0.001	-12.71853288	Down	1.30E-45	3.77E-45
novel_pir891	198	0	6.74	0.001	-12.71853288	Down	1.30E-45	3.76E-45
novel_pir705	198	0	6.74	0.001	-12.71853288	Down	1.30E-45	3.76E-45
novel_pir258	197	0	6.71	0.001	-12.71209705	Down	2.20E-45	6.33E-45

novel_pir1117	196	0	6.67	0.001	-12.70347105	Down	3.71E-45	1.06E-44
novel_pir1150	195	0	6.64	0.001	-12.69696753	Down	6.26E-45	1.79E-44
novel_pir1761	195	0	6.64	0.001	-12.69696753	Down	6.26E-45	1.79E-44
novel_pir2267	195	0	6.64	0.001	-12.69696753	Down	6.26E-45	1.79E-44
novel_pir2307	194	0	6.61	0.001	-12.69043456	Down	1.06E-44	3.01E-44
novel_pir2009	194	0	6.61	0.001	-12.69043456	Down	1.06E-44	3.00E-44
novel_pir909	194	0	6.61	0.001	-12.69043456	Down	1.06E-44	3.00E-44
novel_pir1589	193	0	6.57	0.001	-12.68167766	Down	1.78E-44	5.04E-44
novel_pir1625	193	0	6.57	0.001	-12.68167766	Down	1.78E-44	5.04E-44
novel_pir286	193	0	6.57	0.001	-12.68167766	Down	1.78E-44	5.03E-44
novel_pir745	193	0	6.57	0.001	-12.68167766	Down	1.78E-44	5.02E-44
novel_pir2102	192	0	6.54	0.001	-12.67507492	Down	3.00E-44	8.46E-44
novel_pir952	191	0	6.5	0.001	-12.666224	Down	5.07E-44	1.42E-43
novel_pir2313	191	0	6.5	0.001	-12.666224	Down	5.07E-44	1.42E-43
novel_pir1513	191	0	6.5	0.001	-12.666224	Down	5.07E-44	1.42E-43
novel_pir831	190	0	6.47	0.001	-12.65955	Down	8.54E-44	2.39E-43
novel_pir762	190	0	6.47	0.001	-12.65955	Down	8.54E-44	2.39E-43
novel_pir1553	190	0	6.47	0.001	-12.65955	Down	8.54E-44	2.39E-43
novel_pir561	189	0	6.44	0.001	-12.65284497	Down	1.44E-43	4.01E-43
novel_pir838	189	0	6.44	0.001	-12.65284497	Down	1.44E-43	4.01E-43
novel_pir1342	188	0	6.4	0.001	-12.64385619	Down	2.43E-43	6.75E-43
novel_pir2021	188	0	6.4	0.001	-12.64385619	Down	2.43E-43	6.74E-43
novel_pir868	188	0	6.4	0.001	-12.64385619	Down	2.43E-43	6.73E-43
novel_pir953	187	0	6.37	0.001	-12.63707766	Down	4.10E-43	1.13E-42
novel_pir734	187	0	6.37	0.001	-12.63707766	Down	4.10E-43	1.13E-42
novel_pir1163	187	0	6.37	0.001	-12.63707766	Down	4.10E-43	1.13E-42
novel_pir1362	187	0	6.37	0.001	-12.63707766	Down	4.10E-43	1.13E-42
novel_pir1925	187	0	6.37	0.001	-12.63707766	Down	4.10E-43	1.13E-42
novel_pir1505	186	0	6.33	0.001	-12.62798978	Down	6.92E-43	1.90E-42
novel_pir1560	186	0	6.33	0.001	-12.62798978	Down	6.92E-43	1.89E-42
novel_pir280	185	0	6.3	0.001	-12.62113611	Down	1.17E-42	3.19E-42
novel_pir643	184	0	6.27	0.001	-12.61424973	Down	1.97E-42	5.36E-42
novel_pir2249	183	0	6.23	0.001	-12.60501645	Down	3.32E-42	9.03E-42
novel_pir2377	183	0	6.23	0.001	-12.60501645	Down	3.32E-42	9.02E-42
novel_pir1966	183	0	6.23	0.001	-12.60501645	Down	3.32E-42	9.01E-42
novel_pir1286	182	0	6.2	0.001	-12.5980525	Down	5.60E-42	1.52E-41

novel_pir2290	182	0	6.2	0.001	-12.5980525	Down	5.60E-42	1.52E-41
novel_pir1658	182	0	6.2	0.001	-12.5980525	Down	5.60E-42	1.51E-41
novel_pir323	182	0	6.2	0.001	-12.5980525	Down	5.60E-42	1.51E-41
novel_pir1315	181	0	6.16	0.001	-12.58871464	Down	9.45E-42	2.55E-41
novel_pir1473	181	0	6.16	0.001	-12.58871464	Down	9.45E-42	2.54E-41
novel_pir57	179	0	6.1	0.001	-12.57459353	Down	2.69E-41	7.21E-41
novel_pir1622	178	0	6.06	0.001	-12.56510208	Down	4.53E-41	1.21E-40
novel_pir1529	178	0	6.06	0.001	-12.56510208	Down	4.53E-41	1.21E-40
novel_pir1470	178	0	6.06	0.001	-12.56510208	Down	4.53E-41	1.21E-40
novel_pir2458	178	0	6.06	0.001	-12.56510208	Down	4.53E-41	1.21E-40
novel_pir1119	178	0	6.06	0.001	-12.56510208	Down	4.53E-41	1.21E-40
novel_pir1419	178	0	6.06	0.001	-12.56510208	Down	4.53E-41	1.21E-40
novel_pir1607	178	0	6.06	0.001	-12.56510208	Down	4.53E-41	1.20E-40
novel_pir874	178	0	6.06	0.001	-12.56510208	Down	4.53E-41	1.20E-40
novel_pir1048	177	0	6.03	0.001	-12.55794229	Down	7.65E-41	2.03E-40
novel_pir1577	176	0	5.99	0.001	-12.54834029	Down	1.29E-40	3.40E-40
novel_pir1799	176	0	5.99	0.001	-12.54834029	Down	1.29E-40	3.39E-40
novel_pir2442	175	0	5.96	0.001	-12.54109662	Down	2.18E-40	5.71E-40
novel_pir1537	175	0	5.96	0.001	-12.54109662	Down	2.18E-40	5.70E-40
novel_pir248	175	0	5.96	0.001	-12.54109662	Down	2.18E-40	5.70E-40
novel_pir1692	174	0	5.93	0.001	-12.53381639	Down	3.67E-40	9.60E-40
novel_pir970	174	0	5.93	0.001	-12.53381639	Down	3.67E-40	9.58E-40
novel_pir2186	172	0	5.86	0.001	-12.51668495	Down	1.04E-39	2.72E-39
novel_pir1321	172	0	5.86	0.001	-12.51668495	Down	1.04E-39	2.72E-39
novel_pir1018	172	0	5.86	0.001	-12.51668495	Down	1.04E-39	2.72E-39
novel_pir1783	172	0	5.86	0.001	-12.51668495	Down	1.04E-39	2.71E-39
novel_pir2454	171	0	5.82	0.001	-12.50680344	Down	1.76E-39	4.56E-39
novel_pir110	171	0	5.82	0.001	-12.50680344	Down	1.76E-39	4.55E-39
novel_pir2343	170	0	5.79	0.001	-12.49934763	Down	2.97E-39	7.65E-39
novel_pir1270	170	0	5.79	0.001	-12.49934763	Down	2.97E-39	7.64E-39
novel_pir742	170	0	5.79	0.001	-12.49934763	Down	2.97E-39	7.63E-39
novel_pir1802	170	0	5.79	0.001	-12.49934763	Down	2.97E-39	7.62E-39
novel_pir2274	167	0	5.69	0.001	-12.47421294	Down	1.43E-38	3.65E-38
novel_pir979	167	0	5.69	0.001	-12.47421294	Down	1.43E-38	3.64E-38
novel_pir2285	167	0	5.69	0.001	-12.47421294	Down	1.43E-38	3.64E-38
novel_pir918	167	0	5.69	0.001	-12.47421294	Down	1.43E-38	3.63E-38

novel_pir2180	167	0	5.69	0.001	-12.47421294	Down	1.43E-38	3.63E-38
novel_pir1758	165	0	5.62	0.001	-12.45635442	Down	4.06E-38	1.03E-37
novel_pir1806	165	0	5.62	0.001	-12.45635442	Down	4.06E-38	1.03E-37
novel_pir1906	164	0	5.59	0.001	-12.44863257	Down	6.84E-38	1.73E-37
novel_pir2355	164	0	5.59	0.001	-12.44863257	Down	6.84E-38	1.73E-37
novel_pir2045	164	0	5.59	0.001	-12.44863257	Down	6.84E-38	1.73E-37
novel_pir1661	164	0	5.59	0.001	-12.44863257	Down	6.84E-38	1.73E-37
novel_pir1877	163	0	5.55	0.001	-12.43827206	Down	1.15E-37	2.91E-37
novel_pir712	163	0	5.55	0.001	-12.43827206	Down	1.15E-37	2.90E-37
novel_pir1873	163	0	5.55	0.001	-12.43827206	Down	1.15E-37	2.90E-37
novel_pir1948	162	0	5.52	0.001	-12.43045255	Down	1.95E-37	4.89E-37
novel_pir1125	161	0	5.48	0.001	-12.41996018	Down	3.28E-37	8.23E-37
novel_pir312	158	0	5.38	0.001	-12.39339046	Down	1.58E-36	3.93E-36
novel_pir1872	157	0	5.35	0.001	-12.38532318	Down	2.66E-36	6.61E-36
novel_pir1675	157	0	5.35	0.001	-12.38532318	Down	2.66E-36	6.60E-36
novel_pir1525	157	0	5.35	0.001	-12.38532318	Down	2.66E-36	6.60E-36
novel_pir29	157	0	5.35	0.001	-12.38532318	Down	2.66E-36	6.59E-36
novel_pir828	157	0	5.35	0.001	-12.38532318	Down	2.66E-36	6.58E-36
novel_pir1860	156	0	5.31	0.001	-12.37449615	Down	4.49E-36	1.11E-35
novel_pir1719	155	0	5.28	0.001	-12.36632221	Down	7.57E-36	1.87E-35
novel_pir2190	155	0	5.28	0.001	-12.36632221	Down	7.57E-36	1.86E-35
novel_pir2116	154	0	5.24	0.001	-12.3553511	Down	1.28E-35	3.14E-35
novel_pir1708	154	0	5.24	0.001	-12.3553511	Down	1.28E-35	3.14E-35
novel_pir1169	153	0	5.21	0.001	-12.34706766	Down	2.15E-35	5.27E-35
novel_pir1089	153	0	5.21	0.001	-12.34706766	Down	2.15E-35	5.27E-35
novel_pir301	152	0	5.18	0.001	-12.33873638	Down	3.63E-35	8.87E-35
novel_pir1784	151	0	5.14	0.001	-12.32755264	Down	6.13E-35	1.49E-34
novel_pir814	150	0	5.11	0.001	-12.31910758	Down	1.03E-34	2.51E-34
novel_pir1178	149	0	5.07	0.001	-12.30777003	Down	1.74E-34	4.22E-34
novel_pir2430	149	0	5.07	0.001	-12.30777003	Down	1.74E-34	4.21E-34
novel_pir657	149	0	5.07	0.001	-12.30777003	Down	1.74E-34	4.21E-34
novel_pir1667	149	0	5.07	0.001	-12.30777003	Down	1.74E-34	4.20E-34
novel_pir794	148	0	5.04	0.001	-12.29920802	Down	2.94E-34	7.08E-34
novel_pir929	148	0	5.04	0.001	-12.29920802	Down	2.94E-34	7.07E-34
novel_pir916	147	0	5.01	0.001	-12.29059489	Down	4.96E-34	1.19E-33
novel_pir310	147	0	5.01	0.001	-12.29059489	Down	4.96E-34	1.19E-33

novel_pir1487	146	0	4.97	0.001	-12.27903014	Down	8.37E-34	1.99E-33
novel_pir424	145	0	4.94	0.001	-12.27029533	Down	1.41E-33	3.35E-33
novel_pir732	145	0	4.94	0.001	-12.27029533	Down	1.41E-33	3.35E-33
novel_pir1698	145	0	4.94	0.001	-12.27029533	Down	1.41E-33	3.34E-33
novel_pir236	145	0	4.94	0.001	-12.27029533	Down	1.41E-33	3.34E-33
novel_pir744	144	0	4.9	0.001	-12.25856603	Down	2.38E-33	5.62E-33
novel_pir614	143	0	4.87	0.001	-12.24970606	Down	4.02E-33	9.45E-33
novel_pir1773	143	0	4.87	0.001	-12.24970606	Down	4.02E-33	9.44E-33
novel_pir1122	143	0	4.87	0.001	-12.24970606	Down	4.02E-33	9.43E-33
novel_pir1372	142	0	4.84	0.001	-12.24079133	Down	6.77E-33	1.59E-32
novel_pir1969	142	0	4.84	0.001	-12.24079133	Down	6.77E-33	1.59E-32
novel_pir2167	142	0	4.84	0.001	-12.24079133	Down	6.77E-33	1.58E-32
novel_pir1947	141	0	4.8	0.001	-12.22881869	Down	1.14E-32	2.67E-32
novel_pir2465	141	0	4.8	0.001	-12.22881869	Down	1.14E-32	2.67E-32
novel_pir2117	141	0	4.8	0.001	-12.22881869	Down	1.14E-32	2.66E-32
novel_pir394	140	0	4.77	0.001	-12.21977355	Down	1.93E-32	4.48E-32
novel_pir1093	140	0	4.77	0.001	-12.21977355	Down	1.93E-32	4.47E-32
novel_pir1809	140	0	4.77	0.001	-12.21977355	Down	1.93E-32	4.47E-32
novel_pir935	138	0	4.7	0.001	-12.19844504	Down	5.48E-32	1.26E-31
novel_pir593	138	0	4.7	0.001	-12.19844504	Down	5.48E-32	1.26E-31
novel_pir844	138	0	4.7	0.001	-12.19844504	Down	5.48E-32	1.26E-31
novel_pir2025	138	0	4.7	0.001	-12.19844504	Down	5.48E-32	1.26E-31
novel_pir2061	137	0	4.67	0.001	-12.18920683	Down	9.25E-32	2.12E-31
novel_pir798	137	0	4.67	0.001	-12.18920683	Down	9.25E-32	2.12E-31
novel_pir1240	137	0	4.67	0.001	-12.18920683	Down	9.25E-32	2.12E-31
novel_pir585	136	0	4.63	0.001	-12.17679648	Down	1.56E-31	3.56E-31
novel_pir1797	136	0	4.63	0.001	-12.17679648	Down	1.56E-31	3.55E-31
novel_pir2350	136	0	4.63	0.001	-12.17679648	Down	1.56E-31	3.55E-31
novel_pir1242	135	0	4.6	0.001	-12.16741815	Down	2.63E-31	5.98E-31
novel_pir1991	135	0	4.6	0.001	-12.16741815	Down	2.63E-31	5.97E-31
novel_pir2238	135	0	4.6	0.001	-12.16741815	Down	2.63E-31	5.97E-31
novel_pir1171	134	0	4.56	0.001	-12.15481811	Down	4.44E-31	1.00E-30
novel_pir925	134	0	4.56	0.001	-12.15481811	Down	4.44E-31	1.00E-30
novel_pir2150	133	0	4.53	0.001	-12.14529533	Down	7.49E-31	1.69E-30
novel_pir266	133	0	4.53	0.001	-12.14529533	Down	7.49E-31	1.69E-30
novel_pir64	132	0	4.5	0.001	-12.13570929	Down	1.26E-30	2.84E-30

novel_pir2213	132	0	4.5	0.001	-12.13570929	Down	1.26E-30	2.83E-30
novel_pir318	132	0	4.5	0.001	-12.13570929	Down	1.26E-30	2.83E-30
novel_pir994	132	0	4.5	0.001	-12.13570929	Down	1.26E-30	2.83E-30
novel_pir1612	131	0	4.46	0.001	-12.12282799	Down	2.13E-30	4.76E-30
novel_pir1790	131	0	4.46	0.001	-12.12282799	Down	2.13E-30	4.75E-30
novel_pir1138	131	0	4.46	0.001	-12.12282799	Down	2.13E-30	4.75E-30
novel_pir1472	130	0	4.43	0.001	-12.11309098	Down	3.59E-30	8.00E-30
novel_pir827	130	0	4.43	0.001	-12.11309098	Down	3.59E-30	7.99E-30
novel_pir2141	128	0	4.36	0.001	-12.09011242	Down	1.02E-29	2.27E-29
novel_pir879	128	0	4.36	0.001	-12.09011242	Down	1.02E-29	2.26E-29
novel_pir1683	127	0	4.33	0.001	-12.08015131	Down	1.72E-29	3.80E-29
novel_pir1147	127	0	4.33	0.001	-12.08015131	Down	1.72E-29	3.79E-29
novel_pir1212	126	0	4.29	0.001	-12.06676193	Down	2.91E-29	6.37E-29
novel_pir2145	125	0	4.26	0.001	-12.05663772	Down	4.91E-29	1.07E-28
novel_pir2284	125	0	4.26	0.001	-12.05663772	Down	4.91E-29	1.07E-28
novel_pir1399	124	0	4.22	0.001	-12.04302728	Down	8.28E-29	1.80E-28
novel_pir500	124	0	4.22	0.001	-12.04302728	Down	8.28E-29	1.80E-28
novel_pir2146	124	0	4.22	0.001	-12.04302728	Down	8.28E-29	1.80E-28
novel_pir1211	124	0	4.22	0.001	-12.04302728	Down	8.28E-29	1.79E-28
novel_pir2125	123	0	4.19	0.001	-12.03273453	Down	1.40E-28	3.02E-28
novel_pir2341	123	0	4.19	0.001	-12.03273453	Down	1.40E-28	3.02E-28
novel_pir2344	122	0	4.15	0.001	-12.01889562	Down	2.36E-28	5.08E-28
novel_pir405	122	0	4.15	0.001	-12.01889562	Down	2.36E-28	5.08E-28
novel_pir1182	122	0	4.15	0.001	-12.01889562	Down	2.36E-28	5.07E-28
novel_pir846	121	0	4.12	0.001	-12.00842862	Down	3.97E-28	8.52E-28
novel_pir1536	120	0	4.09	0.001	-11.99788513	Down	6.70E-28	1.44E-27
novel_pir34	120	0	4.09	0.001	-11.99788513	Down	6.70E-28	1.43E-27
novel_pir644	119	0	4.05	0.001	-11.98370619	Down	1.13E-27	2.41E-27
novel_pir2475	119	0	4.05	0.001	-11.98370619	Down	1.13E-27	2.41E-27
novel_pir1725	119	0	4.05	0.001	-11.98370619	Down	1.13E-27	2.40E-27
novel_pir543	119	0	4.05	0.001	-11.98370619	Down	1.13E-27	2.40E-27
novel_pir1076	118	0	4.02	0.001	-11.97297979	Down	1.91E-27	4.04E-27
novel_pir1811	118	0	4.02	0.001	-11.97297979	Down	1.91E-27	4.04E-27
novel_pir1530	118	0	4.02	0.001	-11.97297979	Down	1.91E-27	4.03E-27
novel_pir2203	117	0	3.98	0.001	-11.95855272	Down	3.22E-27	6.78E-27
novel_pir1143	117	0	3.98	0.001	-11.95855272	Down	3.22E-27	6.77E-27

novel_pir1361	117	0	3.98	0.001	-11.95855272	Down	3.22E-27	6.76E-27
novel_pir987	117	0	3.98	0.001	-11.95855272	Down	3.22E-27	6.75E-27
novel_pir72	116	0	3.95	0.001	-11.94763694	Down	5.43E-27	1.14E-26
novel_pir1554	115	0	3.92	0.001	-11.93663794	Down	9.15E-27	1.92E-26
novel_pir397	114	0	3.88	0.001	-11.92184094	Down	1.54E-26	3.22E-26
novel_pir803	114	0	3.88	0.001	-11.92184094	Down	1.54E-26	3.22E-26
novel_pir2242	113	0	3.85	0.001	-11.91064273	Down	2.60E-26	5.43E-26
novel_pir2320	113	0	3.85	0.001	-11.91064273	Down	2.60E-26	5.42E-26
novel_pir472	113	0	3.85	0.001	-11.91064273	Down	2.60E-26	5.42E-26
novel_pir284	113	0	3.85	0.001	-11.91064273	Down	2.60E-26	5.41E-26
novel_pir1228	112	0	3.81	0.001	-11.89557528	Down	4.39E-26	9.11E-26
novel_pir355	112	0	3.81	0.001	-11.89557528	Down	4.39E-26	9.10E-26
novel_pir2445	112	0	3.81	0.001	-11.89557528	Down	4.39E-26	9.09E-26
novel_pir1071	111	0	3.78	0.001	-11.88417052	Down	7.41E-26	1.53E-25
novel_pir1049	110	0	3.75	0.001	-11.87267488	Down	1.25E-25	2.57E-25
novel_pir903	110	0	3.75	0.001	-11.87267488	Down	1.25E-25	2.57E-25
novel_pir1202	110	0	3.75	0.001	-11.87267488	Down	1.25E-25	2.57E-25
novel_pir1768	109	0	3.71	0.001	-11.85720347	Down	2.11E-25	4.33E-25
novel_pir1981	108	0	3.68	0.001	-11.84549005	Down	3.56E-25	7.24E-25
novel_pir771	107	0	3.64	0.001	-11.82972274	Down	6.00E-25	1.22E-24
novel_pir1796	107	0	3.64	0.001	-11.82972274	Down	6.00E-25	1.22E-24
novel_pir332	106	0	3.61	0.001	-11.81778312	Down	1.01E-24	2.05E-24
novel_pir2194	106	0	3.61	0.001	-11.81778312	Down	1.01E-24	2.05E-24
novel_pir1422	105	0	3.58	0.001	-11.80574387	Down	1.71E-24	3.45E-24
novel_pir333	105	0	3.58	0.001	-11.80574387	Down	1.71E-24	3.44E-24
novel_pir1201	105	0	3.58	0.001	-11.80574387	Down	1.71E-24	3.44E-24
novel_pir227	105	0	3.58	0.001	-11.80574387	Down	1.71E-24	3.43E-24
novel_pir2079	104	0	3.54	0.001	-11.78953364	Down	2.88E-24	5.79E-24
novel_pir518	103	0	3.51	0.001	-11.77725532	Down	4.86E-24	9.74E-24
novel_pir1837	102	0	3.47	0.001	-11.76071995	Down	8.19E-24	1.63E-23
novel_pir1063	102	0	3.47	0.001	-11.76071995	Down	8.19E-24	1.63E-23
novel_pir1549	101	0	3.44	0.001	-11.74819285	Down	1.38E-23	2.75E-23
novel_pir750	101	0	3.44	0.001	-11.74819285	Down	1.38E-23	2.75E-23
novel_pir1491	101	0	3.44	0.001	-11.74819285	Down	1.38E-23	2.75E-23
novel_pir21	100	0	3.41	0.001	-11.73555602	Down	2.33E-23	4.62E-23
novel_pir1944	100	0	3.41	0.001	-11.73555602	Down	2.33E-23	4.62E-23

novel_pir654	98	0	3.34	0.001	-11.70563239	Down	6.63E-23	1.31E-22
novel_pir1619	97	0	3.3	0.001	-11.68825031	Down	1.12E-22	2.21E-22
novel_pir1782	97	0	3.3	0.001	-11.68825031	Down	1.12E-22	2.20E-22
novel_pir2264	97	0	3.3	0.001	-11.68825031	Down	1.12E-22	2.20E-22
novel_pir2370	95	0	3.24	0.001	-11.6617781	Down	3.18E-22	6.22E-22
novel_pir1287	95	0	3.24	0.001	-11.6617781	Down	3.18E-22	6.22E-22
novel_pir1483	94	0	3.2	0.001	-11.64385619	Down	5.37E-22	1.05E-21
novel_pir1276	93	0	3.17	0.001	-11.63026713	Down	9.06E-22	1.76E-21
novel_pir1880	93	0	3.17	0.001	-11.63026713	Down	9.06E-22	1.75E-21
novel_pir2147	93	0	3.17	0.001	-11.63026713	Down	9.06E-22	1.75E-21
novel_pir968	93	0	3.17	0.001	-11.63026713	Down	9.06E-22	1.75E-21
novel_pir1309	92	0	3.13	0.001	-11.61194694	Down	1.53E-21	2.95E-21
novel_pir1714	92	0	3.13	0.001	-11.61194694	Down	1.53E-21	2.95E-21
novel_pir1357	92	0	3.13	0.001	-11.61194694	Down	1.53E-21	2.95E-21
novel_pir1946	91	0	3.1	0.001	-11.5980525	Down	2.58E-21	4.94E-21
novel_pir403	90	0	3.06	0.001	-11.57931594	Down	4.35E-21	8.31E-21
novel_pir696	89	0	3.03	0.001	-11.56510208	Down	7.33E-21	1.40E-20
novel_pir886	89	0	3.03	0.001	-11.56510208	Down	7.33E-21	1.40E-20
novel_pir478	88	0	3	0.001	-11.55074679	Down	1.24E-20	2.36E-20
novel_pir776	88	0	3	0.001	-11.55074679	Down	1.24E-20	2.35E-20
novel_pir322	88	0	3	0.001	-11.55074679	Down	1.24E-20	2.35E-20
novel_pir179	88	0	3	0.001	-11.55074679	Down	1.24E-20	2.35E-20
novel_pir624	87	0	2.96	0.001	-11.53138146	Down	2.09E-20	3.96E-20
novel_pir924	87	0	2.96	0.001	-11.53138146	Down	2.09E-20	3.96E-20
novel_pir1289	87	0	2.96	0.001	-11.53138146	Down	2.09E-20	3.95E-20
novel_pir912	86	0	2.93	0.001	-11.51668495	Down	3.52E-20	6.64E-20
novel_pir1410	86	0	2.93	0.001	-11.51668495	Down	3.52E-20	6.63E-20
novel_pir2259	86	0	2.93	0.001	-11.51668495	Down	3.52E-20	6.63E-20
novel_pir2106	85	0	2.89	0.001	-11.49685378	Down	5.94E-20	1.12E-19
novel_pir1132	84	0	2.86	0.001	-11.48179943	Down	1.00E-19	1.88E-19
novel_pir1861	84	0	2.86	0.001	-11.48179943	Down	1.00E-19	1.88E-19
novel_pir2431	84	0	2.86	0.001	-11.48179943	Down	1.00E-19	1.87E-19
novel_pir1235	83	0	2.83	0.001	-11.46658634	Down	1.69E-19	3.16E-19
novel_pir1051	83	0	2.83	0.001	-11.46658634	Down	1.69E-19	3.16E-19
novel_pir1741	82	0	2.79	0.001	-11.44604941	Down	2.85E-19	5.31E-19
novel_pir1844	82	0	2.79	0.001	-11.44604941	Down	2.85E-19	5.31E-19

novel_pir2440	80	0	2.72	0.001	-11.40939094	Down	8.11E-19	1.51E-18
novel_pir2261	79	0	2.69	0.001	-11.39339046	Down	1.37E-18	2.52E-18
novel_pir307	79	0	2.69	0.001	-11.39339046	Down	1.37E-18	2.52E-18
novel_pir960	78	0	2.66	0.001	-11.37721053	Down	2.31E-18	4.24E-18
novel_pir1352	78	0	2.66	0.001	-11.37721053	Down	2.31E-18	4.23E-18
novel_pir1869	78	0	2.66	0.001	-11.37721053	Down	2.31E-18	4.23E-18
novel_pir1835	77	0	2.62	0.001	-11.3553511	Down	3.89E-18	7.12E-18
novel_pir1686	77	0	2.62	0.001	-11.3553511	Down	3.89E-18	7.12E-18
novel_pir30	77	0	2.62	0.001	-11.3553511	Down	3.89E-18	7.11E-18
novel_pir810	77	0	2.62	0.001	-11.3553511	Down	3.89E-18	7.10E-18
novel_pir562	76	0	2.59	0.001	-11.33873638	Down	6.56E-18	1.19E-17
novel_pir2428	75	0	2.55	0.001	-11.31628153	Down	1.11E-17	2.01E-17
novel_pir1810	74	0	2.52	0.001	-11.29920802	Down	1.87E-17	3.38E-17
novel_pir2426	74	0	2.52	0.001	-11.29920802	Down	1.87E-17	3.38E-17
novel_pir2473	73	0	2.49	0.001	-11.28193003	Down	3.15E-17	5.69E-17
novel_pir1555	73	0	2.49	0.001	-11.28193003	Down	3.15E-17	5.68E-17
novel_pir244	72	0	2.45	0.001	-11.25856603	Down	5.31E-17	9.53E-17
novel_pir2345	72	0	2.45	0.001	-11.25856603	Down	5.31E-17	9.52E-17
novel_pir361	72	0	2.45	0.001	-11.25856603	Down	5.31E-17	9.51E-17
novel_pir1085	71	0	2.42	0.001	-11.24079133	Down	8.96E-17	1.60E-16
novel_pir1521	71	0	2.42	0.001	-11.24079133	Down	8.96E-17	1.60E-16
novel_pir2342	71	0	2.42	0.001	-11.24079133	Down	8.96E-17	1.60E-16
novel_pir1124	71	0	2.42	0.001	-11.24079133	Down	8.96E-17	1.59E-16
novel_pir2028	71	0	2.42	0.001	-11.24079133	Down	8.96E-17	1.59E-16
novel_pir1268	70	0	2.38	0.001	-11.21674586	Down	1.51E-16	2.68E-16
novel_pir1550	69	0	2.35	0.001	-11.19844504	Down	2.55E-16	4.51E-16
novel_pir1164	69	0	2.35	0.001	-11.19844504	Down	2.55E-16	4.50E-16
novel_pir777	69	0	2.35	0.001	-11.19844504	Down	2.55E-16	4.50E-16
novel_pir2337	69	0	2.35	0.001	-11.19844504	Down	2.55E-16	4.49E-16
novel_pir2019	68	0	2.32	0.001	-11.17990909	Down	4.30E-16	7.57E-16
novel_pir1081	68	0	2.32	0.001	-11.17990909	Down	4.30E-16	7.57E-16
novel_pir2187	67	0	2.28	0.001	-11.15481811	Down	7.25E-16	1.27E-15
novel_pir1923	67	0	2.28	0.001	-11.15481811	Down	7.25E-16	1.27E-15
novel_pir1959	66	0	2.25	0.001	-11.13570929	Down	1.22E-15	2.12E-15
novel_pir962	66	0	2.25	0.001	-11.13570929	Down	1.22E-15	2.12E-15
novel_pir2188	65	0	2.21	0.001	-11.10983065	Down	2.06E-15	3.57E-15

novel_pir661	65	0	2.21	0.001	-11.10983065	Down	2.06E-15	3.56E-15
novel_pir1938	64	0	2.18	0.001	-11.09011242	Down	3.48E-15	5.97E-15
novel_pir1299	64	0	2.18	0.001	-11.09011242	Down	3.48E-15	5.97E-15
novel_pir522	64	0	2.18	0.001	-11.09011242	Down	3.48E-15	5.96E-15
novel_pir1257	63	0	2.15	0.001	-11.07012094	Down	5.87E-15	1.01E-14
novel_pir896	63	0	2.15	0.001	-11.07012094	Down	5.87E-15	1.00E-14
novel_pir1193	63	0	2.15	0.001	-11.07012094	Down	5.87E-15	1.00E-14
novel_pir2143	60	0	2.04	0.001	-10.99435344	Down	2.82E-14	4.76E-14
novel_pir1841	60	0	2.04	0.001	-10.99435344	Down	2.82E-14	4.76E-14
novel_pir453	60	0	2.04	0.001	-10.99435344	Down	2.82E-14	4.75E-14
novel_pir2196	60	0	2.04	0.001	-10.99435344	Down	2.82E-14	4.75E-14
novel_pir1776	59	0	2.01	0.001	-10.97297979	Down	4.75E-14	7.92E-14
novel_pir760	58	0	1.98	0.001	-10.95128471	Down	8.02E-14	1.33E-13
novel_pir1300	58	0	1.98	0.001	-10.95128471	Down	8.02E-14	1.33E-13
novel_pir476	58	0	1.98	0.001	-10.95128471	Down	8.02E-14	1.33E-13
novel_pir1464	58	0	1.98	0.001	-10.95128471	Down	8.02E-14	1.33E-13
novel_pir867	58	0	1.98	0.001	-10.95128471	Down	8.02E-14	1.33E-13
novel_pir1839	58	0	1.98	0.001	-10.95128471	Down	8.02E-14	1.33E-13
novel_pir251	57	0	1.94	0.001	-10.92184094	Down	1.35E-13	2.23E-13
novel_pir2124	57	0	1.94	0.001	-10.92184094	Down	1.35E-13	2.23E-13
novel_pir1563	56	0	1.91	0.001	-10.89935692	Down	2.28E-13	3.75E-13
novel_pir1198	56	0	1.91	0.001	-10.89935692	Down	2.28E-13	3.75E-13
novel_pir692	56	0	1.91	0.001	-10.89935692	Down	2.28E-13	3.75E-13
novel_pir1197	56	0	1.91	0.001	-10.89935692	Down	2.28E-13	3.74E-13
novel_pir885	55	0	1.87	0.001	-10.86882255	Down	3.85E-13	6.27E-13
novel_pir80	55	0	1.87	0.001	-10.86882255	Down	3.85E-13	6.27E-13
novel_pir2151	55	0	1.87	0.001	-10.86882255	Down	3.85E-13	6.26E-13
novel_pir530	54	0	1.84	0.001	-10.84549005	Down	6.49E-13	1.06E-12
novel_pir325	54	0	1.84	0.001	-10.84549005	Down	6.49E-13	1.05E-12
novel_pir2069	54	0	1.84	0.001	-10.84549005	Down	6.49E-13	1.05E-12
novel_pir699	54	0	1.84	0.001	-10.84549005	Down	6.49E-13	1.05E-12
novel_pir565	54	0	1.84	0.001	-10.84549005	Down	6.49E-13	1.05E-12
novel_pir2063	53	0	1.8	0.001	-10.81378119	Down	1.10E-12	1.76E-12
novel_pir1000	53	0	1.8	0.001	-10.81378119	Down	1.10E-12	1.76E-12
novel_pir716	52	0	1.77	0.001	-10.78953364	Down	1.85E-12	2.95E-12
novel_pir1353	52	0	1.77	0.001	-10.78953364	Down	1.85E-12	2.95E-12

novel_pir2065	52	0	1.77	0.001	-10.78953364	Down	1.85E-12	2.95E-12
mmu_piR_000634	52	0	1.77	0.001	-10.78953364	Down	1.85E-12	2.95E-12
novel_pir2459	52	0	1.77	0.001	-10.78953364	Down	1.85E-12	2.95E-12
novel_pir2030	52	0	1.77	0.001	-10.78953364	Down	1.85E-12	2.94E-12
novel_pir2326	51	0	1.74	0.001	-10.76487159	Down	3.12E-12	4.96E-12
novel_pir499	50	0	1.7	0.001	-10.73131903	Down	5.26E-12	8.27E-12
novel_pir977	50	0	1.7	0.001	-10.73131903	Down	5.26E-12	8.26E-12
novel_pir739	49	0	1.67	0.001	-10.70563239	Down	8.87E-12	1.39E-11
novel_pir1699	49	0	1.67	0.001	-10.70563239	Down	8.87E-12	1.39E-11
novel_pir267	49	0	1.67	0.001	-10.70563239	Down	8.87E-12	1.39E-11
novel_pir2208	49	0	1.67	0.001	-10.70563239	Down	8.87E-12	1.39E-11
novel_pir471	48	0	1.63	0.001	-10.67065625	Down	1.50E-11	2.32E-11
novel_pir965	48	0	1.63	0.001	-10.67065625	Down	1.50E-11	2.32E-11
novel_pir2152	47	0	1.6	0.001	-10.64385619	Down	2.52E-11	3.88E-11
novel_pir1087	47	0	1.6	0.001	-10.64385619	Down	2.52E-11	3.88E-11
novel_pir2328	47	0	1.6	0.001	-10.64385619	Down	2.52E-11	3.87E-11
novel_pir1606	46	0	1.57	0.001	-10.61654884	Down	4.26E-11	6.53E-11
novel_pir1929	46	0	1.57	0.001	-10.61654884	Down	4.26E-11	6.53E-11
novel_pir1165	46	0	1.57	0.001	-10.61654884	Down	4.26E-11	6.52E-11
novel_pir2361	45	0	1.53	0.001	-10.57931594	Down	7.18E-11	1.09E-10
novel_pir1254	45	0	1.53	0.001	-10.57931594	Down	7.18E-11	1.09E-10
novel_pir1344	45	0	1.53	0.001	-10.57931594	Down	7.18E-11	1.08E-10
novel_pir1200	44	0	1.5	0.001	-10.55074679	Down	1.21E-10	1.83E-10
novel_pir1367	44	0	1.5	0.001	-10.55074679	Down	1.21E-10	1.83E-10
novel_pir2177	44	0	1.5	0.001	-10.55074679	Down	1.21E-10	1.82E-10
novel_pir2163	44	0	1.5	0.001	-10.55074679	Down	1.21E-10	1.82E-10
novel_pir94	44	0	1.5	0.001	-10.55074679	Down	1.21E-10	1.82E-10
novel_pir1570	44	0	1.5	0.001	-10.55074679	Down	1.21E-10	1.82E-10
novel_pir1396	44	0	1.5	0.001	-10.55074679	Down	1.21E-10	1.82E-10
novel_pir134	43	0	1.46	0.001	-10.51175265	Down	2.04E-10	3.03E-10
novel_pir2472	43	0	1.46	0.001	-10.51175265	Down	2.04E-10	3.03E-10
novel_pir385	43	0	1.46	0.001	-10.51175265	Down	2.04E-10	3.03E-10
novel_pir1781	43	0	1.46	0.001	-10.51175265	Down	2.04E-10	3.02E-10
novel_pir1713	43	0	1.46	0.001	-10.51175265	Down	2.04E-10	3.02E-10
novel_pir1687	42	0	1.43	0.001	-10.48179943	Down	3.45E-10	5.09E-10
novel_pir2294	42	0	1.43	0.001	-10.48179943	Down	3.45E-10	5.09E-10

novel_pir592	42	0	1.43	0.001	-10.48179943	Down	3.45E-10	5.09E-10
novel_pir1168	42	0	1.43	0.001	-10.48179943	Down	3.45E-10	5.08E-10
novel_pir1972	42	0	1.43	0.001	-10.48179943	Down	3.45E-10	5.08E-10
novel_pir1703	41	0	1.4	0.001	-10.45121111	Down	5.81E-10	8.45E-10
novel_pir847	41	0	1.4	0.001	-10.45121111	Down	5.81E-10	8.44E-10
novel_pir1760	41	0	1.4	0.001	-10.45121111	Down	5.81E-10	8.44E-10
novel_pir1069	41	0	1.4	0.001	-10.45121111	Down	5.81E-10	8.43E-10
novel_pir1280	41	0	1.4	0.001	-10.45121111	Down	5.81E-10	8.42E-10
novel_pir813	40	0	1.36	0.001	-10.40939094	Down	9.80E-10	1.41E-09
novel_pir2132	40	0	1.36	0.001	-10.40939094	Down	9.80E-10	1.40E-09
novel_pir923	40	0	1.36	0.001	-10.40939094	Down	9.80E-10	1.40E-09
novel_pir1613	40	0	1.36	0.001	-10.40939094	Down	9.80E-10	1.40E-09
novel_pir1672	40	0	1.36	0.001	-10.40939094	Down	9.80E-10	1.40E-09
novel_pir62	39	0	1.33	0.001	-10.37721053	Down	1.65E-09	2.36E-09
novel_pir2322	39	0	1.33	0.001	-10.37721053	Down	1.65E-09	2.36E-09
novel_pir1724	39	0	1.33	0.001	-10.37721053	Down	1.65E-09	2.36E-09
novel_pir61	39	0	1.33	0.001	-10.37721053	Down	1.65E-09	2.36E-09
novel_pir2221	39	0	1.33	0.001	-10.37721053	Down	1.65E-09	2.35E-09
novel_pir848	38	0	1.29	0.001	-10.33315535	Down	2.79E-09	3.94E-09
novel_pir2115	38	0	1.29	0.001	-10.33315535	Down	2.79E-09	3.93E-09
novel_pir829	37	0	1.26	0.001	-10.29920802	Down	4.71E-09	6.63E-09
novel_pir1988	37	0	1.26	0.001	-10.29920802	Down	4.71E-09	6.63E-09
novel_pir1253	36	0	1.23	0.001	-10.2644426	Down	7.94E-09	1.11E-08
novel_pir993	36	0	1.23	0.001	-10.2644426	Down	7.94E-09	1.11E-08
novel_pir2317	36	0	1.23	0.001	-10.2644426	Down	7.94E-09	1.11E-08
novel_pir1600	36	0	1.23	0.001	-10.2644426	Down	7.94E-09	1.11E-08
novel_pir252	35	0	1.19	0.001	-10.21674586	Down	1.34E-08	1.84E-08
novel_pir452	34	0	1.16	0.001	-10.17990909	Down	2.26E-08	3.10E-08
novel_pir309	34	0	1.16	0.001	-10.17990909	Down	2.26E-08	3.10E-08
novel_pir1652	34	0	1.16	0.001	-10.17990909	Down	2.26E-08	3.10E-08
novel_pir1819	34	0	1.16	0.001	-10.17990909	Down	2.26E-08	3.10E-08
novel_pir1082	33	0	1.12	0.001	-10.12928302	Down	3.81E-08	5.15E-08
mmu_piR_032974	33	0	1.12	0.001	-10.12928302	Down	3.81E-08	5.14E-08
novel_pir1298	33	0	1.12	0.001	-10.12928302	Down	3.81E-08	5.14E-08
novel_pir529	33	0	1.12	0.001	-10.12928302	Down	3.81E-08	5.14E-08
novel_pir934	33	0	1.12	0.001	-10.12928302	Down	3.81E-08	5.13E-08

novel_pir1512	32	0	1.09	0.001	-10.09011242	Down	6.43E-08	8.65E-08
novel_pir893	32	0	1.09	0.001	-10.09011242	Down	6.43E-08	8.64E-08
novel_pir414	31	0	1.06	0.001	-10.04984855	Down	1.08E-07	1.44E-07
novel_pir331	30	0	1.02	0.001	-9.994353437	Down	1.83E-07	2.42E-07
novel_pir1742	30	0	1.02	0.001	-9.994353437	Down	1.83E-07	2.42E-07
novel_pir1330	29	0	0.99	0.001	-9.951284715	Down	3.08E-07	4.01E-07
novel_pir2359	29	0	0.99	0.001	-9.951284715	Down	3.08E-07	4.01E-07
novel_pir2016	28	0	0.95	0.001	-9.891783703	Down	5.20E-07	6.66E-07
novel_pir976	28	0	0.95	0.001	-9.891783703	Down	5.20E-07	6.65E-07
novel_pir602	28	0	0.95	0.001	-9.891783703	Down	5.20E-07	6.65E-07
novel_pir256	28	0	0.95	0.001	-9.891783703	Down	5.20E-07	6.64E-07
novel_pir650	27	0	0.92	0.001	-9.845490051	Down	8.77E-07	1.12E-06
novel_pir272	27	0	0.92	0.001	-9.845490051	Down	8.77E-07	1.12E-06
novel_pir1891	27	0	0.92	0.001	-9.845490051	Down	8.77E-07	1.12E-06
novel_pir1249	27	0	0.92	0.001	-9.845490051	Down	8.77E-07	1.12E-06
novel_pir623	26	0	0.89	0.001	-9.797661526	Down	1.48E-06	1.86E-06
novel_pir626	26	0	0.89	0.001	-9.797661526	Down	1.48E-06	1.86E-06
novel_pir857	26	0	0.89	0.001	-9.797661526	Down	1.48E-06	1.86E-06
novel_pir1849	26	0	0.89	0.001	-9.797661526	Down	1.48E-06	1.85E-06
novel_pir869	26	0	0.89	0.001	-9.797661526	Down	1.48E-06	1.85E-06
novel_pir1305	26	0	0.89	0.001	-9.797661526	Down	1.48E-06	1.85E-06
novel_pir461	26	0	0.89	0.001	-9.797661526	Down	1.48E-06	1.85E-06
novel_pir1527	26	0	0.89	0.001	-9.797661526	Down	1.48E-06	1.85E-06
novel_pir1663	26	0	0.89	0.001	-9.797661526	Down	1.48E-06	1.85E-06
novel_pir691	25	0	0.85	0.001	-9.731319031	Down	2.50E-06	3.12E-06
novel_pir2070	25	0	0.85	0.001	-9.731319031	Down	2.50E-06	3.11E-06
novel_pir383	25	0	0.85	0.001	-9.731319031	Down	2.50E-06	3.11E-06
novel_pir188	25	0	0.85	0.001	-9.731319031	Down	2.50E-06	3.11E-06
novel_pir2073	24	0	0.82	0.001	-9.6794801	Down	4.21E-06	5.16E-06
novel_pir384	24	0	0.82	0.001	-9.6794801	Down	4.21E-06	5.15E-06
novel_pir1617	24	0	0.82	0.001	-9.6794801	Down	4.21E-06	5.15E-06
novel_pir1580	24	0	0.82	0.001	-9.6794801	Down	4.21E-06	5.15E-06
novel_pir865	24	0	0.82	0.001	-9.6794801	Down	4.21E-06	5.14E-06
novel_pir1778	24	0	0.82	0.001	-9.6794801	Down	4.21E-06	5.14E-06
novel_pir2323	24	0	0.82	0.001	-9.6794801	Down	4.21E-06	5.14E-06
novel_pir50	24	0	0.82	0.001	-9.6794801	Down	4.21E-06	5.13E-06

novel_pir1273	23	0	0.78	0.001	-9.607330314	Down	7.10E-06	8.44E-06
novel_pir259	23	0	0.78	0.001	-9.607330314	Down	7.10E-06	8.43E-06
novel_pir1250	23	0	0.78	0.001	-9.607330314	Down	7.10E-06	8.42E-06
novel_pir1905	23	0	0.78	0.001	-9.607330314	Down	7.10E-06	8.42E-06
novel_pir1244	22	0	0.75	0.001	-9.550746785	Down	1.20E-05	1.42E-05
novel_pir2394	22	0	0.75	0.001	-9.550746785	Down	1.20E-05	1.42E-05
novel_pir1746	22	0	0.75	0.001	-9.550746785	Down	1.20E-05	1.42E-05
novel_pir1820	22	0	0.75	0.001	-9.550746785	Down	1.20E-05	1.42E-05
novel_pir939	22	0	0.75	0.001	-9.550746785	Down	1.20E-05	1.42E-05
novel_pir200	22	0	0.75	0.001	-9.550746785	Down	1.20E-05	1.41E-05
novel_pir2384	22	0	0.75	0.001	-9.550746785	Down	1.20E-05	1.41E-05
novel_pir1106	21	0	0.72	0.001	-9.491853096	Down	2.02E-05	2.33E-05
novel_pir326	21	0	0.72	0.001	-9.491853096	Down	2.02E-05	2.33E-05
novel_pir320	21	0	0.72	0.001	-9.491853096	Down	2.02E-05	2.33E-05
novel_pir753	21	0	0.72	0.001	-9.491853096	Down	2.02E-05	2.32E-05
novel_pir2311	21	0	0.72	0.001	-9.491853096	Down	2.02E-05	2.32E-05
novel_pir2056	20	0	0.68	0.001	-9.409390936	Down	3.41E-05	3.92E-05
novel_pir2362	20	0	0.68	0.001	-9.409390936	Down	3.41E-05	3.91E-05
novel_pir908	20	0	0.68	0.001	-9.409390936	Down	3.41E-05	3.91E-05
novel_pir714	19	0	0.65	0.001	-9.344295908	Down	5.75E-05	6.41E-05
novel_pir2339	19	0	0.65	0.001	-9.344295908	Down	5.75E-05	6.40E-05
novel_pir1003	19	0	0.65	0.001	-9.344295908	Down	5.75E-05	6.40E-05
novel_pir45	19	0	0.65	0.001	-9.344295908	Down	5.75E-05	6.40E-05
novel_pir1757	19	0	0.65	0.001	-9.344295908	Down	5.75E-05	6.39E-05
novel_pir1134	19	0	0.65	0.001	-9.344295908	Down	5.75E-05	6.39E-05
novel_pir410	19	0	0.65	0.001	-9.344295908	Down	5.75E-05	6.39E-05
novel_pir1401	19	0	0.65	0.001	-9.344295908	Down	5.75E-05	6.38E-05
novel_pir2149	18	0	0.61	0.001	-9.252665432	Down	9.70E-05	0.000107525
novel_pir1836	18	0	0.61	0.001	-9.252665432	Down	9.70E-05	0.000107463
novel_pir2215	18	0	0.61	0.001	-9.252665432	Down	9.70E-05	0.000107401
novel_pir652	17	0	0.58	0.001	-9.17990909	Down	0.00016363	0.000173987
novel_pir2189	17	0	0.58	0.001	-9.17990909	Down	0.00016363	0.000173891
novel_pir255	17	0	0.58	0.001	-9.17990909	Down	0.00016363	0.000173795
novel_pir1409	17	0	0.58	0.001	-9.17990909	Down	0.00016363	0.0001737
novel_pir1614	17	0	0.58	0.001	-9.17990909	Down	0.00016363	0.000173605
novel_pir302	17	0	0.58	0.001	-9.17990909	Down	0.00016363	0.000173509

novel_pir873	17	0	0.58	0.001	-9.17990909	Down	0.00016363	0.000173414
novel_pir1356	17	0	0.58	0.001	-9.17990909	Down	0.00016363	0.000173319
novel_pir1576	17	0	0.58	0.001	-9.17990909	Down	0.00016363	0.000173224
novel_pir647	17	0	0.58	0.001	-9.17990909	Down	0.00016363	0.000173129
novel_pir2234	17	0	0.58	0.001	-9.17990909	Down	0.00016363	0.000173034
novel_pir44	16	0	0.54	0.001	-9.076815597	Down	0.000276012	0.000291556
novel_pir454	16	0	0.54	0.001	-9.076815597	Down	0.000276012	0.000291396
novel_pir1743	16	0	0.54	0.001	-9.076815597	Down	0.000276012	0.000291237
novel_pir1176	16	0	0.54	0.001	-9.076815597	Down	0.000276012	0.000291078
novel_pir1054	16	0	0.54	0.001	-9.076815597	Down	0.000276012	0.000290919
novel_pir1685	16	0	0.54	0.001	-9.076815597	Down	0.000276012	0.000290761
novel_pir1756	16	0	0.54	0.001	-9.076815597	Down	0.000276012	0.000290602
novel_pir1779	16	0	0.54	0.001	-9.076815597	Down	0.000276012	0.000290444
novel_pir2064	16	0	0.54	0.001	-9.076815597	Down	0.000276012	0.000290286
novel_pir812	16	0	0.54	0.001	-9.076815597	Down	0.000276012	0.000290128
novel_pir594	15	0	0.51	0.001	-8.994353437	Down	0.000465578	0.000488857
novel_pir1104	15	0	0.51	0.001	-8.994353437	Down	0.000465578	0.000488591
novel_pir1428	15	0	0.51	0.001	-8.994353437	Down	0.000465578	0.000488326
novel_pir1471	15	0	0.51	0.001	-8.994353437	Down	0.000465578	0.000488061
novel_pir359	14	0	0.48	0.001	-8.906890596	Down	0.00078534	0.000821927
novel_pir2358	14	0	0.48	0.001	-8.906890596	Down	0.00078534	0.000821482
novel_pir1350	14	0	0.48	0.001	-8.906890596	Down	0.00078534	0.000821037
novel_pir2182	14	0	0.48	0.001	-8.906890596	Down	0.00078534	0.000820593
novel_pir1892	14	0	0.48	0.001	-8.906890596	Down	0.00078534	0.00082015
novel_pir1053	14	0	0.48	0.001	-8.906890596	Down	0.00078534	0.000819707
novel_pir932	14	0	0.48	0.001	-8.906890596	Down	0.00078534	0.000819264
mmu_piR_024749	1110	2	37.8	0.1	-8.562242424	Down	1.11E-247	8.56E-246
novel_pir231	3004	10	102.3	0.5	-7.676662335	Down	0	0
novel_pir399	6067	29	206.61	1.44	-7.164697461	Down	0	0
novel_pir807	1826	13	62.18	0.64	-6.602234901	Down	0	0
mmu_piR_010309	1894	16	64.5	0.79	-6.351302697	Down	0	0
novel_pir613	1945	20	66.24	0.99	-6.064130337	Down	0	0
mmu_piR_023189	3681	42	125.36	2.08	-5.913349746	Down	0	0
novel_pir1720	949	11	32.32	0.55	-5.876851769	Down	3.08E-195	1.92E-193
novel_pir398	2111	33	71.89	1.64	-5.454023384	Down	0	0
novel_pir1272	2888	47	98.35	2.33	-5.399523193	Down	0	0

novel_pir2416	697	12	23.74	0.6	-5.306213624	Down	4.29E-138	1.48E-136
novel_pir984	3461	62	117.87	3.07	-5.262814108	Down	0	0
novel_pir133	1144	22	38.96	1.09	-5.159593637	Down	1.19E-222	8.24E-221
novel_pir274	1281	26	43.62	1.29	-5.079546799	Down	2.45E-247	1.82E-245
mmu_piR_002962	388	9	13.21	0.45	-4.875561655	Down	1.88E-74	1.14E-73
novel_pir287	574	14	19.55	0.69	-4.824428435	Down	1.16E-108	2.58E-107
novel_pir806	1617	41	55.07	2.03	-4.761714976	Down	1.58E-301	1.39E-299
mmu_piR_017405	802	21	27.31	1.04	-4.714773881	Down	1.60E-149	6.45E-148
novel_pir155	1175	32	40.01	1.59	-4.653261958	Down	6.75E-217	4.49E-215
novel_pir717	2955	84	100.63	4.17	-4.592869274	Down	0	0
mmu_piR_013763	303	9	10.32	0.45	-4.519374159	Down	4.27E-56	1.56E-55
novel_pir1609	333	11	11.34	0.55	-4.365845211	Down	2.72E-60	1.11E-59
novel_pir945	377	13	12.84	0.64	-4.326429487	Down	1.72E-67	8.58E-67
novel_pir149	370	14	12.6	0.69	-4.190683562	Down	5.93E-65	2.73E-64
novel_pir2420	453	18	15.43	0.89	-4.115788916	Down	2.50E-78	1.75E-77
novel_pir334	294	13	10.01	0.64	-3.967226259	Down	5.22E-50	1.64E-49
novel_pir1601	569	25	19.38	1.24	-3.966156545	Down	1.25E-95	1.82E-94
novel_pir1914	3286	149	111.91	7.39	-3.920620783	Down	0	0
novel_pir145	1029	47	35.04	2.33	-3.910600915	Down	5.92E-170	3.01E-168
novel_pir2033	879	44	29.93	2.18	-3.779192239	Down	9.11E-142	3.39E-140
novel_pir2385	735	37	25.03	1.83	-3.773742737	Down	1.32E-118	3.80E-117
novel_pir719	565	31	19.24	1.54	-3.643106543	Down	3.29E-89	3.69E-88
mmu_piR_031448	65076	3863	2216.18	191.55	-3.532282132	Down	0	0
novel_pir277	357	22	12.16	1.09	-3.479743189	Down	7.99E-55	2.84E-54
novel_pir147	636	40	21.66	1.98	-3.451460908	Down	7.84E-96	1.15E-94
mmu_piR_028252	323	21	11	1.04	-3.40284809	Down	7.87E-49	2.39E-48
mmu_piR_016069	69	5	2.35	0.25	-3.232660757	Down	5.47E-11	8.38E-11
novel_pir142	1140	87	38.82	4.31	-3.171040344	Down	2.52E-158	1.16E-156
novel_pir2122	3899	315	132.78	15.62	-3.087571498	Down	0	0
novel_pir194	684	60	23.29	2.98	-2.966326404	Down	5.70E-90	6.59E-89
novel_pir2425	347	31	11.82	1.54	-2.940227779	Down	5.25E-46	1.53E-45
novel_pir928	124	11	4.22	0.55	-2.939739475	Down	2.35E-17	4.25E-17
novel_pir459	219	21	7.46	1.04	-2.842592102	Down	1.38E-28	2.99E-28
novel_pir2422	313	31	10.66	1.54	-2.791205182	Down	1.34E-39	3.46E-39
novel_pir2035	149	15	5.07	0.74	-2.776388571	Down	2.07E-19	3.86E-19
novel_pir1591	446	48	15.19	2.38	-2.674088391	Down	2.34E-53	8.03E-53

novel_pir608	295	33	10.05	1.64	-2.615427781	Down	4.97E-35	1.21E-34
novel_pir1696	572	67	19.48	3.32	-2.552738531	Down	1.06E-64	4.83E-64
novel_pir2468	459	57	15.63	2.83	-2.46544382	Down	2.30E-50	7.25E-50
mmu_piR_000691	318	41	10.83	2.03	-2.41548161	Down	1.63E-34	3.95E-34
novel_pir296	569	84	19.38	4.17	-2.216449282	Down	5.85E-55	2.10E-54
mmu_piR_020120	633	94	21.56	4.66	-2.209955318	Down	1.10E-60	4.55E-60
novel_pir153	66	10	2.25	0.5	-2.169925001	Down	2.12E-07	2.77E-07
novel_pir718	781	138	26.6	6.84	-1.959358016	Down	2.97E-64	1.33E-63
novel_pir2040	539	105	18.36	5.21	-1.817210781	Down	8.56E-41	2.26E-40
novel_pir1452	688	137	23.43	6.79	-1.786873475	Down	1.87E-50	5.93E-50
mmu_piR_025576	7161	1453	243.87	72.05	-1.759041958	Down	0	0
novel_pir290	88	20	3	0.99	-1.59946207	Down	1.02E-06	1.30E-06
novel_pir336	452	107	15.39	5.31	-1.535209466	Down	1.34E-27	2.85E-27
novel_pir2246	118	28	4.02	1.39	-1.532110618	Down	3.44E-08	4.65E-08
novel_pir148	67	17	2.28	0.84	-1.440572591	Down	8.23E-05	9.13E-05
