# Supplementary material for: Pathogenic Effects and Potential Regulatory Mechanisms of Tea Polyphenols on Obesity
Source: Biomed Res Int. 2019 Jun 11;2019:2579734. doi: 10.1155/2019/2579734 (PMC6595166; doi:10.1155/2019/2579734)
Supplement: Supplementary 4 — Supplementary Material S4. Differential expressed piRNAs in the control group vs. high-dose group. [file 2579734.f4.pdf]

miRNA id	Count (DUI4)	Count (XIA01)	TPM (DUI4)	TPM (XIA01)	log2 Ratio (XIA01/DUI4)	Up-Down-Regulation (XIA01/DUI4)	P-value	FDR
novel_pir2005	0	960	0.001	24.03	14.55254903	Up	2.09E-230	8.03E-229
novel_pir1997	0	456	0.001	11.41	13.47801117	Up	8.60E-110	4.36E-109
novel_pir2418	0	362	0.001	9.06	13.14529533	Up	2.69E-87	9.49E-87
novel_pir2004	0	237	0.001	5.93	12.53381639	Up	2.21E-57	5.73E-57
novel_pir2419	0	167	0.001	4.18	12.02928723	Up	1.25E-40	2.80E-40
novel_pir1451	0	148	0.001	3.7	11.85330956	Up	4.40E-36	9.58E-36
novel_pir610	0	144	0.001	3.6	11.81378119	Up	3.99E-35	8.62E-35
novel_pir1456	0	126	0.001	3.15	11.62113611	Up	8.09E-31	1.70E-30
novel_pir1653	0	118	0.001	2.95	11.52649924	Up	6.65E-29	1.37E-28
novel_pir1608	0	116	0.001	2.9	11.50183718	Up	2.00E-28	4.13E-28
novel_pir2006	0	112	0.001	2.8	11.45121111	Up	1.81E-27	3.71E-27
mmu_piR_002643	0	108	0.001	2.7	11.39874369	Up	1.64E-26	3.32E-26
novel_pir2414	0	105	0.001	2.63	11.36084708	Up	8.59E-26	1.73E-25
novel_pir117	0	96	0.001	2.4	11.22881869	Up	1.22E-23	2.42E-23
novel_pir2415	0	96	0.001	2.4	11.22881869	Up	1.22E-23	2.42E-23
novel_pir2000	0	89	0.001	2.23	11.12282799	Up	5.79E-22	1.13E-21
novel_pir169	0	87	0.001	2.18	11.09011242	Up	1.74E-21	3.38E-21
novel_pir2003	0	84	0.001	2.1	11.03617361	Up	9.11E-21	1.74E-20
novel_pir854	0	83	0.001	2.08	11.02236781	Up	1.58E-20	3.01E-20
mmu_piR_002435	0	78	0.001	1.95	10.92925841	Up	2.48E-19	4.69E-19
novel_pir2436	0	76	0.001	1.9	10.8917837	Up	7.48E-19	1.40E-18
novel_pir2036	0	75	0.001	1.88	10.87651695	Up	1.30E-18	2.43E-18
novel_pir172	0	72	0.001	1.8	10.81378119	Up	6.78E-18	1.26E-17
novel_pir2296	0	71	0.001	1.78	10.79766153	Up	1.18E-17	2.18E-17
novel_pir1455	0	69	0.001	1.73	10.75655632	Up	3.54E-17	6.50E-17
mmu_piR_028975	0	68	0.001	1.7	10.73131903	Up	6.14E-17	1.13E-16
novel_pir2007	0	66	0.001	1.65	10.68825031	Up	1.85E-16	3.35E-16
novel_pir2424	0	62	0.001	1.55	10.5980525	Up	1.68E-15	3.01E-15
novel_pir2413	0	60	0.001	1.5	10.55074679	Up	5.05E-15	9.00E-15
novel_pir1668	0	59	0.001	1.48	10.53138146	Up	8.75E-15	1.56E-14
novel_pir514	0	58	0.001	1.45	10.50183718	Up	1.52E-14	2.69E-14
novel_pir1604	0	55	0.001	1.38	10.43045255	Up	7.93E-14	1.40E-13

novel_pir2412	0	54	0.001	1.35	10.39874369	Up	1.38E-13	2.42E-13
novel_pir2417	0	53	0.001	1.33	10.37721053	Up	2.39E-13	4.18E-13
novel_pir417	0	52	0.001	1.3	10.34429591	Up	4.14E-13	7.25E-13
novel_pir1557	0	52	0.001	1.3	10.34429591	Up	4.14E-13	7.24E-13
novel_pir2039	0	51	0.001	1.28	10.32192809	Up	7.19E-13	1.25E-12
novel_pir1766	0	51	0.001	1.28	10.32192809	Up	7.19E-13	1.25E-12
novel_pir2001	0	51	0.001	1.28	10.32192809	Up	7.19E-13	1.25E-12
mmu_piR_000362	0	47	0.001	1.18	10.20457114	Up	6.52E-12	1.12E-11
novel_pir2409	0	47	0.001	1.18	10.20457114	Up	6.52E-12	1.12E-11
novel_pir295	0	47	0.001	1.18	10.20457114	Up	6.52E-12	1.12E-11
novel_pir144	0	45	0.001	1.13	10.14210706	Up	1.96E-11	3.37E-11
novel_pir2002	0	45	0.001	1.13	10.14210706	Up	1.96E-11	3.37E-11
novel_pir2387	0	45	0.001	1.13	10.14210706	Up	1.96E-11	3.36E-11
novel_pir1446	0	45	0.001	1.13	10.14210706	Up	1.96E-11	3.36E-11
novel_pir2410	0	44	0.001	1.1	10.10328781	Up	3.40E-11	5.80E-11
novel_pir1996	0	44	0.001	1.1	10.10328781	Up	3.40E-11	5.80E-11
mmu_piR_038428	0	43	0.001	1.08	10.0768156	Up	5.91E-11	1.00E-10
novel_pir1998	0	43	0.001	1.08	10.0768156	Up	5.91E-11	1.00E-10
mmu_piR_000536	0	42	0.001	1.05	10.03617361	Up	1.02E-10	1.73E-10
novel_pir850	0	42	0.001	1.05	10.03617361	Up	1.02E-10	1.73E-10
novel_pir531	0	42	0.001	1.05	10.03617361	Up	1.02E-10	1.73E-10
novel_pir570	0	41	0.001	1.03	10.00842862	Up	1.78E-10	2.97E-10
novel_pir1999	0	41	0.001	1.03	10.00842862	Up	1.78E-10	2.97E-10
novel_pir2403	0	39	0.001	0.98	9.936637939	Up	5.35E-10	8.91E-10
novel_pir2398	0	39	0.001	0.98	9.936637939	Up	5.35E-10	8.90E-10
novel_pir1448	0	39	0.001	0.98	9.936637939	Up	5.35E-10	8.89E-10
novel_pir1702	0	39	0.001	0.98	9.936637939	Up	5.35E-10	8.88E-10
novel_pir2421	0	39	0.001	0.98	9.936637939	Up	5.35E-10	8.87E-10
novel_pir116	0	36	0.001	0.9	9.813781191	Up	2.80E-09	4.59E-09
mmu_piR_011141	0	36	0.001	0.9	9.813781191	Up	2.80E-09	4.58E-09
novel_pir2091	0	36	0.001	0.9	9.813781191	Up	2.80E-09	4.58E-09
novel_pir43	0	35	0.001	0.88	9.781359714	Up	4.85E-09	7.94E-09
novel_pir2404	0	35	0.001	0.88	9.781359714	Up	4.85E-09	7.93E-09
novel_pir2406	0	34	0.001	0.85	9.731319031	Up	8.41E-09	1.37E-08
novel_pir1539	0	34	0.001	0.85	9.731319031	Up	8.41E-09	1.37E-08
novel_pir222	0	33	0.001	0.83	9.696967526	Up	1.46E-08	2.35E-08

novel_pir170	0	33	0.001	0.83	9.696967526	Up	1.46E-08	2.35E-08
novel_pir113	0	33	0.001	0.83	9.696967526	Up	1.46E-08	2.35E-08
novel_pir299	0	33	0.001	0.83	9.696967526	Up	1.46E-08	2.35E-08
novel_pir1050	0	33	0.001	0.83	9.696967526	Up	1.46E-08	2.35E-08
novel_pir894	0	32	0.001	0.8	9.64385619	Up	2.53E-08	4.07E-08
novel_pir1588	0	32	0.001	0.8	9.64385619	Up	2.53E-08	4.06E-08
novel_pir505	0	32	0.001	0.8	9.64385619	Up	2.53E-08	4.06E-08
novel_pir864	0	31	0.001	0.78	9.607330314	Up	4.40E-08	7.02E-08
novel_pir2011	0	31	0.001	0.78	9.607330314	Up	4.40E-08	7.02E-08
novel_pir2109	0	30	0.001	0.75	9.550746785	Up	7.63E-08	1.21E-07
novel_pir294	0	30	0.001	0.75	9.550746785	Up	7.63E-08	1.21E-07
novel_pir2466	0	29	0.001	0.73	9.511752654	Up	1.32E-07	2.09E-07
mmu_piR_004567	0	29	0.001	0.73	9.511752654	Up	1.32E-07	2.09E-07
novel_pir1453	0	29	0.001	0.73	9.511752654	Up	1.32E-07	2.09E-07
novel_pir1531	0	29	0.001	0.73	9.511752654	Up	1.32E-07	2.09E-07
novel_pir217	0	29	0.001	0.73	9.511752654	Up	1.32E-07	2.09E-07
novel_pir2408	0	28	0.001	0.7	9.451211112	Up	2.30E-07	3.61E-07
novel_pir1584	0	28	0.001	0.7	9.451211112	Up	2.30E-07	3.60E-07
novel_pir2017	0	27	0.001	0.68	9.409390936	Up	3.98E-07	6.19E-07
novel_pir157	0	27	0.001	0.68	9.409390936	Up	3.98E-07	6.18E-07
novel_pir1214	0	27	0.001	0.68	9.409390936	Up	3.98E-07	6.18E-07
novel_pir1631	0	27	0.001	0.68	9.409390936	Up	3.98E-07	6.17E-07
novel_pir642	0	26	0.001	0.65	9.344295908	Up	6.91E-07	1.07E-06
novel_pir2301	0	26	0.001	0.65	9.344295908	Up	6.91E-07	1.07E-06
novel_pir2155	0	26	0.001	0.65	9.344295908	Up	6.91E-07	1.07E-06
novel_pir1123	0	26	0.001	0.65	9.344295908	Up	6.91E-07	1.07E-06
novel_pir1030	0	26	0.001	0.65	9.344295908	Up	6.91E-07	1.07E-06
novel_pir382	0	25	0.001	0.63	9.299208018	Up	1.20E-06	1.83E-06
novel_pir609	0	25	0.001	0.63	9.299208018	Up	1.20E-06	1.83E-06
novel_pir434	0	25	0.001	0.63	9.299208018	Up	1.20E-06	1.83E-06
mmu_piR_024221	0	25	0.001	0.63	9.299208018	Up	1.20E-06	1.83E-06
novel_pir1506	0	25	0.001	0.63	9.299208018	Up	1.20E-06	1.83E-06
novel_pir898	0	25	0.001	0.63	9.299208018	Up	1.20E-06	1.82E-06
novel_pir161	0	25	0.001	0.63	9.299208018	Up	1.20E-06	1.82E-06
novel_pir733	0	25	0.001	0.63	9.299208018	Up	1.20E-06	1.82E-06
novel_pir1486	0	24	0.001	0.6	9.22881869	Up	2.08E-06	3.16E-06

novel_pir646	0	24	0.001	0.6	9.22881869	Up	2.08E-06	3.16E-06
novel_pir2351	0	24	0.001	0.6	9.22881869	Up	2.08E-06	3.15E-06
novel_pir40	0	24	0.001	0.6	9.22881869	Up	2.08E-06	3.15E-06
novel_pir1040	0	24	0.001	0.6	9.22881869	Up	2.08E-06	3.15E-06
novel_pir1574	0	24	0.001	0.6	9.22881869	Up	2.08E-06	3.15E-06
novel_pir936	0	24	0.001	0.6	9.22881869	Up	2.08E-06	3.14E-06
novel_pir2202	0	24	0.001	0.6	9.22881869	Up	2.08E-06	3.14E-06
novel_pir1371	0	24	0.001	0.6	9.22881869	Up	2.08E-06	3.14E-06
novel_pir1940	0	24	0.001	0.6	9.22881869	Up	2.08E-06	3.14E-06
novel_pir379	0	24	0.001	0.6	9.22881869	Up	2.08E-06	3.13E-06
novel_pir1149	0	23	0.001	0.58	9.17990909	Up	3.61E-06	5.41E-06
novel_pir305	0	23	0.001	0.58	9.17990909	Up	3.61E-06	5.40E-06
novel_pir1765	0	23	0.001	0.58	9.17990909	Up	3.61E-06	5.40E-06
novel_pir568	0	23	0.001	0.58	9.17990909	Up	3.61E-06	5.40E-06
novel_pir441	0	23	0.001	0.58	9.17990909	Up	3.61E-06	5.39E-06
novel_pir176	0	23	0.001	0.58	9.17990909	Up	3.61E-06	5.39E-06
mmu_piR_012500	0	23	0.001	0.58	9.17990909	Up	3.61E-06	5.38E-06
novel_pir1645	0	23	0.001	0.58	9.17990909	Up	3.61E-06	5.38E-06
novel_pir306	0	23	0.001	0.58	9.17990909	Up	3.61E-06	5.37E-06
novel_pir825	0	23	0.001	0.58	9.17990909	Up	3.61E-06	5.37E-06
novel_pir1188	0	23	0.001	0.58	9.17990909	Up	3.61E-06	5.37E-06
novel_pir1457	0	22	0.001	0.55	9.103287808	Up	6.26E-06	9.25E-06
novel_pir1449	0	22	0.001	0.55	9.103287808	Up	6.26E-06	9.24E-06
novel_pir1712	0	22	0.001	0.55	9.103287808	Up	6.26E-06	9.23E-06
mmu_piR_000802	0	22	0.001	0.55	9.103287808	Up	6.26E-06	9.22E-06
novel_pir1297	0	22	0.001	0.55	9.103287808	Up	6.26E-06	9.22E-06
novel_pir2133	0	22	0.001	0.55	9.103287808	Up	6.26E-06	9.21E-06
novel_pir339	0	22	0.001	0.55	9.103287808	Up	6.26E-06	9.20E-06
novel_pir223	0	22	0.001	0.55	9.103287808	Up	6.26E-06	9.19E-06
novel_pir2324	0	22	0.001	0.55	9.103287808	Up	6.26E-06	9.19E-06
novel_pir2295	0	22	0.001	0.55	9.103287808	Up	6.26E-06	9.18E-06
mmu_piR_028055	0	21	0.001	0.53	9.049848549	Up	1.09E-05	1.59E-05
novel_pir1271	0	21	0.001	0.53	9.049848549	Up	1.09E-05	1.59E-05
novel_pir1630	0	21	0.001	0.53	9.049848549	Up	1.09E-05	1.59E-05
novel_pir20	0	21	0.001	0.53	9.049848549	Up	1.09E-05	1.59E-05
novel_pir1640	0	20	0.001	0.5	8.965784285	Up	1.89E-05	2.73E-05

novel_pir2130	0	20	0.001	0.5	8.965784285	Up	1.89E-05	2.73E-05
novel_pir631	0	20	0.001	0.5	8.965784285	Up	1.89E-05	2.73E-05
novel_pir430	0	20	0.001	0.5	8.965784285	Up	1.89E-05	2.73E-05
novel_pir298	0	20	0.001	0.5	8.965784285	Up	1.89E-05	2.72E-05
novel_pir2042	0	20	0.001	0.5	8.965784285	Up	1.89E-05	2.72E-05
novel_pir1358	0	20	0.001	0.5	8.965784285	Up	1.89E-05	2.72E-05
novel_pir146	0	20	0.001	0.5	8.965784285	Up	1.89E-05	2.72E-05
novel_pir440	0	20	0.001	0.5	8.965784285	Up	1.89E-05	2.72E-05
novel_pir1184	0	20	0.001	0.5	8.965784285	Up	1.89E-05	2.71E-05
novel_pir558	0	19	0.001	0.48	8.906890596	Up	3.27E-05	4.66E-05
novel_pir182	0	19	0.001	0.48	8.906890596	Up	3.27E-05	4.66E-05
novel_pir625	0	19	0.001	0.48	8.906890596	Up	3.27E-05	4.66E-05
novel_pir1378	0	19	0.001	0.48	8.906890596	Up	3.27E-05	4.65E-05
novel_pir701	0	19	0.001	0.48	8.906890596	Up	3.27E-05	4.65E-05
novel_pir209	0	19	0.001	0.48	8.906890596	Up	3.27E-05	4.65E-05
novel_pir1031	0	19	0.001	0.48	8.906890596	Up	3.27E-05	4.64E-05
novel_pir1939	0	19	0.001	0.48	8.906890596	Up	3.27E-05	4.64E-05
novel_pir756	0	19	0.001	0.48	8.906890596	Up	3.27E-05	4.64E-05
novel_pir943	0	19	0.001	0.48	8.906890596	Up	3.27E-05	4.63E-05
novel_pir1450	0	19	0.001	0.48	8.906890596	Up	3.27E-05	4.63E-05
novel_pir2055	0	19	0.001	0.48	8.906890596	Up	3.27E-05	4.63E-05
novel_pir966	0	19	0.001	0.48	8.906890596	Up	3.27E-05	4.62E-05
novel_pir81	0	19	0.001	0.48	8.906890596	Up	3.27E-05	4.62E-05
novel_pir2205	0	18	0.001	0.45	8.813781191	Up	5.68E-05	8.01E-05
novel_pir1061	0	18	0.001	0.45	8.813781191	Up	5.68E-05	8.00E-05
novel_pir1764	0	18	0.001	0.45	8.813781191	Up	5.68E-05	8.00E-05
novel_pir1507	0	18	0.001	0.45	8.813781191	Up	5.68E-05	7.99E-05
novel_pir2397	0	18	0.001	0.45	8.813781191	Up	5.68E-05	7.98E-05
novel_pir1162	0	18	0.001	0.45	8.813781191	Up	5.68E-05	7.98E-05
novel_pir88	0	18	0.001	0.45	8.813781191	Up	5.68E-05	7.97E-05
novel_pir1569	0	18	0.001	0.45	8.813781191	Up	5.68E-05	7.97E-05
novel_pir247	0	18	0.001	0.45	8.813781191	Up	5.68E-05	7.96E-05
novel_pir1374	0	18	0.001	0.45	8.813781191	Up	5.68E-05	7.95E-05
novel_pir129	0	18	0.001	0.45	8.813781191	Up	5.68E-05	7.95E-05
novel_pir1516	0	18	0.001	0.45	8.813781191	Up	5.68E-05	7.94E-05
novel_pir381	0	18	0.001	0.45	8.813781191	Up	5.68E-05	7.94E-05

novel_pir560	0	18	0.001	0.45	8.813781191	Up	5.68E-05	7.93E-05
novel_pir1	0	18	0.001	0.45	8.813781191	Up	5.68E-05	7.92E-05
novel_pir87	0	18	0.001	0.45	8.813781191	Up	5.68E-05	7.92E-05
novel_pir1233	0	18	0.001	0.45	8.813781191	Up	5.68E-05	7.91E-05
novel_pir726	0	18	0.001	0.45	8.813781191	Up	5.68E-05	7.91E-05
novel_pir1752	0	18	0.001	0.45	8.813781191	Up	5.68E-05	7.90E-05
novel_pir2052	0	18	0.001	0.45	8.813781191	Up	5.68E-05	7.90E-05
novel_pir71	0	18	0.001	0.45	8.813781191	Up	5.68E-05	7.89E-05
novel_pir666	0	17	0.001	0.43	8.74819285	Up	9.85E-05	0.000135691
novel_pir432	0	17	0.001	0.43	8.74819285	Up	9.85E-05	0.000135592
novel_pir1933	0	17	0.001	0.43	8.74819285	Up	9.85E-05	0.000135493
novel_pir619	0	17	0.001	0.43	8.74819285	Up	9.85E-05	0.000135394
novel_pir1303	0	17	0.001	0.43	8.74819285	Up	9.85E-05	0.000135295
novel_pir1777	0	17	0.001	0.43	8.74819285	Up	9.85E-05	0.000135196
novel_pir1931	0	17	0.001	0.43	8.74819285	Up	9.85E-05	0.000135098
novel_pir2464	0	17	0.001	0.43	8.74819285	Up	9.85E-05	0.000134999
novel_pir765	0	17	0.001	0.43	8.74819285	Up	9.85E-05	0.000134901
mmu_piR_022956	0	17	0.001	0.43	8.74819285	Up	9.85E-05	0.000134803
novel_pir395	0	17	0.001	0.43	8.74819285	Up	9.85E-05	0.000134705
novel_pir2455	0	17	0.001	0.43	8.74819285	Up	9.85E-05	0.000134607
novel_pir2089	0	16	0.001	0.4	8.64385619	Up	0.000170882	0.0002312
novel_pir114	0	16	0.001	0.4	8.64385619	Up	0.000170882	0.000231034
novel_pir1329	0	16	0.001	0.4	8.64385619	Up	0.000170882	0.000230868
novel_pir527	0	16	0.001	0.4	8.64385619	Up	0.000170882	0.000230703
novel_pir1889	0	16	0.001	0.4	8.64385619	Up	0.000170882	0.000230537
novel_pir870	0	16	0.001	0.4	8.64385619	Up	0.000170882	0.000230372
novel_pir2456	0	16	0.001	0.4	8.64385619	Up	0.000170882	0.000230207
novel_pir2371	0	16	0.001	0.4	8.64385619	Up	0.000170882	0.000230043
novel_pir1626	0	16	0.001	0.4	8.64385619	Up	0.000170882	0.000229878
novel_pir86	0	16	0.001	0.4	8.64385619	Up	0.000170882	0.000229714
novel_pir1903	0	16	0.001	0.4	8.64385619	Up	0.000170882	0.00022955
novel_pir1654	0	16	0.001	0.4	8.64385619	Up	0.000170882	0.000229386
novel_pir1414	0	16	0.001	0.4	8.64385619	Up	0.000170882	0.000229223
novel_pir26	0	16	0.001	0.4	8.64385619	Up	0.000170882	0.00022906
novel_pir992	0	16	0.001	0.4	8.64385619	Up	0.000170882	0.000228896
novel_pir1697	0	16	0.001	0.4	8.64385619	Up	0.000170882	0.000228734

novel_pir679	0	16	0.001	0.4	8.64385619	Up	0.000170882	0.000228571
novel_pir300	0	16	0.001	0.4	8.64385619	Up	0.000170882	0.000228409
novel_pir673	0	16	0.001	0.4	8.64385619	Up	0.000170882	0.000228247
novel_pir1204	0	16	0.001	0.4	8.64385619	Up	0.000170882	0.000228085
novel_pir2127	0	16	0.001	0.4	8.64385619	Up	0.000170882	0.000227923
novel_pir578	0	16	0.001	0.4	8.64385619	Up	0.000170882	0.000227762
novel_pir2262	0	16	0.001	0.4	8.64385619	Up	0.000170882	0.000227601
novel_pir92	0	16	0.001	0.4	8.64385619	Up	0.000170882	0.00022744
novel_pir1101	0	16	0.001	0.4	8.64385619	Up	0.000170882	0.000227279
novel_pir138	0	16	0.001	0.4	8.64385619	Up	0.000170882	0.000227118
novel_pir1447	0	16	0.001	0.4	8.64385619	Up	0.000170882	0.000226958
novel_pir1634	0	15	0.001	0.38	8.569855608	Up	0.00029649	0.000393508
novel_pir83	0	15	0.001	0.38	8.569855608	Up	0.00029649	0.000393231
novel_pir1346	0	15	0.001	0.38	8.569855608	Up	0.00029649	0.000392954
novel_pir1403	0	15	0.001	0.38	8.569855608	Up	0.00029649	0.000392677
novel_pir1311	0	15	0.001	0.38	8.569855608	Up	0.00029649	0.000392401
novel_pir2270	0	15	0.001	0.38	8.569855608	Up	0.00029649	0.000392125
novel_pir128	0	15	0.001	0.38	8.569855608	Up	0.00029649	0.00039185
novel_pir1789	0	15	0.001	0.38	8.569855608	Up	0.00029649	0.000391575
novel_pir528	0	15	0.001	0.38	8.569855608	Up	0.00029649	0.0003913
novel_pir1130	0	15	0.001	0.38	8.569855608	Up	0.00029649	0.000391026
novel_pir1010	0	15	0.001	0.38	8.569855608	Up	0.00029649	0.000390752
novel_pir391	0	15	0.001	0.38	8.569855608	Up	0.00029649	0.000390479
novel_pir1263	0	15	0.001	0.38	8.569855608	Up	0.00029649	0.000390206
novel_pir2231	0	15	0.001	0.38	8.569855608	Up	0.00029649	0.000389933
novel_pir1102	0	15	0.001	0.38	8.569855608	Up	0.00029649	0.000389661
novel_pir439	0	15	0.001	0.38	8.569855608	Up	0.00029649	0.000389389
novel_pir1014	0	15	0.001	0.38	8.569855608	Up	0.00029649	0.000389117
novel_pir1217	0	15	0.001	0.38	8.569855608	Up	0.00029649	0.000388846
novel_pir988	0	15	0.001	0.38	8.569855608	Up	0.00029649	0.000388575
novel_pir989	0	15	0.001	0.38	8.569855608	Up	0.00029649	0.000388305
novel_pir2407	0	15	0.001	0.38	8.569855608	Up	0.00029649	0.000388035
novel_pir2051	0	15	0.001	0.38	8.569855608	Up	0.00029649	0.000387765
novel_pir2366	0	15	0.001	0.38	8.569855608	Up	0.00029649	0.000387496
novel_pir1180	0	15	0.001	0.38	8.569855608	Up	0.00029649	0.000387227
novel_pir106	0	15	0.001	0.38	8.569855608	Up	0.00029649	0.000386959

novel_pir1961	0	15	0.001	0.38	8.569855608	Up	0.00029649	0.00038669
novel_pir930	0	15	0.001	0.38	8.569855608	Up	0.00029649	0.000386423
novel_pir582	0	15	0.001	0.38	8.569855608	Up	0.00029649	0.000386155
novel_pir58	0	15	0.001	0.38	8.569855608	Up	0.00029649	0.000385888
novel_pir270	0	15	0.001	0.38	8.569855608	Up	0.00029649	0.000385621
novel_pir2131	0	15	0.001	0.38	8.569855608	Up	0.00029649	0.000385355
novel_pir1717	0	15	0.001	0.38	8.569855608	Up	0.00029649	0.000385089
novel_pir56	0	15	0.001	0.38	8.569855608	Up	0.00029649	0.000384824
novel_pir2094	0	15	0.001	0.38	8.569855608	Up	0.00029649	0.000384558
novel_pir2269	0	15	0.001	0.38	8.569855608	Up	0.00029649	0.000384294
novel_pir127	0	15	0.001	0.38	8.569855608	Up	0.00029649	0.000384029
novel_pir438	0	15	0.001	0.38	8.569855608	Up	0.00029649	0.000383765
novel_pir1216	0	15	0.001	0.38	8.569855608	Up	0.00029649	0.000383501
novel_pir2255	0	15	0.001	0.38	8.569855608	Up	0.00029649	0.000383238
novel_pir583	0	14	0.001	0.35	8.451211112	Up	0.000514426	0.000664026
novel_pir2280	0	14	0.001	0.35	8.451211112	Up	0.000514426	0.000663571
novel_pir171	0	14	0.001	0.35	8.451211112	Up	0.000514426	0.000663116
novel_pir338	0	14	0.001	0.35	8.451211112	Up	0.000514426	0.000662662
novel_pir241	0	14	0.001	0.35	8.451211112	Up	0.000514426	0.000662209
novel_pir1397	0	14	0.001	0.35	8.451211112	Up	0.000514426	0.000661756
novel_pir972	0	14	0.001	0.35	8.451211112	Up	0.000514426	0.000661304
novel_pir1774	0	14	0.001	0.35	8.451211112	Up	0.000514426	0.000660853
novel_pir2128	0	14	0.001	0.35	8.451211112	Up	0.000514426	0.000660402
novel_pir31	0	14	0.001	0.35	8.451211112	Up	0.000514426	0.000659952
novel_pir1247	0	14	0.001	0.35	8.451211112	Up	0.000514426	0.000659503
novel_pir763	0	14	0.001	0.35	8.451211112	Up	0.000514426	0.000659054
novel_pir1648	0	14	0.001	0.35	8.451211112	Up	0.000514426	0.000658605
novel_pir1651	0	14	0.001	0.35	8.451211112	Up	0.000514426	0.000658158
novel_pir1109	0	14	0.001	0.35	8.451211112	Up	0.000514426	0.00065771
novel_pir1639	0	14	0.001	0.35	8.451211112	Up	0.000514426	0.000657264
novel_pir2157	0	14	0.001	0.35	8.451211112	Up	0.000514426	0.000656818
novel_pir436	0	14	0.001	0.35	8.451211112	Up	0.000514426	0.000656373
novel_pir167	0	14	0.001	0.35	8.451211112	Up	0.000514426	0.000655928
novel_pir1155	0	14	0.001	0.35	8.451211112	Up	0.000514426	0.000655484
novel_pir795	0	14	0.001	0.35	8.451211112	Up	0.000514426	0.00065504
novel_pir687	0	14	0.001	0.35	8.451211112	Up	0.000514426	0.000654598

novel_pir2029	0	14	0.001	0.35	8.451211112	Up	0.000514426	0.000654155
novel_pir1222	0	14	0.001	0.35	8.451211112	Up	0.000514426	0.000653714
novel_pir297	0	14	0.001	0.35	8.451211112	Up	0.000514426	0.000653272
novel_pir366	0	14	0.001	0.35	8.451211112	Up	0.000514426	0.000652832
novel_pir515	0	14	0.001	0.35	8.451211112	Up	0.000514426	0.000652392
novel_pir2172	0	14	0.001	0.35	8.451211112	Up	0.000514426	0.000651953
novel_pir221	0	14	0.001	0.35	8.451211112	Up	0.000514426	0.000651514
novel_pir392	0	14	0.001	0.35	8.451211112	Up	0.000514426	0.000651076
novel_pir2232	0	14	0.001	0.35	8.451211112	Up	0.000514426	0.000650638
novel_pir1637	0	14	0.001	0.35	8.451211112	Up	0.000514426	0.000650201
novel_pir1755	0	14	0.001	0.35	8.451211112	Up	0.000514426	0.000649765
novel_pir1172	0	14	0.001	0.35	8.451211112	Up	0.000514426	0.000649329
novel_pir538	0	14	0.001	0.35	8.451211112	Up	0.000514426	0.000648894
novel_pir96	0	14	0.001	0.35	8.451211112	Up	0.000514426	0.000648459
novel_pir1875	0	14	0.001	0.35	8.451211112	Up	0.000514426	0.000648025
novel_pir574	0	14	0.001	0.35	8.451211112	Up	0.000514426	0.000647592
novel_pir781	0	14	0.001	0.35	8.451211112	Up	0.000514426	0.000647159
novel_pir1695	0	14	0.001	0.35	8.451211112	Up	0.000514426	0.000646727
novel_pir1380	0	14	0.001	0.35	8.451211112	Up	0.000514426	0.000646295
novel_pir2013	0	14	0.001	0.35	8.451211112	Up	0.000514426	0.000645864
mmu_piR_022097	32	4833	1.09	120.98	6.794296621	Up	0	0
mmu_piR_032015	4	343	0.14	8.59	5.939159399	Up	1.86E-75	5.72E-75
mmu_piR_000958	1	65	0.03	1.63	5.763765654	Up	9.29E-15	1.65E-14
mmu_piR_000935	2838	179644	96.65	4496.95	5.540033301	Up	0	0
mmu_piR_000870	1	55	0.03	1.38	5.523561956	Up	1.96E-12	3.40E-12
mmu_piR_033077	2	109	0.07	2.73	5.285402219	Up	1.08E-23	2.15E-23
mmu_piR_034411	2	107	0.07	2.68	5.258734268	Up	3.15E-23	6.22E-23
mmu_piR_038322	1	30	0.03	0.75	4.64385619	Up	1.08E-06	1.65E-06
mmu_piR_038312	1	26	0.03	0.65	4.437405312	Up	8.60E-06	1.26E-05
novel_pir2034	1123	19612	38.24	490.94	3.682392289	Up	0	0
mmu_piR_000159	3	42	0.1	1.05	3.392317423	Up	1.30E-07	2.06E-07
mmu_piR_000273	117	759	3.98	19	2.255159083	Up	1.25E-77	3.89E-77
novel_pir125	4195	0	142.86	0.001	-17.1242425	Down	0	0
novel_pir613	1945	0	66.24	0.001	-16.01541505	Down	0	0
novel_pir13	1438	0	48.97	0.001	-15.57961058	Down	0	0
novel_pir274	1281	0	43.62	0.001	-15.41270215	Down	0	0

novel_pir14	1223	0	41.65	0.001	-15.34602888	Down	0	0
mmu_piR_024749	1110	0	37.8	0.001	-15.20609861	Down	0	0
novel_pir615	991	0	33.75	0.001	-15.04259988	Down	0	0
novel_pir340	856	0	29.15	0.001	-14.83120826	Down	0	0
novel_pir123	853	0	29.05	0.001	-14.82625054	Down	0	0
novel_pir863	778	0	26.5	0.001	-14.69370474	Down	5.49E-291	3.56E-289
novel_pir728	764	0	26.02	0.001	-14.66733334	Down	9.15E-286	5.74E-284
novel_pir961	743	0	25.3	0.001	-14.62684976	Down	6.23E-278	3.66E-276
novel_pir136	720	0	24.52	0.001	-14.58167136	Down	2.36E-269	1.35E-267
novel_pir1524	686	0	23.36	0.001	-14.51175265	Down	1.14E-256	6.28E-255
novel_pir194	684	0	23.29	0.001	-14.50742302	Down	6.32E-256	3.40E-254
novel_pir611	675	0	22.99	0.001	-14.48871885	Down	1.44E-252	7.32E-251
novel_pir2175	670	0	22.82	0.001	-14.47801117	Down	1.05E-250	5.22E-249
novel_pir1517	663	0	22.58	0.001	-14.46275787	Down	4.30E-248	2.03E-246
novel_pir778	661	0	22.51	0.001	-14.45827844	Down	2.40E-247	1.07E-245
novel_pir1581	637	0	21.69	0.001	-14.40474243	Down	2.15E-238	9.39E-237
novel_pir967	627	0	21.35	0.001	-14.38194845	Down	1.15E-234	4.71E-233
novel_pir2332	625	0	21.28	0.001	-14.37721053	Down	6.42E-234	2.57E-232
novel_pir1994	616	0	20.98	0.001	-14.35672706	Down	1.46E-230	5.73E-229
novel_pir2237	612	0	20.84	0.001	-14.34706766	Down	4.53E-229	1.71E-227
novel_pir79	599	0	20.4	0.001	-14.31628153	Down	3.20E-224	1.18E-222
novel_pir780	597	0	20.33	0.001	-14.31132259	Down	1.78E-223	6.46E-222
novel_pir10	588	0	20.02	0.001	-14.28915435	Down	4.06E-220	1.44E-218
novel_pir47	580	0	19.75	0.001	-14.26956503	Down	3.91E-217	1.36E-215
novel_pir287	574	0	19.55	0.001	-14.25488099	Down	6.76E-215	2.31E-213
novel_pir2321	568	0	19.34	0.001	-14.23930017	Down	1.17E-212	3.93E-211
novel_pir1707	560	0	19.07	0.001	-14.21901722	Down	1.13E-209	3.66E-208
novel_pir1262	556	0	18.93	0.001	-14.20838679	Down	3.50E-208	1.12E-206
novel_pir1368	550	0	18.73	0.001	-14.19306328	Down	6.05E-206	1.90E-204
novel_pir1334	532	0	18.12	0.001	-14.14529533	Down	3.13E-199	9.67E-198
novel_pir1179	519	0	17.67	0.001	-14.10901442	Down	2.21E-194	6.72E-193
novel_pir166	517	0	17.61	0.001	-14.10410729	Down	1.23E-193	3.68E-192
novel_pir766	512	0	17.44	0.001	-14.09011242	Down	9.03E-192	2.62E-190
novel_pir228	510	0	17.37	0.001	-14.08431013	Down	5.03E-191	1.43E-189
novel_pir551	508	0	17.3	0.001	-14.07848442	Down	2.80E-190	7.88E-189
novel_pir2184	507	0	17.27	0.001	-14.07598046	Down	6.62E-190	1.83E-188

novel_pir126	502	0	17.1	0.001	-14.0617087	Down	4.85E-188	1.32E-186
novel_pir1382	500	0	17.03	0.001	-14.05579081	Down	2.70E-187	7.26E-186
novel_pir1207	495	0	16.86	0.001	-14.04131692	Down	1.98E-185	5.25E-184
novel_pir2257	491	0	16.72	0.001	-14.02928723	Down	6.15E-184	1.61E-182
novel_pir2099	484	0	16.48	0.001	-14.00842862	Down	2.51E-181	6.47E-180
novel_pir67	484	0	16.48	0.001	-14.00842862	Down	2.51E-181	6.38E-180
novel_pir1734	484	0	16.48	0.001	-14.00842862	Down	2.51E-181	6.30E-180
novel_pir2083	483	0	16.45	0.001	-14.00579996	Down	5.92E-181	1.47E-179
novel_pir3	480	0	16.35	0.001	-13.99700302	Down	7.79E-180	1.90E-178
novel_pir617	476	0	16.21	0.001	-13.98459647	Down	2.42E-178	5.83E-177
novel_pir2049	476	0	16.21	0.001	-13.98459647	Down	2.42E-178	5.76E-177
novel_pir6	476	0	16.21	0.001	-13.98459647	Down	2.42E-178	5.69E-177
novel_pir628	472	0	16.07	0.001	-13.97208231	Down	7.51E-177	1.74E-175
novel_pir364	471	0	16.04	0.001	-13.96938652	Down	1.77E-176	4.07E-175
novel_pir329	469	0	15.97	0.001	-13.96307669	Down	9.87E-176	2.24E-174
novel_pir573	468	0	15.94	0.001	-13.96036401	Down	2.33E-175	5.22E-174
novel_pir2086	466	0	15.87	0.001	-13.95401451	Down	1.30E-174	2.88E-173
novel_pir226	464	0	15.8	0.001	-13.94763694	Down	7.23E-174	1.58E-172
novel_pir872	461	0	15.7	0.001	-13.93847694	Down	9.51E-173	2.06E-171
novel_pir1406	460	0	15.67	0.001	-13.93571756	Down	2.25E-172	4.80E-171
novel_pir111	459	0	15.63	0.001	-13.93203016	Down	5.30E-172	1.12E-170
novel_pir2214	458	0	15.6	0.001	-13.92925841	Down	1.25E-171	2.62E-170
novel_pir195	458	0	15.6	0.001	-13.92925841	Down	1.25E-171	2.59E-170
novel_pir2306	458	0	15.6	0.001	-13.92925841	Down	1.25E-171	2.56E-170
novel_pir68	458	0	15.6	0.001	-13.92925841	Down	1.25E-171	2.53E-170
novel_pir547	456	0	15.53	0.001	-13.92277021	Down	6.97E-171	1.40E-169
novel_pir2093	455	0	15.5	0.001	-13.9199806	Down	1.65E-170	3.26E-169
novel_pir336	452	0	15.39	0.001	-13.90970561	Down	2.16E-169	4.24E-168
novel_pir1148	450	0	15.32	0.001	-13.90312868	Down	1.21E-168	2.34E-167
novel_pir1215	449	0	15.29	0.001	-13.90030079	Down	2.85E-168	5.47E-167
novel_pir1161	447	0	15.22	0.001	-13.89368074	Down	1.59E-167	3.01E-166
novel_pir2026	445	0	15.15	0.001	-13.88703017	Down	8.84E-167	1.66E-165
novel_pir2271	443	0	15.09	0.001	-13.88130519	Down	4.92E-166	9.17E-165
novel_pir348	441	0	15.02	0.001	-13.87459719	Down	2.74E-165	5.06E-164
novel_pir469	438	0	14.92	0.001	-13.86495992	Down	3.61E-164	6.59E-163
novel_pir1705	438	0	14.92	0.001	-13.86495992	Down	3.61E-164	6.53E-163

novel_pir380	436	0	14.85	0.001	-13.85817531	Down	2.01E-163	3.60E-162
novel_pir2263	436	0	14.85	0.001	-13.85817531	Down	2.01E-163	3.57E-162
novel_pir1767	434	0	14.78	0.001	-13.85135865	Down	1.12E-162	1.97E-161
novel_pir575	433	0	14.75	0.001	-13.84842733	Down	2.64E-162	4.61E-161
novel_pir1258	433	0	14.75	0.001	-13.84842733	Down	2.64E-162	4.56E-161
novel_pir347	432	0	14.71	0.001	-13.84450963	Down	6.24E-162	1.07E-160
novel_pir400	432	0	14.71	0.001	-13.84450963	Down	6.24E-162	1.06E-160
novel_pir1937	432	0	14.71	0.001	-13.84450963	Down	6.24E-162	1.05E-160
novel_pir1689	430	0	14.64	0.001	-13.83762793	Down	3.48E-161	5.74E-160
novel_pir1716	430	0	14.64	0.001	-13.83762793	Down	3.48E-161	5.69E-160
novel_pir809	429	0	14.61	0.001	-13.83466856	Down	8.21E-161	1.33E-159
novel_pir1073	429	0	14.61	0.001	-13.83466856	Down	8.21E-161	1.32E-159
novel_pir2139	429	0	14.61	0.001	-13.83466856	Down	8.21E-161	1.31E-159
novel_pir1218	429	0	14.61	0.001	-13.83466856	Down	8.21E-161	1.30E-159
novel_pir2095	427	0	14.54	0.001	-13.82773965	Down	4.57E-160	7.17E-159
novel_pir1881	427	0	14.54	0.001	-13.82773965	Down	4.57E-160	7.11E-159
novel_pir1055	426	0	14.51	0.001	-13.8247599	Down	1.08E-159	1.66E-158
novel_pir2059	422	0	14.37	0.001	-13.81077244	Down	3.35E-158	5.13E-157
novel_pir651	420	0	14.3	0.001	-13.80372753	Down	1.87E-157	2.83E-156
novel_pir820	417	0	14.2	0.001	-13.79360331	Down	2.46E-156	3.70E-155
novel_pir2226	416	0	14.17	0.001	-13.79055214	Down	5.80E-156	8.66E-155
novel_pir204	416	0	14.17	0.001	-13.79055214	Down	5.80E-156	8.59E-155
novel_pir1065	413	0	14.06	0.001	-13.77930897	Down	7.62E-155	1.12E-153
novel_pir119	412	0	14.03	0.001	-13.77622739	Down	1.80E-154	2.62E-153
novel_pir2018	412	0	14.03	0.001	-13.77622739	Down	1.80E-154	2.60E-153
novel_pir1395	412	0	14.03	0.001	-13.77622739	Down	1.80E-154	2.58E-153
novel_pir1684	412	0	14.03	0.001	-13.77622739	Down	1.80E-154	2.57E-153
novel_pir1441	411	0	14	0.001	-13.77313921	Down	4.25E-154	6.01E-153
novel_pir1159	408	0	13.89	0.001	-13.76175898	Down	5.58E-153	7.79E-152
novel_pir495	407	0	13.86	0.001	-13.75863964	Down	1.32E-152	1.82E-151
novel_pir1100	405	0	13.79	0.001	-13.75133484	Down	7.35E-152	1.01E-150
novel_pir22	405	0	13.79	0.001	-13.75133484	Down	7.35E-152	1.00E-150
novel_pir1775	404	0	13.76	0.001	-13.74819285	Down	1.73E-151	2.35E-150
novel_pir2105	403	0	13.72	0.001	-13.74399286	Down	4.09E-151	5.50E-150
novel_pir2395	403	0	13.72	0.001	-13.74399286	Down	4.09E-151	5.46E-150
novel_pir1900	403	0	13.72	0.001	-13.74399286	Down	4.09E-151	5.42E-150

novel_pir783	401	0	13.66	0.001	-13.73766986	Down	2.28E-150	3.00E-149
novel_pir1465	400	0	13.62	0.001	-13.73343908	Down	5.38E-150	7.03E-149
novel_pir396	399	0	13.59	0.001	-13.73025784	Down	1.27E-149	1.65E-148
novel_pir2193	398	0	13.55	0.001	-13.72600523	Down	3.00E-149	3.87E-148
novel_pir917	397	0	13.52	0.001	-13.72280753	Down	7.08E-149	9.06E-148
novel_pir1643	397	0	13.52	0.001	-13.72280753	Down	7.08E-149	9.00E-148
novel_pir315	397	0	13.52	0.001	-13.72280753	Down	7.08E-149	8.94E-148
novel_pir612	397	0	13.52	0.001	-13.72280753	Down	7.08E-149	8.88E-148
novel_pir1114	395	0	13.45	0.001	-13.71531855	Down	3.94E-148	4.92E-147
novel_pir1028	394	0	13.42	0.001	-13.71209705	Down	9.31E-148	1.15E-146
novel_pir2354	394	0	13.42	0.001	-13.71209705	Down	9.31E-148	1.15E-146
novel_pir1659	393	0	13.38	0.001	-13.7077905	Down	2.20E-147	2.69E-146
novel_pir516	393	0	13.38	0.001	-13.7077905	Down	2.20E-147	2.67E-146
novel_pir1359	391	0	13.32	0.001	-13.70130646	Down	1.22E-146	1.48E-145
novel_pir876	388	0	13.21	0.001	-13.68934285	Down	1.61E-145	1.93E-144
novel_pir751	388	0	13.21	0.001	-13.68934285	Down	1.61E-145	1.92E-144
novel_pir1088	388	0	13.21	0.001	-13.68934285	Down	1.61E-145	1.91E-144
novel_pir1862	388	0	13.21	0.001	-13.68934285	Down	1.61E-145	1.89E-144
novel_pir1340	387	0	13.18	0.001	-13.68606275	Down	3.80E-145	4.44E-144
novel_pir23	387	0	13.18	0.001	-13.68606275	Down	3.80E-145	4.42E-144
novel_pir1241	386	0	13.15	0.001	-13.68277518	Down	8.97E-145	1.04E-143
novel_pir314	386	0	13.15	0.001	-13.68277518	Down	8.97E-145	1.03E-143
novel_pir346	386	0	13.15	0.001	-13.68277518	Down	8.97E-145	1.02E-143
novel_pir2192	385	0	13.11	0.001	-13.67838007	Down	2.12E-144	2.40E-143
novel_pir1481	384	0	13.08	0.001	-13.67507492	Down	5.00E-144	5.63E-143
novel_pir1670	382	0	13.01	0.001	-13.66733334	Down	2.79E-143	3.12E-142
novel_pir713	381	0	12.98	0.001	-13.66400276	Down	6.57E-143	7.32E-142
novel_pir540	381	0	12.98	0.001	-13.66400276	Down	6.57E-143	7.28E-142
novel_pir481	381	0	12.98	0.001	-13.66400276	Down	6.57E-143	7.24E-142
novel_pir282	381	0	12.98	0.001	-13.66400276	Down	6.57E-143	7.19E-142
novel_pir974	381	0	12.98	0.001	-13.66400276	Down	6.57E-143	7.15E-142
novel_pir959	381	0	12.98	0.001	-13.66400276	Down	6.57E-143	7.11E-142
novel_pir548	380	0	12.94	0.001	-13.65955	Down	1.55E-142	1.66E-141
novel_pir669	380	0	12.94	0.001	-13.65955	Down	1.55E-142	1.65E-141
novel_pir1740	379	0	12.91	0.001	-13.65620138	Down	3.66E-142	3.87E-141
novel_pir1727	379	0	12.91	0.001	-13.65620138	Down	3.66E-142	3.85E-141

novel_pir2254	378	0	12.87	0.001	-13.65172443	Down	8.65E-142	9.04E-141
novel_pir545	378	0	12.87	0.001	-13.65172443	Down	8.65E-142	8.99E-141
novel_pir697	377	0	12.84	0.001	-13.64835758	Down	2.04E-141	2.10E-140
novel_pir2289	376	0	12.8	0.001	-13.64385619	Down	4.82E-141	4.93E-140
novel_pir2101	376	0	12.8	0.001	-13.64385619	Down	4.82E-141	4.90E-140
novel_pir2375	375	0	12.77	0.001	-13.6404709	Down	1.14E-140	1.15E-139
novel_pir1985	374	0	12.74	0.001	-13.63707766	Down	2.68E-140	2.70E-139
novel_pir1721	373	0	12.7	0.001	-13.63254088	Down	6.34E-140	6.34E-139
novel_pir1034	373	0	12.7	0.001	-13.63254088	Down	6.34E-140	6.31E-139
novel_pir1294	372	0	12.67	0.001	-13.6291289	Down	1.50E-139	1.48E-138
novel_pir1750	371	0	12.63	0.001	-13.62456702	Down	3.53E-139	3.48E-138
novel_pir2176	371	0	12.63	0.001	-13.62456702	Down	3.53E-139	3.46E-138
novel_pir688	370	0	12.6	0.001	-13.62113611	Down	8.33E-139	8.12E-138
novel_pir1388	370	0	12.6	0.001	-13.62113611	Down	8.33E-139	8.08E-138
novel_pir1984	369	0	12.57	0.001	-13.61769703	Down	1.97E-138	1.90E-137
novel_pir1942	368	0	12.53	0.001	-13.61309879	Down	4.64E-138	4.46E-137
novel_pir1269	367	0	12.5	0.001	-13.60964047	Down	1.10E-137	1.05E-136
novel_pir634	367	0	12.5	0.001	-13.60964047	Down	1.10E-137	1.04E-136
novel_pir205	366	0	12.46	0.001	-13.60501645	Down	2.59E-137	2.45E-136
novel_pir124	366	0	12.46	0.001	-13.60501645	Down	2.59E-137	2.43E-136
novel_pir2265	364	0	12.4	0.001	-13.5980525	Down	1.44E-136	1.35E-135
novel_pir774	364	0	12.4	0.001	-13.5980525	Down	1.44E-136	1.34E-135
novel_pir2471	364	0	12.4	0.001	-13.5980525	Down	1.44E-136	1.34E-135
novel_pir1379	364	0	12.4	0.001	-13.5980525	Down	1.44E-136	1.33E-135
novel_pir954	364	0	12.4	0.001	-13.5980525	Down	1.44E-136	1.32E-135
novel_pir448	363	0	12.36	0.001	-13.59339112	Down	3.40E-136	3.11E-135
novel_pir374	362	0	12.33	0.001	-13.58988518	Down	8.03E-136	7.30E-135
novel_pir1676	362	0	12.33	0.001	-13.58988518	Down	8.03E-136	7.27E-135
novel_pir2378	361	0	12.29	0.001	-13.5851973	Down	1.90E-135	1.71E-134
novel_pir1804	361	0	12.29	0.001	-13.5851973	Down	1.90E-135	1.70E-134
novel_pir1232	361	0	12.29	0.001	-13.5851973	Down	1.90E-135	1.69E-134
novel_pir775	360	0	12.26	0.001	-13.58167136	Down	4.47E-135	3.97E-134
novel_pir556	360	0	12.26	0.001	-13.58167136	Down	4.47E-135	3.95E-134
novel_pir682	360	0	12.26	0.001	-13.58167136	Down	4.47E-135	3.93E-134
novel_pir416	359	0	12.23	0.001	-13.57813678	Down	1.06E-134	9.24E-134
novel_pir377	359	0	12.23	0.001	-13.57813678	Down	1.06E-134	9.20E-134

novel_pir2314	358	0	12.19	0.001	-13.57341051	Down	2.49E-134	2.16E-133
novel_pir743	358	0	12.19	0.001	-13.57341051	Down	2.49E-134	2.15E-133
novel_pir2097	356	0	12.12	0.001	-13.56510208	Down	1.39E-133	1.19E-132
novel_pir667	356	0	12.12	0.001	-13.56510208	Down	1.39E-133	1.19E-132
novel_pir1260	354	0	12.06	0.001	-13.55794229	Down	7.74E-133	6.56E-132
novel_pir591	354	0	12.06	0.001	-13.55794229	Down	7.74E-133	6.53E-132
novel_pir748	354	0	12.06	0.001	-13.55794229	Down	7.74E-133	6.50E-132
novel_pir1647	353	0	12.02	0.001	-13.55314928	Down	1.83E-132	1.53E-131
novel_pir1814	352	0	11.99	0.001	-13.54954404	Down	4.31E-132	3.59E-131
novel_pir1791	352	0	11.99	0.001	-13.54954404	Down	4.31E-132	3.57E-131
novel_pir1523	352	0	11.99	0.001	-13.54954404	Down	4.31E-132	3.56E-131
novel_pir313	351	0	11.95	0.001	-13.544723	Down	1.02E-131	8.36E-131
novel_pir37	350	0	11.92	0.001	-13.54109662	Down	2.40E-131	1.97E-130
novel_pir1493	350	0	11.92	0.001	-13.54109662	Down	2.40E-131	1.96E-130
novel_pir369	349	0	11.89	0.001	-13.53746109	Down	5.67E-131	4.60E-130
novel_pir7	349	0	11.89	0.001	-13.53746109	Down	5.67E-131	4.58E-130
novel_pir1319	349	0	11.89	0.001	-13.53746109	Down	5.67E-131	4.56E-130
novel_pir664	349	0	11.89	0.001	-13.53746109	Down	5.67E-131	4.54E-130
novel_pir862	349	0	11.89	0.001	-13.53746109	Down	5.67E-131	4.52E-130
novel_pir1415	348	0	11.85	0.001	-13.53259944	Down	1.34E-130	1.06E-129
novel_pir1012	348	0	11.85	0.001	-13.53259944	Down	1.34E-130	1.06E-129
novel_pir186	348	0	11.85	0.001	-13.53259944	Down	1.34E-130	1.05E-129
novel_pir206	347	0	11.82	0.001	-13.52894242	Down	3.16E-130	2.48E-129
novel_pir767	347	0	11.82	0.001	-13.52894242	Down	3.16E-130	2.47E-129
novel_pir242	347	0	11.82	0.001	-13.52894242	Down	3.16E-130	2.46E-129
novel_pir724	346	0	11.78	0.001	-13.52405192	Down	7.46E-130	5.78E-129
novel_pir1203	346	0	11.78	0.001	-13.52405192	Down	7.46E-130	5.75E-129
novel_pir243	346	0	11.78	0.001	-13.52405192	Down	7.46E-130	5.73E-129
novel_pir2107	345	0	11.75	0.001	-13.52037314	Down	1.76E-129	1.35E-128
novel_pir2168	344	0	11.72	0.001	-13.51668495	Down	4.15E-129	3.17E-128
novel_pir656	344	0	11.72	0.001	-13.51668495	Down	4.15E-129	3.15E-128
novel_pir1135	343	0	11.68	0.001	-13.51175265	Down	9.81E-129	7.41E-128
novel_pir283	343	0	11.68	0.001	-13.51175265	Down	9.81E-129	7.38E-128
novel_pir102	342	0	11.65	0.001	-13.50804233	Down	2.31E-128	1.74E-127
novel_pir910	342	0	11.65	0.001	-13.50804233	Down	2.31E-128	1.73E-127
novel_pir1190	341	0	11.61	0.001	-13.50308035	Down	5.46E-128	4.06E-127

novel_pir655	341	0	11.61	0.001	-13.50308035	Down	5.46E-128	4.05E-127
novel_pir1644	341	0	11.61	0.001	-13.50308035	Down	5.46E-128	4.03E-127
novel_pir203	340	0	11.58	0.001	-13.49934763	Down	1.29E-127	9.48E-127
novel_pir493	340	0	11.58	0.001	-13.49934763	Down	1.29E-127	9.45E-127
novel_pir2476	340	0	11.58	0.001	-13.49934763	Down	1.29E-127	9.41E-127
novel_pir2	340	0	11.58	0.001	-13.49934763	Down	1.29E-127	9.37E-127
novel_pir489	340	0	11.58	0.001	-13.49934763	Down	1.29E-127	9.34E-127
novel_pir2080	339	0	11.54	0.001	-13.4943556	Down	3.04E-127	2.20E-126
novel_pir66	339	0	11.54	0.001	-13.4943556	Down	3.04E-127	2.19E-126
novel_pir1296	339	0	11.54	0.001	-13.4943556	Down	3.04E-127	2.18E-126
novel_pir567	339	0	11.54	0.001	-13.4943556	Down	3.04E-127	2.17E-126
novel_pir2219	339	0	11.54	0.001	-13.4943556	Down	3.04E-127	2.16E-126
novel_pir1066	338	0	11.51	0.001	-13.49060021	Down	7.19E-127	5.08E-126
novel_pir995	338	0	11.51	0.001	-13.49060021	Down	7.19E-127	5.07E-126
novel_pir1080	338	0	11.51	0.001	-13.49060021	Down	7.19E-127	5.05E-126
novel_pir260	337	0	11.48	0.001	-13.48683502	Down	1.70E-126	1.19E-125
novel_pir949	336	0	11.44	0.001	-13.48179943	Down	4.00E-126	2.79E-125
novel_pir596	336	0	11.44	0.001	-13.48179943	Down	4.00E-126	2.78E-125
novel_pir940	336	0	11.44	0.001	-13.48179943	Down	4.00E-126	2.77E-125
novel_pir1140	335	0	11.41	0.001	-13.47801117	Down	9.45E-126	6.52E-125
novel_pir2103	335	0	11.41	0.001	-13.47801117	Down	9.45E-126	6.49E-125
novel_pir1629	335	0	11.41	0.001	-13.47801117	Down	9.45E-126	6.47E-125
novel_pir485	334	0	11.37	0.001	-13.47294463	Down	2.23E-125	1.52E-124
novel_pir1432	334	0	11.37	0.001	-13.47294463	Down	2.23E-125	1.51E-124
novel_pir1611	333	0	11.34	0.001	-13.46913302	Down	5.27E-125	3.55E-124
novel_pir1730	333	0	11.34	0.001	-13.46913302	Down	5.27E-125	3.54E-124
novel_pir2195	331	0	11.27	0.001	-13.4601999	Down	2.93E-124	1.97E-123
novel_pir1231	330	0	11.24	0.001	-13.45635442	Down	6.93E-124	4.62E-123
novel_pir1079	330	0	11.24	0.001	-13.45635442	Down	6.93E-124	4.61E-123
novel_pir1874	330	0	11.24	0.001	-13.45635442	Down	6.93E-124	4.59E-123
novel_pir1385	329	0	11.2	0.001	-13.45121111	Down	1.63E-123	1.08E-122
novel_pir74	328	0	11.17	0.001	-13.44734157	Down	3.86E-123	2.54E-122
novel_pir2224	328	0	11.17	0.001	-13.44734157	Down	3.86E-123	2.53E-122
novel_pir1958	328	0	11.17	0.001	-13.44734157	Down	3.86E-123	2.52E-122
novel_pir1459	328	0	11.17	0.001	-13.44734157	Down	3.86E-123	2.51E-122
novel_pir51	327	0	11.14	0.001	-13.44346161	Down	9.11E-123	5.91E-122

novel_pir2227	326	0	11.1	0.001	-13.43827206	Down	2.15E-122	1.39E-121
novel_pir1979	326	0	11.1	0.001	-13.43827206	Down	2.15E-122	1.39E-121
novel_pir2266	325	0	11.07	0.001	-13.4343676	Down	5.07E-122	3.24E-121
novel_pir69	325	0	11.07	0.001	-13.4343676	Down	5.07E-122	3.23E-121
novel_pir211	325	0	11.07	0.001	-13.4343676	Down	5.07E-122	3.22E-121
novel_pir180	325	0	11.07	0.001	-13.4343676	Down	5.07E-122	3.20E-121
novel_pir2220	325	0	11.07	0.001	-13.4343676	Down	5.07E-122	3.19E-121
novel_pir1323	323	0	11	0.001	-13.4252159	Down	2.83E-121	1.77E-120
novel_pir973	323	0	11	0.001	-13.4252159	Down	2.83E-121	1.76E-120
novel_pir2260	323	0	11	0.001	-13.4252159	Down	2.83E-121	1.76E-120
novel_pir1794	322	0	10.97	0.001	-13.42127591	Down	6.67E-121	4.13E-120
novel_pir1993	321	0	10.93	0.001	-13.41600578	Down	1.58E-120	9.72E-120
novel_pir577	319	0	10.86	0.001	-13.40673648	Down	8.78E-120	5.40E-119
novel_pir1389	318	0	10.83	0.001	-13.40274562	Down	2.07E-119	1.27E-118
novel_pir1904	316	0	10.76	0.001	-13.39339046	Down	1.15E-118	7.05E-118
novel_pir1373	316	0	10.76	0.001	-13.39339046	Down	1.15E-118	7.03E-118
novel_pir2292	315	0	10.73	0.001	-13.38936246	Down	2.72E-118	1.65E-117
novel_pir389	315	0	10.73	0.001	-13.38936246	Down	2.72E-118	1.65E-117
novel_pir1443	315	0	10.73	0.001	-13.38936246	Down	2.72E-118	1.64E-117
novel_pir720	315	0	10.73	0.001	-13.38936246	Down	2.72E-118	1.64E-117
novel_pir1277	315	0	10.73	0.001	-13.38936246	Down	2.72E-118	1.63E-117
novel_pir175	315	0	10.73	0.001	-13.38936246	Down	2.72E-118	1.63E-117
novel_pir2273	314	0	10.69	0.001	-13.38397423	Down	6.43E-118	3.83E-117
novel_pir1490	313	0	10.66	0.001	-13.37991982	Down	1.52E-117	9.01E-117
novel_pir2357	313	0	10.66	0.001	-13.37991982	Down	1.52E-117	8.98E-117
novel_pir1918	312	0	10.63	0.001	-13.37585398	Down	3.58E-117	2.11E-116
novel_pir721	311	0	10.59	0.001	-13.37041497	Down	8.46E-117	4.97E-116
novel_pir1151	310	0	10.56	0.001	-13.36632221	Down	2.00E-116	1.17E-115
novel_pir535	309	0	10.52	0.001	-13.36084708	Down	4.71E-116	2.75E-115
novel_pir579	309	0	10.52	0.001	-13.36084708	Down	4.71E-116	2.75E-115
novel_pir627	309	0	10.52	0.001	-13.36084708	Down	4.71E-116	2.74E-115
novel_pir1392	309	0	10.52	0.001	-13.36084708	Down	4.71E-116	2.73E-115
novel_pir525	309	0	10.52	0.001	-13.36084708	Down	4.71E-116	2.72E-115
novel_pir1526	307	0	10.45	0.001	-13.35121532	Down	2.63E-115	1.50E-114
novel_pir1974	307	0	10.45	0.001	-13.35121532	Down	2.63E-115	1.50E-114
novel_pir1366	306	0	10.42	0.001	-13.34706766	Down	6.20E-115	3.52E-114

novel_pir635	306	0	10.42	0.001	-13.34706766	Down	6.20E-115	3.51E-114
novel_pir1718	306	0	10.42	0.001	-13.34706766	Down	6.20E-115	3.50E-114
novel_pir1564	305	0	10.39	0.001	-13.34290803	Down	1.46E-114	8.24E-114
novel_pir950	305	0	10.39	0.001	-13.34290803	Down	1.46E-114	8.22E-114
novel_pir1739	305	0	10.39	0.001	-13.34290803	Down	1.46E-114	8.19E-114
novel_pir1029	304	0	10.35	0.001	-13.33734315	Down	3.45E-114	1.93E-113
novel_pir2233	304	0	10.35	0.001	-13.33734315	Down	3.45E-114	1.92E-113
novel_pir1335	304	0	10.35	0.001	-13.33734315	Down	3.45E-114	1.92E-113
novel_pir1445	303	0	10.32	0.001	-13.33315535	Down	8.15E-114	4.51E-113
novel_pir140	303	0	10.32	0.001	-13.33315535	Down	8.15E-114	4.50E-113
novel_pir1950	303	0	10.32	0.001	-13.33315535	Down	8.15E-114	4.49E-113
novel_pir2043	302	0	10.28	0.001	-13.32755264	Down	1.92E-113	1.06E-112
novel_pir38	302	0	10.28	0.001	-13.32755264	Down	1.92E-113	1.05E-112
novel_pir563	301	0	10.25	0.001	-13.32333629	Down	4.54E-113	2.48E-112
novel_pir303	301	0	10.25	0.001	-13.32333629	Down	4.54E-113	2.47E-112
novel_pir2181	301	0	10.25	0.001	-13.32333629	Down	4.54E-113	2.46E-112
novel_pir875	301	0	10.25	0.001	-13.32333629	Down	4.54E-113	2.46E-112
novel_pir882	301	0	10.25	0.001	-13.32333629	Down	4.54E-113	2.45E-112
novel_pir784	301	0	10.25	0.001	-13.32333629	Down	4.54E-113	2.44E-112
novel_pir1006	300	0	10.22	0.001	-13.31910758	Down	1.07E-112	5.75E-112
novel_pir1002	300	0	10.22	0.001	-13.31910758	Down	1.07E-112	5.73E-112
novel_pir1152	299	0	10.18	0.001	-13.31344994	Down	2.53E-112	1.35E-111
novel_pir1910	299	0	10.18	0.001	-13.31344994	Down	2.53E-112	1.34E-111
novel_pir546	298	0	10.15	0.001	-13.30919211	Down	5.97E-112	3.16E-111
novel_pir207	298	0	10.15	0.001	-13.30919211	Down	5.97E-112	3.15E-111
novel_pir121	298	0	10.15	0.001	-13.30919211	Down	5.97E-112	3.14E-111
novel_pir1932	296	0	10.08	0.001	-13.29920802	Down	3.33E-111	1.74E-110
novel_pir1234	296	0	10.08	0.001	-13.29920802	Down	3.33E-111	1.74E-110
novel_pir76	295	0	10.05	0.001	-13.29490788	Down	7.86E-111	4.07E-110
novel_pir1605	295	0	10.05	0.001	-13.29490788	Down	7.86E-111	4.06E-110
novel_pir860	294	0	10.01	0.001	-13.28915435	Down	1.85E-110	9.56E-110
novel_pir1826	294	0	10.01	0.001	-13.28915435	Down	1.85E-110	9.54E-110
novel_pir2474	294	0	10.01	0.001	-13.28915435	Down	1.85E-110	9.51E-110
novel_pir334	294	0	10.01	0.001	-13.28915435	Down	1.85E-110	9.48E-110
novel_pir387	293	0	9.98	0.001	-13.2848241	Down	4.38E-110	2.23E-109
novel_pir694	293	0	9.98	0.001	-13.2848241	Down	4.38E-110	2.23E-109

novel_pir832	292	0	9.94	0.001	-13.27903014	Down	1.03E-109	5.23E-109
novel_pir1408	292	0	9.94	0.001	-13.27903014	Down	1.03E-109	5.21E-109
novel_pir2210	292	0	9.94	0.001	-13.27903014	Down	1.03E-109	5.20E-109
novel_pir1072	292	0	9.94	0.001	-13.27903014	Down	1.03E-109	5.19E-109
novel_pir1922	291	0	9.91	0.001	-13.27466934	Down	2.44E-109	1.22E-108
novel_pir1603	291	0	9.91	0.001	-13.27466934	Down	2.44E-109	1.22E-108
novel_pir190	291	0	9.91	0.001	-13.27466934	Down	2.44E-109	1.21E-108
novel_pir1894	290	0	9.88	0.001	-13.27029533	Down	5.76E-109	2.86E-108
novel_pir368	289	0	9.84	0.001	-13.2644426	Down	1.36E-108	6.73E-108
novel_pir1037	288	0	9.81	0.001	-13.26003742	Down	3.21E-108	1.58E-107
novel_pir978	288	0	9.81	0.001	-13.26003742	Down	3.21E-108	1.58E-107
novel_pir1502	288	0	9.81	0.001	-13.26003742	Down	3.21E-108	1.58E-107
novel_pir2390	287	0	9.77	0.001	-13.25414285	Down	7.57E-108	3.71E-107
novel_pir483	287	0	9.77	0.001	-13.25414285	Down	7.57E-108	3.70E-107
novel_pir2153	287	0	9.77	0.001	-13.25414285	Down	7.57E-108	3.69E-107
novel_pir1688	287	0	9.77	0.001	-13.25414285	Down	7.57E-108	3.68E-107
novel_pir779	287	0	9.77	0.001	-13.25414285	Down	7.57E-108	3.67E-107
novel_pir541	286	0	9.74	0.001	-13.24970606	Down	1.79E-107	8.65E-107
novel_pir2197	285	0	9.71	0.001	-13.24525558	Down	4.22E-107	2.04E-106
novel_pir1853	285	0	9.71	0.001	-13.24525558	Down	4.22E-107	2.03E-106
novel_pir2050	285	0	9.71	0.001	-13.24525558	Down	4.22E-107	2.03E-106
novel_pir2441	284	0	9.67	0.001	-13.23930017	Down	9.96E-107	4.77E-106
novel_pir1965	284	0	9.67	0.001	-13.23930017	Down	9.96E-107	4.76E-106
novel_pir1173	284	0	9.67	0.001	-13.23930017	Down	9.96E-107	4.74E-106
novel_pir630	284	0	9.67	0.001	-13.23930017	Down	9.96E-107	4.73E-106
novel_pir281	284	0	9.67	0.001	-13.23930017	Down	9.96E-107	4.72E-106
novel_pir1745	283	0	9.64	0.001	-13.23481743	Down	2.35E-106	1.11E-105
novel_pir805	283	0	9.64	0.001	-13.23481743	Down	2.35E-106	1.11E-105
novel_pir1855	283	0	9.64	0.001	-13.23481743	Down	2.35E-106	1.11E-105
novel_pir460	282	0	9.6	0.001	-13.22881869	Down	5.55E-106	2.60E-105
novel_pir702	281	0	9.57	0.001	-13.22430321	Down	1.31E-105	6.13E-105
novel_pir1971	281	0	9.57	0.001	-13.22430321	Down	1.31E-105	6.12E-105
novel_pir1225	281	0	9.57	0.001	-13.22430321	Down	1.31E-105	6.10E-105
novel_pir497	281	0	9.57	0.001	-13.22430321	Down	1.31E-105	6.08E-105
novel_pir1508	280	0	9.54	0.001	-13.21977355	Down	3.09E-105	1.43E-104
novel_pir349	280	0	9.54	0.001	-13.21977355	Down	3.09E-105	1.43E-104

novel_pir183	280	0	9.54	0.001	-13.21977355	Down	3.09E-105	1.43E-104
novel_pir722	279	0	9.5	0.001	-13.2137118	Down	7.30E-105	3.36E-104
novel_pir1129	279	0	9.5	0.001	-13.2137118	Down	7.30E-105	3.35E-104
novel_pir1194	279	0	9.5	0.001	-13.2137118	Down	7.30E-105	3.34E-104
novel_pir866	278	0	9.47	0.001	-13.20914871	Down	1.72E-104	7.87E-104
novel_pir580	278	0	9.47	0.001	-13.20914871	Down	1.72E-104	7.85E-104
novel_pir1400	277	0	9.43	0.001	-13.20304206	Down	4.07E-104	1.85E-103
novel_pir1086	277	0	9.43	0.001	-13.20304206	Down	4.07E-104	1.84E-103
novel_pir1986	277	0	9.43	0.001	-13.20304206	Down	4.07E-104	1.84E-103
novel_pir1786	276	0	9.4	0.001	-13.19844504	Down	9.60E-104	4.33E-103
novel_pir1377	276	0	9.4	0.001	-13.19844504	Down	9.60E-104	4.32E-103
novel_pir187	275	0	9.37	0.001	-13.19383333	Down	2.26E-103	1.02E-102
novel_pir990	275	0	9.37	0.001	-13.19383333	Down	2.26E-103	1.01E-102
novel_pir1238	275	0	9.37	0.001	-13.19383333	Down	2.26E-103	1.01E-102
novel_pir2383	274	0	9.33	0.001	-13.18766137	Down	5.35E-103	2.38E-102
novel_pir97	274	0	9.33	0.001	-13.18766137	Down	5.35E-103	2.38E-102
novel_pir2363	273	0	9.3	0.001	-13.183015	Down	1.26E-102	5.60E-102
novel_pir1749	273	0	9.3	0.001	-13.183015	Down	1.26E-102	5.59E-102
novel_pir2137	272	0	9.26	0.001	-13.17679648	Down	2.98E-102	1.32E-101
novel_pir502	271	0	9.23	0.001	-13.17211493	Down	7.03E-102	3.10E-101
novel_pir98	271	0	9.23	0.001	-13.17211493	Down	7.03E-102	3.09E-101
novel_pir1336	270	0	9.19	0.001	-13.16584915	Down	1.66E-101	7.26E-101
novel_pir337	269	0	9.16	0.001	-13.16113188	Down	3.92E-101	1.71E-100
novel_pir1156	269	0	9.16	0.001	-13.16113188	Down	3.92E-101	1.71E-100
novel_pir1302	268	0	9.13	0.001	-13.15639914	Down	9.25E-101	4.02E-100
novel_pir754	268	0	9.13	0.001	-13.15639914	Down	9.25E-101	4.01E-100
novel_pir1715	268	0	9.13	0.001	-13.15639914	Down	9.25E-101	4.00E-100
novel_pir981	267	0	9.09	0.001	-13.15006458	Down	2.18E-100	9.42E-100
novel_pir1890	267	0	9.09	0.001	-13.15006458	Down	2.18E-100	9.40E-100
novel_pir1500	267	0	9.09	0.001	-13.15006458	Down	2.18E-100	9.38E-100
novel_pir1787	266	0	9.06	0.001	-13.14529533	Down	5.15E-100	2.21E-99
novel_pir48	266	0	9.06	0.001	-13.14529533	Down	5.15E-100	2.20E-99
novel_pir408	265	0	9.02	0.001	-13.13891172	Down	1.22E-99	5.18E-99
novel_pir1883	264	0	8.99	0.001	-13.1341054	Down	2.87E-99	1.22E-98
novel_pir1429	264	0	8.99	0.001	-13.1341054	Down	2.87E-99	1.22E-98
novel_pir9	263	0	8.96	0.001	-13.12928302	Down	6.78E-99	2.86E-98

novel_pir1681	263	0	8.96	0.001	-13.12928302	Down	6.78E-99	2.85E-98
novel_pir1992	262	0	8.92	0.001	-13.12282799	Down	1.60E-98	6.72E-98
novel_pir2243	262	0	8.92	0.001	-13.12282799	Down	1.60E-98	6.70E-98
novel_pir1153	261	0	8.89	0.001	-13.1179677	Down	3.78E-98	1.58E-97
novel_pir2216	261	0	8.89	0.001	-13.1179677	Down	3.78E-98	1.58E-97
novel_pir1848	261	0	8.89	0.001	-13.1179677	Down	3.78E-98	1.57E-97
novel_pir971	261	0	8.89	0.001	-13.1179677	Down	3.78E-98	1.57E-97
novel_pir1829	261	0	8.89	0.001	-13.1179677	Down	3.78E-98	1.56E-97
novel_pir2078	261	0	8.89	0.001	-13.1179677	Down	3.78E-98	1.56E-97
novel_pir1845	261	0	8.89	0.001	-13.1179677	Down	3.78E-98	1.56E-97
novel_pir293	260	0	8.85	0.001	-13.11146174	Down	8.91E-98	3.67E-97
novel_pir1391	259	0	8.82	0.001	-13.10656294	Down	2.10E-97	8.64E-97
novel_pir2305	259	0	8.82	0.001	-13.10656294	Down	2.10E-97	8.62E-97
novel_pir1105	259	0	8.82	0.001	-13.10656294	Down	2.10E-97	8.61E-97
novel_pir1425	259	0	8.82	0.001	-13.10656294	Down	2.10E-97	8.59E-97
novel_pir801	259	0	8.82	0.001	-13.10656294	Down	2.10E-97	8.57E-97
novel_pir2302	259	0	8.82	0.001	-13.10656294	Down	2.10E-97	8.55E-97
novel_pir1009	258	0	8.79	0.001	-13.10164745	Down	4.97E-97	2.01E-96
novel_pir1209	257	0	8.75	0.001	-13.0950673	Down	1.17E-96	4.74E-96
novel_pir892	257	0	8.75	0.001	-13.0950673	Down	1.17E-96	4.73E-96
novel_pir1310	257	0	8.75	0.001	-13.0950673	Down	1.17E-96	4.72E-96
novel_pir1041	257	0	8.75	0.001	-13.0950673	Down	1.17E-96	4.71E-96
novel_pir1348	256	0	8.72	0.001	-13.09011242	Down	2.77E-96	1.11E-95
novel_pir1822	256	0	8.72	0.001	-13.09011242	Down	2.77E-96	1.11E-95
novel_pir1339	256	0	8.72	0.001	-13.09011242	Down	2.77E-96	1.11E-95
novel_pir1753	255	0	8.68	0.001	-13.08347933	Down	6.53E-96	2.60E-95
novel_pir2212	255	0	8.68	0.001	-13.08347933	Down	6.53E-96	2.60E-95
novel_pir1126	254	0	8.65	0.001	-13.07848442	Down	1.54E-95	6.12E-95
novel_pir1882	253	0	8.62	0.001	-13.07347215	Down	3.64E-95	1.44E-94
novel_pir947	253	0	8.62	0.001	-13.07347215	Down	3.64E-95	1.44E-94
novel_pir1420	252	0	8.58	0.001	-13.06676193	Down	8.59E-95	3.39E-94
novel_pir1096	251	0	8.55	0.001	-13.0617087	Down	2.03E-94	7.98E-94
novel_pir122	251	0	8.55	0.001	-13.0617087	Down	2.03E-94	7.96E-94
novel_pir8	251	0	8.55	0.001	-13.0617087	Down	2.03E-94	7.95E-94
novel_pir1035	250	0	8.51	0.001	-13.05494342	Down	4.78E-94	1.87E-93
novel_pir1534	250	0	8.51	0.001	-13.05494342	Down	4.78E-94	1.87E-93

novel_pir584	250	0	8.51	0.001	-13.05494342	Down	4.78E-94	1.86E-93
novel_pir2253	249	0	8.48	0.001	-13.04984855	Down	1.13E-93	4.39E-93
novel_pir2229	249	0	8.48	0.001	-13.04984855	Down	1.13E-93	4.38E-93
novel_pir1248	249	0	8.48	0.001	-13.04984855	Down	1.13E-93	4.37E-93
novel_pir855	249	0	8.48	0.001	-13.04984855	Down	1.13E-93	4.36E-93
novel_pir2118	249	0	8.48	0.001	-13.04984855	Down	1.13E-93	4.36E-93
novel_pir1095	248	0	8.45	0.001	-13.04473563	Down	2.67E-93	1.03E-92
novel_pir135	248	0	8.45	0.001	-13.04473563	Down	2.67E-93	1.02E-92
novel_pir1128	248	0	8.45	0.001	-13.04473563	Down	2.67E-93	1.02E-92
novel_pir1800	248	0	8.45	0.001	-13.04473563	Down	2.67E-93	1.02E-92
novel_pir2372	247	0	8.41	0.001	-13.03789009	Down	6.29E-93	2.40E-92
novel_pir2111	247	0	8.41	0.001	-13.03789009	Down	6.29E-93	2.39E-92
novel_pir534	247	0	8.41	0.001	-13.03789009	Down	6.29E-93	2.39E-92
novel_pir1514	246	0	8.38	0.001	-13.03273453	Down	1.49E-92	5.62E-92
novel_pir1384	246	0	8.38	0.001	-13.03273453	Down	1.49E-92	5.61E-92
novel_pir1108	246	0	8.38	0.001	-13.03273453	Down	1.49E-92	5.60E-92
novel_pir444	244	0	8.31	0.001	-13.02063276	Down	8.28E-92	3.12E-91
novel_pir1098	243	0	8.28	0.001	-13.01541505	Down	1.95E-91	7.34E-91
novel_pir467	243	0	8.28	0.001	-13.01541505	Down	1.95E-91	7.32E-91
novel_pir1519	243	0	8.28	0.001	-13.01541505	Down	1.95E-91	7.31E-91
novel_pir12	243	0	8.28	0.001	-13.01541505	Down	1.95E-91	7.29E-91
novel_pir2165	243	0	8.28	0.001	-13.01541505	Down	1.95E-91	7.28E-91
novel_pir1092	242	0	8.24	0.001	-13.00842862	Down	4.61E-91	1.72E-90
novel_pir1662	241	0	8.21	0.001	-13.00316651	Down	1.09E-90	4.04E-90
novel_pir1416	241	0	8.21	0.001	-13.00316651	Down	1.09E-90	4.03E-90
novel_pir65	240	0	8.17	0.001	-12.99612036	Down	2.57E-90	9.50E-90
novel_pir1660	239	0	8.14	0.001	-12.99081308	Down	6.06E-90	2.24E-89
novel_pir1393	239	0	8.14	0.001	-12.99081308	Down	6.06E-90	2.23E-89
novel_pir1592	239	0	8.14	0.001	-12.99081308	Down	6.06E-90	2.23E-89
novel_pir1706	238	0	8.11	0.001	-12.9854862	Down	1.43E-89	5.24E-89
novel_pir590	238	0	8.11	0.001	-12.9854862	Down	1.43E-89	5.23E-89
novel_pir1770	238	0	8.11	0.001	-12.9854862	Down	1.43E-89	5.22E-89
novel_pir1462	238	0	8.11	0.001	-12.9854862	Down	1.43E-89	5.21E-89
novel_pir1650	238	0	8.11	0.001	-12.9854862	Down	1.43E-89	5.20E-89
novel_pir1723	237	0	8.07	0.001	-12.97835296	Down	3.38E-89	1.23E-88
novel_pir817	237	0	8.07	0.001	-12.97835296	Down	3.38E-89	1.22E-88

novel_pir821	237	0	8.07	0.001	-12.97835296	Down	3.38E-89	1.22E-88
novel_pir559	236	0	8.04	0.001	-12.97297979	Down	7.98E-89	2.88E-88
novel_pir2084	235	0	8	0.001	-12.96578428	Down	1.88E-88	6.77E-88
novel_pir464	235	0	8	0.001	-12.96578428	Down	1.88E-88	6.76E-88
novel_pir1430	235	0	8	0.001	-12.96578428	Down	1.88E-88	6.75E-88
novel_pir1019	234	0	7.97	0.001	-12.96036401	Down	4.44E-88	1.59E-87
novel_pir1978	233	0	7.93	0.001	-12.95310515	Down	1.05E-87	3.75E-87
novel_pir639	233	0	7.93	0.001	-12.95310515	Down	1.05E-87	3.74E-87
novel_pir658	233	0	7.93	0.001	-12.95310515	Down	1.05E-87	3.73E-87
novel_pir2236	233	0	7.93	0.001	-12.95310515	Down	1.05E-87	3.72E-87
novel_pir246	232	0	7.9	0.001	-12.94763694	Down	2.48E-87	8.78E-87
novel_pir2283	232	0	7.9	0.001	-12.94763694	Down	2.48E-87	8.76E-87
novel_pir576	232	0	7.9	0.001	-12.94763694	Down	2.48E-87	8.74E-87
novel_pir915	231	0	7.87	0.001	-12.94214792	Down	5.84E-87	2.06E-86
novel_pir859	231	0	7.87	0.001	-12.94214792	Down	5.84E-87	2.05E-86
novel_pir1533	230	0	7.83	0.001	-12.93479659	Down	1.38E-86	4.83E-86
novel_pir549	230	0	7.83	0.001	-12.93479659	Down	1.38E-86	4.83E-86
novel_pir249	229	0	7.8	0.001	-12.92925841	Down	3.26E-86	1.14E-85
novel_pir250	229	0	7.8	0.001	-12.92925841	Down	3.26E-86	1.13E-85
novel_pir822	229	0	7.8	0.001	-12.92925841	Down	3.26E-86	1.13E-85
novel_pir109	229	0	7.8	0.001	-12.92925841	Down	3.26E-86	1.13E-85
novel_pir496	229	0	7.8	0.001	-12.92925841	Down	3.26E-86	1.13E-85
novel_pir629	228	0	7.76	0.001	-12.92184094	Down	7.69E-86	2.66E-85
novel_pir327	228	0	7.76	0.001	-12.92184094	Down	7.69E-86	2.65E-85
novel_pir1870	228	0	7.76	0.001	-12.92184094	Down	7.69E-86	2.65E-85
novel_pir1060	228	0	7.76	0.001	-12.92184094	Down	7.69E-86	2.64E-85
novel_pir1593	227	0	7.73	0.001	-12.9162527	Down	1.81E-85	6.23E-85
novel_pir607	227	0	7.73	0.001	-12.9162527	Down	1.81E-85	6.22E-85
novel_pir1558	226	0	7.7	0.001	-12.91064273	Down	4.28E-85	1.47E-84
novel_pir46	226	0	7.7	0.001	-12.91064273	Down	4.28E-85	1.46E-84
novel_pir55	225	0	7.66	0.001	-12.90312868	Down	1.01E-84	3.45E-84
novel_pir835	225	0	7.66	0.001	-12.90312868	Down	1.01E-84	3.44E-84
novel_pir1793	225	0	7.66	0.001	-12.90312868	Down	1.01E-84	3.43E-84
novel_pir2144	224	0	7.63	0.001	-12.89746734	Down	2.39E-84	8.08E-84
novel_pir729	224	0	7.63	0.001	-12.89746734	Down	2.39E-84	8.06E-84
novel_pir35	224	0	7.63	0.001	-12.89746734	Down	2.39E-84	8.05E-84

novel_pir1312	223	0	7.59	0.001	-12.88988417	Down	5.63E-84	1.90E-83
novel_pir1278	222	0	7.56	0.001	-12.88417052	Down	1.33E-83	4.47E-83
novel_pir1259	222	0	7.56	0.001	-12.88417052	Down	1.33E-83	4.46E-83
novel_pir941	222	0	7.56	0.001	-12.88417052	Down	1.33E-83	4.45E-83
novel_pir2469	222	0	7.56	0.001	-12.88417052	Down	1.33E-83	4.44E-83
novel_pir1468	221	0	7.53	0.001	-12.87843415	Down	3.14E-83	1.05E-82
novel_pir1293	221	0	7.53	0.001	-12.87843415	Down	3.14E-83	1.04E-82
novel_pir2239	221	0	7.53	0.001	-12.87843415	Down	3.14E-83	1.04E-82
novel_pir919	221	0	7.53	0.001	-12.87843415	Down	3.14E-83	1.04E-82
novel_pir818	221	0	7.53	0.001	-12.87843415	Down	3.14E-83	1.04E-82
novel_pir11	220	0	7.49	0.001	-12.87075	Down	7.41E-83	2.45E-82
novel_pir488	220	0	7.49	0.001	-12.87075	Down	7.41E-83	2.44E-82
novel_pir689	220	0	7.49	0.001	-12.87075	Down	7.41E-83	2.44E-82
novel_pir459	219	0	7.46	0.001	-12.86495992	Down	1.75E-82	5.74E-82
novel_pir2126	219	0	7.46	0.001	-12.86495992	Down	1.75E-82	5.73E-82
novel_pir1722	219	0	7.46	0.001	-12.86495992	Down	1.75E-82	5.72E-82
novel_pir2225	219	0	7.46	0.001	-12.86495992	Down	1.75E-82	5.71E-82
novel_pir1418	218	0	7.42	0.001	-12.85720347	Down	4.13E-82	1.35E-81
novel_pir1146	218	0	7.42	0.001	-12.85720347	Down	4.13E-82	1.34E-81
novel_pir213	217	0	7.39	0.001	-12.85135865	Down	9.74E-82	3.17E-81
novel_pir100	217	0	7.39	0.001	-12.85135865	Down	9.74E-82	3.16E-81
novel_pir477	216	0	7.36	0.001	-12.84549005	Down	2.30E-81	7.45E-81
novel_pir1239	216	0	7.36	0.001	-12.84549005	Down	2.30E-81	7.44E-81
novel_pir2081	216	0	7.36	0.001	-12.84549005	Down	2.30E-81	7.42E-81
novel_pir465	216	0	7.36	0.001	-12.84549005	Down	2.30E-81	7.41E-81
novel_pir1328	216	0	7.36	0.001	-12.84549005	Down	2.30E-81	7.40E-81
novel_pir2347	215	0	7.32	0.001	-12.83762793	Down	5.43E-81	1.74E-80
novel_pir1047	215	0	7.32	0.001	-12.83762793	Down	5.43E-81	1.74E-80
novel_pir404	215	0	7.32	0.001	-12.83762793	Down	5.43E-81	1.73E-80
novel_pir1930	214	0	7.29	0.001	-12.8317031	Down	1.28E-80	4.09E-80
novel_pir139	213	0	7.25	0.001	-12.82376528	Down	3.02E-80	9.63E-80
novel_pir2148	213	0	7.25	0.001	-12.82376528	Down	3.02E-80	9.61E-80
novel_pir2298	212	0	7.22	0.001	-12.81778312	Down	7.14E-80	2.27E-79
novel_pir735	212	0	7.22	0.001	-12.81778312	Down	7.14E-80	2.26E-79
novel_pir1710	210	0	7.15	0.001	-12.80372753	Down	3.98E-79	1.26E-78
novel_pir104	209	0	7.12	0.001	-12.79766153	Down	9.39E-79	2.96E-78

novel_pir1423	209	0	7.12	0.001	-12.79766153	Down	9.39E-79	2.96E-78
novel_pir2183	208	0	7.08	0.001	-12.78953364	Down	2.22E-78	6.97E-78
novel_pir589	207	0	7.05	0.001	-12.78340754	Down	5.23E-78	1.64E-77
novel_pir2222	207	0	7.05	0.001	-12.78340754	Down	5.23E-78	1.64E-77
novel_pir1901	206	0	7.02	0.001	-12.77725532	Down	1.23E-77	3.87E-77
novel_pir2161	206	0	7.02	0.001	-12.77725532	Down	1.23E-77	3.86E-77
novel_pir1754	204	0	6.95	0.001	-12.76279726	Down	6.88E-77	2.14E-76
novel_pir1295	203	0	6.91	0.001	-12.75447	Down	1.62E-76	5.05E-76
novel_pir683	203	0	6.91	0.001	-12.75447	Down	1.62E-76	5.04E-76
novel_pir1094	203	0	6.91	0.001	-12.75447	Down	1.62E-76	5.03E-76
novel_pir1674	202	0	6.88	0.001	-12.74819285	Down	3.83E-76	1.19E-75
novel_pir723	202	0	6.88	0.001	-12.74819285	Down	3.83E-76	1.18E-75
novel_pir509	202	0	6.88	0.001	-12.74819285	Down	3.83E-76	1.18E-75
novel_pir455	202	0	6.88	0.001	-12.74819285	Down	3.83E-76	1.18E-75
novel_pir54	201	0	6.85	0.001	-12.74188827	Down	9.05E-76	2.78E-75
novel_pir1243	201	0	6.85	0.001	-12.74188827	Down	9.05E-76	2.78E-75
novel_pir948	200	0	6.81	0.001	-12.73343908	Down	2.14E-75	6.53E-75
novel_pir2114	200	0	6.81	0.001	-12.73343908	Down	2.14E-75	6.52E-75
novel_pir1187	200	0	6.81	0.001	-12.73343908	Down	2.14E-75	6.51E-75
novel_pir1897	199	0	6.78	0.001	-12.72706956	Down	5.04E-75	1.54E-74
novel_pir1251	199	0	6.78	0.001	-12.72706956	Down	5.04E-75	1.53E-74
novel_pir2352	199	0	6.78	0.001	-12.72706956	Down	5.04E-75	1.53E-74
novel_pir672	198	0	6.74	0.001	-12.71853288	Down	1.19E-74	3.61E-74
novel_pir891	198	0	6.74	0.001	-12.71853288	Down	1.19E-74	3.60E-74
novel_pir705	198	0	6.74	0.001	-12.71853288	Down	1.19E-74	3.59E-74
novel_pir258	197	0	6.71	0.001	-12.71209705	Down	2.81E-74	8.47E-74
novel_pir1117	196	0	6.67	0.001	-12.70347105	Down	6.63E-74	2.00E-73
novel_pir1150	195	0	6.64	0.001	-12.69696753	Down	1.56E-73	4.70E-73
novel_pir1761	195	0	6.64	0.001	-12.69696753	Down	1.56E-73	4.70E-73
novel_pir2267	195	0	6.64	0.001	-12.69696753	Down	1.56E-73	4.69E-73
novel_pir2307	194	0	6.61	0.001	-12.69043456	Down	3.69E-73	1.11E-72
novel_pir2009	194	0	6.61	0.001	-12.69043456	Down	3.69E-73	1.10E-72
novel_pir909	194	0	6.61	0.001	-12.69043456	Down	3.69E-73	1.10E-72
novel_pir1589	193	0	6.57	0.001	-12.68167766	Down	8.72E-73	2.60E-72
novel_pir1625	193	0	6.57	0.001	-12.68167766	Down	8.72E-73	2.59E-72
novel_pir286	193	0	6.57	0.001	-12.68167766	Down	8.72E-73	2.59E-72

novel_pir745	193	0	6.57	0.001	-12.68167766	Down	8.72E-73	2.58E-72
novel_pir2102	192	0	6.54	0.001	-12.67507492	Down	2.06E-72	6.09E-72
novel_pir952	191	0	6.5	0.001	-12.666224	Down	4.86E-72	1.44E-71
novel_pir2313	191	0	6.5	0.001	-12.666224	Down	4.86E-72	1.43E-71
novel_pir1513	191	0	6.5	0.001	-12.666224	Down	4.86E-72	1.43E-71
novel_pir831	190	0	6.47	0.001	-12.65955	Down	1.15E-71	3.37E-71
novel_pir762	190	0	6.47	0.001	-12.65955	Down	1.15E-71	3.37E-71
novel_pir1553	190	0	6.47	0.001	-12.65955	Down	1.15E-71	3.36E-71
novel_pir838	189	0	6.44	0.001	-12.65284497	Down	2.71E-71	7.92E-71
novel_pir1342	188	0	6.4	0.001	-12.64385619	Down	6.39E-71	1.86E-70
novel_pir2021	188	0	6.4	0.001	-12.64385619	Down	6.39E-71	1.86E-70
novel_pir868	188	0	6.4	0.001	-12.64385619	Down	6.39E-71	1.86E-70
novel_pir953	187	0	6.37	0.001	-12.63707766	Down	1.51E-70	4.38E-70
novel_pir734	187	0	6.37	0.001	-12.63707766	Down	1.51E-70	4.37E-70
novel_pir1163	187	0	6.37	0.001	-12.63707766	Down	1.51E-70	4.37E-70
novel_pir1362	187	0	6.37	0.001	-12.63707766	Down	1.51E-70	4.36E-70
novel_pir1925	187	0	6.37	0.001	-12.63707766	Down	1.51E-70	4.35E-70
novel_pir1505	186	0	6.33	0.001	-12.62798978	Down	3.56E-70	1.03E-69
novel_pir1560	186	0	6.33	0.001	-12.62798978	Down	3.56E-70	1.02E-69
novel_pir280	185	0	6.3	0.001	-12.62113611	Down	8.40E-70	2.41E-69
novel_pir643	184	0	6.27	0.001	-12.61424973	Down	1.98E-69	5.69E-69
novel_pir2249	183	0	6.23	0.001	-12.60501645	Down	4.68E-69	1.34E-68
novel_pir2377	183	0	6.23	0.001	-12.60501645	Down	4.68E-69	1.34E-68
novel_pir1966	183	0	6.23	0.001	-12.60501645	Down	4.68E-69	1.34E-68
novel_pir1286	182	0	6.2	0.001	-12.5980525	Down	1.11E-68	3.15E-68
novel_pir2290	182	0	6.2	0.001	-12.5980525	Down	1.11E-68	3.15E-68
novel_pir1658	182	0	6.2	0.001	-12.5980525	Down	1.11E-68	3.14E-68
novel_pir323	182	0	6.2	0.001	-12.5980525	Down	1.11E-68	3.14E-68
novel_pir1315	181	0	6.16	0.001	-12.58871464	Down	2.61E-68	7.39E-68
novel_pir1473	181	0	6.16	0.001	-12.58871464	Down	2.61E-68	7.38E-68
novel_pir1622	178	0	6.06	0.001	-12.56510208	Down	3.43E-67	9.69E-67
novel_pir1529	178	0	6.06	0.001	-12.56510208	Down	3.43E-67	9.68E-67
novel_pir1470	178	0	6.06	0.001	-12.56510208	Down	3.43E-67	9.66E-67
novel_pir2458	178	0	6.06	0.001	-12.56510208	Down	3.43E-67	9.65E-67
novel_pir1119	178	0	6.06	0.001	-12.56510208	Down	3.43E-67	9.64E-67
novel_pir1419	178	0	6.06	0.001	-12.56510208	Down	3.43E-67	9.62E-67

novel_pir1607	178	0	6.06	0.001	-12.56510208	Down	3.43E-67	9.61E-67
novel_pir874	178	0	6.06	0.001	-12.56510208	Down	3.43E-67	9.59E-67
novel_pir1048	177	0	6.03	0.001	-12.55794229	Down	8.10E-67	2.26E-66
novel_pir1577	176	0	5.99	0.001	-12.54834029	Down	1.91E-66	5.33E-66
novel_pir1799	176	0	5.99	0.001	-12.54834029	Down	1.91E-66	5.32E-66
novel_pir2442	175	0	5.96	0.001	-12.54109662	Down	4.51E-66	1.25E-65
novel_pir1537	175	0	5.96	0.001	-12.54109662	Down	4.51E-66	1.25E-65
novel_pir248	175	0	5.96	0.001	-12.54109662	Down	4.51E-66	1.25E-65
novel_pir1692	174	0	5.93	0.001	-12.53381639	Down	1.06E-65	2.94E-65
novel_pir970	174	0	5.93	0.001	-12.53381639	Down	1.06E-65	2.93E-65
novel_pir2186	172	0	5.86	0.001	-12.51668495	Down	5.93E-65	1.63E-64
novel_pir1321	172	0	5.86	0.001	-12.51668495	Down	5.93E-65	1.63E-64
novel_pir1018	172	0	5.86	0.001	-12.51668495	Down	5.93E-65	1.63E-64
novel_pir1783	172	0	5.86	0.001	-12.51668495	Down	5.93E-65	1.63E-64
novel_pir2454	171	0	5.82	0.001	-12.50680344	Down	1.40E-64	3.83E-64
novel_pir110	171	0	5.82	0.001	-12.50680344	Down	1.40E-64	3.83E-64
novel_pir2343	170	0	5.79	0.001	-12.49934763	Down	3.31E-64	9.02E-64
novel_pir1270	170	0	5.79	0.001	-12.49934763	Down	3.31E-64	9.00E-64
novel_pir742	170	0	5.79	0.001	-12.49934763	Down	3.31E-64	8.99E-64
novel_pir1802	170	0	5.79	0.001	-12.49934763	Down	3.31E-64	8.98E-64
novel_pir2274	167	0	5.69	0.001	-12.47421294	Down	4.35E-63	1.18E-62
novel_pir979	167	0	5.69	0.001	-12.47421294	Down	4.35E-63	1.18E-62
novel_pir2285	167	0	5.69	0.001	-12.47421294	Down	4.35E-63	1.18E-62
novel_pir918	167	0	5.69	0.001	-12.47421294	Down	4.35E-63	1.17E-62
novel_pir2180	167	0	5.69	0.001	-12.47421294	Down	4.35E-63	1.17E-62
novel_pir1758	165	0	5.62	0.001	-12.45635442	Down	2.42E-62	6.51E-62
novel_pir1806	165	0	5.62	0.001	-12.45635442	Down	2.42E-62	6.50E-62
novel_pir1906	164	0	5.59	0.001	-12.44863257	Down	5.72E-62	1.53E-61
novel_pir2355	164	0	5.59	0.001	-12.44863257	Down	5.72E-62	1.53E-61
novel_pir2045	164	0	5.59	0.001	-12.44863257	Down	5.72E-62	1.53E-61
novel_pir1661	164	0	5.59	0.001	-12.44863257	Down	5.72E-62	1.53E-61
novel_pir1877	163	0	5.55	0.001	-12.43827206	Down	1.35E-61	3.60E-61
novel_pir712	163	0	5.55	0.001	-12.43827206	Down	1.35E-61	3.59E-61
novel_pir1873	163	0	5.55	0.001	-12.43827206	Down	1.35E-61	3.59E-61
novel_pir1948	162	0	5.52	0.001	-12.43045255	Down	3.19E-61	8.46E-61
novel_pir1125	161	0	5.48	0.001	-12.41996018	Down	7.52E-61	1.99E-60

novel_pir1872	157	0	5.35	0.001	-12.38532318	Down	2.33E-59	6.18E-59
novel_pir1675	157	0	5.35	0.001	-12.38532318	Down	2.33E-59	6.17E-59
novel_pir1525	157	0	5.35	0.001	-12.38532318	Down	2.33E-59	6.16E-59
novel_pir29	157	0	5.35	0.001	-12.38532318	Down	2.33E-59	6.15E-59
novel_pir828	157	0	5.35	0.001	-12.38532318	Down	2.33E-59	6.14E-59
novel_pir1860	156	0	5.31	0.001	-12.37449615	Down	5.51E-59	1.45E-58
novel_pir1719	155	0	5.28	0.001	-12.36632221	Down	1.30E-58	3.41E-58
novel_pir2190	155	0	5.28	0.001	-12.36632221	Down	1.30E-58	3.41E-58
novel_pir2116	154	0	5.24	0.001	-12.3553511	Down	3.07E-58	8.04E-58
novel_pir1708	154	0	5.24	0.001	-12.3553511	Down	3.07E-58	8.03E-58
novel_pir1169	153	0	5.21	0.001	-12.34706766	Down	7.25E-58	1.89E-57
novel_pir1089	153	0	5.21	0.001	-12.34706766	Down	7.25E-58	1.89E-57
novel_pir301	152	0	5.18	0.001	-12.33873638	Down	1.71E-57	4.44E-57
novel_pir1784	151	0	5.14	0.001	-12.32755264	Down	4.04E-57	1.05E-56
novel_pir814	150	0	5.11	0.001	-12.31910758	Down	9.53E-57	2.46E-56
novel_pir1178	149	0	5.07	0.001	-12.30777003	Down	2.25E-56	5.81E-56
novel_pir2430	149	0	5.07	0.001	-12.30777003	Down	2.25E-56	5.80E-56
novel_pir657	149	0	5.07	0.001	-12.30777003	Down	2.25E-56	5.79E-56
novel_pir794	148	0	5.04	0.001	-12.29920802	Down	5.31E-56	1.37E-55
novel_pir929	148	0	5.04	0.001	-12.29920802	Down	5.31E-56	1.36E-55
novel_pir916	147	0	5.01	0.001	-12.29059489	Down	1.25E-55	3.21E-55
novel_pir310	147	0	5.01	0.001	-12.29059489	Down	1.25E-55	3.21E-55
novel_pir1487	146	0	4.97	0.001	-12.27903014	Down	2.96E-55	7.57E-55
novel_pir424	145	0	4.94	0.001	-12.27029533	Down	6.98E-55	1.78E-54
novel_pir732	145	0	4.94	0.001	-12.27029533	Down	6.98E-55	1.78E-54
novel_pir1698	145	0	4.94	0.001	-12.27029533	Down	6.98E-55	1.78E-54
novel_pir236	145	0	4.94	0.001	-12.27029533	Down	6.98E-55	1.78E-54
novel_pir744	144	0	4.9	0.001	-12.25856603	Down	1.65E-54	4.19E-54
novel_pir614	143	0	4.87	0.001	-12.24970606	Down	3.89E-54	9.86E-54
novel_pir1773	143	0	4.87	0.001	-12.24970606	Down	3.89E-54	9.84E-54
novel_pir1122	143	0	4.87	0.001	-12.24970606	Down	3.89E-54	9.83E-54
novel_pir1372	142	0	4.84	0.001	-12.24079133	Down	9.18E-54	2.32E-53
novel_pir1969	142	0	4.84	0.001	-12.24079133	Down	9.18E-54	2.31E-53
novel_pir2167	142	0	4.84	0.001	-12.24079133	Down	9.18E-54	2.31E-53
novel_pir1947	141	0	4.8	0.001	-12.22881869	Down	2.17E-53	5.45E-53
novel_pir2465	141	0	4.8	0.001	-12.22881869	Down	2.17E-53	5.44E-53

novel_pir2117	141	0	4.8	0.001	-12.22881869	Down	2.17E-53	5.43E-53
novel_pir394	140	0	4.77	0.001	-12.21977355	Down	5.12E-53	1.28E-52
novel_pir1093	140	0	4.77	0.001	-12.21977355	Down	5.12E-53	1.28E-52
novel_pir1809	140	0	4.77	0.001	-12.21977355	Down	5.12E-53	1.28E-52
novel_pir935	138	0	4.7	0.001	-12.19844504	Down	2.85E-52	7.10E-52
novel_pir593	138	0	4.7	0.001	-12.19844504	Down	2.85E-52	7.09E-52
novel_pir844	138	0	4.7	0.001	-12.19844504	Down	2.85E-52	7.08E-52
novel_pir2025	138	0	4.7	0.001	-12.19844504	Down	2.85E-52	7.07E-52
novel_pir2061	137	0	4.67	0.001	-12.18920683	Down	6.73E-52	1.67E-51
novel_pir798	137	0	4.67	0.001	-12.18920683	Down	6.73E-52	1.66E-51
novel_pir1240	137	0	4.67	0.001	-12.18920683	Down	6.73E-52	1.66E-51
novel_pir585	136	0	4.63	0.001	-12.17679648	Down	1.59E-51	3.91E-51
novel_pir1797	136	0	4.63	0.001	-12.17679648	Down	1.59E-51	3.91E-51
novel_pir2350	136	0	4.63	0.001	-12.17679648	Down	1.59E-51	3.90E-51
novel_pir1242	135	0	4.6	0.001	-12.16741815	Down	3.75E-51	9.20E-51
novel_pir1991	135	0	4.6	0.001	-12.16741815	Down	3.75E-51	9.19E-51
novel_pir2238	135	0	4.6	0.001	-12.16741815	Down	3.75E-51	9.18E-51
novel_pir1171	134	0	4.56	0.001	-12.15481811	Down	8.85E-51	2.16E-50
novel_pir925	134	0	4.56	0.001	-12.15481811	Down	8.85E-51	2.16E-50
novel_pir2150	133	0	4.53	0.001	-12.14529533	Down	2.09E-50	5.09E-50
novel_pir266	133	0	4.53	0.001	-12.14529533	Down	2.09E-50	5.09E-50
novel_pir64	132	0	4.5	0.001	-12.13570929	Down	4.93E-50	1.20E-49
novel_pir2213	132	0	4.5	0.001	-12.13570929	Down	4.93E-50	1.20E-49
novel_pir318	132	0	4.5	0.001	-12.13570929	Down	4.93E-50	1.20E-49
novel_pir994	132	0	4.5	0.001	-12.13570929	Down	4.93E-50	1.19E-49
novel_pir1612	131	0	4.46	0.001	-12.12282799	Down	1.16E-49	2.81E-49
novel_pir1790	131	0	4.46	0.001	-12.12282799	Down	1.16E-49	2.81E-49
novel_pir1138	131	0	4.46	0.001	-12.12282799	Down	1.16E-49	2.81E-49
novel_pir1472	130	0	4.43	0.001	-12.11309098	Down	2.75E-49	6.61E-49
novel_pir827	130	0	4.43	0.001	-12.11309098	Down	2.75E-49	6.60E-49
novel_pir2141	128	0	4.36	0.001	-12.09011242	Down	1.53E-48	3.68E-48
novel_pir879	128	0	4.36	0.001	-12.09011242	Down	1.53E-48	3.67E-48
novel_pir1683	127	0	4.33	0.001	-12.08015131	Down	3.61E-48	8.65E-48
novel_pir1147	127	0	4.33	0.001	-12.08015131	Down	3.61E-48	8.64E-48
novel_pir1212	126	0	4.29	0.001	-12.06676193	Down	8.53E-48	2.04E-47
novel_pir2145	125	0	4.26	0.001	-12.05663772	Down	2.01E-47	4.80E-47

novel_pir2284	125	0	4.26	0.001	-12.05663772	Down	2.01E-47	4.80E-47
novel_pir1399	124	0	4.22	0.001	-12.04302728	Down	4.75E-47	1.13E-46
novel_pir500	124	0	4.22	0.001	-12.04302728	Down	4.75E-47	1.13E-46
novel_pir2146	124	0	4.22	0.001	-12.04302728	Down	4.75E-47	1.13E-46
novel_pir1211	124	0	4.22	0.001	-12.04302728	Down	4.75E-47	1.13E-46
novel_pir2125	123	0	4.19	0.001	-12.03273453	Down	1.12E-46	2.66E-46
novel_pir2341	123	0	4.19	0.001	-12.03273453	Down	1.12E-46	2.65E-46
novel_pir2344	122	0	4.15	0.001	-12.01889562	Down	2.65E-46	6.25E-46
novel_pir405	122	0	4.15	0.001	-12.01889562	Down	2.65E-46	6.24E-46
novel_pir1182	122	0	4.15	0.001	-12.01889562	Down	2.65E-46	6.23E-46
novel_pir846	121	0	4.12	0.001	-12.00842862	Down	6.25E-46	1.47E-45
novel_pir1536	120	0	4.09	0.001	-11.99788513	Down	1.48E-45	3.46E-45
novel_pir34	120	0	4.09	0.001	-11.99788513	Down	1.48E-45	3.45E-45
novel_pir644	119	0	4.05	0.001	-11.98370619	Down	3.48E-45	8.14E-45
novel_pir2475	119	0	4.05	0.001	-11.98370619	Down	3.48E-45	8.13E-45
novel_pir1725	119	0	4.05	0.001	-11.98370619	Down	3.48E-45	8.12E-45
novel_pir543	119	0	4.05	0.001	-11.98370619	Down	3.48E-45	8.11E-45
novel_pir1076	118	0	4.02	0.001	-11.97297979	Down	8.22E-45	1.91E-44
novel_pir2246	118	0	4.02	0.001	-11.97297979	Down	8.22E-45	1.91E-44
novel_pir1811	118	0	4.02	0.001	-11.97297979	Down	8.22E-45	1.91E-44
novel_pir1530	118	0	4.02	0.001	-11.97297979	Down	8.22E-45	1.90E-44
novel_pir2203	117	0	3.98	0.001	-11.95855272	Down	1.94E-44	4.49E-44
novel_pir1143	117	0	3.98	0.001	-11.95855272	Down	1.94E-44	4.48E-44
novel_pir1361	117	0	3.98	0.001	-11.95855272	Down	1.94E-44	4.48E-44
novel_pir987	117	0	3.98	0.001	-11.95855272	Down	1.94E-44	4.47E-44
novel_pir72	116	0	3.95	0.001	-11.94763694	Down	4.58E-44	1.05E-43
novel_pir1554	115	0	3.92	0.001	-11.93663794	Down	1.08E-43	2.48E-43
novel_pir803	114	0	3.88	0.001	-11.92184094	Down	2.55E-43	5.84E-43
novel_pir2242	113	0	3.85	0.001	-11.91064273	Down	6.02E-43	1.38E-42
novel_pir2320	113	0	3.85	0.001	-11.91064273	Down	6.02E-43	1.38E-42
novel_pir472	113	0	3.85	0.001	-11.91064273	Down	6.02E-43	1.37E-42
novel_pir284	113	0	3.85	0.001	-11.91064273	Down	6.02E-43	1.37E-42
novel_pir1228	112	0	3.81	0.001	-11.89557528	Down	1.42E-42	3.24E-42
novel_pir355	112	0	3.81	0.001	-11.89557528	Down	1.42E-42	3.23E-42
novel_pir2445	112	0	3.81	0.001	-11.89557528	Down	1.42E-42	3.23E-42
novel_pir1071	111	0	3.78	0.001	-11.88417052	Down	3.36E-42	7.61E-42

novel_pir1049	110	0	3.75	0.001	-11.87267488	Down	7.92E-42	1.79E-41
novel_pir903	110	0	3.75	0.001	-11.87267488	Down	7.92E-42	1.79E-41
novel_pir1202	110	0	3.75	0.001	-11.87267488	Down	7.92E-42	1.79E-41
novel_pir1768	109	0	3.71	0.001	-11.85720347	Down	1.87E-41	4.22E-41
novel_pir1981	108	0	3.68	0.001	-11.84549005	Down	4.41E-41	9.95E-41
novel_pir771	107	0	3.64	0.001	-11.82972274	Down	1.04E-40	2.35E-40
novel_pir1796	107	0	3.64	0.001	-11.82972274	Down	1.04E-40	2.34E-40
novel_pir332	106	0	3.61	0.001	-11.81778312	Down	2.46E-40	5.52E-40
novel_pir2194	106	0	3.61	0.001	-11.81778312	Down	2.46E-40	5.51E-40
novel_pir1422	105	0	3.58	0.001	-11.80574387	Down	5.81E-40	1.30E-39
novel_pir333	105	0	3.58	0.001	-11.80574387	Down	5.81E-40	1.30E-39
novel_pir1201	105	0	3.58	0.001	-11.80574387	Down	5.81E-40	1.30E-39
novel_pir227	105	0	3.58	0.001	-11.80574387	Down	5.81E-40	1.29E-39
novel_pir2079	104	0	3.54	0.001	-11.78953364	Down	1.37E-39	3.05E-39
novel_pir518	103	0	3.51	0.001	-11.77725532	Down	3.23E-39	7.20E-39
novel_pir1837	102	0	3.47	0.001	-11.76071995	Down	7.63E-39	1.70E-38
novel_pir1063	102	0	3.47	0.001	-11.76071995	Down	7.63E-39	1.69E-38
novel_pir1549	101	0	3.44	0.001	-11.74819285	Down	1.80E-38	3.99E-38
novel_pir750	101	0	3.44	0.001	-11.74819285	Down	1.80E-38	3.99E-38
novel_pir1491	101	0	3.44	0.001	-11.74819285	Down	1.80E-38	3.98E-38
novel_pir21	100	0	3.41	0.001	-11.73555602	Down	4.25E-38	9.37E-38
novel_pir1944	100	0	3.41	0.001	-11.73555602	Down	4.25E-38	9.36E-38
novel_pir654	98	0	3.34	0.001	-11.70563239	Down	2.37E-37	5.21E-37
novel_pir1619	97	0	3.3	0.001	-11.68825031	Down	5.59E-37	1.23E-36
novel_pir1782	97	0	3.3	0.001	-11.68825031	Down	5.59E-37	1.23E-36
novel_pir2264	97	0	3.3	0.001	-11.68825031	Down	5.59E-37	1.23E-36
novel_pir2370	95	0	3.24	0.001	-11.6617781	Down	3.12E-36	6.81E-36
novel_pir1287	95	0	3.24	0.001	-11.6617781	Down	3.12E-36	6.80E-36
novel_pir1483	94	0	3.2	0.001	-11.64385619	Down	7.36E-36	1.60E-35
novel_pir1276	93	0	3.17	0.001	-11.63026713	Down	1.74E-35	3.77E-35
novel_pir1880	93	0	3.17	0.001	-11.63026713	Down	1.74E-35	3.77E-35
novel_pir2147	93	0	3.17	0.001	-11.63026713	Down	1.74E-35	3.77E-35
novel_pir968	93	0	3.17	0.001	-11.63026713	Down	1.74E-35	3.76E-35
novel_pir1309	92	0	3.13	0.001	-11.61194694	Down	4.10E-35	8.86E-35
novel_pir1714	92	0	3.13	0.001	-11.61194694	Down	4.10E-35	8.85E-35
novel_pir1357	92	0	3.13	0.001	-11.61194694	Down	4.10E-35	8.84E-35

novel_pir1946	91	0	3.1	0.001	-11.5980525	Down	9.68E-35	2.08E-34
novel_pir403	90	0	3.06	0.001	-11.57931594	Down	2.28E-34	4.91E-34
novel_pir696	89	0	3.03	0.001	-11.56510208	Down	5.39E-34	1.16E-33
novel_pir886	89	0	3.03	0.001	-11.56510208	Down	5.39E-34	1.16E-33
novel_pir478	88	0	3	0.001	-11.55074679	Down	1.27E-33	2.72E-33
novel_pir776	88	0	3	0.001	-11.55074679	Down	1.27E-33	2.72E-33
novel_pir322	88	0	3	0.001	-11.55074679	Down	1.27E-33	2.72E-33
novel_pir179	88	0	3	0.001	-11.55074679	Down	1.27E-33	2.72E-33
novel_pir624	87	0	2.96	0.001	-11.53138146	Down	3.00E-33	6.40E-33
novel_pir924	87	0	2.96	0.001	-11.53138146	Down	3.00E-33	6.39E-33
novel_pir1289	87	0	2.96	0.001	-11.53138146	Down	3.00E-33	6.38E-33
novel_pir912	86	0	2.93	0.001	-11.51668495	Down	7.09E-33	1.50E-32
novel_pir1410	86	0	2.93	0.001	-11.51668495	Down	7.09E-33	1.50E-32
novel_pir2259	86	0	2.93	0.001	-11.51668495	Down	7.09E-33	1.50E-32
novel_pir2106	85	0	2.89	0.001	-11.49685378	Down	1.67E-32	3.54E-32
novel_pir1132	84	0	2.86	0.001	-11.48179943	Down	3.95E-32	8.34E-32
novel_pir1861	84	0	2.86	0.001	-11.48179943	Down	3.95E-32	8.33E-32
novel_pir2431	84	0	2.86	0.001	-11.48179943	Down	3.95E-32	8.33E-32
novel_pir1235	83	0	2.83	0.001	-11.46658634	Down	9.32E-32	1.96E-31
novel_pir1051	83	0	2.83	0.001	-11.46658634	Down	9.32E-32	1.96E-31
novel_pir1741	82	0	2.79	0.001	-11.44604941	Down	2.20E-31	4.62E-31
novel_pir1844	82	0	2.79	0.001	-11.44604941	Down	2.20E-31	4.62E-31
novel_pir2440	80	0	2.72	0.001	-11.40939094	Down	1.23E-30	2.57E-30
novel_pir2261	79	0	2.69	0.001	-11.39339046	Down	2.89E-30	6.05E-30
novel_pir307	79	0	2.69	0.001	-11.39339046	Down	2.89E-30	6.04E-30
novel_pir960	78	0	2.66	0.001	-11.37721053	Down	6.83E-30	1.42E-29
novel_pir1352	78	0	2.66	0.001	-11.37721053	Down	6.83E-30	1.42E-29
novel_pir1869	78	0	2.66	0.001	-11.37721053	Down	6.83E-30	1.42E-29
novel_pir1835	77	0	2.62	0.001	-11.3553511	Down	1.61E-29	3.35E-29
novel_pir1686	77	0	2.62	0.001	-11.3553511	Down	1.61E-29	3.35E-29
novel_pir30	77	0	2.62	0.001	-11.3553511	Down	1.61E-29	3.34E-29
novel_pir810	77	0	2.62	0.001	-11.3553511	Down	1.61E-29	3.34E-29
novel_pir2428	75	0	2.55	0.001	-11.31628153	Down	8.99E-29	1.85E-28
novel_pir1810	74	0	2.52	0.001	-11.29920802	Down	2.12E-28	4.37E-28
novel_pir2426	74	0	2.52	0.001	-11.29920802	Down	2.12E-28	4.36E-28
novel_pir2473	73	0	2.49	0.001	-11.28193003	Down	5.01E-28	1.03E-27

novel_pir1555	73	0	2.49	0.001	-11.28193003	Down	5.01E-28	1.03E-27
novel_pir2345	72	0	2.45	0.001	-11.25856603	Down	1.18E-27	2.42E-27
novel_pir361	72	0	2.45	0.001	-11.25856603	Down	1.18E-27	2.42E-27
novel_pir1085	71	0	2.42	0.001	-11.24079133	Down	2.79E-27	5.69E-27
novel_pir1521	71	0	2.42	0.001	-11.24079133	Down	2.79E-27	5.69E-27
novel_pir2342	71	0	2.42	0.001	-11.24079133	Down	2.79E-27	5.68E-27
novel_pir1124	71	0	2.42	0.001	-11.24079133	Down	2.79E-27	5.68E-27
novel_pir2028	71	0	2.42	0.001	-11.24079133	Down	2.79E-27	5.67E-27
novel_pir1268	70	0	2.38	0.001	-11.21674586	Down	6.58E-27	1.34E-26
novel_pir1550	69	0	2.35	0.001	-11.19844504	Down	1.55E-26	3.15E-26
mmu_piR_016069	69	0	2.35	0.001	-11.19844504	Down	1.55E-26	3.15E-26
novel_pir777	69	0	2.35	0.001	-11.19844504	Down	1.55E-26	3.14E-26
novel_pir2337	69	0	2.35	0.001	-11.19844504	Down	1.55E-26	3.14E-26
novel_pir2019	68	0	2.32	0.001	-11.17990909	Down	3.67E-26	7.39E-26
novel_pir1081	68	0	2.32	0.001	-11.17990909	Down	3.67E-26	7.38E-26
novel_pir2187	67	0	2.28	0.001	-11.15481811	Down	8.66E-26	1.74E-25
novel_pir1923	67	0	2.28	0.001	-11.15481811	Down	8.66E-26	1.74E-25
novel_pir1959	66	0	2.25	0.001	-11.13570929	Down	2.04E-25	4.10E-25
novel_pir962	66	0	2.25	0.001	-11.13570929	Down	2.04E-25	4.09E-25
novel_pir2188	65	0	2.21	0.001	-11.10983065	Down	4.83E-25	9.65E-25
novel_pir661	65	0	2.21	0.001	-11.10983065	Down	4.83E-25	9.64E-25
novel_pir1938	64	0	2.18	0.001	-11.09011242	Down	1.14E-24	2.27E-24
novel_pir1299	64	0	2.18	0.001	-11.09011242	Down	1.14E-24	2.27E-24
novel_pir522	64	0	2.18	0.001	-11.09011242	Down	1.14E-24	2.27E-24
novel_pir1257	63	0	2.15	0.001	-11.07012094	Down	2.69E-24	5.35E-24
novel_pir896	63	0	2.15	0.001	-11.07012094	Down	2.69E-24	5.34E-24
novel_pir1193	63	0	2.15	0.001	-11.07012094	Down	2.69E-24	5.34E-24
novel_pir2143	60	0	2.04	0.001	-10.99435344	Down	3.54E-23	6.98E-23
novel_pir1841	60	0	2.04	0.001	-10.99435344	Down	3.54E-23	6.98E-23
novel_pir453	60	0	2.04	0.001	-10.99435344	Down	3.54E-23	6.97E-23
novel_pir2196	60	0	2.04	0.001	-10.99435344	Down	3.54E-23	6.96E-23
novel_pir1776	59	0	2.01	0.001	-10.97297979	Down	8.35E-23	1.64E-22
novel_pir760	58	0	1.98	0.001	-10.95128471	Down	1.97E-22	3.87E-22
novel_pir1300	58	0	1.98	0.001	-10.95128471	Down	1.97E-22	3.87E-22
novel_pir476	58	0	1.98	0.001	-10.95128471	Down	1.97E-22	3.86E-22
novel_pir1464	58	0	1.98	0.001	-10.95128471	Down	1.97E-22	3.86E-22

novel_pir867	58	0	1.98	0.001	-10.95128471	Down	1.97E-22	3.85E-22
novel_pir1839	58	0	1.98	0.001	-10.95128471	Down	1.97E-22	3.85E-22
novel_pir251	57	0	1.94	0.001	-10.92184094	Down	4.65E-22	9.08E-22
novel_pir2124	57	0	1.94	0.001	-10.92184094	Down	4.65E-22	9.07E-22
novel_pir1563	56	0	1.91	0.001	-10.89935692	Down	1.10E-21	2.13E-21
novel_pir1198	56	0	1.91	0.001	-10.89935692	Down	1.10E-21	2.13E-21
novel_pir692	56	0	1.91	0.001	-10.89935692	Down	1.10E-21	2.13E-21
novel_pir1197	56	0	1.91	0.001	-10.89935692	Down	1.10E-21	2.13E-21
novel_pir885	55	0	1.87	0.001	-10.86882255	Down	2.59E-21	5.01E-21
novel_pir80	55	0	1.87	0.001	-10.86882255	Down	2.59E-21	5.00E-21
novel_pir2151	55	0	1.87	0.001	-10.86882255	Down	2.59E-21	5.00E-21
novel_pir1980	54	0	1.84	0.001	-10.84549005	Down	6.12E-21	1.18E-20
novel_pir530	54	0	1.84	0.001	-10.84549005	Down	6.12E-21	1.18E-20
novel_pir325	54	0	1.84	0.001	-10.84549005	Down	6.12E-21	1.17E-20
novel_pir2069	54	0	1.84	0.001	-10.84549005	Down	6.12E-21	1.17E-20
novel_pir699	54	0	1.84	0.001	-10.84549005	Down	6.12E-21	1.17E-20
novel_pir565	54	0	1.84	0.001	-10.84549005	Down	6.12E-21	1.17E-20
novel_pir2063	53	0	1.8	0.001	-10.81378119	Down	1.44E-20	2.76E-20
novel_pir1000	53	0	1.8	0.001	-10.81378119	Down	1.44E-20	2.76E-20
novel_pir716	52	0	1.77	0.001	-10.78953364	Down	3.41E-20	6.49E-20
novel_pir1353	52	0	1.77	0.001	-10.78953364	Down	3.41E-20	6.48E-20
novel_pir2065	52	0	1.77	0.001	-10.78953364	Down	3.41E-20	6.48E-20
novel_pir2459	52	0	1.77	0.001	-10.78953364	Down	3.41E-20	6.47E-20
novel_pir2030	52	0	1.77	0.001	-10.78953364	Down	3.41E-20	6.46E-20
novel_pir2326	51	0	1.74	0.001	-10.76487159	Down	8.04E-20	1.52E-19
novel_pir499	50	0	1.7	0.001	-10.73131903	Down	1.90E-19	3.59E-19
novel_pir977	50	0	1.7	0.001	-10.73131903	Down	1.90E-19	3.58E-19
novel_pir739	49	0	1.67	0.001	-10.70563239	Down	4.48E-19	8.44E-19
novel_pir1699	49	0	1.67	0.001	-10.70563239	Down	4.48E-19	8.43E-19
novel_pir267	49	0	1.67	0.001	-10.70563239	Down	4.48E-19	8.43E-19
novel_pir2208	49	0	1.67	0.001	-10.70563239	Down	4.48E-19	8.42E-19
novel_pir471	48	0	1.63	0.001	-10.67065625	Down	1.06E-18	1.98E-18
novel_pir965	48	0	1.63	0.001	-10.67065625	Down	1.06E-18	1.98E-18
novel_pir2152	47	0	1.6	0.001	-10.64385619	Down	2.50E-18	4.67E-18
novel_pir1087	47	0	1.6	0.001	-10.64385619	Down	2.50E-18	4.66E-18
novel_pir2328	47	0	1.6	0.001	-10.64385619	Down	2.50E-18	4.66E-18

novel_pir1606	46	0	1.57	0.001	-10.61654884	Down	5.89E-18	1.10E-17
novel_pir1929	46	0	1.57	0.001	-10.61654884	Down	5.89E-18	1.10E-17
novel_pir1165	46	0	1.57	0.001	-10.61654884	Down	5.89E-18	1.10E-17
novel_pir2361	45	0	1.53	0.001	-10.57931594	Down	1.39E-17	2.58E-17
novel_pir1254	45	0	1.53	0.001	-10.57931594	Down	1.39E-17	2.58E-17
novel_pir1344	45	0	1.53	0.001	-10.57931594	Down	1.39E-17	2.57E-17
novel_pir1200	44	0	1.5	0.001	-10.55074679	Down	3.28E-17	6.07E-17
novel_pir1367	44	0	1.5	0.001	-10.55074679	Down	3.28E-17	6.06E-17
novel_pir2177	44	0	1.5	0.001	-10.55074679	Down	3.28E-17	6.06E-17
novel_pir2163	44	0	1.5	0.001	-10.55074679	Down	3.28E-17	6.05E-17
novel_pir94	44	0	1.5	0.001	-10.55074679	Down	3.28E-17	6.05E-17
novel_pir1570	44	0	1.5	0.001	-10.55074679	Down	3.28E-17	6.04E-17
novel_pir1396	44	0	1.5	0.001	-10.55074679	Down	3.28E-17	6.04E-17
novel_pir134	43	0	1.46	0.001	-10.51175265	Down	7.75E-17	1.42E-16
novel_pir2472	43	0	1.46	0.001	-10.51175265	Down	7.75E-17	1.42E-16
novel_pir385	43	0	1.46	0.001	-10.51175265	Down	7.75E-17	1.42E-16
novel_pir1781	43	0	1.46	0.001	-10.51175265	Down	7.75E-17	1.42E-16
novel_pir1713	43	0	1.46	0.001	-10.51175265	Down	7.75E-17	1.41E-16
novel_pir1687	42	0	1.43	0.001	-10.48179943	Down	1.83E-16	3.33E-16
novel_pir2294	42	0	1.43	0.001	-10.48179943	Down	1.83E-16	3.33E-16
novel_pir592	42	0	1.43	0.001	-10.48179943	Down	1.83E-16	3.33E-16
novel_pir1168	42	0	1.43	0.001	-10.48179943	Down	1.83E-16	3.32E-16
novel_pir1972	42	0	1.43	0.001	-10.48179943	Down	1.83E-16	3.32E-16
novel_pir1703	41	0	1.4	0.001	-10.45121111	Down	4.32E-16	7.82E-16
novel_pir847	41	0	1.4	0.001	-10.45121111	Down	4.32E-16	7.81E-16
novel_pir1760	41	0	1.4	0.001	-10.45121111	Down	4.32E-16	7.81E-16
novel_pir1069	41	0	1.4	0.001	-10.45121111	Down	4.32E-16	7.80E-16
novel_pir1280	41	0	1.4	0.001	-10.45121111	Down	4.32E-16	7.79E-16
novel_pir813	40	0	1.36	0.001	-10.40939094	Down	1.02E-15	1.84E-15
novel_pir2132	40	0	1.36	0.001	-10.40939094	Down	1.02E-15	1.84E-15
novel_pir923	40	0	1.36	0.001	-10.40939094	Down	1.02E-15	1.83E-15
novel_pir1613	40	0	1.36	0.001	-10.40939094	Down	1.02E-15	1.83E-15
novel_pir1672	40	0	1.36	0.001	-10.40939094	Down	1.02E-15	1.83E-15
novel_pir62	39	0	1.33	0.001	-10.37721053	Down	2.41E-15	4.31E-15
novel_pir2322	39	0	1.33	0.001	-10.37721053	Down	2.41E-15	4.31E-15
novel_pir1724	39	0	1.33	0.001	-10.37721053	Down	2.41E-15	4.30E-15

novel_pir61	39	0	1.33	0.001	-10.37721053	Down	2.41E-15	4.30E-15
novel_pir2221	39	0	1.33	0.001	-10.37721053	Down	2.41E-15	4.30E-15
novel_pir848	38	0	1.29	0.001	-10.33315535	Down	5.68E-15	1.01E-14
novel_pir2115	38	0	1.29	0.001	-10.33315535	Down	5.68E-15	1.01E-14
novel_pir829	37	0	1.26	0.001	-10.29920802	Down	1.34E-14	2.38E-14
novel_pir1988	37	0	1.26	0.001	-10.29920802	Down	1.34E-14	2.38E-14
novel_pir1253	36	0	1.23	0.001	-10.2644426	Down	3.16E-14	5.60E-14
novel_pir993	36	0	1.23	0.001	-10.2644426	Down	3.16E-14	5.59E-14
novel_pir2317	36	0	1.23	0.001	-10.2644426	Down	3.16E-14	5.59E-14
novel_pir1600	36	0	1.23	0.001	-10.2644426	Down	3.16E-14	5.58E-14
novel_pir252	35	0	1.19	0.001	-10.21674586	Down	7.47E-14	1.32E-13
novel_pir309	34	0	1.16	0.001	-10.17990909	Down	1.76E-13	3.10E-13
novel_pir1652	34	0	1.16	0.001	-10.17990909	Down	1.76E-13	3.09E-13
novel_pir1819	34	0	1.16	0.001	-10.17990909	Down	1.76E-13	3.09E-13
novel_pir1082	33	0	1.12	0.001	-10.12928302	Down	4.16E-13	7.27E-13
novel_pir1298	33	0	1.12	0.001	-10.12928302	Down	4.16E-13	7.26E-13
novel_pir529	33	0	1.12	0.001	-10.12928302	Down	4.16E-13	7.25E-13
novel_pir934	33	0	1.12	0.001	-10.12928302	Down	4.16E-13	7.25E-13
novel_pir1512	32	0	1.09	0.001	-10.09011242	Down	9.82E-13	1.70E-12
novel_pir893	32	0	1.09	0.001	-10.09011242	Down	9.82E-13	1.70E-12
novel_pir414	31	0	1.06	0.001	-10.04984855	Down	2.32E-12	4.01E-12
novel_pir331	30	0	1.02	0.001	-9.994353437	Down	5.47E-12	9.46E-12
novel_pir1742	30	0	1.02	0.001	-9.994353437	Down	5.47E-12	9.45E-12
novel_pir1330	29	0	0.99	0.001	-9.951284715	Down	1.29E-11	2.22E-11
novel_pir2359	29	0	0.99	0.001	-9.951284715	Down	1.29E-11	2.22E-11
novel_pir2016	28	0	0.95	0.001	-9.891783703	Down	3.05E-11	5.22E-11
novel_pir976	28	0	0.95	0.001	-9.891783703	Down	3.05E-11	5.21E-11
novel_pir602	28	0	0.95	0.001	-9.891783703	Down	3.05E-11	5.21E-11
novel_pir256	28	0	0.95	0.001	-9.891783703	Down	3.05E-11	5.20E-11
novel_pir650	27	0	0.92	0.001	-9.845490051	Down	7.20E-11	1.22E-10
novel_pir272	27	0	0.92	0.001	-9.845490051	Down	7.20E-11	1.22E-10
novel_pir1891	27	0	0.92	0.001	-9.845490051	Down	7.20E-11	1.22E-10
novel_pir1249	27	0	0.92	0.001	-9.845490051	Down	7.20E-11	1.22E-10
novel_pir623	26	0	0.89	0.001	-9.797661526	Down	1.70E-10	2.87E-10
novel_pir626	26	0	0.89	0.001	-9.797661526	Down	1.70E-10	2.86E-10
novel_pir857	26	0	0.89	0.001	-9.797661526	Down	1.70E-10	2.86E-10

novel_pir1849	26	0	0.89	0.001	-9.797661526	Down	1.70E-10	2.86E-10
novel_pir869	26	0	0.89	0.001	-9.797661526	Down	1.70E-10	2.86E-10
novel_pir1305	26	0	0.89	0.001	-9.797661526	Down	1.70E-10	2.85E-10
novel_pir461	26	0	0.89	0.001	-9.797661526	Down	1.70E-10	2.85E-10
novel_pir1527	26	0	0.89	0.001	-9.797661526	Down	1.70E-10	2.85E-10
novel_pir1663	26	0	0.89	0.001	-9.797661526	Down	1.70E-10	2.84E-10
novel_pir691	25	0	0.85	0.001	-9.731319031	Down	4.01E-10	6.70E-10
novel_pir2070	25	0	0.85	0.001	-9.731319031	Down	4.01E-10	6.69E-10
novel_pir383	25	0	0.85	0.001	-9.731319031	Down	4.01E-10	6.69E-10
novel_pir188	25	0	0.85	0.001	-9.731319031	Down	4.01E-10	6.68E-10
novel_pir2073	24	0	0.82	0.001	-9.6794801	Down	9.47E-10	1.57E-09
novel_pir384	24	0	0.82	0.001	-9.6794801	Down	9.47E-10	1.57E-09
novel_pir1617	24	0	0.82	0.001	-9.6794801	Down	9.47E-10	1.57E-09
novel_pir1580	24	0	0.82	0.001	-9.6794801	Down	9.47E-10	1.56E-09
novel_pir865	24	0	0.82	0.001	-9.6794801	Down	9.47E-10	1.56E-09
novel_pir1778	24	0	0.82	0.001	-9.6794801	Down	9.47E-10	1.56E-09
novel_pir2323	24	0	0.82	0.001	-9.6794801	Down	9.47E-10	1.56E-09
novel_pir50	24	0	0.82	0.001	-9.6794801	Down	9.47E-10	1.56E-09
novel_pir259	23	0	0.78	0.001	-9.607330314	Down	2.23E-09	3.68E-09
novel_pir1250	23	0	0.78	0.001	-9.607330314	Down	2.23E-09	3.67E-09
novel_pir1905	23	0	0.78	0.001	-9.607330314	Down	2.23E-09	3.67E-09
novel_pir1244	22	0	0.75	0.001	-9.550746785	Down	5.27E-09	8.62E-09
novel_pir2394	22	0	0.75	0.001	-9.550746785	Down	5.27E-09	8.61E-09
novel_pir1746	22	0	0.75	0.001	-9.550746785	Down	5.27E-09	8.60E-09
novel_pir1820	22	0	0.75	0.001	-9.550746785	Down	5.27E-09	8.59E-09
novel_pir939	22	0	0.75	0.001	-9.550746785	Down	5.27E-09	8.59E-09
novel_pir200	22	0	0.75	0.001	-9.550746785	Down	5.27E-09	8.58E-09
novel_pir2384	22	0	0.75	0.001	-9.550746785	Down	5.27E-09	8.57E-09
novel_pir1106	21	0	0.72	0.001	-9.491853096	Down	1.25E-08	2.02E-08
novel_pir326	21	0	0.72	0.001	-9.491853096	Down	1.25E-08	2.02E-08
novel_pir320	21	0	0.72	0.001	-9.491853096	Down	1.25E-08	2.01E-08
novel_pir753	21	0	0.72	0.001	-9.491853096	Down	1.25E-08	2.01E-08
novel_pir150	21	0	0.72	0.001	-9.491853096	Down	1.25E-08	2.01E-08
novel_pir2311	21	0	0.72	0.001	-9.491853096	Down	1.25E-08	2.01E-08
novel_pir2056	20	0	0.68	0.001	-9.409390936	Down	2.94E-08	4.71E-08
novel_pir2362	20	0	0.68	0.001	-9.409390936	Down	2.94E-08	4.70E-08

novel_pir908	20	0	0.68	0.001	-9.409390936	Down	2.94E-08	4.70E-08
novel_pir714	19	0	0.65	0.001	-9.344295908	Down	6.94E-08	1.11E-07
novel_pir2339	19	0	0.65	0.001	-9.344295908	Down	6.94E-08	1.11E-07
novel_pir1003	19	0	0.65	0.001	-9.344295908	Down	6.94E-08	1.10E-07
novel_pir1757	19	0	0.65	0.001	-9.344295908	Down	6.94E-08	1.10E-07
novel_pir1134	19	0	0.65	0.001	-9.344295908	Down	6.94E-08	1.10E-07
novel_pir410	19	0	0.65	0.001	-9.344295908	Down	6.94E-08	1.10E-07
novel_pir1401	19	0	0.65	0.001	-9.344295908	Down	6.94E-08	1.10E-07
novel_pir2149	18	0	0.61	0.001	-9.252665432	Down	1.64E-07	2.58E-07
novel_pir1836	18	0	0.61	0.001	-9.252665432	Down	1.64E-07	2.58E-07
novel_pir2215	18	0	0.61	0.001	-9.252665432	Down	1.64E-07	2.57E-07
novel_pir652	17	0	0.58	0.001	-9.17990909	Down	3.87E-07	6.06E-07
novel_pir2189	17	0	0.58	0.001	-9.17990909	Down	3.87E-07	6.05E-07
novel_pir255	17	0	0.58	0.001	-9.17990909	Down	3.87E-07	6.05E-07
novel_pir1409	17	0	0.58	0.001	-9.17990909	Down	3.87E-07	6.04E-07
novel_pir1614	17	0	0.58	0.001	-9.17990909	Down	3.87E-07	6.04E-07
novel_pir302	17	0	0.58	0.001	-9.17990909	Down	3.87E-07	6.03E-07
novel_pir873	17	0	0.58	0.001	-9.17990909	Down	3.87E-07	6.03E-07
novel_pir1356	17	0	0.58	0.001	-9.17990909	Down	3.87E-07	6.02E-07
novel_pir1576	17	0	0.58	0.001	-9.17990909	Down	3.87E-07	6.02E-07
novel_pir647	17	0	0.58	0.001	-9.17990909	Down	3.87E-07	6.01E-07
novel_pir2234	17	0	0.58	0.001	-9.17990909	Down	3.87E-07	6.01E-07
novel_pir44	16	0	0.54	0.001	-9.076815597	Down	9.12E-07	1.41E-06
novel_pir454	16	0	0.54	0.001	-9.076815597	Down	9.12E-07	1.41E-06
novel_pir1743	16	0	0.54	0.001	-9.076815597	Down	9.12E-07	1.40E-06
novel_pir1176	16	0	0.54	0.001	-9.076815597	Down	9.12E-07	1.40E-06
novel_pir1054	16	0	0.54	0.001	-9.076815597	Down	9.12E-07	1.40E-06
novel_pir1685	16	0	0.54	0.001	-9.076815597	Down	9.12E-07	1.40E-06
novel_pir1756	16	0	0.54	0.001	-9.076815597	Down	9.12E-07	1.40E-06
novel_pir1779	16	0	0.54	0.001	-9.076815597	Down	9.12E-07	1.40E-06
novel_pir2064	16	0	0.54	0.001	-9.076815597	Down	9.12E-07	1.40E-06
novel_pir812	16	0	0.54	0.001	-9.076815597	Down	9.12E-07	1.40E-06
novel_pir594	15	0	0.51	0.001	-8.994353437	Down	2.15E-06	3.24E-06
novel_pir1104	15	0	0.51	0.001	-8.994353437	Down	2.15E-06	3.24E-06
novel_pir1428	15	0	0.51	0.001	-8.994353437	Down	2.15E-06	3.23E-06
novel_pir1471	15	0	0.51	0.001	-8.994353437	Down	2.15E-06	3.23E-06

novel_pir399	6067	17	206.61	0.43	-8.908357708	Down	0	0
novel_pir359	14	0	0.48	0.001	-8.906890596	Down	5.08E-06	7.54E-06
novel_pir2358	14	0	0.48	0.001	-8.906890596	Down	5.08E-06	7.54E-06
novel_pir1350	14	0	0.48	0.001	-8.906890596	Down	5.08E-06	7.53E-06
novel_pir2182	14	0	0.48	0.001	-8.906890596	Down	5.08E-06	7.53E-06
novel_pir1892	14	0	0.48	0.001	-8.906890596	Down	5.08E-06	7.52E-06
novel_pir1053	14	0	0.48	0.001	-8.906890596	Down	5.08E-06	7.52E-06
novel_pir932	14	0	0.48	0.001	-8.906890596	Down	5.08E-06	7.51E-06
novel_pir1914	3286	10	111.91	0.25	-8.806195147	Down	0	0
novel_pir103	13	0	0.44	0.001	-8.781359714	Down	1.20E-05	1.75E-05
novel_pir1547	13	0	0.44	0.001	-8.781359714	Down	1.20E-05	1.75E-05
novel_pir1318	13	0	0.44	0.001	-8.781359714	Down	1.20E-05	1.75E-05
novel_pir761	13	0	0.44	0.001	-8.781359714	Down	1.20E-05	1.75E-05
novel_pir2457	13	0	0.44	0.001	-8.781359714	Down	1.20E-05	1.75E-05
novel_pir1026	13	0	0.44	0.001	-8.781359714	Down	1.20E-05	1.74E-05
novel_pir2020	13	0	0.44	0.001	-8.781359714	Down	1.20E-05	1.74E-05
novel_pir210	13	0	0.44	0.001	-8.781359714	Down	1.20E-05	1.74E-05
novel_pir2463	13	0	0.44	0.001	-8.781359714	Down	1.20E-05	1.74E-05
novel_pir1424	12	0	0.41	0.001	-8.6794801	Down	2.83E-05	4.07E-05
novel_pir1221	12	0	0.41	0.001	-8.6794801	Down	2.83E-05	4.06E-05
novel_pir1504	12	0	0.41	0.001	-8.6794801	Down	2.83E-05	4.06E-05
novel_pir234	12	0	0.41	0.001	-8.6794801	Down	2.83E-05	4.06E-05
novel_pir1064	12	0	0.41	0.001	-8.6794801	Down	2.83E-05	4.05E-05
novel_pir409	12	0	0.41	0.001	-8.6794801	Down	2.83E-05	4.05E-05
novel_pir922	12	0	0.41	0.001	-8.6794801	Down	2.83E-05	4.05E-05
novel_pir905	12	0	0.41	0.001	-8.6794801	Down	2.83E-05	4.04E-05
novel_pir1186	12	0	0.41	0.001	-8.6794801	Down	2.83E-05	4.04E-05
novel_pir636	11	0	0.37	0.001	-8.531381461	Down	6.69E-05	9.28E-05
novel_pir27	11	0	0.37	0.001	-8.531381461	Down	6.69E-05	9.27E-05
novel_pir1762	11	0	0.37	0.001	-8.531381461	Down	6.69E-05	9.26E-05
novel_pir2460	11	0	0.37	0.001	-8.531381461	Down	6.69E-05	9.26E-05
novel_pir1542	11	0	0.37	0.001	-8.531381461	Down	6.69E-05	9.25E-05
novel_pir1230	11	0	0.37	0.001	-8.531381461	Down	6.69E-05	9.24E-05
novel_pir1275	11	0	0.37	0.001	-8.531381461	Down	6.69E-05	9.24E-05
novel_pir70	11	0	0.37	0.001	-8.531381461	Down	6.69E-05	9.23E-05
novel_pir715	11	0	0.37	0.001	-8.531381461	Down	6.69E-05	9.22E-05

novel_pir2138	11	0	0.37	0.001	-8.531381461	Down	6.69E-05	9.22E-05
novel_pir231	3004	12	102.3	0.3	-8.413627929	Down	0	0
novel_pir1812	10	0	0.34	0.001	-8.409390936	Down	0.000157801	0.000215517
novel_pir914	10	0	0.34	0.001	-8.409390936	Down	0.000157801	0.00021536
novel_pir2123	10	0	0.34	0.001	-8.409390936	Down	0.000157801	0.000215204
novel_pir1801	10	0	0.34	0.001	-8.409390936	Down	0.000157801	0.000215048
novel_pir2191	10	0	0.34	0.001	-8.409390936	Down	0.000157801	0.000214893
novel_pir99	10	0	0.34	0.001	-8.409390936	Down	0.000157801	0.000214737
novel_pir1831	10	0	0.34	0.001	-8.409390936	Down	0.000157801	0.000214582
novel_pir1170	10	0	0.34	0.001	-8.409390936	Down	0.000157801	0.000214427
novel_pir1954	10	0	0.34	0.001	-8.409390936	Down	0.000157801	0.000214273
novel_pir1136	10	0	0.34	0.001	-8.409390936	Down	0.000157801	0.000214118
novel_pir2251	10	0	0.34	0.001	-8.409390936	Down	0.000157801	0.000213964
novel_pir2140	10	0	0.34	0.001	-8.409390936	Down	0.000157801	0.00021381
novel_pir1022	10	0	0.34	0.001	-8.409390936	Down	0.000157801	0.000213656
mmu_piR_000705	9	0	0.31	0.001	-8.276124405	Down	0.00037248	0.000481131
mmu_piR_023189	3681	39	125.36	0.98	-6.99907962	Down	0	0
mmu_piR_010309	1894	23	64.5	0.58	-6.79710245	Down	0	0
novel_pir273	745	12	25.37	0.3	-6.402017208	Down	1.05E-255	5.47E-254
mmu_piR_022820	355	6	12.09	0.15	-6.332707934	Down	3.65E-122	2.34E-121
novel_pir807	1826	31	62.18	0.78	-6.316832682	Down	0	0
novel_pir2385	735	15	25.03	0.38	-6.041515062	Down	1.42E-247	6.52E-246
novel_pir229	857	20	29.19	0.5	-5.867402306	Down	7.65E-285	4.64E-283
novel_pir710	352	10	11.99	0.25	-5.583759754	Down	1.72E-115	9.87E-115
novel_pir230	1224	35	41.68	0.88	-5.565707944	Down	0	0
novel_pir1272	2888	88	98.35	2.2	-5.482349624	Down	0	0
novel_pir2411	387	12	13.18	0.3	-5.457244059	Down	1.55E-125	1.06E-124
novel_pir1720	949	34	32.32	0.85	-5.248820547	Down	7.48E-300	5.03E-298
novel_pir711	617	25	21.01	0.63	-5.059580523	Down	4.28E-192	1.26E-190
novel_pir278	370	15	12.6	0.38	-5.051280505	Down	8.07E-116	4.64E-115
novel_pir806	1617	74	55.07	1.85	-4.895669433	Down	0	0
novel_pir1655	462	21	15.73	0.53	-4.891382501	Down	1.19E-141	1.23E-140
novel_pir1609	333	16	11.34	0.4	-4.82527683	Down	1.38E-101	6.07E-101
novel_pir131	786	40	26.77	1	-4.742545234	Down	3.73E-235	1.56E-233
novel_pir1440	309	16	10.52	0.4	-4.716990894	Down	3.88E-93	1.48E-92
novel_pir945	377	21	12.84	0.53	-4.598509033	Down	9.59E-112	5.03E-111

novel_pir557	284	16	9.67	0.4	-4.595443985	Down	2.23E-84	7.55E-84
mmu_piR_002962	388	24	13.21	0.6	-4.460524156	Down	1.51E-112	8.02E-112
novel_pir571	386	25	13.15	0.63	-4.383567161	Down	7.08E-111	3.68E-110
novel_pir1601	569	38	19.38	0.95	-4.350497247	Down	1.51E-161	2.52E-160
novel_pir57	179	12	6.1	0.3	-4.345774837	Down	7.57E-52	1.87E-51
novel_pir277	357	26	12.16	0.65	-4.2255597	Down	6.06E-100	2.58E-99
novel_pir1627	354	26	12.06	0.65	-4.213646379	Down	6.45E-99	2.73E-98
novel_pir398	2111	160	71.89	4.01	-4.164116962	Down	0	0
novel_pir984	3461	331	117.87	8.29	-3.829680662	Down	0	0
novel_pir397	114	11	3.88	0.28	-3.79255792	Down	1.30E-30	2.72E-30
novel_pir130	837	84	28.5	2.1	-3.762500686	Down	3.07E-212	1.01E-210
novel_pir296	569	60	19.38	1.5	-3.691534165	Down	1.42E-142	1.52E-141
novel_pir2122	3899	418	132.78	10.46	-3.6660831	Down	0	0
novel_pir2035	149	18	5.07	0.45	-3.493988841	Down	8.38E-37	1.83E-36
novel_pir561	189	23	6.44	0.58	-3.472935883	Down	3.75E-46	8.80E-46
novel_pir132	358	47	12.19	1.18	-3.368839361	Down	1.50E-83	5.02E-83
novel_pir290	88	12	3	0.3	-3.321928095	Down	2.26E-21	4.37E-21
novel_pir312	158	22	5.38	0.55	-3.290102649	Down	6.56E-37	1.44E-36
mmu_piR_031448	65076	9736	2216.18	243.72	-3.184778512	Down	0	0
novel_pir155	1175	185	40.01	4.63	-3.11127653	Down	7.87E-250	3.80E-248
novel_pir1696	572	92	19.48	2.3	-3.082287911	Down	2.24E-121	1.40E-120
novel_pir928	124	20	4.22	0.5	-3.077242999	Down	1.54E-27	3.16E-27
novel_pir717	2955	477	100.63	11.94	-3.075185726	Down	0	0
novel_pir133	1144	191	38.96	4.78	-3.026911154	Down	1.36E-236	5.82E-235
mmu_piR_013763	303	53	10.32	1.33	-2.95594482	Down	1.52E-62	4.09E-62
mmu_piR_028252	323	57	11	1.43	-2.943416472	Down	2.88E-66	7.99E-66
novel_pir1618	214	39	7.29	0.98	-2.89506516	Down	9.52E-44	2.19E-43
mmu_piR_017405	802	156	27.31	3.91	-2.804188801	Down	1.03E-153	1.45E-152
novel_pir1164	69	14	2.35	0.35	-2.74723393	Down	2.57E-14	4.55E-14
mmu_piR_000634	52	11	1.77	0.28	-2.660250628	Down	6.92E-11	1.18E-10
mmu_piR_025576	7161	1563	243.87	39.13	-2.639765279	Down	0	0
mmu_piR_032974	33	7	1.12	0.18	-2.637429921	Down	2.31E-07	3.62E-07
novel_pir244	72	16	2.45	0.4	-2.614709844	Down	3.67E-14	6.46E-14
novel_pir2416	697	158	23.74	3.96	-2.5837476	Down	3.66E-122	2.34E-121
novel_pir354	192	44	6.54	1.1	-2.571787112	Down	1.19E-34	2.56E-34
mmu_piR_018323	265	65	9.02	1.63	-2.468255469	Down	3.64E-45	8.47E-45

novel_pir719	565	149	19.24	3.73	-2.366861264	Down	1.31E-89	4.80E-89
novel_pir2422	313	84	10.66	2.1	-2.343746205	Down	8.73E-50	2.11E-49
novel_pir2468	459	126	15.63	3.15	-2.310894045	Down	5.54E-71	1.62E-70
novel_pir2033	879	243	29.93	6.08	-2.29944905	Down	1.59E-133	1.36E-132
novel_pir789	375	104	12.77	2.6	-2.296174997	Down	7.31E-58	1.90E-57
novel_pir1591	446	130	15.19	3.25	-2.224610247	Down	7.31E-66	2.02E-65
novel_pir2423	397	118	13.52	2.95	-2.196308292	Down	6.76E-58	1.76E-57
novel_pir608	295	100	10.05	2.5	-2.007195501	Down	8.62E-39	1.91E-38
novel_pir1667	149	52	5.07	1.3	-1.963474124	Down	7.50E-20	1.42E-19
novel_pir115	401	162	13.66	4.06	-1.750405851	Down	1.55E-43	3.56E-43
novel_pir684	45	21	1.53	0.53	-1.529467388	Down	2.50E-05	3.59E-05
mmu_piR_020120	633	304	21.56	7.61	-1.502388819	Down	2.05E-54	5.21E-54
novel_pir147	636	314	21.66	7.86	-1.462432025	Down	1.48E-52	3.69E-52
novel_pir145	1029	519	35.04	12.99	-1.431601344	Down	2.77E-81	8.89E-81
novel_pir149	370	192	12.6	4.81	-1.389314935	Down	4.29E-29	8.88E-29
novel_pir2425	347	183	11.82	4.58	-1.367810532	Down	1.01E-26	2.04E-26
novel_pir2420	453	243	15.43	6.08	-1.343594833	Down	1.79E-33	3.82E-33
novel_pir275	73	41	2.49	1.03	-1.273501405	Down	3.36E-06	5.04E-06
novel_pir718	781	469	26.6	11.74	-1.179993837	Down	2.52E-46	5.96E-46
novel_pir562	76	48	2.59	1.2	-1.109917692	Down	2.32E-05	3.33E-05
