# Supplementary material for: Pathogenic Effects and Potential Regulatory Mechanisms of Tea Polyphenols on Obesity
Source: Biomed Res Int. 2019 Jun 11;2019:2579734. doi: 10.1155/2019/2579734 (PMC6595166; doi:10.1155/2019/2579734)
Supplement: Supplementary 5 — Supplementary Material S5. Small RNA target prediction result. [file 2579734.f5.pdf]

miRNA id	Target id	RNAhybrid MFE	RNAhybrid pvalue	miRanda MFE	miRanda score	GO term	Pathway	NR id	NR evalue	NR description
mmu-let-7a-1-3p	NM_173785	-27.8	0.517532	-25.48	173	GO:0043231,GO:0008168,GO:0006779,GO:0006730,GO:0006544,	K06980|1|0.0|705|mmu:216792|	gi|148675734|gb|EDL07681.1|	0	RIKEN cDNA A230051G13 [Mus musculus]
mmu-let-7a-1-3p	NR_029725	-29.3	0.000661	-27.99	179	-	-	-	-	-
mmu-let-7a-1-3p	NR_029726	-28.3	0.001341	-27.96	179	-	-	-	-	-
mmu-let-7a-1-3p	NR_029731	-28	0.001359	-28.31	183	-	-	-	-	-
mmu-let-7a-1-3p	NR_029732	-29.9	0.000322	-28.26	183	-	-	-	-	-
mmu-let-7a-1-3p	NM_009103	-26.2	0.76834	-24.19	183	GO:0043234,GO:0031981,GO:0044444,GO:0016728,GO:0032559,GO:0006259,GO:0009165,GO:0051262,	K10807|1|0.0|1528|mmu:20133|ribonucleoside-diphosphate reductase subunit M1 [EC:1.17.4.1]	gi|31982026|ref|NP_033129.2|	0	ribonucleoside-diphosphate reductase large subunit [Mus musculus]

mmu-let-7a-1-3p	NM_019827	-26.6	0.916657	-22.47	180	GO:0030427,GO:0031981,GO:0043234,GO:0044425,GO:0030425,GO:0044444,GO:0044297,GO:0004672,GO:0051018,GO:0008134,GO:0019900,GO:0032559,GO:0008092,GO:0035254,GO:0006461,GO:0001952,GO:0032321,GO:0043405,GO:0048017,GO:0048167,GO:0007169,GO:00488	K03083|1|0.0|788|ecb:100061033|glycogen synthase kinase 3 beta [EC:2.7.11.26]	gi|387766242|pdb|4ACC|A	0	Chain A, Gsk3b In Complex With Inhibitor
mmu-let-7a-1-3p	NM_009767	-25.4	0.975109	-21.06	178	GO:0016020,GO:0031410,	-	gi|291407705|ref|XP_002720161.1|	1.03E-68	PREDICTED: cysteine-rich hydrophobic domain 2-like [Oryctolagus cuniculus]

mmu-let-7a-1-3p	NM_023502	-25.1	0.796618	-21.39	176	GO:0031981,GO:0003677,GO:0001071,GO:0006355,	K09428|1|0.0|995|mmu:69257|E74-like factor 1/2/4	gi|148703252|gb|EDL35199.1|	0	E74-like factor 2, isoform CRA_e [Mus musculus]
mmu-let-7a-1-3p	NM_033604	-25.2	0.936074	-20.16	174	GO:0032991,GO:0044424,GO:0046872,GO:0005515,GO:0019787,GO:0006355,GO:0016567,GO:0007179,GO:0007275,	K13201|1|7e-09|64.7|bfo:BRAFLDRAFT_124262|nucleolysin TIA-1/TIAR!K11980|2|9e-09|64.3|ame:408648|E3 ubiquitin-protein ligase RNF11	gi|15809022|ref|NP_291082.1|	0	E3 ubiquitin-protein ligase Arkadia [Mus musculus]

mmu-let-7a-1-3p	NM_001113204	-22.6	0.999988	-20.69	172	GO:0030427,GO:0043005,GO:0044297,GO:0009986,GO:0031224,GO:0019904,GO:0005539,GO:0019899,GO:0021536,GO:0014073,GO:0016337,GO:0010941,GO:0007610,GO:0051931,GO:0008284,GO:0031103,GO:0009967,GO:0010038,GO:00098	K06491|1|0.0|1561|gga:428253|neural cell adhesion molecule	gi|148693783|gb|EDL25730.1|	0	neural cell adhesion molecule 1, isoform CRA_d [Mus musculus]
mmu-let-7a-1-3p	NR_029730	-24.5	0.014993	-23.33	171	-	-	-	-	-
mmu-let-7a-1-3p	NM_028538	-24.5	0.980085	-23.02	168	-	K09228|1|0.0|1213|mmu:338354|KRAB domain-containing zinc finger protein	gi|223462786|gb|AAI41374.1|	0	1700049G17Rik protein [Mus musculus]

mmu-let-7a-1-3p	NM_001033173	-23.7	0.99994	-22.37	167	GO:0008233,	K11852|1|0.0|1986|mmu:76179|ubiquitin carboxyl-terminal hydrolase 31 [EC:3.1.2.15]	gi|226443061|ref|NP_001028345.1|	0	ubiquitin specific protease 31 [Mus musculus]
mmu-let-7a-1-3p	NM_153165	-22.8	0.995164	-24.29	166	-	K10452|1|1e-120|435|mmu:236904|kelch-like protein	gi|23346619|ref|NP_694805.1|	6.84E-120	kelch-like 15 isoform c [Mus musculus]
mmu-let-7a-1-3p	NM_026647	-23.5	0.999913	-21.28	165	GO:0012505,GO:0031224,GO:0046914,GO:0006807,GO:0032768,	K05766|1|7e-10|68.9|spu:575378|slingshot	gi|148699086|gb|EDL31033.1|	8.86E-160	zinc finger, DHHC domain containing 21 [Mus musculus]
mmu-let-7a-1-3p	NM_025534	-22.2	0.999939	-20.59	164	GO:0005488,	-	gi|61098118|ref|NP_079810.2|	0	coiled-coil domain-containing protein 82 [Mus musculus]
mmu-let-7a-1-3p	NM_182992	-23.4	0.998306	-21.38	163	GO:0030016,GO:0043231,GO:0008092,	K12567|1|9e-58|227|bta:540561|titin [EC:2.7.11.1]	gi|60360554|dbj|BAD90521.1|	0	mKIAA4170 protein [Mus musculus]

mmu-let-7a-1-3p	NM_025812	-24.3	0.951499	-23.44	162	GO:0031981,GO:0003676,GO:0001071,GO:0006351,GO:0006325,	K09272|1|7e-11|70.9|tad:TRIADDRAFT_64155|structure-specific recognition protein 1!K10802|2|9e-11|70.5|dya:Dyak_GE17263|high mobility group	gi|148693919|gb|EDL25866.1|	0	high mobility group 20A, isoform CRA_b [Mus musculus]

mmu-let-7a-1-3p	NM_007540	-20.7	1	-28.95	159	GO:0030136,GO:0005167,GO:0023061,GO:0033555,GO:0045597,GO:0007405,GO:0031175,GO:0007610,GO:0051899,GO:0014070,GO:0008037,GO:0033273,GO:0016202,GO:0006935,GO:0006954,GO:0035295,GO:0009719,GO:0048169,GO:0043066,GO:00	K04355|1|2e-134|481|mmu:12064|brain-derived neurotrophic factor	gi|114326463|ref|NP_031566.4|	2.03E-138	brain-derived neurotrophic factor isoform 1 precursor [Mus musculus]
mmu-let-7a-1-3p	NM_175104	-20.3	1	-21.11	158	-	-	gi|148664700|gb|EDK97116.1|	0	RIKEN cDNA 2810012G03, isoform CRA_b [Mus musculus]
mmu-let-7a-1-3p	NM_001003910	-21	1	-20.83	157	GO:0044424,	-	gi|145966853|ref|NP_001003910.2|	0	tetratricopeptide repeat protein GNN isoform 3 [Mus musculus]

mmu-let-7a-1-3p	NM_022313	-20.7	0.999914	-20.14	155	GO:0019866,GO:0044429,GO:0043021,GO:0032561,GO:0003723,GO:0022618,	-	gi|165377197|ref|NP_071708.2|	0	GTPase Era, mitochondrial [Mus musculus]
mmu-let-7a-1-3p	NM_198416	-21.8	1	-22.28	154	GO:0043231,GO:0046914,GO:0003676,GO:0006351,	-	gi|124053447|ref|NP_940808.2|	0	ZZ-type zinc finger-containing protein 3 [Mus musculus]
mmu-let-7a-1-3p	NM_178736	-23.1	0.998661	-20.9	153	GO:0043232,GO:0008047,GO:0051707,GO:0043087,GO:0002697,GO:0006897,	-	gi|283436077|ref|NP_848851.3|	3.10E-172	ELMO domain-containing protein 2 [Mus musculus]
mmu-let-7a-1-3p	NM_145590	-23.2	0.974681	-22.81	146	GO:0031224,	K14382|1|2e-24|88.2|bta:399680|solute carrier family 5 (sodium/glucose cotransporter), member 2	gi|225579041|ref|NP_663565.2|	0	UPF0420 protein C16orf58 homolog [Mus musculus]
mmu-let-7a-1-3p	NM_181649	NA	NA	-21.2	151	GO:0044464,GO:0005488,	-	gi|194473718|ref|NP_857632.4|	3.31E-133	coiled-coil domain-containing protein 75 [Mus musculus]

mmu-let-7a-1-3p	NM_021452	NA	NA	-20.24	150	GO:0005887,GO:0005488,GO:0005227,GO:0001508,GO:0001775,GO:0050804,GO:0009593,GO:0042310,GO:0007599,GO:0034220,GO:0030001,	K04941|1|8e-114|410|mmu:58802|potassium large conductance calcium-activated channel subfamily M beta member 4	gi|10946834|ref|NP_067427.1|	5.61E-113	calcium-activated potassium channel subunit beta-4 [Mus musculus]
mmu-let-7a-1-3p	NR_073378	NA	NA	-20.7	149	-	-	-	-	-
mmu-let-7a-1-3p	NM_001128181	-20.2	1	NA	NA	GO:0044459,GO:0044430,GO:0005515,GO:0007267,	K15008|1|0.0|1041|cfa:480194|discs, large-associated protein 1	gi|189491682|ref|NP_001121653.1|	0	disks large-associated protein 1 isoform 4 [Mus musculus]
mmu-let-7a-1-3p	NM_001025311	-21.4	0.999418	NA	NA	GO:0031228,GO:0008373,GO:0001573,GO:0006464,	K03376|1|4e-175|615|mmu:50935|N-acetylgalactosaminide alpha-2,6-sialyltransferase (sialyltransferase 7F) [EC:2.4.99.	gi|70778764|ref|NP_001020482.1|	0	alpha-N-acetylgalactosaminide alpha-2,6-sialyltransferase 6 isoform 3 [Mus musculus]

mmu-let-7a-1-3p	NM_027547	-20.4	0.999988	NA	NA	GO:0043231,GO:0046914,GO:0043565,GO:0008134,GO:0034968,GO:0006476,GO:0007049,GO:0010467,	K09228|1|6e-93|342|api:100159445|KRAB domain-containing zinc finger protein	gi|124286828|ref|NP_081823.2|	0	PR domain zinc finger protein 5 [Mus musculus]
mmu-let-7a-1-3p	NM_001142948	-20.6	1	NA	NA	GO:0043231,GO:0003676,GO:0006351,GO:0032501,	K09228|1|8e-14|81.6|ssc:100737868|KRAB domain-containing zinc finger protein	gi|219273495|ref|NP_001136420.1|	0	pogo transposable element with KRAB domain isoform 2 [Mus musculus]
mmu-let-7a-1-3p	NR_045280	-23.4	0.561095	NA	NA	-	-	gi|148676890|gb|EDL08837.1|	2.05E-40	mCG145115 [Mus musculus]
mmu-let-7a-1-3p	NM_013780	-24.1	0.994596	NA	NA	GO:0031981,GO:0003676,GO:0060089,GO:0060746,GO:0023052,GO:0051705,GO:00063	K09098|1|0.0|1507|mmu:27386|neuronal PAS domain-containing protein 1/3	gi|154090947|ref|NP_038808.2|	0	neuronal PAS domain-containing protein 3 [Mus musculus]
mmu-let-7a-1-3p	NM_019967	-23.8	0.971875	NA	NA	GO:0044424,GO:0005488,GO:0022402,GO:0051726,	-	gi|85677501|ref|NP_064351.2|	0	deleted in bladder cancer protein 1 homolog precursor [Mus musculus]

mmu-let-7a-1-3p	NM_178698	-25.6	0.751908	NA	NA	GO:0031090,GO:0043231,GO:0031224,GO:0000030,GO:0006506,	K07542|1|0.0|832|rno:366478|phosphatidylinositol glycan, class V [EC:2.4.1.-]	gi|26338704|dbj|BAC33023.1|	0	unnamed protein product [Mus musculus]
mmu-let-7a-1-3p	NM_001163085	-23.5	0.994374	NA	NA	GO:0043169,GO:0004702,GO:0032559,GO:0006464,GO:0023052,	K13986|1|0.0|2426|mmu:270672|mitogen-activated protein kinase kinase kinase 15 [EC:2.7.11.25]	gi|282847404|ref|NP_001156557.2|	0	mitogen-activated protein kinase kinase kinase 15 [Mus musculus]
mmu-let-7a-1-3p	NM_198656	-20.2	1	NA	NA	GO:0031224,GO:0046872,GO:0016337,	K06736|1|2e-80|301|mdo:100010120|cadherin 2, type 1, N-	gi|38348546|ref|NP_941058.1|	0	cadherin-like protein 26 precursor [Mus musculus]

mmu-let-7a-1-3p	NM_008716	-21.6	1	NA	NA	GO:0044432,GO:0016021,GO:0031981,GO:0044431,GO:0005488,GO:0045596,GO:0044267,GO:0007420,GO:0045165,GO:0006351,GO:0007166,	K02599|1|0.0|4566|mmu:18131|Notch	gi|6679096|ref|NP_032742.1|	0	neurogenic locus notch homolog protein 3 precursor [Mus musculus]
mmu-let-7a-1-3p	NM_008359	-20.7	1	NA	NA	GO:0016021,GO:0004896,GO:0001775,GO:0001819,GO:0007165,	K05164|1|0.0|1596|mmu:16172|interleukin 17 receptor	gi|6680411|ref|NP_032385.1|	0	interleukin-17 receptor A precursor [Mus musculus]
mmu-let-7a-1-3p	NM_001081394	-25.4	0.847216	NA	NA	GO:0031224,	K12662|1|2e-11|72.4|smm:Smp_169060|U4/U6 small nuclear ribonucleoprotein PRP4	gi|124487091|ref|NP_001074863.1|	0	transmembrane protein 248 [Mus musculus]

mmu-let-7a-1-3p	NM_001195544	-20.5	0.990218	NA	NA	GO:0031224,	K06839|1|7e-13|75.1|tca:660980|slit 2!K06838|3|3e-12|73.2|ptr:100609860|slit 1	gi|306966158|ref|NP_001182473.1|	4.81E-123	leucine-rich repeat-containing protein 3C precursor [Mus musculus]
mmu-let-7a-1-3p	NM_001163672	-23.9	0.990445	NA	NA	GO:0031981,GO:0044444,GO:0016505,GO:0032559,GO:0019901,GO:0016909,GO:0070555,GO:0010557,GO:0010243,GO:0016567,GO:0010742,GO:0031328,GO:0010952,GO:0000904,GO:0009411,GO:0032305,GO:0043408,GO:0009605,GO:0001516,GO:0001819,GO:0007399,GO:00066	K04440|1|0.0|768|rno:50658|c-Jun N-terminal kinase [EC:2.7.11.24]	gi|8394233|ref|NP_059018.1|	0	mitogen-activated protein kinase 9 isoform 1 [Rattus norvegicus]

mmu-let-7a-1-3p	NM_144837	-20.9	1	NA	NA	-	-	gi|256818804|ref|NP_659086.2|	0	uncharacterized protein LOC218333 [Mus musculus]
mmu-let-7a-1-3p	NM_001163572	-21.2	1	NA	NA	GO:0031224,	-	gi|254675263|ref|NP_001157044.1|	2.73E-66	transmembrane protein 170B [Mus musculus]
mmu-let-7a-1-3p	NM_001033135	-20.3	0.999998	NA	NA	GO:0016020,GO:0046872,	K15704|1|0.0|694|mmu:67702|E3 ubiquitin-protein ligase RNF149 [EC:6.3.2.19]	gi|224809379|ref|NP_001028307.2|	0	E3 ubiquitin-protein ligase RNF149 precursor [Mus musculus]

mmu-let-7a-1-3p	NM_001165902	-20.8	0.999999	NA	NA	GO:0043231,GO:0005902,GO:0005815,GO:0005913,GO:0019897,GO:0031252,GO:0071664,GO:0031674,GO:0030425,GO:0046332,GO:0019902,GO:0008134,GO:0019900,GO:0043566,GO:0035258,GO:0050839,GO:0001071,GO:0003713,GO:0035254,GO:0060089,GO:0000975,GO:00435	K02105|1|0.0|1522|mmu:12387|catenin beta 1	gi|6671684|ref|NP_031640.1|	0	catenin beta-1 [Mus musculus]

mmu-let-7a-1-3p	NM_001035854	-22	0.999994	NA	NA	GO:0030119,GO:0044431,GO:0030139,GO:0012505,GO:0030136,GO:0005515,GO:0022892,GO:0007267,GO:0006901,GO:0015031,GO:0007173,GO:0050688,GO:00069	K11825|1|0.0|1737|mmu:71770|AP-2 complex subunit beta-1	gi|78711838|ref|NP_001030931.1|	0	AP-2 complex subunit beta isoform a [Mus musculus]
mmu-let-7a-1-3p	NM_207654	-25.6	0.916534	NA	NA	GO:0044459,GO:0031225,GO:0044421,GO:0005102,GO:0007411,GO:0007417,GO:00071	K05462|1|1e-125|452|aml:100465489|ephrin-A	gi|46560570|ref|NP_997537.1|	1.44E-125	ephrin-A5 isoform 1 precursor [Mus musculus]
mmu-let-7a-1-3p	NR_030693	-21.9	0.999936	NA	NA	-	-	gi|148673719|gb|EDL05666.1|	1.05E-56	mCG145028 [Mus musculus]
mmu-let-7a-1-3p	NM_201367	-21.9	0.999928	NA	NA	GO:0016021,GO:0004888,GO:0007166,GO:0007267,	K08442|1|0.0|988|mmu:381413|G protein-coupled receptor 176	gi|148695958|gb|EDL27905.1|	0	G protein-coupled receptor 176 [Mus musculus]

mmu-let-7a-1-3p	NM_001111015	-23.8	0.97156	NA	NA	GO:0008021,GO:0044459,GO:0005515,GO:0003824,GO:0032559,GO:0050804,GO:00015	-	gi|149049703|gb|EDM02157.1|	0	synapsin II, isoform CRA_a [Rattus norvegicus]
mmu-let-7a-1-3p	NM_021536	-20.3	1	NA	NA	GO:0031306,GO:0046872,GO:0032561,GO:0003824,GO:0012501,GO:0035556,GO:00346	K07870|1|0.0|1281|rno:303351|Ras homolog gene family, member T1	gi|148683684|gb|EDL15631.1|	0	ras homolog gene family, member T1, isoform CRA_b [Mus musculus]
mmu-let-7a-1-3p	NM_025937	-20.5	1	NA	NA	GO:0031981,GO:0005488,GO:0045582,	-	gi|301789639|ref|XP_002930232.1|	1.34E-86	PREDICTED: LOW QUALITY PROTEIN: NKAP-like protein-like [Ailuropoda melanoleuca]
mmu-let-7a-1-3p	NR_027803	-21.3	0.999902	NA	NA	-	-	gi|148678715|gb|EDL10662.1|	7.26E-46	mCG1027169, isoform CRA_a [Mus musculus]
mmu-let-7a-1-3p	NM_133919	-20	1	NA	NA	GO:0043231,GO:0001071,GO:0006355,	K15184|1|0.0|1660|mmu:17355|AF4/FMR2 family member 1	gi|124248527|ref|NP_598680.3|	0	AF4/FMR2 family member 1 isoform 2 [Mus musculus]

mmu-let-7a-1-3p	NM_178601	-20.1	0.999999	NA	NA	GO:0032991,GO:0031981,GO:0016876,GO:0032559,GO:0042254,GO:0010467,	K14561|1|2e-155|550|rno:316317|U3 small nucleolar ribonucleoprotein protein	gi|148682503|gb|EDL14450.1|	1.67E-154	IMP4, U3 small nucleolar ribonucleoprotein, homolog (yeast) [Mus musculus]
mmu-let-7a-1-3p	NM_080428	-24.6	0.937175	NA	NA	GO:0031981,GO:0031461,GO:0005488,GO:0001071,GO:0043161,GO:0032446,GO:0051704,GO:0007059,GO:0007166,GO:00109	K10260|1|0.0|1210|mdo:100617365|F-box and WD-40 domain protein 7	gi|148683452|gb|EDL15399.1|	0	F-box and WD-40 domain protein 7, archipelago homolog (Drosophila), isoform CRA_a [Mus musculus]
mmu-let-7a-1-3p	NM_207683	-20.3	1	NA	NA	GO:0043231,GO:0043234,GO:0012505,GO:0016020,GO:0005543,GO:0016307,GO:0035004,GO:0032559,GO:0046488,GO:00480	K00923|1|0.0|2628|rno:116720|phosphatidylinositol-4-phosphate 3-kinase [EC:2.7.1.154]	gi|46810282|ref|NP_997566.1|	0	phosphatidylinositol 4-phosphate 3-kinase C2 domain-containing subunit gamma isoform 1 [Mus musculus]
mmu-let-7a-1-3p	NR_040637	-20	1	NA	NA	-	-	gi|148695815|gb|EDL27762.1|	8.52E-59	mCG146272 [Mus musculus]

mmu-let-7a-1-3p	NM_001042591	-21.7	0.99998	NA	NA	GO:0044424,GO:0005488,GO:0023052,	K15183|1|1e-07|60.1|mgp:100549853|RNA polymerase II elongation factor ELL	gi|28972730|dbj|BAC65781.1|		0	mKIAA1376 protein [Mus musculus]
mmu-let-7a-1-3p	NM_021337	-20.9	1	NA	NA	GO:0043231,GO:0003724,GO:0003676,GO:0042623,GO:0032559,	K12599|1|0.0|2274|mmu:108077|antiviral helicase SKI2 [EC:3.6.4.-	gi|148694814|gb|EDL26761.1|		0	mCG15924, isoform CRA_i [Mus musculus]
mmu-let-7a-1-3p	NM_001080798	-20	1	NA	NA	GO:0043231,GO:0001071,GO:0006355,	K15184|1|0.0|1688|mmu:17355|AF4/FMR2 family member 1	gi|124248529|ref|NP_001074267.1|		0	AF4/FMR2 family member 1 isoform 1 [Mus musculus]
mmu-let-7a-1-3p	NM_001102458	-20.9	1	NA	NA	GO:0044444,GO:0008047,GO:0005488,GO:0004857,GO:0019222,GO:0006595,GO:00065	K01581|1|5e-123|444|xla:379859|ornithine decarboxylase [EC:4.1.1.17]	gi|9055298|ref|NP_061215.1|		0	antizyme inhibitor 1 [Mus musculus]

mmu-let-7a-1-3p	NM_021495	-22.4	0.997959	NA	NA	GO:0005911,GO:0031224,GO:0042802,GO:0002088,GO:0045216,GO:0016337,GO:0009653,GO:0019953,	K06592|1|0.0|1037|mmu:58998|poliovirus receptor-related protein 3	gi|12957492|ref|NP_067470.1|	0	poliovirus receptor-related protein 3 isoform alpha [Mus musculus]
mmu-let-7a-1-3p	NM_001033348	-25.1	0.994722	NA	NA	GO:0043231,GO:0044444,GO:0005099,GO:0046983,GO:0032852,	K07207|1|2e-22|110|bfo:BRAFLDRAFT_64809|tuberous sclerosis 2	gi|158187505|ref|NP_001028520.2|	0	ral GTPase-activating protein subunit alpha-2 [Mus musculus]
mmu-let-7a-1-3p	NM_021345	-22.4	0.996122	NA	NA	GO:0031224,GO:0043231,GO:0031090,GO:0008047,GO:0005488,GO:0003824,GO:0007265,GO:0006631,GO:0000187,	K10703|1|4e-22|107|bta:613886|3-hydroxyacyl-CoA dehydratase [EC:4.2.1.-]	gi|171184435|ref|NP_067320.2|	0	3-hydroxyacyl-CoA dehydratase 3 [Mus musculus]
mmu-let-7a-1-3p	NM_174996	-20.2	1	NA	NA	-	-	gi|395862204|ref|XP_003803351.1|	1.09E-72	PREDICTED: uncharacterized protein LOC100960564 [Otolemur garnettii]
mmu-let-7a-1-3p	NM_026782	-21.2	0.993674	NA	NA	GO:0031224,	-	gi|148690682|gb|EDL22629.1|	1.82E-54	mCG21143, isoform CRA_a [Mus musculus]

mmu-let-7a-1-3p	NM_138648	-21.9	0.999855	NA	NA	GO:0031224,GO:0030169,GO:0006952,GO:0016337,GO:0000302,GO:0044260,	K08763|1|5e-177|622|mmu:108078|oxidised low-density lipoprotein receptor 1	gi|148667512|gb|EDK99928.1|	2.38E-177	oxidized low density lipoprotein (lectin-like) receptor 1 [Mus musculus]
mmu-let-7a-1-3p	NM_028720	-22.5	0.998296	NA	NA	GO:0043231,GO:0003676,GO:0042393,GO:0016616,GO:0048037,GO:0006007,	K00020|1|0.0|1071|mcc:706933|3-hydroxyisobutyrate dehydrogenase [EC:1.1.1.31]	gi|119605657|gb|EAW85251.1|	0	cytokine-like nuclear factor n-pac, isoform CRA_a [Homo sapiens]
mmu-let-7a-1-3p	NM_001167680	-20.5	1	NA	NA	GO:0031090,GO:0043231,GO:0031224,GO:0004175,GO:0019538,	K02857|1|3e-09|65.5|nvi:100122487|rhomboid-related protein 1/2/3 [EC:3.4.21.	gi|264681541|ref|NP_766160.2|	0	inactive rhomboid protein 2 [Mus musculus]

mmu-let-7a-1-3p	NM_145708	-20.8	0.997456	NA	NA	GO:0043231,GO:0003676,	K09296|1|2e-06|40.0|mdo:100026219|homeobox protein HoxA/C/D11!K09311|3|4e-06|36.6|tca:641600|Antp family, other!K09312|5|6e-06|52.8|mdo:100024158|homeobox protein CDX	gi|84579823|ref|NP_001033765.1|	1.05E-95	uncharacterized protein LOC194588 [Mus musculus]
mmu-let-7a-1-3p	NM_001242360	-22.9	0.955715	NA	NA	GO:0043231,GO:0003723,GO:0070566,GO:0032559,GO:0008033,GO:0006605,	K00974|1|8e-115|415|mmu:70047|tRNA nucleotidyltransferase (CCA-adding enzyme) [EC:2.7.7.72 3.1.3.- 3.1.4.-]	gi|334358911|ref|NP_001229289.1|	4.46E-121	CCA tRNA nucleotidyltransferase 1, mitochondrial isoform b [Mus musculus]

mmu-let-7a-1-3p	NM_013540	-25.9	0.938616	NA	NA	GO:0030427,GO:0008328,GO:0044430,GO:0030425,GO:0030139,GO:0043025,GO:0031090,GO:0008021,GO:0033267,GO:0005231,GO:0004970,GO:0019904,GO:0016247,GO:0019900,GO:0006898,GO:0010038,GO:0050804,GO:0009636,	K05198|1|0.0|1708|mdo:100023645|glutamate receptor 2	gi|355687686|gb|EHH26270.1|	0	hypothetical protein EGK_16189 [Macaca mulatta]
mmu-let-7a-1-3p	NM_139269	-20.3	1	NA	NA	GO:0005737,GO:0016020,GO:0005488,GO:0016787,GO:0051726,	K00678|1|5e-07|58.2|xtr:394746|phosphatidylcholine-retinol O-acyltransferase [EC:2.3.1.1	gi|148701366|gb|EDL33313.1|	1.56E-64	HRAS like suppressor 3, isoform CRA_b [Mus musculus]

mmu-let-7a-1-3p	NM_175137	-20.1	1	NA	NA	-	K01873|1|0.0|2132|mmu:68915|valyl-tRNA synthetase [EC:6.1.1.9]	gi|160333671|ref|NP_780346.3|	0	valine--tRNA ligase, mitochondrial precursor [Mus musculus]
mmu-let-7a-1-3p	NM_013793	-21.3	0.976548	NA	NA	GO:0016020,GO:0060089,	K10069|1|1e-15|84.7|mcc:717526|C-type lectin domain family 1, member A!K08763|2|1e-15|84.3|acs:100564370|oxidised low-density lipoprotein receptor 1	gi|7549771|ref|NP_038821.2|	5.77E-156	killer cell lectin-like receptor, subfamily A, member 15 [Mus musculus]

mmu-let-7a-1-3p	NM_001081401	-20.5	1	NA	NA	GO:0031012,GO:0004175,GO:0046914,GO:0005539,GO:0007166,GO:0032963,GO:0006508,GO:0030198,	K08619|1|0.0|2476|mmu:330119|a disintegrin and metalloproteinase with thrombospondin motifs 3 [EC:3.4.24.-]	gi|124487141|ref|NP_001074870.1|	0	a disintegrin-like and metalloprotease with thrombospondin type 1 motif, 3 isoform 2 precursor [Mus musculus]
mmu-let-7a-1-3p	NM_007881	-20.7	1	NA	NA	GO:0043231,GO:0003712,GO:0005102,GO:0019901,GO:0019748,GO:0050767,	K05626|1|0.0|1175|mmu:13498|atrophin-1 (dentatorubral-pallidoluysian atrophy protein)	gi|34328110|ref|NP_031907.2|	0	atrophin-1 [Mus musculus]
mmu-let-7a-1-3p	NM_172586	-20.8	1	NA	NA	GO:0043231,GO:0046914,GO:0003676,GO:0006351,	K09228|1|8e-98|360|mmu:626316|KRAB domain-containing zinc finger protein	gi|27369830|ref|NP_766174.1|	0	zinc finger protein 322 [Mus musculus]

mmu-let-7a-1-3p	NM_013508	-20.1	1	NA	NA	GO:0031981,GO:0003712,GO:0001071,GO:0043565,GO:0048646,GO:0009611,GO:0023052,GO:0006351,	K09430|1|5e-163|576|mmu:13713|ETS domain-containing protein Elk-3 (SRF accessory protein 2)	gi|157909833|ref|NP_038536.2|	3.39E-162	ETS domain-containing protein Elk-3 isoform a [Mus musculus]
mmu-let-7a-1-3p	NM_025730	-23.1	0.999973	NA	NA	GO:0044431,GO:0044455,GO:0030136,GO:0032561,GO:0004712,GO:0008047,GO:0004674,GO:0042802,GO:0032559,GO:0012501,GO:0010259,GO:0006468,GO:0016567,GO:0006950,GO:0000165,	K08844|1|0.0|4778|mmu:66725|leucine-rich repeat kinase 2 [EC:2.7.11.1]	gi|146231954|ref|NP_080006.3|	0	leucine-rich repeat serine/threonine-protein kinase 2 [Mus musculus]
mmu-let-7a-1-3p	NM_012045	-21.4	0.999625	NA	NA	GO:0046872,GO:0004091,GO:0019637,GO:0006629,	K01047|1|1e-116|421|mmu:26971|phospholipase A2 [EC:3.1.1.4	gi|38454186|ref|NP_036175.2|	9.81E-116	group IIF secretory phospholipase A2 [Mus musculus]

mmu-let-7a-1-3p	NM_007395	-21.8	0.999808	NA	NA	GO:0016021,GO:0043235,GO:0032559,GO:0043169,GO:0017002,GO:0032403,GO:0019899,GO:0001934,GO:0008544,GO:0006357,GO:0045137,GO:0045639,GO:0043009,GO:0022402,GO:0012502,GO:0007399,GO:0016049,GO:0032924,	K13567|1|0.0|937|mmu:11479|activin receptor type-1B [EC:2.7.11.30]	gi|6680630|ref|NP_031421.1|	0	activin receptor type-1B precursor [Mus musculus]
mmu-let-7a-1-3p	NM_029509	-21.7	0.99902	NA	NA	-	-	gi|83776551|ref|NP_083785.3|	0	guanylate binding protein 8 [Mus musculus]
mmu-let-7a-1-3p	NR_027952	-30.9	0.098831	NA	NA	-	-	gi|148706443|gb|EDL38390.1|	2.74E-60	mCG146330, isoform CRA_b [Mus musculus]
mmu-let-7a-1-3p	NR_029729	-28.7	0.001006	NA	NA	-	-	-	-	-

mmu-let-7a-1-3p	NM_153538	-21.4	1	NA	NA	-	K13291|1|0.0|2665|mmu:214290|terminal uridylyltransferase [EC:2.7.7.52]	gi|148709347|gb|EDL41293.1|	0	zinc finger, CCHC domain containing 6, isoform CRA_b [Mus musculus]
mmu-let-7a-1-3p	NM_013463	-20	1	NA	NA	GO:0000323,GO:0048029,GO:0042802,GO:0043167,GO:0015925,GO:0001503,GO:0006687,GO:0016137,GO:0006677,GO:0031327,GO:0032769,	K01189|1|0.0|893|mmu:11605|alpha-galactosidase [EC:3.2.1.22]	gi|133778924|ref|NP_038491.2|	0	alpha-galactosidase A [Mus musculus]

mmu-let-7a-1-3p	NM_028898	-21	1	NA	NA	GO:0043234,GO:0043005,GO:0000323,GO:0044297,GO:0019900,GO:0009967,GO:0035556,GO:0022402,GO:0031667,GO:0040007,GO:0032535,GO:0007169,GO:0045860,GO:00	K07204|1|0.0|2623|mmu:74370|regulatory associated protein of mTOR	gi|133777706|gb|AAI18972.1|	0	4932417H02Rik protein [Mus musculus]
mmu-let-7a-1-3p	NR_045433	-22.7	0.596212	NA	NA	-	-	gi|148670143|gb|EDL02090.1|	9.32E-47	mCG144944 [Mus musculus]
mmu-let-7a-1-3p	NM_001163512	-21.1	0.999995	NA	NA	GO:0000228,GO:0016020,GO:0008047,GO:0004871,GO:0060589,GO:0007186,	K13765|1|8e-18|94.0|bta:281453|regulator of G-protein signaling 9	gi|254553475|ref|NP_001156984.1|	0	regulator of G-protein signaling 12 isoform B [Mus musculus]
mmu-let-7a-1-3p	NM_028779	-21.8	0.999917	NA	NA	GO:0044444,GO:0043169,GO:0016814,GO:0009112,GO:0009127,	K01490|1|0.0|1593|mmu:109674|AMP deaminase [EC:3.5.4.6]	gi|21311925|ref|NP_083055.1|	0	AMP deaminase 2 [Mus musculus]

mmu-let-7a-1-3p	NM_026444	-21.1	0.999967	NA	NA	GO:0044429,GO:0046912,GO:0009060,GO:0005975,GO:0072350,GO:0006732,GO:00436	K01647|1|0.0|917|mmu:12974|citrate synthase [EC:2.3.3.1]	gi|13385942|ref|NP_080720.1|	0	citrate synthase, mitochondrial precursor [Mus musculus]
mmu-let-7a-1-3p	NM_009622	-23	1	NA	NA	GO:0043231,GO:0031224,GO:0005515,GO:0004016,GO:0032559,GO:0043169,GO:0015980,GO:0007267,GO:0007613,GO:0045762,GO:0033762,GO:0032147,GO:0010863,GO:0007169,GO:0048667,GO:0007188,GO:00	K08041|1|0.0|1955|mmu:432530|adenylate cyclase 1 [EC:4.6.1.1]	gi|61888832|ref|NP_033752.1|	0	adenylate cyclase type 1 [Mus musculus]

mmu-let-7a-1-3p	NM_025682	-22.5	0.990335	NA	NA	GO:0016604,GO:0003676,GO:0006351,	K13219|1|4e-108|393|mdo:100031251|splicing factor, proline- and glutamine-rich!K13214|2|7e-108|392|mdo:100011224|non-POU domain-containing octamer-binding	gi|225543409|ref|NP_079958.3|	0	paraspeckle component 1 [Mus musculus]
mmu-let-7a-1-3p	NM_020267	-20.8	1	NA	NA	GO:0044464,GO:0046914,	K12020|1|6e-98|360|mmu:80985|tripartite motif-containing protein 44	gi|148695720|gb|EDL27667.1|	3.94E-97	tripartite motif-containing 44, isoform CRA_a [Mus musculus]
mmu-let-7a-1-3p	NM_133744	-20.4	1	NA	NA	-	-	gi|19526948|ref|NP_598505.1|	0	coiled-coil domain-containing protein 71 [Mus musculus]

mmu-let-7a-2-3p	NM_001003917	-25.4	0.903983	-25.65	162	GO:0031224,GO:0005774,GO:0031982,GO:0005770,GO:0006810,GO:0044248,GO:00070	-	gi|86990446|ref|NP_001003917.2|	0	autophagy-related protein 9A [Mus musculus]
mmu-let-7a-2-3p	NM_001004182	-25.6	0.829493	-27.13	153	-	-	gi|51921359|ref|NP_001004182.1|	1.03E-80	uncharacterized protein LOC434008 [Mus musculus]
mmu-let-7a-2-3p	NM_001024504	-28.5	0.388458	-26.58	160	-	K10624|1|6e-40|167|ame:724670|E3 ubiquitin-protein ligase RBBP6 [EC:6.3.2.19]	gi|111162659|ref|NP_001019675.2|	1.35E-149	DCN1-like protein 2 isoform a [Mus musculus]

mmu-let-7a-2-3p	NM_001037539	-27	0.331976	-25.49	160	GO:0005102,GO:0042221,GO:0048511,GO:0051480,GO:0048232,GO:0007186,GO:0007600,GO:0006939,GO:0048646,GO:0006952,GO:0043066,GO:0000165,	-	gi|82830395|ref|NP_001032628.1|	4.91E-50	prokineticin-2 isoform 2 precursor [Mus musculus]
mmu-let-7a-2-3p	NM_001038699	-29.4	0.352697	-25.51	163	GO:0044444,GO:0016301,GO:0006520,GO:0006040,GO:0006796,	K15522|1|3e-116|421|mmu:63828|protein-fructosamine 3-kinase [EC:2.7.1.1	gi|84662727|ref|NP_001033788.1|	2.16E-115	fructosamine-3-kinase isoform b [Mus musculus]
mmu-let-7a-2-3p	NM_001042649	-28.5	0.379187	-26.58	160	-	K10624|1|1e-31|94.4|ame:724670|E3 ubiquitin-protein ligase RBBP6 [EC:6.3.2.19]	gi|148690187|gb|EDL22134.1|	2.05E-133	mCG3700, isoform CRA_e [Mus musculus]

mmu-let-7a-2-3p	NM_001042650	-28.5	0.368158	-26.58	160	-	K10624|1|1e-17|93.2|ame:724670|E3 ubiquitin-protein ligase RBBP6 [EC:6.3.2.19]	gi|148690187|gb|EDL22134.1|	8.48E-138	mCG3700, isoform CRA_e [Mus musculus]
mmu-let-7a-2-3p	NM_001042651	-28.5	0.377608	-26.58	160	-	K10624|1|1e-24|109|ame:724670|E3 ubiquitin-protein ligase RBBP6 [EC:6.3.2.19]	gi|148690187|gb|EDL22134.1|	2.91E-132	mCG3700, isoform CRA_e [Mus musculus]
mmu-let-7a-2-3p	NM_001042707	-37.8	0.009843	-34.75	177	GO:0032991,GO:0031981,GO:0003723,GO:0001071,GO:0005515,GO:0006355,GO:00082	K13090|1|0.0|1099|mmu:16201|interleukin enhancer-binding factor 3	gi|148693238|gb|EDL25185.1|	0	interleukin enhancer binding factor 3, isoform CRA_b [Mus musculus]

mmu-let-7a-2-3p	NM_001042708	-37.8	0.010804	-34.75	177	GO:0032991,GO:0031981,GO:0003723,GO:0001071,GO:0005515,GO:0006355,GO:0008213,GO:0022403,	K13090|1|0.0|1107|rno:84472|interleukin enhancer-binding factor 3	gi|148693239|gb|EDL25186.1|	0	interleukin enhancer binding factor 3, isoform CRA_c [Mus musculus]
mmu-let-7a-2-3p	NM_001042709	-37.8	0.010638	-34.75	177	GO:0032991,GO:0031981,GO:0003723,GO:0001071,GO:0005515,GO:0006355,GO:0008213,GO:0022403,	K13090|1|0.0|1099|mmu:16201|interleukin enhancer-binding factor 3	gi|148693239|gb|EDL25186.1|	0	interleukin enhancer binding factor 3, isoform CRA_c [Mus musculus]
mmu-let-7a-2-3p	NM_001081068	-31.6	0.308586	-26.15	175	GO:0003824,GO:0046914,	-	gi|50510603|dbj|BAD32287.1|	0	mKIAA0714 protein [Mus musculus]
mmu-let-7a-2-3p	NM_001085535	-34	0.021068	-32.24	177	GO:0016020,	-	gi|147905830|ref|NP_001079004.1|	2.75E-64	transmembrane protein 235 precursor [Mus musculus]

mmu-let-7a-2-3p	NM_001109757	-27.4	0.878716	-42.89	310	GO:0031224,GO:0005903,GO:0044459,GO:0005798,GO:0044297,GO:0005768,GO:0043682,GO:0032559,GO:0016531,GO:0005515,GO:0010467,GO:0021859,GO:0002294,GO:0035434,GO:0042415,GO:0030003,GO:0043009,GO:0043085,GO:0009072,GO:0085029,GO:0009207,GO:00435	K01533|1|0.0|2765|mmu:11977|Cu2+-exporting ATPase [EC:3.6.3.4]	gi|148682115|gb|EDL14062.1|	0	ATPase, Cu++ transporting, alpha polypeptide, isoform CRA_b [Mus musculus]

mmu-let-7a-2-3p	NM_001136062	-31.8	0.04111	-26.1	174	GO:0043234,GO:0044444,GO:0046872,GO:0042802,GO:0046983,GO:0016836,GO:0006007,GO:0042221,GO:0042246,	K01689|1|0.0|837|mmu:13808|enolase [EC:4.2.1.11]	gi|148680653|gb|EDL12600.1|	0	enolase 3, beta muscle, isoform CRA_a [Mus musculus]
mmu-let-7a-2-3p	NM_001145896	-29.2	0.563178	-25.89	167	GO:0005488,	-	gi|225543323|ref|NP_941073.2|	0	genetic suppressor element 1 isoform 1 [Mus musculus]
mmu-let-7a-2-3p	NM_001145897	-29.2	0.560058	-25.89	167	GO:0005488,	-	gi|225543323|ref|NP_941073.2|	0	genetic suppressor element 1 isoform 1 [Mus musculus]
mmu-let-7a-2-3p	NM_001146059	-28.5	0.574233	-29.19	157	GO:0031410,GO:0008047,GO:0005088,GO:0005515,GO:0006996,GO:0043087,GO:0033036,GO:0007266,	K04575|1|3e-139|498|mdo:100019560|amyotrophic lateral sclerosis 2 (juvenile)	gi|225690620|ref|NP_666340.3|	0	ALS2 C-terminal-like protein [Mus musculus]

mmu-let-7a-2-3p	NM_001146060	-28.5	0.580049	-29.19	157	GO:0031410,GO:0008047,GO:0005088,GO:0005515,GO:0006996,GO:0043087,GO:0033036,GO:0007266,	K04575|1|3e-139|498|mdo:100019560|amyotrophic lateral sclerosis 2 (juvenile)	gi|225690620|ref|NP_666340.3|	0	ALS2 C-terminal-like protein [Mus musculus]
mmu-let-7a-2-3p	NM_001159536	-30	0.338583	-28.41	160	GO:0043231,GO:0016021,GO:0005515,GO:0004016,GO:0032559,GO:0043169,GO:0015980,GO:0007267,GO:0045762,GO:0033762,GO:0032147,GO:0010863,GO:0007169,GO:0007126,GO:0007188,GO:0042044,GO:00	K08043|1|0.0|1968|mmu:104111|adenylate cyclase 3 [EC:4.6.1.1]	gi|28972253|dbj|BAC65580.1|	0	mKIAA0511 protein [Mus musculus]

mmu-let-7a-2-3p	NM_001159537	-30	0.342373	-28.41	160	GO:0043231,GO:0016021,GO:0005515,GO:0004016,GO:0032559,GO:0043169,GO:0015980,GO:0007267,GO:0045762,GO:0033762,GO:0032147,GO:0010863,GO:0007169,GO:0007126,GO:0007188,GO:0042044,GO:00	K08043|1|0.0|1968|mmu:104111|adenylate cyclase 3 [EC:4.6.1.1]	gi|28972253|dbj|BAC65580.1|	0	mKIAA0511 protein [Mus musculus]
mmu-let-7a-2-3p	NM_001159907	-29.1	0.482131	-47.32	310	GO:0043231,GO:0005488,GO:0016740,GO:0006355,	K03239|1|1e-103|379|cin:100175767|translation initiation factor eIF-2B subunit alpha	gi|229577450|ref|NP_001153379.1|	0	TAR (HIV-1) RNA binding protein 1 [Mus musculus]
mmu-let-7a-2-3p	NM_001163567	-25.6	0.958334	-43.64	313	-	-	gi|254675227|ref|NP_001157039.1|	0	protein FAM102B [Mus musculus]

mmu-let-7a-2-3p	NM_001163754	-28.4	0.704928	-25.66	159	GO:0044424,GO:0008047,GO:0046983,GO:0033124,GO:0015031,	-	gi|255003810|ref|NP_001157226.1|	0	rab3 GTPase-activating protein non-catalytic subunit [Mus musculus]
mmu-let-7a-2-3p	NM_001164604	-26.3	0.683245	-26.05	158	GO:0043231,GO:0046914,GO:0003824,GO:0006259,GO:0033554,	-	gi|148697381|gb|EDL29328.1|	2.46E-62	non-SMC element 2 homolog (MMS21, S. cerevisiae), isoform CRA_a [Mus musculus]
mmu-let-7a-2-3p	NM_001168292	-26.2	0.832584	-25.78	162	GO:0005488,	K02360|1|1e-26|123|aag:AaeL_AAEL009189|a component of the cytoplasm (encore)	gi|28972564|dbj|BAC65698.1|	0	mKIAA1002 protein [Mus musculus]
mmu-let-7a-2-3p	NM_001168293	-26.2	0.847712	-25.78	162	GO:0043231,GO:0005488,	K02360|1|5e-28|128|aag:AaeL_AAEL009189|a component of the cytoplasm (encore)	gi|28972564|dbj|BAC65698.1|	0	mKIAA1002 protein [Mus musculus]

mmu-let-7a-2-3p	NM_001170419	-27	0.302658	-25.49	160	GO:0005102,GO:0048646,GO:0007186,GO:0048511,GO:0042221,GO:0006939,GO:0048232,GO:0043066,	-	gi|281371486|ref|NP_001163890.1|	5.01E-22	prokineticin-2 isoform 3 precursor [Mus musculus]
mmu-let-7a-2-3p	NM_001177759	-27.7	0.523166	-25.64	160	GO:0044424,GO:0004869,GO:0005488,GO:0010466,	-	gi|295444881|ref|NP_001171230.1|	1.90E-101	tumor necrosis factor alpha-induced protein 8 isoform 2 [Mus musculus]
mmu-let-7a-2-3p	NM_001177760	-27.7	0.425342	-25.64	160	GO:0044424,GO:0004869,GO:0005488,GO:0010466,	-	gi|295444883|ref|NP_001171231.1|	5.06E-67	tumor necrosis factor alpha-induced protein 8 isoform 3 [Mus musculus]
mmu-let-7a-2-3p	NM_001252292	-27.8	0.438229	-47.05	309	GO:0031090,GO:0043231,GO:0031224,GO:0005488,GO:0003824,GO:0033189,GO:0009888,GO:0019915,	-	gi|356582503|ref|NP_001239221.1|	0	mesoderm-specific transcript protein isoform 1 [Mus musculus]

mmu-let-7a-2-3p	NM_001252293	-28.7	0.288363	-47.05	309	GO:0031090,GO:0043231,GO:0031224,GO:0005488,GO:0003824,GO:0033189,GO:0009888,GO:0019915,	-	gi|356582503|ref|NP_001239221.1|	0	mesoderm-specific transcript protein isoform 1 [Mus musculus]
mmu-let-7a-2-3p	NM_007390	-30.6	0.114657	-25.14	170	-	K04809|1|0.0|976|mmu:11441|nicotinic acetylcholine receptor alpha-7	gi|156766082|ref|NP_031416.3|	0	neuronal acetylcholine receptor subunit alpha-7 precursor [Mus musculus]
mmu-let-7a-2-3p	NM_007933	-31.8	0.043793	-26.1	174	GO:0043234,GO:0044444,GO:0046872,GO:0042802,GO:0046983,GO:0016836,GO:0006007,GO:0042221,GO:0042246,	K01689|1|0.0|837|mmu:13808|enolase [EC:4.2.1.11]	gi|148680653|gb|EDL12600.1|	0	enolase 3, beta muscle, isoform CRA_a [Mus musculus]
mmu-let-7a-2-3p	NM_007993	-25.8	0.991223	-45.39	308	GO:0043205,GO:0046872,GO:0005198,GO:0030514,GO:0030512,GO:0048513,	K06825|1|0.0|6138|mmu:14118|fibrillin 1	gi|118197277|ref|NP_032019.2|	0	fibrillin-1 precursor [Mus musculus]

mmu-let-7a-2-3p	NM_008135	-28.7	0.384974	-29.11	159	GO:0016021,GO:0005488,GO:0015175,GO:0005283,GO:0005326,GO:0015804,GO:0035725,	K05038|1|0.0|1275|mmu:14664|solute carrier family 6 (neurotransmitter transporter, amino acid) member 5/7/9/14	gi|148698593|gb|EDL30540.1|	0	solute carrier family 6 (neurotransmitter transporter, glycine), member 9, isoform CRA_a [Mus musculus]
mmu-let-7a-2-3p	NM_008397	-27.5	0.655734	-25.64	159	GO:0008305,GO:0009986,GO:0042995,GO:0005911,GO:0016323,GO:0005578,GO:0004871,GO:0005102,GO:0042476,GO:0016337,GO:0030035,GO:0006915,GO:0007044,GO:0002376,GO:0007599,GO:0007154,GO:0007166,GO:0045444,GO:00315	K06485|1|0.0|2134|mmu:16403|integrin alpha 6	gi|31982236|ref|NP_032423.2|	0	integrin alpha-6 precursor [Mus musculus]

mmu-let-7a-2-3p	NM_008590	-27.8	0.424299	-47.05	309	GO:0031090,GO:0043231,GO:0031224,GO:0005488,GO:0003824,GO:0033189,GO:0009888,GO:0019915,	-	gi|356582507|ref|NP_001239222.1|	0	mesoderm-specific transcript protein isoform 1 precursor [Mus musculus]
mmu-let-7a-2-3p	NM_008820	-28.6	0.229321	-25.94	157	GO:0008235,GO:0046914,GO:0019538,GO:0032963,GO:0019752,	K14213|1|0.0|982|mmu:18624|Xaa-Pro dipeptidase [EC:3.4.13.9]	gi|170650724|ref|NP_032846.2|	0	xaa-Pro dipeptidase [Mus musculus]
mmu-let-7a-2-3p	NM_008832	-25.2	0.981523	-45.6	316	GO:0043234,GO:0043227,GO:0044444,GO:0016020,GO:0005515,GO:0015926,GO:0070567,GO:0004683,GO:0006468,GO:0019318,GO:0006656,GO:0005977,	K07190|1|0.0|2407|mmu:18679|phosphorylase kinase alpha/beta subunit	gi|153945840|ref|NP_032858.2|	0	phosphorylase b kinase regulatory subunit alpha, skeletal muscle isoform isoform 1 [Mus musculus]

mmu-let-7a-2-3p	NM_009061	-30.3	0.196913	-25.45	174	GO:0043231,GO:0016020,GO:0008047,GO:0060089,GO:0005515,GO:0043087,GO:0007186,GO:0048232,GO:0045444,	K07524|1|3e-46|187|pon:100445111|regulator of G-protein signalling 3	gi|31543586|ref|NP_033087.2|	3.51E-118	regulator of G-protein signaling 2 [Mus musculus]
mmu-let-7a-2-3p	NM_009576	-27.3	0.67633	-25.74	158	GO:0043231,GO:0046914,GO:0003676,	K09225|1|0.0|679|mmu:22774|zinc finger protein	gi|70780386|ref|NP_033602.2|	0	zinc finger protein ZIC 4 [Mus musculus]

mmu-let-7a-2-3p	NM_009660	-30.3	0.154125	-28.23	165	GO:0031981,GO:0005886,GO:0044444,GO:0046914,GO:0008200,GO:0016702,GO:0016803,GO:0000038,GO:0006636,GO:0006884,GO:0008283,GO:0001503,GO:0006691,GO:0016049,GO:0006873,GO:0006952,GO:00	K00458|1|0.0|1296|mmu:11687|arachidonate 12-lipoxygenase [EC:1.13.11.31]!K00460|2|0.0|1232|rno:81639|arachidonate 15-lipoxygenase [EC:1.13.11.33]	gi|134948633|ref|NP_033790.3|		0	arachidonate 12-lipoxygenase, leukocyte-type [Mus musculus]

mmu-let-7a-2-3p	NM_009726	-27.4	0.87863	-42.89	310	GO:0031224,GO:0005903,GO:0044459,GO:0005798,GO:0044297,GO:0005768,GO:0043682,GO:0032559,GO:0016531,GO:0005515,GO:0010467,GO:0021859,GO:0002294,GO:0035434,GO:0042415,GO:0030003,GO:0043009,GO:0043085,GO:0009072,GO:0085029,GO:0009207,GO:00435	K01533|1|0.0|2759|mmu:11977|Cu2+-exporting ATPase [EC:3.6.3.4]	gi|148682115|gb|EDL14062.1|	0	ATPase, Cu++ transporting, alpha polypeptide, isoform CRA_b [Mus musculus]

mmu-let-7a-2-3p	NM_009940	-27	0.188533	-25.4	157	GO:0043231,GO:0019866,GO:0046872,GO:0003824,GO:0006979,GO:0043009,GO:0043170,GO:0010259,GO:0001838,GO:0006743,GO:0042773,GO:0007399,GO:00070	K06134|1|1e-103|376|mmu:12850|ubiquinone biosynthesis monooxygenase Coq7 [EC:1.14.13.-]	gi|20587962|ref|NP_034070.1|	6.81E-103	ubiquinone biosynthesis protein COQ7 homolog [Mus musculus]

mmu-let-7a-2-3p	NM_010301	-29.2	0.322741	-25.4	164	GO:0032991,GO:0031234,GO:0060089,GO:0005102,GO:0032561,GO:0008047,GO:0017111,GO:0032403,GO:0030326,GO:0001775,GO:0033673,GO:0007200,GO:0021879,GO:0042551,GO:0035411,GO:0042391,GO:0009207,GO:0006464,GO:0007599,GO:0007188,GO:0031647,GO:00509	K04635|1|0.0|714|mmu:14672|guanine nucleotide-binding protein subunit alpha-11	gi|6754004|ref|NP_034431.1|	0	guanine nucleotide-binding protein subunit alpha-11 [Mus musculus]
mmu-let-7a-2-3p	NM_010481	-33	0.066527	-28.49	170	GO:0005759,GO:0005515,GO:0032559,GO:0044267,GO:0006605,GO:0043066,	K04043|1|0.0|1160|mmu:15526|molecular chaperone DnaK	gi|162461907|ref|NP_034611.2|	0	stress-70 protein, mitochondrial [Mus musculus]

mmu-let-7a-2-3p	NM_010561	-37.8	0.010007	-34.75	177	GO:0032991,GO:0031981,GO:0003723,GO:0001071,GO:0005515,GO:0006355,GO:00082	K13090|1|0.0|1107|rno:84472|interleukin enhancer-binding factor 3	gi|148693240|gb|EDL25187.1|	0	interleukin enhancer binding factor 3, isoform CRA_d [Mus musculus]
mmu-let-7a-2-3p	NM_011053	-31.1	0.29387	-25.91	171	GO:0044444,GO:0031981,GO:0003676,GO:0005515,GO:0042254,GO:0006396,	K14792|1|0.0|3299|mmu:18572|rRNA biogenesis protein RRP5	gi|54607128|ref|NP_035183.2|	0	protein RRP5 homolog [Mus musculus]
mmu-let-7a-2-3p	NM_011874	-25	0.639437	-25.86	157	GO:0031981,GO:0043234,GO:0017111,GO:0032559,GO:0043161,GO:0051320,GO:0007050,GO:0016070,GO:0009207,GO:0051439,GO:0006520,GO:0006915,GO:0030330,GO:00	K03063|1|0.0|758|rno:117262|26S proteasome regulatory subunit T3	gi|25742677|ref|NP_476463.1|	0	26S protease regulatory subunit 6B [Rattus norvegicus]

mmu-let-7a-2-3p	NM_011970	-28.4	0.097824	-26.14	162	GO:0043231,GO:0000502,GO:0004175,GO:0043161,GO:0010033,GO:0051320,GO:0007050,GO:0016070,GO:0051439,GO:0006520,GO:0006915,GO:0030330,GO:0009719,GO:00	K02734|1|2e-99|362|mmu:26445|20S proteasome subunit beta 4 [EC:3.4.25.1]	gi|227116345|ref|NP_036100.3|	9.99E-99	proteasome subunit beta type-2 [Mus musculus]
mmu-let-7a-2-3p	NM_015768	-27	0.346205	-25.49	160	GO:0005102,GO:0042221,GO:0048511,GO:0051480,GO:0048232,GO:0007186,GO:0007600,GO:0006939,GO:0048646,GO:0006952,GO:0043066,GO:0000165,	-	gi|7656932|ref|NP_056583.1|	9.17E-55	prokineticin-2 isoform 1 precursor [Mus musculus]

mmu-let-7a-2-3p	NM_016964	-26	0.869676	-25.79	159	GO:0043073,GO:0008278,GO:0000795,GO:0007140,GO:0007143,GO:0007129,	K13055|1|0.0|2189|mmu:50878|cohesin complex subunit SA-3	gi|158854018|ref|NP_058660.2|	0	cohesin subunit SA-3 [Mus musculus]
mmu-let-7a-2-3p	NM_019421	-32.7	0.049444	-27.3	175	GO:0043231,GO:0016020,GO:0005102,	K06734|1|8e-113|408|mmu:54219|CD320 antigen	gi|12861991|dbj|BAB32321.1|	5.87E-112	unnamed protein product [Mus musculus]
mmu-let-7a-2-3p	NM_019482	-30.8	0.106568	-27.26	165	GO:0005911,GO:0043231,GO:0031224,GO:0031090,GO:0042995,GO:0022842,GO:0046983,GO:0005261,GO:0022829,GO:0032730,GO:0007267,GO:0010033,GO:0006816,GO:0032731,GO:0051259,GO:00507	K03443|1|0.0|773|mmu:55991|pannexin	gi|8515126|gb|AAF75838.1|AF207817_1	0	putative gap junction protein pannexin [Mus musculus]
mmu-let-7a-2-3p	NM_023320	-28.8	0.148582	-27.29	162	GO:0043231,GO:0016020,	-	gi|12963637|ref|NP_075809.1|	0	pleckstrin homology domain-containing family O member 1 [Mus

mmu-let-7a-2-3p	NM_023631	-30.1	0.346302	-26.59	162	GO:0046914,GO:0003824,GO:0051536,GO:0050662,GO:0000166,GO:0008152,	K00157|1|0.0|1774|aml:100471086|aldehyde oxidase [EC:1.2.3.1]	gi|114205422|ref|NP_076120.2|	0	aldehyde oxidase 4 [Mus musculus]
mmu-let-7a-2-3p	NM_023721	-28	0.216091	-25.14	160	GO:0005774,GO:0016469,GO:0019829,GO:0005488,GO:0015988,GO:0015031,GO:0009207,GO:0007169,GO:0006875,	K02149|1|1e-113|410|rno:299159|V-type H+-transporting ATPase subunit D [EC:3.6.3.14]	gi|40786463|ref|NP_955418.1|	8.78E-113	V-type proton ATPase subunit D [Rattus norvegicus]
mmu-let-7a-2-3p	NM_024273	-33.5	0.031946	-28.27	173	GO:0019866,GO:0043231,GO:0031224,GO:0022892,GO:0006810,	-	gi|225543444|ref|NP_077235.2|	1.12E-157	translocase of inner mitochondrial membrane domain-containing protein 1 [Mus musculus]
mmu-let-7a-2-3p	NM_025844	-25.5	0.727772	-40.49	330	GO:0031072,GO:0046914,GO:0006457,GO:0071901,GO:0046605,GO:0006950,	K12795|1|9e-06|53.1|cin:100179637|suppressor of G2 allele of SKP1	gi|13385324|ref|NP_080120.1|	2.68E-165	cysteine and histidine-rich domain-containing protein 1 [Mus musculus]

mmu-let-7a-2-3p	NM_026121	-31.1	0.23527	-25.31	174	GO:0031981,GO:0005488,GO:0004871,GO:0009451,GO:0044267,GO:0051259,GO:00430	K09558|1|6e-175|616|mmu:67384|BCL2-associated athanogene 4	gi|23273683|gb|AAH37239.1|	1.79E-175	Bag4 protein [Mus musculus]
mmu-let-7a-2-3p	NM_026887	-26	0.785653	-25.01	165	GO:0005764,GO:0030117,GO:0012506,GO:0022892,GO:0048193,GO:0050688,GO:00150	K12394|1|4e-87|323|mmu:108012|AP-1 complex subunit sigma 1/2	gi|194389590|dbj|BAG61756.1|	3.69E-89	unnamed protein product [Homo sapiens]
mmu-let-7a-2-3p	NM_027900	-26.2	0.845132	-25.78	162	GO:0043231,GO:0005488,	K02360|1|5e-28|128|aag:AaeL_AAEL009189|a component of the cytoplasm (encore)	gi|269973927|ref|NP_082176.4|	0	R3H domain-containing protein 2 isoform 2 [Mus musculus]
mmu-let-7a-2-3p	NM_027982	-25.4	0.64888	-26.33	162	GO:0004721,GO:0005488,GO:0006796,GO:0006464,	K01090|1|3e-137|489|xtr:550059|protein phosphatase [EC:3.1.3.16]	gi|114205424|ref|NP_082258.2|	0	protein phosphatase 1J [Mus musculus]

mmu-let-7a-2-3p	NM_028234	-28	0.851947	-26.33	157	-	K14007|1|2e-06|57.4|dya:Dyak_GE15184|protein transport protein SEC24	gi|145966792|ref|NP_082510.1|	0	RNA-binding protein 33 [Mus musculus]
mmu-let-7a-2-3p	NM_028846	-27.7	0.620074	-25.33	155	GO:0044444,GO:0016790,GO:0004175,GO:0046914,GO:0005102,GO:0016192,GO:0007186,GO:0019941,GO:0016579,	K11848|1|0.0|1764|mmu:74270|ubiquitin carboxyl-terminal hydrolase 20/33 [EC:3.1.2.15]	gi|50510751|dbj|BAD32361.1|	0	mKIAA1003 protein [Mus musculus]
mmu-let-7a-2-3p	NM_030150	-32.7	0.056005	-29.28	162	GO:0044424,GO:0046914,GO:0003676,GO:0017111,GO:0032559,GO:0006952,	K12649|1|0.0|1321|mmu:80861|ATP-dependent RNA helicase DHX58 [EC:3.6.3.14]	gi|70608133|ref|NP_084426.2|	0	probable ATP-dependent RNA helicase DHX58 [Mus musculus]
mmu-let-7a-2-3p	NM_030153	-29.4	0.243894	-26.64	163	GO:0016020,GO:0031981,GO:0005488,GO:0033002,	-	gi|172072615|ref|NP_084429.2|	0	N-alpha-acetyltransferase 35, NatC auxiliary subunit [Mus musculus]

mmu-let-7a-2-3p	NM_133937	-29.6	0.079023	-25.8	159	-	K05752|1|5e-29|129|ssc:100522899|chromosome 3 open reading frame 10	gi|6841250|gb|AAF28978.1|AF161418_1	8.59E-30	HSPC300 [Homo sapiens]
mmu-let-7a-2-3p	NM_133952	-28.4	0.426672	-25.1	162	GO:0044444,GO:0043231,GO:0005488,GO:0048513,GO:0048869,	K04460|1|4e-13|78.2|smm:Smp_090860|protein phosphatase 5 [EC:3.1.3.16]	gi|227908790|ref|NP_598713.2|	0	protein unc-45 homolog A [Mus musculus]
mmu-let-7a-2-3p	NM_134131	-27.7	0.539218	-25.64	160	GO:0044424,GO:0004869,GO:0005488,GO:0010466,	-	gi|295444879|ref|NP_598892.2|	5.38E-115	tumor necrosis factor alpha-induced protein 8 isoform 1 [Mus musculus]

mmu-let-7a-2-3p	NM_138305	-30	0.31758	-28.41	160	GO:0043231,GO:0016021,GO:0005515,GO:0004016,GO:0032559,GO:0043169,GO:0015980,GO:0007267,GO:0045762,GO:0033762,GO:0032147,GO:0010863,GO:0007169,GO:0007126,GO:0007188,GO:0042044,GO:00	K08043|1|0.0|1964|mmu:104111|adenylate cyclase 3 [EC:4.6.1.1]	gi|28972253|dbj|BAC65580.1|	0	mKIAA0511 protein [Mus musculus]
mmu-let-7a-2-3p	NM_138590	-28.1	0.37869	-25.06	159	GO:0046914,	K12597|1|0.0|962|mmu:319885|protein AIR1/2	gi|169646700|ref|NP_613056.2|	0	zinc finger CCHC domain-containing protein 7 [Mus musculus]
mmu-let-7a-2-3p	NM_146228	-28.5	0.574013	-29.19	157	GO:0031410,GO:0008047,GO:0005088,GO:0005515,GO:0006996,GO:0043087,GO:0033036,GO:0007266,	K04575|1|3e-139|498|mdo:100019560|amyotrophic lateral sclerosis 2 (juvenile)	gi|225690620|ref|NP_666340.3|	0	ALS2 C-terminal-like protein [Mus musculus]

mmu-let-7a-2-3p	NM_146261	-25.7	0.985738	-44.47	317	-	-	gi|148691944|gb|EDL23891.1|	0	cDNA sequence BC031748 [Mus musculus]
mmu-let-7a-2-3p	NM_153153	-27.8	0.807354	-25.77	158	GO:0044459,GO:0043231,GO:0030016,GO:0015629,GO:0042995,GO:0003779,GO:0014706,GO:0006996,	K10369|1|0.0|4275|mmu:225115|supervillin	gi|23346601|ref|NP_694793.1|	0	supervillin [Mus musculus]

mmu-let-7a-2-3p	NM_172674	-29	0.543318	-25.09	158	GO:0035097,GO:0046914,GO:0003676,GO:0010467,	K09189|1|9e-09|64.7|api:100161573|histone-lysine N-methyltransferase MLL5 [EC:2.1.1.43]!K13165|2|4e-08|62.4|aml:100466086|splicing factor, arginine/serine-rich 12!K13100|3|6e-08|62.0|dan:Dana_GF14847|pre-mRNA-splicing factor CWC22!K04574|4|7	gi|124487307|ref|NP_766262.2|	0	PHD finger protein 20 [Mus musculus]
mmu-let-7a-2-3p	NM_172887	-29.9	0.636547	-25	161	GO:0031224,	-	gi|255069717|ref|NP_766475.2|	0	furry homolog [Mus musculus]

mmu-let-7a-2-3p	NM_172891	-30	0.249189	-27.03	160	GO:0031224,GO:0004713,GO:0032559,GO:0006464,	K08253|1|0.0|788|mmu:243659|non-specific protein-tyrosine kinase [EC:2.7.10.2]	gi|27370362|ref|NP_766479.1|	0	tyrosine-protein kinase STYK1 [Mus musculus]
mmu-let-7a-2-3p	NM_173021	-25.2	0.979532	-45.6	316	GO:0043234,GO:0043227,GO:0044444,GO:0016020,GO:0005515,GO:0015926,GO:0070567,GO:0004683,GO:0006468,GO:0019318,GO:0006656,GO:0005977,	K07190|1|0.0|2233|mmu:18679|phosphorylase kinase alpha/beta subunit	gi|153945788|ref|NP_766609.2|	0	phosphorylase b kinase regulatory subunit alpha, skeletal muscle isoform isoform 2 [Mus musculus]
mmu-let-7a-2-3p	NM_173414	-28.8	0.433597	-26.84	160	-	-	gi|238637328|ref|NP_775590.2|	0	lanC-like protein 3 [Mus musculus]

mmu-let-7a-2-3p	NM_175333	-34.9	0.007522	-31.63	166	GO:0019866,GO:0031224,GO:0043231,GO:0006810,	K14684|1|9e-169|593|mmu:103775|solute carrier family 25 (mitochondrial phosphate transporter), member 23/24/25/41	gi|30425020|ref|NP_780542.1|	6.02E-168	solute carrier family 25 member 41 [Mus musculus]
mmu-let-7a-2-3p	NM_175366	-28.1	0.483924	-26.99	162	GO:0044424,GO:0046872,GO:0006468,	K15686|1|0.0|721|mmu:108797|RNA-binding protein MEX3	gi|50511245|dbj|BAD32608.1|	0	mKIAA2009 protein [Mus musculus]
mmu-let-7a-2-3p	NM_175480	-29	0.48503	-25.62	158	-	K09228|1|0.0|1405|mmu:234725|KRAB domain-containing zinc finger protein	gi|283837868|ref|NP_780689.3|	0	zinc finger protein 612 [Mus musculus]

mmu-let-7a-2-3p	NM_175499	-35.1	0.041641	-30.97	166	GO:0031224,GO:0048667,	K06838|1|9e-32|140|xla:380270|slit 1!K06850|4|8e-31|137|mdo:100030923|slit 3	gi|30425254|ref|NP_780708.1|	0	SLIT and NTRK-like protein 6 precursor [Mus musculus]
mmu-let-7a-2-3p	NM_177036	-26.6	0.712385	-26.08	155	-	K06499|1|4e-07|58.2|xtr:100493418|carcinoembryonic antigen-related cell adhesion	gi|28892919|ref|NP_796010.1|	8.55E-121	carcinoembryonic antigen-related cell adhesion molecule 19 precursor [Mus musculus]
mmu-let-7a-2-3p	NM_178791	-30.5	0.164263	-27.76	159	GO:0031224,	-	gi|30520277|ref|NP_848906.1|	5.01E-148	uncharacterized protein LOC320736 precursor [Mus musculus]
mmu-let-7a-2-3p	NM_181848	-25.9	0.697972	-66.82	443	GO:0044431,GO:0031410,GO:0005515,GO:0007030,GO:0006886,GO:0000301,GO:0023052,	K07210|1|1e-42|176|xtr:100145270|inhibitor of nuclear factor kappa-B kinase subunit	gi|148676014|gb|EDL07961.1|	0	optineurin, isoform CRA_b [Mus musculus]

mmu-let-7a-2-3p	NM_198671	-29.2	0.57043	-25.89	167	GO:0005488,	-	gi|225543323|ref|NP_941073.2|	0	genetic suppressor element 1 isoform 1 [Mus musculus]
mmu-let-7a-2-3p	NM_199056	-28.8	0.32006	-26.65	163	GO:0043231,GO:0032559,GO:0006020,GO:0006796,	K10572|1|0.0|1005|mmu:75678|inositol-pentakisphosphate 2-kinase [EC:2.7.1.158]	gi|39979616|ref|NP_951011.1|	0	inositol-pentakisphosphate 2-kinase [Mus musculus]
mmu-let-7a-2-3p	NR_033514	-26.9	0.795954	-26.95	160	-	-	gi|148670710|gb|EDL02657.1|	2.17E-19	mCG63843, isoform CRA_b [Mus musculus]
mmu-let-7a-2-3p	NR_040531	-32.8	0.00815	-30.28	174	-	-	gi|148685748|gb|EDL17695.1|	3.56E-23	mCG144665 [Mus musculus]
mmu-let-7a-2-3p	NR_045139	-32.5	0.070196	-28.99	161	-	-	gi|148683880|gb|EDL15827.1|	3.54E-21	mCG147542 [Mus musculus]
mmu-let-7a-2-3p	NM_025545	-23.1	0.999869	-60.13	452	GO:0031981,GO:0000785,GO:0003723,GO:0046914,GO:0035312,GO:0005515,GO:0019204,GO:0043566,GO:0010608,GO:0006281,GO:0000302,GO:0006796,	K10863|1|0.0|689|mmu:66408|aprataxin [EC:3.-.-.-]	gi|32526865|ref|NP_079821.3|	0	aprataxin isoform a [Mus musculus]

mmu-let-7a-2-3p	NM_183310	-21.6	1	-46.62	318	-	-	gi|256222323|ref|NP_899133.2|	0	protein FAM190A [Mus musculus]
mmu-let-7a-2-3p	NM_172053	-20.5	1	-47.32	312	GO:0031012,GO:0046914,GO:0004175,GO:0019538,	K08630|1|0.0|2464|mmu:271127|a disintegrin and metalloproteinase with thrombospondin motifs 16 [EC:3.4.24.-]	gi|50511261|dbj|BAD32616.1|	0	mKIAA2029 protein [Mus musculus]
mmu-let-7a-2-3p	NM_027238	-22.3	1	-41.07	306	-	-	gi|58037187|ref|NP_081514.1|	0	tetratricopeptide repeat protein 39B [Mus musculus]
mmu-let-7a-2-3p	NM_145124	-22.4	0.99985	-40.47	304	GO:0043234,GO:0005768,GO:0008092,GO:0046914,GO:0060089,GO:0019787,GO:0032446,GO:0007249,GO:0007166,	K10645|1|0.0|1733|mmu:76580|E3 ubiquitin-protein ligase mind-bomb [EC:6.3.2.19]	gi|110347531|ref|NP_660106.2|	0	E3 ubiquitin-protein ligase MIB2 isoform 2 [Mus musculus]

mmu-let-7a-2-3p	NM_011904	-28.2	0.464157	-22.59	179	GO:0046914,GO:0004175,GO:0032501,GO:0019538,GO:0048869,	K13047|1|0.0|2071|mmu:24087|tolloid-like protein 2 [EC:3.4.24.-]	gi|6755807|ref|NP_036034.1|	0	tolloid-like protein 2 precursor [Mus musculus]
mmu-let-7a-2-3p	NM_026796	-24.1	0.830228	-24.21	174	GO:0043231,GO:0044444,GO:0046914,GO:0018024,GO:0001099,GO:0000122,GO:0008283,GO:0018022,GO:0009966,GO:00349	K11426|1|0.0|894|mmu:226830|SET and MYND domain-containing protein	gi|30840984|ref|NP_081072.1|	0	N-lysine methyltransferase SMYD2 [Mus musculus]
mmu-let-7a-5p	NM_001007589	-33	0.027873	-31.31	167	GO:0043231,GO:0043234,GO:0006357,GO:0001819,GO:0002237,GO:0007275,GO:00082	-	gi|86604728|ref|NP_001007590.2|	2.67E-69	akirin-2 [Mus musculus]
mmu-let-7a-5p	NM_001013774	-28	0.329907	-41.77	305	GO:0005634,GO:0006886,	K15043|1|2e-132|474|xla:398027|importin subunit alpha-2	gi|144446032|ref|NP_001013796.2|	0	importin subunit alpha-8 [Mus musculus]

mmu-let-7a-5p	NM_001024504	-28.2	0.406472	-25.69	156	-	K10624|1|6e-40|167|ame:724670|E3 ubiquitin-protein ligase RBBP6 [EC:6.3.2.19]	gi|111162659|ref|NP_001019675.2|	1.35E-149	DCN1-like protein 2 isoform a [Mus musculus]
mmu-let-7a-5p	NM_001033343	-25.7	0.867865	-25	160	GO:0030134,GO:0031090,GO:0006810,	K14005|1|0.0|2187|mmu:240667|protein transport protein SEC31	gi|133504851|ref|NP_001028515.1|	0	protein transport protein Sec31B [Mus musculus]
mmu-let-7a-5p	NM_001042649	-28.2	0.396955	-25.69	156	-	K10624|1|1e-31|94.4|ame:724670|E3 ubiquitin-protein ligase RBBP6 [EC:6.3.2.19]	gi|148690187|gb|EDL22134.1|	2.05E-133	mCG3700, isoform CRA_e [Mus musculus]

mmu-let-7a-5p	NM_001042650	-28.2	0.385621	-25.69	156	-	K10624|1|1e-17|93.2|ame:724670|E3 ubiquitin-protein ligase RBBP6 [EC:6.3.2.19]	gi|148690187|gb|EDL22134.1|	8.48E-138	mCG3700, isoform CRA_e [Mus musculus]
mmu-let-7a-5p	NM_001042651	-28.2	0.395333	-25.69	156	-	K10624|1|1e-24|109|ame:724670|E3 ubiquitin-protein ligase RBBP6 [EC:6.3.2.19]	gi|148690187|gb|EDL22134.1|	2.91E-132	mCG3700, isoform CRA_e [Mus musculus]
mmu-let-7a-5p	NM_001085378	-29.4	0.48487	-25.41	168	GO:0016459,GO:0008092,GO:0017111,GO:0032559,	K10352|1|0.0|3052|mmu:668940|myosin heavy chain	gi|148674189|gb|EDL06136.1|	0	mCG21082 [Mus musculus]

mmu-let-7a-5p	NM_001105245	-26.7	0.961121	-25.05	168	GO:0031224,GO:0046872,GO:0016337,	K04601|1|2e-64|250|bfo:BRAFLDRAFT_130073|cadherin EGF LAG seven-pass G-type receptor 2 (flamingo)	gi|157426849|ref|NP_001098715.1|	0	protocadherin-19 isoform a precursor [Mus musculus]
mmu-let-7a-5p	NM_001105246	-26.7	0.959564	-25.05	168	GO:0031224,GO:0046872,GO:0016337,	K04601|1|2e-64|250|bfo:BRAFLDRAFT_130073|cadherin EGF LAG seven-pass G-type receptor 2 (flamingo)	gi|157426851|ref|NP_001098716.1|	0	protocadherin-19 isoform b precursor [Mus musculus]

mmu-let-7a-5p	NM_001110140	-31.2	0.20022	-28.13	159	GO:0032991,GO:0016021,GO:0005789,GO:0001664,GO:0019829,GO:0046872,GO:0032559,GO:0009888,GO:0007599,GO:0008016,GO:0006816,GO:0006936,GO:0009207,GO:0007165,GO:0001775,GO:0009206,GO:0009725,GO:0006875,	K05853|1|0.0|2035|mmu:11938|Ca2+ transporting ATPase, sarcoplasmic/endoplasmic reticulum [EC:3.6.3.8]	gi|60360584|dbj|BAD90532.1|	0	mKIAA4195 protein [Mus musculus]
mmu-let-7a-5p	NM_001122989	-28	0.660186	-25.73	163	GO:0031224,GO:0031981,GO:0005488,GO:0004721,GO:0006470,GO:0006259,GO:0051443,GO:0000077,	K06639|1|0.0|934|mmu:218294|cell division cycle 14 [EC:3.1.3.48]	gi|172072673|ref|NP_001116461.1|	0	dual specificity protein phosphatase CDC14B isoform 2 [Mus musculus]

mmu-let-7a-5p	NM_001160012	-30.4	0.101299	-28.44	172	GO:0005921,GO:0031224,GO:0005488,GO:0022829,GO:0043009,GO:0008544,GO:0050954,GO:0009987,	K07622|1|4e-136|485|mmu:14620|gap junction protein, beta 3	gi|6680013|ref|NP_032152.1|	3.12E-135	gap junction beta-3 protein [Mus musculus]
mmu-let-7a-5p	NM_001161433	-28.6	0.252431	-26.57	166	GO:0016021,GO:0005035,GO:0005488,GO:0043410,GO:0048869,GO:0009888,GO:0051091,GO:0008219,GO:0019221,GO:00072	K05163|1|5e-165|582|mmu:245527|tumor necrosis factor receptor superfamily member 27	gi|148682260|gb|EDL14207.1|	1.81E-166	ectodysplasin A2 isoform receptor, isoform CRA_b [Mus musculus]
mmu-let-7a-5p	NM_001164709	-28.7	0.346202	-25.03	162	GO:0031981,GO:0046914,GO:0016879,GO:0043161,GO:0043009,GO:0043066,	K10601|1|0.0|679|rno:361712|E3 ubiquitin-protein ligase synoviolin [EC:6.3.2.19]	gi|149062129|gb|EDM12552.1|	0	synovial apoptosis inhibitor 1, synoviolin, isoform CRA_b [Rattus norvegicus]

mmu-let-7a-5p	NM_001168321	-30.2	0.37687	-25.11	166	GO:0043231,GO:0043234,GO:0003712,GO:0043565,GO:0019787,GO:0019899,GO:0000122,GO:0030279,GO:0007389,GO:0070168,GO:0009887,GO:0032446,GO:0000414,GO:00	K10683|1|3e-16|89.7|ecb:100053962|BRCA1-associated RING domain protein 1 [EC:6.3.2.19]	gi|37360460|dbj|BAC98208.1|	0	mKIAA1575 protein [Mus musculus]
mmu-let-7a-5p	NM_001205068	-26.6	0.785852	-42.94	311	-	K11323|1|1e-17|94.0|dwi:Dwil_GK14253|histone arginine demethylase JMJD6 [EC:1.14.1	gi|326633177|ref|NP_001191997.1|	0	jmjC domain-containing protein 4 isoform 2 [Mus musculus]

mmu-let-7a-5p	NM_007719	-27.5	0.350718	-27.05	159	GO:0009986,GO:0016021,GO:0004950,GO:0006952,GO:0051480,GO:0002376,GO:0007166,GO:0042221,	K04182|1|0.0|716|mmu:12775|C-C chemokine receptor type 7	gi|116268121|ref|NP_031745.2|	0	C-C chemokine receptor type 7 precursor [Mus musculus]
mmu-let-7a-5p	NM_008126	-30.4	0.082129	-28.44	172	GO:0005921,GO:0031224,GO:0005488,GO:0022829,GO:0043009,GO:0008544,GO:0050954,GO:0009987,	K07622|1|4e-136|485|mmu:14620|gap junction protein, beta 3	gi|6680013|ref|NP_032152.1|	2.54E-135	gap junction beta-3 protein [Mus musculus]
mmu-let-7a-5p	NM_008340	-27.5	0.373228	-25.11	151	GO:0016942,GO:0019838,GO:0023052,GO:0009987,	K13023|1|2e-41|171|mcc:707574|carboxypeptidase N regulatory subunit	gi|60359992|dbj|BAD90215.1|	0	mKIAA4111 protein [Mus musculus]

mmu-let-7a-5p	NM_008355	-29.5	0.086157	-25.74	170	GO:0044424,GO:0044421,GO:0005126,GO:0015992,GO:0010524,GO:0009612,GO:0001818,GO:0030888,GO:0023052,GO:0002377,GO:0007154,GO:0008284,GO:0002696,GO:0014070,GO:0002237,GO:00069	K05435|1|3e-56|219|mmu:16163|interleukin 13	gi|6680403|ref|NP_032381.1|	1.79E-55	interleukin-13 precursor [Mus musculus]
mmu-let-7a-5p	NM_008767	-28.5	0.17784	-25.99	163	GO:0016021,GO:0044459,GO:0012505,GO:0044424,GO:0015238,GO:0008509,GO:0019899,GO:0015291,GO:0015893,GO:0003008,GO:0006812,	K08214|1|2e-168|592|mmu:18400|MFS transporter, OCT family, solute carrier family 22 (organic cation transporter), member 18	gi|112363086|ref|NP_001036225.1|	1.38E-167	solute carrier family 22 member 18 [Mus musculus]

mmu-let-7a-5p	NM_008824	-37.4	0.003593	-33.42	184	GO:0043234,GO:0044444,GO:0070061,GO:0008443,GO:0019899,GO:0019203,GO:0032559,GO:0006007,GO:0035556,GO:0033131,GO:0009887,GO:0043434,GO:0031960,GO:0015980,GO:0006000,GO:00069	K01103|1|0.0|910|mmu:18639|6-phosphofructo-2-kinase / fructose-2,6-bisphosphatase [EC:2.7.1.105 3.1.3.46]	gi|7434494|pir||S77704	0	6-phosphofructo-2-kinase (EC 2.7.1.105) / fructose-2, 6-bisphosphate 2-phosphatase (EC 3.1.3.46) clone 5c, skeletal muscle - rat
mmu-let-7a-5p	NM_009826	-29.8	0.475503	-25.85	171	GO:0043231,GO:0043234,GO:0044444,GO:0005488,GO:0044248,GO:0001932,GO:0006915,GO:0000165,GO:0008361,GO:0048513,GO:0007033,GO:0010467,	K11498|1|3e-15|86.7|hsa:1062|centromeric protein E	gi|226693374|ref|NP_033956.2|	0	RB1-inducible coiled-coil protein 1 [Mus musculus]

mmu-let-7a-5p	NM_010473	-28	0.356786	-25.14	165	GO:0005789,GO:0044057,	-	gi|133778931|ref|NP_034603.2|	0	histidine rich calcium binding protein precursor [Mus musculus]
mmu-let-7a-5p	NM_011060	-26	0.749867	-25.05	165	GO:0044424,GO:0046872,GO:0016813,GO:0006527,GO:0018195,	K01481|1|0.0|1349|mmu:18601|protein-arginine deiminase [EC:3.5.3.15]	gi|156938255|ref|NP_035190.3|	0	protein-arginine deiminase type-3 [Mus musculus]
mmu-let-7a-5p	NM_011150	-27.4	0.396182	-42.86	321	-	K13912|1|3e-29|131|ssc:751862|deleted in malignant brain tumors 1 protein	gi|6755144|ref|NP_035280.1|	0	galectin-3-binding protein precursor [Mus musculus]

mmu-let-7a-5p	NM_011532	-28.5	0.212519	-25.49	167	GO:0043231,GO:0001071,GO:0042802,GO:0003677,GO:0003007,GO:0051705,GO:0048771,GO:0048729,GO:0006357,GO:0001667,GO:0048534,GO:0045165,GO:0048732,GO:0009855,GO:0003002,GO:0050954,GO:0042471,GO:0035239,GO:0048514,GO:0035270,GO:0048646,GO:00487	K10175|1|1e-168|593|mmu:21380|T-box protein 1	gi|148665113|gb|EDK97529.1|	7.57E-168	T-box 1 [Mus musculus]
mmu-let-7a-5p	NM_013581	-30.8	0.152174	-27.35	175	GO:0012505,GO:0043234,GO:0043231,GO:0005488,GO:0048193,GO:0006996,	-	gi|159110754|ref|NP_038609.3|	0	conserved oligomeric Golgi complex subunit 1 [Mus musculus]

mmu-let-7a-5p	NM_016695	-28.4	0.486544	-26.59	176	GO:0016021,GO:0019904,GO:0023052,	K06103|1|1e-115|419|ptr:100608149|calcium/calmodulin-dependent serine protein kinase [EC:2.7.11.1]	gi|7710062|ref|NP_057904.1|	0	MAGUK p55 subfamily member 2 [Mus musculus]
mmu-let-7a-5p	NM_019674	-28.9	0.127196	-44.96	315	GO:0005815,GO:0043231,GO:0008287,GO:0043169,GO:0004721,GO:0004702,GO:0048646,GO:0006796,GO:0007010,GO:00064	K15423|1|0.0|636|mdo:100012739|serine/threonine-protein phosphatase 4 catalytic subunit [EC:3.1.3.16]	gi|4506027|ref|NP_002711.1|	0	serine/threonine-protein phosphatase 4 catalytic subunit [Homo sapiens]
mmu-let-7a-5p	NM_021362	-25.5	0.996073	-63.74	479	GO:0044424,GO:0044421,GO:0046914,GO:0004175,GO:0048869,GO:0022414,	K07762|1|0.0|3240|mmu:18491|pappalysin-1 [EC:3.4.24.79]	gi|20148857|gb|AAM12687.1|AF439513_1	0	pregnancy-associated plasma protein-A, partial [Mus musculus]

mmu-let-7a-5p	NM_021390	-27.6	0.700061	-44.86	322	-	K09228|1|2e-24|116|ssc:100156843|KRAB domain-containing zinc finger protein	gi|159032064|ref|NP_067365.2|	0	sal-like protein 1 [Mus musculus]
mmu-let-7a-5p	NM_021463	-28.4	0.25124	-25.25	167	GO:0032991,GO:0044444,GO:0016778,GO:0046872,GO:0042802,GO:0032561,GO:0030246,GO:0032559,GO:0006220,GO:0006796,GO:0009887,GO:0046390,GO:0009168,GO:00	K00948|1|4e-179|629|ssc:100517744|ribose-phosphate pyrophosphokinase [EC:2.7.6.1]	gi|118137847|pdb|2H06|A	2.45E-178	Chain A, Crystal Structure Of Human Phosphoribosyl Pyrophosphate Synthetase 1
mmu-let-7a-5p	NM_023320	-28.9	0.131748	-25.83	159	GO:0043231,GO:0016020,	-	gi|12963637|ref|NP_075809.1|	0	pleckstrin homology domain-containing family O member 1 [Mus

mmu-let-7a-5p	NM_025304	-30	0.080105	-28.29	168	GO:0051998,GO:0005488,GO:0006479,	K15451|1|3e-161|568|ecb:100061739|tRNA wybutosine-synthesizing protein 4 [EC:2.1.1.-	gi|42794773|ref|NP_079580.2|	0	leucine carboxyl methyltransferase 1 [Mus musculus]
mmu-let-7a-5p	NM_026936	-25.8	0.710564	-46.44	312	GO:0032991,GO:0043231,GO:0019866,GO:0031301,GO:0010257,GO:0043462,GO:0045333,GO:0043086,GO:0034613,GO:00070	K03217|1|0.0|764|mmu:69089|preprotein translocase subunit YidC	gi|58037139|ref|NP_081212.1|	0	mitochondrial inner membrane protein OXA1L [Mus musculus]
mmu-let-7a-5p	NM_028544	-27.4	0.538138	-26.41	163	GO:0005794,	K05702|1|6e-07|57.8|nve:NEMVE_v1g174672|afadin	gi|61676215|ref|NP_082820.1|	0	ras-interacting protein 1 [Mus musculus]

mmu-let-7a-5p	NM_028769	-28.7	0.382515	-25.03	162	GO:0031981,GO:0046914,GO:0016879,GO:0043161,GO:0043009,GO:0043066,	K10601|1|0.0|679|rno:361712|E3 ubiquitin-protein ligase synoviolin [EC:6.3.2.19]	gi|149062129|gb|EDM12552.1|	0	synovial apoptosis inhibitor 1, synoviolin, isoform CRA_b [Rattus norvegicus]
mmu-let-7a-5p	NM_029965	-28.4	0.478232	-25.44	168	GO:0031224,GO:0046914,	K15707|1|1e-137|491|mmu:77733|RING finger protein 170	gi|148692281|gb|EDL24228.1|	1.05E-136	mCG1050561 [Mus musculus]
mmu-let-7a-5p	NM_054044	-28.1	0.639763	-25.74	166	GO:0031224,GO:0004888,GO:0001525,GO:0016477,GO:0032101,GO:0022603,GO:0007399,GO:0007186,	K08461|1|0.0|2430|mmu:78560|G protein-coupled receptor 124	gi|300934759|ref|NP_473385.2|	0	G-protein coupled receptor 124 precursor [Mus musculus]

mmu-let-7a-5p	NM_080437	-25.5	0.996836	-43.46	314	GO:0031224,GO:0046872,GO:0004888,GO:0007409,GO:0016337,GO:0007186,	K04602|1|0.0|5914|mmu:107934|cadherin EGF LAG seven-pass G-type receptor 3 (flamingo)	gi|125719165|ref|NP_536685.2|	0	cadherin EGF LAG seven-pass G-type receptor 3 precursor [Mus musculus]
mmu-let-7a-5p	NM_138952	-28.8	0.205058	-25.22	159	-	K08846|1|0.0|1095|mmu:192656|receptor-interacting serine/threonine-protein kinase 2 [EC:2.7.11.1]	gi|20336736|ref|NP_620402.1|	0	receptor-interacting serine/threonine-protein kinase 2 [Mus musculus]
mmu-let-7a-5p	NM_146155	-31.3	0.276922	-73.34	485	-	K02350|1|2e-08|63.5|tgu:100217592|DNA polymerase zeta subunit [EC:2.7.7.7]	gi|148698133|gb|EDL30080.1|	0	AT hook, DNA binding motif, containing 1, isoform CRA_b [Mus musculus]

mmu-let-7a-5p	NM_172587	-28	0.677045	-25.73	163	GO:0031224,GO:0031981,GO:0005488,GO:0004721,GO:0006470,GO:0006259,GO:0051443,GO:0000077,	K06639|1|0.0|905|mmu:218294|cell division cycle 14 [EC:3.1.3.48]	gi|148684287|gb|EDL16234.1|	0	CDC14 cell division cycle 14 homolog B (S. cerevisiae), isoform CRA_d [Mus musculus]
mmu-let-7a-5p	NM_172710	-29.4	0.380177	-25.35	170	GO:0031224,	K07126|1|9e-35|150|ptr:469885|	gi|28972381|dbj|BAC65644.1|	0	mKIAA0746 protein [Mus musculus]
mmu-let-7a-5p	NM_172715	-31	0.130606	-25.4	167	GO:0031090,GO:0043231,GO:0031224,GO:0008374,GO:0006644,GO:0006641,GO:0009966,	K13506|1|0.0|857|mmu:231510|glycerol-3-phosphate O-acyltransferase 3/4 [EC:2.3.1.15]	gi|27370046|ref|NP_766303.1|	0	glycerol-3-phosphate acyltransferase 3 [Mus musculus]
mmu-let-7a-5p	NM_172716	-31.1	0.168051	-48.68	323	GO:0046914,GO:0010467,	K11488|1|2e-144|514|ptr:461045|polycomb group RING finger protein 3	gi|148688164|gb|EDL20111.1|	3.18E-148	polycomb group ring finger 3, isoform CRA_c [Mus musculus]

mmu-let-7a-5p	NM_177656	-27.1	0.348948	-25.65	151	-	K07847|1|1e-23|112|mgp:100542192|Rad and Gem related GTP binding protein 1!K04514|2|3e-06|54.3|tca:663533|Rho-associated, coiled-coil containing protein kinase [EC:2.7.11.1]!K11547|3|6e-06|53.1|tgu:100226934|kinetochore protein	gi|29244072|ref|NP_808324.1|	0	uncharacterized protein LOC228778 [Mus musculus]

mmu-let-7a-5p	NM_178659	-26.6	0.788509	-42.94	311	-	K11323|1|1e-20|103|dwi:Dwil_GK14253|histone arginine demethylase JMJD6 [EC:1.14.1	gi|30520031|ref|NP_848774.1|	0	jmjC domain-containing protein 4 isoform 1 [Mus musculus]
mmu-let-7a-5p	NM_178774	-27.3	0.566998	-41.72	310	-	-	gi|255308901|ref|NP_848889.3|	2.86E-129	proline-rich protein 18 [Mus musculus]
mmu-let-7a-5p	NM_178923	-28.5	0.476346	-26.8	168	GO:0031981,GO:0005488,	K13167|1|2e-145|518|mmu:224432|splicing factor, arginine/serine-rich 15	gi|148665971|gb|EDK98387.1|	1.51E-146	mCG129573, isoform CRA_b [Mus musculus]
mmu-let-7a-5p	NM_183106	-26.8	0.771858	-44.54	324	-	-	gi|124430551|ref|NP_898929.2|	0	tetratricopeptide repeat domain 17 [Mus musculus]
mmu-let-7a-5p	NR_027652	-29.5	0.153113	-25.33	164	-	-	gi|148686746|gb|EDL18693.1|	1.84E-74	GTL2, imprinted maternally expressed untranslated mRNA [Mus musculus]

mmu-let-7a-5p	NR_027838	-31.2	0.252514	-28.13	159	GO:0032991,GO:0016021,GO:0005789,GO:0001664,GO:0019829,GO:0046872,GO:0032559,GO:0009888,GO:0007599,GO:0008016,GO:0006816,GO:0006936,GO:0009207,GO:0007165,GO:0001775,GO:0009206,GO:0009725,GO:0006875,	K05853|1|0.0|2035|mmu:11938|Ca2+ transporting ATPase, sarcoplasmic/endoplasmic reticulum [EC:3.6.3.8]	gi|60360584|dbj|BAD90532.1|	0	mKIAA4195 protein [Mus musculus]
mmu-let-7a-5p	NR_028280	-27.3	0.585089	-41.72	310	-	-	gi|255308901|ref|NP_848889.3|	3.00E-129	proline-rich protein 18 [Mus musculus]
mmu-let-7a-5p	NR_037992	-26.6	0.7821	-42.94	311	-	K11323|1|2e-09|66.6|nvi:100116682|histone arginine demethylase JMJD6 [EC:1.14.1	gi|30520031|ref|NP_848774.1|	0	jmjC domain-containing protein 4 isoform 1 [Mus musculus]

mmu-let-7a-5p	NR_046069	-28.5	0.068602	-25.22	165	-	-	gi|148709166|gb|EDL41112.1|	2.52E-54	mCG1051124 [Mus musculus]
mmu-let-7a-5p	NR_046475	-29.5	0.160951	-25.33	164	-	-	-	-	-
mmu-let-7a-5p	NM_181729	-24.2	0.999495	-64.98	477	-	K13908|1|0.0|868|ecb:100147340|mucin-5B	gi|32171192|ref|NP_859418.1|	0	mucin-6 precursor [Mus musculus]
mmu-let-7a-5p	NM_133199	-21.3	1	-65.42	455	GO:0005887,GO:0005272,GO:0034220,GO:0003012,GO:0006814,	K04837|1|0.0|3362|mmu:110880|voltage-gated sodium channel type IV alpha	gi|134948032|ref|NP_573462.2|	0	sodium channel protein type 4 subunit alpha [Mus musculus]
mmu-let-7a-5p	NM_008480	-22.1	1	-67.32	445	-	K05637|1|0.0|6084|mmu:16772|laminin, alpha 1/2	gi|117168301|ref|NP_032506.2|	0	laminin subunit alpha-1 precursor [Mus musculus]

mmu-let-7a-5p	NM_148948	-20.6	1	-54.61	345	GO:0031332,GO:0044444,GO:0016891,GO:0032559,GO:0043169,GO:0003723,GO:0042623,GO:0016246,GO:0006357,GO:0031641,GO:0022011,GO:0010624,GO:0048858,GO:00	K11592|1|0.0|3661|mmu:192119|endoribonuclease Dicer [EC:3.1.26.-]	gi|148686840|gb|EDL18787.1|	0	Dicer1, Dcr-1 homolog (Drosophila), isoform CRA_a [Mus musculus]
mmu-let-7a-5p	NM_007548	-23.2	0.999473	-47.25	329	GO:0043231,GO:0046914,GO:0001071,GO:0003677,GO:0019899,GO:0000122,GO:0002832,GO:0003006,GO:0001890,GO:0030888,GO:0045577,GO:0001701,	K09228|1|1e-40|170|bfo:BRAFLDRAFT_71028|KRAB domain-containing zinc finger protein	gi|125859109|gb|AAI29802.1|	0	Prdm1 protein [Mus musculus]

mmu-let-7a-5p	NM_026298	-22	0.999998	-53.72	323	GO:0005815,GO:0043231,GO:0005515,GO:0003002,GO:0006357,GO:0010927,GO:0001838,GO:0051604,GO:0009855,GO:00071	-	gi|148705414|gb|EDL37361.1|	0	intraflagellar transport 172 homolog (Chlamydomonas), isoform CRA_a [Mus musculus]
mmu-let-7a-5p	NM_153459	-23.7	0.982072	-47.16	322	GO:0031981,GO:0044444,GO:0005488,GO:0008138,GO:0006470,GO:0000165,GO:0002224,GO:0007169,GO:0043405,	K04459|1|0.0|728|mmu:235584|dual specificity phosphatase [EC:3.1.3.16 3.1.3.48]	gi|238231443|ref|NP_703189.3|	0	dual specificity protein phosphatase 7 [Mus musculus]
mmu-let-7a-5p	NR_015467	-20.9	1	-43.11	321	-	-	gi|148682130|gb|EDL14077.1|	9.17E-47	mCG1031049, isoform CRA_d [Mus musculus]

mmu-let-7a-5p	NM_001164679	-23.5	0.993695	-42.51	320	GO:0016021,GO:0005216,	K13982|1|9e-81|303|dgr:Dgri_GH10918|probable ATP-dependent RNA helicase DDX4 [EC:3.6.4.13]!K03018|3|2e-19|99.4|smm:Smp_002890|DNA-directed RNA polymerase III subunit RPC1 [EC:2.7.7.6]	gi|50511029|dbj|BAD32500.1|	0	mKIAA1623 protein [Mus musculus]

mmu-let-7a-5p	NM_013462	-24.5	0.91421	-43.13	317	GO:0043231,GO:0016021,GO:0043234,GO:0043176,GO:0031690,GO:0042802,GO:0004939,GO:0015980,GO:0001659,GO:0001993,GO:0045762,GO:0002029,GO:0045444,GO:0010740,GO:0006897,GO:0002021,GO:0007188,GO:0006950,GO:00076	K04143|1|0.0|654|mmu:11556|adrenergic receptor beta-3	gi|85861166|ref|NP_038490.2|	0	beta-3 adrenergic receptor [Mus musculus]
mmu-let-7a-5p	NR_045891	-24.5	0.336634	-43	316	-	-	gi|148700318|gb|EDL32265.1|	1.10E-47	mCG144848 [Mus musculus]

mmu-let-7a-5p	NM_201395	-23.6	0.991882	-44.33	313	GO:0000785,GO:0043234,GO:0046914,GO:0003676,GO:0014020,GO:0006357,GO:0035108,GO:0001832,GO:0003231,	K09228|1|2e-13|79.3|bta:507236|KRAB domain-containing zinc finger protein	gi|117553623|ref|NP_958797.2|	0	sal-like protein 4 isoform b [Mus musculus]
mmu-let-7a-5p	NR_001461	-22.9	1	-45.29	312	-	K11849|1|6e-55|213|acs:100555596|ubiquitin carboxyl-terminal hydrolase 25/28 [EC:3.1.2.15]!K06478|4|4e-43|182|acs:100563672|protein tyrosine phosphatase, receptor type, C [EC:3.1.3.4	gi|209553980|gb|AAA39398.2|	0	ORF2 [Mus musculus domesticus]

mmu-let-7a-5p	NM_010264	-24.2	0.996977	-43.27	309	GO:0043234,GO:0031981,GO:0046914,GO:0042802,GO:0001071,GO:0003677,GO:0004879,GO:0048869,GO:0009755,GO:00482	K08561|1|0.0|892|ssc:100038028|germ cell nuclear factor	gi|6753956|ref|NP_034394.1|	0	nuclear receptor subfamily 6 group A member 1 isoform 1 [Mus musculus]
mmu-let-7a-5p	NM_172416	-21.9	0.999767	-40.3	308	GO:0016020,GO:0044444,GO:0005488,GO:0002573,	-	gi|27369545|ref|NP_766004.1|	9.49E-140	osteopetrosis-associated transmembrane protein 1 precursor [Mus musculus]
mmu-let-7a-5p	NM_030263	-24.6	0.999152	-40.69	307	GO:0044459,GO:0044424,GO:0005083,GO:0019222,GO:0032011,	K12494|1|0.0|937|rno:306380|PH and SEC7 domain-containing protein	gi|83921581|ref|NP_084539.2|	0	PH and SEC7 domain-containing protein 3 isoform 1 [Mus musculus]

mmu-let-7a-5p	NM_018877	-22.6	0.999869	-45.25	306	GO:0031981,GO:0043232,GO:0016020,GO:0003676,GO:0046914,GO:0016279,GO:0001832,GO:0006479,GO:0006351,GO:00063	K11421|1|0.0|2232|mmu:84505|histone-lysine N-methyltransferase SETDB [EC:2.1.1.43]	gi|148706861|gb|EDL38808.1|	0	mCG16729, isoform CRA_b [Mus musculus]
mmu-let-7a-5p	NM_001024139	-20.8	1	-41.28	305	GO:0031012,GO:0046914,GO:0004175,GO:0019538,	K08629|1|0.0|1966|mmu:235130|a disintegrin and metalloproteinase with thrombospondin motifs 15 [EC:3.4.24.-]	gi|71725383|ref|NP_001019310.1|	0	A disintegrin and metalloproteinase with thrombospondin motifs 15 precursor [Mus musculus]
mmu-let-7a-5p	NM_001171512	-24.6	0.999999	-42.2	303	-	K12567|1|0.0|1030|mmu:22138|titin [EC:2.7.11.1]	gi|345842335|ref|NP_001164983.2|	0	obscurin isoform 1 [Mus musculus]
mmu-let-7a-5p	NR_038041	-23.6	0.358428	-30.84	180	-	-	gi|148700034|gb|EDL31981.1|	1.62E-60	mCG146286 [Mus musculus]

mmu-let-7a-5p	NM_173753	-23.3	0.999806	-25.06	176	GO:0044424,GO:0005488,GO:0006468,	K08020|1|5e-34|148|ptr:743392|Rap guanine nucleotide exchange factor (GEF) 6	gi|55925634|ref|NP_776114.2|	0	folliculin-interacting protein 1 [Mus musculus]
mmu-let-7a-5p	NM_177638	-20.9	0.995731	-28.37	175	GO:0043296,GO:0031224,GO:0019904,GO:0007009,GO:0045216,GO:0034329,	K06090|1|4e-50|199|mmu:224912|crumbs 3	gi|29244038|ref|NP_808306.1|	3.06E-49	crumbs protein homolog 3 precursor [Mus musculus]
mmu-let-7a-5p	NM_178687	-25	0.825096	-21.54	174	GO:0016021,GO:0004871,GO:0005102,GO:0019900,GO:0002420,GO:0023052,GO:0002696,GO:0001912,GO:0002714,GO:00325	K06567|1|3e-159|563|rno:307199|CD226 antigen	gi|85540456|ref|NP_848802.2|	3.75E-179	CD226 antigen isoform a precursor [Mus musculus]

mmu-let-7a-5p	NM_177639	-21.2	1	-22.6	172	GO:0044459,GO:0044430,GO:0005515,GO:0007267,	K15008|1|0.0|1680|rno:65040|discs, large-associated protein 1	gi|51339023|ref|NP_808307.2|	0	disks large-associated protein 1 isoform 1 [Mus musculus]
mmu-let-7a-5p	NM_145857	-24.9	0.964398	-24.56	171	GO:0043226,GO:0044444,GO:0016020,GO:0019900,GO:0042834,GO:0032559,GO:0019904,GO:0004520,GO:0006461,GO:0006915,GO:0051091,GO:0045807,GO:0016567,GO:0043331,GO:0043433,GO:0002437,GO:0002455,GO:0002385,GO:0032495,GO:0001934,GO:0002371,GO:00328	K10165|1|0.0|1827|mmu:257632|nucleotide-binding oligomerization domain-containing protein 2	gi|29427819|sp|Q8K3Z0.1|NOD2_MOUSE	0	RecName: Full=Nucleotide-binding oligomerization domain-containing protein 2; AltName: Full=Caspase recruitment domain-containing protein 15

mmu-let-7a-5p	NM_201368	-22.4	0.999879	-24.41	170	GO:0016020,	-	gi|41235770|ref|NP_958756.1|	0	XK-related protein 8 [Mus musculus]
mmu-let-7a-5p	NR_044986	-24.2	0.800998	-21.2	169	-	-	gi|148704939|gb|EDL36886.1|	8.89E-35	mCG54161, isoform CRA_a [Mus musculus]
mmu-let-7a-5p	NR_045156	-22.6	0.999105	-20.6	168	-	-	gi|74196599|dbj|BAE34410.1|	3.04E-20	unnamed protein product [Mus musculus]
mmu-let-7a-5p	NM_201519	-21.2	1	-20.33	167	GO:0044424,GO:0030695,GO:0004672,GO:0032559,GO:0000187,	K08833|1|0.0|1620|mmu:399510|mitogen-activated protein kinase kinase kinase kinase 5 [EC:2.7.11.1]	gi|148704648|gb|EDL36595.1|	0	mitogen-activated protein kinase kinase kinase kinase 5, isoform CRA_c [Mus musculus]
mmu-let-7a-5p	NM_207671	-21.9	1	-22.04	166	GO:0031981,GO:0046914,GO:0022403,GO:0010467,	-	gi|157823950|ref|NP_997554.2|	0	zinc finger protein 318 isoform 1 [Mus musculus]
mmu-let-7a-5p	NM_177091	-24.2	0.992754	-20.83	165	-	K06252|1|2e-13|80.1|spu:576745|tenascin!K05717|5|2e-08|63.2|acs:100563512|fibronectin 1	gi|260166640|ref|NP_796065.2|	0	fibronectin type III domain-containing protein 7 isoform 1 precursor [Mus musculus]

mmu-let-7a-5p	NR_033388	-25.6	0.939729	-20.18	164	-	K04332|1|7e-07|58.2|mmu:19214|prostaglandin D2 receptor	gi|309270125|ref|XP_003085044.1|	1.96E-94	PREDICTED: uncharacterized protein LOC100169868 isoform 1 [Mus musculus]
mmu-let-7a-5p	NR_046181	-20.9	0.999988	-22.85	163	-	-	gi|148703515|gb|EDL35462.1|	1.53E-34	mCG148208 [Mus musculus]
mmu-let-7a-5p	NR_027809	-22.6	0.999927	-21.09	162	GO:0044444,GO:0016646,GO:0000166,GO:0050662,GO:0000097,GO:0001101,GO:0033273,GO:0034097,GO:0006760,	K00297|1|0.0|1347|mmu:17769|methylenetetrahydrofolate reductase (NADPH) [EC:1.5.1.20]	gi|187466137|emb|CAQ51916.1|	0	5,10-methylenetetrahydrofolate reductase [Mus musculus]
mmu-let-7a-5p	NM_178732	-22.3	0.99993	-25.55	161	GO:0005622,GO:0046872,	K09228|1|0.0|1177|mmu:243834|KRAB domain-containing zinc finger protein	gi|148706142|gb|EDL38089.1|	0	zinc finger protein 324 [Mus musculus]
mmu-let-7a-5p	NR_045984	-24.8	0.589137	-21.25	160	-	-	gi|148666212|gb|EDK98628.1|	4.37E-57	mCG1036764 [Mus musculus]

mmu-let-7a-5p	NR_045601	-22.9	0.992508	-21.29	159	GO:0031224,GO:0005789,GO:0000030,GO:0006497,GO:0006487,	K03846|1|0.0|800|mmu:102580|alpha-1,2-mannosyltransferase [EC:2.4.1.259	gi|148693819|gb|EDL25766.1|	0	asparagine-linked glycosylation 9 homolog (yeast, alpha 1,2 mannosyltransferase) [Mus musculus]
mmu-let-7a-5p	NR_046162	-21.6	0.999656	-20.92	158	-	-	gi|148665186|gb|EDK97602.1|	7.08E-114	mCG144829 [Mus musculus]
mmu-let-7a-5p	NR_046022	-24.5	0.652695	-21.69	157	-	-	gi|148709053|gb|EDL40999.1|	1.90E-71	mCG145077 [Mus musculus]
mmu-let-7a-5p	NR_033218	-21.2	1	-21.14	156	GO:0044451,GO:0003676,GO:0046914,GO:0007264,GO:0032501,GO:0006351,	K09228|1|7e-25|119|xla:397958|KRAB domain-containing zinc finger protein	gi|152060560|sp|Q3UH06.2|RREB1_MOUSE	0	RecName: Full=Ras-responsive element-binding protein 1; Short=RREB-1; AltName: Full=RAS-responsive zinc finger transcription factor RREB

mmu-let-7a-5p	NR_045676	-28	0.586756	-22.74	155	GO:0044424,GO:0016020,GO:0005083,GO:0046872,GO:0008289,GO:0005515,GO:0046578,GO:0048869,GO:0010863,GO:0007186,GO:0030097,GO:0040007,GO:0007167,GO:00	K12363|1|0.0|1009|mmu:233046|RAS guanyl-releasing protein 4	gi|74185872|dbj|BAE32802.1|	0	unnamed protein product [Mus musculus]
mmu-let-7b-3p	NM_001039059	-29.3	0.345876	-28.19	173	-	K10452|1|1e-120|435|mmu:236904|kelch-like protein	gi|23346619|ref|NP_694805.1|	6.72E-120	kelch-like 15 isoform c [Mus musculus]
mmu-let-7b-3p	NM_001039060	-29.3	0.425985	-28.19	173	-	K10452|1|1e-120|436|ecb:100052043|kelch-like protein	gi|84794653|ref|NP_001034149.1|	3.68E-126	kelch-like 15 isoform b [Mus musculus]
mmu-let-7b-3p	NM_001080755	-26.3	0.970386	-25.15	164	GO:0043231,GO:0046914,GO:0003676,GO:0006351,	-	gi|124053447|ref|NP_940808.2|	0	ZZ-type zinc finger-containing protein 3 [Mus musculus]

mmu-let-7b-3p	NM_001163539	-26	0.880829	-43.75	318	-	-	gi|254692863|ref|NP_001157011.1|	7.25E-156	uncharacterized protein CXorf21 homolog [Mus musculus]
mmu-let-7b-3p	NM_009622	-29.8	0.748002	-26.65	168	GO:0043231,GO:0031224,GO:0005515,GO:0004016,GO:0032559,GO:0043169,GO:0015980,GO:0007267,GO:0007613,GO:0045762,GO:0033762,GO:0032147,GO:0010863,GO:0007169,GO:0048667,GO:0007188,GO:00	K08041|1|0.0|1955|mmu:432530|adenylate cyclase 1 [EC:4.6.1.1]	gi|61888832|ref|NP_033752.1|	0	adenylate cyclase type 1 [Mus musculus]

mmu-let-7b-3p	NM_025812	-28.8	0.453614	-27.02	166	GO:0031981,GO:0003676,GO:0001071,GO:0006351,GO:0006325,	K09272|1|7e-11|70.9|tad:TRIADDRAFT_64155|structure-specific recognition protein 1!K10802|2|9e-11|70.5|dya:Dyak_GE17263|high mobility group	gi|148693919|gb|EDL25866.1|	0	high mobility group 20A, isoform CRA_b [Mus musculus]
mmu-let-7b-3p	NM_026641	-27.6	0.685913	-25.69	163	GO:0005815,GO:0005930,	K04508|1|6e-12|74.7|smm:Smp_098760|transducin (beta)-like 1	gi|28076907|ref|NP_080917.1|	0	intraflagellar transport protein 80 homolog [Mus musculus]
mmu-let-7b-3p	NM_028538	-29.2	0.530647	-28.2	170	-	K09228|1|0.0|1213|mmu:338354|KRAB domain-containing zinc finger protein	gi|223462786|gb|AAI41374.1|	0	1700049G17Rik protein [Mus musculus]

mmu-let-7b-3p	NM_145590	-28.1	0.439396	-27.25	154	GO:0031224,	K14382|1|2e-24|88.2|bta:399680|solute carrier family 5 (sodium/glucose cotransporter), member 2	gi|225579041|ref|NP_663565.2|	0	UPF0420 protein C16orf58 homolog [Mus musculus]
mmu-let-7b-3p	NM_153165	-29.3	0.350942	-28.19	173	-	K10452|1|1e-120|435|mmu:236904|kelch-like protein	gi|23346619|ref|NP_694805.1|	6.84E-120	kelch-like 15 isoform c [Mus musculus]
mmu-let-7b-3p	NM_172516	-27	0.897371	-25.73	163	GO:0044424,GO:0004672,GO:0032559,GO:0006464,	K08282|1|0.0|1709|mmu:213452|non-specific serine/threonine protein kinase [EC:2.7.11.1]	gi|40556610|ref|NP_766104.2|	0	dual serine/threonine and tyrosine protein kinase [Mus musculus]
mmu-let-7b-3p	NM_198416	-26.3	0.969637	-25.15	164	GO:0043231,GO:0046914,GO:0003676,GO:0006351,	-	gi|124053447|ref|NP_940808.2|	0	ZZ-type zinc finger-containing protein 3 [Mus musculus]
mmu-let-7b-3p	NR_029725	-39.9	0.000002	-32.91	187	-	-	-	-	-
mmu-let-7b-3p	NR_029726	-36.5	0.000016	-32.91	187	-	-	-	-	-

mmu-let-7b-3p	NR_029730	-34.5	0.00005	-28.28	179	-	-	-	-	-
mmu-let-7b-3p	NR_029731	-28.7	0.001692	-26.29	179	-	-	-	-	-
mmu-let-7b-3p	NR_029732	-33.3	0.000077	-26.04	179	-	-	-	-	-
mmu-let-7b-3p	NM_001033173	-22.3	1	-25.26	176	GO:0008233,	K11852|1|0.0|1986|mmu:76179|ubiquitin carboxyl-terminal hydrolase 31 [EC:3.1.2.15]	gi|226443061|ref|NP_001028345.1|	0	ubiquitin specific protease 31 [Mus musculus]

mmu-let-7b-3p	NM_019827	-26.9	0.950036	-22.38	174	GO:0030427,GO:0031981,GO:0043234,GO:0044425,GO:0030425,GO:0044444,GO:0044297,GO:0004672,GO:0051018,GO:0008134,GO:0019900,GO:0032559,GO:0008092,GO:0035254,GO:0006461,GO:0001952,GO:0032321,GO:0043405,GO:0048017,GO:0048167,GO:0007169,GO:00488	K03083|1|0.0|788|ecb:100061033|glycogen synthase kinase 3 beta [EC:2.7.11.26]	gi|387766242|pdb|4ACC|A	0	Chain A, Gsk3b In Complex With Inhibitor
mmu-let-7b-3p	NM_023502	-25.3	0.867857	-20.99	171	GO:0031981,GO:0003677,GO:0001071,GO:0006355,	K09428|1|0.0|995|mmu:69257|E74-like factor 1/2/4	gi|148703252|gb|EDL35199.1|	0	E74-like factor 2, isoform CRA_e [Mus musculus]

mmu-let-7b-3p	NM_011445	-22.9	0.999999	-21.15	170	GO:0043231,GO:0043234,GO:0001071,GO:0046983,GO:0043565,GO:0009888,GO:0032989,GO:0006357,GO:0007507,GO:0048534,GO:0048468,GO:0043009,GO:00217	K09269|1|0.0|1365|bta:534321|transcription factor SOX5/6/13 (SOX group D)	gi|6755610|ref|NP_035575.1|	0	transcription factor SOX-6 isoform 1 [Mus musculus]
mmu-let-7b-3p	NM_173785	-25.9	0.902205	-22.78	169	GO:0043231,GO:0008168,GO:0006779,GO:0006730,GO:0006544,	K06980|1|0.0|705|mmu:216792|	gi|148675734|gb|EDL07681.1|	0	RIKEN cDNA A230051G13 [Mus musculus]
mmu-let-7b-3p	NR_030693	-28.1	0.597737	-24.62	168	-	-	gi|148673719|gb|EDL05666.1|	1.05E-56	mCG145028 [Mus musculus]

mmu-let-7b-3p	NM_175473	-25.1	0.999957	-20.25	167	GO:0031224,GO:0005578,GO:0043169,GO:0008544,GO:0006810,GO:0009987,	K08654|1|6e-103|379|gga:395456|proprotein convertase subtilisin/kexin type 5 [EC:3.4.21.-]	gi|126157515|ref|NP_780682.3|	0	extracellular matrix protein FRAS1 precursor [Mus musculus]
mmu-let-7b-3p	NM_026319	-22.6	0.994939	-21.28	166	GO:0031410,GO:0030990,	K12478|1|9e-14|79.7|mcc:710381|early endosome antigen 1!K11498|2|1e-13|79.3|ecb:100068227|centromeric protein E	gi|151101194|ref|NP_080595.2|	0	intraflagellar transport protein 74 homolog [Mus musculus]
mmu-let-7b-3p	NM_145976	-26.6	0.772378	-21.11	165	-	-	gi|60359824|dbj|BAD90131.1|	4.40E-80	mFLJ00292 protein [Mus musculus]
mmu-let-7b-3p	NM_178906	-27	0.8682	-20.33	163	-	-	gi|26343487|dbj|BAC35400.1|	4.87E-63	unnamed protein product [Mus musculus]
mmu-let-7b-3p	NR_015580	-26	0.808482	-21.57	162	-	-	gi|148703101|gb|EDL35048.1|	3.77E-56	mCG142516, isoform CRA_b [Mus musculus]

mmu-let-7b-3p	NR_015609	-22.8	0.989993	-21.81	161	GO:0000153,GO:0031981,GO:0005515,GO:0019787,GO:0006357,GO:0032446,GO:0065003,GO:0045935,GO:0006354,	K03873|1|4e-30|134|rno:81807|transcription elongation factor B, polypeptide 2	gi|392349787|ref|XP_003750469.1|	4.58E-31	PREDICTED: transcription elongation factor B polypeptide 2-like [Rattus norvegicus]
mmu-let-7b-3p	NR_027323	-24.5	0.977703	-23.54	160	GO:0031224,	K13023|1|2e-31|139|pon:100174713|carboxypeptidase N regulatory subunit	gi|149036465|gb|EDL91083.1|	0	rCG56516 [Rattus norvegicus]
mmu-let-7b-3p	NM_207649	-20.8	1	-20.73	159	GO:0019903,GO:0019932,GO:0007399,	-	gi|46560587|ref|NP_997532.1|	2.86E-132	calcipressin-2 isoform 1 [Mus musculus]

mmu-let-7b-3p	NM_173396	-22.6	0.998851	-21.17	158	GO:0043231,GO:0001071,GO:0003677,GO:0048856,GO:0045597,GO:0006351,	K11649|1|6e-20|100|tgu:100229325|SWI/SNF related-matrix-associated actin-dependent regulator of chromatin subfamily C!K15613|2|3e-13|78.2|spu:757216|homeobox protein Meis 1	gi|27734180|ref|NP_775572.1|	8.76E-132	homeobox protein TGIF2 [Mus musculus]
mmu-let-7b-3p	NM_033144	-21.5	1	-21.04	157	GO:0032156,GO:0032561,GO:0009987,	K04557|1|7e-65|250|xla:779425|peanut-like protein 1 (cell division control related protein 1)	gi|148701638|gb|EDL33585.1|	0	septin 8, isoform CRA_c [Mus musculus]
mmu-let-7b-3p	NR_038092	-22.8	0.993906	-20.08	156	GO:0003676,	-	gi|74138274|dbj|BAE28773.1|	1.82E-92	unnamed protein product [Mus musculus]

mmu-let-7b-3p	NR_003625	-20.2	1	-20.96	155	GO:0015934,GO:0003723,GO:0042802,GO:0005198,GO:0006412,GO:0019080,GO:0031016,GO:0042254,	K02937|1|1e-70|269|cfa:477912|large subunit ribosomal protein L7e	gi|296226698|ref|XP_002759041.1|	7.42E-70	PREDICTED: 60S ribosomal protein L7-like [Callithrix jacchus]

mmu-let-7b-3p	NM_009523	-23.8	0.995281	-20.84	154	GO:0043231,GO:0016020,GO:0031012,GO:0060089,GO:0005198,GO:0003712,GO:0001664,GO:0061370,GO:0030177,GO:0006915,GO:0010557,GO:0042692,GO:0030282,GO:0006497,GO:0016337,GO:0035567,GO:0072075,GO:0033077,GO:0003382,GO:0032342,GO:0045597,GO:00352	K00408|1|0.0|699|mmu:22417|wingless-type MMTV integration site family, member 4	gi|6678595|ref|NP_033549.1|	0	protein Wnt-4 precursor [Mus musculus]

mmu-let-7b-3p	NM_026082	-22.5	1	-21.4	153	GO:0030427,GO:0043005,GO:0032561,GO:0030695,GO:0017048,GO:0001934,GO:0007010,GO:0032855,GO:0048667,GO:0030030,GO:0030010,	K05727|1|7e-13|78.6|nvi:100118634|dedicator of cytokinesis 3/4/5	gi|78191789|ref|NP_080358.3|	0	dedicator of cytokinesis protein 7 [Mus musculus]
mmu-let-7b-3p	NM_181649	-21.6	1	-20.72	152	GO:0044464,GO:0005488,	-	gi|194473718|ref|NP_857632.4|	3.31E-133	coiled-coil domain-containing protein 75 [Mus musculus]
mmu-let-7b-3p	NM_027878	-23.2	0.994727	-22.42	149	GO:0005764,GO:0031224,GO:0012501,GO:0044248,	-	gi|27229121|ref|NP_082154.2|	1.90E-134	DNA damage-regulated autophagy modulator protein 1 [Mus musculus]
mmu-let-7b-3p	NM_001003910	NA	NA	-24.65	162	GO:0044424,	-	gi|145966853|ref|NP_001003910.2|	0	tetratricopeptide repeat protein GNN isoform 3 [Mus musculus]

mmu-let-7b-3p	NM_145501	-22	0.999988	NA	NA	GO:0032991,GO:0016021,GO:0030133,GO:0008021,GO:0005769,GO:0043025,GO:0001727,GO:0046872,GO:0005515,GO:0032559,GO:0002276,GO:0046488,GO:0046474,GO:00	K13711|1|0.0|865|mmu:84095|phosphatidylinositol 4-kinase type 2 [EC:2.7.1.67]	gi|21703986|ref|NP_663476.1|	0	phosphatidylinositol 4-kinase type 2-alpha [Mus musculus]

mmu-let-7b-3p	NM_001081417	-20.2	1	NA	NA	GO:0043231,GO:0044427,GO:0017111,GO:0003676,GO:0032559,GO:0006351,GO:0090276,GO:0007507,GO:0001944,GO:0030326,GO:0043009,GO:0050954,GO:0001654,GO:0021675,GO:0042471,GO:0046545,GO:0030098,GO:0035264,GO:0003013,GO:0006325,GO	K14437|1|0.0|3897|mmu:320790|chromodomain-helicase-DNA-binding protein 7 [EC:3.6.4.12]	gi|124487249|ref|NP_001074886.1|	0	chromodomain-helicase-DNA-binding protein 7 [Mus musculus]
mmu-let-7b-3p	NM_001081093	-20.8	1	NA	NA	GO:0012505,GO:0044444,GO:0046914,GO:0005515,GO:0051046,GO:0015031,	-	gi|124487362|ref|NP_001074562.1|	0	arfaptin-1 [Mus musculus]

mmu-let-7b-3p	NM_001080130	-22	0.99997	NA	NA	GO:0031224,GO:0044427,GO:0019866,GO:0005635,GO:0003676,GO:0005515,GO:0006998,GO:0006351,	-	gi|121949767|ref|NP_001073599.1|	0	lamina-associated polypeptide 2 isoform delta [Mus musculus]
mmu-let-7b-3p	NM_153805	-23.6	0.990099	NA	NA	GO:0043231,GO:0044444,GO:0004674,GO:0032559,GO:0006464,GO:0023052,	K06071|1|0.0|1569|mmu:263803|protein kinase N [EC:2.7.11.13]	gi|24418929|ref|NP_722500.1|	0	serine/threonine-protein kinase N3 [Mus musculus]
mmu-let-7b-3p	NM_001162904	-24.1	0.981132	NA	NA	GO:0048856,	K03995|1|5e-40|167|bfo:BRAFLDRAFT_76018|complement component 6	gi|251823814|ref|NP_001156376.1|	0	nuclear protein MDM1 isoform 3 [Mus musculus]

mmu-let-7b-3p	NM_001039388	-22.8	0.99993	NA	NA	-	K14863|1|3e-18|95.9|mmu:57750|ribosome biogenesis protein YTM1!K01062|2|4e-18|95.5|cin:100180225|1-alkyl-2-acetylglycerophosphocholine esterase [EC:3.1.1.47]	gi|37360172|dbj|BAC98064.1|	0	mKIAA0982 protein [Mus musculus]
mmu-let-7b-3p	NM_030110	-26.1	0.824972	NA	NA	GO:0031224,GO:0046872,	-	gi|255003807|ref|NP_084386.1|	0	EF-hand domain-containing family member A2 [Mus musculus]
mmu-let-7b-3p	NM_173392	-20.7	1	NA	NA	GO:0005769,GO:0022892,GO:0046872,GO:0035091,GO:0006623,GO:0006897,GO:0007178,	K04679|1|0.0|2753|mmu:218441|MAD, mothers against decapentaplegic interacting protein	gi|28972139|dbj|BAC65523.1|	0	mKIAA0305 protein [Mus musculus]
mmu-let-7b-3p	NM_025556	-20.4	0.996607	NA	NA	GO:0043231,GO:0005515,GO:0034969,GO:0006351,	-	gi|13384986|ref|NP_079832.1|	1.37E-99	cooperator of PRMT5 [Mus musculus]

mmu-let-7b-3p	NM_007985	-26.5	0.755252	NA	NA	GO:0043231,GO:0044427,GO:0044444,GO:0006281,GO:0006801,GO:0002376,GO:0019953,GO:0003006,	K10890|1|0.0|1048|mmu:14088|fanconi anemia group C protein	gi|74150968|dbj|BAE27618.1|	0	unnamed protein product [Mus musculus]
mmu-let-7b-3p	NM_011819	-25	0.579478	NA	NA	GO:0044421,GO:0005102,GO:0007154,GO:0007178,	K05504|1|4e-131|468|mmu:23886|growth differentiation factor 15	gi|170784848|ref|NP_035949.2|	2.43E-130	growth/differentiation factor 15 precursor [Mus musculus]
mmu-let-7b-3p	NM_011216	-24.4	0.998767	NA	NA	GO:0016021,GO:0004725,GO:0005488,GO:0004871,GO:0048731,GO:0006464,	K01104|1|0.0|2263|mmu:19277|protein-tyrosine phosphatase [EC:3.1.3.48]	gi|257096040|ref|NP_035346.3|	0	receptor-type tyrosine-protein phosphatase O isoform 1 precursor [Mus musculus]

mmu-let-7b-3p	NM_029081	-23.2	0.999541	NA	NA	-	K06130|1|2e-08|63.2|spu:584566|lysophospholipase II [EC:3.1.1.5]!K15290|2|2e-06|56.6|tgu:751605|synaptotagmin-1	gi|148678709|gb|EDL10656.1|	0	RIKEN cDNA 5730419I09, isoform CRA_b [Mus musculus]
mmu-let-7b-3p	NM_001163548	-25.1	0.962577	NA	NA	GO:0031252,GO:0016020,GO:0044424,GO:0004872,GO:0005083,GO:0035091,GO:0019222,GO:0030155,GO:0006810,GO:00320	K12495|1|8e-41|170|bfo:BRAFLDRAFT_106693|IQ motif and SEC7 domain-containing protein	gi|13938146|gb|AAH07189.1|	0	Cyth3 protein [Mus musculus]

mmu-let-7b-3p	NM_153589	-24	0.993702	NA	NA	GO:0034702,GO:0005488,GO:0005254,GO:0015698,	K13982|1|8e-32|140|dgr:Dgri_GH10918|probable ATP-dependent RNA helicase DDX4 [EC:3.6.4.13]!K05169|2|3e-25|118|ptr:743996|single Ig IL-1R-related molecule	gi|209862776|ref|NP_705817.2|	0	anoctamin-2 [Mus musculus]
mmu-let-7b-3p	NM_013924	-26.4	0.874855	NA	NA	GO:0043234,GO:0031981,GO:0003676,GO:0021515,GO:0006351,	K14785|1|4e-121|437|mmu:30946|ESF2/ABP1 family	gi|2065210|emb|CAA73251.1|	2.78E-160	Pro-Pol-dUTPase polyprotein [Mus musculus]
mmu-let-7b-3p	NM_133218	-21.1	1	NA	NA	GO:0044464,GO:0046914,	-	gi|18875370|ref|NP_573481.1|	0	zinc finger protein 704 [Mus musculus]

mmu-let-7b-3p	NM_146121	-20.2	1	NA	NA	GO:0005815,GO:0008092,GO:0005099,GO:0032313,	K11837|1|3e-12|75.9|hsa:9098|ubiquitin carboxyl-terminal hydrolase 6/32 [EC:3.1.2.15]!K10352|2|4e-09|65.5|xla:432231|myosin heavy chain	gi|60360530|dbj|BAD90509.1|	0	mKIAA4104 protein [Mus musculus]
mmu-let-7b-3p	NM_031376	-21.3	0.999987	NA	NA	GO:0044424,GO:0016020,GO:0005488,	K12230|1|0.0|1528|mmu:83490|phosphoinositide 3-kinase adapter protein 1	gi|13878195|ref|NP_113553.1|	0	phosphoinositide 3-kinase adapter protein 1 [Mus musculus]
mmu-let-7b-3p	NM_001146046	-20.3	1	NA	NA	GO:0015630,	K11293|1|6e-18|94.0|cbr:CBG03541|protein HIRA/HIR1	gi|225690570|ref|NP_001139518.1|	0	leucine-rich repeat-containing protein 49 isoform 1 [Mus musculus]

mmu-let-7b-3p	NM_001102458	-21.5	1	NA	NA	GO:0044444,GO:0008047,GO:0005488,GO:0004857,GO:0019222,GO:0006595,GO:00065	K01581|1|5e-123|444|xla:379859|ornithine decarboxylase [EC:4.1.1.17]	gi|9055298|ref|NP_061215.1|	0	antizyme inhibitor 1 [Mus musculus]
mmu-let-7b-3p	NM_001040686	-23.8	0.936279	NA	NA	GO:0043231,GO:0046914,GO:0003676,GO:0006351,	K09228|1|3e-24|114|mmu:432769|KRAB domain-containing zinc finger protein	gi|100817070|ref|NP_001035776.1|	0	zinc finger protein 692 [Mus musculus]
mmu-let-7b-3p	NM_009366	-20.1	0.999999	NA	NA	GO:0043231,GO:0005488,GO:0001071,GO:0006351,GO:0006950,GO:0043066,	-	gi|6678315|ref|NP_033392.1|	1.09E-54	TSC22 domain family protein 1 isoform 2 [Mus musculus]

mmu-let-7b-3p	NM_172161	-24	0.985033	NA	NA	GO:0031090,GO:0044444,GO:0016301,GO:0046983,GO:0042802,GO:0032559,GO:0051091,GO:0002224,GO:0043433,GO:0019221,GO:0006468,GO:0007249,GO:0006915,GO:00	K04731|1|0.0|1142|mmu:108960|interleukin-1 receptor-associated kinase 2 [EC:2.7.11.1]	gi|165905629|ref|NP_751893.3|	0	interleukin-1 receptor-associated kinase-like 2 isoform a [Mus musculus]

mmu-let-7b-3p	NM_013454	-25.5	0.997943	NA	NA	GO:0030139,GO:0034358,GO:0016021,GO:0032934,GO:0032559,GO:0015405,GO:0015075,GO:0017111,GO:0019905,GO:0015248,GO:0051020,GO:0030226,GO:0007033,GO:0033363,GO:0015914,GO:0006811,GO:0055092,GO:0009207,GO:0016125,GO:0006464,GO:0010742,GO:00550	K05641|1|0.0|4380|mmu:11303|ATP-binding cassette, subfamily A (ABC1), member 1	gi|90568038|ref|NP_038482.3|	0	ATP-binding cassette sub-family A member 1 [Mus musculus]
mmu-let-7b-3p	NM_177823	-22.1	0.999853	NA	NA	GO:0043231,GO:0044444,GO:0001816,GO:0050856,	-	gi|165905557|ref|NP_808491.2|	0	ubiquitin-associated and SH3 domain-containing protein A [Mus musculus]

mmu-let-7b-3p	NM_013723	-20.9	1	NA	NA	GO:0005815,GO:0031224,GO:0031981,GO:0044459,GO:0031252,GO:0005902,GO:0005488,GO:0016477,GO:0030033,GO:0022409,GO:0035148,GO:0007162,GO:00023	K06817|1|0.0|733|mmu:27205|podocalyxin-like	gi|6448471|dbj|BAA86912.1|	0	podocalyxin-like protein 1 [Mus musculus]
mmu-let-7b-3p	NM_001165985	-22.6	0.999838	NA	NA	GO:0016020,GO:0043492,GO:0005488,GO:0005548,GO:0016820,GO:0035036,GO:00068	-	gi|260166711|ref|NP_001159457.1|	0	ubiquitin-associated protein 2-like isoform 5 [Mus musculus]
mmu-let-7b-3p	NM_023852	-20.1	1	NA	NA	GO:0030136,GO:0016020,GO:0032561,GO:0035556,GO:0006887,GO:0001505,	K07883|1|8e-120|434|mmu:67295|Ras-related protein Rab-3C	gi|13470090|ref|NP_076341.1|	5.63E-119	ras-related protein Rab-3C [Mus musculus]

mmu-let-7b-3p	NM_030241	-24	0.973316	NA	NA	GO:0043231,GO:0043232,GO:0005515,GO:0003712,GO:0016279,GO:0000122,GO:0016571,GO:0018022,GO:0009966,GO:00000	K11428|1|0.0|683|mmu:67956|histone-lysine N-methyltransferase SETD8 [EC:2.1.1.43]	gi|45580702|ref|NP_084517.2|	0	N-lysine methyltransferase SETD8 [Mus musculus]

mmu-let-7b-3p	NM_008628	-21.1	0.999999	NA	NA	GO:0032300,GO:0043231,GO:0005694,GO:0032404,GO:0046872,GO:0032356,GO:0019900,GO:0032139,GO:0032559,GO:0032138,GO:0042623,GO:0042802,GO:0043565,GO:0000217,GO:0006298,GO:0030098,GO:0006091,GO:0051095,GO:0008406,GO:0043009,GO:0048232,GO:00092	K08735|1|0.0|1806|mmu:17685|DNA mismatch repair protein MSH2	gi|6678938|ref|NP_032654.1|	0	DNA mismatch repair protein Msh2 [Mus musculus]
mmu-let-7b-3p	NM_022329	-26	0.761639	NA	NA	GO:0031224,GO:0031090,GO:0043231,GO:0005488,	K11794|1|3e-71|270|mcc:716691|IFRG15 protein	gi|11612513|ref|NP_071724.1|	4.61E-71	interferon alpha responsive protein isoform a [Mus musculus]

mmu-let-7b-3p	NM_178873	-28.5	0.494201	NA	NA	GO:0043231,GO:0031224,GO:0016301,GO:0032559,GO:0006796,	K08869|1|0.0|1110|mmu:57869|aarF domain-containing kinase	gi|30725855|ref|NP_849204.1|	0	uncharacterized aarF domain-containing protein kinase 2 [Mus musculus]
mmu-let-7b-3p	NR_040539	-23.9	0.748042	NA	NA	GO:0044421,GO:0071813,GO:0032934,GO:0010883,GO:0002455,GO:0010742,	-	gi|148683526|gb|EDL15473.1|	6.73E-58	mCG145237 [Mus musculus]
mmu-let-7b-3p	NM_010476	-26.6	0.8593	NA	NA	GO:0031090,GO:0043231,GO:0031224,GO:0005126,GO:0033764,GO:0008203,	K13373|1|0.0|670|mmu:15490|estradiol 17beta-dehydrogenase / 3-keto steroid reductase [EC:1.1.1.62 1.1.1.270]	gi|87162470|ref|NP_034606.3|	0	3-keto-steroid reductase [Mus musculus]

mmu-let-7b-3p	NM_001110223	-20.6	1	NA	NA	GO:0015630,GO:0043005,GO:0044444,GO:0015631,GO:0016477,GO:0009628,GO:0006935,GO:0048588,GO:0007165,GO:00219	K08805|1|2e-144|516|ssc:100623824|doublecortin and CaM kinase-like 1 [EC:2.7.11.1]	gi|397502923|ref|XP_003822087.1|	0	PREDICTED: neuronal migration protein doublecortin isoform 3 [Pan paniscus]
mmu-let-7b-3p	NM_177694	-20.7	1	NA	NA	GO:0031090,GO:0043231,GO:0034702,GO:0005253,GO:0015698,	K13982|1|2e-29|134|dgr:Dgri_GH10918|probable ATP-dependent RNA helicase DDX4 [EC:3.6.4.13]!K05169|2|1e-24|117|ptr:743996|single Ig IL-1R-related molecule	gi|55742666|ref|NP_808362.2|	0	anoctamin-5 [Mus musculus]
mmu-let-7b-3p	NM_173386	-20.1	1	NA	NA	-	-	gi|116812912|ref|NP_775562.2|	0	uncharacterized protein LOC214763 [Mus musculus]

mmu-let-7b-3p	NM_001177941	-20.4	1	NA	NA	GO:0016021,GO:0031090,GO:0005515,GO:0008544,GO:0010467,GO:0042476,GO:0048732,GO:0060605,GO:0042345,GO:0007249,GO:0031589,GO:0060788,GO:00301	K05480|1|7e-103|377|mmu:13607|ectodysplasin-A	gi|295789026|ref|NP_001171412.1|	6.88E-106	ectodysplasin-A isoform 6 [Mus musculus]
mmu-let-7b-3p	NM_012051	-20.4	1	NA	NA	GO:0043231,GO:0043234,GO:0001071,GO:0003677,GO:0006357,	K09433|1|0.0|1000|mmu:27049|ETS translocation variant 3	gi|134031941|ref|NP_001076787.1|	0	ETS translocation variant 3 [Mus musculus]
mmu-let-7b-3p	NM_026154	-23.7	0.922955	NA	NA	GO:0000315,GO:0043231,GO:0005198,GO:0010467,GO:0022613,	-	gi|13385658|ref|NP_080430.1|	1.44E-120	39S ribosomal protein L10, mitochondrial precursor [Mus musculus]
mmu-let-7b-3p	NM_019918	-24.4	0.952517	NA	NA	GO:0043005,GO:0031224,GO:0044444,GO:0004888,GO:0010033,GO:0023052,	K04613|1|0.0|1756|mmu:56544|vomeronasal 2 receptor	gi|114145503|ref|NP_064302.2|	0	vomeronasal type-2 receptor 1 precursor [Mus musculus]

mmu-let-7b-3p	NM_001164047	-23.4	0.853097	NA	NA	-	K13826|1|2e-11|70.9|ecb:100065056|hemoglobin subunit zeta!K13825|2|1e-10|67.8|ecb:100068926|hemoglobin subunit epsilon	gi|21359820|ref|NP_038621.2|	4.02E-83	myoglobin [Mus musculus]
mmu-let-7b-3p	NM_001164423	-20.2	1	NA	NA	GO:0031224,	-	gi|257096059|ref|NP_001157895.1|	1.59E-63	PQ-loop repeat-containing protein 1 isoform c [Mus musculus]
mmu-let-7b-3p	NM_011565	-20.8	0.999992	NA	NA	GO:0043231,GO:0043234,GO:0003676,GO:0000981,GO:0014020,GO:0035556,GO:00063	K09449|1|0.0|743|mmu:21677|TEA domain family member 2	gi|74201288|dbj|BAE26103.1|	0	unnamed protein product [Mus musculus]
mmu-let-7b-3p	NR_027907	-22.3	0.999995	NA	NA	-	-	gi|297269657|ref|XP_002799933.1|	4.69E-06	PREDICTED: hypothetical protein LOC100423696 [Macaca mulatta]
mmu-let-7b-3p	NM_010836	-25.9	0.732378	NA	NA	GO:0003677,GO:0006357,GO:0016573,	K09341|1|2e-82|308|mmu:17703|homeobox protein MSX	gi|30704771|gb|AAH51983.1|	1.85E-84	Msx3 protein [Mus musculus]

mmu-let-7b-3p	NM_001081362	-20.1	1	NA	NA	GO:0070461,GO:0005667,GO:0043189,GO:0016772,GO:0005488,GO:0003712,GO:0000075,GO:0016573,	K08874|1|0.0|7158|mmu:100683|transformation/transcription domain-associated protein	gi|124486949|ref|NP_001074831.1|	0	transformation/transcription domain-associated protein [Mus musculus]
mmu-let-7b-3p	NM_026175	-29.3	0.498706	NA	NA	GO:0005681,GO:0031981,GO:0003676,GO:0006376,	K12825|1|0.0|1134|mmu:67465|splicing factor 3A	gi|26324776|dbj|BAC26142.1|	0	unnamed protein product [Mus musculus]
mmu-let-7b-3p	NM_001085373	-22.7	1	NA	NA	-	-	gi|145699114|ref|NP_001078842.1|	0	mutated in colorectal cancers isoform 1 [Mus musculus]
mmu-let-7b-3p	NM_177872	-21.4	1	NA	NA	GO:0031012,GO:0004175,GO:0046914,GO:0005539,GO:0007166,GO:0032963,GO:0006508,GO:0030198,	K08619|1|0.0|2433|mmu:330119|a disintegrin and metalloproteinase with thrombospondin motifs 3 [EC:3.4.24.-]	gi|294345398|ref|NP_808540.2|	0	a disintegrin-like and metalloprotease with thrombospondin type 1 motif, 3 isoform 1 precursor [Mus musculus]

mmu-let-7b-3p	NM_177191	-23.2	0.999932	NA	NA	GO:0000795,GO:0003676,GO:0046983,GO:0012501,GO:0019953,GO:0007129,GO:00351	-	gi|109689713|ref|NP_796165.2|	0	synaptonemal complex protein 2 [Mus musculus]
mmu-let-7b-3p	NM_177608	-23.7	0.999882	NA	NA	-	-	gi|50510425|dbj|BAD32198.1|	0	mKIAA0256 protein [Mus musculus]
mmu-let-7b-3p	NM_027266	-25.1	0.80842	NA	NA	GO:0016740,GO:0006730,	K15445|1|2e-23|111|rno:295496|tRNA (guanine9-N1)-methyltransferase [EC:2.1.1.221]	gi|31560100|ref|NP_081542.2|	1.79E-160	RNA (guanine-9-)-methyltransferase domain-containing protein 3 [Mus musculus]
mmu-let-7b-3p	NM_008123	-26	0.974959	NA	NA	GO:0005921,GO:0031224,GO:0005488,GO:0022829,GO:0043010,GO:0051259,GO:00071	K07617|1|0.0|775|mmu:14616|gap junction protein, alpha 8	gi|6680009|ref|NP_032149.1|	0	gap junction alpha-8 protein [Mus musculus]
mmu-let-7b-3p	NM_001034903	-22.1	0.971279	NA	NA	GO:0044464,	-	gi|85702266|ref|NP_001030075.1|	0	MANSC domain-containing protein 4 precursor [Mus musculus]

mmu-let-7b-3p	NM_009922	-22.3	0.996545	NA	NA	GO:0043232,GO:0008092,GO:0006937,GO:0030036,	K06084|1|6e-12|73.6|bfo:BRAFLDRAFT_117217|F-box protein	gi|332852612|ref|XP_001168396.2|	2.80E-158	PREDICTED: calponin-1 isoform 3 [Pan troglodytes]
mmu-let-7b-3p	NM_011251	-26	0.873238	NA	NA	-	K13094|1|0.0|1013|tgu:100221173|RNA-binding protein 5/10	gi|160333291|ref|NP_035381.2|	0	RNA binding motif protein 6 isoform a [Mus musculus]
mmu-let-7b-3p	NM_015745	-26.6	0.894031	NA	NA	GO:0005578,GO:0005501,GO:0070011,GO:0051234,GO:0019538,GO:0050953,GO:00017	-	gi|21729751|ref|NP_056560.1|	0	retinol-binding protein 3 precursor [Mus musculus]
mmu-let-7b-5p	NM_001001335	-27.9	0.80973	-27.04	167	GO:0044424,GO:0005319,GO:0008289,GO:0006869,	K08051|1|0.0|944|rno:500132|pleckstrin homology domain containing, family A (phosphoinositide binding specific) member 8	gi|148666292|gb|EDK98708.1|	0	pleckstrin homology domain containing, family A (phosphoinositide binding specific) member 8, isoform CRA_a [Mus musculus]

mmu-let-7b-5p	NM_001007460	-28.5	0.18775	-43.16	300	GO:0031224,GO:0046914,GO:0016747,	K05766|1|9e-12|72.0|spu:575378|slingshot	gi|74184789|dbj|BAE27992.1|	0	unnamed protein product [Mus musculus]
mmu-let-7b-5p	NM_001007465	-33.6	0.075712	-26.04	167	GO:0043227,GO:0031410,GO:0046914,GO:0003824,GO:0012501,GO:0015031,	K10871|1|5e-70|267|mcc:718665|RAD51-like protein 3!K10641|3|3e-10|68.6|tad:TRIADDRAFT_53391|E3 ubiquitin-protein ligase LRSAM1 [EC:6.3.2.19]!K16060|4|4e-09|65.1|spu:581540|baculoviral IAP repeat-containing protein 2/3!K10637|5|5e-	gi|148683726|gb|EDL15673.1|	0	ring finger and FYVE like domain containing protein, isoform CRA_c [Mus musculus]

mmu-let-7b-5p	NM_001007589	-29.2	0.198782	-28.77	159	GO:0043231,GO:0043234,GO:0006357,GO:0001819,GO:0002237,GO:0007275,GO:00082	-	gi|86604728|ref|NP_001007590.2|	2.67E-69	akirin-2 [Mus musculus]
mmu-let-7b-5p	NM_001010973	-25.6	0.844932	-26.41	163	GO:0016021,GO:0008227,GO:0005488,GO:0002376,GO:0007186,	K04150|1|0.0|733|mmu:15466|histamine receptor H2	gi|58331134|ref|NP_001010973.1|	0	histamine H2 receptor isoform 1 [Mus musculus]
mmu-let-7b-5p	NM_001013774	-29.8	0.208931	-49.4	314	GO:0005634,GO:0006886,	K15043|1|2e-132|474|xla:398027|importin subunit alpha-2	gi|144446032|ref|NP_001013796.2|	0	importin subunit alpha-8 [Mus musculus]

mmu-let-7b-5p	NM_001014974	-30.2	0.344249	-49.46	316	GO:0005815,GO:0042995,GO:0016881,GO:0008092,GO:0032559,GO:0018200,	K06228|1|5e-52|207|ssc:100622459|fused [EC:2.7.11.1]!K00273|2|3e-51|205|ame:551507|D-amino-acid oxidase [EC:1.4.3.3]!K06047|3|2e-16|89.4|bta:514559|tubulin---tyrosine ligase [EC:6.3.2.2	gi|62510079|ref|NP_001014974.1|	0	tubulin polyglutamylase TTLL4 [Mus musculus]
mmu-let-7b-5p	NM_001025067	-25.8	0.983503	-44.41	316	GO:0044424,GO:0031224,	K06753|1|1e-30|137|acs:100559253|roundabout, axon guidance receptor	gi|148675623|gb|EDL07570.1|	0	leucine-rich repeats and immunoglobulin-like domains 2, isoform CRA_b [Mus musculus]

mmu-let-7b-5p	NM_001033171	-27.4	0.49691	-49.39	332	GO:0031224,GO:0030246,	K10069|1|1e-12|75.9|aml:100471954|C-type lectin domain family 1, member A!K10076|3|2e-12|75.5|ptr:736225|killer cell lectin-like receptor subfamily G, member 1!K10071|4|2e-12|75.1|mcc:717347|C-type lectin domain family 2, member	gi|119964698|ref|NP_001028343.2|	0	killer cell lectin-like receptor subfamily G member 2 [Mus musculus]

mmu-let-7b-5p	NM_001033263	-29.5	0.523538	-26.92	164	GO:0031981,GO:0044444,GO:0019900,GO:0032561,GO:0032559,GO:0008047,GO:0017111,GO:0005083,GO:0019887,GO:0046914,GO:0006357,GO:0001932,GO:0007010,GO:0032012,GO:0033674,GO:0009895,GO:0006935,GO:0043066,GO:0010740,GO:0043087,GO:0033598,GO:00161	K12491|1|0.0|1466|mmu:216439|Arf-GAP, GTPase, ANK repeat and PH domain-containing protein 1/2/3	gi|84370300|ref|NP_001028435.1|	0	arf-GAP with GTPase, ANK repeat and PH domain-containing protein 2 [Mus musculus]

mmu-let-7b-5p	NM_001033276	-25.6	0.999951	-44.39	306	GO:0043231,GO:0034708,GO:0016740,GO:0043565,GO:0008283,GO:0048545,GO:0030520,GO:0007292,GO:0016568,	K09187|1|0.0|4273|mmu:381022|histone-lysine N-methyltransferase MLL2 [EC:2.1.1.43]	gi|359718904|ref|NP_001028448.3|	0	histone-lysine N-methyltransferase MLL2 [Mus musculus]
mmu-let-7b-5p	NM_001033343	-26.4	0.84399	-33.74	170	GO:0030134,GO:0031090,GO:0006810,	K14005|1|0.0|2187|mmu:240667|protein transport protein SEC31	gi|133504851|ref|NP_001028515.1|	0	protein transport protein Sec31B [Mus musculus]
mmu-let-7b-5p	NM_001037493	-28	0.408012	-48.16	309	GO:0031224,GO:0015075,GO:0005488,GO:0006811,	K15122|1|0.0|838|mmu:71699|solute carrier family 41	gi|82734225|ref|NP_001032570.1|	0	solute carrier family 41 member 3 isoform 2 [Mus musculus]
mmu-let-7b-5p	NM_001037762	-25.7	0.491984	-48.62	317	-	-	gi|83649751|ref|NP_001032851.1|	7.39E-80	probable palmitoyltransferase ZDHHC12 isoform b [Mus musculus]
mmu-let-7b-5p	NM_001037842	-29.5	0.188699	-27.98	168	GO:0019866,GO:0016740,	-	gi|83745106|ref|NP_001032931.1|	2.86E-85	probable N-acetyltransferase CML3 isoform 1 [Mus musculus]

mmu-let-7b-5p	NM_001039090	-25.8	0.981726	-26.78	154	GO:0016604,GO:0030141,GO:0005515,GO:0003712,GO:0003676,GO:0051291,GO:0000122,GO:0007179,GO:0014706,GO:0010033,GO:0031346,GO:0001824,GO:0022402,GO:0006915,GO:0045595,GO:0048232,GO:0051260,GO:0001776,	-	gi|84875520|ref|NP_001034179.1|	0	ski-like protein isoform b [Mus musculus]
mmu-let-7b-5p	NM_001039653	-28.9	0.289135	-25.32	150	GO:0043231,GO:0043234,GO:0046914,GO:0001071,GO:0003677,GO:0007411,GO:0021536,GO:0006357,GO:0003002,GO:0021523,GO:0006915,	K09374|1|0.0|686|mmu:16871|LIM homeobox protein 3/4	gi|148676345|gb|EDL08292.1|	0	mCG18748, isoform CRA_b [Mus musculus]

mmu-let-7b-5p	NM_001040111	-25	0.991464	-25.37	158	GO:0000139,GO:0046914,GO:0035091,GO:0005099,GO:0032879,GO:0065008,GO:0031346,GO:0032232,GO:0032012,GO:00323	K12490|1|0.0|2544|mmu:69710|Arf-GAP, Rho-GAP domain, ANK repeat and PH domain-containing protein	gi|187954747|gb|AAI41180.1|	0	ArfGAP with RhoGAP domain, ankyrin repeat and PH domain 1 [Mus musculus]
mmu-let-7b-5p	NM_001042591	-31.5	0.20571	-27.28	174	GO:0044424,GO:0005488,GO:0023052,	K15183|1|1e-07|60.1|mgp:100549853|RNA polymerase II elongation factor ELL	gi|28972730|dbj|BAC65781.1|	0	mKIAA1376 protein [Mus musculus]

mmu-let-7b-5p	NM_001042760	-27.4	0.357653	-28.78	167	GO:0016021,GO:0044459,GO:0012505,GO:0044424,GO:0015238,GO:0008509,GO:0019899,GO:0015291,GO:0015893,GO:0003008,GO:0006812,	K08214|1|2e-168|592|mmu:18400|MFS transporter, OCT family, solute carrier family 22 (organic cation transporter), member 18	gi|112363086|ref|NP_001036225.1|	1.43E-167	solute carrier family 22 member 18 [Mus musculus]

mmu-let-7b-5p	NM_001081044	-33.2	0.072114	-31.1	169	GO:0044430,GO:0044297,GO:0030016,GO:0030425,GO:0033267,GO:0030136,GO:0017022,GO:0004674,GO:0032559,GO:0006937,GO:0060415,GO:0048167,GO:0010627,GO:0016192,GO:0006468,GO:0031443,GO:0048522,GO:0007049,GO:00420	K00907|1|0.0|1063|mmu:228785|myosin-light-chain kinase [EC:2.7.11.18]	gi|163644275|ref|NP_001074513.2|	0	myosin light chain kinase 2, skeletal/cardiac muscle [Mus musculus]

mmu-let-7b-5p	NM_001081125	-28	0.800559	-44.45	314	GO:0072372,GO:0043231,GO:0001071,GO:0043565,GO:0046914,GO:0030326,GO:0007224,GO:0001763,GO:0006357,GO:0021520,GO:0021521,GO:0048732,GO:0043009,GO:0021952,GO:0002062,GO:0014070,GO:0007418,GO:0033077,GO:0042476,GO:0048565,GO:0008544,GO:00215	K06230|1|0.0|2855|mmu:14633|zinc finger protein GLI	gi|124487481|ref|NP_001074594.1|	0	zinc finger protein GLI2 [Mus musculus]
mmu-let-7b-5p	NM_001081193	-29.1	0.519644	-25.62	158	GO:0031224,GO:0005637,GO:0005488,GO:0030509,GO:0007179,GO:0032924,	K13091|1|9e-23|110|tad:TRIADDRAFT_37071|RNA-binding protein	gi|145864461|ref|NP_001074662.2|	0	inner nuclear membrane protein Man1 [Mus musculus]

mmu-let-7b-5p	NM_001081347	-32.5	0.149251	-50.78	317	GO:0044444,GO:0016020,GO:0032561,GO:0035556,GO:0009966,	K07868|1|0.0|1371|mmu:69288|Rho-related BTB domain-containing protein 1/2	gi|124486809|ref|NP_001074816.1|	0	rho-related BTB domain-containing protein 1 isoform 1 [Mus musculus]
mmu-let-7b-5p	NM_001081477	-27.2	0.882086	-25.34	157	-	K11798|1|0.0|3097|mmu:382236|bromodomain and WD repeat domain containing protein 1/3	gi|125490358|ref|NP_001074946.1|	0	bromodomain and WD repeat-containing protein 3 [Mus musculus]
mmu-let-7b-5p	NM_001085370	-27.4	0.944903	-51.78	330	GO:0043231,GO:0004672,GO:0032559,GO:0030154,GO:0006464,GO:0008283,GO:0048513,	K08809|1|0.0|4939|rno:363256|striated muscle-specific serine/threonine protein kinase [EC:2.7.11.1]	gi|153792586|ref|NP_031489.4|	0	striated muscle-specific serine/threonine-protein kinase isoform 1 [Mus musculus]
mmu-let-7b-5p	NM_001085378	-26.5	0.934439	-25.41	167	GO:0016459,GO:0008092,GO:0017111,GO:0032559,	K10352|1|0.0|3052|mmu:668940|myosin heavy chain	gi|148674189|gb|EDL06136.1|	0	mCG21082 [Mus musculus]

mmu-let-7b-5p	NM_001085502	-28.7	0.144377	-26.24	162	GO:0044444,GO:0008252,GO:0046872,GO:0006213,GO:0006163,GO:0009112,GO:0046128,GO:0006796,	K01081|1|0.0|682|mmu:230718|5'-nucleotidase [EC:3.1.3.5]	gi|148227160|ref|NP_001078971.1|	0	cytosolic 5'-nucleotidase 1A [Mus musculus]
mmu-let-7b-5p	NM_001098203	-27.4	0.634823	-43.1	300	GO:0005622,GO:0003676,GO:0001071,GO:0006351,	K09221|1|3e-28|128|bta:523865|myeloid zinc finger	gi|148228529|ref|NP_001091673.1|	0	hypermethylated in cancer 1 protein isoform 2 [Mus musculus]
mmu-let-7b-5p	NM_001101479	-26.3	0.920997	-42.34	305	GO:0005488,	K13126|1|0.0|729|mmu:241989|polyadenylate-binding protein	gi|168229272|ref|NP_001094949.1|	0	poly(A) binding protein, cytoplasmic 4-like [Mus musculus]
mmu-let-7b-5p	NM_001109686	-27.1	0.823349	-28.16	160	GO:0043231,GO:0043232,GO:0046914,GO:0018024,GO:0005515,GO:0034968,GO:0006357,GO:0006305,GO:0018022,GO:00072	K11420|1|0.0|2303|mmu:77683|euchromatic histone-lysine N-methyltransferase [EC:2.1.1.43]	gi|157838004|ref|NP_001103156.1|	0	histone-lysine N-methyltransferase EHMT1 isoform 3 [Mus musculus]

mmu-let-7b-5p	NM_001109691	-25.4	0.988171	-25.03	163	GO:0031981,GO:0003676,GO:0046914,GO:0006357,GO:0007599,GO:0006325,GO:0007631,	K11643|1|4e-15|85.9|isc:IscW_ISCW005933|chromodomain-helicase-DNA-binding protein 4 [EC:3.6.4.12]	gi|157841178|ref|NP_001103161.1|	0	PHD finger protein 21A isoform 3 [Mus musculus]
mmu-let-7b-5p	NM_001109748	-25	0.759431	-25.2	168	GO:0031306,GO:0031301,GO:0008565,GO:0022829,GO:0005244,GO:0006605,GO:0034220,GO:0043269,	K11518|1|5e-160|565|mmu:53333|mitochondrial import receptor subunit TOM40	gi|6650562|gb|AAF21906.1|AF109918_1	3.69E-162	mitochondrial outer membrane protein MOM35 [Mus musculus]

mmu-let-7b-5p	NM_001110140	-31.4	0.237572	-46.26	312	GO:0032991,GO:0016021,GO:0005789,GO:0001664,GO:0019829,GO:0046872,GO:0032559,GO:0009888,GO:0007599,GO:0008016,GO:0006816,GO:0006936,GO:0009207,GO:0007165,GO:0001775,GO:0009206,GO:0009725,GO:0006875,	K05853|1|0.0|2035|mmu:11938|Ca2+ transporting ATPase, sarcoplasmic/endoplasmic reticulum [EC:3.6.3.8]	gi|60360584|dbj|BAD90532.1|	0	mKIAA4195 protein [Mus musculus]

mmu-let-7b-5p	NM_001111062	-26.5	0.622545	-25.22	153	GO:0043231,GO:0031224,GO:0044444,GO:0008171,GO:0046872,GO:0008202,GO:0006575,GO:0007611,GO:0033555,GO:0042053,GO:0006730,GO:0008285,GO:0042133,GO:0031337,GO:0002237,GO:00224	K00545|1|7e-136|485|mmu:12846|catechol O-methyltransferase [EC:2.1.1.6]	gi|161484634|ref|NP_031770.2|	4.88E-135	catechol O-methyltransferase [Mus musculus]
mmu-let-7b-5p	NM_001113360	-31.7	0.236255	-55	315	GO:0016020,GO:0060089,GO:0046872,GO:0004629,GO:0007165,GO:0006650,	K15370|1|6e-104|380|nve:NEMVE_v1g89019|inactive phospholipase C-like	gi|164565357|ref|NP_001106831.1|	0	1-phosphatidylinositol 4,5-bisphosphate phosphodiesterase eta-2 isoform b [Mus musculus]

mmu-let-7b-5p	NM_001114098	-27.7	0.859904	-42.4	311	-	K10388|1|2e-09|67.0|dre:567310|plectin!K10352|5|2e-08|63.9|smm:Smp_085540.6|myosin heavy chain	gi|166235131|ref|NP_766551.3|	0	coiled-coil domain-containing protein 165 isoform 2 [Mus musculus]
mmu-let-7b-5p	NM_001128169	-27.4	0.915327	-46.2	311	GO:0005874,GO:0044444,GO:0030529,GO:0009898,GO:0046914,GO:0005198,GO:0019783,GO:0016790,GO:0019900,GO:0000278,GO:0016579,GO:0001818,GO:0032886,GO:0043433,GO:0030178,GO:0042345,GO:00	K08601|1|0.0|1884|mmu:74256|ubiquitin thioesterase CYLD [EC:3.1.2.15]	gi|28972435|dbj|BAC65671.1|	0	mKIAA0849 protein [Mus musculus]

mmu-let-7b-5p	NM_001128170	-27.4	0.910374	-46.2	311	GO:0005874,GO:0044444,GO:0030529,GO:0009898,GO:0046914,GO:0005198,GO:0019783,GO:0016790,GO:0019900,GO:0000278,GO:0016579,GO:0001818,GO:0032886,GO:0043433,GO:0030178,GO:0042345,GO:00	K08601|1|0.0|1884|mmu:74256|ubiquitin thioesterase CYLD [EC:3.1.2.15]	gi|28972435|dbj|BAC65671.1|	0	mKIAA0849 protein [Mus musculus]

mmu-let-7b-5p	NM_001128171	-27.4	0.913333	-46.2	311	GO:0005874,GO:0044444,GO:0030529,GO:0009898,GO:0046914,GO:0005198,GO:0019783,GO:0016790,GO:0019900,GO:0000278,GO:0016579,GO:0001818,GO:0032886,GO:0043433,GO:0030178,GO:0042345,GO:00	K08601|1|0.0|1874|mmu:74256|ubiquitin thioesterase CYLD [EC:3.1.2.15]	gi|28972435|dbj|BAC65671.1|	0	mKIAA0849 protein [Mus musculus]
mmu-let-7b-5p	NM_001136240	-28.4	0.687549	-27.13	161	GO:0019866,GO:0043231,GO:0016614,GO:0050662,GO:0019695,	K00108|1|0.0|1245|mmu:218865|choline dehydrogenase [EC:1.1.99.1]	gi|148692803|gb|EDL24750.1|	0	choline dehydrogenase [Mus musculus]

mmu-let-7b-5p	NM_001142731	-28.2	0.534236	-26.81	162	-	K15074|1|8e-10|67.4|cbr:CBG18386|BTB/POZ domain-containing adapter for CUL3-mediated RhoA degradation protein	gi|218505696|ref|NP_001136203.1|	0	BTB/POZ domain-containing protein KCTD1 isoform 1 [Mus musculus]
mmu-let-7b-5p	NM_001145854	-30.2	0.294805	-26.59	166	GO:0004672,GO:0032559,GO:0006464,	K05735|1|0.0|1364|mmu:214230|p21-activated kinase 6 [EC:2.7.11.1]	gi|224994207|ref|NP_001028426.2|	0	serine/threonine-protein kinase PAK 6 [Mus musculus]
mmu-let-7b-5p	NM_001145970	-33.4	0.073089	-30.2	164	GO:0043232,	K10433|1|1e-18|96.7|acs:100551816|microtubule-associated protein 7	gi|148698350|gb|EDL30297.1|	0	arginine/proline rich coiled-coil 1, isoform CRA_c [Mus musculus]

mmu-let-7b-5p	NM_001160012	-29.9	0.165038	-25.42	164	GO:0005921,GO:0031224,GO:0005488,GO:0022829,GO:0043009,GO:0008544,GO:0050954,GO:0009987,	K07622|1|4e-136|485|mmu:14620|gap junction protein, beta 3	gi|6680013|ref|NP_032152.1|	3.12E-135	gap junction beta-3 protein [Mus musculus]
mmu-let-7b-5p	NM_001160019	-30.4	0.278471	-27.46	174	GO:0012505,GO:0019866,GO:0031224,GO:0043231,GO:0005515,	-	gi|229608946|ref|NP_001153490.1|	0	torsin-1A-interacting protein 1 isoform 1 [Mus musculus]
mmu-let-7b-5p	NM_001161365	-27.7	0.64274	-25.35	160	GO:0005768,GO:0031410,GO:0008047,GO:0031267,GO:0016192,GO:0043087,GO:00230	-	gi|238624151|ref|NP_808288.2|	0	ras and Rab interactor 3 isoform 1 [Mus musculus]

mmu-let-7b-5p	NM_001161433	-28.3	0.352474	-25.85	158	GO:0016021,GO:0005035,GO:0005488,GO:0043410,GO:0048869,GO:0009888,GO:0051091,GO:0008219,GO:0019221,GO:00072	K05163|1|5e-165|582|mmu:245527|tumor necrosis factor receptor superfamily member 27	gi|148682260|gb|EDL14207.1|	1.81E-166	ectodysplasin A2 isoform receptor, isoform CRA_b [Mus musculus]
mmu-let-7b-5p	NM_001161763	-26.3	0.920427	-65.91	458	GO:0005789,GO:0031224,GO:0016709,GO:0050662,GO:0000166,GO:0008152,	K00485|1|0.0|1078|mmu:14263|dimethylaniline monooxygenase (N-oxide forming) [EC:1.14.13.8]	gi|46559382|ref|NP_034362.2|	0	dimethylaniline monooxygenase [N-oxide-forming] 5 [Mus musculus]
mmu-let-7b-5p	NM_001161765	-26.3	0.923336	-65.91	458	GO:0005789,GO:0031224,GO:0016709,GO:0050662,GO:0000166,GO:0008152,	K00485|1|0.0|1078|mmu:14263|dimethylaniline monooxygenase (N-oxide forming) [EC:1.14.13.8]	gi|46559382|ref|NP_034362.2|	0	dimethylaniline monooxygenase [N-oxide-forming] 5 [Mus musculus]

mmu-let-7b-5p	NM_001163175	-29.6	0.275356	-27.45	163	GO:0044424,GO:0042802,	K06105|1|3e-45|184|dre:566060|tight junction protein 4 (peripheral)	gi|253683505|ref|NP_001156647.1|	0	brain-enriched guanylate kinase-associated protein [Mus musculus]
mmu-let-7b-5p	NM_001163625	-29.2	0.240401	-25.11	163	GO:0044424,GO:0016782,	K01025|1|7e-42|172|xla:447130|[EC:2.8.2.-]	gi|254692894|ref|NP_001157097.1|	8.16E-177	sulfotransferase 6B1 [Mus musculus]
mmu-let-7b-5p	NM_001163676	-26	0.95083	-41.54	305	GO:0044459,GO:0030667,GO:0042625,GO:0032559,GO:0009719,GO:0008284,GO:0010033,	K05673|1|0.0|2261|mmu:239273|ATP-binding cassette, subfamily C (CFTR/MRP), member 4	gi|255683328|ref|NP_001157148.1|	0	ATP-binding cassette, sub-family C (CFTR/MRP), member 4 isoform 3 [Mus musculus]

mmu-let-7b-5p	NM_001164361	-27.9	0.813934	-27.04	167	GO:0044424,GO:0005319,GO:0008289,GO:0006869,	K08051|1|0.0|972|rno:500132|pleckstrin homology domain containing, family A (phosphoinositide binding specific) member 8	gi|256355025|ref|NP_001157833.1|	0	pleckstrin homology domain-containing family A member 8 isoform 1 [Mus musculus]
mmu-let-7b-5p	NM_001164401	-27.9	0.806197	-29.85	165	GO:0016021,GO:0044424,GO:0004725,GO:0005488,GO:0004871,GO:0048731,GO:0006464,GO:0006796,	K01104|1|0.0|2278|mmu:19277|protein-tyrosine phosphatase [EC:3.1.3.48]	gi|257096042|ref|NP_001157873.1|	0	receptor-type tyrosine-protein phosphatase O isoform 2 precursor [Mus musculus]

mmu-let-7b-5p	NM_001164569	-33.6	0.079492	-26.04	167	GO:0043227,GO:0031410,GO:0046914,GO:0003824,GO:0012501,GO:0015031,	K10871|1|5e-70|267|mcc:718665|RAD51-like protein 3!K10641|3|4e-10|68.6|tad:TRIADDRAFT_53391|E3 ubiquitin-protein ligase LRSAM1 [EC:6.3.2.19]!K16060|4|4e-09|65.1|spu:581540|baculoviral IAP repeat-containing protein 2/3!K10637|5|5e-	gi|257196123|ref|NP_001158041.1|	0	E3 ubiquitin-protein ligase rififylin isoform 3 [Mus musculus]

mmu-let-7b-5p	NM_001164570	-33.6	0.07797	-26.04	167	GO:0043227,GO:0031410,GO:0046914,GO:0003824,GO:0012501,GO:0015031,	K10871|1|5e-70|267|mcc:718665|RAD51-like protein 3!K10641|3|4e-10|68.6|tad:TRIADDRAFT_53391|E3 ubiquitin-protein ligase LRSAM1 [EC:6.3.2.19]!K16060|4|4e-09|65.1|spu:581540|baculoviral IAP repeat-containing protein 2/3!K10637|5|5e-	gi|257153330|ref|NP_001158042.1|	0	E3 ubiquitin-protein ligase rififylin isoform 4 [Mus musculus]

mmu-let-7b-5p	NM_001164571	-33.6	0.073585	-26.04	167	GO:0043227,GO:0031410,GO:0046914,GO:0003824,GO:0012501,GO:0015031,	K10871|1|1e-69|266|mcc:718665|RAD51-like protein 3!K10641|3|3e-10|68.6|tad:TRIADDRAFT_53391|E3 ubiquitin-protein ligase LRSAM1 [EC:6.3.2.19]!K16060|4|4e-09|65.1|spu:581540|baculoviral IAP repeat-containing protein 2/3!K10637|5|5e-	gi|148683726|gb|EDL15673.1|	0	ring finger and FYVE like domain containing protein, isoform CRA_c [Mus musculus]

mmu-let-7b-5p	NM_001164679	-27.7	0.644652	-49.24	324	GO:0016021,GO:0005216,	K13982|1|9e-81|303|dgr:Dgri_GH10918|probable ATP-dependent RNA helicase DDX4 [EC:3.6.4.13]!K03018|3|2e-19|99.4|smm:Smp_002890|DNA-directed RNA polymerase III subunit RPC1 [EC:2.7.7.6]	gi|50511029|dbj|BAD32500.1|	0	mKIAA1623 protein [Mus musculus]
mmu-let-7b-5p	NM_001165952	-25.8	0.870856	-26.43	164	GO:0043231,GO:0046914,	K12021|1|0.0|1128|mmu:229644|tripartite motif-containing protein	gi|260099702|ref|NP_919324.2|	0	tripartite motif-containing protein 45 isoform b [Mus musculus]

mmu-let-7b-5p	NM_001165965	-31.7	0.226961	-29.57	178	-	K06252|1|5e-12|75.1|spu:576745|tenascin!K05717|3|1e-07|60.5|xtr:595087|fibronectin 1!K05698|4|2e-06|56.6|mdo:100619665|protein tyrosine phosphatase, receptor type, J [EC:3.1.3.48]	gi|260166644|ref|NP_001159437.1|	0	fibronectin type III domain-containing protein 7 isoform 2 precursor [Mus musculus]

mmu-let-7b-5p	NM_001168321	-30.7	0.392273	-25.17	162	GO:0043231,GO:0043234,GO:0003712,GO:0043565,GO:0019787,GO:0019899,GO:0000122,GO:0030279,GO:0007389,GO:0070168,GO:0009887,GO:0032446,GO:0000414,GO:00	K10683|1|3e-16|89.7|ecb:100053962|BRCA1-associated RING domain protein 1 [EC:6.3.2.19]	gi|37360460|dbj|BAC98208.1|	0	mKIAA1575 protein [Mus musculus]
mmu-let-7b-5p	NM_001177362	-31.8	0.051352	-27.52	167	GO:0031224,GO:0004871,	-	gi|148701316|gb|EDL33263.1|	3.65E-114	G protein-coupled receptor 137, isoform CRA_a [Mus musculus]
mmu-let-7b-5p	NM_001177561	-31.8	0.063358	-27.16	167	GO:0016021,GO:0044459,GO:0005343,GO:0008028,GO:0006820,GO:0035725,GO:0015718,GO:0008202,	K14341|1|0.0|664|mmu:20493|solute carrier family 10 (sodium/bile acid cotransporter), member 1	gi|294774569|ref|NP_001171032.1|	0	sodium/bile acid cotransporter isoform 1 [Mus musculus]

mmu-let-7b-5p	NM_001177995	-28.3	0.829767	-27.25	166	GO:0043231,GO:0043234,GO:0046914,GO:0005515,GO:0003712,GO:0003677,GO:0007423,GO:0002762,GO:0007179,GO:0006357,GO:0043467,GO:0045444,GO:0019827,GO:00	K04462|1|0.0|1054|cfa:488148|ecotropic virus integration site 1 protein	gi|295789156|ref|NP_001171466.1|	0	PR domain zinc finger protein 16 isoform 2 [Mus musculus]
mmu-let-7b-5p	NM_001190187	-28.8	0.500743	-25.16	158	GO:0016021,GO:0030296,GO:0005102,GO:0035556,GO:0060788,GO:0048732,GO:00160	K05457|1|0.0|1175|mmu:18183|neuregulin 3	gi|298231177|ref|NP_001177116.1|	0	pro-neuregulin-3, membrane-bound isoform isoform 2 [Mus musculus]
mmu-let-7b-5p	NM_001190188	-28.8	0.496601	-25.16	158	GO:0016021,GO:0030296,GO:0005102,GO:0035556,GO:0060788,GO:0048732,GO:00160	K05457|1|0.0|1098|mmu:18183|neuregulin 3	gi|298231179|ref|NP_001177117.1|	0	pro-neuregulin-3, membrane-bound isoform isoform 3 [Mus musculus]

mmu-let-7b-5p	NM_001190297	-31.9	0.212029	-29.76	154	GO:0031224,GO:0007165,GO:0006810,	K12365|1|4e-07|58.9|oaa:100075289|phosphatidylinositol-3,4,5-trisphosphate-dependent Rac exchanger 1 protein!K04351|2|5e-07|58.5|ecb:100064121|Rap guanine nucleotide exchange factor (GEF) 4	gi|298231160|ref|NP_001177226.1|	0	integral membrane protein GPR155 isoform 1 [Mus musculus]
mmu-let-7b-5p	NM_001195023	-27.4	0.767165	-50.04	327	GO:0043231,GO:0044444,GO:0016020,GO:0046914,GO:0005515,GO:0016044,GO:0043161,GO:0006996,	K14015|1|0.0|1221|mmu:217365|nuclear protein localization protein 4 homolog	gi|37360414|dbj|BAC98185.1|	0	mKIAA1499 protein [Mus musculus]

mmu-let-7b-5p	NM_001201332	-30.1	0.143714	-27.24	174	-	K13696|1|9e-17|89.4|bfo:BRAFLDRAFT_117028|abhydrolase domain-containing protein 1/3	gi|318083343|ref|NP_001188261.1|	3.42E-83	uncharacterized protein LOC73667 isoform 1 [Mus musculus]
mmu-let-7b-5p	NM_001201333	-30.1	0.139925	-27.24	174	-	K13696|1|6e-07|47.8|bfo:BRAFLDRAFT_117028|abhydrolase domain-containing protein 1/3	gi|318804024|ref|NP_001188262.1|	2.88E-119	uncharacterized protein LOC73667 isoform 2 [Mus musculus]
mmu-let-7b-5p	NM_001205068	-28.5	0.580807	-46.62	312	-	K11323|1|1e-17|94.0|dwi:Dwil_GK14253|histone arginine demethylase JMJD6 [EC:1.14.1	gi|326633177|ref|NP_001191997.1|	0	jmjC domain-containing protein 4 isoform 2 [Mus musculus]

mmu-let-7b-5p	NM_001205239	-25.3	0.873223	-25.06	159	GO:0016763,	K15258|1|0.0|1186|mmu:67287|poly [ADP-ribose] polymerase 6/8 [EC:2.4.2.30]	gi|327365350|ref|NP_001192168.1|	0	poly [ADP-ribose] polymerase 6 isoform 1 [Mus musculus]
mmu-let-7b-5p	NM_001252067	-26.6	0.779534	-43.65	318	GO:0044431,GO:0030119,GO:0031267,GO:0015031,	K12404|1|0.0|972|rno:360658|ADP-ribosylation factor-binding protein GGA	gi|148702556|gb|EDL34503.1|	0	golgi associated, gamma adaptin ear containing, ARF binding protein 3, isoform CRA_b [Mus musculus]
mmu-let-7b-5p	NM_001252636	-32.5	0.148614	-50.78	317	GO:0044444,GO:0016020,GO:0032561,GO:0035556,GO:0009966,	K07868|1|0.0|1371|mmu:69288|Rho-related BTB domain-containing protein 1/2	gi|124486809|ref|NP_001074816.1|	0	rho-related BTB domain-containing protein 1 isoform 1 [Mus musculus]
mmu-let-7b-5p	NM_001252637	-25.1	0.972265	-50.78	317	GO:0044444,GO:0016020,GO:0032561,GO:0035556,GO:0009966,	K07868|1|0.0|1371|mmu:69288|Rho-related BTB domain-containing protein 1/2	gi|124486809|ref|NP_001074816.1|	0	rho-related BTB domain-containing protein 1 isoform 1 [Mus musculus]

mmu-let-7b-5p	NM_001252638	-25.1	0.973438	-50.78	317	GO:0044444,GO:0016020,GO:0032561,GO:0035556,GO:0009966,	K07868|1|0.0|1167|mmu:69288|Rho-related BTB domain-containing protein 1/2	gi|124486809|ref|NP_001074816.1|	0	rho-related BTB domain-containing protein 1 isoform 1 [Mus musculus]
mmu-let-7b-5p	NM_001271772	-25.8	0.978549	-26.78	154	-	-	-	-	-
mmu-let-7b-5p	NM_007379	-25.4	0.995168	-27.26	161	GO:0015630,GO:0005764,GO:0031224,GO:0031410,GO:0043234,GO:0017111,GO:0015405,GO:0032559,GO:0016125,GO:0006355,GO:0009207,GO:0032367,GO:0009725,GO:00	K05642|1|0.0|4579|mmu:11305|ATP-binding cassette, subfamily A (ABC1), member 2	gi|110225379|ref|NP_031405.2|	0	ATP-binding cassette sub-family A member 2 [Mus musculus]

mmu-let-7b-5p	NM_007419	-31	0.14529	-44.96	299	GO:0043231,GO:0016021,GO:0031690,GO:0043176,GO:0046983,GO:0004939,GO:0002027,GO:0050804,GO:0033555,GO:0010613,GO:0001659,GO:0032414,GO:0046541,GO:0045762,GO:0002021,GO:0005977,GO:0006940,GO:0007611,GO:0007600,GO:0045444,GO:0007265,GO:00019	K04141|1|0.0|660|mmu:11554|adrenergic receptor beta-1	gi|158517827|ref|NP_031445.2|	0	beta-1 adrenergic receptor [Mus musculus]

mmu-let-7b-5p	NM_007463	-30.5	0.612186	-51.78	330	GO:0043231,GO:0004672,GO:0032559,GO:0030154,GO:0006464,GO:0008283,GO:0048513,	K08809|1|0.0|5477|rno:363256|striated muscle-specific serine/threonine protein kinase [EC:2.7.11.1]	gi|148667988|gb|EDL00405.1|	0	SPEG complex locus, isoform CRA_b [Mus musculus]
mmu-let-7b-5p	NM_007566	-25.3	0.999916	-28.07	162	-	K10586|1|0.0|8523|mmu:12211|baculoviral IAP repeat-containing protein 6 (apollon) [EC:6.3.2.19]	gi|380876899|sp|O88738.2|BIRC6_MOUSE	0	RecName: Full=Baculoviral IAP repeat-containing protein 6; AltName: Full=BIR repeat-containing ubiquitin-conjugating enzyme; Short=BRUCE; AltName: Full=Ubiquitin-conjugating BIR domain enzyme apollon; Short=APOLLON

mmu-let-7b-5p	NM_007583	-25.2	0.898997	-28.19	176	GO:0030139,GO:0034704,GO:0005262,GO:0016247,GO:0035254,GO:0048741,GO:0007267,GO:0003008,GO:0032412,GO:0006816,GO:0042391,	K04867|1|1e-164|581|mmu:12300|voltage-dependent calcium channel gamma-2	gi|148697713|gb|EDL29660.1|	6.25E-165	calcium channel, voltage-dependent, gamma subunit 2, isoform CRA_a [Mus musculus]
mmu-let-7b-5p	NM_007719	-27	0.502207	-44.89	311	GO:0009986,GO:0016021,GO:0004950,GO:0006952,GO:0051480,GO:0002376,GO:0007166,GO:0042221,	K04182|1|0.0|716|mmu:12775|C-C chemokine receptor type 7	gi|116268121|ref|NP_031745.2|	0	C-C chemokine receptor type 7 precursor [Mus musculus]

mmu-let-7b-5p	NM_007744	-26.5	0.611957	-25.22	153	GO:0043231,GO:0031224,GO:0044444,GO:0008171,GO:0046872,GO:0008202,GO:0006575,GO:0007611,GO:0033555,GO:0042053,GO:0006730,GO:0008285,GO:0042133,GO:0031337,GO:0002237,GO:00224	K00545|1|7e-136|485|mmu:12846|catechol O-methyltransferase [EC:2.1.1.6]	gi|161484634|ref|NP_031770.2|	4.72E-135	catechol O-methyltransferase [Mus musculus]
mmu-let-7b-5p	NM_007780	-27.9	0.701335	-25.22	158	GO:0005887,GO:0004896,GO:0002237,GO:0032501,GO:0007166,	K04738|1|0.0|1665|mmu:12983|cytokine receptor common subunit beta	gi|165972513|ref|NP_031806.3|	0	cytokine receptor common subunit beta precursor [Mus musculus]

mmu-let-7b-5p	NM_008052	-26.1	0.864497	-28.21	159	GO:0043231,GO:0003712,GO:0046914,GO:0019899,GO:0005102,GO:0019904,GO:0045596,GO:0007219,GO:0006351,GO:00455	K06058|1|0.0|1070|rno:687424|deltex	gi|60360548|dbj|BAD90518.1|	0	mKIAA4160 protein [Mus musculus]
mmu-let-7b-5p	NM_008126	-29.9	0.136044	-25.42	164	GO:0005921,GO:0031224,GO:0005488,GO:0022829,GO:0043009,GO:0008544,GO:0050954,GO:0009987,	K07622|1|4e-136|485|mmu:14620|gap junction protein, beta 3	gi|6680013|ref|NP_032152.1|	2.54E-135	gap junction beta-3 protein [Mus musculus]
mmu-let-7b-5p	NM_008158	-32.2	0.030893	-28.46	163	GO:0016020,GO:0004871,GO:0007166,	K04301|1|2e-128|459|rno:65275|G protein-coupled receptor	gi|6680073|ref|NP_032184.1|	1.23E-127	probable G-protein coupled receptor 27 [Mus musculus]

mmu-let-7b-5p	NM_008254	-30	0.112024	-27.8	151	GO:0019866,GO:0044429,GO:0043169,GO:0016833,GO:0005504,GO:0033993,GO:0044249,GO:0006996,GO:0031667,GO:0006732,GO:0048513,GO:0006950,GO:00434	K01640|1|0.0|638|mmu:15356|hydroxymethylglutaryl-CoA lyase [EC:4.1.3.4]	gi|171543858|ref|NP_032280.2|	0	hydroxymethylglutaryl-CoA lyase, mitochondrial precursor [Mus musculus]
mmu-let-7b-5p	NM_008305	-26.9	0.991303	-41.99	305	GO:0005604,GO:0016020,GO:0005515,GO:0007417,GO:0043062,GO:0001503,GO:0044238,GO:0051216,GO:0048646,GO:0044260,GO:0048562,GO:0030154,GO:0014706,GO:00	K06255|1|0.0|8774|mmu:15530|heparan sulfate proteoglycan 2 (perlecan)	gi|183979966|ref|NP_032331.2|	0	basement membrane-specific heparan sulfate proteoglycan core protein precursor [Mus musculus]

mmu-let-7b-5p	NM_008355	-33	0.023508	-30.56	174	GO:0044424,GO:0044421,GO:0005126,GO:0015992,GO:0010524,GO:0009612,GO:0001818,GO:0030888,GO:0023052,GO:0002377,GO:0007154,GO:0008284,GO:0002696,GO:0014070,GO:0002237,GO:00069	K05435|1|3e-56|219|mmu:16163|interleukin 13	gi|6680403|ref|NP_032381.1|	1.79E-55	interleukin-13 precursor [Mus musculus]
mmu-let-7b-5p	NM_008480	-27	0.962278	-48.52	305	-	K05637|1|0.0|6084|mmu:16772|laminin, alpha 1/2	gi|117168301|ref|NP_032506.2|	0	laminin subunit alpha-1 precursor [Mus musculus]
mmu-let-7b-5p	NM_008734	-28.8	0.501018	-25.16	158	GO:0016021,GO:0030296,GO:0005102,GO:0035556,GO:0060788,GO:0048732,GO:00160	K05457|1|0.0|1169|mmu:18183|neuregulin 3	gi|6679128|ref|NP_032760.1|	0	pro-neuregulin-3, membrane-bound isoform isoform 1 [Mus musculus]

mmu-let-7b-5p	NM_008767	-33.5	0.025961	-28.78	167	GO:0016021,GO:0044459,GO:0012505,GO:0044424,GO:0015238,GO:0008509,GO:0019899,GO:0015291,GO:0015893,GO:0003008,GO:0006812,	K08214|1|2e-168|592|mmu:18400|MFS transporter, OCT family, solute carrier family 22 (organic cation transporter), member 18	gi|112363086|ref|NP_001036225.1|	1.38E-167	solute carrier family 22 member 18 [Mus musculus]

mmu-let-7b-5p	NM_008771	-28.2	0.40638	-27.42	151	GO:0032991,GO:0016021,GO:0009279,GO:0046914,GO:0005231,GO:0005261,GO:0032559,GO:0016502,GO:0006940,GO:0007599,GO:0001508,GO:0006672,GO:0010959,GO:0002442,GO:0010033,GO:0006816,GO:0035587,GO:0042310,GO:0051259,GO:0006915,GO:0001775,GO:00072	K05215|1|0.0|828|mmu:18436|purinergic receptor P2X, ligand-gated ion channel 1	gi|158711719|ref|NP_032797.3|	0	P2X purinoceptor 1 [Mus musculus]

mmu-let-7b-5p	NM_008824	-40.2	0.001521	-39.8	188	GO:0043234,GO:0044444,GO:0070061,GO:0008443,GO:0019899,GO:0019203,GO:0032559,GO:0006007,GO:0035556,GO:0033131,GO:0009887,GO:0043434,GO:0031960,GO:0015980,GO:0006000,GO:00069	K01103|1|0.0|910|mmu:18639|6-phosphofructo-2-kinase / fructose-2,6-bisphosphatase [EC:2.7.1.105 3.1.3.46]	gi|7434494|pir||S77704	0	6-phosphofructo-2-kinase (EC 2.7.1.105) / fructose-2, 6-bisphosphate 2-phosphatase (EC 3.1.3.46) clone 5c, skeletal muscle - rat
mmu-let-7b-5p	NM_008906	-26.6	0.798347	-48	309	GO:0000323,GO:0031981,GO:0005488,GO:0004180,GO:0030234,GO:0019538,GO:0015031,	K13289|1|0.0|979|mmu:19025|cathepsin A (carboxypeptidase C) [EC:3.4.16.5]	gi|84042525|ref|NP_032932.2|	0	lysosomal protective protein isoform a precursor [Mus musculus]

mmu-let-7b-5p	NM_008970	-25.5	0.638539	-31.91	151	GO:0043231,GO:0044421,GO:0005102,GO:0002062,GO:0001649,GO:0060560,GO:0051604,GO:0008283,GO:0043392,GO:0007154,GO:0006171,GO:0007188,GO:0048875,GO:0048646,GO:0022414,	-	gi|148678784|gb|EDL10731.1|	9.70E-81	parathyroid hormone-like peptide, isoform CRA_a [Mus musculus]
mmu-let-7b-5p	NM_009211	-25.5	0.977622	-41.76	308	GO:0070603,GO:0001739,GO:0003712,GO:0003676,GO:0005515,GO:0016568,GO:0048731,GO:0006357,GO:0007169,GO:0009653,	K11649|1|0.0|1677|mmu:20588|SWI/SNF related-matrix-associated actin-dependent regulator of chromatin subfamily C	gi|148677078|gb|EDL09025.1|	0	SWI/SNF related, matrix associated, actin dependent regulator of chromatin, subfamily c, member 1, isoform CRA_b [Mus musculus]

mmu-let-7b-5p	NM_001170746	-21	1	-68.3	477	GO:0044459,GO:0032991,GO:0019904,GO:0019899,GO:0007172,GO:0051259,	K05629|1|0.0|2370|rno:113970|atrophin-1 interacting protein 1	gi|282721034|ref|NP_001164217.1|	0	membrane-associated guanylate kinase, WW and PDZ domain-containing protein 2 isoform 1 [Mus musculus]
mmu-let-7c-1-3p	NM_001033438	-28.8	0.35781	-25.23	163	-	-	gi|85701949|ref|NP_001028610.1|	0	uncharacterized protein LOC380882 [Mus musculus]
mmu-let-7c-1-3p	NM_001033770	-27.6	0.096716	-25.23	163	-	-	gi|347582644|ref|NP_001231578.1|	5.24E-66	uncharacterized protein LOC667693 [Mus musculus]
mmu-let-7c-1-3p	NM_001168535	-29.3	0.304547	-25.48	166	GO:0046914,	K01493|1|1e-08|63.5|phu:Phum_PHUM008070|dCMP deaminase [EC:3.5.4.1	gi|148704183|gb|EDL36130.1|	0	mCG13855, isoform CRA_f [Mus musculus]
mmu-let-7c-1-3p	NM_001168538	-29.3	0.300652	-25.48	166	GO:0003824,GO:0046914,	K01493|1|1e-08|63.5|phu:Phum_PHUM008070|dCMP deaminase [EC:3.5.4.1	gi|148704184|gb|EDL36131.1|	0	mCG13855, isoform CRA_g [Mus musculus]

mmu-let-7c-1-3p	NM_001244649	-28.8	0.353404	-25.23	163	-	-	gi|347582644|ref|NP_001231578.1|	0	uncharacterized protein LOC667693 [Mus musculus]
mmu-let-7c-1-3p	NM_001256104	-29.6	0.160617	-26.53	160	GO:0031224,GO:0005343,GO:0006820,GO:0033189,GO:0015718,GO:0035725,	K14343|1|0.0|731|mmu:214601|solute carrier family 10 (sodium/bile acid cotransporter), member 3/5	gi|21703812|ref|NP_663381.1|	0	P3 protein [Mus musculus]
mmu-let-7c-1-3p	NM_008498	-29.2	0.271849	-26.42	162	GO:0043231,GO:0043234,GO:0046914,GO:0001071,GO:0003677,GO:0007267,GO:0048568,GO:0048793,GO:0006357,GO:0021533,GO:0001667,GO:0003002,GO:0009792,GO:0009798,GO:0022037,GO:0001704,GO:00	K09372|1|0.0|725|rno:257634|LIM homeobox protein 1/5	gi|6678688|ref|NP_032524.1|	0	LIM/homeobox protein Lhx1 [Mus musculus]

mmu-let-7c-1-3p	NM_010500	-27.8	0.510652	-27.54	158	-	-	gi|6754284|ref|NP_034630.1|	9.23E-112	immediate early response gene 5 protein [Mus musculus]
mmu-let-7c-1-3p	NM_011076	-32.8	0.126503	-27.23	173	GO:0005911,GO:0031224,GO:0012505,GO:0032559,GO:0042626,GO:0048511,GO:0007589,GO:0010033,GO:0015893,GO:0010941,GO:0009207,GO:0033273,GO:00100	K05658|1|0.0|2296|mmu:18671|ATP-binding cassette, subfamily B (MDR/TAP), member 1	gi|225734206|pdb|3G60|A	0	Chain A, Structure Of P-Glycoprotein Reveals A Molecular Basis For Poly-Specific Drug Binding

mmu-let-7c-1-3p	NM_011261	-28.8	0.811909	-26.42	157	GO:0043005,GO:0044424,GO:0043025,GO:0031012,GO:0070011,GO:0004672,GO:0043169,GO:0033674,GO:0051705,GO:0009611,GO:0021536,GO:0006575,GO:0007611,GO:0033555,GO:0021766,GO:0000902,GO:0031175,GO:0003002,GO:0006468,GO:0009967,GO:0010038,GO:00011	K06249|1|0.0|7111|mmu:19699|reelin [EC:3.4.21.-]	gi|117320554|ref|NP_035391.2|	0	reelin precursor [Mus musculus]
mmu-let-7c-1-3p	NM_011855	-28.4	0.762141	-25.47	169	GO:0016021,GO:0005539,GO:0048731,GO:0008283,GO:0002376,GO:0023052,	K06252|1|1e-55|220|oaa:100076692|tenascin	gi|7657413|ref|NP_035985.2|	0	teneurin-1 [Mus musculus]

mmu-let-7c-1-3p	NM_018782	-26.9	0.787596	-25.38	171	GO:0016021,GO:0004930,GO:0031348,GO:0007188,GO:0048513,GO:0008284,GO:00069	K04577|1|0.0|889|mmu:54598|calcitonin receptor-like	gi|213385308|ref|NP_061252.2|	0	calcitonin gene-related peptide type 1 receptor precursor [Mus musculus]
mmu-let-7c-1-3p	NM_025605	-27.6	0.212019	-25.76	161	GO:0005764,GO:0031902,GO:0044425,GO:0005488,GO:0051640,GO:0040007,GO:0001881,GO:0007033,GO:0019218,GO:0008104,GO:0010740,GO:0043200,GO:0032374,GO:00	-	gi|242332593|ref|NP_079881.2|	4.74E-85	ragulator complex protein LAMTOR1 [Mus musculus]
mmu-let-7c-1-3p	NM_026572	-28.5	0.163455	-25.4	166	GO:0043234,GO:0043231,GO:0005515,GO:0008168,GO:0006546,GO:0006730,	K02437|1|8e-91|334|mmu:68133|glycine cleavage system H protein	gi|13386066|ref|NP_080848.1|	5.68E-90	glycine cleavage system H protein, mitochondrial precursor [Mus musculus]

mmu-let-7c-1-3p	NM_027399	-30.1	0.070542	-27.99	167	GO:0030054,GO:0031090,GO:0043231,GO:0016021,GO:0046914,GO:0022803,GO:0050662,GO:0003824,GO:0000041,GO:00060	K14737|1|0.0|662|mmu:70358|metalloreductase STEAP1 [EC:1.16.1.-]	gi|74188352|dbj|BAE25828.1|	0	unnamed protein product [Mus musculus]

mmu-let-7c-1-3p	NM_027801	-29.2	0.354629	-25.09	160	-	K11423|1|1e-08|63.5|dgr:Dgri_GH16034|histone-lysine N-methyltransferase SETD2 [EC:2.1.1.43]!K11515|2|2e-07|59.3|oaa:100090319|inner centromere protein!K10352|3|3e-06|55.5|dwi:Dwil_GK20720|myosin heavy chain	gi|298358637|ref|NP_082077.1|	0	uncharacterized protein LOC212153 [Mus musculus]
mmu-let-7c-1-3p	NM_028627	-26	0.849204	-25.01	154	GO:0031224,GO:0044424,GO:0005083,GO:0060089,GO:0015216,GO:0006863,GO:0019222,GO:0032011,GO:0030154,	K12494|1|0.0|1716|mmu:73728|PH and SEC7 domain-containing protein	gi|148710042|gb|EDL41988.1|	0	pleckstrin and Sec7 domain containing, isoform CRA_b [Mus musculus]

mmu-let-7c-1-3p	NM_139304	-28.6	0.235476	-26.13	165	GO:0031981,GO:0046914,GO:0003677,GO:0001071,GO:0006351,	K10310|1|4e-20|100|nvi:100122742|F-box protein 33	gi|21314854|ref|NP_647465.1|	0	transcriptional repressor p66-beta [Mus musculus]
mmu-let-7c-1-3p	NM_144789	-28.6	0.636859	-25.5	161	GO:0043231,GO:0046914,GO:0003676,GO:0006351,	K09228|1|2e-74|283|ssc:100512531|KRAB domain-containing zinc finger protein	gi|226531251|ref|NP_659038.2|	0	PR domain containing 15 [Mus musculus]
mmu-let-7c-1-3p	NM_145406	-29.6	0.167168	-26.53	160	GO:0031224,GO:0005343,GO:0006820,GO:0033189,GO:0015718,GO:0035725,	K14343|1|0.0|731|mmu:214601|solute carrier family 10 (sodium/bile acid cotransporter), member 3/5	gi|21703812|ref|NP_663381.1|	0	P3 protein [Mus musculus]
mmu-let-7c-1-3p	NM_172476	-26.5	0.823389	-25.12	169	-	-	gi|31542282|ref|NP_766064.2|	0	transmembrane channel-like protein 7 [Mus musculus]

mmu-let-7c-1-3p	NM_172711	-30.2	0.269782	-25.18	170	GO:0043231,GO:0019866,GO:0017111,GO:0032561,GO:0009207,GO:0010467,	K03596|1|0.0|753|nve:NEMVE_v1g106275|GTP-binding protein LepA!K02355|2|4e-28|128|cbr:CBG15144|elongation factor G!K14536|3|1e-25|120|bta:527607|ribosome assembly protein 1 [EC:3.6.5.-	gi|27370038|ref|NP_766299.1|	0	translation factor Guf1, mitochondrial [Mus musculus]
mmu-let-7c-1-3p	NM_197943	-30.8	0.259622	-27.81	166	GO:0044464,GO:0005099,GO:0032313,	K13136|1|6e-27|124|nvi:100114269|gem associated protein 8	gi|28972203|dbj|BAC65555.1|	0	mKIAA0397 protein [Mus musculus]
mmu-let-7c-1-3p	NR_045335	-28.7	0.316245	-25.72	163	-	-	gi|74209595|dbj|BAE23557.1|	6.97E-30	unnamed protein product [Mus musculus]

mmu-let-7c-1-3p	NM_012005	-21.6	1	-42.78	309	GO:0043234,GO:0031981,GO:0004871,GO:0008134,GO:0003713,GO:0006357,GO:0030518,GO:0006352,	K15156|1|0.0|2559|mmu:26896|mediator of RNA polymerase II transcription subunit 14	gi|115270972|ref|NP_036135.3|	0	mediator of RNA polymerase II transcription subunit 14 isoform b [Mus musculus]

mmu-let-7c-1-3p	NM_172946	-28.5	0.349473	-24.55	182	GO:0043234,	K07604|1|2e-137|491|cfa:491009|type I keratin, acidic!K11651|4|7e-30|133|tgu:100232351|SWI/SNF-related matrix-associated actin-dependent regulator of chromatin subfamily E, member 1!K10379|5|3e-21|105|dre:494090|beaded	gi|27370460|ref|NP_766534.1|	3.34E-152	keratin-like protein KRT222 [Mus musculus]
mmu-let-7c-1-3p	NM_001166497	-27.8	0.62085	-23.76	179	GO:0046872,	K09228|1|0.0|881|rno:499056|KRAB domain-containing zinc finger protein	gi|262073018|ref|NP_001159969.1|	0	zinc finger protein 51-like [Mus musculus]

mmu-let-7c-1-3p	NM_009827	-27.2	0.561619	-22.08	178	GO:0016021,GO:0000323,GO:0033267,GO:0008188,GO:0032414,GO:0007589,GO:0010646,GO:0030036,GO:0051480,GO:0048667,GO:0007420,GO:0070253,GO:0009628,GO:0002021,GO:0009887,GO:0007186,GO:0031960,GO:0006950,GO:0007631,GO:0006813,GO:0002237,GO:00226	K04194|1|0.0|747|rno:24889|cholecystokinin A receptor	gi|6978615|ref|NP_036820.1|	0	cholecystokinin receptor type A [Rattus norvegicus]

mmu-let-7c-1-3p	NM_008054	-31.2	0.16301	-24.6	176	GO:0016020,GO:0043231,GO:0044444,GO:0051427,GO:0004713,GO:0008092,GO:0032559,GO:0043169,GO:0019900,GO:0007599,GO:0007611,GO:0010467,GO:0010033,GO:0050851,GO:0009895,GO:0050974,GO:0007420,GO:0006468,GO:0031294,GO:0050688,GO:0070838,GO:00069	K05703|1|0.0|1068|mmu:14360|proto-oncogene tyrosine-protein kinase Fyn [EC:2.7.10.2]	gi|171543837|ref|NP_032080.2|	0	tyrosine-protein kinase Fyn isoform b [Mus musculus]

mmu-let-7c-1-3p	NM_183276	-26.7	0.949137	-21.92	172	-	K07295|1|4e-26|122|dpe:Dper_GL19043|nuclear receptor subfamily 2 group A!K11887|2|3e-09|67.0|ame:409686|proteasomal ATPase-associated factor 1!K10342|5|7e-08|62.4|mcc:710401|WD repeat and SOCS box-containing protein 2	gi|254911027|ref|NP_899099.2|	0	neurobeachin-like protein 2 [Mus musculus]
mmu-let-7c-1-3p	NM_207281	-26.2	0.752361	-22.34	171	-	K02357|1|3e-06|55.5|cfa:481134|elongation factor Ts	gi|46402315|ref|NP_997164.1|	1.12E-138	methyltransferase-like protein LOC121952 homolog [Mus musculus]
mmu-let-7c-1-3p	NR_045940	-27.5	0.178277	-21.51	170	-	-	gi|148675213|gb|EDL07160.1|	4.65E-16	mCG144577 [Mus musculus]

mmu-let-7c-1-3p	NM_023294	-26.9	0.497194	-20.12	169	GO:0044444,GO:0000778,GO:0005488,GO:0000819,GO:0040001,GO:0048017,GO:00070	K11547|1|0.0|1180|mmu:67052|kinetochore protein NDC80	gi|217035142|ref|NP_075783.2|	0	kinetochore protein NDC80 homolog [Mus musculus]
mmu-let-7c-1-3p	NM_029282	-29.1	0.380548	-21.9	168	-	-	gi|33859732|ref|NP_083558.1|	0	TELO2-interacting protein 1 homolog [Mus musculus]
mmu-let-7c-1-3p	NR_038062	-28.4	0.255916	-24	167	GO:0016023,GO:0031224,	-	gi|148693255|gb|EDL25202.1|	1.57E-158	Yip1 domain family, member 2, isoform CRA_a [Mus musculus]
mmu-let-7c-1-3p	NR_003188	-21.8	0.998366	-20.19	166	-	-	gi|148697167|gb|EDL29114.1|	1.64E-12	mCG147982 [Mus musculus]

mmu-let-7c-1-3p	NM_178804	-25.4	0.990164	-21.16	165	GO:0044424,GO:0016020,GO:0044421,GO:0004857,GO:0005102,GO:0046872,GO:0005539,GO:0042802,GO:0043394,GO:0043236,GO:0048675,GO:0010467,GO:0001763,GO:0006915,GO:0001932,GO:0031290,GO:0043009,GO:0002689,GO:0031348,GO:0051384,GO:0021827,GO:00313	K06839|1|0.0|2991|mmu:20563|slit 2	gi|148705684|gb|EDL37631.1|	0	slit homolog 2 (Drosophila), isoform CRA_a [Mus musculus]

mmu-let-7c-1-3p	NM_173001	-24.2	0.992851	-22.19	164	GO:0043231,GO:0046914,GO:0016701,GO:0035258,GO:0048869,GO:0030518,GO:0070076,GO:0048232,GO:0006351,GO:00442	K15601|1|0.0|2693|mmu:104263|lysine-specific demethylase 3 [EC:1.14.11.-]	gi|37360072|dbj|BAC98014.1|	0	mKIAA0742 protein [Mus musculus]
mmu-let-7c-1-3p	NR_031761	-24.6	0.957674	-20.65	163	-	K10990|1|0.0|1165|mmu:74386|RecQ-mediated genome instability protein 1	gi|270265788|ref|NP_083180.3|	0	recQ-mediated genome instability protein 1 [Mus musculus]
mmu-let-7c-1-3p	NM_172992	-26	0.899816	-24.64	162	GO:0043231,GO:0003676,GO:0010467,	-	gi|148671275|gb|EDL03222.1|	0	putative homeodomain transcription factor 2, isoform CRA_a [Mus musculus]
mmu-let-7c-1-3p	NM_133365	-24.4	0.999991	-20.98	161	GO:0030286,GO:0005930,GO:0003774,GO:0032559,GO:0048870,GO:0007017,	K10408|1|0.0|2060|dre:556023|dynein heavy chain, axonemal	gi|148676944|gb|EDL08891.1|	0	dynein, axonemal, heavy chain 5, isoform CRA_a [Mus musculus]

mmu-let-7c-1-3p	NM_178716	-24.6	0.989641	-23.06	160	GO:0043231,GO:0044444,GO:0005048,GO:0022892,GO:0006606,GO:0051704,GO:00160	K14293|1|1e-16|90.5|cqu:CpipJ_CPIJ004613|importin subunit beta-1	gi|115385968|ref|NP_848831.2|	0	transportin-1 isoform 1 [Mus musculus]
mmu-let-7c-1-3p	NM_198683	-26	0.894333	-20.97	159	GO:0032991,GO:0043231,GO:0030371,GO:0003723,GO:0005515,GO:0000245,GO:0035264,GO:0035194,GO:0007281,GO:00072	K13207|1|0.0|722|mmu:13046|CUG-BP- and ETR3-like factor	gi|148695568|gb|EDL27515.1|	0	CUG triplet repeat, RNA binding protein 1, isoform CRA_d [Mus musculus]
mmu-let-7c-1-3p	NM_201239	-24	0.880233	-20.51	158	GO:0016892,GO:0005488,	K01172|1|1e-74|281|mmu:58809|[EC:3.1.27.-]	gi|47679082|ref|NP_067447.2|	8.62E-74	ribonuclease 4 precursor [Mus musculus]
mmu-let-7c-1-3p	NM_175335	-21.7	1	-20.24	157	GO:0005667,GO:0003676,GO:0046983,GO:0008134,GO:0006352,GO:0006354,GO:0006366,	K03122|1|2e-110|402|mmu:83602|transcription initiation factor TFIIA large subunit	gi|148687003|gb|EDL18950.1|	1.56E-109	general transcription factor II A, 1, isoform CRA_a [Mus musculus]

mmu-let-7c-1-3p	NM_153561	-24.6	0.584704	-20.51	156	GO:0043231,GO:0003824,GO:0005102,	-	gi|23956340|ref|NP_705789.1|	0	nucleoside diphosphate-linked moiety X motif 6 [Mus musculus]
mmu-let-7c-1-3p	NM_199038	-20.9	1	-20.18	155	GO:0044464,	-	gi|158711675|ref|NP_950239.2|	0	protein CASC4 isoform 2 [Mus musculus]
mmu-let-7c-1-3p	NR_040627	-22.8	0.968468	-20.1	154	-	-	-	-	-
mmu-let-7c-1-3p	NM_031261	-24.4	0.529374	-21.53	153	GO:0016491,GO:0005506,GO:0008283,GO:0002376,GO:0008152,GO:0000041,GO:00068	K00522|1|3e-51|202|tgu:100190515|ferritin heavy chain [EC:1.16.3.1]	gi|148678224|gb|EDL10171.1|	9.19E-108	mCG65721 [Mus musculus]
mmu-let-7c-1-3p	NM_025892	-20.8	0.999821	-20.36	152	-	-	gi|149060724|gb|EDM11438.1|	2.05E-90	similar to RIKEN cDNA 1500031L02, isoform CRA_b [Rattus norvegicus]
mmu-let-7c-1-3p	NR_045820	-23.3	0.952222	-21.5	151	-	-	gi|148687710|gb|EDL19657.1|	3.76E-65	mCG1030587 [Mus musculus]
mmu-let-7c-1-3p	NM_176920	-24.1	0.99931	-21.76	150	GO:0031224,	K06850|1|2e-18|97.1|ssc:100513444|slit 3	gi|28892715|ref|NP_795894.1|	8.95E-174	leucine-rich repeat and transmembrane domain-containing protein 1 precursor [Mus musculus]

mmu-let-7c-1-3p	NM_172481	-22.8	0.998234	-20.8	149	GO:0005488,GO:0006950,	K12800|1|3e-95|350|ecb:100073322|NACHT, LRR and PYD domains-containing protein 3	gi|27369660|ref|NP_766069.1|	0	NACHT, LRR and PYD domains-containing protein 4B [Mus musculus]

mmu-let-7c-1-3p	NM_008514	-21.4	1	-23.17	148	GO:0031224,GO:0005768,GO:0031982,GO:0044297,GO:0044459,GO:0042802,GO:0005215,GO:0030169,GO:0004857,GO:0004872,GO:0048048,GO:0051480,GO:0060536,GO:0048754,GO:0021536,GO:0061349,GO:0014020,GO:0003143,GO:0001702,GO:0030278,GO:0048771,GO:00517	K03068|1|0.0|3112|mmu:16974|low density lipoprotein receptor-related protein 5/6	gi|148727327|ref|NP_032540.2|	0	low-density lipoprotein receptor-related protein 6 precursor [Mus musculus]
mmu-let-7c-1-3p	NM_001081081	-20.6	1	-21.85	147	GO:0044429,GO:0016811,GO:0050896,GO:0006541,GO:0023061,GO:0001505,	K01425|1|0.0|1211|mmu:14660|glutaminase [EC:3.5.1.2]	gi|124487313|ref|NP_001074550.1|	0	glutaminase kidney isoform, mitochondrial isoform 1 [Mus musculus]

mmu-let-7c-1-3p	NM_175490	-21.1	0.999995	-21.13	144	GO:0016021,GO:0004888,GO:0007166,	K08417|1|0.0|1003|mmu:237716|G protein-coupled receptor 75	gi|47059091|ref|NP_780699.2|	0	probable G-protein coupled receptor 75 [Mus musculus]
mmu-let-7c-1-3p	NM_011811	NA	NA	-20.22	153	GO:0044444,GO:0004812,GO:0046872,GO:0003676,GO:0032559,GO:0006418,	K01890|1|0.0|1142|mmu:23874|phenylalanyl-tRNA synthetase beta chain [EC:6.1.1.20]	gi|31981400|ref|NP_035941.2|	0	phenylalanine--tRNA ligase beta subunit [Mus musculus]
mmu-let-7c-1-3p	NM_016967	NA	NA	-20.95	150	GO:0031981,GO:0016020,GO:0000981,GO:0045596,GO:0021529,GO:0006357,GO:0045165,GO:0045687,GO:0008366,	K09085|1|3e-105|362|pon:100438715|class B basic helix-loop-helix protein 1/6/7	gi|114684349|ref|XP_001172282.1|	2.18E-104	PREDICTED: oligodendrocyte transcription factor 2 isoform 1 [Pan troglodytes]

mmu-let-7c-1-3p	NM_001111075	NA	NA	-22.33	143	GO:0015630,GO:0031981,GO:0044444,GO:0005488,GO:0004721,GO:0006470,GO:0000087,GO:0007127,GO:0033674,GO:0003006,GO:0008283,	K05866|1|0.0|1026|mmu:12531|cell division cycle 25B [EC:3.1.3.48]	gi|161621261|ref|NP_001104545.1|	0	M-phase inducer phosphatase 2 isoform b [Mus musculus]
mmu-let-7c-1-3p	NM_023117	NA	NA	-22.33	143	GO:0015630,GO:0031981,GO:0044444,GO:0005488,GO:0004721,GO:0006470,GO:0000087,GO:0007127,GO:0033674,GO:0003006,GO:0008283,	K05866|1|0.0|1017|mmu:12531|cell division cycle 25B [EC:3.1.3.48]	gi|12963487|ref|NP_075606.1|	0	M-phase inducer phosphatase 2 isoform a [Mus musculus]

mmu-let-7c-1-3p	NM_172498	NA	NA	-24.79	142	GO:0030427,GO:0043005,GO:0044430,GO:0043231,GO:0044444,GO:0005924,GO:0004713,GO:0060089,GO:0032559,GO:0019901,GO:0001894,GO:0003006,GO:0007169,GO:0043406,GO:0016049,GO:0007259,GO:0001503,GO:0048869,GO:0043542,GO:0009605,GO:0048646,GO:00003	K05871|1|0.0|1800|mmu:19229|protein tyrosine kinase 2 beta [EC:2.7.10.2]	gi|148704055|gb|EDL36002.1|	0	PTK2 protein tyrosine kinase 2 beta, isoform CRA_b [Mus musculus]
mmu-let-7c-1-3p	NR_029725	-28.5	0.001619	NA	NA	-	-	-	-	-

mmu-let-7c-1-3p	NM_001111028	-22.2	0.999763	NA	NA	GO:0005887,GO:0005267,GO:0030001,	K15074|1|5e-08|61.2|cfa:489942|BTB/POZ domain-containing adapter for CUL3-mediated RhoA degradation protein	gi|161333815|ref|NP_001104498.1|	0	BTB/POZ domain-containing protein KCTD9 isoform a [Mus musculus]
mmu-let-7c-1-3p	NM_182993	-23	0.995419	NA	NA	GO:0030658,GO:0031224,GO:0008021,GO:0044459,GO:0005436,GO:0015172,GO:0023061,GO:0007613,GO:0030001,GO:0006817,GO:0015813,GO:0051899,GO:0001505,GO:0007270,	K12302|1|0.0|1050|rno:116638|MFS transporter, ACS family, solute carrier family 17 (sodium-dependent inorganic phosphate cotransporter), member 6/7/8	gi|218156282|ref|NP_892038.2|	0	vesicular glutamate transporter 1 [Mus musculus]

mmu-let-7c-1-3p	NM_015771	-20.5	1	NA	NA	GO:0043231,GO:0005819,GO:0043169,GO:0004672,GO:0032559,GO:0035556,GO:0030178,GO:0000087,GO:0000079,	K08791|1|0.0|1999|mmu:50523|LATS, large tumor suppressor [EC:2.7.11.1]	gi|148704219|gb|EDL36166.1|	0	large tumor suppressor 2, isoform CRA_b [Mus musculus]
mmu-let-7c-1-3p	NM_001009949	-20.4	1	NA	NA	GO:0019866,GO:0031224,GO:0043231,GO:0051234,	K15110|1|4e-16|88.6|isc:IscW_ISCW017282|solute carrier family 25 (mitochondrial 2-oxodicarboxylate transporter), member 21	gi|74220685|dbj|BAE33610.1|	0	unnamed protein product [Mus musculus]
mmu-let-7c-1-3p	NM_001199632	-21	0.999991	NA	NA	-	K10279|1|0.0|1555|mmu:320118|F-box and leucine-rich repeat protein	gi|313851009|ref|NP_001186561.1|	0	F-box/LRR-repeat protein 13 isoform a [Mus musculus]

mmu-let-7c-1-3p	NM_011631	-21.8	0.999788	NA	NA	GO:0044432,GO:0048770,GO:0044425,GO:0031090,GO:0003676,GO:0046872,GO:0070325,GO:0032559,GO:0019902,GO:0006810,GO:0043161,GO:0006874,GO:0033198,GO:0010921,GO:0007015,GO:0006950,GO:00	K09487|1|0.0|1283|mmu:22027|heat shock protein 90kDa beta	gi|332241648|ref|XP_003269991.1|	0	PREDICTED: endoplasmin-like [Nomascus leucogenys]
mmu-let-7c-1-3p	NM_001008426	-22.6	0.995811	NA	NA	GO:0016020,	-	gi|56606033|ref|NP_001008426.1|	3.49E-156	uncharacterized protein LOC433365 [Mus musculus]
mmu-let-7c-1-3p	NM_001166581	-20.8	1	NA	NA	-	K12879|1|0.0|2560|aml:100473142|THO complex subunit 2	gi|262205179|ref|NP_001160053.1|	0	cDNA sequence BC005561 [Mus musculus]

mmu-let-7c-1-3p	NM_001163284	-23.4	0.9996	NA	NA	GO:0043231,GO:0046914,GO:0003676,GO:0010467,	K10492|1|0.0|1161|mmu:230119|zinc finger and BTB domain-containing protein 5	gi|37359912|dbj|BAC97934.1|	0	mKIAA0354 protein [Mus musculus]
mmu-let-7c-1-3p	NM_010997	-20.2	0.998542	NA	NA	GO:0004871,GO:0023052,	K04257|1|1e-170|598|mmu:18354|olfactory	gi|22203767|ref|NP_035127.1|	9.68E-170	olfactory receptor 54 [Mus musculus]
mmu-let-7c-1-3p	NM_010626	-23.1	0.99939	NA	NA	GO:0042995,GO:0003774,GO:0032559,GO:0008589,GO:0007017,	K10395|1|0.0|2214|mmu:16576|kinesin family member 4/7/21/27	gi|254553479|ref|NP_034756.2|	0	kinesin-like protein KIF7 [Mus musculus]
mmu-let-7c-1-3p	NR_029732	-21.9	0.083689	NA	NA	-	-	-	-	-
mmu-let-7c-1-3p	NM_172424	-21.6	1	NA	NA	GO:0043234,GO:0043231,GO:0006355,	K15164|1|0.0|4141|mmu:76199|mediator of RNA polymerase II transcription subunit	gi|146134449|ref|NP_766012.3|	0	mediator of RNA polymerase II transcription subunit 13-like [Mus musculus]

mmu-let-7c-1-3p	NM_001164672	-20.8	1	NA	NA	GO:0044424,GO:0043169,GO:0070325,GO:0032559,GO:0044267,GO:0048870,GO:0030518,GO:0048232,GO:0006974,	K09502|1|0.0|724|rno:65028|DnaJ homolog subfamily A member 1	gi|6680297|ref|NP_032324.1|	0	dnaJ homolog subfamily A member 1 [Mus musculus]
mmu-let-7c-1-3p	NM_001114102	-23	0.693976	NA	NA	GO:0042802,GO:0046914,GO:0003723,GO:0016705,GO:0016701,GO:0006400,	-	gi|166235142|ref|NP_001032831.2|	1.10E-80	tRNA wybutosine-synthesizing protein 5 isoform 1 [Mus musculus]
mmu-let-7c-1-3p	NM_019762	-22.5	0.998342	NA	NA	GO:0005913,GO:0043231,GO:0009987,	K12642|1|1e-73|278|xla:443776|plakophilin 2	gi|242332585|ref|NP_062736.2|	0	plakophilin-3 isoform 1 [Mus musculus]
mmu-let-7c-1-3p	NM_001168571	-20.8	1	NA	NA	GO:0043231,GO:0044459,GO:0044444,GO:0046872,GO:0005496,GO:0016879,GO:0032559,GO:0006220,GO:0009064,	K01937|1|0.0|953|mmu:55936|CTP synthase [EC:6.3.4.2]	gi|270483776|ref|NP_001162043.1|	0	CTP synthase 2 isoform b [Mus musculus]

mmu-let-7c-1-3p	NM_024242	-22.3	0.998824	NA	NA	GO:0004672,GO:0032559,GO:0006796,	K07178|1|0.0|1050|mmu:71340|RIO kinase 1 [EC:2.7.11.1]	gi|22208995|ref|NP_077204.2|	0	serine/threonine-protein kinase RIO1 [Mus musculus]
mmu-let-7c-1-3p	NM_001177669	-20.6	0.999999	NA	NA	-	K15620|1|2e-114|414|ssc:100153347|golgi phosphoprotein 3	gi|295054280|ref|NP_001171140.1|	3.57E-151	Golgi phosphoprotein 3-like isoform 2 [Mus musculus]
mmu-let-7c-1-3p	NM_030255	-20.1	1	NA	NA	-	K01500|1|0.0|818|mmu:80287|[EC:3.5.4.-]	gi|148672677|gb|EDL04624.1|	0	apolipoprotein B editing complex 3, isoform CRA_c [Mus musculus]
mmu-let-7c-1-3p	NM_201394	-21.6	1	NA	NA	-	K10388|1|0.0|3881|mmu:18810|plectin	gi|256418964|ref|NP_958788.2|	0	plectin isoform 1e [Mus musculus]
mmu-let-7c-1-3p	NM_023858	-22	0.99978	NA	NA	GO:0031090,GO:0043231,GO:0042802,GO:0004721,GO:0006470,GO:0051259,	K01112|1|0.0|1233|mmu:77116|[EC:3.1.3.-]	gi|194394153|ref|NP_076347.3|	0	myotubularin-related protein 2 [Mus musculus]

mmu-let-7c-1-3p	NM_001171615	-21.8	1	NA	NA	GO:0043231,GO:0046914,GO:0003676,GO:0001071,GO:0006351,GO:0007275,GO:00488	-	gi|284448128|ref|NP_001165086.1|	0	myelin transcription factor 1 isoform 3 [Mus musculus]
mmu-let-7c-1-3p	NM_001040426	-20.5	1	NA	NA	GO:0031012,GO:0004175,	K08630|1|7e-87|324|acs:100566040|a disintegrin and metalloproteinase with thrombospondin motifs 16 [EC:3.4.24.-]	gi|283135196|ref|NP_001035516.2|	0	thrombospondin type-1 domain-containing protein 4 isoform a precursor [Mus musculus]
mmu-let-7c-1-3p	NR_026733	-23.2	0.744671	NA	NA	-	-	gi|148704847|gb|EDL36794.1|	1.03E-50	mCG5478 [Mus musculus]
mmu-let-7c-1-3p	NM_026864	-23.7	0.762474	NA	NA	GO:0031981,GO:0017111,GO:0032561,GO:0035556,GO:0006356,GO:0009207,	K07852|1|5e-129|461|mmu:68895|Ras-like, family 11, member	gi|110625728|ref|NP_081140.1|	3.15E-128	ras-like protein family member 11A [Mus musculus]

mmu-let-7c-1-3p	NM_001252106	-23	0.99963	NA	NA	GO:0044459,GO:0030136,GO:0043169,GO:0016079,GO:0017157,	K15293|1|7e-11|71.2|xtr:100492289|protein unc-13	gi|355390341|ref|NP_001239035.1|	0	calcium-dependent secretion activator 2 isoform 3 [Mus musculus]
mmu-let-7c-1-3p	NM_030138	-22	1	NA	NA	GO:0031252,GO:0005083,GO:0008047,GO:0046914,GO:0043087,GO:0032012,	K12489|1|0.0|1484|mmu:78618|Arf-GAP with coiled-coil, ANK repeat and PH domain-containing protein	gi|37359746|dbj|BAC97851.1|	0	mKIAA0041 protein [Mus musculus]
mmu-let-7c-1-3p	NM_001201470	-20.2	1	NA	NA	GO:0044444,GO:0004779,GO:0016301,GO:0032559,GO:0048731,GO:0006796,GO:0006790,GO:0034036,GO:0007599,	K13811|1|0.0|1236|mmu:23972|3'-phosphoadenosine 5'-phosphosulfate synthase [EC:2.7.7.4 2.7.1.25]	gi|319918850|ref|NP_001188399.1|	0	bifunctional 3'-phosphoadenosine 5'-phosphosulfate synthase 2 isoform 2 [Mus musculus]

mmu-let-7c-1-3p	NM_001168624	-27.5	0.81054	NA	NA	-	K08534|1|7e-13|78.6|ecb:100060134|RAR-related orphan receptor gamma	gi|58037552|ref|NP_941016.2|	0	C2 calcium-dependent domain-containing protein 4C [Mus musculus]
mmu-let-7c-1-3p	NM_021459	-23.9	0.953211	NA	NA	GO:0043231,GO:0046914,GO:0001071,GO:0003677,GO:0007411,GO:0021536,GO:0045596,GO:0031016,GO:0055007,GO:0006357,GO:0001667,GO:0001708,GO:0050796,GO:0009887,GO:0021522,	K09370|1|0.0|725|ssc:100511417|insulin gene enhancer protein ISL	gi|94400881|ref|NP_059035.3|	0	insulin gene enhancer protein ISL-1 [Rattus norvegicus]

mmu-let-7c-1-3p	NM_001048142	-20.5	1	NA	NA	GO:0030136,GO:0005167,GO:0023061,GO:0033555,GO:0045597,GO:0007405,GO:0031175,GO:0007610,GO:0051899,GO:0014070,GO:0008037,GO:0033273,GO:0016202,GO:0006935,GO:0006954,GO:0035295,GO:0009719,GO:0048169,GO:0043066,GO:00	K04355|1|3e-145|517|pon:100447350|brain-derived neurotrophic factor	gi|397174764|ref|NP_001257559.1|	7.86E-161	brain-derived neurotrophic factor isoform 1 [Rattus norvegicus]
mmu-let-7c-1-3p	NR_015580	-21	0.999997	NA	NA	-	-	gi|148703101|gb|EDL35048.1|	3.77E-56	mCG142516, isoform CRA_b [Mus musculus]
mmu-let-7c-1-3p	NM_030110	-23.8	0.983268	NA	NA	GO:0031224,GO:0046872,	-	gi|255003807|ref|NP_084386.1|	0	EF-hand domain-containing family member A2 [Mus musculus]

mmu-let-7c-1-3p	NM_028190	-21.4	1	NA	NA	GO:0043231,GO:0043169,GO:0019904,GO:0016202,	K13212|1|3e-112|408|xtr:548778|RNA-binding protein Luc7-like 2	gi|148690554|gb|EDL22501.1|	8.16E-127	Luc7 homolog (S. cerevisiae)-like, isoform CRA_a [Mus musculus]
mmu-let-7c-1-3p	NM_007594	-21	0.999999	NA	NA	GO:0005788,GO:0048770,GO:0046872,GO:0005515,GO:0001775,GO:0031103,GO:0006887,GO:0007599,GO:0010033,	K12042|1|2e-06|55.8|dre:100002038|troponin C, skeletal muscle	gi|6680840|ref|NP_031620.1|	0	calumenin isoform 1 precursor [Mus musculus]
mmu-let-7c-1-3p	NM_146886	-22.3	0.901748	NA	NA	GO:0004888,GO:0023052,	K04257|1|2e-172|605|mmu:258888|olfactory	gi|22129115|ref|NP_667097.1|	1.34E-171	olfactory receptor 1298 [Mus musculus]

mmu-let-7c-1-3p	NM_008465	-27.5	0.636704	NA	NA	GO:0031981,GO:0044444,GO:0005635,GO:0022892,GO:0005048,GO:0006310,GO:0006915,GO:0051704,GO:0022415,GO:0006606,GO:0000737,	K15042|1|0.0|987|mmu:16646|importin subunit alpha-1	gi|31543047|ref|NP_032491.2|	0	importin subunit alpha-1 [Mus musculus]
mmu-let-7c-2-3p	NM_173785	-27.8	0.517532	-25.48	173	GO:0043231,GO:0008168,GO:0006779,GO:0006730,GO:0006544,	K06980|1|0.0|705|mmu:216792|	gi|148675734|gb|EDL07681.1|	0	RIKEN cDNA A230051G13 [Mus musculus]
mmu-let-7c-2-3p	NR_029725	-29.3	0.000661	-27.99	179	-	-	-	-	-
mmu-let-7c-2-3p	NR_029726	-28.3	0.001341	-27.96	179	-	-	-	-	-
mmu-let-7c-2-3p	NR_029731	-28	0.001359	-28.31	183	-	-	-	-	-
mmu-let-7c-2-3p	NR_029732	-29.9	0.000322	-28.26	183	-	-	-	-	-
mmu-let-7c-2-3p	NM_009103	-26.2	0.76834	-24.19	183	GO:0043234,GO:0031981,GO:0044444,GO:0016728,GO:0032559,GO:0006259,GO:0009165,GO:0051262,	K10807|1|0.0|1528|mmu:20133|ribonucleoside-diphosphate reductase subunit M1 [EC:1.17.4.1]	gi|31982026|ref|NP_033129.2|	0	ribonucleoside-diphosphate reductase large subunit [Mus musculus]

mmu-let-7c-2-3p	NM_019827	-26.6	0.916657	-22.47	180	GO:0030427,GO:0031981,GO:0043234,GO:0044425,GO:0030425,GO:0044444,GO:0044297,GO:0004672,GO:0051018,GO:0008134,GO:0019900,GO:0032559,GO:0008092,GO:0035254,GO:0006461,GO:0001952,GO:0032321,GO:0043405,GO:0048017,GO:0048167,GO:0007169,GO:00488	K03083|1|0.0|788|ecb:100061033|glycogen synthase kinase 3 beta [EC:2.7.11.26]	gi|387766242|pdb|4ACC|A	0	Chain A, Gsk3b In Complex With Inhibitor
mmu-let-7c-2-3p	NM_009767	-25.4	0.975109	-21.06	178	GO:0016020,GO:0031410,	-	gi|291407705|ref|XP_002720161.1|	1.03E-68	PREDICTED: cysteine-rich hydrophobic domain 2-like [Oryctolagus cuniculus]

mmu-let-7c-2-3p	NM_023502	-25.1	0.796618	-21.39	176	GO:0031981,GO:0003677,GO:0001071,GO:0006355,	K09428|1|0.0|995|mmu:69257|E74-like factor 1/2/4	gi|148703252|gb|EDL35199.1|	0	E74-like factor 2, isoform CRA_e [Mus musculus]
mmu-let-7c-2-3p	NM_033604	-25.2	0.936074	-20.16	174	GO:0032991,GO:0044424,GO:0046872,GO:0005515,GO:0019787,GO:0006355,GO:0016567,GO:0007179,GO:0007275,	K13201|1|7e-09|64.7|bfo:BRAFLDRAFT_124262|nucleolysin TIA-1/TIAR!K11980|2|9e-09|64.3|ame:408648|E3 ubiquitin-protein ligase RNF11	gi|15809022|ref|NP_291082.1|	0	E3 ubiquitin-protein ligase Arkadia [Mus musculus]

mmu-let-7c-2-3p	NM_001113204	-22.6	0.999988	-20.69	172	GO:0030427,GO:0043005,GO:0044297,GO:0009986,GO:0031224,GO:0019904,GO:0005539,GO:0019899,GO:0021536,GO:0014073,GO:0016337,GO:0010941,GO:0007610,GO:0051931,GO:0008284,GO:0031103,GO:0009967,GO:0010038,GO:00098	K06491|1|0.0|1561|gga:428253|neural cell adhesion molecule	gi|148693783|gb|EDL25730.1|	0	neural cell adhesion molecule 1, isoform CRA_d [Mus musculus]
mmu-let-7c-2-3p	NR_029730	-24.5	0.014993	-23.33	171	-	-	-	-	-
mmu-let-7c-2-3p	NM_028538	-24.5	0.980085	-23.02	168	-	K09228|1|0.0|1213|mmu:338354|KRAB domain-containing zinc finger protein	gi|223462786|gb|AAI41374.1|	0	1700049G17Rik protein [Mus musculus]

mmu-let-7c-2-3p	NM_001033173	-23.7	0.99994	-22.37	167	GO:0008233,	K11852|1|0.0|1986|mmu:76179|ubiquitin carboxyl-terminal hydrolase 31 [EC:3.1.2.15]	gi|226443061|ref|NP_001028345.1|	0	ubiquitin specific protease 31 [Mus musculus]
mmu-let-7c-2-3p	NM_153165	-22.8	0.995164	-24.29	166	-	K10452|1|1e-120|435|mmu:236904|kelch-like protein	gi|23346619|ref|NP_694805.1|	6.84E-120	kelch-like 15 isoform c [Mus musculus]
mmu-let-7c-2-3p	NM_026647	-23.5	0.999913	-21.28	165	GO:0012505,GO:0031224,GO:0046914,GO:0006807,GO:0032768,	K05766|1|7e-10|68.9|spu:575378|slingshot	gi|148699086|gb|EDL31033.1|	8.86E-160	zinc finger, DHHC domain containing 21 [Mus musculus]
mmu-let-7c-2-3p	NM_025534	-22.2	0.999939	-20.59	164	GO:0005488,	-	gi|61098118|ref|NP_079810.2|	0	coiled-coil domain-containing protein 82 [Mus musculus]
mmu-let-7c-2-3p	NM_182992	-23.4	0.998306	-21.38	163	GO:0030016,GO:0043231,GO:0008092,	K12567|1|9e-58|227|bta:540561|titin [EC:2.7.11.1]	gi|60360554|dbj|BAD90521.1|	0	mKIAA4170 protein [Mus musculus]

mmu-let-7c-2-3p	NM_025812	-24.3	0.951499	-23.44	162	GO:0031981,GO:0003676,GO:0001071,GO:0006351,GO:0006325,	K09272|1|7e-11|70.9|tad:TRIADDRAFT_64155|structure-specific recognition protein 1!K10802|2|9e-11|70.5|dya:Dyak_GE17263|high mobility group	gi|148693919|gb|EDL25866.1|	0	high mobility group 20A, isoform CRA_b [Mus musculus]

mmu-let-7c-2-3p	NM_007540	-20.7	1	-28.95	159	GO:0030136,GO:0005167,GO:0023061,GO:0033555,GO:0045597,GO:0007405,GO:0031175,GO:0007610,GO:0051899,GO:0014070,GO:0008037,GO:0033273,GO:0016202,GO:0006935,GO:0006954,GO:0035295,GO:0009719,GO:0048169,GO:0043066,GO:00	K04355|1|2e-134|481|mmu:12064|brain-derived neurotrophic factor	gi|114326463|ref|NP_031566.4|	2.03E-138	brain-derived neurotrophic factor isoform 1 precursor [Mus musculus]
mmu-let-7c-2-3p	NM_175104	-20.3	1	-21.11	158	-	-	gi|148664700|gb|EDK97116.1|	0	RIKEN cDNA 2810012G03, isoform CRA_b [Mus musculus]
mmu-let-7c-2-3p	NM_001003910	-21	1	-20.83	157	GO:0044424,	-	gi|145966853|ref|NP_001003910.2|	0	tetratricopeptide repeat protein GNN isoform 3 [Mus musculus]

mmu-let-7c-2-3p	NM_022313	-20.7	0.999914	-20.14	155	GO:0019866,GO:0044429,GO:0043021,GO:0032561,GO:0003723,GO:0022618,	-	gi|165377197|ref|NP_071708.2|	0	GTPase Era, mitochondrial [Mus musculus]
mmu-let-7c-2-3p	NM_198416	-21.8	1	-22.28	154	GO:0043231,GO:0046914,GO:0003676,GO:0006351,	-	gi|124053447|ref|NP_940808.2|	0	ZZ-type zinc finger-containing protein 3 [Mus musculus]
mmu-let-7c-2-3p	NM_178736	-23.1	0.998661	-20.9	153	GO:0043232,GO:0008047,GO:0051707,GO:0043087,GO:0002697,GO:0006897,	-	gi|283436077|ref|NP_848851.3|	3.10E-172	ELMO domain-containing protein 2 [Mus musculus]
mmu-let-7c-2-3p	NM_145590	-23.2	0.974681	-22.81	146	GO:0031224,	K14382|1|2e-24|88.2|bta:399680|solute carrier family 5 (sodium/glucose cotransporter), member 2	gi|225579041|ref|NP_663565.2|	0	UPF0420 protein C16orf58 homolog [Mus musculus]
mmu-let-7c-2-3p	NM_181649	NA	NA	-21.2	151	GO:0044464,GO:0005488,	-	gi|194473718|ref|NP_857632.4|	3.31E-133	coiled-coil domain-containing protein 75 [Mus musculus]

mmu-let-7c-2-3p	NM_021452	NA	NA	-20.24	150	GO:0005887,GO:0005488,GO:0005227,GO:0001508,GO:0001775,GO:0050804,GO:0009593,GO:0042310,GO:0007599,GO:0034220,GO:0030001,	K04941|1|8e-114|410|mmu:58802|potassium large conductance calcium-activated channel subfamily M beta member 4	gi|10946834|ref|NP_067427.1|	5.61E-113	calcium-activated potassium channel subunit beta-4 [Mus musculus]
mmu-let-7c-2-3p	NR_073378	NA	NA	-20.7	149	-	-	-	-	-
mmu-let-7c-2-3p	NM_011749	-21.6	1	NA	NA	GO:0031981,GO:0046914,GO:0001071,GO:0043565,GO:0043566,GO:0006952,GO:0006357,GO:0019953,GO:0065003,	K09228|1|1e-27|127|mmu:242747|KRAB domain-containing zinc finger protein	gi|6756049|ref|NP_035879.1|	0	zinc finger protein 148 [Mus musculus]
mmu-let-7c-2-3p	NM_145930	-20.2	1	NA	NA	-	-	gi|52630434|ref|NP_666042.2|	1.80E-149	UPF0600 protein C5orf51 homolog [Mus musculus]

mmu-let-7c-2-3p	NM_172943	-21.1	1	NA	NA	GO:0031224,GO:0043169,GO:0016701,GO:0008152,	K10767|1|0.0|727|mmu:268420|alkylated DNA repair protein alkB homolog 5 [EC:1.14.11.-]	gi|31044423|ref|NP_766531.2|	0	probable alpha-ketoglutarate-dependent dioxygenase ABH5 [Mus musculus]
mmu-let-7c-2-3p	NM_027497	-20.7	0.999999	NA	NA	GO:0043189,GO:0005635,GO:0004468,GO:0006357,GO:0010629,GO:0016573,	K11322|1|0.0|1210|mmu:13831|enhancer of polycomb-like protein	gi|27532963|ref|NP_081773.1|	0	enhancer of polycomb homolog 1 isoform 2 [Mus musculus]
mmu-let-7c-2-3p	NM_145556	-20.1	1	NA	NA	GO:0043231,GO:0003729,GO:0043566,GO:0006396,GO:0032897,GO:0048255,	K03993|1|1e-139|499|mmu:17175|mannan-binding lectin serine protease 2 [EC:3.4.21.104]	gi|148682887|gb|EDL14834.1|	1.28E-167	mCG16669, isoform CRA_e [Mus musculus]
mmu-let-7c-2-3p	NM_026175	-23.6	0.996039	NA	NA	GO:0005681,GO:0031981,GO:0003676,GO:0006376,	K12825|1|0.0|1134|mmu:67465|splicing factor 3A	gi|26324776|dbj|BAC26142.1|	0	unnamed protein product [Mus musculus]

mmu-let-7c-2-3p	NM_008265	-22.6	0.942131	NA	NA	GO:0043231,GO:0001071,GO:0003677,GO:0003002,GO:0048562,GO:0006351,	K09304|1|1e-81|304|mmu:15401|homeobox protein HoxA/B/C/D4	gi|46275810|ref|NP_032291.1|	9.86E-81	homeobox protein Hox-A4 [Mus musculus]
mmu-let-7c-2-3p	NM_178413	-20.7	0.999812	NA	NA	GO:0016838,GO:0048037,GO:0006566,	K06037|1|3e-146|519|dre:641568|[EC:4.2.3.-]	gi|76677922|ref|NP_848500.3|	0	threonine synthase-like 2 [Mus musculus]
mmu-let-7c-2-3p	NM_025730	-23.1	0.999973	NA	NA	GO:0044431,GO:0044455,GO:0030136,GO:0032561,GO:0004712,GO:0008047,GO:0004674,GO:0042802,GO:0032559,GO:0012501,GO:0010259,GO:0006468,GO:0016567,GO:0006950,GO:0000165,	K08844|1|0.0|4778|mmu:66725|leucine-rich repeat kinase 2 [EC:2.7.11.1]	gi|146231954|ref|NP_080006.3|	0	leucine-rich repeat serine/threonine-protein kinase 2 [Mus musculus]

mmu-let-7c-2-3p	NM_030121	-21.5	0.998841	NA	NA	GO:0043231,GO:0007165,	K10330|1|5e-161|568|mmu:78541|ankyrin repeat and SOCS box protein 8	gi|282397100|ref|NP_084397.3|	3.31E-160	ankyrin repeat and SOCS box protein 8 [Mus musculus]
mmu-let-7c-2-3p	NM_023764	-22.7	0.998315	NA	NA	GO:0043235,GO:0044444,GO:0060089,GO:0005102,GO:0019899,GO:0001775,GO:0006952,GO:0007165,GO:0006796,GO:00071	K05402|1|3e-125|451|mmu:54473|toll-interacting protein	gi|13591860|ref|NP_076253.1|	1.86E-124	toll-interacting protein [Mus musculus]
mmu-let-7c-2-3p	NR_045323	-20	1	NA	NA	-	-	-	-	-
mmu-let-7c-2-3p	NM_201367	-21.9	0.999928	NA	NA	GO:0016021,GO:0004888,GO:0007166,GO:0007267,	K08442|1|0.0|988|mmu:381413|G protein-coupled receptor 176	gi|148695958|gb|EDL27905.1|	0	G protein-coupled receptor 176 [Mus musculus]

mmu-let-7c-2-3p	NM_001037926	-22.1	0.998634	NA	NA	-	K09228|1|0.0|1366|mmu:626316|KRAB domain-containing zinc finger protein	gi|123244705|emb|CAM22692.1|	0	novel protein [Mus musculus]
mmu-let-7c-2-3p	NM_134131	-20.4	1	NA	NA	GO:0044424,GO:0004869,GO:0005488,GO:0010466,	-	gi|295444879|ref|NP_598892.2|	5.38E-115	tumor necrosis factor alpha-induced protein 8 isoform 1 [Mus musculus]
mmu-let-7c-2-3p	NR_027930	-20.7	0.980548	NA	NA	-	-	-	-	-
mmu-let-7c-2-3p	NM_010156	-21.1	1	NA	NA	-	-	gi|226958514|ref|NP_034286.2|	0	sterile alpha motif domain-containing protein 9-like [Mus musculus]
mmu-let-7c-2-3p	NM_011050	-24	0.906036	NA	NA	GO:0031981,GO:0044444,GO:0003676,GO:0012501,GO:0051726,GO:0043407,GO:00075	K03260|1|1e-08|62.8|hmg:100206797|translation initiation factor 4G	gi|148669763|gb|EDL01710.1|	0	programmed cell death 4, isoform CRA_a [Mus musculus]
mmu-let-7c-2-3p	NM_001039051	-20.8	1	NA	NA	GO:0030291,GO:0016772,GO:0006469,	-	gi|148673173|gb|EDL05120.1|	5.38E-35	protein kinase inhibitor beta, cAMP dependent, testis specific, isoform CRA_b [Mus musculus]
mmu-let-7c-2-3p	NM_001111118	-20	1	NA	NA	-	-	gi|162135969|ref|NP_001104588.1|	0	RAD51-associated protein 2 [Mus musculus]

mmu-let-7c-2-3p	NM_027288	-20.9	0.999999	NA	NA	GO:0000323,GO:0048029,GO:0043167,GO:0015923,GO:0009057,GO:0043412,GO:00442	K01192|1|0.0|1801|mmu:110173|beta-mannosidase [EC:3.2.1.25]	gi|158533990|ref|NP_081564.3|	0	beta-mannosidase precursor [Mus musculus]
mmu-let-7c-2-3p	NM_001146199	-20.1	1	NA	NA	GO:0043232,GO:0004721,GO:0006470,GO:0006996,	K01104|1|0.0|2277|mmu:24000|protein-tyrosine phosphatase [EC:3.1.3.48]	gi|226246561|ref|NP_036007.2|	0	tyrosine-protein phosphatase non-receptor type 21 [Mus musculus]
mmu-let-7c-2-3p	NM_001160251	-20	1	NA	NA	GO:0043231,GO:0046914,GO:0001071,GO:0043565,GO:0006357,	K09228|1|4e-28|128|bfo:BRAFLDRAFT_71028|KRAB domain-containing zinc finger protein	gi|29244048|ref|NP_808311.1|	0	zinc finger protein 281 [Mus musculus]
mmu-let-7c-2-3p	NM_177117	-24.2	0.98929	NA	NA	GO:0044424,GO:0008283,	-	gi|226371688|ref|NP_796091.2|	0	deleted in lung and esophageal cancer protein 1 homolog [Mus musculus]

mmu-let-7c-2-3p	NM_198122	-22.1	0.999995	NA	NA	GO:0043231,GO:0003676,GO:0010467,GO:0009987,	K07874|1|9e-33|144|ame:409456|Ras-related protein Rab-1A!K11653|2|7e-09|65.1|cin:100186822|AT-rich interactive domain-containing protein 1!K14832|5|7e-08|61.6|spu:580482|ribosome biogenesi	gi|37694070|ref|NP_937755.1|	0	AT-rich interactive domain-containing protein 4B isoform 2 [Mus musculus]
mmu-let-7c-2-3p	NM_021451	-20.1	1	NA	NA	GO:0031966,GO:0005488,GO:0010033,GO:0008285,GO:0006006,GO:0009416,GO:0010212,GO:0001836,GO:0007006,GO:00092	K10131|1|2e-52|208|mmu:58801|phorbol-12-myristate-13-acetate-induced protein 1	gi|10946832|ref|NP_067426.1|	1.10E-51	phorbol-12-myristate-13-acetate-induced protein 1 [Mus musculus]

mmu-let-7c-2-3p	NM_173444	-20.3	1	NA	NA	-	K07295|1|1e-24|118|dpe:Dper_GL19043|nuclear receptor subfamily 2 group A!K11887|2|4e-09|67.0|ame:409686|proteasomal ATPase-associated factor 1	gi|153791557|ref|NP_775620.2|	0	neurobeachin like 1 [Mus musculus]
mmu-let-7c-2-3p	NM_018831	-20.6	1	NA	NA	GO:0009987,	K15340|1|0.0|1985|mmu:55947|DNA cross-link repair 1A protein	gi|347543745|ref|NP_061301.3|	0	DNA cross-link repair 1A protein [Mus musculus]
mmu-let-7c-2-3p	NM_001122851	-20.3	0.997981	NA	NA	GO:0044424,GO:0010720,GO:0007243,GO:0043066,	-	gi|171184430|ref|NP_001116323.1|	1.04E-112	fas apoptotic inhibitory molecule 1 Faim-L [Mus musculus]
mmu-let-7c-2-3p	NM_177740	-24.8	0.912727	NA	NA	GO:0031224,GO:0043231,GO:0014020,GO:0001932,GO:0006935,GO:0030509,	K06847|1|0.0|743|mmu:244058|RGM domain family	gi|148675189|gb|EDL07136.1|	0	RGM domain family, member A, isoform CRA_b [Mus musculus]

mmu-let-7c-2-3p	NM_016671	-22.1	0.998016	NA	NA	GO:0016021,GO:0004896,GO:0042742,GO:0019221,GO:0001819,GO:0045191,GO:0002828,GO:0002824,	K05064|1|2e-25|118|ptr:469348|interleukin 12 receptor beta-2	gi|170932522|ref|NP_057880.2|	0	interleukin-27 receptor subunit alpha precursor [Mus musculus]
mmu-let-7c-2-3p	NM_028794	-21.2	0.99243	NA	NA	GO:0043231,GO:0019144,GO:0017110,GO:0009181,	K13988|1|0.0|643|mmu:74167|ADP-ribose pyrophosphatase [EC:3.6.1.13]	gi|27753998|ref|NP_083070.2|	0	ADP-ribose pyrophosphatase, mitochondrial [Mus musculus]
mmu-let-7c-2-3p	NM_028140	-26.3	0.652241	NA	NA	GO:0005764,GO:0031224,GO:0009987,GO:0006810,	K12307|1|0.0|939|mmu:72175|MFS transporter, ceroid-lipofuscinosis neuronal protein 7	gi|31541926|ref|NP_082416.2|	0	major facilitator superfamily domain-containing protein 8 [Mus musculus]
mmu-let-7c-2-3p	NM_178717	-21.5	0.999995	NA	NA	GO:0031224,GO:0008528,GO:0007166,GO:0007204,	K08397|1|0.0|767|mmu:239336|relaxin family peptide receptor 3	gi|30520143|ref|NP_848832.1|	0	relaxin-3 receptor 1 [Mus musculus]

mmu-let-7c-2-3p	NM_178647	-20.6	1	NA	NA	GO:0043231,GO:0043566,GO:0010467,	-	gi|30520001|ref|NP_848762.1|	7.22E-91	CGG triplet repeat-binding protein 1 [Mus musculus]
mmu-let-7c-2-3p	NM_001102458	-20.9	1	NA	NA	GO:0044444,GO:0008047,GO:0005488,GO:0004857,GO:0019222,GO:0006595,GO:00065	K01581|1|5e-123|444|xla:379859|ornithine decarboxylase [EC:4.1.1.17]	gi|9055298|ref|NP_061215.1|	0	antizyme inhibitor 1 [Mus musculus]
mmu-let-7c-2-3p	NM_172053	-20.2	1	NA	NA	GO:0031012,GO:0046914,GO:0004175,GO:0019538,	K08630|1|0.0|2464|mmu:271127|a disintegrin and metalloproteinase with thrombospondin motifs 16 [EC:3.4.24.-]	gi|50511261|dbj|BAD32616.1|	0	mKIAA2029 protein [Mus musculus]

mmu-let-7c-2-3p	NM_001177626	-23.8	0.985721	NA	NA	GO:0044444,GO:0005088,GO:0017016,GO:0060089,GO:0006917,GO:0019222,GO:0032989,GO:0007169,GO:0009966,GO:0007249,GO:0007266,	K10728|1|6e-32|140|bfo:BRAFLDRAFT_82314|topoisomerase (DNA) II binding protein 1	gi|60360116|dbj|BAD90277.1|	0	mKIAA4037 protein [Mus musculus]
mmu-let-7c-2-3p	NM_010443	-21.2	0.990553	NA	NA	GO:0043231,GO:0031090,GO:0043169,GO:0016705,GO:0006810,GO:0055114,GO:0006787,GO:0006875,GO:0006950,	K00510|1|5e-170|597|mmu:15369|heme oxygenase [EC:1.14.99.3]	gi|209862939|ref|NP_001129538.1|	3.38E-169	heme oxygenase 2 [Mus musculus]

mmu-let-7c-2-3p	NM_172161	-22.9	0.994074	NA	NA	GO:0031090,GO:0044444,GO:0016301,GO:0046983,GO:0042802,GO:0032559,GO:0051091,GO:0002224,GO:0043433,GO:0019221,GO:0006468,GO:0007249,GO:0006915,GO:00	K04731|1|0.0|1142|mmu:108960|interleukin-1 receptor-associated kinase 2 [EC:2.7.11.1]	gi|165905629|ref|NP_751893.3|	0	interleukin-1 receptor-associated kinase-like 2 isoform a [Mus musculus]
mmu-let-7c-2-3p	NM_010893	-20.7	1	NA	NA	GO:0005764,GO:0016020,GO:0031410,GO:0016997,GO:0005488,	K01186|1|0.0|819|mmu:18010|sialidase-1 [EC:3.2.1.18]	gi|254939721|ref|NP_035023.3|	0	sialidase-1 precursor [Mus musculus]
mmu-let-7c-2-3p	NM_011828	-20.1	1	NA	NA	GO:0031224,GO:0043231,GO:0012505,GO:0034483,GO:0005975,GO:0001838,GO:0015012,	K02513|1|0.0|699|mmu:23908|heparan sulfate 2-O-sulfotransferase HS2ST1 [EC:2.8.2.-]	gi|170172560|ref|NP_035958.3|	0	heparan sulfate 2-O-sulfotransferase 1 [Mus musculus]

mmu-let-7c-2-3p	NM_001253746	-24.1	0.849029	NA	NA	GO:0031224,GO:0009986,GO:0004871,GO:0030246,GO:0032770,GO:0002455,GO:0031343,GO:0023052,GO:0010557,	K06468|1|1e-161|570|mmu:14128|Fc receptor, IgE, low affinity II	gi|359279923|ref|NP_001240675.1|	5.22E-168	low affinity immunoglobulin epsilon Fc receptor isoform F [Mus musculus]
mmu-let-7c-2-3p	NR_027798	-21.3	0.989933	NA	NA	-	-	gi|148708535|gb|EDL40482.1|	9.86E-79	hypothetical protein A930010I02 [Mus musculus]
mmu-let-7c-2-3p	NM_001177960	-20.9	0.999998	NA	NA	GO:0031224,GO:0048731,GO:0048869,	-	gi|295789076|ref|NP_001171430.1|	7.66E-154	neuronal membrane glycoprotein M6-b isoform 5 [Mus

mmu-let-7c-2-3p	NM_138661	-20	1	NA	NA	GO:0016021,GO:0046872,GO:0048731,GO:0016337,GO:0048232,GO:0023052,	K04601|1|1e-60|236|cin:100185193|cadherin EGF LAG seven-pass G-type receptor 2 (flamingo)!K04600|5|4e-60|234|acs:100557297|cadherin EGF LAG seven-pass G-type receptor	gi|21426885|ref|NP_619602.1|	0	protocadherin alpha-10 [Mus musculus]
mmu-let-7c-2-3p	NM_175368	-21	0.998479	NA	NA	GO:0031224,	-	gi|148680506|gb|EDL12453.1|	0	RIKEN cDNA 4933402P03 [Mus musculus]
mmu-let-7c-2-3p	NM_133701	-20.8	0.999997	NA	NA	GO:0030532,GO:0005681,GO:0031981,GO:0003712,GO:0035258,GO:0006357,GO:00003	K12855|1|0.0|1739|mmu:68879|pre-mRNA-processing factor 6	gi|21539655|ref|NP_598462.1|	0	pre-mRNA-processing factor 6 [Mus musculus]

mmu-let-7c-2-3p	NM_001081269	-23.3	0.999987	NA	NA	GO:0043232,GO:0046914,GO:0016279,GO:0016571,GO:0040007,GO:0048869,	K11425|1|0.0|2520|pon:100459660|histone-lysine N-methyltransferase NSD3 [EC:2.1.1.43]	gi|124486903|ref|NP_001074738.1|	0	histone-lysine N-methyltransferase NSD3 isoform 2 [Mus musculus]
mmu-let-7c-2-3p	NM_178069	-21	0.999905	NA	NA	GO:0016604,GO:0032561,GO:0003824,GO:0006913,	K14539|1|0.0|1234|mmu:224092|large subunit GTPase 1 [EC:3.6.1.-]	gi|30017373|ref|NP_835170.1|	0	large subunit GTPase 1 homolog [Mus musculus]
mmu-let-7c-2-3p	NM_001033316	-20.9	0.998937	NA	NA	GO:0016021,GO:0005488,GO:0004888,GO:0007166,	K04326|1|2e-160|566|mmu:233080|free fatty acid receptor 3	gi|85701812|ref|NP_001028488.1|	1.50E-159	free fatty acid receptor 3 [Mus musculus]
mmu-let-7c-2-3p	NM_172632	-24.1	0.985674	NA	NA	GO:0004702,GO:0032559,GO:0006464,GO:0023052,	K06855|1|0.0|1143|mmu:225724|mitogen-activated protein kinase 4/6 [EC:2.7.11.24]	gi|89337268|ref|NP_766220.2|	0	mitogen-activated protein kinase 4 [Mus musculus]

mmu-let-7c-2-3p	NM_001083806	-25.9	0.938616	NA	NA	GO:0030427,GO:0008328,GO:0044430,GO:0030425,GO:0030139,GO:0043025,GO:0031090,GO:0008021,GO:0033267,GO:0005231,GO:0004970,GO:0019904,GO:0016247,GO:0019900,GO:0001881,GO:0006898,GO:0010038,GO:0050804,GO:0006811,GO:00	K05198|1|0.0|1700|mdo:100023645|glutamate receptor 2	gi|332217591|ref|XP_003257942.1|	0	PREDICTED: glutamate receptor 2 isoform 3 [Nomascus leucogenys]
mmu-let-7c-2-3p	NM_001039231	-20.1	1	NA	NA	-	K09228|1|0.0|1009|mmu:626391|KRAB domain-containing zinc finger protein	gi|162287421|ref|NP_001034320.3|	0	kruppel-related zinc finger protein-like [Mus musculus]

mmu-let-7c-2-3p	NM_181470	-24.5	0.74281	NA	NA	-	K14798|1| 0.0|854|m<br>mu:35325<br>8|protein<br>LTV1	gi|313245<br>39|ref|NP_<br>852135.1|	0	protein LTV1<br>homolog [Mus<br>musculus]
mmu-let-7c-2-3p	NM_001122989	-21.1	1	NA	NA	GO:00312<br>24,GO:00<br>31981,GO<br>:0005488,<br>GO:00047<br>21,GO:00<br>06470,GO<br>:0006259,<br>GO:00514<br>43,GO:00<br>00077,	K06639|1| 0.0|934|m<br>mu:21829<br>4|cell<br>division<br>cycle 14<br>[EC:3.1.3.4<br>8]	gi|172072<br>673|ref|N<br>P_001116<br>461.1|	0	dual specificity<br>protein<br>phosphatase<br>CDC14B isoform 2<br>[Mus musculus]
mmu-let-7c-2-3p	NM_177393	-21.5	1	NA	NA	GO:00312<br>24,GO:00<br>05261,GO<br>:0030001,	K05668|1| 0.0|1398|a<br>me:41394<br>7|ATP-<br>binding<br>cassette,<br>subfamily<br>C<br>(CFTR/MR<br>P),<br>member<br>5!K04849| 3|3e-<br>60|236|cin<br>:1001843<br>82|voltag<br>e-<br>dependen<br>t calcium<br>channel N<br>type<br>alpha-1B	gi|123173<br>782|ref|N<br>P_796367.<br>3|	0	sodium leak<br>channel non-<br>selective protein<br>[Mus musculus]

mmu-let-7c-2-3p	NM_008857	-26.8	0.715813	NA	NA	GO:0030427,GO:0043231,GO:0043296,GO:0044444,GO:0046914,GO:0004674,GO:0032559,GO:0008289,GO:0032868,GO:0007043,GO:0006900,GO:0006605,GO:0006796,GO:0007169,GO:0030036,GO:0034097,GO:0035088,GO:0048522,GO:0006464,GO:0010828,GO:0043066,GO:00017	K06069|1|0.0|1209|mmu:18759|atypical protein kinase C [EC:2.7.11.13]	gi|60360172|dbj|BAD90305.1|	0	mKIAA4165 protein [Mus musculus]
mmu-let-7c-2-3p	NM_011877	-20.1	1	NA	NA	GO:0043232,GO:0004721,GO:0006470,GO:0006996,	K01104|1|0.0|2277|mmu:24000|protein-tyrosine phosphatase [EC:3.1.3.48]	gi|226246561|ref|NP_036007.2|	0	tyrosine-protein phosphatase non-receptor type 21 [Mus musculus]

mmu-let-7c-2-3p	NM_001195003	-21.3	1	NA	NA	GO:0043234,GO:0031981,GO:0004468,GO:0046914,GO:0001071,GO:0043967,GO:0006259,GO:0006351,	K11307|1|0.0|1191|ptr:455108|histone acetyltransferase MYST2 [EC:2.3.1.48]	gi|303521965|ref|NP_001181932.1|	0	histone acetyltransferase KAT7 isoform 1 [Mus musculus]
mmu-let-7c-2-3p	NM_173402	-21.1	1	NA	NA	GO:0000228,GO:0016020,GO:0008047,GO:0004871,GO:0060589,GO:0007186,	K13765|1|1e-17|94.0|bta:281453|regulator of G-protein signaling 9	gi|148705526|gb|EDL37473.1|	0	regulator of G-protein signaling 12, isoform CRA_b [Mus musculus]
mmu-let-7c-2-3p	NM_172875	-22.6	0.972449	NA	NA	GO:0043231,GO:0044444,GO:0008047,GO:0005488,GO:0016831,GO:0006595,GO:0006520,GO:0048232,	K01583|1|0.0|780|rno:366473|arginine decarboxylase [EC:4.1.1.19]	gi|62079257|ref|NP_001014283.1|	0	uncharacterized protein LOC366473 [Rattus norvegicus]

mmu-let-7c-2-3p	NM_001033794	-20.7	0.999146	NA	NA	-	K10267|1|0.0|693|mmu:434440|F-box and WD-40 domain protein 12/13/14/15/16/17/19	gi|85702119|ref|NP_001028966.1|	0	F-box and WD-40 domain protein 18 [Mus musculus]
mmu-let-7c-2-3p	NM_178748	-21.8	0.999992	NA	NA	GO:0044459,GO:0005578,GO:0030247,GO:0043062,GO:0010810,GO:0018149,	K06254|1|3e-94|348|bta:525795|agrin!K06255|4|3e-88|328|dre:565429|heparan sulfate proteoglycan 2 (perlecan)	gi|148671398|gb|EDL03345.1|	0	expressed sequence AU040377, isoform CRA_a [Mus musculus]
mmu-let-7c-2-3p	NM_008176	-20.2	0.996872	NA	NA	GO:0044421,GO:0005125,GO:0030595,GO:0006952,GO:0009607,	K05505|1|8e-36|151|mmu:14825|C-X-C motif chemokine 1/2/3	gi|54887349|gb|AAH37997.1|	1.41E-38	Cxcl1 protein, partial [Mus musculus]
mmu-let-7c-2-3p	NM_008338	-21.5	0.998467	NA	NA	GO:0043231,GO:0016021,GO:0004904,GO:0051707,GO:0001959,	K05133|1|0.0|642|mmu:15980|interferon gamma receptor 2	gi|6680373|ref|NP_032364.1|	0	interferon gamma receptor 2 precursor [Mus musculus]

mmu-let-7c-2-3p	NM_207221	-20.5	1	NA	NA	GO:0031981,GO:0043169,GO:0032452,GO:0016701,GO:0008134,GO:0070076,GO:0007599,GO:0006351,	K11449|1|0.0|4581|mmu:108829|jumonji domain-containing protein 1C [EC:1.14.11.-]	gi|226531205|ref|NP_997104.2|	0	probable JmjC domain-containing histone demethylation protein 2C isoform 1 [Mus musculus]
mmu-let-7c-2-3p	NM_028720	-22.5	0.998296	NA	NA	GO:0043231,GO:0003676,GO:0042393,GO:0016616,GO:0048037,GO:0006007,	K00020|1|0.0|1071|mcc:706933|3-hydroxyisobutyrate dehydrogenase [EC:1.1.1.31]	gi|119605657|gb|EAW85251.1|	0	cytokine-like nuclear factor n-pac, isoform CRA_a [Homo sapiens]
mmu-let-7c-2-3p	NM_177342	-20	1	NA	NA	GO:0005856,GO:0005667,GO:0004468,GO:0001071,GO:0043565,GO:0005515,GO:0051704,GO:0006352,GO:0006325,GO:00063	K03130|1|0.0|1285|mmu:226182|transcription initiation factor TFIID subunit 5	gi|148710078|gb|EDL42024.1|	0	TAF5 RNA polymerase II, TATA box binding protein (TBP)-associated factor [Mus musculus]

mmu-let-7c-2-3p	NM_008792	-21.6	0.999997	NA	NA	GO:0044421,GO:0016023,GO:0004175,GO:0005515,GO:0048731,GO:0016486,	K01360|1|0.0|1289|mmu:18549|proprotein convertase subtilisin/kexin type 2 [EC:3.4.21.94]	gi|74179798|dbj|BAE36477.1|	0	unnamed protein product [Mus musculus]
mmu-let-7c-2-3p	NM_028385	-20.9	1	NA	NA	-	K09189|1|9e-95|350|aml:100468393|histone-lysine N-methyltransferase MLL5 [EC:2.1.1.4	gi|28972834|dbj|BAC65833.1|	0	mKIAA1757 protein [Mus musculus]
mmu-let-7c-5p	NM_001007589	-31.8	0.056896	-28.47	163	GO:0043231,GO:0043234,GO:0006357,GO:0001819,GO:0002237,GO:0007275,GO:00082	-	gi|86604728|ref|NP_001007590.2|	2.67E-69	akirin-2 [Mus musculus]
mmu-let-7c-5p	NM_001013256	-25.1	0.954611	-41.81	328	GO:0000783,GO:0043566,GO:0006259,	-	gi|219689064|ref|NP_001013274.2|	0	CST complex subunit CTC1 isoform a [Mus musculus]

mmu-let-7c-5p	NM_001013774	-33.8	0.033517	-50.82	318	GO:0005634,GO:0006886,	K15043|1|2e-132|474|xla:398027|importin subunit alpha-2	gi|144446032|ref|NP_001013796.2|	0	importin subunit alpha-8 [Mus musculus]
mmu-let-7c-5p	NM_001014974	-25.1	0.959264	-41.1	306	GO:0005815,GO:0042995,GO:0016881,GO:0008092,GO:0032559,GO:0018200,	K06228|1|5e-52|207|ssc:100622459|fused [EC:2.7.11.1]!K00273|2|3e-51|205|ame:551507|D-amino-acid oxidase [EC:1.4.3.3]!K06047|3|2e-16|89.4|bta:514559|tubulin---tyrosine ligase [EC:6.3.2.2	gi|62510079|ref|NP_001014974.1|	0	tubulin polyglutamylase TTLL4 [Mus musculus]
mmu-let-7c-5p	NM_001024927	-27.2	0.864628	-25	160	GO:0031224,GO:0005548,GO:0046872,GO:0005102,GO:0050953,GO:0006650,	K13619|1|6e-09|65.5|ame:724115|phospholipase DDHD1 [EC:3.1.1.-]	gi|67972419|ref|NP_001020098.1|	0	membrane-associated phosphatidylinositol transfer protein 3 isoform 1 [Mus musculus]

mmu-let-7c-5p	NM_001033343	-25.7	0.895177	-29.4	162	GO:0030134,GO:0031090,GO:0006810,	K14005|1|0.0|2187|mmu:240667|protein transport protein SEC31	gi|133504851|ref|NP_001028515.1|	0	protein transport protein Sec31B [Mus musculus]
mmu-let-7c-5p	NM_001037842	-27.4	0.378132	-25.99	160	GO:0019866,GO:0016740,	-	gi|83745106|ref|NP_001032931.1|	2.86E-85	probable N-acetyltransferase CML3 isoform 1 [Mus musculus]
mmu-let-7c-5p	NM_001038492	-26.6	0.750697	-42.19	310	GO:0000323,GO:0031981,GO:0005488,GO:0004180,GO:0030234,GO:0019538,GO:0015031,	K13289|1|0.0|979|mmu:19025|cathepsin A (carboxypeptidase C) [EC:3.4.16.5]	gi|84042525|ref|NP_032932.2|	0	lysosomal protective protein isoform a precursor [Mus musculus]
mmu-let-7c-5p	NM_001040130	-26.9	0.191582	-25.88	158	GO:0016020,	-	gi|158517816|ref|NP_001035219.2|	7.99E-47	transmembrane protein 141 isoform 1 [Mus musculus]
mmu-let-7c-5p	NM_001081641	-27.2	0.862805	-25	160	GO:0031224,GO:0005548,GO:0046872,GO:0005102,GO:0050953,GO:0006650,	K13619|1|6e-09|65.5|ame:724115|phospholipase DDHD1 [EC:3.1.1.-]	gi|126032319|ref|NP_001075110.1|	0	membrane-associated phosphatidylinositol transfer protein 3 isoform 2 [Mus musculus]

mmu-let-7c-5p	NM_001083895	-30.9	0.117846	-26.33	158	GO:0044424,GO:0005515,GO:0002042,GO:0007155,GO:0016310,	K14407|1|0.0|669|oaa:100082393|cleavage stimulation factor subunit 2!K06496|2|4e-22|107|gga:424364|selectin, platele	gi|123226189|emb|CAM17838.1|	0	sushi-repeat-containing protein, X-linked 2 [Mus musculus]
mmu-let-7c-5p	NM_001109993	-26.9	0.265307	-25.88	158	GO:0016020,	-	gi|158517816|ref|NP_001035219.2|	3.45E-36	transmembrane protein 141 isoform 1 [Mus musculus]

mmu-let-7c-5p	NM_001110140	-29.9	0.359625	-45.35	311	GO:0032991,GO:0016021,GO:0005789,GO:0001664,GO:0019829,GO:0046872,GO:0032559,GO:0009888,GO:0007599,GO:0008016,GO:0006816,GO:0006936,GO:0009207,GO:0007165,GO:0001775,GO:0009206,GO:0009725,GO:0006875,	K05853|1|0.0|2035|mmu:11938|Ca2+ transporting ATPase, sarcoplasmic/endoplasmic reticulum [EC:3.6.3.8]	gi|60360584|dbj|BAD90532.1|	0	mKIAA4195 protein [Mus musculus]
mmu-let-7c-5p	NM_001113360	-27	0.811759	-46.48	300	GO:0016020,GO:0060089,GO:0046872,GO:0004629,GO:0007165,GO:0006650,	K15370|1|6e-104|380|nve:NEMVE_v1g89019|inactive phospholipase C-like	gi|164565357|ref|NP_001106831.1|	0	1-phosphatidylinositol 4,5-bisphosphate phosphodiesterase eta-2 isoform b [Mus musculus]

mmu-let-7c-5p	NM_001142731	-28.2	0.492912	-25.46	160	-	K15074|1|8e-10|67.4|cbr:CBG18386|BTB/POZ domain-containing adapter for CUL3-mediated RhoA degradation protein	gi|218505696|ref|NP_001136203.1|	0	BTB/POZ domain-containing protein KCTD1 isoform 1 [Mus musculus]
mmu-let-7c-5p	NM_001143790	-25.1	0.954517	-41.81	328	GO:0000783,GO:0043566,GO:0006259,	-	gi|219689066|ref|NP_001137262.1|	0	CST complex subunit CTC1 isoform c [Mus musculus]
mmu-let-7c-5p	NM_001145970	-28.7	0.39365	-25.48	156	GO:0043232,	K10433|1|1e-18|96.7|acs:100551816|microtubule-associated protein 7	gi|148698350|gb|EDL30297.1|	0	arginine/proline rich coiled-coil 1, isoform CRA_c [Mus musculus]
mmu-let-7c-5p	NM_001160012	-29.6	0.164491	-25.6	168	GO:0005921,GO:0031224,GO:0005488,GO:0022829,GO:0043009,GO:0008544,GO:0050954,GO:0009987,	K07622|1|4e-136|485|mmu:14620|gap junction protein, beta 3	gi|6680013|ref|NP_032152.1|	3.12E-135	gap junction beta-3 protein [Mus musculus]

mmu-let-7c-5p	NM_001161433	-28.3	0.318117	-25.88	162	GO:0016021,GO:0005035,GO:0005488,GO:0043410,GO:0048869,GO:0009888,GO:0051091,GO:0008219,GO:0019221,GO:00072	K05163|1|5e-165|582|mmu:245527|tumor necrosis factor receptor superfamily member 27	gi|148682260|gb|EDL14207.1|	1.81E-166	ectodysplasin A2 isoform receptor, isoform CRA_b [Mus musculus]
mmu-let-7c-5p	NM_001164679	-26.4	0.80019	-48.12	319	GO:0016021,GO:0005216,	K13982|1|9e-81|303|dgr:Dgri_GH10918|probable ATP-dependent RNA helicase DDX4 [EC:3.6.4.13]!K03018|3|2e-19|99.4|smm:Smp_002890|DNA-directed RNA polymerase III subunit RPC1 [EC:2.7.7.6]	gi|50511029|dbj|BAD32500.1|	0	mKIAA1623 protein [Mus musculus]

mmu-let-7c-5p	NM_001170746	-25	0.992764	-49.05	321	GO:0044459,GO:0032991,GO:0019904,GO:0019899,GO:0007172,GO:0051259,	K05629|1|0.0|2370|rno:113970|atrophin-1 interacting protein 1	gi|282721034|ref|NP_001164217.1|	0	membrane-associated guanylate kinase, WW and PDZ domain-containing protein 2 isoform 1 [Mus musculus]
mmu-let-7c-5p	NM_001205068	-26.4	0.84692	-46.83	316	-	K11323|1|1e-17|94.0|dwi:Dwil_GK14253|histone arginine demethylase JMJD6 [EC:1.14.1	gi|326633177|ref|NP_001191997.1|	0	jmjC domain-containing protein 4 isoform 2 [Mus musculus]
mmu-let-7c-5p	NM_001214911	-25.6	0.919251	-65.62	455	-	K13254|1|1e-25|120|xtr:549207|spastin [EC:3.6.4.3]	gi|148672121|gb|EDL04068.1|	6.82E-147	mCG52237 [Mus musculus]
mmu-let-7c-5p	NM_001253905	-25.6	0.98941	-44.63	314	-	K06065|1|0.0|3390|mmu:20602|nuclear receptor co-repressor 2	gi|359806952|ref|NP_001240834.1|	0	nuclear receptor corepressor 2 isoform 3 [Mus musculus]

mmu-let-7c-5p	NM_007419	-25	0.857852	-43.98	306	GO:0043231,GO:0016021,GO:0031690,GO:0043176,GO:0046983,GO:0004939,GO:0002027,GO:0050804,GO:0033555,GO:0010613,GO:0001659,GO:0032414,GO:0046541,GO:0045762,GO:0002021,GO:0005977,GO:0006940,GO:0007611,GO:0007600,GO:0045444,GO:0007265,GO:00019	K04141|1|0.0|660|mmu:11554|adrenergic receptor beta-1	gi|158517827|ref|NP_031445.2|		0	beta-1 adrenergic receptor [Mus musculus]

mmu-let-7c-5p	NM_007719	-27.2	0.431884	-48.21	318	GO:0009986,GO:0016021,GO:0004950,GO:0006952,GO:0051480,GO:0002376,GO:0007166,GO:0042221,	K04182|1|0.0|716|mmu:12775|C-C chemokine receptor type 7	gi|116268121|ref|NP_031745.2|	0	C-C chemokine receptor type 7 precursor [Mus musculus]
mmu-let-7c-5p	NM_007854	-25.4	0.804648	-25.81	158	GO:0016021,GO:0031981,GO:0044459,GO:0015932,GO:0015855,GO:0006863,GO:0015864,GO:0007589,GO:0032868,GO:0006139,	K15014|1|0.0|668|mmu:13340|solute carrier family 29 (equilibrative nucleoside transporter), member 1/2/3	gi|194248086|ref|NP_031880.2|	0	equilibrative nucleoside transporter 2 [Mus musculus]
mmu-let-7c-5p	NM_008126	-29.6	0.135448	-25.6	168	GO:0005921,GO:0031224,GO:0005488,GO:0022829,GO:0043009,GO:0008544,GO:0050954,GO:0009987,	K07622|1|4e-136|485|mmu:14620|gap junction protein, beta 3	gi|6680013|ref|NP_032152.1|	2.54E-135	gap junction beta-3 protein [Mus musculus]

mmu-let-7c-5p	NM_008313	-25.5	0.944228	-28.82	167	GO:0043231,GO:0016021,GO:0008227,GO:0007188,GO:0008283,GO:0050877,	K04160|1|0.0|797|mmu:15562|5-hydroxytryptamine receptor 4	gi|84370240|ref|NP_032339.2|	0	5-hydroxytryptamine receptor 4 [Mus musculus]
mmu-let-7c-5p	NM_008355	-29.2	0.114658	-26.05	166	GO:0044424,GO:0044421,GO:0005126,GO:0015992,GO:0010524,GO:0009612,GO:0001818,GO:0030888,GO:0023052,GO:0002377,GO:0007154,GO:0008284,GO:0002696,GO:0014070,GO:0002237,GO:00069	K05435|1|3e-56|219|mmu:16163|interleukin 13	gi|6680403|ref|NP_032381.1|	1.79E-55	interleukin-13 precursor [Mus musculus]

mmu-let-7c-5p	NM_008767	-27.2	0.339482	-25.02	159	GO:0016021,GO:0044459,GO:0012505,GO:0044424,GO:0015238,GO:0008509,GO:0019899,GO:0015291,GO:0015893,GO:0003008,GO:0006812,	K08214|1|2e-168|592|mmu:18400|MFS transporter, OCT family, solute carrier family 22 (organic cation transporter), member 18	gi|112363086|ref|NP_001036225.1|	1.38E-167	solute carrier family 22 member 18 [Mus musculus]
mmu-let-7c-5p	NM_008824	-43.2	0.00031	-40.73	192	GO:0043234,GO:0044444,GO:0070061,GO:0008443,GO:0019899,GO:0019203,GO:0032559,GO:0006007,GO:0035556,GO:0033131,GO:0009887,GO:0043434,GO:0031960,GO:0015980,GO:0006000,GO:00069	K01103|1|0.0|910|mmu:18639|6-phosphofructo-2-kinase / fructose-2,6-bisphosphatase [EC:2.7.1.105 3.1.3.46]	gi|7434494|pir||S77704	0	6-phosphofructo-2-kinase (EC 2.7.1.105) / fructose-2, 6-bisphosphate 2-phosphatase (EC 3.1.3.46) clone 5c, skeletal muscle - rat

mmu-let-7c-5p	NM_008906	-26.6	0.762933	-42.19	310	GO:0000323,GO:0031981,GO:0005488,GO:0004180,GO:0030234,GO:0019538,GO:0015031,	K13289|1|0.0|979|mmu:19025|cathepsin A (carboxypeptidase C) [EC:3.4.16.5]	gi|84042525|ref|NP_032932.2|	0	lysosomal protective protein isoform a precursor [Mus musculus]
mmu-let-7c-5p	NM_009191	-30.3	0.309304	-26.29	163	GO:0043231,GO:0016462,GO:0032559,GO:0009408,	K03695|1|0.0|1324|mmu:20480|ATP-dependent Clp protease ATP-binding subunit ClpB	gi|6677983|ref|NP_033217.1|	0	caseinolytic peptidase B protein homolog [Mus musculus]
mmu-let-7c-5p	NM_009288	-25.6	0.950238	-28.51	170	GO:0004672,GO:0032559,GO:0006464,	K08837|1|0.0|1758|mmu:20868|serine/threonine kinase 10 [EC:2.7.11.1]	gi|60360472|dbj|BAD90480.1|	0	mKIAA4026 protein [Mus musculus]
mmu-let-7c-5p	NM_009942	-31.3	0.014075	-27.18	184	GO:0019866,GO:0043231,GO:0043169,GO:0015002,GO:0009725,	K02265|1|3e-72|271|mmu:12859|cytochrome c oxidase subunit 5b	gi|148705042|gb|EDL36989.1|	2.14E-80	mCG18900 [Mus musculus]

mmu-let-7c-5p	NM_010269	-28.3	0.411469	-48.47	320	GO:0033189,	K15212|1|2e-21|105|bfo:BRAFLDRAFT_123909|TATA box-binding protein-associated factor RNA polymerase I subunit A!K15260|2|5e-12|74.3|xla:431935|poly [ADP-ribose] polymerase 9 [EC:2.4.2.30]!K15261|4|3e-11|71.6|dre:562648|	gi|22094097|ref|NP_034399.1|	0	ganglioside-induced differentiation-associated protein 2 [Mus musculus]

mmu-let-7c-5p	NM_010577	-30.2	0.31404	-26.24	162	GO:0044424,GO:0009986,GO:0001726,GO:0005924,GO:0043234,GO:0044421,GO:0005887,GO:0004871,GO:0005172,GO:0001934,GO:0007166,GO:0007599,GO:0007611,GO:0016337,GO:0010810,GO:0034329,GO:0044319,GO:0009967,GO:0006935,GO:0002376,GO:0051704,GO:00486	K06484|1|0.0|1961|mmu:16402|integrin alpha 5	gi|148671975|gb|EDL03922.1|	0	integrin alpha 5 (fibronectin receptor alpha), isoform CRA_b [Mus musculus]

mmu-let-7c-5p	NM_011015	-28.8	0.363797	-25.06	161	GO:0044444,GO:0000808,GO:0016020,GO:0031981,GO:0003676,GO:0016462,GO:0032559,GO:0006259,	K02603|1|0.0|1562|mmu:18392|origin recognition complex subunit 1	gi|158517935|ref|NP_035145.2|	0	origin recognition complex subunit 1 isoform A [Mus musculus]
mmu-let-7c-5p	NM_011150	-29.7	0.184977	-48.48	323	-	K13912|1|3e-29|131|ssc:751862|deleted in malignant brain tumors 1 protein	gi|6755144|ref|NP_035280.1|	0	galectin-3-binding protein precursor [Mus musculus]
mmu-let-7c-5p	NM_011424	-25.6	0.991997	-44.63	314	GO:0043234,GO:0044451,GO:0003712,GO:0019899,GO:0005102,GO:0006629,GO:00001	K06065|1|0.0|3771|mmu:20602|nuclear receptor co-repressor 2	gi|148687620|gb|EDL19567.1|	0	nuclear receptor co-repressor 2, isoform CRA_c [Mus musculus]

mmu-let-7c-5p	NM_011532	-32.6	0.0413	-30.04	169	GO:0043231,GO:0001071,GO:0042802,GO:0003677,GO:0003007,GO:0051705,GO:0048771,GO:0048729,GO:0006357,GO:0001667,GO:0048534,GO:0045165,GO:0048732,GO:0009855,GO:0003002,GO:0050954,GO:0042471,GO:0035239,GO:0048514,GO:0035270,GO:0048646,GO:00487	K10175|1|1e-168|593|mmu:21380|T-box protein 1	gi|148665113|gb|EDK97529.1|	7.57E-168	T-box 1 [Mus musculus]

mmu-let-7c-5p	NM_011682	-27.2	0.968269	-27.99	163	GO:0031981,GO:0044459,GO:0043232,GO:0008092,GO:0046914,GO:0003012,GO:0048513,GO:0001952,	K10366|1|0.0|1570|gga:396236|dystrophin	gi|110431378|ref|NP_035812.3|	0	utrophin [Mus musculus]
mmu-let-7c-5p	NM_011731	-31.6	0.137299	-28.09	160	GO:0044459,GO:0016021,GO:0015291,GO:0005326,GO:0005275,GO:0015804,GO:0035725,	K05048|1|0.0|1254|mmu:22599|solute carrier family 6 (neurotransmitter transporter, amino acid/orphan) member 15/16/17/18/20	gi|31981421|ref|NP_035861.2|	0	sodium- and chloride-dependent transporter XTRP3B [Mus musculus]
mmu-let-7c-5p	NM_011779	-28.8	0.406105	-26.94	170	GO:0005856,GO:0003779,GO:0030866,GO:0023052,GO:0006897,	K13887|1|0.0|968|mmu:23790|coronin-1C	gi|31542413|ref|NP_035909.2|	0	coronin-1C [Mus musculus]

mmu-let-7c-5p	NM_012031	-29.6	0.294483	-41.85	310	GO:0044424,GO:0032561,GO:0003824,GO:0009566,	K09553|1|2e-28|129|hmg:100203295|stress-induced-phosphoprotein 1	gi|148676879|gb|EDL08826.1|	0	sperm associated antigen 1, isoform CRA_b [Mus musculus]
mmu-let-7c-5p	NM_013581	-31.4	0.137182	-28.5	171	GO:0012505,GO:0043234,GO:0043231,GO:0005488,GO:0048193,GO:0006996,	-	gi|159110754|ref|NP_038609.3|	0	conserved oligomeric Golgi complex subunit 1 [Mus musculus]
mmu-let-7c-5p	NM_017395	-27.2	0.719841	-42.01	306	GO:0003676,GO:0006357,	K08061|1|0.0|759|mmu:53970|regulatory factor X	gi|7716046|gb|AAF68260.1|	0	regulator factor X 5 [Mus musculus]
mmu-let-7c-5p	NM_019674	-27.6	0.253304	-43.38	318	GO:0005815,GO:0043231,GO:0008287,GO:0043169,GO:0004721,GO:0004702,GO:0048646,GO:0006796,GO:0007010,GO:00064	K15423|1|0.0|636|mdo:100012739|serine/threonine-protein phosphatase 4 catalytic subunit [EC:3.1.3.16]	gi|4506027|ref|NP_002711.1|	0	serine/threonine-protein phosphatase 4 catalytic subunit [Homo sapiens]

mmu-let-7c-5p	NM_021362	-25.5	0.997697	-70.5	478	GO:0044424,GO:0044421,GO:0046914,GO:0004175,GO:0048869,GO:0022414,	K07762|1|0.0|3240|mmu:18491|pappalysin-1 [EC:3.4.24.79]	gi|20148857|gb|AAM12687.1|AF439513_1	0	pregnancy-associated plasma protein-A, partial [Mus musculus]
mmu-let-7c-5p	NM_021390	-29.6	0.445633	-93.07	633	-	K09228|1|2e-24|116|ssc:100156843|KRAB domain-containing zinc finger protein	gi|159032064|ref|NP_067365.2|	0	sal-like protein 1 [Mus musculus]
mmu-let-7c-5p	NM_021441	-26.2	0.779728	-25.42	151	GO:0005667,GO:0003676,GO:0006351,	K15214|1|0.0|1535|mmu:21341|TATA box-binding protein-associated factor RNA polymerase I subunit C	gi|133778985|ref|NP_067416.2|	0	TATA box-binding protein-associated factor RNA polymerase I subunit C [Mus musculus]

mmu-let-7c-5p	NM_021463	-30.6	0.115634	-27.37	167	GO:0032991,GO:0044444,GO:0016778,GO:0046872,GO:0042802,GO:0032561,GO:0030246,GO:0032559,GO:0006220,GO:0006796,GO:0009887,GO:0046390,GO:0009168,GO:00	K00948|1|4e-179|629|ssc:100517744|ribose-phosphate pyrophosphokinase [EC:2.7.6.1]	gi|118137847|pdb|2H06|A	2.45E-178	Chain A, Crystal Structure Of Human Phosphoribosyl Pyrophosphate Synthetase 1
mmu-let-7c-5p	NM_022031	-26.2	0.605353	-43.69	311	GO:0031012,GO:0005539,GO:0009987,	K06851|1|0.0|690|mmu:73940|hyaluronan and proteoglycan link protein 2	gi|15042971|ref|NP_071314.1|	0	hyaluronan and proteoglycan link protein 2 precursor [Mus musculus]
mmu-let-7c-5p	NM_025304	-28.7	0.165067	-25.45	164	GO:0051998,GO:0005488,GO:0006479,	K15451|1|3e-161|568|ecb:100061739|tRNA wybutosine-synthesizing protein 4 [EC:2.1.1.-	gi|42794773|ref|NP_079580.2|	0	leucine carboxyl methyltransferase 1 [Mus musculus]

mmu-let-7c-5p	NM_025841	-30.1	0.131997	-25.97	175	GO:0044431,GO:0031090,GO:0031224,GO:0046923,GO:0004871,GO:0035437,GO:0023052,GO:0015031,	K10949|1|2e-118|426|bta:531676|ER lumen protein retaining receptor	gi|13385318|ref|NP_080117.1|	1.70E-117	ER lumen protein retaining receptor 2 [Mus musculus]
mmu-let-7c-5p	NM_026353	-28.1	0.394041	-25.58	170	GO:0005764,GO:0031224,GO:0031090,GO:0051234,	K15380|1|1e-67|259|mmu:67739|solute carrier family 48 (heme transporter), member 1	gi|74219814|dbj|BAE40496.1|	3.21E-101	unnamed protein product [Mus musculus]
mmu-let-7c-5p	NM_026376	-26.7	0.920177	-65.34	464	GO:0031224,GO:0005488,GO:0004888,GO:0001525,GO:0006935,GO:0023052,GO:00606	K06822|1|0.0|3678|mmu:67784|plexin D	gi|51243714|gb|AAT99561.1|	0	plexin D1 [Mus musculus]
mmu-let-7c-5p	NM_026566	-28.4	0.165213	-25.03	160	GO:0044444,GO:0005515,GO:0044248,GO:0007033,	-	gi|21311929|ref|NP_080842.1|	5.54E-121	autophagy-related protein 101 [Mus musculus]

mmu-let-7c-5p	NM_026838	-30.9	0.12111	-26.33	158	GO:0044424,GO:0005515,GO:0002042,GO:0007155,GO:0016310,	K14407|1|0.0|669|oaa:100082393|cleavage stimulation factor subunit 2!K06496|2|4e-22|107|gga:424364|selectin, platele	gi|123226189|emb|CAM17838.1|	0	sushi-repeat-containing protein, X-linked 2 [Mus musculus]
mmu-let-7c-5p	NM_027052	-26.1	0.851385	-45.04	305	GO:0031224,GO:0015291,GO:0005275,GO:0006865,GO:0006807,GO:0030001,	K14991|1|0.0|1010|mmu:69354|solute carrier family 38 (sodium-coupled neutral amino acid transporter), member 4	gi|31543737|ref|NP_081328.2|	0	sodium-coupled neutral amino acid transporter 4 [Mus musculus]
mmu-let-7c-5p	NM_028317	-26.7	0.807123	-42.74	301	GO:0043231,GO:0005488,	-	gi|148806918|ref|NP_082593.2|	0	protein DLN-1 [Mus musculus]
mmu-let-7c-5p	NM_028544	-27.4	0.582906	-27.63	167	GO:0005794,	K05702|1|6e-07|57.8|nve:NEMVE_v1g174672|afadin	gi|61676215|ref|NP_082820.1|	0	ras-interacting protein 1 [Mus musculus]

mmu-let-7c-5p	NM_028769	-30.4	0.232388	-25.82	162	GO:0031981,GO:0046914,GO:0016879,GO:0043161,GO:0043009,GO:0043066,	K10601|1|0.0|679|rno:361712|E3 ubiquitin-protein ligase synoviolin [EC:6.3.2.19]	gi|149062129|gb|EDM12552.1|	0	synovial apoptosis inhibitor 1, synoviolin, isoform CRA_b [Rattus norvegicus]
mmu-let-7c-5p	NM_029157	-26	0.563582	-26.69	155	GO:0005681,GO:0016604,GO:0046914,GO:0006376,	K12827|1|0.0|971|rno:313583|splicing factor 3A subunit 3	gi|148698409|gb|EDL30356.1|	0	mCG17252, isoform CRA_b [Mus musculus]
mmu-let-7c-5p	NM_030219	-25.7	0.756933	-25.43	154	GO:0044464,GO:0046914,	K12018|1|0.0|1503|mmu:78911|tripartite motif-containing protein 42	gi|116174784|ref|NP_084495.2|	0	tripartite motif-containing protein 42 [Mus musculus]
mmu-let-7c-5p	NM_053214	-30.2	0.276091	-25.6	171	GO:0016459,GO:0005884,GO:0008092,GO:0017111,GO:0032559,GO:0016477,GO:0002444,GO:0002283,GO:0030036,GO:0007155,GO:0031347,	K10356|1|0.0|2111|mmu:17916|myosin I	gi|255069756|ref|NP_444444.2|	0	unconventional myosin-If [Mus musculus]

mmu-let-7c-5p	NM_054044	-27.2	0.811621	-25.61	164	GO:0031224,GO:0004888,GO:0001525,GO:0016477,GO:0032101,GO:0022603,GO:0007399,GO:0007186,	K08461|1|0.0|2430|mmu:78560|G protein-coupled receptor 124	gi|300934759|ref|NP_473385.2|		0	G-protein coupled receptor 124 precursor [Mus musculus]
mmu-let-7c-5p	NM_080437	-31.3	0.483471	-48.66	319	GO:0031224,GO:0046872,GO:0004888,GO:0007409,GO:0016337,GO:0007186,	K04602|1|0.0|5914|mmu:107934|cadherin EGF LAG seven-pass G-type receptor 3 (flamingo)	gi|125719165|ref|NP_536685.2|		0	cadherin EGF LAG seven-pass G-type receptor 3 precursor [Mus musculus]
mmu-let-7c-5p	NM_133355	-26	0.829928	-46.2	307	GO:0030425,GO:0044459,GO:0008092,GO:0007186,GO:0007010,GO:0048167,	K04512|1|3e-41|168|tca:658864|dishevelled associated activator of morphogenesis	gi|19111166|ref|NP_579933.1|		0	delphilin isoform 2 [Mus musculus]

mmu-let-7c-5p	NM_133721	-30.3	0.44809	-28.63	172	GO:0005887,GO:0004871,GO:0006935,GO:0007166,	K06585|1|0.0|1915|rno:685004|integrin alpha 9	gi|165377045|ref|NP_598482.2|	0	integrin alpha 9 isoform a precursor [Mus musculus]
mmu-let-7c-5p	NM_139142	-31.6	0.070129	-28.09	160	GO:0044459,GO:0016021,GO:0015291,GO:0005326,GO:0005275,GO:0015804,GO:0006807,GO:0035725,	K05048|1|0.0|1121|mmu:102680|solute carrier family 6 (neurotransmitter transporter, amino acid/orphan) member 15/16/17/18/20	gi|20982825|ref|NP_631881.1|	0	sodium- and chloride-dependent transporter XTRP3A [Mus musculus]
mmu-let-7c-5p	NM_144941	-28.7	0.416126	-25.48	156	GO:0044424,	K10433|1|7e-34|147|acs:100551816|microtubule-associated protein 7	gi|148698349|gb|EDL30296.1|	0	arginine/proline rich coiled-coil 1, isoform CRA_b [Mus musculus]
mmu-let-7c-5p	NM_145418	-31.3	0.061804	-28.22	160	GO:0031224,	-	gi|21703834|ref|NP_663393.1|	2.19E-168	uncharacterized protein C6orf72 homolog precursor [Mus musculus]

mmu-let-7c-5p	NM_145942	-30.4	0.219678	-27.65	169	GO:0044444,GO:0046912,GO:0042802,GO:0009642,GO:0007417,GO:0010038,GO:0033273,GO:0032354,GO:0014070,GO:0006720,GO:0006694,GO:0070723,GO:0008406,GO:00	K01641|1|0.0|1052|mmu:208715|hydroxymethylglutaryl-CoA synthase [EC:2.3.3.10]	gi|148686390|gb|EDL18337.1|	0	3-hydroxy-3-methylglutaryl-Coenzyme A synthase 1, isoform CRA_b [Mus musculus]
mmu-let-7c-5p	NM_146071	-30.3	0.174395	-26.69	157	GO:0016020,GO:0005488,GO:0000165,GO:0007169,	-	gi|225007557|ref|NP_666183.2|	0	mucin-20 isoform b precursor [Mus musculus]
mmu-let-7c-5p	NM_146155	-31.6	0.280665	-51.36	328	-	K02350|1|2e-08|63.5|tgu:100217592|DNA polymerase zeta subunit [EC:2.7.7.7]	gi|148698133|gb|EDL30080.1|	0	AT hook, DNA binding motif, containing 1, isoform CRA_b [Mus musculus]
mmu-let-7c-5p	NM_153161	-31.7	0.098073	-27.21	158	-	-	gi|264681507|ref|NP_694801.2|	0	uncharacterized protein C7orf43 homolog [Mus musculus]

mmu-let-7c-5p	NM_153459	-26.4	0.747199	-48.71	316	GO:0031981,GO:0044444,GO:0005488,GO:0008138,GO:0006470,GO:0000165,GO:0002224,GO:0007169,GO:0043405,	K04459|1|0.0|728|mmu:235584|dual specificity phosphatase [EC:3.1.3.16 3.1.3.48]	gi|238231443|ref|NP_703189.3|	0	dual specificity protein phosphatase 7 [Mus musculus]
mmu-let-7c-5p	NM_172710	-34.2	0.069896	-30.78	175	GO:0031224,	K07126|1|9e-35|150|ptr:469885|	gi|28972381|dbj|BAC65644.1|	0	mKIAA0746 protein [Mus musculus]
mmu-let-7c-5p	NM_172716	-30.9	0.207232	-47.91	319	GO:0046914,GO:0010467,	K11488|1|2e-144|514|ptr:461045|polycomb group RING finger protein 3	gi|148688164|gb|EDL20111.1|	3.18E-148	polycomb group ring finger 3, isoform CRA_c [Mus musculus]
mmu-let-7c-5p	NM_172717	-27.6	0.551079	-26.38	162	GO:0016604,GO:0046914,GO:0019787,GO:0000075,GO:0019941,GO:0000087,GO:0016567,	K10644|1|0.0|1262|mmu:231600|E3 ubiquitin-protein ligase CHFR [EC:6.3.2.19]	gi|148688094|gb|EDL20041.1|	0	checkpoint with forkhead and ring finger domains, isoform CRA_d [Mus musculus]

mmu-let-7c-5p	NM_172800	-26.4	0.935423	-43.29	296	GO:0031224,GO:0009987,	K06252|1|2e-97|359|bfo:BRAFLDRAFT_124749|tenascin	gi|40254254|ref|NP_766388.2|	0	protein sidekick-2 precursor [Mus musculus]
mmu-let-7c-5p	NM_175520	-27.7	0.54973	-26.66	172	GO:0031224,GO:0001608,GO:0043627,GO:0007166,	K08401|1|0.0|647|mmu:243270|G protein-coupled receptor 81	gi|259490314|ref|NP_780729.2|	0	hydroxycarboxylic acid receptor 1 [Mus musculus]
mmu-let-7c-5p	NM_175556	-27	0.840807	-46.48	300	GO:0016020,GO:0060089,GO:0046872,GO:0004629,GO:0007165,GO:0006650,	K15370|1|7e-104|380|nve:NEMVE_v1g89019|inactive phospholipase C-like	gi|148683042|gb|EDL14989.1|	0	phospholipase C-like 4 [Mus musculus]
mmu-let-7c-5p	NM_175730	-29.5	0.255776	-27.23	161	GO:0031981,GO:0044459,GO:0001071,GO:0003677,GO:0003002,GO:0006355,GO:00015	K09305|1|9e-107|389|ecb:100064385|homeobox protein HoxA/B/C5	gi|359323087|ref|XP_849475.2|	2.49E-115	PREDICTED: uncharacterized protein LOC607625 [Canis lupus familiaris]

mmu-let-7c-5p	NM_177265	-26.8	0.685693	-25.03	154	GO:0031224,GO:0050896,	K06566|1|4e-08|61.6|xla:734801|interferon induced transmembrane protein	gi|148686194|gb|EDL18141.1|	4.81E-121	RIKEN cDNA 6330512M04 [Mus musculus]
mmu-let-7c-5p	NM_177342	-29.8	0.272789	-27.54	158	GO:0005856,GO:0005667,GO:0004468,GO:0001071,GO:0043565,GO:0005515,GO:0051704,GO:0006352,GO:0006325,GO:00063	K03130|1|0.0|1285|mmu:226182|transcription initiation factor TFIID subunit 5	gi|148710078|gb|EDL42024.1|	0	TAF5 RNA polymerase II, TATA box binding protein (TBP)-associated factor [Mus musculus]

mmu-let-7c-5p	NM_177475	-30.1	0.356063	-27.91	158	-	K09228|1|2e-12|76.6|gga:430731|KRAB domain-containing zinc finger protein!K08582|3|2e-12|76.3|cqu:CpipJ_CPIJ010344|calpain-15 [EC:3.4.22.-]	gi|158749569|ref|NP_803426.2|	0	zinc finger protein 280b [Mus musculus]
mmu-let-7c-5p	NM_177613	-26.9	0.324439	-25.68	164	GO:0031981,GO:0016787,GO:0032559,GO:0019787,GO:0000209,GO:0010033,GO:0009968,GO:0006259,GO:0006511,GO:0022402,GO:0043065,	K02207|1|1e-115|417|rno:299602|ubiquitin-conjugating enzyme E2 R [EC:6.3.2.19]	gi|397502346|ref|XP_003821822.1|	8.37E-115	PREDICTED: ubiquitin-conjugating enzyme E2 R1 [Pan paniscus]

mmu-let-7c-5p	NM_177637	-28.8	0.162843	-26.48	158	GO:0031224,	K06751|1|1e-174|613|mmu:224761|major histocompatibility complex, class I	gi|114842391|ref|NP_808305.2|	6.79E-174	histocompatibility 2, M region locus 10.5 precursor [Mus musculus]
mmu-let-7c-5p	NM_177897	-28.7	0.384973	-26.14	158	GO:0000139,GO:0031224,GO:0008376,	K09657|1|0.0|1837|mmu:330671|beta-1,4-N-acetylgalactosaminyltransferase 4 [EC:2.4.1.244]	gi|58372116|ref|NP_808565.2|	0	N-acetyl-beta-glucosaminyl-glycoprotein 4-beta-N-acetylgalactosaminyltransferase 1 [Mus musculus]
mmu-let-7c-5p	NM_178659	-26.4	0.849175	-46.83	316	-	K11323|1|1e-20|103|dwi:Dwil_GK14253|histone arginine demethylase JMJD6 [EC:1.14.1	gi|30520031|ref|NP_848774.1|	0	jmjC domain-containing protein 4 isoform 1 [Mus musculus]

mmu-let-7c-5p	NM_178752	-25.4	0.900676	-44.5	307	-	K15630|1|0.0|780|mmu:269952|GDP-D-glucose phosphorylase [EC:2.7.7.78]	gi|123702129|ref|NP_848867.2|	0	GDP-D-glucose phosphorylase 1 [Mus musculus]
mmu-let-7c-5p	NM_178774	-30.6	0.202943	-26.69	168	-	-	gi|255308901|ref|NP_848889.3|	2.86E-129	proline-rich protein 18 [Mus musculus]
mmu-let-7c-5p	NM_183106	-31.6	0.197827	-51.57	325	-	-	gi|124430551|ref|NP_898929.2|	0	tetratricopeptide repeat domain 17 [Mus musculus]
mmu-let-7c-5p	NM_198421	-26.7	0.875077	-41.23	310	GO:0046914,GO:0016790,GO:0070011,GO:0019941,	K11834|1|0.0|1292|mmu:224836|ubiquitin carboxyl-terminal hydrolase 44/49 [EC:3.1.2.15]	gi|38259220|ref|NP_940813.1|	0	ubiquitin carboxyl-terminal hydrolase 49 [Mus musculus]
mmu-let-7c-5p	NM_198422	-28.4	0.188495	-26.88	162	GO:0012505,GO:0043231,GO:0031224,GO:0004871,GO:0023052,	K07297|1|5e-32|139|cqu:CpipJ_CPIJ002779|adiponectin receptor	gi|66272333|gb|AAH96380.1|	8.06E-180	Paqr3 protein [Mus musculus]

mmu-let-7c-5p	NR_026565	-25.1	0.954108	-41.81	328	GO:0000783,GO:0043566,GO:0006259,	-	gi|148678523|gb|EDL10470.1|	0	RIKEN cDNA 1500010J02, isoform CRA_b [Mus musculus]
mmu-let-7c-5p	NR_027838	-29.9	0.435536	-45.35	311	GO:0032991,GO:0016021,GO:0005789,GO:0001664,GO:0019829,GO:0046872,GO:0032559,GO:0009888,GO:0007599,GO:0008016,GO:0006816,GO:0006936,GO:0009207,GO:0007165,GO:0001775,GO:0009206,GO:0009725,GO:0006875,	K05853|1|0.0|2035|mmu:11938|Ca2+ transporting ATPase, sarcoplasmic/endoplasmic reticulum [EC:3.6.3.8]	gi|60360584|dbj|BAD90532.1|	0	mKIAA4195 protein [Mus musculus]
mmu-let-7c-5p	NM_181729	-24.2	0.999747	-93.31	638	-	K13908|1|0.0|868|ecb:100147340|mucin-5B	gi|32171192|ref|NP_859418.1|	0	mucin-6 precursor [Mus musculus]

mmu-let-7d-3p	NM_010395	-28.5	0.427209	-26.79	163	GO:0044425,	K06751|1|0.0|736|mmu:15024|major histocompatibility complex, class I	gi|199664|gb|AAA39695.1|	0	MHC H2-TL-T10-129 [Mus musculus]
mmu-let-7d-3p	NM_010397	-28.4	0.282053	-26.79	163	GO:0016020,	K06751|1|0.0|724|mmu:15039|major histocompatibility complex, class I	gi|148691329|gb|EDL23276.1|	0	mCG132391, isoform CRA_b [Mus musculus]
mmu-let-7d-3p	NM_010399	-28.5	0.252294	-26.79	163	GO:0016020,	K06751|1|0.0|731|mmu:15051|major histocompatibility complex, class I	gi|148691329|gb|EDL23276.1|	0	mCG132391, isoform CRA_b [Mus musculus]
mmu-let-7d-3p	NM_176999	-33.3	0.18477	-29.15	179	GO:0031224,GO:0042625,GO:0046872,GO:0005548,GO:0032559,GO:0009206,GO:0006869,	K01530|1|0.0|1825|oaa:100074214|phospholipid-translocating ATPase [EC:3.6.3.1	gi|125628644|ref|NP_795973.2|	0	probable phospholipid-transporting ATPase VB [Mus musculus]
mmu-let-7d-3p	NR_045063	-23.4	0.987349	-20.32	170	-	-	gi|148671346|gb|EDL03293.1|	8.39E-22	mCG141159, isoform CRA_c [Mus musculus]

mmu-let-7d-3p	NM_025596	-26	0.433752	-21.18	168	GO:0031981,GO:0032501,GO:0002376,	-	gi|46518501|ref|NP_079872.4|	1.17E-103	PRELI domain-containing protein 1, mitochondrial precursor [Mus musculus]
mmu-let-7d-3p	NM_177589	-24	0.995257	-23	167	GO:0004672,GO:0032559,GO:0006464,	K06228|1|2e-35|152|bfo:BRAFLDRAFT_175974|fused [EC:2.7.11.1]	gi|226958505|ref|NP_808257.2|	0	serine/threonine-protein kinase ULK4 [Mus musculus]
mmu-let-7d-3p	NM_008585	-24.9	0.932382	-20.31	166	GO:0044421,GO:0016021,GO:0004175,GO:0046914,GO:0019538,GO:0032501,	K01395|1|0.0|1541|mmu:17287|meprin A, alpha [EC:3.4.24.18]	gi|31982199|ref|NP_032611.2|	0	meprin A subunit alpha [Mus musculus]
mmu-let-7d-3p	NM_175642	-27.8	0.771481	-22.58	165	GO:0031224,GO:0004888,GO:0001525,GO:0007186,	K04598|1|0.0|3007|mmu:210933|brain-specific angiogenesis inhibitor 3	gi|169790905|ref|NP_783573.4|	0	brain-specific angiogenesis inhibitor 3 precursor [Mus musculus]

mmu-let-7d-3p	NM_033552	-26.1	0.945872	-20.94	163	GO:0044459,GO:0031224,GO:0015301,GO:0006464,GO:0055067,GO:0030001,	K13861|1|0.0|2028|mmu:94229|solute carrier family 4 (sodium bicarbonate transporter), member 10	gi|148695037|gb|EDL26984.1|	0	solute carrier family 4, sodium bicarbonate cotransporter-like, member 10, isoform CRA_b [Mus musculus]
mmu-let-7d-3p	NM_177584	-21.7	0.99999	-21.11	160	GO:0016021,GO:0009986,GO:0005488,GO:0004871,GO:0042130,GO:0030888,GO:0002764,	K06707|1|3e-161|570|mmu:208154|B- and T-lymphocyte attenuator	gi|29243938|ref|NP_808252.1|	5.62E-162	B- and T-lymphocyte attenuator isoform 2 precursor [Mus musculus]
mmu-let-7d-3p	NM_080795	-21.7	1	-24.29	159	GO:0046914,GO:0019904,GO:0051259,	K10692|1|0.0|1338|mmu:140887|ligand of Numb protein X	gi|165932387|ref|NP_542985.4|	0	ligand of Numb protein X 2 [Mus musculus]
mmu-let-7d-3p	NR_046286	-25.4	0.882834	-23.5	158	GO:0044425,	K06751|1|0.0|460|mmu:15024|major histocompatibility complex, class I	gi|199664|gb|AAA39695.1|	0	MHC H2-TL-T10-129 [Mus musculus]

mmu-let-7d-3p	NM_199304	-22.7	0.999481	-20.54	155	GO:0031981,GO:0046914,GO:0003676,	K09228|1|7e-63|243|bfo:BRAFLDRAFT_71028|KRAB domain-containing zinc finger protein	gi|76559897|ref|NP_955008.2|	0	zinc finger protein 341 [Mus musculus]
mmu-let-7d-3p	NM_178879	-25.1	0.861256	-20.49	151	GO:0012505,GO:0043231,GO:0031224,GO:0016758,GO:0006464,	K09664|1|1e-74|282|mdo:100020682|beta-1,3-N-acetylglucosaminyltransferase 7 [EC:2.4.1.-]	gi|30725708|ref|NP_849210.1|	0	UDP-GlcNAc:betaGal beta-1,3-N-acetylglucosaminyltransferase 9 precursor [Mus musculus]
mmu-let-7d-3p	NM_008527	-24.7	0.928647	-21.9	148	GO:0016021,GO:0004871,GO:0042802,GO:0030246,GO:0001912,GO:0023052,	K06543|1|1e-159|564|mmu:17059|killer cell lectin-like receptor subfamily	gi|229577430|ref|NP_032553.2|	2.44E-160	killer cell lectin-like receptor subfamily B member 1C isoform 2 [Mus musculus]

mmu-let-7d-3p	NM_198644	-23.9	0.997412	-21.08	146	GO:0043231,GO:0044444,GO:0046914,GO:0003676,GO:0006351,	K09228|1|5e-51|204|xtr:100216012|KRAB domain-containing zinc finger protein	gi|148697448|gb|EDL29395.1|	0	zinc finger protein 406, isoform CRA_b [Mus musculus]
mmu-let-7d-3p	NM_001163548	-24.9	0.974281	NA	NA	GO:0031252,GO:0016020,GO:0044424,GO:0004872,GO:0005083,GO:0035091,GO:0019222,GO:0030155,GO:0006810,GO:00320	K12495|1|8e-41|170|bfo:BRAFLDRAFT_106693|IQ motif and SEC7 domain-containing protein	gi|13938146|gb|AAH07189.1|	0	Cyth3 protein [Mus musculus]
mmu-let-7d-3p	NM_001013757	-20.6	1	NA	NA	-	-	gi|147903647|ref|NP_001013779.2|	0	von Willebrand factor D and EGF domain-containing protein precursor [Mus musculus]

mmu-let-7d-3p	NM_010586	-20.2	1	NA	NA	GO:0043005,GO:0030133,GO:0005789,GO:0031224,GO:0005262,GO:0005217,GO:0046873,GO:0005543,GO:0004872,GO:0015980,GO:0007599,GO:0006816,GO:0010863,GO:0007169,GO:0030073,GO:0006950,GO:00	K04959|1|0.0|5027|mmu:16439|inositol 1,4,5-triphosphate receptor type 2	gi|60592758|ref|NP_034716.1|	0	inositol 1,4,5-trisphosphate receptor type 2 isoform 2 [Mus musculus]
mmu-let-7d-3p	NM_009680	-25.1	0.964108	NA	NA	GO:0031982,GO:0044431,GO:0043234,GO:0022892,GO:0019902,GO:0007599,GO:0048753,GO:0006623,GO:0019884,GO:0016192,GO:0000041,GO:0046638,	K12397|1|0.0|1833|mmu:11774|AP-3 complex subunit beta	gi|163310776|ref|NP_033810.2|	0	AP-3 complex subunit beta-1 [Mus musculus]

mmu-let-7d-3p	NM_018867	-21.8	0.999993	NA	NA	GO:0044421,GO:0046914,GO:0008235,GO:0019538,GO:0009987,	K08639|1|0.0|1383|mmu:55987|carboxypeptidase X2	gi|229092758|ref|NP_061355.3|	0	inactive carboxypeptidase-like protein X2 precursor [Mus musculus]
mmu-let-7d-3p	NM_153395	-21.9	1	NA	NA	GO:0044424,GO:0005083,GO:0006810,	-	gi|148692491|gb|EDL24438.1|	0	MON2 homolog (yeast), isoform CRA_d [Mus musculus]
mmu-let-7d-3p	NM_001170710	-21.3	0.999943	NA	NA	GO:0043231,GO:0007165,	K10330|1|5e-161|568|mmu:78541|ankyrin repeat and SOCS box protein 8	gi|282397100|ref|NP_084397.3|	3.32E-160	ankyrin repeat and SOCS box protein 8 [Mus musculus]
mmu-let-7d-3p	NM_001163549	-21.7	1	NA	NA	-	K10388|1|0.0|3881|mmu:18810|plectin	gi|256418964|ref|NP_958788.2|	0	plectin isoform 1e [Mus musculus]
mmu-let-7d-3p	NM_008251	-30.1	0.088532	NA	NA	GO:0043231,GO:0005488,GO:0009411,GO:0006289,GO:0051276,GO:0042127,GO:0048050,	K11299|1|7e-22|105|mmu:15312|high-mobility group nucleosome-binding domain-containing protein 1	gi|119630061|gb|EAX09656.1|	2.82E-28	high-mobility group nucleosome binding domain 1, isoform CRA_g [Homo sapiens]

mmu-let-7d-3p	NM_028319	-20.6	1	NA	NA	GO:0043231,GO:0046914,GO:0003676,GO:0006351,	K09228|1|7e-15|85.1|bfo:BRAFLDRAFT_66688|KRAB domain-containing zinc finger protein	gi|133507240|ref|NP_082595.1|	0	zinc finger protein 518A [Mus musculus]
mmu-let-7d-3p	NM_001166669	-21.3	1	NA	NA	GO:0016604,GO:0044444,GO:0030529,GO:0003723,GO:0000398,GO:0065003,	K13133|1|0.0|2805|mmu:216766|gem associated protein 5	gi|262263299|ref|NP_001160141.1|	0	gem-associated protein 5 isoform 1 [Mus musculus]
mmu-let-7d-3p	NM_172775	-27.3	0.933465	NA	NA	GO:0043235,GO:0016021,GO:0008047,GO:0005102,GO:0004888,GO:0065008,GO:0048870,GO:0031346,GO:0006935,GO:0033043,GO:0032501,GO:0009966,GO:00071	K06821|1|0.0|4042|mmu:235611|plexin B	gi|148689398|gb|EDL21345.1|	0	plexin B1, isoform CRA_b [Mus musculus]

mmu-let-7d-3p	NM_021539	-21.9	0.999756	NA	NA	GO:0007165,	K10342|1|0.0|813|mmu:59043|WD repeat and SOCS box-containing protein 2	gi|31982686|ref|NP_067514.2|	0	WD repeat and SOCS box-containing protein 2 [Mus musculus]
mmu-let-7d-3p	NM_175335	-20.5	1	NA	NA	GO:0005667,GO:0003676,GO:0046983,GO:0008134,GO:0006352,GO:0006354,GO:0006366,	K03122|1|2e-110|402|mmu:83602|transcription initiation factor TFIIA large subunit	gi|148687003|gb|EDL18950.1|	1.56E-109	general transcription factor II A, 1, isoform CRA_a [Mus musculus]
mmu-let-7d-3p	NM_053096	-20.8	1	NA	NA	GO:0019866,GO:0031224,GO:0016407,GO:0007369,	-	gi|31542409|ref|NP_444326.2|	2.52E-109	probable N-acetyltransferase CML2 [Mus musculus]

mmu-let-7d-3p	NM_134086	-28.2	0.823144	NA	NA	GO:0031224,GO:0043005,GO:0015175,GO:0005343,GO:0015804,GO:0001505,GO:0006807,GO:0030001,	K14990|1|0.0|841|mmu:105727|solute carrier family 38 (sodium-coupled neutral amino acid transporter), member 1	gi|148672291|gb|EDL04238.1|	0	solute carrier family 38, member 1, isoform CRA_a [Mus musculus]
mmu-let-7d-3p	NM_010109	-24.2	0.997608	NA	NA	GO:0044421,GO:0044459,GO:0031225,GO:0005102,GO:0007417,GO:0007411,GO:00071	K05462|1|8e-110|400|mmu:13640|ephrin-A	gi|6753724|ref|NP_034239.1|	5.39E-109	ephrin-A5 isoform 2 precursor [Mus musculus]
mmu-let-7d-3p	NM_146786	-20.6	0.998097	NA	NA	GO:0004888,GO:0023052,	K04257|1|1e-172|605|mmu:258782|olfactory	gi|121583649|ref|NP_666997.2|	8.05E-172	olfactory receptor 914 [Mus musculus]
mmu-let-7d-3p	NR_029728	-35.5	0.000029	NA	NA	-	-	-	-	-
mmu-let-7d-3p	NM_181819	-21.9	0.999984	NA	NA	GO:0005488,GO:0004866,GO:0007178,GO:0051346,	K03909|1|7e-16|87.4|dre:359833|tissue factor pathway inhibitor	gi|267844918|ref|NP_861540.2|	0	WAP, kazal, immunoglobulin, kunitz and NTR domain-containing protein 2 precursor [Mus musculus]

mmu-let-7d-3p	NM_009764	-23.6	0.999921	NA	NA	GO:0000931,GO:0000228,GO:0044429,GO:0000152,GO:0019787,GO:0008092,GO:0003677,GO:0046914,GO:0019899,GO:0031327,GO:0031667,GO:0009314,GO:0046599,GO:0007549,GO:0000077,GO:0030330,GO:0009792,GO:0043627,GO:0000725,GO:0006282,GO:0007417,GO:00082	K10605|1|0.0|3555|mmu:12189|breast cancer type 1 susceptibility protein	gi|161016835|ref|NP_033894.3|	0	breast cancer type 1 susceptibility protein homolog [Mus musculus]
mmu-let-7d-3p	NM_172785	-21.4	1	NA	NA	GO:0046914,	-	gi|226423912|ref|NP_766373.2|	0	probable ribonuclease ZC3H12D [Mus musculus]

mmu-let-7d-3p	NM_001122666	-23.4	0.984519	NA	NA	GO:0070822,GO:0019899,GO:0006355,GO:0006325,	-	gi|169881250|ref|NP_001116138.1|	1.65E-136	sin3 histone deacetylase corepressor complex component SDS3 isoform b [Mus musculus]
mmu-let-7d-3p	NM_172832	-22	0.998789	NA	NA	GO:0016667,GO:0030329,	K05906|1|0.0|811|ecb:100060629|prenylcysteine oxidase / farnesylcysteine lyase [EC:1.8.3.5 1.8.3.6]	gi|27370248|ref|NP_766420.1|	0	prenylcysteine oxidase-like precursor [Mus musculus]
mmu-let-7d-3p	NM_133774	-24.6	0.955486	NA	NA	GO:0043231,GO:0005488,GO:0051234,GO:0010876,	-	gi|19526998|ref|NP_598535.1|	2.57E-129	stAR-related lipid transfer protein 4 [Mus musculus]

mmu-let-7d-3p	NM_030210	-20.9	1	NA	NA	GO:0044444,GO:0016878,GO:0032559,GO:0050796,GO:0009888,GO:0044238,GO:0033574,GO:0031667,GO:0032787,GO:0070542,GO:0001678,GO:0014070,GO:0048513,GO:0070723,GO:0006950,GO:00454	K01907|1|0.0|1368|mmu:78894|acetoacetyl-CoA synthetase [EC:6.2.1.16]	gi|21313520|ref|NP_084486.1|	0	acetoacetyl-CoA synthetase [Mus musculus]
mmu-let-7d-3p	NM_146030	-27.3	0.626689	NA	NA	GO:0043232,GO:0023052,	K12559|1|1e-101|372|dre:566674|myosin X	gi|22122503|ref|NP_666142.1|	0	pleckstrin homology domain-containing family H member 3 precursor [Mus
mmu-let-7d-3p	NM_001104621	-22.3	0.999299	NA	NA	-	K04613|1|0.0|1412|mmu:211223|vomeronasal 2 receptor	gi|157277962|ref|NP_001098091.1|	0	vomeronasal 2, receptor 9 precursor [Mus musculus]

mmu-let-7d-3p	NM_026438	-27.5	0.290079	NA	NA	GO:0044444,GO:0046872,GO:0016462,GO:0006412,GO:0006796,	K01507|1|1e-174|613|mmu:67895|inorganic pyrophosphatase [EC:3.6.1.1	gi|148700191|gb|EDL32138.1|	0	pyrophosphatase (inorganic) 1 [Mus musculus]
mmu-let-7d-3p	NM_001101488	-20.6	1	NA	NA	GO:0031224,	K14356|1|3e-15|85.5|bfo:BRAFLDRAFT_199004|solute carrier organic anion transporter family, member 5A!K12495|3|5e-06|45.1|dre:560461|IQ motif and SEC7 domain-containing protein	gi|157951733|ref|NP_001094958.1|	2.64E-161	GSG1-like precursor [Mus musculus]

mmu-let-7d-3p	NM_013829	-24.4	0.983882	NA	NA	GO:0043005,GO:0044430,GO:0005783,GO:0004629,GO:0046872,GO:0060089,GO:0019901,GO:0006629,GO:0006813,GO:00071	K05858|1|0.0|2301|mmu:18798|phosphatidylinositol phospholipase C, beta [EC:3.1.4.11]	gi|29293803|ref|NP_038857.1|	0	1-phosphatidylinositol-4,5-bisphosphate phosphodiesterase beta-4 [Mus musculus]
mmu-let-7d-3p	NR_024328	-21.5	0.999831	NA	NA	-	-	gi|148707601|gb|EDL39548.1|	7.88E-26	mCG148400 [Mus musculus]
mmu-let-7d-3p	NM_001253807	-22.8	0.999157	NA	NA	GO:0032991,GO:0044430,GO:0048770,GO:0005515,GO:0042221,GO:0007599,GO:0006605,GO:0016070,GO:0002441,GO:0001775,GO:0043066,	K06630|1|1e-118|428|rno:25578|tyrosine 3-monooxygenase/tryptophan 5-monooxygenase activation protein	gi|148676868|gb|EDL08815.1|	8.28E-118	tyrosine 3-monooxygenase/tryptophan 5-monooxygenase activation protein, zeta polypeptide, isoform CRA_b [Mus musculus]

mmu-let-7d-3p	NM_007595	-22.4	0.999966	NA	NA	GO:0031981,GO:0016020,GO:0044430,GO:0030139,GO:0043234,GO:0005515,GO:0004674,GO:0032559,GO:0048168,GO:0002029,GO:0019221,GO:0006468,GO:0010038,GO:0070838,GO:0045836,GO:0007271,GO:00	K04515|1|0.0|1054|bta:525416|calcium/calmodulin-dependent protein kinase (CaM kinase) II [EC:2.7.11.17]	gi|108796657|ref|NP_068507.2|	0	calcium/calmodulin-dependent protein kinase type II subunit beta isoform 2 [Rattus norvegicus]
mmu-let-7d-3p	NM_198052	-23.6	0.999457	NA	NA	-	K10177|1|0.0|1065|mmu:21386|T-box protein 3	gi|38327623|ref|NP_932169.1|	0	T-box transcription factor TBX3 isoform 2 [Mus musculus]
mmu-let-7d-3p	NM_011810	-31.2	0.043558	NA	NA	GO:0044424,GO:0010720,GO:0007243,GO:0043066,	-	gi|171184430|ref|NP_001116323.1|	1.07E-103	fas apoptotic inhibitory molecule 1 Faim-L [Mus musculus]

mmu-let-7d-3p	NM_028121	-24.9	0.899709	NA	NA	GO:0043169,GO:0016301,GO:0006796,GO:0006007,	K08074|1|0.0|922|mmu:72141|ADP-dependent glucokinase [EC:2.7.1.147]	gi|148694017|gb|EDL25964.1|	0	ADP-dependent glucokinase, isoform CRA_b [Mus musculus]
mmu-let-7d-3p	NM_025661	-20.4	0.999999	NA	NA	GO:0017059,GO:0031224,GO:0005488,GO:0046519,	K12667|1|1e-37|158|smm:Smp_144800|oligosaccharyltransferase complex subunit delta (ribophorin II)	gi|149252419|ref|XP_889452.2|	1.15E-90	PREDICTED: ORM1-like protein 3-like [Mus musculus]
mmu-let-7d-3p	NM_027914	-28.2	0.455662	NA	NA	GO:0032559,GO:0019538,GO:0042384,GO:0001894,GO:0043954,	-	gi|254553416|ref|NP_082190.1|	0	Bardet-Biedl syndrome 10 protein homolog [Mus musculus]
mmu-let-7d-3p	NM_001267593	-21	1	NA	NA	-	-	-	-	-
mmu-let-7d-3p	NM_001195066	-20.7	1	NA	NA	-	K14798|1|6e-27|125|ssc:100627735|protein LTV1	gi|304361727|ref|NP_001181995.1|	0	phosphatase and actin regulator 2 isoform B [Mus musculus]

mmu-let-7d-3p	NM_026154	-20.7	0.999963	NA	NA	GO:0000315,GO:0043231,GO:0005198,GO:0010467,GO:0022613,	-	gi|13385658|ref|NP_080430.1|	1.44E-120	39S ribosomal protein L10, mitochondrial precursor [Mus musculus]
mmu-let-7d-3p	NM_001101433	-20.6	1	NA	NA	GO:0046914,	-	gi|160333288|ref|NP_001094903.1|	4.62E-130	zinc finger CCHC domain-containing protein 24 [Mus musculus]
mmu-let-7d-3p	NM_001177955	-21.5	0.999998	NA	NA	GO:0031224,GO:0048731,GO:0048869,	-	gi|295789068|ref|NP_001171426.1|	0	neuronal membrane glycoprotein M6-b isoform 1 [Mus
mmu-let-7d-3p	NM_001081407	-20.9	1	NA	NA	GO:0044459,GO:0031224,GO:0004091,GO:0016101,	K14621|1|0.0|2901|mmu:665270|phospholipase B1, membrane-associated [EC:3.1.1.4 3.1.1.5]	gi|124487227|ref|NP_001074876.1|	0	phospholipase B1, membrane-associated isoform 1 precursor [Mus musculus]
mmu-let-7d-3p	NM_008013	-21.5	0.999999	NA	NA	GO:0005615,GO:0044464,GO:0008047,GO:0005515,GO:0016787,GO:0022414,GO:0051345,GO:0023052,	K10104|1|1e-55|219|nve:NEMVE_v1g90597|ficolin!K05466|2|4e-50|201|mdo:100028850|angi	gi|31542814|ref|NP_032039.2|	0	fibroleukin precursor [Mus musculus]

mmu-let-7d-3p	NM_001256101	-22.3	0.999998	NA	NA	GO:0043231,GO:0043021,GO:0008135,GO:0006413,GO:0032988,	K02520|1|2e-148|528|mmu:76366|translation initiation factor IF-	gi|21313176|ref|NP_083857.1|	1.30E-147	translation initiation factor IF-3, mitochondrial [Mus musculus]
mmu-let-7d-3p	NM_001270792	-20.5	0.999994	NA	NA	-	-	-	-	-
mmu-let-7d-3p	NM_001271725	-21.4	1	NA	NA	-	-	-	-	-
mmu-let-7d-3p	NM_001115085	-33.6	0.021722	NA	NA	-	-	gi|9938004|ref|NP_064681.1|	2.26E-142	protein TSSC4 isoform 1 [Mus musculus]
mmu-let-7d-3p	NM_001001983	-23.2	0.99998	NA	NA	GO:0000139,GO:0016301,GO:0046488,GO:0048017,	K00888|1|0.0|3944|mmu:224020|phosphatidylinositol 4-kinase [EC:2.7.1.67]	gi|241982771|ref|NP_001001983.2|	0	phosphatidylinositol 4-kinase type 3 alpha [Mus musculus]
mmu-let-7d-3p	NM_001198872	-22	0.999822	NA	NA	GO:0005875,GO:0044444,GO:0005515,GO:0003774,GO:0051234,GO:0007017,GO:00224	K10415|1|0.0|1191|mmu:13427|dynein intermediate chain, cytosolic	gi|311893374|ref|NP_001185801.1|	0	cytoplasmic dynein 1 intermediate chain 2 isoform 1 [Mus musculus]

mmu-let-7d-3p	NM_053187	-20.3	1	NA	NA	GO:0043005,GO:0044430,GO:0012505,GO:0005798,GO:0044459,GO:0030695,GO:0005515,GO:0043002,GO:0008105,GO:0051259,GO:0006997,GO:0044248,	K14548|1|4e-10|68.6|tgu:100229140|U3 small nucleolar RNA-associated protein 4!K06092|2|6e-09|64.7|acs:100561106|InaD-like protein!K12075|4|8e-09|64.3|bmy:Bm1_32060|discs large protein	gi|31543485|ref|NP_444417.2|	0	Golgi-associated PDZ and coiled-coil motif-containing protein isoform b [Mus musculus]
mmu-let-7d-3p	NM_148931	-21.3	0.999982	NA	NA	GO:0016021,GO:0005326,GO:0015175,GO:0005283,GO:0015804,GO:0007267,GO:0035725,	K05038|1|0.0|1638|mmu:104245|solute carrier family 6 (neurotransmitter transporter, amino acid) member 5/7/9/14	gi|225579049|ref|NP_001139485.1|	0	sodium- and chloride-dependent glycine transporter 2 isoform a [Mus musculus]

mmu-let-7d-3p	NM_001256132	-20.4	1	NA	NA	GO:0031224,GO:0005768,GO:0070838,	-	gi|148689926|gb|EDL21873.1|	1.03E-165	non imprinted in Prader-Willi/Angelman syndrome 2 homolog (human), isoform CRA_b [Mus musculus]
mmu-let-7d-3p	NM_001083894	-22.9	0.999655	NA	NA	GO:0044464,GO:0003824,GO:0006629,	K01066|1|7e-123|442|dre:445105|esterase / lipase [EC:3.1.1.-]!K13618|2|8e-74|280|mgp:100546329|phospholipase A1 member A [EC:3.1.1.-]	gi|148665200|gb|EDK97616.1|	0	lipase, member H, isoform CRA_d [Mus musculus]
mmu-let-7d-3p	NM_176839	-23.4	0.88095	NA	NA	GO:0016021,GO:0044444,GO:0017111,GO:0008509,GO:0015405,GO:0032559,GO:0006820,GO:0043434,GO:0009207,GO:00022	K05668|1|1e-112|406|mmu:27416|ATP-binding cassette, subfamily C (CFTR/MRP), member 5	gi|66932956|ref|NP_789809.1|	3.27E-118	multidrug resistance-associated protein 5 isoform 2 [Mus musculus]

mmu-let-7d-3p	NM_001025392	-21.6	1	NA	NA	GO:0031981,GO:0003676,GO:0006915,GO:0006355,	K13087|1|0.0|1152|mmu:72567|bcl2 associated transcription factor 1	gi|148671484|gb|EDL03431.1|	0	mCG2822, isoform CRA_b [Mus musculus]
mmu-let-7d-3p	NM_010740	-21	1	NA	NA	GO:0031224,GO:0031410,GO:0046872,GO:0004871,GO:0030246,GO:0001846,GO:0002274,GO:0007155,GO:0023052,GO:00068	K06702|1|0.0|1217|mmu:17064|CDW93 antigen	gi|6754578|ref|NP_034870.1|	0	complement component C1q receptor precursor [Mus musculus]
mmu-let-7d-3p	NM_001034903	-23.3	0.873686	NA	NA	GO:0044464,	-	gi|85702266|ref|NP_001030075.1|	0	MANSC domain-containing protein 4 precursor [Mus musculus]

mmu-let-7d-3p	NM_145707	-23.3	0.950338	NA	NA	GO:0043231,GO:0003676,	K09325|1|2e-10|67.8|mmu:195333|homeobox protein goosecoid-like!K09314|2|9e-10|65.9|pon:100459176|homeobox protein DLX1/4/6!K09315|5|3e-09|64.3|xla:779428|homeobox protein DLX2/3/5	gi|188528635|ref|NP_663753.3|	0	oocyte specific homeobox 3 [Mus musculus]
mmu-let-7d-3p	NM_007705	-21.5	0.995428	NA	NA	GO:0031981,GO:0030529,GO:0003729,GO:0019843,GO:0045182,GO:0006412,GO:0009416,GO:0043488,GO:0022618,GO:00069	K13195|1|1e-42|174|rno:81825|cold-inducible RNA-binding protein	gi|119589927|gb|EAW69521.1|	1.82E-42	cold inducible RNA binding protein, isoform CRA_b [Homo sapiens]

mmu-let-7d-3p	NM_053076	-23.9	0.982297	NA	NA	GO:0044421,GO:0012505,GO:0031090,GO:0043231,GO:0031224,GO:0005488,GO:0051704,GO:0050896,GO:0007029,GO:0006810,GO:0012501,	K00688|1|2e-37|159|ame:409267|starch phosphorylase [EC:2.4.1.1]	gi|62089128|dbj|BAD93008.1|	1.54E-107	reticulon 3 isoform a variant [Homo sapiens]
mmu-let-7d-3p	NM_028004	-25	1	NA	NA	-	K12567|1|0.0|4.870e+04|hsa:7273|titin [EC:2.7.11.1]	gi|77812699|ref|NP_082280.2|	0	titin isoform N2-B [Mus musculus]
mmu-let-7d-3p	NM_145556	-21.3	1	NA	NA	GO:0043231,GO:0003729,GO:0043566,GO:0006396,GO:0032897,GO:0048255,	K03993|1|1e-139|499|mmu:17175|mannan-binding lectin serine protease 2 [EC:3.4.21.104]	gi|148682887|gb|EDL14834.1|	1.28E-167	mCG16669, isoform CRA_e [Mus musculus]

mmu-let-7d-3p	NM_001103156	-20.8	1	NA	NA	GO:0005768,GO:0031228,GO:0005798,GO:0046914,GO:0050662,GO:0003824,GO:0006892,GO:0009719,GO:0006091,GO:0000041,GO:0006887,	K14738|1|0.0|935|mmu:74051|metalloreductase STEAP2 [EC:1.16.1.-]	gi|124249344|ref|NP_083010.2|	0	metalloreductase STEAP2 [Mus musculus]
mmu-let-7d-3p	NM_001204979	-20.4	1	NA	NA	-	K01875|1|0.0|966|mmu:20226|seryl-tRNA synthetase [EC:6.1.1.11]	gi|148670021|gb|EDL01968.1|	0	seryl-aminoacyl-tRNA synthetase, isoform CRA_a [Mus musculus]
mmu-let-7d-3p	NM_001161782	-21.4	0.999998	NA	NA	GO:0043231,GO:0005859,GO:0008092,GO:0003712,GO:0046914,GO:0019787,GO:0032182,GO:0009896,GO:0016567,GO:00301	K10607|1|0.0|1239|mmu:69807|tripartite motif-containing protein 32 [EC:6.3.2.19]	gi|148699134|gb|EDL31081.1|	0	tripartite motif protein 32, isoform CRA_a [Mus musculus]

mmu-let-7d-3p	NM_001165992	-21.6	0.999952	NA	NA	GO:0043231,GO:0017111,GO:0032561,GO:0035556,GO:0048598,GO:0009207,	K07952|1|2e-113|411|rno:117051|ADP-ribosylation factor related protein 1	gi|148675460|gb|EDL07407.1|	1.08E-112	ADP-ribosylation factor related protein 1, isoform CRA_b [Mus musculus]
mmu-let-7d-3p	NR_028392	-20.3	1	NA	NA	GO:0016020,GO:0043231,GO:0031252,GO:0043232,GO:0044444,GO:0046914,GO:0005088,GO:0051020,GO:0007010,GO:0065008,GO:0030035,GO:0006917,GO:0032319,	K14800|1|8e-61|200|mmu:69499|pre-rRNA-processing protein TSR2!K05720|5|9e-54|213|mmu:14163|FYVE, RhoGEF and PH domain containing 1	gi|257196134|ref|NP_780355.2|	1.34E-61	pre-rRNA-processing protein TSR2 homolog isoform 2 [Mus musculus]

mmu-let-7d-3p	NM_029600	-24.4	0.995481	NA	NA	GO:0016021,GO:0044459,GO:0042626,GO:0008509,GO:0032559,GO:0015721,GO:0015893,GO:0043627,GO:0006820,GO:0009207,GO:0002237,GO:0008202,	K05667|1|0.0|3005|mmu:76408|ATP-binding cassette, subfamily C (CFTR/MRP), member 3	gi|90403595|ref|NP_083876.3|	0	canalicular multispecific organic anion transporter 2 [Mus musculus]
mmu-let-7d-3p	NM_138674	-22.5	1	NA	NA	GO:0044444,GO:0031224,GO:0044421,GO:0004871,GO:0002376,	K05099|1|1e-11|75.5|xla:373551|proto-oncogene tyrosine-protein kinase Met [EC:2.7.10.1]	gi|29135339|ref|NP_619615.2|	0	fibrocystin-L precursor [Mus musculus]
mmu-let-7d-3p	NM_001146707	-26.6	0.825991	NA	NA	GO:0005488,GO:0009987,	K11279|1|9e-124|446|mmu:53605|nucleosome assembly protein 1-like 1	gi|226443026|ref|NP_001140179.1|	6.40E-123	nucleosome assembly protein 1-like 1 isoform 1 precursor [Mus musculus]

mmu-let-7d-3p	NM_018734	-20.2	1	NA	NA	GO:0005774,GO:0031982,GO:0017111,GO:0032561,GO:0001562,GO:0042742,GO:0035456,GO:0051702,	-	gi|134053871|ref|NP_061204.3|	0	guanylate-binding protein 4 [Mus musculus]
mmu-let-7d-3p	NM_001079904	-22.3	0.999821	NA	NA	GO:0043231,GO:0046914,GO:0003676,GO:0006259,	K09228|1|3e-105|384|pon:100459001|KRAB domain-containing zinc finger protein	gi|347658946|ref|NP_001073373.2|	0	replication initiator 1 isoform d [Mus musculus]
mmu-let-7d-3p	NM_178029	-26.4	0.938181	NA	NA	GO:0044451,GO:0034708,GO:0005488,GO:0008168,	K11422|1|0.0|1335|mmu:233904|histone-lysine N-methyltransferase SETD1 [EC:2.1.1.43]	gi|119508422|ref|NP_821172.2|	0	SET domain containing 1A [Mus musculus]

mmu-let-7d-3p	NM_183428	-21.1	1	NA	NA	GO:0043231,GO:0030864,GO:0044425,GO:0032991,GO:0035091,GO:0008092,GO:0005198,GO:0043393,GO:0065008,GO:0003013,GO:0030036,	K06107|1|0.0|1343|hsa:2035|erythrocyte membrane protein band 4.1	gi|190684669|ref|NP_906273.3|	0	protein 4.1 isoform 1 [Mus musculus]
mmu-let-7d-3p	NM_026995	-25	0.692171	NA	NA	-	-	gi|298676450|ref|NP_081271.2|	0	ATP-dependent (S)-NAD(P)H-hydrate dehydratase isoform 1 [Mus musculus]
mmu-let-7d-3p	NR_045946	-20	1	NA	NA	GO:0048522,GO:0002684,	-	gi|74203689|dbj|BAE23092.1|	2.30E-48	unnamed protein product [Mus musculus]
mmu-let-7d-3p	NM_199028	-20.2	1	NA	NA	-	-	gi|39841055|ref|NP_950193.1|	0	BEN domain-containing protein 3 [Mus musculus]

mmu-let-7d-3p	NM_001114596	-20.2	1	NA	NA	-	K06115|1|1e-15|86.7|tsp:Tsp_02784|spectrin beta!K05699|2|3e-15|85.5|nve:NEMVE_v1g206855|actinin alpha	gi|167736349|ref|NP_001108068.1|	0	EH domain-binding protein 1-like protein 1 isoform D [Mus musculus]
mmu-let-7d-3p	NM_001164403	-22.8	0.999854	NA	NA	GO:0016021,GO:0044424,GO:0004725,GO:0005488,GO:0004871,GO:0048731,GO:0006464,GO:0006796,	K01104|1|0.0|884|mmu:19277|protein-tyrosine phosphatase [EC:3.1.3.48]	gi|257096042|ref|NP_001157873.1|	0	receptor-type tyrosine-protein phosphatase O isoform 2 precursor [Mus musculus]
mmu-let-7d-3p	NM_001253769	-22.4	0.999916	NA	NA	GO:0005911,GO:0043231,GO:0032991,GO:0005515,GO:0007010,	K06085|1|0.0|1149|mmu:99167|synovial sarcoma, X breakpoint 2 interactin	gi|148680030|gb|EDL11977.1|	0	synovial sarcoma, X breakpoint 2 interacting protein, isoform CRA_b [Mus musculus]

mmu-let-7d-5p	NM_001010941	-29.3	0.24472	-27.69	160	GO:0016021,GO:0045125,GO:0007166,GO:0006875,	K04315|1|3e-178|626|mmu:14738|G protein-coupled receptor 12	gi|6680061|ref|NP_032177.1|	2.06E-177	G-protein coupled receptor 12 [Mus musculus]
mmu-let-7d-5p	NM_001024504	-29.5	0.279789	-25.04	160	-	K10624|1|6e-40|167|ame:724670|E3 ubiquitin-protein ligase RBBP6 [EC:6.3.2.19]	gi|111162659|ref|NP_001019675.2|	1.35E-149	DCN1-like protein 2 isoform a [Mus musculus]
mmu-let-7d-5p	NM_001042649	-29.5	0.272312	-25.04	160	-	K10624|1|1e-31|94.4|ame:724670|E3 ubiquitin-protein ligase RBBP6 [EC:6.3.2.19]	gi|148690187|gb|EDL22134.1|	2.05E-133	mCG3700, isoform CRA_e [Mus musculus]

mmu-let-7d-5p	NM_001042650	-29.5	0.263472	-25.04	160	-	K10624|1|1e-17|93.2|ame:724670|E3 ubiquitin-protein ligase RBBP6 [EC:6.3.2.19]	gi|148690187|gb|EDL22134.1|	8.48E-138	mCG3700, isoform CRA_e [Mus musculus]
mmu-let-7d-5p	NM_001042651	-29.5	0.271043	-25.04	160	-	K10624|1|1e-24|109|ame:724670|E3 ubiquitin-protein ligase RBBP6 [EC:6.3.2.19]	gi|148690187|gb|EDL22134.1|	2.91E-132	mCG3700, isoform CRA_e [Mus musculus]
mmu-let-7d-5p	NM_001081089	-25.2	0.842787	-28.13	169	GO:0031966,GO:0008374,GO:0006071,GO:0006644,GO:0006641,	K00629|1|0.0|1393|mmu:215456|glycerol-3-phosphate O-acyltransferase 1/2 [EC:2.3.1.15]	gi|47847464|dbj|BAD21404.1|	0	mFLJ00202 protein [Mus musculus]
mmu-let-7d-5p	NM_001081235	-25.4	0.977207	-25.41	167	-	-	gi|124486985|ref|NP_001074704.1|	0	probable tumor suppressor protein MN1 [Mus musculus]

mmu-let-7d-5p	NM_001081347	-27.2	0.723363	-41.93	307	GO:0044444,GO:0016020,GO:0032561,GO:0035556,GO:0009966,	K07868|1|0.0|1371|mmu:69288|Rho-related BTB domain-containing protein 1/2	gi|124486809|ref|NP_001074816.1|	0	rho-related BTB domain-containing protein 1 isoform 1 [Mus musculus]
mmu-let-7d-5p	NM_001109992	-27.1	0.822699	-27.76	164	GO:0032991,GO:0032432,GO:0043231,GO:0044459,GO:0044444,GO:0051427,GO:0004725,GO:0019899,GO:0006639,GO:0006470,GO:0007169,GO:0035264,GO:0060123,GO:0046888,GO:0022414,GO:0006935,GO:0006974,GO:0007599,GO:0033500,GO:0001959,GO:0048667,GO:00343	K07293|1|0.0|1165|mmu:19247|protein tyrosine phosphatase, non-receptor type 11 [EC:3.1.3.48]	gi|158508568|ref|NP_001103462.1|	0	tyrosine-protein phosphatase non-receptor type 11 isoform b [Mus musculus]

mmu-let-7d-5p	NM_001110140	-31.7	0.18383	-27.23	163	GO:0032991,GO:0016021,GO:0005789,GO:0001664,GO:0019829,GO:0046872,GO:0032559,GO:0009888,GO:0007599,GO:0008016,GO:0006816,GO:0006936,GO:0009207,GO:0007165,GO:0001775,GO:0009206,GO:0009725,GO:0006875,	K05853|1|0.0|2035|mmu:11938|Ca2+ transporting ATPase, sarcoplasmic/endoplasmic reticulum [EC:3.6.3.8]	gi|60360584|dbj|BAD90532.1|	0	mKIAA4195 protein [Mus musculus]
mmu-let-7d-5p	NM_001114311	-29.2	0.736541	-25.45	165	GO:0001890,	-	gi|166851842|ref|NP_001107783.1|	0	storkhead-box protein 2 isoform 1 [Mus musculus]

mmu-let-7d-5p	NM_001159548	-26	0.94183	-47.69	317	GO:0043234,GO:0031981,GO:0046914,GO:0042802,GO:0001071,GO:0003677,GO:0004879,GO:0048869,GO:0009755,GO:00482	K08561|1|0.0|871|mmu:14536|germ cell nuclear factor	gi|227116316|ref|NP_001153020.1|	0	nuclear receptor subfamily 6 group A member 1 isoform 2 [Mus musculus]
mmu-let-7d-5p	NM_001159549	-26	0.941753	-47.69	317	GO:0043234,GO:0031981,GO:0046914,GO:0042802,GO:0001071,GO:0003677,GO:0004879,GO:0048869,GO:0009755,GO:00482	K08561|1|0.0|899|mmu:14536|germ cell nuclear factor	gi|227116318|ref|NP_001153021.1|	0	nuclear receptor subfamily 6 group A member 1 isoform 3 [Mus musculus]
mmu-let-7d-5p	NM_001160012	-31.3	0.077118	-27.29	176	GO:0005921,GO:0031224,GO:0005488,GO:0022829,GO:0043009,GO:0008544,GO:0050954,GO:0009987,	K07622|1|4e-136|485|mmu:14620|gap junction protein, beta 3	gi|6680013|ref|NP_032152.1|	3.12E-135	gap junction beta-3 protein [Mus musculus]

mmu-let-7d-5p	NM_001166625	-25.3	0.945705	-25.95	163	GO:0016021,GO:0004950,GO:0006952,GO:0051480,GO:0002376,GO:0007166,GO:00422	K04184|1|0.0|712|mmu:12769|C-C chemokine receptor type 9	gi|6753462|ref|NP_034043.1|	0	C-C chemokine receptor type 9 [Mus musculus]
mmu-let-7d-5p	NM_001174047	-26.9	0.843518	-25.36	169	GO:0034704,GO:0005262,GO:0043169,GO:0016247,GO:0015980,GO:0048741,GO:0006816,GO:0032412,GO:0045927,GO:0030073,GO:0050804,GO:0035264,	K04859|1|0.0|2153|mmu:56808|voltage-dependent calcium channel alpha-2/delta-2	gi|291290985|ref|NP_001167518.1|	0	voltage-dependent calcium channel subunit alpha-2/delta-2 isoform 1 precursor [Mus musculus]

mmu-let-7d-5p	NM_001174048	-26.9	0.842616	-25.36	169	GO:0034704,GO:0005262,GO:0043169,GO:0016247,GO:0015980,GO:0048741,GO:0006816,GO:0032412,GO:0045927,GO:0030073,GO:0050804,GO:0035264,	K04859|1|0.0|2126|mmu:56808|voltage-dependent calcium channel alpha-2/delta-2	gi|291290987|ref|NP_001167519.1|	0	voltage-dependent calcium channel subunit alpha-2/delta-2 isoform 3 precursor [Mus musculus]
mmu-let-7d-5p	NM_001174049	-26.9	0.842164	-25.36	169	GO:0034704,GO:0005262,GO:0043169,GO:0016247,GO:0015980,GO:0048741,GO:0006816,GO:0032412,GO:0045927,GO:0030073,GO:0050804,GO:0035264,	K04859|1|0.0|2118|mmu:56808|voltage-dependent calcium channel alpha-2/delta-2	gi|291290989|ref|NP_001167520.1|	0	voltage-dependent calcium channel subunit alpha-2/delta-2 isoform 4 precursor [Mus musculus]

mmu-let-7d-5p	NM_001174050	-26.9	0.842315	-25.36	169	GO:0034704,GO:0005262,GO:0043169,GO:0016247,GO:0015980,GO:0048741,GO:0006816,GO:0032412,GO:0045927,GO:0030073,GO:0050804,GO:0035264,	K04859|1|0.0|2114|mmu:56808|voltage-dependent calcium channel alpha-2/delta-2	gi|148689241|gb|EDL21188.1|	0	calcium channel, voltage-dependent, alpha 2/delta subunit 2, isoform CRA_b [Mus musculus]
mmu-let-7d-5p	NM_001190409	-29.6	0.385968	-25.31	171	GO:0005789,GO:0031224,GO:0019725,	K12669|1|1e-148|529|xla:494750|oligosaccharyltransferase complex subunit gamma	gi|298676496|ref|NP_080228.4|	0	magnesium transporter protein 1 [Mus musculus]
mmu-let-7d-5p	NM_001252636	-27.2	0.721956	-41.93	307	GO:0044444,GO:0016020,GO:0032561,GO:0035556,GO:0009966,	K07868|1|0.0|1371|mmu:69288|Rho-related BTB domain-containing protein 1/2	gi|124486809|ref|NP_001074816.1|	0	rho-related BTB domain-containing protein 1 isoform 1 [Mus musculus]

mmu-let-7d-5p	NM_007719	-28.6	0.252837	-26.06	163	GO:0009986,GO:0016021,GO:0004950,GO:0006952,GO:0051480,GO:0002376,GO:0007166,GO:0042221,	K04182|1|0.0|716|mmu:12775|C-C chemokine receptor type 7	gi|116268121|ref|NP_031745.2|	0	C-C chemokine receptor type 7 precursor [Mus musculus]
mmu-let-7d-5p	NM_008126	-31.3	0.06215	-27.29	176	GO:0005921,GO:0031224,GO:0005488,GO:0022829,GO:0043009,GO:0008544,GO:0050954,GO:0009987,	K07622|1|4e-136|485|mmu:14620|gap junction protein, beta 3	gi|6680013|ref|NP_032152.1|	2.54E-135	gap junction beta-3 protein [Mus musculus]
mmu-let-7d-5p	NM_008151	-29.3	0.22259	-27.69	160	GO:0016021,GO:0045125,GO:0007166,GO:0006875,	K04315|1|3e-178|626|mmu:14738|G protein-coupled receptor 12	gi|6680061|ref|NP_032177.1|	1.86E-177	G-protein coupled receptor 12 [Mus musculus]
mmu-let-7d-5p	NM_008480	-25.3	0.996741	-45.67	298	-	K05637|1|0.0|6084|mmu:16772|laminin, alpha 1/2	gi|117168301|ref|NP_032506.2|	0	laminin subunit alpha-1 precursor [Mus musculus]

mmu-let-7d-5p	NM_008824	-32	0.050045	-26.81	176	GO:0043234,GO:0044444,GO:0070061,GO:0008443,GO:0019899,GO:0019203,GO:0032559,GO:0006007,GO:0035556,GO:0033131,GO:0009887,GO:0043434,GO:0031960,GO:0015980,GO:0006000,GO:00069	K01103|1|0.0|910|mmu:18639|6-phosphofructo-2-kinase / fructose-2,6-bisphosphatase [EC:2.7.1.105 3.1.3.46]	gi|7434494|pir||S77704	0	6-phosphofructo-2-kinase (EC 2.7.1.105) / fructose-2, 6-bisphosphate 2-phosphatase (EC 3.1.3.46) clone 5c, skeletal muscle - rat
mmu-let-7d-5p	NM_009913	-28.2	0.568383	-25.95	163	GO:0016021,GO:0004950,GO:0006952,GO:0051480,GO:0002376,GO:0007166,GO:00422	K04184|1|0.0|712|mmu:12769|C-C chemokine receptor type 9	gi|6753462|ref|NP_034043.1|	0	C-C chemokine receptor type 9 [Mus musculus]

mmu-let-7d-5p	NM_010264	-26	0.949176	-47.69	317	GO:0043234,GO:0031981,GO:0046914,GO:0042802,GO:0001071,GO:0003677,GO:0004879,GO:0048869,GO:0009755,GO:00482	K08561|1|0.0|892|ssc:100038028|germ cell nuclear factor	gi|6753956|ref|NP_034394.1|	0	nuclear receptor subfamily 6 group A member 1 isoform 1 [Mus musculus]
mmu-let-7d-5p	NM_010473	-29.6	0.213813	-27.01	165	GO:0005789,GO:0044057,	-	gi|133778931|ref|NP_034603.2|	0	histidine rich calcium binding protein precursor [Mus musculus]
mmu-let-7d-5p	NM_010489	-28.4	0.258519	-25.41	162	GO:0031224,GO:0000323,GO:0005488,GO:0004871,GO:0015929,GO:0023052,GO:0030260,GO:0019048,GO:0006027,	K01197|1|0.0|998|mmu:15587|hyaluronoglucosaminidase [EC:3.2.1.35]	gi|45331202|ref|NP_034619.2|	0	hyaluronidase-2 precursor [Mus musculus]

mmu-let-7d-5p	NM_010577	-30.1	0.317962	-26.5	165	GO:0044424,GO:0009986,GO:0001726,GO:0005924,GO:0043234,GO:0044421,GO:0005887,GO:0004871,GO:0005172,GO:0001934,GO:0007166,GO:0007599,GO:0007611,GO:0016337,GO:0010810,GO:0034329,GO:0044319,GO:0009967,GO:0006935,GO:0002376,GO:0051704,GO:00486	K06484|1|0.0|1961|mmu:16402|integrin alpha 5	gi|148671975|gb|EDL03922.1|	0	integrin alpha 5 (fibronectin receptor alpha), isoform CRA_b [Mus musculus]

mmu-let-7d-5p	NM_011202	-27.1	0.823325	-27.76	164	GO:0032991,GO:0032432,GO:0043231,GO:0044459,GO:0044444,GO:0051427,GO:0004725,GO:0019899,GO:0006639,GO:0006470,GO:0007169,GO:0035264,GO:0060123,GO:0046888,GO:0022414,GO:0006935,GO:0006974,GO:0007599,GO:0033500,GO:0001959,GO:0048667,GO:00343	K07293|1|0.0|1164|rno:25622|protein tyrosine phosphatase, non-receptor type 11 [EC:3.1.3.48]	gi|6755228|ref|NP_035332.1|	0	tyrosine-protein phosphatase non-receptor type 11 isoform a [Mus musculus]
mmu-let-7d-5p	NM_011426	-27.7	0.785037	-65.69	459	GO:0031224,GO:0030246,GO:0006952,GO:0031589,GO:0016192,	K06548|1|0.0|3231|mmu:20612|sialoadhesin	gi|226958332|ref|NP_035556.3|	0	sialoadhesin precursor [Mus musculus]

mmu-let-7d-5p	NM_017471	-28.5	0.088682	-26.23	169	GO:0043231,GO:0005488,GO:0051234,	-	gi|8567372|ref|NP_059499.1|	1.57E-95	probasin precursor [Mus musculus]
mmu-let-7d-5p	NM_020263	-26.9	0.843218	-25.36	169	GO:0034704,GO:0005262,GO:0043169,GO:0016247,GO:0015980,GO:0048741,GO:0006816,GO:0032412,GO:0045927,GO:0030073,GO:0050804,GO:0035264,	K04859|1|0.0|2141|mmu:56808|voltage-dependent calcium channel alpha-2/delta-2	gi|161353447|ref|NP_064659.2|	0	voltage-dependent calcium channel subunit alpha-2/delta-2 isoform 2 precursor [Mus musculus]
mmu-let-7d-5p	NM_021463	-29.9	0.151054	-26.46	167	GO:0032991,GO:0044444,GO:0016778,GO:0046872,GO:0042802,GO:0032561,GO:0030246,GO:0032559,GO:0006220,GO:0006796,GO:0009887,GO:0046390,GO:0009168,GO:00	K00948|1|4e-179|629|ssc:100517744|ribose-phosphate pyrophosphokinase [EC:2.7.6.1]	gi|118137847|pdb|2H06|A	2.45E-178	Chain A, Crystal Structure Of Human Phosphoribosyl Pyrophosphate Synthetase 1

mmu-let-7d-5p	NM_022887	-27.4	0.87209	-47.58	327	GO:0044446,GO:0030427,GO:0031252,GO:0043234,GO:0015629,GO:0044444,GO:0060589,GO:0005515,GO:0014020,GO:0032321,GO:0001932,GO:0043434,GO:0032231,GO:0042254,GO:0043062,GO:0007169,GO:0021543,GO:0016567,GO:0022402,GO:0030001,GO:0008366,GO:00083	K07206|1|0.0|1951|mmu:64930|tuberous sclerosis 1	gi|79750409|ref|NP_075025.2|	0	hamartin [Mus musculus]
mmu-let-7d-5p	NM_023229	-31.6	0.065426	-26.67	161	GO:0043231,GO:0004674,GO:0032559,GO:0006917,GO:0006796,GO:0010468,GO:0006464,	K08290|1|0.0|986|mmu:66587|Fas-activated serine/threonine kinase [EC:2.7.11.8]	gi|85861160|ref|NP_075718.2|	0	fas-activated serine/threonine kinase [Mus musculus]

mmu-let-7d-5p	NM_024203	-29.3	0.426298	-26.51	154	GO:0043231,GO:0010467,GO:0048869,	-	gi|51988904|ref|NP_077165.2|	0	constitutive coactivator of peroxisome proliferator-activated receptor gamma [Mus musculus]
mmu-let-7d-5p	NM_025557	-28.8	0.181608	-26.3	170	-	-	gi|12833000|dbj|BAB22346.1|	1.23E-49	unnamed protein product [Mus musculus]
mmu-let-7d-5p	NM_025952	-29.6	0.355743	-25.31	171	GO:0005789,GO:0031224,GO:0019725,	K12669|1|9e-149|529|xla:494750|oligosaccharyltransferase complex subunit gamma	gi|298676496|ref|NP_080228.4|	0	magnesium transporter protein 1 [Mus musculus]
mmu-let-7d-5p	NM_026566	-30.3	0.068513	-26.49	159	GO:0044444,GO:0005515,GO:0044248,GO:0007033,	-	gi|21311929|ref|NP_080842.1|	5.54E-121	autophagy-related protein 101 [Mus musculus]
mmu-let-7d-5p	NM_080437	-27.7	0.927539	-43.58	312	GO:0031224,GO:0046872,GO:0004888,GO:0007409,GO:0016337,GO:0007186,	K04602|1|0.0|5914|mmu:107934|cadherin EGF LAG seven-pass G-type receptor 3 (flamingo)	gi|125719165|ref|NP_536685.2|	0	cadherin EGF LAG seven-pass G-type receptor 3 precursor [Mus musculus]

mmu-let-7d-5p	NM_145401	-25.2	0.903869	-43.67	316	GO:0031981,GO:0043234,GO:0044444,GO:0030291,GO:0032559,GO:0019900,GO:0001934,GO:0033674,GO:0015980,GO:0033673,GO:0006073,GO:0010827,GO:0006694,GO:0006633,GO:0007169,GO:0006096,GO:0003333,GO:0022402,GO:0019217,GO:0009206,GO	K07200|1|0.0|832|ptr:463879|5'-AMP-activated protein kinase, regulatory gamma subunit	gi|282847325|ref|NP_663376.2|	0	5'-AMP-activated protein kinase subunit gamma-2 isoform 1 [Mus musculus]
mmu-let-7d-5p	NM_146155	-29.7	0.501695	-74.76	486	-	K02350|1|2e-08|63.5|tgu:100217592|DNA polymerase zeta subunit [EC:2.7.7.7]	gi|148698133|gb|EDL30080.1|	0	AT hook, DNA binding motif, containing 1, isoform CRA_b [Mus musculus]

mmu-let-7d-5p	NM_153459	-25.4	0.874558	-26.3	164	GO:0031981,GO:0044444,GO:0005488,GO:0008138,GO:0006470,GO:0000165,GO:0002224,GO:0007169,GO:0043405,	K04459|1|0.0|728|mmu:235584|dual specificity phosphatase [EC:3.1.3.16 3.1.3.48]	gi|238231443|ref|NP_703189.3|	0	dual specificity protein phosphatase 7 [Mus musculus]
mmu-let-7d-5p	NM_172371	-26.7	0.597486	-44.75	311	GO:0031224,GO:0015291,GO:0006810,	K08189|1|0.0|729|mmu:69309|MFS transporter, MCP family, solute carrier family 16 (monocarboxylic acid transporters), member 13	gi|29336097|ref|NP_758959.1|	0	monocarboxylate transporter 13 [Mus musculus]

mmu-let-7d-5p	NM_172715	-34.6	0.031985	-29.2	171	GO:0031090,GO:0043231,GO:0031224,GO:0008374,GO:0006644,GO:0006641,GO:0009966,	K13506|1|0.0|857|mmu:231510|glycerol-3-phosphate O-acyltransferase 3/4 [EC:2.3.1.15]	gi|27370046|ref|NP_766303.1|	0	glycerol-3-phosphate acyltransferase 3 [Mus musculus]
mmu-let-7d-5p	NM_172716	-32.3	0.116365	-26.29	166	GO:0046914,GO:0010467,	K11488|1|2e-144|514|ptr:461045|polycomb group RING finger protein 3	gi|148688164|gb|EDL20111.1|	3.18E-148	polycomb group ring finger 3, isoform CRA_c [Mus musculus]
mmu-let-7d-5p	NM_172784	-26.5	0.747086	-42.22	310	GO:0031224,GO:0004871,GO:0023052,	K15619|1|3e-30|135|tgu:100226787|Kunitz-type protease inhibitor 1	gi|31542055|ref|NP_766372.1|	0	low-density lipoprotein receptor-related protein 11 precursor [Mus musculus]
mmu-let-7d-5p	NM_172887	-26	0.991794	-42.96	321	GO:0031224,	-	gi|255069717|ref|NP_766475.2|	0	furry homolog [Mus musculus]
mmu-let-7d-5p	NM_175162	-28.4	0.806554	-25.45	165	GO:0001890,	-	gi|166851842|ref|NP_001107783.1|	0	storkhead-box protein 2 isoform 1 [Mus musculus]

mmu-let-7d-5p	NM_177030	-27.6	0.805457	-25.26	159	GO:0044444,GO:0032561,GO:0030695,GO:0019899,GO:0007599,	K05727|1|9e-13|78.2|nvi:100118634|dedicator of cytokinesis 3/4/5!K13708|5|2e-11|73.9|phu:Phum_PHUM252200|dedicator of cytokinesis 1	gi|124301208|ref|NP_796004.2|	0	dedicator of cytokinesis protein 6 [Mus musculus]
mmu-let-7d-5p	NM_177243	-28.5	0.459606	-66.69	476	GO:0044459,GO:0031224,GO:0015291,GO:0005253,GO:0015103,GO:0015698,GO:0055067,	K14706|1|0.0|1545|mmu:320718|solute carrier family 26 (sulfate anion transporter), member 9	gi|154090973|ref|NP_796217.2|	0	solute carrier family 26 member 9 [Mus musculus]
mmu-let-7d-5p	NM_178923	-31.5	0.181452	-30.6	172	GO:0031981,GO:0005488,	K13167|1|2e-145|518|mmu:224432|splicing factor, arginine/serine-rich 15	gi|148665971|gb|EDK98387.1|	1.51E-146	mCG129573, isoform CRA_b [Mus musculus]

mmu-let-7d-5p	NM_181729	-26.4	0.965838	-89.86	642	-	K13908|1|0.0|868|ecb:100147340|mucin-5B	gi|32171192|ref|NP_859418.1|	0	mucin-6 precursor [Mus musculus]
mmu-let-7d-5p	NM_207671	-27.8	0.83061	-26.32	170	GO:0031981,GO:0046914,GO:0022403,GO:0010467,	-	gi|157823950|ref|NP_997554.2|	0	zinc finger protein 318 isoform 1 [Mus musculus]
mmu-let-7d-5p	NR_003248	-26	0.84264	-25.13	159	-	-	gi|74228501|dbj|BAE25354.1|	1.79E-42	unnamed protein product [Mus musculus]
mmu-let-7d-5p	NR_027651	-27.1	0.962567	-27.83	168	-	-	gi|148686746|gb|EDL18693.1|	5.00E-78	GTL2, imprinted maternally expressed untranslated mRNA [Mus musculus]
mmu-let-7d-5p	NR_027652	-31.7	0.066383	-27.83	168	-	-	gi|148686746|gb|EDL18693.1|	1.84E-74	GTL2, imprinted maternally expressed untranslated mRNA [Mus musculus]

mmu-let-7d-5p	NR_027838	-31.7	0.232647	-27.23	163	GO:0032991,GO:0016021,GO:0005789,GO:0001664,GO:0019829,GO:0046872,GO:0032559,GO:0009888,GO:0007599,GO:0008016,GO:0006816,GO:0006936,GO:0009207,GO:0007165,GO:0001775,GO:0009206,GO:0009725,GO:0006875,	K05853|1|0.0|2035|mmu:11938|Ca2+ transporting ATPase, sarcoplasmic/endoplasmic reticulum [EC:3.6.3.8]	gi|60360584|dbj|BAD90532.1|	0	mKIAA4195 protein [Mus musculus]
mmu-let-7d-5p	NR_033586	-29.3	0.514623	-26.51	154	-	-	gi|54887404|gb|AAH85163.1|	0	Fam120b protein [Mus musculus]
mmu-let-7d-5p	NR_046475	-31.7	0.070187	-27.83	168	-	-	-	-	-

mmu-let-7d-5p	NM_148948	-21.8	1	-67.21	486	GO:0031332,GO:0044444,GO:0016891,GO:0032559,GO:0043169,GO:0003723,GO:0042623,GO:0016246,GO:0006357,GO:0031641,GO:0022011,GO:0010624,GO:0048858,GO:00	K11592|1|0.0|3661|mmu:192119|endoribonuclease Dicer [EC:3.1.26.-]	gi|148686840|gb|EDL18787.1|	0	Dicer1, Dcr-1 homolog (Drosophila), isoform CRA_a [Mus musculus]
mmu-let-7d-5p	NM_145908	-23.9	0.904759	-64.06	458	GO:0031227,GO:0016747,GO:0005488,GO:0044260,GO:0007166,	K00181|1|0.0|855|rno:317368|porcupine	gi|148702018|gb|EDL33965.1|	0	porcupine homolog (Drosophila), isoform CRA_b [Mus musculus]

mmu-let-7d-5p	NM_007698	-24.7	0.993902	-43.55	331	GO:0044430,GO:0016021,GO:0033267,GO:0044459,GO:0045202,GO:0004629,GO:0016907,GO:0048731,GO:0007200,GO:0008283,GO:0043412,GO:0007268,	K04129|1|0.0|738|mmu:12669|muscarinic acetylcholine receptor M1	gi|31542388|ref|NP_031724.2|	0	muscarinic acetylcholine receptor M1 [Mus musculus]

mmu-let-7d-5p	NM_007949	-24.6	0.959873	-45.37	329	GO:0005667,GO:0005819,GO:0004674,GO:0003678,GO:0032559,GO:0042623,GO:0005515,GO:0003676,GO:0051540,GO:0043169,GO:0051704,GO:0006915,GO:0006357,GO:0022010,GO:0043009,GO:0043062,GO:0045935,GO:0009207,GO:0001503,GO:0048863,GO:0022405,GO:00094	K10844|1|0.0|1469|mmu:13871|DNA excision repair protein ERCC-2 [EC:3.6.4.12]	gi|31542614|ref|NP_031975.2|	0	TFIIH basal transcription factor complex helicase XPD subunit [Mus musculus]

mmu-let-7d-5p	NM_026298	-22.7	0.999971	-50.49	327	GO:0005815,GO:0043231,GO:0005515,GO:0003002,GO:0006357,GO:0010927,GO:0001838,GO:0051604,GO:0009855,GO:00071	-	gi|148705414|gb|EDL37361.1|	0	intraflagellar transport 172 homolog (Chlamydomonas), isoform CRA_a [Mus musculus]
mmu-let-7d-5p	NM_028785	-23.1	0.999992	-44.15	322	GO:0044444,GO:0032561,GO:0030695,GO:0019899,GO:0007599,	K05727|1|2e-13|80.5|nvi:100118634|dedicator of cytokinesis 3/4/5!K13708|2|4e-13|79.7|tca:661070|dedicator of cytokinesis 1	gi|62241030|ref|NP_083061.2|	0	dedicator of cytokinesis protein 8 [Mus musculus]

mmu-let-7d-5p	NM_199469	-22.2	0.999986	-47.23	321	GO:0043231,GO:0044444,GO:0016020,GO:0046914,GO:0005515,GO:0016044,GO:0043161,GO:0006996,	K14015|1|0.0|1139|mmu:217365|nuclear protein localization protein 4 homolog	gi|148702796|gb|EDL34743.1|		0	nuclear protein localization 4 homolog (S. cerevisiae), isoform CRA_a [Mus musculus]
mmu-let-7d-5p	NM_010026	-22.4	0.999968	-41.64	320	GO:0044424,GO:0030674,GO:0005083,GO:0008047,GO:0046914,GO:0019904,GO:0030035,GO:0048858,GO:0043087,GO:00320	K12488|1|0.0|1808|mmu:13196|Arf-GAP with SH3 domain, ANK repeat and PH domain-containing protein	gi|65301464|ref|NP_034156.2|		0	arf-GAP with SH3 domain, ANK repeat and PH domain-containing protein 1 [Mus musculus]
mmu-let-7d-5p	NM_207298	-20.7	0.999999	-42.56	314	GO:0044432,GO:0016020,GO:0016740,GO:0016337,GO:0008610,	K11703|1|0.0|651|ssc:100517457|collagen beta-1,O-galactosyltransferase [EC:2.4.1.50]	gi|46411176|ref|NP_997181.1|		0	glycosyltransferase 25 family member 3 precursor [Mus musculus]

mmu-let-7d-5p	NM_153407	-23.5	0.997286	-42.51	312	GO:0043231,GO:0003676,GO:0001071,GO:0012501,GO:0006357,	-	gi|23510287|ref|NP_700456.1|	0	cysteine/serine-rich nuclear protein 2 [Mus musculus]
mmu-let-7d-5p	NM_025809	-23.5	0.998371	-43.83	309	GO:0030246,	K06702|1|1e-25|120|gga:421234|CDW93 antigen!K06706|2|4e-25|118|ssc:100523587|CD248 antigen, endosialin	gi|148704745|gb|EDL36692.1|	0	C-type lectin domain family 14, member a [Mus musculus]
mmu-let-7d-5p	NM_001252638	-23.1	0.999465	-41.93	307	GO:0044444,GO:0016020,GO:0032561,GO:0035556,GO:0009966,	K07868|1|0.0|1167|mmu:69288|Rho-related BTB domain-containing protein 1/2	gi|124486809|ref|NP_001074816.1|	0	rho-related BTB domain-containing protein 1 isoform 1 [Mus musculus]
mmu-let-7d-5p	NM_172885	-24.1	0.991163	-41.36	306	GO:0031224,	-	gi|148687591|gb|EDL19538.1|	0	transmembrane protein 132D [Mus musculus]

mmu-let-7d-5p	NM_194333	-20.8	0.999975	-45.13	302	GO:0031224,GO:0005488,GO:0006810,	K14612|1|0.0|1042|mmu:22626|solute carrier family 23 (nucleobase transporter), member 3	gi|148667947|gb|EDL00364.1|	0	solute carrier family 23 (nucleobase transporters), member 3 [Mus musculus]
mmu-let-7d-5p	NM_008305	-24.3	0.99999	-41.62	300	GO:0005604,GO:0016020,GO:0005515,GO:0007417,GO:0043062,GO:0001503,GO:0044238,GO:0051216,GO:0048646,GO:0044260,GO:0048562,GO:0030154,GO:0014706,GO:00	K06255|1|0.0|8774|mmu:15530|heparan sulfate proteoglycan 2 (perlecan)	gi|183979966|ref|NP_032331.2|	0	basement membrane-specific heparan sulfate proteoglycan core protein precursor [Mus musculus]
mmu-let-7d-5p	NR_038041	-23.5	0.405861	-33.34	184	-	-	gi|148700034|gb|EDL31981.1|	1.62E-60	mCG146286 [Mus musculus]

mmu-let-7d-5p	NM_026184	-23.6	0.997027	-30.44	180	GO:0031090,GO:0043231,GO:0016667,GO:0005515,GO:0050662,GO:0051234,GO:0006457,GO:0006091,	K10976|1|0.0|881|mmu:67475|ERO1-like protein beta [EC:1.8.4.-]	gi|38348230|ref|NP_080460.2|	0	ERO1-like protein beta precursor [Mus musculus]
mmu-let-7d-5p	NM_177638	-24.4	0.66718	-32.17	179	GO:0043296,GO:0031224,GO:0019904,GO:0007009,GO:0045216,GO:0034329,	K06090|1|4e-50|199|mmu:224912|crumbs 3	gi|29244038|ref|NP_808306.1|	3.06E-49	crumbs protein homolog 3 precursor [Mus musculus]
mmu-let-7d-5p	NM_177034	-22.6	0.999997	-27.85	176	GO:0032991,GO:0030136,GO:0016020,GO:0035091,GO:0019904,GO:0048731,GO:0043009,GO:0040007,GO:0023061,GO:0006887,GO:0010467,GO:0007610,GO:0065003,GO:0015031,GO:0010970,	K04531|1|0.0|1538|mmu:319924|amyloid beta (A4) precursor protein-binding, family A, member 1 (X11)	gi|187954913|gb|AAI41182.1|	0	Apba1 protein [Mus musculus]

mmu-let-7d-5p	NM_201368	-24.6	0.977462	-27.03	174	GO:0016020,	-	gi|41235770|ref|NP_958756.1|	0	XK-related protein 8 [Mus musculus]
mmu-let-7d-5p	NM_029933	-21.2	1	-25.89	173	GO:0005488,GO:0007179,	K06236|1|5e-12|57.8|aml:100472350|collagen, type I/II/III/V/XI, alpha!K06237|4|3e-08|62.4|hsa:1287|collagen, type IV, alpha	gi|56606137|ref|NP_084209.3|	0	B-cell CLL/lymphoma 9 protein [Mus musculus]
mmu-let-7d-5p	NR_040398	-30.8	0.173677	-24.1	172	-	-	gi|149026310|gb|EDL82553.1|	5.64E-10	rCG63030 [Rattus norvegicus]
mmu-let-7d-5p	NM_198703	-20.3	1	-27.47	171	GO:0044424,GO:0004860,GO:0004672,GO:0019900,GO:0046872,GO:0008200,GO:0032559,GO:0006810,GO:0035556,GO:0006469,GO:0048468,GO:0003073,	K08867|1|0.0|2065|ptr:451739|WNK lysine deficient protein kinase [EC:2.7.11.1]	gi|148667213|gb|EDK99629.1|	0	WNK lysine deficient protein kinase 1, isoform CRA_b [Mus musculus]

mmu-let-7d-5p	NM_172641	-25.9	0.705064	-20.45	170	-	-	gi|148669812|gb|EDL01759.1|	0	RIKEN cDNA 9930023K05, isoform CRA_c [Mus musculus]
mmu-let-7d-5p	NM_173451	-29.2	0.370218	-24.27	169	GO:0016788,GO:0043169,	K12375|1|0.0|1174|mmu:271970|arylsulfatase I/J [EC:3.1.6.-]	gi|27734088|ref|NP_775627.1|	0	arylsulfatase J precursor [Mus musculus]
mmu-let-7d-5p	NM_178691	-24.7	0.963872	-25.2	168	GO:0044464,GO:0046914,GO:0019783,GO:0043161,GO:0019752,GO:0016579,GO:0006950,GO:0006984,	K13719|1|0.0|701|mmu:226418|ubiquitin thioesterase OTU1 [EC:3.1.2.-]	gi|148707790|gb|EDL39737.1|	0	mCG2637 [Mus musculus]
mmu-let-7d-5p	NM_178066	-22.3	0.997659	-27.68	167	-	-	gi|30017375|ref|NP_835167.1|	1.04E-130	uncharacterized protein LOC73827 [Mus musculus]

mmu-let-7d-5p	NR_001461	-23.4	1	-23.18	166	-	K11849|1|6e-55|213|acs:100555596|ubiquitin carboxyl-terminal hydrolase 25/28 [EC:3.1.2.15]!K06478|4|4e-43|182|acs:100563672|protein tyrosine phosphatase, receptor type, C [EC:3.1.3.4	gi|209553980|gb|AAA39398.2|	0	ORF2 [Mus musculus domesticus]
mmu-let-7d-5p	NR_033617	-27.6	0.423166	-22.78	165	-	-	gi|148698724|gb|EDL30671.1|	1.48E-89	mCG144794 [Mus musculus]
mmu-let-7d-5p	NR_036449	-22.3	0.999847	-20.14	164	-	-	gi|148709350|gb|EDL41296.1|	2.83E-61	hypothetical protein A030007L22 [Mus musculus]
mmu-let-7d-5p	NR_026733	-25	0.441337	-20.47	163	-	-	gi|148704847|gb|EDL36794.1|	1.03E-50	mCG5478 [Mus musculus]
mmu-let-7d-5p	NR_045831	-24.7	0.741646	-20.28	162	-	-	-	-	-

mmu-let-7d-5p	NR_037992	-27.2	0.725827	-20.32	161	-	K11323|1|2e-09|66.6|nvi:100116682|histone arginine demethylase JMJD6 [EC:1.14.1	gi|30520031|ref|NP_848774.1|	0	jmjC domain-containing protein 4 isoform 1 [Mus musculus]
mmu-let-7d-5p	NR_033398	-26.4	0.590961	-23.6	160	-	K11290|1|7e-06|53.5|rno:317165|template-activating factor I	gi|82793879|ref|XP_728215.1|	1.64E-17	hypothetical protein [Plasmodium yoelii yoelii 17XNL]
mmu-let-7d-5p	NR_046162	-23.3	0.981764	-21.92	159	-	-	gi|148665186|gb|EDK97602.1|	7.08E-114	mCG144829 [Mus musculus]
mmu-let-7d-5p	NR_045888	-20.4	0.999999	-20.55	158	-	-	gi|148687022|gb|EDL18969.1|	2.86E-77	mCG144675 [Mus musculus]
mmu-let-7d-5p	NR_046142	-21	1	-22.29	157	-	-	gi|148709348|gb|EDL41294.1|	1.58E-30	mCG145641 [Mus musculus]
mmu-let-7e-3p	NM_001081383	-29.2	0.917256	-27.42	157	GO:0043231,GO:0046872,GO:0008168,GO:0050794,	K09188|1|0.0|8511|mmu:231051|histone-lysine N-methyltransferase MLL3 [EC:2.1.1.43]	gi|124487063|ref|NP_001074852.1|	0	histone-lysine N-methyltransferase MLL3 [Mus musculus]

mmu-let-7e-3p	NM_001165965	-32.5	0.186319	-26.7	179	-	K06252|1|5e-12|75.1|spu:576745|tenascin!K05717|3|1e-07|60.5|xtr:595087|fibronectin 1!K05698|4|2e-06|56.6|mdo:100619665|protein tyrosine phosphatase, receptor type, J [EC:3.1.3.48]	gi|260166644|ref|NP_001159437.1|	0	fibronectin type III domain-containing protein 7 isoform 2 precursor [Mus musculus]

mmu-let-7e-3p	NM_009157	-33.4	0.097614	-27.87	167	GO:0043231,GO:0030425,GO:0043025,GO:0030424,GO:0044444,GO:0004708,GO:0019901,GO:0032559,GO:0007166,GO:0006275,GO:0009612,GO:0002224,GO:0000187,GO:00	K04430|1|0.0|752|rno:287398|mitogen-activated protein kinase kinase 4 [EC:2.7.12.2]	gi|22095023|ref|NP_033183.1|	0	dual specificity mitogen-activated protein kinase kinase 4 [Mus musculus]
mmu-let-7e-3p	NM_153589	-31.8	0.194475	-31.59	168	GO:0034702,GO:0005488,GO:0005254,GO:0015698,	K13982|1|8e-32|140|dgr:Dgri_GH10918|probable ATP-dependent RNA helicase DDX4 [EC:3.6.4.13]!K05169|2|3e-25|118|ptr:743996|single Ig IL-1R-related molecule	gi|209862776|ref|NP_705817.2|	0	anoctamin-2 [Mus musculus]

mmu-let-7e-3p	NM_175324	-29.6	0.3618	-26.93	163	GO:0042579,GO:0042995,GO:0016627,GO:0016740,GO:0050662,GO:0045596,GO:0030178,GO:0043954,GO:0008152,	K11730|1|0.0|1579|mmu:102632|acyl-CoA dehydrogenase family member 11	gi|74271799|ref|NP_780533.2|	0	acyl-CoA dehydrogenase family member 11 [Mus musculus]
mmu-let-7e-3p	NM_177091	-32.5	0.196407	-26.7	179	-	K06252|1|2e-13|80.1|spu:576745|tenascin!K05717|5|2e-08|63.2|acs:100563512|fibronectin 1	gi|260166640|ref|NP_796065.2|	0	fibronectin type III domain-containing protein 7 isoform 1 precursor [Mus musculus]
mmu-let-7e-3p	NR_029916	-26.8	0.005517	-26.69	172	-	-	-	-	-

mmu-let-7e-3p	NM_001045540	-23.6	0.999731	-20.28	176	GO:0044464,GO:0016787,	K14139|1|2e-42|176|cfa:481473|immunity-related GTPase family M protein [EC:3.6.5.-]!K14140|3|3e-39|165|rno:303163|interferon gamma induced GTPase	gi|113865923|ref|NP_001039005.1|	0	uncharacterized protein LOC620913 [Mus musculus]
mmu-let-7e-3p	NM_019924	-24.2	0.983026	-21.47	170	GO:0031981,GO:0004674,GO:0046872,GO:0051019,GO:0032559,GO:0035556,GO:0006796,GO:0006935,GO:0006351,GO:00064	K04445|1|0.0|1434|mmu:56613|mitogen-,stress activated protein kinases [EC:2.7.11.1]	gi|9910454|ref|NP_064308.1|	0	ribosomal protein S6 kinase alpha-4 [Mus musculus]

mmu-let-7e-3p	NR_001461	-25.9	1	-23.36	168	-	K11849|1|6e-55|213|acs:100555596|ubiquitin carboxyl-terminal hydrolase 25/28 [EC:3.1.2.15]!K06478|4|4e-43|182|acs:100563672|protein tyrosine phosphatase, receptor type, C [EC:3.1.3.4	gi|209553980|gb|AAA39398.2|	0	ORF2 [Mus musculus domesticus]
mmu-let-7e-3p	NM_033074	-28.4	0.43635	-24.44	165	GO:0044444,GO:0042802,GO:0004812,GO:0032559,GO:0006418,	K01868|1|0.0|1437|mmu:110960|threonyl-tRNA synthetase [EC:6.1.1.3]	gi|27229277|ref|NP_149065.2|	0	threonine--tRNA ligase, cytoplasmic [Mus musculus]

mmu-let-7e-3p	NM_001081091	-27.6	0.831582	-22.42	164	GO:0044444,	K14692|1|6e-20|101|smm:Smp_170590|solute carrier family 30 (zinc transporter), member 5!K12478|2|8e-15|84.7|oaa:100079644|early endosome antigen 1!K10352|3|8e-15|84.7|pon:100450821|myosin heavy chain	gi|50510705|dbj|BAD32338.1|	0	mKIAA0912 protein [Mus musculus]
mmu-let-7e-3p	NR_045063	-22.7	0.998077	-20.2	162	-	-	gi|148671346|gb|EDL03293.1|	8.39E-22	mCG141159, isoform CRA_c [Mus musculus]
mmu-let-7e-3p	NM_026010	-26.4	0.441855	-21.15	161	GO:0043231,GO:0005488,GO:0006633,GO:0043412,	K03801|1|2e-125|449|mmu:67164|lipoyl(octanoyl) transferase [EC:2.3.1.181]	gi|109731836|gb|AAI15473.1|	1.09E-124	RIKEN cDNA 2610209A20 gene [Mus musculus]

mmu-let-7e-3p	NM_001034880	-27.1	0.504241	-24.54	160	-	-	gi|85702223|ref|NP_001030052.1|	2.63E-73	uncharacterized protein LOC432838 [Mus musculus]
mmu-let-7e-3p	NM_172477	-21.2	1	-20.39	158	-	K12861|1|8e-159|562|gga:419884|pre-mRNA-splicing factor SPF27!K01289|2|7e-16|87.8|dme:Dmel_CG18659|carboxypeptidase [EC:3.4.16.	gi|148681657|gb|EDL13604.1|	0	DENN/MADD domain containing 2A, isoform CRA_c [Mus musculus]
mmu-let-7e-3p	NM_172592	-23.8	0.997747	-20.31	157	GO:0005488,	K13165|1|3e-102|374|bta:507801|splicing factor, arginine/serine-rich 12	gi|148686563|gb|EDL18510.1|	5.43E-109	splicing factor, arginine/serine-rich 12, isoform CRA_a [Mus musculus]

mmu-let-7e-3p	NM_016763	-24.8	0.588852	-23.98	156	GO:0043231,GO:0019866,GO:0033764,GO:0008289,GO:0035258,GO:0000166,GO:0051260,GO:0006399,GO:0007568,GO:0003006,	K08683|1|4e-130|464|mmu:15108|3-hydroxyacyl-CoA dehydrogenase / 3-hydroxy-2-methylbutyryl-CoA dehydrogenase [EC:1.1.1.35 1.1.1.178]	gi|2078284|gb|AAB57689.1|	5.68E-130	amyloid beta-peptide binding protein [Mus musculus]
mmu-let-7e-3p	NM_133208	-26.2	0.936349	-20.3	155	GO:0043231,GO:0046914,GO:0003676,GO:0001071,GO:0006351,GO:0001817,	K09229|1|0.0|1588|mmu:170740|KRAB and SCAN domains-containing zinc finger	gi|113865959|ref|NP_573471.2|	0	zinc finger protein 287 [Mus musculus]
mmu-let-7e-3p	NM_144515	-22	0.999971	-21.43	154	GO:0005622,GO:0046914,GO:0006351,	K09228|1|0.0|1573|mmu:22710|KRAB domain-containing zinc finger protein	gi|122937377|ref|NP_653098.2|	0	zinc finger protein 52 [Mus musculus]

mmu-let-7e-3p	NM_153062	-26.2	0.80779	-21.07	153	GO:0031224,GO:0071702,	K13783|1|0.0|1033|mmu:224674|MFS transporter, OPA family, solute carrier family 37 (glycerol-3-phosphate transporter), member 1/2	gi|23346457|ref|NP_694702.1|	0	glycerol-3-phosphate transporter [Mus musculus]
mmu-let-7e-3p	NM_172587	-21.2	1	-21.71	152	GO:0031224,GO:0031981,GO:0005488,GO:0004721,GO:0006470,GO:0006259,GO:0051443,GO:0000077,	K06639|1|0.0|905|mmu:218294|cell division cycle 14 [EC:3.1.3.48]	gi|148684287|gb|EDL16234.1|	0	CDC14 cell division cycle 14 homolog B (S. cerevisiae), isoform CRA_d [Mus musculus]
mmu-let-7e-3p	NM_027410	-20.6	1	-20.22	151	GO:0031224,GO:0005488,	-	gi|148687074|gb|EDL19021.1|	0	mCG122828, isoform CRA_b [Mus musculus]

mmu-let-7e-3p	NR_028367	-22.6	0.999678	-20.33	149	GO:0031981,GO:0003676,GO:0042254,	K07565|1|3e-94|347|rno:192180|60S ribosome subunit biogenesis protein NIP7	gi|257153336|ref|NP_079667.2|	9.73E-95	60S ribosome subunit biogenesis protein NIP7 homolog isoform 1 [Mus musculus]
mmu-let-7e-3p	NM_008051	-23	0.996705	-20.5	148	GO:0000139,GO:0031224,GO:0031127,GO:0044238,	K00718|1|0.0|760|mmu:14343|galactoside 2-L-fucosyltransferase 1/2 [EC:2.4.1.69]	gi|49355810|ref|NP_032077.2|	0	galactoside 2-alpha-L-fucosyltransferase 1 [Mus musculus]
mmu-let-7e-3p	NM_001008425	-24.3	0.841624	-20.57	147	GO:0000346,GO:0003676,GO:0006406,GO:0006396,	K13175|1|0.0|642|mmu:386612|THO complex subunit 6	gi|56605680|ref|NP_001008425.1|	0	THO complex subunit 6 homolog [Mus musculus]

mmu-let-7e-3p	NM_183355	-23.1	0.999996	-21.33	146	GO:0031981,GO:0043234,GO:0001071,GO:0046983,GO:0003677,GO:0001501,GO:0030097,GO:0045596,GO:0008202,GO:0006357,GO:0003006,GO:0008283,GO:0035108,GO:0001503,GO:0043392,GO:0003002,GO:0048754,GO:0009887,GO:00352	K09355|1|0.0|738|ecb:100058426|pre-B-cell leukemia transcription factor 1	gi|149755772|ref|XP_001493290.1|	0	PREDICTED: pre-B-cell leukemia transcription factor 1 isoform 1 [Equus caballus]
mmu-let-7e-3p	NM_178759	-22	0.999484	-20.38	145	-	K15148|1|5e-23|110|mdo:100029969|mediator of RNA polymerase II transcription subunit 7	gi|38532495|gb|AAR23483.1|	1.08E-126	smuckler [Mus musculus]

mmu-let-7e-3p	NM_177075	NA	NA	-23.89	152	GO:0031224,	K06252|1|1e-06|56.2|oaa:100084981|tenascin	gi|148701878|gb|EDL33825.1|	7.31E-136	mCG51652 [Mus musculus]
mmu-let-7e-3p	NM_172656	NA	NA	-21.35	147	GO:0043231,GO:0044444,GO:0016301,GO:0032559,GO:0032989,GO:0006605,GO:0007169,GO:0006996,GO:0022402,GO:0045860,GO:0019217,GO:0043066,GO:00001	K08271|1|4e-83|310|xtr:493513|protein kinase LYK5	gi|148667693|gb|EDL00110.1|	0	mCG117847, isoform CRA_g [Mus musculus]
mmu-let-7e-3p	NM_009567	NA	NA	-21.93	143	GO:0043231,GO:0046872,GO:0003676,GO:0006351,	K09228|1|0.0|1398|mmu:22755|KRAB domain-containing zinc finger protein	gi|6677629|ref|NP_033593.1|	0	zinc finger protein 235 [Mus musculus]
mmu-let-7e-3p	NM_001001880	-21.6	0.999907	NA	NA	GO:0016021,GO:0005488,GO:0007154,GO:0007167,	K06770|1|4e-88|326|mmu:68481|myelin protein zero	gi|49457857|ref|NP_001001880.1|	3.00E-87	myelin protein zero-like protein 1 isoform b precursor [Mus musculus]

mmu-let-7e-3p	NM_001029856	-20.1	1	NA	NA	GO:0043231,GO:0016462,GO:0032559,	K10754|1|1e-14|84.7|cel:C54G10.2|replication factor C subunit	gi|71143102|ref|NP_001025027.1|	0	ATPase family AAA domain-containing protein 5 [Mus musculus]
mmu-let-7e-3p	NM_010275	-20	1	NA	NA	GO:0044421,GO:0005126,GO:0042802,GO:0070851,GO:0014046,GO:0007178,GO:0007420,GO:0051094,GO:0001667,GO:0014821,GO:0006935,GO:0003156,GO:0007626,GO:0043066,GO:0043488,GO:0032225,GO:0001838,GO:0016044,GO:0003337,GO:0001658,GO:0032768,GO:00074	K05452|1|6e-119|429|mmu:14573|glial cell derived neurotrophic factor	gi|7110601|ref|NP_034405.1|	4.17E-118	glial cell line-derived neurotrophic factor [Mus musculus]

mmu-let-7e-3p	NM_001085374	-21.9	1	NA	NA	GO:0043231,GO:0016020,GO:0005488,GO:0004871,GO:0010631,GO:0030178,GO:00082	-	gi|145699135|ref|NP_001078843.1|	0	mutated in colorectal cancers isoform 2 [Mus musculus]
mmu-let-7e-3p	NM_010576	-20.3	1	NA	NA	-	K06483|1|0.0|2050|mmu:16401|integrin alpha 4	gi|114326554|ref|NP_034706.3|	0	integrin alpha-4 precursor [Mus musculus]
mmu-let-7e-3p	NM_027937	-20.8	1	NA	NA	GO:0044424,GO:0005515,GO:0023052,	K15502|1|6e-34|148|aag:AaeL_AAEL014742|serine/threonine-protein phosphatase 6 regulatory ankyrin repeat subunit A	gi|161168996|ref|NP_082213.2|	0	caskin-1 [Mus musculus]
mmu-let-7e-3p	NM_146179	-20.2	1	NA	NA	-	K09228|1|0.0|1371|mmu:232854|KRAB domain-containing zinc finger protein	gi|124339857|ref|NP_666291.2|	0	zinc finger protein 418 [Mus musculus]

mmu-let-7e-3p	NM_009981	-20.5	1	NA	NA	GO:0031090,GO:0044444,GO:0044425,GO:0005488,GO:0070567,GO:0050896,GO:0006656,	K00968|1|0.0|644|mmu:13026|choline-phosphate cytidylyltransferase [EC:2.7.7.15]	gi|6753552|ref|NP_034111.1|	0	choline-phosphate cytidylyltransferase A [Mus musculus]
mmu-let-7e-3p	NM_027804	-20.4	1	NA	NA	GO:0031090,GO:0043231,GO:0031224,GO:0044444,GO:0046914,GO:0016790,GO:0070011,GO:0006511,GO:0007049,	K11847|1|0.0|2483|mmu:71472|ubiquitin carboxyl-terminal hydrolase 19 [EC:3.1.2.15]!K06243|3|0.0|2333|ssc:100187735|laminin, beta 2	gi|148689348|gb|EDL21295.1|	0	ubiquitin specific peptidase 19, isoform CRA_c [Mus musculus]
mmu-let-7e-3p	NM_172743	-23.5	0.999295	NA	NA	-	K06102|1|1e-06|57.0|mgp:100544052|cingulin	gi|170785877|gb|ACB38004.1|	0	heart adaptor protein 1b [Mus musculus]

mmu-let-7e-3p	NM_027187	-20.8	0.999995	NA	NA	GO:0043234,GO:0043231,GO:0043169,GO:0003676,GO:0016891,GO:0006259,GO:00160	K10743|1|5e-174|612|mmu:69724|ribonuclease H2 subunit A [EC:3.1.26.4]	gi|58037175|ref|NP_081463.1|	3.18E-173	ribonuclease H2 subunit A [Mus musculus]
mmu-let-7e-3p	NM_028153	-23.1	0.992945	NA	NA	GO:0043227,GO:0015630,GO:0005515,GO:0050953,GO:0050954,	K15420|1|0.0|811|tgu:100219108|echinoderm microtubule-associated protein-like 4	gi|244790233|ref|NP_082429.1|	0	echinoderm microtubule-associated protein-like 2 isoform 1 [Mus musculus]
mmu-let-7e-3p	NM_153064	-21.1	0.999814	NA	NA	GO:0005746,GO:0043169,GO:0051536,GO:0000166,GO:0050136,GO:0048037,GO:0022904,GO:0051234,GO:0006950,	K03935|1|0.0|953|mmu:226646|NADH dehydrogenase (ubiquinone) Fe-S protein 2 [EC:1.6.5.3 1.6.99.3]	gi|23346461|ref|NP_694704.1|	0	NADH dehydrogenase [ubiquinone] iron-sulfur protein 2, mitochondrial precursor [Mus musculus]

mmu-let-7e-3p	NM_001201470	-22.6	0.999883	NA	NA	GO:0044444,GO:0004779,GO:0016301,GO:0032559,GO:0048731,GO:0006796,GO:0006790,GO:0034036,GO:0007599,	K13811|1|0.0|1236|mmu:23972|3'-phosphoadenosine 5'-phosphosulfate synthase [EC:2.7.7.4 2.7.1.25]	gi|319918850|ref|NP_001188399.1|	0	bifunctional 3'-phosphoadenosine 5'-phosphosulfate synthase 2 isoform 2 [Mus musculus]

mmu-let-7e-3p	NM_033217	-23.9	0.993343	NA	NA	GO:0031981,GO:0016021,GO:0044444,GO:0043121,GO:0004888,GO:0010467,GO:0031346,GO:0045683,GO:0042475,GO:0001942,GO:0010769,GO:0008284,GO:0051726,GO:0006917,GO:0006935,GO:0006950,GO:0009266,GO:0007399,GO:0008543,GO:0031345,GO:0043066,GO:00486	K02583|1|0.0|803|mmu:18053|nerve growth factor receptor (TNFR superfamily member 16)	gi|70794803|ref|NP_150086.2|	0	tumor necrosis factor receptor superfamily member 16 precursor [Mus musculus]

mmu-let-7e-3p	NM_010956	-20.2	1	NA	NA	GO:0043234,GO:0005740,GO:0019842,GO:0034601,GO:0016624,GO:0005515,GO:0006007,GO:0043648,GO:0006553,GO:0009060,GO:0019674,	K00164|1|0.0|2076|mmu:18293|2-oxoglutarate dehydrogenase E1 component [EC:1.2.4.2]	gi|60360580|dbj|BAD90530.1|	0	mKIAA4192 protein [Mus musculus]
mmu-let-7e-3p	NM_001243792	-23.1	0.999964	NA	NA	GO:0031981,GO:0046914,GO:0001071,GO:0003677,GO:0004879,GO:0019904,GO:0030522,GO:0032501,GO:0009755,	K08554|1|0.0|850|ecb:100050530|estrogen-related receptor gamma	gi|354465166|ref|XP_003495051.1|	0	PREDICTED: estrogen-related receptor gamma-like [Cricetulus griseus]

mmu-let-7e-3p	NM_009723	-23.7	0.999957	NA	NA	GO:0031224,GO:0043005,GO:0043231,GO:0044459,GO:0044297,GO:0005102,GO:0046872,GO:0032559,GO:0019904,GO:0019829,GO:0001775,GO:0050804,GO:0043062,GO:0022037,GO:0032989,GO:0050954,GO:0009207,GO:0042471,GO:0031214,GO:0006874,GO:0006586,GO:00075	K05850|1|0.0|2107|mmu:11941|Ca2+ transporting ATPase, plasma membrane [EC:3.6.3.8]	gi|148667088|gb|EDK99504.1|	0	ATPase, Ca++ transporting, plasma membrane 2, isoform CRA_b [Mus musculus]
mmu-let-7e-3p	NM_011337	-20.9	0.992034	NA	NA	GO:0044421,GO:0005125,GO:0005539,GO:0006952,GO:0002763,GO:0030595,GO:00514	K05408|1|4e-48|191|mmu:20302|C-C motif chemokine 3	gi|6755432|ref|NP_035467.1|	2.71E-47	C-C motif chemokine 3 precursor [Mus musculus]

mmu-let-7e-3p	NM_172821	-21.6	1	NA	NA	GO:0043234,GO:0044424,GO:0019900,GO:0046872,GO:0042802,GO:0032559,GO:0004702,GO:0006468,GO:0051091,GO:00001	K04422|1|0.0|1600|mmu:71751|mitogen-activated protein kinase kinase kinase 13 [EC:2.7.11.25]	gi|95113638|ref|NP_766409.2|	0	mitogen-activated protein kinase kinase kinase 13 [Mus musculus]
mmu-let-7e-3p	NM_024440	-20.6	0.99989	NA	NA	GO:0031227,GO:0005488,GO:0043161,GO:0006984,	K13989|1|2e-88|326|mmu:70377|Derlin-2/3	gi|18859601|ref|NP_077760.1|	1.55E-87	derlin-3 [Mus musculus]
mmu-let-7e-3p	NM_028817	-24.3	0.99206	NA	NA	GO:0031224,GO:0031966,GO:0044439,GO:0016405,GO:0015645,GO:0032559,GO:0016408,GO:0007417,GO:0006631,GO:0035336,GO:0031667,GO:0006641,GO:00100	K01897|1|0.0|1416|mmu:74205|long-chain acyl-CoA synthetase [EC:6.2.1.3]	gi|75992920|ref|NP_083093.2|	0	long-chain-fatty-acid--CoA ligase 3 isoform a [Mus musculus]

mmu-let-7e-3p	NM_145561	-21.1	0.999976	NA	NA	GO:0016021,GO:0004175,GO:0019538,GO:0032501,GO:0050789,	K09641|1|0.0|843|mmu:231382|transmembrane protease, serine 11D [EC:3.4.21.-]	gi|21704106|ref|NP_663536.1|	0	transmembrane protease serine 11D [Mus musculus]
mmu-let-7e-3p	NM_009262	-20.4	1	NA	NA	GO:0031012,GO:0046872,GO:0048731,GO:0023052,GO:0009987,	K08136|1|0.0|778|ptr:462085|sparc/osteonectin, cwcv and kazal-like domains proteoglycan (testican)	gi|148709301|gb|EDL41247.1|	0	sparc/osteonectin, cwcv and kazal-like domains proteoglycan 1, isoform CRA_b [Mus musculus]
mmu-let-7e-3p	NM_001042711	-23.3	0.95649	NA	NA	GO:0044421,GO:0046872,GO:0016160,GO:0005975,	K01176|1|0.0|1090|mmu:100043684|alpha-amylase [EC:3.2.1.1]	gi|148680466|gb|EDL12413.1|	0	mCG11265, isoform CRA_a [Mus musculus]

mmu-let-7e-3p	NM_011428	-20.3	1	NA	NA	GO:0030426,GO:0044431,GO:0044297,GO:0031252,GO:0005856,GO:0005887,GO:0031201,GO:0005267,GO:0008092,GO:0019905,GO:0015980,GO:0007613,GO:0007269,GO:0006904,GO:0050796,GO:0044087,GO:0048667,GO:0034220,	K08508|1|1e-112|408|mgp:100545824|synaptosomal-associated protein, 23kDa	gi|6755588|ref|NP_035558.1|	7.36E-112	synaptosomal-associated protein 25 [Mus musculus]
mmu-let-7e-3p	NM_177898	-22.6	0.998301	NA	NA	GO:0043169,GO:0004672,GO:0032559,GO:0006464,	K08857|1|0.0|1259|mmu:330721|NIMA (never in mitosis gene a)-related kinase [EC:2.7.11.1]	gi|31559852|ref|NP_808566.2|	0	serine/threonine-protein kinase Nek5 [Mus musculus]

mmu-let-7e-3p	NM_016963	-24.9	0.96844	NA	NA	GO:0031252,GO:0005884,GO:0008017,GO:0051693,	K10370|1|1e-179|631|mmu:50875|tropomodulin	gi|8394460|ref|NP_058659.1|	7.42E-179	tropomodulin-3 [Mus musculus]
mmu-let-7e-3p	NM_029023	-20	1	NA	NA	GO:0044444,GO:0004180,GO:0019538,GO:0001523,	K09646|1|0.0|863|mmu:74617|serine carboxypeptidase 1 [EC:3.4.16.-]	gi|253970508|ref|NP_083299.3|	0	retinoid-inducible serine carboxypeptidase precursor [Mus musculus]
mmu-let-7e-3p	NM_022983	-28.5	0.403178	NA	NA	GO:0016021,GO:0043227,GO:0005515,GO:0008289,GO:0045125,GO:0007267,GO:0043270,GO:0007204,GO:0030031,GO:0010740,GO:0007186,GO:0000165,	K04294|1|0.0|674|mmu:65086|lysophosphatidic acid receptor 3	gi|12667796|ref|NP_075359.1|	0	lysophosphatidic acid receptor 3 [Mus musculus]

mmu-let-7e-3p	NM_020559	-20	1	NA	NA	GO:0044429,GO:0016749,GO:0048037,GO:0010038,GO:0014070,GO:0009991,GO:0032868,GO:0006779,GO:0006950,	K00643|1|0.0|1249|mmu:11655|5-aminolevulinate synthase [EC:2.3.1.37]	gi|160707967|ref|NP_065584.2|	0	5-aminolevulinate synthase, nonspecific, mitochondrial precursor [Mus musculus]
mmu-let-7e-3p	NM_013663	-21.2	0.999996	NA	NA	GO:0031981,GO:0003676,GO:0000377,GO:0006405,GO:0006397,GO:0006353,	K12892|1|1e-49|194|cfa:403687|splicing factor, arginine/serine-rich 3	gi|148690658|gb|EDL22605.1|	2.61E-74	mCG21131, isoform CRA_c [Mus musculus]
mmu-let-7e-3p	NR_015549	-20.4	1	NA	NA	-	-	gi|148706443|gb|EDL38390.1|	2.85E-60	mCG146330, isoform CRA_b [Mus musculus]
mmu-let-7e-3p	NM_001177673	-29.6	0.321454	NA	NA	GO:0016021,GO:0004888,GO:0007166,GO:0006952,	K08408|1|0.0|729|mmu:238377|G protein-coupled receptor 68	gi|148686940|gb|EDL18887.1|	0	G protein-coupled receptor 68, isoform CRA_a [Mus musculus]

mmu-let-7e-3p	NM_001145922	-23.5	0.999856	NA	NA	GO:0044464,GO:0005088,GO:0019222,GO:0007266,	K15048|1|5e-22|108|isc:IscW_ISCW021530|kalirin [EC:2.7.11.1]	gi|47847428|dbj|BAD21386.1|	0	mFLJ00128 protein [Mus musculus]
mmu-let-7e-3p	NM_001030014	-27.1	0.502176	NA	NA	GO:0043231,GO:0004312,GO:0006631,	K00645|1|0.0|725|mmu:223722|[acyl-carrier-protein] S-malonyltransferase [EC:2.3.1.39]	gi|71725343|ref|NP_001025185.1|	0	malonyl-CoA-acyl carrier protein transacylase, mitochondrial [Mus musculus]
mmu-let-7e-3p	NM_001195748	-31.8	0.197484	NA	NA	GO:0016020,GO:0044424,GO:0060089,GO:0008047,GO:0007165,	-	gi|307691211|ref|NP_001182677.1|	0	regulator of G-protein signaling 22 [Mus musculus]
mmu-let-7e-3p	NM_028941	-23	0.999876	NA	NA	GO:0043231,	-	gi|254028208|ref|NP_083217.1|	0	C-Maf-inducing protein isoform 2 [Mus musculus]

mmu-let-7e-3p	NM_011989	-20.7	1	NA	NA	GO:0031224,GO:0043231,GO:0005903,GO:0005488,GO:0015645,GO:0005319,GO:0000038,GO:0015908,GO:0031667,GO:00085	K08745|1|0.0|1276|mmu:26569|solute carrier family 27 (fatty acid transporter), member 1/4 [EC:6.2.1.-]	gi|45597453|ref|NP_036119.1|	0	long-chain fatty acid transport protein 4 [Mus musculus]
mmu-let-7e-3p	NM_153157	-21.8	0.999999	NA	NA	GO:0044421,GO:0043231,GO:0005488,GO:0001754,	K04594|1|7e-47|190|dre:560631|latrophilin 3	gi|21955259|ref|NP_665720.1|	0	noelin-3 precursor [Rattus norvegicus]
mmu-let-7e-3p	NM_183017	-24	0.994784	NA	NA	GO:0016881,GO:0043412,	-	gi|148672520|gb|EDL04467.1|	0	tubulin tyrosine ligase-like family, member 12 [Mus musculus]
mmu-let-7e-3p	NM_173019	-21	1	NA	NA	GO:0044444,GO:0008443,GO:0019203,GO:0005515,GO:0032559,GO:0006000,GO:0006796,GO:0006007,	K01103|1|0.0|918|mmu:270198|6-phosphofructo-2-kinase / fructose-2,6-bisphosphatase [EC:2.7.1.105 3.1.3.46]	gi|84662740|ref|NP_766607.3|	0	6-phosphofructo-2-kinase/fructose-2,6-bisphosphatase 4 [Mus musculus]

mmu-let-7e-3p	NM_030081	-21.5	1	NA	NA	GO:0005769,GO:0031090,GO:0046914,GO:0007599,GO:0016192,	K12481|1|0.0|1402|mmu:78287|rabenosyn-5	gi|31541996|ref|NP_084357.2|	0	rabenosyn-5 [Mus musculus]
mmu-let-7e-3p	NR_045155	-22.1	0.994785	NA	NA	-	-	gi|148691452|gb|EDL23399.1|	2.84E-27	mCG144726 [Mus musculus]
mmu-let-7e-3p	NM_001163387	-22.6	0.999985	NA	NA	GO:0043005,GO:0044430,GO:0016021,GO:0044459,GO:0045202,GO:0004871,GO:0005515,GO:0060078,GO:0051094,GO:0007243,GO:0016044,GO:0016337,GO:0006886,GO:0050767,GO:0051899,GO:0010469,GO:0051969,GO:0043062,GO:0006903,GO:0051262,GO	K07378|1|0.0|1480|mmu:192167|neuroligin	gi|254281191|ref|NP_001156859.1|	0	neuroligin-1 isoform 2 [Mus musculus]

mmu-let-7e-3p	NM_183193	-24.1	0.972035	NA	NA	-	K09401|1|3e-138|493|mmu:270004|forkhead box protein I	gi|34147250|ref|NP_899016.1|	2.18E-137	forkhead box protein I2 [Mus musculus]
mmu-let-7e-3p	NM_007472	-28.9	0.385599	NA	NA	GO:0033267,GO:0005774,GO:0005903,GO:0016323,GO:0016021,GO:0005635,GO:0033655,GO:0043025,GO:0030667,GO:0005372,GO:0005221,GO:0015166,GO:0046873,GO:0005488,GO:0019725,GO:0034614,GO:0006915,GO:0015670,GO:0008284,GO:0071241,GO:0048592,GO:00096	K09864|1|4e-148|526|mmu:11826|aquaporin-1	gi|6680710|ref|NP_031498.1|	2.51E-147	aquaporin-1 [Mus musculus]
mmu-let-7e-3p	NM_153560	-24.6	0.989186	NA	NA	-	-	gi|23956346|ref|NP_705788.1|	2.33E-171	protein FAM102A [Mus musculus]

mmu-let-7e-3p	NM_025662	-23	0.999935	NA	NA	GO:0030176,GO:0004175,GO:0035091,GO:0006457,GO:0006497,	K05290|1|0.0|775|mmu:329777|phosphatidylinositol glycan, class K [EC:2.6.-.-]	gi|29788753|ref|NP_079938.1|	0	GPI-anchor transamidase isoform 1 precursor [Mus musculus]
mmu-let-7e-3p	NM_172532	-24.2	0.998931	NA	NA	GO:0044429,GO:0016620,GO:0000166,GO:0042802,GO:0043648,GO:0009063,GO:0022900,GO:0009064,GO:0006631,GO:0042133,GO:0006790,GO:0019318,GO:0007399,GO:0051260,GO:0006644,GO:00066	K00139|1|0.0|972|mmu:214579|succinate-semialdehyde dehydrogenase [EC:1.2.1.24]	gi|27369748|ref|NP_766120.1|	0	succinate-semialdehyde dehydrogenase, mitochondrial precursor [Mus musculus]
mmu-let-7e-3p	NM_026438	-28	0.255464	NA	NA	GO:0044444,GO:0046872,GO:0016462,GO:0006412,GO:0006796,	K01507|1|1e-174|613|mmu:67895|inorganic pyrophosphatase [EC:3.6.1.1	gi|148700191|gb|EDL32138.1|	0	pyrophosphatase (inorganic) 1 [Mus musculus]

mmu-let-7e-3p	NM_001243161	-21.4	1	NA	NA	GO:0031090,GO:0043231,GO:0031224,GO:0005488,GO:0016757,GO:0006487,	K03844|1|0.0|932|rno:361174|alpha-1,2-mannosyltransferase [EC:2.4.1.-	gi|341926298|ref|NP_001230090.1|	0	GDP-Man:Man(3)GlcNAc(2)-PP-Dol alpha-1,2-mannosyltransferase 1 [Mus musculus]
mmu-let-7e-3p	NM_026609	-22.2	0.999711	NA	NA	GO:0031224,	-	gi|355699236|gb|AES01062.1|	3.82E-73	leptin receptor overlapping transcript-like 1 [Mustela putorius furo]
mmu-let-7e-3p	NM_013688	-20.1	1	NA	NA	-	K10165|1|3e-13|78.6|mmu:257632|nucleotide-binding oligomerization domain-containing protein 2	gi|124430564|ref|NP_038716.2|	0	T-complex-associated testis-expressed protein 1 [Mus musculus]
mmu-let-7e-3p	NM_152802	-21.5	0.989148	NA	NA	GO:0042742,	-	gi|23680955|ref|NP_690015.1|	8.09E-45	beta-defensin 12 [Mus musculus]
mmu-let-7e-3p	NM_176920	-20.8	1	NA	NA	GO:0031224,	K06850|1|2e-18|97.1|ssc:100513444|slit 3	gi|28892715|ref|NP_795894.1|	8.95E-174	leucine-rich repeat and transmembrane domain-containing protein 1 precursor [Mus musculus]

mmu-let-7e-3p	NM_001008793	-22.2	0.999992	NA	NA	GO:0030425,GO:0044297,GO:0015629,GO:0005902,GO:0002141,GO:0031513,GO:0030427,GO:0005515,GO:0001894,GO:0050954,GO:0030030,GO:0050953,	K12076|1|1e-16|90.1|ssc:100157729|disks large protein 1!K06095|2|3e-16|89.0|tad:TRIADDRAFT_27973|multiple PDZ domain protein	gi|28972778|dbj|BAC65805.1|	0	mKIAA1526 protein [Mus musculus]
mmu-let-7e-3p	NM_001190817	-26.2	0.939003	NA	NA	GO:0031224,GO:0044432,GO:0031090,GO:0005635,GO:0008047,GO:0003676,GO:0043021,GO:0005515,GO:0006412,GO:0051046,GO:0030162,GO:0006351,	K09521|1|0.0|874|mmu:13418|DnaJ homolog subfamily C member 1	gi|7106295|ref|NP_031895.1|	0	dnaJ homolog subfamily C member 1 precursor [Mus musculus]
mmu-let-7e-3p	NM_153165	-25.7	0.889955	NA	NA	-	K10452|1|1e-120|435|mmu:236904|kelch-like protein	gi|23346619|ref|NP_694805.1|	6.84E-120	kelch-like 15 isoform c [Mus musculus]

mmu-let-7e-3p	NM_001098172	-25.3	0.966978	NA	NA	GO:0016021,GO:0046872,GO:0016337,	K04602|1|1e-63|246|dre:100008568|cadherin EGF LAG seven-pass G-type receptor 3 (flamingo)	gi|28972738|dbj|BAC65785.1|	0	mKIAA1400 protein [Mus musculus]
mmu-let-7e-3p	NM_133681	-22.7	0.993821	NA	NA	GO:0031224,GO:0000323,	K06509|1|5e-21|103|xtr:394799|kangai 1	gi|19526840|ref|NP_598442.1|	3.01E-112	tetraspanin-1 [Mus musculus]
mmu-let-7e-3p	NM_172471	-26.4	0.977959	NA	NA	GO:0004866,GO:0010466,	K05316|1|2e-09|67.0|cqu:CpipJ_CPIJ005979|voltage-dependent calcium channel alpha-2/delta, invertebrate	gi|27369644|ref|NP_766059.1|	0	inter-alpha-trypsin inhibitor heavy chain H5 precursor [Mus musculus]

mmu-let-7e-3p	NM_145407	-20.4	1	NA	NA	GO:0043231,GO:0031224,GO:0044444,GO:0046914,GO:0016790,GO:0070011,GO:0006511,GO:0007049,	K11847|1|0.0|2507|mmu:71472|ubiquitin carboxyl-terminal hydrolase 19 [EC:3.1.2.15]!K06243|2|0.0|2357|ssc:100187735|laminin, beta 2	gi|148689349|gb|EDL21296.1|	0	ubiquitin specific peptidase 19, isoform CRA_d [Mus musculus]

mmu-let-7e-3p	NM_001176528	-20.6	1	NA	NA	GO:0031981,GO:0043005,GO:0044444,GO:0044297,GO:0030371,GO:0003712,GO:0003729,GO:0004879,GO:0001071,GO:0003682,GO:0005501,GO:0046914,GO:0046983,GO:0019207,GO:0055007,GO:0050804,GO:0044093,GO:0035295,GO:0008406,GO:0048232,GO:0021543,GO:00517	K08527|1|0.0|817|ptr:454647|retinoic acid receptor alpha	gi|332847666|ref|XP_003315498.1|	0	PREDICTED: retinoic acid receptor alpha isoform 1 [Pan troglodytes]
mmu-let-7e-3p	NM_001271409	-29.4	0.226801	NA	NA	-	-	-	-	-
mmu-let-7e-3p	NM_001145759	-23.1	0.990299	NA	NA	-	-	gi|224809588|ref|NP_001139231.1|	6.37E-71	uncharacterized protein LOC75861 [Mus musculus]

mmu-let-7e-3p	NM_133680	-21.6	0.999998	NA	NA	GO:0031224,GO:0005488,GO:0008493,GO:0015893,GO:0015850,GO:0006818,GO:0006811,	K14613|1|9e-11|70.5|tca:664418|MFS transporter, PCFT/HCP family, solute carrier family 46 (folate transporter), member 1/3!K08214|2|1e-06|56.6|dre:559752|MFS transporter, OCT family, solute carrier family 22 (organic cation	gi|142349203|ref|NP_598441.3|	0	hippocampus abundant transcript-like protein 1 [Mus musculus]
mmu-let-7e-3p	NM_173425	-23.2	0.996859	NA	NA	GO:0005488,	-	gi|27734128|ref|NP_775601.1|	0	protein FAM124B [Mus musculus]

mmu-let-7e-3p	NM_001081378	-24.3	0.999723	NA	NA	GO:0031224,GO:0044444,GO:0000165,GO:0007169,	K12460|1|0.0|3213|mmu:77480|ankyrin repeat-rich membrane spanning protein	gi|124487039|ref|NP_001074847.1|	0	kinase D-interacting substrate of 220 kDa [Mus musculus]
mmu-let-7e-5p	NM_001003815	-25.3	0.984405	-27.42	166	GO:0043232,GO:0044444,GO:0044425,GO:0008092,GO:0007267,GO:0030036,	K06107|1|0.0|1568|mmu:13821|erythrocyte membrane protein band 4.1	gi|54873604|ref|NP_001003815.1|	0	band 4.1-like protein 1 isoform b [Mus musculus]
mmu-let-7e-5p	NM_001006664	-25.3	0.983594	-27.42	166	GO:0043232,GO:0044444,GO:0044425,GO:0008092,GO:0007267,GO:0030036,	K06107|1|0.0|1555|mmu:13821|erythrocyte membrane protein band 4.1	gi|7305029|ref|NP_038538.1|	0	band 4.1-like protein 1 isoform a [Mus musculus]

mmu-let-7e-5p	NM_001007465	-32.1	0.122603	-27.36	163	GO:0043227,GO:0031410,GO:0046914,GO:0003824,GO:0012501,GO:0015031,	K10871|1|5e-70|267|mcc:718665|RAD51-like protein 3!K10641|3|3e-10|68.6|tad:TRIADDRAFT_53391|E3 ubiquitin-protein ligase LRSAM1 [EC:6.3.2.19]!K16060|4|4e-09|65.1|spu:581540|baculoviral IAP repeat-containing protein 2/3!K10637|5|5e-	gi|148683726|gb|EDL15673.1|	0	ring finger and FYVE like domain containing protein, isoform CRA_c [Mus musculus]
mmu-let-7e-5p	NM_001007589	-30.7	0.093895	-29.97	163	GO:0043231,GO:0043234,GO:0006357,GO:0001819,GO:0002237,GO:0007275,GO:00082	-	gi|86604728|ref|NP_001007590.2|	2.67E-69	akirin-2 [Mus musculus]

mmu-let-7e-5p	NM_001024504	-29.2	0.322744	-26.45	158	-	K10624|1|6e-40|167|ame:724670|E3 ubiquitin-protein ligase RBBP6 [EC:6.3.2.19]	gi|111162659|ref|NP_001019675.2|	1.35E-149	DCN1-like protein 2 isoform a [Mus musculus]
mmu-let-7e-5p	NM_001037493	-27.5	0.44395	-46.26	313	GO:0031224,GO:0015075,GO:0005488,GO:0006811,	K15122|1|0.0|838|mmu:71699|solute carrier family 41	gi|82734225|ref|NP_001032570.1|	0	solute carrier family 41 member 3 isoform 2 [Mus musculus]
mmu-let-7e-5p	NM_001039039	-25.3	0.873336	-25.43	165	GO:0005887,GO:0005267,GO:0034220,GO:0030001,	K15074|1|1e-16|89.7|pon:100450153|BTB/POZ domain-containing adapter for CUL3-mediated RhoA degradation protein	gi|119595459|gb|EAW75053.1|	7.16E-148	hypothetical protein LOC283219, isoform CRA_a [Homo sapiens]
mmu-let-7e-5p	NM_001040130	-30.6	0.034101	-27.76	163	GO:0016020,	-	gi|158517816|ref|NP_001035219.2|	7.99E-47	transmembrane protein 141 isoform 1 [Mus musculus]

mmu-let-7e-5p	NM_001042649	-29.2	0.314512	-26.45	158	-	K10624|1|1e-31|94.4|ame:724670|E3 ubiquitin-protein ligase RBBP6 [EC:6.3.2.19]	gi|148690187|gb|EDL22134.1|	2.05E-133	mCG3700, isoform CRA_e [Mus musculus]
mmu-let-7e-5p	NM_001042650	-29.2	0.304755	-26.45	158	-	K10624|1|1e-17|93.2|ame:724670|E3 ubiquitin-protein ligase RBBP6 [EC:6.3.2.19]	gi|148690187|gb|EDL22134.1|	8.48E-138	mCG3700, isoform CRA_e [Mus musculus]
mmu-let-7e-5p	NM_001042651	-29.2	0.313113	-26.45	158	-	K10624|1|1e-24|109|ame:724670|E3 ubiquitin-protein ligase RBBP6 [EC:6.3.2.19]	gi|148690187|gb|EDL22134.1|	2.91E-132	mCG3700, isoform CRA_e [Mus musculus]

mmu-let-7e-5p	NM_001045521	-27.9	0.208975	-25.54	167	GO:0004175,GO:0019538,	K01324|1|6e-51|202|aml:100468355|plasma kallikrein [EC:3.4.21.34]!K09628|2|1e-50|201|rno:287108|protease, serine 27 [EC:3.4.21.-]!K09629|4|4e-50|199|mdo:100018399|protease, serine, 33!K09640|5|5e-50|199|xtr:448231|transmembrane	gi|113865889|ref|NP_001038986.1|	4.23E-169	serine protease 38 precursor [Mus musculus]
mmu-let-7e-5p	NM_001080979	-29.1	0.322977	-47.37	327	GO:0043234,GO:0003676,GO:0001071,GO:0035556,GO:0006357,GO:0045165,GO:00018	K09448|1|0.0|714|mmu:21679|TEA domain family member 1/3/4	gi|148667455|gb|EDK99871.1|	0	TEA domain family member 4, isoform CRA_b [Mus musculus]

mmu-let-7e-5p	NM_001081135	-25.1	0.985853	-45.85	309	GO:0031224,GO:0046872,	K04949|1|1e-17|94.0|ecb:100069276|cyclic nucleotide gated channel alpha 2!K01314|2|5e-09|65.5|cfa:476993|coagulation factor X [EC:3.4.21.6]!K01320|3|7e-09|65.1|aml:100471728|coagulation factor VII [EC:3.4.21.21]!K01313|4|1e-08|64.3|xtr:548514|	gi|124487491|ref|NP_001074604.1|	4.00E-108	transmembrane gamma-carboxyglutamic acid protein 3 precursor [Mus musculus]
mmu-let-7e-5p	NM_001081197	-29.1	0.165486	-27.86	170	GO:0046914,GO:0016810,	K01500|1|9e-132|471|hsa:403314|[EC:3.5.4.-]	gi|148707507|gb|EDL39454.1|	0	mCG1047261 [Mus musculus]

mmu-let-7e-5p	NM_001081347	-26.5	0.832554	-42.31	313	GO:0044444,GO:0016020,GO:0032561,GO:0035556,GO:0009966,	K07868|1|0.0|1371|mmu:69288|Rho-related BTB domain-containing protein 1/2	gi|124486809|ref|NP_001074816.1|	0	rho-related BTB domain-containing protein 1 isoform 1 [Mus musculus]
mmu-let-7e-5p	NM_001109993	-30.6	0.051522	-27.76	163	GO:0016020,	-	gi|158517816|ref|NP_001035219.2|	3.45E-36	transmembrane protein 141 isoform 1 [Mus musculus]
mmu-let-7e-5p	NM_001110140	-28.7	0.526382	-26.05	155	GO:0032991,GO:0016021,GO:0005789,GO:0001664,GO:0019829,GO:0046872,GO:0032559,GO:0009888,GO:0007599,GO:0008016,GO:0006816,GO:0006936,GO:0009207,GO:0007165,GO:0001775,GO:0009206,GO:0009725,GO:0006875,	K05853|1|0.0|2035|mmu:11938|Ca2+ transporting ATPase, sarcoplasmic/endoplasmic reticulum [EC:3.6.3.8]	gi|60360584|dbj|BAD90532.1|	0	mKIAA4195 protein [Mus musculus]

mmu-let-7e-5p	NM_001111017	-28.4	0.649704	-25.3	164	GO:0005789,GO:0044421,GO:0031224,GO:0016787,GO:0043062,GO:0046488,GO:0015031,	K01099|1|1e-32|144|mmu:20975|phosphatidylinositol-bisphosphatase [EC:3.1.3.36]	gi|161169010|ref|NP_001104487.1|	0	protein SERAC1 isoform 3 [Mus musculus]
mmu-let-7e-5p	NM_001113360	-25.3	0.967442	-46	308	GO:0016020,GO:0060089,GO:0046872,GO:0004629,GO:0007165,GO:0006650,	K15370|1|6e-104|380|nve:NEMVE_v1g89019|inactive phospholipase C-like	gi|164565357|ref|NP_001106831.1|	0	1-phosphatidylinositol 4,5-bisphosphate phosphodiesterase eta-2 isoform b [Mus musculus]
mmu-let-7e-5p	NM_001114098	-27.8	0.821159	-25.33	157	-	K10388|1|2e-09|67.0|dre:567310|plectin!K10352|5|2e-08|63.9|smm:Smp_085540.6|myosin heavy chain	gi|166235131|ref|NP_766551.3|	0	coiled-coil domain-containing protein 165 isoform 2 [Mus musculus]
mmu-let-7e-5p	NM_001115077	-27.4	0.29998	-26.27	158	-	-	gi|169234924|ref|NP_001108549.1|	0	uncharacterized protein LOC627009 [Mus musculus]

mmu-let-7e-5p	NM_001122730	-26.3	0.985622	-46.75	317	-	-	gi|170172511|ref|NP_001116202.1|	0	trinucleotide repeat-containing gene 18 protein isoform A [Mus musculus]
mmu-let-7e-5p	NM_001142697	-30	0.296571	-26.45	170	GO:0015630,	-	gi|218156330|ref|NP_001004361.2|	2.24E-133	tubulin polyglutamylase complex subunit 2 isoform a [Mus musculus]
mmu-let-7e-5p	NM_001145899	-26.3	0.35216	-26.19	160	GO:0016021,GO:0005488,GO:0015238,GO:0015198,GO:0015295,GO:0015893,GO:0006857,	K14637|1|1e-119|430|rno:60577|solute carrier family 15 (oligopeptide transporter), member 2	gi|149060554|gb|EDM11268.1|	1.91E-138	solute carrier family 15 (H+/peptide transporter), member 2, isoform CRA_b [Rattus norvegicus]
mmu-let-7e-5p	NM_001160012	-27.9	0.32632	-26.36	168	GO:0005921,GO:0031224,GO:0005488,GO:0022829,GO:0043009,GO:0008544,GO:0050954,GO:0009987,	K07622|1|4e-136|485|mmu:14620|gap junction protein, beta 3	gi|6680013|ref|NP_032152.1|	3.12E-135	gap junction beta-3 protein [Mus musculus]

mmu-let-7e-5p	NM_001161433	-30.8	0.118728	-28.81	166	GO:0016021,GO:0005035,GO:0005488,GO:0043410,GO:0048869,GO:0009888,GO:0051091,GO:0008219,GO:0019221,GO:00072	K05163|1|5e-165|582|mmu:245527|tumor necrosis factor receptor superfamily member 27	gi|148682260|gb|EDL14207.1|	1.81E-166	ectodysplasin A2 isoform receptor, isoform CRA_b [Mus musculus]
mmu-let-7e-5p	NM_001162381	-26.1	0.432594	-25.66	158	-	-	gi|193806567|sp|A2AIP0.1|F166B_MOUSE	1.99E-145	RecName: Full=Protein FAM166B
mmu-let-7e-5p	NM_001163641	-25	0.973036	-44.49	301	GO:0031981,GO:0043232,GO:0016020,GO:0003676,GO:0046914,GO:0016279,GO:0001832,GO:0006479,GO:0006351,GO:00063	K11421|1|0.0|2232|mmu:84505|histone-lysine N-methyltransferase SETDB [EC:2.1.1.43]	gi|148706861|gb|EDL38808.1|	0	mCG16729, isoform CRA_b [Mus musculus]

mmu-let-7e-5p	NM_001163642	-25	0.97225	-44.49	301	GO:0031981,GO:0043232,GO:0016020,GO:0003676,GO:0046914,GO:0016279,GO:0001832,GO:0006479,GO:0006351,GO:00063	K11421|1|0.0|2225|mmu:84505|histone-lysine N-methyltransferase SETDB [EC:2.1.1.43]	gi|254692954|ref|NP_001157114.1|	0	histone-lysine N-methyltransferase SETDB1 isoform b [Mus musculus]
mmu-let-7e-5p	NM_001163676	-30.1	0.398422	-27.62	166	GO:0044459,GO:0030667,GO:0042625,GO:0032559,GO:0009719,GO:0008284,GO:0010033,	K05673|1|0.0|2261|mmu:239273|ATP-binding cassette, subfamily C (CFTR/MRP), member 4	gi|255683328|ref|NP_001157148.1|	0	ATP-binding cassette, sub-family C (CFTR/MRP), member 4 isoform 3 [Mus musculus]

mmu-let-7e-5p	NM_001164569	-32.1	0.128365	-27.36	163	GO:0043227,GO:0031410,GO:0046914,GO:0003824,GO:0012501,GO:0015031,	K10871|1|5e-70|267|mcc:718665|RAD51-like protein 3!K10641|3|4e-10|68.6|tad:TRIADDRAFT_53391|E3 ubiquitin-protein ligase LRSAM1 [EC:6.3.2.19]!K16060|4|4e-09|65.1|spu:581540|baculoviral IAP repeat-containing protein 2/3!K10637|5|5e-	gi|257196123|ref|NP_001158041.1|	0	E3 ubiquitin-protein ligase rififylin isoform 3 [Mus musculus]

mmu-let-7e-5p	NM_001164570	-32.1	0.126048	-27.36	163	GO:0043227,GO:0031410,GO:0046914,GO:0003824,GO:0012501,GO:0015031,	K10871|1|5e-70|267|mcc:718665|RAD51-like protein 3!K10641|3|4e-10|68.6|tad:TRIADDRAFT_53391|E3 ubiquitin-protein ligase LRSAM1 [EC:6.3.2.19]!K16060|4|4e-09|65.1|spu:581540|baculoviral IAP repeat-containing protein 2/3!K10637|5|5e-	gi|257153330|ref|NP_001158042.1|	0	E3 ubiquitin-protein ligase rififylin isoform 4 [Mus musculus]

mmu-let-7e-5p	NM_001164571	-32.1	0.119351	-27.36	163	GO:0043227,GO:0031410,GO:0046914,GO:0003824,GO:0012501,GO:0015031,	K10871|1|1e-69|266|mcc:718665|RAD51-like protein 3!K10641|3|3e-10|68.6|tad:TRIADDRAFT_53391|E3 ubiquitin-protein ligase LRSAM1 [EC:6.3.2.19]!K16060|4|4e-09|65.1|spu:581540|baculoviral IAP repeat-containing protein 2/3!K10637|5|5e-	gi|148683726|gb|EDL15673.1|	0	ring finger and FYVE like domain containing protein, isoform CRA_c [Mus musculus]
mmu-let-7e-5p	NM_001166549	-26.9	0.727574	-44.36	313	GO:0043231,GO:0044444,GO:0008135,GO:0006810,GO:0006412,	-	gi|262118204|ref|NP_001160020.1|	0	eukaryotic translation initiation factor 4E transporter isoform b [Mus musculus]

mmu-let-7e-5p	NM_001168369	-32.7	0.032895	-26.66	168	-	-	gi|270265823|ref|NP_001161841.1|	0	uncharacterized protein LOC75453 isoform 1 [Mus musculus]
mmu-let-7e-5p	NM_001190187	-29.1	0.422878	-25.11	169	GO:0016021,GO:0030296,GO:0005102,GO:0035556,GO:0060788,GO:0048732,GO:00160	K05457|1|0.0|1175|mmu:18183|neuregulin 3	gi|298231177|ref|NP_001177116.1|	0	pro-neuregulin-3, membrane-bound isoform isoform 2 [Mus musculus]
mmu-let-7e-5p	NM_001190188	-29.1	0.419	-25.11	169	GO:0016021,GO:0030296,GO:0005102,GO:0035556,GO:0060788,GO:0048732,GO:00160	K05457|1|0.0|1098|mmu:18183|neuregulin 3	gi|298231179|ref|NP_001177117.1|	0	pro-neuregulin-3, membrane-bound isoform isoform 3 [Mus musculus]
mmu-let-7e-5p	NM_001195673	-29.7	0.106342	-25.02	155	-	-	gi|307548932|ref|NP_001182602.1|	0	proline-rich protein 22 [Mus musculus]
mmu-let-7e-5p	NM_001205068	-26.6	0.82522	-45.69	315	-	K11323|1|1e-17|94.0|dwi:Dwil_GK14253|histone arginine demethylase JMJD6 [EC:1.14.1	gi|326633177|ref|NP_001191997.1|	0	jmjC domain-containing protein 4 isoform 2 [Mus musculus]

mmu-let-7e-5p	NM_001214911	-25.2	0.950771	-68.36	467	-	K13254|1|1e-25|120|xtr:549207|spastin [EC:3.6.4.3]	gi|148672121|gb|EDL04068.1|	6.82E-147	mCG52237 [Mus musculus]
mmu-let-7e-5p	NM_001242363	-32.3	0.349048	-42.55	321	GO:0043234,GO:0005488,GO:0007041,GO:0016197,GO:0007033,	-	gi|39930595|ref|NP_950174.1|	0	FTS and Hook-interacting protein isoform a [Mus musculus]
mmu-let-7e-5p	NM_001242364	-32.3	0.347774	-42.55	321	GO:0043234,GO:0007041,GO:0016197,GO:0007033,	-	gi|334358879|ref|NP_001229293.1|	0	FTS and Hook-interacting protein isoform b [Mus musculus]
mmu-let-7e-5p	NM_001252636	-26.5	0.831404	-42.31	313	GO:0044444,GO:0016020,GO:0032561,GO:0035556,GO:0009966,	K07868|1|0.0|1371|mmu:69288|Rho-related BTB domain-containing protein 1/2	gi|124486809|ref|NP_001074816.1|	0	rho-related BTB domain-containing protein 1 isoform 1 [Mus musculus]

mmu-let-7e-5p	NM_001252637	-25.4	0.945263	-42.31	313	GO:0044444,GO:0016020,GO:0032561,GO:0035556,GO:0009966,	K07868|1|0.0|1371|mmu:69288|Rho-related BTB domain-containing protein 1/2	gi|124486809|ref|NP_001074816.1|	0	rho-related BTB domain-containing protein 1 isoform 1 [Mus musculus]
mmu-let-7e-5p	NM_001252638	-25.4	0.947194	-42.31	313	GO:0044444,GO:0016020,GO:0032561,GO:0035556,GO:0009966,	K07868|1|0.0|1167|mmu:69288|Rho-related BTB domain-containing protein 1/2	gi|124486809|ref|NP_001074816.1|	0	rho-related BTB domain-containing protein 1 isoform 1 [Mus musculus]
mmu-let-7e-5p	NM_001271578	-25.4	0.805553	-64.56	472	-	-	-	-	-
mmu-let-7e-5p	NM_001271768	-30.9	0.336258	-27.15	167	-	-	-	-	-

mmu-let-7e-5p	NM_007426	-29.9	0.200119	-25.31	167	GO:0016020,GO:0043231,GO:0044421,GO:0005126,GO:0043169,GO:0050922,GO:0009888,GO:0007599,GO:0003006,GO:0007162,GO:0007169,GO:0010596,GO:0009628,GO:0048869,GO:0009887,GO:0048609,GO:0009746,GO:0002376,GO:0045765,GO:00	K05466|1|0.0|1007|mmu:11601|angiopoietin 2	gi|31982508|ref|NP_031452.2|	0	angiopoietin-2 precursor [Mus musculus]
mmu-let-7e-5p	NM_008126	-27.9	0.276675	-26.36	168	GO:0005921,GO:0031224,GO:0005488,GO:0022829,GO:0043009,GO:0008544,GO:0050954,GO:0009987,	K07622|1|4e-136|485|mmu:14620|gap junction protein, beta 3	gi|6680013|ref|NP_032152.1|	2.54E-135	gap junction beta-3 protein [Mus musculus]

mmu-let-7e-5p	NM_008734	-29.1	0.423135	-25.11	169	GO:0016021,GO:0030296,GO:0005102,GO:0035556,GO:0060788,GO:0048732,GO:00160	K05457|1|0.0|1169|mmu:18183|neuregulin 3	gi|6679128|ref|NP_032760.1|	0	pro-neuregulin-3, membrane-bound isoform isoform 1 [Mus musculus]
mmu-let-7e-5p	NM_008763	-29.4	0.087579	-27.98	154	GO:0031224,GO:0004930,GO:0023052,GO:0007606,	K04257|1|9e-164|576|mmu:18313|olfactory	gi|6679172|ref|NP_032789.1|	6.08E-163	olfactory receptor 16 [Mus musculus]
mmu-let-7e-5p	NM_008824	-32.8	0.036721	-28.79	176	GO:0043234,GO:0044444,GO:0070061,GO:0008443,GO:0019899,GO:0019203,GO:0032559,GO:0006007,GO:0035556,GO:0033131,GO:0009887,GO:0043434,GO:0031960,GO:0015980,GO:0006000,GO:00069	K01103|1|0.0|910|mmu:18639|6-phosphofructo-2-kinase / fructose-2,6-bisphosphatase [EC:2.7.1.105 3.1.3.46]	gi|7434494|pir||S77704	0	6-phosphofructo-2-kinase (EC 2.7.1.105) / fructose-2, 6-bisphosphate 2-phosphatase (EC 3.1.3.46) clone 5c, skeletal muscle - rat

mmu-let-7e-5p	NM_008832	-25.1	0.987657	-47.27	334	GO:0043234,GO:0043227,GO:0044444,GO:0016020,GO:0005515,GO:0015926,GO:0070567,GO:0004683,GO:0006468,GO:0019318,GO:0006656,GO:0005977,	K07190|1|0.0|2407|mmu:18679|phosphorylase kinase alpha/beta subunit	gi|153945840|ref|NP_032858.2|	0	phosphorylase b kinase regulatory subunit alpha, skeletal muscle isoform isoform 1 [Mus musculus]
mmu-let-7e-5p	NM_008988	-27.7	0.537522	-48.44	311	GO:0016021,GO:0050905,	K06765|1|2e-68|261|mdo:100010164|deleted in colorectal carcinoma	gi|6679571|ref|NP_033014.1|	0	immunoglobulin superfamily DCC subclass member 3 [Mus musculus]
mmu-let-7e-5p	NM_009074	-33	0.119737	-28.82	175	GO:0031224,GO:0032432,GO:0004871,GO:0004713,GO:0032559,GO:0051707,GO:0007167,GO:0032501,GO:0006464,	K05100|1|0.0|2660|mmu:19882|macrophage-stimulating 1 receptor [EC:2.7.10.1]	gi|254826769|ref|NP_033100.2|	0	macrophage-stimulating protein receptor precursor [Mus musculus]

mmu-let-7e-5p	NM_009218	-25.6	0.907592	-25.33	162	GO:0016021,GO:0072372,GO:0044424,GO:0005488,GO:0004872,GO:0022037,GO:0007186,GO:0051384,GO:0048232,GO:0032355,GO:0007154,GO:0008283,GO:00069	K04219|1|3e-165|583|mmu:20607|somatostatin receptor 3	gi|84370258|ref|NP_033244.2|	1.96E-164	somatostatin receptor type 3 [Mus musculus]
mmu-let-7e-5p	NM_009298	-29.8	0.218781	-25.51	166	GO:0031981,GO:0003676,GO:0022613,	-	gi|6678185|ref|NP_033324.1|	6.34E-137	surfeit locus protein 6 [Mus musculus]
mmu-let-7e-5p	NM_009805	-25	0.798294	-25.36	157	GO:0031264,GO:0044424,GO:0005488,GO:0004175,GO:0019538,GO:0010466,GO:0007249,GO:0043066,	K04724|1|2e-115|417|mmu:12633|CASP8 and FADD-like apoptosis regulator	gi|148667680|gb|EDL00097.1|	1.59E-124	CASP8 and FADD-like apoptosis regulator, isoform CRA_c [Mus musculus]

mmu-let-7e-5p	NM_010360	-27.4	0.180107	-25.79	162	GO:0044424,GO:0005515,GO:0016765,GO:0006790,GO:0001885,GO:0048545,	K00799|1|3e-130|464|mmu:14866|glutathione S-transferase [EC:2.5.1.18]	gi|6754086|ref|NP_034490.1|	2.05E-129	glutathione S-transferase Mu 5 [Mus musculus]
mmu-let-7e-5p	NM_011026	-33.4	0.034503	-29.61	177	GO:0033267,GO:0044430,GO:0044459,GO:0016021,GO:0030425,GO:0044444,GO:0044297,GO:0005231,GO:0050839,GO:0032559,GO:0016502,GO:0042802,GO:0046914,GO:0001775,GO:0055118,GO:0006915,GO:0035587,GO:0048871,GO:0006814,GO:0051899,GO:0001508,GO:00099	K05218|1|0.0|758|mmu:18438|purinergic receptor P2X, ligand-gated ion channel 4	gi|117676374|ref|NP_035156.2|	0	P2X purinoceptor 4 [Mus musculus]

mmu-let-7e-5p	NM_011060	-26	0.792247	-26.68	168	GO:0044424,GO:0046872,GO:0016813,GO:0006527,GO:0018195,	K01481|1|0.0|1349|mmu:18601|protein-arginine deiminase [EC:3.5.3.15]	gi|156938255|ref|NP_035190.3|	0	protein-arginine deiminase type-3 [Mus musculus]
mmu-let-7e-5p	NM_011101	-28.1	0.823892	-25.02	169	GO:0031981,GO:0043005,GO:0032991,GO:0031513,GO:0044425,GO:0044444,GO:0044297,GO:0004697,GO:0035184,GO:0032559,GO:0005515,GO:0046914,GO:0001775,GO:0010829,GO:0001932,GO:0008284,GO:0033673,GO:0010595,GO:0015980,GO:0007169,GO:0051384,GO:00313	K02677|1|0.0|1407|mmu:18750|classical protein kinase C [EC:2.7.11.13]	gi|164663791|ref|NP_035231.2|	0	protein kinase C alpha type [Mus musculus]

mmu-let-7e-5p	NM_011150	-25.3	0.775635	-51.71	333	-	K13912|1|3e-29|131|ssc:751862|deleted in malignant brain tumors 1 protein	gi|6755144|ref|NP_035280.1|	0	galectin-3-binding protein precursor [Mus musculus]
mmu-let-7e-5p	NM_011426	-28.4	0.697424	-43.54	309	GO:0031224,GO:0030246,GO:0006952,GO:0031589,GO:0016192,	K06548|1|0.0|3231|mmu:20612|sialoadhesin	gi|226958332|ref|NP_035556.3|	0	sialoadhesin precursor [Mus musculus]
mmu-let-7e-5p	NM_011836	-32.6	0.178881	-28.36	163	GO:0044464,GO:0005578,GO:0021782,GO:0050953,GO:0000902,	K06247|1|0.0|3086|mmu:23928|laminin, gamma 3	gi|66392579|ref|NP_035966.2|	0	laminin subunit gamma-3 precursor [Mus musculus]
mmu-let-7e-5p	NM_013510	-25.3	0.984315	-27.42	166	GO:0043232,GO:0044444,GO:0044425,GO:0008092,GO:0007267,GO:0030036,	K06107|1|0.0|1555|mmu:13821|erythrocyte membrane protein band 4.1	gi|28972159|dbj|BAC65533.1|	0	mKIAA0338 protein [Mus musculus]
mmu-let-7e-5p	NM_013581	-29.2	0.322843	-27.68	170	GO:0012505,GO:0043234,GO:0043231,GO:0005488,GO:0048193,GO:0006996,	-	gi|159110754|ref|NP_038609.3|	0	conserved oligomeric Golgi complex subunit 1 [Mus musculus]

mmu-let-7e-5p	NM_013672	-25.2	0.994512	-25.73	154	GO:0031981,GO:0032991,GO:0001071,GO:0046872,GO:0044212,GO:0042802,GO:0043565,GO:0043566,GO:0019899,GO:0001501,GO:0048048,GO:0030323,GO:0030218,GO:0048609,GO:0001825,GO:00439	K04684|1|0.0|1107|mmu:20683|transcription factor Sp1	gi|119226255|ref|NP_038700.2|	0	transcription factor Sp1 [Mus musculus]	
mmu-let-7e-5p	NM_013753	-29.4	0.404849	-25.78	160	-	-	gi|187951755|gb|AAI37696.1|	0	X99384 protein [Mus musculus]	
mmu-let-7e-5p	NM_013792	-27.7	0.451118	-26.86	164	GO:0000323,GO:0015929,GO:0042471,GO:0042462,GO:0021695,GO:0007626,GO:0007033,	K01205|1|0.0|1461|mmu:27419|alpha-N-acetylglucosaminidase [EC:3.2.1.50]	gi|254910995|ref|NP_038820.2|	0	alpha-N-acetylglucosaminidase precursor [Mus musculus]	

mmu-let-7e-5p	NM_013907	-26.5	0.53851	-25.44	159	GO:0043234,GO:0008284,GO:0019941,GO:0007166,GO:0009888,GO:0030326,	K10262|1|0.0|776|mmu:30838|F-box and WD-40 domain protein 4	gi|14780880|ref|NP_038935.1|	0	F-box/WD repeat-containing protein 4 [Mus musculus]
mmu-let-7e-5p	NM_016695	-35.2	0.042155	-33.46	184	GO:0016021,GO:0019904,GO:0023052,	K06103|1|1e-115|419|ptr:100608149|calcium/calmodulin-dependent serine protein kinase [EC:2.7.11.1]	gi|7710062|ref|NP_057904.1|	0	MAGUK p55 subfamily member 2 [Mus musculus]
mmu-let-7e-5p	NM_018877	-25	0.972309	-44.49	301	GO:0031981,GO:0043232,GO:0016020,GO:0003676,GO:0046914,GO:0016279,GO:0001832,GO:0006479,GO:0006351,GO:00063	K11421|1|0.0|2232|mmu:84505|histone-lysine N-methyltransferase SETDB [EC:2.1.1.43]	gi|148706861|gb|EDL38808.1|	0	mCG16729, isoform CRA_b [Mus musculus]

mmu-let-7e-5p	NM_021301	-26.3	0.835253	-26.19	160	GO:0016021,GO:0005488,GO:0015238,GO:0015198,GO:0015295,GO:0015893,GO:0006857,	K14637|1|0.0|1248|rno:60577|solute carrier family 15 (oligopeptide transporter), member 2	gi|225543357|ref|NP_067276.2|	0	solute carrier family 15 member 2 isoform 1 [Mus musculus]
mmu-let-7e-5p	NM_021362	-27.7	0.925695	-47.15	324	GO:0044424,GO:0044421,GO:0046914,GO:0004175,GO:0048869,GO:0022414,	K07762|1|0.0|3240|mmu:18491|pappalysin-1 [EC:3.4.24.79]	gi|20148857|gb|AAM12687.1|AF439513_1	0	pregnancy-associated plasma protein-A, partial [Mus musculus]
mmu-let-7e-5p	NM_021390	-32	0.198266	-50.84	332	-	K09228|1|2e-24|116|ssc:100156843|KRAB domain-containing zinc finger protein	gi|159032064|ref|NP_067365.2|	0	sal-like protein 1 [Mus musculus]

mmu-let-7e-5p	NM_021485	-29.9	0.110454	-46.12	312	GO:0031981,GO:0030529,GO:0004674,GO:0032559,GO:0007243,GO:0006796,GO:0048017,GO:0007169,GO:0006446,GO:00064	K04688|1|0.0|796|mmu:58988|p70 ribosomal S6 kinase [EC:2.7.11.1]	gi|10946894|ref|NP_067460.1|	0	ribosomal protein S6 kinase beta-2 [Mus musculus]
mmu-let-7e-5p	NM_021877	-25.9	0.946325	-42.95	318	GO:0044451,GO:0043169,GO:0003712,GO:0003676,GO:0001071,GO:0019899,GO:0008134,GO:0051259,GO:0006355,GO:0043392,	K11449|1|2e-62|243|cfa:479221|jumonji domain-containing protein 1C [EC:1.14.11.-]!K15601|3|9e-53|210|isc:IscW_ISCW018588|lysine-specific demethylase 3 [EC:1.14.11.-]	gi|31544072|ref|NP_068677.2|	0	protein hairless [Mus musculus]

mmu-let-7e-5p	NM_023186	-25.2	0.664512	-46.67	299	GO:0044424,GO:0044421,GO:0030247,GO:0004553,GO:0019899,GO:0005126,GO:0043167,GO:0012501,GO:0006952,GO:0051707,GO:0006026,GO:0002376,GO:0042221,GO:0032722,GO:0006030,	K01183|1|0.0|931|mmu:81600|chitinase [EC:3.2.1.14]	gi|6934190|gb|AAF31644.1|AF154571_1	0	putative chitinase precursor [Mus musculus]
mmu-let-7e-5p	NM_023320	-28.9	0.153682	-25.83	159	GO:0043231,GO:0016020,	-	gi|12963637|ref|NP_075809.1|	0	pleckstrin homology domain-containing family O member 1 [Mus
mmu-let-7e-5p	NM_023815	-29.7	0.377166	-25.99	175	GO:0043231,GO:0005515,GO:0004672,GO:0032559,GO:0006464,GO:0008610,GO:00067	K08851|1|5e-112|407|mmu:76367|TP53 regulating kinase [EC:2.7.11.1]	gi|31560052|ref|NP_076304.2|	3.71E-111	TP53-regulating kinase [Mus musculus]

mmu-let-7e-5p	NM_024469	-30.9	0.330088	-27.15	167	GO:0043234,GO:0003712,GO:0006357,GO:0009605,	K03730|1|1e-167|590|mmu:79362|class B basic helix-loop-helix	gi|13277378|ref|NP_077789.1|	8.18E-167	class E basic helix-loop-helix protein 41 [Mus musculus]
mmu-let-7e-5p	NM_025271	-25.6	0.559292	-25.92	152	GO:0005856,GO:0005198,	K05692|1|2e-81|303|smm:Smp_046590|actin beta/gamma 1	gi|146198843|ref|NP_079547.2|	0	actin-like protein 7B [Mus musculus]
mmu-let-7e-5p	NM_025404	-32.8	0.027239	-29.89	167	GO:0031981,GO:0016020,GO:0017111,GO:0032561,GO:0035556,GO:0009207,GO:00150	K07948|1|4e-102|372|rno:303559|ADP-ribosylation factor-like 4D	gi|13384792|ref|NP_079680.1|	2.62E-101	ADP-ribosylation factor-like protein 4D [Mus musculus]
mmu-let-7e-5p	NM_025708	-29.1	0.315853	-25.43	165	GO:0031224,GO:0043231,	-	gi|27228988|ref|NP_079984.2|	2.24E-122	transmembrane protein 186 [Mus musculus]
mmu-let-7e-5p	NM_025869	-27	0.419591	-43.19	297	GO:0043231,GO:0005488,GO:0004721,GO:0006470,	K14165|1|2e-112|406|mmu:66959|dual specificity phosphatase [EC:3.1.3.16 3.1.3.48]	gi|27229024|ref|NP_080145.1|	1.67E-111	dual specificity protein phosphatase 26 [Mus musculus]

mmu-let-7e-5p	NM_025907	-26.7	0.498116	-49.31	320	GO:0016741,GO:0006730,	K00599|1|4e-163|575|mmu:67011|[EC:2.1.1.-]	gi|13385400|ref|NP_080183.1|	3.03E-162	methyltransferase-like protein 6 [Mus musculus]
mmu-let-7e-5p	NM_025994	-28.2	0.364732	-25.98	153	GO:0016020,GO:0046872,	-	gi|31981086|ref|NP_080270.2|	2.07E-94	EF-hand domain-containing protein D2 [Mus musculus]
mmu-let-7e-5p	NM_026010	-29.4	0.108418	-26.53	163	GO:0043231,GO:0005488,GO:0006633,GO:0043412,	K03801|1|2e-125|449|mmu:67164|lipoyl(octanoyl) transferase [EC:2.3.1.181]	gi|109731836|gb|AAI15473.1|	1.09E-124	RIKEN cDNA 2610209A20 gene [Mus musculus]

mmu-let-7e-5p	NM_026097	-32.1	0.123686	-27.36	163	GO:0043227,GO:0031410,GO:0046914,GO:0003824,GO:0012501,GO:0015031,	K10871|1|1e-69|266|mcc:718665|RAD51-like protein 3!K10641|3|4e-10|68.6|tad:TRIADDRAFT_53391|E3 ubiquitin-protein ligase LRSAM1 [EC:6.3.2.19]!K16060|4|4e-09|65.1|spu:581540|baculoviral IAP repeat-containing protein 2/3!K10637|5|5e-	gi|16975488|ref|NP_080373.1|	0	E3 ubiquitin-protein ligase rififylin isoform 2 [Mus musculus]

mmu-let-7e-5p	NM_026184	-29.6	0.377877	-28.49	180	GO:0031090,GO:0043231,GO:0016667,GO:0005515,GO:0050662,GO:0051234,GO:0006457,GO:0006091,	K10976|1|0.0|881|mmu:67475|ERO1-like protein beta [EC:1.8.4.-]	gi|38348230|ref|NP_080460.2|	0	ERO1-like protein beta precursor [Mus musculus]
mmu-let-7e-5p	NM_026240	-30.7	0.154128	-28.31	171	-	-	gi|12858310|dbj|BAB31270.1|	0	unnamed protein product [Mus musculus]
mmu-let-7e-5p	NM_026376	-31.3	0.327208	-27.14	163	GO:0031224,GO:0005488,GO:0004888,GO:0001525,GO:0006935,GO:0023052,GO:00606	K06822|1|0.0|3678|mmu:67784|plexin D	gi|51243714|gb|AAT99561.1|	0	plexin D1 [Mus musculus]
mmu-let-7e-5p	NM_026533	-30.8	0.018528	-29.34	172	GO:0015935,GO:0031981,GO:0003723,GO:0005198,GO:0003824,GO:0006412,GO:0019080,GO:0031016,GO:0008380,	K02953|1|3e-81|300|acs:100562063|small subunit ribosomal protein S13e	gi|4506685|ref|NP_001008.1|	2.14E-80	40S ribosomal protein S13 [Homo sapiens]
mmu-let-7e-5p	NM_027029	-28.4	0.088389	-27.08	167	GO:0012501,GO:0048232,	-	gi|22208836|ref|NP_081305.1|	6.95E-39	spermatogenesis-associated protein 3 isoform 1 [Mus musculus]

mmu-let-7e-5p	NM_027081	-26.1	0.899259	-25.02	159	-	-	gi|49355814|ref|NP_081357.2|	0	protein FAM116B [Mus musculus]
mmu-let-7e-5p	NM_027298	-27.7	0.431083	-25.75	167	GO:0043231,	K09291|1|4e-13|77.8|xtr:100496307|nucleoprotein TPR!K11498|2|3e-12|74.7|mdo:100013897|centromeric protein E	gi|226437669|ref|NP_081574.1|	0	coiled-coil domain-containing protein 89 [Mus musculus]
mmu-let-7e-5p	NM_027917	-30.2	0.241085	-28.23	171	GO:0016459,GO:0015630,GO:0003779,GO:0007015,GO:0032989,	-	gi|125628648|ref|NP_082193.2|	0	protein Shroom1 [Mus musculus]
mmu-let-7e-5p	NM_028061	-26.3	0.840256	-25.52	168	GO:0031224,GO:0043169,GO:0051234,GO:0006091,	K11318|1|5e-80|300|aml:100466161|ataxin-7!K08371|4|3e-38|162|mdo:100022046|cytochrome b-561 domain containin	gi|37805394|gb|AAH60107.1|	1.26E-97	Cyb561d1 protein, partial [Mus musculus]

mmu-let-7e-5p	NM_026917	-24.7	0.996957	-65.24	470	GO:0012505,GO:0043231,GO:0031224,GO:0046914,GO:0006497,GO:0006886,	K05766|1|8e-15|85.1|spu:575378|slingshot	gi|27754040|ref|NP_081193.2|	1.67E-161	palmitoyltransferase ZDHHC3 [Mus musculus]
mmu-let-7f-1-3p	NM_001004364	-30.2	0.335226	-26.62	175	GO:0000139,GO:0005083,GO:0046914,GO:0032012,	K12488|1|0.0|1715|mmu:211914|Arf-GAP with SH3 domain, ANK repeat and PH domain-containing protein	gi|147907212|ref|NP_001004364.2|	0	arf-GAP with SH3 domain, ANK repeat and PH domain-containing protein 2 isoform b [Mus musculus]
mmu-let-7f-1-3p	NM_001098168	-30.2	0.335704	-26.62	175	GO:0000139,GO:0005083,GO:0046914,GO:0032012,	K12488|1|0.0|1710|mmu:211914|Arf-GAP with SH3 domain, ANK repeat and PH domain-containing protein	gi|50510473|dbj|BAD32222.1|	0	mKIAA0400 protein [Mus musculus]

mmu-let-7f-1-3p	NM_001135192	-30.2	0.342043	-26.62	175	GO:0000139,GO:0005083,GO:0046914,GO:0032012,	K12488|1|0.0|1677|rno:362719|Arf-GAP with SH3 domain, ANK repeat and PH domain-containin	gi|206597526|ref|NP_001128664.1|	0	arf-GAP with SH3 domain, ANK repeat and PH domain-containing protein 2 isoform c [Mus musculus]
mmu-let-7f-1-3p	NM_001163539	-26.4	0.756085	-45.65	330	-	-	gi|254692863|ref|NP_001157011.1|	7.25E-156	uncharacterized protein CXorf21 homolog [Mus musculus]
mmu-let-7f-1-3p	NM_012003	-29	0.165332	-27.49	179	GO:0043234,GO:0031981,	K12180|1|7e-133|474|ssc:100157341|COP9 signalosome complex subunit 7	gi|148667367|gb|EDK99783.1|	2.90E-135	COP9 (constitutive photomorphogenic) homolog, subunit 7a (Arabidopsis thaliana), isoform CRA_b [Mus musculus]
mmu-let-7f-1-3p	NM_145590	-26.5	0.592751	-25.71	150	GO:0031224,	K14382|1|2e-24|88.2|bta:399680|solute carrier family 5 (sodium/glucose cotransporter), member 2	gi|225579041|ref|NP_663565.2|	0	UPF0420 protein C16orf58 homolog [Mus musculus]
mmu-let-7f-1-3p	NR_029725	-35	0.00002	-28.34	179	-	-	-	-	-

mmu-let-7f-1-3p	NR_029726	-31.6	0.000192	-28.34	179	-	-	-	-	-
mmu-let-7f-1-3p	NR_029731	-31	0.000231	-28.86	183	-	-	-	-	-
mmu-let-7f-1-3p	NR_029732	-35.6	0.000009	-28.61	183	-	-	-	-	-
mmu-let-7f-1-3p	NM_001145452	-26	0.761355	-21.44	176	-	K08878|1|2e-06|56.2|nve:NEMVE_v1g239294|breakpoint cluster region protein [EC:2.7.11.1]	gi|224282176|ref|NP_001138924.1|	0	rho guanine nucleotide exchange factor 33 [Mus musculus]
mmu-let-7f-1-3p	NM_173785	-27.9	0.53832	-24.87	173	GO:0043231,GO:0008168,GO:0006779,GO:0006730,GO:0006544,	K06980|1|0.0|705|mmu:216792|	gi|148675734|gb|EDL07681.1|	0	RIKEN cDNA A230051G13 [Mus musculus]
mmu-let-7f-1-3p	NR_029730	-30	0.000497	-24.27	171	-	-	-	-	-
mmu-let-7f-1-3p	NM_177811	-29.1	0.309681	-23.99	170	GO:0043169,GO:0003006,GO:0006355,	K09228|1|2e-162|573|mmu:328274|KRAB domain-containing zinc finger protein	gi|29244364|ref|NP_808479.1|	1.68E-161	zinc finger protein 459 [Mus musculus]

mmu-let-7f-1-3p	NM_145956	-23.2	0.998245	-20.57	169	GO:0043231,GO:0000151,GO:0043169,GO:0016790,GO:0043130,GO:0019783,GO:0006282,GO:0010212,GO:0016578,GO:00000	K11864|1|7e-144|513|mmu:210766|BRCA1/BRCA2-containing complex subunit 3	gi|12848275|dbj|BAB27894.1|	4.71E-143	unnamed protein product [Mus musculus]
mmu-let-7f-1-3p	NM_001033173	-20.6	1	-20.69	168	GO:0008233,	K11852|1|0.0|1986|mmu:76179|ubiquitin carboxyl-terminal hydrolase 31 [EC:3.1.2.15]	gi|226443061|ref|NP_001028345.1|	0	ubiquitin specific protease 31 [Mus musculus]
mmu-let-7f-1-3p	NM_026319	-25.3	0.71226	-22.55	167	GO:0031410,GO:0030990,	K12478|1|9e-14|79.7|mcc:710381|early endosome antigen 1!K11498|2|1e-13|79.3|ecb:100068227|centromeric protein E	gi|151101194|ref|NP_080595.2|	0	intraflagellar transport protein 74 homolog [Mus musculus]

mmu-let-7f-1-3p	NM_153165	-24.1	0.960523	-23.14	165	-	K10452|1|1e-120|435|mmu:236904|kelch-like protein	gi|23346619|ref|NP_694805.1|	6.84E-120	kelch-like 15 isoform c [Mus musculus]
mmu-let-7f-1-3p	NR_030693	-25.5	0.884709	-22.27	164	-	-	gi|148673719|gb|EDL05666.1|	1.05E-56	mCG145028 [Mus musculus]
mmu-let-7f-1-3p	NM_028538	-25.6	0.929897	-23.96	163	-	K09228|1|0.0|1213|mmu:338354|KRAB domain-containing zinc finger protein	gi|223462786|gb|AAI41374.1|	0	1700049G17Rik protein [Mus musculus]

mmu-let-7f-1-3p	NM_008063	-24.8	0.788369	-22.07	162	GO:0031227,GO:0015119,GO:0002262,GO:0033500,GO:0006639,GO:0001818,GO:0006073,GO:0009791,GO:0015712,GO:0002244,GO:0019318,GO:0044093,GO:0032787,GO:0009746,GO:0030595,GO:0055092,GO:0031667,GO:0006875,	K08171|1|0.0|739|mmu:14385|MFS transporter, OPA family, solute carrier family 37 (glycerol-6-phosphate transporter), member 4	gi|51592076|ref|NP_032089.2|	0	glucose-6-phosphate translocase [Mus musculus]
mmu-let-7f-1-3p	NM_001003910	-22.9	0.9993	-23.17	161	GO:0044424,	-	gi|145966853|ref|NP_001003910.2|	0	tetratricopeptide repeat protein GNN isoform 3 [Mus musculus]
mmu-let-7f-1-3p	NR_073452	-21.6	1	-21.11	159	-	-	-	-	-
mmu-let-7f-1-3p	NM_178601	-22	0.997483	-20.55	158	GO:0032991,GO:0031981,GO:0016876,GO:0032559,GO:0042254,GO:0010467,	K14561|1|2e-155|550|rno:316317|U3 small nucleolar ribonucleoprotein protein	gi|148682503|gb|EDL14450.1|	1.67E-154	IMP4, U3 small nucleolar ribonucleoprotein, homolog (yeast) [Mus musculus]

mmu-let-7f-1-3p	NM_198416	-23.6	0.999803	-22	157	GO:0043231,GO:0046914,GO:0003676,GO:0006351,	-	gi|124053447|ref|NP_940808.2|	0	ZZ-type zinc finger-containing protein 3 [Mus musculus]
mmu-let-7f-1-3p	NM_146019	-28.5	0.605754	-23.01	156	GO:0015630,GO:0044427,GO:0000118,GO:0003678,GO:0003676,GO:0046914,GO:0042623,GO:0032559,GO:0006355,GO:00063	K11642|1|0.0|3174|mmu:216848|chromodomain-helicase-DNA-binding protein 3 [EC:3.6.4.12]	gi|357527416|ref|NP_666131.3|	0	chromodomain helicase DNA binding protein 3 [Mus musculus]
mmu-let-7f-1-3p	NR_003625	-20.1	1	-20.44	153	GO:0015934,GO:0003723,GO:0042802,GO:0005198,GO:0006412,GO:0019080,GO:0031016,GO:0042254,	K02937|1|1e-70|269|cfa:477912|large subunit ribosomal protein L7e	gi|296226698|ref|XP_002759041.1|	7.42E-70	PREDICTED: 60S ribosomal protein L7-like [Callithrix jacchus]
mmu-let-7f-1-3p	NM_009412	-23.2	0.969242	-21.12	152	GO:0043231,GO:0044444,GO:0042802,GO:0046872,GO:0046983,GO:0030098,GO:00068	-	gi|6678407|ref|NP_033438.1|	8.00E-72	tumor protein D52 isoform 5 [Mus musculus]

mmu-let-7f-1-3p	NM_007540	-21.4	0.999999	-25.27	151	GO:0030136,GO:0005167,GO:0023061,GO:0033555,GO:0045597,GO:0007405,GO:0031175,GO:0007610,GO:0051899,GO:0014070,GO:0008037,GO:0033273,GO:0016202,GO:0006935,GO:0006954,GO:0035295,GO:0009719,GO:0048169,GO:0043066,GO:00	K04355|1|2e-134|481|mmu:12064|brain-derived neurotrophic factor	gi|114326463|ref|NP_031566.4|	2.03E-138	brain-derived neurotrophic factor isoform 1 precursor [Mus musculus]
mmu-let-7f-1-3p	NM_027878	-24.4	0.917721	-22.34	149	GO:0005764,GO:0031224,GO:0012501,GO:0044248,	-	gi|27229121|ref|NP_082154.2|	1.90E-134	DNA damage-regulated autophagy modulator protein 1 [Mus musculus]
mmu-let-7f-1-3p	NM_009832	-21.5	0.999827	-20.74	147	GO:0005488,GO:0006351,GO:0051726,GO:0000087,	K15188|1|1e-34|149|oaa:100081231|cyclin T	gi|397525923|ref|XP_003832897.1|	3.41E-154	PREDICTED: cyclin-K [Pan paniscus]

mmu-let-7f-1-3p	NM_173386	-23	0.998417	NA	NA	-	-	gi|116812912|ref|NP_775562.2|	0	uncharacterized protein LOC214763 [Mus musculus]
mmu-let-7f-1-3p	NM_001167751	-20.6	1	NA	NA	-	-	gi|268370061|ref|NP_001161223.1|	0	coiled-coil domain-containing protein 132 isoform 3 [Mus musculus]
mmu-let-7f-1-3p	NM_175162	-21.4	1	NA	NA	GO:0001890,	-	gi|166851842|ref|NP_001107783.1|	0	storkhead-box protein 2 isoform 1 [Mus musculus]
mmu-let-7f-1-3p	NM_015782	-21	0.997546	NA	NA	GO:0030529,GO:0031981,GO:0005488,GO:0000377,	K11091|1|3e-98|359|mmu:53607|U1 small nuclear ribonucleoprotein A	gi|12851426|dbj|BAB29037.1|	2.26E-97	unnamed protein product [Mus musculus]
mmu-let-7f-1-3p	NM_145478	-21	0.999958	NA	NA	GO:0004672,GO:0032559,GO:0000278,GO:0006915,GO:0006468,	K08807|1|5e-168|592|mmu:223775|proto-oncogene serine/threonine-protein kinase Pim-3 [EC:2.7.11.	gi|3800869|gb|AAC68900.1|	0	serine threonine kinase [Rattus norvegicus]
mmu-let-7f-1-3p	NM_174996	-22.2	0.999491	NA	NA	-	-	gi|395862204|ref|XP_003803351.1|	1.09E-72	PREDICTED: uncharacterized protein LOC100960564 [Otolemur garnettii]

mmu-let-7f-1-3p	NM_177056	-27.2	0.417286	NA	NA	-	-	gi|28892951|ref|NP_796030.1|	1.26E-106	transmembrane protein 198 [Mus musculus]
mmu-let-7f-1-3p	NM_001113180	-25.2	0.963737	NA	NA	GO:0044297,GO:0030139,GO:0033267,GO:0008328,GO:0044430,GO:0005488,GO:0004970,GO:0005231,GO:0065008,GO:0006810,GO:0009636,GO:0050806,	K05200|1|0.0|1721|mmu:14802|glutamate receptor 4	gi|164419753|ref|NP_001106651.1|	0	glutamate receptor 4 isoform 2 precursor [Mus musculus]
mmu-let-7f-1-3p	NM_029509	-21.5	0.999712	NA	NA	-	-	gi|83776551|ref|NP_083785.3|	0	guanylate binding protein 8 [Mus musculus]

mmu-let-7f-1-3p	NM_001170985	-22.7	0.999994	NA	NA	GO:0031224,GO:0030054,GO:0030425,GO:0003008,	K14319|1|5e-31|139|bfo:BRAFLDRAFT_126584|Ran GTPase-activating protein 1!K04910|2|3e-24|116|spu:578213|potassium voltage-gated channel Eag-related subfamily H member 7!K06755|4|2e-22|110|ptr:466839|roundabout, axon	gi|70608146|ref|NP_570937.2|	0	kin of IRRE-like protein 1 precursor [Mus musculus]
mmu-let-7f-1-3p	NM_028894	-20.1	1	NA	NA	GO:0046914,GO:0019538,GO:0009207,	K09553|1|7e-11|72.0|tca:655405|stress-induced-phosphoprotein 1	gi|71043955|ref|NP_083170.1|	0	LON peptidase N-terminal domain and RING finger protein 3 [Mus musculus]

mmu-let-7f-1-3p	NM_029838	-20.9	1	NA	NA	GO:0005576,GO:0005488,	K06237|1|6e-22|72.4|gga:395530|collagen, type IV, alpha	gi|38570077|ref|NP_084114.2|	0	collagen alpha-1(XXV) chain isoform 1 [Mus musculus]
mmu-let-7f-1-3p	NM_029835	-20.2	1	NA	NA	GO:0043231,GO:0005488,GO:0009314,GO:0006413,GO:0033314,	-	gi|78354983|ref|NP_084111.1|	0	treslin [Mus musculus]
mmu-let-7f-1-3p	NM_001110142	-21.1	1	NA	NA	GO:0080008,GO:0043231,GO:0019899,GO:0006259,GO:0009896,GO:0019941,GO:00070	K10609|1|0.0|1613|mmu:72584|cullin 4	gi|158635985|ref|NP_001103612.1|	0	cullin-4B [Mus musculus]
mmu-let-7f-1-3p	NM_001253739	-21.5	0.999174	NA	NA	GO:0031224,GO:0009986,GO:0004871,GO:0030246,GO:0032770,GO:0002455,GO:0031343,GO:0023052,GO:0010557,	K06468|1|3e-177|622|mmu:14128|Fc receptor, IgE, low affinity II	gi|359279906|ref|NP_001240668.1|	3.65E-180	low affinity immunoglobulin epsilon Fc receptor isoform C [Mus musculus]

mmu-let-7f-1-3p	NM_001025568	-20.8	1	NA	NA	GO:0044297,GO:0044444,GO:0047555,GO:0043169,GO:0005515,GO:0016208,GO:0007606,GO:0050796,GO:0006195,GO:0010038,GO:0010863,GO:0007169,	K13755|1|0.0|1171|mmu:18575|calcium/calmodulin-dependent 3',5'-cyclic nucleotide phosphodiesterase [EC:3.1.4.17]	gi|148672760|gb|EDL04707.1|	0	phosphodiesterase 1C, isoform CRA_f [Mus musculus]
mmu-let-7f-1-3p	NM_175692	-20.9	1	NA	NA	-	-	gi|26348733|dbj|BAC38006.1|	2.84E-55	unnamed protein product [Mus musculus]
mmu-let-7f-1-3p	NM_139149	-27.6	0.305562	NA	NA	GO:0043231,GO:0016070,GO:0010467,	K13098|1|2e-75|284|mmu:233908|RNA-binding protein FUS	gi|148685673|gb|EDL17620.1|	1.34E-74	fusion, derived from t(12;16) malignant liposarcoma (human), isoform CRA_f [Mus musculus]

mmu-let-7f-1-3p	NM_182959	-21	1	NA	NA	GO:0031224,GO:0008021,GO:0044459,GO:0015172,GO:0015291,GO:0050954,GO:0015711,GO:0030001,	K12302|1|0.0|1206|mmu:216227|MFS transporter, ACS family, solute carrier family 17 (sodium-dependent inorganic phosphate cotransporter), member 6/7/8	gi|33695072|ref|NP_892004.1|	0	vesicular glutamate transporter 3 [Mus musculus]
mmu-let-7f-1-3p	NM_008951	-23.6	0.7878	NA	NA	-	K03029|1|2e-171|602|mmu:19185|26S proteasome regulatory subunit N10	gi|6679505|ref|NP_032977.1|	1.03E-170	26S proteasome non-ATPase regulatory subunit 4 [Mus musculus]
mmu-let-7f-1-3p	NM_001033254	-26.2	0.810012	NA	NA	GO:0004672,GO:0032559,GO:0006464,	K05735|1|0.0|1364|mmu:214230|p21-activated kinase 6 [EC:2.7.11.1]	gi|224994207|ref|NP_001028426.2|	0	serine/threonine-protein kinase PAK 6 [Mus musculus]

mmu-let-7f-1-3p	NM_009183	-20.5	1	NA	NA	GO:0031228,GO:0008373,GO:0048731,GO:0006935,GO:0006487,	K06614|1|0.0|900|rno:116696|alpha-N-acetyl-neuraminate alpha-2,8-sialyltransferase (sialyltransferase 8D) [EC:2.4.99.	gi|122425617|ref|NP_446366.1|	0	CMP-N-acetylneuraminate-poly-alpha-2,8-sialyltransferase [Rattus norvegicus]
mmu-let-7f-1-3p	NM_001081223	-21.8	0.999954	NA	NA	GO:0003677,GO:0004520,GO:0022402,GO:0001824,GO:0000729,	-	gi|124487005|ref|NP_001074692.1|	0	DNA endonuclease RBBP8 [Mus musculus]
mmu-let-7f-1-3p	NM_028874	-20.1	1	NA	NA	-	-	gi|28972119|dbj|BAC65513.1|	0	mKIAA0254 protein [Mus musculus]
mmu-let-7f-1-3p	NM_001253753	-24.6	0.985101	NA	NA	GO:0043231,GO:0046914,	K02981|1|6e-39|149|ptr:456848|small subunit ribosomal protein S2e	gi|140972030|ref|NP_659091.3|	0	zinc finger MYM-type protein 5 [Mus musculus]

mmu-let-7f-1-3p	NM_011630	-21.7	1	NA	NA	-	K08544|1|0.0|1179|cfa:484641|testicular receptor 4	gi|912822|gb|AAB33314.1|	0	type II zinc finger DNA binding transcription factor [Mus sp.]
mmu-let-7f-1-3p	NR_027824	-21.8	0.985678	NA	NA	-	-	gi|74217401|dbj|BAC38306.2|	4.69E-53	unnamed protein product [Mus musculus]
mmu-let-7f-1-3p	NR_045546	-20.2	1	NA	NA	GO:0043231,GO:0046914,GO:0003676,GO:0010467,	K09228|1|6e-34|147|dre:796272|KRAB domain-containing zinc finger protein	gi|21450221|ref|NP_659082.1|	0	zinc finger protein 410 isoform 1 [Mus musculus]
mmu-let-7f-1-3p	NM_080850	-20.4	1	NA	NA	GO:0043231,GO:0004672,GO:0060089,GO:0005515,GO:0032559,GO:0023052,GO:0006351,GO:0006464,	K08801|1|0.0|2635|mmu:269224|PAS domain containing serine/threonine kinase [EC:2.7.11.1]	gi|37359790|dbj|BAC97873.1|	0	mKIAA0135 protein [Mus musculus]

mmu-let-7f-1-3p	NM_028279	-20.1	1	NA	NA	GO:0031224,GO:0043169,GO:0008238,GO:0019538,GO:0042133,	K01301|1|0.0|1501|mmu:72560|glutamate carboxypeptidase II [EC:3.4.17.21]	gi|148693094|gb|EDL25041.1|	0	N-acetylated alpha-linked acidic dipeptidase 2, isoform CRA_a [Mus musculus]
mmu-let-7f-1-3p	NM_013504	-20	1	NA	NA	GO:0005911,GO:0031224,GO:0046872,GO:0016337,	K07600|1|0.0|1626|mmu:13505|desmocollin 1	gi|161016841|ref|NP_038532.3|	0	desmocollin-1 precursor [Mus musculus]
mmu-let-7f-1-3p	NM_183225	-20.7	1	NA	NA	GO:0016790,GO:0070011,GO:0019941,	K11840|1|0.0|4787|mmu:329908|ubiquitin carboxyl-terminal hydrolase 9/24 [EC:3.1.2.15]	gi|260064007|ref|NP_899048.2|	0	ubiquitin carboxyl-terminal hydrolase 24 [Mus musculus]
mmu-let-7f-1-3p	NM_026438	-27.7	0.199279	NA	NA	GO:0044444,GO:0046872,GO:0016462,GO:0006412,GO:0006796,	K01507|1|1e-174|613|mmu:67895|inorganic pyrophosphatase [EC:3.6.1.1	gi|148700191|gb|EDL32138.1|	0	pyrophosphatase (inorganic) 1 [Mus musculus]

mmu-let-7f-1-3p	NM_018778	-20.7	0.999988	NA	NA	GO:0043296,GO:0043231,GO:0031224,GO:0005515,GO:0016337,	K06087|1|1e-114|414|mmu:54420|claudin	gi|9055192|ref|NP_061248.1|	8.88E-114	claudin-8 [Mus musculus]
mmu-let-7f-1-3p	NM_177561	-20.2	1	NA	NA	GO:0019783,GO:0005488,GO:0016790,GO:0070646,GO:0050896,GO:0019941,GO:0050804,	K11842|1|0.0|708|bta:516420|ubiquitin carboxyl-terminal hydrolase 12/46 [EC:3.1.2.15]	gi|29243896|ref|NP_808229.1|	0	ubiquitin carboxyl-terminal hydrolase 46 [Mus musculus]
mmu-let-7f-1-3p	NM_001104625	-22.2	0.997824	NA	NA	-	K04613|1|0.0|1512|mmu:211223|vomeronasal 2 receptor	gi|157277977|ref|NP_001098095.1|	0	vomeronasal 2, receptor 14 precursor [Mus musculus]
mmu-let-7f-1-3p	NM_172574	-23.7	0.90467	NA	NA	GO:0031224,	K09660|1|2e-08|61.6|ame:410429|mannose-P-dolichol utilization defect 1	gi|238550113|ref|NP_766162.2|	7.21E-103	PQ-loop repeat-containing protein 3 isoform 1 precursor [Mus musculus]

mmu-let-7f-1-3p	NM_026214	-20.6	1	NA	NA	GO:0005887,GO:0005267,GO:0034220,GO:0030001,	K15074|1|1e-12|76.6|mmu:330171|BTB/POZ domain-containing adapter for CUL3-mediated RhoA degradation protein	gi|50980305|ref|NP_080490.3|	1.37E-138	BTB/POZ domain-containing protein KCTD4 [Mus musculus]
mmu-let-7f-1-3p	NM_021450	-21.1	1	NA	NA	GO:0031224,GO:0031252,GO:0008092,GO:0043169,GO:0004672,GO:0004871,GO:0005261,GO:0032559,GO:0006468,GO:0007160,GO:0023052,GO:0030036,GO:00068	K04982|1|0.0|3664|mmu:58800|transient receptor potential cation channel subfamily M member 7 [EC:2.7.11.1]	gi|256773285|ref|NP_067425.2|	0	transient receptor potential cation channel subfamily M member 7 isoform 1 [Mus musculus]

mmu-let-7f-1-3p	NM_022022	-20.2	1	NA	NA	GO:0043231,GO:0043234,GO:0004842,GO:0005515,GO:0012501,GO:0055010,GO:0030030,GO:0009416,GO:0033554,GO:0019941,GO:0016567,	K10597|1|0.0|2051|mmu:63958|ubiquitin conjugation factor E4 B [EC:6.3.2.19]	gi|28972347|dbj|BAC65627.1|	0	mKIAA0684 protein [Mus musculus]
mmu-let-7f-1-3p	NM_001085521	-23.3	0.952384	NA	NA	GO:0005769,GO:0031224,GO:0050896,	-	gi|147903397|ref|NP_001078990.1|	1.16E-130	synapse differentiation-inducing gene protein 1 [Mus musculus]
mmu-let-7f-1-3p	NM_001160182	-24.6	0.87682	NA	NA	GO:0031224,GO:0031090,GO:0043231,GO:0005488,	K11794|1|3e-71|270|mcc:716691|IFRG15 protein	gi|11612513|ref|NP_071724.1|	4.59E-71	interferon alpha responsive protein isoform a [Mus musculus]
mmu-let-7f-1-3p	NM_008569	-21.6	1	NA	NA	GO:0031981,GO:0044444,GO:0000152,GO:0005488,GO:0043161,GO:0007050,GO:0045841,GO:0051439,	K03348|1|0.0|3780|mmu:17222|anaphase-promoting complex subunit 1	gi|228008323|ref|NP_032595.2|	0	anaphase-promoting complex subunit 1 [Mus musculus]

mmu-let-7f-1-3p	NM_027478	-20	0.999985	NA	NA	GO:0031224,	-	gi|348581376|ref|XP_003476453.1|	1.49E-42	PREDICTED: UPF0414 transmembrane protein C20orf30-like [Cavia
mmu-let-7f-1-3p	NM_001168497	-20.4	1	NA	NA	GO:0031224,GO:0005548,GO:0046872,GO:0019904,GO:0055092,GO:0033500,GO:0007009,	K08885|1|2e-40|110|mmu:83813|non-receptor tyrosine-protein kinase TNK1 [EC:2.7.10.2]	gi|74196739|dbj|BAE43107.1|	3.33E-118	unnamed protein product [Mus musculus]
mmu-let-7f-1-3p	NM_207654	-23.4	0.998972	NA	NA	GO:0044459,GO:0031225,GO:0044421,GO:0005102,GO:0007411,GO:0007417,GO:00071	K05462|1|1e-125|452|aml:100465489|ephrin-A	gi|46560570|ref|NP_997537.1|	1.44E-125	ephrin-A5 isoform 1 precursor [Mus musculus]
mmu-let-7f-1-3p	NM_009616	-20.5	1	NA	NA	GO:0031224,GO:0004175,GO:0046914,GO:0019904,GO:0007166,GO:0033619,GO:0048513,	K08608|1|0.0|1703|mmu:11492|disintegrin and metalloproteinase domain-containing protein 19 [EC:3.4.24.-]	gi|6752966|ref|NP_033746.1|	0	disintegrin and metalloproteinase domain-containing protein 19 preproprotein [Mus musculus]

mmu-let-7f-1-3p	NM_019691	-25.2	0.963737	NA	NA	GO:0044297,GO:0030139,GO:0033267,GO:0008328,GO:0044430,GO:0005488,GO:0004970,GO:0005231,GO:0065008,GO:0006810,GO:0009636,GO:0007166,GO:00508	K05200|1|0.0|1756|aml:100464250|glutamate receptor 4	gi|164419751|ref|NP_062665.3|	0	glutamate receptor 4 isoform 1 precursor [Mus musculus]
mmu-let-7f-1-3p	NM_001159627	-21	1	NA	NA	GO:0031224,GO:0044444,GO:0046914,GO:0003824,GO:0030099,GO:0008152,GO:0000041,GO:0006875,	K14735|1|0.0|2287|mmu:15203|hephaestin	gi|148682267|gb|EDL14214.1|	0	hephaestin, isoform CRA_a [Mus musculus]

mmu-let-7f-1-3p	NM_010655	-22	0.996069	NA	NA	GO:0031981,GO:0005635,GO:0022892,GO:0005048,GO:0019899,GO:0006310,GO:0051704,GO:0006606,GO:0000279,GO:0051319,GO:0045990,	K15043|1|0.0|1014|mmu:16647|importin subunit alpha-2	gi|6754474|ref|NP_034785.1|	0	importin subunit alpha-2 [Mus musculus]
mmu-let-7f-1-3p	NM_001164200	-24	0.988465	NA	NA	GO:0031981,GO:0043234,GO:0044444,GO:0004690,GO:0046872,GO:0032559,GO:0015980,GO:0007599,GO:0033762,GO:0045786,GO:0032147,GO:0010863,GO:0007169,GO:0006006,GO:0030073,GO:0006641,GO:0007187,GO:0042044,	K04345|1|0.0|762|cfa:479975|protein kinase A [EC:2.7.11.11]	gi|255958156|ref|NP_001157672.1|	0	cAMP-dependent protein kinase catalytic subunit beta isoform 4 [Mus musculus]

mmu-let-7f-1-3p	NM_026752	-23.9	0.756086	NA	NA	GO:0005924,GO:0031410,GO:0046914,	K04679|1|9e-15|82.0|bfo:BRAFLDRAFT_91658|MAD, mothers against decapentaplegic interacting protein	gi|326937469|ref|NP_081028.3|	1.06E-133	zinc finger FYVE domain-containing protein 21 [Mus musculus]
mmu-let-7f-1-3p	NR_040297	-20.3	1	NA	NA	-	-	gi|68073695|ref|XP_678762.1|	2.44E-23	Pb-fam-2 protein [Plasmodium berghei strain ANKA]

mmu-let-7f-1-3p	NM_007499	-22.7	1	NA	NA	GO:0031410,GO:0031981,GO:0044427,GO:0015630,GO:0004674,GO:0032559,GO:0005515,GO:0003676,GO:0035004,GO:0045841,GO:0010332,GO:0042157,GO:0009967,GO:0016572,GO:0002329,GO:0046488,GO:0000077,GO:0030330,GO:0007276,GO:0030888,GO:0009952,GO:00009	K04728|1|0.0|5925|mmu:11920|ataxia telangiectasia mutated family protein [EC:2.7.11.1]	gi|163838660|ref|NP_031525.2|	0	serine-protein kinase ATM [Mus musculus]
mmu-let-7f-1-3p	NM_008664	-24.3	0.988358	NA	NA	GO:0016459,GO:0005198,GO:0003012,	K12567|1|4e-71|271|spu:590007|titin [EC:2.7.11.1]	gi|170763465|ref|NP_032690.2|	0	myomesin 2 [Mus musculus]
mmu-let-7f-1-3p	NR_027803	-20.1	1	NA	NA	-	-	gi|148678715|gb|EDL10662.1|	7.26E-46	mCG1027169, isoform CRA_a [Mus musculus]

mmu-let-7f-1-3p	NM_013463	-21.2	0.999986	NA	NA	GO:0000323,GO:0048029,GO:0042802,GO:0043167,GO:0015925,GO:0001503,GO:0006687,GO:0016137,GO:0006677,GO:0031327,GO:0032769,	K01189|1|0.0|893|mmu:11605|alpha-galactosidase [EC:3.2.1.22]	gi|133778924|ref|NP_038491.2|	0	alpha-galactosidase A [Mus musculus]
mmu-let-7f-1-3p	NM_026175	-25.4	0.937113	NA	NA	GO:0005681,GO:0031981,GO:0003676,GO:0006376,	K12825|1|0.0|1134|mmu:67465|splicing factor 3A	gi|26324776|dbj|BAC26142.1|	0	unnamed protein product [Mus musculus]
mmu-let-7f-1-3p	NM_009426	-21.4	0.992975	NA	NA	GO:0016023,GO:0016020,GO:0005179,GO:0050796,GO:0008344,GO:0009746,GO:0014051,GO:0014047,GO:0051384,GO:0007165,GO:0006950,GO:0007631,	K05253|1|3e-127|455|mmu:22044|thyrotropin-releasing hormone	gi|134031937|ref|NP_033452.2|	2.32E-126	prothyroliberin precursor [Mus musculus]

mmu-let-7f-1-3p	NM_001253745	-21.5	0.99896	NA	NA	GO:0031224,GO:0009986,GO:0004871,GO:0030246,GO:0032770,GO:0002455,GO:0031343,GO:0023052,GO:0010557,	K06468|1|3e-161|569|mmu:14128|Fc receptor, IgE, low affinity II	gi|359279919|ref|NP_001240674.1|	1.52E-167	low affinity immunoglobulin epsilon Fc receptor isoform E [Mus musculus]
mmu-let-7f-1-3p	NM_001160268	-21.2	1	NA	NA	-	K03426|1|2e-07|60.5|ssc:100512459|NAD+ diphosphatase [EC:3.6.1.22]!K06082|2|8e-06|55.1|tgu:100221501|FERM, RhoGEF and pleckstrin domain protein 2	gi|50510733|dbj|BAD32352.1|	0	mKIAA0969 protein [Mus musculus]
mmu-let-7f-1-3p	NM_028905	-22.7	0.994112	NA	NA	-	-	gi|19920334|ref|NP_083181.1|	6.42E-124	uncharacterized protein C21orf62 homolog precursor [Mus musculus]
mmu-let-7f-1-3p	NR_015580	-22.9	0.994235	NA	NA	-	-	gi|148703101|gb|EDL35048.1|	3.77E-56	mCG142516, isoform CRA_b [Mus musculus]

mmu-let-7f-1-3p	NM_001166027	-20.8	0.998726	NA	NA	GO:0044464,	K06712|1|3e-10|67.0|mmu:100038862|butyrophilin	gi|260447054|ref|NP_001159499.1|	0	selection and upkeep of intraepithelial T-cells protein 11 isoform 1 precursor [Mus musculus]
mmu-let-7f-1-3p	NM_001012310	-23.3	0.904426	NA	NA	GO:0019866,GO:0031224,GO:0043231,GO:0006810,	K15123|1|4e-145|515|mmu:104910|solute carrier family 25, member 45/47	gi|32822820|gb|AAH55027.1|	6.71E-152	AI132487 protein, partial [Mus musculus]
mmu-let-7f-1-3p	NM_182930	-21.2	1	NA	NA	-	K03426|1|3e-09|66.6|ssc:100512459|NAD+ diphosphatase [EC:3.6.1.22]!K06082|2|2e-08|63.5|tgu:100221501|FERM, RhoGEF and pleckstrin domain protein 2	gi|148707707|gb|EDL39654.1|	0	pleckstrin homology domain containing, family A member 6, isoform CRA_a [Mus musculus]

mmu-let-7f-1-3p	NM_177566	-23.7	0.993162	NA	NA	GO:0043005,GO:0008047,GO:0005488,GO:0005088,GO:0048523,GO:0043087,GO:0007266,	K07525|1|2e-64|248|xtr:100487663|neuronal guanine nucleotide exchange	gi|60279700|ref|NP_808234.2|	0	rho guanine nucleotide exchange factor 15 [Mus musculus]
mmu-let-7f-1-3p	NM_001160713	-21.3	0.999916	NA	NA	GO:0031224,GO:0043231,GO:0004871,GO:0023052,	K06719|1|3e-23|111|mcc:702540|CD300 antigen	gi|148702134|gb|EDL34081.1|	0	CD300 antigen like family member G, isoform CRA_a [Mus musculus]
mmu-let-7f-1-3p	NM_001159960	-20.8	1	NA	NA	GO:0044297,GO:0044444,GO:0047555,GO:0043169,GO:0005515,GO:0016208,GO:0007606,GO:0050796,GO:0006195,GO:0010038,GO:0010863,GO:0007169,	K13755|1|0.0|1171|mmu:18575|calcium/calmodulin-dependent 3',5'-cyclic nucleotide phosphodiesterase [EC:3.1.4.17]	gi|148672760|gb|EDL04707.1|	0	phosphodiesterase 1C, isoform CRA_f [Mus musculus]

mmu-let-7f-2-3p	NM_001025444	-25.4	0.92425	-48.06	314	GO:0031981,GO:0000785,GO:0003723,GO:0046914,GO:0035312,GO:0005515,GO:0019204,GO:0043566,GO:0010608,GO:0006281,GO:0000302,GO:0006796,	K10863|1|0.0|689|mmu:66408|aprataxin [EC:3.-.-.-]	gi|48734599|gb|AAH71213.1|	0	Aptx protein, partial [Mus musculus]
mmu-let-7f-2-3p	NM_001029889	-26.9	0.945083	-26.39	167	GO:0043231,GO:0046872,GO:0003676,GO:0015924,	K10955|1|1e-07|62.0|gga:423101|intestinal mucin-2	gi|148665619|gb|EDK98035.1|	0	gene model 608, (NCBI), isoform CRA_b [Mus musculus]
mmu-let-7f-2-3p	NM_001085355	-26.8	0.935938	-25.26	168	GO:0031981,GO:0070603,GO:0005488,GO:0003712,GO:0006338,	K11653|1|0.0|2711|mmu:239985|AT-rich interactive domain-containing protein	gi|145553997|ref|NP_001078824.1|	0	AT rich interactive domain 1B (Swi1 like) [Mus musculus]

mmu-let-7f-2-3p	NM_025545	-25.4	0.928814	-48.06	314	GO:0031981,GO:0000785,GO:0003723,GO:0046914,GO:0035312,GO:0005515,GO:0019204,GO:0043566,GO:0010608,GO:0006281,GO:0000302,GO:0006796,	K10863|1|0.0|689|mmu:66408|aprataxin [EC:3.-.-.-]	gi|32526865|ref|NP_079821.3|	0	aprataxin isoform a [Mus musculus]
mmu-let-7f-2-3p	NM_175210	-29.8	0.446647	-28.07	183	GO:0031224,GO:0017111,GO:0032559,GO:0010876,GO:0051234,GO:0009987,	K05646|1|0.0|5073|mmu:74591|ATP-binding cassette, subfamily A (ABC1), member 12	gi|225703056|ref|NP_780419.2|	0	ATP-binding cassette, sub-family A (ABC1), member 12 [Mus musculus]
mmu-let-7f-2-3p	NR_027778	-28.3	0.158633	-28.28	171	GO:0044444,GO:0043169,GO:0016702,GO:0006558,GO:0006570,	K00451|1|0.0|823|mmu:15233|homogentisate 1,2-dioxygenase [EC:1.13.1	gi|148665546|gb|EDK97962.1|	0	homogentisate 1,2-dioxygenase, isoform CRA_b [Mus musculus]

mmu-let-7f-2-3p	NR_033229	-30.3	0.340841	-26.23	175	GO:0043231,GO:0043234,GO:0044444,GO:0003712,GO:0019787,GO:0005488,GO:0000209,GO:0006357,GO:0003006,GO:0030518,GO:0022602,GO:0009967,GO:0006897,GO:00	K10587|1|3e-35|152|mmu:22215|ubiquitin-protein ligase E3A [EC:6.3.2.19]	gi|354498222|ref|XP_003511214.1|	2.14E-34	PREDICTED: ubiquitin-protein ligase E3A-like [Cricetulus griseus]
mmu-let-7f-2-3p	NM_175204	-20.7	1	-43.07	302	GO:0043231,GO:0000502,GO:0004175,GO:0043161,GO:0051320,GO:0007050,GO:0016070,GO:0051439,GO:0006520,GO:0006915,GO:0030330,	K11598|1|2e-171|604|mmu:73902|20S proteasome subunit beta 11 [EC:3.4.25.1]	gi|30424826|ref|NP_780413.1|	1.53E-170	proteasome subunit beta type-11 precursor [Mus musculus]

mmu-let-7f-2-3p	NM_018739	-25.2	0.398474	-20.3	179	GO:0030867,GO:0016604,GO:0046914,GO:0050953,GO:0006396,	-	gi|74222240|dbj|BAE26927.1|	2.60E-103	unnamed protein product [Mus musculus]
mmu-let-7f-2-3p	NM_028273	-25.5	0.591	-20.37	175	GO:0031224,GO:0031966,GO:0016791,GO:0006796,	K15637|1|7e-154|545|mmu:72542|serine/threonine-protein phosphatase PGAM5 [EC:3.1.3.16]	gi|148688083|gb|EDL20030.1|	9.79E-162	phosphoglycerate mutase family member 5, isoform CRA_a [Mus musculus]
mmu-let-7f-2-3p	NM_009784	-25.3	0.970289	-20.69	174	GO:0034704,GO:0042383,GO:0005783,GO:0043169,GO:0005262,GO:0032412,GO:0006816,GO:0010959,	K04858|1|0.0|2150|mmu:12293|voltage-dependent calcium channel alpha-2/delta-1	gi|6753234|ref|NP_033914.1|	0	voltage-dependent calcium channel subunit alpha-2/delta-1 isoform e precursor [Mus musculus]

mmu-let-7f-2-3p	NM_008594	-27.7	0.279772	-22.01	172	GO:0019898,GO:0009986,GO:0031410,GO:0044421,GO:0005543,GO:0005102,GO:0048545,GO:0045807,GO:0006909,GO:0048646,GO:0009566,GO:0008283,	K03899|1|9e-70|265|tgu:100223378|coagulation factor VIII!K03902|3|4e-69|263|mmu:14067|coagulation factor V (labile factor)	gi|113865979|ref|NP_032620.2|	0	lactadherin isoform 1 precursor [Mus musculus]
mmu-let-7f-2-3p	NM_026778	-25	0.437483	-23.68	170	GO:0031012,GO:0001664,GO:0035567,GO:0043393,GO:0030030,GO:0048870,GO:0030178,GO:0042472,GO:0001736,	K13649|1|4e-17|89.7|bfo:BRAFLDRAFT_123559|folate receptor!K06823|3|6e-07|55.8|acs:100562332|collagen, type XVIII, alpha!K06237|4|9e-06|52.0|xtr:100492984|collagen, type IV, alpha	gi|149066481|gb|EDM16354.1|	1.89E-139	collagen triple helix repeat containing 1 [Rattus norvegicus]

mmu-let-7f-2-3p	NM_010570	-23.7	0.999734	-23.16	167	GO:0043231,GO:0005887,GO:0044444,GO:0004714,GO:0005102,GO:0019904,GO:0019901,GO:0030674,GO:0010467,GO:0032868,GO:0008284,GO:0048017,GO:0048732,GO:0010828,GO:0007169,GO:0007243,GO:0009967,GO:0005979,GO:0016477,GO:0033500,GO:0009968,GO:00435	K07187|1|0.0|1994|mmu:16367|insulin receptor substrate	gi|29825829|ref|NP_034700.2|	0	insulin receptor substrate 1 [Mus musculus]
mmu-let-7f-2-3p	NR_045377	-26	0.165413	-23.44	166	-	-	gi|148688038|gb|EDL19985.1|	1.16E-24	mCG144681 [Mus musculus]

mmu-let-7f-2-3p	NR_027492	-24.5	0.946267	-21.88	165	GO:0043231,GO:0032559,	K09491|1|0.0|872|mmu:110920|stress 70 protein chaperone microsome-associated 60kDa	gi|30089677|ref|NP_084477.1|	0	heat shock 70 kDa protein 13 precursor [Mus musculus]
mmu-let-7f-2-3p	NR_045003	-26.5	0.54926	-21.26	164	-	-	gi|40556843|gb|AAR87779.1|	9.52E-33	unknown [Mus musculus]
mmu-let-7f-2-3p	NR_001461	-20.8	1	-22.25	162	-	K11849|1|6e-55|213|acs:100555596|ubiquitin carboxyl-terminal hydrolase 25/28 [EC:3.1.2.15]!K06478|4|4e-43|182|acs:100563672|protein tyrosine phosphatase, receptor type, C [EC:3.1.3.4	gi|209553980|gb|AAA39398.2|	0	ORF2 [Mus musculus domesticus]

mmu-let-7f-2-3p	NM_177140	-21.4	0.973053	-20.45	161	GO:0031224,	-	gi|354489678|ref|XP_003506988.1|	1.55E-89	PREDICTED: UPF0694 transmembrane protein C14orf109 homolog [Cricetulus griseus]
mmu-let-7f-2-3p	NM_177099	-22.5	0.988818	-20.43	160	-	K04668|1|0.0|692|xla:100505435|left-right determination	gi|148681196|gb|EDL13143.1|	0	mCG19828 [Mus musculus]
mmu-let-7f-2-3p	NM_145229	-25.4	0.217648	-20.29	159	-	-	gi|21644595|ref|NP_660264.1|	4.06E-46	cleft palate-related protein 1 [Mus musculus]
mmu-let-7f-2-3p	NR_040466	-20.8	0.999822	-22.53	158	GO:0043234,GO:0043232,GO:0005488,GO:0005198,GO:0048468,GO:0050953,GO:0007010,GO:0021700,	K10379|1|5e-31|137|mmu:107993|beaded filament structural protein 2, phakinin	gi|148689128|gb|EDL21075.1|	4.78E-74	mCG145984 [Mus musculus]

mmu-let-7f-2-3p	NM_175194	-23.3	0.978089	-20.11	157	GO:0019866,GO:0043231,GO:0031224,GO:0015297,GO:0006766,GO:0006732,GO:0006810,GO:0006575,	K15084|1|2e-179|630|mmu:73132|solute carrier family 25 (mitochondrial carrier protein), member 16	gi|30424808|ref|NP_780403.1|	1.42E-178	graves disease carrier protein homolog [Mus musculus]
mmu-let-7f-2-3p	NM_030066	-22.2	0.993226	-20.01	155	GO:0031224,	-	gi|148688430|gb|EDL20377.1|	0	armadillo repeat containing, X-linked 1, isoform CRA_a [Mus musculus]
mmu-let-7f-2-3p	NR_038022	-20.3	1	-21.55	154	-	-	gi|148671508|gb|EDL03455.1|	7.75E-43	mCG144972 [Mus musculus]
mmu-let-7f-2-3p	NM_029936	-22.3	0.998372	-20.75	153	GO:0003676,GO:0004386,GO:0032559,GO:0042623,	K14776|1|0.0|1502|mmu:77591|ATP-dependent RNA helicase DDX10/DBP4 [EC:3.6.4.13]	gi|29351650|gb|AAH49261.1|	0	Ddx10 protein, partial [Mus musculus]

mmu-let-7f-2-3p	NR_040359	-20.6	0.999016	-21.27	150	GO:0031982,GO:0043231,GO:0044444,GO:0016020,GO:0046872,GO:0019787,GO:0032446,	K10657|1|6e-11|69.7|mmu:224703|E3 ubiquitin-protein ligase MARCH2 [EC:6.3.2.19]	gi|148674327|gb|EDL06274.1|	1.43E-34	mCG141217 [Mus musculus]
mmu-let-7f-2-3p	NM_001081242	-20.1	1	-20.52	149	GO:0005856,GO:0005924,GO:0031252,GO:0008092,GO:0005102,GO:0005198,GO:0034329,GO:0007010,	K06271|1|0.0|4323|mmu:70549|talin	gi|163310736|ref|NP_001074711.2|	0	talin-2 [Mus musculus]
mmu-let-7f-2-3p	NM_153103	-20	1	-21.43	148	GO:0005875,GO:0043231,GO:0003774,GO:0032559,GO:0048193,GO:0007017,	K10392|1|0.0|1836|mmu:16562|kinesin family member 1/13/14	gi|50510599|dbj|BAD32285.1|	0	mKIAA0706 protein [Mus musculus]
mmu-let-7f-2-3p	NR_037998	-20	1	-20.52	146	-	-	gi|148682404|gb|EDL14351.1|	2.60E-59	mCG145226 [Mus musculus]

mmu-let-7f-2-3p	NM_133643	NA	NA	-20.55	154	GO:0044424,GO:0004871,GO:0032813,GO:0042476,GO:0008544,GO:0048732,GO:0007243,GO:0048869,	-	gi|127139913|ref|NP_598398.3|	4.26E-116	ectodysplasin-A receptor-associated adapter protein isoform 1 [Mus musculus]
mmu-let-7f-2-3p	NM_029296	NA	NA	-25.53	152	-	-	gi|58037411|ref|NP_083572.1|	3.06E-112	uncharacterized protein LOC75462 isoform 1 [Mus musculus]
mmu-let-7f-2-3p	NM_010175	NA	NA	-20.52	149	GO:0042995,GO:0031264,GO:0044444,GO:0032813,GO:0009612,GO:0001819,GO:0051259,GO:0007249,	K02373|1|2e-84|314|mmu:14082|FAS (TNFRSF6)-associated via death domain	gi|26327281|dbj|BAC27384.1|	1.57E-83	unnamed protein product [Mus musculus]
mmu-let-7f-2-3p	NM_009398	NA	NA	-20.62	146	GO:0005539,GO:0006952,GO:0007154,GO:0023052,	K14616|1|5e-21|103|dre:100144772|cubilin	gi|6678379|ref|NP_033424.1|	2.29E-160	tumor necrosis factor-inducible gene 6 protein precursor [Mus musculus]
mmu-let-7f-2-3p	NM_010893	NA	NA	-20.43	145	GO:0005764,GO:0016020,GO:0031410,GO:0016997,GO:0005488,	K01186|1|0.0|819|mmu:18010|sialidase-1 [EC:3.2.1.18]	gi|254939721|ref|NP_035023.3|	0	sialidase-1 precursor [Mus musculus]

mmu-let-7f-2-3p	NM_001122899	-22.8	0.999661	NA	NA	GO:0016021,GO:0044421,GO:0042277,GO:0004930,GO:0022602,GO:0048545,GO:0048646,GO:0031667,GO:0019221,GO:0016125,GO:0014070,GO:0043086,GO:0010740,GO:0044237,GO:0006950,GO:00076	K05062|1|0.0|1850|mmu:16847|leptin receptor	gi|148698917|gb|EDL30864.1|	0	leptin receptor, isoform CRA_b [Mus musculus]
mmu-let-7f-2-3p	NM_025778	-21.3	0.999038	NA	NA	GO:0043226,GO:0044444,GO:0005488,GO:0006915,	K03211|1|9e-11|69.7|mgp:100542717|ETS translocation variant	gi|38194232|ref|NP_080054.1|	3.64E-170	apoptosis facilitator Bcl-2-like protein 14 [Mus musculus]
mmu-let-7f-2-3p	NM_013835	-20.2	1	NA	NA	GO:0032991,GO:0031981,GO:0003676,GO:0006351,	K11089|1|0.0|1073|mmu:20822|60 kDa SS-A/Ro ribonucleoprotein	gi|7305523|ref|NP_038863.1|	0	60 kDa SS-A/Ro ribonucleoprotein [Mus musculus]

mmu-let-7f-2-3p	NM_008385	-21.9	0.999817	NA	NA	GO:0005856,GO:0031224,GO:0044444,GO:0008047,GO:0046030,GO:0034593,GO:0048870,GO:0035556,GO:0009966,GO:00482	K01099|1|0.0|1988|mmu:16330|phosphatidylinositol-bisphosphatase [EC:3.1.3.36]	gi|30231213|ref|NP_032411.3|	0	type II inositol 1,4,5-trisphosphate 5-phosphatase [Mus musculus]

mmu-let-7f-2-3p	NM_008510	-25.5	0.13124	NA	NA	GO:0044421,GO:0005125,GO:0042802,GO:0001664,GO:0010524,GO:0046631,GO:0023052,GO:0002369,GO:0001912,GO:0043392,GO:0031347,GO:0001819,GO:0002690,GO:0042130,GO:0002702,GO:0051283,GO:0007154,GO:0003013,GO:0002823,GO:0001818,GO	K05507|1|1e-42|172|mmu:16963|C motif chemokine 1/2	gi|6678712|ref|NP_032536.1|	5.70E-42	lymphotactin precursor [Mus musculus]

mmu-let-7f-2-3p	NM_153080	-27	0.686919	NA	NA	GO:0044464,GO:0015031,	K12404|1|5e-21|105|pon:100172741|ADP-ribosylation factor-binding protein GGA!K04705|3|7e-20|101|nvi:100119432|signal transducing adaptor	gi|84875530|ref|NP_694720.2|	0	TOM1-like protein 2 isoform a [Mus musculus]
mmu-let-7f-2-3p	NM_178901	-22.7	0.955138	NA	NA	GO:0031224,	-	gi|30725748|ref|NP_849232.1|	2.46E-111	transmembrane protein C16orf54 homolog [Mus musculus]
mmu-let-7f-2-3p	NM_001165955	-21.5	1	NA	NA	GO:0005488,	K11458|1|0.0|1202|mmu:241915|polyhomeotic-like protein 3	gi|260099711|ref|NP_001159426.1|	0	polyhomeotic-like protein 3 isoform 1 [Mus musculus]
mmu-let-7f-2-3p	NM_009990	-20.9	1	NA	NA	GO:0043005,GO:0005874,GO:0030141,GO:0008017,GO:0007019,	K10422|1|0.0|1788|rno:29264|CAP-Gly domain-containing linker protein 2	gi|148687481|gb|EDL19428.1|	0	cytoplasmic linker 2, isoform CRA_c [Mus musculus]

mmu-let-7f-2-3p	NM_144549	-20.6	1	NA	NA	GO:0043231,GO:0004871,GO:0016301,GO:0019901,GO:0055103,GO:0032559,GO:0019887,GO:0014909,GO:0002832,GO:0033673,GO:0031398,GO:0043392,GO:0008285,GO:0009966,GO:0031331,GO:0002237,GO:00	K08814|1|0.0|637|mmu:211770|tribbles [EC:2.7.11.-]	gi|21362355|ref|NP_653132.1|	1.17E-180	tribbles homolog 1 [Mus musculus]
mmu-let-7f-2-3p	NM_080451	-20.2	1	NA	NA	-	-	gi|226958463|ref|NP_536699.2|	0	synaptopodin-2 [Mus musculus]
mmu-let-7f-2-3p	NR_027710	-20.2	1	NA	NA	-	K07202|1|0.0|584|mmu:19017|peroxisome proliferative activated receptor, gamma, coactivator 1, alpha	gi|45219869|gb|AAH66868.1|	0	Peroxisome proliferative activated receptor, gamma, coactivator 1 alpha [Mus musculus]

mmu-let-7f-2-3p	NM_173785	-20.7	1	NA	NA	GO:0043231,GO:0008168,GO:0006779,GO:0006730,GO:0006544,	K06980|1|0.0|705|mmu:216792|	gi|148675734|gb|EDL07681.1|	0	RIKEN cDNA A230051G13 [Mus musculus]
mmu-let-7f-2-3p	NM_001018031	-23.3	0.928544	NA	NA	-	K07296|1|1e-40|168|oaa:100075105|adiponectin	gi|260166678|ref|NP_001018041.2|	0	otolin-1 precursor [Mus musculus]
mmu-let-7f-2-3p	NM_147221	-21.8	0.999133	NA	NA	GO:0043231,GO:0003676,GO:0046914,GO:0006357,	K09232|1|0.0|1283|mmu:230587|zinc finger protein GLIS1/3	gi|124377993|ref|NP_671754.2|	0	zinc finger protein GLIS1 [Mus musculus]
mmu-let-7f-2-3p	NM_001170868	-25.3	0.813093	NA	NA	GO:0012510,GO:0046872,GO:0015631,GO:0006810,GO:0007267,	K13764|1|4e-53|211|cfa:489500|recoverin	gi|27806017|ref|NP_776823.1|	5.49E-107	neurocalcin-delta [Bos taurus]
mmu-let-7f-2-3p	NM_174997	-20.9	1	NA	NA	-	-	gi|60360118|dbj|BAD90278.1|	9.89E-100	mKIAA4042 protein [Mus musculus]

mmu-let-7f-2-3p	NM_010604	-21.6	0.999917	NA	NA	GO:0005887,GO:0005488,GO:0005249,GO:0034220,GO:0007267,GO:0030001,	K05009|1|0.0|827|mmu:16517|potassium inwardly-rectifying channel subfamily J member	gi|130505929|ref|NP_034734.3|	0	inward rectifier potassium channel 16 [Mus musculus]
mmu-let-7f-2-3p	NM_177388	-24.1	0.974194	NA	NA	GO:0031224,GO:0046873,GO:0070838,	K15122|1|0.0|1059|mmu:338365|solute carrier family 41	gi|28893565|ref|NP_796362.1|	0	solute carrier family 41 member 2 [Mus musculus]
mmu-let-7f-2-3p	NM_029974	-22.9	0.966926	NA	NA	GO:0016020,GO:0046872,	-	gi|116812883|ref|NP_084250.1|	0	DC-STAMP domain-containing protein 1 [Mus musculus]
mmu-let-7f-2-3p	NM_053090	-20.1	1	NA	NA	GO:0044424,GO:0060089,GO:0023052,	-	gi|148671210|gb|EDL03157.1|	0	down-regulated by Ctnnb1, a, isoform CRA_a [Mus musculus]
mmu-let-7f-2-3p	NM_016868	-23.9	0.997553	NA	NA	GO:0043231,GO:0003676,GO:0060089,GO:0001071,GO:0006357,GO:0006950,	K09096|1|0.0|1192|mmu:53417|hypoxia-inducible factor 3 alpha	gi|170295859|ref|NP_058564.2|	0	hypoxia-inducible factor 3-alpha isoform 2 [Mus musculus]

mmu-let-7f-2-3p	NM_009885	-21.2	0.998825	NA	NA	GO:0032991,GO:0044425,GO:0005783,GO:0030141,GO:0044421,GO:0005515,GO:0005539,GO:0046625,GO:0004091,GO:0006672,GO:0007589,GO:0006639,GO:0008203,GO:0044240,GO:0006631,GO:0030301,GO:0006464,GO:0051969,	K12298|1|0.0|1134|mmu:12613|bile salt-stimulated lipase [EC:3.1.1.3 3.1.1.13]	gi|6753406|ref|NP_034015.1|	0	bile salt-activated lipase precursor [Mus musculus]
mmu-let-7f-2-3p	NM_028779	-20	1	NA	NA	GO:0044444,GO:0043169,GO:0016814,GO:0009112,GO:0009127,	K01490|1|0.0|1593|mmu:109674|AMP deaminase [EC:3.5.4.6]	gi|21311925|ref|NP_083055.1|	0	AMP deaminase 2 [Mus musculus]

mmu-let-7f-2-3p	NM_021421	-20.7	0.999997	NA	NA	-	K12603|1|1e-24|116|cqu:CpipJ_CPIJ005352|CCR4-NOT transcription complex subunit 6	gi|70794813|ref|NP_067396.3|	0	protein angel homolog 2 isoform 1 [Mus musculus]
mmu-let-7f-2-3p	NM_028545	-20.1	0.999985	NA	NA	GO:0016620,GO:0044238,GO:0044237,GO:0044281,	K00129|1|0.0|841|mmu:621603|aldehyde dehydrogenase (NAD(P)+) [EC:1.2.1.5	gi|343098457|ref|NP_082821.2|	0	aldehyde dehydrogenase 3 family, member B2-like [Mus musculus]
mmu-let-7f-2-3p	NM_144811	-22.2	0.997113	NA	NA	GO:0000785,GO:0043231,GO:0042393,GO:0003723,GO:0006325,GO:0010467,	K11454|1|3e-70|267|mmu:52609|chromobox protein 7	gi|21450179|ref|NP_659060.1|	2.22E-69	chromobox protein homolog 7 [Mus musculus]
mmu-let-7f-2-3p	NM_025329	-21	0.897924	NA	NA	GO:0005488,	K10420|1|2e-08|59.3|xtr:496911|dynein light chain Tctex-	gi|21426853|ref|NP_079605.1|	6.94E-79	tctex1 domain-containing protein 2 [Mus musculus]

mmu-let-7f-2-3p	NM_019417	-20.2	0.998477	NA	NA	GO:0008092,GO:0046914,	K08202|1|3e-16|86.7|oaa:100079308|MFS transporter, OCT family, solute carrier family 22 (organic cation transporter), member 4/5!K05743|2|1e-06|54.7|xtr:780362|LIM domain kinase 1 [EC:2.7.11.1]!K05760|3|3e-06|53.5|cbr:CBG09041|paxillin	gi|251823786|ref|NP_062290.2|	0	PDZ and LIM domain protein 4 [Mus musculus]

mmu-let-7f-2-3p	NM_172838	-22.1	0.999647	NA	NA	GO:0031224,GO:0015291,GO:0006810,	K11810|1|0.0|984|mmu:240638|MFS transporter, MCP family, solute carrier family 16 (monocarboxylic acid transporters), member 12	gi|27370260|ref|NP_766426.1|	0	monocarboxylate transporter 12 [Mus musculus]
mmu-let-7f-2-3p	NM_028004	-22.2	1	NA	NA	-	K12567|1|0.0|4.870e+04|hsa:7273|titin [EC:2.7.11.1]	gi|77812699|ref|NP_082280.2|	0	titin isoform N2-B [Mus musculus]
mmu-let-7f-2-3p	NM_023431	-20.5	1	NA	NA	GO:0043231,GO:0003682,GO:0006259,GO:0051276,	-	gi|50054188|ref|NP_075920.4|	0	PWWP domain-containing protein MUM1 [Mus musculus]
mmu-let-7f-2-3p	NM_029335	-20.2	0.999205	NA	NA	-	-	gi|268370161|ref|NP_083611.2|	2.12E-174	uncharacterized protein C15orf26 homolog [Mus musculus]

mmu-let-7f-2-3p	NM_010299	-21	0.999999	NA	NA	GO:0044448,GO:0000323,GO:0031981,GO:0005887,GO:0004563,GO:0005488,GO:0022892,GO:0060229,GO:0050905,GO:0016044,GO:0001573,GO:0010876,GO:0007610,GO:0009311,GO:0043085,	K12383|1|3e-94|348|mmu:14667|ganglioside GM2 activator	gi|6806917|ref|NP_034429.1|	1.97E-93	ganglioside GM2 activator precursor [Mus musculus]
mmu-let-7f-2-3p	NM_023627	-20.8	0.999403	NA	NA	GO:0044424,GO:0016872,GO:0006644,GO:0006020,	K01858|1|0.0|1097|mmu:71780|myo-inositol-1-phosphate synthase [EC:5.5.1.4]	gi|12963757|ref|NP_076116.1|	0	inositol-3-phosphate synthase 1 [Mus musculus]

mmu-let-7f-2-3p	NM_013886	-26.7	0.788364	NA	NA	GO:0043231,GO:0005488,	K00020|1|3e-08|63.2|aag:AaeL_AAEL006684|3-hydroxyisobutyrate dehydrogenase [EC:1.1.1.31]	gi|26337455|dbj|BAC32413.1|	2.02E-83	unnamed protein product [Mus musculus]
mmu-let-7f-2-3p	NM_172524	-21.3	0.99995	NA	NA	GO:0031224,GO:0004871,GO:0023052,GO:0070838,	-	gi|27369726|ref|NP_766112.1|	0	magnesium transporter NIPA4 [Mus musculus]
mmu-let-7f-2-3p	NM_001146122	-21.6	0.999212	NA	NA	GO:0005764,GO:0044421,GO:0006643,GO:0040007,GO:0010627,GO:0003006,	K12382|1|0.0|1059|mmu:19156|saposin	gi|225735653|ref|NP_001139594.1|	0	sulfated glycoprotein 1 isoform D preproprotein [Mus musculus]
mmu-let-7f-2-3p	NM_001146060	-20.9	1	NA	NA	GO:0031410,GO:0008047,GO:0005088,GO:0005515,GO:0006996,GO:0043087,GO:0033036,GO:0007266,	K04575|1|3e-139|498|mdo:100019560|amyotrophic lateral sclerosis 2 (juvenile)	gi|225690620|ref|NP_666340.3|	0	ALS2 C-terminal-like protein [Mus musculus]

mmu-let-7f-2-3p	NM_024181	-20.7	1	NA	NA	GO:0044432,GO:0043234,GO:0015036,GO:0008047,GO:0019899,GO:0043462,GO:0043161,GO:0018904,GO:0001932,GO:0019725,GO:0006915,GO:0033554,	K09530|1|0.0|1623|mmu:66861|DnaJ homolog subfamily C member 10	gi|119508443|ref|NP_077143.2|	0	dnaJ homolog subfamily C member 10 precursor [Mus musculus]
mmu-let-7f-2-3p	NM_021381	-20.5	1	NA	NA	GO:0031224,GO:0008188,GO:0007267,GO:0007186,GO:0048511,GO:0043066,	K08379|1|0.0|718|mmu:58182|prokineticin receptor 1	gi|40254343|ref|NP_067356.2|	0	prokineticin receptor 1 [Mus musculus]
mmu-let-7f-2-3p	NM_026319	-21.7	0.996027	NA	NA	GO:0031410,GO:0030990,	K12478|1|9e-14|79.7|mcc:710381|early endosome antigen 1!K11498|2|1e-13|79.3|ecb:100068227|centromeric protein E	gi|151101194|ref|NP_080595.2|	0	intraflagellar transport protein 74 homolog [Mus musculus]

mmu-let-7f-2-3p	NM_021450	-21.7	1	NA	NA	GO:0031224,GO:0031252,GO:0008092,GO:0043169,GO:0004672,GO:0004871,GO:0005261,GO:0032559,GO:0006468,GO:0007160,GO:0023052,GO:0030036,GO:00068	K04982|1|0.0|3664|mmu:58800|transient receptor potential cation channel subfamily M member 7 [EC:2.7.11.1]	gi|256773285|ref|NP_067425.2|	0	transient receptor potential cation channel subfamily M member 7 isoform 1 [Mus musculus]
mmu-let-7f-2-3p	NM_145840	-20.8	1	NA	NA	GO:0031224,GO:0050953,GO:0009584,GO:0007165,	-	gi|109735053|gb|AAI18007.1|	5.53E-90	Regulator of G-protein signalling 9 binding protein [Mus musculus]
mmu-let-7f-2-3p	NM_027860	-21.5	0.999983	NA	NA	-	-	gi|37360572|dbj|BAC98264.1|	0	mKIAA1841 protein [Mus musculus]
mmu-let-7f-2-3p	NM_011667	-21.6	0.999962	NA	NA	GO:0003824,GO:0032559,GO:0043412,GO:0009987,	K03178|1|0.0|2066|mmu:22202|ubiquitin-activating enzyme E1 [EC:6.3.2.19]	gi|6755923|ref|NP_035797.1|	0	ubiquitin-like modifier-activating enzyme 1 Y [Mus musculus]

mmu-let-7f-2-3p	NM_001195096	-20.3	1	NA	NA	-	K14798|1|6e-27|125|ssc:100627735|protein LTV1	gi|304361740|ref|NP_001182025.1|	0	phosphatase and actin regulator 2 isoform C [Mus musculus]
mmu-let-7f-2-3p	NM_130860	-23.7	0.967514	NA	NA	GO:0008023,GO:0003723,GO:0004674,GO:0043565,GO:0032559,GO:0006796,GO:0006352,GO:0045935,GO:0006354,GO:00064	K02211|1|0.0|755|rno:362110|cyclin-dependent kinase 9 [EC:2.7.11.22 2.7.11.23]	gi|18699998|ref|NP_570930.1|	0	cyclin-dependent kinase 9 [Mus musculus]
mmu-let-7f-2-3p	NR_030763	-21.9	0.995744	NA	NA	GO:0004672,GO:0032559,GO:0006464,	K08798|1|2e-61|238|ame:410717|MAP/microtubule affinity-regulating kinase [EC:2.7.11.1]	gi|149268414|ref|XP_001475924.1|	0	PREDICTED: sperm motility kinase 4A [Mus musculus]

mmu-let-7f-2-3p	NM_053138	-20.4	1	NA	NA	GO:0016021,GO:0046872,GO:0016337,GO:0007399,GO:0007267,	K04601|1|3e-59|231|cin:100185193|cadherin EGF LAG seven-pass G-type receptor 2	gi|18087789|ref|NP_444368.1|	0	protocadherin beta 13 precursor [Mus musculus]
mmu-let-7f-2-3p	NM_009806	-21	1	NA	NA	GO:0043231,GO:0005856,GO:0044459,GO:0044444,GO:0004672,GO:0016776,GO:0005515,GO:0032559,GO:0006357,GO:00064	K06103|1|0.0|1794|mmu:12361|calcium/calmodulin-dependent serine protein kinase [EC:2.7.11.1]	gi|133504495|ref|NP_033936.2|	0	peripheral plasma membrane protein CASK [Mus musculus]

mmu-let-7f-2-3p	NM_024433	-22.4	0.991345	NA	NA	GO:0043231,GO:0016763,GO:0016758,GO:0016705,GO:0005515,GO:0046495,GO:0048545,GO:0030097,GO:0001890,GO:0019725,GO:0002237,GO:0006950,	K00772|1|2e-163|577|mmu:66902|5'-methylthioadenosine phosphorylase [EC:2.4.2.28]	gi|148699009|gb|EDL30956.1|	9.27E-173	methylthioadenosine phosphorylase, isoform CRA_b [Mus musculus]
mmu-let-7f-2-3p	NR_002873	-20.2	0.999986	NA	NA	-	-	gi|148666681|gb|EDK99097.1|	2.01E-61	mCG133599, isoform CRA_b [Mus musculus]
mmu-let-7f-2-3p	NM_028687	-23.7	0.930859	NA	NA	-	K11498|1|2e-16|88.6|mcc:711186|centromeric protein E!K03257|4|2e-14|82.4|tca:659090|translation initiation factor 4A!K11499|5|2e-14|82.4|spu:592172|centromere protein	gi|256542283|ref|NP_082963.1|	0	uncharacterized protein LOC73936 [Mus musculus]

mmu-let-7f-2-3p	NM_001163852	-20.3	1	NA	NA	-	K02155|1|0.0|838|gga:416753|V-type H+-transporting ATPase 16kDa proteolipid subunit [EC:3.6.3.14]!K12826|2|3e-09|66.6|mdo:100014243|splicing factor 3A subunit 2	gi|37360272|dbj|BAC98114.1|	0	mKIAA1171 protein [Mus musculus]
mmu-let-7f-2-3p	NM_201389	-23.2	1	NA	NA	-	K10388|1|0.0|3881|mmu:18810|plectin	gi|256418964|ref|NP_958788.2|	0	plectin isoform 1e [Mus musculus]
mmu-let-7f-2-3p	NM_001081225	-23.1	0.999771	NA	NA	-	K06521|1|4e-12|75.9|acs:100564148|semaphorin 4	gi|148709994|gb|EDL41940.1|	0	mCG127475, isoform CRA_b [Mus musculus]

mmu-let-7f-2-3p	NM_181730	-23.4	0.61716	NA	NA	GO:0044452,GO:0034062,GO:0003676,GO:0005515,GO:0006352,GO:0006354,GO:0006353,	K03020|1|8e-28|124|hsa:51082|DNA-directed RNA polymerases I and III subunit RPAC2	gi|19354425|gb|AAH24394.1|	7.01E-53	Polymerase (RNA) I polypeptide D [Mus musculus]
mmu-let-7f-2-3p	NM_010020	-21.5	0.99992	NA	NA	GO:0043005,GO:0044297,GO:0016021,GO:0044424,GO:0005515,GO:0043176,GO:0005328,GO:0008504,GO:0035725,GO:0051937,GO:0007589,GO:0014073,GO:0021983,GO:0042069,GO:0042417,GO:0007610,GO:0032102,GO:0042133,GO:0010038,GO:0035264,GO	K05036|1|0.0|1228|mmu:13162|solute carrier family 6 (neurotransmitter transporter, dopamine) member 3	gi|6753606|ref|NP_034150.1|	0	sodium-dependent dopamine transporter [Mus musculus]

mmu-let-7f-2-3p	NM_001006664	-23.5	0.998481	NA	NA	GO:0043232,GO:0044444,GO:0044425,GO:0008092,GO:0007267,GO:0030036,	K06107|1|0.0|1555|mmu:13821|erythrocyte membrane protein band 4.1	gi|7305029|ref|NP_038538.1|	0	band 4.1-like protein 1 isoform a [Mus musculus]
mmu-let-7f-2-3p	NM_008088	-21.6	1	NA	NA	GO:0031252,GO:0015629,GO:0001071,GO:0003779,GO:0065008,GO:0048731,GO:0048858,GO:0022402,GO:0008154,GO:0030154,GO:0010467,	K12804|1|6e-07|58.9|bfo:BRAFLDRAFT_226416|proline-serine-threonine phosphatase interacting protein 1	gi|157817137|ref|NP_032114.2|	0	growth arrest-specific protein 7 isoform a [Mus musculus]
mmu-let-7f-2-3p	NM_001081664	-22.2	0.968458	NA	NA	GO:0043231,GO:0031224,GO:0005506,GO:0016705,GO:0051234,GO:0006631,GO:0006091,	K10226|1|3e-164|578|acs:100551818|fatty acid desaturase 2 (delta-6 desaturase) [EC:1.14.19.-]	gi|126157490|ref|NP_001075133.1|	0	fatty acid desaturase 2-like protein FADS2P1 [Mus musculus]

mmu-let-7f-2-3p	NM_001161797	-21.5	0.999993	NA	NA	-	K14798|1|9e-14|80.9|ssc:100627735|protein LTV1	gi|60360150|dbj|BAD90294.1|	0	mKIAA4120 protein [Mus musculus]
mmu-let-7f-2-3p	NM_026381	-20.5	0.999287	NA	NA	GO:0044464,GO:0009987,	K10135|1|4e-13|77.0|mmu:66940|scotin	gi|194385126|dbj|BAG60969.1|	2.51E-31	unnamed protein product [Homo sapiens]
mmu-let-7f-2-3p	NM_001243167	-22.9	0.984711	NA	NA	GO:0044421,GO:0005126,GO:0030098,GO:0007166,GO:0007049,GO:0051707,GO:00125	K05414|1|3e-16|88.6|ecb:100629951|interferon alpha	gi|149588682|ref|NP_001092311.1|	9.68E-102	interferon zeta-like precursor [Mus musculus]
mmu-let-7f-5p	NM_001007589	-26.1	0.462819	-26.63	159	GO:0043231,GO:0043234,GO:0006357,GO:0001819,GO:0002237,GO:0007275,GO:00082	-	gi|86604728|ref|NP_001007590.2|	2.67E-69	akirin-2 [Mus musculus]
mmu-let-7f-5p	NM_001037842	-28.1	0.228925	-26.02	168	GO:0019866,GO:0016740,	-	gi|83745106|ref|NP_001032931.1|	2.86E-85	probable N-acetyltransferase CML3 isoform 1 [Mus musculus]

mmu-let-7f-5p	NM_001105245	-25.7	0.987613	-25.05	168	GO:0031224,GO:0046872,GO:0016337,	K04601|1|2e-64|250|bfo:BRAFLDRAFT_130073|cadherin EGF LAG seven-pass G-type receptor 2 (flamingo)	gi|157426849|ref|NP_001098715.1|	0	protocadherin-19 isoform a precursor [Mus musculus]
mmu-let-7f-5p	NM_001105246	-25.7	0.986952	-25.05	168	GO:0031224,GO:0046872,GO:0016337,	K04601|1|2e-64|250|bfo:BRAFLDRAFT_130073|cadherin EGF LAG seven-pass G-type receptor 2 (flamingo)	gi|157426851|ref|NP_001098716.1|	0	protocadherin-19 isoform b precursor [Mus musculus]
mmu-let-7f-5p	NM_001160012	-31	0.066235	-28.71	176	GO:0005921,GO:0031224,GO:0005488,GO:0022829,GO:0043009,GO:0008544,GO:0050954,GO:0009987,	K07622|1|4e-136|485|mmu:14620|gap junction protein, beta 3	gi|6680013|ref|NP_032152.1|	3.12E-135	gap junction beta-3 protein [Mus musculus]

mmu-let-7f-5p	NM_001161432	-29.5	0.346724	-26.48	170	GO:0016021,GO:0005035,GO:0005488,GO:0043410,GO:0048869,GO:0009888,GO:0051091,GO:0008219,GO:0019221,GO:00072	K05163|1|1e-164|582|mmu:245527|tumor necrosis factor receptor superfamily member 27	gi|148682260|gb|EDL14207.1|	4.43E-166	ectodysplasin A2 isoform receptor, isoform CRA_b [Mus musculus]
mmu-let-7f-5p	NM_001161433	-29.5	0.152732	-26.48	170	GO:0016021,GO:0005035,GO:0005488,GO:0043410,GO:0048869,GO:0009888,GO:0051091,GO:0008219,GO:0019221,GO:00072	K05163|1|5e-165|582|mmu:245527|tumor necrosis factor receptor superfamily member 27	gi|148682260|gb|EDL14207.1|	1.81E-166	ectodysplasin A2 isoform receptor, isoform CRA_b [Mus musculus]

mmu-let-7f-5p	NM_001177505	-29.7	0.145093	-25.33	164	GO:0005622,GO:0046872,GO:0003676,GO:0006351,	K09230|1|0.0|1506|mmu:382118|SCAN domain-containing zinc finger protein!K09229|3|0.0|1057|ptr:470805|KRAB and SCAN domains-containing zinc finger protein	gi|294610662|ref|NP_001170976.1|	0	zinc finger protein 167 [Mus musculus]
mmu-let-7f-5p	NM_007719	-28.4	0.218698	-26.96	163	GO:0009986,GO:0016021,GO:0004950,GO:0006952,GO:0051480,GO:0002376,GO:0007166,GO:0042221,	K04182|1|0.0|716|mmu:12775|C-C chemokine receptor type 7	gi|116268121|ref|NP_031745.2|	0	C-C chemokine receptor type 7 precursor [Mus musculus]

mmu-let-7f-5p	NM_008126	-31	0.053082	-28.71	176	GO:0005921,GO:0031224,GO:0005488,GO:0022829,GO:0043009,GO:0008544,GO:0050954,GO:0009987,	K07622|1|4e-136|485|mmu:14620|gap junction protein, beta 3	gi|6680013|ref|NP_032152.1|	2.54E-135	gap junction beta-3 protein [Mus musculus]
mmu-let-7f-5p	NM_008355	-28.7	0.107045	-25.63	170	GO:0044424,GO:0044421,GO:0005126,GO:0015992,GO:0010524,GO:0009612,GO:0001818,GO:0030888,GO:0023052,GO:0002377,GO:0007154,GO:0008284,GO:0002696,GO:0014070,GO:0002237,GO:00069	K05435|1|3e-56|219|mmu:16163|interleukin 13	gi|6680403|ref|NP_032381.1|	1.79E-55	interleukin-13 precursor [Mus musculus]

mmu-let-7f-5p	NM_008824	-30.5	0.073692	-27.91	176	GO:0043234,GO:0044444,GO:0070061,GO:0008443,GO:0019899,GO:0019203,GO:0032559,GO:0006007,GO:0035556,GO:0033131,GO:0009887,GO:0043434,GO:0031960,GO:0015980,GO:0006000,GO:00069	K01103|1|0.0|910|mmu:18639|6-phosphofructo-2-kinase / fructose-2,6-bisphosphatase [EC:2.7.1.105 3.1.3.46]	gi|7434494|pir||S77704	0	6-phosphofructo-2-kinase (EC 2.7.1.105) / fructose-2, 6-bisphosphate 2-phosphatase (EC 3.1.3.46) clone 5c, skeletal muscle - rat
mmu-let-7f-5p	NM_010473	-28.6	0.253173	-25.41	169	GO:0005789,GO:0044057,	-	gi|133778931|ref|NP_034603.2|	0	histidine rich calcium binding protein precursor [Mus musculus]

mmu-let-7f-5p	NM_010484	-28.6	0.285221	-26.52	172	GO:0043231,GO:0016021,GO:0031090,GO:0044444,GO:0008144,GO:0005083,GO:0003779,GO:0005328,GO:0042802,GO:0008504,GO:0019899,GO:0019905,GO:0015844,GO:0021536,GO:0048511,GO:0045596,GO:0007611,GO:0010467,GO:0035725,GO:0032526,GO:0007320,GO:00019	K05037|1|0.0|1244|mmu:15567|solute carrier family 6 (neurotransmitter transporter, serotonin) member 4	gi|195972807|ref|NP_034614.2|	0	sodium-dependent serotonin transporter [Mus musculus]

mmu-let-7f-5p	NM_010595	-25.7	0.979727	-25.05	171	GO:0033267,GO:0005887,GO:0005488,GO:0005249,GO:0046873,GO:0007267,GO:0034220,GO:0030001,	K04874|1|0.0|847|mmu:16485|potassium voltage-gated channel Shaker-related subfamily A member	gi|119395752|ref|NP_034725.3|	0	potassium voltage-gated channel subfamily A member 1 [Mus musculus]
mmu-let-7f-5p	NM_011060	-28.3	0.354234	-26.22	169	GO:0044424,GO:0046872,GO:0016813,GO:0006527,GO:0018195,	K01481|1|0.0|1349|mmu:18601|protein-arginine deiminase [EC:3.5.3.15]	gi|156938255|ref|NP_035190.3|	0	protein-arginine deiminase type-3 [Mus musculus]
mmu-let-7f-5p	NM_011116	-26.9	0.439562	-25.38	162	GO:0031090,GO:0043231,GO:0031224,GO:0005488,GO:0004620,GO:0006629,	-	gi|7242181|ref|NP_035246.1|	0	phospholipase D3 [Mus musculus]
mmu-let-7f-5p	NM_015764	-29.3	0.56006	-25.83	170	GO:0031224,	K09390|1|6e-141|504|ssc:100511192|E2F transcription factor 6	gi|154090987|ref|NP_056579.2|	0	protein GREB1 isoform 1 [Mus musculus]

mmu-let-7f-5p	NM_019674	-29.5	0.083153	-25.2	158	GO:0005815,GO:0043231,GO:0008287,GO:0043169,GO:0004721,GO:0004702,GO:0048646,GO:0006796,GO:0007010,GO:00064	K15423|1|0.0|636|mdo:100012739|serine/threonine-protein phosphatase 4 catalytic subunit [EC:3.1.3.16]	gi|4506027|ref|NP_002711.1|	0	serine/threonine-protein phosphatase 4 catalytic subunit [Homo sapiens]
mmu-let-7f-5p	NM_025304	-32.7	0.018909	-29.5	172	GO:0051998,GO:0005488,GO:0006479,	K15451|1|3e-161|568|ecb:100061739|tRNA wybutosine-synthesizing protein 4 [EC:2.1.1.-	gi|42794773|ref|NP_079580.2|	0	leucine carboxyl methyltransferase 1 [Mus musculus]
mmu-let-7f-5p	NM_026298	-29.9	0.337327	-48.11	319	GO:0005815,GO:0043231,GO:0005515,GO:0003002,GO:0006357,GO:0010927,GO:0001838,GO:0051604,GO:0009855,GO:00071	-	gi|148705414|gb|EDL37361.1|	0	intraflagellar transport 172 homolog (Chlamydomonas), isoform CRA_a [Mus musculus]

mmu-let-7f-5p	NM_028544	-29.6	0.223718	-26.89	167	GO:0005794,	K05702|1|6e-07|57.8|nve:NEMVE_v1g174672|afadin	gi|61676215|ref|NP_082820.1|	0	ras-interacting protein 1 [Mus musculus]
mmu-let-7f-5p	NM_029965	-28.4	0.43484	-25.23	168	GO:0031224,GO:0046914,	K15707|1|1e-137|491|mmu:77733|RING finger protein 170	gi|148692281|gb|EDL24228.1|	1.05E-136	mCG1050561 [Mus musculus]
mmu-let-7f-5p	NM_145505	-27.7	0.664532	-25.72	165	-	-	gi|148669833|gb|EDL01780.1|	0	expressed sequence AI450540, isoform CRA_a [Mus musculus]
mmu-let-7f-5p	NM_148948	-26.5	0.952648	-47.91	341	GO:0031332,GO:0044444,GO:0016891,GO:0032559,GO:0043169,GO:0003723,GO:0042623,GO:0016246,GO:0006357,GO:0031641,GO:0022011,GO:0010624,GO:0048858,GO:00	K11592|1|0.0|3661|mmu:192119|endoribonuclease Dicer [EC:3.1.26.-]	gi|148686840|gb|EDL18787.1|	0	Dicer1, Dcr-1 homolog (Drosophila), isoform CRA_a [Mus musculus]

mmu-let-7f-5p	NM_175540	-29.5	0.343838	-26.48	170	GO:0016021,GO:0005035,GO:0005488,GO:0043410,GO:0048869,GO:0009888,GO:0051091,GO:0008219,GO:0019221,GO:00072	K05163|1|1e-164|582|mmu:245527|tumor necrosis factor receptor superfamily member 27	gi|148682260|gb|EDL14207.1|	4.38E-166	ectodysplasin A2 isoform receptor, isoform CRA_b [Mus musculus]
mmu-let-7f-5p	NM_178923	-29.4	0.317962	-26.69	172	GO:0031981,GO:0005488,	K13167|1|2e-145|518|mmu:224432|splicing factor, arginine/serine-rich 15	gi|148665971|gb|EDK98387.1|	1.51E-146	mCG129573, isoform CRA_b [Mus musculus]
mmu-let-7f-5p	NM_183106	-24.7	0.962019	-42.81	328	-	-	gi|124430551|ref|NP_898929.2|	0	tetratricopeptide repeat domain 17 [Mus musculus]
mmu-let-7f-5p	NM_172416	-22.8	0.993927	-40.85	312	GO:0016020,GO:0044444,GO:0005488,GO:0002573,	-	gi|27369545|ref|NP_766004.1|	9.49E-140	osteopetrosis-associated transmembrane protein 1 precursor [Mus musculus]

mmu-let-7f-5p	NM_201395	-24.1	0.969827	-41.31	311	GO:0000785,GO:0043234,GO:0046914,GO:0003676,GO:0014020,GO:0006357,GO:0035108,GO:0001832,GO:0003231,	K09228|1|2e-13|79.3|bta:507236|KRAB domain-containing zinc finger protein	gi|117553623|ref|NP_958797.2|	0	sal-like protein 4 isoform b [Mus musculus]
mmu-let-7f-5p	NM_008679	-23.4	0.999842	-41.55	304	GO:0031981,GO:0004871,GO:0004468,GO:0003676,GO:0003713,GO:0035258,GO:0008134,GO:0006357,GO:0040007,GO:0016570,GO:0048608,GO:0030520,GO:0006629,GO:00	K11256|1|0.0|2313|mmu:17979|nuclear receptor coactivator 3 [EC:2.3.1.48]	gi|118026946|ref|NP_032705.2|	0	nuclear receptor coactivator 3 [Mus musculus]
mmu-let-7f-5p	NM_028721	-21.3	1	-42.17	303	GO:0042995,GO:0005488,GO:0045596,GO:0044238,GO:0060071,GO:0030178,GO:00439	K10407|1|2e-38|163|bfo:BRAFLDRAFT_77030|kinesin light chain	gi|159131889|ref|NP_082997.3|	0	nephrocystin-3 isoform a [Mus musculus]

mmu-let-7f-5p	NM_173753	-21.2	1	-25.02	180	GO:0044424,GO:0005488,GO:0006468,	K08020|1|5e-34|148|ptr:743392|Rap guanine nucleotide exchange factor (GEF) 6	gi|55925634|ref|NP_776114.2|	0	folliculin-interacting protein 1 [Mus musculus]
mmu-let-7f-5p	NM_178687	-25.6	0.692984	-21.81	178	GO:0016021,GO:0004871,GO:0005102,GO:0019900,GO:0002420,GO:0023052,GO:0002696,GO:0001912,GO:0002714,GO:00325	K06567|1|3e-159|563|rno:307199|CD226 antigen	gi|85540456|ref|NP_848802.2|	3.75E-179	CD226 antigen isoform a precursor [Mus musculus]
mmu-let-7f-5p	NM_177639	-26.7	0.84607	-22.77	176	GO:0044459,GO:0044430,GO:0005515,GO:0007267,	K15008|1|0.0|1680|rno:65040|discs, large-associated protein 1	gi|51339023|ref|NP_808307.2|	0	disks large-associated protein 1 isoform 1 [Mus musculus]

mmu-let-7f-5p	NM_172926	-26	0.713406	-21.62	175	GO:0031224,GO:0005543,GO:0060089,GO:0023052,GO:0009987,GO:0006810,	K13304|1|3e-07|58.5|mgp:100542838|serum/glucocorticoid-regulated kinase 3 [EC:2.7.11.1]	gi|238814328|ref|NP_766514.2|	0	sorting nexin-14 [Mus musculus]
mmu-let-7f-5p	NM_201368	-22.6	0.999488	-24.68	174	GO:0016020,	-	gi|41235770|ref|NP_958756.1|	0	XK-related protein 8 [Mus musculus]
mmu-let-7f-5p	NR_040398	-27.6	0.457438	-24.79	172	-	-	gi|149026310|gb|EDL82553.1|	5.64E-10	rCG63030 [Rattus norvegicus]
mmu-let-7f-5p	NM_198703	-27.6	0.874893	-23.46	171	GO:0044424,GO:0004860,GO:0004672,GO:0019900,GO:0046872,GO:0008200,GO:0032559,GO:0006810,GO:0035556,GO:0006469,GO:0048468,GO:0003073,	K08867|1|0.0|2065|ptr:451739|WNK lysine deficient protein kinase [EC:2.7.11.1]	gi|148667213|gb|EDK99629.1|	0	WNK lysine deficient protein kinase 1, isoform CRA_b [Mus musculus]

mmu-let-7f-5p	NM_178379	-21.6	0.999805	-23.06	170	GO:0031224,GO:0005740,GO:0015002,GO:0004311,GO:0006783,GO:0018342,GO:0043623,GO:0022904,GO:0007005,	K02257|1|0.0|843|mmu:70383|protoheme IX farnesyltransferase [EC:2.5.1.-]	gi|30410008|ref|NP_848466.1|	0	protoheme IX farnesyltransferase, mitochondrial [Mus musculus]
mmu-let-7f-5p	NM_172471	-22.1	1	-20.98	169	GO:0004866,GO:0010466,	K05316|1|2e-09|67.0|cqu:CpipJ_CPIJ005979|voltage-dependent calcium channel alpha-2/delta, invertebrate	gi|27369644|ref|NP_766059.1|	0	inter-alpha-trypsin inhibitor heavy chain H5 precursor [Mus musculus]
mmu-let-7f-5p	NR_033388	-24.5	0.982542	-20.49	168	-	K04332|1|7e-07|58.2|mmu:19214|prostaglandin D2 receptor	gi|309270125|ref|XP_003085044.1|	1.96E-94	PREDICTED: uncharacterized protein LOC100169868 isoform 1 [Mus musculus]

mmu-let-7f-5p	NM_182785	-22.3	0.903242	-20.81	167	GO:0031224,	K06552|1|1e-23|111|mmu:68891|polycythemia rubra vera protein 1	gi|33354251|ref|NP_877586.1|	7.43E-135	ly6/PLAUR domain-containing protein 4 precursor [Mus musculus]
mmu-let-7f-5p	NR_045156	-22.6	0.998352	-20.87	166	-	-	gi|74196599|dbj|BAE34410.1|	3.04E-20	unnamed protein product [Mus musculus]
mmu-let-7f-5p	NR_046069	-28.7	0.05273	-24.22	165	-	-	gi|148709166|gb|EDL41112.1|	2.52E-54	mCG1051124 [Mus musculus]
mmu-let-7f-5p	NM_199469	-21.9	0.999983	-22.96	164	GO:0043231,GO:0044444,GO:0016020,GO:0046914,GO:0005515,GO:0016044,GO:0043161,GO:0006996,	K14015|1|0.0|1139|mmu:217365|nuclear protein localization protein 4 homolog	gi|148702796|gb|EDL34743.1|	0	nuclear protein localization 4 homolog (S. cerevisiae), isoform CRA_a [Mus musculus]
mmu-let-7f-5p	NR_033299	-22.1	1	-21.03	163	-	-	gi|26338105|dbj|BAC32738.1|	1.85E-55	unnamed protein product [Mus musculus]
mmu-let-7f-5p	NR_046162	-25.2	0.752482	-22.09	162	-	-	gi|148665186|gb|EDK97602.1|	7.08E-114	mCG144829 [Mus musculus]
mmu-let-7f-5p	NR_040627	-22.7	0.955221	-20.22	161	-	-	-	-	-

mmu-let-7f-5p	NM_201396	-24.1	0.922515	-21.03	160	GO:0000785,GO:0043234,GO:0046914,GO:0003676,GO:0014020,GO:0006357,GO:0035108,GO:0001832,GO:0003231,	K09228|1|1e-13|79.3|bta:507236|KRAB domain-containing zinc finger protein	gi|117553633|ref|NP_958798.2|	1.64E-135	sal-like protein 4 isoform c [Mus musculus]
mmu-let-7f-5p	NR_028521	-25.5	0.00324	-22.25	159	-	-	-	-	-
mmu-let-7f-5p	NR_040309	-23.9	0.555697	-22.54	158	GO:0031224,	-	gi|148676787|gb|EDL08734.1|	2.01E-25	mCG146092 [Mus musculus]
mmu-let-7f-5p	NM_175429	-24.3	0.982684	-20.06	157	GO:0005887,GO:0005267,GO:0030001,	K15074|1|1e-09|67.4|tad:TRIADDRAFT_4579|BTB/POZ domain-containing adapter for CUL3-mediated RhoA degradation protein	gi|30425146|ref|NP_780638.1|	3.89E-170	potassium channel tetramerisation domain containing 12b [Mus musculus]
mmu-let-7f-5p	NR_046475	-24.5	0.790297	-20.17	156	-	-	-	-	-
mmu-let-7f-5p	NR_030734	-28.5	0.097686	-24.02	155	-	-	gi|148704546|gb|EDL36493.1|	4.82E-91	mCG146312 [Mus musculus]
mmu-let-7f-5p	NR_045491	-22.9	0.92005	-21.22	154	-	-	-	-	-

mmu-let-7f-5p	NM_181751	-20.8	0.999949	-21.86	153	GO:0031224,GO:0005488,GO:0045125,GO:0030072,GO:0007166,	K08424|1|2e-175|617|mmu:236781|G protein-coupled receptor 119	gi|32306530|ref|NP_861416.1|	1.12E-174	glucose-dependent insulinotropic receptor [Mus musculus]
mmu-let-7f-5p	NM_178782	-22.9	0.999888	-20.26	152	GO:0031981,GO:0006325,GO:0010467,	K10683|1|6e-21|105|ecb:100053962|BRCA1-associated RING domain protein 1 [EC:6.3.2.19]	gi|125347764|ref|NP_848897.3|	0	BCL-6 corepressor-like protein 1 [Mus musculus]
mmu-let-7f-5p	NM_194345	-24.8	0.937553	-22.48	151	-	-	gi|257196165|ref|NP_919326.1|	0	protein FAM160B2 [Mus musculus]
mmu-let-7f-5p	NR_028280	-20.4	1	-20.62	150	-	-	gi|255308901|ref|NP_848889.3|	3.00E-129	proline-rich protein 18 [Mus musculus]

mmu-let-7f-5p	NM_011372	-20.2	1	-20.52	149	GO:0031228,GO:0016757,GO:0006464,	K03373|1|0.0|649|mmu:20447|N-acetylgalactosaminide alpha-2,6-sialyltransferase (sialyltransferase 7C) [EC:2.4.99.	gi|10304989|ref|NP_035502.1|	0	alpha-N-acetylgalactosaminide alpha-2,6-sialyltransferase 3 [Mus musculus]
mmu-let-7f-5p	NR_045544	-24.3	0.798929	-21.17	148	GO:0016020,	K15280|1|4e-147|522|mmu:228875|solute carrier family 35, member C2	gi|21450265|ref|NP_659142.1|	3.01E-146	solute carrier family 35 member C2 isoform 1 [Mus musculus]

mmu-let-7f-5p	NM_153551	-23.5	0.957478	-20.76	147	GO:0031982,GO:0044444,GO:0030695,	K01289|1|6e-54|213|dme:Dmel_CG18659|carboxypeptidase [EC:3.4.16.-]!K12861|2|3e-23|111|gga:419884|pre-mRNA-splicing factor SPF27	gi|24025656|ref|NP_705779.1|	0	DENN domain-containing protein 1C [Mus musculus]
mmu-let-7f-5p	NM_134086	-21.1	1	-20.36	146	GO:0031224,GO:0043005,GO:0015175,GO:0005343,GO:0015804,GO:0001505,GO:0006807,GO:0030001,	K14990|1|0.0|841|mmu:105727|solute carrier family 38 (sodium-coupled neutral amino acid transporter), member 1	gi|148672291|gb|EDL04238.1|	0	solute carrier family 38, member 1, isoform CRA_a [Mus musculus]
mmu-let-7f-5p	NM_021564	-21.9	0.985318	-20.91	145	GO:0044421,GO:0004866,GO:0010466,	K03898|1|1e-13|78.6|gga:424957|kininog	gi|37719755|gb|AAN34519.1|	0	GUGU alpha [Mus musculus]

mmu-let-7f-5p	NM_025902	-21.3	0.999937	-20.62	144	GO:0031224,GO:0032991,GO:0031966,GO:0042802,GO:0043169,GO:0051536,GO:0007275,GO:0006914,	-	gi|21728370|ref|NP_080178.1|	2.71E-59	CDGSH iron-sulfur domain-containing protein 2 [Mus musculus]

mmu-let-7f-5p	NM_177656	-22.3	0.974464	-20.2	143	-	K07847|1|1e-23|112|mgp:100542192|Rad and Gem related GTP binding protein 1!K04514|2|3e-06|54.3|tca:663533|Rho-associated, coiled-coil containing protein kinase [EC:2.7.11.1]!K11547|3|6e-06|53.1|tgu:100226934|kinetochore protein	gi|29244072|ref|NP_808324.1|	0	uncharacterized protein LOC228778 [Mus musculus]
mmu-let-7f-5p	NM_177184	-22.8	1	-20.06	142	GO:0033036,	-	gi|122114537|ref|NP_796158.2|	0	vacuolar protein sorting-associated protein 13C [Mus musculus]

mmu-let-7f-5p	NM_001008427	NA	NA	-20.26	143	-	K09228|1|0.0|1156|mmu:434179|KRAB domain-containing zinc finger protein	gi|148680477|gb|EDL12424.1|	0	mCG140430, isoform CRA_b [Mus musculus]
mmu-let-7f-5p	NM_025298	-21.1	1	NA	NA	GO:0031981,GO:0034062,GO:0006952,GO:0006354,GO:0051707,GO:0006353,	K14721|1|0.0|1358|rno:361640|DNA-directed RNA polymerase III subunit RPC5	gi|31981264|ref|NP_079574.2|	0	DNA-directed RNA polymerase III subunit RPC5 isoform 1 [Mus musculus]
mmu-let-7f-5p	NM_178695	-21.7	0.999657	NA	NA	GO:0031224,GO:0043231,GO:0046872,	K01320|1|5e-10|67.8|hsa:2155|coagulation factor VII [EC:3.4.21.21]	gi|224994242|ref|NP_848810.3|	7.87E-120	transmembrane gamma-carboxyglutamic acid protein 4 precursor [Mus musculus]
mmu-let-7f-5p	NM_007625	-20.8	1	NA	NA	GO:0031981,GO:0043232,GO:0003723,GO:0003712,GO:0042393,GO:0006357,GO:0006325,GO:0043066,	K11452|1|0.0|900|mmu:12418|chromobox protein 4	gi|161353508|ref|NP_031651.2|	0	E3 SUMO-protein ligase CBX4 [Mus musculus]

mmu-let-7f-5p	NM_144887	-20.1	1	NA	NA	-	K05766|1|1e-58|230|spu:575378|slingshot	gi|50511087|dbj|BAD32529.1|	0	mKIAA1748 protein [Mus musculus]
mmu-let-7f-5p	NM_013557	-20.5	0.999999	NA	NA	GO:0044424,GO:0004674,GO:0042802,GO:0008135,GO:0005506,GO:0032559,GO:0050896,GO:0001932,GO:0006447,GO:0046984,GO:0006725,GO:0008283,	K08860|1|0.0|1087|mmu:15467|eukaryotic translation initiation factor 2-alpha kinase [EC:2.7.11.1]	gi|50510905|dbj|BAD32438.1|	0	mKIAA1369 protein [Mus musculus]
mmu-let-7f-5p	NM_001143790	-23.9	0.985558	NA	NA	GO:0000783,GO:0043566,GO:0006259,	-	gi|219689066|ref|NP_001137262.1|	0	CST complex subunit CTC1 isoform c [Mus musculus]
mmu-let-7f-5p	NM_201369	-20.7	1	NA	NA	GO:0043231,GO:0032559,GO:0001701,	K15720|1|2e-41|173|xtr:100493484|NEDD4-binding protein 2 [EC:3.-.-.-]	gi|158186629|ref|NP_958757.2|	0	NEDD4-binding protein 2-like 2 [Mus musculus]

mmu-let-7f-5p	NM_001142731	-22.7	0.997658	NA	NA	-	K15074|1|8e-10|67.4|cbr:CBG18386|BTB/POZ domain-containing adapter for CUL3-mediated RhoA degradation protein	gi|218505696|ref|NP_001136203.1|	0	BTB/POZ domain-containing protein KCTD1 isoform 1 [Mus musculus]
mmu-let-7f-5p	NM_019948	-20.5	0.999996	NA	NA	GO:0031224,GO:0030246,	K10059|1|4e-120|433|mmu:56619|C-type lectin domain family 4, member E	gi|9910162|ref|NP_064332.1|	2.60E-119	C-type lectin domain family 4 member E [Mus musculus]
mmu-let-7f-5p	NM_001033236	-21.3	0.972252	NA	NA	-	-	gi|98986329|ref|NP_001028408.2|	1.73E-122	methyltransferase-like protein 21D [Mus musculus]

mmu-let-7f-5p	NM_001113392	-22.7	0.992162	NA	NA	GO:0031224,GO:0042101,GO:0044424,GO:0004872,GO:0042802,GO:0050851,GO:0050688,GO:0031294,	K06453|1|2e-77|291|mmu:12503|CD3Z antigen, zeta polypeptide	gi|164664502|ref|NP_001106863.1|	8.85E-97	CD247 antigen isoform iota precursor [Mus musculus]
mmu-let-7f-5p	NM_001037136	-21	1	NA	NA	GO:0044424,GO:0005083,GO:0008047,GO:0046914,GO:0032561,GO:0006810,GO:0043087,GO:0032012,	K12491|1|0.0|1498|mmu:347722|Arf-GAP, GTPase, ANK repeat and PH domain-containing protein 1/2/3	gi|80978937|ref|NP_001032213.1|	0	arf-GAP with GTPase, ANK repeat and PH domain-containing protein 1 isoform 2 [Mus musculus]
mmu-let-7f-5p	NM_019552	-22.2	0.999897	NA	NA	GO:0043231,GO:0019866,GO:0031301,GO:0042802,GO:0015440,GO:0032559,GO:0006810,GO:0009207,	K05657|1|0.0|1286|mmu:56199|ATP-binding cassette, subfamily B (MDR/TAP), member 10	gi|9506367|ref|NP_062425.1|	0	ATP-binding cassette sub-family B member 10, mitochondrial [Mus musculus]

mmu-let-7f-5p	NM_026481	-20.2	0.998138	NA	NA	GO:0015630,GO:0046872,GO:0008092,GO:0000226,	-	gi|332227513|ref|XP_003262935.1|	8.25E-96	PREDICTED: tubulin polymerization-promoting protein family member 3-like isoform 2 [Nomascus leucogenys]
mmu-let-7f-5p	NM_001159638	-20	0.999952	NA	NA	-	-	gi|21311853|ref|NP_083023.1|	6.89E-119	uncharacterized protein LOC74088 isoform 1 [Mus musculus]
mmu-let-7f-5p	NM_146311	-23	0.751871	NA	NA	GO:0016020,GO:0004888,GO:0007166,	K04257|1|1e-148|526|mmu:258308|olfactory	gi|22129665|ref|NP_666423.1|	8.02E-148	olfactory receptor 510 [Mus musculus]
mmu-let-7f-5p	NM_009353	-20.5	0.999998	NA	NA	GO:0031981,GO:0000782,GO:0043234,GO:0043073,GO:0042802,GO:0043565,GO:0003690,GO:0001304,GO:0034502,GO:0043009,GO:0016233,GO:0006278,GO:00322	K11111|1|0.0|796|rno:361403|telomeric repeat-binding factor 2	gi|133904142|ref|NP_033379.2|	0	telomeric repeat-binding factor 2 isoform 1 [Mus musculus]

mmu-let-7f-5p	NM_028385	-21.5	1	NA	NA	-	K09189|1|9e-95|350|aml:100468393|histone-lysine N-methyltransferase MLL5 [EC:2.1.1.4	gi|28972834|dbj|BAC65833.1|	0	mKIAA1757 protein [Mus musculus]
mmu-let-7f-5p	NM_178646	-23.3	0.998133	NA	NA	GO:0043231,GO:0044427,GO:0003676,	K11496|1|3e-26|122|mdo:100031122|centromere protein B	gi|111185889|ref|NP_848761.2|	0	tigger transposable element derived 5 [Mus musculus]
mmu-let-7f-5p	NM_172589	-26	0.827456	NA	NA	GO:0031224,	-	gi|27369844|ref|NP_766177.1|	2.29E-113	lipoma HMGIC fusion partner-like 2 protein [Mus musculus]
mmu-let-7f-5p	NM_019696	-23.3	0.963856	NA	NA	GO:0044421,GO:0046914,GO:0008235,GO:0019538,GO:0009987,	K08638|1|0.0|1428|mmu:56264|carboxypeptidase X1 [EC:3.4.17.-]	gi|228008326|ref|NP_062670.2|	0	probable carboxypeptidase X1 precursor [Mus musculus]
mmu-let-7f-5p	NM_173750	-21.4	0.999966	NA	NA	-	-	gi|148695824|gb|EDL27771.1|	4.37E-146	RIKEN cDNA 2700007P21, isoform CRA_b [Mus musculus]

mmu-let-7f-5p	NM_001242381	-20.2	1	NA	NA	GO:0044459,GO:0031224,GO:0015301,GO:0006464,GO:0055067,GO:0030001,	K13861|1|0.0|2024|ptr:459688|solute carrier family 4 (sodium bicarbonate transporter), member 10	gi|148695037|gb|EDL26984.1|	0	solute carrier family 4, sodium bicarbonate cotransporter-like, member 10, isoform CRA_b [Mus musculus]
mmu-let-7f-5p	NM_001270667	-21.8	0.994752	NA	NA	-	-	-	-	-
mmu-let-7f-5p	NM_023662	-20.2	1	NA	NA	GO:0005813,GO:0072372,GO:0005635,GO:0005488,GO:0021846,GO:0031023,GO:0010927,GO:0033365,GO:0010721,	K07847|1|1e-99|367|ssc:100517200|Rad and Gem related GTP binding protein 1	gi|170763496|ref|NP_076151.2|	0	pericentriolar material 1 protein [Mus musculus]

mmu-let-7f-5p	NM_029771	-21.7	0.999225	NA	NA	GO:0043231,GO:0016021,GO:0031090,GO:0012505,GO:0008289,GO:0004879,GO:0004888,GO:0043410,GO:0030522,GO:0009755,GO:0007166,	K04246|1|0.0|726|mmu:76854|G protein-coupled receptor 30	gi|254588034|ref|NP_084047.2|	0	G-protein coupled estrogen receptor 1 [Mus musculus]
mmu-let-7f-5p	NM_001126182	-21	1	NA	NA	-	K12807|1|0.0|2870|mmu:17948|baculoviral IAP repeat-containing protein 1	gi|187133241|ref|NP_035002.2|	0	baculoviral IAP repeat-containing protein 1b [Mus musculus]
mmu-let-7f-5p	NM_001252374	-20.9	0.99853	NA	NA	GO:0043231,GO:0044444,GO:0008252,GO:0046872,GO:0016740,GO:0006213,GO:0006753,GO:0009112,GO:0046128,GO:00067	K01081|1|8e-168|590|rno:312373|5'-nucleotidase [EC:3.1.3.5]	gi|356991161|ref|NP_001239303.1|	2.14E-168	cytosolic 5'-nucleotidase 3 isoform 2 [Mus musculus]

mmu-let-7f-5p	NM_001252472	-24	0.968411	NA	NA	GO:0031224,GO:0004871,GO:0023052,	K06511|1|7e-62|240|mmu:12523|CD84 antigen	gi|357197147|ref|NP_001239401.1|	2.33E-68	SLAM family member 5 isoform 2 precursor [Mus musculus]
mmu-let-7f-5p	NM_001024952	-24.7	0.999041	NA	NA	GO:0044424,GO:0003676,GO:0046914,GO:0002698,	K15690|1|0.0|2014|mmu:381305|RING finger and CCCH-type zinc finger domain-containin	gi|50511255|dbj|BAD32613.1|	0	mKIAA2025 protein [Mus musculus]
mmu-let-7f-5p	NM_001103171	-20.2	1	NA	NA	GO:0005902,GO:0015629,GO:0008092,GO:0017111,GO:0032559,GO:0042471,GO:0050954,GO:0007610,	K10361|1|0.0|6171|mmu:17910|myosin XV	gi|157041248|ref|NP_001096641.1|	0	unconventional myosin-XV isoform 3 [Mus musculus]
mmu-let-7g-3p	NM_001001152	-28.1	0.628372	-26.02	159	-	K09228|1|0.0|1632|mmu:238690|KRAB domain-containing zinc finger protein	gi|47564121|ref|NP_001001152.1|	0	zinc finger protein 43 [Mus musculus]

mmu-let-7g-3p	NM_001001186	-27.9	0.716008	-25.15	163	GO:0046872,GO:0003006,GO:0006355,	K09228|1|0.0|997|mmu:408065|KRAB domain-containing zinc finger protein	gi|123702020|ref|NP_001001186.3|	0	zinc finger protein 456 [Mus musculus]
mmu-let-7g-3p	NM_001001297	-28.7	0.391603	-25.21	158	GO:0044424,GO:0046872,GO:0016838,GO:0016301,GO:0048037,GO:0032559,GO:0006566,GO:0008652,GO:0006796,	K06037|1|5e-10|67.4|dre:641568|[EC:4.2.3.-]	gi|211826759|gb|AAH27743.2|	1.25E-120	Thnsl1 protein [Mus musculus]
mmu-let-7g-3p	NM_001002238	-27.1	0.875125	-45.6	306	GO:0043186,GO:0030529,GO:0046914,GO:0048869,GO:0016458,GO:0022403,GO:0048232,GO:0034660,GO:0003006,GO:00063	K15979|1|5e-12|75.1|nve:NEMVE_v1g183195|staphylococcal nuclease domain-containing protein 1	gi|50355696|ref|NP_001002238.1|	0	tudor domain-containing protein 1 [Mus musculus]

mmu-let-7g-3p	NM_001002240	-27.1	0.873782	-45.6	306	GO:0043186,GO:0030529,GO:0046914,GO:0048869,GO:0016458,GO:0022403,GO:0048232,GO:0034660,GO:0003006,GO:00063	K15979|1|5e-12|75.1|nve:NEMVE_v1g183195|staphylococcal nuclease domain-containing protein 1	gi|50355696|ref|NP_001002238.1|	0	tudor domain-containing protein 1 [Mus musculus]
mmu-let-7g-3p	NM_001002241	-27.1	0.887844	-45.6	306	GO:0043186,GO:0030529,GO:0046914,GO:0048869,GO:0016458,GO:0022403,GO:0048232,GO:0034660,GO:0003006,GO:00063	K15979|1|6e-12|75.1|nve:NEMVE_v1g183195|staphylococcal nuclease domain-containing protein 1	gi|50355696|ref|NP_001002238.1|	0	tudor domain-containing protein 1 [Mus musculus]
mmu-let-7g-3p	NM_001003666	-26.9	0.620847	-25.15	163	GO:0046872,	K09228|1|0.0|1378|mmu:431706|KRAB domain-containing zinc finger protein	gi|110665714|ref|NP_001003666.2|	0	zinc finger protein 457 [Mus musculus]

mmu-let-7g-3p	NM_001003933	-31	0.358092	-27.22	159	GO:0044421,GO:0031090,GO:0043231,GO:0031224,GO:0005488,GO:0007029,GO:0006810,GO:0012501,	K00688|1|3e-37|159|ame:409267|starch phosphorylase [EC:2.4.1.1]	gi|148701361|gb|EDL33308.1|	0	reticulon 3, isoform CRA_c [Mus musculus]
mmu-let-7g-3p	NM_001003934	-31	0.361557	-27.22	159	GO:0044421,GO:0031090,GO:0043231,GO:0031224,GO:0005488,GO:0007029,GO:0006810,GO:0012501,	K00688|1|3e-37|159|ame:409267|starch phosphorylase [EC:2.4.1.1]	gi|54607016|ref|NP_001003934.1|	0	reticulon-3 isoform 1 [Mus musculus]
mmu-let-7g-3p	NM_001005477	-30.6	0.198043	-25.44	165	GO:0043231,GO:0001071,GO:0003677,GO:0016477,GO:0006412,GO:0006355,GO:0001558,GO:0045165,GO:0050767,	K09360|1|1e-179|630|mmu:104382|BarH-like	gi|56699436|ref|NP_001005477.1|	1.01E-178	barH-like 2 homeobox protein [Mus musculus]

mmu-let-7g-3p	NM_001007575	-27.3	0.601591	-25.15	163	GO:0044464,GO:0046914,GO:0003676,GO:0006351,	K09228|1|0.0|1071|mmu:238693|KRAB domain-containing zinc finger protein	gi|56119156|ref|NP_001007576.1|	0	zinc finger protein 58 [Mus musculus]
mmu-let-7g-3p	NM_001011775	-29.8	0.104584	-26.68	166	GO:0004888,GO:0023052,	K04257|1|5e-95|348|ecb:100060799|olfactory receptor	gi|58801326|ref|NP_001011775.1|	1.16E-166	olfactory receptor 1419 [Mus musculus]
mmu-let-7g-3p	NM_001013769	-29.7	0.242813	-26.85	167	GO:0005488,GO:0003006,GO:0006355,	K09228|1|0.0|989|mmu:380855|KRAB domain-containing zinc finger protein	gi|71480078|ref|NP_001013791.1|	0	regulator of sex limited protein 1 [Mus musculus]
mmu-let-7g-3p	NM_001014900	-31.1	0.096965	-25.57	163	GO:0044464,GO:0046914,	-	gi|225543354|ref|NP_001014900.2|	0	zinc finger, MYND domain containing 12 [Mus musculus]
mmu-let-7g-3p	NM_001025163	-29.5	0.536687	-27.31	163	GO:0043231,GO:0005488,	K09228|1|0.0|1027|mmu:330463|KRAB domain-containing zinc finger protein	gi|68448539|ref|NP_001020334.1|	0	zinc finger protein 78 isoform 1 [Mus musculus]

mmu-let-7g-3p	NM_001025444	-30.1	0.508097	-249.87	1452	GO:0031981,GO:0000785,GO:0003723,GO:0046914,GO:0035312,GO:0005515,GO:0019204,GO:0043566,GO:0010608,GO:0006281,GO:0000302,GO:0006796,	K10863|1|0.0|689|mmu:66408|aprataxin [EC:3.-.-.-]	gi|48734599|gb|AAH71213.1|	0	Aptx protein, partial [Mus musculus]
mmu-let-7g-3p	NM_001033314	-26.8	0.616097	-25.79	161	-	-	gi|148691146|gb|EDL23093.1|	0	RIKEN cDNA C530028I08, isoform CRA_b [Mus musculus]
mmu-let-7g-3p	NM_001033432	-31.7	0.211572	-28.76	178	GO:0035295,	-	gi|84370019|ref|NP_001028604.1|	0	headcase protein homolog [Mus musculus]
mmu-let-7g-3p	NM_001035231	-28	0.743759	-25.15	163	-	K09228|1|0.0|1926|mmu:212276|KRAB domain-containing zinc finger protein	gi|60360632|dbj|BAD90328.1|	0	mKIAA4236 protein [Mus musculus]

mmu-let-7g-3p	NM_001038612	-27.5	0.278383	-25.1	150	GO:0044421,GO:0044432,GO:0005488,GO:0006461,GO:0048731,	-	gi|84370349|ref|NP_001033701.1|	1.30E-80	noelin isoform b precursor [Mus musculus]
mmu-let-7g-3p	NM_001038613	-27.5	0.620188	-25.1	150	GO:0044421,GO:0044432,GO:0005488,GO:0006461,GO:0048731,	K04593|1|3e-55|217|mmu:99633|latrophilin 2	gi|84370353|ref|NP_001033702.1|	0	noelin isoform c precursor [Mus musculus]
mmu-let-7g-3p	NM_001038614	-27.5	0.266663	-25.1	150	GO:0044421,GO:0044432,GO:0005488,GO:0006461,GO:0048731,	-	gi|338720148|ref|XP_003364130.1|	9.04E-60	PREDICTED: noelin isoform 3 [Equus caballus]
mmu-let-7g-3p	NM_001038695	-35.3	0.07369	-53.74	325	GO:0043231,GO:0046914,GO:0016701,GO:0035258,GO:0048869,GO:0030518,GO:0070076,GO:0048232,GO:0006351,GO:00442	K15601|1|0.0|2693|mmu:104263|lysine-specific demethylase 3 [EC:1.14.11.-]	gi|37360072|dbj|BAC98014.1|	0	mKIAA0742 protein [Mus musculus]

mmu-let-7g-3p	NM_001039653	-29.7	0.262565	-25.66	170	GO:0043231,GO:0043234,GO:0046914,GO:0001071,GO:0003677,GO:0007411,GO:0021536,GO:0006357,GO:0003002,GO:0021523,GO:0006915,	K09374|1|0.0|686|mmu:16871|LIM homeobox protein 3/4	gi|148676345|gb|EDL08292.1|	0	mCG18748, isoform CRA_b [Mus musculus]
mmu-let-7g-3p	NM_001040106	-29.1	0.959366	-26.45	164	-	K08853|1|0.0|802|mmu:269774|AP2-associated kinase [EC:2.7.11.1]	gi|40789031|dbj|BAA83000.2|	0	KIAA1048 protein [Homo sapiens]
mmu-let-7g-3p	NM_001042655	-34.7	0.043415	-30.66	174	GO:0044464,GO:0005099,GO:0032313,	K13136|1|4e-98|360|nvi:100114269|gem associated protein 8!K11452|2|2e-13|78.6|tgu:100222038|chromobox protein 4	gi|74208127|dbj|BAE29165.1|	0	unnamed protein product [Mus musculus]

mmu-let-7g-3p	NM_001076791	-26.7	0.852247	-25.24	163	GO:0043169,	K09228|1|0.0|962|mmu:408067|KRAB domain-containing zinc finger protein	gi|115647917|ref|NP_001070259.1|	0	regulator of sex-limitation candidate 16 [Mus musculus]
mmu-let-7g-3p	NM_001079883	-26.1	0.990841	-50.49	315	GO:0043231,GO:0000982,GO:0000977,GO:0046872,GO:0042127,GO:0008544,GO:0006357,GO:0021756,GO:0042476,GO:0045058,GO:0009791,GO:0048667,GO:0002681,GO:0006629,GO:0006915,GO:00020	K09228|1|1e-18|97.8|api:100159445|KRAB domain-containing zinc finger protein	gi|120587007|ref|NP_001073352.1|	0	B-cell lymphoma/leukemia 11B isoform a [Mus musculus]
mmu-let-7g-3p	NM_001080941	-27.9	0.452234	-25.15	163	GO:0005488,GO:0003006,GO:0006355,	K09228|1|0.0|958|mmu:380855|KRAB domain-containing zinc finger protein	gi|148678364|gb|EDL10311.1|	0	mCG140745, isoform CRA_a [Mus musculus]

mmu-let-7g-3p	NM_001080942	-29.9	0.038665	-26.5	171	-	K01403|1|1e-09|63.2|xtr:448591|matrix metalloproteinase-9 (gelatinase B) [EC:3.4.24.35]	gi|124301182|ref|NP_001074411.1|	6.97E-79	binder of sperm protein homolog 2 precursor [Mus musculus]
mmu-let-7g-3p	NM_001081064	-30.1	0.805284	-25.4	158	GO:0030054,GO:0043231,GO:0044430,GO:0007165,	K06095|1|5e-31|139|gga:427360|multiple PDZ domain protein	gi|124486716|ref|NP_001074533.1|	0	PDZ domain-containing protein 2 [Mus musculus]
mmu-let-7g-3p	NM_001081185	-30.4	0.644251	-29.28	160	GO:0030016,GO:0016020,GO:0008092,GO:0034330,	K04437|1|0.0|5400|mmu:68794|filamin	gi|124487139|ref|NP_001074654.1|	0	filamin-C [Mus musculus]
mmu-let-7g-3p	NM_001081217	-27.5	0.879596	-25.83	156	GO:0005856,GO:0031981,GO:0046914,GO:0042802,GO:0001071,GO:0043565,GO:00001	K09230|1|0.0|829|mmu:385674|SCAN domain-containing zinc finger protein	gi|124487047|ref|NP_001074686.1|	0	zinc finger protein 174 [Mus musculus]

mmu-let-7g-3p	NM_001081260	-28.5	0.751181	-25.22	155	GO:0044444,GO:0000784,GO:0008092,GO:0006278,GO:0000288,	-	gi|124486923|ref|NP_001074729.1|	0	182 kDa tankyrase-1-binding protein [Mus musculus]
mmu-let-7g-3p	NM_001081441	-32	0.08354	-29.38	155	-	K10260|1|3e-38|160|gga:419074|F-box and WD-40 domain protein 7	gi|124517674|ref|NP_001074910.1|	0	WD repeat domain 86 [Mus musculus]
mmu-let-7g-3p	NM_001081650	-26.9	0.843594	-27.98	165	GO:0043231,GO:0016020,GO:0008047,GO:0060089,GO:0043087,GO:0007166,	K07524|1|0.0|868|mmu:50780|regulator of G-protein signalling 3	gi|126032301|ref|NP_001075119.1|	0	regulator of G-protein signaling 3 isoform 3 [Mus musculus]
mmu-let-7g-3p	NM_001082535	-29.5	0.287756	-47.25	329	GO:0031224,GO:0004175,	K14212|1|0.0|1025|mmu:237958|signal peptide peptidase-like 2C [EC:3.4.23.-]	gi|126722669|ref|NP_001076004.1|	0	signal peptide peptidase-like 2C isoform b precursor [Mus musculus]
mmu-let-7g-3p	NM_001085535	-30.4	0.162762	-27.25	166	GO:0016020,	-	gi|147905830|ref|NP_001079004.1|	2.75E-64	transmembrane protein 235 precursor [Mus musculus]

mmu-let-7g-3p	NM_001110513	-27.8	0.573978	-26.32	165	GO:0043231,GO:0043169,GO:0003676,GO:0005515,GO:0006351,GO:0032501,	K09103|1|0.0|1055|mmu:228598|early B-cell factor	gi|160333394|ref|NP_001103983.1|	0	transcription factor COE4 [Mus musculus]
mmu-let-7g-3p	NM_001111096	-28.8	0.520256	-28.21	158	GO:0044430,GO:0005911,GO:0005743,GO:0008305,GO:0004713,GO:0046625,GO:0032559,GO:0005126,GO:0019904,GO:0015631,GO:0019899,GO:0070851,GO:0051704,GO:0008284,GO:0043434,GO:0021782,GO:0050851,GO:0002441,GO:0010522,GO:0001934,GO:0030099,GO:00140	K05854|1|0.0|1051|mmu:17096|tyrosine-protein kinase Lyn [EC:2.7.10.2]	gi|161760636|ref|NP_001104566.1|	0	tyrosine-protein kinase Lyn isoform A [Mus musculus]

mmu-let-7g-3p	NM_001113563	-32.7	0.198897	-29.56	161	GO:0031981,GO:0044444,GO:0060089,GO:0035258,GO:0046872,GO:0001071,GO:0043566,GO:0001779,GO:0042108,GO:0072350,GO:0006915,GO:0006357,GO:0010876,GO:0007346,GO:0043648,GO:0002260,GO:0045638,GO:0001912,GO:0032787,GO:0031349,GO:0006599,GO:00352	K11224|1|0.0|1558|mmu:20851|signal transducer and activator of transcription 5B	gi|165932366|ref|NP_035619.3|		0	signal transducer and activator of transcription 5B [Mus musculus]

mmu-let-7g-3p	NM_001114140	-32.7	0.29111	-28.85	167	GO:0043231,GO:0003712,GO:0046914,GO:0001071,GO:0043565,	K09188|1|5e-11|72.4|dya:Dyak_GE16999|histone-lysine N-methyltransferase MLL3 [EC:2.1.1.43]	gi|166706891|ref|NP_001107612.1|	0	transcription factor 20 isoform a [Mus musculus]
mmu-let-7g-3p	NM_001145896	-31.4	0.397867	-28.57	168	GO:0005488,	-	gi|225543323|ref|NP_941073.2|	0	genetic suppressor element 1 isoform 1 [Mus musculus]
mmu-let-7g-3p	NM_001145897	-31.4	0.39518	-28.57	168	GO:0005488,	-	gi|225543323|ref|NP_941073.2|	0	genetic suppressor element 1 isoform 1 [Mus musculus]
mmu-let-7g-3p	NM_001159572	-28.8	0.646044	-25.72	164	GO:0031224,GO:0004871,GO:0023052,	-	gi|47847392|dbj|BAD21368.1|	2.14E-168	mFLJ00041 protein [Mus musculus]
mmu-let-7g-3p	NM_001159907	-29.5	0.559727	-46.25	328	GO:0043231,GO:0005488,GO:0016740,GO:0006355,	K03239|1|1e-103|379|cin:100175767|translation initiation factor eIF-2B subunit alpha	gi|229577450|ref|NP_001153379.1|	0	TAR (HIV-1) RNA binding protein 1 [Mus musculus]

mmu-let-7g-3p	NM_001161618	-27.6	0.869343	-47.25	320	GO:0043231,GO:0031461,GO:0044444,GO:0008528,GO:0019899,GO:0005261,GO:0019787,GO:0006917,GO:0022402,GO:0051704,GO:0019941,GO:0006874,GO:0006816,GO:0008283,GO:0006950,	K10612|1|0.0|1604|mmu:75717|cullin 5	gi|239051082|ref|NP_001155090.1|	0	cullin-5 isoform 2 [Mus musculus]
mmu-let-7g-3p	NM_001161852	-29.4	0.221962	-25.37	157	GO:0031461,GO:0005488,GO:0009057,	K10103|1|2e-103|377|ptr:457952|F-box protein	gi|240255559|ref|NP_001155324.1|	1.89E-120	F-box only protein 44 isoform 2 [Mus musculus]
mmu-let-7g-3p	NM_001162414	-28.7	0.601906	-26.08	159	GO:0043231,GO:0005515,GO:0032559,GO:0006915,GO:0006952,GO:0012502,	K12798|1|0.0|1933|mmu:637515|NACHT, LRR and PYD domains-containing protein 1	gi|241666419|ref|NP_001155886.1|	0	NLR family, pyrin domain containing 1B isoform 3 [Mus musculus]

mmu-let-7g-3p	NM_001162425	-30.8	0.104769	-58.69	329	GO:0016021,GO:0031225,GO:0005102,GO:0010594,GO:0000122,GO:0003176,GO:0001837,GO:0003188,GO:0010769,GO:0007154,GO:0007169,GO:0007399,GO:0003174,GO:00	K05462|1|1e-84|314|rno:94268|ephrin-A	gi|247269690|ref|NP_034237.3|	7.03E-89	ephrin-A1 isoform 1 precursor [Mus musculus]
mmu-let-7g-3p	NM_001163246	-28.1	0.74655	-26.02	159	GO:0005622,GO:0046872,GO:0006351,	K09228|1|0.0|1641|mmu:212281|KRAB domain-containing zinc finger protein	gi|253735886|ref|NP_001156718.1|	0	zinc finger protein [Mus musculus]
mmu-let-7g-3p	NM_001163703	-31.5	0.338395	-29.21	170	GO:0044444,GO:0007090,GO:0006915,GO:0016049,GO:0010212,GO:0009411,	K10624|1|7e-58|227|ame:724670|E3 ubiquitin-protein ligase RBBP6 [EC:6.3.2.19]	gi|27734156|ref|NP_775584.1|	7.76E-178	DCN1-like protein 3 [Mus musculus]

mmu-let-7g-3p	NM_001163749	-27.6	0.776786	-25.01	171	GO:0015630,GO:0005913,GO:0008017,GO:0016337,GO:0045217,GO:0000226,GO:00328	-	gi|148689975|gb|EDL21922.1|	0	RIKEN cDNA 2310057J16, isoform CRA_b [Mus musculus]
mmu-let-7g-3p	NM_001163847	-25.2	0.998498	-42.84	307	-	K02155|1|0.0|832|gga:416753|V-type H+-transporting ATPase 16kDa proteolipid subunit [EC:3.6.3.14]!K12826|2|3e-09|66.6|mdo:100014243|splicing factor 3A subunit 2	gi|148690351|gb|EDL22298.1|	0	TBC1 domain family, member 24, isoform CRA_c [Mus musculus]

mmu-let-7g-3p	NM_001163848	-25.2	0.997938	-42.84	307	-	K02155|1|0.0|832|gga:416753|V-type H+-transporting ATPase 16kDa proteolipid subunit [EC:3.6.3.14]!K12826|2|3e-09|66.6|mdo:100014243|splicing factor 3A subunit 2	gi|37360272|dbj|BAC98114.1|	0	mKIAA1171 protein [Mus musculus]

mmu-let-7g-3p	NM_001163849	-25.2	0.997811	-42.84	307	-	K02155|1|0.0|832|gga:416753|V-type H+-transporting ATPase 16kDa proteolipid subunit [EC:3.6.3.14]!K12826|2|3e-09|66.6|mdo:100014243|splicing factor 3A subunit 2	gi|148690351|gb|EDL22298.1|	0	TBC1 domain family, member 24, isoform CRA_c [Mus musculus]

mmu-let-7g-3p	NM_001163850	-25.2	0.998479	-42.84	307	-	K02155|1|0.0|838|gga:416753|V-type H+-transporting ATPase 16kDa proteolipid subunit [EC:3.6.3.14]!K12826|2|3e-09|66.6|mdo:100014243|splicing factor 3A subunit 2	gi|148690351|gb|EDL22298.1|	0	TBC1 domain family, member 24, isoform CRA_c [Mus musculus]

mmu-let-7g-3p	NM_001163851	-25.2	0.997912	-42.84	307	-	K02155|1|0.0|838|gga:416753|V-type H+-transporting ATPase 16kDa proteolipid subunit [EC:3.6.3.14]!K12826|2|3e-09|66.6|mdo:100014243|splicing factor 3A subunit 2	gi|37360272|dbj|BAC98114.1|	0	mKIAA1171 protein [Mus musculus]

mmu-let-7g-3p	NM_001163852	-25.2	0.998703	-42.84	307	-	K02155|1|0.0|838|gga:416753|V-type H+-transporting ATPase 16kDa proteolipid subunit [EC:3.6.3.14]!K12826|2|3e-09|66.6|mdo:100014243|splicing factor 3A subunit 2	gi|37360272|dbj|BAC98114.1|	0	mKIAA1171 protein [Mus musculus]

mmu-let-7g-3p	NM_001163853	-25.2	0.998544	-42.84	307	-	K02155|1|0.0|838|gga:416753|V-type H+-transporting ATPase 16kDa proteolipid subunit [EC:3.6.3.14]!K12826|2|3e-09|66.6|mdo:100014243|splicing factor 3A subunit 2	gi|37360272|dbj|BAC98114.1|	0	mKIAA1171 protein [Mus musculus]
mmu-let-7g-3p	NM_001164497	-31.9	0.248879	-27.56	170	GO:0043231,GO:0003676,GO:0034061,GO:0006259,GO:0000956,	K03514|1|0.0|659|xtr:100491336|DNA polymerase sigma subunit [EC:2.7.7.7]	gi|256818784|ref|NP_001157969.1|	0	PAP-associated domain-containing protein 5 isoform a [Mus musculus]
mmu-let-7g-3p	NM_001164498	-31.9	0.247539	-27.56	170	GO:0043231,GO:0003676,GO:0034061,GO:0006259,GO:0000956,	K03514|1|0.0|640|xtr:100491336|DNA polymerase sigma subunit [EC:2.7.7.7]	gi|256818784|ref|NP_001157969.1|	0	PAP-associated domain-containing protein 5 isoform a [Mus musculus]

mmu-let-7g-3p	NM_001164499	-31.9	0.2422	-27.56	170	GO:0043231,GO:0003676,GO:0034061,GO:0006259,GO:0000956,	K03514|1|1e-175|618|xtr:100491336|DNA polymerase sigma subunit [EC:2.7.7.7]	gi|256818788|ref|NP_001157971.1|	0	PAP-associated domain-containing protein 5 isoform b [Mus musculus]
mmu-let-7g-3p	NM_001164671	-29.3	0.627932	-28.76	158	GO:0044424,GO:0043169,GO:0070325,GO:0032559,GO:0044267,GO:0048870,GO:0030518,GO:0048232,GO:0006974,	K09502|1|0.0|724|rno:65028|DnaJ homolog subfamily A member 1	gi|6680297|ref|NP_032324.1|	0	dnaJ homolog subfamily A member 1 [Mus musculus]
mmu-let-7g-3p	NM_001164768	-28.6	0.477017	-27.31	162	GO:0043231,GO:0005488,GO:0019787,GO:0032446,	K10261|1|0.0|907|ptr:464698|F-box and WD-40 domain	gi|397526456|ref|XP_003833140.1|	0	PREDICTED: F-box/WD repeat-containing protein 2 isoform 3 [Pan paniscus]
mmu-let-7g-3p	NM_001164769	-28.6	0.39971	-27.31	162	GO:0043231,GO:0005488,GO:0019787,GO:0032446,	K10261|1|0.0|713|ptr:464698|F-box and WD-40 domain	gi|148676676|gb|EDL08623.1|	0	F-box and WD-40 domain protein 2, isoform CRA_d [Mus musculus]

mmu-let-7g-3p	NM_001164770	-28.6	0.472723	-27.31	162	GO:0043231,GO:0005488,GO:0019787,GO:0032446,	K10261|1|0.0|855|mmu:30050|F-box and WD-40 domain protein 2	gi|148676677|gb|EDL08624.1|	0	F-box and WD-40 domain protein 2, isoform CRA_e [Mus musculus]
mmu-let-7g-3p	NM_001164772	-28.6	0.434504	-27.31	162	GO:0043231,	K10261|1|2e-133|477|mcc:700958|F-box and WD-40 domain protein 2	gi|148676676|gb|EDL08623.1|	3.63E-137	F-box and WD-40 domain protein 2, isoform CRA_d [Mus musculus]
mmu-let-7g-3p	NM_001165954	-25.8	0.998963	-48.12	322	GO:0046872,	K11458|1|0.0|1244|mmu:241915|polyhomeotic-like protein 3	gi|260099711|ref|NP_001159426.1|	0	polyhomeotic-like protein 3 isoform 1 [Mus musculus]
mmu-let-7g-3p	NM_001165956	-30.2	0.730667	-48.12	322	GO:0005488,	K11458|1|0.0|1142|mmu:241915|polyhomeotic-like protein 3	gi|260099715|ref|NP_001159428.1|	0	polyhomeotic-like protein 3 isoform 4 [Mus musculus]

mmu-let-7g-3p	NM_001166218	-27.9	0.706319	-25.15	163	-	K09228|1|0.0|1542|mmu:78251|KRAB domain-containing zinc finger protein	gi|148678380|gb|EDL10327.1|	0	mCG67989 [Mus musculus]
mmu-let-7g-3p	NM_001177732	-32.1	0.307909	-27.68	154	GO:0044444,GO:0060089,GO:0046872,GO:0004629,GO:0006629,GO:0048017,	K05861|1|2e-97|359|mdo:100012056|phosphatidylinositol phospholipase C, zeta [EC:3.1.4.11]!K15370|2|5e-90|334|nve:NEMVE_v1g89019|inactive phospholipase C-like protein 2!K05857|4|1e-85|320|xla:735225|phosphatidylinositol phospholipase C,	gi|295148200|ref|NP_899014.2|	0	1-phosphatidylinositol 4,5-bisphosphate phosphodiesterase eta-1 isoform 1 [Mus musculus]

mmu-let-7g-3p	NM_001177733	-32.1	0.307694	-27.68	154	GO:0044444,GO:0060089,GO:0046872,GO:0004629,GO:0006629,GO:0048017,	K05861|1|2e-97|359|mdo:100012056|phosphatidylinositol phospholipase C, zeta [EC:3.1.4.11]!K15370|2|5e-90|334|nve:NEMVE_v1g89019|inactive phospholipase C-like protein 2!K05857|4|1e-85|320|xla:735225|phosphatidylinositol phospholipase C,	gi|295148204|ref|NP_001171204.1|	0	1-phosphatidylinositol 4,5-bisphosphate phosphodiesterase eta-1 isoform 2 [Mus musculus]

mmu-let-7g-3p	NM_001198933	-30.6	0.285162	-27.86	156	GO:0043231,GO:0044444,GO:0046914,GO:0004470,GO:0032559,GO:0006629,GO:0043648,GO:0051259,GO:0006739,GO:0009719,GO:0010033,	K00029|1|0.0|1048|mmu:17436|malate dehydrogenase (oxaloacetate-decarboxylating)(NADP+) [EC:1.1.1.40]	gi|312147392|ref|NP_001185862.1|	0	NADP-dependent malic enzyme isoform 2 [Mus musculus]
mmu-let-7g-3p	NM_001205353	-26.1	0.936352	-27.1	149	GO:0005740,GO:0031224,GO:0012501,	-	gi|329663552|ref|NP_001192282.1|	0	GRAM domain-containing protein 4 isoform 2 [Mus musculus]
mmu-let-7g-3p	NM_001252437	-28.8	0.502304	-25.09	161	GO:0005768,GO:0016020,GO:0046914,GO:0019787,GO:0007041,GO:0009968,GO:0007186,GO:0016567,	K10604|1|0.0|961|mmu:17237|E3 ubiquitin-protein ligase MGRN1 [EC:6.3.2.19]	gi|356995926|ref|NP_001239366.1|	0	E3 ubiquitin-protein ligase MGRN1 isoform 1 [Mus musculus]
mmu-let-7g-3p	NM_001252489	-30.8	0.225497	-29.67	154	GO:0044464,GO:0008289,GO:0016125,GO:0006810,GO:0010876,	-	gi|52345396|ref|NP_997413.2|	0	oxysterol-binding protein-related protein 1 isoform 1 [Mus musculus]

mmu-let-7g-3p	NM_001252490	-30.8	0.215093	-29.67	154	GO:0044464,GO:0008289,GO:0016125,GO:0006810,GO:0010876,	-	gi|357394760|ref|NP_001239418.1|	0	oxysterol-binding protein-related protein 1 isoform 2 [Mus musculus]
mmu-let-7g-3p	NM_001252491	-30.8	0.232029	-29.67	154	GO:0044464,GO:0008289,GO:0016125,GO:0006810,GO:0010876,	-	gi|148669624|gb|EDL01571.1|	0	oxysterol binding protein-like 1A, isoform CRA_a [Mus musculus]
mmu-let-7g-3p	NM_001252492	-30.8	0.24516	-29.67	154	GO:0044464,GO:0008289,GO:0016125,GO:0006810,GO:0010876,	-	gi|148669624|gb|EDL01571.1|	0	oxysterol binding protein-like 1A, isoform CRA_a [Mus musculus]
mmu-let-7g-3p	NM_001252493	-30.8	0.237778	-29.67	154	GO:0044464,GO:0008289,GO:0016125,GO:0006810,GO:0010876,	-	gi|148669624|gb|EDL01571.1|	0	oxysterol binding protein-like 1A, isoform CRA_a [Mus musculus]

mmu-let-7g-3p	NM_001253808	-33.5	0.082682	-26.77	169	GO:0015630,GO:0030141,GO:0008047,GO:0043169,GO:0015631,GO:0043087,GO:0048699,GO:0007599,GO:0007017,GO:0015698,GO:0000912,GO:0009966,GO:0048232,GO:0000920,GO:0035556,	K04450|1|7e-25|117|ptr:739083|activating transcription factor 2!K10360|2|7e-22|107|ssc:100520722|myosin IX	gi|148672172|gb|EDL04119.1|	0	Rac GTPase-activating protein 1, isoform CRA_a [Mus musculus]

mmu-let-7g-3p	NM_001253809	-33.5	0.082341	-26.77	169	GO:0015630,GO:0030141,GO:0008047,GO:0043169,GO:0015631,GO:0043087,GO:0048699,GO:0007599,GO:0007017,GO:0015698,GO:0000912,GO:0009966,GO:0048232,GO:0000920,GO:0035556,	K04450|1|7e-25|117|ptr:739083|activating transcription factor 2!K10360|2|7e-22|107|ssc:100520722|myosin IX	gi|148672172|gb|EDL04119.1|	0	Rac GTPase-activating protein 1, isoform CRA_a [Mus musculus]
mmu-let-7g-3p	NM_001256052	-27.9	0.59882	-25.15	163	-	K09228|1|3e-121|423|mmu:78251|KRAB domain-containing zinc finger protein	gi|365812506|ref|NP_001242981.1|	0	regulator of sex-limitation candidate 18 [Mus musculus]

mmu-let-7g-3p	NM_007463	-25.9	0.998495	-46.6	315	GO:0043231,GO:0004672,GO:0032559,GO:0030154,GO:0006464,GO:0008283,GO:0048513,	K08809|1|0.0|5477|rno:363256|striated muscle-specific serine/threonine protein kinase [EC:2.7.11.1]	gi|148667988|gb|EDL00405.1|	0	SPEG complex locus, isoform CRA_b [Mus musculus]
mmu-let-7g-3p	NM_007769	-27.6	0.881716	-81.08	502	GO:0030666,GO:0031224,GO:0030667,GO:0031012,GO:0051635,GO:0019899,GO:0004888,GO:0006810,GO:0019731,GO:0048545,GO:0002758,GO:0001832,GO:00308	K13912|1|0.0|3868|mmu:12945|deleted in malignant brain tumors 1 protein	gi|148685745|gb|EDL17692.1|	0	deleted in malignant brain tumors 1, isoform CRA_c [Mus musculus]
mmu-let-7g-3p	NM_007897	-32.2	0.100954	-30.54	168	GO:0043231,GO:0043169,GO:0003676,GO:0001071,GO:0019904,GO:0006355,GO:00325	K09103|1|0.0|1119|mmu:13591|early B-cell factor	gi|148701892|gb|EDL33839.1|	0	early B-cell factor 1, isoform CRA_c [Mus musculus]

mmu-let-7g-3p	NM_008051	-29.7	0.305729	-26.75	166	GO:0000139,GO:0031224,GO:0031127,GO:0044238,	K00718|1|0.0|760|mmu:14343|galactoside 2-L-fucosyltransferase 1/2 [EC:2.4.1.69]	gi|49355810|ref|NP_032077.2|	0	galactoside 2-alpha-L-fucosyltransferase 1 [Mus musculus]
mmu-let-7g-3p	NM_008101	-29.5	0.246203	-27.57	165	GO:0031224,GO:0043231,GO:0042277,GO:0004930,GO:0016192,GO:0031667,GO:0007188,GO:0033762,GO:0015980,GO:00080	K04583|1|0.0|932|mmu:14527|glucagon receptor	gi|160298176|ref|NP_032127.2|	0	glucagon receptor precursor [Mus musculus]
mmu-let-7g-3p	NM_008135	-29.7	0.379452	-26.24	155	GO:0016021,GO:0005488,GO:0015175,GO:0005283,GO:0005326,GO:0015804,GO:0035725,	K05038|1|0.0|1275|mmu:14664|solute carrier family 6 (neurotransmitter transporter, amino acid) member 5/7/9/14	gi|148698593|gb|EDL30540.1|	0	solute carrier family 6 (neurotransmitter transporter, glycine), member 9, isoform CRA_a [Mus musculus]

mmu-let-7g-3p	NM_008397	-30.6	0.352078	-28.67	163	GO:0008305,GO:0009986,GO:0042995,GO:0005911,GO:0016323,GO:0005578,GO:0004871,GO:0005102,GO:0042476,GO:0016337,GO:0030035,GO:0006915,GO:0007044,GO:0002376,GO:0007599,GO:0007154,GO:0007166,GO:0045444,GO:00315	K06485|1|0.0|2134|mmu:16403|integrin alpha 6	gi|31982236|ref|NP_032423.2|	0	integrin alpha-6 precursor [Mus musculus]
mmu-let-7g-3p	NM_008482	-30.9	0.416822	-25.9	167	GO:0043256,GO:0044444,GO:0005198,GO:0016477,GO:0030030,GO:0006935,GO:0009887,GO:0008284,GO:0000904,	K05636|1|0.0|3679|mmu:16777|laminin, beta 1	gi|148704971|gb|EDL36918.1|	0	laminin B1 subunit 1 [Mus musculus]

mmu-let-7g-3p	NM_008512	-31.8	0.618788	-26.11	164	GO:0043005,GO:0030135,GO:0044297,GO:0016021,GO:0012505,GO:0004871,GO:0005102,GO:0008565,GO:0046872,GO:0019899,GO:0034185,GO:0006909,GO:0044260,GO:0016055,GO:0051050,GO:0015031,GO:0007186,GO:0010874,GO:0010517,GO:0032502,GO:0010466,GO:00325	K04550|1|0.0|9441|mmu:16971|low density lipoprotein-related protein 1 (alpha-2-macroglobulin receptor)	gi|124494256|ref|NP_032538.2|	0	prolow-density lipoprotein receptor-related protein 1 precursor [Mus musculus]

mmu-let-7g-3p	NM_008600	-30.4	0.195399	-26.32	163	GO:0005911,GO:0031224,GO:0005488,GO:0005372,GO:0005198,GO:0050953,GO:0015721,GO:0042044,GO:0043010,	K09863|1|1e-133|477|mmu:17339|aquaporin-0	gi|31543250|ref|NP_032626.2|	9.91E-133	lens fiber major intrinsic protein [Mus musculus]
mmu-let-7g-3p	NM_008615	-30.6	0.278784	-27.86	156	GO:0043231,GO:0044444,GO:0046914,GO:0004470,GO:0032559,GO:0006629,GO:0043648,GO:0051259,GO:0006739,GO:0009719,GO:0010033,	K00029|1|0.0|1064|rno:24552|malate dehydrogenase (oxaloacetate-decarboxylating)(NADP+) [EC:1.1.1.40]	gi|162139827|ref|NP_032641.2|	0	NADP-dependent malic enzyme isoform 1 [Mus musculus]

mmu-let-7g-3p	NM_008947	-25.2	0.756082	-25.53	160	GO:0031981,GO:0043234,GO:0017111,GO:0032559,GO:0043161,GO:0051320,GO:0007050,GO:0016070,GO:0009207,GO:0051439,GO:0006520,GO:0006915,GO:00303	K03062|1|0.0|783|cfa:100688636|26S proteasome regulatory subunit T2	gi|395503740|ref|XP_003756221.1|	0	PREDICTED: 26S protease regulatory subunit 4 [Sarcophilus harrisii]

mmu-let-7g-3p	NM_009212	-25.7	0.982774	-26.06	166	GO:0030427,GO:0043005,GO:0043231,GO:0044297,GO:0043234,GO:0044444,GO:0046914,GO:0005515,GO:0043021,GO:0032559,GO:0003723,GO:0004003,GO:0043566,GO:0004004,GO:0006357,GO:0006259,GO:0009207,GO:0051259,GO:00215	K14326|1|6e-34|148|dwi:Dwil_GK25498|regulator of nonsense transcripts 1 [EC:3.6.4.-]	gi|74191771|dbj|BAE32841.1|	0	unnamed protein product [Mus musculus]
mmu-let-7g-3p	NM_009557	-26.1	0.947409	-44.52	322	GO:0043231,GO:0046914,GO:0003676,GO:0006351,	K09228|1|0.0|981|mmu:22704|KRAB domain-containing zinc finger protein	gi|50511065|dbj|BAD32518.1|	0	mKIAA1710 protein [Mus musculus]

mmu-let-7g-3p	NM_009660	-26.9	0.672287	-25.39	166	GO:0031981,GO:0005886,GO:0044444,GO:0046914,GO:0008200,GO:0016702,GO:0016803,GO:0000038,GO:0006636,GO:0006884,GO:0008283,GO:0001503,GO:0006691,GO:0016049,GO:0006873,GO:0006952,GO:00	K00458|1|0.0|1296|mmu:11687|arachidonate 12-lipoxygenase [EC:1.13.11.31]!K00460|2|0.0|1232|rno:81639|arachidonate 15-lipoxygenase [EC:1.13.11.33]	gi|134948633|ref|NP_033790.3|	0	arachidonate 12-lipoxygenase, leukocyte-type [Mus musculus]
mmu-let-7g-3p	NM_009761	-29.5	0.400912	-27.03	167	GO:0031966,GO:0044425,GO:0012505,GO:0042802,GO:0046983,GO:0012502,GO:0006915,GO:0010629,GO:0007005,GO:00022	K15465|1|4e-78|294|rno:140923|BCL2/adenovirus E1B 19 kDa protein-interacting protein 3-like	gi|74151578|dbj|BAE41138.1|	2.51E-77	unnamed protein product [Mus musculus]

mmu-let-7g-3p	NM_007567	-23.3	1	-43.42	328	GO:0044459,GO:0043232,GO:0043231,GO:0044456,GO:0044463,GO:0046914,GO:0007267,GO:0006996,	K13171|1|2e-08|51.6|mgp:100546733|serine/arginine repetitive matrix protein 1!K10955|5|2e-06|58.5|hsa:4583|intestinal mucin-2	gi|148689323|gb|EDL21270.1|	0	bassoon [Mus musculus]
mmu-let-7g-5p	NM_001007589	-28	0.245176	-27.49	163	GO:0043231,GO:0043234,GO:0006357,GO:0001819,GO:0002237,GO:0007275,GO:00082	-	gi|86604728|ref|NP_001007590.2|	2.67E-69	akirin-2 [Mus musculus]
mmu-let-7g-5p	NM_001024504	-28.7	0.332381	-25.01	162	-	K10624|1|6e-40|167|ame:724670|E3 ubiquitin-protein ligase RBBP6 [EC:6.3.2.19]	gi|111162659|ref|NP_001019675.2|	1.35E-149	DCN1-like protein 2 isoform a [Mus musculus]

mmu-let-7g-5p	NM_001033759	-29.5	0.48305	-25.6	170	GO:0031224,	K09666|1|6e-09|65.5|spu:575152|beta-1,2-N-acetylglucosaminyltransferase [EC:2.4.1.-]	gi|76253922|ref|NP_001028931.1|	0	transmembrane protein 2 [Mus musculus]
mmu-let-7g-5p	NM_001042649	-28.7	0.323925	-25.01	162	-	K10624|1|1e-31|94.4|ame:724670|E3 ubiquitin-protein ligase RBBP6 [EC:6.3.2.19]	gi|148690187|gb|EDL22134.1|	2.05E-133	mCG3700, isoform CRA_e [Mus musculus]
mmu-let-7g-5p	NM_001042650	-28.7	0.313899	-25.01	162	-	K10624|1|1e-17|93.2|ame:724670|E3 ubiquitin-protein ligase RBBP6 [EC:6.3.2.19]	gi|148690187|gb|EDL22134.1|	8.48E-138	mCG3700, isoform CRA_e [Mus musculus]

mmu-let-7g-5p	NM_001042651	-28.7	0.322487	-25.01	162	-	K10624|1|1e-24|109|ame:724670|E3 ubiquitin-protein ligase RBBP6 [EC:6.3.2.19]	gi|148690187|gb|EDL22134.1|	2.91E-132	mCG3700, isoform CRA_e [Mus musculus]
mmu-let-7g-5p	NM_001042760	-32.2	0.033308	-28.6	171	GO:0016021,GO:0044459,GO:0012505,GO:0044424,GO:0015238,GO:0008509,GO:0019899,GO:0015291,GO:0015893,GO:0003008,GO:0006812,	K08214|1|2e-168|592|mmu:18400|MFS transporter, OCT family, solute carrier family 22 (organic cation transporter), member 18	gi|112363086|ref|NP_001036225.1|	1.43E-167	solute carrier family 22 member 18 [Mus musculus]

mmu-let-7g-5p	NM_001105245	-28.7	0.758291	-27.04	180	GO:0031224,GO:0046872,GO:0016337,	K04601|1|2e-64|250|bfo:BRAFLDRAFT_130073|cadherin EGF LAG seven-pass G-type receptor 2 (flamingo)	gi|157426849|ref|NP_001098715.1|	0	protocadherin-19 isoform a precursor [Mus musculus]
mmu-let-7g-5p	NM_001105246	-28.7	0.753771	-27.04	180	GO:0031224,GO:0046872,GO:0016337,	K04601|1|2e-64|250|bfo:BRAFLDRAFT_130073|cadherin EGF LAG seven-pass G-type receptor 2 (flamingo)	gi|157426851|ref|NP_001098716.1|	0	protocadherin-19 isoform b precursor [Mus musculus]

mmu-let-7g-5p	NM_001110140	-30.9	0.217245	-26.69	163	GO:0032991,GO:0016021,GO:0005789,GO:0001664,GO:0019829,GO:0046872,GO:0032559,GO:0009888,GO:0007599,GO:0008016,GO:0006816,GO:0006936,GO:0009207,GO:0007165,GO:0001775,GO:0009206,GO:0009725,GO:0006875,	K05853|1|0.0|2035|mmu:11938|Ca2+ transporting ATPase, sarcoplasmic/endoplasmic reticulum [EC:3.6.3.8]	gi|60360584|dbj|BAD90532.1|	0	mKIAA4195 protein [Mus musculus]

mmu-let-7g-5p	NM_001112697	-31.4	0.248141	-27.26	180	GO:0044430,GO:0016021,GO:0033267,GO:0044459,GO:0045202,GO:0004629,GO:0016907,GO:0048731,GO:0007200,GO:0008283,GO:0043412,GO:0007268,	K04129|1|0.0|738|mmu:12669|muscarinic acetylcholine receptor M1	gi|31542388|ref|NP_031724.2|	0	muscarinic acetylcholine receptor M1 [Mus musculus]
mmu-let-7g-5p	NM_001122989	-31.2	0.237908	-29.24	170	GO:0031224,GO:0031981,GO:0005488,GO:0004721,GO:0006470,GO:0006259,GO:0051443,GO:0000077,	K06639|1|0.0|934|mmu:218294|cell division cycle 14 [EC:3.1.3.48]	gi|172072673|ref|NP_001116461.1|	0	dual specificity protein phosphatase CDC14B isoform 2 [Mus musculus]
mmu-let-7g-5p	NM_001160012	-28.3	0.238422	-27.15	172	GO:0005921,GO:0031224,GO:0005488,GO:0022829,GO:0043009,GO:0008544,GO:0050954,GO:0009987,	K07622|1|4e-136|485|mmu:14620|gap junction protein, beta 3	gi|6680013|ref|NP_032152.1|	3.12E-135	gap junction beta-3 protein [Mus musculus]

mmu-let-7g-5p	NM_001164679	-27.5	0.578449	-25.3	169	GO:0016021,GO:0005216,	K13982|1|9e-81|303|dgr:Dgri_GH10918|probable ATP-dependent RNA helicase DDX4 [EC:3.6.4.13]!K03018|3|2e-19|99.4|smm:Smp_002890|DNA-directed RNA polymerase III subunit RPC1 [EC:2.7.7.6]	gi|50511029|dbj|BAD32500.1|	0	mKIAA1623 protein [Mus musculus]

mmu-let-7g-5p	NM_001168321	-29.7	0.430926	-26.5	166	GO:0043231,GO:0043234,GO:0003712,GO:0043565,GO:0019787,GO:0019899,GO:0000122,GO:0030279,GO:0007389,GO:0070168,GO:0009887,GO:0032446,GO:0000414,GO:00	K10683|1|3e-16|89.7|ecb:100053962|BRCA1-associated RING domain protein 1 [EC:6.3.2.19]	gi|37360460|dbj|BAC98208.1|	0	mKIAA1575 protein [Mus musculus]
mmu-let-7g-5p	NM_007698	-31.4	0.244665	-27.26	180	GO:0044430,GO:0016021,GO:0033267,GO:0044459,GO:0045202,GO:0004629,GO:0016907,GO:0048731,GO:0007200,GO:0008283,GO:0043412,GO:0007268,	K04129|1|0.0|738|mmu:12669|muscarinic acetylcholine receptor M1	gi|31542388|ref|NP_031724.2|	0	muscarinic acetylcholine receptor M1 [Mus musculus]

mmu-let-7g-5p	NM_008126	-28.3	0.198844	-27.15	172	GO:0005921,GO:0031224,GO:0005488,GO:0022829,GO:0043009,GO:0008544,GO:0050954,GO:0009987,	K07622|1|4e-136|485|mmu:14620|gap junction protein, beta 3	gi|6680013|ref|NP_032152.1|	2.54E-135	gap junction beta-3 protein [Mus musculus]
mmu-let-7g-5p	NM_008355	-30.3	0.057017	-27.15	162	GO:0044424,GO:0044421,GO:0005126,GO:0015992,GO:0010524,GO:0009612,GO:0001818,GO:0030888,GO:0023052,GO:0002377,GO:0007154,GO:0008284,GO:0002696,GO:0014070,GO:0002237,GO:00069	K05435|1|3e-56|219|mmu:16163|interleukin 13	gi|6680403|ref|NP_032381.1|	1.79E-55	interleukin-13 precursor [Mus musculus]

mmu-let-7g-5p	NM_008767	-32.2	0.032086	-28.6	171	GO:0016021,GO:0044459,GO:0012505,GO:0044424,GO:0015238,GO:0008509,GO:0019899,GO:0015291,GO:0015893,GO:0003008,GO:0006812,	K08214|1|2e-168|592|mmu:18400|MFS transporter, OCT family, solute carrier family 22 (organic cation transporter), member 18	gi|112363086|ref|NP_001036225.1|	1.38E-167	solute carrier family 22 member 18 [Mus musculus]
mmu-let-7g-5p	NM_008824	-32.1	0.04009	-29.17	180	GO:0043234,GO:0044444,GO:0070061,GO:0008443,GO:0019899,GO:0019203,GO:0032559,GO:0006007,GO:0035556,GO:0033131,GO:0009887,GO:0043434,GO:0031960,GO:0015980,GO:0006000,GO:00069	K01103|1|0.0|910|mmu:18639|6-phosphofructo-2-kinase / fructose-2,6-bisphosphatase [EC:2.7.1.105 3.1.3.46]	gi|7434494|pir||S77704	0	6-phosphofructo-2-kinase (EC 2.7.1.105) / fructose-2, 6-bisphosphate 2-phosphatase (EC 3.1.3.46) clone 5c, skeletal muscle - rat

mmu-let-7g-5p	NM_010473	-28.8	0.25674	-25.67	165	GO:0005789,GO:0044057,	-	gi|133778931|ref|NP_034603.2|	0	histidine rich calcium binding protein precursor [Mus musculus]
mmu-let-7g-5p	NM_010484	-28.7	0.300004	-26.46	168	GO:0043231,GO:0016021,GO:0031090,GO:0044444,GO:0008144,GO:0005083,GO:0003779,GO:0005328,GO:0042802,GO:0008504,GO:0019899,GO:0019905,GO:0015844,GO:0021536,GO:0048511,GO:0045596,GO:0007611,GO:0010467,GO:0035725,GO:0032526,GO:0007320,GO:00019	K05037|1|0.0|1244|mmu:15567|solute carrier family 6 (neurotransmitter transporter, serotonin) member 4	gi|195972807|ref|NP_034614.2|	0	sodium-dependent serotonin transporter [Mus musculus]

mmu-let-7g-5p	NM_019674	-27.3	0.245935	-25.33	157	GO:0005815,GO:0043231,GO:0008287,GO:0043169,GO:0004721,GO:0004702,GO:0048646,GO:0006796,GO:0007010,GO:00064	K15423|1|0.0|636|mdo:100012739|serine/threonine-protein phosphatase 4 catalytic subunit [EC:3.1.3.16]	gi|4506027|ref|NP_002711.1|	0	serine/threonine-protein phosphatase 4 catalytic subunit [Homo sapiens]
mmu-let-7g-5p	NM_020575	-29.3	0.235971	-30.37	171	GO:0003824,GO:0046914,	K10662|1|0.0|1066|mmu:57438|E3 ubiquitin-protein ligase MARCH7 [EC:6.3.2.19]	gi|10181210|ref|NP_065600.1|	0	E3 ubiquitin-protein ligase MARCH7 [Mus musculus]
mmu-let-7g-5p	NM_025304	-32.6	0.022795	-29.49	168	GO:0051998,GO:0005488,GO:0006479,	K15451|1|3e-161|568|ecb:100061739|tRNA wybutosine-synthesizing protein 4 [EC:2.1.1.-	gi|42794773|ref|NP_079580.2|	0	leucine carboxyl methyltransferase 1 [Mus musculus]
mmu-let-7g-5p	NM_025994	-30.7	0.114177	-27.25	165	GO:0016020,GO:0046872,	-	gi|31981086|ref|NP_080270.2|	2.07E-94	EF-hand domain-containing protein D2 [Mus musculus]

mmu-let-7g-5p	NM_027318	-29.3	0.072698	-26.31	164	GO:0043169,	K11663|1|7e-68|258|rno:100360077|zinc finger HIT domain-containing protein 1	gi|148687347|gb|EDL19294.1|	1.07E-95	zinc finger, HIT domain containing 1, isoform CRA_c [Mus musculus]
mmu-let-7g-5p	NM_028402	-27.7	0.492944	-25.21	164	-	K09228|1|0.0|736|mmu:78251|KRAB domain-containing zinc finger protein	gi|38049077|tpg|DAA01863.1|	0	TPA: TPA_exp: regulator of sex-limitation candidate 12 [Mus musculus]
mmu-let-7g-5p	NM_029510	-29.7	0.474214	-26.5	166	GO:0043231,GO:0043234,GO:0003712,GO:0043565,GO:0019787,GO:0019899,GO:0000122,GO:0030279,GO:0007389,GO:0070168,GO:0009887,GO:0032446,GO:0000414,GO:00	K10683|1|3e-16|89.7|ecb:100053962|BRCA1-associated RING domain protein 1 [EC:6.3.2.19]	gi|269995967|ref|NP_083786.2|	0	BCL-6 corepressor isoform a [Mus musculus]

mmu-let-7g-5p	NM_029965	-34.9	0.036905	-31.22	180	GO:0031224,GO:0046914,	K15707|1|1e-137|491|mmu:77733|RING finger protein 170	gi|148692281|gb|EDL24228.1|	1.05E-136	mCG1050561 [Mus musculus]
mmu-let-7g-5p	NM_031997	-29.5	0.487412	-25.6	170	GO:0031224,	K09666|1|6e-09|65.5|spu:575152|beta-1,2-N-acetylglucosaminyltransferase [EC:2.4.1.-]	gi|76253922|ref|NP_001028931.1|	0	transmembrane protein 2 [Mus musculus]
mmu-let-7g-5p	NM_134141	-30.3	0.257836	-27.06	167	GO:0031981,GO:0043066,GO:0048534,	-	gi|19527376|ref|NP_598902.1|	2.56E-165	anamorsin [Mus musculus]
mmu-let-7g-5p	NM_146814	-28.3	0.104309	-48.53	329	GO:0004871,GO:0023052,	K04257|1|4e-151|534|mmu:258810|olfactory	gi|22129233|ref|NP_667025.1|	2.98E-150	olfactory receptor 665 [Mus musculus]
mmu-let-7g-5p	NM_172716	-29.7	0.278615	-26.43	166	GO:0046914,GO:0010467,	K11488|1|2e-144|514|ptr:461045|polycomb group RING finger protein 3	gi|148688164|gb|EDL20111.1|	3.18E-148	polycomb group ring finger 3, isoform CRA_c [Mus musculus]

mmu-let-7g-5p	NM_175044	-29.7	0.471504	-26.5	166	GO:0043231,GO:0043234,GO:0003712,GO:0043565,GO:0019787,GO:0019899,GO:0000122,GO:0030279,GO:0007389,GO:0070168,GO:0009887,GO:0032446,GO:0000414,GO:00	K10683|1|3e-16|89.7|ecb:100053962|BRCA1-associated RING domain protein 1 [EC:6.3.2.19]	gi|269995971|ref|NP_778209.2|	0	BCL-6 corepressor isoform b [Mus musculus]
mmu-let-7g-5p	NM_175045	-29.7	0.469082	-26.5	166	GO:0043231,GO:0043234,GO:0003712,GO:0043565,GO:0019787,GO:0019899,GO:0000122,GO:0030279,GO:0007389,GO:0070168,GO:0009887,GO:0032446,GO:0000414,GO:00	K10683|1|3e-16|89.7|ecb:100053962|BRCA1-associated RING domain protein 1 [EC:6.3.2.19]	gi|66792788|ref|NP_778210.2|	0	BCL-6 corepressor isoform c [Mus musculus]

mmu-let-7g-5p	NM_175046	-29.7	0.466341	-26.5	166	GO:0043231,GO:0043234,GO:0003712,GO:0043565,GO:0019787,GO:0019899,GO:0000122,GO:0030279,GO:0007389,GO:0070168,GO:0009887,GO:0032446,GO:0000414,GO:00	K10683|1|3e-16|89.7|ecb:100053962|BRCA1-associated RING domain protein 1 [EC:6.3.2.19]	gi|37360460|dbj|BAC98208.1|	0	mKIAA1575 protein [Mus musculus]
mmu-let-7g-5p	NM_175520	-31.4	0.124518	-28.23	178	GO:0031224,GO:0001608,GO:0043627,GO:0007166,	K08401|1|0.0|647|mmu:243270|G protein-coupled receptor 81	gi|259490314|ref|NP_780729.2|	0	hydroxycarboxylic acid receptor 1 [Mus musculus]
mmu-let-7g-5p	NM_183106	-30.5	0.254602	-27.3	172	-	-	gi|124430551|ref|NP_898929.2|	0	tetratricopeptide repeat domain 17 [Mus musculus]

mmu-let-7g-5p	NR_001461	-26.3	1	-63.82	490	-	K11849|1|6e-55|213|acs:100555596|ubiquitin carboxyl-terminal hydrolase 25/28 [EC:3.1.2.15]!K06478|4|4e-43|182|acs:100563672|protein tyrosine phosphatase, receptor type, C [EC:3.1.3.4	gi|209553980|gb|AAA39398.2|	0	ORF2 [Mus musculus domesticus]
mmu-let-7g-5p	NR_027652	-26.7	0.443663	-42.85	315	-	-	gi|148686746|gb|EDL18693.1|	1.84E-74	GTL2, imprinted maternally expressed untranslated mRNA [Mus musculus]

mmu-let-7g-5p	NR_027838	-30.9	0.272836	-26.69	163	GO:0032991,GO:0016021,GO:0005789,GO:0001664,GO:0019829,GO:0046872,GO:0032559,GO:0009888,GO:0007599,GO:0008016,GO:0006816,GO:0006936,GO:0009207,GO:0007165,GO:0001775,GO:0009206,GO:0009725,GO:0006875,	K05853|1|0.0|2035|mmu:11938|Ca2+ transporting ATPase, sarcoplasmic/endoplasmic reticulum [EC:3.6.3.8]	gi|60360584|dbj|BAD90532.1|	0	mKIAA4195 protein [Mus musculus]
mmu-let-7g-5p	NR_033299	-28.2	0.744578	-27.4	175	-	-	gi|26338105|dbj|BAC32738.1|	1.85E-55	unnamed protein product [Mus musculus]
mmu-let-7g-5p	NR_046069	-25	0.333979	-25.17	161	-	-	gi|148709166|gb|EDL41112.1|	2.52E-54	mCG1051124 [Mus musculus]
mmu-let-7g-5p	NR_046475	-26.7	0.459968	-66.31	470	-	-	-	-	-
mmu-let-7g-5p	NM_001013616	-22.4	0.999694	-104.69	762	GO:0044424,GO:0046914,GO:0051259,	K11999|1|0.0|947|mmu:94088|tripartite motif-containing protein 6/22/34	gi|61966933|ref|NP_001013637.1|	0	tripartite motif-containing protein 6 [Mus musculus]

mmu-let-7g-5p	NM_009951	-22.8	0.999998	-68.68	483	GO:0043234,GO:0030425,GO:0031981,GO:0031252,GO:0044444,GO:0030529,GO:0016020,GO:0003729,GO:0006412,GO:0033554,GO:0048255,GO:0033036,GO:00018	K13197|1|0.0|868|acs:100559269|insulin-like growth factor 2 mRNA-binding protein 3	gi|6753518|ref|NP_034081.1|	0	insulin-like growth factor 2 mRNA-binding protein 1 [Mus musculus]
mmu-let-7g-5p	NM_009109	-20.5	1	-65.29	478	GO:0016021,GO:0033017,GO:0030017,GO:0005218,GO:0004871,GO:0046872,GO:0005515,GO:0003012,GO:0051283,GO:0014074,GO:0006816,GO:0006950,	K04961|1|0.0|8764|mmu:20190|ryanodine receptor 1	gi|145046267|ref|NP_033135.2|	0	ryanodine receptor 1 [Mus musculus]
mmu-let-7g-5p	NM_178066	-20.2	0.999999	-44.03	329	-	-	gi|30017375|ref|NP_835167.1|	1.04E-130	uncharacterized protein LOC73827 [Mus musculus]

mmu-let-7g-5p	NM_178730	-20.4	1	-41.36	325	GO:0016021,GO:0004175,GO:0019538,	K09752|1|0.0|833|mmu:243083|transmembrane protease, serine 11F [EC:3.4.21.-]	gi|32189423|ref|NP_848845.1|	0	transmembrane protease serine 11F [Mus musculus]
mmu-let-7g-5p	NM_010264	-23.7	0.999075	-42.94	323	GO:0043234,GO:0031981,GO:0046914,GO:0042802,GO:0001071,GO:0003677,GO:0004879,GO:0048869,GO:0009755,GO:00482	K08561|1|0.0|892|ssc:100038028|germ cell nuclear factor	gi|6753956|ref|NP_034394.1|	0	nuclear receptor subfamily 6 group A member 1 isoform 1 [Mus musculus]

mmu-let-7g-5p	NM_001128151	-22.5	1	-43.09	320	GO:0043231,GO:0005488,GO:0000737,GO:0006810,GO:0012501,GO:0006996,	K06062|1|7e-19|99.0|dre:563942|histone acetyltransferase [EC:2.3.1.48]!K11728|2|2e-18|97.4|tca:100141790|nucleosome-remodeling factor subunit BPTF	gi|189458854|ref|NP_001121623.1|	0	cat eye syndrome critical region protein 2 [Mus musculus]
mmu-let-7g-5p	NM_009483	-22.4	0.999976	-42.34	318	GO:0034708,GO:0043231,GO:0043169,GO:0016701,GO:0034968,	K11447|1|0.0|2720|mmu:22289|histone demethylase [EC:1.14.1	gi|33859492|ref|NP_033509.1|	0	lysine-specific demethylase 6A [Mus musculus]

mmu-let-7g-5p	NM_030695	-23.6	0.999976	-43.89	317	GO:0000323,GO:0044431,GO:0031224,GO:0031410,GO:0005488,GO:0016192,GO:0023052,	K07295|1|2e-25|120|dpe:Dper_GL19043|nuclear receptor subfamily 2 group A!K11887|2|4e-10|70.1|ame:409686|proteasomal ATPase-associated factor 1!K10342|4|3e-06|57.0|mdo:100023636|WD repeat and SOCS box-containing protein 2	gi|117956399|ref|NP_109620.2|	0	lipopolysaccharide-responsive and beige-like anchor protein isoform alpha [Mus musculus]
mmu-let-7g-5p	NR_040329	-24.6	0.909282	-41.25	316	-	-	gi|148671029|gb|EDL02976.1|	2.19E-32	mCG144950 [Mus musculus]
mmu-let-7g-5p	NR_027651	-24	0.999959	-45.95	315	-	-	gi|148686746|gb|EDL18693.1|	5.00E-78	GTL2, imprinted maternally expressed untranslated mRNA [Mus musculus]

mmu-let-7g-5p	NM_146155	-24.7	0.992305	-40.82	313	-	K02350|1|2e-08|63.5|tgu:100217592|DNA polymerase zeta subunit [EC:2.7.7.7]	gi|148698133|gb|EDL30080.1|	0	AT hook, DNA binding motif, containing 1, isoform CRA_b [Mus musculus]
mmu-let-7g-5p	NM_026936	-23.7	0.955436	-42.69	312	GO:0032991,GO:0043231,GO:0019866,GO:0031301,GO:0010257,GO:0043462,GO:0045333,GO:0043086,GO:0034613,GO:00070	K03217|1|0.0|764|mmu:69089|preprotein translocase subunit YidC	gi|58037139|ref|NP_081212.1|	0	mitochondrial inner membrane protein OXA1L [Mus musculus]
mmu-let-7g-5p	NM_172416	-22.6	0.997502	-43.58	311	GO:0016020,GO:0044444,GO:0005488,GO:0002573,	-	gi|27369545|ref|NP_766004.1|	9.49E-140	osteopetrosis-associated transmembrane protein 1 precursor [Mus musculus]

mmu-let-7g-5p	NM_201395	-21.6	0.999987	-42.82	308	GO:0000785,GO:0043234,GO:0046914,GO:0003676,GO:0014020,GO:0006357,GO:0035108,GO:0001832,GO:0003231,	K09228|1|2e-13|79.3|bta:507236|KRAB domain-containing zinc finger protein	gi|117553623|ref|NP_958797.2|	0	sal-like protein 4 isoform b [Mus musculus]
mmu-let-7g-5p	NM_001214911	-20.9	1	-40.48	307	-	K13254|1|1e-25|120|xtr:549207|spastin [EC:3.6.4.3]	gi|148672121|gb|EDL04068.1|	6.82E-147	mCG52237 [Mus musculus]
mmu-let-7g-5p	NM_178280	-24.8	0.99219	-43.27	305	GO:0044424,GO:0005515,	K09228|1|5e-17|92.4|xtr:100216012|KRAB domain-containing zinc finger protein	gi|49257163|gb|AAH72631.1|	0	Sall3 protein, partial [Mus musculus]
mmu-let-7g-5p	NM_028762	-23.8	0.989806	-43.81	302	GO:0043232,GO:0031981,GO:0003676,GO:0009790,	K14787|1|0.0|1643|mmu:74111|multiple RNA-binding domain-containing protein 1	gi|30794154|ref|NP_083038.1|	0	probable RNA-binding protein 19 [Mus musculus]

mmu-let-7g-5p	NM_021716	-20	1	-40.17	299	GO:0034399,GO:0005515,GO:0016462,GO:0032559,	K13254|1|6e-65|251|phu:Phum_PHUM514830|spastin [EC:3.6.4.3]	gi|148695049|gb|EDL26996.1|	0	fidgetin, isoform CRA_b [Mus musculus]
mmu-let-7g-5p	NM_148948	-20.5	1	-27.42	184	GO:0031332,GO:0044444,GO:0016891,GO:0032559,GO:0043169,GO:0003723,GO:0042623,GO:0016246,GO:0006357,GO:0031641,GO:0022011,GO:0010624,GO:0048858,GO:00	K11592|1|0.0|3661|mmu:192119|endoribonuclease Dicer [EC:3.1.26.-]	gi|148686840|gb|EDL18787.1|	0	Dicer1, Dcr-1 homolog (Drosophila), isoform CRA_a [Mus musculus]
mmu-let-7g-5p	NM_172926	-22.4	0.99883	-26.41	178	GO:0031224,GO:0005543,GO:0060089,GO:0023052,GO:0009987,GO:0006810,	K13304|1|3e-07|58.5|mgp:100542838|serum/glucocorticoid-regulated kinase 3 [EC:2.7.11.1]	gi|238814328|ref|NP_766514.2|	0	sorting nexin-14 [Mus musculus]

mmu-let-7g-5p	NR_038041	-24	0.292453	-27.01	176	-	-	gi|148700034|gb|EDL31981.1|	1.62E-60	mCG146286 [Mus musculus]
mmu-let-7g-5p	NM_145857	-23.1	0.999117	-27.31	175	GO:0043226,GO:0044444,GO:0016020,GO:0019900,GO:0042834,GO:0032559,GO:0019904,GO:0004520,GO:0006461,GO:0006915,GO:0051091,GO:0045807,GO:0016567,GO:0043331,GO:0043433,GO:0002437,GO:0002455,GO:0002385,GO:0032495,GO:0001934,GO:0002371,GO:00328	K10165|1|0.0|1827|mmu:257632|nucleotide-binding oligomerization domain-containing protein 2	gi|29427819|sp|Q8K3Z0.1|NOD2_MOUSE	0	RecName: Full=Nucleotide-binding oligomerization domain-containing protein 2; AltName: Full=Caspase recruitment domain-containing protein 15

mmu-let-7g-5p	NR_027809	-23.5	0.998273	-26.68	174	GO:0044444,GO:0016646,GO:0000166,GO:0050662,GO:0000097,GO:0001101,GO:0033273,GO:0034097,GO:0006760,	K00297|1|0.0|1347|mmu:17769|methylenetetrahydrofolate reductase (NADPH) [EC:1.5.1.20]	gi|187466137|emb|CAQ51916.1|	0	5,10-methylenetetrahydrofolate reductase [Mus musculus]
mmu-let-7g-5p	NM_145505	-20.7	1	-27.8	173	-	-	gi|148669833|gb|EDL01780.1|	0	expressed sequence AI450540, isoform CRA_a [Mus musculus]
mmu-let-7g-5p	NM_199469	-21	1	-25.73	172	GO:0043231,GO:0044444,GO:0016020,GO:0046914,GO:0005515,GO:0016044,GO:0043161,GO:0006996,	K14015|1|0.0|1139|mmu:217365|nuclear protein localization protein 4 homolog	gi|148702796|gb|EDL34743.1|	0	nuclear protein localization 4 homolog (S. cerevisiae), isoform CRA_a [Mus musculus]
mmu-let-7g-5p	NM_172458	-23.9	0.99996	-20.83	171	-	K09228|1|0.0|1252|mmu:208292|KRAB domain-containing zinc finger protein	gi|28972890|dbj|BAC65861.1|	0	mKIAA3006 protein [Mus musculus]
mmu-let-7g-5p	NM_201368	-22	0.999972	-21.97	170	GO:0016020,	-	gi|41235770|ref|NP_958756.1|	0	XK-related protein 8 [Mus musculus]

mmu-let-7g-5p	NM_172760	-25.5	0.812933	-23.7	169	GO:0043232,GO:0019904,GO:0012501,GO:0006897,	K12366|1|0.0|732|dre:406364|engulfment and cell motility	gi|255683297|ref|NP_766348.3|	0	engulfment and cell motility protein 3 [Mus musculus]
mmu-let-7g-5p	NR_040398	-31.7	0.10358	-22.49	168	-	-	gi|149026310|gb|EDL82553.1|	5.64E-10	rCG63030 [Rattus norvegicus]
mmu-let-7g-5p	NR_045608	-20.5	1	-26.19	167	GO:0043231,GO:0046914,GO:0003676,	K11485|1|0.0|1184|aml:100480331|polycomb-like protein 2	gi|301765136|ref|XP_002917993.1|	0	PREDICTED: metal-response element-binding transcription factor 2-like [Ailuropoda melanoleuca]
mmu-let-7g-5p	NR_036630	-27.5	0.446511	-23.5	166	-	-	gi|392337668|ref|XP_003753315.1|	0	PREDICTED: inactive serine/threonine-protein kinase TEX14-like [Rattus norvegicus]
mmu-let-7g-5p	NM_199032	-25.2	0.961796	-22.89	165	GO:0005813,GO:0005515,GO:0051297,GO:0022402,	K11499|1|1e-35|153|spu:592172|centromere protein F	gi|148705959|gb|EDL37906.1|	0	centrosomal protein 135, isoform CRA_a [Mus musculus]
mmu-let-7g-5p	NM_212435	-21.4	1	-20.05	164	GO:0044464,GO:0046914,GO:0003677,GO:0001071,GO:0006351,	K09409|1|0.0|798|ecb:100055896|forkhead box protein P	gi|397466201|ref|XP_003804855.1|	0	PREDICTED: forkhead box protein P2-like [Pan paniscus]
mmu-let-7g-5p	NR_046162	-22.1	0.998007	-20.02	163	-	-	gi|148665186|gb|EDK97602.1|	7.08E-114	mCG144829 [Mus musculus]

mmu-let-7g-5p	NR_037698	-22.7	0.952886	-20.91	162	-	-	gi|148672575|gb|EDL04522.1|	6.45E-59	mCG20394, isoform CRA_b [Mus musculus]
mmu-let-7g-5p	NM_178691	-28	0.507567	-24.29	161	GO:0044464,GO:0046914,GO:0019783,GO:0043161,GO:0019752,GO:0016579,GO:0006950,GO:0006984,	K13719|1|0.0|701|mmu:226418|ubiquitin thioesterase OTU1 [EC:3.1.2.-]	gi|148707790|gb|EDL39737.1|	0	mCG2637 [Mus musculus]
mmu-let-7g-5p	NM_212449	-22	0.999809	-20.36	160	-	-	gi|148693799|gb|EDL25746.1|	2.65E-106	expressed sequence AU019823 [Mus musculus]
mmu-let-7g-5p	NR_045545	-21.5	1	-20.18	159	-	K08883|1|1e-16|90.9|bfo:BRAFLDRAFT_69357|tripartite motif-containing protein 33 [EC:6.3.2.19]!K08881|3|8e-16|87.8|mmu:21848|tripartite motif-containing protein 24	gi|357576270|ref|NP_001239513.1|	0	protein kinase C-binding protein 1 isoform 2 [Mus musculus]

mmu-let-7g-5p	NR_040526	-26.6	0.078282	-21.8	158	-	-	gi|148681716|gb|EDL13663.1|	1.70E-49	mCG145950 [Mus musculus]
mmu-let-7g-5p	NR_030694	-20.1	1	-22.98	157	-	-	gi|148669452|gb|EDL01399.1|	5.53E-56	mCG145864, isoform CRA_a [Mus musculus]
mmu-let-7g-5p	NR_045554	-26	0.77007	-21.22	156	GO:0031224,GO:0000139,GO:0046921,GO:0019904,GO:0019317,GO:0048870,GO:0043009,GO:0009311,GO:0007178,GO:00064	K00717|1|0.0|1192|mmu:53618|glycoprotein 6-alpha-L-fucosyltransferase [EC:2.4.1.68]	gi|148704495|gb|EDL36442.1|	0	fucosyltransferase 8, isoform CRA_b [Mus musculus]
mmu-let-7g-5p	NR_046181	-20.8	0.999991	-23.73	155	-	-	gi|148703515|gb|EDL35462.1|	1.53E-34	mCG148208 [Mus musculus]
mmu-let-7g-5p	NR_045891	-23.9	0.418597	-21.19	154	-	-	gi|148700318|gb|EDL32265.1|	1.10E-47	mCG144848 [Mus musculus]
mmu-let-7g-5p	NM_207708	-24	0.987477	-20.86	153	GO:0016021,GO:0008021,GO:0044459,GO:0048168,GO:0006886,	K04403|1|1e-39|166|acs:100555014|TAK1-binding protein 1	gi|46877048|ref|NP_997591.1|	1.64E-92	synaptogyrin-1 isoform 1a [Mus musculus]

mmu-let-7g-5p	NM_178664	-21.8	0.999725	-20.66	152	GO:0016740,	K07204|1|2e-66|254|spu:586135|regulatory associated protein	gi|148702899|gb|EDL34846.1|	0	RIKEN cDNA 6030413G23, isoform CRA_c [Mus musculus]
mmu-let-7g-5p	NR_073462	-24.4	0.873034	-20.05	151	-	-	-	-	-
mmu-let-7g-5p	NR_027664	-23.4	0.986605	-21.07	150	GO:0044429,GO:0043169,GO:0016614,GO:0006818,GO:0043648,	K11173|1|0.0|811|mmu:76187|hydroxyacid-oxoacid transhydrogenase [EC:1.1.99.24]	gi|30424876|ref|NP_780445.1|	0	hydroxyacid-oxoacid transhydrogenase, mitochondrial [Mus musculus]
mmu-let-7g-5p	NR_027904	-21.6	0.978377	-20.56	149	-	-	gi|74187078|dbj|BAE20553.1|	2.31E-36	unnamed protein product [Mus musculus]
mmu-let-7g-5p	NM_207651	-21.1	1	-21.54	148	GO:0044459,GO:0031224,GO:0042887,GO:0005488,GO:0015840,GO:0006950,	K08716|1|0.0|1402|cfa:490471|solute carrier family 14 (urea transporter)	gi|159131880|ref|NP_997534.2|	0	urea transporter 2 isoform a [Mus musculus]
mmu-let-7g-5p	NM_198632	-20.3	1	-22.09	147	GO:0043232,GO:0046914,	K10649|1|0.0|1382|mmu:330863|tripartite motif-containing protein	gi|254939710|ref|NP_941034.2|	0	tripartite motif-containing protein 67 [Mus musculus]

mmu-let-7g-5p	NR_033586	-22.6	0.999974	-20.27	146	-	-	gi|54887404|gb|AAH85163.1|	0	Fam120b protein [Mus musculus]
mmu-let-7g-5p	NM_198424	-21.1	0.99971	-20.62	145	-	K16058|1|3e-164|579|mmu:269999|protein Orai-3	gi|38259222|ref|NP_940816.1|	2.23E-163	protein orai-3 [Mus musculus]
mmu-let-7g-5p	NR_045935	-24.7	0.414966	-21.12	144	-	-	gi|148665704|gb|EDK98120.1|	4.81E-68	hypothetical protein B130050K08 [Mus musculus]

mmu-let-7g-5p	NM_183276	-22.1	1	-20.31	143	-	K07295|1|4e-26|122|dpe:Dper_GL19043|nuclear receptor subfamily 2 group A!K11887|2|3e-09|67.0|ame:409686|proteasomal ATPase-associated factor 1!K10342|5|7e-08|62.4|mcc:710401|WD repeat and SOCS box-containing protein 2	gi|254911027|ref|NP_899099.2|	0	neurobeachin-like protein 2 [Mus musculus]
mmu-let-7i-3p	NM_001004362	-29.4	0.483385	-25.21	144	GO:0005488,	K09228|1|0.0|1469|mmu:72128|KRAB domain-containing zinc finger protein	gi|140971855|ref|NP_001004362.2|	0	uncharacterized protein LOC72128 [Mus musculus]

mmu-let-7i-3p	NM_001024504	-32.9	0.101055	-29.16	158	-	K10624|1|6e-40|167|ame:724670|E3 ubiquitin-protein ligase RBBP6 [EC:6.3.2.19]	gi|111162659|ref|NP_001019675.2|	1.35E-149	DCN1-like protein 2 isoform a [Mus musculus]
mmu-let-7i-3p	NM_001033326	-32.5	0.080136	-25.32	158	-	K11170|1|3e-165|583|mmu:236082|dehydrogenase/reductase SDR family member X [EC:1.1.-.-	gi|124244062|ref|NP_001028498.2|	1.74E-164	dehydrogenase/reductase SDR family member on chromosome X homolog precursor [Mus musculus]

mmu-let-7i-3p	NM_001034856	-28.7	0.184821	-27.21	153	GO:0043231,GO:0000989,GO:0019899,GO:0045661,GO:0008283,GO:0008213,GO:0030858,GO:0048870,GO:0006915,GO:0044093,GO:0007281,GO:0048232,GO:0045765,GO:0006950,GO:0016049,GO:00097	K14443|1|2e-90|333|mmu:194735|protein Tob/BTG	gi|148697056|gb|EDL29003.1|	3.71E-90	mCG141009 [Mus musculus]
mmu-let-7i-3p	NM_001040187	-27.5	0.801016	-25.8	158	GO:0043231,GO:0003676,GO:0042623,GO:0004386,GO:0032559,GO:0009207,GO:0010467,	K13178|1|0.0|1122|aml:100482324|ATP-dependent RNA helicase DDX17 [EC:3.6.4.1	gi|387540018|gb|AFJ70636.1|	0	putative ATP-dependent RNA helicase DDX17 isoform 1 [Macaca mulatta]

mmu-let-7i-3p	NM_001042528	-26.5	0.967037	-25.66	162	GO:0034704,GO:0044297,GO:0030425,GO:0033267,GO:0005515,GO:0005262,GO:0032559,GO:0033555,GO:0007269,GO:0006816,GO:0008015,GO:0044057,GO:0032412,GO:0048545,GO:0007610,GO:00423	K04849|1|0.0|4145|mmu:12287|voltage-dependent calcium channel N type alpha-1B	gi|110225368|ref|NP_001035993.1|	0	voltage-dependent N-type calcium channel subunit alpha-1B isoform 1 [Mus musculus]
mmu-let-7i-3p	NM_001042649	-32.9	0.097814	-29.16	158	-	K10624|1|1e-31|94.4|ame:724670|E3 ubiquitin-protein ligase RBBP6 [EC:6.3.2.19]	gi|148690187|gb|EDL22134.1|	2.05E-133	mCG3700, isoform CRA_e [Mus musculus]

mmu-let-7i-3p	NM_001042650	-32.9	0.09402	-29.16	158	-	K10624|1|1e-17|93.2|ame:724670|E3 ubiquitin-protein ligase RBBP6 [EC:6.3.2.19]	gi|148690187|gb|EDL22134.1|	8.48E-138	mCG3700, isoform CRA_e [Mus musculus]
mmu-let-7i-3p	NM_001042651	-32.9	0.097267	-29.16	158	-	K10624|1|1e-24|109|ame:724670|E3 ubiquitin-protein ligase RBBP6 [EC:6.3.2.19]	gi|148690187|gb|EDL22134.1|	2.91E-132	mCG3700, isoform CRA_e [Mus musculus]
mmu-let-7i-3p	NM_001081041	-31	0.179778	-28.03	158	GO:0031224,GO:0043231,GO:0006810,GO:0010876,	-	gi|148701257|gb|EDL33204.1|	0	mCG11742, isoform CRA_b [Mus musculus]

mmu-let-7i-3p	NM_001081049	-29.6	0.889152	-27.28	171	GO:0035097,GO:0046914,GO:0042393,GO:0018024,GO:0001071,GO:0042802,GO:0043565,GO:0030097,GO:0043967,GO:0006357,GO:0012501,GO:0031060,GO:0008283,GO:0006305,GO:0003002,GO:0065003,GO:00	K09186|1|0.0|4637|mmu:214162|histone-lysine N-methyltransferase MLL1 [EC:2.1.1.43]	gi|124486682|ref|NP_001074518.1|	0	histone-lysine N-methyltransferase MLL [Mus musculus]
mmu-let-7i-3p	NM_001081292	-26.6	0.838225	-25.9	157	GO:0003712,GO:0042802,GO:0004709,GO:0032559,GO:0012502,GO:0000187,	K04418|1|0.0|1573|mmu:269881|mitogen-activated protein kinase kinase kinase 10 [EC:2.7.11.25]	gi|124486847|ref|NP_001074761.1|	0	mitogen-activated protein kinase kinase kinase 10 [Mus musculus]

mmu-let-7i-3p	NM_001110131	-35	0.098917	-29.32	162	GO:0005488,	K09267|1|3e-14|82.8|aga:AgaP_AGAP009957|transcription factor SOX1/2/3/14/21 (SOX group B)!K09270|4|2e-13|80.1|bfo:BRAFLDRAFT_125030|transcription factor SOX7/8/9/10/18 (SOX group E/F)!K09269|5|2e-13|80.1|bta:788459|transcripti	gi|158631198|ref|NP_001103601.1|	0	protein capicua homolog isoform b [Mus musculus]

mmu-let-7i-3p	NM_001110132	-35	0.098971	-29.32	162	GO:0005488,	K09267|1|3e-14|82.8|aga:AgaP_AGAP009957|transcription factor SOX1/2/3/14/21 (SOX group B)!K09270|4|2e-13|80.1|bfo:BRAFLDRAFT_125030|transcription factor SOX7/8/9/10/18 (SOX group E/F)!K09269|5|2e-13|80.1|bta:788459|transcripti	gi|158631217|ref|NP_001103602.1|	0	protein capicua homolog isoform c [Mus musculus]
mmu-let-7i-3p	NM_001113471	-34	0.034172	-29.89	168	GO:0044444,GO:0005488,GO:0006641,	K08768|1|0.0|932|mmu:103968|perilipin	gi|148675107|gb|EDL07054.1|	0	perilipin, isoform CRA_e [Mus musculus]
mmu-let-7i-3p	NM_001163098	-31.5	0.327725	-28.34	161	GO:0043232,GO:0046872,	K10384|1|1e-16|90.9|ecb:100060541|filaggrin	gi|251823773|ref|NP_001156570.1|	1.16E-67	trichohyalin [Mus musculus]

mmu-let-7i-3p	NM_001163445	-33	0.076412	-25.52	160	-	-	gi|148697823|gb|EDL29770.1|	4.34E-66	mCG1039243, isoform CRA_c [Mus musculus]
mmu-let-7i-3p	NM_001163569	-28.8	0.462526	-29.57	149	GO:0015630,GO:0003774,GO:0032559,GO:0007599,GO:0007017,	K10397|1|0.0|1468|mmu:16578|kinesin family member 6/9	gi|254675238|ref|NP_001157041.1|	0	kinesin-like protein KIF9 isoform 1 [Mus musculus]
mmu-let-7i-3p	NM_001163787	-32	0.099104	-26.5	170	-	K10388|1|3e-13|77.8|dre:567639|plectin	gi|148693294|gb|EDL25241.1|	0	RIKEN cDNA C330001K17, isoform CRA_c [Mus musculus]
mmu-let-7i-3p	NM_001164480	-25.9	0.908044	-26.59	152	GO:0032991,GO:0016023,GO:0005515,GO:0005099,GO:0043087,GO:0009414,GO:0007155,GO:0006996,GO:0051726,GO:0009966,GO:0016049,	K08013|1|0.0|1822|mmu:20469|signal-induced proliferation-associated gene 1	gi|60360124|dbj|BAD90281.1|	0	mKIAA4074 protein [Mus musculus]

mmu-let-7i-3p	NM_001164481	-25.9	0.921522	-26.59	152	GO:0032991,GO:0016023,GO:0005515,GO:0005099,GO:0043087,GO:0009414,GO:0007155,GO:0006996,GO:0051726,GO:0009966,GO:0016049,	K08013|1|0.0|1822|mmu:20469|signal-induced proliferation-associated gene 1	gi|60360124|dbj|BAD90281.1|	0	mKIAA4074 protein [Mus musculus]
mmu-let-7i-3p	NM_001164482	-25.9	0.904441	-26.59	152	GO:0032991,GO:0016023,GO:0005515,GO:0005099,GO:0043087,GO:0009414,GO:0007155,GO:0006996,GO:0051726,GO:0009966,GO:0016049,	K08013|1|0.0|1822|mmu:20469|signal-induced proliferation-associated gene 1	gi|60360124|dbj|BAD90281.1|	0	mKIAA4074 protein [Mus musculus]

mmu-let-7i-3p	NM_001164568	-25.9	0.906232	-26.59	152	GO:0032991,GO:0016023,GO:0005515,GO:0005099,GO:0043087,GO:0009414,GO:0007155,GO:0006996,GO:0051726,GO:0009966,GO:0016049,	K08013|1|0.0|1822|mmu:20469|signal-induced proliferation-associated gene 1	gi|60360124|dbj|BAD90281.1|	0	mKIAA4074 protein [Mus musculus]
mmu-let-7i-3p	NM_001165999	-27.8	0.751795	-25.23	151	GO:0031224,	K07523|1|1e-40|170|bfo:BRAFLDRAFT_64092|netrin-G1 ligand	gi|260268510|ref|NP_780725.2|	0	leucine-rich repeat and immunoglobulin-like domain-containing nogo receptor-interacting protein 2 precursor [Mus musculus]
mmu-let-7i-3p	NM_001166000	-27.8	0.708519	-25.23	151	GO:0031224,	K07523|1|1e-40|170|bfo:BRAFLDRAFT_64092|netrin-G1 ligand	gi|260268510|ref|NP_780725.2|	0	leucine-rich repeat and immunoglobulin-like domain-containing nogo receptor-interacting protein 2 precursor [Mus musculus]
mmu-let-7i-3p	NM_001166001	-27.8	0.656618	-25.23	151	GO:0031224,	K07523|1|9e-41|170|bfo:BRAFLDRAFT_64092|netrin-G1 ligand	gi|260268510|ref|NP_780725.2|	0	leucine-rich repeat and immunoglobulin-like domain-containing nogo receptor-interacting protein 2 precursor [Mus musculus]

mmu-let-7i-3p	NM_001193305	-31.5	0.212308	-26.29	165	GO:0043232,GO:0046914,GO:0016491,GO:0008152,	K06115|1|2e-20|102|mmu:20741|spectrin beta	gi|60360344|dbj|BAD90416.1|	0	mKIAA0750 protein [Mus musculus]
mmu-let-7i-3p	NM_001195004	-31.4	0.341117	-26.72	154	GO:0043234,GO:0031981,GO:0004468,GO:0046914,GO:0001071,GO:0043967,GO:0006259,GO:0006351,	K11307|1|0.0|1115|ptr:455108|histone acetyltransferase MYST2 [EC:2.3.1.48]	gi|303521989|ref|NP_001181933.1|	0	histone acetyltransferase KAT7 isoform 2 [Mus musculus]
mmu-let-7i-3p	NM_001195485	-33.9	0.046663	-30.72	161	-	K12896|1|9e-56|216|ptr:459167|splicing factor, arginine/serine-rich 7	gi|148706565|gb|EDL38512.1|	5.62E-57	mCG17902, isoform CRA_k [Mus musculus]
mmu-let-7i-3p	NM_001195486	-33.9	0.046017	-30.72	161	-	K12896|1|9e-56|216|ptr:459167|splicing factor, arginine/serine-rich 7	gi|148706565|gb|EDL38512.1|	5.58E-57	mCG17902, isoform CRA_k [Mus musculus]

mmu-let-7i-3p	NM_001195487	-33.9	0.045695	-30.72	161	-	K12896|1|9e-56|216|ptr:459167|splicing factor, arginine/serine-rich 7	gi|148706565|gb|EDL38512.1|	5.54E-57	mCG17902, isoform CRA_k [Mus musculus]
mmu-let-7i-3p	NM_001205204	-36.7	0.063272	-28.1	162	GO:0031224,GO:0016337,GO:0007610,GO:0048870,	-	gi|327199303|ref|NP_001192133.1|	0	astrotactin-1 isoform 1 precursor [Mus musculus]
mmu-let-7i-3p	NM_001205353	-31.9	0.215434	-26.59	164	GO:0005740,GO:0031224,GO:0012501,	-	gi|329663552|ref|NP_001192282.1|	0	GRAM domain-containing protein 4 isoform 2 [Mus musculus]
mmu-let-7i-3p	NM_001252525	-30.9	0.225154	-26.81	166	GO:0043005,GO:0044459,GO:0030529,GO:0044430,GO:0003723,GO:0006412,GO:0006396,GO:0048167,GO:0007129,	K02602|1|0.0|1076|mmu:12877|oo18 RNA-binding protein	gi|357430798|ref|NP_001239454.1|	0	cytoplasmic polyadenylation element-binding protein 1 isoform 1 [Mus musculus]

mmu-let-7i-3p	NM_001252526	-30.9	0.223833	-26.81	166	GO:0043005,GO:0044459,GO:0030529,GO:0044430,GO:0003723,GO:0006412,GO:0006396,GO:0048167,GO:0007129,	K02602|1|0.0|1063|mmu:12877|oo18 RNA-binding protein	gi|357430802|ref|NP_001239455.1|	0	cytoplasmic polyadenylation element-binding protein 1 isoform 3 [Mus musculus]
mmu-let-7i-3p	NM_007495	-36.7	0.063029	-28.1	162	GO:0031224,GO:0016337,GO:0007610,GO:0048870,	-	gi|46488926|ref|NP_031521.2|	0	astrotactin-1 isoform 2 precursor [Mus musculus]
mmu-let-7i-3p	NM_007579	-26.5	0.969495	-25.66	162	GO:0034704,GO:0044297,GO:0030425,GO:0033267,GO:0005515,GO:0005262,GO:0032559,GO:0033555,GO:0007269,GO:0006816,GO:0008015,GO:0044057,GO:0032412,GO:0048545,GO:0007610,GO:00423	K04849|1|0.0|4026|mmu:12287|voltage-dependent calcium channel N type alpha-1B	gi|110225366|ref|NP_031605.2|	0	voltage-dependent N-type calcium channel subunit alpha-1B isoform 2 [Mus musculus]

mmu-let-7i-3p	NM_007755	-30.9	0.224934	-26.81	166	GO:0043005,GO:0044459,GO:0030529,GO:0044430,GO:0003723,GO:0006412,GO:0006396,GO:0048167,GO:0007129,	K02602|1|0.0|1081|mmu:12877|oo18 RNA-binding protein	gi|6681003|ref|NP_031781.1|	0	cytoplasmic polyadenylation element-binding protein 1 isoform 2 [Mus musculus]
mmu-let-7i-3p	NM_008213	-26.9	0.551555	-27.1	154	GO:0031981,GO:0003712,GO:0001071,GO:0046983,GO:0042802,GO:0043565,GO:0008134,GO:0000122,GO:0045165,GO:0055008,GO:0003143,GO:0003211,GO:0060706,GO:0001825,GO:0001704,	K09071|1|1e-99|364|cfa:489164|heart-and neural crest derivatives-expressed protein	gi|29504798|gb|AAH50182.1|	1.81E-90	Heart and neural crest derivatives expressed transcript 1 [Mus musculus]

mmu-let-7i-3p	NM_008242	-31.7	0.126534	-28	157	GO:0043231,GO:0043234,GO:0005515,GO:0000981,GO:0043565,GO:0043566,GO:0000122,GO:0001656,GO:0001667,GO:0060600,GO:0048675,GO:0050931,GO:0072130,GO:0030509,GO:0021782,	K09397|1|7e-71|269|mmu:15229|forkhead box protein D	gi|157057168|ref|NP_032268.2|	4.84E-70	forkhead box protein D1 [Mus musculus]
mmu-let-7i-3p	NM_008278	-30.4	0.140671	-28.01	147	GO:0031981,GO:0044444,GO:0051287,GO:0016616,GO:0042802,GO:0004955,GO:0000038,GO:0051726,GO:0006692,GO:0022414,GO:0007178,	K00069|1|8e-151|534|mmu:15446|15-hydroxyprostaglandin dehydrogenase (NAD) [EC:1.1.1.141]	gi|124486706|ref|NP_032304.2|	5.21E-150	15-hydroxyprostaglandin dehydrogenase [NAD(+)] [Mus musculus]

mmu-let-7i-3p	NM_008891	-35.8	0.0365	-29.24	169	GO:0043234,GO:0005681,GO:0016604,GO:0005911,GO:0005488,GO:0000377,GO:00071	K13114|1|0.0|726|mmu:18949|pinin	gi|148704736|gb|EDL36683.1|	0	pinin [Mus musculus]
mmu-let-7i-3p	NM_008983	-28.5	0.75023	-26.97	154	GO:0031513,GO:0043005,GO:0070161,GO:0044297,GO:0016021,GO:0031252,GO:0004871,GO:0004725,GO:0019900,GO:0034613,GO:0007178,GO:0000302,GO:0009411,GO:0008285,GO:0007044,GO:0030030,GO:0051726,GO:0006470,GO:00164	K06776|1|0.0|2856|mmu:19272|protein tyrosine phosphatase, receptor type, K [EC:3.1.3.48]	gi|6679561|ref|NP_033009.1|	0	receptor-type tyrosine-protein phosphatase kappa precursor [Mus musculus]

mmu-let-7i-3p	NM_009208	-31.8	0.230062	-26.51	148	GO:0016021,GO:0015301,GO:0015698,	K13856|1|0.0|1868|mmu:20536|solute carrier family 4 (anion exchanger), member 3	gi|165377246|ref|NP_033234.2|	0	anion exchange protein 3 [Mus musculus]
mmu-let-7i-3p	NM_009349	-30.7	0.063388	-28.57	157	-	K00562|1|3e-137|488|mmu:21743|methyltransferase [EC:2.1.1.4	gi|6678281|ref|NP_033375.1|	2.09E-136	indolethylamine N-methyltransferase [Mus musculus]

mmu-let-7i-3p	NM_010350	-29.1	0.580138	-25.07	151	GO:0030313,GO:0008328,GO:0044430,GO:0005231,GO:0004970,GO:0019904,GO:0033036,GO:0007166,GO:0009605,GO:0040011,GO:0009895,GO:0007610,GO:0051899,GO:0050804,GO:0006811,GO:0006950,GO:00	K05211|1|0.0|2230|mmu:14813|glutamate [NMDA] receptor subunit epsilon-3	gi|75709202|ref|NP_034480.2|		0	glutamate [NMDA] receptor subunit epsilon-3 precursor [Mus musculus]
mmu-let-7i-3p	NM_010628	-28.8	0.412175	-29.57	149	GO:0015630,GO:0003774,GO:0032559,GO:0007599,GO:0007017,	K10397|1|0.0|1392|mmu:16578|kinesin family member 6/9	gi|254675236|ref|NP_034758.2|		0	kinesin-like protein KIF9 isoform 2 [Mus musculus]
mmu-let-7i-3p	NM_011116	-36.6	0.014332	-32.02	169	GO:0031090,GO:0043231,GO:0031224,GO:0005488,GO:0004620,GO:0006629,	-	gi|7242181|ref|NP_035246.1|		0	phospholipase D3 [Mus musculus]

mmu-let-7i-3p	NM_011379	-25.9	0.92	-26.59	152	GO:0032991,GO:0016023,GO:0005515,GO:0005099,GO:0043087,GO:0009414,GO:0007155,GO:0006996,GO:0051726,GO:0009966,GO:0016049,	K08013|1|0.0|1822|mmu:20469|signal-induced proliferation-associated gene 1	gi|60360124|dbj|BAD90281.1|	0	mKIAA4074 protein [Mus musculus]
mmu-let-7i-3p	NM_011498	-27.3	0.682401	-25.09	155	GO:0031981,GO:0003676,GO:0001071,GO:0009314,GO:0048731,GO:0006355,GO:0048511,GO:0048167,GO:0009605,	K03729|1|0.0|721|mmu:20893|class B basic helix-loop-helix protein 2	gi|6755680|ref|NP_035628.1|	0	class E basic helix-loop-helix protein 40 [Mus musculus]

mmu-let-7i-3p	NM_011714	-30.3	0.508337	-25.18	155	GO:0070603,GO:0000775,GO:0005657,GO:0046914,GO:0042393,GO:0030546,GO:0004713,GO:0032559,GO:0008134,GO:0006351,GO:0006337,GO:0010468,GO:0006468,GO:0010469,GO:0006338,GO:0009887,GO:00	K11658|1|0.0|2409|mmu:22385|bromodomain adjacent to zinc finger domain protein 1B	gi|170295818|ref|NP_035844.2|	0	tyrosine-protein kinase BAZ1B [Mus musculus]

mmu-let-7i-3p	NM_016847	-27.4	0.60801	-25.98	149	GO:0031224,GO:0012506,GO:0008528,GO:0031893,GO:0002027,GO:0009414,GO:0019229,GO:0032846,GO:0019932,GO:0051384,GO:0005981,GO:0060746,GO:0030900,GO:0016049,GO:0044060,GO:0014047,GO:0001516,GO:0001990,GO:0051146,GO:0002118,GO:0008283,GO:00071	K04226|1|0.0|821|mmu:54140|arginine vasopressin receptor 1A	gi|33149329|ref|NP_058543.2|	0	vasopressin V1a receptor [Mus musculus]
mmu-let-7i-3p	NM_019429	-27.7	0.47805	-25.14	157	GO:0031410,GO:0070011,GO:0009057,GO:0019538,	K09649|1|0.0|922|mmu:54373|protease, serine, 16 (thymus) [EC:3.4.-.-]	gi|9506995|ref|NP_062302.1|	0	thymus-specific serine protease precursor [Mus musculus]

mmu-let-7i-3p	NM_022427	-35	0.05021	-30.86	157	GO:0004871,	K08422|1|2e-89|331|mmu:64378|G protein-coupled receptor 88	gi|11967953|ref|NP_071872.1|	1.52E-88	probable G-protein coupled receptor 88 [Mus musculus]
mmu-let-7i-3p	NM_023485	-27.2	0.532785	-26.6	158	GO:0043234,GO:0005886,GO:0045202,GO:0031674,GO:0005488,	K10377|1|0.0|816|mmu:68828|syncoilin 1	gi|47059478|ref|NP_075974.3|	0	syncoilin [Mus musculus]
mmu-let-7i-3p	NM_023644	-33.3	0.083811	-55.93	325	GO:0019866,GO:0044429,GO:0019842,GO:0043169,GO:0016879,GO:0016421,GO:0032559,GO:0006551,GO:0006790,GO:00090	K01968|1|0.0|1346|mmu:72039|3-methylcrotonyl-CoA carboxylase alpha subunit [EC:6.4.1.4]	gi|186700620|ref|NP_076133.3|	0	methylcrotonoyl-CoA carboxylase subunit alpha, mitochondrial [Mus musculus]

mmu-let-7i-3p	NM_025289	-27.7	0.366489	-25.21	159	GO:0043231,GO:0003676,GO:0006259,GO:0008283,GO:0051169,GO:0022402,GO:0031647,	K09188|1|1e-13|79.0|dsi:Dsim_GD16408|histone-lysine N-methyltransferase MLL3 [EC:2.1.1.43]	gi|224967132|ref|NP_079565.2|	5.33E-178	transforming growth factor beta regulator 1 [Mus musculus]
mmu-let-7i-3p	NM_025588	-35	0.065483	-32.94	170	GO:0043234,GO:0017016,GO:0017157,	-	gi|21313438|ref|NP_079864.1|	0	exocyst complex component 2 [Mus musculus]
mmu-let-7i-3p	NM_026609	-29	0.369387	-25.02	168	GO:0031224,	-	gi|355699236|gb|AES01062.1|	3.82E-73	leptin receptor overlapping transcript-like 1 [Mustela putorius furo]
mmu-let-7i-3p	NM_026779	-30.9	0.206075	-26.93	164	GO:0044444,GO:0046914,GO:0048037,GO:0003824,GO:0006766,GO:0009108,GO:0042559,	K15631|1|0.0|1759|mmu:68591|molybdenum cofactor sulfurtransferase [EC:2.8.1.9]	gi|110625679|ref|NP_081055.1|	0	molybdenum cofactor sulfurase [Mus musculus]
mmu-let-7i-3p	NM_027834	-30.6	0.168854	-30.86	157	-	-	gi|58037251|ref|NP_082110.1|	1.56E-101	uncharacterized protein LOC71583 [Mus musculus]

mmu-let-7i-3p	NM_027864	-32.1	0.107993	-26.29	169	GO:0012505,GO:0043231,GO:0031224,GO:0030246,GO:0008376,	K00710|1|0.0|1145|mmu:71685|polypeptide N-acetylgalactosaminyltransferase [EC:2.4.1.41]	gi|148706466|gb|EDL38413.1|	0	UDP-N-acetyl-alpha-D-galactosamine:polypeptide N-acetylgalactosaminyltransferase 14, isoform CRA_b [Mus musculus]

mmu-let-7i-3p	NM_027882	-35	0.098971	-29.32	162	GO:0005488,	K09267|1|3e-14|82.8|aga:AgaP_AGAP009957|transcription factor SOX1/2/3/14/21 (SOX group B)!K09270|4|2e-13|80.1|bfo:BRAFLDRAFT_125030|transcription factor SOX7/8/9/10/18 (SOX group E/F)!K09269|5|2e-13|80.1|bta:788459|transcripti	gi|158631191|ref|NP_082158.2|	0	protein capicua homolog isoform a [Mus musculus]

mmu-let-7i-3p	NM_028474	-26.5	0.965691	-25.13	159	-	K12385|1|4e-21|105|phu:Phum_PHUM580700|Niemann-Pick C1 protein!K11101|4|3e-16|89.7|xla:397836|patched 2	gi|257743473|ref|NP_082750.1|	0	patched domain-containing protein 4 [Mus musculus]
mmu-let-7i-3p	NM_029933	-36.2	0.062684	-29.29	173	GO:0005488,GO:0007179,	K06236|1|5e-12|57.8|aml:100472350|collagen, type I/II/III/V/XI, alpha!K06237|4|3e-08|62.4|hsa:1287|collagen, type IV, alpha	gi|56606137|ref|NP_084209.3|	0	B-cell CLL/lymphoma 9 protein [Mus musculus]

mmu-let-7i-3p	NM_029939	-32	0.098941	-26.5	170	-	K10388|1|3e-13|77.8|dre:567639|plectin!K09291|5|1e-11|72.4|mdo:100019129|nucleoprotein TPR	gi|148693294|gb|EDL25241.1|	0	RIKEN cDNA C330001K17, isoform CRA_c [Mus musculus]
mmu-let-7i-3p	NM_134086	-30.1	0.602105	-27.49	164	GO:0031224,GO:0043005,GO:0015175,GO:0005343,GO:0015804,GO:0001505,GO:0006807,GO:0030001,	K14990|1|0.0|841|mmu:105727|solute carrier family 38 (sodium-coupled neutral amino acid transporter), member 1	gi|148672291|gb|EDL04238.1|	0	solute carrier family 38, member 1, isoform CRA_a [Mus musculus]
mmu-let-7i-3p	NM_144838	-41.6	0.002261	-38.84	180	GO:0031072,GO:0044267,	K09553|1|6e-21|103|nvi:100119701|stress-induced-phosphoprotein 1	gi|21450231|ref|NP_659087.1|	3.65E-159	small glutamine-rich tetratricopeptide repeat-containing protein beta [Mus musculus]

mmu-let-7i-3p	NM_145829	-31.5	0.11515	-26.51	162	GO:0044429,GO:0008080,GO:0006525,GO:0019627,	K11067|1|0.0|904|mmu:217214|N-acetylglutamate synthase [EC:2.3.1.1]	gi|22003874|ref|NP_665828.1|	0	N-acetylglutamate synthase, mitochondrial isoform 1 [Mus musculus]
mmu-let-7i-3p	NM_146083	-33.9	0.046905	-30.72	161	-	K12896|1|9e-56|216|ptr:459167|splicing factor, arginine/serine-rich 7	gi|148706565|gb|EDL38512.1|	5.65E-57	mCG17902, isoform CRA_k [Mus musculus]
mmu-let-7i-3p	NM_172283	-36.2	0.035792	-28.28	161	GO:0044424,GO:0016301,GO:0032559,GO:0006796,	K05305|1|0.0|2045|mmu:234730|fucokinase [EC:2.7.1.52]	gi|148679529|gb|EDL11476.1|	0	fucokinase, isoform CRA_b [Mus musculus]
mmu-let-7i-3p	NM_172296	-33.2	0.085678	-27.41	154	-	-	gi|112821685|ref|NP_758500.2|	3.15E-172	doublesex- and mab-3-related transcription factor A2 [Mus musculus]
mmu-let-7i-3p	NM_172487	-29.8	0.348532	-25.92	164	-	-	gi|148667066|gb|EDK99482.1|	0	proline-rich transmembrane protein 3, isoform CRA_c [Mus musculus]

mmu-let-7i-3p	NM_172549	-30.4	0.54713	-26.6	161	GO:0043231,GO:0019903,GO:0019212,GO:0006325,GO:0010941,GO:0007165,	-	gi|70995287|ref|NP_766137.2|	0	calcineurin binding protein 1 [Mus musculus]
mmu-let-7i-3p	NM_172663	-33.7	0.111174	-30.52	179	GO:0043231,GO:0006259,GO:0006351,GO:0006325,GO:0033554,	K11322|1|0.0|1316|mmu:227867|enhancer of polycomb-like protein	gi|157042787|ref|NP_766251.3|	0	enhancer of polycomb homolog 2 [Mus musculus]
mmu-let-7i-3p	NM_175183	-31.7	0.159082	-25.14	153	-	K11318|1|0.0|1261|mmu:72522|ataxin-7	gi|148670011|gb|EDL01958.1|	0	ataxin 7-like 2, isoform CRA_b [Mus musculus]
mmu-let-7i-3p	NM_175399	-31.8	0.082278	-25.27	154	GO:0043234,GO:0031981,GO:0000785,GO:0044444,GO:0003723,GO:0008408,GO:0016049,GO:0006364,GO:0006304,GO:00002	K11600|1|2e-121|437|mmu:109075|exosome complex component RRP41	gi|148697598|gb|EDL29545.1|	5.00E-135	exosome component 4, isoform CRA_b [Mus musculus]

mmu-let-7i-3p	NM_175450	-35.7	0.030763	-29.41	172	-	K14829|1|2e-148|527|xla:734559|pre-rRNA-processing protein IPI3	gi|83649741|ref|NP_780659.2|	0	WD repeat-containing protein 18 [Mus musculus]
mmu-let-7i-3p	NM_175516	-27.8	0.667709	-25.23	151	GO:0031224,	K07523|1|9e-41|170|bfo:BRAFLDRAFT_64092|netrin-G1 ligand	gi|260268510|ref|NP_780725.2|	0	leucine-rich repeat and immunoglobulin-like domain-containing nogo receptor-interacting protein 2 precursor [Mus musculus]
mmu-let-7i-3p	NM_175631	-28.8	0.419095	-46.68	319	GO:0044459,	K03987|1|5e-06|54.3|bta:617435|complement C1q subcomponent subunit B	gi|157822519|ref|NP_001102680.1|	2.68E-88	cerebellin-4 precursor [Rattus norvegicus]
mmu-let-7i-3p	NM_175640	-34	0.035585	-29.89	168	GO:0044444,GO:0005488,GO:0006641,	K08768|1|0.0|932|mmu:103968|perilipin	gi|148675107|gb|EDL07054.1|	0	perilipin, isoform CRA_e [Mus musculus]
mmu-let-7i-3p	NM_176833	-29.6	0.510691	-27.34	158	GO:0043234,GO:0043169,GO:0004721,GO:0006464,GO:0012501,	K01090|1|0.0|848|mmu:68606|protein phosphatase [EC:3.1.3.16]	gi|28849881|ref|NP_789803.1|	0	protein phosphatase 1F [Mus musculus]

mmu-let-7i-3p	NM_177282	-31.5	0.368438	-26.29	165	GO:0043232,GO:0046914,GO:0016491,GO:0008152,	K06115|1|4e-20|102|mmu:20741|spectrin beta	gi|148685080|gb|EDL17027.1|	0	microtubule associated monoxygenase, calponin and LIM domain containing 2, isoform CRA_b [Mus musculus]
mmu-let-7i-3p	NM_177362	-29.6	0.1311	-25.34	158	GO:0043231,GO:0046914,GO:0003676,GO:0010467,	K09228|1|9e-81|301|mmu:54201|KRAB domain-containing zinc finger protein	gi|38571662|gb|AAH62882.1|	4.05E-164	Zfp771 protein [Mus musculus]
mmu-let-7i-3p	NM_177619	-31.4	0.332256	-26.72	154	GO:0043234,GO:0031981,GO:0004468,GO:0046914,GO:0001071,GO:0043967,GO:0006259,GO:0006351,	K11307|1|0.0|981|cfa:491072|histone acetyltransferase MYST2 [EC:2.3.1.48]	gi|148684025|gb|EDL15972.1|	0	MYST histone acetyltransferase 2, isoform CRA_b [Mus musculus]
mmu-let-7i-3p	NM_178679	-28.8	0.574518	-26.7	154	GO:0043231,GO:0046914,	K10316|1|1e-32|143|acs:100562459|F-box protein	gi|30520065|ref|NP_848794.1|	0	protein ZNF365 [Mus musculus]

mmu-let-7i-3p	NM_183152	-38.3	0.006266	-33.85	171	GO:0043169,GO:0004672,GO:0032559,GO:0006464,GO:0023052,	K08861|1|6e-122|439|xtr:100125189|polo-like kinase 2 [EC:2.7.11.21]	gi|65508357|ref|NP_898975.2|	0	inactive serine/threonine-protein kinase PLK5 [Mus musculus]
mmu-let-7i-3p	NM_199079	-27.5	0.618998	-25.8	158	GO:0043231,GO:0003676,GO:0042623,GO:0004386,GO:0032559,GO:0009207,GO:0010467,	K13178|1|0.0|817|ssc:100514347|ATP-dependent RNA helicase DDX17 [EC:3.6.4.13]	gi|387542724|gb|AFJ71989.1|	0	putative ATP-dependent RNA helicase DDX17 isoform 3 [Macaca mulatta]
mmu-let-7i-3p	NM_199080	-27.5	0.801405	-25.8	158	GO:0043231,GO:0003676,GO:0042623,GO:0004386,GO:0032559,GO:0009207,GO:0010467,	K13178|1|0.0|1126|aml:100482324|ATP-dependent RNA helicase DDX17 [EC:3.6.4.1	gi|387542724|gb|AFJ71989.1|	0	putative ATP-dependent RNA helicase DDX17 isoform 3 [Macaca mulatta]

mmu-let-7i-3p	NM_201639	-25.7	0.994241	-44.63	304	GO:0043234,GO:0045111,GO:0030016,GO:0070161,GO:0008092,GO:0032403,GO:0005198,GO:0007010,	K10376|1|0.0|2403|rno:308709|desmuslin	gi|153791809|ref|NP_964001.2|	0	synemin isoform H [Mus musculus]
mmu-let-7i-3p	NM_207663	-25.7	0.990494	-44.63	304	GO:0043234,GO:0045111,GO:0030016,GO:0070161,GO:0008092,GO:0032403,GO:0005198,GO:0007010,	K10376|1|0.0|1764|rno:308709|desmuslin	gi|153792766|ref|NP_997546.2|	0	synemin isoform M [Mus musculus]
mmu-let-7i-3p	NR_036615	-33.9	0.059138	-30.72	161	-	K12896|1|1e-55|216|ptr:459167|splicing factor, arginine/serine-rich 7	gi|148706565|gb|EDL38512.1|	6.97E-57	mCG17902, isoform CRA_k [Mus musculus]
mmu-let-7i-3p	NR_045866	-31.7	0.064294	-28	159	-	-	gi|74184390|dbj|BAE25724.1|	5.18E-60	unnamed protein product [Mus musculus]
mmu-let-7i-3p	NR_052009	-36.2	0.036962	-28.28	161	-	-	-	-	-

mmu-let-7i-3p	NM_027497	-20.5	1	-31.99	172	GO:0043189,GO:0005635,GO:0004468,GO:0006357,GO:0010629,GO:0016573,	K11322|1|0.0|1210|mmu:13831|enhancer of polycomb-like protein	gi|27532963|ref|NP_081773.1|	0	enhancer of polycomb homolog 1 isoform 2 [Mus musculus]
mmu-let-7i-3p	NM_138753	-20	1	-27.86	170	GO:0031981,GO:0003723,GO:0016538,GO:0006357,GO:0048513,GO:0000079,	K15189|1|1e-168|595|mmu:192231|protein HEXIM1/2	gi|20270289|ref|NP_620092.1|	7.23E-168	protein HEXIM1 [Mus musculus]
mmu-let-7i-3p	NM_019636	-26.8	0.911156	-22.58	169	GO:0043231,GO:0005099,GO:0032313,GO:0008104,	K11837|1|6e-12|75.1|hsa:9098|ubiquitin carboxyl-terminal hydrolase 6/32 [EC:3.1.2.15]!K13136|2|4e-10|68.9|nvi:100114269|gem associated protein 8	gi|120587003|ref|NP_062610.2|	0	TBC1 domain family member 1 [Mus musculus]

mmu-let-7i-3p	NM_011742	-29.5	0.216993	-24.31	168	GO:0043231,GO:0046914,GO:0003676,GO:0006351,	K09228|1|0.0|813|mmu:22640|KRAB domain-containing zinc finger protein	gi|55483|emb|CAA34510.1|	0	unnamed protein product [Mus musculus]
mmu-let-7i-3p	NM_139130	-29.3	0.176038	-23.88	167	GO:0003779,GO:0007010,	K13887|1|0.0|699|dre:562849|coronin-1C	gi|41281703|ref|NP_624356.1|	0	coronin-6 isoform B [Mus musculus]
mmu-let-7i-3p	NM_205823	-27.9	0.597314	-21.86	166	GO:0031224,GO:0004872,GO:0006952,GO:0023052,	K14142|1|1e-127|458|ecb:100072493|toll-like receptor 11!K05401|4|4e-20|101|bfo:BRAFLDRAFT_97448|toll-like receptor 3	gi|45430001|ref|NP_991392.1|	0	toll-like receptor 12 precursor [Mus musculus]

mmu-let-7i-5p	NM_001007465	-30.2	0.236576	-28.38	167	GO:0043227,GO:0031410,GO:0046914,GO:0003824,GO:0012501,GO:0015031,	K10871|1|5e-70|267|mcc:718665|RAD51-like protein 3!K10641|3|3e-10|68.6|tad:TRIADDRAFT_53391|E3 ubiquitin-protein ligase LRSAM1 [EC:6.3.2.19]!K16060|4|4e-09|65.1|spu:581540|baculoviral IAP repeat-containing protein 2/3!K10637|5|5e-	gi|148683726|gb|EDL15673.1|	0	ring finger and FYVE like domain containing protein, isoform CRA_c [Mus musculus]
mmu-let-7i-5p	NM_001013774	-26.7	0.535062	-40.89	312	GO:0005634,GO:0006886,	K15043|1|2e-132|474|xla:398027|importin subunit alpha-2	gi|144446032|ref|NP_001013796.2|	0	importin subunit alpha-8 [Mus musculus]

mmu-let-7i-5p	NM_001031772	-25.7	0.944172	-46.49	314	GO:0043231,GO:0003676,GO:0046914,GO:0035196,GO:0010586,GO:0006351,	K09276|1|2e-10|70.1|spu:580680|Y-box-binding protein 1!K09277|2|2e-10|70.1|xtr:100170415|Y-box-binding	gi|78042603|ref|NP_001026942.1|	7.05E-160	protein lin-28 homolog B [Mus musculus]
mmu-let-7i-5p	NM_001042591	-28.2	0.532878	-25.36	167	GO:0044424,GO:0005488,GO:0023052,	K15183|1|1e-07|60.1|mgp:100549853|RNA polymerase II elongation factor ELL	gi|28972730|dbj|BAC65781.1|	0	mKIAA1376 protein [Mus musculus]
mmu-let-7i-5p	NM_001081068	-28.9	0.661237	-25.53	167	GO:0003824,GO:0046914,	-	gi|50510603|dbj|BAD32287.1|	0	mKIAA0714 protein [Mus musculus]
mmu-let-7i-5p	NM_001081298	-31.3	0.269469	-28.8	179	GO:0031224,GO:0019904,GO:0030246,GO:0004888,GO:0007417,GO:0007186,	K04593|1|0.0|2921|mmu:99633|latrophilin 2	gi|124486821|ref|NP_001074767.1|	0	latrophilin-2 precursor [Mus musculus]

mmu-let-7i-5p	NM_001085502	-29.4	0.084755	-25.07	168	GO:0044444,GO:0008252,GO:0046872,GO:0006213,GO:0006163,GO:0009112,GO:0046128,GO:0006796,	K01081|1|0.0|682|mmu:230718|5'-nucleotidase [EC:3.1.3.5]	gi|148227160|ref|NP_001078971.1|	0	cytosolic 5'-nucleotidase 1A [Mus musculus]
mmu-let-7i-5p	NM_001109743	-30	0.22812	-27.63	154	GO:0043231,GO:0005488,GO:0003712,GO:0006357,	-	gi|157841282|ref|NP_001103213.1|	0	SKI family transcriptional corepressor 2 [Mus musculus]

mmu-let-7i-5p	NM_001110140	-29.5	0.387719	-48.12	316	GO:0032991,GO:0016021,GO:0005789,GO:0001664,GO:0019829,GO:0046872,GO:0032559,GO:0009888,GO:0007599,GO:0008016,GO:0006816,GO:0006936,GO:0009207,GO:0007165,GO:0001775,GO:0009206,GO:0009725,GO:0006875,	K05853|1|0.0|2035|mmu:11938|Ca2+ transporting ATPase, sarcoplasmic/endoplasmic reticulum [EC:3.6.3.8]	gi|60360584|dbj|BAD90532.1|	0	mKIAA4195 protein [Mus musculus]
mmu-let-7i-5p	NM_001113283	-33.8	0.071933	-29.4	166	-	-	gi|50510907|dbj|BAD32439.1|	0	mKIAA1370 protein [Mus musculus]
mmu-let-7i-5p	NM_001122730	-30.3	0.558047	-28.86	159	-	-	gi|170172511|ref|NP_001116202.1|	0	trinucleotide repeat-containing gene 18 protein isoform A [Mus musculus]
mmu-let-7i-5p	NM_001122843	-30.7	0.2725	-25.26	145	GO:0043231,GO:0005048,GO:0022892,GO:0015031,	K14293|1|4e-14|82.0|acs:100557760|importin subunit beta-1	gi|170932528|ref|NP_663365.3|	0	transportin-2 [Mus musculus]

mmu-let-7i-5p	NM_001142539	-26.5	0.431754	-25.68	163	GO:0031224,	-	gi|187956197|gb|AAI47831.1|	0	Unc-93 homolog A (C. elegans) [Mus musculus]
mmu-let-7i-5p	NM_001145948	-27.6	0.41281	-63.38	463	-	-	gi|225543132|ref|NP_001139420.1|	0	tetratricopeptide repeat protein 39A isoform 1 [Mus musculus]
mmu-let-7i-5p	NM_001159548	-25	0.9843	-41.28	319	GO:0043234,GO:0031981,GO:0046914,GO:0042802,GO:0001071,GO:0003677,GO:0004879,GO:0048869,GO:0009755,GO:00482	K08561|1|0.0|871|mmu:14536|germ cell nuclear factor	gi|227116316|ref|NP_001153020.1|	0	nuclear receptor subfamily 6 group A member 1 isoform 2 [Mus musculus]
mmu-let-7i-5p	NM_001159549	-25	0.984271	-41.28	319	GO:0043234,GO:0031981,GO:0046914,GO:0042802,GO:0001071,GO:0003677,GO:0004879,GO:0048869,GO:0009755,GO:00482	K08561|1|0.0|899|mmu:14536|germ cell nuclear factor	gi|227116318|ref|NP_001153021.1|	0	nuclear receptor subfamily 6 group A member 1 isoform 3 [Mus musculus]

mmu-let-7i-5p	NM_001161763	-26.7	0.839034	-46.02	311	GO:0005789,GO:0031224,GO:0016709,GO:0050662,GO:0000166,GO:0008152,	K00485|1|0.0|1078|mmu:14263|dimethylaniline monooxygenase (N-oxide forming) [EC:1.14.13.8]	gi|46559382|ref|NP_034362.2|	0	dimethylaniline monooxygenase [N-oxide-forming] 5 [Mus musculus]
mmu-let-7i-5p	NM_001161765	-26.7	0.843475	-46.02	311	GO:0005789,GO:0031224,GO:0016709,GO:0050662,GO:0000166,GO:0008152,	K00485|1|0.0|1078|mmu:14263|dimethylaniline monooxygenase (N-oxide forming) [EC:1.14.13.8]	gi|46559382|ref|NP_034362.2|	0	dimethylaniline monooxygenase [N-oxide-forming] 5 [Mus musculus]
mmu-let-7i-5p	NM_001161798	-27	0.846186	-25.18	170	GO:0044444,GO:0016646,GO:0000166,GO:0050662,GO:0000097,GO:0001101,GO:0033273,GO:0034097,GO:0006760,	K00297|1|0.0|1347|mmu:17769|methylenetetrahydrofolate reductase (NADPH) [EC:1.5.1.20]	gi|187466137|emb|CAQ51916.1|	0	5,10-methylenetetrahydrofolate reductase [Mus musculus]

mmu-let-7i-5p	NM_001161853	-25.8	0.557007	-25.06	154	GO:0070603,GO:0001739,GO:0005515,GO:0048731,GO:0019079,GO:0008283,GO:0001824,GO:0006259,GO:0048869,GO:0051704,GO:0043388,GO:0016568,	K11648|1|0.0|735|xla:100505439|SWI/SNF-related matrix-associated actin-dependent regulator of chromatin subfamily B member	gi|55956801|ref|NP_001007469.1|	0	SWI/SNF-related matrix-associated actin-dependent regulator of chromatin subfamily B member 1 isoform b [Homo sapiens]
mmu-let-7i-5p	NM_001163796	-25	0.709971	-25.16	165	GO:0023052,	K06742|1|4e-26|120|ecb:100066291|natural cytotoxicity triggering receptor 2	gi|148691658|gb|EDL23605.1|	4.89E-140	triggering receptor expressed on myeloid cells-like 4, isoform CRA_b [Mus musculus]

mmu-let-7i-5p	NM_001164569	-30.2	0.24637	-28.38	167	GO:0043227,GO:0031410,GO:0046914,GO:0003824,GO:0012501,GO:0015031,	K10871|1|5e-70|267|mcc:718665|RAD51-like protein 3!K10641|3|4e-10|68.6|tad:TRIADDRAFT_53391|E3 ubiquitin-protein ligase LRSAM1 [EC:6.3.2.19]!K16060|4|4e-09|65.1|spu:581540|baculoviral IAP repeat-containing protein 2/3!K10637|5|5e-	gi|257196123|ref|NP_001158041.1|	0	E3 ubiquitin-protein ligase rififylin isoform 3 [Mus musculus]

mmu-let-7i-5p	NM_001164570	-30.2	0.242441	-28.38	167	GO:0043227,GO:0031410,GO:0046914,GO:0003824,GO:0012501,GO:0015031,	K10871|1|5e-70|267|mcc:718665|RAD51-like protein 3!K10641|3|4e-10|68.6|tad:TRIADDRAFT_53391|E3 ubiquitin-protein ligase LRSAM1 [EC:6.3.2.19]!K16060|4|4e-09|65.1|spu:581540|baculoviral IAP repeat-containing protein 2/3!K10637|5|5e-	gi|257153330|ref|NP_001158042.1|	0	E3 ubiquitin-protein ligase rififylin isoform 4 [Mus musculus]

mmu-let-7i-5p	NM_001164571	-30.2	0.231008	-28.38	167	GO:0043227,GO:0031410,GO:0046914,GO:0003824,GO:0012501,GO:0015031,	K10871|1|1e-69|266|mcc:718665|RAD51-like protein 3!K10641|3|3e-10|68.6|tad:TRIADDRAFT_53391|E3 ubiquitin-protein ligase LRSAM1 [EC:6.3.2.19]!K16060|4|4e-09|65.1|spu:581540|baculoviral IAP repeat-containing protein 2/3!K10637|5|5e-	gi|148683726|gb|EDL15673.1|	0	ring finger and FYVE like domain containing protein, isoform CRA_c [Mus musculus]

mmu-let-7i-5p	NM_001164679	-28.5	0.457607	-25.59	168	GO:0016021,GO:0005216,	K13982|1|9e-81|303|dgr:Dgri_GH10918|probable ATP-dependent RNA helicase DDX4 [EC:3.6.4.13]!K03018|3|2e-19|99.4|smm:Smp_002890|DNA-directed RNA polymerase III subunit RPC1 [EC:2.7.7.6]	gi|50511029|dbj|BAD32500.1|	0	mKIAA1623 protein [Mus musculus]
mmu-let-7i-5p	NM_001164709	-25.1	0.888315	-25.31	163	GO:0031981,GO:0046914,GO:0016879,GO:0043161,GO:0043009,GO:0043066,	K10601|1|0.0|679|rno:361712|E3 ubiquitin-protein ligase synoviolin [EC:6.3.2.19]	gi|149062129|gb|EDM12552.1|	0	synovial apoptosis inhibitor 1, synoviolin, isoform CRA_b [Rattus norvegicus]

mmu-let-7i-5p	NM_001165965	-28.3	0.581708	-25.32	161	-	K06252|1|5e-12|75.1|spu:576745|tenascin!K05717|3|1e-07|60.5|xtr:595087|fibronectin 1!K05698|4|2e-06|56.6|mdo:100619665|protein tyrosine phosphatase, receptor type, J [EC:3.1.3.48]	gi|260166644|ref|NP_001159437.1|		0	fibronectin type III domain-containing protein 7 isoform 2 precursor [Mus musculus]
mmu-let-7i-5p	NM_001168521	-32.7	0.136039	-26.81	174	GO:0031312,GO:0015630,GO:0004872,GO:0005488,GO:0006915,GO:0006952,GO:0009746,GO:0023052,	-	gi|281183137|ref|NP_001161993.1|		0	sterile alpha and TIR motif-containing protein 1 isoform 1 [Mus musculus]

mmu-let-7i-5p	NM_001253880	-31.8	0.148773	-27.01	171	GO:0043231,GO:0046914,GO:0003676,	K11485|1|0.0|1004|mmu:17765|polycomb-like protein 2	gi|70887769|ref|NP_038855.2|	0	metal-response element-binding transcription factor 2 isoform 1 [Mus musculus]
mmu-let-7i-5p	NM_007496	-28.2	0.940374	-43.55	314	GO:0043231,GO:0043234,GO:0046914,GO:0005515,GO:0000981,GO:0043565,GO:0045661,GO:0006357,	K09378|1|0.0|5228|mmu:11906|AT-binding transcription factor 1	gi|148679469|gb|EDL11416.1|	0	AT motif binding factor 1 [Mus musculus]
mmu-let-7i-5p	NM_007625	-29.6	0.418707	-25.3	160	GO:0031981,GO:0043232,GO:0003723,GO:0003712,GO:0042393,GO:0006357,GO:0006325,GO:0043066,	K11452|1|0.0|900|mmu:12418|chromobox protein 4	gi|161353508|ref|NP_031651.2|	0	E3 SUMO-protein ligase CBX4 [Mus musculus]
mmu-let-7i-5p	NM_007732	-28.1	0.653501	-25.16	158	GO:0009925,GO:0030054,GO:0016021,GO:0007044,	K07603|1|0.0|1482|mmu:12821|collagen, type XVII, alpha 1	gi|226423931|ref|NP_031758.2|	0	collagen alpha-1(XVII) chain [Mus musculus]

mmu-let-7i-5p	NM_008587	-30.1	0.249738	-25.07	174	GO:0031513,GO:0016021,GO:0004871,GO:0004713,GO:0032559,GO:0006909,GO:0007243,GO:0007599,GO:0050953,GO:0006796,GO:0048608,GO:0007154,GO:0002695,GO:0000904,GO:0048232,GO:0006464,GO:0030098,GO:0046903,	K05117|1|0.0|1907|mmu:17289|c-mer proto-oncogene tyrosine kinase [EC:2.7.10.1]	gi|7106355|ref|NP_032613.1|	0	tyrosine-protein kinase Mer precursor [Mus musculus]
mmu-let-7i-5p	NM_008611	-29.8	0.189535	-28.18	171	GO:0031012,GO:0004175,GO:0046914,GO:0019538,GO:0032963,	K01402|1|0.0|975|mmu:17394|matrix metalloproteinase-8 (neutrophil collagenase) [EC:3.4.24.34]	gi|160333381|ref|NP_032637.3|	0	neutrophil collagenase precursor [Mus musculus]

mmu-let-7i-5p	NM_008767	-34.9	0.010353	-30.31	171	GO:0016021,GO:0044459,GO:0012505,GO:0044424,GO:0015238,GO:0008509,GO:0019899,GO:0015291,GO:0015893,GO:0003008,GO:0006812,	K08214|1|2e-168|592|mmu:18400|MFS transporter, OCT family, solute carrier family 22 (organic cation transporter), member 18	gi|112363086|ref|NP_001036225.1|	1.38E-167	solute carrier family 22 member 18 [Mus musculus]
mmu-let-7i-5p	NM_008824	-31.9	0.049415	-28.66	176	GO:0043234,GO:0044444,GO:0070061,GO:0008443,GO:0019899,GO:0019203,GO:0032559,GO:0006007,GO:0035556,GO:0033131,GO:0009887,GO:0043434,GO:0031960,GO:0015980,GO:0006000,GO:00069	K01103|1|0.0|910|mmu:18639|6-phosphofructo-2-kinase / fructose-2,6-bisphosphatase [EC:2.7.1.105 3.1.3.46]	gi|7434494|pir||S77704	0	6-phosphofructo-2-kinase (EC 2.7.1.105) / fructose-2, 6-bisphosphate 2-phosphatase (EC 3.1.3.46) clone 5c, skeletal muscle - rat

mmu-let-7i-5p	NM_008906	-28.1	0.50664	-43.58	304	GO:0000323,GO:0031981,GO:0005488,GO:0004180,GO:0030234,GO:0019538,GO:0015031,	K13289|1|0.0|979|mmu:19025|cathepsin A (carboxypeptidase C) [EC:3.4.16.5]	gi|84042525|ref|NP_032932.2|	0	lysosomal protective protein isoform a precursor [Mus musculus]
mmu-let-7i-5p	NM_009662	-27.4	0.514118	-40.68	312	GO:0043005,GO:0034399,GO:0044444,GO:0005635,GO:0005886,GO:0046914,GO:0016702,GO:0000038,GO:0007600,GO:0002538,GO:0031667,GO:0009058,GO:0019229,GO:00	K00461|1|0.0|1356|mmu:11689|arachidonate 5-lipoxygenase [EC:1.13.11.34]	gi|62526037|ref|NP_033792.1|	0	arachidonate 5-lipoxygenase [Mus musculus]
mmu-let-7i-5p	NM_010232	-26.7	0.838163	-46.02	311	GO:0005789,GO:0031224,GO:0016709,GO:0050662,GO:0000166,GO:0008152,	K00485|1|0.0|1078|mmu:14263|dimethylaniline monooxygenase (N-oxide forming) [EC:1.14.13.8]	gi|46559382|ref|NP_034362.2|	0	dimethylaniline monooxygenase [N-oxide-forming] 5 [Mus musculus]

mmu-let-7i-5p	NM_010264	-25	0.987023	-41.28	319	GO:0043234,GO:0031981,GO:0046914,GO:0042802,GO:0001071,GO:0003677,GO:0004879,GO:0048869,GO:0009755,GO:00482	K08561|1|0.0|892|ssc:100038028|germ cell nuclear factor	gi|6753956|ref|NP_034394.1|	0	nuclear receptor subfamily 6 group A member 1 isoform 1 [Mus musculus]
mmu-let-7i-5p	NM_010840	-27	0.842694	-25.18	170	GO:0044444,GO:0016646,GO:0000166,GO:0050662,GO:0000097,GO:0001101,GO:0033273,GO:0034097,GO:0006760,	K00297|1|0.0|1272|mmu:17769|methylenetetrahydrofolate reductase (NADPH) [EC:1.5.1.20]	gi|187466137|emb|CAQ51916.1|	0	5,10-methylenetetrahydrofolate reductase [Mus musculus]

mmu-let-7i-5p	NM_011158	-34.2	0.043917	-28.7	174	GO:0044425,GO:0043234,GO:0019866,GO:0044444,GO:0016772,GO:0016208,GO:0051018,GO:0019887,GO:0009653,GO:0015980,GO:0007599,GO:0007611,GO:0033762,GO:0032147,GO:0010863,GO:0007169,GO:0022402,GO:0030073,GO:0032787,GO:00	K04739|1|0.0|775|mmu:19088|cAMP-dependent protein kinase regulator	gi|148704952|gb|EDL36899.1|	0	protein kinase, cAMP dependent regulatory, type II beta, isoform CRA_b [Mus musculus]

mmu-let-7i-5p	NM_011418	-25.8	0.563715	-25.06	154	GO:0070603,GO:0001739,GO:0005515,GO:0048731,GO:0019079,GO:0008283,GO:0001824,GO:0006259,GO:0048869,GO:0051704,GO:0043388,GO:0016568,	K11648|1|0.0|754|ssc:100154735|SWI/SNF-related matrix-associated actin-dependent regulator of chromatin subfamily B member 1	gi|6755578|ref|NP_035548.1|	0	SWI/SNF-related matrix-associated actin-dependent regulator of chromatin subfamily B member 1 isoform 1 [Mus musculus]
mmu-let-7i-5p	NM_012031	-26.3	0.745023	-44.56	311	GO:0044424,GO:0032561,GO:0003824,GO:0009566,	K09553|1|2e-28|129|hmg:100203295|stress-induced-phosphoprotein 1	gi|148676879|gb|EDL08826.1|	0	sperm associated antigen 1, isoform CRA_b [Mus musculus]

mmu-let-7i-5p	NM_013477	-30.8	0.074577	-28.41	161	GO:0005774,GO:0033176,GO:0005768,GO:0033267,GO:0044459,GO:0030136,GO:0005515,GO:0015077,GO:0015662,GO:0007417,GO:0015988,GO:0015031,GO:0007169,GO:00	K02146|1|0.0|712|rno:291969|V-type H+-transporting ATPase subunit AC39 [EC:3.6.3.14]	gi|31981304|ref|NP_038505.2|	0	V-type proton ATPase subunit d 1 [Mus musculus]
mmu-let-7i-5p	NM_013539	-27.5	0.215186	-25.14	162	GO:0044424,GO:0005488,GO:0007165,	K10344|1|1e-154|546|mmu:14794|SPRY domain-containing SOCS box protein 2	gi|7305113|ref|NP_038567.1|	8.01E-154	SPRY domain-containing SOCS box protein 2 [Mus musculus]
mmu-let-7i-5p	NM_015755	-25.2	0.963504	-25.95	162	GO:0004672,GO:0032559,GO:0006464,GO:0032501,GO:0006796,GO:0023052,	K08797|1|0.0|1317|mmu:26559|hormonally upregulated Neu-associated kinase [EC:2.7.11.1]	gi|7657216|ref|NP_056570.1|	0	hormonally up-regulated neu tumor-associated kinase [Mus musculus]

mmu-let-7i-5p	NM_019742	-26.2	0.494693	-25.66	152	GO:0005488,GO:0007154,	-	gi|157817851|ref|NP_001102767.1|	4.65E-47	tumor suppressor candidate 2 [Rattus norvegicus]
mmu-let-7i-5p	NM_019757	-26.5	0.569339	-25.22	166	GO:0031981,GO:0000152,GO:0044444,GO:0005488,GO:0043161,GO:0051320,GO:0006259,GO:0000077,GO:0051439,GO:0051443,GO:0007126,GO:0030154,GO:0000087,GO:00	K03364|1|0.0|1002|mmu:56371|cell division cycle 20-like protein 1, cofactor of APC complex	gi|9789959|ref|NP_062731.1|	0	fizzy-related protein homolog [Mus musculus]
mmu-let-7i-5p	NM_019965	-28.5	0.226704	-25.72	161	GO:0031224,GO:0043231,GO:0005515,GO:0044267,	K09518|1|2e-180|632|ecb:100063614|DnaJ homolog subfamily B member	gi|344274603|ref|XP_003409104.1|	5.64E-179	PREDICTED: dnaJ homolog subfamily B member 12-like [Loxodonta africana]

mmu-let-7i-5p	NM_020575	-25.4	0.813786	-25.52	159	GO:0003824,GO:0046914,	K10662|1|0.0|1066|mmu:57438|E3 ubiquitin-protein ligase MARCH7 [EC:6.3.2.19]	gi|10181210|ref|NP_065600.1|	0	E3 ubiquitin-protein ligase MARCH7 [Mus musculus]
mmu-let-7i-5p	NM_020614	-29.9	0.145675	-26.58	163	GO:0005667,GO:0003676,GO:0006351,	K15213|1|0.0|1081|mmu:21340|TATA box-binding protein-associated factor RNA polymerase I subunit B	gi|256773270|ref|NP_065639.2|	0	TATA box-binding protein-associated factor RNA polymerase I subunit B [Mus musculus]
mmu-let-7i-5p	NM_025902	-25	0.881249	-40.45	287	GO:0031224,GO:0032991,GO:0031966,GO:0042802,GO:0043169,GO:0051536,GO:0007275,GO:0006914,	-	gi|21728370|ref|NP_080178.1|	2.71E-59	CDGSH iron-sulfur domain-containing protein 2 [Mus musculus]

mmu-let-7i-5p	NM_026097	-30.2	0.238422	-28.38	167	GO:0043227,GO:0031410,GO:0046914,GO:0003824,GO:0012501,GO:0015031,	K10871|1|1e-69|266|mcc:718665|RAD51-like protein 3!K10641|3|4e-10|68.6|tad:TRIADDRAFT_53391|E3 ubiquitin-protein ligase LRSAM1 [EC:6.3.2.19]!K16060|4|4e-09|65.1|spu:581540|baculoviral IAP repeat-containing protein 2/3!K10637|5|5e-	gi|16975488|ref|NP_080373.1|	0	E3 ubiquitin-protein ligase rififylin isoform 2 [Mus musculus]
mmu-let-7i-5p	NM_026240	-29.6	0.21957	-26.02	176	-	-	gi|12858310|dbj|BAB31270.1|	0	unnamed protein product [Mus musculus]

mmu-let-7i-5p	NM_026298	-25.3	0.967349	-51.05	324	GO:0005815,GO:0043231,GO:0005515,GO:0003002,GO:0006357,GO:0010927,GO:0001838,GO:0051604,GO:0009855,GO:00071	-	gi|148705414|gb|EDL37361.1|	0	intraflagellar transport 172 homolog (Chlamydomonas), isoform CRA_a [Mus musculus]
mmu-let-7i-5p	NM_027917	-26.4	0.733119	-25.44	170	GO:0016459,GO:0015630,GO:0003779,GO:0007015,GO:0032989,	-	gi|125628648|ref|NP_082193.2|	0	protein Shroom1 [Mus musculus]
mmu-let-7i-5p	NM_028544	-30.2	0.213139	-29.3	171	GO:0005794,	K05702|1|6e-07|57.8|nve:NEMVE_v1g174672|afadin	gi|61676215|ref|NP_082820.1|	0	ras-interacting protein 1 [Mus musculus]
mmu-let-7i-5p	NM_028769	-27.9	0.515448	-25.31	163	GO:0031981,GO:0046914,GO:0016879,GO:0043161,GO:0043009,GO:0043066,	K10601|1|0.0|679|rno:361712|E3 ubiquitin-protein ligase synoviolin [EC:6.3.2.19]	gi|149062129|gb|EDM12552.1|	0	synovial apoptosis inhibitor 1, synoviolin, isoform CRA_b [Rattus norvegicus]

mmu-let-7i-5p	NM_029554	-30.9	0.08615	-27.48	165	-	-	gi|148705758|gb|EDL37705.1|	0	RIKEN cDNA 0610040J01, isoform CRA_c [Mus musculus]
mmu-let-7i-5p	NM_029965	-31	0.202355	-26.37	168	GO:0031224,GO:0046914,	K15707|1|1e-137|491|mmu:77733|RING finger protein 170	gi|148692281|gb|EDL24228.1|	1.05E-136	mCG1050561 [Mus musculus]
mmu-let-7i-5p	NM_030219	-28.9	0.271255	-27.14	162	GO:0044464,GO:0046914,	K12018|1|0.0|1503|mmu:78911|tripartite motif-containing protein 42	gi|116174784|ref|NP_084495.2|	0	tripartite motif-containing protein 42 [Mus musculus]
mmu-let-7i-5p	NM_030262	-25.8	0.678335	-25.96	154	GO:0043231,GO:0008417,GO:0019318,	K03691|1|0.0|867|mmu:80294|peptide-O-fucosyltransferase [EC:2.4.1.221]	gi|19525731|ref|NP_084538.2|	0	GDP-fucose protein O-fucosyltransferase 2 precursor [Mus musculus]

mmu-let-7i-5p	NM_052976	-29.9	0.295665	-27.55	162	GO:0030425,GO:0005856,GO:0033267,GO:0044459,GO:0044444,GO:0030674,GO:0008092,GO:0019904,GO:0035254,GO:0005099,GO:0048731,GO:0007010,GO:0030035,GO:0006929,GO:0006897,GO:0006935,GO:0050804,GO:0032319,	K13736|1|7e-169|595|gga:422471|Rho GTPase-activating protein 10	gi|148682255|gb|EDL14202.1|	0	oligophrenin 1, isoform CRA_b [Mus musculus]
mmu-let-7i-5p	NM_053214	-27.6	0.600269	-25.33	165	GO:0016459,GO:0005884,GO:0008092,GO:0017111,GO:0032559,GO:0016477,GO:0002444,GO:0002283,GO:0030036,GO:0007155,GO:0031347,	K10356|1|0.0|2111|mmu:17916|myosin I	gi|255069756|ref|NP_444444.2|	0	unconventional myosin-If [Mus musculus]

mmu-let-7i-5p	NM_054044	-28.4	0.613671	-44.77	313	GO:0031224,GO:0004888,GO:0001525,GO:0016477,GO:0032101,GO:0022603,GO:0007399,GO:0007186,	K08461|1|0.0|2430|mmu:78560|G protein-coupled receptor 124	gi|300934759|ref|NP_473385.2|	0	G-protein coupled receptor 124 precursor [Mus musculus]
mmu-let-7i-5p	NM_133355	-28.6	0.416538	-44.05	300	GO:0030425,GO:0044459,GO:0008092,GO:0007186,GO:0007010,GO:0048167,	K04512|1|3e-41|168|tca:658864|dishevelled associated activator of morphogenesis	gi|19111166|ref|NP_579933.1|	0	delphilin isoform 2 [Mus musculus]
mmu-let-7i-5p	NM_133698	-28.6	0.808138	-373.91	2465	-	K10384|1|2e-49|200|ecb:100060541|filaggrin	gi|351702692|gb|EHB05611.1|	0	Hornerin [Heterocephalus glaber]
mmu-let-7i-5p	NM_133821	-27.7	0.755784	-44.64	332	GO:0043231,GO:0044444,GO:0043169,GO:0004721,GO:0048017,GO:0012501,GO:0048511,GO:0007169,	K01090|1|0.0|2610|mmu:98432|protein phosphatase [EC:3.1.3.16]	gi|257467468|ref|NP_598582.3|	0	PH domain leucine-rich repeat-containing protein phosphatase 1 [Mus musculus]

mmu-let-7i-5p	NM_139061	-26.7	0.809765	-46.63	315	GO:0045104,GO:0001894,GO:0016197,GO:0050879,	-	gi|226530401|ref|NP_620692.3|	0	vacuolar protein sorting-associated protein 54 [Mus musculus]
mmu-let-7i-5p	NM_139232	-30.9	0.165113	-27.71	171	GO:0043232,GO:0031252,GO:0042995,GO:0044444,GO:0005088,GO:0046914,GO:0003779,GO:0065008,GO:0006917,GO:0000187,GO:0007266,GO:0030031,	K05723|1|0.0|1549|mmu:224014|FYVE, RhoGEF and PH domain containing 4	gi|148665004|gb|EDK97420.1|	0	FYVE, RhoGEF and PH domain containing 4, isoform CRA_a [Mus musculus]
mmu-let-7i-5p	NM_139233	-30.9	0.106532	-27.71	171	GO:0043232,GO:0031252,GO:0042995,GO:0044444,GO:0005088,GO:0046914,GO:0003779,GO:0065008,GO:0006917,GO:0000187,GO:0007266,GO:0030031,	K05723|1|0.0|1181|mmu:224014|FYVE, RhoGEF and PH domain containing 4	gi|148665004|gb|EDK97420.1|	0	FYVE, RhoGEF and PH domain containing 4, isoform CRA_a [Mus musculus]

mmu-let-7i-5p	NM_139234	-30.9	0.117689	-27.71	171	GO:0043232,GO:0031252,GO:0042995,GO:0044444,GO:0005088,GO:0046914,GO:0003779,GO:0065008,GO:0006917,GO:0000187,GO:0007266,GO:0030031,	K05723|1|0.0|1004|mmu:224014|FYVE, RhoGEF and PH domain containing 4	gi|148665004|gb|EDK97420.1|	0	FYVE, RhoGEF and PH domain containing 4, isoform CRA_a [Mus musculus]
mmu-let-7i-5p	NM_145140	-25.6	0.951926	-44.59	322	GO:0031224,GO:0017111,GO:0015405,GO:0032559,GO:0006810,GO:0009207,	K05674|1|0.0|2328|mmu:224814|ATP-binding cassette, subfamily C (CFTR/MRP), member 10	gi|21553091|ref|NP_660122.1|	0	multidrug resistance-associated protein 7 isoform mrp7A [Mus musculus]
mmu-let-7i-5p	NM_145390	-30.7	0.271546	-25.26	145	GO:0043231,GO:0005048,GO:0022892,GO:0015031,	K14293|1|4e-14|82.0|acs:100557760|importin subunit beta-1	gi|170932528|ref|NP_663365.3|	0	transportin-2 [Mus musculus]

mmu-let-7i-5p	NM_145833	-25.1	0.913934	-46.94	318	GO:0031981,GO:0030529,GO:0046914,GO:0005515,GO:0003723,GO:0006351,GO:0045597,GO:0006412,GO:0003006,GO:0014014,GO:0010586,GO:0035196,	K09277|1|2e-10|69.3|xtr:100170415|Y-box-binding protein 2!K09276|3|3e-09|65.5|ecb:100067336|Y-box-binding protein 1	gi|297677568|ref|XP_002816670.1|	8.09E-106	PREDICTED: protein lin-28 homolog A-like [Pongo abelii]
mmu-let-7i-5p	NM_146155	-25.7	0.968209	-63.76	475	-	K02350|1|2e-08|63.5|tgu:100217592|DNA polymerase zeta subunit [EC:2.7.7.7]	gi|148698133|gb|EDL30080.1|	0	AT hook, DNA binding motif, containing 1, isoform CRA_b [Mus musculus]
mmu-let-7i-5p	NM_153392	-27.6	0.414189	-63.38	463	-	-	gi|148698762|gb|EDL30709.1|	0	RIKEN cDNA 4922503N01, isoform CRA_d [Mus musculus]
mmu-let-7i-5p	NM_153584	-33.8	0.068758	-29.4	166	-	-	gi|164452925|ref|NP_705812.2|	0	protein FAM214A isoform 2 [Mus musculus]

mmu-let-7i-5p	NM_170680	-25.6	0.934553	-44.59	322	GO:0031224,GO:0017111,GO:0015405,GO:0032559,GO:0006810,GO:0009207,	K05674|1|0.0|2305|mmu:224814|ATP-binding cassette, subfamily C (CFTR/MRP), member 10	gi|24850123|ref|NP_733780.1|	0	multidrug resistance-associated protein 7 isoform mrp7B [Mus musculus]
mmu-let-7i-5p	NM_172795	-32.7	0.132572	-26.81	174	GO:0031312,GO:0015630,GO:0004872,GO:0005488,GO:0006915,GO:0006952,GO:0009746,GO:0023052,	-	gi|50510513|dbj|BAD32242.1|	0	mKIAA0524 protein [Mus musculus]
mmu-let-7i-5p	NM_172800	-27.8	0.766655	-50.07	310	GO:0031224,GO:0009987,	K06252|1|2e-97|359|bfo:BRAFLDRAFT_124749|tenascin	gi|40254254|ref|NP_766388.2|	0	protein sidekick-2 precursor [Mus musculus]

mmu-let-7i-5p	NM_174876	-31.6	0.277346	-27.76	169	GO:0031012,GO:0031224,GO:0004871,GO:0005539,GO:0005198,GO:0050953,GO:0023052,	K08116|1|2e-08|63.5|gga:395466|chondroitin sulfate proteoglycan 5 (neurogly	gi|148665738|gb|EDK98154.1|	0	interphotoreceptor matrix proteoglycan 2, isoform CRA_a [Mus musculus]
mmu-let-7i-5p	NM_175520	-33.1	0.068077	-31.74	178	GO:0031224,GO:0001608,GO:0043627,GO:0007166,	K08401|1|0.0|647|mmu:243270|G protein-coupled receptor 81	gi|259490314|ref|NP_780729.2|	0	hydroxycarboxylic acid receptor 1 [Mus musculus]
mmu-let-7i-5p	NM_177028	-28.6	0.410145	-28.79	163	GO:0016746,	K00680|1|2e-21|105|dme:Dmel_CG33337|[EC:2.3.1.-	gi|28892903|ref|NP_796002.1|	0	acyltransferase 3 precursor [Mus musculus]
mmu-let-7i-5p	NM_177091	-28.3	0.601103	-25.32	161	-	K06252|1|2e-13|80.1|spu:576745|tenascin!K05717|5|2e-08|63.2|acs:100563512|fibronectin 1	gi|260166640|ref|NP_796065.2|	0	fibronectin type III domain-containing protein 7 isoform 1 precursor [Mus musculus]

mmu-let-7i-5p	NM_177566	-25.8	0.887222	-50.39	353	GO:0043005,GO:0008047,GO:0005488,GO:0005088,GO:0048523,GO:0043087,GO:0007266,	K07525|1|2e-64|248|xtr:100487663|neuronal guanine nucleotide exchange	gi|60279700|ref|NP_808234.2|	0	rho guanine nucleotide exchange factor 15 [Mus musculus]
mmu-let-7i-5p	NM_177656	-30.7	0.082108	-27.22	161	-	K07847|1|1e-23|112|mgp:100542192|Rad and Gem related GTP binding protein 1!K04514|2|3e-06|54.3|tca:663533|Rho-associated, coiled-coil containing protein kinase [EC:2.7.11.1]!K11547|3|6e-06|53.1|tgu:100226934|kinetochore protein	gi|29244072|ref|NP_808324.1|	0	uncharacterized protein LOC228778 [Mus musculus]

mmu-let-7i-5p	NM_178641	-27.5	0.695827	-26.62	167	GO:0016788,	K01099|1|7e-46|187|dre:337236|phosphatidylinositol-bisphosphatase [EC:3.1.3.36]	gi|37360162|dbj|BAC98059.1|	0	mKIAA0966 protein [Mus musculus]
mmu-let-7i-5p	NM_183106	-32.5	0.12972	-73.4	498	-	-	gi|124430551|ref|NP_898929.2|	0	tetratricopeptide repeat domain 17 [Mus musculus]
mmu-let-7i-5p	NM_194345	-30.3	0.263152	-25.03	160	-	-	gi|257196165|ref|NP_919326.1|	0	protein FAM160B2 [Mus musculus]
mmu-let-7i-5p	NM_198622	-25.9	0.389857	-25.12	154	GO:0000785,GO:0043231,GO:0003676,GO:0034728,	K11275|1|4e-57|222|mmu:243529|histone H1/5	gi|38348566|ref|NP_941024.1|	2.48E-56	H1 histone family, member X [Mus musculus]
mmu-let-7i-5p	NM_199252	-26.5	0.635672	-25.68	163	GO:0031224,	-	gi|40445395|ref|NP_954860.1|	0	protein unc-93 homolog A [Mus musculus]

mmu-let-7i-5p	NM_199322	-28.3	0.640499	-46.27	313	GO:0005488,GO:0016279,GO:0016571,	K11427|1|0.0|2615|mmu:208266|histone-lysine N-methyltransferase, H3 lysine-79 specific [EC:2.1.1.4	gi|148699559|gb|EDL31506.1|	0	DOT1-like, histone H3 methyltransferase (S. cerevisiae), isoform CRA_c [Mus musculus]
mmu-let-7i-5p	NR_027809	-27	0.78869	-25.18	170	GO:0044444,GO:0016646,GO:0000166,GO:0050662,GO:0000097,GO:0001101,GO:0033273,GO:0034097,GO:0006760,	K00297|1|0.0|1347|mmu:17769|methylenetetrahydrofolate reductase (NADPH) [EC:1.5.1.20]	gi|187466137|emb|CAQ51916.1|	0	5,10-methylenetetrahydrofolate reductase [Mus musculus]

mmu-let-7i-5p	NR_027838	-29.5	0.466735	-48.12	316	GO:0032991,GO:0016021,GO:0005789,GO:0001664,GO:0019829,GO:0046872,GO:0032559,GO:0009888,GO:0007599,GO:0008016,GO:0006816,GO:0006936,GO:0009207,GO:0007165,GO:0001775,GO:0009206,GO:0009725,GO:0006875,	K05853|1|0.0|2035|mmu:11938|Ca2+ transporting ATPase, sarcoplasmic/endoplasmic reticulum [EC:3.6.3.8]	gi|60360584|dbj|BAD90532.1|	0	mKIAA4195 protein [Mus musculus]
mmu-let-7i-5p	NR_030733	-34.4	0.007387	-27.41	162	-	-	gi|148704546|gb|EDL36493.1|	4.86E-91	mCG146312 [Mus musculus]
mmu-let-7i-5p	NR_030734	-34.4	0.00733	-27.41	162	-	-	gi|148704546|gb|EDL36493.1|	4.82E-91	mCG146312 [Mus musculus]

mmu-let-7i-5p	NR_001461	-20.4	1	-93.13	643	-	K11849|1|6e-55|213|acs:100555596|ubiquitin carboxyl-terminal hydrolase 25/28 [EC:3.1.2.15]!K06478|4|4e-43|182|acs:100563672|protein tyrosine phosphatase, receptor type, C [EC:3.1.3.4	gi|209553980|gb|AAA39398.2|	0	ORF2 [Mus musculus domesticus]

mmu-let-7i-5p	NM_172882	-22.4	1	-70.7	496	GO:0005773,GO:0012505,GO:0044425,GO:0035091,GO:0046914,GO:0035250,	K07295|1|3e-49|200|dpe:Dper_GL19043|nuclear receptor subfamily 2 group A!K01104|2|1e-12|79.0|spu:585727|protein-tyrosine phosphatase [EC:3.1.3.48]!K12182|3|3e-12|77.4|cqu:CpipJ_CPIJ001538|hepatocyte growth factor-regulated tyrosine kinase	gi|39930599|ref|NP_766470.2|	0	WD repeat and FYVE domain-containing protein 3 [Mus musculus]
mmu-let-7j	NM_001025371	-26.7	0.62511	-42.52	327	GO:0031224,GO:0006644,	-	gi|148696114|gb|EDL28061.1|	0	hypothetical LOC574418, isoform CRA_b [Mus musculus]

mmu-let-7j	NM_001034869	-27.4	0.3839	-27.28	160	-	K09381|1|3e-06|54.3|dya:Dyak_GE12435|paired box protein 3/7!K09371|5|7e-06|53.1|hsa:4010|LIM homeobox transcription factor 1	gi|85702209|ref|NP_001030041.1|	4.71E-149	uncharacterized protein LOC245263 [Mus musculus]
mmu-let-7j	NM_001146174	-33.1	0.066994	-26.25	172	GO:0000776,GO:0005819,GO:0005635,GO:0044444,GO:0005099,GO:0005488,GO:0007067,GO:0032316,	K14319|1|0.0|972|mmu:19387|Ran GTPase-activating protein 1	gi|37360568|dbj|BAC98262.1|	0	mKIAA1835 protein [Mus musculus]

mmu-let-7j	NM_008439	-33.2	0.016498	-29.39	177	GO:0044444,GO:0016773,GO:0032559,GO:0006000,GO:0043434,GO:0016310,GO:0009746,GO:0034285,GO:0010038,	K00846|1|8e-172|603|mmu:16548|ketohexokinase [EC:2.7.1.3]	gi|31982229|ref|NP_032465.2|	5.09E-171	ketohexokinase [Mus musculus]
mmu-let-7j	NM_009185	-29.2	0.515095	-26.94	171	GO:0044444,GO:0014020,GO:0006915,GO:0009880,GO:0003143,GO:0007420,GO:0040007,GO:0009855,GO:0048568,GO:00071	-	gi|164698492|ref|NP_033211.2|	0	SCL-interrupting locus protein homolog [Mus musculus]
mmu-let-7j	NM_009267	-28.4	0.074496	-25.7	173	-	-	gi|254692869|ref|NP_033293.2|	7.46E-62	16.5 kDa submandibular gland glycoprotein precursor [Mus musculus]

mmu-let-7j	NM_011241	-33.1	0.068356	-26.25	172	GO:0000776,GO:0005819,GO:0005635,GO:0044444,GO:0005099,GO:0005488,GO:0007067,GO:0032316,	K14319|1|0.0|972|mmu:19387|Ran GTPase-activating protein 1	gi|148672614|gb|EDL04561.1|	0	RAN GTPase activating protein 1, isoform CRA_b [Mus musculus]
mmu-let-7j	NM_011981	-27.3	0.684446	-45.96	334	GO:0043231,GO:0046914,GO:0003676,GO:0006351,GO:0032501,	K09228|1|0.0|722|mcc:712978|KRAB domain-containing zinc finger protein	gi|31544063|ref|NP_036111.2|	0	zinc finger protein 260 [Mus musculus]
mmu-let-7j	NM_013886	-29	0.579628	-26.03	180	GO:0043231,GO:0005488,	K00020|1|3e-08|63.2|aag:AaeL_AAEL006684|3-hydroxyisobutyrate dehydrogenase [EC:1.1.1.31]	gi|26337455|dbj|BAC32413.1|	2.02E-83	unnamed protein product [Mus musculus]
mmu-let-7j	NM_021477	-30.7	0.314882	-26.86	166	GO:0044431,GO:0003676,GO:0005515,GO:0050657,GO:0006396,	K14946|1|0.0|654|mmu:268859|RNA binding protein fox-1	gi|66792808|ref|NP_067452.2|	0	RNA binding protein fox-1 homolog 1 isoform gamma [Mus musculus]

mmu-let-7j	NM_028771	-30.7	0.181686	-25.96	167	-	-	gi|21311899|ref|NP_083047.1|	8.52E-141	coiled-coil domain-containing protein 97 [Mus musculus]
mmu-let-7j	NM_029294	-27.9	0.295775	-26.04	168	GO:0032991,GO:0044444,GO:0016778,GO:0046872,GO:0042802,GO:0032561,GO:0030246,GO:0032559,GO:0006220,GO:0006796,GO:0009887,GO:0046390,GO:0009168,GO:00	K00948|1|6e-179|627|mmu:75456|ribose-phosphate pyrophosphokinase [EC:2.7.6.1]	gi|30794182|ref|NP_083570.1|	4.38E-178	phosphoribosyl pyrophosphate synthetase 1-like 1 [Mus musculus]
mmu-let-7j	NM_053162	-28.3	0.080256	-25.62	164	GO:0000315,GO:0043231,GO:0005198,GO:0010467,	K11792|1|5e-22|104|ptr:748388|DET1- and DDB1-associated protein 1	gi|16716449|ref|NP_444392.1|	8.37E-48	39S ribosomal protein L34, mitochondrial [Mus musculus]

mmu-let-7j	NM_172301	-26.7	0.583842	-25.83	161	GO:0005819,GO:0000778,GO:0044444,GO:0004672,GO:0019900,GO:0008284,GO:0055013,GO:0000070,GO:0003006,GO:0051495,GO:0043009,GO:0010041,GO:0048232,GO:0014070,GO:0033044,GO:0001666,GO:0001934,GO:0009605,GO:0043161,GO:0051439,GO:0065003,GO:00705	K05868|1|0.0|748|mmu:268697|cyclin B	gi|51762951|ref|XP_485921.1|	0	PREDICTED: G2/mitotic-specific cyclin-B1-like isoform 1 [Mus musculus]

mmu-let-7j	NM_172855	-26.6	0.730018	-25.23	176	GO:0012505,GO:0043231,GO:0031224,GO:0030246,GO:0008376,GO:0006023,	K00710|1|0.0|1862|mmu:241391|polypeptide N-acetylgalactosaminyltransferase [EC:2.4.1.41]	gi|158749624|ref|NP_766443.2|	0	polypeptide N-acetylgalactosaminyltransferase 5 [Mus musculus]
mmu-let-7j	NM_001081954	-24.8	0.83219	-71.86	452	GO:0003676,	K09360|1|5e-08|60.5|dvi:Dvir_GJ18855|BarH-like	gi|126432556|ref|NP_001075423.1|	0	double homeobox [Mus musculus]
mmu-let-7j	NM_029928	-24.2	0.999983	-41.85	330	GO:0016021,GO:0004725,GO:0005488,GO:0004871,GO:0048646,GO:0006470,GO:0023052,	K05694|1|0.0|3919|mmu:19263|protein tyrosine phosphatase, receptor type, B [EC:3.1.3.48]	gi|258613900|ref|NP_084204.2|	0	receptor-type tyrosine-protein phosphatase beta precursor [Mus musculus]

mmu-let-7j	NM_183355	-21.3	1	-47.49	326	GO:0031981,GO:0043234,GO:0001071,GO:0046983,GO:0003677,GO:0001501,GO:0030097,GO:0045596,GO:0008202,GO:0006357,GO:0003006,GO:0008283,GO:0035108,GO:0001503,GO:0043392,GO:0003002,GO:0048754,GO:0009887,GO:00352	K09355|1|0.0|738|ecb:100058426|pre-B-cell leukemia transcription factor 1	gi|149755772|ref|XP_001493290.1|	0	PREDICTED: pre-B-cell leukemia transcription factor 1 isoform 1 [Equus caballus]
mmu-let-7j	NM_001163026	-24.1	0.999707	-42.4	306	GO:0005515,GO:0023052,	K09533|1|0.0|4270|mmu:235567|DnaJ homolog subfamily C member 13	gi|247494234|ref|NP_001156498.1|	0	DnaJ (Hsp40) homolog, subfamily C, member 13 [Mus musculus]

mmu-let-7j	NM_178746	-23.1	0.999999	-22.68	182	GO:0031224,GO:0030001,GO:0015837,	K14995|1|0.0|1042|mmu:268706|solute carrier family 38 (sodium-coupled neutral amino acid transporter), member 9	gi|30520193|ref|NP_848861.1|	0	putative sodium-coupled neutral amino acid transporter 9 [Mus musculus]
mmu-let-7j	NM_145575	-28.7	0.486709	-24.75	181	GO:0030425,GO:0044297,GO:0030864,GO:0005924,GO:0008092,GO:0043393,GO:0007015,GO:0006937,GO:0022414,	K12327|1|3e-94|347|mmu:109624|caldesmon	gi|148681738|gb|EDL13685.1|	2.43E-93	caldesmon 1, isoform CRA_a [Mus musculus]
mmu-let-7j	NM_027619	-24.7	0.999462	-24.42	180	GO:0003676,	-	gi|26334203|dbj|BAC30819.1|	0	unnamed protein product [Mus musculus]
mmu-let-7j	NM_175133	-20.3	1	-28.94	178	-	-	gi|355563527|gb|EHH20089.1|	5.06E-146	hypothetical protein EGK_02875, partial [Macaca mulatta]

mmu-let-7j	NM_001083897	-23.9	0.955593	-23.1	177	GO:0016021,GO:0005488,GO:0007154,GO:0007167,	K06770|1|7e-126|452|rno:360871|myelin protein zero	gi|140969905|ref|NP_001077366.1|	6.96E-127	myelin protein zero-like protein 1 isoform a precursor [Mus musculus]
mmu-let-7j	NM_173771	-24.9	0.772316	-20.36	176	GO:0044464,GO:0016757,	K13748|1|7e-49|196|mgp:100550363|alpha-1,3-mannosylglycoprotein beta-1,4-N-acetylglucosaminyltransferase C [EC:2.4.1.201 2.4.1.145]	gi|237757320|ref|NP_776132.2|	0	uncharacterized protein LOC240755 [Mus musculus]
mmu-let-7j	NM_001081477	-20.9	1	-23.86	175	-	K11798|1|0.0|3097|mmu:382236|bromodomain and WD repeat domain containing protein 1/3	gi|125490358|ref|NP_001074946.1|	0	bromodomain and WD repeat-containing protein 3 [Mus musculus]

mmu-let-7j	NM_177806	-24.7	0.975357	-21.43	174	GO:0031981,GO:0006396,	K13217|1|0.0|1284|mmu:328110|pre-mRNA-processing factor 39	gi|156546892|ref|NP_808474.2|	0	pre-mRNA-processing factor 39 [Mus musculus]
mmu-let-7j	NR_045497	-24.3	0.736059	-21.67	173	-	-	gi|148703576|gb|EDL35523.1|	4.68E-40	mCG1042816 [Mus musculus]
mmu-let-7j	NR_030769	-25.8	0.914133	-20.66	172	GO:0043231,GO:0005515,GO:0001071,GO:0003677,GO:0007166,GO:0045596,GO:0048664,GO:0021529,GO:0006357,GO:0003312,GO:0003310,GO:0001708,GO:0045687,GO:0003309,GO:0021782,	K08029|1|1e-46|189|ssc:100626520|homeobox protein Nkx-2.2	gi|148696575|gb|EDL28522.1|	2.31E-73	mCG146261 [Mus musculus]
mmu-let-7j	NM_173185	-22	1	-24.51	171	GO:0044424,GO:0004672,GO:0032559,GO:0006464,GO:0007166,	K08958|1|0.0|898|mmu:214897|casein kinase 1, gamma [EC:2.7.11.1]	gi|148694162|gb|EDL26109.1|	0	casein kinase 1, gamma 1 [Mus musculus]

mmu-let-7j	NM_175937	-21.7	1	-20.31	170	GO:0044424,GO:0003727,GO:0006412,	K02602|1|2e-54|216|spu:581876|oo18 RNA-binding protein	gi|293651586|ref|NP_787951.2|	0	cytoplasmic polyadenylation element-binding protein 2 isoform 1 [Mus musculus]
mmu-let-7j	NM_139272	-21.4	1	-20.54	169	GO:0031228,GO:0046914,GO:0008376,GO:0030246,GO:0006493,GO:0002377,	K00710|1|0.0|1139|mmu:108148|polypeptide N-acetylgalactosaminyltransferase [EC:2.4.1.41]	gi|46877109|ref|NP_644678.2|	0	polypeptide N-acetylgalactosaminyltransferase 2 precursor [Mus musculus]
mmu-let-7j	NR_045151	-20.1	1	-25.2	168	-	-	gi|148664438|gb|EDK96854.1|	1.08E-104	mCG144467 [Mus musculus]
mmu-let-7j	NM_177664	-21.4	1	-21.29	167	GO:0009986,GO:0016021,GO:0016791,GO:0016311,GO:0048667,	K01080|1|6e-26|121|dre:557680|phosphatidate phosphatase [EC:3.1.3.4]	gi|37359962|dbj|BAC97959.1|	0	mKIAA0455 protein [Mus musculus]
mmu-let-7j	NR_015459	-28.3	0.657092	-22.92	166	-	-	gi|74145126|dbj|BAE22222.1|	5.95E-58	unnamed protein product [Mus musculus]

mmu-let-7j	NM_198022	-20.7	1	-22.04	165	GO:0003676,GO:0016458,	K06237|1|2e-08|64.3|dan:Dana_GF14161|collagen, type IV, alpha	gi|124378035|ref|NP_932139.2|	0	trinucleotide repeat-containing gene 6C protein [Mus musculus]
mmu-let-7j	NR_045293	-25.5	0.549686	-22.92	164	GO:0031228,GO:0016811,GO:0033578,GO:0006670,GO:0007162,GO:0033189,GO:0008283,GO:0001952,	K04613|1|5e-10|66.2|rno:297590|vomeronasal 2 receptor	gi|148705102|gb|EDL37049.1|	5.44E-37	mCG1049986 [Mus musculus]
mmu-let-7j	NR_073122	-28.6	0.400066	-22.47	163	-	-	-	-	-
mmu-let-7j	NR_045906	-21.4	0.998747	-20.67	162	-	-	gi|148691869|gb|EDL23816.1|	5.06E-59	mCG144731 [Mus musculus]
mmu-let-7j	NR_045945	-24.1	0.970994	-22.15	161	GO:0043231,GO:0046914,	K04511|1|5e-21|104|acs:100560390|prickle	gi|309271021|ref|XP_003085199.1|	1.34E-37	PREDICTED: prickle-like protein 4-like [Mus musculus]
mmu-let-7j	NR_045744	-24	0.963431	-20.63	160	-	-	gi|148674962|gb|EDL06909.1|	9.00E-22	mCG140937 [Mus musculus]

mmu-let-7j	NM_181413	-20.2	1	-20.32	159	GO:0044424,GO:0005488,	K15503|1|4e-31|139|aag:AaeL_AAEL014668|serine/threonine-protein phosphatase 6 regulatory ankyrin repeat subunit B	gi|148690611|gb|EDL22558.1|	0	ankyrin repeat and SAM domain containing 1, isoform CRA_c [Mus musculus]
mmu-let-7j	NM_178724	-27.7	0.430042	-22.02	158	GO:0043231,GO:0016788,GO:0043169,	-	gi|62871735|gb|AAH94315.1|	0	Harbi1 protein [Mus musculus]
mmu-let-7j	NM_181039	-20.6	1	-21.83	157	GO:0031224,GO:0044430,GO:0004888,GO:0030246,GO:0007417,GO:0007186,GO:00068	K04592|1|0.0|2529|mmu:330814|latrophilin 1	gi|395850996|ref|XP_003798056.1|	0	PREDICTED: latrophilin-1, partial [Otolemur garnettii]
mmu-let-7j	NM_198303	-22.1	1	-20.95	156	GO:0044444,GO:0008135,GO:0000166,GO:0006413,	K03243|1|0.0|1711|mmu:226982|translation initiation factor 5B	gi|84043961|ref|NP_938045.2|	0	eukaryotic translation initiation factor 5B [Mus musculus]

mmu-let-7j	NM_181734	-22.7	0.999921	-21.66	155	GO:0044464,GO:0051234,	K01104|1|4e-21|105|phu:Phum_PHUM243230|protein-tyrosine phosphatase [EC:3.1.3.48]	gi|148674393|gb|EDL06340.1|	0	RIKEN cDNA 5830472M02, isoform CRA_b [Mus musculus]
mmu-let-7j	NR_027710	-26.6	0.922288	-21.42	154	-	K07202|1|0.0|584|mmu:19017|peroxisome proliferative activated receptor, gamma, coactivator 1, alpha	gi|45219869|gb|AAH66868.1|	0	Peroxisome proliferative activated receptor, gamma, coactivator 1 alpha [Mus musculus]
mmu-let-7j	NR_040262	-24.6	0.377039	-21.67	153	-	-	gi|148681769|gb|EDL13716.1|	6.13E-30	mCG4750, isoform CRA_b [Mus musculus]
mmu-let-7j	NM_172153	-21.5	1	-20.23	152	GO:0043231,GO:0004871,GO:0003676,GO:0006351,	-	gi|253683551|ref|NP_001156545.1|	0	ligand-dependent nuclear receptor corepressor-like protein isoform 1 [Mus musculus]
mmu-let-7j	NR_045608	-23.7	0.997616	-20.02	151	GO:0043231,GO:0046914,GO:0003676,	K11485|1|0.0|1184|aml:100480331|polycomb-like protein 2	gi|301765136|ref|XP_002917993.1|	0	PREDICTED: metal-response element-binding transcription factor 2-like [Ailuropoda melanoleuca]

mmu-let-7j	NM_145599	-22.9	0.995987	-20.21	150	GO:0031224,	K14360|1|2e-10|68.9|dre:799052|organic solute transporter subunit alpha	gi|269973921|ref|NP_663574.3|	0	transmembrane protein 184C [Mus musculus]
mmu-let-7j	NM_182698	-20.2	1	-22.59	149	GO:0005902,GO:0015629,GO:0008092,GO:0017111,GO:0032559,GO:0042471,GO:0050954,GO:0007610,	K10361|1|0.0|4514|mmu:17910|myosin XV	gi|157041248|ref|NP_001096641.1|	0	unconventional myosin-XV isoform 3 [Mus musculus]

mmu-let-7j	NM_172732	-20.4	1	-21.8	148	GO:0031224,GO:0004871,GO:0030246,GO:0023052,	K08763|1|9e-24|113|acs:100564370|oxidised low-density lipoprotein receptor 1!K10070|2|5e-21|104|cfa:486696|C-type lectin domain family 1, member B!K10074|5|4e-18|94.7|ecb:100053863|C-type lectin domain family 7, member	gi|148667507|gb|EDK99923.1|	1.48E-154	C-type lectin domain family 9, member a, isoform CRA_b [Mus musculus]
mmu-let-7j	NR_046162	-23.6	0.975185	-20.05	147	-	-	gi|148665186|gb|EDK97602.1|	7.08E-114	mCG144829 [Mus musculus]

mmu-let-7j	NM_001039347	-21.9	1	-20.28	145	GO:0005887,GO:0044297,GO:0042995,GO:0043169,GO:0015271,GO:0034220,GO:0030001,	K04893|1|0.0|1125|mmu:56543|potassium voltage-gated channel Shal-related subfamily D member 3	gi|86991434|ref|NP_001034436.1|	0	potassium voltage-gated channel subfamily D member 3 isoform 1 precursor [Mus musculus]

mmu-let-7j	NM_011035	-20.3	1	-20.68	140	GO:0030427,GO:0043005,GO:0005635,GO:0031674,GO:0044291,GO:0005924,GO:0019900,GO:0032559,GO:0004702,GO:0007172,GO:0032868,GO:0048858,GO:0050851,GO:0031175,GO:0043406,GO:0007243,GO:0001934,GO:0006935,GO:0048741,GO:0030036,GO:0006950,GO:00125	K04409|1|0.0|1001|mmu:18479|p21-activated kinase 1 [EC:2.7.11.1]	gi|112181194|ref|NP_035165.2|	0	serine/threonine-protein kinase PAK 1 [Mus musculus]
mmu-let-7j	NM_172967	-20.4	1	NA	NA	-	-	gi|148677635|gb|EDL09582.1|	0	RIKEN cDNA 4930503L19, isoform CRA_e [Mus musculus]

mmu-let-7j	NM_001134384	-23.3	0.999946	NA	NA	-	K12495|1|0.0|1498|hsa:9922|IQ motif and SEC7 domain-containing protein	gi|197304784|ref|NP_001127856.1|	0	IQ motif and SEC7 domain-containing protein 1 isoform b [Mus musculus]
mmu-let-7j	NM_011308	-25.3	0.99604	NA	NA	GO:0031981,GO:0005819,GO:0044444,GO:0004871,GO:0030234,GO:0042974,GO:0003712,GO:0001071,GO:0043565,GO:0019899,GO:0022037,GO:0055092,GO:0035264,GO:0007051,GO:0043409,GO:0006629,GO:0021536,GO:0016575,GO:0000122,GO:0007623,GO:0030218,GO:00433	K04650|1|0.0|3835|mmu:20185|nuclear receptor co-repressor 1	gi|356640157|ref|NP_035438.3|	0	nuclear receptor corepressor 1 isoform 2 [Mus musculus]

mmu-let-7j	NM_027182	-20.4	0.999999	NA	NA	-	K13525|1|9e-12|73.2|ssc:397524|transitional endoplasmic reticulum ATPase!K12196|3|1e-11|72.8|tad:TRIADDRAFT_63201|vacuolar protein-sorting-associated protein	gi|110625724|ref|NP_081458.1|	0	pachytene checkpoint protein 2 homolog [Mus musculus]
mmu-let-7j	NM_009548	-26.1	0.7769	NA	NA	GO:0043231,GO:0017111,GO:0046914,GO:0032561,	K12008|1|5e-07|57.8|mdo:100022140|tripartite motif-containing protein 26!K12031|4|2e-06|56.2|tgu:100220742|tripartite motif-containing protein	gi|20127402|ref|NP_033574.2|	0	RING finger protein 112 [Mus musculus]

mmu-let-7j	NM_172400	-21.7	0.994257	NA	NA	GO:0031981,GO:0005515,GO:0000377,GO:0044267,	K09528|1|4e-122|438|ssc:100621067|DnaJ homolog subfamily C member 8	gi|148698151|gb|EDL30098.1|	1.55E-125	DnaJ (Hsp40) homolog, subfamily C, member 8, isoform CRA_a [Mus musculus]
mmu-let-7j	NM_024244	-23.2	0.996936	NA	NA	-	-	gi|148700024|gb|EDL31971.1|	0	RIKEN cDNA 1200015N20, isoform CRA_b [Mus musculus]
mmu-let-7j	NM_172257	-22.6	0.999905	NA	NA	GO:0005764,GO:0031224,	K07994|1|3e-126|384|gga:395683|matrix metalloproteinase-13 (collagenase 3) [EC:3.4.24.-]	gi|148693723|gb|EDL25670.1|	0	SID1 transmembrane family, member 2, isoform CRA_c [Mus musculus]
mmu-let-7j	NM_001177550	-25	0.893886	NA	NA	-	K09228|1|0.0|1341|mmu:668923|KRAB domain-containing zinc finger protein	gi|294712573|ref|NP_001171021.1|	0	zinc finger protein 442 [Mus musculus]

mmu-let-7j	NM_001252541	-22.2	0.999991	NA	NA	GO:0005856,GO:0043231,GO:0044444,GO:0046914,GO:0050662,GO:0003824,GO:0000166,GO:0009987,	K00327|1|2e-60|235|bta:532512|NADPH-ferrihemoprotein reductase [EC:1.6.2.4]	gi|357527449|ref|NP_001239470.1|	0	NADPH-dependent diflavin oxidoreductase 1 isoform 2 [Mus musculus]
mmu-let-7j	NM_175836	-21.4	1	NA	NA	GO:0031981,GO:0032991,GO:0016020,GO:0030016,GO:0030864,GO:0005198,GO:0008092,GO:0007178,GO:0030835,GO:0006935,GO:0006606,	K06115|1|0.0|4308|hsa:6711|spectrin beta	gi|117938332|ref|NP_787030.2|	0	spectrin beta chain, brain 1 isoform 1 [Mus musculus]
mmu-let-7j	NM_001111118	-23.6	0.990975	NA	NA	-	-	gi|162135969|ref|NP_001104588.1|	0	RAD51-associated protein 2 [Mus musculus]
mmu-let-7j	NM_030061	-23.6	0.720655	NA	NA	GO:0004866,GO:0010466,GO:0030162,	-	gi|27229257|ref|NP_084337.1|	9.44E-47	serine protease inhibitor Kazal-type 12 [Mus musculus]

mmu-let-7j	NM_001077407	-21.9	0.999999	NA	NA	GO:0031224,GO:0043005,GO:0005488,GO:0004714,GO:0048869,GO:0001667,GO:0006935,GO:0007169,GO:0048513,	K06819|1|0.0|1773|cfa:488487|neuropilin 2	gi|116686144|ref|NP_001070875.1|	0	neuropilin-2 isoform 6 precursor [Mus musculus]
mmu-let-7j	NM_175374	-24.8	0.901662	NA	NA	GO:0043231,GO:0003747,GO:0006412,	K02835|1|0.0|739|mmu:108853|peptide chain release factor 1	gi|30425080|ref|NP_780583.1|	0	peptide chain release factor 1-like, mitochondrial precursor [Mus musculus]
mmu-let-7j	NM_134009	-20.6	1	NA	NA	GO:0031090,GO:0031224,GO:0031981,GO:0005488,GO:0016787,GO:0019538,GO:00071	-	gi|344247008|gb|EGW03112.1|	0	Nicalin [Cricetulus griseus]

mmu-let-7j	NM_001163671	-24.7	0.984608	NA	NA	GO:0031981,GO:0044444,GO:0016505,GO:0032559,GO:0019901,GO:0016909,GO:0070555,GO:0001932,GO:0010557,GO:0010243,GO:0016567,GO:0010742,GO:0031328,GO:0010952,GO:0000904,GO:0009411,GO:0032305,GO:0043408,GO:0009605,GO:0001516,GO:0001819,GO:00073	K04440|1|0.0|798|mmu:26420|c-Jun N-terminal kinase [EC:2.7.11.24]	gi|254750733|ref|NP_001157143.1|	0	mitogen-activated protein kinase 9 isoform beta2 [Mus musculus]

mmu-let-7j	NM_009351	-21.8	1	NA	NA	GO:0044427,GO:0034399,GO:0030529,GO:0003676,GO:0003964,GO:0032559,GO:0006278,GO:0000723,	K11127|1|0.0|4957|mmu:21745|telomerase protein component 1	gi|6678285|ref|NP_033377.1|	0	telomerase protein component 1 [Mus musculus]
mmu-let-7j	NM_028115	-24.8	0.963414	NA	NA	GO:0003676,GO:0016866,GO:0010467,GO:0009451,GO:0006399,	K03177|1|6e-165|582|mmu:72133|tRNA pseudouridine55 synthase [EC:5.4.99.25]	gi|21312414|ref|NP_082391.1|	4.19E-164	probable tRNA pseudouridine synthase 1 isoform 1 [Mus musculus]
mmu-let-7j	NM_027810	-20	1	NA	NA	GO:0043234,GO:0031253,GO:0044424,GO:0005488,GO:0048858,GO:0003143,GO:0050953,GO:0030154,GO:0009855,GO:00324	-	gi|148703141|gb|EDL35088.1|	0	Bardet-Biedl syndrome 7, isoform CRA_f [Mus musculus]

mmu-let-7j	NM_009758	-24.2	0.997268	NA	NA	GO:0031224,GO:0044459,GO:0004675,GO:0032559,GO:0042802,GO:0043169,GO:0030326,GO:0007178,GO:0008284,GO:0030282,GO:0003006,GO:0043009,GO:0003203,GO:0042476,GO:0021536,GO:0001934,GO:0009798,GO:0045597,GO:0040007,GO:0042307,GO:0009952,GO:00023	K04673|1|0.0|1076|mmu:12166|bone morphogenetic protein receptor type-1A [EC:2.7.11.30]	gi|46519168|ref|NP_033888.2|	0	bone morphogenetic protein receptor type-1A precursor [Mus musculus]

mmu-let-7j	NM_029054	-23.5	0.912273	NA	NA	GO:0019866,GO:0043231,GO:0031224,GO:0046872,GO:0006810,	K14684|1|0.0|950|mmu:74686|solute carrier family 25 (mitochondrial phosphate transporter), member 23/24/25/41	gi|198278549|ref|NP_083330.1|	0	RIKEN cDNA 4930443G12 [Mus musculus]
mmu-let-7j	NM_172637	-23.6	0.998813	NA	NA	GO:0044464,GO:0019787,GO:0016567,	K12232|1|0.0|1369|mmu:226098|E3 ubiquitin-protein ligase HECTD2 [EC:6.3.2.19]	gi|254939513|ref|NP_766225.2|	0	probable E3 ubiquitin-protein ligase HECTD2 isoform 2 [Mus musculus]
mmu-let-7j	NM_007807	-21.9	0.999999	NA	NA	-	K08008|1|0.0|1105|mmu:13058|NADPH oxidase	gi|161333819|ref|NP_031833.3|	0	cytochrome b-245 heavy chain [Mus musculus]
mmu-let-7j	NM_177373	-21.4	1	NA	NA	GO:0044424,GO:0005488,GO:0031589,	K10352|1|5e-16|89.0|aml:100466547|myosin heavy chain	gi|60360140|dbj|BAD90289.1|	0	mKIAA4112 protein [Mus musculus]

mmu-let-7j	NM_198415	-26	0.52767	NA	NA	GO:0043231,GO:0030016,GO:0019866,GO:0016301,GO:0032559,GO:0006796,GO:0003012,GO:0006603,	K00933|1|0.0|857|rno:688698|creatine kinase [EC:2.7.3.2]	gi|38259206|ref|NP_940807.1|	0	creatine kinase S-type, mitochondrial precursor [Mus musculus]
mmu-let-7j	NM_028201	-21	0.999995	NA	NA	GO:0005488,	-	gi|84579818|ref|NP_082477.1|	6.62E-179	UPF0492 protein C20orf94 homolog isoform 2 [Mus musculus]
mmu-let-7j	NM_023041	-23.4	0.993721	NA	NA	GO:0031224,GO:0005903,GO:0044439,GO:0005515,GO:0007031,GO:0006605,	K13337|1|3e-130|467|rno:289233|peroxin-19	gi|226958490|ref|NP_075528.3|	3.20E-138	peroxisomal biogenesis factor 19 isoform a [Mus musculus]
mmu-let-7j	NM_001173553	-20.7	1	NA	NA	GO:0031981,GO:0015630,GO:0005488,GO:0004721,GO:0006470,	K06639|1|0.0|966|mmu:229776|cell division cycle 14 [EC:3.1.3.48]	gi|291167780|ref|NP_001167024.1|	0	dual specificity protein phosphatase CDC14A isoform 2 [Mus musculus]

mmu-let-7j	NM_183149	-21.1	0.999999	NA	NA	GO:0046872,	K14859|1|1e-27|126|smm:Smp_173890|ribosome biogenesis protein SSF1/2	gi|34147169|ref|NP_898972.1|	0	zinc finger protein 598 [Mus musculus]
mmu-let-7j	NM_015805	-23.9	0.9987	NA	NA	GO:0031224,GO:0042625,GO:0004871,GO:0046872,GO:0005548,GO:0032559,GO:0009206,GO:0006811,GO:0023052,GO:0006869,GO:0009207,	K01530|1|0.0|2102|mmu:50771|phospholipid-translocating ATPase [EC:3.6.3.1]	gi|40807502|ref|NP_056620.2|	0	probable phospholipid-transporting ATPase IIB isoform 2 [Mus musculus]
mmu-let-7j	NM_024166	-22.7	0.868457	NA	NA	-	K11837|1|1e-21|103|bfo:BRAFLDRAFT_121867|ubiquitin carboxyl-terminal hydrolase 6/32 [EC:3.1.2.15]	gi|148687546|gb|EDL19493.1|	6.11E-49	mCG145321, isoform CRA_a [Mus musculus]

mmu-let-7j	NM_133224	-20.2	1	NA	NA	GO:0031224,GO:0043169,GO:0042625,GO:0032559,GO:0009206,GO:0006811,GO:0009207,	K14950|1|0.0|2165|mmu:170759|cation-transporting ATPase 13A1 [EC:3.6.3.-]	gi|283135194|ref|NP_573487.2|	0	probable cation-transporting ATPase 13A1 [Mus musculus]
mmu-let-7j	NM_138582	-20.8	1	NA	NA	-	-	gi|20147795|ref|NP_613048.1|	0	von Willebrand factor A domain-containing protein 7 precursor [Mus musculus]
mmu-let-7j	NM_010880	-21.2	1	NA	NA	GO:0044444,GO:0032991,GO:0031981,GO:0043565,GO:0005515,GO:0048646,	K11294|1|0.0|784|mmu:17975|nucleolin	gi|13529464|gb|AAH05460.1|	0	Nucleolin [Mus musculus]
mmu-let-7j	NM_001045864	-21.8	0.996423	NA	NA	GO:0031981,GO:0003676,GO:0045935,GO:0006354,	K15182|1|1e-139|497|mmu:27632|negative elongatio	gi|148694798|gb|EDL26745.1|	8.55E-139	RD RNA-binding protein, isoform CRA_a [Mus musculus]

mmu-let-7j	NM_001099632	-20.3	0.999962	NA	NA	GO:0044424,GO:0046914,GO:0048167,	K12015|1|1e-29|131|mgp:100541069|tripartite motif-containing protein 39!K12000|4|3e-28|127|mdo:100018451|tripartite motif-containing protein 7	gi|153791477|ref|NP_001093102.1|	0	RING finger protein 39 [Mus musculus]
mmu-let-7j	NM_001083912	-22.5	0.999986	NA	NA	GO:0044444,GO:0005088,GO:0019222,GO:0006917,GO:0007266,GO:0009966,GO:0007169,	K12365|1|1e-21|107|rno:311647|phosphatidylinositol-3,4,5-trisphosphate-dependent Rac exchanger 1 protein	gi|47847376|dbj|BAD21360.1|	0	mFLJ00018 protein [Mus musculus]
mmu-let-7j	NR_015613	-23.4	0.99195	NA	NA	-	-	gi|12853321|dbj|BAB29714.1|	5.89E-47	unnamed protein product [Mus musculus]

mmu-let-7j	NM_023128	-20.7	0.999999	NA	NA	GO:0016021,GO:0044459,GO:0031410,GO:0050780,GO:0065008,GO:0006996,GO:0033036,GO:0006171,GO:0031280,	-	gi|239985639|ref|NP_075617.3|	0	paralemmin-1 isoform 1 [Mus musculus]
mmu-let-7j	NM_001142746	-20.1	1	NA	NA	GO:0031513,GO:0005902,GO:0031224,GO:0046872,GO:0016337,GO:0043954,GO:00509	K06813|1|3e-79|299|gga:423718|cadherin 23	gi|218505743|ref|NP_001136218.1|	0	protocadherin-15 isoform CD3-1 precursor [Mus musculus]
mmu-let-7j	NM_001205239	-20.4	1	NA	NA	GO:0016763,	K15258|1|0.0|1186|mmu:67287|poly [ADP-ribose] polymerase 6/8 [EC:2.4.2.30]	gi|327365350|ref|NP_001192168.1|	0	poly [ADP-ribose] polymerase 6 isoform 1 [Mus musculus]

mmu-let-7j	NM_029081	-20	1	NA	NA	-	K06130|1|2e-08|63.2|spu:584566|lysophospholipase II [EC:3.1.1.5]!K15290|2|2e-06|56.6|tgu:751605|synaptotagmin-1	gi|148678709|gb|EDL10656.1|	0	RIKEN cDNA 5730419I09, isoform CRA_b [Mus musculus]
mmu-let-7j	NM_016676	-24.1	0.984689	NA	NA	GO:0044431,GO:0016020,GO:0032561,GO:0035556,GO:0046677,GO:0015031,	K07903|1|4e-112|407|ecb:100055747|Ras-related protein Rab-10	gi|7710086|ref|NP_057885.1|	2.92E-111	ras-related protein Rab-10 [Mus musculus]
mmu-let-7k	NM_001002775	-27.5	0.505992	-26.77	164	-	-	gi|377834441|ref|XP_003689482.1|	0	PREDICTED: uncharacterized protein LOC442809 [Mus musculus]

mmu-let-7k	NM_001007465	-36.7	0.011633	-32.81	168	GO:0043227,GO:0031410,GO:0046914,GO:0003824,GO:0012501,GO:0015031,	K10871|1|5e-70|267|mcc:718665|RAD51-like protein 3!K10641|3|3e-10|68.6|tad:TRIADDRAFT_53391|E3 ubiquitin-protein ligase LRSAM1 [EC:6.3.2.19]!K16060|4|4e-09|65.1|spu:581540|baculoviral IAP repeat-containing protein 2/3!K10637|5|5e-	gi|148683726|gb|EDL15673.1|	0	ring finger and FYVE like domain containing protein, isoform CRA_c [Mus musculus]

mmu-let-7k	NM_001033263	-29.6	0.373843	-26.14	164	GO:0031981,GO:0044444,GO:0019900,GO:0032561,GO:0032559,GO:0008047,GO:0017111,GO:0005083,GO:0019887,GO:0046914,GO:0006357,GO:0001932,GO:0007010,GO:0032012,GO:0033674,GO:0009895,GO:0006935,GO:0043066,GO:0010740,GO:0043087,GO:0033598,GO:00161	K12491|1|0.0|1466|mmu:216439|Arf-GAP, GTPase, ANK repeat and PH domain-containing protein 1/2/3	gi|84370300|ref|NP_001028435.1|	0	arf-GAP with GTPase, ANK repeat and PH domain-containing protein 2 [Mus musculus]
mmu-let-7k	NM_001033383	-27.4	0.562954	-25.61	160	GO:0005488,	K09228|1|4e-62|241|ecb:100062322|KRAB domain-containing zinc finger protein	gi|75677466|ref|NP_001028555.1|	0	zinc finger protein 865 [Mus musculus]

mmu-let-7k	NM_001033424	-27.4	0.532442	-25.08	164	-	K12567|1|5e-37|157|spu:590007|titin [EC:2.7.11.1]	gi|257196223|ref|NP_001028596.2|	0	fibronectin type III domain containing 3C2 [Mus musculus]
mmu-let-7k	NM_001039039	-26.3	0.629398	-28.7	163	GO:0005887,GO:0005267,GO:0034220,GO:0030001,	K15074|1|1e-16|89.7|pon:100450153|BTB/POZ domain-containing adapter for CUL3-mediated RhoA degradation protein	gi|119595459|gb|EAW75053.1|	7.16E-148	hypothetical protein LOC283219, isoform CRA_a [Homo sapiens]
mmu-let-7k	NM_001040130	-30.9	0.018844	-28.03	163	GO:0016020,	-	gi|158517816|ref|NP_001035219.2|	7.99E-47	transmembrane protein 141 isoform 1 [Mus musculus]
mmu-let-7k	NM_001042591	-28.5	0.41219	-25.06	165	GO:0044424,GO:0005488,GO:0023052,	K15183|1|1e-07|60.1|mgp:100549853|RNA polymerase II elongation factor ELL	gi|28972730|dbj|BAC65781.1|	0	mKIAA1376 protein [Mus musculus]

mmu-let-7k	NM_001079875	-30.4	0.095087	-26.38	169	GO:0009986,GO:0031224,GO:0030141,GO:0015929,GO:0009987,GO:0007338,GO:00442	K01197|1|0.0|1066|mmu:20690|hyaluronoglucosaminidase [EC:3.2.1.35]	gi|120407035|ref|NP_001073344.1|	0	hyaluronidase PH-20 precursor [Mus musculus]
mmu-let-7k	NM_001080979	-25.7	0.724104	-44.94	314	GO:0043234,GO:0003676,GO:0001071,GO:0035556,GO:0006357,GO:0045165,GO:00018	K09448|1|0.0|714|mmu:21679|TEA domain family member 1/3/4	gi|148667455|gb|EDK99871.1|	0	TEA domain family member 4, isoform CRA_b [Mus musculus]

mmu-let-7k	NM_001081044	-33.5	0.03753	-32.06	163	GO:0044430,GO:0044297,GO:0030016,GO:0030425,GO:0033267,GO:0030136,GO:0017022,GO:0004674,GO:0032559,GO:0006937,GO:0060415,GO:0048167,GO:0010627,GO:0016192,GO:0006468,GO:0031443,GO:0048522,GO:0007049,GO:00420	K00907|1|0.0|1063|mmu:228785|myosin-light-chain kinase [EC:2.7.11.18]	gi|163644275|ref|NP_001074513.2|	0	myosin light chain kinase 2, skeletal/cardiac muscle [Mus musculus]

mmu-let-7k	NM_001081102	-25.5	0.961124	-26.34	167	GO:0031981,GO:0043232,GO:0005635,GO:0003676,GO:0046914,GO:0016279,GO:0006357,GO:0003285,GO:0003284,GO:0006325,GO:0009653,	K11424|1|0.0|2639|mmu:107823|histone-lysine N-methyltransferase NSD2 [EC:2.1.1.43]	gi|295424164|ref|NP_001074571.2|	0	probable histone-lysine N-methyltransferase NSD2 isoform 1 [Mus musculus]
mmu-let-7k	NM_001081181	-27.3	0.21104	-25.48	159	-	-	gi|148667637|gb|EDL00054.1|	3.97E-128	mCG113908 [Mus musculus]
mmu-let-7k	NM_001081197	-29.1	0.116838	-27.76	168	GO:0046914,GO:0016810,	K01500|1|9e-132|471|hsa:403314|[EC:3.5.4.-]	gi|148707507|gb|EDL39454.1|	0	mCG1047261 [Mus musculus]
mmu-let-7k	NM_001081220	-26	0.967374	-50.54	320	GO:0031224,GO:0004888,	K08469|1|0.0|683|tgu:100229682|G protein-coupled receptor 158	gi|124487045|ref|NP_001074689.1|	0	probable G-protein coupled receptor 179 precursor [Mus musculus]

mmu-let-7k	NM_001081347	-29.1	0.3522	-46.59	312	GO:0044444,GO:0016020,GO:0032561,GO:0035556,GO:0009966,	K07868|1|0.0|1371|mmu:69288|Rho-related BTB domain-containing protein 1/2	gi|124486809|ref|NP_001074816.1|	0	rho-related BTB domain-containing protein 1 isoform 1 [Mus musculus]
mmu-let-7k	NM_001109993	-30.9	0.029319	-28.03	163	GO:0016020,	-	gi|158517816|ref|NP_001035219.2|	3.45E-36	transmembrane protein 141 isoform 1 [Mus musculus]
mmu-let-7k	NM_001113360	-28.3	0.517369	-53.93	314	GO:0016020,GO:0060089,GO:0046872,GO:0004629,GO:0007165,GO:0006650,	K15370|1|6e-104|380|nve:NEMVE_v1g89019|inactive phospholipase C-like	gi|164565357|ref|NP_001106831.1|	0	1-phosphatidylinositol 4,5-bisphosphate phosphodiesterase eta-2 isoform b [Mus musculus]

mmu-let-7k	NM_001113553	-31.2	0.107225	-27.6	164	GO:0031090,GO:0044444,GO:0016301,GO:0046983,GO:0042802,GO:0032559,GO:0051091,GO:0002224,GO:0043433,GO:0019221,GO:0006468,GO:0007249,GO:0006915,GO:00	K04731|1|0.0|1164|mmu:108960|interleukin-1 receptor-associated kinase 2 [EC:2.7.11.1]	gi|148667078|gb|EDK99494.1|	0	mCG132967, isoform CRA_b [Mus musculus]
mmu-let-7k	NM_001122732	-25.3	0.393727	-25.42	164	GO:0012501,GO:0048232,	-	gi|22208842|ref|NP_082923.1|	9.46E-37	spermatogenesis-associated protein 3 isoform 3 [Mus musculus]
mmu-let-7k	NM_001142697	-29.2	0.30016	-25.74	165	GO:0015630,	-	gi|218156330|ref|NP_001004361.2|	2.24E-133	tubulin polyglutamylase complex subunit 2 isoform a [Mus musculus]

mmu-let-7k	NM_001145830	-31.8	0.209746	-29.86	163	GO:0016604,GO:0000785,GO:0044444,GO:0035091,GO:0060089,GO:0046872,GO:0008047,GO:0004629,GO:0042802,GO:0007186,GO:0045661,GO:0045836,GO:0007169,GO:0021543,GO:0009790,GO:0007611,GO:0019221,GO:0009101,GO:0045927,GO:0001819,GO:0006650,GO:00073	K05858|1|0.0|2138|mmu:18795|phosphatidylinositol phospholipase C, beta [EC:3.1.4.11]	gi|224967070|ref|NP_001139302.1|	0	1-phosphatidylinositol 4,5-bisphosphate phosphodiesterase beta-1 isoform 1 [Mus musculus]
mmu-let-7k	NM_001145970	-27.1	0.526167	-25.01	154	GO:0043232,	K10433|1|1e-18|96.7|acs:100551816|microtubule-associated protein 7	gi|148698350|gb|EDL30297.1|	0	arginine/proline rich coiled-coil 1, isoform CRA_c [Mus musculus]

mmu-let-7k	NM_001163175	-28.7	0.261893	-25.23	156	GO:0044424,GO:0042802,	K06105|1|3e-45|184|dre:566060|tight junction protein 4 (peripheral)	gi|253683505|ref|NP_001156647.1|	0	brain-enriched guanylate kinase-associated protein [Mus musculus]
mmu-let-7k	NM_001163662	-26.6	0.532046	-25.23	155	GO:0005856,GO:0044444,GO:0044421,GO:0043231,GO:0046872,GO:0003676,	K12385|1|1e-66|255|isc:IscW_ISCW020300|Niemann-Pick C1 protein	gi|148690913|gb|EDL22860.1|	0	nucleobindin 1, isoform CRA_a [Mus musculus]
mmu-let-7k	NM_001163676	-29.3	0.404759	-46.33	307	GO:0044459,GO:0030667,GO:0042625,GO:0032559,GO:0009719,GO:0008284,GO:0010033,	K05673|1|0.0|2261|mmu:239273|ATP-binding cassette, subfamily C (CFTR/MRP), member 4	gi|255683328|ref|NP_001157148.1|	0	ATP-binding cassette, sub-family C (CFTR/MRP), member 4 isoform 3 [Mus musculus]

mmu-let-7k	NM_001164569	-36.7	0.012333	-32.81	168	GO:0043227,GO:0031410,GO:0046914,GO:0003824,GO:0012501,GO:0015031,	K10871|1|5e-70|267|mcc:718665|RAD51-like protein 3!K10641|3|4e-10|68.6|tad:TRIADDRAFT_53391|E3 ubiquitin-protein ligase LRSAM1 [EC:6.3.2.19]!K16060|4|4e-09|65.1|spu:581540|baculoviral IAP repeat-containing protein 2/3!K10637|5|5e-	gi|257196123|ref|NP_001158041.1|	0	E3 ubiquitin-protein ligase rififylin isoform 3 [Mus musculus]

mmu-let-7k	NM_001164570	-36.7	0.01205	-32.81	168	GO:0043227,GO:0031410,GO:0046914,GO:0003824,GO:0012501,GO:0015031,	K10871|1|5e-70|267|mcc:718665|RAD51-like protein 3!K10641|3|4e-10|68.6|tad:TRIADDRAFT_53391|E3 ubiquitin-protein ligase LRSAM1 [EC:6.3.2.19]!K16060|4|4e-09|65.1|spu:581540|baculoviral IAP repeat-containing protein 2/3!K10637|5|5e-	gi|257153330|ref|NP_001158042.1|	0	E3 ubiquitin-protein ligase rififylin isoform 4 [Mus musculus]

mmu-let-7k	NM_001164571	-36.7	0.011242	-32.81	168	GO:0043227,GO:0031410,GO:0046914,GO:0003824,GO:0012501,GO:0015031,	K10871|1|1e-69|266|mcc:718665|RAD51-like protein 3!K10641|3|3e-10|68.6|tad:TRIADDRAFT_53391|E3 ubiquitin-protein ligase LRSAM1 [EC:6.3.2.19]!K16060|4|4e-09|65.1|spu:581540|baculoviral IAP repeat-containing protein 2/3!K10637|5|5e-	gi|148683726|gb|EDL15673.1|	0	ring finger and FYVE like domain containing protein, isoform CRA_c [Mus musculus]

mmu-let-7k	NM_001164743	-27.7	0.534201	-43.92	306	GO:0016021,GO:0005903,GO:0044459,GO:0044444,GO:0031090,GO:0008509,GO:0015081,GO:0005244,GO:0005342,GO:0006863,GO:0015893,GO:0015698,GO:0006082,GO:0043269,GO:0030001,	K12300|1|0.0|881|mmu:105355|MFS transporter, ACS family, solute carrier family 17 (sodium-dependent inorganic phosphate cotransporter), member 1/2/3/4	gi|56237850|emb|CAI25831.1|	0	solute carrier family 17 (sodium phosphate), member 3 [Mus musculus]
mmu-let-7k	NM_001166549	-29.1	0.306953	-44.78	315	GO:0043231,GO:0044444,GO:0008135,GO:0006810,GO:0006412,	-	gi|262118204|ref|NP_001160020.1|	0	eukaryotic translation initiation factor 4E transporter isoform b [Mus musculus]
mmu-let-7k	NM_001170746	-26.7	0.852255	-44.64	327	GO:0044459,GO:0032991,GO:0019904,GO:0019899,GO:0007172,GO:0051259,	K05629|1|0.0|2370|rno:113970|atrophin-1 interacting protein 1	gi|282721034|ref|NP_001164217.1|	0	membrane-associated guanylate kinase, WW and PDZ domain-containing protein 2 isoform 1 [Mus musculus]

mmu-let-7k	NM_001190187	-28.7	0.377806	-25.21	165	GO:0016021,GO:0030296,GO:0005102,GO:0035556,GO:0060788,GO:0048732,GO:00160	K05457|1|0.0|1175|mmu:18183|neuregulin 3	gi|298231177|ref|NP_001177116.1|	0	pro-neuregulin-3, membrane-bound isoform isoform 2 [Mus musculus]
mmu-let-7k	NM_001190188	-28.7	0.374083	-25.21	165	GO:0016021,GO:0030296,GO:0005102,GO:0035556,GO:0060788,GO:0048732,GO:00160	K05457|1|0.0|1098|mmu:18183|neuregulin 3	gi|298231179|ref|NP_001177117.1|	0	pro-neuregulin-3, membrane-bound isoform isoform 3 [Mus musculus]
mmu-let-7k	NM_001205068	-26.1	0.804763	-25.46	158	-	K11323|1|1e-17|94.0|dwi:Dwil_GK14253|histone arginine demethylase JMJD6 [EC:1.14.1	gi|326633177|ref|NP_001191997.1|	0	jmjC domain-containing protein 4 isoform 2 [Mus musculus]
mmu-let-7k	NM_001242363	-33.7	0.163033	-40.78	314	GO:0043234,GO:0005488,GO:0007041,GO:0016197,GO:0007033,	-	gi|39930595|ref|NP_950174.1|	0	FTS and Hook-interacting protein isoform a [Mus musculus]

mmu-let-7k	NM_001242364	-33.7	0.162294	-40.78	314	GO:0043234,GO:0007041,GO:0016197,GO:0007033,	-	gi|334358879|ref|NP_001229293.1|	0	FTS and Hook-interacting protein isoform b [Mus musculus]
mmu-let-7k	NM_001252636	-29.1	0.350962	-46.59	312	GO:0044444,GO:0016020,GO:0032561,GO:0035556,GO:0009966,	K07868|1|0.0|1371|mmu:69288|Rho-related BTB domain-containing protein 1/2	gi|124486809|ref|NP_001074816.1|	0	rho-related BTB domain-containing protein 1 isoform 1 [Mus musculus]
mmu-let-7k	NM_001252637	-27.9	0.528161	-46.59	312	GO:0044444,GO:0016020,GO:0032561,GO:0035556,GO:0009966,	K07868|1|0.0|1371|mmu:69288|Rho-related BTB domain-containing protein 1/2	gi|124486809|ref|NP_001074816.1|	0	rho-related BTB domain-containing protein 1 isoform 1 [Mus musculus]
mmu-let-7k	NM_001252638	-27.9	0.533174	-46.59	312	GO:0044444,GO:0016020,GO:0032561,GO:0035556,GO:0009966,	K07868|1|0.0|1167|mmu:69288|Rho-related BTB domain-containing protein 1/2	gi|124486809|ref|NP_001074816.1|	0	rho-related BTB domain-containing protein 1 isoform 1 [Mus musculus]
mmu-let-7k	NM_001271578	-25.4	0.705239	-67.8	471	-	-	-	-	-

mmu-let-7k	NM_007400	-26.2	0.930071	-44.49	314	GO:0031224,GO:0004175,GO:0046914,GO:0019904,GO:0019538,GO:0007169,GO:0000768,	K06835|1|0.0|1772|mmu:11489|disintegrin and metalloproteinase domain-containing protein 12 [EC:3.4.24.-]	gi|117606341|ref|NP_031426.2|	0	disintegrin and metalloproteinase domain-containing protein 12 precursor [Mus musculus]
mmu-let-7k	NM_007548	-27	0.725472	-46.67	308	GO:0043231,GO:0046914,GO:0001071,GO:0003677,GO:0019899,GO:0000122,GO:0002832,GO:0003006,GO:0001890,GO:0030888,GO:0045577,GO:0001701,	K09228|1|1e-40|170|bfo:BRAFLDRAFT_71028|KRAB domain-containing zinc finger protein	gi|125859109|gb|AAI29802.1|	0	Prdm1 protein [Mus musculus]
mmu-let-7k	NM_008158	-28.2	0.117003	-25.86	159	GO:0016020,GO:0004871,GO:0007166,	K04301|1|2e-128|459|rno:65275|G protein-coupled receptor	gi|6680073|ref|NP_032184.1|	1.23E-127	probable G-protein coupled receptor 27 [Mus musculus]

mmu-let-7k	NM_008255	-32.5	0.096995	-29.63	171	GO:0031224,GO:0031090,GO:0044439,GO:0048037,GO:0000166,GO:0016616,GO:0042802,GO:0006732,GO:0006694,GO:0008203,GO:0045137,GO:0007632,GO:0016114,GO:0043405,GO:0007276,GO:0045844,GO:00	K00021|1|0.0|1693|mmu:15357|hydroxymethylglutaryl-CoA reductase (NADPH) [EC:1.1.1.34]	gi|160358778|ref|NP_032281.2|	0	3-hydroxy-3-methylglutaryl-coenzyme A reductase [Mus musculus]

mmu-let-7k	NM_008355	-29.7	0.063183	-27.26	164	GO:0044424,GO:0044421,GO:0005126,GO:0015992,GO:0010524,GO:0009612,GO:0001818,GO:0030888,GO:0023052,GO:0002377,GO:0007154,GO:0008284,GO:0002696,GO:0014070,GO:0002237,GO:00069	K05435|1|3e-56|219|mmu:16163|interleukin 13	gi|6680403|ref|NP_032381.1|	1.79E-55	interleukin-13 precursor [Mus musculus]
mmu-let-7k	NM_008734	-28.7	0.378053	-25.21	165	GO:0016021,GO:0030296,GO:0005102,GO:0035556,GO:0060788,GO:0048732,GO:00160	K05457|1|0.0|1169|mmu:18183|neuregulin 3	gi|6679128|ref|NP_032760.1|	0	pro-neuregulin-3, membrane-bound isoform isoform 1 [Mus musculus]
mmu-let-7k	NM_008749	-26.6	0.530233	-25.23	155	GO:0005856,GO:0044444,GO:0044421,GO:0043231,GO:0046872,GO:0003676,	K12385|1|1e-66|255|isc:IscW_ISCW020300|Niemann-Pick C1 protein	gi|148690913|gb|EDL22860.1|	0	nucleobindin 1, isoform CRA_a [Mus musculus]

mmu-let-7k	NM_008824	-29.6	0.105267	-29.11	169	GO:0043234,GO:0044444,GO:0070061,GO:0008443,GO:0019899,GO:0019203,GO:0032559,GO:0006007,GO:0035556,GO:0033131,GO:0009887,GO:0043434,GO:0031960,GO:0015980,GO:0006000,GO:00069	K01103|1|0.0|910|mmu:18639|6-phosphofructo-2-kinase / fructose-2,6-bisphosphatase [EC:2.7.1.105 3.1.3.46]	gi|7434494|pir||S77704		0	6-phosphofructo-2-kinase (EC 2.7.1.105) / fructose-2, 6-bisphosphate 2-phosphatase (EC 3.1.3.46) clone 5c, skeletal muscle - rat
mmu-let-7k	NM_008988	-27.7	0.430435	-48.13	310	GO:0016021,GO:0050905,	K06765|1|2e-68|261|mdo:100010164|deleted in colorectal carcinoma	gi|6679571|ref|NP_033014.1|		0	immunoglobulin superfamily DCC subclass member 3 [Mus musculus]

mmu-let-7k	NM_009074	-32.2	0.117238	-27.61	163	GO:0031224,GO:0032432,GO:0004871,GO:0004713,GO:0032559,GO:0051707,GO:0007167,GO:0032501,GO:0006464,	K05100|1|0.0|2660|mmu:19882|macrophage-stimulating 1 receptor [EC:2.7.10.1]	gi|254826769|ref|NP_033100.2|	0	macrophage-stimulating protein receptor precursor [Mus musculus]
mmu-let-7k	NM_009218	-27.2	0.591963	-25.77	164	GO:0016021,GO:0072372,GO:0044424,GO:0005488,GO:0004872,GO:0022037,GO:0007186,GO:0051384,GO:0048232,GO:0032355,GO:0007154,GO:0008283,GO:00069	K04219|1|3e-165|583|mmu:20607|somatostatin receptor 3	gi|84370258|ref|NP_033244.2|	1.96E-164	somatostatin receptor type 3 [Mus musculus]
mmu-let-7k	NM_009241	-30.4	0.094742	-26.38	169	GO:0009986,GO:0031224,GO:0030141,GO:0015929,GO:0009987,GO:0007338,GO:00442	K01197|1|0.0|1066|mmu:20690|hyaluronoglucosaminidase [EC:3.2.1.35]	gi|120407035|ref|NP_001073344.1|	0	hyaluronidase PH-20 precursor [Mus musculus]

mmu-let-7k	NM_009298	-33	0.039367	-26.36	166	GO:0031981,GO:0003676,GO:0022613,	-	gi|6678185|ref|NP_033324.1|	6.34E-137	surfeit locus protein 6 [Mus musculus]
mmu-let-7k	NM_009483	-26.8	0.766341	-44.77	310	GO:0034708,GO:0043231,GO:0043169,GO:0016701,GO:0034968,	K11447|1|0.0|2720|mmu:22289|histone demethylase [EC:1.14.1	gi|33859492|ref|NP_033509.1|	0	lysine-specific demethylase 6A [Mus musculus]
mmu-let-7k	NM_010213	-28.4	0.174016	-27.1	161	GO:0032432,GO:0043231,GO:0031674,GO:0046872,GO:0008092,GO:0007010,GO:00485	K14380|1|6e-102|372|dre:450007|four and a half LIM domains protein 2	gi|130488506|ref|NP_034343.2|	3.49E-175	four and a half LIM domains protein 3 [Mus musculus]
mmu-let-7k	NM_010411	-28.3	0.221385	-25.4	162	-	K11404|1|0.0|904|mmu:15183|histone deacetylase 3 [EC:3.5.1.98]	gi|89257352|ref|NP_034541.2|	0	histone deacetylase 3 [Mus musculus]

mmu-let-7k	NM_010436	-29.6	0.081372	-28.45	164	GO:0001739,GO:0043073,GO:0005515,GO:0003677,GO:0000725,GO:0007568,GO:0034728,GO:0006282,GO:0048232,GO:0022403,GO:0009314,	K11251|1|3e-61|236|mmu:15270|histone H2A	gi|7106331|ref|NP_034566.1|	1.74E-60	histone H2A.x [Mus musculus]
mmu-let-7k	NM_010752	-27.7	0.368917	-25.07	161	GO:0005815,GO:0000776,GO:0005635,GO:0005488,GO:0000075,GO:0045841,	K06638|1|0.0|1258|mmu:17120|mitotic spindle assembly checkpoint protein MAD1	gi|88014564|ref|NP_034882.2|	0	mitotic spindle assembly checkpoint protein MAD1 [Mus musculus]
mmu-let-7k	NM_010917	-26.6	0.834404	-47.62	330	GO:0005604,GO:0044424,GO:0046872,GO:0043236,GO:0043062,GO:0010810,GO:00064	K06826|1|0.0|2522|mmu:18073|nidogen (entactin)	gi|171543883|ref|NP_035047.2|	0	nidogen-1 precursor [Mus musculus]

mmu-let-7k	NM_011026	-32.5	0.034293	-28.93	173	GO:0033267,GO:0044430,GO:0044459,GO:0016021,GO:0030425,GO:0044444,GO:0044297,GO:0005231,GO:0050839,GO:0032559,GO:0016502,GO:0042802,GO:0046914,GO:0001775,GO:0055118,GO:0006915,GO:0035587,GO:0048871,GO:0006814,GO:0051899,GO:0001508,GO:00099	K05218|1|0.0|758|mmu:18438|purinergic receptor P2X, ligand-gated ion channel 4	gi|117676374|ref|NP_035156.2|	0	P2X purinoceptor 4 [Mus musculus]

mmu-let-7k	NM_011101	-27.9	0.761165	-25.17	167	GO:0031981,GO:0043005,GO:0032991,GO:0031513,GO:0044425,GO:0044444,GO:0044297,GO:0004697,GO:0035184,GO:0032559,GO:0005515,GO:0046914,GO:0001775,GO:0010829,GO:0001932,GO:0008284,GO:0033673,GO:0010595,GO:0015980,GO:0007169,GO:0051384,GO:00313	K02677|1|0.0|1407|mmu:18750|classical protein kinase C [EC:2.7.11.13]	gi|164663791|ref|NP_035231.2|	0	protein kinase C alpha type [Mus musculus]

mmu-let-7k	NM_013474	-29.4	0.0198	-25.77	159	GO:0034364,GO:0034385,GO:0044432,GO:0004857,GO:0070325,GO:0005543,GO:0032934,GO:0008047,GO:0042802,GO:0071813,GO:0046983,GO:0015248,GO:0006954,GO:0051704,GO:0042108,GO:0010608,GO:0046470,GO:0032787,GO:0015914,GO:0055092,GO:0016125,GO:00108	K08758|1|8e-51|199|mmu:11807|apolipoprotein A-II	gi|157951676|ref|NP_038502.2|	4.92E-50	apolipoprotein A-II precursor [Mus musculus]
mmu-let-7k	NM_013581	-28.3	0.340708	-25.9	159	GO:0012505,GO:0043234,GO:0043231,GO:0005488,GO:0048193,GO:0006996,	-	gi|159110754|ref|NP_038609.3|	0	conserved oligomeric Golgi complex subunit 1 [Mus musculus]

mmu-let-7k	NM_013792	-29.6	0.16584	-25	160	GO:0000323,GO:0015929,GO:0042471,GO:0042462,GO:0021695,GO:0007626,GO:0007033,	K01205|1|0.0|1461|mmu:27419|alpha-N-acetylglucosaminidase [EC:3.2.1.50]	gi|254910995|ref|NP_038820.2|	0	alpha-N-acetylglucosaminidase precursor [Mus musculus]
mmu-let-7k	NM_016695	-34.1	0.045346	-30.19	176	GO:0016021,GO:0019904,GO:0023052,	K06103|1|1e-115|419|ptr:100608149|calcium/calmodulin-dependent serine protein kinase [EC:2.7.11.1]	gi|7710062|ref|NP_057904.1|	0	MAGUK p55 subfamily member 2 [Mus musculus]

mmu-let-7k	NM_019677	-31.8	0.213269	-29.86	163	GO:0016604,GO:0000785,GO:0044444,GO:0035091,GO:0060089,GO:0046872,GO:0008047,GO:0004629,GO:0042802,GO:0007186,GO:0045661,GO:0045836,GO:0007169,GO:0021543,GO:0009790,GO:0007611,GO:0019221,GO:0009101,GO:0045927,GO:0001819,GO:0006650,GO:00073	K05858|1|0.0|1993|mmu:18795|phosphatidylinositol phospholipase C, beta [EC:3.1.4.11]	gi|224967068|ref|NP_062651.2|	0	1-phosphatidylinositol 4,5-bisphosphate phosphodiesterase beta-1 isoform 2 [Mus musculus]

mmu-let-7k	NM_019777	-25.5	0.78503	-26.17	154	GO:0016604,GO:0031090,GO:0044444,GO:0004702,GO:0032559,GO:0007166,GO:0001818,GO:0006796,GO:0002224,GO:0007249,GO:0006464,GO:0033554,	K07211|1|0.0|1439|mmu:56489|inhibitor of nuclear factor kappa-B kinase subunit epsilon [EC:2.7.11.10]	gi|148707764|gb|EDL39711.1|	0	inhibitor of kappaB kinase epsilon, isoform CRA_b [Mus musculus]
mmu-let-7k	NM_020576	-30.9	0.020451	-26.46	162	-	-	gi|5731131|gb|AAD48782.1|AF159090_1	3.30E-54	MHC psoriasis candidate protein [Mus musculus]
mmu-let-7k	NM_021362	-27.7	0.865488	-48.62	325	GO:0044424,GO:0044421,GO:0046914,GO:0004175,GO:0048869,GO:0022414,	K07762|1|0.0|3240|mmu:18491|pappalysin-1 [EC:3.4.24.79]	gi|20148857|gb|AAM12687.1|AF439513_1	0	pregnancy-associated plasma protein-A, partial [Mus musculus]
mmu-let-7k	NM_021390	-33.8	0.069624	-74.22	485	-	K09228|1|2e-24|116|ssc:100156843|KRAB domain-containing zinc finger protein	gi|159032064|ref|NP_067365.2|	0	sal-like protein 1 [Mus musculus]

mmu-let-7k	NM_022015	-27	0.488805	-26.13	165	GO:0005667,GO:0044444,GO:0003676,GO:0045595,GO:0001832,GO:0006355,GO:0072595,	K14649|1|3e-140|500|mmu:63856|transcription initiation factor TFIID subunit 8	gi|26352151|dbj|BAC39712.1|	1.94E-139	unnamed protein product [Mus musculus]
mmu-let-7k	NM_025404	-30.8	0.045614	-28.47	169	GO:0031981,GO:0016020,GO:0017111,GO:0032561,GO:0035556,GO:0009207,GO:00150	K07948|1|4e-102|372|rno:303559|ADP-ribosylation factor-like 4D	gi|13384792|ref|NP_079680.1|	2.62E-101	ADP-ribosylation factor-like protein 4D [Mus musculus]
mmu-let-7k	NM_025708	-30.1	0.157987	-26.47	163	GO:0031224,GO:0043231,	-	gi|27228988|ref|NP_079984.2|	2.24E-122	transmembrane protein 186 [Mus musculus]
mmu-let-7k	NM_025907	-26.1	0.48277	-47.61	316	GO:0016741,GO:0006730,	K00599|1|4e-163|575|mmu:67011|[EC:2.1.1.-]	gi|13385400|ref|NP_080183.1|	3.03E-162	methyltransferase-like protein 6 [Mus musculus]
mmu-let-7k	NM_026045	-27.9	0.433064	-25.78	156	GO:0016604,GO:0030529,GO:0008200,GO:0006396,	K12817|1|0.0|681|mmu:67229|pre-mRNA-splicing factor 18	gi|40254503|ref|NP_080321.2|	0	pre-mRNA-splicing factor 18 [Mus musculus]

mmu-let-7k	NM_026097	-36.7	0.011763	-32.81	168	GO:0043227,GO:0031410,GO:0046914,GO:0003824,GO:0012501,GO:0015031,	K10871|1|1e-69|266|mcc:718665|RAD51-like protein 3!K10641|3|4e-10|68.6|tad:TRIADDRAFT_53391|E3 ubiquitin-protein ligase LRSAM1 [EC:6.3.2.19]!K16060|4|4e-09|65.1|spu:581540|baculoviral IAP repeat-containing protein 2/3!K10637|5|5e-	gi|16975488|ref|NP_080373.1|	0	E3 ubiquitin-protein ligase rififylin isoform 2 [Mus musculus]

mmu-let-7k	NM_026810	-30.8	0.104034	-28.37	171	GO:0043073,GO:0000794,GO:0032300,GO:0017111,GO:0030983,GO:0032559,GO:0032404,GO:0002566,GO:0007131,GO:0000019,GO:0000726,GO:0000288,GO:0009207,GO:0007128,GO:0014070,GO:0051255,GO:0007292,GO:0048232,GO:0007140,GO:0002204,GO	K08734|1|0.0|1419|mmu:17350|DNA mismatch repair protein MLH1	gi|255958238|ref|NP_081086.2|	0	DNA mismatch repair protein Mlh1 [Mus musculus]
mmu-let-7k	NM_027029	-29.4	0.03586	-25.42	164	GO:0012501,GO:0048232,	-	gi|22208836|ref|NP_081305.1|	6.95E-39	spermatogenesis-associated protein 3 isoform 1 [Mus musculus]
mmu-let-7k	NM_027300	-25.3	0.394765	-25.42	164	GO:0012501,GO:0048232,	-	gi|170172533|ref|NP_081576.2|	3.26E-37	spermatogenesis-associated protein 3 isoform 2 [Mus musculus]

mmu-let-7k	NM_027891	-31.3	0.067611	-25.87	156	GO:0043231,GO:0000808,GO:0000781,GO:0000775,GO:0042393,GO:0000166,GO:0006259,GO:0070199,GO:0051318,GO:00063	K13131|1|1e-49|198|smm:Smp_171170|ATP-dependent RNA helicase DDX20 [EC:3.6.4.13]	gi|148687374|gb|EDL19321.1|	0	RIKEN cDNA 1200011O22, isoform CRA_a [Mus musculus]
mmu-let-7k	NM_027917	-32.3	0.073439	-29.24	171	GO:0016459,GO:0015630,GO:0003779,GO:0007015,GO:0032989,	-	gi|125628648|ref|NP_082193.2|	0	protein Shroom1 [Mus musculus]
mmu-let-7k	NM_028004	-25.8	1	-41.66	305	-	K12567|1|0.0|4.870e+04|hsa:7273|titin [EC:2.7.11.1]	gi|77812699|ref|NP_082280.2|	0	titin isoform N2-B [Mus musculus]
mmu-let-7k	NM_028033	-28.1	0.215155	-25.61	164	-	-	gi|197927238|ref|NP_082309.1|	3.78E-98	proline-rich protein 23 [Mus musculus]
mmu-let-7k	NM_030262	-30.1	0.116059	-26.85	154	GO:0043231,GO:0008417,GO:0019318,	K03691|1|0.0|867|mmu:80294|peptide-O-fucosyltransferase [EC:2.4.1.221]	gi|19525731|ref|NP_084538.2|	0	GDP-fucose protein O-fucosyltransferase 2 precursor [Mus musculus]

mmu-let-7k	NM_033269	-26.6	0.604882	-25.02	160	GO:0016021,GO:0033267,GO:0044459,GO:0045202,GO:0004629,GO:0016907,GO:0048731,GO:0015980,GO:0007200,GO:0006940,GO:0043412,GO:0042310,GO:0030073,GO:00	K04131|1|0.0|1115|mmu:12671|muscarinic acetylcholine receptor M3	gi|15149489|ref|NP_150372.1|	0	muscarinic acetylcholine receptor M3 [Mus musculus]
mmu-let-7k	NM_054044	-26.6	0.807128	-46.37	300	GO:0031224,GO:0004888,GO:0001525,GO:0016477,GO:0032101,GO:0022603,GO:0007399,GO:0007186,	K08461|1|0.0|2430|mmu:78560|G protein-coupled receptor 124	gi|300934759|ref|NP_473385.2|	0	G-protein coupled receptor 124 precursor [Mus musculus]

mmu-let-7k	NM_080434	-26.7	0.465807	-26.33	159	GO:0034385,GO:0005543,GO:0070325,GO:0005539,GO:0060229,GO:0051004,GO:0044260,GO:0009888,GO:0010901,GO:0009887,GO:0009719,GO:0045807,GO:0010896,GO:0009896,GO:0010876,GO:0055092,GO:00	K09025|1|0.0|701|mmu:66113|apolipoprotein A-V	gi|31560003|ref|NP_536682.2|	0	apolipoprotein A-V precursor [Mus musculus]
mmu-let-7k	NM_134013	-27.1	0.788046	-89.33	629	GO:0016604,GO:0043234,GO:0043161,GO:0051320,GO:0007050,GO:0016070,GO:0051439,GO:0006520,GO:0006915,GO:0048869,GO:0048232,GO:0030330,	K06699|1|0.0|3494|mmu:103554|proteasome activator subunit 4	gi|117956381|ref|NP_598774.2|	0	proteasome activator complex subunit 4 [Mus musculus]

mmu-let-7k	NM_134069	-27.7	0.545459	-43.92	306	GO:0016021,GO:0005903,GO:0044459,GO:0044444,GO:0031090,GO:0008509,GO:0015081,GO:0005244,GO:0005342,GO:0006863,GO:0015893,GO:0015698,GO:0006082,GO:0043269,GO:0030001,	K12300|1|0.0|881|mmu:105355|MFS transporter, ACS family, solute carrier family 17 (sodium-dependent inorganic phosphate cotransporter), member 1/2/3/4	gi|259155326|ref|NP_598830.2|	0	solute carrier family 17 (sodium phosphate), member 3 isoform 1 [Mus musculus]
mmu-let-7k	NM_134078	-29	0.230798	-25.48	164	GO:0010008,GO:0022892,GO:0016192,GO:0007034,	K15053|1|0.0|721|mmu:105513|charged multivesicular body protein 7	gi|21359827|ref|NP_598839.2|	0	charged multivesicular body protein 7 [Mus musculus]

mmu-let-7k	NM_138656	-33.8	0.015602	-32.72	168	GO:0044439,GO:0016772,GO:0031072,GO:0042802,GO:0016831,GO:0032559,GO:0006796,GO:0006084,GO:0042221,GO:0006720,GO:0008283,GO:0008203,	K01597|1|0.0|721|mmu:192156|diphosphomevalonate decarboxylase [EC:4.1.1.33]	gi|256985114|ref|NP_619597.2|	0	diphosphomevalonate decarboxylase [Mus musculus]
mmu-let-7k	NM_138952	-28.2	0.220287	-27.65	153	-	K08846|1|0.0|1095|mmu:192656|receptor-interacting serine/threonine-protein kinase 2 [EC:2.7.11.1]	gi|20336736|ref|NP_620402.1|	0	receptor-interacting serine/threonine-protein kinase 2 [Mus musculus]

mmu-let-7k	NM_138955	-25.5	0.825446	-61.74	468	GO:0031224,GO:0017111,GO:0042802,GO:0046983,GO:0032559,GO:0030301,GO:0009207,	K05680|1|0.0|1188|mmu:192663|ATP-binding cassette, subfamily G (WHITE), member 4	gi|148693626|gb|EDL25573.1|	0	mCG141862, isoform CRA_c [Mus musculus]
mmu-let-7k	NM_144941	-27.1	0.551811	-25.01	154	GO:0044424,	K10433|1|7e-34|147|acs:100551816|microtubule-associated protein 7	gi|148698349|gb|EDL30296.1|	0	arginine/proline rich coiled-coil 1, isoform CRA_b [Mus musculus]
mmu-let-7k	NM_145066	-32.1	0.070077	-29.38	164	GO:0031224,GO:0004888,GO:0007166,	K04302|1|0.0|720|ssc:100736987|G protein-coupled receptor 85	gi|11560065|ref|NP_071590.1|	0	probable G-protein coupled receptor 85 [Rattus norvegicus]
mmu-let-7k	NM_145418	-27.1	0.274736	-25.72	153	GO:0031224,	-	gi|21703834|ref|NP_663393.1|	2.19E-168	uncharacterized protein C6orf72 homolog precursor [Mus musculus]
mmu-let-7k	NM_145456	-27.5	0.670944	-28.15	160	GO:0046914,	-	gi|210147530|ref|NP_663431.2|	0	zinc finger SWIM domain-containing protein 6 [Mus musculus]

mmu-let-7k	NM_146497	-31.8	0.015451	-26.94	165	GO:0016020,GO:0004888,GO:0007166,	K04257|1|5e-166|583|mmu:258490|olfactory	gi|33239116|ref|NP_666708.1|	3.24E-165	olfactory receptor 492 [Mus musculus]
mmu-let-7k	NM_146568	-30.2	0.033739	-25.87	169	GO:0004871,GO:0023052,	K04257|1|7e-168|590|mmu:258561|olfactory	gi|22129507|ref|NP_666779.1|	4.45E-167	olfactory receptor 1012 [Mus musculus]
mmu-let-7k	NM_001171512	-24.5	0.999998	-72.93	473	-	K12567|1|0.0|1030|mmu:22138|titin [EC:2.7.11.1]	gi|345842335|ref|NP_001164983.2|	0	obscurin isoform 1 [Mus musculus]
mmu-miR-100-3p	NM_001080814	-30.7	0.668316	-26.74	171	GO:0031224,GO:0032501,GO:0016337,	K06813|1|0.0|868|gga:423718|cadherin 23	gi|172046767|sp|Q8BNA6.2|FAT3_MOUSE	0	RecName: Full=Protocadherin Fat 3; AltName: Full=FAT tumor suppressor homolog 3; Flags: Precursor
mmu-miR-100-3p	NM_001081255	-29	0.51496	-25.62	176	-	K10130|1|7e-20|101|acs:100564298|leucine-rich repeats and death domain-containing protein	gi|124486939|ref|NP_001074724.1|	0	leucine-rich repeat and calponin homology domain-containing protein 3 precursor [Mus musculus]

mmu-miR-100-3p	NM_001195601	-31.3	0.005253	-27.72	179	-	K14014|1|1e-17|88.2|cfa:476890|small VCP/p97-interacting protein	gi|307548826|ref|NP_001182530.1|	1.28E-33	uncharacterized protein LOC100503036 [Mus musculus]
mmu-miR-100-3p	NM_007547	-31.1	0.174283	-27.83	179	-	K06551|1|0.0|909|mmu:19261|signal-regulatory protein	gi|148696296|gb|EDL28243.1|	0	signal-regulatory protein alpha, isoform CRA_c [Mus musculus]
mmu-miR-100-3p	NM_009481	-25.5	0.996761	-42.3	327	GO:0044424,GO:0070011,GO:0016790,GO:0046332,GO:0007276,GO:0019941,GO:0000087,GO:0007178,GO:0070646,	K11840|1|0.0|4960|mmu:22284|ubiquitin carboxyl-terminal hydrolase 9/24 [EC:3.1.2.15]	gi|115511018|ref|NP_033507.2|	0	probable ubiquitin carboxyl-terminal hydrolase FAF-X [Mus musculus]
mmu-miR-100-3p	NM_010517	-31.1	0.079926	-25.2	167	GO:0044421,GO:0019838,GO:0006952,GO:0048731,GO:0016049,GO:0044260,GO:0010740,GO:0006006,GO:0008283,	K10138|1|3e-28|127|bta:282261|insulin-like growth factor-binding protein 3	gi|6981086|ref|NP_034647.1|	1.37E-131	insulin-like growth factor-binding protein 4 precursor [Mus musculus]

mmu-miR-100-3p	NM_026283	-29.3	0.531398	-27.06	178	GO:0031224,GO:0006684,	K04714|1|9e-88|327|bfo:BRAFLDRAFT_104157|shingomyelin synthase [EC:2.7.8.27]	gi|21312864|ref|NP_080559.1|	0	sphingomyelin synthase-related protein 1 [Mus musculus]
mmu-miR-100-3p	NM_027442	-30.2	0.182287	-46.98	330	GO:0042579,GO:0015922,GO:0007620,GO:0006531,GO:0007610,	K00272|1|0.0|676|mmu:70503|D-aspartate oxidase [EC:1.4.3.1]	gi|148673010|gb|EDL04957.1|	0	D-aspartate oxidase, isoform CRA_a [Mus musculus]
mmu-miR-100-3p	NM_030732	-30.1	0.470093	-25.64	180	GO:0031981,GO:0005819,GO:0005515,GO:0003712,GO:0043565,GO:0000122,GO:0016055,GO:0006511,GO:0006629,GO:00063	K04508|1|0.0|973|mmu:81004|transducin (beta)-like 1	gi|31543001|ref|NP_109657.2|	0	F-box-like/WD repeat-containing protein TBL1XR1 [Mus musculus]

mmu-miR-100-3p	NM_133821	-28.3	0.633822	-25.08	166	GO:0043231,GO:0044444,GO:0043169,GO:0004721,GO:0048017,GO:0012501,GO:0048511,GO:0007169,	K01090|1|0.0|2610|mmu:98432|protein phosphatase [EC:3.1.3.16]	gi|257467468|ref|NP_598582.3|	0	PH domain leucine-rich repeat-containing protein phosphatase 1 [Mus musculus]
mmu-miR-100-3p	NM_138582	-31.8	0.143072	-27.46	184	-	-	gi|20147795|ref|NP_613048.1|	0	von Willebrand factor A domain-containing protein 7 precursor [Mus musculus]
mmu-miR-100-3p	NM_175234	-31	0.398167	-27.75	175	-	-	gi|167234386|ref|NP_780443.2|	0	failed axon connections homolog [Mus musculus]
mmu-miR-100-3p	NR_015615	-30.9	0.012226	-25.71	169	-	-	-	-	-
mmu-miR-100-3p	NR_024619	-29.6	0.130047	-25.74	180	-	-	gi|148681957|gb|EDL13904.1|	5.18E-39	RIKEN cDNA 2610001J05 [Mus musculus]
mmu-miR-100-3p	NM_148943	-20.1	1	-40.87	321	GO:0044424,GO:0070011,GO:0016790,GO:0046332,GO:0007276,GO:0019941,GO:0000087,GO:0007178,GO:0070646,	K11840|1|0.0|4999|mmu:107868|ubiquitin carboxyl-terminal hydrolase 9/24 [EC:3.1.2.15]	gi|120300980|ref|NP_683745.2|	0	ubiquitin specific peptidase 9, Y chromosome [Mus musculus]

mmu-miR-100-3p	NM_007495	-24.6	0.9957	-42.67	314	GO:0031224,GO:0016337,GO:0007610,GO:0048870,	-	gi|46488926|ref|NP_031521.2|	0	astrotactin-1 isoform 2 precursor [Mus musculus]
mmu-miR-100-3p	NR_033538	-27.9	0.108935	-20.93	184	-	-	gi|74146077|dbj|BAE24225.1|	1.19E-43	unnamed protein product [Mus musculus]
mmu-miR-100-3p	NM_010825	-27.9	0.59061	-24.84	180	GO:0043231,GO:0003712,GO:0001071,GO:0003677,GO:0006357,	K15613|1|0.0|773|aml:100465236|homeobox protein Meis 1	gi|148695936|gb|EDL27883.1|	0	myeloid ecotropic viral integration site-related gene 1, isoform CRA_b [Mus musculus]

mmu-miR-100-3p	NM_026081	-28.2	0.635538	-24.35	179	GO:0043231,GO:0005488,	K15176|1|3e-08|62.8|tca:657922|RNA polymerase-associated protein CTR9!K04574|2|2e-07|60.1|mcc:717705|neurofilament heavy polypeptide!K12893|3|3e-06|56.2|ecb:100070608|splicing factor, arginine/serine-rich 4/5/6	gi|26006165|dbj|BAC41425.1|	0	mKIAA0443 protein [Mus musculus]
mmu-miR-100-3p	NM_010889	-24.5	0.999999	-22.01	178	GO:0031674,	K11498|1|2e-07|62.0|ptr:461413|centromeric protein E	gi|148222065|ref|NP_035019.1|	0	nebulin [Mus musculus]
mmu-miR-100-3p	NM_146599	-27.4	0.178007	-23.29	176	GO:0004871,GO:0023052,	K04257|1|1e-164|579|mmu:258592|olfactory	gi|22129455|ref|NP_666810.1|	9.42E-164	olfactory receptor 697 [Mus musculus]

mmu-miR-100-3p	NM_001033416	-24.9	0.894476	-24.24	175	GO:0000139,GO:0031224,GO:0030554,GO:0050694,GO:0005975,GO:0006029,GO:00071	K09677|1|0.0|904|mmu:330217|galactose-3-O-sulfotransferase 4 [EC:2.8.2.-]	gi|84370027|ref|NP_001028588.1|	0	galactose-3-O-sulfotransferase 4 [Mus musculus]
mmu-miR-100-3p	NM_145998	-27.3	0.310264	-23.21	174	GO:0043231,GO:0001071,GO:0003677,GO:0007417,GO:0048869,GO:0042471,GO:0006351,GO:0008283,	K09349|1|5e-141|501|mmu:15372|homeobox protein Nkx-5	gi|70608207|ref|NP_666110.1|	3.60E-140	homeobox protein HMX2 [Mus musculus]
mmu-miR-100-3p	NM_015796	-27.3	0.366993	-23.53	173	GO:0031461,GO:0005515,GO:0009057,	K10101|1|3e-164|579|mmu:50760|F-box protein	gi|171543844|ref|NP_056611.2|	2.21E-163	F-box only protein 17 [Mus musculus]
mmu-miR-100-3p	NR_033123	-27.9	0.584814	-23	172	-	-	gi|49117662|gb|AAH72647.1|	0	RIKEN cDNA 4933409K07 gene [Mus musculus]
mmu-miR-100-3p	NM_178695	-20.6	0.999999	-20.11	171	GO:0031224,GO:0043231,GO:0046872,	K01320|1|5e-10|67.8|hsa:2155|coagulation factor VII [EC:3.4.21.21]	gi|224994242|ref|NP_848810.3|	7.87E-120	transmembrane gamma-carboxyglutamic acid protein 4 precursor [Mus musculus]

mmu-miR-100-3p	NM_198006	-25.8	0.842076	-21.34	170	GO:0043231,	-	gi|113461996|ref|NP_932123.3|	8.94E-37	cytochrome c oxidase assembly factor 5 [Mus musculus]
mmu-miR-100-3p	NR_040391	-28	0.263953	-22.5	169	-	-	gi|148675669|gb|EDL07616.1|	7.01E-55	mCG145090 [Mus musculus]
mmu-miR-100-3p	NM_181040	-23.2	0.972062	-20.13	168	GO:0043231,GO:0005488,GO:0065003,	K07555|1|2e-176|620|mmu:230649|ATP synthase mitochondrial F1 complex assembly factor 1	gi|344925843|ref|NP_851383.2|	4.24E-177	ATP synthase mitochondrial F1 complex assembly factor 1 [Mus musculus]
mmu-miR-100-3p	NM_199419	-24.5	0.649205	-21.62	167	GO:0035036,	-	gi|148684200|gb|EDL16147.1|	1.92E-151	zona pellucida binding protein 2, isoform CRA_a [Mus musculus]
mmu-miR-100-3p	NR_033209	-24.5	0.434885	-21.67	166	-	-	gi|62185789|gb|AAH92352.1|	5.87E-100	Predicted gene, EG215472 [Mus musculus]

mmu-miR-100-3p	NM_198703	-26.6	0.96493	-23.12	165	GO:0044424,GO:0004860,GO:0004672,GO:0019900,GO:0046872,GO:0008200,GO:0032559,GO:0006810,GO:0035556,GO:0006469,GO:0048468,GO:0003073,	K08867|1|0.0|2065|ptr:451739|WNK lysine deficient protein kinase [EC:2.7.11.1]	gi|148667213|gb|EDK99629.1|	0	WNK lysine deficient protein kinase 1, isoform CRA_b [Mus musculus]
mmu-miR-100-3p	NM_178687	-24.2	0.911266	-20.7	164	GO:0016021,GO:0004871,GO:0005102,GO:0019900,GO:0002420,GO:0023052,GO:0002696,GO:0001912,GO:0002714,GO:00325	K06567|1|3e-159|563|rno:307199|CD226 antigen	gi|85540456|ref|NP_848802.2|	3.75E-179	CD226 antigen isoform a precursor [Mus musculus]

mmu-miR-100-3p	NM_198295	-20.9	1	-23.94	163	GO:0031090,GO:0043231,GO:0031224,GO:0016862,GO:0015036,GO:0018904,GO:0019725,	K09585|1|0.0|843|rno:682967|thioredoxin domain-containing protein 10 [EC:5.3.4.1]	gi|37360566|dbj|BAC98261.1|	0	mKIAA1830 protein [Mus musculus]
mmu-miR-100-3p	NR_045071	-23.1	0.999267	-20.28	162	-	-	gi|1791243|gb|AAB41224.1|	7.63E-09	ORF2 [Rattus norvegicus]
mmu-miR-100-3p	NR_046291	-24.5	0.877044	-20.12	161	-	K11502|1|8e-70|266|ecb:100049819|centromere protein J	gi|61098085|ref|NP_035683.2|	0	t-complex protein 10a [Mus musculus]
mmu-miR-100-3p	NR_015562	-26.2	0.687937	-20.49	160	-	-	gi|148705285|gb|EDL37232.1|	2.57E-49	mCG146320 [Mus musculus]
mmu-miR-100-3p	NR_027870	-25.7	0.698218	-21.53	159	GO:0044424,GO:0046914,	K10366|1|4e-50|200|hmg:100213780|dystrophin	gi|148669448|gb|EDL01395.1|	0	dystrobrevin, beta, isoform CRA_e [Mus musculus]
mmu-miR-100-3p	NR_028403	-21.6	0.998266	-22.16	158	GO:0043231,GO:0044444,GO:0019783,GO:0005515,GO:0048731,GO:0019941,	K14016|1|2e-162|515|mmu:22230|ubiquitin fusion degradation protein 1	gi|257153392|ref|NP_035802.3|	1.72E-161	ubiquitin fusion degradation protein 1 homolog [Mus musculus]

mmu-miR-100-3p	NR_033326	-24.9	0.276293	-20.88	157	-	-	gi|148668293|gb|EDL00623.1|	4.58E-49	mCG144908 [Mus musculus]
mmu-miR-100-3p	NM_145400	-25.9	0.934227	-21.38	156	GO:0043234,GO:0044424,GO:0005488,GO:0004842,GO:0019941,GO:0016567,	K10596|1|0.0|2026|mmu:140630|ubiquitin conjugation factor E4 A [EC:6.3.2.19]	gi|50510371|dbj|BAD32171.1|	0	mKIAA0126 protein [Mus musculus]
mmu-miR-100-3p	NM_207208	-22.5	0.998002	-20.08	155	GO:0044459,GO:0016021,GO:0005253,GO:0015698,	K05030|1|0.0|1785|mmu:99663|calcium-activated chloride channel family member 4	gi|110556625|ref|NP_997091.3|	0	calcium-activated chloride channel regulator 4 precursor [Mus musculus]
mmu-miR-100-3p	NM_175417	-21.7	0.999385	-20.04	154	GO:0031224,	-	gi|30425128|ref|NP_780626.1|	2.24E-152	androgen-dependent TFPI-regulating protein isoform 1 [Mus musculus]
mmu-miR-100-3p	NM_177394	-21.3	1	-21.28	153	GO:0004518,GO:0006259,	K03849|1|3e-18|95.9|cel:C08H9.3|alpha-1,3-glucosyltransferase [EC:2.4.1.265]	gi|257467544|ref|NP_001158108.1|	0	endonuclease V isoform 1 [Mus musculus]

mmu-miR-100-3p	NR_045189	-20.5	1	-20.77	152	GO:0043231,GO:0003676,GO:0046914,GO:0001071,GO:0006357,	K11307|1|3e-06|56.2|spu:577009|histone acetyltransferase MYST2 [EC:2.3.1.48]	gi|28972263|dbj|BAC65585.1|	0	mKIAA0535 protein [Mus musculus]
mmu-miR-100-3p	NR_001461	-27.3	0.999994	-20.16	151	-	K11849|1|6e-55|213|acs:100555596|ubiquitin carboxyl-terminal hydrolase 25/28 [EC:3.1.2.15]!K06478|4|4e-43|182|acs:100563672|protein tyrosine phosphatase, receptor type, C [EC:3.1.3.4	gi|209553980|gb|AAA39398.2|	0	ORF2 [Mus musculus domesticus]

mmu-miR-100-3p	NM_130865	-23.3	0.996748	-23.59	150	GO:0043234,GO:0042802,GO:0001071,GO:0003677,GO:0046983,GO:0007417,GO:0006357,GO:0040007,GO:0007626,GO:0007631,	K09324|1|3e-17|92.0|bfo:BRAFLDRAFT_116854|homeobox protein goosecoid!K09329|2|8e-17|90.9|bfo:BRAFLDRAFT_204029|paired mesoderm homeobox protein!K09451|3|3e-16|89.0|mdo:100013656|homeobox protein aristaless-	gi|70909343|ref|NP_570935.3|	2.04E-148	diencephalon/mesencephalon homeobox protein 1 isoform a [Mus musculus]
mmu-miR-100-3p	NM_024475	-20.9	0.999927	-20.69	149	GO:0043231,GO:0016791,GO:0006796,	K01090|1|5e-166|585|mmu:79560|protein phosphatase [EC:3.1.3.16]	gi|46575895|ref|NP_077795.2|	3.40E-165	ubiquitin-like domain-containing CTD phosphatase 1 [Mus musculus]

mmu-miR-100-3p	NR_045419	-24.7	0.747994	-20.42	148	-	-	gi|148677827|gb|EDL09774.1|	4.21E-44	mCG144594 [Mus musculus]
mmu-miR-100-3p	NR_033514	-22.1	0.999986	-20.63	147	-	-	gi|148670710|gb|EDL02657.1|	2.17E-19	mCG63843, isoform CRA_b [Mus musculus]
mmu-miR-100-3p	NM_026984	-22.6	0.999996	-22.34	146	GO:0035097,GO:0005515,GO:0018024,GO:0003712,GO:0046872,GO:0002274,GO:0032526,GO:0006355,GO:0002763,GO:0006305,GO:0002444,GO:0030522,GO:0034968,GO:00	K09189|1|0.0|3114|mmu:69188|histone-lysine N-methyltransferase MLL5 [EC:2.1.1.43]	gi|262527254|ref|NP_081260.1|	0	histone-lysine N-methyltransferase MLL5 [Mus musculus]
mmu-miR-100-3p	NM_008136	-21	0.999987	-20.93	145	GO:0044421,GO:0044464,GO:0032561,GO:0002449,GO:0033554,GO:0023052,	K14539|1|1e-41|172|acs:100556150|large subunit GTPase 1 [EC:3.6.1.-]	gi|122937361|ref|NP_032162.2|	0	guanine nucleotide-binding protein-like 1 [Mus musculus]

mmu-miR-100-3p	NM_198599	NA	NA	-23.17	158	GO:0043232,GO:0016023,GO:0044431,GO:0015631,GO:0006500,	K10432|1|5e-09|64.7|aml:100477292|microtubule-associated protein 6	gi|74201189|dbj|BAE37442.1|	1.63E-108	unnamed protein product [Mus musculus]
mmu-miR-100-3p	NM_016704	NA	NA	-20.2	152	GO:0044421,GO:0005887,GO:0005488,GO:0002699,GO:0002455,GO:0001905,GO:0043009,GO:0006915,GO:0012502,GO:0045765,GO:0006952,	K03995|1|0.0|1624|mmu:12274|complement component 6	gi|161086891|ref|NP_057913.2|	0	complement component 6 precursor [Mus musculus]

mmu-miR-100-3p	NM_011504	NA	NA	-20.11	151	GO:0031981,GO:0030141,GO:0044459,GO:0019905,GO:0007599,GO:0002283,GO:0007417,GO:0034109,GO:0022406,GO:0008645,GO:0043434,GO:0051259,GO:0030072,GO:00	K15301|1|0.0|1193|mmu:20912|syntaxin-binding protein 3	gi|6755690|ref|NP_035634.1|	0	syntaxin-binding protein 3 [Mus musculus]
mmu-miR-100-3p	NM_026565	-24.5	0.536691	NA	NA	GO:0019866,GO:0043231,	-	gi|13386062|ref|NP_080841.1|	8.19E-138	apolipoprotein O-like precursor [Mus musculus]
mmu-miR-100-3p	NM_001254754	-28.9	0.112859	NA	NA	GO:0043231,GO:0003824,GO:0044238,GO:0032787,	K05607|1|2e-70|266|tgu:100231431|methylglutaconyl-CoA hydratase [EC:4.2.1.18]	gi|74150361|dbj|BAE32227.1|	1.07E-135	unnamed protein product [Mus musculus]
mmu-miR-100-3p	NM_001163589	-22.9	0.956054	NA	NA	GO:0030424,GO:0005882,GO:0044297,GO:0005488,GO:0007010,	K07607|1|1e-178|627|rno:24688|peripherin	gi|254675339|ref|NP_001157061.1|	0	peripherin isoform 3 [Mus musculus]

mmu-miR-100-3p	NM_013518	-21.2	1	NA	NA	GO:0044424,GO:0005578,GO:0070851,GO:0005539,GO:0001501,GO:0010467,GO:0060485,GO:0006605,GO:0035108,GO:0008406,GO:0001503,GO:0040034,GO:0007169,GO:0008284,GO:0010740,GO:0030323,GO:0042471,GO:0030154,GO:0048565,GO:0008589,GO:0048646,GO:00301	K04358|1|1e-109|399|rno:25444|fibroblast growth factor	gi|1706792|sp|P54130.1|FGF9_MOUSE	9.47E-109	RecName: Full=Fibroblast growth factor 9; Short=FGF-9; AltName: Full=Glia-activating factor; Short=GAF; AltName: Full=HBGF-9; Flags: Precursor
mmu-miR-100-3p	NM_011650	-25.3	0.836317	NA	NA	GO:0043231,GO:0043566,GO:0003723,GO:0005515,GO:0006259,	-	gi|21730459|pdb|1KEY|A	2.02E-113	Chain A, Crystal Structure Of Mouse TestisBRAIN RNA-Binding Protein (Tb-Rbp)

mmu-miR-100-3p	NM_027908	-23	0.982869	NA	NA	GO:0044444,GO:0043169,GO:0016812,GO:0006536,GO:0006547,	K01468|1|0.0|803|mmu:71761|imidazolonepropionase [EC:3.5.2.7]	gi|254675292|ref|NP_082184.1|	0	probable imidazolonepropionase [Mus musculus]
mmu-miR-100-3p	NM_197945	-20.6	1	NA	NA	GO:0030425,GO:0044430,GO:0016020,GO:0019904,GO:0051259,	-	gi|161086990|ref|NP_922936.3|	0	proSAP-interacting protein 1 [Mus musculus]
mmu-miR-100-3p	NM_001145897	-21.8	1	NA	NA	GO:0005488,	-	gi|225543323|ref|NP_941073.2|	0	genetic suppressor element 1 isoform 1 [Mus musculus]
mmu-miR-100-3p	NM_170669	-23.6	0.998003	NA	NA	GO:0043231,GO:0015935,GO:0003676,GO:0005198,GO:0006412,GO:0019080,GO:0007417,GO:0051707,GO:0051726,GO:0031016,GO:0008283,	K02957|1|1e-68|263|ptr:100614776|small subunit ribosomal protein S15Ae	gi|30109302|gb|AAH51205.1|	3.76E-70	Rps15a protein [Mus musculus]

mmu-miR-100-3p	NM_001007222	-25	0.507304	NA	NA	-	K10523|1|9e-114|410|acs:100564527|speckle-type POZ protein	gi|124126981|ref|NP_001007223.2|	0	TD and POZ domain-containing protein 2 [Mus musculus]
mmu-miR-100-3p	NM_026846	-21.2	0.992574	NA	NA	GO:0043169,	K07059|1|7e-14|79.0|hsa:79752|	gi|229577434|ref|NP_081122.2|	9.02E-103	AN1-type zinc finger protein 2B [Mus musculus]
mmu-miR-100-3p	NM_146245	-20.1	1	NA	NA	GO:0031227,	K06838|1|6e-15|84.7|mmu:20562|slit 1!K06850|5|1e-14|83.6|xtr:780195|slit 3	gi|148692949|gb|EDL24896.1|	0	leucine rich repeat containing 21 [Mus musculus]
mmu-miR-100-3p	NM_001159485	-22.8	0.999848	NA	NA	GO:0031252,GO:0044444,GO:0016020,GO:0005088,GO:0035091,GO:0006917,GO:0007266,GO:0009966,GO:0007169,	K15048|1|3e-56|222|tgu:100232281|kalirin [EC:2.7.11.1]	gi|226958677|ref|NP_001152957.1|	0	guanine nucleotide exchange factor DBS isoform 3 [Mus musculus]

mmu-miR-100-3p	NM_001093778	-20.6	1	NA	NA	GO:0043231,GO:0046914,GO:0003676,GO:0001071,GO:0006351,GO:0032501,GO:004886 9,	K11307|1|7e-08|62.0|acs:100564728|histone acetyltransferase MYST2 [EC:2.3.1.4	gi|148271075|ref|NP_032692.2|	0	myelin transcription factor 1-like protein isoform 2 [Mus musculus]
mmu-miR-100-3p	NM_207682	-21.9	1	NA	NA	GO:0012506,GO:0043231,GO:0015630,GO:0030246,GO:0016616,GO:0032559,GO:0003774,GO:0015631,GO:0006098,GO:0007275,GO:0012501,GO:0009207,GO:0019520,GO:0007268,GO:0034643,GO:00080	K10392|1|0.0|3154|ecb:100057126|kinesin family member 1/13/14	gi|86990460|ref|NP_997565.2|	0	kinesin-like protein KIF1B isoform b [Mus musculus]

mmu-miR-100-3p	NM_177271	-22.1	0.998153	NA	NA	-	K14079|1|4e-22|107|dwi:Dwil_GK14246|poly(A) RNA polymerase GLD2 [EC:2.7.7.19]!K05106|5|8e-06|53.5|rno:79208|Eph receptor A5 [EC:2.7.10.1]	gi|110556634|ref|NP_796245.2|	7.06E-85	sterile alpha motif domain-containing protein 5 [Mus musculus]
mmu-miR-100-3p	NM_153804	-21.6	0.999999	NA	NA	GO:0044464,GO:0005088,GO:0007266,	K05769|1|7e-31|137|acs:100562417|Rho guanine nucleotide exchange factor 4	gi|167621502|ref|NP_722499.4|	0	pleckstrin homology domain-containing family G member 3 [Mus musculus]
mmu-miR-100-3p	NM_178777	-21.9	0.999871	NA	NA	GO:0009987,GO:0007618,GO:0048511,GO:0007399,	K09075|1|2e-37|159|cfa:483135|nescient helix-loop-helix	gi|30520253|ref|NP_848892.1|	1.30E-36	helix-loop-helix protein 2 [Mus musculus]
mmu-miR-100-3p	NR_038022	-20.7	1	NA	NA	-	-	gi|148671508|gb|EDL03455.1|	7.75E-43	mCG144972 [Mus musculus]

mmu-miR-100-3p	NM_028773	-29.1	0.240502	NA	NA	GO:0043231,GO:0002369,GO:0030888,GO:0002377,GO:0001819,GO:0002696,GO:0045927,GO:0043370,GO:0042098,GO:00018	K10356|1|2e-06|55.5|hmg:100211911|myosin I	gi|21311907|ref|NP_083049.1|	0	SAM and SH3 domain-containing protein 3 [Mus musculus]
mmu-miR-100-3p	NM_008415	-21.4	1	NA	NA	GO:0044427,GO:0043231,GO:0030529,GO:0003723,GO:0006351,	K11496|1|1e-19|100|oaa:100077513|centromere protein B	gi|153792154|ref|NP_032441.4|	0	jerky protein [Mus musculus]
mmu-miR-100-3p	NM_001081453	-20.8	1	NA	NA	-	K00688|1|5e-163|577|tgu:100231603|starch phosphorylase [EC:2.4.1.1]!K11499|3|5e-28|129|spu:592172|centromere protein F	gi|125346101|ref|NP_001074922.1|	0	ninein isoform 1 [Mus musculus]

mmu-miR-100-3p	NM_001168476	-20.6	0.999969	NA	NA	-	-	gi|270309132|ref|NP_001161947.1|	0	tetratricopeptide repeat protein 23 isoform 2 [Mus musculus]
mmu-miR-100-3p	NM_001252479	-22.3	0.911923	NA	NA	GO:0031224,GO:0044421,GO:0005102,GO:0042221,	-	gi|74198935|dbj|BAE30687.1|	8.46E-104	unnamed protein product [Mus musculus]
mmu-miR-100-3p	NM_001013024	-20.9	1	NA	NA	GO:0019783,GO:0004175,GO:0046914,GO:0008238,GO:0016790,GO:0019941,	K11836|1|0.0|1649|mmu:72607|ubiquitin carboxyl-terminal hydrolase 5/13 [EC:3.1.2.15]	gi|61316410|ref|NP_001013042.1|	0	ubiquitin carboxyl-terminal hydrolase 13 [Mus musculus]
mmu-miR-100-3p	NM_001081239	-21.9	0.997261	NA	NA	-	K06512|1|4e-60|233|ptr:450147|leukocyte immunoglobulin-like receptor	gi|156766061|ref|NP_001074708.2|	7.37E-165	leukocyte immunoglobulin-like receptor precursor [Mus musculus]

mmu-miR-100-3p	NM_001111017	-24.7	0.985195	NA	NA	GO:0005789,GO:0044421,GO:0031224,GO:0016787,GO:0043062,GO:0046488,GO:0015031,	K01099|1|1e-32|144|mmu:20975|phosphatidylinositol-bisphosphatase [EC:3.1.3.36]	gi|161169010|ref|NP_001104487.1|	0	protein SERAC1 isoform 3 [Mus musculus]
mmu-miR-100-3p	NM_172463	-21.6	0.999998	NA	NA	GO:0046872,GO:0031589,	K02599|1|6e-145|516|aga:AgaP_AGAP001015|Notc	gi|153791169|ref|NP_766051.4|	0	sushi, nidogen and EGF-like domain-containing protein 1 precursor [Mus musculus]
mmu-miR-100-3p	NM_025884	-21.2	0.999994	NA	NA	GO:0031981,GO:0043169,GO:0001824,GO:0000087,	K13104|1|0.0|659|mmu:66983|zinc finger protein	gi|31560213|ref|NP_080160.2|	0	zinc finger protein 830 [Mus musculus]

mmu-miR-100-3p	NM_021483	-22.5	0.99856	NA	NA	GO:0030427,GO:0043005,GO:0034703,GO:0044439,GO:0005217,GO:0005083,GO:0000268,GO:0006887,GO:0046887,GO:0051235,GO:0009966,GO:0006811,GO:0019222,GO:0006873,GO:0006625,	K13342|1|0.0|883|tgu:100222110|peroxin-5	gi|254588036|ref|NP_067458.2|	0	PEX5-related protein isoform 1 [Mus musculus]
mmu-miR-100-3p	NM_020597	-21.3	0.834413	NA	NA	GO:0005576,	-	gi|10181104|ref|NP_065622.1|	4.90E-66	beta-microseminoprotein precursor [Mus musculus]

mmu-miR-100-3p	NM_007560	-24	0.9956	NA	NA	GO:0016021,GO:0043234,GO:0005515,GO:0005024,GO:0032559,GO:0043169,GO:0007411,GO:0035107,GO:0007178,GO:0045597,GO:0006694,GO:0016337,GO:0001547,GO:0006796,GO:0003002,GO:0030282,GO:0001654,GO:0006464,GO:00069	K13578|1|0.0|1013|mmu:12167|bone morphogenetic protein receptor type-1B [EC:2.7.11.30]	gi|6680802|ref|NP_031586.1|	0	bone morphogenetic protein receptor type-1B precursor [Mus musculus]
mmu-miR-100-3p	NM_001001885	-23.3	0.99655	NA	NA	GO:0031224,	-	gi|49457865|ref|NP_001001885.1|	0	transmembrane protein 151A [Mus musculus]

mmu-miR-100-3p	NM_001079932	-20.3	0.999999	NA	NA	GO:0012506,GO:0005886,GO:0046914,GO:0005543,GO:0016192,GO:0051259,GO:0007009,GO:0003008,GO:0048513,	K12036|1|0.0|931|mmu:434246|tripartite motif-containing protein 72	gi|121247302|ref|NP_001073401.1|	0	tripartite motif-containing protein 72 [Mus musculus]
mmu-miR-100-3p	NM_001252444	-22.1	0.997271	NA	NA	GO:0043234,GO:0031981,GO:0044431,GO:0016859,GO:0004842,GO:0002376,GO:0016567,GO:0007599,	K10598|1|0.0|1014|mmu:66053|peptidyl-prolyl cis-trans isomerase-like 2 [EC:5.2.1.8]	gi|26349387|dbj|BAC38333.1|	0	unnamed protein product [Mus musculus]
mmu-miR-100-3p	NM_001080819	-20.2	1	NA	NA	GO:0031981,GO:0070603,GO:0003676,GO:0048731,GO:0034728,GO:0010467,GO:0006338,GO:0031958,	K11653|1|0.0|2794|mmu:93760|AT-rich interactive domain-containing protein 1	gi|124249109|ref|NP_001074288.1|	0	AT-rich interactive domain-containing protein 1A [Mus musculus]

mmu-miR-100-3p	NM_008140	-22.7	0.986353	NA	NA	GO:0001750,GO:0031234,GO:0044297,GO:0032561,GO:0005102,GO:0017111,GO:0060089,GO:0032403,GO:0019900,GO:0051602,GO:0050953,GO:0050912,GO:0009207,GO:0051342,GO:0007603,GO:0007187,GO:00	K04631|1|0.0|670|mmu:14685|guanine nucleotide-binding protein G(t) subunit alpha	gi|6680041|ref|NP_032166.1|	0	guanine nucleotide-binding protein G(t) subunit alpha-1 [Mus musculus]
mmu-miR-100-3p	NR_073190	-20.1	1	NA	NA	-	-	-	-	-

mmu-miR-100-3p	NM_010927	-22	0.999952	NA	NA	GO:0042579,GO:0005856,GO:0000166,GO:0016597,GO:0050662,GO:0042802,GO:0005506,GO:0016709,GO:0019887,GO:0032496,GO:0009611,GO:0048771,GO:0033280,GO:0050673,GO:0008015,GO:0044057,GO:0016310,GO:0043627,GO:0042742,GO:0022602,GO:0044271,GO:00170	K13241|1|0.0|2334|mmu:18126|nitric-oxide synthase, inducible [EC:1.14.13.39]	gi|6754872|ref|NP_035057.1|	0	nitric oxide synthase, inducible [Mus musculus]
mmu-miR-100-3p	NM_001141921	-28.7	0.280777	NA	NA	GO:0044459,GO:0031224,GO:0044430,	K07523|1|6e-30|133|xtr:100188921|netrin-G1 ligand	gi|213972562|ref|NP_001135393.1|	0	leucine-rich repeat and fibronectin type III domain-containing protein 1 isoform 1 precursor [Mus musculus]

mmu-miR-100-3p	NM_026380	-20	1	NA	NA	GO:0044424,GO:0016020,GO:0008047,GO:0060089,GO:0043087,GO:0007166,	K07524|1|8e-44|181|mdo:100009962|regulator of G-protein signalling 3	gi|40789289|ref|NP_080656.2|	3.04E-92	regulator of G-protein signaling 8 [Mus musculus]
mmu-miR-100-3p	NR_045941	-20.2	0.992628	NA	NA	-	-	gi|148704600|gb|EDL36547.1|	7.79E-41	mCG1041734 [Mus musculus]
mmu-miR-100-3p	NM_001037758	-20.8	1	NA	NA	GO:0043231,GO:0031461,GO:0044444,GO:0019787,GO:0005488,GO:0007166,GO:0043161,GO:0031647,GO:0030162,GO:0051439,GO:0051704,	K03362|1|0.0|1249|mmu:12234|F-box and WD-40 domain protein 1/11	gi|60360248|dbj|BAD90368.1|	0	mKIAA4123 protein [Mus musculus]
mmu-miR-100-3p	NM_001081400	-22.5	0.999708	NA	NA	-	-	gi|145587086|ref|NP_001074869.1|	3.63E-129	UPF0472 protein C16orf72 homolog [Mus musculus]
mmu-miR-100-3p	NR_027975	-20.1	1	NA	NA	-	-	gi|42557306|gb|AAH66076.1|	1.64E-110	Predicted gene, EG434280 [Mus musculus]

mmu-miR-100-5p	NM_001033262	-29.2	0.248615	-26.66	151	GO:0044431,GO:0016020,	K10659|1|4e-128|443|ptr:452026|E3 ubiquitin-protein ligase MARCH4/9/11 [EC:6.3.2.19]	gi|395835542|ref|XP_003790736.1|	4.94E-130	PREDICTED: E3 ubiquitin-protein ligase MARCH9, partial [Otolemur garnettii]
mmu-miR-100-5p	NM_001033542	-26.4	0.695097	-26.94	159	GO:0016020,GO:0015299,GO:0015893,GO:0006818,GO:0006812,	K03327|1|0.0|995|mmu:380701|multidrug resistance protein, MATE family	gi|85702045|ref|NP_001028714.1|	0	multidrug and toxin extrusion protein 2 [Mus musculus]
mmu-miR-100-5p	NM_001043335	-32.8	0.137256	-28.32	164	GO:0015630,GO:0046872,	K15420|1|0.0|920|gga:771345|echinoderm microtubule-associated protein-like 4	gi|112807168|ref|NP_001036800.1|	0	echinoderm microtubule-associated protein-like 1 isoform 1 [Mus musculus]

mmu-miR-100-5p	NM_001043336	-32.8	0.122415	-28.32	164	GO:0015630,GO:0046872,	K15420|1|0.0|908|gga:771345|echinoderm microtubule-associated protein-like 4	gi|223462311|gb|AAI50976.1|	0	Eml1 protein [Mus musculus]
mmu-miR-100-5p	NM_001130150	-26.1	0.860802	-47.62	334	GO:0016020,GO:0044444,GO:0005088,GO:0008047,GO:0005488,GO:0043087,GO:0007266,GO:0031346,GO:0010769,GO:0007169,GO:0031345,	K12330|1|0.0|1680|mmu:16801|Rho guanine nucleotide exchange factor 1	gi|148692356|gb|EDL24303.1|	0	Rho guanine nucleotide exchange factor (GEF) 1, isoform CRA_b [Mus musculus]

mmu-miR-100-5p	NM_001130151	-26.1	0.8601	-47.62	334	GO:0016020,GO:0044444,GO:0005088,GO:0008047,GO:0005488,GO:0043087,GO:0007266,GO:0031346,GO:0010769,GO:0007169,GO:0031345,	K12330|1|0.0|1659|mmu:16801|Rho guanine nucleotide exchange factor 1	gi|148692356|gb|EDL24303.1|	0	Rho guanine nucleotide exchange factor (GEF) 1, isoform CRA_b [Mus musculus]
mmu-miR-100-5p	NM_001130152	-26.1	0.847042	-47.62	334	GO:0016020,GO:0044444,GO:0005088,GO:0008047,GO:0005488,GO:0043087,GO:0007266,GO:0031346,GO:0010769,GO:0007169,GO:0031345,	K12330|1|0.0|1545|rno:60323|Rho guanine nucleotide exchange factor 1	gi|148692357|gb|EDL24304.1|	0	Rho guanine nucleotide exchange factor (GEF) 1, isoform CRA_c [Mus musculus]

mmu-miR-100-5p	NM_001130153	-26.1	0.846264	-47.62	334	GO:0016020,GO:0044444,GO:0005088,GO:0008047,GO:0005488,GO:0043087,GO:0007266,GO:0031346,GO:0010769,GO:0007169,GO:0031345,	K12330|1|0.0|1545|rno:60323|Rho guanine nucleotide exchange factor 1	gi|148692358|gb|EDL24305.1|	0	Rho guanine nucleotide exchange factor (GEF) 1, isoform CRA_d [Mus musculus]
mmu-miR-100-5p	NM_008488	-26.1	0.846003	-47.62	334	GO:0016020,GO:0044444,GO:0005088,GO:0008047,GO:0005488,GO:0043087,GO:0007266,GO:0031346,GO:0010769,GO:0007169,GO:0031345,	K12330|1|0.0|1549|rno:60323|Rho guanine nucleotide exchange factor 1	gi|148692358|gb|EDL24305.1|	0	Rho guanine nucleotide exchange factor (GEF) 1, isoform CRA_d [Mus musculus]

mmu-miR-100-5p	NM_011980	-32	0.08193	-42.85	302	GO:0031981,GO:0046914,GO:0003676,GO:0005539,GO:0006351,	K09228|1|3e-161|569|ssc:100525521|KRAB domain-containing zinc finger protein	gi|392337664|ref|XP_001078904.3|	1.33E-179	PREDICTED: zinc finger protein OZF-like [Rattus norvegicus]
mmu-miR-100-5p	NM_013500	-29.4	0.522416	-25.32	167	GO:0031012,GO:0005539,GO:0009987,	K06848|1|0.0|755|mmu:12950|hyaluronan and proteoglycan link protein 1	gi|162138936|ref|NP_038528.3|	0	hyaluronan and proteoglycan link protein 1 precursor [Mus musculus]
mmu-miR-100-5p	NM_015729	-27.6	0.710615	-25.96	148	GO:0044439,GO:0003997,GO:0008289,GO:0005515,GO:0050662,GO:0006692,GO:0048232,GO:0006635,	K00232|1|0.0|1335|mmu:11430|acyl-CoA oxidase [EC:1.3.3.6]	gi|66793429|ref|NP_056544.2|	0	peroxisomal acyl-coenzyme A oxidase 1 [Mus musculus]

mmu-miR-100-5p	NM_019549	-27.8	0.685282	-25.69	165	GO:0001726,GO:0044444,GO:0019901,GO:0035091,GO:0042802,GO:0030097,GO:0007200,GO:0007243,GO:0010543,GO:0051017,GO:0034109,GO:0009306,GO:0009968,GO:0006887,GO:0006650,GO:0030030,GO:0008361,GO:0031334,GO:0010922,GO:0030042,GO	K06082|1|2e-06|56.6|cin:100177029|FERM, RhoGEF and pleckstrin domain protein 2	gi|11464971|ref|NP_062422.1|	0	pleckstrin [Mus musculus]
mmu-miR-100-5p	NM_022814	-30.6	0.637016	-25.65	169	GO:0044424,GO:0046872,GO:0009987,	K04011|1|0.0|640|ecb:100057298|complement component (3b/4b) receptor 1	gi|124783268|ref|NP_073725.2|	0	sushi, von Willebrand factor type A, EGF and pentraxin domain-containing protein 1 precursor [Mus musculus]

mmu-miR-100-5p	NM_031377	-34	0.044115	-31.71	179	-	-	gi|13878197|ref|NP_113554.1|	0	preferentially expressed antigen in melanoma-like 1 [Mus musculus]
mmu-miR-100-5p	NM_144880	-26.6	0.759858	-25.54	157	GO:0043231,GO:0044427,GO:0008287,GO:0019888,GO:0019899,GO:0048523,GO:0006470,GO:0023052,GO:0033673,	K11584|1|0.0|757|mmu:226849|protein phosphatase 2 (formerly 2A), regulatory subunit B'	gi|47059051|ref|NP_659129.2|	0	serine/threonine-protein phosphatase 2A 56 kDa regulatory subunit alpha isoform [Mus musculus]
mmu-miR-100-5p	NM_153388	-27.8	0.555933	-26.19	156	GO:0031224,	K07523|1|2e-25|118|oaa:100073707|netrin-G1	gi|31559904|ref|NP_700437.2|	0	leucine-rich repeat and fibronectin type-III domain-containing protein 4 precursor [Mus musculus]
mmu-miR-100-5p	NM_172551	-32.5	0.137856	-30.55	166	GO:0005759,GO:0034062,GO:0003676,GO:0006352,	K10908|1|0.0|2251|mmu:216151|DNA-directed RNA polymerase, mitochondrial [EC:2.7.7.6]	gi|281332113|ref|NP_766139.2|	0	DNA-directed RNA polymerase, mitochondrial precursor [Mus musculus]

mmu-miR-100-5p	NM_177006	-27.3	0.939456	-26.28	151	-	K11127|1|3e-13|80.1|bfo:BRAFLDRAFT_128562|telomerase protein component 1	gi|222418589|ref|NP_795980.2|	0	leucine-rich repeat and WD repeat-containing protein KIAA1239 [Mus musculus]
mmu-miR-100-5p	NM_182992	-28.6	0.654176	-46.04	311	GO:0030016,GO:0043231,GO:0008092,	K12567|1|9e-58|227|bta:540561|titin [EC:2.7.11.1]	gi|60360554|dbj|BAD90521.1|	0	mKIAA4170 protein [Mus musculus]

mmu-miR-100-5p	NM_194334	-28.1	0.76987	-25.87	171	GO:0044464,GO:0005099,GO:0005488,GO:0032318,	K11837|1|9e-12|74.7|hsa:9098|ubiquitin carboxyl-terminal hydrolase 6/32 [EC:3.1.2.15]!K13136|2|2e-06|57.0|nvi:100114269|gem associated protein 8!K02155|3|9e-06|54.7|gga:416753|V-type H+-transporting ATPase 16kDa proteolipid subunit	gi|57165377|ref|NP_919315.2|	0	TBC1 domain family member 2B [Mus musculus]
mmu-miR-100-5p	NR_003293	-28.1	0.495974	-27.24	165	-	-	gi|148684109|gb|EDL16056.1|	1.06E-84	mCG60780, isoform CRA_a [Mus musculus]
mmu-miR-100-5p	NR_038051	-29	0.311329	-26.18	167	-	-	gi|148677656|gb|EDL09603.1|	1.00E-77	mCG145150 [Mus musculus]

mmu-miR-100-5p	NM_177124	-23.5	1	-44.55	306	GO:0030529,GO:0003676,GO:0006412,GO:0016458,	K13098|1|4e-07|60.8|acs:100556650|RNA-binding protein FUS	gi|159110982|ref|NP_796098.3|	0	trinucleotide repeat-containing gene 6B protein isoform 2 [Mus musculus]
mmu-miR-100-5p	NM_178247	-27.6	0.501363	-24.6	167	-	-	gi|30142691|ref|NP_839978.1|	4.62E-38	developmental pluripotency associated 1 precursor [Mus musculus]
mmu-miR-100-5p	NM_024200	-26.2	0.914083	-21.78	166	GO:0031224,GO:0031966,GO:0017111,GO:0032561,GO:0009207,GO:0032501,GO:00069	K06030|1|0.0|1370|mmu:67414|mitofusin [EC:3.6.5.-]	gi|60360220|dbj|BAD90354.1|	0	mKIAA4032 protein [Mus musculus]
mmu-miR-100-5p	NM_026911	-30.3	0.069224	-24.14	165	GO:0043234,GO:0031227,GO:0006508,GO:0030073,GO:0015980,	K12946|1|7e-92|337|mmu:69019|signal peptidase complex subunit 1 [EC:3.4.-.-]	gi|148692833|gb|EDL24780.1|	2.41E-100	signal peptidase complex subunit 1 homolog (S. cerevisiae), isoform CRA_a [Mus musculus]
mmu-miR-100-5p	NR_040715	-25.2	0.667706	-21.22	164	-	-	gi|148693445|gb|EDL25392.1|	7.20E-32	mCG7469 [Mus musculus]
mmu-miR-100-5p	NM_199028	-25.8	0.976467	-23.07	163	-	-	gi|39841055|ref|NP_950193.1|	0	BEN domain-containing protein 3 [Mus musculus]

mmu-miR-100-5p	NM_011155	-20.5	0.999999	-22.78	162	-	K04460|1|0.0|974|mmu:19060|protein phosphatase 5 [EC:3.1.3.16]	gi|199559777|ref|NP_035285.2|	0	serine/threonine-protein phosphatase 5 [Mus musculus]
mmu-miR-100-5p	NM_027297	-25.1	0.987506	-22.3	161	GO:0030532,GO:0016604,GO:0005488,GO:0000377,	K12662|1|0.0|1014|ptr:464671|U4/U6 small nuclear ribonucleoprotein PRP4	gi|62089180|dbj|BAD93034.1|	0	PRPF4 protein variant [Homo sapiens]
mmu-miR-100-5p	NR_033442	-24.7	0.983997	-21.28	160	GO:0000808,GO:0031981,GO:0043565,GO:0016462,GO:0032559,GO:0007050,GO:0006259,GO:0051320,	K02606|1|0.0|523|rno:295596|origin recognition complex subunit 4	gi|124286818|ref|NP_036088.3|	0	origin recognition complex subunit 4 isoform 1 [Mus musculus]
mmu-miR-100-5p	NR_045537	-25.5	0.862887	-23.71	159	GO:0019866,GO:0043231,GO:0031224,GO:0005488,GO:0010876,GO:0006810,	-	gi|170671720|ref|NP_076201.2|	0	protein spinster homolog 1 [Mus musculus]

mmu-miR-100-5p	NM_178688	-28.4	0.76455	-24.18	158	GO:0005856,GO:0008092,GO:0046914,GO:0006996,GO:0006357,GO:0006935,	K07520|1|0.0|1427|cfa:477828|actin-binding LIM protein	gi|157057145|ref|NP_848803.3|	0	actin-binding LIM protein 1 isoform 1 [Mus musculus]
mmu-miR-100-5p	NM_024436	-25.3	0.783011	-23.91	157	GO:0005768,GO:0031090,GO:0017111,GO:0032561,GO:0016192,GO:0035556,GO:0006996,GO:0009207,	K07891|1|2e-108|393|mmu:19334|Ras-related protein Rab-22A	gi|397525605|ref|XP_003832751.1|	2.21E-112	PREDICTED: ras-related protein Rab-22A-like [Pan paniscus]
mmu-miR-100-5p	NR_040689	-25.1	0.333252	-23.55	156	-	-	gi|148705897|gb|EDL37844.1|	7.39E-22	mCG1046397, isoform CRA_a [Mus musculus]
mmu-miR-100-5p	NR_038163	-24.5	0.963293	-20.17	155	-	-	gi|148666262|gb|EDK98678.1|	8.00E-27	mCG140829 [Mus musculus]
mmu-miR-100-5p	NM_172819	-24.3	0.999829	-20.64	154	GO:0043231,GO:0005515,	-	gi|226823266|ref|NP_766407.2|	0	disco-interacting protein 2 homolog B isoform 2 [Mus musculus]
mmu-miR-100-5p	NM_181420	-23.3	0.980482	-22.21	153	GO:0016772,GO:0006796,	K15523|1|3e-173|609|mmu:238024|protein-ribulosamine 3-kinase [EC:2.7.1.1	gi|253970463|ref|NP_852085.2|	2.04E-172	ketosamine-3-kinase [Mus musculus]

mmu-miR-100-5p	NM_172049	-24.2	0.978125	-20.64	152	GO:0005635,GO:0031224,GO:0048870,	-	gi|157951600|ref|NP_742046.2|	1.69E-56	transmembrane protein 18 [Mus musculus]
mmu-miR-100-5p	NM_199310	-22.6	0.993047	-24.24	151	-	-	gi|160298152|ref|NP_955014.2|	3.01E-98	uncharacterized protein C15orf57 homolog [Mus musculus]
mmu-miR-100-5p	NR_046233	-27.4	0.980772	-20.03	150	-	K05287|1|5e-23|74.3|oaa:100077127|phosphatidylinositol glycan, class F [EC:2.7.-.-]!K05692|3|6e-16|89.7|ptr:746570|actin beta/gamma 1!K13157|4|1e-10|72.0|mdo:100032985|U11/U12 small nuclear ribonucleoprotein 65 kDa	gi|14669161|gb|AAK71346.1|	8.77E-74	ETS-related transcription factor ERF [Mus musculus]

mmu-miR-100-5p	NM_178651	-23.3	0.998234	-20.11	149	GO:0031224,GO:0043231,GO:0043232,GO:0005488,GO:0001071,GO:0015075,GO:0006281,GO:0000041,GO:0006351,	K14696|1|0.0|1038|mmu:109108|solute carrier family 30 (zinc transporter), member 9	gi|125660458|ref|NP_848766.2|	0	zinc transporter 9 [Mus musculus]	
mmu-miR-100-5p	NM_008427	-23.5	0.978536	-20.9	148	GO:0043005,GO:0044297,GO:0005887,GO:0005249,GO:0019904,GO:0007267,GO:0034220,GO:0030001,	K04998|1|0.0|729|mmu:16520|potassium inwardly-rectifying channel subfamily J member 4	gi|158711668|ref|NP_032453.3|	0	inward rectifier potassium channel 4 [Mus musculus]	
mmu-miR-100-5p	NM_023842	-22.1	1	-21.63	147	GO:0043231,GO:0005856,GO:0005913,GO:0005515,GO:0005198,GO:0006915,GO:0009611,GO:0009913,GO:0045216,GO:0007155,GO:0006464,	K10381|1|0.0|5019|mmu:109620|desmoplakin	gi|190194418|ref|NP_076331.2|	0	desmoplakin [Mus musculus]	

mmu-miR-100-5p	NR_027627	-23	0.999102	-20.42	146	GO:0005887,GO:0005249,GO:0042391,GO:0034220,GO:0007166,GO:0030001,	K04913|1|0.0|788|mmu:16526|potassium channel subfamily K member	gi|38566067|gb|AAH62094.1|	0	Kcnk2 protein [Mus musculus]
mmu-miR-100-5p	NM_183024	-20.2	1	-20.49	145	GO:0044424,GO:0032991,GO:0003676,	K11219|1|3e-102|374|cfa:476714|non-receptor tyrosine kinase [EC:2.7.10.2]!K13208|2|4e-08|62.0|dre:30737|ELAV like protein 2/3/4!K13219|3|4e-08|62.0|dre:406564|splicing factor, proline- and glutamine-rich!K13214|4|4e-08|62.0|tgu:100223	gi|33942106|ref|NP_898845.1|	0	ribonucleoprotein PTB-binding 2 [Mus musculus]

mmu-miR-100-5p	NM_029426	-22.4	0.999977	-22.24	144	GO:0046872,GO:0004672,GO:0032559,GO:0048858,GO:0030154,GO:0007163,GO:00064	K08796|1|0.0|1215|mmu:75770|BR serine/threonine kinase [EC:2.7.11.1]	gi|57864120|ref|NP_083702.1|	0	serine/threonine-protein kinase BRSK2 isoform alpha [Mus musculus]
mmu-miR-100-5p	NM_001163553	NA	NA	-26.76	156	GO:0044431,GO:0030119,GO:0022892,GO:0015031,	K12401|1|0.0|1367|mmu:67489|AP-4 complex subunit beta-1	gi|254588022|ref|NP_001157025.1|	0	AP-4 complex subunit beta-1 isoform b [Mus musculus]
mmu-miR-100-5p	NM_026193	NA	NA	-26.76	156	GO:0044431,GO:0030119,GO:0005488,GO:0022892,GO:0015031,	K12401|1|0.0|1449|mmu:67489|AP-4 complex subunit beta-1	gi|148675637|gb|EDL07584.1|	0	adaptor-related protein complex AP-4, beta 1, isoform CRA_a [Mus musculus]
mmu-miR-100-5p	NR_027323	NA	NA	-20.3	151	GO:0031224,	K13023|1|2e-31|139|pon:100174713|carboxypeptidase N regulatory subunit	gi|149036465|gb|EDL91083.1|	0	rCG56516 [Rattus norvegicus]

mmu-miR-100-5p	NM_173212	NA	NA	-20.37	147	GO:0005887,GO:0004871,GO:0042165,GO:0005231,GO:0051259,GO:0006811,GO:00072	K04814|1|0.0|868|rno:171131|nicotinic acetylcholine receptor beta-3	gi|148700842|gb|EDL32789.1|	0	cholinergic receptor, nicotinic, beta polypeptide 3, isoform CRA_b [Mus musculus]
mmu-miR-100-5p	NM_008635	NA	NA	-20.77	146	GO:0015630,GO:0016020,GO:0005488,GO:0040007,GO:0032989,GO:0042592,GO:0006664,GO:0000226,GO:0048232,GO:0033189,GO:0003006,GO:0006950,	K10433|1|2e-163|577|mmu:17761|microtubule-associated protein 7	gi|159110787|ref|NP_032661.2|	8.68E-165	ensconsin isoform 1 [Mus musculus]

mmu-miR-100-5p	NM_001166437	NA	NA	-20.18	145	GO:0044464,GO:0005099,GO:0016769,GO:0032313,	K13136|1|1e-48|195|nvi:100114269|gem associated protein 8!K12813|2|3e-16|88.2|nvi:100117259|pre-mRNA-splicing factor ATP-dependent RNA helicase DHX16 [EC:3.6.4.13]!K11452|3|2e-09|65.9|tgu:100222038|chromobox protein 4	gi|261878622|ref|NP_001159909.1|	0	TBC1 domain family member 25 isoform 2 [Mus musculus]
mmu-miR-100-5p	NM_022811	-22.7	0.999244	NA	NA	GO:0044459,GO:0031981,GO:0034062,GO:0003676,GO:0006351,	K03005|1|0.0|857|mmu:64424|DNA-directed RNA polymerase I subunit RPA49	gi|12328816|ref|NP_073722.1|	0	DNA-directed RNA polymerase I subunit RPA49 [Mus musculus]

mmu-miR-100-5p	NM_001143672	-20.7	0.999451	NA	NA	GO:0031224,	-	gi|119369434|sp|Q5FW56.2|FA70B_MOUSE	0	RecName: Full=Protein FAM70B
mmu-miR-100-5p	NM_009313	-26.3	0.925639	NA	NA	GO:0043005,GO:0016021,GO:0044424,GO:0004995,GO:0006954,GO:0007186,GO:0033555,GO:0030278,GO:0019229,GO:0007631,GO:0032946,GO:0009612,GO:0007613,GO:0001508,GO:0008217,GO:0014072,GO:0007271,GO:0007600,GO:0007612,GO:0051705,GO:0032233,GO:00106	K04222|1|0.0|773|mmu:21336|tachykinin receptor 1	gi|31543840|ref|NP_033339.2|	0	substance-P receptor [Mus musculus]

mmu-miR-100-5p	NM_001198933	-23.6	0.995815	NA	NA	GO:0043231,GO:0044444,GO:0046914,GO:0004470,GO:0032559,GO:0006629,GO:0043648,GO:0051259,GO:0006739,GO:0009719,GO:0010033,	K00029|1|0.0|1048|mmu:17436|malate dehydrogenase (oxaloacetate-decarboxylating)(NADP+) [EC:1.1.1.40]	gi|312147392|ref|NP_001185862.1|	0	NADP-dependent malic enzyme isoform 2 [Mus musculus]
mmu-miR-100-5p	NR_045578	-24.8	0.99616	NA	NA	GO:0031224,	-	gi|23346515|ref|NP_694731.1|	0	suppressor of tumorigenicity 7 protein-like isoform 1 [Mus musculus]
mmu-miR-100-5p	NM_001081549	-20.5	1	NA	NA	GO:0043231,GO:0003676,GO:0001071,GO:0048545,GO:0019932,GO:0048747,GO:0007399,GO:0003013,GO:0051148,GO:0010467,GO:0010921,	-	gi|125656176|ref|NP_001075018.1|	1.93E-121	calcipressin-1 isoform 1 [Mus musculus]

mmu-miR-100-5p	NM_019814	-20.8	0.999985	NA	NA	GO:0032991,GO:0031224,GO:0043231,GO:0050896,	-	gi|293363057|ref|XP_002730318.1|	4.43E-49	PREDICTED: HIG1 domain family member 1A-like [Rattus norvegicus]
mmu-miR-100-5p	NM_001166411	-20.7	0.999711	NA	NA	GO:0043231,GO:0043005,GO:0030529,GO:0003676,GO:0043021,GO:0006412,GO:0006950,GO:0035195,	K13186|1|6e-40|165|ecb:100051736|RNA-binding protein 3	gi|148702008|gb|EDL33955.1|	3.04E-45	mCG116386, isoform CRA_e [Mus musculus]
mmu-miR-100-5p	NM_029402	-20.1	1	NA	NA	GO:0000153,GO:0019899,GO:0006917,GO:0022402,GO:0051704,GO:0019941,GO:00082	K03870|1|0.0|1463|mmu:71745|cullin 2	gi|60359986|dbj|BAD90212.1|	0	mKIAA4106 protein [Mus musculus]
mmu-miR-100-5p	NM_146211	-21.9	0.99998	NA	NA	GO:0044432,GO:0035250,GO:0008610,	K11703|1|0.0|1115|mmu:234407|collagen beta-1,O-galactosyltransferase [EC:2.4.1.50]	gi|170784829|ref|NP_666323.2|	0	procollagen galactosyltransferase 1 precursor [Mus musculus]

mmu-miR-100-5p	NM_133945	-22.7	0.989519	NA	NA	GO:0043231,GO:0019902,GO:0016301,GO:0032559,GO:0010922,GO:0043409,GO:00064	K08816|1|0.0|935|mmu:101568|vaccinia related kinase [EC:2.7.11.1]	gi|148690802|gb|EDL22749.1|	0	vaccinia related kinase 3, isoform CRA_a [Mus musculus]
mmu-miR-100-5p	NM_178906	-21.4	1	NA	NA	-	-	gi|26343487|dbj|BAC35400.1|	4.87E-63	unnamed protein product [Mus musculus]
mmu-miR-100-5p	NM_029000	-22.4	1	NA	NA	-	K12797|1|5e-16|89.4|aml:100471523|caspase recruitment domain-containing protein 6	gi|115270958|ref|NP_083276.2|	0	interferon-induced very large GTPase 1 [Mus musculus]

mmu-miR-100-5p	NM_025564	-20.1	0.998711	NA	NA	GO:0043234,GO:0005681,GO:0016604,GO:0044444,GO:0003676,GO:0006412,GO:0007276,GO:0000377,GO:0006405,GO:0000956,GO:0006397,GO:0006353,	K12877|1|2e-82|305|mmu:66441|protein mago nashi	gi|256985211|ref|NP_079840.2|	1.55E-81	protein mago nashi homolog 1-related [Mus musculus]
mmu-miR-100-5p	NM_001005787	-20	1	NA	NA	GO:0043296,GO:0044444,GO:0005488,GO:0034330,	K06092|1|0.0|987|rno:140581|InaD-like protein	gi|557695576|ref|NP_001005787.1|	0	inaD-like protein isoform 4 [Mus musculus]

mmu-miR-100-5p	NM_001164148	-23	0.999107	NA	NA	GO:0031981,GO:0000786,GO:0043234,GO:0001071,GO:0046983,GO:0042802,GO:0008134,GO:0031647,GO:0045597,GO:0016447,GO:0030183,GO:0048537,GO:0030888,GO:0051318,GO:0051726,GO:0002237,GO:00	K09063|1|0.0|996|mmu:21423|transcription factor E2-alpha	gi|148699595|gb|EDL31542.1|	0	transcription factor E2a, isoform CRA_b [Mus musculus]
mmu-miR-100-5p	NM_144830	-22.4	0.998602	NA	NA	GO:0031224,	-	gi|74204009|dbj|BAE29006.1|	4.32E-156	unnamed protein product [Mus musculus]
mmu-miR-100-5p	NM_178942	-23.2	0.991464	NA	NA	GO:0044464,GO:0004871,GO:0023052,	K05166|1|0.0|1325|mmu:171095|interleukin 17 receptor C	gi|359751380|ref|NP_849273.1|	0	interleukin-17 receptor C isoform 1 precursor [Mus musculus]

mmu-miR-100-5p	NM_153803	-24.9	0.969612	NA	NA	GO:0015925,GO:0043167,GO:0044238,	K12309|1|2e-105|385|bta:507188|beta-galactosidase [EC:3.2.1.23]	gi|24418925|ref|NP_722498.1|	0	beta-galactosidase-1-like protein 2 [Mus musculus]
mmu-miR-100-5p	NM_008319	-20.6	1	NA	NA	GO:0016021,GO:0005488,GO:0007155,GO:0006897,	K06769|1|0.0|1625|mmu:15898|intercellular adhesion molecule 5	gi|159110562|ref|NP_032345.2|	0	intercellular adhesion molecule 5 precursor [Mus musculus]
mmu-miR-100-5p	NM_031408	-24.1	0.998886	NA	NA	GO:0005488,GO:0007169,	-	gi|154091042|ref|NP_113596.2|	0	PERQ amino acid-rich with GYF domain-containing protein 1 [Mus musculus]
mmu-miR-100-5p	NM_027998	-21.4	0.999774	NA	NA	GO:0043296,GO:0007043,	K06087|1|4e-139|495|mmu:71908|claudin	gi|21312576|ref|NP_082274.1|	2.94E-138	claudin-23 [Mus musculus]
mmu-miR-100-5p	NM_172275	-20.3	1	NA	NA	GO:0044464,GO:0046914,GO:0031348,GO:0010033,	-	gi|254553256|ref|NP_758479.2|	0	TRAF-type zinc finger domain-containing protein 1 isoform 2 [Mus musculus]
mmu-miR-100-5p	NR_034030	-21	1	NA	NA	-	-	gi|300797303|ref|NP_001177930.1|	0	protein Lines homolog isoform 2 [Mus musculus]

mmu-miR-100-5p	NM_025446	-21.1	0.996548	NA	NA	GO:0031224,	-	gi|13384848|ref|NP_079722.1|	3.16E-143	androgen-induced gene 1 protein [Mus musculus]
mmu-miR-100-5p	NM_018778	-20.5	1	NA	NA	GO:0043296,GO:0043231,GO:0031224,GO:0005515,GO:0016337,	K06087|1|1e-114|414|mmu:54420|claudin	gi|9055192|ref|NP_061248.1|	8.88E-114	claudin-8 [Mus musculus]
mmu-miR-100-5p	NM_011068	-20.5	0.998155	NA	NA	GO:0031231,GO:0006629,GO:0045444,GO:0007031,GO:0023052,	K13351|1|1e-118|426|mmu:18631|peroxin-11A	gi|148675102|gb|EDL07049.1|	2.08E-131	peroxisomal biogenesis factor 11a, isoform CRA_b [Mus musculus]
mmu-miR-100-5p	NM_025788	-20.4	1	NA	NA	GO:0044451,GO:0012502,GO:0051259,GO:0006351,GO:0008283,	K10486|1|0.0|911|mmu:66830|BTB/POZ domain-containing protein 14	gi|268837281|ref|NP_080064.3|	0	nucleus accumbens-associated protein 1 [Mus musculus]
mmu-miR-100-5p	NM_181853	-21.4	1	NA	NA	GO:0044427,GO:0031981,GO:0016234,GO:0046914,GO:0042802,GO:0006355,	K12032|1|0.0|2440|mmu:330627|tripartite motif-containing protein	gi|284004881|ref|NP_001164383.1|	0	tripartite motif-containing protein 66 isoform 1 [Mus musculus]

mmu-miR-100-5p	NM_175362	-21	1	NA	NA	-	K07367|1|0.0|2109|mmu:108723|caspase recruitment domain-containing protein 11	gi|47847424|dbj|BAD21384.1|	0	mFLJ00120 protein [Mus musculus]
mmu-miR-100-5p	NM_026184	-23.5	0.999136	NA	NA	GO:0031090,GO:0043231,GO:0016667,GO:0005515,GO:0050662,GO:0051234,GO:0006457,GO:0006091,	K10976|1|0.0|881|mmu:67475|ERO1-like protein beta [EC:1.8.4.-]	gi|38348230|ref|NP_080460.2|	0	ERO1-like protein beta precursor [Mus musculus]
mmu-miR-100-5p	NM_001109661	-20.2	1	NA	NA	GO:0043231,GO:0003677,GO:0001071,GO:0005515,GO:0006351,	K09042|1|0.0|1570|mmu:12014|transcription regulator BACH	gi|157817436|ref|NP_001103131.1|	0	transcription regulator protein BACH2 [Mus musculus]

mmu-miR-100-5p	NM_016980	-22.1	0.998781	NA	NA	GO:0043234,GO:0031981,GO:0015934,GO:0019843,GO:0005198,GO:0006412,GO:0019080,GO:0010035,GO:0031016,GO:0042254,GO:0010921,	K02932|1|1e-157|557|ptr:457025|large subunit ribosomal protein L5e	gi|390461662|ref|XP_002746579.2|	1.21E-156	PREDICTED: 60S ribosomal protein L5 [Callithrix jacchus]
mmu-miR-100-5p	NM_010029	-20.5	1	NA	NA	GO:0043231,GO:0043186,GO:0030529,GO:0042623,GO:0032559,GO:0048870,GO:0009207,GO:0048232,GO:0008104,GO:00071	K13982|1|0.0|1258|mmu:13206|probable ATP-dependent RNA helicase DDX4 [EC:3.6.4.13]	gi|148686462|gb|EDL18409.1|	0	DEAD (Asp-Glu-Ala-Asp) box polypeptide 4 [Mus musculus]
mmu-miR-100-5p	NM_016742	-24	0.969234	NA	NA	GO:0044444,GO:0031072,GO:0032559,GO:0051726,GO:0001959,GO:0006886,	K09554|1|0.0|680|mmu:12539|cell division cycle protein 37	gi|7949018|ref|NP_058022.1|	0	hsp90 co-chaperone Cdc37 [Mus musculus]

mmu-miR-100-5p	NM_001033493	-22.1	0.999962	NA	NA	GO:0031224,GO:0004888,GO:0007186,	K08454|1|0.0|1231|mmu:435529|G protein-coupled receptor 111	gi|134949047|ref|NP_001028665.2|		0	G protein-coupled receptor 111 precursor [Mus musculus]
mmu-miR-100-5p	NM_009021	-20.5	1	NA	NA	GO:0043231,GO:0046914,GO:0035264,GO:0048731,GO:0006355,	K09188|1|3e-13|79.7|dme:Dmel_CG3848|histone-lysine N-methyltransferase MLL3 [EC:2.1.1.43]	gi|83649745|ref|NP_001032853.1|		0	retinoic acid-induced protein 1 [Mus musculus]
mmu-miR-100-5p	NM_011215	-21.4	1	NA	NA	GO:0016021,GO:0033267,GO:0016023,GO:0005488,GO:0004871,GO:0004725,GO:0006470,GO:0043087,GO:0023052,	K07817|1|0.0|1833|mmu:19276|protein tyrosine phosphatase, receptor type, N [EC:3.1.3.48]	gi|226052731|ref|NP_035345.2|		0	receptor-type tyrosine-protein phosphatase N2 precursor [Mus musculus]

mmu-miR-100-5p	NM_139153	-25.7	0.88626	NA	NA	GO:0044424,GO:0017111,GO:0005083,GO:0008047,GO:0046914,GO:0032561,GO:0043087,GO:0032012,	K12491|1|0.0|1548|mmu:213990|Arf-GAP, GTPase, ANK repeat and PH domain-containing protein 1/2/3	gi|124301219|ref|NP_631892.2|	0	arf-GAP with GTPase, ANK repeat and PH domain-containing protein 3 isoform a [Mus musculus]
mmu-miR-100-5p	NM_015830	-20.5	1	NA	NA	GO:0044464,GO:0046914,GO:0001071,GO:0004197,GO:0019538,GO:0010467,	K08582|1|0.0|1956|mmu:50817|calpain-15 [EC:3.4.22.-]	gi|7657601|ref|NP_056645.1|	0	calpain-15 [Mus musculus]
mmu-miR-100-5p	NM_177379	-20.2	1	NA	NA	GO:0043231,GO:0030425,GO:0044459,GO:0044444,GO:0031090,GO:0012505,GO:0044430,GO:0008047,GO:0005543,GO:0007265,GO:0043087,GO:0009966,	K04450|1|1e-25|121|ptr:739083|activating transcription factor 2!K08878|2|1e-24|118|cin:100176232|breakpoint cluster region protein [EC:2.7.11.	gi|307344692|ref|NP_001182561.1|	0	rho GTPase-activating protein 32 isoform 1 [Mus musculus]

mmu-miR-100-5p	NM_133762	-26	0.976183	NA	NA	GO:0031981,GO:0042393,GO:0051276,GO:0000087,	K11492|1|0.0|2241|mmu:76044|condensin-2 complex subunit G2	gi|91208439|ref|NP_598523.3|	0	condensin-2 complex subunit G2 [Mus musculus]
mmu-miR-100-5p	NM_001110499	-33.1	0.126473	NA	NA	GO:0031224,GO:0044432,GO:0030425,GO:0044297,GO:0031090,GO:0030529,GO:0048770,GO:0046872,GO:0035254,GO:0030246,GO:0044267,GO:0032502,	K08054|1|0.0|1038|mmu:12330|calnexin	gi|6671664|ref|NP_031623.1|	0	calnexin precursor [Mus musculus]
mmu-miR-100-5p	NM_001195130	-20.3	1	NA	NA	GO:0043234,GO:0043231,GO:0046872,GO:0005515,GO:0048232,	K11457|1|0.0|1130|rno:313038|polyhomeotic-like protein 2	gi|9055280|ref|NP_061244.1|	0	polyhomeotic-like protein 2 isoform A [Mus musculus]

mmu-miR-100-5p	NR_027957	-24.1	0.996791	NA	NA	-	K15424|1|3e-19|93.6|ecb:100056167|serine/threonine-protein phosphatase 4 regulatory subunit	gi|148674700|gb|EDL06647.1|	1.60E-69	mCG145743, isoform CRA_c [Mus musculus]
mmu-miR-100-5p	NM_001122737	-25.9	0.898642	NA	NA	GO:0044421,GO:0005102,GO:0009653,GO:0031016,GO:0006357,GO:0008283,GO:0043627,GO:0001503,GO:0007169,GO:0007088,GO:0014070,GO:0010740,GO:0019318,GO:0009628,GO:0009991,GO:0042692,GO:00	K13769|1|1e-78|295|mmu:16002|insulin-like growth factor 2	gi|298231180|ref|NP_034644.2|	1.39E-79	insulin-like growth factor II isoform 1 preproprotein [Mus musculus]

mmu-miR-101a-3p	NM_001004762	-27.7	0.419159	-25.11	175	GO:0044444,GO:0004623,GO:0008289,GO:0000038,GO:0006952,GO:0022414,GO:0007165,GO:0006650,	K01047|1|5e-40|167|xtr:100145731|phospholipase A2 [EC:3.1.1.4]	gi|52317152|ref|NP_001004762.1|	0	phospholipase A2, group IVC isoform 2 [Mus musculus]
mmu-miR-101a-3p	NM_001033535	-32.3	0.037393	-28.94	183	-	-	gi|109484717|ref|XP_001072284.1|	2.40E-121	PREDICTED: tumor necrosis factor alpha-induced protein 8-like protein 3-like [Rattus norvegicus]
mmu-miR-101a-3p	NM_001168504	-27.7	0.420842	-25.11	175	GO:0044444,GO:0004623,GO:0008289,GO:0000038,GO:0006952,GO:0022414,GO:0007165,GO:0006650,	K01047|1|7e-40|167|xtr:100145731|phospholipase A2 [EC:3.1.1.4]	gi|270341357|ref|NP_001161976.1|	0	phospholipase A2, group IVC isoform 1 [Mus musculus]
mmu-miR-101a-3p	NM_015765	-27	0.300387	-25.15	173	GO:0044444,GO:0030529,GO:0032559,GO:0006458,	K03283|1|1e-86|321|cbr:CBG00457|heat shock 70kDa protein 1/8	gi|82880662|ref|NP_056580.2|	0	heat shock 70 kDa protein 14 isoform 1 [Mus musculus]

mmu-miR-101a-3p	NM_023203	-28.1	0.04838	-26.61	167	GO:0044444,GO:0046872,GO:0047429,GO:0005515,GO:0019103,GO:0009141,GO:00512	-	gi|12963573|ref|NP_075692.1|	2.36E-84	dCTP pyrophosphatase 1 [Mus musculus]
mmu-miR-101a-3p	NM_146039	-28.8	0.309121	-25.66	168	-	K10415|1|1e-10|70.1|nve:NEMVE_v1g158934|dynein intermediate chain, cytosolic	gi|113199781|ref|NP_666151.3|	0	WD repeat-containing protein 60 [Mus musculus]
mmu-miR-101a-3p	NM_172417	-30.4	0.170746	-28.94	178	-	K10431|1|5e-08|61.6|tgu:100222640|microtubule-associated protein 4!K04989|2|1e-07|60.1|rno:100364048|polycystin 1L3	gi|161377418|ref|NP_766005.2|	0	uncharacterized protein C1orf170 homolog [Mus musculus]

mmu-miR-101a-3p	NM_176832	-29.3	0.323695	-26.7	165	GO:0043232,GO:0043231,GO:0044444,GO:0046914,GO:0008092,GO:0016192,	K02098|1|0.0|888|ptr:468603|spire	gi|148677693|gb|EDL09640.1|		0	spire homolog 1 (Drosophila), isoform CRA_d [Mus musculus]
mmu-miR-101a-3p	NM_194355	-29.3	0.340403	-26.7	165	GO:0043231,GO:0044444,GO:0005515,GO:0016192,	K02098|1|0.0|1063|rno:307348|spire	gi|148677692|gb|EDL09639.1|		0	spire homolog 1 (Drosophila), isoform CRA_c [Mus musculus]
mmu-miR-101a-3p	NM_205810	-27.8	0.482763	-25.2	170	GO:0031224,GO:0042277,GO:0004888,GO:0007166,	K08396|1|7e-135|483|mmu:243978|Mas-related G protein-coupled receptor member X	gi|154240718|ref|NP_991379.3|		0	mas-related G-protein coupled receptor member B1 [Mus musculus]
mmu-miR-101a-3p	NM_178667	-21.1	1	-28.12	174	GO:0043231,GO:0043234,GO:0012505,GO:0000989,GO:0003676,GO:0001071,GO:0005515,GO:0006351,	K09392|1|0.0|649|aml:100464401|transcription factor Dp-2	gi|8039811|sp|Q64163.2|TFDP2_MOUSE		0	RecName: Full=Transcription factor Dp-2; AltName: Full=Dp-3; AltName: Full=E2F dimerization partner 2

mmu-miR-101a-3p	NM_023799	-26.4	0.756858	-23.99	171	GO:0043231,GO:0004563,GO:0004468,GO:0009057,GO:0016570,GO:0010561,GO:0032846,GO:0010828,GO:0006041,GO:0030162,GO:0010522,GO:0060123,GO:0009215,GO:0032502,GO:0006605,GO:0010612,GO:0008219,GO:0043241,GO:0043270,GO:0050796,GO:0010604,GO:00097	K15719|1|0.0|1807|mmu:76055|protein O-GlcNAcase / histone acetyltransferase [EC:3.2.1.169 2.3.1.48]	gi|37360040|dbj|BAC97998.1|	0	mKIAA0679 protein [Mus musculus]
mmu-miR-101a-3p	NM_177124	-21	1	-24.81	170	GO:0030529,GO:0003676,GO:0006412,GO:0016458,	K13098|1|4e-07|60.8|acs:100556650|RNA-binding protein FUS	gi|159110982|ref|NP_796098.3|	0	trinucleotide repeat-containing gene 6B protein isoform 2 [Mus musculus]

mmu-miR-101a-3p	NM_178654	-24.2	0.990934	-20.07	169	GO:0043231,GO:0019899,GO:0004674,GO:0032559,GO:0006464,GO:0023052,	K06071|1|0.0|1677|mmu:109333|protein kinase N [EC:2.7.11.13]	gi|260099670|ref|NP_848769.2|	0	serine/threonine-protein kinase N2 [Mus musculus]
mmu-miR-101a-3p	NM_178803	-22.4	0.999947	-22.01	168	GO:0031519,GO:0003712,GO:0003676,GO:0046914,GO:0006351,GO:0006325,	K09232|1|3e-13|79.7|cin:778878|zinc finger protein GLIS1/3	gi|190358164|sp|Q9Z248.2|AEBP2_MOUSE	3.48E-152	RecName: Full=Zinc finger protein AEBP2; AltName: Full=Adipocyte enhancer-binding protein 2; Short=AE-binding protein 2
mmu-miR-101a-3p	NR_037692	-23.7	0.658509	-21.41	167	-	-	gi|12855626|dbj|BAB30402.1|	1.91E-75	unnamed protein product [Mus musculus]
mmu-miR-101a-3p	NR_033597	-24.2	0.848592	-21.03	166	GO:0043231,GO:0007049,GO:0050794,	K10401|1|1e-75|269|mgp:100547630|kinesin family member 18/19	gi|254939627|ref|NP_932063.2|	0	kinesin-like protein KIF18B [Mus musculus]

mmu-miR-101a-3p	NM_201518	-27.9	0.665282	-24.46	165	GO:0016021,GO:0031012,GO:0004871,GO:0005515,GO:0009987,	K13023|1|7e-29|131|hsa:1370|carboxypeptidase N regulatory subunit!K07523|2|2e-28|130|xtr:100188921|netrin-	gi|41462393|ref|NP_958926.1|	0	fibronectin leucine rich transmembrane protein 2 [Mus musculus]
mmu-miR-101a-3p	NM_177186	-21	1	-26.33	164	GO:0031224,	K15284|1|0.0|716|mmu:320541|solute carrier family 35, member E2	gi|28893193|ref|NP_796160.1|	0	solute carrier family 35 member E2 [Mus musculus]
mmu-miR-101a-3p	NM_031374	-22.3	0.999999	-20.13	163	-	-	gi|118722338|ref|NP_113551.2|	0	testis expressed gene 15 [Mus musculus]
mmu-miR-101a-3p	NM_197959	-24.2	0.928663	-20.06	162	GO:0043231,GO:0015630,GO:0003774,GO:0032559,GO:0050794,GO:0007017,GO:00000	K10401|1|8e-126|452|xtr:548999|kinesin family member 18/19	gi|254939627|ref|NP_932063.2|	0	kinesin-like protein KIF18B [Mus musculus]
mmu-miR-101a-3p	NR_039546	-24.1	0.009668	-21.56	161	-	-	-	-	-

mmu-miR-101a-3p	NR_045057	-24	0.595177	-21.33	160	-	-	gi|148680200|gb|EDL12147.1|	4.36E-16	mCG145184 [Mus musculus]
mmu-miR-101a-3p	NM_198634	-23.5	0.902991	-20.23	159	GO:0044427,GO:0043231,GO:0003676,GO:0006351,	K11496|1|5e-33|143|mmu:12616|centromere protein B	gi|148701235|gb|EDL33182.1|	0	tigger transposable element derived 3, isoform CRA_a [Mus musculus]
mmu-miR-101a-3p	NM_183408	-24.8	0.938058	-20.72	158	GO:0043231,GO:0001726,GO:0044444,GO:0016020,GO:0043169,GO:0004114,GO:0006195,GO:0023052,GO:0042221,	K01120|1|0.0|1425|rno:25638|3',5'-cyclic-nucleotide phosphodiesterase [EC:3.1.4.17]	gi|41282015|ref|NP_899668.1|	0	cAMP-specific 3',5'-cyclic phosphodiesterase 4A isoform 5 [Mus musculus]
mmu-miR-101a-3p	NR_030675	-23.1	0.990636	-20.32	157	-	-	gi|74180681|dbj|BAE25568.1|	9.47E-49	unnamed protein product [Mus musculus]
mmu-miR-101a-3p	NR_052009	-23.1	0.993928	-20.76	156	-	-	-	-	-

mmu-miR-101a-3p	NM_178795	-21.3	1	-21.7	155	GO:0044444,GO:0016791,GO:0000828,GO:0032559,GO:0019751,GO:0006796,	K13024|1|0.0|2731|mmu:327655|inositol hexakisphosphate/diphosphoinositol-pentakisphosphate kinase [EC:2.7.4.21 2.7.4.24]	gi|28972185|dbj|BAC65546.1|		0	mKIAA0377 protein [Mus musculus]
mmu-miR-101a-3p	NM_177747	-23.9	0.979106	-22.4	154	GO:0043231,GO:0046914,GO:0003677,GO:0006355,	K09228|1|2e-64|249|bfo:BRAFLDRAFT_71028|KRAB domain-containing zinc finger protein	gi|125347348|ref|NP_808415.2|		0	zinc finger protein 711 [Mus musculus]
mmu-miR-101a-3p	NM_010569	-21.5	0.999999	-20.33	153	GO:0043231,GO:0015630,GO:0005515,GO:0060972,GO:0030178,GO:0048513,	K15503|1|2e-67|259|aag:AaeL_AAEL014668|serine/threonine-protein phosphatase 6 regulatory ankyrin repeat subunit B	gi|148670390|gb|EDL02337.1|		0	inversin, isoform CRA_a [Mus musculus]

mmu-miR-101a-3p	NM_013569	-22.2	0.999682	-20.82	151	GO:0044424,GO:0012505,GO:0005887,GO:0004673,GO:0060089,GO:0005249,GO:0006351,GO:0003012,GO:0030001,GO:0044057,GO:0006796,GO:0051259,GO:0007165,GO:0034220,GO:0006873,GO:00030	K04905|1|0.0|2048|mmu:16511|potassium voltage-gated channel Eag-related subfamily H member 2	gi|157042785|ref|NP_038597.2|	0	potassium voltage-gated channel subfamily H member 2 [Mus musculus]
mmu-miR-101a-3p	NM_172941	-22.5	0.998893	-20.01	150	GO:0044451,GO:0003676,GO:0046914,GO:0001071,GO:0005515,GO:0006357,	K09229|1|0.0|1098|mmu:268417|KRAB and SCAN domains-containing zinc finger	gi|194473739|ref|NP_766529.3|	0	zinc finger protein 496 isoform 1 [Mus musculus]

mmu-miR-101a-3p	NM_001033443	-24	0.920924	-20.12	149	GO:0004674,GO:0032559,GO:0006464,	K08824|1|0.0|667|mmu:381113|cyclin-dependent kinase-like [EC:2.7.11.22]	gi|84370314|ref|NP_001028615.1|	0	cyclin-dependent kinase-like 4 [Mus musculus]
mmu-miR-101a-3p	NM_029494	-21.2	0.998501	-21.54	148	GO:0044431,GO:0016020,GO:0017111,GO:0032561,GO:0006810,GO:0035556,GO:00092	K07917|1|2e-112|407|ecb:100051006|Ras-related protein Rab-30	gi|21361418|ref|NP_055303.2|	1.17E-111	ras-related protein Rab-30 [Homo sapiens]
mmu-miR-101a-3p	NM_001114660	-20.2	1	-20.67	147	GO:0006887,	-	gi|167900448|ref|NP_001108132.1|	0	sec1 family domain-containing protein 2 isoform a [Mus musculus]
mmu-miR-101a-3p	NM_172819	-20.1	1	-20.44	146	GO:0043231,GO:0005515,	-	gi|226823266|ref|NP_766407.2|	0	disco-interacting protein 2 homolog B isoform 2 [Mus musculus]
mmu-miR-101a-3p	NM_145495	NA	NA	-20.42	157	GO:0043232,GO:0016020,GO:0008047,GO:0005488,GO:0043087,GO:0023052,	-	gi|148701147|gb|EDL33094.1|	0	Ras and Rab interactor 1, isoform CRA_a [Mus musculus]

mmu-miR-101a-3p	NM_172882	NA	NA	-21.2	149	GO:0005773,GO:0012505,GO:0044425,GO:0035091,GO:0046914,GO:0035250,	K07295|1|3e-49|200|dpe:Dper_GL19043|nuclear receptor subfamily 2 group A!K01104|2|1e-12|79.0|spu:585727|protein-tyrosine phosphatase [EC:3.1.3.48]!K12182|3|3e-12|77.4|cqu:CpipJ_CPIJ001538|hepatocyte growth factor-regulated tyrosine kinase	gi|39930599|ref|NP_766470.2|	0	WD repeat and FYVE domain-containing protein 3 [Mus musculus]
mmu-miR-101a-3p	NM_178672	NA	NA	-20.67	147	GO:0006887,	-	gi|31982305|ref|NP_848787.2|	0	sec1 family domain-containing protein 2 isoform b [Mus musculus]

mmu-miR-101a-3p	NM_028900	-20.9	0.999999	NA	NA	GO:0016020,GO:0043231,GO:0005488,	K02084|1|7e-63|243|spu:591503|apoptotic protease-activating factor!K06236|2|5e-08|61.6|xla:380515|collagen, type I/II/III/V/XI, alpha	gi|268370067|ref|NP_083176.3|	0	GRIP and coiled-coil domain-containing protein 1 [Mus musculus]
mmu-miR-101a-3p	NM_019671	-20	1	NA	NA	GO:0043231,GO:0044444,GO:0005088,GO:0016049,GO:0006917,GO:0019222,GO:0007169,GO:0009966,GO:0007266,	K07532|1|6e-20|100|cfa:479409|Rho guanine nucleotide exchange factor 12	gi|114145477|ref|NP_062645.2|	0	neuroepithelial cell-transforming gene 1 protein isoform 1 [Mus musculus]
mmu-miR-101a-3p	NM_001177464	-22	0.999999	NA	NA	GO:0043231,GO:0046914,GO:0003676,GO:0010467,	K09228|1|4e-26|122|mcc:694939|KRAB domain-containing zinc finger protein	gi|33942118|ref|NP_898854.1|	0	zinc finger protein 516 [Mus musculus]

mmu-miR-101a-3p	NM_146320	-20.1	0.995875	NA	NA	GO:0004888,GO:0023052,	K04257|1|0.0|634|mmu:258317|olfactory receptor	gi|121247419|ref|NP_666432.2|	1.70E-180	olfactory receptor 1145 [Mus musculus]
mmu-miR-101a-3p	NM_021534	-25.1	0.50674	NA	NA	GO:0044439,GO:0031224,	K13350|1|4e-104|379|mmu:59038|peroxisomal membrane protein 4	gi|10946966|ref|NP_067509.1|	2.97E-103	peroxisomal membrane protein 4 [Mus musculus]
mmu-miR-101a-3p	NM_027992	-21.6	0.999999	NA	NA	GO:0031224,	-	gi|188035856|ref|NP_082268.2|	7.50E-142	transmembrane protein 106B [Mus musculus]

mmu-miR-101a-3p	NM_017468	-21.4	0.999999	NA	NA	GO:0031012,GO:0009888,	K15712|1|4e-11|72.4|cin:100175215|E3 ubiquitin-protein ligase TTC3 [EC:6.3.2.19]!K06238|2|1e-09|67.8|mmu:12835|collagen, type VI, alpha!K03006|4|1e-06|57.8|dpo:Dpse_GA13798|DNA-directed RNA polymerase II subunit RPB1 [EC:2.7.7.6	gi|148673413|gb|EDL05360.1|	0	enamelin, isoform CRA_c [Mus musculus]

mmu-miR-101a-3p	NR_029430	-21.8	0.90042	NA	NA	GO:0015630,GO:0044444,GO:0008092,GO:0060089,GO:0000278,GO:0007417,GO:0051707,GO:0048667,GO:0031111,GO:0048522,GO:0007165,	K04381|1|1e-22|68.6|xtr:493340|stathmin	gi|56118724|ref|NP_001007972.1|	9.20E-22	stathmin 1 [Xenopus (Silurana) tropicalis]

mmu-miR-101a-3p	NM_178804	-20.4	1	NA	NA	GO:0044424,GO:0016020,GO:0044421,GO:0004857,GO:0005102,GO:0046872,GO:0005539,GO:0042802,GO:0043394,GO:0043236,GO:0048675,GO:0010467,GO:0001763,GO:0006915,GO:0001932,GO:0031290,GO:0043009,GO:0002689,GO:0031348,GO:0051384,GO:0021827,GO:00313	K06839|1|0.0|2991|mmu:20563|slit 2	gi|148705684|gb|EDL37631.1|	0	slit homolog 2 (Drosophila), isoform CRA_a [Mus musculus]

mmu-miR-101a-3p	NM_011732	-24.3	0.804593	NA	NA	GO:0043005,GO:0005681,GO:0043234,GO:0044444,GO:0005515,GO:0003723,GO:0043566,GO:0006357,GO:0009968,GO:0008283,GO:0043009,GO:0048255,GO:0008380,GO:00	K09276|1|1e-122|258|mcc:700140|Y-box-binding protein 1	gi|297278427|ref|XP_001088540.2|	7.55E-122	PREDICTED: hypothetical protein LOC700140 [Macaca mulatta]
mmu-miR-101a-3p	NM_019428	-22.5	0.861862	NA	NA	GO:0030681,GO:0031981,GO:0005488,GO:0004549,GO:0006399,	K03539|1|1e-149|529|mmu:54364|ribonuclease P/MRP protein subunit RPP1 [EC:3.1.26.5]	gi|257196209|ref|NP_062301.3|	1.01E-148	ribonuclease P protein subunit p30 [Mus musculus]
mmu-miR-101a-3p	NM_178398	-22.1	0.999623	NA	NA	GO:0043234,GO:0000407,GO:0005773,GO:0035091,GO:0016236,GO:0007033,	-	gi|30409972|ref|NP_848485.1|	0	WD repeat domain phosphoinositide-interacting protein 2 [Mus musculus]

mmu-miR-101a-3p	NM_181421	-20.7	1	NA	NA	-	K10595|1|1e-32|145|mdo:100012223|E3 ubiquitin-protein ligase HERC2 [EC:6.3.2.19]!K10594|4|2e-32|144|spu:592249|E3 ubiquitin-protein ligase HERC1 [EC:6.3.2.19]	gi|359718915|ref|NP_852086.3|	0	probable E3 ubiquitin-protein ligase C12orf51 homolog [Mus musculus]

mmu-miR-101a-3p	NM_178220	-21.2	1	NA	NA	GO:0031410,GO:0005764,GO:0042995,GO:0031234,GO:0044427,GO:0012506,GO:0004468,GO:0008047,GO:0001664,GO:0043565,GO:0019899,GO:0001775,GO:0001541,GO:0002029,GO:0050954,GO:0016567,GO:0006198,GO:0007187,GO:0043433,GO:0007599,GO:0016573,GO:00481	K04439|1|0.0|665|mmu:109689|beta-arrestin	gi|30089688|ref|NP_835738.1|	0	beta-arrestin-1 isoform B [Mus musculus]

mmu-miR-101a-3p	NM_011625	-22.5	0.999283	NA	NA	GO:0016020,GO:0031981,GO:0005488,GO:0012502,GO:0051726,	K06270|1|3e-15|85.9|dpo:Dpse_GA16721|protein phosphatase 1 regulatory subunit 12A!K12329|2|6e-15|84.7|acs:100566099|protein phosphatase 1 regulatory subunit 12B	gi|45592936|ref|NP_035755.1|	0	apoptosis-stimulating of p53 protein 1 [Mus musculus]
mmu-miR-101a-3p	NM_009608	-20.7	0.996977	NA	NA	-	K12314|1|0.0|764|acs:100560364|actin, alpha cardiac muscle	gi|4885049|ref|NP_005150.1|	0	actin, alpha cardiac muscle 1 proprotein [Homo sapiens]
mmu-miR-101a-3p	NM_145369	-24.1	0.851848	NA	NA	GO:0004866,GO:0051346,	-	gi|70778837|ref|NP_663344.2|	5.51E-68	WAP four-disulfide core domain 5 precursor [Mus musculus]

mmu-miR-101a-3p	NM_001045521	-23.8	0.667376	NA	NA	GO:0004175,GO:0019538,	K01324|1|6e-51|202|aml:100468355|plasma kallikrein [EC:3.4.21.34]!K09628|2|1e-50|201|rno:287108|protease, serine 27 [EC:3.4.21.-]!K09629|4|4e-50|199|mdo:100018399|protease, serine, 33!K09640|5|5e-50|199|xtr:448231|transmembrane	gi|113865889|ref|NP_001038986.1|	4.23E-169	serine protease 38 precursor [Mus musculus]

mmu-miR-101a-3p	NM_177343	-22.5	0.999982	NA	NA	GO:0043234,GO:0043231,GO:0005515,GO:0004674,GO:0032559,GO:0006915,GO:0006464,	K08794|1|0.0|692|rno:307124|calcium/calmodulin-dependent protein kinase I [EC:2.7.11.17]	gi|74188615|dbj|BAE28053.1|	0	unnamed protein product [Mus musculus]
mmu-miR-101a-3p	NM_178115	-22.1	0.999965	NA	NA	GO:0043231,GO:0010467,	-	gi|295389569|ref|NP_835216.3|	0	erythroid differentiation-related factor 1 [Mus musculus]
mmu-miR-101a-3p	NR_001461	-21.2	1	NA	NA	-	K11849|1|6e-55|213|acs:100555596|ubiquitin carboxyl-terminal hydrolase 25/28 [EC:3.1.2.15]!K06478|4|4e-43|182|acs:100563672|protein tyrosine phosphatase, receptor type, C [EC:3.1.3.4	gi|209553980|gb|AAA39398.2|	0	ORF2 [Mus musculus domesticus]

mmu-miR-101a-3p	NM_023764	-25.8	0.758105	NA	NA	GO:0043235,GO:0044444,GO:0060089,GO:0005102,GO:0019899,GO:0001775,GO:0006952,GO:0007165,GO:0006796,GO:00071	K05402|1|3e-125|451|mmu:54473|toll-interacting protein	gi|13591860|ref|NP_076253.1|	1.86E-124	toll-interacting protein [Mus musculus]
mmu-miR-101a-3p	NR_033575	-21.2	0.809502	NA	NA	GO:0031090,GO:0043231,GO:0005506,GO:0016712,GO:0042493,GO:0042537,	K07426|1|8e-30|130|mmu:13120|cytochrome P450, family 4, subfamily B [EC:1.14.14.1]	gi|148698695|gb|EDL30642.1|	1.37E-33	mCG52000 [Mus musculus]
mmu-miR-101a-3p	NM_019479	-21.2	0.987095	NA	NA	GO:0043231,GO:0043234,GO:0000989,GO:0003676,GO:0001071,GO:0048869,GO:0048731,GO:0006355,	K09087|1|2e-102|373|mmu:55927|hairy and enhancer of split 2/6/7	gi|9506777|ref|NP_062352.1|	1.10E-101	transcription cofactor HES-6 [Mus musculus]

mmu-miR-101a-3p	NM_178779	-20.1	1	NA	NA	-	K15705|1|1e-109|400|mmu:320311|E3 ubiquitin-protein ligase RNF152 [EC:6.3.2.19]	gi|148707931|gb|EDL39878.1|	4.62E-121	ring finger protein 152 [Mus musculus]
mmu-miR-101a-3p	NM_027782	-21.6	0.988644	NA	NA	GO:0005887,GO:0005267,GO:0034220,GO:0030001,	K15074|1|2e-09|64.7|hmg:100213269|BTB/POZ domain-containing adapter for CUL3-mediated RhoA degradation protein	gi|351710048|gb|EHB12967.1|	1.24E-132	BTB/POZ domain-containing protein KCTD6 [Heterocephalus glaber]
mmu-miR-101a-3p	NM_175540	-20.8	1	NA	NA	GO:0016021,GO:0005035,GO:0005488,GO:0043410,GO:0048869,GO:0009888,GO:0051091,GO:0008219,GO:0019221,GO:00072	K05163|1|1e-164|582|mmu:245527|tumor necrosis factor receptor superfamily member 27	gi|148682260|gb|EDL14207.1|	4.38E-166	ectodysplasin A2 isoform receptor, isoform CRA_b [Mus musculus]

mmu-miR-101a-3p	NM_180974	-25	0.937762	NA	NA	-	K09407|1|0.0|818|mmu:14236|forkhead box protein N	gi|163965351|ref|NP_851305.2|	0	forkhead box N2 [Mus musculus]
mmu-miR-101a-3p	NM_171826	-21.8	0.995136	NA	NA	GO:0031224,	-	gi|356995848|ref|NP_001239379.1|	8.87E-161	claudin domain-containing protein 1 isoform 2 [Mus musculus]
mmu-miR-101a-3p	NM_001164184	-20.5	0.999928	NA	NA	GO:0034358,GO:0043231,GO:0016020,GO:0071813,GO:0008289,GO:0004888,GO:0006629,GO:0048513,GO:0015031,	-	gi|1236083|gb|AAA92719.1|	0	Lisch7, partial [Mus musculus]
mmu-miR-101a-3p	NM_001081061	-20.6	1	NA	NA	-	K15198|1|0.0|4487|mmu:544971|transcription factor TFIIIB component B''	gi|145587077|ref|NP_001074530.1|	0	transcription factor TFIIIB component B'' homolog [Mus musculus]
mmu-miR-101a-3p	NR_030709	-20.2	0.951092	NA	NA	-	-	gi|74188359|dbj|BAE25830.1|	7.11E-47	unnamed protein product [Mus musculus]

mmu-miR-101a-3p	NM_001161823	-21.4	0.999996	NA	NA	GO:0005815,GO:0031981,GO:0046914,GO:0016706,GO:0016701,GO:0051704,GO:0070076,GO:0006351,	K06709|1|0.0|1993|mmu:230674|jumonji domain-containing protein 2 [EC:1.14.11.-]	gi|37360038|dbj|BAC97997.1|	0	mKIAA0677 protein [Mus musculus]
mmu-miR-101a-3p	NM_028227	-20.9	0.999998	NA	NA	-	K10632|1|0.0|1164|mmu:72399|BRCA1-associated protein [EC:6.3.2.19]	gi|70608139|ref|NP_082503.2|	0	BRCA1-associated protein [Mus musculus]
mmu-miR-101a-3p	NM_001081252	-21.2	1	NA	NA	GO:0044432,GO:0035251,GO:0006487,	K11718|1|0.0|2798|mmu:66435|UDP-glucose:glycoprotein glucosyltransferase [EC:2.4.1.-]	gi|158749642|ref|NP_001074721.2|	0	UDP-glucose:glycoprotein glucosyltransferase 2 precursor [Mus musculus]

mmu-miR-101a-3p	NM_028956	-20.8	0.999993	NA	NA	GO:0003676,GO:0016866,GO:0009451,GO:0006399,	K07583|1|0.0|1027|mmu:74467|tRNA pseudouridine synthase 10 [EC:5.4.99.-]	gi|148675947|gb|EDL07894.1|	0	RIKEN cDNA 4933435A13, isoform CRA_b [Mus musculus]
mmu-miR-101a-3p	NM_001099624	-20.4	1	NA	NA	GO:0016021,GO:0005088,GO:0060089,GO:0046872,GO:0008289,GO:0005099,GO:0019935,GO:0007243,GO:0009966,	K08018|1|0.0|2651|mmu:76089|Rap guanine nucleotide exchange factor (GEF) 2	gi|44890797|gb|AAH67056.1|	0	Rapgef2 protein [Mus musculus]
mmu-miR-101a-3p	NM_025711	-21.7	0.997491	NA	NA	GO:0016021,GO:0031012,GO:0046872,GO:0022829,GO:0006810,GO:0001503,GO:0070168,GO:0007179,	K08120|1|0.0|727|mmu:66695|asporin	gi|27754118|ref|NP_079987.2|	0	asporin preproprotein [Mus musculus]

mmu-miR-101a-3p	NM_008886	-21.3	0.999937	NA	NA	GO:0032300,GO:0043231,GO:0017111,GO:0032135,GO:0004518,GO:0032404,GO:0032559,GO:0006281,GO:0002566,GO:0009207,GO:0042221,GO:0022403,GO:00025	K10858|1|0.0|1680|mmu:18861|DNA mismatch repair protein PMS2	gi|121583910|ref|NP_032912.2|	0	mismatch repair endonuclease PMS2 [Mus musculus]
mmu-miR-101a-3p	NM_146184	-22.2	0.976646	NA	NA	GO:0043231,GO:0031224,GO:0008375,GO:0006464,GO:0006023,	K09665|1|0.0|770|mmu:232984|beta-1,3-N-acetylglucosaminyltransferase 8 [EC:2.4.1.-]	gi|22122729|ref|NP_666296.1|	0	UDP-GlcNAc:betaGal beta-1,3-N-acetylglucosaminyltransferase 8 [Mus musculus]
mmu-miR-101a-3p	NM_153516	-21	1	NA	NA	GO:0043231,GO:0031224,GO:0016505,GO:0006915,GO:0012502,	K15485|1|0.0|719|mmu:94044|Bcl-2-like protein 13	gi|23943828|ref|NP_705736.1|	0	bcl-2-like protein 13 [Mus musculus]
mmu-miR-101a-3p	NM_029870	-22.4	0.999993	NA	NA	GO:0003677,GO:0001071,GO:0006351,	-	gi|30794196|ref|NP_084146.1|	0	CREB3 regulatory factor [Mus musculus]

mmu-miR-101a-3p	NR_046178	-20.5	0.999578	NA	NA	-	-	gi|148687840|gb|EDL19787.1|	2.61E-69	mCG145322 [Mus musculus]
mmu-miR-101a-3p	NM_008493	-20.8	0.999994	NA	NA	GO:0044421,GO:0051427,GO:0006915,GO:0006357,GO:0019229,GO:0043434,GO:0007631,GO:0032275,GO:0030282,GO:0015980,GO:0019935,GO:0006811,GO:0007159,GO:0016125,GO:0072593,GO:0032891,GO:0019221,GO:0030073,GO:0032096,GO:0045597,GO:0045927,GO:00060	K05424|1|5e-80|300|mmu:16846|leptin	gi|6678678|ref|NP_032519.1|	3.52E-79	leptin precursor [Mus musculus]

mmu-miR-101a-3p	NM_001167730	-24.8	0.800678	NA	NA	GO:0001739,GO:0000217,GO:0046914,GO:0019899,GO:0003824,GO:0009416,GO:0033554,GO:0000018,GO:0048232,	K10627|1|0.0|935|mmu:58186|E3 ubiquitin-protein ligase RAD18 [EC:6.3.2.19]	gi|148667015|gb|EDK99431.1|	0	RAD18 homolog (S. cerevisiae), isoform CRA_c [Mus musculus]
mmu-miR-101a-3p	NM_001083320	-21.6	1	NA	NA	GO:0043296,GO:0043231,GO:0032559,	K05631|1|0.0|2283|mmu:14924|atrophin-1 interacting protein 3 (BAI1-associated protein 1)	gi|71533173|ref|NP_001025021.1|	0	membrane-associated guanylate kinase, WW and PDZ domain-containing protein 1 isoform c [Mus musculus]
mmu-miR-101a-3p	NM_198942	-24.3	0.974259	NA	NA	-	K13026|1|0.0|2435|mmu:106794|ATP-dependent RNA helicase DHX57 [EC:3.6.4.13]	gi|254939651|ref|NP_945180.2|	0	putative ATP-dependent RNA helicase DHX57 isoform 2 [Mus musculus]

mmu-miR-101a-3p	NM_001081418	-20.8	1	NA	NA	GO:0005488,	K05626|1|6e-06|55.1|rno:29515|atrophin-1 (dentatorubral-pallidoluysian atrophy protein)	gi|124487237|ref|NP_001074887.1|	0	glioma tumor suppressor candidate region gene 1 protein [Mus musculus]
mmu-miR-101a-3p	NM_001033436	-22.2	0.994073	NA	NA	-	K11318|1|0.0|1227|mmu:380753|ataxin-7	gi|295317311|ref|NP_001028608.3|	0	ataxin-7-like protein 1 isoform 1 [Mus musculus]

mmu-miR-101a-3p	NM_008092	-20.6	0.999999	NA	NA	GO:0031981,GO:0046914,GO:0001071,GO:0043565,GO:0008134,GO:0051094,GO:0060411,GO:0007599,GO:0042127,GO:0007369,GO:0048562,GO:0009952,GO:0002065,GO:0008406,GO:0048545,GO:0035050,GO:0043009,GO:2000026,GO:0007154,GO:0003283,GO:0048522,GO:00328	K09183|1|5e-170|599|mmu:14463|GATA-binding protein 4/5/6	gi|46909569|ref|NP_032118.2|	3.82E-169	transcription factor GATA-4 [Mus musculus]

mmu-miR-101a-3p	NM_001172481	-21.7	0.997387	NA	NA	GO:0016021,GO:0031012,GO:0046872,GO:0022829,GO:0006810,GO:0001503,GO:0070168,GO:0007179,	K08120|1|0.0|727|mmu:66695|asporin	gi|27754118|ref|NP_079987.2|	0	asporin preproprotein [Mus musculus]

mmu-miR-101a-3p	NM_183220	-23.6	0.872971	NA	NA	GO:0042802,GO:0016740,GO:0048037,GO:0016846,GO:0008652,	K00815|1|5e-13|77.0|tad:TRIADDRAFT_35383|tyrosine aminotransferase [EC:2.6.1.5]!K00816|3|7e-12|73.2|tsp:Tsp_07645|kynurenine---oxoglutarate transaminase / cysteine-S-conjugate beta-lyase / glutamine---phenylpyruvate	gi|167017114|sp|A2AIG8.1|1A1L1_MOUSE	0	RecName: Full=1-aminocyclopropane-1-carboxylate synthase-like protein 1; Short=ACC synthase-like protein 1
mmu-miR-101a-3p	NM_008533	-21.6	0.997942	NA	NA	GO:0031224,GO:0004871,GO:0005488,GO:0006952,GO:0023052,	K06555|1|0.0|1179|mmu:17079|lymphocyte antigen 78	gi|117320545|ref|NP_032559.2|	0	CD180 antigen precursor [Mus musculus]

mmu-miR-101a-3p	NM_013471	-23.9	0.833587	NA	NA	GO:0044424,GO:0046872,GO:0005543,GO:0055102,GO:0016192,GO:0043066,GO:00230	-	gi|161016799|ref|NP_038499.2|	6.06E-177	annexin A4 [Mus musculus]
mmu-miR-101a-3p	NM_028981	-21.2	1	NA	NA	GO:0034704,GO:0044459,GO:0005245,GO:0015980,GO:0007611,GO:0032412,GO:0050954,GO:0070838,GO:0030073,GO:0006935,GO:0007187,	K04851|1|0.0|3754|mmu:12289|voltage-dependent calcium channel L type alpha-1D	gi|134288900|ref|NP_083257.2|	0	voltage-dependent L-type calcium channel subunit alpha-1D isoform 1 [Mus musculus]
mmu-miR-101a-3p	NM_139200	-25.2	0.941643	NA	NA	GO:0005768,GO:0005488,GO:0007155,	K08789|1|2e-09|67.0|dre:100535762|microtubule-associated serine/threonine kinase [EC:2.7.11.1]	gi|21105855|ref|NP_631939.1|	6.72E-177	cytohesin-interacting protein [Mus musculus]

mmu-miR-101a-3p	NM_001198826	-20.1	1	NA	NA	GO:0031224,GO:0031982,GO:0044430,GO:0012505,GO:0043231,GO:0045202,GO:0030425,GO:0044444,GO:0005102,GO:0005539,GO:0008047,GO:0019904,GO:0003676,GO:0043169,GO:0004866,GO:0016198,GO:0006915,GO:0006357,GO:0007346,GO:0007631,GO:0007416,GO:00425	K04520|1|0.0|875|mmu:11820|amyloid beta (A4) protein	gi|311893408|ref|NP_001185755.1|	0	amyloid beta A4 protein isoform 6 precursor [Mus musculus]
mmu-miR-101a-3p	NM_001039389	-22.5	0.998368	NA	NA	-	-	gi|86476061|ref|NP_001034478.1|	5.24E-90	WD repeat-containing protein 37 isoform b [Mus musculus]
mmu-miR-101a-3p	NM_001271002	-20	0.999724	NA	NA	-	-	-	-	-

mmu-miR-101a-3p	NM_008523	-20.5	0.999961	NA	NA	GO:0043231,GO:0016021,GO:0004871,GO:0004713,GO:0032559,GO:0007167,GO:0016310,GO:0006464,	K05118|1|0.0|1037|mmu:17005|leukocyte tyrosine kinase [EC:2.7.10.1]	gi|159032532|ref|NP_996825.2|	0	leukocyte tyrosine kinase receptor isoform C precursor [Mus musculus]
mmu-miR-101a-3p	NM_009557	-20.9	1	NA	NA	GO:0043231,GO:0046914,GO:0003676,GO:0006351,	K09228|1|0.0|981|mmu:22704|KRAB domain-containing zinc finger protein	gi|50511065|dbj|BAD32518.1|	0	mKIAA1710 protein [Mus musculus]
mmu-miR-101a-5p	NM_001008423	-33.8	0.077496	-28.44	175	-	-	gi|119601406|gb|EAW81000.1|	2.12E-66	hCG1644165 [Homo sapiens]
mmu-miR-101a-5p	NM_001011825	-29.4	0.072333	-26.27	178	GO:0004888,GO:0023052,	K04257|1|9e-91|333|mcc:707282|olfactory receptor	gi|58801414|ref|NP_001011825.1|	4.03E-176	olfactory receptor 1105 [Mus musculus]
mmu-miR-101a-5p	NM_001042438	-30.7	0.303238	-26.78	175	GO:0043231,GO:0046914,GO:0001071,GO:0003677,GO:0006355,	-	gi|60360160|dbj|BAD90299.1|	0	mKIAA4149 protein [Mus musculus]

mmu-miR-101a-5p	NM_001127189	-27.7	0.472524	-25.46	165	-	K09228|1|0.0|1191|mmu:100041677|KRAB domain-containing zinc finger protein	gi|187608663|ref|NP_001120661.1|	0	uncharacterized protein LOC100041677 [Mus musculus]
mmu-miR-101a-5p	NM_001193266	-28.1	0.858365	-25.95	168	GO:0031224,GO:0021515,	K06762|1|4e-32|142|hsa:152330|contactin 4	gi|300863061|ref|NP_001180195.1|	0	MAM domain-containing glycosylphosphatidylinositol anchor protein 2 isoform A [Mus musculus]
mmu-miR-101a-5p	NM_001256100	-31.5	0.224944	-29.71	168	GO:0043231,GO:0043021,GO:0008135,GO:0006413,GO:0032988,	K02520|1|2e-148|528|mmu:76366|translation initiation factor IF-	gi|21313176|ref|NP_083857.1|	1.30E-147	translation initiation factor IF-3, mitochondrial [Mus musculus]
mmu-miR-101a-5p	NM_001256101	-31.5	0.224466	-29.71	168	GO:0043231,GO:0043021,GO:0008135,GO:0006413,GO:0032988,	K02520|1|2e-148|528|mmu:76366|translation initiation factor IF-	gi|21313176|ref|NP_083857.1|	1.30E-147	translation initiation factor IF-3, mitochondrial [Mus musculus]

mmu-miR-101a-5p	NM_001256102	-31.5	0.217482	-29.71	168	GO:0043231,GO:0043021,GO:0008135,GO:0006413,GO:0032988,	K02520|1|2e-148|528|mmu:76366|translation initiation factor IF-	gi|21313176|ref|NP_083857.1|	1.25E-147	translation initiation factor IF-3, mitochondrial [Mus musculus]
mmu-miR-101a-5p	NM_009572	-30.7	0.296518	-26.78	175	GO:0043231,GO:0046914,GO:0001071,GO:0003677,GO:0006355,	-	gi|60360160|dbj|BAD90299.1|	0	mKIAA4149 protein [Mus musculus]
mmu-miR-101a-5p	NM_029288	-26.1	0.332471	-25.28	171	-	-	gi|309270198|ref|XP_001477082.2|	1.00E-114	PREDICTED: uncharacterized protein LOC100041651 isoform 1 [Mus musculus]
mmu-miR-101a-5p	NM_146867	-27.9	0.145801	-26.35	161	GO:0004871,GO:0023052,	K04257|1|4e-167|587|mmu:258867|olfactory	gi|22129143|ref|NP_667078.1|	2.93E-166	olfactory receptor 131 [Mus musculus]

mmu-miR-101a-5p	NM_172554	-28.1	0.606377	-26.43	168	GO:0031224,GO:0000139,GO:0031982,GO:0046914,GO:0060089,GO:0016409,GO:0046873,GO:0007249,GO:0006497,GO:0070838,GO:0015031,	K11420|1|2e-23|113|oaa:100078328|euchromatic histone-lysine N-methyltransferase [EC:2.1.1.43]	gi|4589536|dbj|BAA76790.1|	0	KIAA0946 protein [Homo sapiens]
mmu-miR-101a-5p	NM_207010	-28.1	0.857829	-25.95	168	GO:0031224,GO:0021515,	K06762|1|4e-32|142|hsa:152330|contactin 4	gi|300863063|ref|NP_996893.2|	0	MAM domain-containing glycosylphosphatidylinositol anchor protein 2 isoform B [Mus musculus]
mmu-miR-101a-5p	NR_029537	-35.6	0.000013	-33.81	186	-	-	-	-	-
mmu-miR-101a-5p	NR_039546	-37.4	0.000003	-34.99	186	-	-	-	-	-
mmu-miR-101a-5p	NR_040708	-35.6	0.002415	-33.81	186	-	-	gi|148698926|gb|EDL30873.1|	3.90E-42	mCG148069 [Mus musculus]
mmu-miR-101a-5p	NR_045296	-31.9	0.008996	-29.39	185	-	-	gi|148686391|gb|EDL18338.1|	1.11E-29	mCG1033006 [Mus musculus]

mmu-miR-101a-5p	NM_011663	-21.5	1	-45.2	323	GO:0005681,GO:0003729,GO:0046914,GO:0000398,	K03105|1|2e-118|428|ptr:737811|signal recognition particle subunit SRP19!K00816|2|8e-28|127|smm:Smp_123750|kynurenine---oxoglutarate transaminase / cysteine-S-conjugate beta-lyase / glutamine---phenylpyruvate	gi|6755917|ref|NP_035793.1|	3.99E-161	U2 small nuclear ribonucleoprotein auxiliary factor 35 kDa subunit-related protein 1 [Mus musculus]
mmu-miR-101a-5p	NM_020506	-27.5	0.594036	-24.8	184	GO:0043231,GO:0005488,GO:0006810,	-	gi|10048438|ref|NP_065252.1|	0	exportin-4 [Mus musculus]

mmu-miR-101a-5p	NM_016980	-28	0.329492	-23.19	178	GO:0043234,GO:0031981,GO:0015934,GO:0019843,GO:0005198,GO:0006412,GO:0019080,GO:0010035,GO:0031016,GO:0042254,GO:0010921,	K02932|1|1e-157|557|ptr:457025|large subunit ribosomal protein L5e	gi|390461662|ref|XP_002746579.2|	1.21E-156	PREDICTED: 60S ribosomal protein L5 [Callithrix jacchus]
mmu-miR-101a-5p	NR_015483	-25.1	0.850598	-23.02	175	-	-	gi|148693587|gb|EDL25534.1|	7.18E-32	mCG147890 [Mus musculus]
mmu-miR-101a-5p	NM_026213	-21	0.999829	-23.82	173	-	K07198|1|1e-15|85.9|mgp:100034740|5'-AMP-activated protein kinase, catalytic alpha subunit [EC:2.7.11.11]!K09553|2|7e-06|53.1|tca:655405|stress-induced-phosphoprotein 1	gi|21312920|ref|NP_080489.1|	3.55E-136	tetratricopeptide repeat protein 33 [Mus musculus]

mmu-miR-101a-5p	NM_181649	-26.4	0.830664	-21.66	172	GO:0044464,GO:0005488,	-	gi|194473718|ref|NP_857632.4|	3.31E-133	coiled-coil domain-containing protein 75 [Mus musculus]
mmu-miR-101a-5p	NM_177630	-26.9	0.77847	-24.16	171	-	-	gi|110681693|ref|NP_808298.2|	5.94E-133	protein LDOC1L [Mus musculus]
mmu-miR-101a-5p	NM_153542	-25.6	0.789564	-23.34	170	-	K10641|1|6e-10|67.4|aga:AgaP_AGAP005053|E3 ubiquitin-protein ligase LRSAM1 [EC:6.3.2.19]!K01090|3|6e-09|63.9|bfo:BRAFLDRAFT_214316|protein phosphatase [EC:3.1.3.16]	gi|23956308|ref|NP_705770.1|	6.54E-97	leucine-rich repeat-containing protein 20 [Mus musculus]
mmu-miR-101a-5p	NM_178141	-25.9	0.712095	-21.14	169	-	-	gi|148688656|gb|EDL20603.1|	1.20E-65	RIKEN cDNA 3830406C13, isoform CRA_b [Mus musculus]

mmu-miR-101a-5p	NM_207668	-24.5	0.984819	-20.96	168	GO:0005770,GO:0005795,GO:0016023,GO:0005764,GO:0043169,GO:0016791,GO:00067	K14410|1|0.0|689|mmu:56318|acid phosphatase [EC:3.1.3.2]	gi|148689143|gb|EDL21090.1|	0	acid phosphatase, prostate, isoform CRA_b [Mus musculus]
mmu-miR-101a-5p	NR_027805	-21	0.999948	-20.02	167	-	-	gi|148709434|gb|EDL41380.1|	3.66E-63	RIKEN cDNA A430093F15, isoform CRA_b [Mus musculus]
mmu-miR-101a-5p	NR_015527	-26.5	0.313499	-23.13	166	-	-	gi|148667406|gb|EDK99822.1|	1.54E-31	mCG1037138 [Mus musculus]
mmu-miR-101a-5p	NM_181407	-27.6	0.667696	-20.1	165	GO:0044429,GO:0043169,GO:0004470,GO:0000166,GO:0045333,GO:0032787,GO:0043648,	K00029|1|0.0|1193|mmu:109264|malate dehydrogenase (oxaloacetate-decarboxylating)(NADP+) [EC:1.1.1.40]	gi|225579045|ref|NP_852072.2|	0	NADP-dependent malic enzyme, mitochondrial [Mus musculus]
mmu-miR-101a-5p	NM_138304	-25.3	0.392816	-23.57	164	GO:0046872,	K02183|1|1e-28|127|ptr:456153|calmodulin	gi|148694085|gb|EDL26032.1|	5.31E-92	calmodulin-like 4, isoform CRA_c [Mus musculus]
mmu-miR-101a-5p	NR_015526	-21.6	1	-23.97	163	-	-	gi|148672931|gb|EDL04878.1|	7.98E-19	mCG147136 [Mus musculus]

mmu-miR-101a-5p	NR_045083	-26	0.904224	-22.47	162	-	-	gi|148686518|gb|EDL18465.1|	4.68E-51	mCG142514, isoform CRA_a [Mus musculus]
mmu-miR-101a-5p	NM_181347	-21.6	1	-20.84	161	GO:0030135,GO:0030695,	K01289|1|8e-67|258|dme:Dmel_CG18659|carboxypeptidase [EC:3.4.16.-]!K12861|2|2e-28|130|gga:419884|pre-mRNA-splicing factor SPF27	gi|262073029|ref|NP_001159973.1|	0	DENN domain-containing protein 1B isoform 1 [Mus musculus]
mmu-miR-101a-5p	NM_177604	-25.9	0.783027	-22.13	160	GO:0016020,GO:0044424,GO:0004871,GO:0023052,	-	gi|162417980|ref|NP_808272.2|	0	specifically androgen-regulated gene protein [Mus musculus]
mmu-miR-101a-5p	NM_172832	-25.6	0.690877	-24.17	159	GO:0016667,GO:0030329,	K05906|1|0.0|811|ecb:100060629|prenylcysteine oxidase / farnesylcysteine lyase [EC:1.8.3.5 1.8.3.6]	gi|27370248|ref|NP_766420.1|	0	prenylcysteine oxidase-like precursor [Mus musculus]

mmu-miR-101a-5p	NM_175290	-29.8	0.289477	-20.86	158	GO:0032559,GO:0006950,	K12800|1|4e-111|404|mcc:701278|NACHT, LRR and PYD domains-containing protein 3	gi|140972011|ref|NP_780499.3|	0	NACHT, LRR and PYD domains-containing protein 4F [Mus musculus]
mmu-miR-101a-5p	NR_045071	-21.3	1	-21.51	157	-	-	gi|1791243|gb|AAB41224.1|	7.63E-09	ORF2 [Rattus norvegicus]
mmu-miR-101a-5p	NM_198170	-21	1	-20.04	156	GO:0007399,	-	gi|253314408|ref|NP_937813.3|	0	protein SZT2 [Mus musculus]
mmu-miR-101a-5p	NM_025727	-24.9	0.799812	-21.02	155	GO:0044424,GO:0007281,GO:0001894,GO:0035112,GO:0032989,GO:0008406,	K10448|1|0.0|1257|mdo:100012063|kelch-like protein 10	gi|395826410|ref|XP_003786411.1|	0	PREDICTED: kelch-like protein 10 isoform 1 [Otolemur garnettii]

mmu-miR-101a-5p	NR_003564	-25.5	0.342525	-22	154	GO:0000153,GO:0031981,GO:0008135,GO:0005515,GO:0019787,GO:0006412,GO:0006357,GO:0032446,GO:0065003,GO:0045935,GO:0006354,	K03873|1|4e-34|145|rno:81807|transcription elongation factor B, polypeptide 2	gi|151358120|emb|CAO77939.1|	2.40E-83	novel protein [Mus musculus]
mmu-miR-101a-5p	NM_028004	-25.9	1	-20.96	153	-	K12567|1|0.0|4.870e+04|hsa:7273|titin [EC:2.7.11.1]	gi|77812699|ref|NP_082280.2|	0	titin isoform N2-B [Mus musculus]
mmu-miR-101a-5p	NR_027357	-25.9	0.836066	-21.31	152	GO:0019866,GO:0031224,GO:0043231,GO:0006810,	K15121|1|1e-177|625|mmu:229517|solute carrier family 25, member 44	gi|148683335|gb|EDL15282.1|	0	RIKEN cDNA B430110G05, isoform CRA_b [Mus musculus]

mmu-miR-101a-5p	NM_133360	-21.1	1	-20.23	150	GO:0043231,GO:0044444,GO:0019842,GO:0043169,GO:0016879,GO:0016421,GO:0032559,GO:0009893,GO:0006631,GO:0035336,GO:0006641,GO:0048878,GO:0051260,GO:0019538,GO:0015980,GO:0048871,GO:00	K11262|1|0.0|4551|mmu:107476|acetyl-CoA carboxylase / biotin carboxylase [EC:6.4.1.2 6.3.4.14]	gi|125656173|ref|NP_579938.2|	0	acetyl-CoA carboxylase 1 [Mus musculus]
mmu-miR-101a-5p	NM_177806	-26.4	0.820548	-20.61	149	GO:0031981,GO:0006396,	K13217|1|0.0|1284|mmu:328110|pre-mRNA-processing factor 39	gi|156546892|ref|NP_808474.2|	0	pre-mRNA-processing factor 39 [Mus musculus]

mmu-miR-101a-5p	NM_010546	-22.8	0.999827	-20.21	147	GO:0043234,GO:0005887,GO:0044444,GO:0046983,GO:0004674,GO:0032559,GO:0006941,GO:0042221,GO:0050851,GO:0051091,GO:0002224,GO:0007169,GO:0006468,GO:0007249,GO:0002260,GO:00430	K07209|1|0.0|1446|mmu:16150|inhibitor of nuclear factor kappa-B kinase subunit beta [EC:2.7.11.10]	gi|148700933|gb|EDL32880.1|	0	inhibitor of kappaB kinase beta, isoform CRA_a [Mus musculus]
mmu-miR-101a-5p	NM_021361	-21.1	1	-20.65	146	GO:0043231,GO:0003723,GO:0000377,GO:0048731,GO:0007267,GO:0007610,	K14944|1|1e-145|519|mmu:664883|RNA-binding protein Nova	gi|256017197|ref|NP_067336.1|	8.37E-145	RNA-binding protein Nova-1 [Mus musculus]
mmu-miR-101a-5p	NR_046026	NA	NA	-23.65	162	-	-	gi|148665603|gb|EDK98019.1|	1.31E-64	mCG144482 [Mus musculus]
mmu-miR-101a-5p	NM_011652	NA	NA	-20.96	153	-	K12567|1|0.0|4.370e+04|mmu:22138|titin [EC:2.7.11.	gi|77812697|ref|NP_035782.3|	0	titin isoform N2-A [Mus musculus]

mmu-miR-101a-5p	NM_016917	NA	NA	-23.15	152	GO:0016021,GO:0030136,GO:0005488,GO:0022890,GO:0012501,GO:0006875,GO:0060343,GO:0048534,GO:0006826,GO:0060429,GO:0001776,GO:0048871,	K14685|1|0.0|1054|mmu:53945|solute carrier family 40 (iron-regulated transporter), member 1	gi|124248585|ref|NP_058613.2|	0	solute carrier family 40 member 1 [Mus musculus]
mmu-miR-101a-5p	NM_008626	NA	NA	-20.81	149	GO:0031224,GO:0004871,GO:0030246,GO:0016192,GO:0023052,	K06560|1|0.0|3009|mmu:17534|mannose receptor, C type	gi|113930720|ref|NP_032652.3|	0	C-type mannose receptor 2 precursor [Mus musculus]
mmu-miR-101a-5p	NM_019583	-24.2	0.896153	NA	NA	GO:0016021,GO:0044424,GO:0004888,GO:0007166,GO:0006950,GO:0016049,	K05165|1|0.0|971|mmu:50905|interleukin 17 receptor B	gi|148692799|gb|EDL24746.1|	0	interleukin 17 receptor B, isoform CRA_b [Mus musculus]

mmu-miR-101a-5p	NM_053201	-20.2	1	NA	NA	GO:0043005,GO:0016020,GO:0043234,GO:0044444,GO:0043231,GO:0005488,	K04976|1|1e-43|179|ssc:100158130|transient receptor potential cation channel subfamily M member 1!K12464|3|1e-39|166|ssc:414852|melanoma-associate	gi|111378377|ref|NP_444431.3|	0	melanoma-associated antigen E1 [Mus musculus]
mmu-miR-101a-5p	NM_001037922	-22.2	0.998402	NA	NA	GO:0031981,GO:0005488,GO:0019887,GO:0051726,GO:0019222,GO:0051320,GO:0016310,	K02219|1|6e-40|166|mdo:100021385|cyclin-dependent kinase regulatory subunit CKS1	gi|148679055|gb|EDL11002.1|	6.80E-53	mCG132387 [Mus musculus]
mmu-miR-101a-5p	NM_144511	-20.2	0.999999	NA	NA	GO:0005783,GO:0004091,GO:0051969,	K15743|1|0.0|1003|mmu:13909|carboxylesterase 3/5 [EC:3.1.1.1]	gi|226874914|ref|NP_653094.2|	0	carboxylesterase 3B isoform 1 precursor [Mus musculus]

mmu-miR-101a-5p	NM_001099792	-26.1	0.631482	NA	NA	GO:0043231,GO:0016426,GO:0005488,GO:0006399,GO:0001510,	K07442|1|3e-169|596|mmu:328162|tRNA (adenine57-N1/adenine58-N1)-methyltransferase [EC:2.1.1.219 2.1.1.220]	gi|152963551|ref|NP_796348.2|	1.91E-168	tRNA (adenine(58)-N(1))-methyltransferase catalytic subunit TRMT61A [Mus musculus]
mmu-miR-101a-5p	NM_018771	-22.6	0.975801	NA	NA	GO:0030425,GO:0012505,GO:0030136,GO:0008047,GO:0008092,GO:0019904,GO:0042802,GO:0043087,GO:0023061,GO:0007179,GO:0006886,GO:0031330,GO:0010608,GO:0050804,GO:0016477,	-	gi|9055336|ref|NP_061241.1|	9.45E-162	PDZ domain-containing protein GIPC1 [Mus musculus]
mmu-miR-101a-5p	NR_028298	-23.2	0.835489	NA	NA	-	-	-	-	-

mmu-miR-101a-5p	NM_013910	-22.7	0.999336	NA	NA	GO:0043231,GO:0003723,GO:0046914,GO:0016706,GO:0070076,GO:0006351,	K10276|1|0.0|1330|mmu:30841|F-box and leucine-rich repeat protein 10/11 [EC:1.14.11.27]	gi|39930339|ref|NP_038938.1|	0	lysine-specific demethylase 2B isoform 2 [Mus musculus]
mmu-miR-101a-5p	NM_183285	-20.5	0.999977	NA	NA	GO:0005887,GO:0005267,GO:0030001,	K08023|1|1e-08|62.0|aml:100472196|latent transforming growth factor beta binding	gi|397484499|ref|XP_003813412.1|	9.83E-99	PREDICTED: BTB/POZ domain-containing protein KCTD2 [Pan paniscus]
mmu-miR-101a-5p	NM_001252568	-20.6	0.999453	NA	NA	GO:0016701,GO:0043169,GO:0008152,	K00477|1|6e-06|52.8|dre:550521|phytanoyl-CoA hydroxylase [EC:1.14.11.18]	gi|357588425|ref|NP_001239497.1|	4.03E-170	phytanoyl-CoA dioxygenase domain-containing protein 1 isoform 2 [Mus musculus]

mmu-miR-101a-5p	NM_207031	-21.8	0.999952	NA	NA	GO:0044459,GO:0044444,GO:0034702,GO:0005253,GO:0015698,	K13982|1|6e-31|137|dgr:Dgri_GH10918|probable ATP-dependent RNA helicase DDX4 [EC:3.6.4.13]!K05169|2|7e-18|94.0|ptr:743996|single Ig IL-1R-related molecule	gi|46309609|ref|NP_996914.1|	0	anoctamin-7 [Mus musculus]
mmu-miR-101a-5p	NM_173428	-20.4	1	NA	NA	GO:0044421,GO:0009987,	K03900|1|4e-169|598|xtr:100492314|von Willebrand factor	gi|345842432|ref|NP_775604.3|	0	SCO-spondin precursor [Mus musculus]

mmu-miR-101a-5p	NM_001114098	-26.8	0.918705	NA	NA	-	K10388|1|2e-09|67.0|dre:567310|plectin!K10352|5|2e-08|63.9|smm:Smp_085540.6|myosin heavy chain	gi|166235131|ref|NP_766551.3|	0	coiled-coil domain-containing protein 165 isoform 2 [Mus musculus]
mmu-miR-101a-5p	NM_025644	-21.3	0.998832	NA	NA	GO:0043234,GO:0031981,GO:0044444,GO:0003676,GO:0004527,GO:0000291,GO:0042254,GO:0010467,	K07573|1|4e-107|389|rno:679140|exosome complex component CSL4	gi|22267446|ref|NP_079920.1|	2.27E-107	exosome complex component CSL4 isoform 1 [Mus musculus]
mmu-miR-101a-5p	NR_046208	-22.8	0.958594	NA	NA	-	-	gi|148675217|gb|EDL07164.1|	2.21E-72	mCG13839 [Mus musculus]

mmu-miR-101a-5p	NM_001163457	-21.8	0.999997	NA	NA	GO:0044432,GO:0005903,GO:0044459,GO:0031982,GO:0005902,GO:0022892,GO:0046983,GO:0055092,GO:0010038,GO:0016125,GO:0010876,GO:0006639,GO:0015031,GO:00	K14463|1|0.0|1736|mmu:17777|microsomal triglyceride transfer protein large subunit	gi|254540023|ref|NP_001156929.1|	0	microsomal triglyceride transfer protein large subunit isoform 1 precursor [Mus musculus]

mmu-miR-101a-5p	NM_023612	-27.1	0.47478	NA	NA	GO:0019838,GO:0005102,GO:0016049,	K08785|1|1e-07|59.3|acs:100560592|HtrA serine peptidase 3 [EC:3.4.21.-]!K08786|2|4e-07|57.8|bta:514946|HtrA serine peptidase 4 [EC:3.4.21.-]!K08784|3|8e-07|56.6|dre:565082|HtrA serine peptidase 1 [EC:3.4.21.	gi|14196342|ref|NP_076101.1|	1.20E-101	endothelial cell-specific molecule 1 precursor [Mus musculus]

mmu-miR-101a-5p	NM_181421	-21	1	NA	NA	-	K10595|1|1e-32|145|mdo:100012223|E3 ubiquitin-protein ligase HERC2 [EC:6.3.2.19]!K10594|4|2e-32|144|spu:592249|E3 ubiquitin-protein ligase HERC1 [EC:6.3.2.19]	gi|359718915|ref|NP_852086.3|	0	probable E3 ubiquitin-protein ligase C12orf51 homolog [Mus musculus]
mmu-miR-101a-5p	NM_001144855	-20.8	1	NA	NA	-	K10352|1|2e-14|83.6|ecb:100054482|myosin heavy chain	gi|221554491|ref|NP_001138327.1|	0	liprin-alpha-4 [Mus musculus]
mmu-miR-101a-5p	NM_172721	-20.8	1	NA	NA	GO:0005488,GO:0048568,	K10264|1|0.0|1045|mmu:231672|F-box and WD-40 domain protein 8	gi|120407054|ref|NP_766309.2|	0	F-box/WD repeat-containing protein 8 [Mus musculus]

mmu-miR-101a-5p	NM_177582	-22.1	0.998193	NA	NA	GO:0005739,GO:0016020,GO:0005488,GO:0051169,GO:0006351,	K09113|1|0.0|855|mmu:208104|MAX-like protein X	gi|148687690|gb|EDL19637.1|	0	MLX interacting protein, isoform CRA_b [Mus musculus]
mmu-miR-101a-5p	NM_008427	-20.2	0.999999	NA	NA	GO:0043005,GO:0044297,GO:0005887,GO:0005249,GO:0019904,GO:0007267,GO:0034220,GO:0030001,	K04998|1|0.0|729|mmu:16520|potassium inwardly-rectifying channel subfamily J member 4	gi|158711668|ref|NP_032453.3|	0	inward rectifier potassium channel 4 [Mus musculus]

mmu-miR-101a-5p	NM_010612	-22.2	0.999998	NA	NA	GO:0042995,GO:0016021,GO:0044424,GO:0044297,GO:0004714,GO:0005102,GO:0032559,GO:0031072,GO:0051704,GO:0010720,GO:0021700,GO:0001935,GO:0001763,GO:0048169,GO:0048858,GO:0008284,GO:0008406,GO:0045765,GO:0048534,GO:0007169,GO:0043523,GO:00423	K05098|1|0.0|2645|mmu:16542|kinase insert domain protein receptor [EC:2.7.10.1]	gi|27777648|ref|NP_034742.2|	0	vascular endothelial growth factor receptor 2 precursor [Mus musculus]
mmu-miR-101a-5p	NM_001163756	-21	0.999919	NA	NA	GO:0004091,GO:0051969,GO:0006464,GO:0010033,	K03927|1|0.0|1133|mmu:234673|carboxylesterase 2 [EC:3.1.1.1 3.1.1.84 3.1.1.56]	gi|148679291|gb|EDL11238.1|	0	carboxylesterase 5, isoform CRA_a [Mus musculus]

mmu-miR-101a-5p	NM_001271616	-20.3	0.999782	NA	NA	-	-	-	-	-
mmu-miR-101a-5p	NM_011351	-20.5	1	NA	NA	GO:0031224,GO:0004871,GO:0005102,GO:0006935,GO:0048731,GO:0023052,GO:00488	K06842|1|0.0|1567|mmu:20360|semaphorin 6	gi|6755464|ref|NP_035481.1|	0	semaphorin-6C precursor [Mus musculus]
mmu-miR-101a-5p	NM_030559	-20.8	1	NA	NA	GO:0005770,GO:0043005,GO:0005764,GO:0005774,GO:0044297,GO:0015629,GO:0003779,GO:0015031,	-	gi|254939640|ref|NP_085036.3|	0	vacuolar protein sorting-associated protein 16 homolog [Mus musculus]
mmu-miR-101a-5p	NM_001128146	-24	0.980968	NA	NA	GO:0002360,GO:0001816,	K06545|1|0.0|688|cfa:477704|CD163 antigen	gi|189458797|ref|NP_001121618.1|	0	scavenger receptor protein family member isoform 2 precursor [Mus musculus]

mmu-miR-101a-5p	NM_010907	-20	0.999995	NA	NA	GO:0043234,GO:0043231,GO:0044444,GO:0016020,GO:0008134,GO:0005048,GO:0019899,GO:0042348,GO:0010883,GO:0031325,GO:0051091,GO:0007169,GO:0050851,GO:0010742,GO:0043331,GO:0042994,GO:0043433,GO:0045596,GO:0043066,GO:0070423,GO:0032494,GO:00082	K04734|1|9e-172|603|mmu:18035|NF-kappa-B inhibitor alpha	gi|148704779|gb|EDL36726.1|	5.75E-172	nuclear factor of kappa light chain gene enhancer in B-cells inhibitor, alpha [Mus musculus]

mmu-miR-101a-5p	NM_010178	-21.2	0.999999	NA	NA	GO:0016604,GO:0003676,GO:0019904,GO:0000245,GO:0033119,GO:0006405,GO:0000387,GO:0006351,	K12900|1|5e-62|240|ssc:100625186|FUS-interacting serine-arginine-rich protein 1	gi|301754964|ref|XP_002913325.1|	3.48E-61	PREDICTED: splicing factor, arginine/serine-rich 13A-like [Ailuropoda melanoleuca]
mmu-miR-101a-5p	NM_023438	-20.2	1	NA	NA	GO:0031224,	-	gi|40254491|ref|NP_075927.2|	0	transmembrane protein 132E precursor [Mus musculus]
mmu-miR-101a-5p	NM_023879	-20.2	1	NA	NA	GO:0043231,GO:0042995,GO:0005488,GO:0050953,GO:0001754,	K11499|1|1e-07|60.8|gga:395357|centromere protein F	gi|13569852|ref|NP_076368.1|	0	X-linked retinitis pigmentosa GTPase regulator-interacting protein 1 isoform 1 [Mus musculus]
mmu-miR-101a-5p	NM_009444	-22	0.998753	NA	NA	-	-	gi|6678461|ref|NP_033470.1|	1.74E-151	trans-Golgi network integral membrane protein 2 precursor [Mus musculus]

mmu-miR-101a-5p	NM_010074	-20.4	1	NA	NA	GO:0005911,GO:0030027,GO:0016023,GO:0031253,GO:0031224,GO:0004175,GO:0004871,GO:0008238,GO:0042802,GO:0019899,GO:0022407,GO:0008283,GO:0023052,GO:0031294,GO:0010715,GO:0002456,GO:0006950,GO:0016477,GO:00195	K01278|1|0.0|1441|mmu:13482|dipeptidyl-peptidase 4 [EC:3.4.14.5]	gi|6753674|ref|NP_034204.1|	0	dipeptidyl peptidase 4 isoform 1 [Mus musculus]
mmu-miR-101a-5p	NM_027434	-21	0.999999	NA	NA	-	K15559|1|3e-158|556|cfa:485866|regulator of Ty1 transposition protein 103	gi|148674304|gb|EDL06251.1|	3.15E-176	RIKEN cDNA 2610304G08, isoform CRA_b [Mus musculus]

mmu-miR-101a-5p	NM_009836	-20.7	0.999974	NA	NA	GO:0043234,GO:0043232,GO:0044444,GO:0016020,GO:0005515,GO:0032559,GO:00064	K09495|1|0.0|1067|mmu:12462|T-complex protein 1 subunit gamma	gi|6753320|ref|NP_033966.1|	0	T-complex protein 1 subunit gamma [Mus musculus]
mmu-miR-101a-5p	NM_028722	-22.2	0.999979	NA	NA	GO:0000123,GO:0043967,	K13163|1|0.0|850|mmu:74026|male-specific lethal 1	gi|166091474|ref|NP_082998.2|	0	male-specific lethal 1 homolog [Mus musculus]
mmu-miR-101a-5p	NM_177644	-21.8	1	NA	NA	GO:0044464,GO:0009966,	K04352|1|7e-43|178|cin:100184835|Ras GTPase-activating protein 1	gi|226958516|ref|NP_808312.3|	0	RAS protein activator like 2 [Mus musculus]
mmu-miR-101a-5p	NM_007724	-27.3	0.580106	NA	NA	GO:0031224,GO:0016208,GO:0005267,GO:0034220,GO:0007606,GO:0030001,	K04949|1|0.0|1205|mmu:12789|cyclic nucleotide gated channel alpha 2	gi|31982859|ref|NP_031750.2|	0	cyclic nucleotide-gated olfactory channel [Mus musculus]

mmu-miR-101a-5p	NM_001024602	-23.3	0.999933	NA	NA	GO:0005856,	K13108|1|5e-07|58.9|cqu:CpipJ_CPIJ010869|smad nuclear-interacting protein 1	gi|28972129|dbj|BAC65518.1|	0	mKIAA0284 protein [Mus musculus]
mmu-miR-101a-5p	NM_011192	-24.5	0.919587	NA	NA	GO:0022624,GO:0043231,GO:0031224,GO:0061135,GO:0048037,GO:0046914,GO:0005515,GO:0016787,GO:0016641,GO:0010952,GO:0043161,GO:0051320,GO:0007050,GO:0016070,GO:0051439,GO:0006520,GO:0006915,GO:0030330,	K06698|1|6e-140|499|ecb:100065788|proteasome activator subunit 3 (PA28 gamma)	gi|332260752|ref|XP_003279444.1|	2.79E-140	PREDICTED: proteasome activator complex subunit 3-like [Nomascus leucogenys]

mmu-miR-101a-5p	NM_008359	-24.8	0.962972	NA	NA	GO:0016021,GO:0004896,GO:0001775,GO:0001819,GO:0007165,	K05164|1|0.0|1596|mmu:16172|interleukin 17 receptor	gi|6680411|ref|NP_032385.1|	0	interleukin-17 receptor A precursor [Mus musculus]
mmu-miR-101a-5p	NM_026850	-20.8	0.999865	NA	NA	GO:0044424,GO:0005488,GO:0012501,GO:0051704,	K08327|1|4e-08|60.5|tgu:100219066|phosducin	gi|31560120|ref|NP_081126.2|	4.90E-100	phosducin-like protein 3 [Mus musculus]
mmu-miR-101a-5p	NR_001581	-23.6	0.73795	NA	NA	-	-	gi|12839835|dbj|BAB24683.1|	1.62E-106	unnamed protein product [Mus musculus]
mmu-miR-101a-5p	NM_001199236	-26.3	0.8723	NA	NA	GO:0034399,GO:0016604,GO:0033267,GO:0030864,GO:0008092,GO:0007010,GO:0019953,GO:0051179,GO:0007154,GO:0048667,GO:0050877,GO:0035264,GO:00083	K06115|1|0.0|2056|mmu:80297|spectrin beta	gi|148692241|gb|EDL24188.1|	0	spectrin beta 4, isoform CRA_a [Mus musculus]

mmu-miR-101a-5p	NM_134013	-24.1	0.998852	NA	NA	GO:0016604,GO:0043234,GO:0043161,GO:0051320,GO:0007050,GO:0016070,GO:0051439,GO:0006520,GO:0006915,GO:0048869,GO:0048232,GO:0030330,	K06699|1|0.0|3494|mmu:103554|proteasome activator subunit 4	gi|117956381|ref|NP_598774.2|	0	proteasome activator complex subunit 4 [Mus musculus]
mmu-miR-101a-5p	NM_177767	-24	0.997528	NA	NA	GO:0046914,GO:0016705,GO:0019842,GO:0016701,GO:0008152,	-	gi|147901538|ref|NP_808435.3|	0	2-oxoglutarate and iron-dependent oxygenase domain-containing protein 1 isoform 1 [Mus musculus]
mmu-miR-101a-5p	NM_001177604	-20.3	1	NA	NA	GO:0044429,GO:0019205,GO:0032561,GO:0016776,GO:0032559,GO:0009719,GO:0010033,GO:0009117,GO:0006796,	K00939|1|5e-125|450|mmu:11639|adenylate kinase [EC:2.7.4.3]	gi|6753022|ref|NP_033777.1|	3.31E-124	adenylate kinase isoenzyme 4, mitochondrial [Mus musculus]

mmu-miR-101a-5p	NM_001113356	-24.1	0.917257	NA	NA	GO:0046872,GO:0004175,GO:0019538,GO:0006952,GO:0002455,	K01330|1|0.0|1446|mmu:50909|complement component 1, r subcomponent [EC:3.4.21.41]	gi|164518919|ref|NP_001106827.1|	0	complement C1r-B subcomponent precursor [Mus musculus]
mmu-miR-101b-3p	NM_001033535	-29.5	0.106597	-24.42	175	-	-	gi|109484717|ref|XP_001072284.1|	2.40E-121	PREDICTED: tumor necrosis factor alpha-induced protein 8-like protein 3-like [Rattus norvegicus]
mmu-miR-101b-3p	NM_011576	-27.1	0.193668	-24.89	172	GO:0044421,GO:0016020,GO:0004866,GO:0007599,GO:0010466,GO:0072376,	K03909|1|8e-130|464|mmu:21788|tissue factor pathway inhibitor	gi|148695335|gb|EDL27282.1|	5.08E-175	tissue factor pathway inhibitor, isoform CRA_b [Mus musculus]
mmu-miR-101b-3p	NM_007641	-25.2	0.558104	-23.4	171	GO:0016021,GO:0009986,GO:0046649,	K06466|1|2e-139|497|mmu:12482|membrane-spanning 4-domains, subfamily A, member 1	gi|6671710|ref|NP_031667.1|	1.15E-138	B-lymphocyte antigen CD20 [Mus musculus]

mmu-miR-101b-3p	NM_178640	-28.2	0.322273	-22.5	170	GO:0012505,GO:0043231,GO:0031224,GO:0016758,GO:0006464,	K09654|1|0.0|962|mmu:97884|beta-1,3-N-acetylgalactosaminyltransferase 2 [EC:2.4.1.-]	gi|47716521|ref|NP_848755.1|	0	UDP-GalNAc:beta-1,3-N-acetylgalactosaminyltransferase 2 precursor [Mus musculus]
mmu-miR-101b-3p	NM_175224	-28	0.240482	-21.64	169	GO:0044424,GO:0043169,GO:0008238,GO:0008237,GO:0018193,GO:0006412,	K01265|1|0.0|815|mmu:75624|methionyl aminopeptidase [EC:3.4.11.	gi|28202007|ref|NP_780433.1|	0	methionine aminopeptidase 1 [Mus musculus]
mmu-miR-101b-3p	NM_183408	-25.8	0.77653	-23.59	168	GO:0043231,GO:0001726,GO:0044444,GO:0016020,GO:0043169,GO:0004114,GO:0006195,GO:0023052,GO:0042221,	K01120|1|0.0|1425|rno:25638|3',5'-cyclic-nucleotide phosphodiesterase [EC:3.1.4.17]	gi|41282015|ref|NP_899668.1|	0	cAMP-specific 3',5'-cyclic phosphodiesterase 4A isoform 5 [Mus musculus]
mmu-miR-101b-3p	NM_001164580	-21.4	0.999987	-20.83	167	GO:0044464,GO:0003824,GO:0008152,	-	gi|148236001|ref|NP_001079768.1|	5.58E-92	uncharacterized protein C16orf52 homolog B [Xenopus laevis]

mmu-miR-101b-3p	NM_146039	-27.5	0.419564	-21.41	166	-	K10415|1|1e-10|70.1|nve:NEMVE_v1g158934|dynein intermediate chain, cytosolic	gi|113199781|ref|NP_666151.3|	0	WD repeat-containing protein 60 [Mus musculus]
mmu-miR-101b-3p	NR_045057	-27.4	0.120455	-24.23	165	-	-	gi|148680200|gb|EDL12147.1|	4.36E-16	mCG145184 [Mus musculus]
mmu-miR-101b-3p	NM_178667	-20.2	1	-25.88	164	GO:0043231,GO:0043234,GO:0012505,GO:0000989,GO:0003676,GO:0001071,GO:0005515,GO:0006351,	K09392|1|0.0|649|aml:100464401|transcription factor Dp-2	gi|8039811|sp|Q64163.2|TFDP2_MOUSE	0	RecName: Full=Transcription factor Dp-2; AltName: Full=Dp-3; AltName: Full=E2F dimerization partner 2
mmu-miR-101b-3p	NM_145066	-23.3	0.95026	-20.03	163	GO:0031224,GO:0004888,GO:0007166,	K04302|1|0.0|720|ssc:100736987|G protein-coupled receptor 85	gi|11560065|ref|NP_071590.1|	0	probable G-protein coupled receptor 85 [Rattus norvegicus]
mmu-miR-101b-3p	NM_146868	-21.8	0.841833	-20.21	162	GO:0016020,GO:0004888,GO:0007166,	K04257|1|2e-179|628|mmu:258868|olfactory	gi|22129137|ref|NP_667079.1|	1.15E-178	olfactory receptor 894 [Mus musculus]

mmu-miR-101b-3p	NM_175212	-22.5	0.994994	-21.61	161	-	K01183|1|3e-26|122|dgr:Dgri_GH10759|chitinase [EC:3.2.1.14]	gi|70778812|ref|NP_780421.2|	3.57E-112	transmembrane protein 65 [Mus musculus]
mmu-miR-101b-3p	NR_033731	-24	0.747464	-20.35	160	GO:0005488,GO:0004864,GO:0044237,	K12489|1|5e-11|70.5|ssc:100623422|Arf-GAP with coiled-coil, ANK repeat and PH domain-containing protein!K14809|2|3e-09|64.3|tca:658811|ATP-dependent RNA helicase DDX55/SPB4 [EC:3.6.4.13]	gi|148668392|gb|EDL00716.1|	6.64E-88	mCG1047086 [Mus musculus]

mmu-miR-101b-3p	NM_177271	-20.2	0.999983	-22.98	159	-	K14079|1|4e-22|107|dwi:Dwil_GK14246|poly(A) RNA polymerase GLD2 [EC:2.7.7.19]!K05106|5|8e-06|53.5|rno:79208|Eph receptor A5 [EC:2.7.10.1]	gi|110556634|ref|NP_796245.2|	7.06E-85	sterile alpha motif domain-containing protein 5 [Mus musculus]
mmu-miR-101b-3p	NM_175201	-20	1	-20.51	158	GO:0042995,GO:0046914,	K13201|1|1e-45|187|bfo:BRAFLDRAFT_124262|nucleolysin TIA-1/TIAR!K15692|3|1e-10|70.5|xtr:493377|E3 ubiquitin-protein ligase RNF13 [EC:6.3.2.19]	gi|332228409|ref|XP_0032633 82.1|	0	PREDICTED: RING finger protein 38 isoform 3 [Nomascus leucogenys]

mmu-miR-101b-3p	NM_147026	-26.1	0.172099	-21.18	157	GO:0004871,GO:0023052,	K04257|1|1e-117|422|mmu:258958|olfactory	gi|49227415|ref|NP_667237.1|	1.58E-132	olfactory receptor 532 [Mus musculus]
mmu-miR-101b-3p	NM_172823	-23.1	0.988929	-20.52	156	GO:0044464,GO:0004175,GO:0046914,GO:0019538,GO:0000087,	K13539|1|0.0|1269|mmu:239833|leishmanolysin-like peptidase [EC:3.4.24.-]	gi|148665419|gb|EDK97835.1|	0	leishmanolysin-like (metallopeptidase M8 family), isoform CRA_c [Mus musculus]
mmu-miR-101b-3p	NM_027726	-24.9	0.974148	-21.12	155	GO:0044459,GO:0044424,GO:0032947,GO:0006915,GO:0008283,GO:0007155,GO:0007165,	K05701|1|1e-43|181|bfo:BRAFLDRAFT_93411|tight junction protein 1!K12076|4|3e-43|179|hsa:1739|disks large protein 1	gi|254588085|ref|NP_082002.1|	0	discs large homolog 5 isoform 2 [Mus musculus]

mmu-miR-101b-3p	NM_032610	-26.3	0.89175	-20.22	154	GO:0034399,GO:0016604,GO:0033267,GO:0030864,GO:0005198,GO:0008092,GO:0006810,GO:0030835,GO:0019953,GO:0007154,GO:0048667,GO:0006935,GO:0035264,GO:00	K06115|1|0.0|4142|rno:308458|spectrin beta	gi|148692241|gb|EDL24188.1|	0	spectrin beta 4, isoform CRA_a [Mus musculus]
mmu-miR-101b-3p	NM_213727	-22.3	0.998933	-20.05	153	-	-	gi|47523376|ref|NP_998892.1|	0	APC membrane recruitment protein 3 [Mus musculus]
mmu-miR-101b-3p	NM_019496	-24.3	0.86032	-20.7	152	-	-	gi|397502901|ref|XP_003822076.1|	1.81E-125	PREDICTED: AMME syndrome candidate gene 1 protein [Pan paniscus]
mmu-miR-101b-3p	NM_172274	-21.3	0.999998	-20.55	151	GO:0042995,GO:0005815,	-	gi|26986583|ref|NP_758478.1|	0	coiled-coil and C2 domain-containing protein 2A [Mus musculus]
mmu-miR-101b-3p	NM_019796	-24	0.990137	-20.17	150	GO:0043234,GO:0005681,GO:0031981,GO:0003729,GO:0051704,GO:0000377,GO:00482	K13160|1|0.0|953|cfa:474986|heterogeneous nuclear ribonucleoprotein Q	gi|37589144|gb|AAH58807.1|	0	Syncrip protein, partial [Mus musculus]

mmu-miR-101b-3p	NM_023626	-21.3	0.999921	-21.64	149	GO:0043189,GO:0004468,GO:0046914,GO:0050789,GO:0006915,GO:0006351,GO:00165	K11319|1|0.0|709|mmu:71777|inhibitor of growth protein 3	gi|139948756|ref|NP_076115.3|	0	inhibitor of growth protein 3 [Mus musculus]
mmu-miR-101b-3p	NM_028369	-24.2	0.744841	-23.19	148	GO:0005488,GO:0015031,GO:0006875,	-	gi|21312179|ref|NP_082645.1|	0	vacuolar fusion protein MON1 homolog A [Mus musculus]
mmu-miR-101b-3p	NM_198866	-22.2	0.993682	-20.85	147	-	-	gi|38524610|ref|NP_942566.1|	9.37E-55	DNA binding protein with his-thr domain [Mus musculus]
mmu-miR-101b-3p	NM_027306	NA	NA	-20.21	148	GO:0043231,GO:0043169,GO:0006497,GO:0006886,	K05766|1|3e-11|70.5|spu:575378|slingshot	gi|58037195|ref|NP_081582.1|	3.60E-160	zinc finger, DHHC domain containing 25 [Mus musculus]
mmu-miR-101b-3p	NM_001242934	-20.6	1	NA	NA	GO:0043231,GO:0003712,GO:0046914,GO:0030098,GO:0032446,GO:0006351,	K09228|1|5e-17|92.0|api:100159445|KRAB domain-containing zinc finger protein	gi|339409205|ref|NP_001229863.1|	0	B-cell lymphoma/leukemia 11A isoform 4 [Mus musculus]

mmu-miR-101b-3p	NM_007727	-20.5	1	NA	NA	GO:0031224,GO:0005515,GO:0030030,GO:0006935,GO:0010765,GO:0010467,GO:00071	K06759|1|0.0|1959|mmu:12805|contactin 1	gi|6680954|ref|NP_031753.1|	0	contactin-1 precursor [Mus musculus]
mmu-miR-101b-3p	NM_030679	-25.1	0.929337	NA	NA	GO:0031981,GO:0005924,GO:0016460,GO:0030017,GO:0008092,GO:0017111,GO:0032559,GO:0003012,	K10352|1|0.0|3297|mmu:17879|myosin heavy chain	gi|82524274|ref|NP_109604.1|	0	myosin-1 [Mus musculus]

mmu-miR-101b-3p	NM_172723	-25.6	0.555582	NA	NA	GO:0043231,GO:0016020,GO:0005083,GO:0004871,GO:0046914,GO:0043178,GO:0035091,GO:0019932,GO:0033124,GO:0032012,	K12491|1|4e-23|110|api:100169536|Arf-GAP, GTPase, ANK repeat and PH domain-containing protein 1/2/3!K13051|2|3e-22|107|spu:582857|beta-aspartyl-peptidase (threonine type) [EC:3.4.19.5]!K12489|3|3e-21|104|tad:TRIADDRAFT_57442|Arf-	gi|148368976|ref|NP_766311.2|	0	centaurin, alpha 1 [Mus musculus]
mmu-miR-101b-3p	NM_001024931	-23.8	0.909572	NA	NA	GO:0043231,GO:0044297,GO:0003676,GO:0006396,	K14946|1|3e-146|520|mmu:52897|RNA binding protein fox-1	gi|148702729|gb|EDL34676.1|	2.11E-147	DNA segment, Chr 11, Brigham & Women's Genetics 0517 expressed, isoform CRA_c [Mus musculus]

mmu-miR-101b-3p	NM_013861	-21.6	0.996955	NA	NA	GO:0044444,GO:0016778,GO:0032559,GO:0042723,GO:0009108,GO:0006796,	K00949|1|2e-141|503|mmu:29807|thiamine pyrophosphokinase [EC:2.7.6.2	gi|14278354|pdb|1IG3|A	1.60E-140	Chain A, Mouse Thiamin Pyrophosphokinase Complexed With Thiamin
mmu-miR-101b-3p	NM_146306	-21.2	0.931691	NA	NA	GO:0031224,GO:0004888,GO:0023052,	K04257|1|0.0|632|mmu:258303|olfactory receptor	gi|22129679|ref|NP_666418.1|	6.65E-180	olfactory receptor 518 [Mus musculus]
mmu-miR-101b-3p	NM_053182	-23.4	0.999301	NA	NA	GO:0016021,GO:0030674,GO:0019904,GO:0050851,GO:0050863,GO:0007169,	-	gi|148673233|gb|EDL05180.1|	0	phosphoprotein associated with glycosphingolipid microdomains 1, isoform CRA_b [Mus musculus]
mmu-miR-101b-3p	NM_178618	-22.1	0.999702	NA	NA	-	K14390|1|2e-36|155|mmu:109342|solute carrier family 5 (sodium/glucose cotransporter), member 10	gi|117647255|ref|NP_848733.2|	0	protein FAM83G [Mus musculus]

mmu-miR-101b-3p	NM_030112	-20.9	0.999999	NA	NA	GO:0031981,GO:0043566,GO:0006351,	K15178|1|0.0|1012|mmu:76246|RNA polymerase-associated protein RTF1	gi|195976788|ref|NP_084388.2|	0	RNA polymerase-associated protein RTF1 homolog precursor [Mus musculus]
mmu-miR-101b-3p	NR_030680	-21.5	0.998567	NA	NA	GO:0043231,GO:0003676,GO:0006259,GO:0022403,	-	gi|240848585|ref|NP_084073.2|	7.34E-101	meiotic nuclear division protein 1 homolog [Mus musculus]
mmu-miR-101b-3p	NM_030704	-20.9	0.997014	NA	NA	GO:0043231,GO:0004672,GO:0005515,GO:0006950,GO:0009987,	K08879|1|6e-104|379|mmu:80888|heat shock 22kDa protein 8	gi|13507646|ref|NP_109629.1|	3.90E-103	heat shock protein beta-8 [Mus musculus]
mmu-miR-101b-3p	NM_010856	-22.1	0.999948	NA	NA	-	K10352|1|0.0|3335|mmu:17888|myosin heavy chain	gi|148704367|gb|EDL36314.1|	0	mCG133649, isoform CRA_b [Mus musculus]

mmu-miR-101b-3p	NM_001081191	-21.1	1	NA	NA	-	K15420|1|2e-99|366|tgu:100219108|echinoderm microtubule-associated protein-like 4	gi|124487111|ref|NP_001074660.1|	0	echinoderm microtubule-associated protein-like 5 [Mus musculus]
mmu-miR-101b-3p	NR_028353	-20	1	NA	NA	GO:0044444,GO:0008252,GO:0043169,GO:0006163,GO:0009112,GO:0006796,	K01081|1|0.0|1005|mmu:76952|5'-nucleotidase [EC:3.1.3.5]	gi|256665234|ref|NP_001157835.1|	0	cytosolic purine 5'-nucleotidase isoform 1 [Mus musculus]
mmu-miR-101b-3p	NM_013875	-20.4	1	NA	NA	GO:0044444,GO:0043169,GO:0004114,GO:0019935,GO:0007267,	K01120|1|0.0|921|mmu:29863|3',5'-cyclic-nucleotide phosphodiesterase [EC:3.1.4.17]	gi|148539997|ref|NP_038903.3|	0	cAMP-specific 3',5'-cyclic phosphodiesterase 7B [Mus musculus]

mmu-miR-101b-3p	NM_173755	-24.3	0.961786	NA	NA	GO:0019787,GO:0032559,GO:0032446,	K10581|1|0.0|2164|mmu:217342|ubiquitin-conjugating enzyme E2 O [EC:6.3.2.19]	gi|148702637|gb|EDL34584.1|	0	ubiquitin-conjugating enzyme E2O [Mus musculus]
mmu-miR-101b-3p	NM_001083331	-22.4	0.977412	NA	NA	GO:0031981,GO:0044444,GO:0005635,GO:0022829,GO:0044238,GO:0050658,GO:00157	K14318|1|0.0|1447|mmu:19069|nuclear pore complex protein Nup88	gi|148680682|gb|EDL12629.1|	0	nucleoporin 88, isoform CRA_d [Mus musculus]

mmu-miR-101b-3p	NM_146010	-20	0.999527	NA	NA	GO:0031224,GO:0000323,GO:0060089,GO:0023052,GO:0007596,GO:0006464,	K06497|1|3e-27|123|nve:NEMVE_v1g236446|CD63 antigen!K06537|2|3e-26|120|nve:NEMVE_v1g233307|CD151 antigen!K06460|3|1e-25|118|aml:100480432|CD9 antigen	gi|22122475|ref|NP_666122.1|	7.17E-116	tetraspanin-8 [Mus musculus]
mmu-miR-101b-3p	NM_007925	-20.7	0.999996	NA	NA	GO:0043231,GO:0005576,GO:0005488,GO:0005201,GO:0051017,GO:0008064,GO:0014706,GO:0030198,	K14211|1|6e-14|81.3|rno:25043|elastin	gi|345801223|ref|XP_859408.2|	2.22E-32	PREDICTED: uncharacterized protein LOC607775 [Canis lupus familiaris]
mmu-miR-101b-3p	NM_001167746	-26.3	0.972144	NA	NA	GO:0030286,GO:0005930,GO:0003774,GO:0032559,GO:0048870,GO:0007017,	K10408|1|0.0|2152|tad:TRIADDRAFT_23475|dynein heavy chain, axonemal	gi|283837762|ref|NP_001161218.1|	0	dynein heavy chain 17, axonemal [Mus musculus]

mmu-miR-101b-3p	NM_199009	-23.2	0.999952	NA	NA	GO:0043234,GO:0005488,GO:0007041,GO:0016197,GO:0007033,	-	gi|39930595|ref|NP_950174.1|	0	FTS and Hook-interacting protein isoform a [Mus musculus]
mmu-miR-101b-3p	NM_026641	-25.7	0.746251	NA	NA	GO:0005815,GO:0005930,	K04508|1|6e-12|74.7|smm:Smp_098760|transducin (beta)-like 1	gi|28076907|ref|NP_080917.1|	0	intraflagellar transport protein 80 homolog [Mus musculus]
mmu-miR-101b-3p	NM_207705	-20.5	0.999994	NA	NA	GO:0043232,GO:0044444,GO:0019904,GO:0005102,GO:0003824,GO:0006935,GO:0012501,GO:0006897,	K12366|1|0.0|1005|mcc:705818|engulfment and cell motility protein 1	gi|46877050|ref|NP_997588.1|	0	engulfment and cell motility protein 2 isoform 1 [Mus musculus]
mmu-miR-101b-3p	NM_001009940	-20.7	0.975855	NA	NA	GO:0044421,GO:0005102,GO:0002376,GO:0032635,GO:0012502,GO:0044237,GO:00230	K05444|1|3e-97|355|mmu:329244|interleukin 19/20/24	gi|57977321|ref|NP_001009940.1|	2.26E-96	interleukin-19 precursor [Mus musculus]

mmu-miR-101b-3p	NM_007742	-21	0.999998	NA	NA	-	K06236|1|1e-151|539|mmu:12842|collagen, type I/II/III/V/XI, alpha	gi|148684009|gb|EDL15956.1|	6.73E-151	procollagen, type I, alpha 1, isoform CRA_a [Mus musculus]
mmu-miR-101b-3p	NM_001083906	-23.4	0.979896	NA	NA	GO:0031981,GO:0031090,GO:0046914,GO:0042802,GO:0008289,GO:0001071,GO:0046983,GO:0003707,GO:0043566,GO:0003008,GO:0006875,GO:0009755,GO:0008283,GO:00	K08555|1|0.0|1732|mmu:110784|mineralocorticoid receptor	gi|144227212|ref|NP_001077375.1|	0	mineralocorticoid receptor [Mus musculus]
mmu-miR-101b-3p	NM_172540	-23.1	0.972985	NA	NA	GO:0043231,GO:0008757,GO:0006399,GO:0001510,GO:0010467,	K15331|1|0.0|1015|mmu:215201|tRNA (uracil-5-)-methyltransferase [EC:2.1.1.35]	gi|269784754|ref|NP_766128.2|	0	tRNA (uracil(54)-C(5))-methyltransferase homolog isoform a [Mus musculus]

mmu-miR-101b-3p	NM_001081395	-21.4	1	NA	NA	GO:0044424,GO:0043296,GO:0005515,	K06104|1|0.0|1605|mmu:75723|angiomotin like 1	gi|124487107|ref|NP_001074864.1|	0	angiomotin-like protein 1 [Mus musculus]
mmu-miR-101b-3p	NM_030723	-24.4	0.975342	NA	NA	GO:0003676,	K14844|1|1e-09|67.4|dgr:Dgri_GH24934|pumilio homology domain family member 6	gi|237649070|ref|NP_109648.2|	0	pumilio homolog 2 isoform 1 [Mus musculus]
mmu-miR-101b-3p	NM_198618	-21.7	0.999674	NA	NA	GO:0030425,GO:0005886,GO:0044297,GO:0005515,	K15008|1|2e-106|388|tgu:100226961|discs, large-associated protein 1	gi|149023981|gb|EDL80478.1|	0	discs, large (Drosophila) homolog-associated protein 3, isoform CRA_b [Rattus norvegicus]
mmu-miR-101b-3p	NM_175343	-24.1	0.978606	NA	NA	GO:0019866,GO:0043231,GO:0016614,GO:0050662,GO:0019695,	K00108|1|0.0|1245|mmu:218865|choline dehydrogenase [EC:1.1.99.1]	gi|148692803|gb|EDL24750.1|	0	choline dehydrogenase [Mus musculus]

mmu-miR-101b-3p	NM_001109905	-20.9	0.999911	NA	NA	GO:0043231,GO:0044297,GO:0030529,GO:0042995,GO:0003723,GO:0019903,GO:00064	-	gi|74204587|dbj|BAE35365.1|	0	unnamed protein product [Mus musculus]
mmu-miR-101b-3p	NM_001256520	-20.2	0.999995	NA	NA	GO:0043231,GO:0046914,GO:0003676,GO:0010467,	K09228|1|7e-105|382|pon:100433614|KRAB domain-containing zinc finger protein	gi|148675800|gb|EDL07747.1|	0	zinc finger protein 672, isoform CRA_a [Mus musculus]
mmu-miR-101b-3p	NM_026042	-23.6	0.846258	NA	NA	GO:0043234,GO:0043231,GO:0005488,GO:0010467,	K15142|1|5e-72|273|mmu:67224|mediator of RNA polymerase II transcription subunit	gi|27754101|ref|NP_080318.2|	3.75E-71	mediator of RNA polymerase II transcription subunit 29 [Mus musculus]

mmu-miR-101b-3p	NR_028110	-20.5	1	NA	NA	GO:0008287,GO:0005488,GO:0019888,GO:0006464,GO:0008283,GO:0051318,	K11583|1|2e-139|359|mmu:19054|protein phosphatase 2 (formerly 2A), regulatory subunit	gi|148705237|gb|EDL37184.1|	3.86E-180	mCG8010, isoform CRA_a [Mus musculus]
mmu-miR-101b-3p	NM_145143	-27	0.340506	NA	NA	GO:0044424,GO:0005515,	K00942|1|9e-71|269|aag:AaeL_AAEL014012|guanylate kinase [EC:2.7.4.8]	gi|258547158|ref|NP_001158154.1|	0	MAGUK p55 subfamily member 4 isoform 1 [Mus musculus]
mmu-miR-101b-3p	NM_008187	-20.1	0.99819	NA	NA	GO:0032501,	-	gi|51491831|ref|NP_001003905.1|	1.01E-107	UPF0468 protein C16orf80 homolog [Bos taurus]

mmu-miR-101b-3p	NM_008816	-21	0.999939	NA	NA	GO:0031224,GO:0030667,GO:0044421,GO:0044459,GO:0043292,GO:0005488,GO:0042506,GO:0007265,GO:0009611,GO:0007599,GO:0010033,GO:0050900,GO:0006887,GO:0006897,GO:0001775,	K06471|1|0.0|1300|mmu:18613|platelet/endothelial cell adhesion molecule	gi|74024907|ref|NP_032842.2|	0	platelet endothelial cell adhesion molecule isoform 1 precursor [Mus musculus]
mmu-miR-101b-3p	NM_026400	-20.2	0.999986	NA	NA	GO:0044432,GO:0005515,GO:0044267,GO:0009451,	K09517|1|0.0|685|mmu:67838|DnaJ homolog subfamily B member 11	gi|397470100|ref|XP_003806671.1|	0	PREDICTED: dnaJ homolog subfamily B member 11 [Pan paniscus]
mmu-miR-101b-3p	NM_001271586	-20.4	0.999623	NA	NA	-	-	-	-	-

mmu-miR-101b-3p	NM_025780	-21.8	0.998972	NA	NA	GO:0031981,GO:0003676,GO:0043169,	K10423|1|1e-08|63.2|cfa:484566|CAP-Gly domain-containing linker protein 3/4	gi|13385250|ref|NP_080056.1|	7.37E-112	THAP domain-containing protein 2 [Mus musculus]
mmu-miR-101b-3p	NM_001159965	-24.4	0.983571	NA	NA	GO:0016020,GO:0044424,GO:0005488,GO:0030695,GO:0019222,GO:0035556,	K04349|1|2e-31|140|rno:192213|Ras-specific guanine nucleotide-releasing factor 1	gi|229577166|ref|NP_076373.3|	0	ras-specific guanine nucleotide-releasing factor RalGPS2 isoform a [Mus musculus]
mmu-miR-101b-3p	NM_177466	-22.6	0.996611	NA	NA	GO:0010008,GO:0031966,GO:0015631,GO:0006810,	K12484|1|0.0|968|mcc:713953|Rab11 family-interacting protein 1/2/5	gi|55743143|ref|NP_803417.3|	0	rab11 family-interacting protein 5 isoform 2 [Mus musculus]
mmu-miR-101b-3p	NM_001243139	-24.4	0.831452	NA	NA	-	K09228|1|0.0|1034|mmu:667666|KRAB domain-containing zinc finger protein	gi|295293068|ref|NP_001171238.1|	0	reduced expression 2 [Mus musculus]

mmu-miR-101b-3p	NM_153135	-21.2	1	NA	NA	GO:0031224,GO:0004871,GO:0006935,GO:0032501,GO:0012501,GO:0023052,	K07521|1|0.0|1887|mmu:210801|netrin receptor unc-5	gi|23346575|ref|NP_694775.1|		0	netrin receptor UNC5D precursor [Mus musculus]
mmu-miR-101b-3p	NM_001040112	-21.2	0.999998	NA	NA	GO:0000139,GO:0046914,GO:0035091,GO:0005099,GO:0032879,GO:0065008,GO:0031346,GO:0032232,GO:0032012,GO:00323	K12490|1|0.0|2335|mmu:69710|Arf-GAP, Rho-GAP domain, ANK repeat and PH domain-containing protein	gi|187954747|gb|AAI41180.1|		0	ArfGAP with RhoGAP domain, ankyrin repeat and PH domain 1 [Mus musculus]
mmu-miR-101b-3p	NM_001162999	-20.5	1	NA	NA	GO:0044424,GO:0005488,GO:0006468,	K08020|1|5e-13|79.0|acs:100553829|Rap guanine nucleotide exchange factor (GEF) 6	gi|244790458|ref|NP_001156471.1|		0	folliculin-interacting protein 2 [Mus musculus]

mmu-miR-101b-3p	NM_028821	-23	0.998373	NA	NA	-	K10411|1|9e-104|380|mmu:105000|dynein light chain 1, axonemal	gi|344273553|ref|XP_003408585.1|	4.26E-104	PREDICTED: dynein light chain 1, axonemal-like [Loxodonta africana]
mmu-miR-101b-3p	NR_037708	-20.5	1	NA	NA	-	K08625|1|0.0|1735|mmu:224697|a disintegrin and metalloproteinase with thrombospondin motifs 10 [EC:3.4.24.-]	gi|27502095|gb|AAO17380.1|	0	zinc metalloendopeptidase [Mus musculus]
mmu-miR-101b-3p	NM_182807	-25.8	0.753784	NA	NA	GO:0044424,	-	gi|54607133|ref|NP_877959.2|	3.83E-76	protein FAM19A2 isoform 1 [Mus musculus]
mmu-miR-101b-3p	NM_080459	-21.2	0.999999	NA	NA	GO:0050954,	-	gi|120300985|ref|NP_536707.2|	0	stereocilin precursor [Mus musculus]

mmu-miR-101b-3p	NM_001172588	-23.4	0.681848	NA	NA	GO:0016020,GO:0008528,GO:0050896,	K08396|1|4e-52|206|aml:100475424|Mas-related G protein-coupled receptor member	gi|289191461|ref|NP_001166059.1|	7.86E-150	MAS-related GPR, member A2A [Mus musculus]

mmu-miR-101b-3p	NM_010516	-22.7	0.933179	NA	NA	GO:0031012,GO:0019838,GO:0005102,GO:0005539,GO:0010952,GO:0051094,GO:0060411,GO:0048568,GO:0010033,GO:0046513,GO:0016337,GO:0001503,GO:0010810,GO:0006915,GO:0045595,GO:0045765,GO:0016049,GO:0043062,GO:0010517,GO:0003171,GO:0016477,GO:00032	K06829|1|0.0|695|mmu:16007|cysteine rich protein 61	gi|6753594|ref|NP_034646.1|	0	protein CYR61 precursor [Mus musculus]

mmu-miR-101b-3p	NM_001042655	-24.6	0.757238	NA	NA	GO:0044464,GO:0005099,GO:0032313,	K13136|1|4e-98|360|nvi:100114269|gem associated protein 8!K11452|2|2e-13|78.6|tgu:100222038|chromobox protein 4	gi|74208127|dbj|BAE29165.1|		0	unnamed protein product [Mus musculus]
mmu-miR-101b-3p	NM_019799	-24.5	0.691972	NA	NA	GO:0016021,GO:0044459,GO:0031982,GO:0008092,GO:0015101,GO:0071702,GO:0015696,GO:0030154,GO:0055067,	K06580|1|0.0|852|mmu:56315|ammonium transporter Rh	gi|148675115|gb|EDL07062.1|		0	Rhesus blood group-associated C glycoprotein, isoform CRA_b [Mus musculus]
mmu-miR-101b-3p	NM_027117	-20	0.999937	NA	NA	GO:0043231,GO:0005488,	K10317|1|3e-21|104|nve:NEMVE_v1g100198|F-box protein 42!K14966|2|1e-20|102|mcc:700316|host cell factor	gi|226246638|ref|NP_081393.2|		0	kelch domain-containing protein 2 [Mus musculus]

mmu-miR-101b-3p	NM_001034964	-22.3	0.999689	NA	NA	GO:0031981,GO:0005815,GO:0032432,GO:0005913,GO:0005924,GO:0005901,GO:0005887,GO:0005102,GO:0030674,GO:0008092,GO:0019900,GO:0003012,GO:0032868,GO:0008610,GO:0051017,GO:0007044,GO:0007169,GO:0005979,GO:0031589,GO:0048522,GO	K06086|1|0.0|1275|pon:100171946|sorbin and SH3 domain containing 1	gi|78000154|ref|NP_001030136.1|	0	sorbin and SH3 domain-containing protein 1 isoform 5 [Mus musculus]

mmu-miR-101b-3p	NM_011521	-20.2	0.999981	NA	NA	GO:0031224,GO:0043231,GO:0030016,GO:0005924,GO:0004872,GO:0019901,GO:0001954,GO:0023052,GO:0032233,GO:0033674,GO:0007154,	K06257|1|4e-80|300|mmu:20971|syndecan	gi|6755442|ref|NP_035651.1|	2.56E-79	syndecan-4 precursor [Mus musculus]
mmu-miR-101b-3p	NM_016696	-24.1	0.915656	NA	NA	GO:0031012,GO:0016021,GO:0043394,GO:0006935,GO:0007154,	K08107|1|0.0|1028|mmu:14733|glypican 1	gi|7710028|ref|NP_057905.1|	0	glypican-1 precursor [Mus musculus]
mmu-miR-101b-3p	NM_054046	-22.2	0.998159	NA	NA	GO:0046914,GO:0007165,	-	gi|148679797|gb|EDL11744.1|	0	differentially expressed in FDCP 8, isoform CRA_b [Mus musculus]
mmu-miR-101b-3p	NM_029698	-22.4	0.995971	NA	NA	-	-	gi|254692940|ref|NP_083974.1|	0	tetratricopeptide repeat domain 18 isoform 3 [Mus musculus]

mmu-miR-101b-3p	NM_027570	-23.8	0.828317	NA	NA	GO:0043231,GO:0019866,GO:0004457,GO:0050662,GO:0009206,	K00102|1|0.0|978|mmu:52815|D-lactate dehydrogenase (cytochrome) [EC:1.1.2.4]	gi|34328379|ref|NP_081846.3|	0	probable D-lactate dehydrogenase, mitochondrial [Mus musculus]
mmu-miR-101b-3p	NM_016690	-20.5	0.999971	NA	NA	GO:0043231,GO:0030529,GO:0003729,GO:0043566,GO:0010467,	K13044|1|7e-157|555|mmu:50926|heterogeneous nuclear ribonucleoprotein A/B/D	gi|148664250|ref|NP_057899.2|	5.04E-156	heterogeneous nuclear ribonucleoprotein D-like [Mus musculus]
mmu-miR-101b-3p	NM_033604	-20.1	1	NA	NA	GO:0032991,GO:0044424,GO:0046872,GO:0005515,GO:0019787,GO:0006355,GO:0016567,GO:0007179,GO:0007275,	K13201|1|7e-09|64.7|bfo:BRAFLDRAFT_124262|nucleolysin TIA-1/TIAR!K11980|2|9e-09|64.3|ame:408648|E3 ubiquitin-protein ligase RNF11	gi|15809022|ref|NP_291082.1|	0	E3 ubiquitin-protein ligase Arkadia [Mus musculus]

mmu-miR-101b-3p	NR_037693	-22.7	0.94366	NA	NA	-	-	gi|148677784|gb|EDL09731.1|	2.88E-39	mCG55779, isoform CRA_b [Mus musculus]
mmu-miR-101b-3p	NM_016791	-21	0.999997	NA	NA	GO:0043231,GO:0001071,GO:0043565,GO:0005527,GO:0006357,GO:0022402,GO:0048513,GO:0007165,GO:0070838,GO:00009	K04446|1|0.0|1260|mmu:18018|nuclear factor of activated T-cells, cytoplasmic, calcineurin-dependent	gi|31560459|ref|NP_058071.2|	0	nuclear factor of activated T-cells, cytoplasmic 1 isoform 1 [Mus musculus]
mmu-miR-101b-3p	NM_001159317	-21.7	0.999769	NA	NA	GO:0016021,GO:0005126,GO:0004896,GO:0019838,GO:0006952,GO:0032623,GO:0002376,GO:0065003,GO:0007166,	K04723|1|0.0|1262|mmu:16180|interleukin 1 receptor accessory protein	gi|226693331|ref|NP_001152789.1|	0	interleukin-1 receptor accessory protein isoform c precursor [Mus musculus]

mmu-miR-101b-3p	NM_207263	-22.5	0.962403	NA	NA	GO:0044464,GO:0008329,GO:0016811,GO:0046914,GO:0005539,GO:0042742,GO:0000270,GO:0002758,GO:0009581,	K01446|1|0.0|771|mmu:384997|N-acetylmuramoyl-L-alanine amidase [EC:3.5.1.28]	gi|260166655|ref|NP_997146.2|	0	peptidoglycan recognition protein 4 isoform 2 precursor [Mus musculus]
mmu-miR-101b-3p	NM_181856	-25.1	0.685512	NA	NA	GO:0031224,GO:0043231,	-	gi|304434590|ref|NP_001182017.1|	0	transmembrane channel-like protein 8 isoform A [Mus musculus]
mmu-miR-101b-3p	NM_146023	-23.9	0.950485	NA	NA	GO:0044424,GO:0016020,	-	gi|22122501|ref|NP_666135.1|	0	protein EVI2B precursor [Mus musculus]

mmu-miR-101b-3p	NM_008363	-23.4	0.974612	NA	NA	GO:0031090,GO:0043235,GO:0044444,GO:0019787,GO:0032559,GO:0005126,GO:0004702,GO:0042802,GO:0046983,GO:0006461,GO:0006357,GO:0008284,GO:0051091,GO:0007169,GO:0043406,GO:0043433,GO:0019221,GO:0001666,GO:0032446,GO:0043066,GO:0002224,GO:00072	K04730|1|0.0|1162|mmu:16179|interleukin-1 receptor-associated kinase 1 [EC:2.7.11.1]	gi|117606337|ref|NP_032389.2|	0	interleukin-1 receptor-associated kinase 1 isoform 4 [Mus musculus]

mmu-miR-101b-5p	NM_001005508	-27.5	0.811359	-25.47	156	GO:0044444,GO:0008047,GO:0009966,GO:0035556,	K04450|1|9e-25|117|ptr:739083|activating transcription factor 2!K08878|2|3e-24|115|bfo:BRAFLDRAFT_84243|breakpoint cluster region protein [EC:2.7.11.1]!K08773|3|2e-23|113|bfo:BRAFLDRAFT_80737|RalA-binding protein 1	gi|229608953|ref|NP_001005508.2|	0	rho GTPase-activating protein 30 [Mus musculus]
mmu-miR-101b-5p	NM_001102471	-28.3	0.581208	-44.97	317	GO:0031224,GO:0006810,	-	gi|148710072|gb|EDL42018.1|	0	cyclin M2, isoform CRA_a [Mus musculus]

mmu-miR-101b-5p	NM_001159632	-30.5	0.137619	-27.69	173	GO:0033176,GO:0044444,GO:0005488,GO:0015662,GO:0007169,GO:0006875,GO:0015988,GO:0015031,	K02148|1|0.0|840|mmu:68775|V-type H+-transporting ATPase subunit C [EC:3.6.3.14]	gi|227499977|ref|NP_001153104.1|	0	V-type proton ATPase subunit C 2 isoform 1 [Mus musculus]
mmu-miR-101b-5p	NM_007968	-28.5	0.43699	-26.7	161	GO:0016604,GO:0005488,	K13209|1|7e-86|319|mmu:14030|RNA-binding protein EWS	gi|56206935|emb|CAI25233.1|	5.16E-85	Ewing sarcoma breakpoint region 1 [Mus musculus]
mmu-miR-101b-5p	NM_008067	-32.3	0.176292	-26.95	165	GO:0044459,GO:0034702,GO:0030594,GO:0005253,GO:0016917,GO:0015276,GO:0007267,GO:0007186,GO:0015698,	K05175|1|0.0|913|mmu:14396|gamma-aminobutyric acid receptor subunit alpha	gi|123210733|emb|CAM16951.1|	0	gamma-aminobutyric acid (GABA-A) receptor, subunit alpha 3 [Mus musculus]

mmu-miR-101b-5p	NM_011158	-38	0.016129	-37.21	188	GO:0044425,GO:0043234,GO:0019866,GO:0044444,GO:0016772,GO:0016208,GO:0051018,GO:0019887,GO:0009653,GO:0015980,GO:0007599,GO:0007611,GO:0033762,GO:0032147,GO:0010863,GO:0007169,GO:0022402,GO:0030073,GO:0032787,GO:00	K04739|1|0.0|775|mmu:19088|cAMP-dependent protein kinase regulator	gi|148704952|gb|EDL36899.1|	0	protein kinase, cAMP dependent regulatory, type II beta, isoform CRA_b [Mus musculus]

mmu-miR-101b-5p	NM_011663	-26.6	0.908222	-144.71	976	GO:0005681,GO:0003729,GO:0046914,GO:0000398,	K03105|1|2e-118|428|ptr:737811|signal recognition particle subunit SRP19!K00816|2|8e-28|127|smm:Smp_123750|kynurenine---oxoglutarate transaminase / cysteine-S-conjugate beta-lyase / glutamine---phenylpyruvate	gi|6755917|ref|NP_035793.1|	3.99E-161	U2 small nuclear ribonucleoprotein auxiliary factor 35 kDa subunit-related protein 1 [Mus musculus]

mmu-miR-101b-5p	NM_026138	-28.7	0.593057	-26.26	169	GO:0016020,	K10352|1|1e-10|70.1|hmg:100207631|myosin heavy chain!K10388|3|7e-10|67.8|dre:567639|plectin!K06267|4|9e-10|67.4|cin:493183|hyaluronan-mediated motility receptor	gi|120953219|ref|NP_080414.2|	0	protein SOGA3 precursor [Mus musculus]
mmu-miR-101b-5p	NM_033569	-28.3	0.588828	-44.97	317	GO:0031224,GO:0006810,	-	gi|148710072|gb|EDL42018.1|	0	cyclin M2, isoform CRA_a [Mus musculus]
mmu-miR-101b-5p	NM_133699	-30.5	0.134536	-27.69	173	GO:0033176,GO:0044444,GO:0005488,GO:0015662,GO:0007169,GO:0006875,GO:0015988,GO:0015031,	K02148|1|0.0|811|mmu:68775|V-type H+-transporting ATPase subunit C [EC:3.6.3.14]	gi|19526870|ref|NP_598460.1|	0	V-type proton ATPase subunit C 2 isoform 2 [Mus musculus]

mmu-miR-101b-5p	NM_144786	-26.1	0.824355	-25.19	150	GO:0031224,GO:0016747,GO:0016755,GO:0006749,	K00681|1|0.0|1078|mmu:207182|gamma-glutamyltranspeptidase [EC:2.3.2.2]	gi|224994186|ref|NP_659035.2|	0	gamma-glutamyltransferase 7 [Mus musculus]
mmu-miR-101b-5p	NM_178254	-27.2	0.604357	-26.68	157	GO:0043073,GO:0003676,GO:0001071,GO:0050793,GO:0048232,GO:0006351,GO:00082	K09092|1|0.0|657|mmu:277353|transcription factor-like 5	gi|225007623|ref|NP_839985.2|	0	transcription factor-like 5 protein [Mus musculus]
mmu-miR-101b-5p	NM_027143	-20.6	1	-45.6	328	-	-	gi|28972151|dbj|BAC65529.1|	0	mKIAA0323 protein [Mus musculus]
mmu-miR-101b-5p	NM_001033276	-23.6	1	-47.48	305	GO:0043231,GO:0034708,GO:0016740,GO:0043565,GO:0008283,GO:0048545,GO:0030520,GO:0007292,GO:0016568,	K09187|1|0.0|4273|mmu:381022|histone-lysine N-methyltransferase MLL2 [EC:2.1.1.43]	gi|359718904|ref|NP_001028448.3|	0	histone-lysine N-methyltransferase MLL2 [Mus musculus]

mmu-miR-101b-5p	NM_010312	-20.7	0.999988	-27.93	176	GO:0032991,GO:0031234,GO:0044444,GO:0017111,GO:0005488,GO:0060089,GO:0016247,GO:0007606,GO:0007267,GO:0007186,GO:0033762,GO:0009207,GO:00159	K04537|1|0.0|671|ecb:100067486|guanine nucleotide-binding protein G(I)/G(S)/G(T) subunit beta-2	gi|354497166|ref|XP_003510692.1|	0	PREDICTED: guanine nucleotide-binding protein G(I)/G(S)/G(T) subunit beta-2-like [Cricetulus griseus]
mmu-miR-101b-5p	NM_009294	-23.2	0.960174	-23.97	173	GO:0031224,GO:0043234,GO:0044459,GO:0030141,GO:0044431,GO:0008092,GO:0005083,GO:0001775,GO:0019222,GO:0016044,GO:0007599,GO:0048193,GO:0065003,GO:0006904,GO:0015031,	K13502|1|2e-152|539|mmu:20909|syntaxin 4	gi|148685645|gb|EDL17592.1|	0	syntaxin 4A (placental), isoform CRA_a [Mus musculus]

mmu-miR-101b-5p	NM_029277	-22.5	0.999999	-20.32	169	GO:0044444,GO:0008047,GO:0035556,GO:0043087,GO:0009966,	K04450|1|7e-28|127|ptr:739083|activating transcription factor 2!K08878|2|1e-27|127|xtr:100170543|breakpoint cluster region protein [EC:2.7.11.1]!K13736|3|2e-26|123|acs:100558874|Rho GTPase-activating	gi|148691090|gb|EDL23037.1|	0	Rho GTPase activating protein 12, isoform CRA_b [Mus musculus]
mmu-miR-101b-5p	NR_046286	-24.8	0.964007	-26.27	167	GO:0044425,	K06751|1|0.0|460|mmu:15024|major histocompatibility complex, class I	gi|199664|gb|AAA39695.1|	0	MHC H2-TL-T10-129 [Mus musculus]

mmu-miR-101b-5p	NM_145130	-23.7	0.990097	-22.85	166	GO:0031090,GO:0043231,GO:0031224,GO:0008374,GO:0006644,	K13515|1|0.0|900|mmu:14792|lysophospholipid acyltransferase 5 [EC:2.3.1.23 2.3.1.-]	gi|21699058|ref|NP_660112.1|	0	lysophospholipid acyltransferase 5 [Mus musculus]
mmu-miR-101b-5p	NM_029924	-22.4	1	-21.57	165	GO:0005488,	-	gi|50510937|dbj|BAD32454.1|	0	mKIAA1461 protein [Mus musculus]
mmu-miR-101b-5p	NM_153167	-21.7	1	-20.24	164	GO:0031461,GO:0032446,	K11802|1|0.0|922|mmu:242418|WD repeat-containing protein 32	gi|110815804|ref|NP_694807.2|	0	DDB1- and CUL4-associated factor 10 [Mus musculus]
mmu-miR-101b-5p	NM_146153	-20.1	1	-24.02	163	GO:0043234,GO:0031981,GO:0004871,GO:0008134,GO:0003713,GO:0006357,GO:0030518,GO:0006352,	K13112|1|0.0|1041|xla:100505427|thyroid hormone receptor-associated protein 3	gi|347300437|ref|NP_001184195.1|	0	thyroid hormone receptor-associated protein 3 [Xenopus laevis]

mmu-miR-101b-5p	NM_144847	-24.3	0.973332	-24.14	162	GO:0044424,GO:0016301,GO:0032559,GO:0030154,GO:0048731,GO:0006464,GO:00430	K08875|1|8e-173|608|rno:680451|nuclear receptor-binding protein	gi|298286836|sp|Q91V36.2|NRBP2_MOUSE	7.25E-178	RecName: Full=Nuclear receptor-binding protein 2
mmu-miR-101b-5p	NM_199303	-24	0.935789	-21.03	161	GO:0005488,	K05399|1|5e-09|63.5|bta:512242|lipopolysaccharide-binding protein	gi|254675246|ref|NP_955007.2|	0	BPI fold-containing family B member 6 precursor [Mus musculus]
mmu-miR-101b-5p	NM_011812	-22.9	0.999998	-23.19	160	GO:0043231,GO:0044420,GO:0046872,GO:0005102,GO:0085029,GO:0034613,GO:0019430,GO:0031589,	K08023|1|3e-47|192|xla:398322|latent transforming growth factor beta binding protein	gi|6753824|ref|NP_035942.1|	0	fibulin-5 precursor [Mus musculus]

mmu-miR-101b-5p	NM_182716	-25.3	0.99954	-21.81	159	GO:0031224,GO:0033267,GO:0005911,GO:0005488,GO:0008366,GO:0043062,GO:0007043,GO:0006935,GO:0034613,	K06757|1|0.0|1890|pon:100459576|neurofascin	gi|332811712|ref|XP_003308755.1|	0	PREDICTED: neurofascin isoform 1 [Pan troglodytes]
mmu-miR-101b-5p	NM_138744	-24.9	0.976502	-20.73	158	GO:0005911,GO:0043231,GO:0032991,GO:0005515,GO:0007010,	K06085|1|0.0|1153|mmu:99167|synovial sarcoma, X breakpoint 2 interactin	gi|50510713|dbj|BAD32342.1|	0	mKIAA0923 protein [Mus musculus]
mmu-miR-101b-5p	NM_178697	-21.4	1	-22.11	157	GO:0016323,GO:0016021,GO:0005253,GO:0015698,GO:0009987,	K05028|1|0.0|1821|mmu:229933|calcium-activated chloride channel family member 2	gi|30520101|ref|NP_848812.1|	0	calcium-activated chloride channel regulator 2 precursor [Mus musculus]

mmu-miR-101b-5p	NM_177410	-23	0.99811	-22.93	156	GO:0031966,GO:0031224,GO:0005635,GO:0051400,GO:0019903,GO:0042802,GO:0046983,GO:0003677,GO:0001894,GO:0014074,GO:0001934,GO:0035264,GO:0051384,GO:0002244,GO:0048754,GO:0016567,GO:0007409,GO:0001503,GO:0030183,GO:0003008,GO:0007006,GO:00330	K02161|1|3e-98|360|mmu:12043|apoptosis regulator BCL-2	gi|133893254|ref|NP_803129.2|	1.44E-101	apoptosis regulator Bcl-2 isoform 2 [Mus musculus]
mmu-miR-101b-5p	NM_198247	-20.3	1	-23.62	155	GO:0005488,	-	gi|148681014|gb|EDL12961.1|	0	SERTA domain containing 4, isoform CRA_a [Mus musculus]

mmu-miR-101b-5p	NM_198675	-22.3	0.99888	-20.57	154	GO:0004812,GO:0046872,GO:0003723,GO:0032559,GO:0006418,	K03846|1|5e-112|405|xtr:100497477|alpha-1,2-mannosyltransferase [EC:2.4.1.259	gi|148693816|gb|EDL25763.1|	0	RIKEN cDNA D630004A14, isoform CRA_a [Mus musculus]
mmu-miR-101b-5p	NM_177783	-22.9	1	-22.46	153	GO:0031224,GO:0046914,GO:0019787,GO:0043009,GO:0007631,GO:0019941,GO:0007606,GO:0032446,GO:0007635,	K11978|1|0.0|3519|mmu:68795|E3 ubiquitin-protein ligase UBR3 [EC:6.3.2.19]	gi|160948614|ref|NP_808451.2|	0	E3 ubiquitin-protein ligase UBR3 isoform 2 [Mus musculus]
mmu-miR-101b-5p	NR_033639	-26.5	0.148718	-22.85	152	GO:0015935,GO:0031981,GO:0003676,GO:0005198,GO:0006412,GO:0019080,GO:0006810,GO:0031016,GO:0048522,GO:0030099,GO:0042254,GO:0009605,	K02966|1|2e-71|267|rno:29287|small subunit ribosomal protein S19e	gi|56541224|gb|AAH86775.1|	1.34E-70	Rps19 protein, partial [Mus musculus]

mmu-miR-101b-5p	NM_146200	-21.3	0.999999	-22.32	151	GO:0043234,GO:0044444,GO:0043021,GO:0008135,GO:0006412,	K03252|1|0.0|1498|mmu:56347|translation initiation factor 3 subunit C	gi|22203755|ref|NP_666312.1|	0	eukaryotic translation initiation factor 3 subunit C [Mus musculus]
mmu-miR-101b-5p	NM_025533	-23.4	0.9892	-22.73	150	GO:0044444,GO:0043231,GO:0005488,GO:0006807,GO:0032769,	K13125|1|5e-171|602|rno:292894|nitric oxide synthase-interacting protein	gi|13384956|ref|NP_079809.1|	1.41E-171	nitric oxide synthase-interacting protein isoform 1 [Mus musculus]
mmu-miR-101b-5p	NR_002870	-23.8	0.999991	-21.33	149	-	-	gi|51890509|dbj|BAD42838.1|	2.23E-85	hypothetical protein [Mus musculus]
mmu-miR-101b-5p	NM_213728	-24.8	0.835422	-20.49	148	GO:0005882,	K07605|1|0.0|890|mmu:105866|type II keratin, basic	gi|47523977|ref|NP_998893.1|	0	keratin, type II cytoskeletal 72 [Mus musculus]
mmu-miR-101b-5p	NM_008891	-25.9	0.917388	-21.13	147	GO:0043234,GO:0005681,GO:0016604,GO:0005911,GO:0005488,GO:0000377,GO:00071	K13114|1|0.0|726|mmu:18949|pinin	gi|148704736|gb|EDL36683.1|	0	pinin [Mus musculus]

mmu-miR-101b-5p	NM_001081064	-22.2	1	-20.1	146	GO:0030054,GO:0043231,GO:0044430,GO:0007165,	K06095|1|5e-31|139|gga:427360|multiple PDZ domain protein	gi|124486716|ref|NP_001074533.1|	0	PDZ domain-containing protein 2 [Mus musculus]
mmu-miR-101b-5p	NM_028665	-23.4	0.996973	-21.16	145	-	K15503|1|1e-37|159|spu:755521|serine/threonine-protein phosphatase 6 regulatory ankyrin repeat subunit B	gi|354489835|ref|XP_003507066.1|	0	PREDICTED: ankyrin repeat domain-containing protein 42-like [Cricetulus griseus]
mmu-miR-101b-5p	NR_040438	NA	NA	-20.94	157	-	-	gi|148707218|gb|EDL39165.1|	1.43E-43	mCG145609, isoform CRA_a [Mus musculus]
mmu-miR-101b-5p	NR_028300	NA	NA	-20.55	156	-	-	gi|26342773|dbj|BAC35043.1|	6.10E-72	unnamed protein product [Mus musculus]

mmu-miR-101b-5p	NM_008969	NA	NA	-20.61	150	GO:0031513,GO:0043231,GO:0031090,GO:0012505,GO:0016705,GO:0016701,GO:0005506,GO:0016209,GO:0014060,GO:0008283,GO:0019229,GO:0051384,GO:0001516,GO:0006939,GO:0006950,GO:0014061,GO:00	K00509|1|0.0|1139|mmu:19224|prostaglandin-endoperoxide synthase 1 [EC:1.14.99.1]	gi|6679537|ref|NP_032995.1|	0	prostaglandin G/H synthase 1 precursor [Mus musculus]

mmu-miR-101b-5p	NM_013716	NA	NA	-20.68	146	GO:0005737,GO:0016020,GO:0003724,GO:0003676,GO:0004518,GO:0003678,GO:0000166,GO:0007264,	K12741|1|3e-08|61.6|dan:Dana_GF18582|heterogeneous nuclear ribonucleoprotein A1/A3!K01509|2|9e-08|60.1|dsi:Dsim_GD21374|adenosinetriphosphatase [EC:3.6.1.3]	gi|60359872|dbj|BAD90155.1|	0	mKIAA4115 protein [Mus musculus]
mmu-miR-101b-5p	NM_001177771	-21.3	0.999992	NA	NA	GO:0043231,GO:0031224,GO:0005253,GO:0015698,	-	gi|148670033|gb|EDL01980.1|	0	chloride channel CLIC-like 1, isoform CRA_c [Mus musculus]
mmu-miR-101b-5p	NM_175442	-23.3	0.998404	NA	NA	GO:0031224,GO:0001608,GO:0007166,	K04274|1|1e-141|504|ecb:100072611|purinergic receptor P2Y, G protein-coupled, 10	gi|30425170|ref|NP_780651.1|	0	purinergic receptor P2Y G-protein coupled 10-like [Mus musculus]

mmu-miR-101b-5p	NM_001142642	-21.1	1	NA	NA	-	-	gi|217416345|ref|NP_001136114.1|	0	fibrosin-like 1 isoform 1 [Mus musculus]
mmu-miR-101b-5p	NM_178594	-24.9	0.945007	NA	NA	GO:0031224,GO:0005515,GO:0050863,	K06747|1|2e-135|484|mmu:242122|immune costimulatory protein	gi|260166624|ref|NP_848709.2|	1.34E-134	V-set domain containing T-cell activation inhibitor 1 precursor [Mus musculus]
mmu-miR-101b-5p	NM_146682	-23.9	0.79676	NA	NA	GO:0031224,GO:0004930,GO:0023052,GO:0007606,	K04257|1|0.0|636|mmu:258677|olfactory receptor	gi|33239080|ref|NP_666893.1|	0	olfactory receptor 76 [Mus musculus]
mmu-miR-101b-5p	NM_010636	-22.2	1	NA	NA	GO:0043231,GO:0003712,GO:0003676,GO:0046914,GO:0001071,GO:0006357,	K09205|1|0.0|644|mmu:16597|krueppel-like factor 8/12	gi|46048263|ref|NP_034766.2|	0	Krueppel-like factor 12 [Mus musculus]

mmu-miR-101b-5p	NM_172490	-20.4	1	NA	NA	GO:0043231,GO:0003723,GO:0016740,GO:0048037,GO:0006451,	K03341|1|0.0|1004|mmu:211006|O-phospho-L-seryl-tRNASec:L-selenocysteinyl-tRNA synthase [EC:2.9.1.2]	gi|27369676|ref|NP_766078.1|	0	O-phosphoseryl-tRNA(Sec) selenium transferase [Mus musculus]
mmu-miR-101b-5p	NR_040443	-21.3	0.999999	NA	NA	-	-	gi|148697976|gb|EDL29923.1|	1.61E-27	mCG1049130 [Mus musculus]
mmu-miR-101b-5p	NM_001080707	-22.6	0.999536	NA	NA	GO:0031224,GO:0007165,GO:0006810,	-	gi|123959716|ref|NP_001074176.1|	0	integral membrane protein GPR155 isoform 2 [Mus musculus]
mmu-miR-101b-5p	NM_134127	-20.1	1	NA	NA	GO:0031224,GO:0043231,GO:0031090,GO:0050543,GO:0016709,GO:0016713,GO:0016712,GO:0005506,GO:0000038,GO:0006690,GO:0042221,	K00490|1|0.0|1023|mmu:106648|cytochrome P450, family 4, subfamily F (leukotriene-B4 20-monooxygenase) [EC:1.14.13.30]	gi|148708351|gb|EDL40298.1|	0	cytochrome P450, family 4, subfamily f, polypeptide 15, isoform CRA_b [Mus musculus]

mmu-miR-101b-5p	NM_001242365	-20.1	1	NA	NA	GO:0043234,GO:0007041,GO:0016197,GO:0007033,	-	gi|334358881|ref|NP_001229294.1|	0	FTS and Hook-interacting protein isoform c [Mus musculus]
mmu-miR-101b-5p	NM_183018	-21.1	1	NA	NA	GO:0044459,GO:0005083,GO:0046914,GO:0019904,GO:0019222,GO:0023061,GO:0006887,GO:0048168,GO:0001505,GO:0065003,GO:0031340,GO:0015031,	K15291|1|0.0|2275|mmu:116837|regulating synaptic membrane exocytosis protein 1	gi|60460915|ref|NP_898839.2|	0	regulating synaptic membrane exocytosis protein 1 isoform 2 [Mus musculus]
mmu-miR-101b-5p	NM_001029836	-22.5	0.999976	NA	NA	-	K06824|1|0.0|1041|mmu:114249|nephronectin	gi|71067128|ref|NP_001025007.1|	0	nephronectin isoform b precursor [Mus musculus]
mmu-miR-101b-5p	NM_001135577	-20.2	1	NA	NA	-	-	gi|148709026|gb|EDL40972.1|	2.77E-45	mCG10996 [Mus musculus]
mmu-miR-101b-5p	NM_013917	-20.8	0.999989	NA	NA	-	K06635|1|3e-95|349|mmu:30939|securin	gi|197085601|ref|NP_038945.2|	2.11E-103	securin isoform 2 [Mus musculus]
mmu-miR-101b-5p	NM_025542	-20.8	0.999999	NA	NA	-	-	gi|148674659|gb|EDL06606.1|	1.46E-176	mCG2574 [Mus musculus]

mmu-miR-101b-5p	NM_001243043	-20.8	1	NA	NA	GO:0005764,GO:0030136,GO:0030119,GO:0012506,GO:0044431,GO:0005488,GO:0022892,GO:0006901,GO:0048193,GO:0050688,GO:0015031,	K12392|1|0.0|1668|mmu:11764|AP-1 complex subunit beta-1	gi|339895913|ref|NP_001229972.1|	0	AP-1 complex subunit beta-1 isoform 1 [Mus musculus]
mmu-miR-101b-5p	NM_001193660	-28.7	0.573944	NA	NA	GO:0043296,GO:0031224,GO:0005515,GO:0016337,GO:0007043,	K06087|1|3e-07|58.9|mgp:100540364|claudin	gi|148682691|gb|EDL14638.1|	5.44E-140	claudin 12, isoform CRA_a [Mus musculus]
mmu-miR-101b-5p	NM_183427	-20.8	1	NA	NA	GO:0044459,GO:0034702,GO:0005253,GO:0004871,GO:0016597,GO:0005237,GO:0007186,GO:0007399,GO:0015698,	K05194|1|0.0|831|mmu:237213|glycine receptor alpha-2	gi|148708798|gb|EDL40745.1|	0	glycine receptor, alpha 2 subunit, isoform CRA_a [Mus musculus]

mmu-miR-101b-5p	NR_040407	-27.9	0.342109	NA	NA	-	K04405|1|6e-13|76.3|mmu:26940|evolutionarily conserved signaling intermediate in Toll pathways	gi|150382601|sp|Q9QZH6.2|ECSIT_MOUSE	4.08E-12	RecName: Full=Evolutionarily conserved signaling intermediate in Toll pathway, mitochondrial; AltName: Full=Protein SITPEC; Flags: Precursor
mmu-miR-101b-5p	NM_022325	-23.3	0.95625	NA	NA	GO:0044421,GO:0000323,GO:0004175,GO:0019538,	K08568|1|1e-174|613|mmu:64138|cathepsin X [EC:3.4.18.1]	gi|11968166|ref|NP_071720.1|	8.79E-174	cathepsin Z preproprotein [Mus musculus]
mmu-miR-101b-5p	NM_001093766	-23.7	0.994915	NA	NA	GO:0031224,	-	gi|148699227|gb|EDL31174.1|	2.62E-179	myeloid-associated differentiation marker, isoform CRA_b [Mus musculus]
mmu-miR-101b-5p	NM_053100	-27.1	0.76076	NA	NA	GO:0016604,GO:0042802,GO:0046914,GO:0019787,GO:0032446,	K12001|1|0.0|1224|cfa:486863|tripartite motif-containing protein 8	gi|148710059|gb|EDL42005.1|	0	tripartite motif protein 8, isoform CRA_b [Mus musculus]
mmu-miR-101b-5p	NM_026393	-21.5	0.997586	NA	NA	GO:0044444,GO:0043231,	-	gi|24431937|ref|NP_080669.1|	3.02E-177	nmrA-like family domain-containing protein 1 [Mus musculus]

mmu-miR-101b-5p	NM_001168292	-21.8	1	NA	NA	GO:0005488,	K02360|1|1e-26|123|aag:AaeL_AAEL009189|a component of the cytoplasm (encore)	gi|28972564|dbj|BAC65698.1|	0	mKIAA1002 protein [Mus musculus]
mmu-miR-101b-5p	NM_010729	-23.7	0.997451	NA	NA	GO:0031012,GO:0016638,GO:0046914,GO:0006464,	K14678|1|0.0|990|mmu:16949|lysyl oxidase-like protein 1 [EC:1.4.3.-]	gi|45598390|ref|NP_034859.2|	0	lysyl oxidase homolog 1 precursor [Mus musculus]
mmu-miR-101b-5p	NM_027745	-20.4	1	NA	NA	-	K10421|1|6e-17|90.9|dan:Dana_GF14794|CAP-Gly domain-containing linker protein 1!K10352|4|1e-11|73.6|ame:412092|myosin heavy chain	gi|58037237|ref|NP_082021.1|	0	coiled-coil domain-containing protein 57 [Mus musculus]

mmu-miR-101b-5p	NM_025638	-21.8	0.999965	NA	NA	GO:0044424,GO:0031224,GO:0043169,GO:0008081,GO:0019400,	K01126|1|1e-09|66.2|cel:T12B3.3|glycerophosphoryl diester phosphodiesterase [EC:3.1.4.46]	gi|12847642|dbj|BAB27650.1|	0	unnamed protein product [Mus musculus]
mmu-miR-101b-5p	NM_177006	-20.9	1	NA	NA	-	K11127|1|3e-13|80.1|bfo:BRAFLDRAFT_128562|telomerase protein component 1	gi|222418589|ref|NP_795980.2|	0	leucine-rich repeat and WD repeat-containing protein KIAA1239 [Mus musculus]
mmu-miR-101b-5p	NM_007414	-28.2	0.333363	NA	NA	GO:0046872,GO:0016799,GO:0006464,	K01245|1|0.0|758|mmu:11544|ADP-ribosylarginine hydrolase [EC:3.2.2.19]	gi|6680658|ref|NP_031440.1|	0	[Protein ADP-ribosylarginine] hydrolase [Mus musculus]
mmu-miR-101b-5p	NM_009517	-21.9	1	NA	NA	GO:0031981,GO:0003676,GO:0046914,GO:0012502,GO:0006810,GO:0016049,GO:00335	K10137|1|2e-165|585|mmu:22401|zinc finger, matrin type 3	gi|101943566|ref|NP_033543.2|	1.06E-164	zinc finger matrin-type protein 3 [Mus musculus]

mmu-miR-101b-5p	NM_013624	-24.2	0.999993	NA	NA	GO:0031012,GO:0044444,GO:0016020,GO:0004553,GO:0007599,GO:0019566,GO:0050954,GO:0007626,	K03900|1|1e-180|636|cfa:399544|von Willebrand factor	gi|190358487|ref|NP_038652.2|	0	otogelin precursor [Mus musculus]
mmu-miR-101b-5p	NM_013874	-22.2	0.999678	NA	NA	GO:0070603,GO:0046914,GO:0012502,GO:0048731,GO:0006351,	K13196|1|1e-128|461|xtr:100126056|zinc finger protein ubi-d4	gi|7305309|ref|NP_038902.1|	0	zinc finger protein neuro-d4 [Mus musculus]
mmu-miR-101b-5p	NM_001164576	-22	0.999771	NA	NA	GO:0043231,GO:0046872,GO:0001071,GO:0006351,	K09228|1|5e-51|203|mmu:67911|KRAB domain-containing zinc finger protein	gi|257153352|ref|NP_001158048.1|	3.76E-71	GLI-Kruppel family member HKR1 isoform b [Mus musculus]
mmu-miR-101b-5p	NM_030699	-21.3	1	NA	NA	GO:0031225,GO:0005488,GO:0048667,	K07522|1|0.0|1119|rno:295382|netrin G1	gi|13507636|ref|NP_109624.1|	0	netrin-G1 isoform a precursor [Mus musculus]

mmu-miR-101b-5p	NM_008332	-24.2	0.996403	NA	NA	GO:0005488,GO:0043393,GO:0019221,GO:0035455,GO:0051707,	K14217|1|4e-93|344|ecb:100066067|interferon-induced protein with tetratricopeptide repeats 1	gi|6680363|ref|NP_032358.1|	0	interferon-induced protein with tetratricopeptide repeats 2 [Mus musculus]
mmu-miR-101b-5p	NM_028593	-21.8	1	NA	NA	GO:0005903,GO:0031224,GO:0043169,GO:0016723,GO:0006810,GO:0010038,GO:0006875,GO:0006091,	K08370|1|2e-146|521|mmu:73649|cytochrome b reductase 1	gi|167000071|ref|NP_082869.2|	1.24E-145	cytochrome b reductase 1 [Mus musculus]

mmu-miR-101b-5p	NM_013834	-24.4	0.996594	NA	NA	GO:0016020,GO:0044444,GO:0031012,GO:0001664,GO:0004888,GO:0019904,GO:0061135,GO:0004175,GO:0005539,GO:0010952,GO:0034695,GO:0000737,GO:0033144,GO:0043065,GO:0002762,GO:0006357,GO:0060442,GO:0050796,GO:0010165,GO:0070555,GO:0014020,GO:00713	K02166|1|2e-155|551|mmu:20377|secreted frizzled-related protein 1	gi|65301472|ref|NP_038862.2|	1.58E-154	secreted frizzled-related protein 1 precursor [Mus musculus]
mmu-miR-101b-5p	NM_178730	-22.1	0.999999	NA	NA	GO:0016021,GO:0004175,GO:0019538,	K09752|1|0.0|833|mmu:243083|transmembrane protease, serine 11F [EC:3.4.21.-]	gi|32189423|ref|NP_848845.1|	0	transmembrane protease serine 11F [Mus musculus]

mmu-miR-101b-5p	NM_026696	-24	0.999095	NA	NA	-	-	gi|226437615|ref|NP_080972.1|	3.98E-71	UPF0561 protein C2orf68 homolog [Mus musculus]
mmu-miR-101b-5p	NM_175318	-20.1	1	NA	NA	-	K15193|1|0.0|868|mmu:101685|protein SPT2	gi|26326843|dbj|BAC27165.1|	0	unnamed protein product [Mus musculus]
mmu-miR-101b-5p	NM_172589	-23.8	0.999241	NA	NA	GO:0031224,	-	gi|27369844|ref|NP_766177.1|	2.29E-113	lipoma HMGIC fusion partner-like 2 protein [Mus musculus]
mmu-miR-101b-5p	NM_028651	-21.3	1	NA	NA	GO:0031224,	K09667|1|4e-21|104|nve:NEMVE_v1g239520|polypeptide N-acetylglucosaminyltransferase [EC:2.4.1.-]	gi|148668309|gb|EDL00639.1|	0	transmembrane and tetratricopeptide repeat containing 4, isoform CRA_c [Mus musculus]
mmu-miR-101b-5p	NM_146228	-21.5	1	NA	NA	GO:0031410,GO:0008047,GO:0005088,GO:0005515,GO:0006996,GO:0043087,GO:0033036,GO:0007266,	K04575|1|3e-139|498|mdo:100019560|amyotrophic lateral sclerosis 2 (juvenile)	gi|225690620|ref|NP_666340.3|	0	ALS2 C-terminal-like protein [Mus musculus]

mmu-miR-101b-5p	NM_011933	-21	0.999996	NA	NA	GO:0042579,GO:0030529,GO:0016776,GO:0016628,GO:0046872,GO:0032559,GO:0009209,GO:0006810,GO:0009206,GO:00104	K13237|1|1e-155|551|mmu:26378|peroxisomal 2,4-dienoyl-CoA reductase [EC:1.3.1.34]	gi|6753622|ref|NP_036063.1|	6.68E-155	peroxisomal 2,4-dienoyl-CoA reductase [Mus musculus]

mmu-miR-101b-5p	NM_172833	-21.4	1	NA	NA	GO:0031981,GO:0043234,GO:0044444,GO:0019787,GO:0060089,GO:0008047,GO:0004175,GO:0005515,GO:0006461,GO:0002335,GO:0002369,GO:0051091,GO:0006952,GO:0016567,GO:0050863,GO:0016310,GO:0001819,GO:0043066,GO:0006913,GO:0007249,GO:0050854,GO:00517	K07369|1|0.0|1546|mmu:240354|mucosa-associated lymphoid tissue lymphoma translocation protein 1 [EC:3.4.22.-]	gi|148677744|gb|EDL09691.1|	0	mucosa associated lymphoid tissue lymphoma translocation gene 1, isoform CRA_b [Mus musculus]

mmu-miR-101b-5p	NM_001253806	-20.7	1	NA	NA	GO:0032991,GO:0044430,GO:0048770,GO:0005515,GO:0042221,GO:0007599,GO:0006605,GO:0016070,GO:0002441,GO:0001775,GO:0043066,	K06630|1|8e-129|462|mdo:100030704|tyrosine 3-monooxygenase/tryptophan 5-monooxygenase activation protein	gi|148676868|gb|EDL08815.1|	8.44E-129	tyrosine 3-monooxygenase/tryptophan 5-monooxygenase activation protein, zeta polypeptide, isoform CRA_b [Mus musculus]
mmu-miR-101b-5p	NM_011341	-20.7	1	NA	NA	GO:0005768,GO:0042995,GO:0044431,GO:0016020,GO:0046872,GO:0005515,GO:0009411,GO:0022037,GO:0017156,GO:0030154,GO:0010033,	-	gi|148683118|gb|EDL15065.1|	0	stromal cell derived factor 4, isoform CRA_b [Mus musculus]

mmu-miR-101b-5p	NM_010099	-21	1	NA	NA	GO:0016021,GO:0031090,GO:0005515,GO:0008544,GO:0042476,GO:0051091,GO:0048732,GO:0060605,GO:0042345,GO:0007249,GO:0031589,GO:0060788,GO:00301	K05480|1|5e-106|387|mmu:13607|ectodysplasin-A	gi|6753712|ref|NP_034229.1|	9.68E-108	ectodysplasin-A isoform 1 [Mus musculus]
mmu-miR-101b-5p	NM_001033043	-21.3	1	NA	NA	GO:0043231,GO:0042802,GO:0046914,GO:0016787,GO:0007281,GO:0048232,	K15979|1|5e-07|58.5|nve:NEMVE_v1g183195|staphylococcal nuclease domain-containing protein 1	gi|74271882|ref|NP_001028215.1|	0	RING finger protein 17 [Mus musculus]

mmu-miR-101b-5p	NM_001163852	-23.2	1	NA	NA	-	K02155|1|0.0|838|gga:416753|V-type H+-transporting ATPase 16kDa proteolipid subunit [EC:3.6.3.14]!K12826|2|3e-09|66.6|mdo:100014243|splicing factor 3A subunit 2	gi|37360272|dbj|BAC98114.1|	0	mKIAA1171 protein [Mus musculus]
mmu-miR-101b-5p	NR_045140	-20.3	0.999901	NA	NA	-	-	gi|148697446|gb|EDL29393.1|	6.01E-38	mCG8910 [Mus musculus]
mmu-miR-101b-5p	NM_008883	-22.9	1	NA	NA	GO:0031224,GO:0004872,GO:0021859,GO:0021543,GO:0030517,GO:0006935,GO:00230	K06820|1|0.0|3719|mmu:18846|plexin A	gi|124286839|ref|NP_032909.2|	0	plexin-A3 precursor [Mus musculus]

mmu-miR-101b-5p	NM_001253696	-23.3	0.997826	NA	NA	GO:0043231,GO:0003676,GO:0001071,GO:0010467,	K09228|1|5e-17|90.9|mmu:22688|KRAB domain-containing zinc finger protein	gi|225543502|ref|NP_081524.2|	0	zinc finger protein 219 isoform 1 [Mus musculus]
mmu-miR-101b-5p	NM_001163223	-21.2	1	NA	NA	GO:0046872,	-	gi|253735674|ref|NP_001156695.1|	0	zinc finger protein 804B [Mus musculus]
mmu-miR-101b-5p	NM_001252440	-20.1	1	NA	NA	-	-	gi|165972317|ref|NP_080243.3|	4.21E-166	protein EURL homolog [Mus musculus]
mmu-miR-101c	NM_001033535	-29.9	0.083907	-26.1	177	-	-	gi|109484717|ref|XP_001072284.1|	2.40E-121	PREDICTED: tumor necrosis factor alpha-induced protein 8-like protein 3-like [Rattus norvegicus]
mmu-miR-101c	NM_001081351	-30.7	0.152864	-25.76	164	GO:0043231,	-	gi|124486851|ref|NP_001074820.1|	0	uncharacterized protein LOC214642 [Mus musculus]

mmu-miR-101c	NM_001184706	-29.2	0.402404	-26.31	168	GO:0043231,GO:0043234,GO:0012505,GO:0000989,GO:0003676,GO:0001071,GO:0005515,GO:0048513,GO:0006351,	K09392|1|2e-175|619|aml:100464401|transcription factor Dp-2	gi|62089560|gb|AAH92230.1|	0	Tfdp2 protein, partial [Mus musculus]
mmu-miR-101c	NM_001184708	-29.2	0.390006	-26.31	168	GO:0043231,GO:0043234,GO:0012505,GO:0000989,GO:0003676,GO:0001071,GO:0005515,GO:0048513,GO:0006351,	K09392|1|1e-130|469|mmu:211586|transcription factor Dp-2	gi|296040467|ref|NP_001171639.1|	1.01E-129	transcription factor Dp-2 isoform C [Mus musculus]
mmu-miR-101c	NM_001184709	-29.2	0.385315	-26.31	168	GO:0043231,GO:0043234,GO:0012505,GO:0000989,GO:0003676,GO:0001071,GO:0005515,GO:0048513,GO:0006351,	K09392|1|3e-171|604|aml:100464401|transcription factor Dp-2	gi|62089560|gb|AAH92230.1|	7.50E-178	Tfdp2 protein, partial [Mus musculus]

mmu-miR-101c	NM_001184710	-29.2	0.379554	-26.31	168	GO:0043231,GO:0043234,GO:0012505,GO:0000989,GO:0003676,GO:0001071,GO:0005515,GO:0048513,GO:0006351,	K09392|1|2e-164|582|mmu:211586|transcription factor Dp-2	gi|182765448|ref|NP_848782.2|	1.35E-163	transcription factor Dp-2 isoform A [Mus musculus]
mmu-miR-101c	NM_001184711	-29.2	0.384528	-26.31	168	GO:0043231,GO:0043234,GO:0012505,GO:0000989,GO:0003676,GO:0001071,GO:0005515,GO:0048513,GO:0006351,	K09392|1|7e-178|626|pon:100448153|transcription factor Dp-2	gi|62089560|gb|AAH92230.1|	0	Tfdp2 protein, partial [Mus musculus]
mmu-miR-101c	NM_172417	-30.2	0.143628	-28.64	168	-	K10431|1|5e-08|61.6|tgu:100222640|microtubule-associated protein 4!K04989|2|1e-07|60.1|rno:100364048|polycystin 1L3	gi|161377418|ref|NP_766005.2|	0	uncharacterized protein C1orf170 homolog [Mus musculus]

mmu-miR-101c	NM_172430	-27.2	0.661557	-25.85	163	-	-	gi|170014712|ref|NP_766018.3|	0	A-kinase anchor protein SPHKAP [Mus musculus]
mmu-miR-101c	NM_178667	-29.2	0.402448	-26.31	168	GO:0043231,GO:0043234,GO:0012505,GO:0000989,GO:0003676,GO:0001071,GO:0005515,GO:0006351,	K09392|1|0.0|649|aml:100464401|transcription factor Dp-2	gi|8039811|sp|Q64163.2|TFDP2_MOUSE	0	RecName: Full=Transcription factor Dp-2; AltName: Full=Dp-3; AltName: Full=E2F dimerization partner 2
mmu-miR-101c	NM_001105184	-25.8	0.52949	-24.82	173	-	K04613|1|0.0|1008|rno:293069|vomeronasal 2 receptor	gi|157384952|ref|NP_001098654.1|	0	vomeronasal receptor Vmn2r71 precursor [Mus musculus]
mmu-miR-101c	NM_021716	-22.6	0.999989	-20.71	172	GO:0034399,GO:0005515,GO:0016462,GO:0032559,	K13254|1|6e-65|251|phu:Phum_PHUM514830|spastin [EC:3.6.4.3]	gi|148695049|gb|EDL26996.1|	0	fidgetin, isoform CRA_b [Mus musculus]

mmu-miR-101c	NR_028354	-26.4	0.319365	-23.96	171	-	K01597|1|4e-179|628|mmu:192156|diphosphomevalonate decarboxylase [EC:4.1.1.33]	gi|256985114|ref|NP_619597.2|	2.93E-178	diphosphomevalonate decarboxylase [Mus musculus]
mmu-miR-101c	NM_080788	-20.5	1	-24.43	169	GO:0004672,GO:0032559,GO:0006468,	K08815|1|0.0|2242|mmu:140810|tau tubulin kinase [EC:2.7.11.26]	gi|67906805|ref|NP_542966.2|	0	tau-tubulin kinase 2 isoform 1 [Mus musculus]
mmu-miR-101c	NM_146530	-23.1	0.65273	-20.12	167	GO:0004871,GO:0023052,	K04257|1|2e-166|585|mmu:258523|olfactory	gi|282847422|ref|NP_666741.2|	1.06E-165	olfactory receptor 1186 [Mus musculus]

mmu-miR-101c	NM_001081076	-23.6	0.962236	-21.68	166	GO:0031224,GO:0009986,GO:0044424,GO:0016829,GO:0032561,GO:0009975,GO:0001653,GO:0016301,GO:0032559,GO:0009124,GO:0006164,GO:0007166,GO:00064	K12323|1|6e-154|546|tad:TRIADDRAFT_30655|atrial natriuretic peptide receptor A [EC:4.6.1.2]	gi|124487301|ref|NP_001074545.1|	0	guanylate cyclase 2G precursor [Mus musculus]
mmu-miR-101c	NM_198674	-22.3	0.94122	-20.05	165	-	K10267|1|0.0|797|mmu:320083|F-box and WD-40 domain protein 12/13/14/15/16/17/19	gi|38348578|ref|NP_941076.1|	0	F-box and WD-40 domain protein 26 [Mus musculus]
mmu-miR-101c	NR_033336	-24.8	0.026065	-20.16	164	-	-	-	-	-
mmu-miR-101c	NR_029463	-24.3	0.837125	-21.35	163	-	-	gi|68062078|ref|XP_673041.1|	1.72E-16	hypothetical protein [Plasmodium berghei strain ANKA]
mmu-miR-101c	NR_033502	-26.4	0.476919	-22.94	161	-	-	-	-	-

mmu-miR-101c	NM_194355	-27.7	0.483748	-23.99	160	GO:0043231,GO:0044444,GO:0005515,GO:0016192,	K02098|1|0.0|1063|rno:307348|spire	gi|148677692|gb|EDL09639.1|	0	spire homolog 1 (Drosophila), isoform CRA_c [Mus musculus]
mmu-miR-101c	NM_178744	-20.6	0.999998	-20.09	159	GO:0043231,GO:0046914,GO:0003676,GO:0010467,	K10488|1|0.0|1414|mmu:268564|zinc finger and BTB domain-containing protein 1	gi|148704515|gb|EDL36462.1|	0	zinc finger and BTB domain containing 1 [Mus musculus]
mmu-miR-101c	NM_028785	-25.4	0.94141	-20.89	158	GO:0044444,GO:0032561,GO:0030695,GO:0019899,GO:0007599,	K05727|1|2e-13|80.5|nvi:100118634|dedicator of cytokinesis 3/4/5!K13708|2|4e-13|79.7|tca:661070|dedicator of cytokinesis 1	gi|62241030|ref|NP_083061.2|	0	dedicator of cytokinesis protein 8 [Mus musculus]

mmu-miR-101c	NM_172468	-20	1	-23.32	157	GO:0044424,GO:0005543,GO:0009987,GO:0006810,	K08545|1|2e-08|63.9|ssc:100155153|nuclear receptor subfamily 2 group E member 1	gi|33859789|ref|NP_766056.1|	0	sorting nexin-30 [Mus musculus]
mmu-miR-101c	NM_011623	-24.1	0.968418	-20.5	156	GO:0043234,GO:0000794,GO:0005813,GO:0003916,GO:0001071,GO:0032559,GO:0042623,GO:0042802,GO:0019901,GO:0032182,GO:0046983,GO:0006915,GO:0006357,GO:0048017,GO:0007059,GO:0009790,GO:0009207,GO:0030261,GO:0006260,GO:0021533,GO:0033554,GO:00071	K03164|1|0.0|2713|mmu:21973|DNA topoisomerase II [EC:5.99.1.3]	gi|153945749|ref|NP_035753.2|	0	DNA topoisomerase 2-alpha [Mus musculus]

mmu-miR-101c	NM_172952	-22.8	0.982738	-22.21	155	-	K15376|1|0.0|1389|rno:64845|gephyrin [EC:2.10.1.1 2.7.7.75]	gi|269973915|ref|NP_766540.2|	0	gephyrin isoform 2 [Mus musculus]
mmu-miR-101c	NM_177642	-24.4	0.992946	-22.32	154	GO:0031674,GO:0043231,	K12567|1|1e-76|290|aml:100476638|titin [EC:2.7.11.1]	gi|310923113|ref|NP_808310.2|	0	immunoglobulin-like and fibronectin type III domain-containing protein 1 [Mus musculus]
mmu-miR-101c	NM_025549	-22.3	0.800958	-20.4	153	GO:0023052,	-	gi|110611933|ref|NP_079825.2|	7.91E-161	arrestin domain containing 4 isoform 2 [Mus musculus]
mmu-miR-101c	NM_177185	-21.6	1	-21.56	152	-	-	gi|148681685|gb|EDL13632.1|	0	RIKEN cDNA D130059P03, isoform CRA_a [Mus musculus]
mmu-miR-101c	NR_045808	-21.2	0.727778	-20.6	151	-	-	gi|148682828|gb|EDL14775.1|	4.16E-06	mCG1046009 [Mus musculus]
mmu-miR-101c	NM_025453	-20.8	0.992523	-20.94	150	GO:0031224,	-	gi|13384858|ref|NP_079729.1|	1.43E-116	transmembrane 4 L6 family member 20 [Mus musculus]
mmu-miR-101c	NM_177747	-22.3	0.998598	-20.44	149	GO:0043231,GO:0046914,GO:0003677,GO:0006355,	K09228|1|2e-64|249|bfo:BRAFLDRAFT_71028|KRAB domain-containing zinc finger protein	gi|125347348|ref|NP_808415.2|	0	zinc finger protein 711 [Mus musculus]

mmu-miR-101c	NM_026908	-20.9	0.999893	-21.38	148	GO:0044444,GO:0005488,GO:0019217,GO:0022402,GO:0007169,	K08272|1|0.0|663|mmu:69008|calcium binding protein 39	gi|31541826|ref|NP_081184.3|	0	calcium-binding protein 39-like [Mus musculus]
mmu-miR-101c	NM_175089	-21.8	0.999709	-20.25	145	GO:0043231,GO:0043169,GO:0004672,GO:0032559,GO:0006464,GO:0000087,	K08857|1|0.0|2262|mmu:18004|NIMA (never in mitosis gene a)-related kinase [EC:2.7.11.1]	gi|124107627|ref|NP_780298.2|	0	serine/threonine-protein kinase Nek1 [Mus musculus]
mmu-miR-101c	NM_001040396	NA	NA	-20.9	151	GO:0005488,GO:0019725,GO:0006451,	-	gi|358410384|ref|XP_003581804.1|	3.51E-96	PREDICTED: LOW QUALITY PROTEIN: selenoprotein T [Bos taurus]
mmu-miR-101c	NM_001014976	-20.9	1	NA	NA	GO:0005815,GO:0043231,GO:0070011,GO:0005488,GO:0012501,GO:0007126,GO:0000819,GO:0007063,GO:0019538,GO:0007051,GO:0030071,	K02365|1|0.0|3994|mmu:105988|separase [EC:3.4.22.49]	gi|148672045|gb|EDL03992.1|	0	extra spindle poles-like 1 (S. cerevisiae), isoform CRA_b [Mus musculus]

mmu-miR-101c	NM_001113246	-20.9	0.999992	NA	NA	GO:0044444,GO:0043169,GO:0030674,GO:0008047,GO:0035556,GO:0043087,GO:0009966,	K04450|1|0.0|916|ptr:739083|activating transcription factor 2!K02649|2|3e-38|162|bfo:BRAFLDRAFT_91712|phosphoinositide-3-kinase, regulatory subunit!K10360|3|2e-37|159|tca:656579|	gi|262205420|ref|NP_001106717.2|	0	N-chimaerin isoform 3 [Mus musculus]
mmu-miR-101c	NM_203396	-20.4	0.999993	NA	NA	-	K04257|1|3e-06|55.5|mdo:100012901|olfactory receptor	gi|42794267|ref|NP_981933.1|	0	protein FAM115E [Mus musculus]
mmu-miR-101c	NM_020267	-22.4	0.999626	NA	NA	GO:0044464,GO:0046914,	K12020|1|6e-98|360|mmu:80985|tripartite motif-containing protein 44	gi|148695720|gb|EDL27667.1|	3.94E-97	tripartite motif-containing 44, isoform CRA_a [Mus musculus]

mmu-miR-101c	NM_001042752	-21.5	1	NA	NA	GO:0016021,GO:0031981,GO:0004871,GO:0050839,GO:0006935,GO:0000768,GO:0045597,GO:0006351,	K06766|1|0.0|2664|mmu:18007|neogenin	gi|148694013|gb|EDL25960.1|	0	neogenin, isoform CRA_a [Mus musculus]
mmu-miR-101c	NM_175114	-21.7	0.999935	NA	NA	GO:0005768,GO:0005102,GO:0007041,GO:0006355,GO:0006486,GO:0006886,	K15369|1|0.0|1740|mmu:67095|trafficking kinesin-binding protein 1	gi|124517685|ref|NP_780323.2|	0	trafficking kinesin-binding protein 1 [Mus musculus]
mmu-miR-101c	NM_001145948	-22.4	0.9692	NA	NA	-	-	gi|225543132|ref|NP_001139420.1|	0	tetratricopeptide repeat protein 39A isoform 1 [Mus musculus]
mmu-miR-101c	NM_001029872	-21	0.999984	NA	NA	GO:0005887,GO:0004871,GO:0046983,GO:0031589,GO:0007166,GO:0042098,	K06594|1|0.0|2360|mmu:381924|integrin alpha D	gi|294997269|ref|NP_001025043.3|	0	integrin alpha-D precursor [Mus musculus]

mmu-miR-101c	NM_175749	-20.2	1	NA	NA	GO:0032991,GO:0031981,GO:0005635,GO:0017016,GO:0046914,GO:0044238,GO:0015758,GO:0033036,	K14296|1|0.0|2399|mmu:218210|nuclear pore complex protein Nup153	gi|148709082|gb|EDL41028.1|	0	nucleoporin 153, isoform CRA_a [Mus musculus]
mmu-miR-101c	NM_199159	-21	0.936186	NA	NA	GO:0031224,GO:0004930,GO:0050912,GO:0007166,	K08474|1|1e-176|619|mmu:387512|taste receptor type 2	gi|40255301|ref|NP_954610.1|	9.43E-176	taste receptor type 2 member 135 [Mus musculus]
mmu-miR-101c	NM_001033349	-20.7	0.997582	NA	NA	-	-	gi|85701850|ref|NP_001028521.1|	3.52E-109	protein ANKUB1 [Mus musculus]
mmu-miR-101c	NM_175467	-21	0.999661	NA	NA	GO:0031211,GO:0031224,GO:0048037,GO:0016454,GO:0030148,	K00654|1|0.0|1038|mmu:228677|serine palmitoyltransferase [EC:2.3.1.50]	gi|148696455|gb|EDL28402.1|	0	RIKEN cDNA C130053K05, isoform CRA_a [Mus musculus]

mmu-miR-101c	NM_030595	-22	1	NA	NA	GO:0044459,GO:0044431,GO:0019900,GO:0048193,GO:0006886,	K07295|1|2e-19|101|dpe:Dper_GL19043|nuclear receptor subfamily 2 group A!K10260|2|6e-08|62.8|api:100166211|F-box and WD-40 domain protein 7!K11887|3|1e-07|61.6|ame:409686|proteasomal ATPase-associated factor 1!K10342|4|9e-	gi|158854037|ref|NP_085098.1|	0	neurobeachin [Mus musculus]

mmu-miR-101c	NM_178795	-21.4	0.999995	NA	NA	GO:0044444,GO:0016791,GO:0000828,GO:0032559,GO:0019751,GO:0006796,	K13024|1|0.0|2731|mmu:327655|inositol hexakisphosphate/diphosphoinositol-pentakisphosphate kinase [EC:2.7.4.21 2.7.4.24]	gi|28972185|dbj|BAC65546.1|	0	mKIAA0377 protein [Mus musculus]
mmu-miR-101c	NM_028093	-26.3	0.40731	NA	NA	GO:0031224,GO:0016462,GO:0032559,GO:0009123,GO:0009132,	K01510|1|0.0|1014|mmu:72090|apyrase [EC:3.6.1.5]	gi|148676252|gb|EDL08199.1|	0	mCG20282, isoform CRA_a [Mus musculus]
mmu-miR-101c	NM_009493	-20	1	NA	NA	-	K04613|1|0.0|1638|mmu:22310|vomeronasal 2 receptor	gi|6678543|ref|NP_033519.1|	0	vomeronasal 2, receptor 42 precursor [Mus musculus]
mmu-miR-101c	NM_133917	-23.1	0.999297	NA	NA	GO:0005739,GO:0016020,GO:0005488,GO:0051169,GO:0006351,	K09113|1|0.0|1352|mmu:208104|MAX-like protein X	gi|188497661|ref|NP_598678.2|	0	MLX-interacting protein isoform 2 [Mus musculus]

mmu-miR-101c	NM_146829	-21.7	0.874596	NA	NA	GO:0004888,GO:0023052,	K04257|1|7e-166|583|mmu:258826|olfactory	gi|126215542|ref|NP_667040.2|	4.90E-165	olfactory receptor 27 [Mus musculus]
mmu-miR-101c	NM_019667	-26.7	0.567631	NA	NA	GO:0005769,GO:0005488,GO:0016192,GO:0007173,GO:0015031,	K04705|1|0.0|1014|mmu:56324|signal transducing adaptor	gi|9789975|ref|NP_062641.1|	0	signal transducing adapter molecule 2 [Mus musculus]
mmu-miR-101c	NM_027045	-22.7	0.999855	NA	NA	-	-	gi|80861474|ref|NP_081321.1|	0	protein FAM190B isoform 1 [Mus musculus]
mmu-miR-101c	NM_008787	-24.3	0.995633	NA	NA	GO:0005813,GO:0005515,GO:0016477,GO:0012501,GO:0007049,GO:0010927,GO:0043009,GO:0040007,GO:0021988,GO:0009887,GO:0000226,GO:0035107,GO:00082	K10388|1|1e-13|81.6|bta:786966|plectin!K10352|3|4e-13|79.7|ame:412092|myosin heavy chain	gi|93004085|ref|NP_032813.3|	0	pericentrin [Mus musculus]
mmu-miR-101c	NR_030721	-20	0.999998	NA	NA	-	-	gi|148698720|gb|EDL30667.1|	7.14E-36	mCG1049272, isoform CRA_a [Mus musculus]

mmu-miR-101c	NM_001035123	-20	0.999698	NA	NA	GO:0043231,GO:0008134,GO:0008276,GO:0043433,GO:0018022,GO:0031347,	K05302|1|0.0|833|mmu:66083|SET domain-containing protein 6	gi|78097104|ref|NP_001030295.1|	0	N-lysine methyltransferase SETD6 [Mus musculus]
mmu-miR-101c	NM_172585	-24	0.962341	NA	NA	GO:0003676,	K14000|1|2e-06|56.2|dre:553448|ribosome-binding protein 1	gi|50510417|dbj|BAD32194.1|	0	mKIAA0217 protein [Mus musculus]
mmu-miR-101c	NM_153129	-21.6	0.999888	NA	NA	GO:0005798,GO:0005488,GO:0043393,GO:0050688,GO:0008104,GO:0000301,	-	gi|54291704|ref|NP_694769.2|	0	phosphofurin acidic cluster sorting protein 1 [Mus musculus]
mmu-miR-101c	NM_001190313	-21	0.999992	NA	NA	GO:0031224,GO:0007275,GO:0048856,GO:0050953,	K09608|1|4e-37|158|dre:474335|tolloid-like protein 1 [EC:3.4.24.	gi|298286509|ref|NP_001177243.1|	0	membrane frizzled-related protein isoform 2 [Mus musculus]

mmu-miR-101c	NM_008224	-20.5	1	NA	NA	GO:0035097,GO:0000123,GO:0003712,GO:0001071,GO:0005515,GO:0006461,GO:0031647,GO:0006351,GO:0019042,GO:00073	K14966|1|0.0|2933|mmu:15161|host cell factor	gi|34328130|ref|NP_032250.2|	0	host cell factor 1 [Mus musculus]
mmu-miR-101c	NM_026891	-20.6	1	NA	NA	GO:0031224,GO:0005488,	-	gi|124430553|ref|NP_081167.2|	0	codanin-1 [Mus musculus]

mmu-miR-101c	NM_001081979	-21.4	1	NA	NA	GO:0032991,GO:0043231,GO:0000785,GO:0044444,GO:0003690,GO:0003712,GO:0001071,GO:0000166,GO:0005515,GO:0003682,GO:0003723,GO:0043565,GO:0000217,GO:0016571,GO:0046471,GO:0006357,GO:0048167,GO:0022037,GO:0051899,GO:0002209,GO:0031175,GO:00197	K11588|1|1e-161|573|mmu:17257|methyl CpG binding protein 2	gi|126517469|ref|NP_001075448.1|	7.14E-161	methyl-CpG-binding protein 2 isoform 1 [Mus musculus]
mmu-miR-101c	NM_011250	-20.1	1	NA	NA	GO:0044427,GO:0043234,GO:0031981,GO:0003676,GO:0006325,GO:0007049,GO:00192	K04681|1|0.0|1997|mmu:19651|retinoblastoma-like	gi|170932488|ref|NP_035380.3|	0	retinoblastoma-like protein 2 [Mus musculus]

mmu-miR-101c	NM_133190	-22.7	0.819292	NA	NA	GO:0030139,GO:0034704,GO:0005262,GO:0007267,GO:0032412,GO:0006816,	K04873|1|1e-163|577|mmu:81905|voltage-dependent calcium channel	gi|21687186|ref|NP_573453.1|	6.89E-163	voltage-dependent calcium channel gamma-8 subunit [Mus musculus]
mmu-miR-101c	NM_001252059	-20.3	1	NA	NA	GO:0043005,GO:0034704,GO:0043231,GO:0043025,GO:0005245,GO:0000149,GO:0003676,GO:0007172,GO:0007186,GO:0007270,GO:0050804,GO:0033555,GO:0031327,GO:0019318,GO:0014055,GO:0042391,GO:0050881,GO:0017156,GO:0040007,GO:0030320,GO:0008344,GO:00430	K04344|1|0.0|3792|mmu:12286|voltage-dependent calcium channel P/Q type alpha-1A	gi|355390372|ref|NP_001238988.1|	0	voltage-dependent P/Q-type calcium channel subunit alpha-1A isoform 2 [Mus musculus]

mmu-miR-101c	NM_030557	-20.1	1	NA	NA	GO:0043231,GO:0046914,GO:0003676,GO:0001071,GO:0006351,	K09228|1|2e-71|272|api:100159445|KRAB domain-containing zinc finger protein	gi|41056113|ref|NP_085034.2|	0	myoneurin [Mus musculus]
mmu-miR-101c	NM_008710	-20.2	1	NA	NA	GO:0031224,GO:0005743,GO:0000166,GO:0008746,GO:0006818,GO:0009060,	K00323|1|0.0|2011|mmu:18115|NAD(P) transhydrogenase [EC:1.6.1.2	gi|31543330|ref|NP_032736.2|	0	NAD(P) transhydrogenase, mitochondrial precursor [Mus musculus]

mmu-miR-101c	NM_010009	-20.7	0.999734	NA	NA	GO:0031966,GO:0005506,GO:0016709,GO:0030278,GO:0055065,GO:0003006,GO:0007589,GO:0044237,GO:0006694,GO:0008283,GO:0006775,GO:0048545,GO:0030858,GO:0010979,GO:0007050,GO:0034097,GO:0043434,GO:0009967,GO:0033273,GO:0010038,GO:0070838,GO:00327	K07438|1|0.0|940|mmu:13115|cytochrome P450, family 27, subfamily B (25-hydroxyvitamin D3 1alpha-hydroxylase) [EC:1.14.13.13]	gi|2443371|dbj|BAA22434.1|	0	25-hydroxyvitamin D3 1alpha-hydroxylase [Mus musculus]

mmu-miR-101c	NM_001112698	-22.4	0.770574	NA	NA	GO:0044431,GO:0044432,GO:0044421,GO:0004871,GO:0005123,GO:0050804,GO:0009628,GO:0050767,GO:0006952,GO:0014070,GO:0051726,GO:0009967,GO:0031346,GO:0031960,GO:0016486,GO:0007611,GO:0007600,GO:0001934,GO:0009605,GO:0007626,GO:0043066,GO:00303	K02582|1|6e-161|567|rno:310738|nerve growth factor, beta	gi|148675667|gb|EDL07614.1|	1.57E-166	nerve growth factor, beta, isoform CRA_a [Mus musculus]
mmu-miR-101c	NM_001134384	-21.9	0.999985	NA	NA	-	K12495|1|0.0|1498|hsa:9922|IQ motif and SEC7 domain-containing protein	gi|197304784|ref|NP_001127856.1|	0	IQ motif and SEC7 domain-containing protein 1 isoform b [Mus musculus]

mmu-miR-101c	NM_027782	-21.4	0.982256	NA	NA	GO:0005887,GO:0005267,GO:0034220,GO:0030001,	K15074|1|2e-09|64.7|hmg:100213269|BTB/POZ domain-containing adapter for CUL3-mediated RhoA degradation protein	gi|351710048|gb|EHB12967.1|	1.24E-132	BTB/POZ domain-containing protein KCTD6 [Heterocephalus glaber]
mmu-miR-101c	NM_001159496	-22.7	0.971462	NA	NA	GO:0043234,GO:0046914,GO:0004721,GO:0006464,	K04461|1|0.0|915|mmu:19043|protein phosphatase 1B (formerly 2C) [EC:3.1.3.16]	gi|148706646|gb|EDL38593.1|	0	mCG15599, isoform CRA_e [Mus musculus]

mmu-miR-101c	NM_001168679	-20.6	0.993009	NA	NA	GO:0031224,GO:0000323,GO:0060089,GO:0023052,GO:0007596,GO:0006464,	K06497|1|3e-27|123|nve:NEMVE_v1g236446|CD63 antigen!K06537|2|3e-26|120|nve:NEMVE_v1g233307|CD151 antigen!K06460|3|1e-25|118|aml:100480432|CD9 antigen	gi|22122475|ref|NP_666122.1|	6.30E-116	tetraspanin-8 [Mus musculus]
mmu-miR-101c	NM_001098271	-22.9	0.657996	NA	NA	GO:0031224,	-	gi|148298742|ref|NP_079602.4|	7.83E-128	transmembrane protein 176A [Mus musculus]
mmu-miR-101c	NR_027618	-22.6	0.997128	NA	NA	GO:0005770,GO:0005764,GO:0005774,GO:0005488,GO:0030695,GO:0019222,GO:00068	-	gi|30578384|ref|NP_849182.1|	0	vam6/Vps39-like protein isoform 2 [Mus musculus]

mmu-miR-101c	NM_011183	-20.7	0.999003	NA	NA	GO:0030427,GO:0005764,GO:0032991,GO:0012505,GO:0019866,GO:0044459,GO:0016021,GO:0005815,GO:0045202,GO:0030425,GO:0000775,GO:0031674,GO:0044297,GO:0070011,GO:0005488,GO:0050804,GO:0001932,GO:0044093,GO:0007420,GO:0007169,GO:0050851,GO:00708	K04522|1|0.0|768|mmu:19165|presenilin 2 [EC:3.4.23.-]	gi|190684661|ref|NP_035313.2|	0	presenilin-2 [Mus musculus]

mmu-miR-101c	NM_009009	-20.2	1	NA	NA	GO:0044427,GO:0031981,GO:0000793,GO:0005488,GO:0012501,GO:0006310,GO:0006281,GO:0006357,GO:0034502,GO:00070	K06670|1|0.0|1110|mmu:19357|cohesin complex subunit SCC1	gi|254692855|ref|NP_033035.3|	0	double-strand-break repair protein rad21 homolog [Mus musculus]
mmu-miR-101c	NM_001166282	-21.6	0.9988	NA	NA	-	K15032|1|6e-33|144|xtr:100145192|mTERF domain-containing protein, mitochondrial	gi|261399871|ref|NP_001159754.1|	0	coiled-coil domain-containing protein 33 isoform 1 [Mus musculus]

mmu-miR-101c	NM_145952	-20.2	1	NA	NA	GO:0044464,GO:0005099,GO:0032313,	K11837|1|7e-10|67.8|hsa:9098|ubiquitin carboxyl-terminal hydrolase 6/32 [EC:3.1.2.15]!K13136|2|1e-06|57.0|nvi:100114269|gem associated protein 8	gi|119943133|ref|NP_666064.3|	0	TBC1 domain family member 12 [Mus musculus]
mmu-miR-101c	NM_001177620	-21.1	0.999891	NA	NA	GO:0044424,GO:0009408,	-	gi|26334933|dbj|BAC31167.1|	5.26E-78	unnamed protein product [Mus musculus]
mmu-miR-101c	NM_001177615	-21.1	0.999904	NA	NA	GO:0044424,GO:0009408,	-	gi|26334933|dbj|BAC31167.1|	5.35E-78	unnamed protein product [Mus musculus]
mmu-miR-101c	NM_177899	-20	1	NA	NA	-	K09228|1|0.0|1138|mmu:330788|KRAB domain-containing zinc finger protein	gi|141801938|ref|NP_808567.2|	0	zinc finger protein 866 [Mus musculus]

mmu-miR-101c	NM_023712	-20.1	0.999994	NA	NA	GO:0019866,GO:0043231,GO:0031224,GO:0005488,GO:0010876,GO:0006810,	-	gi|170671720|ref|NP_076201.2|	0	protein spinster homolog 1 [Mus musculus]
mmu-miR-101c	NM_013827	-21.7	0.999827	NA	NA	GO:0043231,GO:0046914,GO:0003676,	K11485|1|0.0|1202|mmu:17765|polycomb-like protein 2	gi|70887769|ref|NP_038855.2|	0	metal-response element-binding transcription factor 2 isoform 1 [Mus musculus]
mmu-miR-101c	NM_019919	-21.8	0.999999	NA	NA	GO:0031012,GO:0046872,GO:0005515,GO:0007178,	K08023|1|0.0|3491|mmu:268977|latent transforming growth factor beta binding protein	gi|46249419|ref|NP_064303.2|	0	latent-transforming growth factor beta-binding protein 1 isoform LTBP-1L precursor [Mus musculus]
mmu-miR-101c	NR_040333	-21.5	0.996782	NA	NA	-	-	gi|148676034|gb|EDL07981.1|	1.30E-54	mCG141243 [Mus musculus]

mmu-miR-101c	NM_001039878	-20.6	0.999994	NA	NA	GO:0044424,GO:0008287,GO:0019903,GO:0019904,	K14963|1|2e-18|95.9|api:100162597|COMPASS component SWD3!K01062|5|6e-18|94.4|nve:NEMVE_v1g242515|1-alkyl-2-acetylglycerophosphocholine esterase [EC:3.1.1.47]	gi|89886486|ref|NP_001034967.1|	0	striatin-4 isoform 2 [Mus musculus]
mmu-miR-101c	NM_001008422	-21.6	0.999848	NA	NA	GO:0043231,GO:0005515,GO:0006351,GO:0006396,	-	gi|148690840|gb|EDL22787.1|	0	expressed sequence AI480556 [Mus musculus]

mmu-miR-101c	NM_001128169	-20.3	1	NA	NA	GO:0005874,GO:0044444,GO:0030529,GO:0009898,GO:0046914,GO:0005198,GO:0019783,GO:0016790,GO:0019900,GO:0000278,GO:0016579,GO:0001818,GO:0032886,GO:0043433,GO:0030178,GO:0042345,GO:00	K08601|1|0.0|1884|mmu:74256|ubiquitin thioesterase CYLD [EC:3.1.2.15]	gi|28972435|dbj|BAC65671.1|	0	mKIAA0849 protein [Mus musculus]
mmu-miR-101c	NM_080638	-22.7	0.973469	NA	NA	GO:0005635,GO:0032991,GO:0005488,GO:0050658,GO:0042221,	-	gi|239052674|ref|NP_542369.2|	0	major vault protein [Mus musculus]

mmu-miR-101c	NM_001113417	-20.6	1	NA	NA	GO:0031981,GO:0046914,GO:0003712,GO:0004879,GO:0001071,GO:0042802,GO:0043566,GO:0009653,GO:0030278,GO:0002062,GO:0045924,GO:0009755,GO:0008283,GO:0044057,GO:0008203,GO:0050954,GO:0007619,GO:0006641,	K08362|1|0.0|919|mmu:21834|thyroid hormone receptor beta	gi|164663877|ref|NP_001106888.1|	0	thyroid hormone receptor beta isoform 1 [Mus musculus]
mmu-miR-101c	NM_030678	-22.1	0.998002	NA	NA	GO:0044444,GO:0048029,GO:0035251,GO:0048513,GO:0005977,GO:0019318,	K00693|1|0.0|1324|mmu:14936|glycogen(starch) synthase [EC:2.4.1.11]	gi|31560022|ref|NP_109603.2|	0	glycogen [starch] synthase, muscle [Mus musculus]

mmu-miR-101c	NM_020256	-20.8	1	NA	NA	GO:0031981,GO:0016020,GO:0003676,GO:0046914,GO:0006351,GO:0007166,	K10507|1|0.0|1233|mmu:56805|zinc finger and BTB domain-containing protein 33	gi|148697061|gb|EDL29008.1|	0	zinc finger and BTB domain containing 33, isoform CRA_b [Mus musculus]
mmu-miR-101c	NM_001165949	-20	0.999999	NA	NA	GO:0043005,GO:0016604,GO:0046914,GO:0005515,GO:0016247,GO:0019787,GO:0016925,GO:0009719,GO:0042391,GO:00104	K16064|1|0.0|1130|mmu:229615|E3 SUMO-protein ligase PIAS3 [EC:6.3.2.-]	gi|148706965|gb|EDL38912.1|	0	protein inhibitor of activated STAT 3, isoform CRA_b [Mus musculus]

mmu-miR-101c	NM_175474	-21.6	0.994029	NA	NA	GO:0031982,GO:0005768,	K04349|1|6e-11|70.5|nvi:100117952|Ras-specific guanine nucleotide-releasing factor 1!K12326|3|9e-09|63.2|aga:AgaP_AGAP001238|Ras-specific guanine nucleotide-releasing factor 2	gi|30425218|ref|NP_780683.1|	9.97E-118	sesquipedalian-1 [Mus musculus]
mmu-miR-101c	NM_144925	-22.1	0.999997	NA	NA	GO:0030529,GO:0003676,GO:0006412,GO:0031669,GO:0035195,	K03165|1|6e-06|55.8|dvi:Dvir_GJ18138|DNA topoisomerase III [EC:5.99.1.2]	gi|117190552|ref|NP_659174.3|	0	trinucleotide repeat-containing gene 6A protein [Mus musculus]
mmu-miR-101c	NM_001126490	-21.1	0.999692	NA	NA	-	K04659|1|1e-06|56.2|cfa:487486|thrombospondin	gi|187608602|ref|NP_001119962.1|	0	isthmin-1 precursor [Mus musculus]

mmu-miR-101c	NM_001081337	-20.3	1	NA	NA	GO:0044464,GO:0008047,GO:0009966,	K08013|1|0.0|758|acs:100553026|signal-induced proliferation-associated gene 1	gi|124487493|ref|NP_001074806.1|	0	signal-induced proliferation-associated 1-like protein 2 [Mus musculus]
mmu-miR-103-1-5p	NM_001004155	-27.6	0.455643	-48.25	320	-	-	gi|21618991|gb|AAH32212.1|	0	9930012K11Rik protein, partial [Mus musculus]
mmu-miR-103-1-5p	NM_001004468	-29.6	0.716769	-71.11	461	GO:0043232,GO:0051427,GO:0021846,GO:0021543,GO:0007051,	K14282|1|0.0|5333|mmu:57752|transforming acidic coiled-coil-containing protein 2	gi|52486843|ref|NP_001004468.1|	0	transforming acidic coiled-coil-containing protein 2 isoform c [Mus musculus]
mmu-miR-103-1-5p	NM_001029933	-29.2	0.28983	-25.92	156	GO:0005622,GO:0043169,GO:0003676,GO:0006351,	K09228|1|0.0|1221|mmu:232966|KRAB domain-containing zinc finger protein	gi|113930749|ref|NP_001025104.2|	0	zinc finger protein 114 [Mus musculus]
mmu-miR-103-1-5p	NM_001033213	-26.3	0.848581	-45.65	319	-	-	gi|148686951|gb|EDL18898.1|	0	mCG145297 [Mus musculus]

mmu-miR-103-1-5p	NM_001033399	-32.2	0.283665	-26.84	158	GO:0003824,GO:0008152,	K00078|1|3e-09|66.6|dpe:Dper_GL19398|dihydrodiol dehydrogenase / D-xylose 1-dehydrogenase (NADP) [EC:1.3.1.20	gi|85701893|ref|NP_001028571.1|	0	glucose-fructose oxidoreductase domain-containing protein 1 precursor [Mus musculus]
mmu-miR-103-1-5p	NM_001033668	-26.8	0.992861	-46.57	321	-	K10408|1|0.0|8267|mmu:110084|dynein heavy chain, axonemal	gi|256773234|ref|NP_001028840.1|	0	dynein, axonemal, heavy chain 1 [Mus musculus]
mmu-miR-103-1-5p	NM_001038643	-28.5	0.457264	-46.57	319	GO:0016323,GO:0016021,GO:0015197,GO:0015291,GO:0008509,GO:0015833,GO:0015711,GO:0071715,	K14353|1|0.0|1216|mmu:108116|solute carrier organic anion transporter family, member 3A	gi|84579878|ref|NP_001033732.1|	0	solute carrier organic anion transporter family member 3A1 isoform 2 [Mus musculus]

mmu-miR-103-1-5p	NM_001039545	-29.7	0.56031	-105.53	759	GO:0031981,GO:0005924,GO:0016460,GO:0030017,GO:0008092,GO:0003774,GO:0005198,GO:0032559,GO:0050896,GO:0007009,GO:0031033,GO:0006936,	K10352|1|0.0|3358|mmu:17882|myosin heavy chain	gi|205830428|ref|NP_001034634.2|	0	myosin heavy chain IIa [Mus musculus]
mmu-miR-103-1-5p	NM_001039700	-28.6	0.748269	-47.15	327	GO:0031224,GO:0046872,GO:0015075,GO:0030246,GO:0006810,GO:0007186,	K04989|1|0.0|3511|mmu:244646|polycystin 1L3	gi|115583681|ref|NP_001034789.2|	0	polycystic kidney disease protein 1-like 3 isoform a precursor [Mus musculus]

mmu-miR-103-1-5p	NM_001042699	-26.3	0.944266	-43.85	324	GO:0031965,GO:0034992,GO:0031224,GO:0008092,GO:0007010,	K10352|1|8e-07|58.2|xtr:448762|myosin heavy chain!K10366|2|1e-06|57.8|bta:537655|dystrophin!K10362|3|1e-06|57.4|bta:540437|myosin XVIII	gi|111607453|ref|NP_001036164.1|	0	nesprin-3 isoform alpha [Mus musculus]
mmu-miR-103-1-5p	NM_001044383	-25.1	0.980462	-45.04	312	GO:0031981,GO:0003676,GO:0006356,GO:0006352,GO:0006342,GO:0006353,GO:00063	K09273|1|0.0|1189|rno:25574|upstream-binding transcription factor	gi|189095264|ref|NP_001099193.2|	0	nucleolar transcription factor 1 isoform 1 [Rattus norvegicus]
mmu-miR-103-1-5p	NM_001076554	-32.7	0.284692	-46.46	333	GO:0032991,GO:0030864,GO:0005913,GO:0031674,GO:0043227,GO:0008092,GO:0046872,GO:0000149,GO:0046983,GO:00308	K06114|1|0.0|4771|mmu:20740|spectrin alpha	gi|115496850|ref|NP_001070022.1|	0	spectrin alpha chain, brain isoform 1 [Mus musculus]

mmu-miR-103-1-5p	NM_001080385	-29.6	0.1227	-26.83	160	GO:0012510,GO:0030118,GO:0005905,GO:0005488,GO:0006935,GO:0048193,GO:0015031,GO:0007173,	K04644|1|4e-113|408|rno:83800|clathrin light chain A	gi|122939194|ref|NP_001073854.1|	2.40E-113	clathrin light chain A isoform b [Mus musculus]
mmu-miR-103-1-5p	NM_001081108	-27.2	0.241426	-29.19	167	-	-	gi|148687256|gb|EDL19203.1|	4.75E-62	mCG7441, isoform CRA_a [Mus musculus]
mmu-miR-103-1-5p	NM_001081128	-27.9	0.715437	-55.15	334	GO:0044444,GO:0019842,GO:0008172,GO:0046914,GO:0016597,GO:0048731,GO:0000097,GO:0006730,GO:0008213,GO:00067	K00548|1|0.0|2512|mmu:238505|5-methyltetrahydrofolate--homocysteine methyltransferase [EC:2.1.1.13]	gi|124487479|ref|NP_001074597.1|	0	methionine synthase [Mus musculus]
mmu-miR-103-1-5p	NM_001081246	-27.9	0.354078	-28.4	174	-	-	gi|348605262|ref|NP_001019523.2|	0	uncharacterized protein C12orf50 homolog [Rattus norvegicus]
mmu-miR-103-1-5p	NM_001081341	-27.9	0.769524	-44.96	318	GO:0043231,GO:0046914,	-	gi|124486797|ref|NP_001074810.1|	0	S phase cyclin A-associated protein in the endoplasmic reticulum [Mus musculus]

mmu-miR-103-1-5p	NM_001081402	-35.1	0.024429	-31.13	176	-	K02208|1|4e-98|359|bmy:Bm1_33825|cyclin-dependent kinase 8/11 [EC:2.7.11.22 2.7.11.23]!K03130|2|2e-10|68.9|bfo:BRAFLDRAFT_65485|transcription initiation factor TFIID subunit 5!K12857|4|8e-10|66.6|dre:393292|Prp8 binding	gi|124487149|ref|NP_001074871.1|	0	WD repeat-containing protein 70 [Mus musculus]
mmu-miR-103-1-5p	NM_001082483	-25.4	0.992311	-50.9	311	-	-	gi|126723102|ref|NP_001075952.1|	0	protein EFR3 homolog B [Mus musculus]

mmu-miR-103-1-5p	NM_001083959	-30.7	0.11319	-26.05	164	GO:0043231,GO:0043232,GO:0003676,GO:0003824,GO:0032559,GO:0022602,GO:0006310,GO:0033365,GO:0048232,GO:0007281,GO:0007128,	K10878|1|0.0|741|mmu:26972|meiotic recombination protein SPO11	gi|145312272|ref|NP_001077428.1|	0	meiotic recombination protein SPO11 isoform c [Mus musculus]
mmu-miR-103-1-5p	NM_001083960	-30.7	0.109878	-26.05	164	GO:0043231,GO:0043232,GO:0003676,GO:0003824,GO:0032559,GO:0022602,GO:0006310,GO:0033365,GO:0048232,GO:0007281,GO:0007128,	K10878|1|0.0|706|mmu:26972|meiotic recombination protein SPO11	gi|145312248|ref|NP_001077429.1|	0	meiotic recombination protein SPO11 isoform b [Mus musculus]

mmu-miR-103-1-5p	NM_001110202	-28.5	0.623356	-63.09	468	GO:0043005,GO:0043232,GO:0030136,GO:0044459,GO:0046914,GO:0042802,GO:0019787,GO:0006511,GO:0031333,GO:0032446,GO:0017158,	K10649|1|0.0|1402|mmu:94090|tripartite motif-containing protein 9/67	gi|37359882|dbj|BAC97919.1|	0	mKIAA0282 protein [Mus musculus]
mmu-miR-103-1-5p	NM_001112735	-27.6	0.455082	-48.25	320	-	-	gi|21618991|gb|AAH32212.1|	0	9930012K11Rik protein, partial [Mus musculus]

mmu-miR-103-1-5p	NM_001113364	-25.9	0.918023	-41.34	312	GO:0044464,GO:0005099,GO:0032313,	K11837|1|4e-11|72.0|hsa:9098|ubiquitin carboxyl-terminal hydrolase 6/32 [EC:3.1.2.15]!K08187|2|8e-08|60.8|tgu:100221433|MFS transporter, MCP family, solute carrier family 16 (monocarboxylic acid transporters), member 10!K13136|3|7e-	gi|164565383|ref|NP_001106835.1|	0	TBC1 domain family member 14 isoform c [Mus musculus]
mmu-miR-103-1-5p	NM_001113560	-29.2	0.10385	-25.66	164	GO:0044424,GO:0043169,GO:0016846,GO:0006790,GO:0006081,GO:0043066,GO:00442	K01759|1|5e-106|384|mmu:109801|lactoylglutathione lyase [EC:4.4.1.5]	gi|165932331|ref|NP_079650.3|	3.58E-105	lactoylglutathione lyase [Mus musculus]

mmu-miR-103-1-5p	NM_001114333	-31.8	0.33815	-27.11	165	GO:0043231,GO:0044430,GO:0044297,GO:0016021,GO:0030425,GO:0035258,GO:0008066,GO:0007200,GO:0051602,GO:0007204,GO:0010627,GO:0007610,GO:0051931,GO:0050804,GO:0042391,GO:0007270,GO:00	K04604|1|0.0|1790|mmu:14816|metabotropic glutamate receptor 1/5	gi|166999785|ref|NP_001107805.1|	0	metabotropic glutamate receptor 1 isoform beta precursor [Mus musculus]
mmu-miR-103-1-5p	NM_001126324	-26.5	0.707906	-45.19	308	-	-	gi|187280849|ref|NP_001119796.1|	0	uncharacterized protein LOC279185 [Mus musculus]
mmu-miR-103-1-5p	NM_001128606	-25.4	0.980774	-27.53	175	GO:0005856,GO:0043231,GO:0044444,GO:0044425,GO:0032991,GO:0035091,GO:0008092,GO:0043393,GO:0065008,GO:00300	K06107|1|0.0|1365|mmu:269587|erythrocyte membrane protein band 4.1	gi|60360122|dbj|BAD90280.1|	0	mKIAA4056 protein [Mus musculus]

mmu-miR-103-1-5p	NM_001128607	-25.4	0.979785	-27.53	175	GO:0005856,GO:0043231,GO:0044444,GO:0044425,GO:0032991,GO:0035091,GO:0008092,GO:0043393,GO:0065008,GO:00300	K06107|1|0.0|1359|mmu:269587|erythrocyte membrane protein band 4.1	gi|45768818|gb|AAH68138.1|	0	Epb4.1 protein, partial [Mus musculus]
mmu-miR-103-1-5p	NM_001130515	-26.9	0.541515	-28.72	164	GO:0031981,GO:0003723,GO:0016538,GO:0006357,GO:0000079,	K15189|1|6e-171|601|mmu:71059|protein HEXIM1/2	gi|110625829|ref|NP_081934.1|	3.94E-170	protein HEXIM2 [Mus musculus]
mmu-miR-103-1-5p	NM_001130516	-26.9	0.545067	-28.72	164	GO:0031981,GO:0003723,GO:0016538,GO:0006357,GO:0000079,	K15189|1|6e-171|601|mmu:71059|protein HEXIM1/2	gi|110625829|ref|NP_081934.1|	3.98E-170	protein HEXIM2 [Mus musculus]
mmu-miR-103-1-5p	NM_001136556	-27.5	0.8625	-45.61	319	GO:0031224,	K15621|1|1e-06|57.4|bta:506146|BAI1-associated protein 3	gi|211971048|ref|NP_001130028.1|	0	fer-1-like protein 4 [Mus musculus]

mmu-miR-103-1-5p	NM_001145100	-29.3	0.421457	-28.92	163	-	K10443|1|5e-11|71.2|tgu:100226815|kelch-like protein 2/3!K10453|4|2e-10|69.3|acs:100566431|kelch-like protein 16 (gigaxonin)!K10494|5|3e-10|68.9|dre:565670|zinc finger and BTB domain-containin	gi|223029434|ref|NP_001138572.1|	0	uncharacterized protein LOC100270744 [Mus musculus]
mmu-miR-103-1-5p	NM_001145351	-27.6	0.432305	-44.87	310	-	-	gi|223890238|ref|NP_001138823.1|	1.15E-121	uncharacterized protein LOC320095 isoform 1 precursor [Mus musculus]

mmu-miR-103-1-5p	NM_001146708	-31.8	0.01417	-25.62	160	GO:0009986,GO:0031224,GO:0031264,GO:0044444,GO:0005035,GO:0019899,GO:0002449,GO:0043029,GO:0048511,GO:0009612,GO:0010939,GO:0008283,GO:0031102,GO:0019221,GO:0050864,GO:0051259,GO:0002440,GO:0045595,GO:0048232,GO:0031960,GO:0043170,GO:00022	K04390|1|4e-32|137|mmu:14102|tumor necrosis factor receptor superfamily member 6	gi|226443053|ref|NP_001140180.1|	2.74E-31	tumor necrosis factor receptor superfamily member 6 isoform 2 precursor [Mus musculus]
mmu-miR-103-1-5p	NM_001159635	-31.1	0.155772	-26.71	168	GO:0003676,	-	gi|227500539|ref|NP_001153107.1|	2.83E-86	probable RNA-binding protein 18 isoform 2 [Mus musculus]

mmu-miR-103-1-5p	NM_001161782	-28.6	0.483645	-25.84	167	GO:0043231,GO:0005859,GO:0008092,GO:0003712,GO:0046914,GO:0019787,GO:0032182,GO:0009896,GO:0016567,GO:00301	K10607|1|0.0|1239|mmu:69807|tripartite motif-containing protein 32 [EC:6.3.2.19]	gi|148699134|gb|EDL31081.1|	0	tripartite motif protein 32, isoform CRA_a [Mus musculus]
mmu-miR-103-1-5p	NM_001162533	-27	0.590298	-47.52	336	-	K12470|1|1e-27|125|gga:418608|SH3 domain-containing kinase-binding protein 1!K13738|2|1e-26|122|ssc:100627990|CD2-associated protein	gi|148698353|gb|EDL30300.1|	0	RIKEN cDNA 1700029G01, isoform CRA_b [Mus musculus]
mmu-miR-103-1-5p	NM_001162905	-30	0.291317	-28.56	172	GO:0048856,	K03995|1|1e-29|133|bfo:BRAFLDRAFT_76018|complement component 6	gi|251823820|ref|NP_001156377.1|	0	nuclear protein MDM1 isoform 4 [Mus musculus]

mmu-miR-103-1-5p	NM_001163016	-29.9	0.36866	-25.59	162	GO:0043231,GO:0005102,	K04574|1|1e-06|56.6|rno:24587|neurofilament heavy polypeptide!K15176|2|6e-06|54.7|dpe:Dper_GL16176|RNA polymerase-associated protein CTR9!K13114|3|6e-06|54.7|oaa:100083077|pinin	gi|244793066|ref|NP_001156487.1|	0	G-protein coupled receptor-associated sorting protein 2 [Mus musculus]

mmu-miR-103-1-5p	NM_001163017	-29.9	0.367397	-25.59	162	GO:0043231,GO:0005102,	K04574|1|1e-06|56.6|rno:24587|neurofilament heavy polypeptide!K15176|2|6e-06|54.7|dpe:Dper_GL16176|RNA polymerase-associated protein CTR9!K13114|3|6e-06|54.7|oaa:100083077|pinin	gi|244793066|ref|NP_001156487.1|	0	G-protein coupled receptor-associated sorting protein 2 [Mus musculus]
mmu-miR-103-1-5p	NM_001163271	-31	0.238663	-52.68	325	GO:0044446,GO:0042995,GO:0046872,GO:0006468,	-	gi|253795534|ref|NP_001156743.1|	0	fibrous sheath CABYR-binding protein [Mus musculus]
mmu-miR-103-1-5p	NM_001163379	-30	0.209805	-26.53	166	GO:0043231,GO:0031224,GO:0016411,GO:0006644,GO:0006641,GO:0046394,GO:0001816,	K13509|1|5e-155|548|mmu:55979|lysophosphatidate acyltransferase [EC:2.3.1.5	gi|26352009|dbj|BAC39641.1|	4.26E-164	unnamed protein product [Mus musculus]

mmu-miR-103-1-5p	NM_001163505	-31.6	0.337573	-28.06	173	-	-	gi|254826716|ref|NP_001156977.1|	0	atlastin-3 isoform 1 [Mus musculus]
mmu-miR-103-1-5p	NM_001163540	-26.3	0.998959	-70.24	473	-	K10388|1|0.0|3881|mmu:18810|plectin	gi|256418964|ref|NP_958788.2|	0	plectin isoform 1e [Mus musculus]
mmu-miR-103-1-5p	NM_001163750	-27.4	0.475414	-27.7	172	-	K10345|1|0.0|848|mmu:79043|SPRY domain-containing SOCS box protein 3	gi|254939631|ref|NP_001157222.1|	0	SPRY domain-containing SOCS box protein 3 isoform 1 [Mus musculus]
mmu-miR-103-1-5p	NM_001163751	-27.4	0.403035	-27.7	172	-	K10345|1|3e-175|615|mmu:79043|SPRY domain-containing SOCS box protein 3	gi|254939633|ref|NP_001157223.1|	0	SPRY domain-containing SOCS box protein 3 isoform 2 [Mus musculus]

mmu-miR-103-1-5p	NM_001164041	-26.7	0.953982	-47.38	320	GO:0031981,GO:0031224,GO:0043234,GO:0044444,GO:0001071,GO:0005072,GO:0019899,GO:0006941,GO:0001649,GO:0007178,GO:0006357,GO:0045597,GO:0003006,GO:0014070,GO:0009790,GO:0030099,GO:0006464,GO:0035295,	K04676|1|0.0|779|mmu:17129|mothers against decapentaplegic homolog 1/5/8	gi|1763543|gb|AAB39737.1|	0	Smad5 [Mus musculus]
mmu-miR-103-1-5p	NM_001164284	-30.6	0.209765	-26.66	163	-	-	gi|256017232|ref|NP_001157756.1|	0	uncharacterized protein LOC381654 [Mus musculus]
mmu-miR-103-1-5p	NM_001164285	-30.6	0.197741	-26.66	163	-	-	gi|256017232|ref|NP_001157756.1|	0	uncharacterized protein LOC381654 [Mus musculus]

mmu-miR-103-1-5p	NM_001164786	-33.7	0.03947	-25.38	163	GO:0031012,GO:0046914,GO:0004175,GO:0019538,	K09609|1|0.0|1012|mmu:223838|a disintegrin and metalloproteinase with thrombospondin motifs 20 [EC:3.4.24.-]	gi|259155423|ref|NP_001158258.1|	0	A disintegrin and metalloproteinase with thrombospondin motifs 20 isoform 3 precursor [Mus musculus]
mmu-miR-103-1-5p	NM_001165919	-30.5	0.120583	-25.89	162	-	-	gi|260436977|ref|NP_001159391.1|	4.86E-126	uncharacterized protein C11orf85 homolog [Mus musculus]
mmu-miR-103-1-5p	NM_001165948	-27.6	0.902938	-31.29	171	-	K10500|1|4e-15|86.3|rno:313666|zinc finger and BTB domain-containing protein	gi|260099686|ref|NP_001159420.1|	0	pogo transposable element with ZNF domain isoform 2 [Mus musculus]

mmu-miR-103-1-5p	NM_001166024	-25.2	0.947857	-25.06	168	GO:0031981,GO:0012505,GO:0016020,GO:0005083,GO:0008047,GO:0046914,GO:0006810,GO:0043087,GO:0032012,	K12493|1|0.0|931|mmu:77038|ADP-ribosylation factor GTPase-activating protein 2/3	gi|260763915|ref|NP_001159496.1|	0	ADP-ribosylation factor GTPase-activating protein 2 isoform 1 [Mus musculus]
mmu-miR-103-1-5p	NM_001166556	-25.3	0.713608	-26.65	167	GO:0031224,GO:0017111,GO:0032559,GO:0051234,GO:0009207,	K05649|1|0.0|731|mmu:76184|ATP-binding cassette, subfamily A (ABC1), member 6	gi|262118234|ref|NP_001160028.1|	0	ATP-binding cassette sub-family A member 6 isoform 2 [Mus musculus]
mmu-miR-103-1-5p	NM_001166557	-25.3	0.691682	-26.65	167	GO:0031224,GO:0017111,GO:0032559,GO:0051234,GO:0009207,	K05649|1|1e-165|583|mmu:76184|ATP-binding cassette, subfamily A (ABC1), member 6	gi|262118236|ref|NP_001160029.1|	5.97E-173	ATP-binding cassette sub-family A member 6 isoform 3 [Mus musculus]

mmu-miR-103-1-5p	NM_001168509	-25.4	0.792689	-43.61	337	GO:0016021,GO:0005488,GO:0004888,GO:0007166,	K04328|1|8e-179|627|mmu:233079|free fatty acid receptor 2	gi|22122727|ref|NP_666299.1|	5.36E-178	free fatty acid receptor 2 [Mus musculus]
mmu-miR-103-1-5p	NM_001172064	-28.3	0.44478	-25.77	161	-	-	gi|60359886|dbj|BAD90162.1|	0	mFLJ00101 protein [Mus musculus]
mmu-miR-103-1-5p	NM_001172065	-28.3	0.424944	-25.77	161	-	-	gi|285026461|ref|NP_001165536.1|	0	leucine-rich repeat-containing protein 56 isoform b [Mus musculus]
mmu-miR-103-1-5p	NM_001177667	-32.7	0.284598	-46.46	333	GO:0032991,GO:0005913,GO:0043227,GO:0031674,GO:0030864,GO:0005198,GO:0000149,GO:0046983,GO:0008092,GO:0046872,GO:0030835,GO:0006915,GO:00069	K06114|1|0.0|4729|rno:64159|spectrin alpha	gi|295054266|ref|NP_001171138.1|	0	spectrin alpha chain, brain isoform 2 [Mus musculus]

mmu-miR-103-1-5p	NM_001177668	-32.7	0.282714	-46.46	333	GO:0032991,GO:0030864,GO:0005913,GO:0031674,GO:0043227,GO:0008092,GO:0046872,GO:0000149,GO:0046983,GO:00308	K06114|1|0.0|4716|mmu:20740|spectrin alpha	gi|295054271|ref|NP_001171139.1|	0	spectrin alpha chain, brain isoform 3 [Mus musculus]

mmu-miR-103-1-5p	NM_001177845	-33.4	0.131638	-30.32	175	GO:0016023,GO:0044425,GO:0001726,GO:0005080,GO:0035091,GO:0004872,GO:0042802,GO:0035325,GO:0046983,GO:0042108,GO:0030097,GO:0070339,GO:0032092,GO:0034142,GO:0051091,GO:0032479,GO:0019221,GO:0042742,GO:0070391,GO:0002690,GO:0032722,GO:00308	K05403|1|8e-103|376|mmu:117149|toll-interleukin 1 receptor (TIR) domain-containing adaptor protein	gi|148693432|gb|EDL25379.1|	1.21E-115	toll-interleukin 1 receptor (TIR) domain-containing adaptor protein, isoform CRA_b [Mus musculus]

mmu-miR-103-1-5p	NM_001177846	-33.4	0.131093	-30.32	175	GO:0016023,GO:0044425,GO:0001726,GO:0005080,GO:0035091,GO:0004872,GO:0042802,GO:0035325,GO:0046983,GO:0042108,GO:0030097,GO:0070339,GO:0032092,GO:0034142,GO:0051091,GO:0032479,GO:0019221,GO:0042742,GO:0070391,GO:0002690,GO:0032722,GO:00308	K05403|1|8e-103|376|mmu:117149|toll-interleukin 1 receptor (TIR) domain-containing adaptor protein	gi|148693432|gb|EDL25379.1|	1.20E-115	toll-interleukin 1 receptor (TIR) domain-containing adaptor protein, isoform CRA_b [Mus musculus]

mmu-miR-103-1-5p	NM_001177847	-33.4	0.129454	-30.32	175	GO:0016023,GO:0044425,GO:0001726,GO:0005080,GO:0035091,GO:0004872,GO:0042802,GO:0035325,GO:0046983,GO:0042108,GO:0030097,GO:0070339,GO:0032092,GO:0034142,GO:0051091,GO:0032479,GO:0019221,GO:0042742,GO:0070391,GO:0002690,GO:0032722,GO:00308	K05403|1|8e-103|376|mmu:117149|toll-interleukin 1 receptor (TIR) domain-containing adaptor protein	gi|148693432|gb|EDL25379.1|	1.19E-115	toll-interleukin 1 receptor (TIR) domain-containing adaptor protein, isoform CRA_b [Mus musculus]

mmu-miR-103-1-5p	NM_001190733	-27.6	0.722103	-47.02	322	-	K10624|1|4e-15|85.1|ame:724670|E3 ubiquitin-protein ligase RBBP6 [EC:6.3.2.19]	gi|300192986|ref|NP_001177662.1|	4.53E-140	DCN1-like protein 4 isoform C [Mus musculus]
mmu-miR-103-1-5p	NM_001195544	-27.6	0.204739	-26.86	149	GO:0031224,	K06839|1|7e-13|75.1|tca:660980|slit 2!K06838|3|3e-12|73.2|ptr:100609860|slit 1	gi|306966158|ref|NP_001182473.1|	4.81E-123	leucine-rich repeat-containing protein 3C precursor [Mus musculus]
mmu-miR-103-1-5p	NM_001199136	-25.9	0.999979	-46.41	322	GO:0005856,GO:0044444,GO:0046872,GO:0015631,GO:0006612,GO:0007166,GO:00017	K10382|1|0.0|3826|mdo:100018543|dystonin	gi|312433955|ref|NP_001186065.1|	0	microtubule-actin cross-linking factor 1 isoform 1 [Mus musculus]

mmu-miR-103-1-5p	NM_001199141	-26.3	0.893579	-46.33	310	GO:0031981,GO:0046914,GO:0003676,GO:0051704,GO:0010467,GO:0009987,	K08881|1|7e-10|67.8|dre:442869|tripartite motif-containing protein 24!K08883|2|1e-06|57.4|isc:IscW_ISCW017611|tripartite motif-containing protein 33 [EC:6.3.2.19]	gi|148700349|gb|EDL32296.1|	0	zinc finger, MYND domain containing 11, isoform CRA_e [Mus musculus]
mmu-miR-103-1-5p	NM_001205057	-30.4	0.098316	-25.81	165	-	K09228|1|3e-31|136|mmu:665211|KRAB domain-containing zinc finger protein	gi|377834938|ref|XP_003688827.1|	1.45E-37	PREDICTED: zinc finger protein 709 [Mus musculus]

mmu-miR-103-1-5p	NM_001205173	-28.7	0.685285	-28.53	156	-	K00294|1|0.0|657|mcc:714870|1-pyrroline-5-carboxylate dehydrogenase [EC:1.5.1.12]!K07606|4|4e-16|89.0|bta:280955|vimentin	gi|350585698|ref|XP_003482029.1|	0	PREDICTED: intermediate filament family orphan 2-like [Sus scrofa]
mmu-miR-103-1-5p	NM_001205312	-34.1	0.103649	-51.86	318	GO:0043231,GO:0043232,GO:0044444,GO:0005126,GO:0004713,GO:0032559,GO:0035556,GO:0001959,GO:0006464,	K11219|1|0.0|2435|mmu:54721|non-receptor tyrosine kinase [EC:2.7.10.2]	gi|328887920|ref|NP_001192241.1|	0	non-receptor tyrosine-protein kinase TYK2 isoform 1 [Mus musculus]
mmu-miR-103-1-5p	NM_001242419	-31.9	0.244943	-50.91	326	GO:0031224,	-	gi|37359864|dbj|BAC97910.1|	2.58E-172	mKIAA0247 protein [Mus musculus]

mmu-miR-103-1-5p	NM_001253904	-26.2	0.989041	-42.27	302	GO:0043234,GO:0044451,GO:0003712,GO:0019899,GO:0005102,GO:0006629,GO:0000122,	K06065|1|0.0|3803|mmu:20602|nuclear receptor co-repressor 2	gi|341942242|sp|Q9WU42.3|NCOR2_MOUSE	0	RecName: Full=Nuclear receptor corepressor 2; Short=N-CoR2; AltName: Full=Silencing mediator of retinoic acid and thyroid hormone receptor; Short=SMRT; Short=SMRTe; AltName: Full=T3 receptor-associating factor; Short=TRAC; AltName: Full=Thyroid-, retinoic-acid-receptor-associated corepressor
mmu-miR-103-1-5p	NM_001271386	-25.2	0.998235	-26.59	176	-	-	-	-	-
mmu-miR-103-1-5p	NM_001271726	-25.3	0.995696	-25.17	168	-	-	-	-	-
mmu-miR-103-1-5p	NM_001271727	-25.3	0.996366	-25.17	168	-	-	-	-	-
mmu-miR-103-1-5p	NM_001271728	-25.3	0.995874	-25.17	168	-	-	-	-	-
mmu-miR-103-1-5p	NM_007439	-27.2	0.886221	-25.65	162	GO:0016021,GO:0004871,GO:0004713,GO:0032559,GO:0006468,GO:0007167,	K05119|1|0.0|2853|mmu:11682|anaplastic lymphoma kinase [EC:2.7.10.1]	gi|110347475|ref|NP_031465.2|	0	ALK tyrosine kinase receptor precursor [Mus musculus]

mmu-miR-103-1-5p	NM_007769	-32.6	0.233854	-26.46	162	GO:0030666,GO:0031224,GO:0030667,GO:0031012,GO:0051635,GO:0019899,GO:0004888,GO:0006810,GO:0019731,GO:0048545,GO:0002758,GO:0001832,GO:00308	K13912|1|0.0|3868|mmu:12945|deleted in malignant brain tumors 1 protein	gi|148685745|gb|EDL17692.1|	0	deleted in malignant brain tumors 1, isoform CRA_c [Mus musculus]
mmu-miR-103-1-5p	NM_008104	-28	0.533557	-25.22	154	GO:0043231,GO:0043169,GO:0001071,GO:0003677,GO:0048869,GO:0072502,GO:0006915,GO:0048732,GO:0006875,GO:00063	-	gi|113680509|ref|NP_032130.2|	0	chorion-specific transcription factor GCMb [Mus musculus]
mmu-miR-103-1-5p	NM_008191	-27.7	0.117421	-25.79	160	GO:0030250,GO:0006164,GO:0046903,GO:0019222,GO:0048878,GO:0008217,GO:00091	-	gi|2058647|gb|AAB53314.1|	6.49E-43	uroguanylin [Mus musculus]

mmu-miR-103-1-5p	NM_008233	-28.5	0.373731	-30.01	173	-	K03243|1|1e-07|59.3|oaa:100075714|translation initiation factor 5B!K09880|2|8e-07|56.6|nvi:100117336|enolase-phosphatase E1 [EC:3.1.3.77]!K15076|3|2e-06|55.1|oaa:100093482|transcription elongation factor B, polypeptide 3!K14721|4|5e-	gi|6680201|ref|NP_032259.1|	0	hepatoma-derived growth factor-related protein 2 [Mus musculus]

mmu-miR-103-1-5p	NM_008852	-29.8	0.133886	-25.68	155	GO:0043231,GO:0003677,GO:0006355,GO:0007420,GO:0048468,GO:0009653,GO:0007610,GO:0002088,GO:0030182,	K09357|1|2e-125|447|ssc:100154216|paired-like homeodomain transcription factor 3	gi|148710035|gb|EDL41981.1|	1.03E-134	paired-like homeodomain transcription factor 3 [Mus musculus]
mmu-miR-103-1-5p	NM_008909	-25.7	0.984521	-29.06	185	GO:0005911,GO:0019898,GO:0043232,GO:0043231,GO:0005488,GO:0005198,GO:00302	K10386|1|0.0|2920|mmu:19041|periplakin	gi|112421039|ref|NP_032935.2|	0	periplakin [Mus musculus]
mmu-miR-103-1-5p	NM_008925	-28.2	0.377441	-25.31	155	GO:0044432,GO:0043234,GO:0016020,GO:0016772,GO:0003676,GO:0046872,GO:0019901,GO:0006952,GO:0035556,GO:0051259,GO:0006796,GO:0001822,GO:00064	K08288|1|0.0|817|mmu:19089|protein kinase C substrate 80K-H	gi|6679465|ref|NP_032951.1|	0	glucosidase 2 subunit beta precursor [Mus musculus]

mmu-miR-103-1-5p	NM_009137	-29.2	0.234054	-25.67	172	GO:0044421,GO:0005125,GO:0006952,GO:0002376,GO:0042221,	K05514|1|2e-33|144|mmu:20299|C-C motif chemokin	gi|5911365|gb|AAD55763.1|AF163476_1	1.24E-32	macrophage-derived chemokine [Mus musculus]
mmu-miR-103-1-5p	NM_009212	-27.2	0.871549	-41.25	317	GO:0030427,GO:0043005,GO:0043231,GO:0044297,GO:0043234,GO:0044444,GO:0046914,GO:0005515,GO:0043021,GO:0032559,GO:0003723,GO:0004003,GO:0043566,GO:0004004,GO:0006357,GO:0006259,GO:0009207,GO:0051259,GO:00215	K14326|1|6e-34|148|dwi:Dwil_GK25498|regulator of nonsense transcripts 1 [EC:3.6.4.-]	gi|74191771|dbj|BAE32841.1|	0	unnamed protein product [Mus musculus]

mmu-miR-103-1-5p	NM_009239	-27.7	0.824049	-25.48	144	GO:0043231,GO:0005488,GO:0003712,GO:0001071,GO:0006355,GO:0044057,	K09194|1|0.0|983|mmu:20688|transcription factor Sp4	gi|148701533|gb|EDL33480.1|	0	trans-acting transcription factor 4 [Mus musculus]
mmu-miR-103-1-5p	NM_009331	-28.5	0.371792	-25.68	148	GO:0043231,GO:0001071,GO:0043565,GO:0002681,GO:0006915,GO:0048562,GO:0007399,GO:0070670,GO:0006351,GO:0008283,GO:0007166,GO:0035112,	K02620|1|8e-143|508|rno:363595|transcription factor 7	gi|148701673|gb|EDL33620.1|	1.72E-143	transcription factor 7, T-cell specific, isoform CRA_a [Mus musculus]
mmu-miR-103-1-5p	NM_009636	-28.1	0.648033	-86.15	580	GO:0044424,GO:0044421,GO:0005488,GO:0003712,GO:0008238,GO:0001071,GO:0048513,GO:0006351,	K08639|1|0.0|773|mdo:100025097|carboxypeptidase X2	gi|160707889|ref|NP_033766.2|	0	adipocyte enhancer-binding protein 1 precursor [Mus musculus]

mmu-miR-103-1-5p	NM_009867	-25.1	0.995369	-25.1	158	GO:0016021,GO:0046872,GO:0016337,GO:0006935,GO:0045216,GO:0030307,	K06797|1|0.0|1649|mmu:12561|cadherin 4, type 1, R-cadherin	gi|6753376|ref|NP_033997.1|	0	cadherin-4 precursor [Mus musculus]
mmu-miR-103-1-5p	NM_009886	-25.9	0.997867	-28.36	156	GO:0016021,GO:0046872,GO:0005515,GO:0004888,GO:0014020,GO:0042471,GO:0016337,GO:0007186,GO:0008105,GO:0007610,GO:0001736,	K04600|1|0.0|5481|mmu:12614|cadherin EGF LAG seven-pass G-type receptor 1 (flamingo)	gi|115648153|ref|NP_034016.2|	0	cadherin EGF LAG seven-pass G-type receptor 1 precursor [Mus musculus]
mmu-miR-103-1-5p	NM_010264	-25.5	0.988404	-44.2	302	GO:0043234,GO:0031981,GO:0046914,GO:0042802,GO:0001071,GO:0003677,GO:0004879,GO:0048869,GO:0009755,GO:00482	K08561|1|0.0|892|ssc:100038028|germ cell nuclear factor	gi|6753956|ref|NP_034394.1|	0	nuclear receptor subfamily 6 group A member 1 isoform 1 [Mus musculus]

mmu-miR-103-1-5p	NM_010472	-31.4	0.180021	-25.75	167	GO:0044297,GO:0005635,GO:0031410,GO:0003676,GO:0046872,GO:0043087,GO:0003006,GO:0048869,GO:0006997,GO:0048232,GO:0006405,	K15044|1|0.0|799|mmu:15463|Arf-GAP domain and FG repeats-containing protein 1	gi|33563260|ref|NP_034602.1|	0	arf-GAP domain and FG repeat-containing protein 1 [Mus musculus]
mmu-miR-103-1-5p	NM_010586	-31.7	0.512901	-69.95	500	GO:0043005,GO:0030133,GO:0005789,GO:0031224,GO:0005262,GO:0005217,GO:0046873,GO:0005543,GO:0004872,GO:0015980,GO:0007599,GO:0006816,GO:0010863,GO:0007169,GO:0030073,GO:0006950,GO:00	K04959|1|0.0|5027|mmu:16439|inositol 1,4,5-triphosphate receptor type 2	gi|60592758|ref|NP_034716.1|	0	inositol 1,4,5-trisphosphate receptor type 2 isoform 2 [Mus musculus]

mmu-miR-103-1-5p	NM_010731	-25.1	0.990303	-40.98	334	GO:0043231,GO:0003676,GO:0046872,GO:0019899,GO:0009888,GO:0006357,GO:00027	K10494|1|0.0|825|mmu:16969|zinc finger and BTB domain-containing protein	gi|117647238|ref|NP_034861.3|	0	zinc finger and BTB domain-containing protein 7A [Mus musculus]
mmu-miR-103-1-5p	NM_010826	-28	0.795712	-51.17	312	GO:0005783,GO:0031224,GO:0031090,	K12337|1|0.0|1487|mmu:17540|inositol 1,4,5-triphosphate receptor-associated cGMP kinase substrate	gi|154275769|ref|NP_034956.2|	0	protein MRVI1 isoform a [Mus musculus]
mmu-miR-103-1-5p	NM_010889	-28.6	0.981024	-42.01	312	GO:0031674,	K11498|1|2e-07|62.0|ptr:461413|centromeric protein E	gi|148222065|ref|NP_035019.1|	0	nebulin [Mus musculus]

mmu-miR-103-1-5p	NM_011161	-25.1	0.893845	-26.47	174	GO:0031981,GO:0044444,GO:0004702,GO:0032559,GO:0045597,GO:0006355,GO:0006796,GO:0002224,GO:0007169,GO:0007264,GO:0006464,GO:0000165,	K04441|1|0.0|700|mmu:19094|p38 MAP kinase [EC:2.7.11.24]	gi|168693637|ref|NP_035291.4|	0	mitogen-activated protein kinase 11 [Mus musculus]
mmu-miR-103-1-5p	NM_011163	-31.9	0.209582	-25.61	159	GO:0043231,GO:0044444,GO:0004674,GO:0032559,GO:0008135,GO:0019888,GO:0006984,GO:0006412,GO:0019049,GO:0008285,GO:0006468,GO:0033273,GO:0006915,GO:0043331,GO:0002237,	K08860|1|0.0|908|mmu:19106|eukaryotic translation initiation factor 2-alpha kinase [EC:2.7.11.1]	gi|6755160|ref|NP_035293.1|	0	interferon-induced, double-stranded RNA-activated protein kinase [Mus musculus]

mmu-miR-103-1-5p	NM_010856	-24.5	0.99848	-111.13	788	-	K10352|1|0.0|3335|mmu:17888|myosin heavy chain	gi|148704367|gb|EDL36314.1|	0	mCG133649, isoform CRA_b [Mus musculus]
mmu-miR-103-2-5p	NM_001004155	-27.6	0.437113	-43.09	320	-	-	gi|21618991|gb|AAH32212.1|	0	9930012K11Rik protein, partial [Mus musculus]
mmu-miR-103-2-5p	NM_001004468	-29.8	0.670002	-27.56	174	GO:0043232,GO:0051427,GO:0021846,GO:0021543,GO:0007051,	K14282|1|0.0|5333|mmu:57752|transforming acidic coiled-coil-containing protein 2	gi|52486843|ref|NP_001004468.1|	0	transforming acidic coiled-coil-containing protein 2 isoform c [Mus musculus]
mmu-miR-103-2-5p	NM_001033668	-26.8	0.99127	-45.15	321	-	K10408|1|0.0|8267|mmu:110084|dynein heavy chain, axonemal	gi|256773234|ref|NP_001028840.1|	0	dynein, axonemal, heavy chain 1 [Mus musculus]
mmu-miR-103-2-5p	NM_001038643	-25.4	0.887692	-43.6	319	GO:0016323,GO:0016021,GO:0015197,GO:0015291,GO:0008509,GO:0015833,GO:0015711,GO:0071715,	K14353|1|0.0|1216|mmu:108116|solute carrier organic anion transporter family, member 3A	gi|84579878|ref|NP_001033732.1|	0	solute carrier organic anion transporter family member 3A1 isoform 2 [Mus musculus]

mmu-miR-103-2-5p	NM_001039376	-26.4	0.979637	-43.52	318	GO:0005815,GO:0043231,GO:0043292,GO:0005515,GO:0006461,	K10352|1|2e-25|120|mdo:100016741|myosin heavy chain	gi|158749578|ref|NP_001034465.2|	0	myomegalin isoform 1 [Mus musculus]
mmu-miR-103-2-5p	NM_001039545	-25.7	0.979477	-40.99	292	GO:0031981,GO:0005924,GO:0016460,GO:0030017,GO:0008092,GO:0003774,GO:0005198,GO:0032559,GO:0050896,GO:0007009,GO:0031033,GO:0006936,	K10352|1|0.0|3358|mmu:17882|myosin heavy chain	gi|205830428|ref|NP_001034634.2|	0	myosin heavy chain IIa [Mus musculus]
mmu-miR-103-2-5p	NM_001039700	-28.6	0.730496	-46.72	327	GO:0031224,GO:0046872,GO:0015075,GO:0030246,GO:0006810,GO:0007186,	K04989|1|0.0|3511|mmu:244646|polycystin 1L3	gi|115583681|ref|NP_001034789.2|	0	polycystic kidney disease protein 1-like 3 isoform a precursor [Mus musculus]

mmu-miR-103-2-5p	NM_001044383	-25.3	0.968893	-25.43	169	GO:0031981,GO:0003676,GO:0006356,GO:0006352,GO:0006342,GO:0006353,GO:00063	K09273|1|0.0|1189|rno:25574|upstream-binding transcription factor	gi|189095264|ref|NP_001099193.2|	0	nucleolar transcription factor 1 isoform 1 [Rattus norvegicus]
mmu-miR-103-2-5p	NM_001076554	-32.7	0.27039	-48.22	333	GO:0032991,GO:0030864,GO:0005913,GO:0031674,GO:0043227,GO:0008092,GO:0046872,GO:0000149,GO:0046983,GO:00308	K06114|1|0.0|4771|mmu:20740|spectrin alpha	gi|115496850|ref|NP_001070022.1|	0	spectrin alpha chain, brain isoform 1 [Mus musculus]
mmu-miR-103-2-5p	NM_001081108	-27.4	0.210133	-25.34	167	-	-	gi|148687256|gb|EDL19203.1|	4.75E-62	mCG7441, isoform CRA_a [Mus musculus]
mmu-miR-103-2-5p	NM_001081128	-27.9	0.696944	-47.45	334	GO:0044444,GO:0019842,GO:0008172,GO:0046914,GO:0016597,GO:0048731,GO:0000097,GO:0006730,GO:0008213,GO:00067	K00548|1|0.0|2512|mmu:238505|5-methyltetrahydrofolate--homocysteine methyltransferase [EC:2.1.1.13]	gi|124487479|ref|NP_001074597.1|	0	methionine synthase [Mus musculus]

mmu-miR-103-2-5p	NM_001081402	-31.1	0.124658	-27.28	176	-	K02208|1|4e-98|359|bmy:Bm1_33825|cyclin-dependent kinase 8/11 [EC:2.7.11.22 2.7.11.23]!K03130|2|2e-10|68.9|bfo:BRAFLDRAFT_65485|transcription initiation factor TFIID subunit 5!K12857|4|8e-10|66.6|dre:393292|Prp8 binding	gi|124487149|ref|NP_001074871.1|	0	WD repeat-containing protein 70 [Mus musculus]
mmu-miR-103-2-5p	NM_001082483	-25.4	0.990622	-48.28	311	-	-	gi|126723102|ref|NP_001075952.1|	0	protein EFR3 homolog B [Mus musculus]

mmu-miR-103-2-5p	NM_001083959	-27.2	0.420681	-26.93	164	GO:0043231,GO:0043232,GO:0003676,GO:0003824,GO:0032559,GO:0022602,GO:0006310,GO:0033365,GO:0048232,GO:0007281,GO:0007128,	K10878|1|0.0|741|mmu:26972|meiotic recombination protein SPO11	gi|145312272|ref|NP_001077428.1|	0	meiotic recombination protein SPO11 isoform c [Mus musculus]
mmu-miR-103-2-5p	NM_001083960	-27.2	0.411842	-26.93	164	GO:0043231,GO:0043232,GO:0003676,GO:0003824,GO:0032559,GO:0022602,GO:0006310,GO:0033365,GO:0048232,GO:0007281,GO:0007128,	K10878|1|0.0|706|mmu:26972|meiotic recombination protein SPO11	gi|145312248|ref|NP_001077429.1|	0	meiotic recombination protein SPO11 isoform b [Mus musculus]
mmu-miR-103-2-5p	NM_001110163	-26.4	0.97856	-43.52	318	GO:0005815,GO:0043231,GO:0043292,GO:0005515,GO:0006461,	K10352|1|2e-25|120|mdo:100016741|myosin heavy chain	gi|158749580|ref|NP_001103633.1|	0	myomegalin isoform 4 [Mus musculus]

mmu-miR-103-2-5p	NM_001110253	-26	0.985322	-45.35	336	GO:0031224,GO:0046872,GO:0051234,	K12478|1|2e-24|117|dre:562943|early endosome antigen 1	gi|159110614|ref|NP_001103723.1|	0	FYVE and coiled-coil domain-containing protein 1 [Mus musculus]
mmu-miR-103-2-5p	NM_001112735	-27.6	0.43656	-43.09	320	-	-	gi|21618991|gb|AAH32212.1|	0	9930012K11Rik protein, partial [Mus musculus]
mmu-miR-103-2-5p	NM_001130515	-26.9	0.522037	-27.41	164	GO:0031981,GO:0003723,GO:0016538,GO:0006357,GO:0000079,	K15189|1|6e-171|601|mmu:71059|protein HEXIM1/2	gi|110625829|ref|NP_081934.1|	3.94E-170	protein HEXIM2 [Mus musculus]
mmu-miR-103-2-5p	NM_001130516	-26.9	0.525569	-27.41	164	GO:0031981,GO:0003723,GO:0016538,GO:0006357,GO:0000079,	K15189|1|6e-171|601|mmu:71059|protein HEXIM1/2	gi|110625829|ref|NP_081934.1|	3.98E-170	protein HEXIM2 [Mus musculus]
mmu-miR-103-2-5p	NM_001136556	-27.7	0.825333	-25.32	169	GO:0031224,	K15621|1|1e-06|57.4|bta:506146|BAI1-associated protein 3	gi|211971048|ref|NP_001130028.1|	0	fer-1-like protein 4 [Mus musculus]

mmu-miR-103-2-5p	NM_001145100	-25.8	0.895591	-27.61	163	-	K10443|1|5e-11|71.2|tgu:100226815|kelch-like protein 2/3!K10453|4|2e-10|69.3|acs:100566431|kelch-like protein 16 (gigaxonin)!K10494|5|3e-10|68.9|dre:565670|zinc finger and BTB domain-containin	gi|223029434|ref|NP_001138572.1|	0	uncharacterized protein LOC100270744 [Mus musculus]
mmu-miR-103-2-5p	NM_001159635	-31.1	0.146617	-27.59	168	GO:0003676,	-	gi|227500539|ref|NP_001153107.1|	2.83E-86	probable RNA-binding protein 18 isoform 2 [Mus musculus]

mmu-miR-103-2-5p	NM_001162533	-27	0.570673	-44.55	336	-	K12470|1|1e-27|125|gga:418608|SH3 domain-containing kinase-binding protein 1!K13738|2|1e-26|122|ssc:100627990|CD2-associated protein	gi|148698353|gb|EDL30300.1|	0	RIKEN cDNA 1700029G01, isoform CRA_b [Mus musculus]
mmu-miR-103-2-5p	NM_001163024	-26.4	0.985505	-25.36	163	GO:0044424,GO:0005083,GO:0006892,	-	gi|28972582|dbj|BAC65707.1|	0	mKIAA1040 protein [Mus musculus]
mmu-miR-103-2-5p	NM_001163025	-26.4	0.985405	-25.36	163	GO:0044424,GO:0005083,GO:0006810,	-	gi|148692491|gb|EDL24438.1|	0	MON2 homolog (yeast), isoform CRA_d [Mus musculus]
mmu-miR-103-2-5p	NM_001163271	-27	0.748511	-44.98	325	GO:0044446,GO:0042995,GO:0046872,GO:0006468,	-	gi|253795534|ref|NP_001156743.1|	0	fibrous sheath CABYR-binding protein [Mus musculus]
mmu-miR-103-2-5p	NM_001163540	-26.3	0.998645	-47.02	319	-	K10388|1|0.0|3881|mmu:18810|plectin	gi|256418964|ref|NP_958788.2|	0	plectin isoform 1e [Mus musculus]

mmu-miR-103-2-5p	NM_001163750	-27.4	0.456591	-28.58	172	-	K10345|1|0.0|848|mmu:79043|SPRY domain-containing SOCS box protein 3	gi|254939631|ref|NP_001157222.1|	0	SPRY domain-containing SOCS box protein 3 isoform 1 [Mus musculus]
mmu-miR-103-2-5p	NM_001163751	-27.4	0.385485	-28.58	172	-	K10345|1|3e-175|615|mmu:79043|SPRY domain-containing SOCS box protein 3	gi|254939633|ref|NP_001157223.1|	0	SPRY domain-containing SOCS box protein 3 isoform 2 [Mus musculus]
mmu-miR-103-2-5p	NM_001164630	-29.5	0.469125	-25.71	172	-	-	gi|148691959|gb|EDL23906.1|	0	melanoma associated antigen (mutated) 1-like 1 [Mus musculus]
mmu-miR-103-2-5p	NM_001164632	-29.5	0.448964	-25.71	172	-	-	gi|148691959|gb|EDL23906.1|	0	melanoma associated antigen (mutated) 1-like 1 [Mus musculus]
mmu-miR-103-2-5p	NM_001164633	-29.5	0.444047	-25.71	172	-	-	gi|148691959|gb|EDL23906.1|	0	melanoma associated antigen (mutated) 1-like 1 [Mus musculus]

mmu-miR-103-2-5p	NM_001165948	-27.8	0.872108	-32.17	171	-	K10500|1|4e-15|86.3|rno:313666|zinc finger and BTB domain-containing protein	gi|260099686|ref|NP_001159420.1|	0	pogo transposable element with ZNF domain isoform 2 [Mus musculus]
mmu-miR-103-2-5p	NM_001172064	-28.5	0.399308	-26.65	161	-	-	gi|60359886|dbj|BAD90162.1|	0	mFLJ00101 protein [Mus musculus]
mmu-miR-103-2-5p	NM_001172065	-28.5	0.380501	-26.65	161	-	-	gi|285026461|ref|NP_001165536.1|	0	leucine-rich repeat-containing protein 56 isoform b [Mus musculus]
mmu-miR-103-2-5p	NM_001177504	-29.1	0.295444	-25.1	161	-	K06236|1|3e-08|61.2|hmg:100211987|collagen, type I/II/III/V/XI, alpha!K08131|2|2e-07|58.5|mdo:100032118|collagen, type IX, alpha	gi|294610658|ref|NP_001170975.1|	0	uncharacterized protein LOC625464 [Mus musculus]

mmu-miR-103-2-5p	NM_001177667		-32.7	0.270299	-48.22	333	GO:0032991,GO:0005913,GO:0043227,GO:0031674,GO:0030864,GO:0005198,GO:0000149,GO:0046983,GO:0008092,GO:0046872,GO:0030835,GO:0006915,GO:00069	K06114|1|0.0|4729|rno:64159|spectrin alpha	gi|295054266|ref|NP_001171138.1|	0	spectrin alpha chain, brain isoform 2 [Mus musculus]
mmu-miR-103-2-5p	NM_001177668		-32.7	0.268478	-48.22	333	GO:0032991,GO:0030864,GO:0005913,GO:0031674,GO:0043227,GO:0008092,GO:0046872,GO:0000149,GO:0046983,GO:00308	K06114|1|0.0|4716|mmu:20740|spectrin alpha	gi|295054271|ref|NP_001171139.1|	0	spectrin alpha chain, brain isoform 3 [Mus musculus]

mmu-miR-103-2-5p	NM_001177845	-30.3	0.375867	-26.47	175	GO:0016023,GO:0044425,GO:0001726,GO:0005080,GO:0035091,GO:0004872,GO:0042802,GO:0035325,GO:0046983,GO:0042108,GO:0030097,GO:0070339,GO:0032092,GO:0034142,GO:0051091,GO:0032479,GO:0019221,GO:0042742,GO:0070391,GO:0002690,GO:0032722,GO:00308	K05403|1|8e-103|376|mmu:117149|toll-interleukin 1 receptor (TIR) domain-containing adaptor protein	gi|148693432|gb|EDL25379.1|	1.21E-115	toll-interleukin 1 receptor (TIR) domain-containing adaptor protein, isoform CRA_b [Mus musculus]

mmu-miR-103-2-5p	NM_001177846	-30.3	0.37467	-26.47	175	GO:0016023,GO:0044425,GO:0001726,GO:0005080,GO:0035091,GO:0004872,GO:0042802,GO:0035325,GO:0046983,GO:0042108,GO:0030097,GO:0070339,GO:0032092,GO:0034142,GO:0051091,GO:0032479,GO:0019221,GO:0042742,GO:0070391,GO:0002690,GO:0032722,GO:00308	K05403|1|8e-103|376|mmu:117149|toll-interleukin 1 receptor (TIR) domain-containing adaptor protein	gi|148693432|gb|EDL25379.1|	1.20E-115	toll-interleukin 1 receptor (TIR) domain-containing adaptor protein, isoform CRA_b [Mus musculus]

mmu-miR-103-2-5p	NM_001177847	-30.3	0.371061	-26.47	175	GO:0016023,GO:0044425,GO:0001726,GO:0005080,GO:0035091,GO:0004872,GO:0042802,GO:0035325,GO:0046983,GO:0042108,GO:0030097,GO:0070339,GO:0032092,GO:0034142,GO:0051091,GO:0032479,GO:0019221,GO:0042742,GO:0070391,GO:0002690,GO:0032722,GO:00308	K05403|1|8e-103|376|mmu:117149|toll-interleukin 1 receptor (TIR) domain-containing adaptor protein	gi|148693432|gb|EDL25379.1|	1.19E-115	toll-interleukin 1 receptor (TIR) domain-containing adaptor protein, isoform CRA_b [Mus musculus]

mmu-miR-103-2-5p	NM_001199141	-26.5	0.860058	-41.17	310	GO:0031981,GO:0046914,GO:0003676,GO:0051704,GO:0010467,GO:0009987,	K08881|1|7e-10|67.8|dre:442869|tripartite motif-containing protein 24!K08883|2|1e-06|57.4|isc:IscW_ISCW017611|tripartite motif-containing protein 33 [EC:6.3.2.19]	gi|148700349|gb|EDL32296.1|	0	zinc finger, MYND domain containing 11, isoform CRA_e [Mus musculus]
mmu-miR-103-2-5p	NM_001205312	-30.6	0.348532	-44.16	318	GO:0043231,GO:0043232,GO:0044444,GO:0005126,GO:0004713,GO:0032559,GO:0035556,GO:0001959,GO:0006464,	K11219|1|0.0|2435|mmu:54721|non-receptor tyrosine kinase [EC:2.7.10.2]	gi|328887920|ref|NP_001192241.1|	0	non-receptor tyrosine-protein kinase TYK2 isoform 1 [Mus musculus]
mmu-miR-103-2-5p	NM_001242419	-27.9	0.741138	-47.94	326	GO:0031224,	-	gi|37359864|dbj|BAC97910.1|	2.58E-172	mKIAA0247 protein [Mus musculus]
mmu-miR-103-2-5p	NM_001271386	-25.4	0.996422	-25.28	176	-	-	-	-	-
mmu-miR-103-2-5p	NM_001271538	-27.8	0.831608	-41.23	319	-	-	-	-	-

mmu-miR-103-2-5p	NM_001271540	-27.8	0.829096	-41.23	319	-	-	-	-	-
mmu-miR-103-2-5p	NM_008909	-25.7	0.98157	-29.94	185	GO:0005911,GO:0019898,GO:0043232,GO:0043231,GO:0005488,GO:0005198,GO:00302	K10386|1|0.0|2920|mmu:19041|periplakin	gi|112421039|ref|NP_032935.2|	0	periplakin [Mus musculus]
mmu-miR-103-2-5p	NM_009137	-29.4	0.204687	-26.55	172	GO:0044421,GO:0005125,GO:0006952,GO:0002376,GO:0042221,	K05514|1|2e-33|144|mmu:20299|C-C motif chemokin	gi|5911365|gb|AAD55763.1|AF163476_1	1.24E-32	macrophage-derived chemokine [Mus musculus]

mmu-miR-103-2-5p	NM_009212	-27.4	0.835366	-42.02	317	GO:0030427,GO:0043005,GO:0043231,GO:0044297,GO:0043234,GO:0044444,GO:0046914,GO:0005515,GO:0043021,GO:0032559,GO:0003723,GO:0004003,GO:0043566,GO:0004004,GO:0006357,GO:0006259,GO:0009207,GO:0051259,GO:00215	K14326|1|6e-34|148|dwi:Dwil_GK25498|regulator of nonsense transcripts 1 [EC:3.6.4.-]	gi|74191771|dbj|BAE32841.1|	0	unnamed protein product [Mus musculus]

mmu-miR-103-2-5p	NM_009886	-25.8	0.997844	-29.24	156	GO:0016021,GO:0046872,GO:0005515,GO:0004888,GO:0014020,GO:0042471,GO:0016337,GO:0007186,GO:0008105,GO:0007610,GO:0001736,	K04600|1|0.0|5481|mmu:12614|cadherin EGF LAG seven-pass G-type receptor 1 (flamingo)	gi|115648153|ref|NP_034016.2|	0	cadherin EGF LAG seven-pass G-type receptor 1 precursor [Mus musculus]
mmu-miR-103-2-5p	NM_010586	-27.7	0.955979	-50.91	349	GO:0043005,GO:0030133,GO:0005789,GO:0031224,GO:0005262,GO:0005217,GO:0046873,GO:0005543,GO:0004872,GO:0015980,GO:0007599,GO:0006816,GO:0010863,GO:0007169,GO:0030073,GO:0006950,GO:00	K04959|1|0.0|5027|mmu:16439|inositol 1,4,5-triphosphate receptor type 2	gi|60592758|ref|NP_034716.1|	0	inositol 1,4,5-trisphosphate receptor type 2 isoform 2 [Mus musculus]

mmu-miR-103-2-5p	NM_010731	-25.1	0.988266	-41.86	334	GO:0043231,GO:0003676,GO:0046872,GO:0019899,GO:0009888,GO:0006357,GO:00027	K10494|1|0.0|825|mmu:16969|zinc finger and BTB domain-containing protein	gi|117647238|ref|NP_034861.3|	0	zinc finger and BTB domain-containing protein 7A [Mus musculus]
mmu-miR-103-2-5p	NM_010910	-28.9	0.277281	-25.58	151	-	K04572|1|0.0|685|mmu:18039|neurofilament light polypeptide	gi|116283387|gb|AAH16436.1|	0	Nefl protein [Mus musculus]
mmu-miR-103-2-5p	NM_011161	-25.3	0.859275	-27.35	174	GO:0031981,GO:0044444,GO:0004702,GO:0032559,GO:0045597,GO:0006355,GO:0006796,GO:0002224,GO:0007169,GO:0007264,GO:0006464,GO:0000165,	K04441|1|0.0|700|mmu:19094|p38 MAP kinase [EC:2.7.11.24]	gi|168693637|ref|NP_035291.4|	0	mitogen-activated protein kinase 11 [Mus musculus]

mmu-miR-103-2-5p	NM_011323	-25.3	0.999468	-25.64	168	GO:0044297,GO:0031410,GO:0033267,GO:0005887,GO:0031420,GO:0005272,GO:0032559,GO:0048513,GO:0006935,GO:0034220,GO:0008366,GO:0006814,GO:00083	K04840|1|0.0|3398|mmu:20273|voltage-gated sodium channel type VIII alpha	gi|172072624|ref|NP_035453.2|	0	sodium channel protein type 8 subunit alpha isoform 2 [Mus musculus]
mmu-miR-103-2-5p	NM_011551	-26	0.934898	-25.43	169	GO:0031981,GO:0003676,GO:0006356,GO:0006352,GO:0006342,GO:0006353,GO:00063	K09273|1|0.0|1248|aml:100483006|upstream-binding transcription factor	gi|113205057|ref|NP_035681.2|	0	nucleolar transcription factor 1 isoform 1 [Mus musculus]
mmu-miR-103-2-5p	NM_011784	-25.2	0.9477	-25.88	160	GO:0031982,GO:0043231,GO:0016021,GO:0005488,GO:0004888,GO:0032501,GO:00071	K04174|1|0.0|670|mmu:23796|apelin receptor	gi|6753014|ref|NP_035914.1|	0	apelin receptor [Mus musculus]

mmu-miR-103-2-5p	NM_011789	-25.3	0.998275	-25.07	168	GO:0043231,GO:0005874,GO:0015629,GO:0044444,GO:0030027,GO:0019897,GO:0015631,GO:0030178,GO:0035411,	K02085|1|0.0|3631|mmu:23805|adenomatosis polyposis coli protein	gi|117938322|ref|NP_035919.2|	0	adenomatous polyposis coli protein 2 [Mus musculus]
mmu-miR-103-2-5p	NM_012046	-29.1	0.216878	-26.93	164	GO:0043231,GO:0043232,GO:0003676,GO:0003824,GO:0032559,GO:0022602,GO:0006310,GO:0033365,GO:0048232,GO:0007281,GO:0007128,	K10878|1|0.0|685|mmu:26972|meiotic recombination protein SPO11	gi|6755624|ref|NP_036176.1|	0	meiotic recombination protein SPO11 isoform a [Mus musculus]
mmu-miR-103-2-5p	NM_013595	-26.7	0.482887	-31.32	156	GO:0000118,GO:0000785,GO:0003676,GO:0006357,GO:0048856,GO:0043009,GO:0006342,GO:0016570,	K11591|1|4e-152|538|rno:362834|methyl-CpG-binding domain protein 3	gi|157818023|ref|NP_001102205.1|	2.51E-151	methyl-CpG-binding domain protein 3 [Rattus norvegicus]

mmu-miR-103-2-5p	NM_013716	-34.5	0.039584	-29.06	175	GO:0005737,GO:0016020,GO:0003724,GO:0003676,GO:0004518,GO:0003678,GO:0000166,GO:0007264,	K12741|1|3e-08|61.6|dan:Dana_GF18582|heterogeneous nuclear ribonucleoprotein A1/A3!K01509|2|9e-08|60.1|dsi:Dsim_GD21374|adenosinetriphosphatase [EC:3.6.1.3]	gi|60359872|dbj|BAD90155.1|	0	mKIAA4115 protein [Mus musculus]
mmu-miR-103-2-5p	NM_015790	-34.8	0.035955	-29.53	170	GO:0016021,GO:0009986,GO:0005515,GO:0042102,GO:0023052,GO:0002204,GO:00421	K06710|1|3e-164|580|mmu:50723|inducible T-cell co-stimulator ligand	gi|7657220|ref|NP_056605.1|	1.90E-163	ICOS ligand precursor [Mus musculus]

mmu-miR-103-2-5p	NM_018793	-30.6	0.345372	-44.16	318	GO:0043231,GO:0043232,GO:0044444,GO:0005126,GO:0004713,GO:0032559,GO:0035556,GO:0001959,GO:0006464,	K11219|1|0.0|2373|mmu:54721|non-receptor tyrosine kinase [EC:2.7.10.2]	gi|133922607|ref|NP_061263.2|	0	non-receptor tyrosine-protein kinase TYK2 isoform 2 [Mus musculus]
mmu-miR-103-2-5p	NM_019438	-31.6	0.187513	-44.41	336	GO:0000793,GO:0043231,GO:0005488,GO:0000070,	K06678|1|0.0|1886|mmu:54392|condensin complex subunit 3	gi|169234780|ref|NP_062311.1|	0	condensin complex subunit 3 [Mus musculus]
mmu-miR-103-2-5p	NM_019568	-27.2	0.465324	-25.84	166	GO:0044421,GO:0043231,GO:0005125,GO:0002376,GO:0042221,GO:0023052,GO:00071	K10033|1|2e-50|181|ecb:100072697|C-X-C motif chemokine 14	gi|354483209|ref|XP_003503787.1|	5.49E-52	PREDICTED: hypothetical protein LOC100754406 [Cricetulus griseus]
mmu-miR-103-2-5p	NM_019570	-25.5	0.953928	-29.09	162	GO:0031981,GO:0046872,GO:0003677,GO:0034061,GO:0019985,GO:0009416,	K03515|1|0.0|2335|mmu:56210|DNA repair protein REV1 [EC:2.7.7.-]	gi|40254551|ref|NP_062516.2|	0	DNA repair protein REV1 [Mus musculus]

mmu-miR-103-2-5p	NM_019793	-29.2	0.212548	-26.53	164	GO:0031224,	K06537|1|4e-29|130|acs:100551648|CD151 antigen!K06497|3|4e-27|123|nve:NEMVE_v1g236446|CD63 antigen	gi|254281189|ref|NP_062767.3|	8.01E-130	tetraspanin-3 [Mus musculus]
mmu-miR-103-2-5p	NM_019923	-27.7	0.956945	-50.91	349	GO:0043005,GO:0030133,GO:0005789,GO:0031224,GO:0005262,GO:0005217,GO:0046873,GO:0005543,GO:0004872,GO:0015980,GO:0007599,GO:0006816,GO:0010863,GO:0007169,GO:0030073,GO:0006950,GO:00	K04959|1|0.0|5024|rno:81678|inositol 1,4,5-triphosphate receptor type 2	gi|60593032|ref|NP_064307.2|	0	inositol 1,4,5-trisphosphate receptor type 2 isoform 1 [Mus musculus]

mmu-miR-103-2-5p	NM_021355	-27	0.667345	-26.33	164	GO:0031012,GO:0044464,GO:0009887,GO:0009611,GO:0007179,	K08121|1|0.0|649|mmu:14264|fibromodulin	gi|30851675|gb|AAH52673.1|	0	Fmod protein [Mus musculus]
mmu-miR-103-2-5p	NM_021544	-28.4	0.832549	-46.83	322	GO:0042383,GO:0031224,GO:0044291,GO:0005272,GO:0006814,	K04838|1|0.0|3652|mmu:20271|voltage-gated sodium channel type V alpha	gi|84875498|ref|NP_067519.2|	0	sodium channel protein type 5 subunit alpha isoform 1 [Mus musculus]
mmu-miR-103-2-5p	NM_023538	-25.8	0.759789	-29.06	174	GO:0005740,GO:0001727,GO:0032559,GO:0007186,GO:0016310,GO:0006672,	K09881|1|0.0|811|mmu:69923|acylglycerol kinase [EC:2.7.1.94]	gi|37537518|ref|NP_076027.1|	0	acylglycerol kinase, mitochondrial precursor [Mus musculus]
mmu-miR-103-2-5p	NM_023908	-25.4	0.969532	-43.6	319	GO:0016323,GO:0016021,GO:0015197,GO:0015291,GO:0008509,GO:0015833,GO:0015711,GO:0071715,	K14353|1|0.0|1247|rno:140915|solute carrier organic anion transporter family, member 3A	gi|84579880|ref|NP_076397.2|	0	solute carrier organic anion transporter family member 3A1 isoform 1 [Mus musculus]

mmu-miR-103-2-5p	NM_025856	-27	0.509902	-44.55	336	-	K12470|1|1e-16|89.4|dre:793623|SH3 domain-containing kinase-binding protein 1!K13738|4|6e-14|80.1|xla:445851|CD2-associated protein	gi|242117962|ref|NP_001156005.1|	0	SH3 domain-containing protein 21 isoform 1 [Mus musculus]
mmu-miR-103-2-5p	NM_026434	-31.1	0.152687	-27.59	168	GO:0003676,	K12842|1|2e-06|55.5|nvi:100118482|U2-associated protein SR140	gi|13385930|ref|NP_080710.1|	2.18E-105	probable RNA-binding protein 18 isoform 1 [Mus musculus]
mmu-miR-103-2-5p	NM_026508	-31.4	0.119107	-28.67	179	GO:0043231,GO:0032813,GO:0032559,GO:0006979,GO:0044267,	K09488|1|0.0|1381|mmu:68015|TNF receptor-associated protein 1	gi|148664808|gb|EDK97224.1|	0	TNF receptor-associated protein 1 [Mus musculus]

mmu-miR-103-2-5p	NM_027027	-28.6	0.164693	-26.75	162	GO:0007165,	K10331|1|1e-169|596|mmu:69299|ankyrin repeat and SOCS box protein 9	gi|21311897|ref|NP_081303.1|	8.24E-169	ankyrin repeat and SOCS box protein 9 [Mus musculus]
mmu-miR-103-2-5p	NM_027141	-30.6	0.109754	-28.58	172	-	K10345|1|0.0|651|rno:302981|SPRY domain-containing SOCS box protein 3	gi|254939629|ref|NP_081417.2|	0	SPRY domain-containing SOCS box protein 3 isoform 3 [Mus musculus]
mmu-miR-103-2-5p	NM_027434	-25.6	0.902116	-48.81	332	-	K15559|1|3e-158|556|cfa:485866|regulator of Ty1 transposition protein 103	gi|148674304|gb|EDL06251.1|	3.15E-176	RIKEN cDNA 2610304G08, isoform CRA_b [Mus musculus]
mmu-miR-103-2-5p	NM_027658	-26.9	0.528045	-27.41	164	GO:0031981,GO:0003723,GO:0016538,GO:0006357,GO:0000079,	K15189|1|6e-171|601|mmu:71059|protein HEXIM1/2	gi|110625829|ref|NP_081934.1|	4.01E-170	protein HEXIM2 [Mus musculus]

mmu-miR-103-2-5p	NM_027742	-30.3	0.26342	-25.7	171	GO:0019904,GO:0007166,	K10352|1|3e-08|62.0|tgu:100219858|myosin heavy chain	gi|110625827|ref|NP_082018.1|	0	leucine-rich repeat flightless-interacting protein 2 isoform 2 [Mus musculus]
mmu-miR-103-2-5p	NM_027801	-34.2	0.066133	-28.9	178	-	K11423|1|1e-08|63.5|dgr:Dgri_GH16034|histone-lysine N-methyltransferase SETD2 [EC:2.1.1.43]!K11515|2|2e-07|59.3|oaa:100090319|inner centromere protein!K10352|3|3e-06|55.5|dwi:Dwil_GK20720|myosin heavy chain	gi|298358637|ref|NP_082077.1|	0	uncharacterized protein LOC212153 [Mus musculus]

mmu-miR-103-2-5p	NM_027830	-30.3	0.3143	-27.1	157	GO:0016462,GO:0032559,GO:0006259,	K10738|1|0.0|2020|mmu:71567|minichromosome maintenance	gi|86439953|ref|NP_082106.2|	0	DNA replication licensing factor MCM9 [Mus musculus]
mmu-miR-103-2-5p	NM_027871	-30.6	0.26289	-26.25	168	GO:0044444,GO:0005088,GO:0019222,GO:0006917,GO:0007266,GO:0009966,GO:0007169,	K07532|1|2e-19|99.4|tgu:100222469|Rho guanine nucleotide exchange factor 12	gi|27229120|ref|NP_082147.1|	0	rho guanine nucleotide exchange factor 3 [Mus musculus]
mmu-miR-103-2-5p	NM_028021	-27.8	0.82623	-41.23	319	GO:0043005,GO:0032432,GO:0030427,GO:0042623,GO:0003774,GO:0003779,GO:0032559,GO:0006935,GO:0030029,	K10352|1|0.0|2806|mmu:71960|myosin heavy chain	gi|148690794|gb|EDL22741.1|	0	myosin, heavy polypeptide 14, isoform CRA_b [Mus musculus]

mmu-miR-103-2-5p	NM_028207	-29.8	0.398481	-26.23	171	GO:0031981,GO:0044459,GO:0044444,GO:0004721,GO:0050856,GO:0043409,GO:0050863,GO:0043009,GO:0002224,GO:0007169,GO:0006470,GO:0007346,	K04459|1|2e-100|368|mmu:72349|dual specificity phosphatase [EC:3.1.3.16 3.1.3.48]	gi|148702127|gb|EDL34074.1|	2.01E-106	dual specificity phosphatase 3 (vaccinia virus phosphatase VH1-related), isoform CRA_a [Mus musculus]
mmu-miR-103-2-5p	NM_029404	-27.6	0.886744	-26.2	155	GO:0046914,	K11347|1|4e-30|135|mdo:100011405|protein Jade-1	gi|270288744|ref|NP_083680.2|	0	PHD finger protein 14 isoform 2 [Mus musculus]
mmu-miR-103-2-5p	NM_029545	-25.5	0.847471	-25.47	174	GO:0044424,GO:0016787,GO:0009063,	K13113|1|6e-23|110|smm:Smp_094530|ubiquitin-like protein 5	gi|31324532|ref|NP_083821.1|	3.38E-90	probable D-tyrosyl-tRNA(Tyr) deacylase 2 [Mus musculus]
mmu-miR-103-2-5p	NM_030237	-26	0.599152	-26.21	165	GO:0043231,GO:0003676,GO:0001071,GO:0006351,	-	gi|242332553|ref|NP_084513.3|	0	spermatogenic leucine zipper protein 1 [Mus musculus]

mmu-miR-103-2-5p	NM_030595	-28.4	0.895657	-26.14	179	GO:0044459,GO:0044431,GO:0019900,GO:0048193,GO:0006886,	K07295|1|2e-19|101|dpe:Dper_GL19043|nuclear receptor subfamily 2 group A!K10260|2|6e-08|62.8|api:100166211|F-box and WD-40 domain protein 7!K11887|3|1e-07|61.6|ame:409686|proteasomal ATPase-associated factor 1!K10342|4|9e-	gi|158854037|ref|NP_085098.1|	0	neurobeachin [Mus musculus]

mmu-miR-103-2-5p	NM_053087	-38.9	0.002929	-32.39	180	GO:0016021,GO:0004712,GO:0070851,GO:0007088,GO:0008284,GO:0043405,GO:0007176,GO:0048646,GO:0000165,	K08774|1|5e-09|63.5|gga:414743|transforming growth factor, alpha!K09784|2|1e-08|62.0|mcc:702502|epireguli	gi|16716373|ref|NP_444317.1|	7.60E-85	epigen precursor [Mus musculus]
mmu-miR-103-2-5p	NM_053196	-27.8	0.588651	-25.88	161	GO:0019866,GO:0043231,GO:0031224,GO:0015075,GO:0000041,	K03351|1|9e-79|296|smm:Smp_136960|anaphase-promoting complex subunit 4	gi|86439984|ref|NP_444426.3|	1.47E-178	sideroflexin-2 [Mus musculus]
mmu-miR-103-2-5p	NM_053263	-25.1	0.986468	-27.09	163	GO:0005681,GO:0031981,GO:0003723,GO:0051032,GO:0050658,GO:0000377,	K12741|1|3e-107|386|ptr:470592|heterogeneous nuclear ribonucleoprotein A1/A3	gi|392332853|ref|XP_003752716.1|	9.81E-111	PREDICTED: regulator of G-protein signaling protein-like [Rattus norvegicus]

mmu-miR-103-2-5p	NM_054096	-30.3	0.375335	-26.47	175	GO:0016023,GO:0044425,GO:0001726,GO:0005080,GO:0035091,GO:0004872,GO:0042802,GO:0035325,GO:0046983,GO:0042108,GO:0030097,GO:0070339,GO:0032092,GO:0034142,GO:0051091,GO:0032479,GO:0019221,GO:0042742,GO:0070391,GO:0002690,GO:0032722,GO:00308	K05403|1|8e-103|376|mmu:117149|toll-interleukin 1 receptor (TIR) domain-containing adaptor protein	gi|148693432|gb|EDL25379.1|	1.21E-115	toll-interleukin 1 receptor (TIR) domain-containing adaptor protein, isoform CRA_b [Mus musculus]
mmu-miR-103-2-5p	NM_130458	-30.6	0.16372	-43.24	320	GO:0043231,GO:0046872,GO:0003676,GO:0001503,GO:0006355,	K09197|1|0.0|702|mmu:170574|transcription factor Sp7	gi|18485518|ref|NP_569725.1|	0	transcription factor Sp7 [Mus musculus]

mmu-miR-103-2-5p	NM_139153	-28.3	0.509986	-42.38	323	GO:0044424,GO:0017111,GO:0005083,GO:0008047,GO:0046914,GO:0032561,GO:0043087,GO:0032012,	K12491|1|0.0|1548|mmu:213990|Arf-GAP, GTPase, ANK repeat and PH domain-containing protein 1/2/3	gi|124301219|ref|NP_631892.2|	0	arf-GAP with GTPase, ANK repeat and PH domain-containing protein 3 isoform a [Mus musculus]
mmu-miR-103-2-5p	NM_145554	-28.3	0.442505	-25.58	154	GO:0043005,GO:0016323,GO:0030130,GO:0005882,GO:0005768,GO:0030128,GO:0004871,GO:0035091,GO:0060090,GO:0070325,GO:0045309,GO:0032947,GO:0006898,GO:0008203,GO:0009100,GO:0007165,GO:0045807,GO:0051098,GO:00550	K12474|1|1e-165|584|mmu:100017|low density lipoprotein receptor adapter protein 1	gi|160333775|ref|NP_663529.2|	7.44E-165	low density lipoprotein receptor adapter protein 1 [Mus musculus]

mmu-miR-103-2-5p	NM_145610	-31.7	0.070557	-47.8	309	GO:0031981,GO:0032559,GO:0006396,GO:0001558,	K14859|1|0.0|902|mmu:235036|ribosome biogenesis protein SSF1/2	gi|255683374|ref|NP_663585.2|	0	suppressor of SWI4 1 homolog [Mus musculus]
mmu-miR-103-2-5p	NM_146130	-25.1	0.987102	-27.09	163	GO:0005681,GO:0031981,GO:0003723,GO:0051032,GO:0050658,GO:0000377,	K12741|1|4e-108|388|ptr:470592|heterogeneous nuclear ribonucleoprotein A1/A3	gi|332815260|ref|XP_525973.3|	2.04E-108	PREDICTED: heterogeneous nuclear ribonucleoprotein A3-like, partial [Pan troglodytes]
mmu-miR-103-2-5p	NM_001168358	-22.1	0.999997	-88.28	684	GO:0016741,GO:0006730,GO:0006399,	K15450|1|8e-70|266|mmu:209584|tRNA wybutosine-synthesizing protein 3 [EC:2.1.1.-]	gi|148679945|gb|EDL11892.1|	1.39E-104	tRNA-yW synthesizing protein 3 homolog (S. cerevisiae), isoform CRA_c [Mus musculus]

mmu-miR-103-3p	NM_001001144	-28.8	0.583038	-47.01	324	GO:0032991,GO:0030134,GO:0031224,GO:0031090,GO:0012505,GO:0005515,GO:0032934,GO:0016125,GO:0006633,GO:0043434,GO:0010894,GO:0010870,GO:00325	K10260|1|6e-17|91.3|pon:100173626|F-box and WD-40 domain protein 7	gi|148677071|gb|EDL09018.1|	0	SREBP cleavage activating protein, isoform CRA_a [Mus musculus]
mmu-miR-103-3p	NM_001001985	-29	0.704217	-30.56	161	GO:0005789,GO:0005740,GO:0008080,	-	gi|134288912|ref|NP_001001985.3|	4.43E-124	N-acetylaspartate synthetase [Mus musculus]
mmu-miR-103-3p	NM_001004062	-29	0.654566	-26.18	164	GO:0031981,GO:0016020,GO:0005488,GO:0003713,GO:0006357,GO:0051704,	K15309|1|0.0|1067|mmu:382056|CREB-regulated transcription coactivator	gi|37360010|dbj|BAC97983.1|	0	mKIAA0616 protein [Mus musculus]

mmu-miR-103-3p	NM_001004177	-28.7	0.877329	-26.16	155	GO:0031224,GO:0044424,GO:0046872,GO:0004888,GO:0016477,GO:0016337,GO:0060897,GO:0007186,GO:0006355,GO:0048812,GO:0048232,	K04601|1|0.0|5459|mmu:53883|cadherin EGF LAG seven-pass G-type receptor 2 (flamingo)	gi|114050897|ref|NP_001004177.2|	0	cadherin EGF LAG seven-pass G-type receptor 2 isoform 2 precursor [Mus musculus]
mmu-miR-103-3p	NM_001004761	-25.9	0.988489	-30.92	173	GO:0031224,GO:0004888,GO:0007166,	K08469|1|0.0|2289|mmu:241263|G protein-coupled receptor 158	gi|52350563|ref|NP_001004761.1|	0	probable G-protein coupled receptor 158 precursor [Mus musculus]
mmu-miR-103-3p	NM_001005420	-34.2	0.120428	-29.95	181	GO:0043231,GO:0005488,	-	gi|50510571|dbj|BAD32271.1|	0	mKIAA0649 protein [Mus musculus]
mmu-miR-103-3p	NM_001024720	-26	0.999876	-42.54	315	GO:0044459,GO:0044444,GO:0005578,GO:0046872,GO:0006464,GO:0050953,	K12567|1|0.0|1253|rno:84015|titin [EC:2.7.11.1]	gi|154689979|ref|NP_001019891.2|	0	hemicentin 1 precursor [Mus musculus]

mmu-miR-103-3p	NM_001024837	-27.8	0.86128	-45.67	318	GO:0031981,GO:0043169,GO:0003723,GO:0004000,GO:0016553,GO:0006396,	K13194|1|0.0|1286|mmu:110532|double stranded RNA-specific editase B [EC:3.5.-.-]	gi|67625747|ref|NP_001020008.1|		0	double-stranded RNA-specific editase 1 isoform 2 [Mus musculus]
mmu-miR-103-3p	NM_001024918	-29.2	0.491532	-25.14	167	GO:0043231,GO:0003676,GO:0006357,GO:0021510,GO:0007420,GO:0009653,	K09174|1|0.0|1402|mmu:71137|regulatory factor X 4	gi|148689463|gb|EDL21410.1|		0	regulatory factor X, 4 (influences HLA class II expression), isoform CRA_c [Mus musculus]
mmu-miR-103-3p	NM_001025106	-27.5	0.796404	-47.36	307	GO:0016020,	-	gi|68342032|ref|NP_001020277.1|		0	transmembrane protein 201 isoform a [Mus musculus]
mmu-miR-103-3p	NM_001029936	-26.2	0.976388	-27.73	156	GO:0043231,	K11498|1|3e-20|102|mdo:100013897|centromeric protein E	gi|60360496|dbj|BAD90492.1|		0	mKIAA4061 protein [Mus musculus]
mmu-miR-103-3p	NM_001033145	-29	0.521147	-25.45	152	GO:0005798,GO:0030120,	-	gi|92058707|sp|Q3USZ8.1|DIA1_MOUSE		0	RecName: Full=Deleted in autism protein 1 homolog; Flags: Precursor

mmu-miR-103-3p	NM_001033208	-30.1	0.221661	-47.62	323	-	K10352|1|1e-14|82.4|cin:100176948|myosin heavy chain!K11499|4|2e-10|68.6|aml:100465502|centromere protein F	gi|85701616|ref|NP_001028380.1|	0	myocardial zonula adherens protein precursor [Mus musculus]
mmu-miR-103-3p	NM_001033331	-28.3	0.808886	-25.04	153	-	K10382|1|1e-08|64.3|spu:756550|dystonin	gi|120407058|ref|NP_001028503.2|	0	GAS2-like protein 3 [Mus musculus]

mmu-miR-103-3p	NM_001033369	-31.8	0.417366	-26.23	175	GO:0044424,GO:0031224,GO:0043235,GO:0070696,GO:0032559,GO:0043169,GO:0017002,GO:0032403,GO:0007179,GO:0006796,GO:0022602,GO:0050796,GO:0009855,GO:0021543,GO:0043434,GO:0006915,GO:0048869,GO:0006464,GO:0009746,GO:0022037,GO:0001832,GO:00108	K13568|1|0.0|774|mcc:697826|activin receptor type-1C [EC:2.7.11.30]	gi|122890143|emb|CAM13261.1|	0	activin A receptor type IC [Mus musculus]
mmu-miR-103-3p	NM_001033448	-32.3	0.13187	-25.72	160	-	K13172|1|8e-06|53.9|hsa:23524|serine/arginine repetitive matrix protein 2	gi|75677454|ref|NP_001028620.1|	0	AP-5 complex subunit beta-1 [Mus musculus]

mmu-miR-103-3p	NM_001034115	-25.9	0.990915	-44.22	315	GO:0016020,GO:0032947,GO:0035254,GO:0071855,	K15009|1|0.0|2565|mmu:243961|SH3 and multiple ankyrin repeat domains protein	gi|283549150|ref|NP_001029287.1|	0	SH3 and multiple ankyrin repeat domains protein 1 [Mus musculus]
mmu-miR-103-3p	NM_001037736	-29.9	0.504109	-27.24	168	GO:0044464,GO:0005088,GO:0019222,GO:0007266,	K05156|1|1e-24|117|gga:419067|tumor necrosis factor receptor superfamily member 19-like!K08878|2|2e-20|103|dre:560226|breakpoint cluster region protein [EC:2.7.11.1]!K04436|3|6e-20|101|ssc:100521718|mitogen-activated protein	gi|60360312|dbj|BAD90400.1|	0	mKIAA0294 protein [Mus musculus]

mmu-miR-103-3p	NM_001042485	-29.9	0.32874	-25.61	170	GO:0031224,	K08443|1|7e-08|60.8|cfa:484897|egf-like module containing, mucin-like, hormone receptor-like 2	gi|148707669|gb|EDL39616.1|	0	RIKEN cDNA 1300007B12, isoform CRA_a [Mus musculus]
mmu-miR-103-3p	NM_001044308	-33.5	0.274092	-68.31	466	GO:0034704,GO:0005488,GO:0005245,GO:0006935,GO:0032412,GO:0023052,GO:0070838,	K04856|1|0.0|3385|mmu:239556|voltage-dependent calcium channel T type alpha-1I	gi|157057194|ref|NP_001037773.2|	0	calcium channel, voltage-dependent, alpha 1I subunit [Mus musculus]

mmu-miR-103-3p	NM_001045536	-30	0.744678	-49.32	333	GO:0000152,GO:0046914,GO:0007088,	K12233|1|7e-20|102|bfo:BRAFLDRAFT_276868|E3 ubiquitin-protein ligase HECTD3 [EC:6.3.2.19]!K11970|2|1e-19|101|xtr:100145662|p53-associated parkin-like cytoplasmic protein!K10613|3|1e-19|101|mcc:699545|cullin 7	gi|113865905|ref|NP_001039001.1|	0	zinc finger ZZ-type and EF-hand domain-containing protein 1 [Mus musculus]
mmu-miR-103-3p	NM_001048267	-34	0.127224	-26.32	171	GO:0043231,GO:0044444,GO:0005048,GO:0022892,GO:0006606,GO:0051704,GO:00160	K14293|1|1e-16|90.5|cqu:CpipJ_CPIJ004613|importin subunit beta-1	gi|115385968|ref|NP_848831.2|	0	transportin-1 isoform 1 [Mus musculus]

mmu-miR-103-3p	NM_001079848	-29.2	0.551842	-25.44	169	GO:0044459,GO:0044424,GO:0031224,GO:0004888,GO:0007186,	K08451|1|0.0|1601|mmu:237175|G protein-coupled receptor 64	gi|148708858|gb|EDL40805.1|	0	G protein-coupled receptor 64 [Mus musculus]
mmu-miR-103-3p	NM_001079876	-28.3	0.809017	-25.04	153	-	K10382|1|1e-08|64.3|spu:756550|dystonin	gi|120407058|ref|NP_001028503.2|	0	GAS2-like protein 3 [Mus musculus]
mmu-miR-103-3p	NM_001081017	-28.2	0.889931	-46.17	319	GO:0044464,	-	gi|313661493|ref|NP_001074486.2|	0	protein unc-79 homolog [Mus musculus]
mmu-miR-103-3p	NM_001081083	-26.9	0.735959	-50.23	325	-	K02105|1|4e-07|58.2|tsp:Tsp_08140|catenin beta 1!K10056|2|3e-06|55.5|xla:398496|junction plakoglobin	gi|124487327|ref|NP_001074552.1|	0	armadillo repeat-containing protein 3 [Mus musculus]
mmu-miR-103-3p	NM_001081158	-27.4	0.853634	-45.16	311	-	K03255|1|0.0|2624|mmu:74148|protein TIF31	gi|124487201|ref|NP_001074627.1|	0	protein KIAA0664 [Mus musculus]

mmu-miR-103-3p	NM_001081191	-26.4	0.992691	-45.59	311	-	K15420|1|2e-99|366|tgu:100219108|echinoderm microtubule-associated protein-like 4	gi|124487111|ref|NP_001074660.1|	0	echinoderm microtubule-associated protein-like 5 [Mus musculus]
mmu-miR-103-3p	NM_001081192	-28.9	0.521194	-41.29	319	GO:0033267,GO:0016020,GO:0005221,GO:0000166,GO:0005267,GO:0015672,GO:0043009,GO:0051969,GO:0003012,GO:0003013,GO:0034220,GO:0030001,	K04957|1|0.0|1503|mmu:330953|hyperpolarization activated cyclic nucleotide-gated potassium channel 4	gi|124487125|ref|NP_001074661.1|	0	potassium/sodium hyperpolarization-activated cyclic nucleotide-gated channel 4 [Mus musculus]
mmu-miR-103-3p	NM_001081315	-25.2	0.993642	-47.88	315	GO:0070775,GO:0044444,GO:0046914,GO:0001775,GO:0006887,GO:0007599,GO:0016573,	K11350|1|0.0|2230|mmu:268936|bromodomain and PHD finger-containing protein 3	gi|124486783|ref|NP_001074784.1|	0	bromodomain and PHD finger-containing protein 3 [Mus musculus]

mmu-miR-103-3p	NM_001081326	-25.1	0.999568	-76.13	471	GO:0043234,GO:0031981,GO:0044444,GO:0043130,GO:0001871,GO:0004133,GO:0016740,GO:0043167,GO:0016787,GO:0019318,GO:0005977,	K01196|1|0.0|3115|mmu:77559|glycogen debranching enzyme [EC:2.4.1.25 3.2.1.33]	gi|124486747|ref|NP_001074795.1|	0	glycogen debranching enzyme [Mus musculus]
mmu-miR-103-3p	NM_001081418	-29.9	0.503645	-46.42	330	GO:0005488,	K05626|1|6e-06|55.1|rno:29515|atrophin-1 (dentatorubral-pallidoluysian atrophy protein)	gi|124487237|ref|NP_001074887.1|	0	glioma tumor suppressor candidate region gene 1 protein [Mus musculus]
mmu-miR-103-3p	NM_001081437	-28.2	0.67519	-70.27	484	GO:0031012,GO:0046872,GO:0005198,GO:0010810,	K06825|1|1e-74|282|xtr:100490545|fibrillin 1	gi|124517693|ref|NP_001074906.1|	0	fibulin-2 isoform b precursor [Mus musculus]

mmu-miR-103-3p	NM_001083807	-32.2	0.151794	-27.87	172	GO:0031981,GO:0030674,	K15691|1|2e-11|72.4|mgp:100545594|E3 ubiquitin-protein ligase RFWD3 [EC:6.3.2.19]	gi|139947575|ref|NP_001077276.1|	0	RUN and SH3 domain-containing protein 1 isoform 2 [Mus musculus]
mmu-miR-103-3p	NM_001083935	-28.2	0.426925	-25.3	156	GO:0043231,GO:0016021,GO:0005903,GO:0044459,GO:0004930,GO:0048731,GO:0001649,GO:0008283,GO:0021700,GO:0051480,GO:0007188,GO:0001894,	K04585|1|0.0|1130|mmu:19228|parathyroid hormone receptor 1	gi|148677056|gb|EDL09003.1|	0	parathyroid hormone receptor 1, isoform CRA_a [Mus musculus]

mmu-miR-103-3p	NM_001083936	-28.2	0.432202	-25.3	156	GO:0043231,GO:0016021,GO:0005903,GO:0044459,GO:0004930,GO:0048731,GO:0001649,GO:0008283,GO:0021700,GO:0051480,GO:0007188,GO:0001894,	K04585|1|0.0|1130|mmu:19228|parathyroid hormone receptor 1	gi|148677056|gb|EDL09003.1|	0	parathyroid hormone receptor 1, isoform CRA_a [Mus musculus]
mmu-miR-103-3p	NM_001085499	-30.1	0.205024	-25.24	168	-	-	gi|148225760|ref|NP_001078968.1|	0	uncharacterized protein LOC209005 [Mus musculus]
mmu-miR-103-3p	NM_001097623	-30.3	0.395559	-26.04	164	GO:0043231,GO:0046872,GO:0001071,GO:0003677,GO:0005515,GO:0003713,GO:0065008,GO:0006355,GO:0032501,GO:00082	K11829|1|4e-10|68.9|xla:398247|REST corepressor 1	gi|148271107|ref|NP_001091092.1|	0	transcriptional-regulating factor 1 isoform 1 [Mus musculus]
mmu-miR-103-3p	NM_001100452	-31.5	0.36755	-28.06	175	-	-	gi|226958520|ref|NP_001093922.1|	0	uncharacterized protein KIAA0240 [Mus musculus]

mmu-miR-103-3p	NM_001101486	-26.4	0.455095	-49.55	322	-	-	gi|162138899|ref|NP_001094956.1|	3.12E-145	uncharacterized protein LOC245884 [Mus musculus]
mmu-miR-103-3p	NM_001103162	-29.3	0.49652	-47.01	324	GO:0032991,GO:0030134,GO:0031224,GO:0031090,GO:0012505,GO:0005515,GO:0032934,GO:0016125,GO:0006633,GO:0043434,GO:0010894,GO:0010870,GO:00325	K10260|1|6e-17|91.3|pon:100173626|F-box and WD-40 domain protein 7	gi|148677071|gb|EDL09018.1|	0	SREBP cleavage activating protein, isoform CRA_a [Mus musculus]

mmu-miR-103-3p	NM_001111030	-31.8	0.430549	-26.23	175	GO:0044424,GO:0031224,GO:0043235,GO:0070696,GO:0032559,GO:0043169,GO:0017002,GO:0032403,GO:0007179,GO:0006796,GO:0022602,GO:0050796,GO:0009855,GO:0021543,GO:0043434,GO:0006915,GO:0048869,GO:0006464,GO:0009746,GO:0022037,GO:0001832,GO:00108	K13568|1|0.0|951|rno:245921|activin receptor type-1C [EC:2.7.11.30]	gi|161333831|ref|NP_001104500.1|	0	activin receptor type-1C isoform 1 precursor [Mus musculus]

mmu-miR-103-3p	NM_001111272	-25.5	0.890588	-43.84	323	GO:0031981,GO:0015630,GO:0003723,GO:0051234,	K13194|1|2e-06|55.8|nve:NEMVE_v1g189235|double stranded RNA-specific editase B [EC:3.5.-.-]	gi|162287083|ref|NP_001104742.1|	0	double-stranded RNA-binding protein Staufen homolog 2 isoform 2 [Mus musculus]
mmu-miR-103-3p	NM_001112729	-28.4	0.701221	-25.27	162	-	-	gi|31542190|ref|NP_659176.2|	0	TELO2-interacting protein 2 isoform a [Mus musculus]
mmu-miR-103-3p	NM_001128145	-27.9	0.580795	-50.59	334	GO:0044464,GO:0002360,GO:0001816,	K06545|1|0.0|679|aml:100470523|CD163	gi|189458795|ref|NP_001121617.1|	0	scavenger receptor protein family member isoform 1 precursor [Mus musculus]
mmu-miR-103-3p	NM_001128146	-29.6	0.381179	-50.59	334	GO:0002360,GO:0001816,	K06545|1|0.0|688|cfa:477704|CD163 antigen	gi|189458797|ref|NP_001121618.1|	0	scavenger receptor protein family member isoform 2 precursor [Mus musculus]
mmu-miR-103-3p	NM_001134385	-30.6	0.175998	-45.96	316	GO:0031224,GO:0004888,GO:0023052,	K08438|1|2e-172|606|mmu:71862|G protein-coupled receptor 160	gi|197304666|ref|NP_001127857.1|	1.47E-171	probable G-protein coupled receptor 160 [Mus musculus]

mmu-miR-103-3p	NM_001134386	-30.6	0.129261	-45.96	316	GO:0031224,GO:0004888,GO:0023052,	K08438|1|2e-172|606|mmu:71862|G protein-coupled receptor 160	gi|197304666|ref|NP_001127857.1|	1.05E-171	probable G-protein coupled receptor 160 [Mus musculus]
mmu-miR-103-3p	NM_001136237	-31.7	0.097077	-26.57	167	GO:0044459,GO:0046915,GO:0072503,GO:0000041,GO:0009267,	K14711|1|0.0|756|mmu:72002|solute carrier family 39 (zinc transporter), member 5	gi|148692617|gb|EDL24564.1|	0	solute carrier family 39 (metal ion transporter), member 5, isoform CRA_b [Mus musculus]
mmu-miR-103-3p	NM_001141921	-26.5	0.741422	-26.58	165	GO:0044459,GO:0031224,GO:0044430,	K07523|1|6e-30|133|xtr:100188921|netrin-G1 ligand	gi|213972562|ref|NP_001135393.1|	0	leucine-rich repeat and fibronectin type III domain-containing protein 1 isoform 1 precursor [Mus musculus]
mmu-miR-103-3p	NM_001146180	-31.3	0.306054	-26.02	161	GO:0005856,GO:0031252,GO:0016023,GO:0030674,GO:0003779,GO:0019904,GO:0030035,GO:0007167,GO:0008154,GO:00485	K05627|1|3e-11|72.8|mgp:100550206|BAI1-associated protein 2	gi|37359942|dbj|BAC97949.1|	0	mKIAA0429 protein [Mus musculus]

mmu-miR-103-3p	NM_001146215	-28.9	0.398108	-25.13	152	GO:0043234,GO:0000794,GO:0003690,GO:0042623,GO:0032559,GO:0007276,GO:0007135,GO:0006310,GO:0009207,GO:0006298,GO:0007127,	K08741|1|0.0|1495|mmu:17687|DNA mismatch repair protein MSH5	gi|226246613|ref|NP_038628.2|	0	mutS protein homolog 5 [Mus musculus]
mmu-miR-103-3p	NM_001159626	-33.1	0.024162	-26.33	164	GO:0044429,GO:0016790,GO:0046914,GO:0006749,GO:0048232,	K01069|1|3e-151|535|rno:24439|hydroxyacylglutathione hydrolase [EC:3.1.2.6]	gi|257051017|sp|Q99KB8.2|GLO2_MOUSE	4.22E-154	RecName: Full=Hydroxyacylglutathione hydrolase, mitochondrial; AltName: Full=Glyoxalase II; Short=Glx II; Flags: Precursor
mmu-miR-103-3p	NM_001159889	-28.4	0.547452	-25.19	161	GO:0043231,	-	gi|229577358|ref|NP_001153360.1|	1.56E-106	OCIA domain-containing protein 1 isoform 3 [Mus musculus]

mmu-miR-103-3p	NM_001160316	-26.1	0.994428	-43.7	308	GO:0031224,GO:0033267,GO:0005911,GO:0008366,GO:0043062,GO:0007043,GO:0006935,GO:0034613,	K06757|1|0.0|2359|mmu:269116|neurofascin	gi|237858630|ref|NP_001153788.1|	0	neurofascin isoform 2 precursor [Mus musculus]
mmu-miR-103-3p	NM_001160317	-26.1	0.994884	-43.7	308	GO:0031224,GO:0033267,GO:0005911,GO:0005488,GO:0008366,GO:0043062,GO:0007043,GO:0006935,GO:0034613,	K06757|1|0.0|2343|ptr:457660|neurofascin	gi|37360078|dbj|BAC98017.1|	0	mKIAA0756 protein [Mus musculus]
mmu-miR-103-3p	NM_001160318	-26.1	0.994996	-43.7	308	GO:0031224,GO:0033267,GO:0005911,GO:0008366,GO:0043062,GO:0007043,GO:0006935,GO:0034613,	K06757|1|0.0|2289|ptr:457660|neurofascin	gi|37360078|dbj|BAC98017.1|	0	mKIAA0756 protein [Mus musculus]

mmu-miR-103-3p	NM_001160319	-27.1	0.995549	-63.1	459	GO:0043232,GO:0043231,GO:0031224,GO:0046914,GO:0005515,GO:0019787,GO:0051704,GO:0032446,	K10691|1|0.0|9307|mmu:69116|E3 ubiquitin-protein ligase UBR4 [EC:6.3.2.19]	gi|237820660|ref|NP_001153791.1|	0	E3 ubiquitin-protein ligase UBR4 [Mus musculus]
mmu-miR-103-3p	NM_001162489	-25.7	0.973175	-41.25	314	-	-	gi|28972856|dbj|BAC65844.1|	0	mKIAA1833 protein [Mus musculus]
mmu-miR-103-3p	NM_001162913	-27.5	0.630186	-47.53	313	GO:0031224,GO:0001871,GO:0004888,GO:0023052,GO:0002376,	K04005|1|3e-07|58.5|rno:54243|complement receptor related protein!K04012|2|3e-07|58.5|rno:289395|complement receptor type 2!K10955|3|1e-06|56.2|xtr:100493286|intestinal mucin-	gi|251823826|ref|NP_001156385.1|	0	sushi domain-containing protein 2 isoform 2 precursor [Mus musculus]

mmu-miR-103-3p	NM_001163487	-28.2	0.522616	-46.97	311	GO:0043234,GO:0044459,GO:0044444,GO:0032559,GO:0008443,GO:0043169,GO:0070061,GO:0019899,GO:0006007,GO:0006000,GO:0016310,GO:0050796,GO:0019725,GO:0051260,GO:0042593,	K00850|1|0.0|1537|mmu:18642|6-phosphofructokinase [EC:2.7.1.11]	gi|332206389|ref|XP_003252273.1|	0	PREDICTED: 6-phosphofructokinase, muscle type isoform 2 [Nomascus leucogenys]

mmu-miR-103-3p	NM_001163488	-29.6	0.328782	-46.97	311	GO:0043234,GO:0044459,GO:0044444,GO:0032559,GO:0008443,GO:0043169,GO:0070061,GO:0019899,GO:0006007,GO:0006000,GO:0016310,GO:0050796,GO:0019725,GO:0051260,GO:0042593,	K00850|1|0.0|1537|mmu:18642|6-phosphofructokinase [EC:2.7.1.11]	gi|254553344|ref|NP_067489.3|	0	6-phosphofructokinase, muscle type [Mus musculus]
mmu-miR-103-3p	NM_001163752	-33.6	0.056914	-27.16	168	-	-	gi|255522949|ref|NP_001157224.1|	0	uncharacterized protein C19orf57 homolog isoform 2 [Mus musculus]
mmu-miR-103-3p	NM_001164233	-32	0.099486	-26.5	174	GO:0016021,GO:0008509,GO:0006820,	K14357|1|3e-133|476|mmu:67854|solute carrier organic anion transporter family, member 6A	gi|255982577|ref|NP_001157705.1|	0	solute carrier organic anion transporter family, member 6d1 isoform 2 [Mus musculus]

mmu-miR-103-3p	NM_001164572	-26.1	0.94583	-30.08	166	GO:0043231,GO:0004672,GO:0046872,GO:0032559,GO:0042981,GO:0006796,GO:0030097,GO:0006464,	K08802|1|0.0|1256|mmu:20623|SNF related kinase [EC:2.7.11.1]	gi|223462828|gb|AAI50743.1|	0	SNF related kinase [Mus musculus]
mmu-miR-103-3p	NM_001166501	-25.6	0.996409	-43.34	304	GO:0030135,GO:0030695,	K01289|1|8e-67|258|dme:Dmel_CG18659|carboxypeptidase [EC:3.4.16.-]!K12861|2|4e-29|132|gga:419884|pre-mRNA-splicing factor SPF27	gi|262073029|ref|NP_001159973.1|	0	DENN domain-containing protein 1B isoform 1 [Mus musculus]
mmu-miR-103-3p	NM_001166506	-26.7	0.895048	-44.05	320	GO:0031224,GO:0043231,GO:0051234,	-	gi|148702667|gb|EDL34614.1|	0	SEC14-like 1 (S. cerevisiae), isoform CRA_c [Mus musculus]
mmu-miR-103-3p	NM_001166507	-26.7	0.876897	-44.05	320	GO:0031224,GO:0043231,GO:0051234,	-	gi|148702667|gb|EDL34614.1|	0	SEC14-like 1 (S. cerevisiae), isoform CRA_c [Mus musculus]

mmu-miR-103-3p	NM_001170552	-28.3	0.774714	-25.13	158	GO:0030054,GO:0032991,GO:0043232,GO:0005488,GO:0030695,GO:0001525,GO:0035556,GO:0043393,GO:0019725,GO:0030010,GO:0001935,GO:0010594,GO:0043066,	K04984|1|3e-11|72.8|gga:420180|transient receptor potential cation channel subfamily A member 1!K08848|2|3e-11|72.8|gga:418529|receptor-interacting serine/threonine-protein kinase 4 [EC:2.7.11.1]!K06694|4|6e-11|72.0|mgp:10054	gi|148682673|gb|EDL14620.1|		0	KRIT1, ankyrin repeat containing, isoform CRA_c [Mus musculus]
mmu-miR-103-3p	NM_001177977	-26	0.982084	-25.43	149	GO:0044429,GO:0043169,GO:0016878,GO:0032559,GO:0032787,GO:0044238,	K01896|1|0.0|1230|mmu:233799|medium-chain acyl-CoA synthetase [EC:6.2.1.2]	gi|295789125|ref|NP_001171448.1|		0	acyl-coenzyme A synthetase ACSM2, mitochondrial isoform 1 [Mus musculus]

mmu-miR-103-3p	NM_001177978	-26	0.981368	-25.43	149	GO:0044429,GO:0043169,GO:0016878,GO:0032559,GO:0032787,GO:0044238,	K01896|1|0.0|1175|mmu:233799|medium-chain acyl-CoA synthetase [EC:6.2.1.2]	gi|295789127|ref|NP_001171449.1|	0	acyl-coenzyme A synthetase ACSM2, mitochondrial isoform 2 [Mus musculus]
mmu-miR-103-3p	NM_001195089	-26.1	0.811908	-46.34	317	GO:0031224,GO:0043231,	-	gi|304434590|ref|NP_001182017.1|	0	transmembrane channel-like protein 8 isoform A [Mus musculus]
mmu-miR-103-3p	NM_001195090	-26.1	0.811554	-46.34	317	GO:0031224,GO:0043231,	-	gi|304434590|ref|NP_001182017.1|	0	transmembrane channel-like protein 8 isoform A [Mus musculus]
mmu-miR-103-3p	NM_001199113	-32.5	0.076841	-28.35	168	GO:0016021,GO:0044459,GO:0005488,GO:0015932,GO:0001666,GO:0015864,GO:0051899,GO:0001678,GO:0007589,GO:0006139,GO:0007610,	K15014|1|0.0|885|mmu:63959|solute carrier family 29 (equilibrative nucleoside transporter), member 1/2/3	gi|74226755|dbj|BAE27024.1|	0	unnamed protein product [Mus musculus]

mmu-miR-103-3p	NM_001199114	-32.5	0.069448	-28.35	168	GO:0016021,GO:0044459,GO:0005488,GO:0015932,GO:0001666,GO:0015864,GO:0051899,GO:0001678,GO:0007589,GO:0006139,GO:0007610,	K15014|1|0.0|885|mmu:63959|solute carrier family 29 (equilibrative nucleoside transporter), member 1/2/3	gi|74226755|dbj|BAE27024.1|	0	unnamed protein product [Mus musculus]
mmu-miR-103-3p	NM_001199115	-32.5	0.074038	-28.35	168	GO:0016021,GO:0044459,GO:0005488,GO:0015932,GO:0001666,GO:0015864,GO:0051899,GO:0001678,GO:0007589,GO:0006139,GO:0007610,	K15014|1|0.0|874|mmu:63959|solute carrier family 29 (equilibrative nucleoside transporter), member 1/2/3	gi|12584968|ref|NP_075018.1|	0	equilibrative nucleoside transporter 1 isoform 2 [Mus musculus]

mmu-miR-103-3p	NM_001199116	-32.5	0.072201	-28.35	168	GO:0016021,GO:0044459,GO:0005488,GO:0015932,GO:0001666,GO:0015864,GO:0051899,GO:0001678,GO:0007589,GO:0006139,GO:0007610,	K15014|1|0.0|874|mmu:63959|solute carrier family 29 (equilibrative nucleoside transporter), member 1/2/3	gi|12584968|ref|NP_075018.1|	0	equilibrative nucleoside transporter 1 isoform 2 [Mus musculus]
mmu-miR-103-3p	NM_001199431	-30.5	0.424064	-27.12	159	GO:0000785,GO:0032991,GO:0043231,GO:0000775,GO:0046914,GO:0000166,GO:0003886,GO:0043565,GO:0043566,GO:0019899,GO:0006575,GO:0006355,GO:0008283,GO:0031062,GO:0032776,GO:0006790,GO:00	K00558|1|0.0|2940|mmu:13433|DNA (cytosine-5-)-methyltransferase [EC:2.1.1.37]	gi|148693194|gb|EDL25141.1|	0	DNA methyltransferase (cytosine-5) 1, isoform CRA_c [Mus musculus]

mmu-miR-103-3p	NM_001199988	-28.4	0.695488	-25.27	162	-	-	gi|316983174|ref|NP_001186917.1|	0	TELO2-interacting protein 2 isoform b [Mus musculus]
mmu-miR-103-3p	NM_001204152	-27.6	0.521266	-25.21	160	-	K01227|1|1e-33|145|acs:100563201|mannosyl-glycoprotein endo-beta-N-acetylglucosaminidase [EC:3.2.1.96]!K07296|2|1e-13|76.3|bta:282865|adiponectin	gi|148540036|ref|NP_082607.3|	3.49E-59	complement C1q tumor necrosis factor-related protein 6 isoform 1 precursor [Mus musculus]

mmu-miR-103-3p	NM_001204153	-27.6	0.510935	-25.21	160	-	K01227|1|1e-33|145|acs:100563201|mannosyl-glycoprotein endo-beta-N-acetylglucosaminidase [EC:3.2.1.96]!K07296|2|3e-12|74.7|bta:282865|adiponectin	gi|148540036|ref|NP_082607.3|	2.20E-58	complement C1q tumor necrosis factor-related protein 6 isoform 1 precursor [Mus musculus]
mmu-miR-103-3p	NM_001204411	-35.3	0.032266	-28.59	163	GO:0043231,GO:0042383,GO:0005887,GO:0005515,GO:0031420,GO:0032559,GO:0005242,GO:0015980,GO:0050796,GO:0048545,GO:0071805,GO:0019318,GO:0006873,GO:00	K05004|1|8e-174|611|rno:83535|potassium inwardly-rectifying channel subfamily J member 11	gi|26328501|dbj|BAC27989.1|	8.77E-174	unnamed protein product [Mus musculus]

mmu-miR-103-3p	NM_001252659	-25.7	0.961541	-25.15	161	GO:0016021,GO:0000323,GO:0009986,GO:0034358,GO:0030665,GO:0044459,GO:0010008,GO:0030228,GO:0030169,GO:0046872,GO:0016125,GO:0034381,GO:0043627,GO:0042157,GO:0023052,GO:0043434,GO:0030301,GO:0006897,GO:0051704,GO:0032502,GO	K12473|1|0.0|1767|mmu:16835|low-density lipoprotein receptor	gi|358030304|ref|NP_001239588.1|	0	low-density lipoprotein receptor isoform 3 presursor precursor [Mus musculus]

mmu-miR-103-3p	NM_001253795	-29.5	0.181227	-50.06	329	GO:0043231,GO:0031332,GO:0044444,GO:0042802,GO:0003723,GO:0016246,GO:0033673,GO:0006412,GO:0006355,GO:0050687,GO:0035196,GO:0045069,	-	gi|359385734|ref|NP_001240724.1|	1.41E-135	RISC-loading complex subunit TARBP2 isoform 2 [Mus musculus]
mmu-miR-103-3p	NM_001254953	-33.1	0.031557	-47.95	327	-	K01395|1|1e-58|227|ptr:462745|meprin A, alpha [EC:3.4.24.18]!K15503|2|9e-13|75.5|tgu:100228130|serine/threonine-protein phosphatase 6 regulatory ankyrin repeat subunit B	gi|363807402|ref|NP_001241882.1|	7.51E-111	predicted gene 4368 [Mus musculus]
mmu-miR-103-3p	NM_001271563	-26.9	0.731629	-50.23	325	-	-	-	-	-
mmu-miR-103-3p	NM_001271564	-26.9	0.730168	-50.23	325	-	-	-	-	-
mmu-miR-103-3p	NM_001271565	-26.9	0.711005	-50.23	325	-	-	-	-	-

mmu-miR-103-3p	NM_007432	-28.8	0.263789	-25.47	155	GO:0009986,GO:0031224,GO:0046914,GO:0016791,GO:0009653,GO:0007399,GO:0040007,GO:0006796,	K01077|1|0.0|969|mmu:11648|alkaline phosphatase [EC:3.1.3.1]	gi|110347479|ref|NP_031458.2|	0	intestinal-type alkaline phosphatase precursor [Mus musculus]
mmu-miR-103-3p	NM_007645	-29.6	0.133314	-25.87	159	GO:0031224,GO:0006959,GO:0002377,	K06475|1|3e-126|452|mmu:12493|CD37 antigen	gi|74181799|dbj|BAE32606.1|	1.96E-126	unnamed protein product [Mus musculus]
mmu-miR-103-3p	NM_007789	-33	0.243482	-28.5	163	GO:0016020,GO:0046872,GO:0005539,GO:0050807,GO:0006935,	K06794|1|0.0|2318|mmu:13004|chondroitin sulfate proteoglycan 3 (neurocan)	gi|40789268|ref|NP_031815.2|	0	neurocan core protein precursor [Mus musculus]

mmu-miR-103-3p	NM_007865	-26.3	0.859412	-26.78	173	GO:0016021,GO:0031982,GO:0046872,GO:0005102,GO:0042476,GO:0045607,GO:0042471,GO:0044267,GO:0045165,GO:0048534,GO:0007155,GO:0009855,GO:0001756,GO:00	K06051|1|0.0|1481|mmu:13388|delta	gi|40789272|ref|NP_031891.2|	0	delta-like protein 1 precursor [Mus musculus]
mmu-miR-103-3p	NM_007930	-25.8	0.961467	-40.86	305	GO:0043232,GO:0034399,GO:0008092,GO:0048731,	K10462|1|0.0|1164|mmu:13803|kelch-like protein 25/37 (ectoderm-neural cortex protein)	gi|40254359|ref|NP_031956.3|	0	ectoderm-neural cortex protein 1 [Mus musculus]
mmu-miR-103-3p	NM_007992	-28.2	0.686826	-70.27	484	GO:0031012,GO:0046872,GO:0005198,GO:0010810,	K06825|1|1e-76|290|mgp:723984|fibrillin 1	gi|124517704|ref|NP_032018.2|	0	fibulin-2 isoform a precursor [Mus musculus]

mmu-miR-103-3p	NM_008344	-27.1	0.384677	-26.63	156	GO:0044421,GO:0043231,GO:0005520,GO:0008283,GO:0016049,GO:0023052,	K10138|1|4e-29|129|acs:100565907|insulin-like growth factor-binding protein 3	gi|168693654|ref|NP_032370.2|	3.79E-123	insulin-like growth factor-binding protein 6 precursor [Mus musculus]
mmu-miR-103-3p	NM_008482	-33	0.197368	-29.8	166	GO:0043256,GO:0044444,GO:0005198,GO:0016477,GO:0030030,GO:0006935,GO:0009887,GO:0008284,GO:0000904,	K05636|1|0.0|3679|mmu:16777|laminin, beta 1	gi|148704971|gb|EDL36918.1|	0	laminin B1 subunit 1 [Mus musculus]
mmu-miR-103-3p	NM_008667	-31.5	0.29483	-26.95	159	GO:0043231,GO:0016020,GO:0008366,GO:0001503,GO:2000026,GO:0006355,GO:00074	-	gi|31543307|ref|NP_032693.2|	0	NGFI-A-binding protein 1 [Mus musculus]
mmu-miR-103-3p	NM_008733	-33.3	0.16967	-27.81	171	-	-	gi|117606368|ref|NP_032759.3|	0	nebulin-related-anchoring protein isoform S [Mus musculus]

mmu-miR-103-3p	NM_008925	-25.5	0.802881	-45.37	307	GO:0044432,GO:0043234,GO:0016020,GO:0016772,GO:0003676,GO:0046872,GO:0019901,GO:0006952,GO:0035556,GO:0051259,GO:0006796,GO:0001822,GO:00064	K08288|1|0.0|817|mmu:19089|protein kinase C substrate 80K-H	gi|6679465|ref|NP_032951.1|	0	glucosidase 2 subunit beta precursor [Mus musculus]
mmu-miR-103-3p	NM_008984	-30.4	0.449208	-25.42	163	GO:0005911,GO:0016021,GO:0031252,GO:0044444,GO:0050839,GO:0004871,GO:0004725,GO:0007411,GO:0001525,GO:0010594,GO:0001935,GO:0016337,GO:0023052,GO:0006464,GO:0003407,	K05693|1|0.0|2887|mmu:19274|protein tyrosine phosphatase, receptor type, M [EC:3.1.3.48]	gi|226054321|ref|NP_033010.2|	0	receptor-type tyrosine-protein phosphatase mu precursor [Mus musculus]

mmu-miR-103-3p	NM_001042699	-23.3	0.999954	-67.41	481	GO:0031965,GO:0034992,GO:0031224,GO:0008092,GO:0007010,	K10352|1|8e-07|58.2|xtr:448762|myosin heavy chain!K10366|2|1e-06|57.8|bta:537655|dystrophin!K10362|3|1e-06|57.4|bta:540437|myosin XVIII	gi|111607453|ref|NP_001036164.1|	0	nesprin-3 isoform alpha [Mus musculus]
mmu-miR-105	NM_001001496	-27.7	0.489017	-52.43	306	GO:0005921,GO:0031224,GO:0009653,GO:0009888,GO:0007043,GO:0007507,GO:0050789,	K07615|1|9e-140|498|mmu:414089|gap junction protein, alpha 6!K07372|3|2e-84|314|gga:395278|gap junction protein, alpha 1	gi|47894401|ref|NP_001001496.1|	6.61E-139	gap junction alpha-6 protein [Mus musculus]
mmu-miR-105	NM_001005424	-27.9	0.774008	-25.55	165	-	-	gi|148762971|ref|NP_001005424.2|	0	uncharacterized protein C9orf172 homolog [Mus musculus]

mmu-miR-105	NM_001008423	-26.1	0.943888	-29.35	168	-	-	gi|119601406|gb|EAW81000.1|	2.12E-66	hCG1644165 [Homo sapiens]
mmu-miR-105	NM_001011760	-28.8	0.185588	-26.66	151	GO:0016020,GO:0004872,GO:0023052,	K04257|1|2e-164|578|mmu:257917|olfactory	gi|58801308|ref|NP_001011760.1|	1.37E-163	olfactory receptor 314 [Mus musculus]
mmu-miR-105	NM_001012402	-26.9	0.419216	-46.8	313	GO:0031224,GO:0034483,	K09679|1|8e-168|590|mmu:328779|[heparan sulfate]-glucosamine 3-sulfotransferase 6 [EC:2.8.2.-	gi|60223081|ref|NP_001012402.1|	5.14E-167	heparan sulfate glucosamine 3-O-sulfotransferase 6 [Mus musculus]
mmu-miR-105	NM_001013256	-26.9	0.863164	-47.23	305	GO:0000783,GO:0043566,GO:0006259,	-	gi|219689064|ref|NP_001013274.2|	0	CST complex subunit CTC1 isoform a [Mus musculus]
mmu-miR-105	NM_001013749	-25.2	0.98903	-25.45	150	GO:0031224,	-	gi|85701444|ref|NP_001013771.1|	0	transmembrane protein 151B [Mus musculus]

mmu-miR-105	NM_001025312	-32.6	0.177477	-26.63	155	GO:0005815,GO:0043231,GO:0044427,GO:0005488,GO:0004527,GO:0007050,GO:0016233,GO:0033554,	K15341|1|0.0|840|mmu:140917|DNA cross-link repair 1B protein [EC:3.1.-.-]	gi|26351057|dbj|BAC39165.1|	0	unnamed protein product [Mus musculus]
mmu-miR-105	NM_001025568	-27.9	0.929772	-50.85	311	GO:0044297,GO:0044444,GO:0047555,GO:0043169,GO:0005515,GO:0016208,GO:0007606,GO:0050796,GO:0006195,GO:0010038,GO:0010863,GO:0007169,	K13755|1|0.0|1171|mmu:18575|calcium/calmodulin-dependent 3',5'-cyclic nucleotide phosphodiesterase [EC:3.1.4.17]	gi|148672760|gb|EDL04707.1|	0	phosphodiesterase 1C, isoform CRA_f [Mus musculus]
mmu-miR-105	NM_001025589	-27.2	0.786393	-25.78	159	GO:0043231,GO:0000989,GO:0003676,GO:0001071,GO:0019899,GO:0006357,GO:0007264,	K08062|1|2e-137|491|mmu:19727|regulatory factor X-associated ankyrin-	gi|148696839|gb|EDL28786.1|	1.34E-151	regulatory factor X-associated ankyrin-containing protein, isoform CRA_b [Mus musculus]

mmu-miR-105	NM_001032413	-30.4	0.396134	-27.08	171	GO:0044459,GO:0031224,GO:0006910,	K06252|1|9e-67|256|bta:282654|tenascin	gi|148683402|gb|EDL15349.1|	0	RIKEN cDNA 3110045G13, isoform CRA_b [Mus musculus]
mmu-miR-105	NM_001032414	-30.4	0.390905	-27.08	171	GO:0044459,GO:0031224,GO:0006910,	K06252|1|9e-67|256|bta:282654|tenascin	gi|148683402|gb|EDL15349.1|	0	RIKEN cDNA 3110045G13, isoform CRA_b [Mus musculus]
mmu-miR-105	NM_001033275	-26.8	0.955365	-25.55	160	GO:0031224,GO:0008194,GO:0006493,	K13676|1|0.0|731|mmu:223827|UDP-xylose:glucoside alpha-1,3-xylosyltransferase [EC:2.4.2.-]	gi|152125897|sp|Q3UHH8.2|GXLT1_MOUSE	0	RecName: Full=Glucoside xylosyltransferase 1; AltName: Full=Glycosyltransferase 8 domain-containing protein 3
mmu-miR-105	NM_001033415	-28.8	0.561445	-25.74	155	GO:0031224,GO:0031090,GO:0043231,GO:0032501,	-	gi|85986585|ref|NP_001028587.1|	7.41E-113	protein shisa-3 homolog precursor [Mus musculus]
mmu-miR-105	NM_001033422	-29.7	0.687912	-27.55	158	GO:0000346,GO:0003676,GO:0006406,GO:0006396,	K12879|1|0.0|2694|mmu:331401|THO complex subunit 2	gi|125656163|ref|NP_001028594.1|	0	THO complex subunit 2 [Mus musculus]

mmu-miR-105	NM_001033430	-25.2	0.999614	-46.05	316	GO:0031981,GO:0032452,GO:0042393,GO:0046914,GO:0016706,GO:0016701,GO:0006355,GO:0007420,GO:0070076,	K11445|1|0.0|1731|mmu:338523|JmjC domain-containing histone demethylation protein 1D/E/F	gi|157909789|ref|NP_001028602.2|	0	lysine-specific demethylase 7 [Mus musculus]
mmu-miR-105	NM_001037754	-30.8	0.251335	-25.52	164	GO:0031981,GO:0044459,GO:0031252,GO:0042995,GO:0030674,GO:0019904,GO:0007265,GO:0030035,GO:0048667,GO:00071	K05627|1|0.0|896|mmu:108100|BAI1-associated protein 2	gi|83649713|ref|NP_001032843.1|	0	brain-specific angiogenesis inhibitor 1-associated protein 2 isoform c [Mus musculus]
mmu-miR-105	NM_001038610	-25.8	0.975008	-25.46	155	GO:0043234,GO:0031981,GO:0003676,GO:0001071,GO:0006357,GO:0008285,GO:00451	-	gi|51260954|gb|AAH78644.1|	0	Dach1 protein, partial [Mus musculus]

mmu-miR-105	NM_001038710	-30	0.735725	-52.34	328	GO:0043232,GO:0042995,GO:0030427,GO:0008092,GO:0048731,GO:0008277,GO:0030036,GO:0007268,GO:0007610,	K10370|1|0.0|646|mmu:50876|tropomodulin	gi|31543876|ref|NP_057920.2|	0	tropomodulin-2 [Mus musculus]

mmu-miR-105	NM_001039647	-25.8	0.873671	-25.07	156	GO:0005774,GO:0031982,GO:0001562,GO:0042742,GO:0034341,GO:0032496,GO:0035456,GO:0051702,	K10388|1|5e-07|57.8|mdo:100014955|plectin!K06669|2|3e-06|55.1|mcc:695123|structural maintenance of chromosome 3 (chondroitin sulfate proteoglycan 6)!K08840|3|5e-06|54.3|ecb:100057722|TRAF2 and NCK interacting kinase [EC:2.7.11.1]	gi|225007564|ref|NP_001034736.2|		0	guanylate binding protein 11 [Mus musculus]
mmu-miR-105	NM_001042611	-28.6	0.663801	-25.48	155	-	K13624|1|0.0|2231|mmu:12870|ceruloplasmin [EC:1.16.3.1]	gi|148702938|gb|EDL34885.1|		0	ceruloplasmin, isoform CRA_c [Mus musculus]

mmu-miR-105	NM_001081012	-27.7	0.788273	-25.19	156	-	K11499|1|3e-24|115|pon:100437688|centromere protein F	gi|124486602|ref|NP_001074481.1|	0	coiled-coil domain-containing protein 171 [Mus musculus]
mmu-miR-105	NM_001081044	-26.1	0.860845	-25.18	159	GO:0044430,GO:0044297,GO:0030016,GO:0030425,GO:0033267,GO:0030136,GO:0017022,GO:0004674,GO:0032559,GO:0006937,GO:0060415,GO:0048167,GO:0010627,GO:0016192,GO:0006468,GO:0031443,GO:0048522,GO:0007049,GO:00420	K00907|1|0.0|1063|mmu:228785|myosin-light-chain kinase [EC:2.7.11.18]	gi|163644275|ref|NP_001074513.2|	0	myosin light chain kinase 2, skeletal/cardiac muscle [Mus musculus]

mmu-miR-105	NM_001081102	-27.5	0.917616	-28.06	168	GO:0031981,GO:0043232,GO:0005635,GO:0003676,GO:0046914,GO:0016279,GO:0006357,GO:0003285,GO:0003284,GO:0006325,GO:0009653,	K11424|1|0.0|2639|mmu:107823|histone-lysine N-methyltransferase NSD2 [EC:2.1.1.43]	gi|295424164|ref|NP_001074571.2|	0	probable histone-lysine N-methyltransferase NSD2 isoform 1 [Mus musculus]
mmu-miR-105	NM_001081122	-29.5	0.350172	-26.63	159	GO:0005815,GO:0005819,GO:0005488,GO:0035556,GO:0000087,GO:0007051,GO:00069	K11498|1|9e-16|86.7|hsa:1062|centromeric protein E	gi|148689105|gb|EDL21052.1|	0	mCG116019, isoform CRA_b [Mus musculus]
mmu-miR-105	NM_001081158	-30.2	0.494236	-25.42	150	-	K03255|1|0.0|2624|mmu:74148|protein TIF31	gi|124487201|ref|NP_001074627.1|	0	protein KIAA0664 [Mus musculus]

mmu-miR-105	NM_001081242	-27.2	0.988842	-42.67	323	GO:0005856,GO:0005924,GO:0031252,GO:0008092,GO:0005102,GO:0005198,GO:0034329,GO:0007010,	K06271|1|0.0|4323|mmu:70549|talin	gi|163310736|ref|NP_001074711.2|	0	talin-2 [Mus musculus]
mmu-miR-105	NM_001081264	-35.3	0.044343	-30.17	176	GO:0031090,GO:0043231,GO:0031224,GO:0046527,GO:0006487,	K03848|1|0.0|956|mmu:320438|alpha-1,3-glucosyltransferase [EC:2.4.1.267]	gi|124486913|ref|NP_001074733.1|	0	dolichyl pyrophosphate Man9GlcNAc2 alpha-1,3-glucosyltransferase precursor [Mus musculus]

mmu-miR-105	NM_001081280	-25.9	0.945803	-48.01	318	GO:0044424,GO:0032559,GO:0007243,GO:0043433,GO:0046649,	K12800|1|2e-82|308|dre:561595|NACHT, LRR and PYD domains-containing protein 3!K10165|5|5e-49|197|mmu:257632|nucleotide-binding oligomerization domain-containing protein 2	gi|124486873|ref|NP_001074749.1|	0	protein NLRC3 [Mus musculus]
mmu-miR-105	NM_001081301	-28.2	0.194954	-25.74	152	GO:0031224,GO:0004871,GO:0007166,	K04257|1|3e-134|478|mmu:546896|olfactory	gi|240255445|ref|NP_001074770.2|	1.95E-133	olfactory receptor 455, pseudogene 1 [Mus musculus]
mmu-miR-105	NM_001081352	-27.5	0.862128	-25.54	150	-	K12600|1|0.0|3033|mmu:218343|superkiller protein 3	gi|124486883|ref|NP_001074821.1|	0	tetratricopeptide repeat protein 37 [Mus musculus]

mmu-miR-105	NM_001083342	-27.2	0.966891	-27.28	165	GO:0005635,GO:0031224,GO:0004888,GO:0055092,GO:0007166,GO:0006869,	-	gi|134053935|ref|NP_001076811.1|	0	patched domain-containing protein 2 [Mus musculus]
mmu-miR-105	NM_001083927	-27.5	0.853378	-47.31	293	GO:0043231,GO:0005488,GO:0048869,GO:0048232,GO:0006351,GO:0009653,GO:00071	K04497|1|0.0|1189|dre:30206|groucho	gi|28972780|dbj|BAC65806.1|	0	mKIAA1547 protein [Mus musculus]
mmu-miR-105	NM_001083928	-27.5	0.850617	-47.31	293	GO:0006351,GO:0007166,	K04497|1|0.0|1194|dre:30206|groucho	gi|28972780|dbj|BAC65806.1|	0	mKIAA1547 protein [Mus musculus]
mmu-miR-105	NM_001101502	-26.8	0.796235	-27.71	159	GO:0043231,GO:0005488,GO:0009653,GO:0033598,GO:0007179,GO:0009966,GO:0030855,GO:0007049,	-	gi|160333268|ref|NP_001094972.1|	0	zinc finger protein 703 isoform 1 [Mus musculus]
mmu-miR-105	NM_001110100	-32.6	0.233439	-27.76	157	GO:0043231,GO:0003676,GO:0006325,GO:0032501,GO:0010467,	-	gi|158534069|ref|NP_001103570.1|	0	protein BANP isoform 1 [Mus musculus]

mmu-miR-105	NM_001110508	-26.8	0.809281	-27.71	159	GO:0043231,GO:0005488,GO:0007049,GO:0033598,GO:0009966,GO:0030855,GO:0007179,GO:0016043,	-	gi|160333268|ref|NP_001094972.1|	0	zinc finger protein 703 isoform 1 [Mus musculus]
mmu-miR-105	NM_001111141	-27.6	0.522542	-26.11	162	GO:0016604,GO:0005488,	-	gi|162138940|ref|NP_001104611.1|	0	uncharacterized protein LOC244666 [Mus musculus]
mmu-miR-105	NM_001111304	-32.2	0.28635	-27.9	154	GO:0044464,GO:0005099,GO:0046872,GO:0032318,	K11837|1|4e-13|79.0|hsa:9098|ubiquitin carboxyl-terminal hydrolase 6/32 [EC:3.1.2.15]	gi|162329599|ref|NP_001104774.1|	0	TBC1 domain family member 9 isoform 1 [Mus musculus]
mmu-miR-105	NM_001113560	-34.2	0.012443	-30.64	166	GO:0044424,GO:0043169,GO:0016846,GO:0006790,GO:0006081,GO:0043066,GO:00442	K01759|1|5e-106|384|mmu:109801|lactoylglutathione lyase [EC:4.4.1.5]	gi|165932331|ref|NP_079650.3|	3.58E-105	lactoylglutathione lyase [Mus musculus]

mmu-miR-105	NM_001122594	-27	0.965788	-28.91	152	GO:0043231,GO:0016791,GO:0043169,GO:0006796,	K01090|1|0.0|2362|mmu:244650|protein phosphatase [EC:3.1.3.16]	gi|295054247|ref|NP_001116066.2|	0	PH domain leucine-rich repeat-containing protein phosphatase 2 [Mus musculus]
mmu-miR-105	NM_001129999	-28.6	0.843074	-41.32	312	GO:0005813,GO:0032991,GO:0042995,GO:0019900,GO:0007098,GO:0033036,	K12478|1|1e-30|137|mgp:100545401|early endosome antigen 1	gi|148674210|gb|EDL06157.1|	0	mCG5221 [Mus musculus]
mmu-miR-105	NM_001130000	-28.6	0.843008	-41.32	312	GO:0005813,GO:0032991,GO:0042995,GO:0019900,GO:0007098,GO:0033036,	K12478|1|1e-30|137|mgp:100545401|early endosome antigen 1	gi|148674210|gb|EDL06157.1|	0	mCG5221 [Mus musculus]
mmu-miR-105	NM_001143790	-26.9	0.862978	-47.23	305	GO:0000783,GO:0043566,GO:0006259,	-	gi|219689066|ref|NP_001137262.1|	0	CST complex subunit CTC1 isoform c [Mus musculus]
mmu-miR-105	NM_001145979	-28.9	0.457629	-28.08	164	GO:0032561,GO:0017111,GO:0009207,	K14416|1|2e-18|95.5|nvi:100116941|elongation factor 1 alpha-like protein	gi|148691529|gb|EDL23476.1|	0	GTP binding protein 2, isoform CRA_b [Mus musculus]

mmu-miR-105	NM_001146007	-31.8	0.213624	-26.88	177	GO:0044424,GO:0042802,GO:0046914,GO:0051259,	K10648|1|0.0|1000|mmu:319236|tripartite motif-containing protein 5 [EC:6.3.2.19]!K11999|3|7e-131|469|bta:505265|tripartite motif-containing protein	gi|225579155|ref|NP_783608.3|	0	tripartite motif-containing 12C [Mus musculus]
mmu-miR-105	NM_001146215	-29	0.412253	-25.2	159	GO:0043234,GO:0000794,GO:0003690,GO:0042623,GO:0032559,GO:0007276,GO:0007135,GO:0006310,GO:0009207,GO:0006298,GO:0007127,	K08741|1|0.0|1495|mmu:17687|DNA mismatch repair protein MSH5	gi|226246613|ref|NP_038628.2|	0	mutS protein homolog 5 [Mus musculus]

mmu-miR-105	NM_001146687	-29.6	0.488517	-26.8	164	GO:0044444,GO:0005886,GO:0016307,GO:0000166,GO:0046488,GO:0048667,GO:0006796,GO:0006935,	K00889|1|0.0|924|mmu:18717|1-phosphatidylinositol-4-phosphate 5-kinase [EC:2.7.1.68]	gi|39104624|dbj|BAC65601.2|	0	mKIAA0589 protein [Mus musculus]
mmu-miR-105	NM_001159288	-28.9	0.659039	-28.05	154	GO:0032559,GO:0046914,	-	gi|50510669|dbj|BAD32320.1|	0	mKIAA0852 protein [Mus musculus]
mmu-miR-105	NM_001159509	-30.1	0.378618	-43.3	310	GO:0044431,GO:0031410,GO:0005515,GO:0016772,GO:0007010,GO:0006796,GO:0023052,GO:0030100,	K10408|1|4e-95|350|tca:664324|dynein heavy chain, axonemal!K12804|2|3e-14|82.0|dre:571331|proline-serine-threonine phosphatase interacting protein 1	gi|7106381|ref|NP_035992.1|	0	protein kinase C and casein kinase substrate in neurons protein 2 [Mus musculus]

mmu-miR-105	NM_001159510	-30.1	0.374339	-43.3	310	GO:0044431,GO:0031410,GO:0005515,GO:0016772,GO:0007010,GO:0006796,GO:0023052,GO:0030100,	K10408|1|4e-95|350|tca:664324|dynein heavy chain, axonemal!K12804|2|3e-14|82.0|dre:571331|proline-serine-threonine phosphatase interacting protein 1	gi|7106381|ref|NP_035992.1|	0	protein kinase C and casein kinase substrate in neurons protein 2 [Mus musculus]
mmu-miR-105	NM_001159591	-31.4	0.144506	-26.35	163	GO:0044444,GO:0046872,GO:0005515,GO:0004672,GO:0032559,GO:0006643,GO:0007166,GO:0006468,	K08958|1|0.0|772|mmu:103236|casein kinase 1, gamma [EC:2.7.11.1]	gi|227430313|ref|NP_001153063.1|	0	casein kinase I isoform gamma-2 isoform 2 [Mus musculus]

mmu-miR-105	NM_001159627	-29.1	0.599641	-47.46	318	GO:0031224,GO:0044444,GO:0046914,GO:0003824,GO:0030099,GO:0008152,GO:0000041,GO:0006875,	K14735|1|0.0|2287|mmu:15203|hephaestin	gi|148682267|gb|EDL14214.1|	0	hephaestin, isoform CRA_a [Mus musculus]
mmu-miR-105	NM_001159628	-29.1	0.597902	-47.46	318	GO:0031224,GO:0044444,GO:0046914,GO:0003824,GO:0030099,GO:0008152,GO:0000041,GO:0006875,	K14735|1|0.0|2281|mmu:15203|hephaestin	gi|148682268|gb|EDL14215.1|	0	hephaestin, isoform CRA_b [Mus musculus]
mmu-miR-105	NM_001159908	-30.6	0.279599	-28.79	172	GO:0043231,GO:0046914,	-	gi|19111158|ref|NP_579927.1|	2.99E-91	AN1-type zinc finger protein 2A [Mus musculus]

mmu-miR-105	NM_001159952	-27.9	0.929277	-50.85	311	GO:0044297,GO:0044444,GO:0047555,GO:0043169,GO:0005515,GO:0016208,GO:0007606,GO:0050796,GO:0006195,GO:0010038,GO:0010863,GO:0007169,	K13755|1|0.0|1145|mmu:18575|calcium/calmodulin-dependent 3',5'-cyclic nucleotide phosphodiesterase [EC:3.1.4.17]	gi|229577068|ref|NP_001153424.1|	0	calcium/calmodulin-dependent 3',5'-cyclic nucleotide phosphodiesterase 1C isoform c [Mus musculus]
mmu-miR-105	NM_001159957	-27.9	0.91878	-50.85	311	GO:0044297,GO:0044444,GO:0047555,GO:0043169,GO:0005515,GO:0016208,GO:0007606,GO:0050796,GO:0006195,GO:0010038,GO:0010863,GO:0007169,	K13755|1|0.0|1103|mmu:18575|calcium/calmodulin-dependent 3',5'-cyclic nucleotide phosphodiesterase [EC:3.1.4.17]	gi|148672758|gb|EDL04705.1|	0	phosphodiesterase 1C, isoform CRA_d [Mus musculus]

mmu-miR-105	NM_001159960	-27.9	0.927428	-50.85	311	GO:0044297,GO:0044444,GO:0047555,GO:0043169,GO:0005515,GO:0016208,GO:0007606,GO:0050796,GO:0006195,GO:0010038,GO:0010863,GO:0007169,	K13755|1|0.0|1171|mmu:18575|calcium/calmodulin-dependent 3',5'-cyclic nucleotide phosphodiesterase [EC:3.1.4.17]	gi|148672760|gb|EDL04707.1|	0	phosphodiesterase 1C, isoform CRA_f [Mus musculus]
mmu-miR-105	NM_001160178	-31.8	0.094804	-25.81	172	-	K10476|1|1e-90|334|dre:557467|kelch repeat and BTB domain-containing protein 11	gi|237513007|ref|NP_001153650.1|	0	kelch domain containing 7B [Mus musculus]

mmu-miR-105	NM_001161432	-28.7	0.667274	-27.02	165	GO:0016021,GO:0005035,GO:0005488,GO:0043410,GO:0048869,GO:0009888,GO:0051091,GO:0008219,GO:0019221,GO:00072	K05163|1|1e-164|582|mmu:245527|tumor necrosis factor receptor superfamily member 27	gi|148682260|gb|EDL14207.1|	4.43E-166	ectodysplasin A2 isoform receptor, isoform CRA_b [Mus musculus]
mmu-miR-105	NM_001161433	-28.7	0.376402	-27.02	165	GO:0016021,GO:0005035,GO:0005488,GO:0043410,GO:0048869,GO:0009888,GO:0051091,GO:0008219,GO:0019221,GO:00072	K05163|1|5e-165|582|mmu:245527|tumor necrosis factor receptor superfamily member 27	gi|148682260|gb|EDL14207.1|	1.81E-166	ectodysplasin A2 isoform receptor, isoform CRA_b [Mus musculus]
mmu-miR-105	NM_001161628	-28.7	0.48291	-27.35	155	GO:0043231,GO:0046872,GO:0010467,	K02183|1|6e-06|54.3|tsp:Tsp_08523|calmodulin	gi|239052655|ref|NP_084222.4|	0	EF-hand calcium-binding domain-containing protein 6 isoform 1 [Mus musculus]

mmu-miR-105	NM_001162863	-33.4	0.037871	-28.09	165	GO:0016892,GO:0005488,	K01168|1|3e-06|53.9|mmu:19752|pancreatic ribonuclease [EC:3.1.27.5]	gi|247269231|ref|NP_083421.2|	7.22E-92	ribonuclease-like protein 10 isoform 1 [Mus musculus]
mmu-miR-105	NM_001163267	-29.2	0.46163	-26.59	177	-	K10352|1|1e-21|106|xla:399266|myosin heavy chain	gi|253795525|ref|NP_001156739.1|	0	coiled-coil domain containing 147 [Mus musculus]
mmu-miR-105	NM_001163473	-25.7	0.559732	-29.24	155	-	-	gi|344243051|gb|EGV99154.1|	5.12E-67	UPF0450 protein C17orf58-like [Cricetulus griseus]
mmu-miR-105	NM_001163531	-26.5	0.813072	-48.22	338	GO:0031224,	-	gi|148688148|gb|EDL20095.1|	0	RIKEN cDNA 3010001K23, isoform CRA_a [Mus musculus]
mmu-miR-105	NM_001163532	-26.5	0.813387	-48.22	338	GO:0031224,	-	gi|148688149|gb|EDL20096.1|	0	RIKEN cDNA 3010001K23, isoform CRA_b [Mus musculus]
mmu-miR-105	NM_001163685	-34.6	0.029754	-26.73	177	GO:0009690,GO:0002376,GO:0022414,	K06499|1|4e-74|280|rno:24926|carcinoembryonic antigen-related cell adhesion molecule	gi|254826757|ref|NP_001157157.1|	1.20E-147	pregnancy specific glycoprotein 18 isoform 2 [Mus musculus]

mmu-miR-105	NM_001163833	-25.7	0.816459	-40.89	309	GO:0044446,GO:0005634,GO:0005488,GO:0016568,GO:0044238,GO:0044260,	K11339|1|7e-06|53.5|tad:TRIADDRAFT_60384|mortality factor 4-like protein 1	gi|255652855|ref|NP_001157305.1|	4.58E-138	male-specific lethal 3-like 2 [Mus musculus]
mmu-miR-105	NM_001164676	-26.8	0.893258	-28.27	160	-	K09228|1|0.0|1536|mmu:381067|KRAB domain-containing zinc finger protein	gi|257743035|ref|NP_001158148.1|	0	zinc finger protein 229 [Mus musculus]

mmu-miR-105	NM_001164679	-26.7	0.858469	-42.07	296	GO:0016021,GO:0005216,	K13982|1|9e-81|303|dgr:Dgri_GH10918|probable ATP-dependent RNA helicase DDX4 [EC:3.6.4.13]!K03018|3|2e-19|99.4|smm:Smp_002890|DNA-directed RNA polymerase III subunit RPC1 [EC:2.7.7.6]	gi|50511029|dbj|BAD32500.1|	0	mKIAA1623 protein [Mus musculus]

mmu-miR-105	NM_001164785	-31.5	0.412998	-28.26	150	-	K09609|1|0.0|3046|mmu:223838|a disintegrin and metalloproteinase with thrombospondin motifs 20 [EC:3.4.24.-]	gi|259155419|ref|NP_001158257.1|	0	A disintegrin and metalloproteinase with thrombospondin motifs 20 isoform 2 precursor [Mus musculus]
mmu-miR-105	NM_001164786	-31.5	0.117849	-28.26	150	GO:0031012,GO:0046914,GO:0004175,GO:0019538,	K09609|1|0.0|1012|mmu:223838|a disintegrin and metalloproteinase with thrombospondin motifs 20 [EC:3.4.24.-]	gi|259155423|ref|NP_001158258.1|	0	A disintegrin and metalloproteinase with thrombospondin motifs 20 isoform 3 precursor [Mus musculus]
mmu-miR-105	NM_001165929	-29.4	0.223326	-26.75	160	-	-	gi|260064083|ref|NP_001159401.1|	0	coiled-coil domain-containing protein 78 [Mus musculus]
mmu-miR-105	NM_001165954	-30.3	0.729586	-28.27	178	GO:0046872,	K11458|1|0.0|1244|mmu:241915|polyhomeotic-like protein 3	gi|260099711|ref|NP_001159426.1|	0	polyhomeotic-like protein 3 isoform 1 [Mus musculus]

mmu-miR-105	NM_001165955	-30.3	0.727129	-28.27	178	GO:0005488,	K11458|1|0.0|1202|mmu:241915|polyhomeotic-like protein 3	gi|260099711|ref|NP_001159426.1|	0	polyhomeotic-like protein 3 isoform 1 [Mus musculus]
mmu-miR-105	NM_001165956	-30.3	0.725889	-28.27	178	GO:0005488,	K11458|1|0.0|1142|mmu:241915|polyhomeotic-like protein 3	gi|260099715|ref|NP_001159428.1|	0	polyhomeotic-like protein 3 isoform 4 [Mus musculus]
mmu-miR-105	NM_001166033	-30.1	0.607246	-27.51	163	GO:0005667,GO:0003676,GO:0004468,GO:0030234,GO:0006383,GO:0006352,GO:0016570,	K11310|1|0.0|1395|rno:685539|general transcription factor 3C polypeptide 4 [EC:2.3.1.48]	gi|260436918|ref|NP_766565.2|	0	general transcription factor 3C polypeptide 4 isoform 1 [Mus musculus]
mmu-miR-105	NM_001166213	-29.9	0.281907	-25.81	152	-	-	gi|148696999|gb|EDL28946.1|	0	mCG145696, isoform CRA_a [Mus musculus]
mmu-miR-105	NM_001167587	-27.5	0.31372	-25.61	171	-	-	gi|262399395|ref|NP_084139.1|	2.85E-51	prostate and testis expressed A precursor [Mus musculus]

mmu-miR-105	NM_001167889	-33.5	0.09755	-29.64	173	GO:0000794,GO:0005488,GO:0018024,GO:0006351,GO:0016571,	K11429|1|0.0|769|rno:361688|histone-lysine N-methyltransferase SUV420H [EC:2.1.1.43]	gi|148701007|gb|EDL32954.1|	0	suppressor of variegation 4-20 homolog 1 (Drosophila), isoform CRA_c [Mus musculus]
mmu-miR-105	NM_001174078	-29.8	0.610239	-26.38	165	GO:0000790,GO:0070603,GO:0035258,GO:0003712,GO:0000166,GO:0042623,GO:0042393,GO:0003676,GO:0030326,GO:0001824,GO:0001709,GO:0007420,GO:0043062,GO:0032989,GO:0007090,GO:0009207,GO:0033144,GO:0006342,GO:0048729,GO:0000122,GO:0032776,GO:00165	K11647|1|0.0|2286|mmu:20586|SWI/SNF-related matrix-associated actin-dependent regulator of chromatin subfamily A member 2/4 [EC:3.6.4.-]	gi|291463269|ref|NP_001167549.1|	0	transcription activator BRG1 isoform 1 [Mus musculus]

mmu-miR-105	NM_001174079	-29.8	0.609562	-26.38	165	GO:0000790,GO:0070603,GO:0035258,GO:0003712,GO:0000166,GO:0042623,GO:0042393,GO:0003676,GO:0030326,GO:0001824,GO:0001709,GO:0007420,GO:0043062,GO:0032989,GO:0007090,GO:0009207,GO:0033144,GO:0006342,GO:0048729,GO:0000122,GO:0032776,GO:00165	K11647|1|0.0|2278|rno:171379|SWI/SNF-related matrix-associated actin-dependent regulator of chromatin subfamily A member 2/4 [EC:3.6.4.-]	gi|291463271|ref|NP_001167550.1|	0	transcription activator BRG1 isoform 3 [Mus musculus]

mmu-miR-105	NM_001177594	-27.1	0.711278	-27.46	164	GO:0043231,GO:0031224,GO:0015291,GO:0015085,GO:0070838,	K13754|1|0.0|1005|mmu:170756|solute carrier family 24 (sodium/potassium/calcium exchanger), member	gi|294862243|ref|NP_001171065.1|	0	sodium/potassium/calcium exchanger 6 isoform 2 precursor [Mus musculus]
mmu-miR-105	NM_001177595	-27.1	0.69818	-27.46	164	GO:0043231,GO:0031224,GO:0015291,GO:0015085,GO:0070838,	K13754|1|0.0|769|mmu:170756|solute carrier family 24 (sodium/potassium/calcium exchanger), member	gi|294862245|ref|NP_001171066.1|	0	sodium/potassium/calcium exchanger 6 isoform 3 precursor [Mus musculus]

mmu-miR-105	NM_001177884	-32.9	0.293808	-28.06	168	GO:0031981,GO:0043232,GO:0005635,GO:0003676,GO:0046914,GO:0016279,GO:0006357,GO:0003285,GO:0003284,GO:0006325,GO:0009653,	K11424|1|0.0|1162|mmu:107823|histone-lysine N-methyltransferase NSD2 [EC:2.1.1.43]	gi|295424169|ref|NP_001171355.1|	0	probable histone-lysine N-methyltransferase NSD2 isoform 3 [Mus musculus]
mmu-miR-105	NM_001177995	-31.9	0.424304	-40.69	308	GO:0043231,GO:0043234,GO:0046914,GO:0005515,GO:0003712,GO:0003677,GO:0007423,GO:0002762,GO:0007179,GO:0006357,GO:0043467,GO:0045444,GO:0019827,GO:00	K04462|1|0.0|1054|cfa:488148|ecotropic virus integration site 1 protein	gi|295789156|ref|NP_001171466.1|	0	PR domain zinc finger protein 16 isoform 2 [Mus musculus]

mmu-miR-105	NM_001195485	-26.9	0.663198	-49.1	320	-	K12896|1|9e-56|216|ptr:459167|splicing factor, arginine/serine-rich 7	gi|148706565|gb|EDL38512.1|	5.62E-57	mCG17902, isoform CRA_k [Mus musculus]
mmu-miR-105	NM_001195486	-26.9	0.65911	-49.1	320	-	K12896|1|9e-56|216|ptr:459167|splicing factor, arginine/serine-rich 7	gi|148706565|gb|EDL38512.1|	5.58E-57	mCG17902, isoform CRA_k [Mus musculus]
mmu-miR-105	NM_001195487	-26.9	0.657046	-49.1	320	-	K12896|1|9e-56|216|ptr:459167|splicing factor, arginine/serine-rich 7	gi|148706565|gb|EDL38512.1|	5.54E-57	mCG17902, isoform CRA_k [Mus musculus]

mmu-miR-105	NM_001199271	-30.4	0.213065	-25.21	167	GO:0016021,GO:0044459,GO:0015238,GO:0051183,GO:0015291,GO:0016597,GO:0006575,GO:0015893,GO:0006766,GO:0006835,GO:0022414,GO:0006865,	K14609|1|0.0|980|mmu:20509|solute carrier family 19 (folate transporter), member 1	gi|13654260|ref|NP_112473.1|	0	folate transporter 1 [Mus musculus]
mmu-miR-105	NM_001199330	-31.6	0.179663	-28.39	176	-	K09228|1|0.0|852|mmu:233168|KRAB domain-containing zinc finger protein	gi|148680500|gb|EDL12447.1|	0	mCG55139, isoform CRA_c [Mus musculus]
mmu-miR-105	NM_001204229	-34.7	0.126648	-30.26	167	-	-	gi|28972167|dbj|BAC65537.1|	0	mKIAA0350 protein [Mus musculus]
mmu-miR-105	NM_001205068	-25	0.988869	-44.29	300	-	K11323|1|1e-17|94.0|dwi:Dwil_GK14253|histone arginine demethylase JMJD6 [EC:1.14.1	gi|326633177|ref|NP_001191997.1|	0	jmjC domain-containing protein 4 isoform 2 [Mus musculus]

mmu-miR-105	NM_001205076	-29	0.569839	-25.29	163	GO:0031674,GO:0033017,GO:0005488,GO:0032412,GO:0007204,	K04575|1|1e-14|84.0|nvi:100117052|amyotrophic lateral sclerosis 2 (juvenile)	gi|10947010|ref|NP_067541.1|	0	junctophilin-2 [Mus musculus]
mmu-miR-105	NM_001205204	-31	0.488359	-25.88	173	GO:0031224,GO:0016337,GO:0007610,GO:0048870,	-	gi|327199303|ref|NP_001192133.1|	0	astrotactin-1 isoform 1 precursor [Mus musculus]
mmu-miR-105	NM_001205313	-30.8	0.400429	-28.17	162	-	K11220|1|0.0|1504|mmu:20846|signal transducer and activator of transcription 1	gi|148667551|gb|EDK99967.1|	0	signal transducer and activator of transcription 1, isoform CRA_c [Mus musculus]

mmu-miR-105	NM_001205314	-30.8	0.401552	-28.17	162	GO:0031981,GO:0043005,GO:0044444,GO:0060089,GO:0046872,GO:0001071,GO:0048020,GO:0003677,GO:0032868,GO:0006915,GO:0008284,GO:0018108,GO:0007243,GO:0072183,GO:0031667,GO:0043331,GO:0012502,GO:0000302,GO:0002237,GO:0000122,GO:0001959,GO:00033	K11220|1|0.0|1485|mmu:20846|signal transducer and activator of transcription 1	gi|148667548|gb|EDK99964.1|	0	signal transducer and activator of transcription 1, isoform CRA_a [Mus musculus]

mmu-miR-105	NM_001205336	-37.5	0.03728	-34.99	176	GO:0031252,GO:0044444,GO:0046914,GO:0035091,GO:0005100,GO:0006810,GO:0007266,GO:0065008,GO:0006996,GO:0032012,GO:0016601,GO:0016477,	K12490|1|0.0|2385|mmu:106952|Arf-GAP, Rho-GAP domain, ANK repeat and PH domain-containing protein	gi|18700711|gb|AAL78678.1|AF469622_1	0	dual-specificity Rho- and Arf-GTPase activating protein 1 [Mus musculus]
mmu-miR-105	NM_001252081	-26	0.981497	-43.86	310	GO:0046914,	-	gi|355390253|ref|NP_001239010.1|	0	zinc finger SWIM domain-containing protein KIAA0913 isoform 2 [Mus musculus]
mmu-miR-105	NM_001163415	-23.6	0.999636	-71.37	469	GO:0008287,GO:0044424,GO:0005488,GO:0004721,GO:0019888,GO:0006464,GO:0008283,GO:0051318,	K11583|1|0.0|913|mmu:19054|protein phosphatase 2 (formerly 2A), regulatory subunit B''	gi|148705238|gb|EDL37185.1|	0	mCG8010, isoform CRA_b [Mus musculus]
mmu-miR-106a-3p	NM_001001979	-32.4	0.317679	-47.58	328	GO:0044459,GO:0031224,GO:0006910,	K02599|1|3e-82|309|bfo:BRAFLDRAFT_99311|Notch	gi|50233828|ref|NP_001001979.1|	0	multiple epidermal growth factor-like domains protein 10 precursor [Mus musculus]

mmu-miR-106a-3p	NM_001004144	-25.3	0.960651	-46.88	320	GO:0005924,GO:0044424,GO:0016772,GO:0005083,GO:0008047,GO:0004871,GO:0046914,GO:0042802,GO:0051020,GO:0043087,GO:0007186,GO:0006796,GO:00320	K05737|1|0.0|1467|mmu:216963|G protein-coupled receptor kinase interactor 1	gi|51921285|ref|NP_001004144.1|	0	ARF GTPase-activating protein GIT1 [Mus musculus]
mmu-miR-106a-3p	NM_001004151	-31.4	0.194405	-25.71	156	GO:0031224,GO:0004871,GO:0001664,GO:0023052,GO:0034613,	-	gi|51921305|ref|NP_001004151.1|	2.96E-160	receptor-transporting protein 1 [Mus musculus]
mmu-miR-106a-3p	NM_001005788	-27.5	0.564811	-25.77	155	GO:0043231,GO:0046914,GO:0003676,GO:0006351,	K09228|1|0.0|761|mcc:694407|KRAB domain-containing zinc finger protein	gi|238859629|ref|NP_001005788.2|	0	zinc finger protein 69 [Mus musculus]

mmu-miR-106a-3p	NM_001008550	-31.3	0.51748	-44.84	310	GO:0005815,GO:0035091,GO:0046914,GO:0006259,	K05724|1|6e-13|79.3|mgp:100546627|FYVE, RhoGEF and PH domain containing 5/6	gi|60360314|dbj|BAD90401.1|	0	mKIAA0321 protein [Mus musculus]
mmu-miR-106a-3p	NM_001011732	-31.2	0.181861	-69.03	480	GO:0031224,	-	gi|59858537|ref|NP_001011732.1|	0	XK-related protein 7 [Mus musculus]
mmu-miR-106a-3p	NM_001012402	-31.1	0.072688	-25.41	162	GO:0031224,GO:0034483,	K09679|1|8e-168|590|mmu:328779|[heparan sulfate]-glucosamine 3-sulfotransferase 6 [EC:2.8.2.-	gi|60223081|ref|NP_001012402.1|	5.14E-167	heparan sulfate glucosamine 3-O-sulfotransferase 6 [Mus musculus]

mmu-miR-106a-3p	NM_001015039	-26.5	0.881254	-26.31	171	GO:0005769,GO:0046914,GO:0035091,GO:0007176,	K01104|1|4e-15|85.1|tca:657269|protein-tyrosine phosphatase [EC:3.1.3.48]!K12182|2|7e-15|84.3|api:100165322|hepatocyte growth factor-regulated tyrosine kinase substrate!K12482|5|4e-14|82.0|tgu:100229989|RUN and FYVE domain-containin	gi|37360492|dbj|BAC98224.1|	0	mKIAA1643 protein [Mus musculus]
mmu-miR-106a-3p	NM_001025067	-28.5	0.809127	-44.83	314	GO:0044424,GO:0031224,	K06753|1|1e-30|137|acs:100559253|roundabout, axon guidance receptor	gi|148675623|gb|EDL07570.1|	0	leucine-rich repeats and immunoglobulin-like domains 2, isoform CRA_b [Mus musculus]

mmu-miR-106a-3p	NM_001029889	-28.1	0.958481	-45.63	330	GO:0043231,GO:0046872,GO:0003676,GO:0015924,	K10955|1|1e-07|62.0|gga:423101|intestinal mucin-2	gi|148665619|gb|EDK98035.1|	0	gene model 608, (NCBI), isoform CRA_b [Mus musculus]
mmu-miR-106a-3p	NM_001033039	-29.2	0.214239	-25.57	164	-	K10455|1|3e-06|54.3|bfo:BRAFLDRAFT_74807|kelch-like protein	gi|85986583|ref|NP_001028211.1|	0	kelch domain-containing protein 9 [Mus musculus]
mmu-miR-106a-3p	NM_001033347	-27.2	0.975584	-45.04	327	GO:0031224,	K15259|1|2e-80|303|cfa:482778|poly [ADP-ribose] polymerase 7/11/12/13 [EC:2.4.2.30]	gi|124358961|ref|NP_001028519.2|	0	uncharacterized protein LOC241589 [Mus musculus]

mmu-miR-106a-3p	NM_001033399	-27.1	0.931961	-25.99	169	GO:0003824,GO:0008152,	K00078|1|3e-09|66.6|dpe:Dper_GL19398|dihydrodiol dehydrogenase / D-xylose 1-dehydrogenase (NADP) [EC:1.3.1.20	gi|85701893|ref|NP_001028571.1|	0	glucose-fructose oxidoreductase domain-containing protein 1 precursor [Mus musculus]
mmu-miR-106a-3p	NM_001035122	-31.1	0.248009	-25.45	167	GO:0016021,GO:0043231,	K10352|1|2e-09|66.2|isc:IscW_ISCW001340|myosin heavy chain!K14972|2|5e-06|54.7|aga:AgaP_AGAP004734|PAX-interacting protein 1	gi|78190500|ref|NP_001030294.1|	0	Golgi membrane protein 1 [Mus musculus]

mmu-miR-106a-3p	NM_001035525	-28.6	0.566155	-26.28	155	GO:0044444,GO:0031224,GO:0005488,	K15619|1|6e-10|67.8|mgp:100540482|Kunitz-type protease inhibitor 1	gi|78482609|ref|NP_001030602.1|	0	dyslexia-associated protein KIAA0319-like protein isoform 1 [Mus musculus]
mmu-miR-106a-3p	NM_001035526	-28.6	0.577981	-26.28	155	GO:0044444,GO:0031224,GO:0005488,	K15619|1|7e-10|67.8|mgp:100540482|Kunitz-type protease inhibitor 1	gi|78482609|ref|NP_001030602.1|	0	dyslexia-associated protein KIAA0319-like protein isoform 1 [Mus musculus]
mmu-miR-106a-3p	NM_001037711	-31	0.353258	-48.14	322	GO:0043296,GO:0015629,GO:0008092,	K06102|1|0.0|1888|mmu:70737|cingulin	gi|50510881|dbj|BAD32426.1|	0	mKIAA1319 protein [Mus musculus]
mmu-miR-106a-3p	NM_001037762	-28.6	0.194564	-25.65	165	-	-	gi|83649751|ref|NP_001032851.1|	7.39E-80	probable palmitoyltransferase ZDHHC12 isoform b [Mus musculus]

mmu-miR-106a-3p	NM_001038627	-28.4	0.729652	-47.99	329	GO:0044444,GO:0016020,GO:0032403,GO:0046332,GO:0019787,GO:0048869,GO:0009888,GO:0001503,GO:0006511,GO:0030509,GO:0006605,GO:0016567,GO:00071	K04678|1|0.0|1495|mmu:75788|E3 ubiquitin ligase SMURF1/2 [EC:6.3.2.19]	gi|87044890|ref|NP_001033716.1|	0	E3 ubiquitin-protein ligase SMURF1 isoform 1 [Mus musculus]
mmu-miR-106a-3p	NM_001039126	-28.8	0.711876	-44.07	331	GO:0007165,GO:0046546,	K10323|1|0.0|658|mmu:65247|ankyrin repeat and SOCS box protein 1	gi|85060520|ref|NP_075533.3|	0	ankyrin repeat and SOCS box protein 1 isoform b [Mus musculus]

mmu-miR-106a-3p	NM_001039180	-34.1	0.104747	-28.11	159	GO:0031982,GO:0043232,GO:0043231,GO:0016020,GO:0005083,GO:0005488,GO:0019222,GO:0034453,GO:0072384,	K10421|1|3e-14|82.4|dse:Dsec_GM17234|CAP-Gly domain-containing linker protein 1!K10352|3|2e-13|79.7|aml:100473449|myosin heavy chain!K12478|5|3e-13|79.0|dre:562943|early endosome antigen 1	gi|85702355|ref|NP_001034268.1|	0	protein bicaudal D homolog 2 isoform 1 [Mus musculus]

mmu-miR-106a-3p	NM_001039484	-29.9	0.517367	-25.01	160	GO:0016021,GO:0043234,GO:0042995,GO:0044459,GO:0005515,GO:0032559,GO:0005242,GO:0055065,GO:0014003,GO:0050953,GO:0048168,GO:0015813,GO:0051931,GO:0071805,GO:0021545,GO:0009416,GO:0031960,GO:0051260,GO:0042391,GO:0006952,GO:0051933,GO:00083	K05003|1|0.0|807|ptr:457431|potassium inwardly-rectifying channel subfamily J member 10	gi|71534081|gb|AAH99932.1|	0	Kcnj10 protein, partial [Mus musculus]

mmu-miR-106a-3p	NM_001039581	-31.6	0.356297	-54.79	325	GO:0031224,GO:0030141,GO:0017111,GO:0015405,GO:0032559,GO:0009207,GO:0042221,GO:0051234,	K05643|1|0.0|3197|mmu:27410|ATP-binding cassette, subfamily A (ABC1), member 3	gi|148690365|gb|EDL22312.1|	0	ATP-binding cassette, sub-family A (ABC1), member 3, isoform CRA_b [Mus musculus]
mmu-miR-106a-3p	NM_001040089	-34.9	0.007029	-28.6	175	GO:0043231,GO:0003677,GO:0006351,	K09452|1|2e-10|66.6|aml:100466274|homeobox protein aristaless-related!K09451|5|1e-09|64.3|ptr:466496|homeobox protein aristaless-like 4	gi|296841121|ref|NP_001035178.3|	3.55E-122	reproductive homeobox 3F [Mus musculus]
mmu-miR-106a-3p	NM_001045863	-30.2	0.14077	-26.77	169	GO:0031981,GO:0003676,GO:0045935,GO:0006354,	K15182|1|1e-139|497|mmu:27632|negative elongatio	gi|148694798|gb|EDL26745.1|	7.98E-139	RD RNA-binding protein, isoform CRA_a [Mus musculus]

mmu-miR-106a-3p	NM_001045864	-30.2	0.150159	-26.77	169	GO:0031981,GO:0003676,GO:0045935,GO:0006354,	K15182|1|1e-139|497|mmu:27632|negative elongatio	gi|148694798|gb|EDL26745.1|	8.55E-139	RD RNA-binding protein, isoform CRA_a [Mus musculus]
mmu-miR-106a-3p	NM_001077184	-34.5	0.019247	-29.77	167	GO:0031224,GO:0012505,GO:0048770,GO:0005886,GO:0048029,GO:0008028,GO:0042476,GO:0010038,GO:0009725,GO:0032787,GO:0015711,GO:0002376,GO:0007599,GO:0007165,GO:0003006,	K06535|1|5e-148|525|dre:100142636|basigin	gi|116014342|ref|NP_001070652.1|	3.18E-147	basigin isoform 2 precursor [Mus musculus]
mmu-miR-106a-3p	NM_001080813	-32	0.377467	-28.37	160	GO:0005768,GO:0005815,GO:0016020,GO:0031410,GO:0006810,	K12484|1|0.0|1850|mmu:75767|Rab11 family-interacting protein 1/2/5	gi|124249097|ref|NP_001074282.1|	0	rab11 family-interacting protein 1 isoform 1 [Mus musculus]

mmu-miR-106a-3p	NM_001081014	-28.6	0.721057	-25.67	151	-	K12861|1|5e-09|65.5|gga:419884|pre-mRNA-splicing factor SPF27	gi|124486610|ref|NP_001074483.1|	0	DENN domain-containing protein 4C isoform 2 [Mus musculus]
mmu-miR-106a-3p	NM_001081061	-25.5	0.999049	-47.19	326	-	K15198|1|0.0|4487|mmu:544971|transcription factor TFIIIB component B''	gi|145587077|ref|NP_001074530.1|	0	transcription factor TFIIIB component B'' homolog [Mus musculus]

mmu-miR-106a-3p	NM_001081091	-29.3	0.627248	-46.74	336	GO:0044444,	K14692|1|6e-20|101|smm:Smp_170590|solute carrier family 30 (zinc transporter), member 5!K12478|2|8e-15|84.7|oaa:100079644|early endosome antigen 1!K10352|3|8e-15|84.7|pon:100450821|myosin heavy chain	gi|50510705|dbj|BAD32338.1|	0	mKIAA0912 protein [Mus musculus]

mmu-miR-106a-3p	NM_001081097	-26.8	0.979079	-46.97	316	GO:0016021,GO:0030425,GO:0043025,GO:0033267,GO:0044459,GO:0005102,GO:0005231,GO:0004970,GO:0004930,GO:0007193,GO:0006811,GO:0006873,GO:00508	K05203|1|0.0|1802|mmu:14807|glutamate receptor, ionotropic kainate 3	gi|124487364|ref|NP_001074566.1|	0	glutamate receptor, ionotropic kainate 3 precursor [Mus musculus]

mmu-miR-106a-3p	NM_001081135	-25.9	0.979331	-54.2	320	GO:0031224,GO:0046872,	K04949|1|1e-17|94.0|ecb:100069276|cyclic nucleotide gated channel alpha 2!K01314|2|5e-09|65.5|cfa:476993|coagulation factor X [EC:3.4.21.6]!K01320|3|7e-09|65.1|aml:100471728|coagulation factor VII [EC:3.4.21.21]!K01313|4|1e-08|64.3|xtr:548514|	gi|124487491|ref|NP_001074604.1|	4.00E-108	transmembrane gamma-carboxyglutamic acid protein 3 precursor [Mus musculus]

mmu-miR-106a-3p	NM_001081171	-26.8	0.991429	-43.33	323	GO:0043256,GO:0004871,GO:0005102,GO:0007166,GO:0072659,GO:0008544,GO:0007155,GO:0001667,GO:0008283,GO:0042476,GO:0061138,GO:2000026,GO:0007044,GO:0006996,GO:0000904,GO:0030323,GO:0048754,GO:0030855,GO:00109	K06240|1|0.0|7470|mmu:16776|laminin, alpha 3/5	gi|124487155|ref|NP_001074640.1|		0	laminin subunit alpha-5 precursor [Mus musculus]
mmu-miR-106a-3p	NM_001081269	-25.3	0.999458	-47.51	316	GO:0043232,GO:0046914,GO:0016279,GO:0016571,GO:0040007,GO:0048869,	K11425|1|0.0|2520|pon:100459660|histone-lysine N-methyltransferase NSD3 [EC:2.1.1.43]	gi|124486903|ref|NP_001074738.1|		0	histone-lysine N-methyltransferase NSD3 isoform 2 [Mus musculus]

mmu-miR-106a-3p	NM_001081330	-28.3	0.957682	-43.48	313	GO:0030286,GO:0005930,GO:0003774,GO:0032559,GO:0048870,GO:0009207,GO:00070	K10408|1|0.0|8777|mmu:327954|dynein heavy chain, axonemal	gi|124486773|ref|NP_001074799.1|	0	dynein heavy chain 2, axonemal [Mus musculus]
mmu-miR-106a-3p	NM_001081407	-27.7	0.794346	-46.66	313	GO:0044459,GO:0031224,GO:0004091,GO:0016101,	K14621|1|0.0|2901|mmu:665270|phospholipase B1, membrane-associated [EC:3.1.1.4 3.1.1.5]	gi|124487227|ref|NP_001074876.1|	0	phospholipase B1, membrane-associated isoform 1 precursor [Mus musculus]
mmu-miR-106a-3p	NM_001082476	-29.1	0.585998	-25.16	159	GO:0005856,GO:0043231,GO:0044444,GO:0046914,GO:0050662,GO:0003824,GO:0000166,GO:0009987,GO:0008152,	K00327|1|5e-71|271|ssc:100170114|NADPH-ferrihemoprotein reductase [EC:1.6.2.4]	gi|126723293|ref|NP_001075945.1|	0	NADPH-dependent diflavin oxidoreductase 1 isoform 1 [Mus musculus]

mmu-miR-106a-3p	NM_001082552	-26.4	0.7815	-41.91	315	GO:0031981,GO:0031461,GO:0030529,GO:0003676,GO:0046914,GO:0019787,GO:0051259,GO:0051726,GO:0043433,GO:0031647,GO:0016567,GO:0016579,	K10651|1|0.0|916|mmu:20821|tripartite motif-containing protein 21	gi|14714855|gb|AAH10580.1|	0	Tripartite motif-containing 21 [Mus musculus]
mmu-miR-106a-3p	NM_001099217	-32.2	0.02705	-27.27	172	GO:0009986,GO:0031224,	K06846|1|6e-61|234|mmu:17067|lymphocyte antigen 6 complex	gi|149773537|ref|NP_001092687.1|	9.11E-63	lymphocyte antigen 6C2 precursor [Mus musculus]
mmu-miR-106a-3p	NM_001101562	-29.6	0.099477	-25.1	161	GO:0031224,GO:0004930,GO:0007166,	K04614|1|5e-174|610|mmu:622032|vomeronasal1 receptor	gi|178057347|ref|NP_001095032.1|	3.25E-173	vomeronasal 1 receptor 167 [Mus musculus]

mmu-miR-106a-3p	NM_001102457	-34.9	0.004564	-28.6	175	GO:0043231,GO:0003677,GO:0006351,	K09296|1|2e-09|63.2|oaa:100077672|homeobox protein HoxA/C/D11!K09451|2|2e-09|63.2|ptr:466496|homeobox protein aristaless-like 4!K09331|5|7e-09|60.8|cfa:485732|short stature homeobox protein	gi|297139732|ref|NP_001095927.3|	2.88E-122	reproductive homeobox 3C [Mus musculus]

mmu-miR-106a-3p	NM_001113326	-30.7	0.108011	-26.54	169	GO:0034358,GO:0016021,GO:0031982,GO:0044444,GO:0071813,GO:0004872,GO:0023052,GO:0015918,GO:0032501,GO:0006897,GO:0010742,GO:0015031,GO:00108	K06558|1|0.0|830|mmu:20288|macrophage scavenger receptor 1	gi|164664520|ref|NP_001106797.1|	0	macrophage scavenger receptor types I and II isoform b [Mus musculus]

mmu-miR-106a-3p	NM_001113364	-30.2	0.36695	-48.65	326	GO:0044464,GO:0005099,GO:0032313,	K11837|1|4e-11|72.0|hsa:9098|ubiquitin carboxyl-terminal hydrolase 6/32 [EC:3.1.2.15]!K08187|2|8e-08|60.8|tgu:100221433|MFS transporter, MCP family, solute carrier family 16 (monocarboxylic acid transporters), member 10!K13136|3|7e-	gi|164565383|ref|NP_001106835.1|	0	TBC1 domain family member 14 isoform c [Mus musculus]
mmu-miR-106a-3p	NM_001113461	-30.6	0.215927	-25.62	169	GO:0044444,GO:0043169,GO:0004713,GO:0032559,GO:0035556,GO:0006464,GO:00098	K07364|1|0.0|1159|mmu:21682|tec protein tyrosine kinase [EC:2.7.10.2]	gi|164698392|ref|NP_001106932.1|	0	tyrosine-protein kinase Tec isoform b [Mus musculus]

mmu-miR-106a-3p	NM_001114079	-31.2	0.154391	-26.44	168	GO:0005488,	K13126|1|0.0|1174|mmu:381404|polyadenylate-binding protein	gi|166157896|ref|NP_001107551.1|	0	polyadenylate-binding protein 1-like [Mus musculus]
mmu-miR-106a-3p	NM_001114157	-34.2	0.014235	-27.34	170	GO:0043231,GO:0003677,GO:0006351,	K09452|1|3e-09|63.5|aml:100466274|homeobox protein aristaless-related!K09451|2|3e-09|63.5|ptr:466496|homeobox protein aristaless-like 4	gi|166706931|ref|NP_001107629.1|	5.23E-122	reproductive homeobox 3H [Mus musculus]
mmu-miR-106a-3p	NM_001122666	-25.4	0.86725	-45.23	309	GO:0070822,GO:0019899,GO:0006355,GO:0006325,	-	gi|169881250|ref|NP_001116138.1|	1.65E-136	sin3 histone deacetylase corepressor complex component SDS3 isoform b [Mus musculus]

mmu-miR-106a-3p	NM_001122756	-27.1	0.860129	-49.31	315	GO:0031224,GO:0004175,GO:0008236,GO:0004888,GO:0030154,GO:0001990,GO:00230	K09614|1|0.0|2098|mmu:53419|corin [EC:3.4.21.-]	gi|170295827|ref|NP_001116228.1|	0	atrial natriuretic peptide-converting enzyme isoform 2 [Mus musculus]
mmu-miR-106a-3p	NM_001122780	-27	0.771425	-42.62	306	GO:0044424,	K10462|1|0.0|1172|mmu:207952|kelch-like protein 25/37 (ectoderm-neural cortex protein)	gi|33354238|ref|NP_877583.1|	0	ectoderm-neural cortex protein 2 [Mus musculus]

mmu-miR-106a-3p	NM_001128103	-28	0.570406	-46.33	334	GO:0034702,GO:0005253,GO:0015698,	K05169|1|3e-27|125|ptr:743996|single Ig IL-1R-related molecule!K13982|2|4e-16|88.2|dgr:Dgri_GH10918|probable ATP-dependent RNA helicase DDX4 [EC:3.6.4.13]	gi|189409057|ref|NP_001121575.1|	0	anoctamin-3 isoform 2 [Mus musculus]
mmu-miR-106a-3p	NM_001130515	-29	0.285287	-25.28	170	GO:0031981,GO:0003723,GO:0016538,GO:0006357,GO:0000079,	K15189|1|6e-171|601|mmu:71059|protein HEXIM1/2	gi|110625829|ref|NP_081934.1|	3.94E-170	protein HEXIM2 [Mus musculus]
mmu-miR-106a-3p	NM_001130516	-29	0.287838	-50.59	346	GO:0031981,GO:0003723,GO:0016538,GO:0006357,GO:0000079,	K15189|1|6e-171|601|mmu:71059|protein HEXIM1/2	gi|110625829|ref|NP_081934.1|	3.98E-170	protein HEXIM2 [Mus musculus]

mmu-miR-106a-3p	NM_001136075	-31.5	0.110536	-25.94	169	GO:0019898,GO:0044459,GO:0005768,GO:0050839,GO:0021846,GO:0048667,GO:0031346,GO:0045216,GO:0010720,GO:0021537,GO:0050767,	K06057|1|0.0|1165|mmu:18222|numb	gi|209862959|ref|NP_001129547.1|	0	protein numb homolog isoform 1 [Mus musculus]
mmu-miR-106a-3p	NM_001136088	-25.1	0.943503	-26.74	158	GO:0030674,GO:0023052,	K07984|1|0.0|943|mmu:24055|SH3-domain binding protein 2	gi|224994231|ref|NP_001139331.1|	0	SH3 domain-binding protein 2 isoform b [Mus musculus]
mmu-miR-106a-3p	NM_001136270	-25.9	0.685848	-27.77	163	GO:0016020,	-	gi|210147464|ref|NP_001129742.1|	6.75E-09	proline-rich protein 24 [Mus musculus]
mmu-miR-106a-3p	NM_001141933	-32.5	0.023107	-28.65	164	GO:0016020,	-	gi|213513245|ref|NP_001135405.1|	9.17E-92	sodium/potassium-transporting ATPase subunit beta-1-interacting protein 4 isoform 2 precursor [Mus

mmu-miR-106a-3p	NM_001142701	-28.6	0.588958	-48.54	309	GO:0044444,GO:0008047,GO:0035556,GO:0043087,GO:0009966,	K02649|1|3e-27|125|bfo:BRAFLDRAFT_91712|phosphoinositide-3-kinase, regulatory subunit!K04450|2|3e-25|119|ptr:739083|activating transcription factor 2!K10360|3|3e-24|115|oaa:100075292|myosin IX!K08878|4|8e-24|114|bfo:BRAFLD	gi|218156343|ref|NP_001136173.1|	0	minor histocompatibility protein HA-1 isoform 1 [Mus musculus]

mmu-miR-106a-3p	NM_001145406	-34.9	0.005486	-28.6	175	GO:0043231,GO:0003677,GO:0006351,	K09451|1|2e-09|62.8|dre:100006399|homeobox protein aristaless-like 4!K09331|3|3e-09|62.4|hsa:6474|short stature homeobox protein!K09452|5|9e-09|60.8|aml:100466274|homeobox protein aristaless-related	gi|166706931|ref|NP_001107629.1|	6.95E-118	reproductive homeobox 3H [Mus musculus]

mmu-miR-106a-3p	NM_001145820	-32.5	0.241114	-29.21	172	GO:0043231,GO:0043234,GO:0019866,GO:0052590,GO:0046872,GO:0016616,GO:0006629,GO:0006071,GO:0019674,GO:00060	K00111|1|0.0|1356|mmu:14571|glycerol-3-phosphate dehydrogenase [EC:1.1.5.3]	gi|224922803|ref|NP_034404.3|	0	glycerol-3-phosphate dehydrogenase, mitochondrial precursor [Mus musculus]
mmu-miR-106a-3p	NM_001145858	-25.3	0.935069	-26.74	158	GO:0030674,GO:0023052,	K07984|1|0.0|1032|mmu:24055|SH3-domain binding protein 2	gi|224994229|ref|NP_001139330.1|	0	SH3 domain-binding protein 2 isoform c [Mus musculus]
mmu-miR-106a-3p	NM_001145859	-25.3	0.935234	-26.74	158	GO:0030674,GO:0023052,	K07984|1|0.0|943|mmu:24055|SH3-domain binding protein 2	gi|224994231|ref|NP_001139331.1|	0	SH3 domain-binding protein 2 isoform b [Mus musculus]
mmu-miR-106a-3p	NM_001145958	-32.4	0.271117	-78.51	500	GO:0005813,GO:0016020,GO:0008092,GO:0031023,GO:0033036,	K10352|1|2e-28|129|smm:Smp_085540.6|myosin heavy chain	gi|225543193|ref|NP_001139430.1|	0	rootletin isoform 2 [Mus musculus]

mmu-miR-106a-3p	NM_001145978	-30.2	0.534932	-47.74	328	GO:0043231,GO:0005819,GO:0032991,GO:0016763,GO:0003676,GO:0005515,GO:0006259,GO:0051234,GO:0006952,GO:0006464,GO:0033554,GO:0042221,	K10798|1|0.0|2730|mmu:328417|poly [ADP-ribose] polymerase [EC:2.4.2.30]	gi|281485553|ref|NP_001139450.2|	0	poly (ADP-ribose) polymerase family, member 4 [Mus musculus]
mmu-miR-106a-3p	NM_001146100	-29.5	0.479229	-26.04	157	GO:0031981,GO:0032991,GO:0031966,GO:0044459,GO:0017111,GO:0004396,GO:0042802,GO:0032559,GO:0048029,GO:0006007,GO:0032412,GO:0009207,GO:0008645,GO:0006468,GO:0006915,	K00844|1|0.0|1826|mmu:15275|hexokinase [EC:2.7.1.1]	gi|225735584|ref|NP_001139572.1|	0	hexokinase-1 isoform HK1 [Mus musculus]

mmu-miR-106a-3p	NM_001146174	-25.1	0.943347	-41.88	311	GO:0000776,GO:0005819,GO:0005635,GO:0044444,GO:0005099,GO:0005488,GO:0007067,GO:0032316,	K14319|1|0.0|972|mmu:19387|Ran GTPase-activating protein 1	gi|37360568|dbj|BAC98262.1|	0	mKIAA1835 protein [Mus musculus]
mmu-miR-106a-3p	NM_001160229	-30.6	0.474224	-32.55	184	-	K09228|1|0.0|944|rno:293849|KRAB domain-containing zinc finger protein	gi|237681073|ref|NP_113682.2|	0	Zinc finger protein 275 isoform 1 [Mus musculus]
mmu-miR-106a-3p	NM_001162417	-28.8	0.381852	-25.86	160	GO:0031981,GO:0003676,	K12887|1|1e-108|394|ecb:100057979|heterogeneous nuclear ribonucleoprotein M	gi|244790087|ref|NP_001155889.1|	0	myelin expression factor 2 isoform 1 [Mus musculus]

mmu-miR-106a-3p	NM_001162418	-28.8	0.450711	-25.86	160	GO:0043231,	K12887|1|1e-96|355|mgp:100550825|heterogeneous nuclear ribonucleoprotein M	gi|244790095|ref|NP_001155890.1|	0	myelin expression factor 2 isoform 3 [Mus musculus]
mmu-miR-106a-3p	NM_001162533	-35.1	0.02878	-30.08	184	-	K12470|1|1e-27|125|gga:418608|SH3 domain-containing kinase-binding protein 1!K13738|2|1e-26|122|ssc:100627990|CD2-associated protein	gi|148698353|gb|EDL30300.1|	0	RIKEN cDNA 1700029G01, isoform CRA_b [Mus musculus]
mmu-miR-106a-3p	NM_001163617	-26.2	0.775556	-44.93	312	GO:0044429,GO:0004812,GO:0032559,GO:0006418,	K01868|1|0.0|1430|mmu:71807|threonyl-tRNA synthetase [EC:6.1.1.3]	gi|254692867|ref|NP_001157089.1|	0	threonine--tRNA ligase, mitochondrial isoform 2 [Mus musculus]

mmu-miR-106a-3p	NM_001163618	-26.2	0.750985	-44.93	312	GO:0044429,GO:0004812,GO:0032559,GO:0006418,	K01868|1|0.0|996|rno:310672|threonyl-tRNA synthetase [EC:6.1.1.3]	gi|254692871|ref|NP_001157090.1|	0	threonine--tRNA ligase, mitochondrial isoform 3 [Mus musculus]
mmu-miR-106a-3p	NM_001163619	-26.2	0.86835	-44.93	312	GO:0044429,GO:0004812,GO:0032559,GO:0006418,	K01868|1|0.0|707|mmu:71807|threonyl-tRNA synthetase [EC:6.1.1.3]	gi|254692873|ref|NP_001157091.1|	0	threonine--tRNA ligase, mitochondrial isoform 4 [Mus musculus]
mmu-miR-106a-3p	NM_001163688	-27.4	0.726704	-44.55	315	GO:0016021,GO:0004871,GO:0004725,GO:0005515,GO:0006470,GO:0045687,GO:0006935,GO:0007169,	K01104|1|0.0|1493|mmu:19262|protein-tyrosine phosphatase [EC:3.1.3.48]	gi|148696327|gb|EDL28274.1|	0	protein tyrosine phosphatase, receptor type, A, isoform CRA_c [Mus musculus]
mmu-miR-106a-3p	NM_001163730	-34.2	0.044442	-29.8	171	-	K10523|1|8e-109|395|mgp:100544737|speckle-type POZ protein	gi|254910979|ref|NP_001157202.1|	0	TD and POZ domain containing-like [Mus musculus]

mmu-miR-106a-3p	NM_001163750	-31.5	0.109922	-25.67	166	-	K10345|1|0.0|848|mmu:79043|SPRY domain-containing SOCS box protein 3	gi|254939631|ref|NP_001157222.1|	0	SPRY domain-containing SOCS box protein 3 isoform 1 [Mus musculus]
mmu-miR-106a-3p	NM_001163755	-25.9	0.917134	-27.41	170	GO:0043231,GO:0043234,GO:0043005,GO:0044297,GO:0003712,GO:0005515,GO:0006357,GO:0030509,GO:0007179,	-	gi|255683429|ref|NP_001157227.1|	0	SKI family transcriptional corepressor 1 isoform 2 [Mus musculus]
mmu-miR-106a-3p	NM_001163757	-25.9	0.916972	-27.41	170	GO:0043231,GO:0043234,GO:0043005,GO:0044297,GO:0003712,GO:0005515,GO:0006357,GO:0030509,GO:0007179,	-	gi|255683431|ref|NP_001157229.1|	0	SKI family transcriptional corepressor 1 isoform 3 [Mus musculus]

mmu-miR-106a-3p	NM_001163758	-25.9	0.916103	-27.41	170	GO:0043231,GO:0043234,GO:0043005,GO:0044297,GO:0003712,GO:0005515,GO:0006357,GO:0030509,GO:0007179,	-	gi|255683426|ref|NP_766034.2|	0	SKI family transcriptional corepressor 1 isoform 1 [Mus musculus]
mmu-miR-106a-3p	NM_001164059	-25.1	0.885429	-45.64	328	GO:0009986,GO:0016021,GO:0046625,GO:0005539,GO:0019899,GO:0007599,GO:0002682,GO:0010033,GO:0009987,	K06495|1|0.0|736|mmu:20343|selectin, lymphocyte	gi|255708471|ref|NP_001157531.1|	0	L-selectin isoform 2 precursor [Mus musculus]
mmu-miR-106a-3p	NM_001164316	-27.6	0.848278	-26.75	169	-	-	gi|256222323|ref|NP_899133.2|	0	protein FAM190A [Mus musculus]

mmu-miR-106a-3p	NM_001164361	-26.9	0.946793	-28.14	174	GO:0044424,GO:0005319,GO:0008289,GO:0006869,	K08051|1|0.0|972|rno:500132|pleckstrin homology domain containing, family A (phosphoinositide binding specific) member 8	gi|256355025|ref|NP_001157833.1|	0	pleckstrin homology domain-containing family A member 8 isoform 1 [Mus musculus]
mmu-miR-106a-3p	NM_001165954	-26.9	0.988537	-50.7	335	GO:0046872,	K11458|1|0.0|1244|mmu:241915|polyhomeotic-like protein 3	gi|260099711|ref|NP_001159426.1|	0	polyhomeotic-like protein 3 isoform 1 [Mus musculus]
mmu-miR-106a-3p	NM_001165955	-26.9	0.988216	-50.7	335	GO:0005488,	K11458|1|0.0|1202|mmu:241915|polyhomeotic-like protein 3	gi|260099711|ref|NP_001159426.1|	0	polyhomeotic-like protein 3 isoform 1 [Mus musculus]
mmu-miR-106a-3p	NM_001165956	-26.9	0.988052	-50.7	335	GO:0005488,	K11458|1|0.0|1142|mmu:241915|polyhomeotic-like protein 3	gi|260099715|ref|NP_001159428.1|	0	polyhomeotic-like protein 3 isoform 4 [Mus musculus]

mmu-miR-106a-3p	NM_001166207	-34.8	0.057373	-28.91	185	GO:0031224,	K13145|1|2e-15|86.3|ptr:464291|integrator complex subunit 8	gi|148693341|gb|EDL25288.1|	0	mCG6268, isoform CRA_a [Mus musculus]
mmu-miR-106a-3p	NM_001166634	-25.8	0.454028	-25.4	151	GO:0005681,GO:0043566,GO:0006396,GO:0006406,	K12881|1|4e-106|384|mmu:100043292|THO complex	gi|262263334|ref|NP_001160106.1|	4.41E-119	uncharacterized protein LOC100043227 [Mus musculus]
mmu-miR-106a-3p	NM_001166638	-25.8	0.446612	-25.4	151	GO:0005681,GO:0043566,GO:0006396,GO:0006406,	K12881|1|4e-106|384|mmu:100043292|THO complex	gi|262263339|ref|NP_001160110.1|	8.36E-115	uncharacterized protein LOC100043231 [Mus musculus]
mmu-miR-106a-3p	NM_001166639	-25.8	0.446612	-25.4	151	GO:0005681,GO:0043566,GO:0006396,GO:0006406,	K12881|1|4e-106|384|mmu:100043292|THO complex	gi|262263339|ref|NP_001160110.1|	8.36E-115	uncharacterized protein LOC100043231 [Mus musculus]
mmu-miR-106a-3p	NM_001166641	-25.8	0.446612	-25.4	151	GO:0005681,GO:0043566,GO:0006396,GO:0006406,	K12881|1|4e-106|384|mmu:100043292|THO complex	gi|262263339|ref|NP_001160110.1|	8.36E-115	uncharacterized protein LOC100043231 [Mus musculus]

mmu-miR-106a-3p	NM_001166722	-29.6	0.09492	-25.1	161	GO:0031224,GO:0004930,GO:0007166,	K04614|1|2e-159|561|mmu:546944|vomeronasal1 receptor	gi|285397745|ref|NP_001160194.1|	1.63E-158	vomeronasal 1 receptor 170 [Mus musculus]
mmu-miR-106a-3p	NM_001167939	-25.5	0.984838	-25.78	168	GO:0044427,GO:0043234,GO:0031981,GO:0005515,GO:0007064,	K11266|1|0.0|1234|mmu:74549|MAternally affected uncoordination	gi|269308215|ref|NP_001161411.1|	0	MAU2 chromatid cohesion factor homolog isoform 1 [Mus musculus]
mmu-miR-106a-3p	NM_001170742	-28.7	0.671371	-48.49	321	-	K14848|1|4e-14|82.4|hmg:100199874|ribosome assembly protein RRB1	gi|50511187|dbj|BAD32579.1|	0	mKIAA1923 protein [Mus musculus]
mmu-miR-106a-3p	NM_001170743	-28.7	0.675363	-48.49	321	-	K14848|1|4e-14|82.4|hmg:100199874|ribosome assembly protein RRB1	gi|50511187|dbj|BAD32579.1|	0	mKIAA1923 protein [Mus musculus]

mmu-miR-106a-3p	NM_001174107	-32.8	0.372966	-29.34	171	GO:0004712,GO:0042802,GO:0004709,GO:0032559,GO:0000187,	K04417|1|0.0|1793|mmu:338372|mitogen-activated protein kinase kinase kinase 9 [EC:2.7.11.25]	gi|291575174|ref|NP_001167578.1|	0	mitogen-activated protein kinase kinase kinase 9 isoform 1 [Mus musculus]
mmu-miR-106a-3p	NM_001177535	-25.8	0.580034	-25.4	151	GO:0005681,GO:0043566,GO:0006396,GO:0006406,	K12881|1|2e-131|469|mmu:100043292|THO complex	gi|294712530|ref|NP_001171006.1|	1.36E-130	THO complex 4-like [Mus musculus]
mmu-miR-106a-3p	NM_001177616	-31.7	0.191552	-28.07	173	GO:0044424,GO:0009408,	K02360|1|1e-33|146|aag:AaeL_AAEL009189|a component of the cytoplasm (encore)	gi|294979157|ref|NP_001171087.1|	0	cAMP-regulated phosphoprotein 21 isoform 4 [Mus musculus]
mmu-miR-106a-3p	NM_001177617	-31.7	0.154497	-28.07	173	GO:0044424,GO:0009408,	K02360|1|3e-19|98.2|aag:AaeL_AAEL009189|a component of the cytoplasm (encore)	gi|294979163|ref|NP_001171088.1|	0	cAMP-regulated phosphoprotein 21 isoform 5 [Mus musculus]

mmu-miR-106a-3p	NM_001177619	-31.7	0.141438	-28.07	173	GO:0044424,GO:0009408,	K02360|1|7e-26|120|aag:AaeL_AAEL009189|a component of the cytoplasm (encore)	gi|294979168|ref|NP_001171090.1|	7.69E-167	cAMP-regulated phosphoprotein 21 isoform 6 [Mus musculus]
mmu-miR-106a-3p	NM_001177623	-31.7	0.14447	-28.07	173	GO:0044424,GO:0009408,	K02360|1|1e-19|99.4|aag:AaeL_AAEL009189|a component of the cytoplasm (encore)	gi|294979177|ref|NP_001171094.1|	4.53E-106	cAMP-regulated phosphoprotein 21 isoform 7 [Mus musculus]
mmu-miR-106a-3p	NM_001024602	-20.2	1	-50.16	342	GO:0005856,	K13108|1|5e-07|58.9|cqu:CpipJ_CPIJ010869|smad nuclear-interacting protein 1	gi|28972129|dbj|BAC65518.1|	0	mKIAA0284 protein [Mus musculus]

mmu-miR-106a-5p	NM_001017959	-28.9	0.237959	-28.11	166	GO:0005768,GO:0030667,GO:0030666,GO:0005764,GO:0031224,	K06528|1|0.0|738|mmu:16784|lysosomal-associated membrane protein 1/2	gi|63054837|ref|NP_001017959.1|	0	lysosome-associated membrane glycoprotein 2 isoform 1 precursor [Mus musculus]
mmu-miR-106a-5p	NM_001024139	-28	0.773106	-26	169	GO:0031012,GO:0046914,GO:0004175,GO:0019538,	K08629|1|0.0|1966|mmu:235130|a disintegrin and metalloproteinase with thrombospondin motifs 15 [EC:3.4.24.-]	gi|71725383|ref|NP_001019310.1|	0	A disintegrin and metalloproteinase with thrombospondin motifs 15 precursor [Mus musculus]
mmu-miR-106a-5p	NM_001025378	-28.1	0.51199	-25.82	163	GO:0000808,GO:0031981,GO:0000785,GO:0000777,GO:0043565,GO:0007050,GO:0006357,GO:0006259,GO:0051320,	K02604|1|0.0|942|mmu:18393|origin recognition complex subunit 2	gi|70909306|ref|NP_001020549.1|	0	origin recognition complex subunit 2 isoform B [Mus musculus]

mmu-miR-106a-5p	NM_001033475	-26.3	0.969222	-25.42	172	-	-	gi|84794627|ref|NP_001028647.1|	7.75E-154	protein TMED8 [Mus musculus]
mmu-miR-106a-5p	NM_001034097	-36.2	0.014367	-34.38	176	-	K05475|1|2e-105|384|mmu:69583|tumor necrosis factor ligand superfamily member	gi|77539772|ref|NP_001029269.1|	0	tumor necrosis factor (ligand) superfamily, member 12-member 13 isoform a [Mus musculus]
mmu-miR-106a-5p	NM_001034867	-32.4	0.208424	-27.76	180	GO:0003824,	-	gi|298493323|ref|NP_001030039.2|	0	peptidase M20 domain-containing protein 2 [Mus musculus]
mmu-miR-106a-5p	NM_001037928	-27	0.59251	-25.7	164	-	-	gi|83776575|ref|NP_001033017.1|	2.35E-154	uncharacterized protein C7orf57 homolog [Mus musculus]
mmu-miR-106a-5p	NM_001077354	-28.1	0.915851	-25.61	175	GO:0042575,GO:0034061,GO:0008408,GO:0000278,GO:0006281,GO:0051320,	K02350|1|2e-28|130|bta:517422|DNA polymerase zeta subunit [EC:2.7.7.7]	gi|148539922|ref|NP_001070822.2|	0	uncharacterized protein KIAA2022 [Mus musculus]

mmu-miR-106a-5p	NM_001081256	-31.1	0.400453	-31.46	162	GO:0031981,GO:0043169,GO:0016701,GO:0006351,GO:0006325,	K15601|1|0.0|3234|mmu:277250|lysine-specific demethylase 3 [EC:1.14.11.-]	gi|124486935|ref|NP_001074725.1|	0	lysine-specific demethylase 3B [Mus musculus]
mmu-miR-106a-5p	NM_001099636	-26.9	0.806772	-26.26	159	-	-	gi|153791731|ref|NP_001093106.1|	0	PNMA-like protein 2 [Mus musculus]
mmu-miR-106a-5p	NM_001128170	-26.5	0.971	-43.72	299	GO:0005874,GO:0044444,GO:0030529,GO:0009898,GO:0046914,GO:0005198,GO:0019783,GO:0016790,GO:0019900,GO:0000278,GO:0016579,GO:0001818,GO:0032886,GO:0043433,GO:0030178,GO:0042345,GO:00	K08601|1|0.0|1884|mmu:74256|ubiquitin thioesterase CYLD [EC:3.1.2.15]	gi|28972435|dbj|BAC65671.1|	0	mKIAA0849 protein [Mus musculus]
mmu-miR-106a-5p	NM_001134299	-27.8	0.551808	-25.6	156	-	-	gi|148668757|gb|EDL01004.1|	1.61E-123	mCG129471 [Mus musculus]

mmu-miR-106a-5p	NM_001134383	-28.8	0.557748	-25.68	169	GO:0043231,GO:0005083,GO:0032011,	K12495|1|0.0|1538|rno:686590|IQ motif and SEC7 domain-containing protein	gi|197304780|ref|NP_001127855.1|		0	IQ motif and SEC7 domain-containing protein 1 isoform a [Mus musculus]
mmu-miR-106a-5p	NM_001134384	-28.8	0.689508	-25.68	169	-	K12495|1|0.0|1498|hsa:9922|IQ motif and SEC7 domain-containing protein	gi|197304784|ref|NP_001127856.1|		0	IQ motif and SEC7 domain-containing protein 1 isoform b [Mus musculus]
mmu-miR-106a-5p	NM_001145902	-28.5	0.438377	-25.85	167	GO:0005815,GO:0031252,GO:0043227,GO:0016020,GO:0005100,GO:0030674,GO:0019904,GO:0032321,	K01514|1|2e-20|102|mdo:100017806|exopolyphosphatase [EC:3.6.1.11]!K10360|2|1e-19|99.8|rno:171296|myosin IX	gi|225543424|ref|NP_001139374.1|		0	rho GTPase-activating protein 1 isoform 1 [Mus musculus]

mmu-miR-106a-5p	NM_001159503	-36.2	0.014311	-34.38	176	-	K05475|1|7e-107|389|mmu:69583|tumor necrosis factor ligand superfamily member	gi|226958391|ref|NP_001152975.1|	0	tumor necrosis factor (ligand) superfamily, member 12-member 13 isoform c [Mus musculus]
mmu-miR-106a-5p	NM_001164224	-28.4	0.258181	-25.35	161	GO:0012505,GO:0044459,GO:0044444,GO:0005488,GO:0010827,GO:0033500,GO:00150	K15627|1|0.0|799|mmu:68938|tether containing UBX domain for GLUT4	gi|148702838|gb|EDL34785.1|	0	alveolar soft part sarcoma chromosome region, candidate 1 (human), isoform CRA_a [Mus musculus]
mmu-miR-106a-5p	NM_001167691	-25.2	0.73526	-25.55	161	GO:0044429,GO:0051287,GO:0016763,GO:0016810,GO:0046914,GO:0033558,GO:0050796,GO:0016458,GO:0035601,GO:00063	K11414|1|0.0|690|mmu:75387|NAD-dependent deacetylase sirtuin 4 [EC:3.5.1.-]	gi|18490478|gb|AAH22653.1|	0	Sirt4 protein, partial [Mus musculus]

mmu-miR-106a-5p	NM_001168621	-28.6	0.581528	-25.58	167	GO:0000323,GO:0044459,GO:0008021,GO:0046914,GO:0003824,	K10694|1|2e-134|481|mmu:170737|E3 ubiquitin-protein ligase ZNRF [EC:6.3.2.19]	gi|148679554|gb|EDL11501.1|	3.35E-152	zinc and ring finger 1, isoform CRA_a [Mus musculus]
mmu-miR-106a-5p	NM_001168622	-28.6	0.657547	-25.58	167	GO:0000323,GO:0044459,GO:0008021,GO:0046914,GO:0003824,	K10694|1|4e-109|397|mmu:170737|E3 ubiquitin-protein ligase ZNRF [EC:6.3.2.19]	gi|148679554|gb|EDL11501.1|	7.94E-127	zinc and ring finger 1, isoform CRA_a [Mus musculus]
mmu-miR-106a-5p	NM_001168623	-28.6	0.50744	-25.58	167	GO:0043231,GO:0016020,GO:0031410,GO:0045202,GO:0046872,	K10694|1|2e-47|192|ptr:736544|E3 ubiquitin-protein ligase ZNRF [EC:6.3.2.19]	gi|274321886|ref|NP_001162094.1|	8.06E-72	E3 ubiquitin-protein ligase ZNRF1 isoform c [Mus musculus]

mmu-miR-106a-5p	NM_001174107	-29.1	0.807503	-27.62	167	GO:0004712,GO:0042802,GO:0004709,GO:0032559,GO:0000187,	K04417|1|0.0|1793|mmu:338372|mitogen-activated protein kinase kinase kinase 9 [EC:2.7.11.25]	gi|291575174|ref|NP_001167578.1|	0	mitogen-activated protein kinase kinase kinase 9 isoform 1 [Mus musculus]
mmu-miR-106a-5p	NM_001177579	-27.8	0.595423	-25.6	156	-	-	gi|148668757|gb|EDL01004.1|	8.01E-111	mCG129471 [Mus musculus]
mmu-miR-106a-5p	NM_001205239	-27.9	0.500308	-26.84	176	GO:0016763,	K15258|1|0.0|1186|mmu:67287|poly [ADP-ribose] polymerase 6/8 [EC:2.4.2.30]	gi|327365350|ref|NP_001192168.1|	0	poly [ADP-ribose] polymerase 6 isoform 1 [Mus musculus]
mmu-miR-106a-5p	NM_001271526	-28.1	0.572733	-25.82	163	-	-	-	-	-

mmu-miR-106a-5p	NM_008155	-27.5	0.581528	-25.49	163	GO:0031981,GO:0016020,GO:0044421,GO:0044444,GO:0005102,GO:0016861,GO:0006007,GO:0006955,GO:0007610,GO:0009438,GO:0050878,GO:0048646,GO:0043066,GO:00	K01810|1|0.0|1137|mmu:14751|glucose-6-phosphate isomerase [EC:5.3.1.9]	gi|254553458|ref|NP_032181.2|	0	glucose-6-phosphate isomerase [Mus musculus]
mmu-miR-106a-5p	NM_008709	-26.3	0.733837	-25.03	162	GO:0043231,GO:0044427,GO:0003676,GO:0030323,GO:0016337,GO:0008219,GO:0006355,GO:0001763,GO:0048562,GO:0008284,GO:0030326,	K09109|1|2e-169|597|mmu:18109|N-myc proto-oncogene protein	gi|148666033|gb|EDK98449.1|	0	v-myc myelocytomatosis viral related oncogene, neuroblastoma derived (avian), isoform CRA_a [Mus musculus]

mmu-miR-106a-5p	NM_008765	-28.1	0.573376	-25.82	163	GO:0000808,GO:0031981,GO:0000785,GO:0000777,GO:0043565,GO:0007050,GO:0006357,GO:0006259,GO:0051320,	K02604|1|0.0|949|rno:301430|origin recognition complex subunit 2	gi|6679176|ref|NP_032791.1|	0	origin recognition complex subunit 2 isoform A [Mus musculus]
mmu-miR-106a-5p	NM_010508	-30.6	0.46012	-27.91	167	GO:0016021,GO:0016879,GO:0004904,GO:0051707,GO:0007243,GO:0001819,GO:0042089,GO:0001959,GO:0046649,GO:00022	K05130|1|0.0|1135|mmu:15975|interferon receptor 1	gi|148671880|gb|EDL03827.1|	0	interferon (alpha and beta) receptor 1, isoform CRA_c [Mus musculus]

mmu-miR-106a-5p	NM_010927	-27.4	0.723175	-26.65	164	GO:0042579,GO:0005856,GO:0000166,GO:0016597,GO:0050662,GO:0042802,GO:0005506,GO:0016709,GO:0019887,GO:0032496,GO:0009611,GO:0048771,GO:0033280,GO:0050673,GO:0008015,GO:0044057,GO:0016310,GO:0043627,GO:0042742,GO:0022602,GO:0044271,GO:00170	K13241|1|0.0|2334|mmu:18126|nitric-oxide synthase, inducible [EC:1.14.13.39]	gi|6754872|ref|NP_035057.1|	0	nitric oxide synthase, inducible [Mus musculus]

mmu-miR-106a-5p	NM_011614	-36.2	0.009483	-34.38	176	GO:0016021,GO:0044444,GO:0044421,GO:0032813,GO:0016477,GO:0012502,GO:0048869,GO:0023052,GO:0002376,GO:0045765,GO:0001935,	K05474|1|1e-91|338|mmu:21944|tumor necrosis factor ligand superfamily member 12	gi|74221419|dbj|BAE42185.1|	7.03E-91	unnamed protein product [Mus musculus]
mmu-miR-106a-5p	NM_013889	-28.9	0.81405	-27.12	160	GO:0043231,GO:0046914,GO:0003676,GO:0010467,	K09228|1|9e-17|92.0|rno:170849|KRAB domain-containing zinc finger protein	gi|169234810|ref|NP_038917.2|	0	zinc finger protein 292 [Mus musculus]
mmu-miR-106a-5p	NM_018821	-26.4	0.792937	-41.83	306	GO:0044424,GO:0005488,GO:0006950,GO:0042593,GO:0007243,	K10322|1|0.0|968|mmu:54607|suppressor of cytokine signaling 6	gi|12860434|dbj|BAB31956.1|	0	unnamed protein product [Mus musculus]

mmu-miR-106a-5p	NM_021435	-28.3	0.557089	-26.35	175	GO:0012505,GO:0043231,GO:0031224,GO:0015165,GO:0015781,	K15278|1|4e-179|629|mmu:58246|solute carrier family 35 (UDP-xylose/UDP-N-acetylglucosamine transporter), member B4	gi|10946804|ref|NP_067410.1|	2.91E-178	UDP-xylose and UDP-N-acetylglucosamine transporter [Mus musculus]
mmu-miR-106a-5p	NM_026877	-28.4	0.305407	-25.35	161	GO:0012505,GO:0044459,GO:0044444,GO:0005488,GO:0010827,GO:0033500,GO:00150	K15627|1|0.0|786|mmu:68938|tether containing UBX domain for GLUT4	gi|30794438|ref|NP_081153.1|	0	tether containing UBX domain for GLUT4 isoform 1 [Mus musculus]
mmu-miR-106a-5p	NM_027421	-26.8	0.909207	-25.32	156	GO:0016591,GO:0005635,GO:0031224,GO:0005488,GO:0016073,	K13139|1|0.0|2277|mmu:70422|integrator complex subunit 2	gi|148683836|gb|EDL15783.1|	0	integrator complex subunit 2, isoform CRA_b [Mus musculus]

mmu-miR-106a-5p	NM_029508	-34.3	0.018762	-31.97	180	GO:0005815,GO:0046914,GO:0010467,	K11489|1|4e-124|445|mmu:76073|polycomb group RING finger protein 5	gi|148709823|gb|EDL41769.1|	7.08E-124	polycomb group ring finger 5, isoform CRA_b [Mus musculus]
mmu-miR-106a-5p	NM_029922	-27.9	0.491853	-26.84	176	GO:0016763,	K15258|1|0.0|1135|pon:100171860|poly [ADP-ribose] polymerase 6/8 [EC:2.4.2.30]	gi|112807189|ref|NP_084198.2|	0	poly [ADP-ribose] polymerase 6 isoform 2 [Mus musculus]
mmu-miR-106a-5p	NM_054087	-31.2	0.204713	-28.13	175	-	K14610|1|0.0|903|mmu:116914|solute carrier family 19 (thiamine transporter), member 2/3	gi|16905117|ref|NP_473428.1|	0	thiamine transporter 1 [Mus musculus]

mmu-miR-106a-5p	NM_054097	-28.1	0.55282	-45.44	327	GO:0043231,GO:0005515,GO:0016307,GO:0032559,GO:0046488,GO:0046474,GO:0006796,	K00920|1|0.0|650|mmu:117150|1-phosphatidylinositol-5-phosphate 4-kinase [EC:2.7.1.149]	gi|17298686|ref|NP_473438.1|	0	phosphatidylinositol 5-phosphate 4-kinase type-2 gamma [Mus musculus]
mmu-miR-106a-5p	NM_133206	-28.6	0.663863	-25.58	167	GO:0000323,GO:0044459,GO:0008021,GO:0046914,GO:0003824,	K10694|1|2e-134|481|mmu:170737|E3 ubiquitin-protein ligase ZNRF [EC:6.3.2.19]	gi|148679554|gb|EDL11501.1|	4.24E-152	zinc and ring finger 1, isoform CRA_a [Mus musculus]
mmu-miR-106a-5p	NM_133760	-25.2	0.712998	-25.55	161	GO:0044429,GO:0051287,GO:0016763,GO:0016810,GO:0046914,GO:0033558,GO:0050796,GO:0016458,GO:0035601,GO:00063	K11414|1|0.0|690|mmu:75387|NAD-dependent deacetylase sirtuin 4 [EC:3.5.1.-]	gi|18490478|gb|AAH22653.1|	0	Sirt4 protein, partial [Mus musculus]
mmu-miR-106a-5p	NM_144853	-28.3	0.343275	-25.49	164	-	-	gi|74190182|dbj|BAE37209.1|	1.62E-68	unnamed protein product [Mus musculus]

mmu-miR-106a-5p	NM_146124	-28.5	0.463442	-25.85	167	GO:0005815,GO:0031252,GO:0043227,GO:0016020,GO:0005100,GO:0030674,GO:0019904,GO:0032321,	K01514|1|2e-20|102|mdo:100017806|exopolyphosphatase [EC:3.6.1.11]!K10360|2|1e-19|99.8|rno:171296|myosin IX	gi|225543424|ref|NP_001139374.1|	0	rho GTPase-activating protein 1 isoform 1 [Mus musculus]
mmu-miR-106a-5p	NM_172566	-28.7	0.448505	-26.67	166	-	-	gi|262263316|ref|NP_766154.2|	0	RUN domain-containing protein 1 [Mus musculus]
mmu-miR-106a-5p	NM_172569	-25.5	0.929857	-26.2	160	GO:0043231,GO:0046914,	K13056|1|2e-06|56.2|xtr:549576|CCCH zinc finger protein	gi|148702600|gb|EDL34547.1|	0	zinc finger CCCH type containing 5, isoform CRA_b [Mus musculus]

mmu-miR-106a-5p	NM_172838	-27.2	0.741909	-41.96	316	GO:0031224,GO:0015291,GO:0006810,	K11810|1|0.0|984|mmu:240638|MFS transporter, MCP family, solute carrier family 16 (monocarboxylic acid transporters), member 12	gi|27370260|ref|NP_766426.1|	0	monocarboxylate transporter 12 [Mus musculus]
mmu-miR-106a-5p	NM_173369	-26.5	0.970901	-43.72	299	GO:0005874,GO:0044444,GO:0030529,GO:0009898,GO:0046914,GO:0005198,GO:0019783,GO:0016790,GO:0019900,GO:0000278,GO:0016579,GO:0001818,GO:0032886,GO:0043433,GO:0030178,GO:0042345,GO:00	K08601|1|0.0|1874|mmu:74256|ubiquitin thioesterase CYLD [EC:3.1.2.15]	gi|28972435|dbj|BAC65671.1|	0	mKIAA0849 protein [Mus musculus]

mmu-miR-106a-5p	NM_173767	-30.7	0.158368	-25.63	161	GO:0044424,GO:0048731,GO:0048869,	-	gi|116248173|sp|Q3HNM7.2|INSC_MOUSE	0	RecName: Full=Protein inscuteable homolog; AltName: Full=Minsc
mmu-miR-106a-5p	NM_178069	-33.9	0.042315	-29.52	166	GO:0016604,GO:0032561,GO:0003824,GO:0006913,	K14539|1|0.0|1234|mmu:224092|large subunit GTPase 1 [EC:3.6.1.-]	gi|30017373|ref|NP_835170.1|	0	large subunit GTPase 1 homolog [Mus musculus]

mmu-miR-106a-5p	NM_178143	-26	0.987624	-26.29	164	GO:0031981,GO:0043234,GO:0044459,GO:0044444,GO:0005515,GO:0004674,GO:0032559,GO:0043169,GO:0015980,GO:0010243,GO:0006694,GO:0008203,GO:0006796,GO:0006633,GO:0007169,GO:0051259,GO:0003333,GO:0022402,GO:0006464,GO:0019217,GO	K07198|1|0.0|1079|mmu:108079|5'-AMP-activated protein kinase, catalytic alpha subunit [EC:2.7.11.11]	gi|227452236|ref|NP_835279.2|	0	5'-AMP-activated protein kinase catalytic subunit alpha-2 [Mus musculus]

mmu-miR-106a-5p	NM_183034	-35.4	0.060664	-55.86	337	GO:0044424,GO:0043169,GO:0007165,	K15348|1|2e-11|73.6|bfo:BRAFLDRAFT_121069|pleckstrin homology domain-containing family M member 2	gi|33942120|ref|NP_898855.1|	0	pleckstrin homology domain-containing family M member 1 [Mus musculus]
mmu-miR-106a-5p	NM_198223	-28.4	0.268911	-25.35	161	GO:0012505,GO:0044459,GO:0044444,GO:0005488,GO:0010827,GO:0033500,GO:00150	K15627|1|0.0|786|mmu:68938|tether containing UBX domain for GLUT4	gi|148702838|gb|EDL34785.1|	0	alveolar soft part sarcoma chromosome region, candidate 1 (human), isoform CRA_a [Mus musculus]
mmu-miR-106a-5p	NR_027371	-28.5	0.44752	-25.85	167	GO:0005815,GO:0031252,GO:0043227,GO:0016020,GO:0005100,GO:0030674,GO:0019904,GO:0032321,	K01514|1|2e-20|102|mdo:100017806|exopolyphosphatase [EC:3.6.1.11]!K10360|2|1e-19|99.8|rno:171296|myosin IX	gi|225543424|ref|NP_001139374.1|	0	rho GTPase-activating protein 1 isoform 1 [Mus musculus]

mmu-miR-106a-5p	NR_027372	-28.5	0.445705	-25.85	167	GO:0005815,GO:0031252,GO:0043227,GO:0016020,GO:0005100,GO:0030674,GO:0019904,GO:0032321,	K01514|1|2e-20|102|mdo:100017806|exopolyphosphatase [EC:3.6.1.11]!K10360|2|1e-19|99.8|rno:171296|myosin IX	gi|225543424|ref|NP_001139374.1|	0	rho GTPase-activating protein 1 isoform 1 [Mus musculus]
mmu-miR-106a-5p	NR_027373	-28.5	0.449328	-25.85	167	GO:0005815,GO:0031252,GO:0043227,GO:0016020,GO:0005100,GO:0030674,GO:0019904,GO:0032321,	K01514|1|2e-20|102|mdo:100017806|exopolyphosphatase [EC:3.6.1.11]!K10360|2|1e-19|99.8|rno:171296|myosin IX	gi|225543424|ref|NP_001139374.1|	0	rho GTPase-activating protein 1 isoform 1 [Mus musculus]
mmu-miR-106a-5p	NR_028280	-28.7	0.470466	-26.78	162	-	-	gi|255308901|ref|NP_848889.3|	3.00E-129	proline-rich protein 18 [Mus musculus]

mmu-miR-106a-5p	NR_038074	-27.9	0.481788	-26.84	176	GO:0016763,	K15258|1|0.0|862|pon:100171860|poly [ADP-ribose] polymerase 6/8 [EC:2.4.2.30]	gi|112807189|ref|NP_084198.2|	0	poly [ADP-ribose] polymerase 6 isoform 2 [Mus musculus]
mmu-miR-106a-5p	NR_038075	-27.9	0.479895	-26.84	176	GO:0016763,	K15258|1|0.0|850|cfa:478358|poly [ADP-ribose] polymerase 6/8 [EC:2.4.2.30]	gi|148694028|gb|EDL25975.1|	0	mCG131573, isoform CRA_b [Mus musculus]
mmu-miR-106a-5p	NR_045471	-31.2	0.131966	-27.94	178	-	-	gi|148679699|gb|EDL11646.1|	3.20E-87	mCG144621 [Mus musculus]
mmu-miR-106a-5p	NR_045801	-28.1	0.334239	-25.9	175	-	-	gi|12857035|dbj|BAB30867.1|	6.42E-53	unnamed protein product [Mus musculus]

mmu-miR-106a-5p	NM_013770	-23.1	0.999575	-46.88	337	GO:0031224,GO:0031981,GO:0019866,GO:0015103,GO:0005488,GO:0015291,GO:0015556,GO:0015743,GO:0015744,GO:0046907,GO:0006006,GO:0006817,	K13577|1|4e-161|570|mmu:27376|solute carrier family 25 (mitochondrial dicarboxylate transporter), member 10	gi|254826790|ref|NP_038798.2|	2.98E-160	mitochondrial dicarboxylate carrier [Mus musculus]
mmu-miR-106a-5p	NM_028021	-24.3	0.999101	-41.38	318	GO:0043005,GO:0032432,GO:0030427,GO:0042623,GO:0003774,GO:0003779,GO:0032559,GO:0006935,GO:0030029,	K10352|1|0.0|2806|mmu:71960|myosin heavy chain	gi|148690794|gb|EDL22741.1|	0	myosin, heavy polypeptide 14, isoform CRA_b [Mus musculus]

mmu-miR-106a-5p	NM_021286	-24	0.995669	-44.86	302	GO:0044424,GO:0044459,GO:0031224,	K03993|1|9e-23|110|aml:100466924|mannan-binding lectin serine protease 2 [EC:3.4.21.104]!K04011|2|4e-22|108|ecb:100057298|complement component (3b/4b) receptor	gi|31981500|ref|NP_067261.2|	0	seizure protein 6 precursor [Mus musculus]
mmu-miR-106a-5p	NM_019994	-20.4	1	-30.16	178	GO:0043231,GO:0044444,GO:0005099,GO:0046983,GO:0032852,GO:0006351,	K07207|1|1e-18|98.2|bfo:BRAFLDRAFT_64809|tuberous sclerosis 2	gi|51230429|ref|NP_064378.4|	0	ral GTPase-activating protein subunit alpha-1 isoform 2 [Mus musculus]

mmu-miR-106a-5p	NM_172523	-30.7	0.263887	-23.49	177	GO:0016021,GO:0030136,GO:0033267,GO:0030658,GO:0030141,GO:0005275,GO:0005515,GO:0001505,GO:0030072,GO:0007275,GO:0007610,GO:0014075,GO:0015837,GO:00	K08155|1|0.0|919|mmu:214084|MFS transporter, DHA1 family, solute carrier family 18 (vesicular amine transporter), member 1/2	gi|148669876|gb|EDL01823.1|	0	solute carrier family 18 (vesicular monoamine), member 2 [Mus musculus]

mmu-miR-106a-5p	NM_201232	-21.2	1	-26.17	175	GO:0043231,GO:0043234,GO:0019904,GO:0019899,GO:0003007,GO:0000122,GO:0010165,GO:0030278,GO:0042633,GO:0007417,GO:0001822,GO:0035115,GO:0043583,GO:0016575,GO:2000026,GO:0050954,GO:0040008,GO:0008104,GO:0030154,GO:0035112,GO:0007064,GO:00335	K06672|1|0.0|4694|mmu:71175|cohesin loading factor subunit SCC2	gi|51371928|ref|NP_957684.1|	0	nipped-B-like protein isoform B [Mus musculus]
mmu-miR-106a-5p	NM_177593	-26.7	0.276907	-23.27	174	-	K06552|1|8e-32|137|mmu:68891|polycythemia rubra vera protein 1	gi|46559428|ref|NP_808261.1|	1.78E-101	uncharacterized protein LOC210155 [Mus musculus]

mmu-miR-106a-5p	NM_181066	-30	0.293688	-23.48	173	GO:0043231,GO:0005488,GO:0009314,	-	gi|148687162|gb|EDL19109.1|	0	EST AA881470, isoform CRA_c [Mus musculus]
mmu-miR-106a-5p	NR_015580	-22.4	0.999696	-20.49	172	-	-	gi|148703101|gb|EDL35048.1|	3.77E-56	mCG142516, isoform CRA_b [Mus musculus]
mmu-miR-106a-5p	NM_031179	-25	0.994236	-22.63	171	GO:0005681,GO:0016604,GO:0005488,GO:0003002,GO:0000377,	K12828|1|0.0|2598|ptr:459850|splicing factor 3B subunit 1	gi|54112117|ref|NP_036565.2|	0	splicing factor 3B subunit 1 isoform 1 [Homo sapiens]
mmu-miR-106a-5p	NR_024051	-24.9	0.989239	-21.73	170	-	K05643|1|0.0|1113|mdo:100013281|ATP-binding cassette, subfamily A (ABC1), member 3	gi|37935731|gb|AAP73046.1|	0	ATP-binding cassette transporter sub-family A member 16 [Mus musculus]

mmu-miR-106a-5p	NR_015457	-26.4	0.680457	-20.17	169	GO:0016020,GO:0043231,GO:0032870,	-	gi|158706129|sp|Q5HZI1.2|MTUS1_MOUSE	3.78E-32	RecName: Full=Microtubule-associated tumor suppressor 1 homolog; AltName: Full=AT2 receptor-binding protein; AltName: Full=Angiotensin-II type 2 receptor-interacting protein; AltName: Full=Coiled-coiled tumor suppressor gene 1 protein; AltName: Full=Mitochondrial tumor suppressor 1 homolog
mmu-miR-106a-5p	NR_036453	-26.6	0.61026	-23.67	168	-	-	gi|148683765|gb|EDL15712.1|	1.65E-87	RIKEN cDNA E230016K23 [Mus musculus]
mmu-miR-106a-5p	NR_033471	-23.2	0.99552	-22.38	167	GO:0044444,GO:0046914,GO:0004871,GO:0012502,GO:0023052,	K11985|1|6e-161|568|mmu:22036|TRAF-interacting protein	gi|31560535|ref|NP_035764.2|	4.43E-160	TRAF-interacting protein [Mus musculus]
mmu-miR-106a-5p	NR_045795	-24.5	0.930171	-21.2	166	GO:0043231,GO:0031224,GO:0043169,GO:0016772,GO:0046469,GO:0006656,	-	gi|148677466|gb|EDL09413.1|	2.42E-26	mCG147326 [Mus musculus]

mmu-miR-106a-5p	NM_198962	-26.3	0.851111	-20.48	165	GO:0016021,GO:0008528,GO:0007267,GO:0007186,GO:0007610,	K04239|1|0.0|880|mmu:387285|hypocretin (orexin) receptor 2	gi|42821120|ref|NP_945200.1|	0	orexin receptor type 2 [Mus musculus]
mmu-miR-106a-5p	NR_030690	-23.1	0.999992	-25.24	164	-	-	gi|67975423|ref|XP_668908.1|	5.38E-16	hypothetical protein [Plasmodium berghei strain ANKA]
mmu-miR-106a-5p	NR_045380	-23.3	0.988101	-20.42	163	GO:0043231,	-	gi|109730699|gb|AAI17740.1|	7.00E-87	RIKEN cDNA 1700034I23 gene [Mus musculus]
mmu-miR-106a-5p	NR_040314	-22.3	0.99994	-21.8	162	-	-	gi|148675117|gb|EDL07064.1|	1.19E-72	mCG144576 [Mus musculus]
mmu-miR-106a-5p	NM_178739	-26.6	0.794556	-21.83	161	-	K11803|1|7e-142|506|mdo:100010360|WD repeat-containing protein 40A	gi|300192937|ref|NP_848854.3|	0	DDB1- and CUL4-associated factor 12-like protein 1 [Mus musculus]
mmu-miR-106a-5p	NR_027465	-25.9	0.949026	-20.75	160	-	-	gi|226371675|ref|NP_694725.3|	0	WW domain-containing adapter protein with coiled-coil isoform 1 [Mus musculus]
mmu-miR-106a-5p	NR_027643	-25.3	0.930043	-22.96	159	-	-	gi|148702486|gb|EDL34433.1|	2.74E-66	RIKEN cDNA 4932435O22 [Mus musculus]
mmu-miR-106a-5p	NR_045100	-21.9	0.994726	-21.08	158	-	-	gi|148701889|gb|EDL33836.1|	8.20E-21	mCG1037788 [Mus musculus]

mmu-miR-106a-5p	NR_073462	-25	0.874162	-24.25	157	-	-	-	-	-
mmu-miR-106a-5p	NR_045615	-23.1	0.997601	-20.54	156	GO:0043231,GO:0046914,GO:0003676,GO:0010467,	K09228|1|6e-34|147|dre:796272|KRAB domain-containing zinc finger protein	gi|21450221|ref|NP_659082.1|	0	zinc finger protein 410 isoform 1 [Mus musculus]
mmu-miR-106a-5p	NM_207682	-23.6	0.999998	-22.06	155	GO:0012506,GO:0043231,GO:0015630,GO:0030246,GO:0016616,GO:0032559,GO:0003774,GO:0015631,GO:0006098,GO:0007275,GO:0012501,GO:0009207,GO:0019520,GO:0007268,GO:0034643,GO:00080	K10392|1|0.0|3154|ecb:100057126|kinesin family member 1/13/14	gi|86990460|ref|NP_997565.2|	0	kinesin-like protein KIF1B isoform b [Mus musculus]
mmu-miR-106a-5p	NR_015469	-23.8	0.990616	-20.34	154	-	-	gi|148708725|gb|EDL40672.1|	4.96E-14	mCG1041253, isoform CRA_d [Mus musculus]

mmu-miR-106a-5p	NM_178624	-21.6	0.999995	-20.77	153	GO:0044424,GO:0019787,GO:0051704,GO:0032446,	K10268|1|0.0|866|mmu:72179|F-box and leucine-rich repeat protein 2/20	gi|30519963|ref|NP_848739.1|	0	F-box/LRR-repeat protein 2 [Mus musculus]
mmu-miR-106a-5p	NR_040601	-20.6	0.997591	-20.66	152	-	-	gi|148667756|gb|EDL00173.1|	6.85E-45	mCG144506 [Mus musculus]
mmu-miR-106a-5p	NR_073122	-21.4	0.999997	-20.28	151	-	-	-	-	-
mmu-miR-106a-5p	NR_028417	-22.3	0.986718	-20.74	150	-	-	gi|148705637|gb|EDL37584.1|	3.85E-39	mCG146113 [Mus musculus]
mmu-miR-106a-5p	NM_198664	-20.5	1	-20.09	149	GO:0043231,GO:0044459,GO:0031410,GO:0005099,GO:0032313,	K11837|1|7e-11|71.2|hsa:9098|ubiquitin carboxyl-terminal hydrolase 6/32 [EC:3.1.2.15]!K13136|2|3e-08|62.4|nvi:100114269|gem associated protein 8	gi|111038126|ref|NP_941066.3|	0	TBC1 domain family member 2A [Mus musculus]
mmu-miR-106a-5p	NM_178671	-22.1	0.999887	-20.95	148	-	-	gi|30520049|ref|NP_848786.1|	1.42E-140	UBX domain-containing protein 10 [Mus musculus]

mmu-miR-106a-5p	NM_153178	-20	1	-20.87	147	GO:0043231,GO:0031332,GO:0044444,GO:0034518,GO:0043169,GO:0016891,GO:0000339,GO:0008135,GO:0006379,GO:0048869,GO:0035196,GO:0032501,GO:0006446,GO:00	K11593|1|0.0|1762|mmu:239528|eukaryotic translation initiation factor 2C	gi|219842353|ref|NP_694818.3|	0	protein argonaute-2 [Mus musculus]
mmu-miR-106a-5p	NR_045164	-22.3	0.999795	-20.67	146	GO:0044424,GO:0005488,GO:0001071,GO:0006351,	-	gi|148675469|gb|EDL07416.1|	4.79E-61	mCG58991 [Mus musculus]
mmu-miR-106a-5p	NM_175938	-23.6	0.992238	-21.64	145	GO:0031224,	K06712|1|0.0|983|mmu:238555|butyrophilin	gi|148700619|gb|EDL32566.1|	0	butyrophilin, subfamily 2, member A2, isoform CRA_b [Mus musculus]

mmu-miR-106a-5p	NM_175185	-22.2	0.999914	-20.54	144	GO:0043231,GO:0003824,GO:0005488,GO:0008152,	K00044|1|1e-63|245|xla:495218|estradiol 17beta-dehydrogenase [EC:1.1.1.62]!K00100|4|2e-56|222|spu:593779|[EC:1.1.1.-	gi|30424792|ref|NP_780394.1|	0	inactive hydroxysteroid dehydrogenase-like protein 1 [Mus musculus]
mmu-miR-106a-5p	NM_027937	NA	NA	-21.22	161	GO:0044424,GO:0005515,GO:0023052,	K15502|1|6e-34|148|aag:AaeL_AAEL014742|serine/threonine-protein phosphatase 6 regulatory ankyrin repeat subunit A	gi|161168996|ref|NP_082213.2|	0	caskin-1 [Mus musculus]
mmu-miR-106a-5p	NR_038041	NA	NA	-23.32	159	-	-	gi|148700034|gb|EDL31981.1|	1.62E-60	mCG146286 [Mus musculus]

mmu-miR-106b-3p	NM_001001491	-26.9	0.635294	-25.55	156	GO:0005865,GO:0005884,GO:0032432,GO:0046872,GO:0005198,GO:0003015,GO:0030239,GO:0006936,GO:0006950,	K10375|1|8e-97|355|mmu:326618|tropomyosin 4	gi|47894398|ref|NP_001001491.1|	5.49E-96	tropomyosin alpha-4 chain [Mus musculus]
mmu-miR-106b-3p	NM_001001982	-30.6	0.406979	-27.67	158	-	-	gi|155029554|ref|NP_001001982.2|	0	uncharacterized protein C15orf52 homolog [Mus musculus]
mmu-miR-106b-3p	NM_001003824	-33.2	0.10278	-32.27	156	GO:0032991,GO:0005887,GO:0005515,GO:0005267,GO:0048731,GO:0007267,GO:0006935,GO:0034220,GO:0030001,	K04927|1|0.0|1436|mmu:16536|potassium voltage-gated channel KQT-like subfamily member 2	gi|148675436|gb|EDL07383.1|	0	potassium voltage-gated channel, subfamily Q, member 2, isoform CRA_j [Mus musculus]

mmu-miR-106b-3p	NM_001003825	-33.2	0.102178	-32.27	156	GO:0032991,GO:0005887,GO:0005515,GO:0005267,GO:0048731,GO:0007267,GO:0006935,GO:0034220,GO:0030001,	K04927|1|0.0|1355|mmu:16536|potassium voltage-gated channel KQT-like subfamily member 2	gi|51474502|ref|NP_001003825.1|	0	potassium voltage-gated channel subfamily KQT member 2 isoform 3 [Mus musculus]
mmu-miR-106b-3p	NM_001004144	-25.2	0.972561	-29.78	166	GO:0005924,GO:0044424,GO:0016772,GO:0005083,GO:0008047,GO:0004871,GO:0046914,GO:0042802,GO:0051020,GO:0043087,GO:0007186,GO:0006796,GO:00320	K05737|1|0.0|1467|mmu:216963|G protein-coupled receptor kinase interactor 1	gi|51921285|ref|NP_001004144.1|	0	ARF GTPase-activating protein GIT1 [Mus musculus]

mmu-miR-106b-3p	NM_001005608	-29	0.72296	-33.36	156	GO:0044444,GO:0016323,GO:0044421,GO:0005887,GO:0004871,GO:0005515,GO:0030035,GO:0007044,GO:0032501,GO:0007166,GO:0031589,	K06525|1|0.0|3564|mmu:192897|integrin beta 4	gi|110735426|ref|NP_001005608.2|	0	integrin beta-4 isoform 1 precursor [Mus musculus]
mmu-miR-106b-3p	NM_001006668	-33.2	0.101335	-32.27	156	GO:0032991,GO:0005887,GO:0005515,GO:0005267,GO:0048731,GO:0007267,GO:0006935,GO:0034220,GO:0030001,	K04927|1|0.0|1405|mmu:16536|potassium voltage-gated channel KQT-like subfamily member 2	gi|54873644|ref|NP_001006669.1|	0	potassium voltage-gated channel subfamily KQT member 2 isoform 4 [Mus musculus]
mmu-miR-106b-3p	NM_001006669	-33.2	0.09844	-32.27	156	GO:0005887,GO:0032991,GO:0005267,GO:0005515,GO:0034220,GO:0030001,	K04927|1|0.0|1340|mmu:16536|potassium voltage-gated channel KQT-like subfamily member 2	gi|54873646|ref|NP_001006670.1|	0	potassium voltage-gated channel subfamily KQT member 2 isoform 5 [Mus musculus]

mmu-miR-106b-3p	NM_001006674	-33.2	0.080475	-32.27	156	GO:0005887,GO:0032991,GO:0005267,GO:0005515,GO:0034220,GO:0030001,	K04927|1|0.0|1058|mmu:16536|potassium voltage-gated channel KQT-like subfamily member 2	gi|148675435|gb|EDL07382.1|	0	potassium voltage-gated channel, subfamily Q, member 2, isoform CRA_i [Mus musculus]
mmu-miR-106b-3p	NM_001006675	-33.2	0.100049	-32.27	156	GO:0032991,GO:0005887,GO:0005515,GO:0005267,GO:0048731,GO:0007267,GO:0006935,GO:0034220,GO:0030001,	K04927|1|0.0|1060|mmu:16536|potassium voltage-gated channel KQT-like subfamily member 2	gi|54873650|ref|NP_001006676.1|	0	potassium voltage-gated channel subfamily KQT member 2 isoform 7 [Mus musculus]
mmu-miR-106b-3p	NM_001007568	-30.9	0.285422	-52.93	327	GO:0005622,GO:0046872,	K09228|1|0.0|1314|mmu:71591|KRAB domain-containing zinc finger protein	gi|56090226|ref|NP_001007569.1|	0	zinc finger protein 251 [Mus musculus]
mmu-miR-106b-3p	NM_001008230	-33.8	0.062196	-53.88	315	GO:0031224,GO:0001664,GO:0034613,	-	gi|148665259|gb|EDK97675.1|	8.51E-136	receptor transporter protein 2, isoform CRA_b [Mus musculus]

mmu-miR-106b-3p	NM_001013370	-25.5	0.900283	-26.17	153	GO:0043231,GO:0022402,GO:0033554,GO:0008283,	K10141|1|0.0|951|mmu:140742|sestrin	gi|253314527|ref|NP_001013388.2|	0	sestrin-1 isoform 2 [Mus musculus]
mmu-miR-106b-3p	NM_001013381	-30.9	0.333531	-27.54	163	GO:0043231,GO:0043169,GO:0051536,GO:0016634,GO:0006778,	-	gi|148683981|gb|EDL15928.1|	0	radical S-adenosyl methionine domain containing 1, isoform CRA_a [Mus musculus]
mmu-miR-106b-3p	NM_001024626	-26.3	0.764761	-25.71	160	-	K09228|1|0.0|788|mmu:69623|KRAB domain-containing zinc finger protein	gi|110665709|ref|NP_001019797.2|	0	zinc finger protein 873 [Mus musculus]
mmu-miR-106b-3p	NM_001025383	-33.7	0.076461	-28.96	172	-	K11275|1|3e-06|55.1|oaa:100093290|histone H1/5	gi|85701574|ref|NP_001020554.1|	0	uncharacterized protein LOC574404 [Mus musculus]
mmu-miR-106b-3p	NM_001025384	-33.7	0.079472	-28.96	172	-	K11275|1|3e-06|55.1|oaa:100093290|histone H1/5	gi|85701574|ref|NP_001020554.1|	0	uncharacterized protein LOC574404 [Mus musculus]

mmu-miR-106b-3p	NM_001029873	-29.3	0.767544	-25.94	155	GO:0032991,GO:0044459,GO:0044424,GO:0043169,GO:0042802,GO:0008289,GO:0046983,GO:0019905,GO:0007270,GO:0048168,GO:0001505,GO:0007165,GO:0006904,GO:0016050,GO:0009100,	K15293|1|0.0|3163|mmu:382018|protein unc-13	gi|283837783|ref|NP_001025044.2|	0	protein unc-13 homolog A [Mus musculus]
mmu-miR-106b-3p	NM_001033208	-31	0.176909	-29.02	154	-	K10352|1|1e-14|82.4|cin:100176948|myosin heavy chain!K11499|4|2e-10|68.6|aml:100465502|centromere protein F	gi|85701616|ref|NP_001028380.1|	0	myocardial zonula adherens protein precursor [Mus musculus]

mmu-miR-106b-3p	NM_001033246	-30.5	0.247353	-27.32	157	GO:0043231,	K12478|1|1e-17|93.2|acs:100552393|early endosome antigen 1	gi|85701744|ref|NP_001028418.1|	0	coiled-coil domain-containing protein 110 [Mus musculus]
mmu-miR-106b-3p	NM_001033256	-36.2	0.021699	-33.72	170	GO:0044424,GO:0016462,GO:0032559,	K13525|1|0.0|1120|mcc:713376|transitional endoplasmic reticulum ATPase!K14575|2|2e-114|414|pon:100457915|AAA family ATPase	gi|348041246|ref|NP_001028428.2|	0	spermatogenesis associated 5-like 1 [Mus musculus]
mmu-miR-106b-3p	NM_001033344	-28.3	0.674248	-25.26	144	GO:0004721,GO:0006464,	K14165|1|0.0|1714|mmu:240892|dual specificity phosphatase [EC:3.1.3.16 3.1.3.48]	gi|229892201|ref|NP_001028516.2|	0	inactive dual specificity phosphatase 27 [Mus musculus]

mmu-miR-106b-3p	NM_001033382	-25.4	0.992989	-29.61	155	GO:0031224,GO:0043169,GO:0005261,GO:0050953,GO:0006810,	K04861|1|0.0|2175|mmu:319734|voltage-dependent calcium channel alpha-2/delta-4	gi|124517718|ref|NP_001028554.2|	0	voltage-dependent calcium channel subunit alpha-2/delta-4 [Mus musculus]
mmu-miR-106b-3p	NM_001033419	-37.5	0.007365	-31.81	175	GO:0031224,	K06499|1|3e-49|196|ecb:100069407|carcinoembryonic antigen-related cell adhesion molecule	gi|126432550|ref|NP_001028591.2|	0	carcinoembryonic antigen-related cell adhesion molecule 16 precursor [Mus musculus]
mmu-miR-106b-3p	NM_001033447	-32.7	0.089158	-31.31	166	-	-	gi|353523843|ref|NP_001028619.2|	0	uncharacterized protein LOC381196 [Mus musculus]
mmu-miR-106b-3p	NM_001033528	-25.5	0.990282	-27.43	159	GO:0031981,GO:0016790,GO:0070011,GO:0019941,	K11855|1|0.0|1919|mmu:72344|ubiquitin carboxyl-terminal hydrolase 36/42 [EC:3.1.2.15]	gi|148702709|gb|EDL34656.1|	0	mCG13907, isoform CRA_c [Mus musculus]

mmu-miR-106b-3p	NM_001034881	-27.9	0.494857	-26	157	-	-	gi|85702228|ref|NP_001030053.1|	1.24E-82	uncharacterized protein LOC432870 [Mus musculus]
mmu-miR-106b-3p	NM_001037539	-31.8	0.073801	-31.21	170	GO:0005102,GO:0042221,GO:0048511,GO:0051480,GO:0048232,GO:0007186,GO:0007600,GO:0006939,GO:0048646,GO:0006952,GO:0043066,GO:0000165,	-	gi|82830395|ref|NP_001032628.1|	4.91E-50	prokineticin-2 isoform 2 precursor [Mus musculus]
mmu-miR-106b-3p	NM_001037711	-25.1	0.992921	-30.53	146	GO:0043296,GO:0015629,GO:0008092,	K06102|1|0.0|1888|mmu:70737|cingulin	gi|50510881|dbj|BAD32426.1|	0	mKIAA1319 protein [Mus musculus]
mmu-miR-106b-3p	NM_001039154	-28.4	0.637288	-28.06	153	GO:0016021,GO:0046872,GO:0016337,GO:0045216,GO:0048232,	K06800|1|0.0|1296|mmu:12564|cadherin 8, type 2	gi|85662402|ref|NP_001034243.1|	0	cadherin-8 isoform 1 precursor [Mus musculus]

mmu-miR-106b-3p	NM_001039179	-28.3	0.823503	-51.44	299	GO:0031982,GO:0043232,GO:0043231,GO:0016020,GO:0005083,GO:0005488,GO:0019222,GO:0034453,GO:0072384,	K11498|1|4e-16|89.4|mdo:100013897|centromeric protein E!K10352|4|3e-14|83.2|aml:100473449|myosin heavy chain	gi|28972353|dbj|BAC65630.1|	0	mKIAA0699 protein [Mus musculus]
mmu-miR-106b-3p	NM_001039180	-28.3	0.715218	-26.16	150	GO:0031982,GO:0043232,GO:0043231,GO:0016020,GO:0005083,GO:0005488,GO:0019222,GO:0034453,GO:0072384,	K10421|1|3e-14|82.4|dse:Dsec_GM17234|CAP-Gly domain-containing linker protein 1!K10352|3|2e-13|79.7|aml:100473449|myosin heavy chain!K12478|5|3e-13|79.0|dre:562943|early endosome antigen 1	gi|85702355|ref|NP_001034268.1|	0	protein bicaudal D homolog 2 isoform 1 [Mus musculus]

mmu-miR-106b-3p	NM_001039376	-28.2	0.902329	-25.66	148	GO:0005815,GO:0043231,GO:0043292,GO:0005515,GO:0006461,	K10352|1|2e-25|120|mdo:100016741|myosin heavy chain	gi|158749578|ref|NP_001034465.2|	0	myomegalin isoform 1 [Mus musculus]
mmu-miR-106b-3p	NM_001040398	-30.4	0.653657	-29.22	161	-	-	-	-	-
mmu-miR-106b-3p	NM_001042557	-27.8	0.424483	-28.1	149	GO:0043231,GO:0044444,GO:0004708,GO:0019901,GO:0046872,GO:0032559,GO:0002224,GO:0000187,GO:0043065,	K04431|1|0.0|838|mmu:26400|mitogen-activated protein kinase kinase 7 [EC:2.7.12.2]	gi|110347549|ref|NP_001036022.1|	0	dual specificity mitogen-activated protein kinase kinase 7 isoform 1 [Mus musculus]
mmu-miR-106b-3p	NM_001042592	-32.8	0.160483	-30.14	158	GO:0023052,	-	gi|110611929|ref|NP_001036057.1|	0	arrestin domain containing 4 isoform 1 [Mus musculus]
mmu-miR-106b-3p	NM_001042726	-26.7	0.861012	-29.36	172	GO:0044459,GO:0043005,GO:0016021,GO:0046872,GO:0007613,GO:0016337,GO:0007267,GO:0009952,GO:0002009,	K04600|1|3e-56|221|der:Dere_GG20142|cadherin EGF LAG seven-pass G-type receptor 1 (flamingo)	gi|112181187|ref|NP_001036191.1|	0	protocadherin-8 isoform 2 precursor [Mus musculus]

mmu-miR-106b-3p	NM_001042760	-32.5	0.060273	-31.27	159	GO:0016021,GO:0044459,GO:0012505,GO:0044424,GO:0015238,GO:0008509,GO:0019899,GO:0015291,GO:0015893,GO:0003008,GO:0006812,	K08214|1|2e-168|592|mmu:18400|MFS transporter, OCT family, solute carrier family 22 (organic cation transporter), member 18	gi|112363086|ref|NP_001036225.1|	1.43E-167	solute carrier family 22 member 18 [Mus musculus]
mmu-miR-106b-3p	NM_001045484	-28.3	0.282177	-25.39	158	GO:0043231,GO:0043234,GO:0001071,GO:0019899,GO:0003677,GO:0006351,	K09261|1|6e-166|584|mmu:17259|MADS-box transcription enhancer factor 2B	gi|113865987|ref|NP_001038949.1|	4.16E-165	myocyte-specific enhancer factor 2B isoform 2 [Mus musculus]
mmu-miR-106b-3p	NM_001045486	-27.6	0.712592	-27.39	156	GO:0043231,GO:0046914,GO:0003676,GO:0006351,	K09228|1|0.0|1390|mmu:676710|KRAB domain-containing zinc finger protein	gi|113866022|ref|NP_001038951.1|	0	zinc finger protein 180 [Mus musculus]

mmu-miR-106b-3p	NM_001048177	-31.3	0.340454	-30.77	167	GO:0034399,GO:0043232,GO:0044459,GO:0044444,GO:0005506,GO:0051427,GO:0035400,GO:0019900,GO:0032559,GO:0005126,GO:0019904,GO:0031958,GO:0042506,GO:0007162,GO:0032611,GO:0007169,GO:0035406,GO:0031349,GO:0008016,GO:0043523,GO:0051100,GO:00099	K04447|1|0.0|2272|mmu:16452|Janus kinase 2 [EC:2.7.10.2]	gi|114326478|ref|NP_001041642.1|	0	tyrosine-protein kinase JAK2 [Mus musculus]

mmu-miR-106b-3p	NM_001079690	-29	0.623474	-27.1	151	GO:0044459,GO:0031224,GO:0015377,GO:0005488,GO:0015698,GO:0042592,GO:0003008,GO:0048513,GO:0030001,	K14425|1|0.0|2013|mmu:20495|solute carrier family 12 (sodium/potassium/chloride transporter), member	gi|119226233|ref|NP_001073158.1|	0	solute carrier family 12 member 1 isoform F [Mus musculus]
mmu-miR-106b-3p	NM_001079844	-31.3	0.43818	-31.75	165	GO:0016021,GO:0031012,GO:0043394,	K08112|1|0.0|1033|mmu:23888|glypican 6	gi|148668227|gb|EDL00557.1|	0	glypican 6, isoform CRA_b [Mus musculus]
mmu-miR-106b-3p	NM_001081014	-27.8	0.849025	-27.59	153	-	K12861|1|5e-09|65.5|gga:419884|pre-mRNA-splicing factor SPF27	gi|124486610|ref|NP_001074483.1|	0	DENN domain-containing protein 4C isoform 2 [Mus musculus]

mmu-miR-106b-3p	NM_001081088	-27.9	0.986709	-48.48	314	GO:0016023,GO:0000323,GO:0031224,GO:0005903,GO:0012505,GO:0044421,GO:0044459,GO:0004871,GO:0008565,GO:0046872,GO:0019904,GO:0017038,GO:0033189,GO:0009914,GO:0006775,GO:0009058,GO:0023052,GO:0007420,GO:0006629,GO:0006897,GO:0009887,GO:00064	K06233|1|0.0|9715|mmu:14725|low density lipoprotein-related protein 2	gi|124487372|ref|NP_001074557.1|	0	low-density lipoprotein receptor-related protein 2 precursor [Mus musculus]

mmu-miR-106b-3p	NM_001081221	-30.3	0.649645	-28.09	161	GO:0043234,GO:0031981,GO:0003676,GO:0042623,GO:0005515,GO:0004386,GO:0032559,GO:0006289,GO:0009416,GO:00063	K10841|1|0.0|2755|mmu:319955|DNA excision repair protein ERCC-6	gi|124487019|ref|NP_001074690.1|	0	DNA excision repair protein ERCC-6 [Mus musculus]
mmu-miR-106b-3p	NM_001081241	-33	0.159218	-28.22	150	-	K10385|1|9e-08|60.8|rno:502541|loricrin	gi|289728 78|dbj|BAC65855.1|	0	mKIAA1930 protein [Mus musculus]
mmu-miR-106b-3p	NM_001081247	-32.9	0.187236	-70.86	454	GO:0044451,GO:0003676,GO:0046914,GO:0034062,GO:0001882,GO:0006952,GO:0051707,GO:0032481,GO:0006353,GO:00063	K03018|1|0.0|2734|mmu:218832|DNA-directed RNA polymerase III subunit RPC1 [EC:2.7.7.6]	gi|124486963|ref|NP_001074716.1|	0	DNA-directed RNA polymerase III subunit RPC1 [Mus musculus]
mmu-miR-106b-3p	NM_001081258	-25.5	0.99876	-48.98	309	GO:0043231,GO:0015630,GO:0003774,GO:0032559,GO:0007017,	K10392|1|0.0|3163|mmu:381293|kinesin family member 1/13/14	gi|124486927|ref|NP_001074727.1|	0	kinesin-like protein KIF14 [Mus musculus]

mmu-miR-106b-3p	NM_001081270	-26.2	0.985019	-32.04	164	GO:0031224,GO:0042802,GO:0007417,GO:0008038,GO:0003002,GO:0016337,GO:0048667,GO:0045165,GO:0048562,	K06768|1|0.0|3999|mmu:114873|Down syndrome cell adhesion molecule like 1	gi|124486901|ref|NP_001074739.1|	0	Down syndrome cell adhesion molecule-like protein 1 homolog [Mus musculus]
mmu-miR-106b-3p	NM_001081290	-26.9	0.989454	-46.53	299	-	-	gi|124486835|ref|NP_001074759.1|	0	protein PRRC2C [Mus musculus]
mmu-miR-106b-3p	NM_001081387	-29.9	0.368217	-26.62	166	GO:0043231,GO:0046914,GO:0043565,GO:0005515,GO:0006351,GO:0006306,GO:0031060,	K09228|1|7e-63|243|mmu:630579|KRAB domain-containing zinc finger protein	gi|124487069|ref|NP_001074856.1|	0	transcriptional repressor CTCFL [Mus musculus]
mmu-miR-106b-3p	NM_001081392	-25.1	0.999998	-25.45	150	GO:0043231,GO:0017111,GO:0005515,GO:0032559,GO:0006461,GO:0009207,	K14572|1|0.0|1.043e+04|mmu:100019|midasin	gi|124487133|ref|NP_001074861.1|	0	midasin [Mus musculus]

mmu-miR-106b-3p	NM_001081441	-29.2	0.272218	-28.31	154	-	K10260|1|3e-38|160|gga:419074|F-box and WD-40 domain protein 7	gi|124517674|ref|NP_001074910.1|	0	WD repeat domain 86 [Mus musculus]
mmu-miR-106b-3p	NM_001081473	-27.9	0.83641	-49.74	305	GO:0044464,GO:0046914,GO:0006351,	K09228|1|1e-43|180|mgp:100547559|KRAB domain-containing zinc finger protein	gi|125490365|ref|NP_001074942.1|	0	zinc finger X-linked protein ZXDA/ZXDB [Mus musculus]
mmu-miR-106b-3p	NM_001085378	-28.8	0.750479	-53.81	318	GO:0016459,GO:0008092,GO:0017111,GO:0032559,	K10352|1|0.0|3052|mmu:668940|myosin heavy chain	gi|148674189|gb|EDL06136.1|	0	mCG21082 [Mus musculus]
mmu-miR-106b-3p	NM_001085518	-29.7	0.443945	-28.35	160	GO:0016021,GO:0005488,GO:0005337,GO:0015858,GO:0001894,GO:0035725,GO:00346	K11536|1|0.0|1142|mmu:269346|pyrimidine nucleoside transport protein	gi|147901770|ref|NP_001078987.1|	0	solute carrier family 28 (sodium-coupled nucleoside transporter), member 2-like [Mus musculus]

mmu-miR-106b-3p	NM_001101471	-29	0.75023	-27.53	159	GO:0044463,GO:0044459,GO:0044444,GO:0019899,GO:0051049,GO:0010737,GO:0015980,GO:0030073,GO:0048522,GO:0006171,GO:0032501,	K04573|1|2e-16|90.5|xla:397995|neurofilament medium polypeptide (neurofilament 3)!K00907|2|4e-16|89.4|dvi:Dvir_GJ20094|myosin-light-chain kinase [EC:2.7.11.18]	gi|158187517|ref|NP_001094941.1|	0	A-kinase anchor protein 5 [Mus musculus]
mmu-miR-106b-3p	NM_001104616	-28.2	0.700562	-27.73	153	GO:0031981,GO:0032155,GO:0043232,GO:0031252,GO:0044425,GO:0005902,GO:0008092,GO:0008105,GO:0030835,GO:00300	K05762|1|0.0|885|mmu:19684|radixin	gi|148693838|gb|EDL25785.1|	0	radixin [Mus musculus]

mmu-miR-106b-3p	NM_001109743	-28.7	0.520069	-26.98	147	GO:0043231,GO:0005488,GO:0003712,GO:0006357,	-	gi|157841282|ref|NP_001103213.1|	0	SKI family transcriptional corepressor 2 [Mus musculus]
mmu-miR-106b-3p	NM_001110163	-28.2	0.899153	-25.66	148	GO:0005815,GO:0043231,GO:0043292,GO:0005515,GO:0006461,	K10352|1|2e-25|120|mdo:100016741|myosin heavy chain	gi|158749580|ref|NP_001103633.1|	0	myomegalin isoform 4 [Mus musculus]
mmu-miR-106b-3p	NM_001111062	-28.5	0.415191	-27.31	160	GO:0043231,GO:0031224,GO:0044444,GO:0008171,GO:0046872,GO:0008202,GO:0006575,GO:0007611,GO:0033555,GO:0042053,GO:0006730,GO:0008285,GO:0042133,GO:0031337,GO:0002237,GO:00224	K00545|1|7e-136|485|mmu:12846|catechol O-methyltransferase [EC:2.1.1.6]	gi|161484634|ref|NP_031770.2|	4.88E-135	catechol O-methyltransferase [Mus musculus]

mmu-miR-106b-3p	NM_001111063	-28.5	0.383674	-27.31	160	GO:0043231,GO:0031224,GO:0044444,GO:0008171,GO:0046872,GO:0008202,GO:0006575,GO:0007611,GO:0033555,GO:0042053,GO:0006730,GO:0008285,GO:0042133,GO:0031337,GO:0002237,GO:00224	K00545|1|6e-136|485|mmu:12846|catechol O-methyltransferase [EC:2.1.1.6]	gi|161484634|ref|NP_031770.2|	4.38E-135	catechol O-methyltransferase [Mus musculus]
mmu-miR-106b-3p	NM_001113533	-29.4	0.287683	-25.98	153	GO:0005635,GO:0031981,GO:0032501,GO:0006396,	-	gi|148670073|gb|EDL02020.1|	3.77E-174	mCG16685, isoform CRA_a [Mus musculus]

mmu-miR-106b-3p	NM_001114125	-36	0.06906	-33.11	167	GO:0009898,GO:0044424,GO:0008047,GO:0060090,GO:0005083,GO:0042802,GO:0019901,GO:0019903,GO:0032813,GO:0006915,GO:0035411,GO:0016049,GO:0032318,GO:0034260,GO:0043433,GO:0001837,GO:0006469,GO:0070849,GO:0030178,GO:0007173,GO:0000122,GO:00082	K04352|1|4e-46|188|cin:100184835|Ras GTPase-activating protein 1	gi|166295214|ref|NP_001107597.1|	0	disabled homolog 2-interacting protein isoform 1 [Mus musculus]

mmu-miR-106b-3p	NM_001114322	-29.1	0.400741	-26.67	149	GO:0012505,GO:0044425,GO:0046872,GO:0016337,	K04600|1|6e-14|80.5|api:100160214|cadherin EGF LAG seven-pass G-type receptor 1 (flamingo)	gi|167004347|ref|NP_001107794.1|	0	cadherin-related family member 5 isoform 1 precursor [Mus musculus]
mmu-miR-106b-3p	NM_001114333	-28	0.881316	-28.26	158	GO:0043231,GO:0044430,GO:0044297,GO:0016021,GO:0030425,GO:0035258,GO:0008066,GO:0007200,GO:0051602,GO:0007204,GO:0010627,GO:0007610,GO:0051931,GO:0050804,GO:0042391,GO:0007270,GO:00	K04604|1|0.0|1790|mmu:14816|metabotropic glutamate receptor 1/5	gi|166999785|ref|NP_001107805.1|	0	metabotropic glutamate receptor 1 isoform beta precursor [Mus musculus]

mmu-miR-106b-3p	NM_001127177	-31.6	0.472985	-27.63	154	GO:0031981,GO:0005488,GO:0004871,GO:0004725,GO:0006470,GO:0007169,GO:00019	K01104|1|0.0|833|mmu:19255|protein-tyrosine phosphatase [EC:3.1.3.48]	gi|187608416|ref|NP_001120649.1|	0	tyrosine-protein phosphatase non-receptor type 2 isoform b [Mus musculus]
mmu-miR-106b-3p	NM_001134383	-30.7	0.381612	-28.45	153	GO:0043231,GO:0005083,GO:0032011,	K12495|1|0.0|1538|rno:686590|IQ motif and SEC7 domain-containing protein	gi|197304780|ref|NP_001127855.1|	0	IQ motif and SEC7 domain-containing protein 1 isoform a [Mus musculus]
mmu-miR-106b-3p	NM_001134384	-27.5	0.910468	-28.45	153	-	K12495|1|0.0|1498|hsa:9922|IQ motif and SEC7 domain-containing protein	gi|197304784|ref|NP_001127856.1|	0	IQ motif and SEC7 domain-containing protein 1 isoform b [Mus musculus]
mmu-miR-106b-3p	NM_001143671	-30.6	0.098966	-27.32	163	GO:0031224,	-	gi|119369434|sp|Q5FW56.2|FA70B_MOUSE	0	RecName: Full=Protein FAM70B
mmu-miR-106b-3p	NM_001143672	-30.6	0.09243	-27.32	163	GO:0031224,	-	gi|119369434|sp|Q5FW56.2|FA70B_MOUSE	0	RecName: Full=Protein FAM70B

mmu-miR-106b-3p	NM_001144953	-31.9	0.258481	-29.07	159	-	K10798|1|7e-08|61.2|tad:TRIADDRAFT_32651|poly [ADP-ribose] polymerase [EC:2.4.2.30]	gi|222352132|ref|NP_001138425.1|	0	von Willebrand factor A domain-containing protein 5B2 isoform 1 [Mus musculus]
mmu-miR-106b-3p	NM_001145900	-34.1	0.042416	-31.54	158	GO:0030529,	K10478|1|0.0|893|mmu:399566|BTB/POZ domain-containing protein 3/6	gi|225543368|ref|NP_964008.2|	0	BTB/POZ domain-containing protein 6 isoform 1 [Mus musculus]
mmu-miR-106b-3p	NM_001145967	-29	0.442657	-27.76	157	GO:0044444,GO:0004175,GO:0019538,GO:0006605,GO:0044248,GO:0007033,	K08342|1|0.0|941|mmu:242557|autophagy-related protein 4 [EC:3.4.22.-]	gi|148698945|gb|EDL30892.1|	0	autophagy-related 4C (yeast), isoform CRA_b [Mus musculus]

mmu-miR-106b-3p	NM_001146022	-27.4	0.971197	-30.91	154	GO:0031224,	K07295|1|2e-36|157|dpe:Dper_GL19043|nuclear receptor subfamily 2 group A!K11127|2|7e-09|65.9|xtr:100485265|telomerase protein component 1!K12862|3|7e-08|62.4|oaa:100083184|pleiotropic regulator 1!K14753|4|2e-07|60.8|cin:100179	gi|225637485|ref|NP_001139494.1|	0	WD repeat and FYVE domain containing 4 [Mus musculus]

mmu-miR-106b-3p	NM_001159392	-30.8	0.309504	-28.73	163	GO:0031981,GO:0031461,GO:0005887,GO:0005267,GO:0017048,GO:0019787,GO:0007266,GO:0006275,GO:0007275,GO:0030001,GO:0012501,GO:0051017,GO:0006511,GO:0048870,GO:0051259,GO:0034220,GO:0002376,GO:0032446,	K15074|1|0.0|641|mmu:21927|BTB/POZ domain-containing adapter for CUL3-mediated RhoA degradation protein	gi|148683641|gb|EDL15588.1|	0	tumor necrosis factor, alpha-induced protein 1 (endothelial), isoform CRA_a [Mus musculus]
mmu-miR-106b-3p	NM_001159743	-36.9	0.008437	-38.65	162	-	-	gi|28204858|gb|AAH46522.1|	1.06E-127	Acp1 protein, partial [Mus musculus]
mmu-miR-106b-3p	NM_001159910	-31.3	0.099071	-28.5	158	-	-	gi|229576815|ref|NP_001153382.1|	7.02E-159	coiled-coil domain-containing protein 85C [Mus musculus]

mmu-miR-106b-3p	NM_001160049	-28.3	0.673885	-25.26	144	GO:0004721,GO:0006464,	K14165|1|0.0|1714|mmu:240892|dual specificity phosphatase [EC:3.1.3.16 3.1.3.48]	gi|229892201|ref|NP_001028516.2|	0	inactive dual specificity phosphatase 27 [Mus musculus]
mmu-miR-106b-3p	NM_001160316	-27.2	0.97606	-63.4	323	GO:0031224,GO:0033267,GO:0005911,GO:0008366,GO:0043062,GO:0007043,GO:0006935,GO:0034613,	K06757|1|0.0|2359|mmu:269116|neurofascin	gi|237858630|ref|NP_001153788.1|	0	neurofascin isoform 2 precursor [Mus musculus]
mmu-miR-106b-3p	NM_001160317	-27.2	0.977525	-63.4	323	GO:0031224,GO:0033267,GO:0005911,GO:0005488,GO:0008366,GO:0043062,GO:0007043,GO:0006935,GO:0034613,	K06757|1|0.0|2343|ptr:457660|neurofascin	gi|37360078|dbj|BAC98017.1|	0	mKIAA0756 protein [Mus musculus]

mmu-miR-106b-3p	NM_001160318	-27.2	0.977891	-63.4	323	GO:0031224,GO:0033267,GO:0005911,GO:0008366,GO:0043062,GO:0007043,GO:0006935,GO:0034613,	K06757|1|0.0|2289|ptr:457660|neurofascin	gi|37360078|dbj|BAC98017.1|	0	mKIAA0756 protein [Mus musculus]
mmu-miR-106b-3p	NM_001161362	-27.5	0.922807	-27.83	150	GO:0008287,GO:0046872,GO:0019888,GO:0006464,	K11583|1|0.0|2281|mmu:235542|protein phosphatase 2 (formerly 2A), regulatory subunit	gi|238624141|ref|NP_001154834.1|	0	protein phosphatase 2, regulatory subunit B'', alpha isoform 1 [Mus musculus]
mmu-miR-106b-3p	NM_001161365	-27.2	0.803659	-25	163	GO:0005768,GO:0031410,GO:0008047,GO:0031267,GO:0016192,GO:0043087,GO:00230	-	gi|238624151|ref|NP_808288.2|	0	ras and Rab interactor 3 isoform 1 [Mus musculus]
mmu-miR-106b-3p	NM_001161665	-28.1	0.884671	-25.06	145	GO:0015630,GO:0003774,GO:0032559,GO:0007017,	K10404|1|0.0|3446|mmu:269152|kinesin family member 26	gi|239582737|ref|NP_001155137.1|	0	kinesin-like protein KIF26B [Mus musculus]

mmu-miR-106b-3p	NM_001162979	-28.4	0.505654	-27.36	163	-	K10358|1|5e-07|57.8|cin:100186550|myosin VI!K06672|2|4e-06|54.7|cbr:CBG07027|cohesin loading factor subunit SCC2!K11515|3|5e-06|54.3|spu:582213|inner centrome	gi|244790085|ref|NP_001156451.1|	0	coiled-coil domain-containing protein 81 [Mus musculus]
mmu-miR-106b-3p	NM_001163464	-25.4	0.964154	-25.32	141	GO:0016791,GO:0043169,GO:0006796,	K06124|1|7e-153|542|rno:287644|phosphoethanolamine/phosphocholine phosphatase [EC:3.1.3.7	gi|157786864|ref|NP_001099303.1|	4.67E-152	phosphoethanolamine/phosphocholine phosphatase [Rattus norvegicus]
mmu-miR-106b-3p	NM_001163473	-29.5	0.138164	-28.61	156	-	-	gi|344243051|gb|EGV99154.1|	5.12E-67	UPF0450 protein C17orf58-like [Cricetulus griseus]

mmu-miR-106b-3p	NM_001163554	-26.2	0.886586	-27.73	154	GO:0043231,GO:0001071,GO:0003677,GO:0005515,GO:0002381,GO:0009306,GO:0006355,GO:0030183,GO:0021700,	K09364|1|0.0|760|mmu:18987|POU domain transcription factor, class 2	gi|53490|emb|CAA41008.1|	0	Oct2.5 transcription factor [Mus musculus]
mmu-miR-106b-3p	NM_001163555	-26.2	0.886014	-27.73	154	GO:0043231,GO:0001071,GO:0003677,GO:0005515,GO:0002381,GO:0009306,GO:0006355,GO:0030183,GO:0021700,	K09364|1|0.0|610|mmu:18987|POU domain transcription factor, class 2	gi|149056612|gb|EDM08043.1|	0	POU domain, class 2, transcription factor 2, isoform CRA_a [Rattus norvegicus]
mmu-miR-106b-3p	NM_001163556	-26.2	0.881163	-27.73	154	GO:0043231,GO:0001071,GO:0003677,GO:0005515,GO:0002381,GO:0009306,GO:0006355,GO:0030183,GO:0021700,	K09364|1|0.0|622|mmu:18987|POU domain transcription factor, class 2	gi|149056612|gb|EDM08043.1|	0	POU domain, class 2, transcription factor 2, isoform CRA_a [Rattus norvegicus]

mmu-miR-106b-3p	NM_001164172	-27.8	0.701237	-28.1	149	GO:0043231,GO:0044444,GO:0004708,GO:0019901,GO:0046872,GO:0032559,GO:0002224,GO:0000187,GO:0043065,	K04431|1|0.0|820|mmu:26400|mitogen-activated protein kinase kinase 7 [EC:2.7.12.2]	gi|81877892|sp|Q8CE90.1|MP2K7_MOUSE	0	RecName: Full=Dual specificity mitogen-activated protein kinase kinase 7; Short=MAP kinase kinase 7; Short=MAPKK 7; AltName: Full=JNK-activating kinase 2; AltName: Full=MAPK/ERK kinase 7; Short=MEK 7; AltName: Full=c-Jun N-terminal kinase kinase 2;
mmu-miR-106b-3p	NM_001164491	-30.5	0.383654	-29.26	162	GO:0044424,GO:0008092,GO:0046914,GO:0006996,GO:0006357,GO:0006935,	K07520|1|0.0|1335|mmu:319713|actin-binding LIM protein	gi|50510665|dbj|BAD32318.1|	0	mKIAA0843 protein [Mus musculus]
mmu-miR-106b-3p	NM_001166065	-27.7	0.832992	-25.86	149	GO:0012505,GO:0043231,GO:0031224,GO:0008375,GO:0006486,	K09663|1|0.0|835|mmu:218476|beta-1,6-N-acetylglucosaminyltransferase 4 [EC:2.4.1.-]	gi|260593704|ref|NP_001159537.1|	0	beta-1,3-galactosyl-O-glycosyl-glycoprotein beta-1,6-N-acetylglucosaminyltransferase 4 [Mus musculus]

mmu-miR-106b-3p	NM_001166210	-28.1	0.84612	-25.55	158	GO:0005737,GO:0043232,GO:0005515,GO:0000166,	K10352|1|0.0|3374|mmu:667772|myosin heavy chain	gi|261245016|ref|NP_001159682.1|	0	myosin-15 [Mus musculus]
mmu-miR-106b-3p	NM_001166463	-28.3	0.72548	-47.85	307	GO:0031012,GO:0046872,GO:0048731,GO:0023052,GO:0009987,	K08136|1|0.0|779|mmu:20745|sparc/osteonectin, cwcv and kazal-like domains proteoglycan (testican)	gi|148709301|gb|EDL41247.1|	0	sparc/osteonectin, cwcv and kazal-like domains proteoglycan 1, isoform CRA_b [Mus musculus]
mmu-miR-106b-3p	NM_001166466	-28.3	0.725926	-47.85	307	GO:0031012,GO:0046872,GO:0023052,	K08136|1|0.0|772|ptr:462085|sparc/osteonectin, cwcv and kazal-like domains proteoglycan (testican)	gi|148709301|gb|EDL41247.1|	0	sparc/osteonectin, cwcv and kazal-like domains proteoglycan 1, isoform CRA_b [Mus musculus]
mmu-miR-106b-3p	NM_001166506	-28.3	0.730705	-26.84	154	GO:0031224,GO:0043231,GO:0051234,	-	gi|148702667|gb|EDL34614.1|	0	SEC14-like 1 (S. cerevisiae), isoform CRA_c [Mus musculus]
mmu-miR-106b-3p	NM_001166507	-28.3	0.703294	-26.84	154	GO:0031224,GO:0043231,GO:0051234,	-	gi|148702667|gb|EDL34614.1|	0	SEC14-like 1 (S. cerevisiae), isoform CRA_c [Mus musculus]

mmu-miR-106b-3p	NM_001162864	-20.6	1	-48.63	320	GO:0044459,GO:0043231,GO:0016740,GO:0000166,	K08815|1|0.0|2012|mmu:106763|tau tubulin kinase [EC:2.7.11.26]	gi|242247093|ref|NP_001156336.1|		0	tau-tubulin kinase 1 [Mus musculus]
mmu-miR-106b-5p	NM_001008550	-27.3	0.886774	-25.06	162	GO:0005815,GO:0035091,GO:0046914,GO:0006259,	K05724|1|6e-13|79.3|mgp:100546627|FYVE, RhoGEF and PH domain containing 5/6	gi|60360314|dbj|BAD90401.1|		0	mKIAA0321 protein [Mus musculus]
mmu-miR-106b-5p	NM_001031811	-28.6	0.441794	-25.43	177	GO:0031224,GO:0004673,GO:0000981,GO:0005267,GO:0006915,GO:0006351,GO:0030001,	K04911|1|0.0|2081|mmu:211468|potassium voltage-gated channel Eag-related subfamily H member 8	gi|122065252|sp|P59111.2|KCNH8_MOUSE		0	RecName: Full=Potassium voltage-gated channel subfamily H member 8; AltName: Full=Ether-a-go-go-like potassium channel 3; Short=ELK channel 3; Short=ELK3; AltName: Full=Voltage-gated potassium channel subunit Kv12.1

mmu-miR-106b-5p	NM_001034097	-32.4	0.04661	-28.59	174	-	K05475|1|2e-105|384|mmu:69583|tumor necrosis factor ligand superfamily member	gi|77539772|ref|NP_001029269.1|	0	tumor necrosis factor (ligand) superfamily, member 12-member 13 isoform a [Mus musculus]
mmu-miR-106b-5p	NM_001034867	-29.2	0.448611	-27.7	175	GO:0003824,	-	gi|298493323|ref|NP_001030039.2|	0	peptidase M20 domain-containing protein 2 [Mus musculus]
mmu-miR-106b-5p	NM_001159503	-32.4	0.046441	-28.59	174	-	K05475|1|7e-107|389|mmu:69583|tumor necrosis factor ligand superfamily member	gi|226958391|ref|NP_001152975.1|	0	tumor necrosis factor (ligand) superfamily, member 12-member 13 isoform c [Mus musculus]
mmu-miR-106b-5p	NM_001168621	-32	0.129849	-27.1	165	GO:0000323,GO:0044459,GO:0008021,GO:0046914,GO:0003824,	K10694|1|2e-134|481|mmu:170737|E3 ubiquitin-protein ligase ZNRF [EC:6.3.2.19]	gi|148679554|gb|EDL11501.1|	3.35E-152	zinc and ring finger 1, isoform CRA_a [Mus musculus]

mmu-miR-106b-5p	NM_001168622	-32	0.162631	-27.1	165	GO:0000323,GO:0044459,GO:0008021,GO:0046914,GO:0003824,	K10694|1|4e-109|397|mmu:170737|E3 ubiquitin-protein ligase ZNRF [EC:6.3.2.19]	gi|148679554|gb|EDL11501.1|	7.94E-127	zinc and ring finger 1, isoform CRA_a [Mus musculus]
mmu-miR-106b-5p	NM_001252562	-30.6	0.076093	-25.52	175	GO:0031981,GO:0003676,GO:0042623,GO:0032559,GO:0006259,GO:0033554,GO:00092	K10869|1|4e-140|499|mmu:19363|RAD51-like protein 1	gi|357588408|ref|NP_001239491.1|	1.85E-142	DNA repair protein RAD51 homolog 2 isoform 2 [Mus musculus]

mmu-miR-106b-5p	NM_008678	-25.1	0.991428	-25.21	161	GO:0044456,GO:0031981,GO:0043005,GO:0044430,GO:0015630,GO:0044297,GO:0005783,GO:0060089,GO:0051427,GO:0010861,GO:0004468,GO:0019904,GO:0003713,GO:0006357,GO:0030518,GO:0006629,GO:0010469,GO:0022037,	K11255|1|0.0|2502|rno:83724|nuclear receptor coactivator 2	gi|118026940|ref|NP_032704.2|	0	nuclear receptor coactivator 2 isoform a [Mus musculus]
mmu-miR-106b-5p	NM_009014	-30.6	0.081856	-25.52	175	GO:0031981,GO:0003676,GO:0042623,GO:0032559,GO:0006310,GO:0033554,GO:0009207,GO:0007599,	K10869|1|0.0|688|mmu:19363|RAD51-like protein 1	gi|31982072|ref|NP_033040.2|	0	DNA repair protein RAD51 homolog 2 isoform 1 [Mus musculus]

mmu-miR-106b-5p	NM_011614	-32.4	0.031675	-28.59	174	GO:0016021,GO:0044444,GO:0044421,GO:0032813,GO:0016477,GO:0012502,GO:0048869,GO:0023052,GO:0002376,GO:0045765,GO:0001935,	K05474|1|1e-91|338|mmu:21944|tumor necrosis factor ligand superfamily member 12	gi|74221419|dbj|BAE42185.1|	7.03E-91	unnamed protein product [Mus musculus]
mmu-miR-106b-5p	NM_013770	-25.2	0.906595	-46.17	335	GO:0031224,GO:0031981,GO:0019866,GO:0015103,GO:0005488,GO:0015291,GO:0015556,GO:0015743,GO:0015744,GO:0046907,GO:0006006,GO:0006817,	K13577|1|4e-161|570|mmu:27376|solute carrier family 25 (mitochondrial dicarboxylate transporter), member 10	gi|254826790|ref|NP_038798.2|	2.98E-160	mitochondrial dicarboxylate carrier [Mus musculus]
mmu-miR-106b-5p	NM_027283	-31.3	0.020764	-27.49	177	-	-	gi|149039179|gb|EDL93399.1|	4.69E-94	Rsb-66 protein, isoform CRA_a [Rattus norvegicus]

mmu-miR-106b-5p	NM_029508	-31.6	0.038975	-28.36	178	GO:0005815,GO:0046914,GO:0010467,	K11489|1|4e-124|445|mmu:76073|polycomb group RING finger protein 5	gi|148709823|gb|EDL41769.1|	7.08E-124	polycomb group ring finger 5, isoform CRA_b [Mus musculus]
mmu-miR-106b-5p	NM_053269	-28.9	0.283855	-25.45	172	GO:0031981,GO:0043234,GO:0044444,GO:0003676,GO:0016889,GO:0032559,GO:0042623,GO:0007127,GO:0007599,GO:0007143,GO:0006310,GO:0009207,GO:0007059,GO:0048232,GO:0033554,	K10870|1|0.0|729|mmu:114714|RAD51-like protein 2	gi|16716605|ref|NP_444499.1|	0	DNA repair protein RAD51 homolog 3 [Mus musculus]

mmu-miR-106b-5p	NM_054087	-29	0.325971	-26.52	175	-	K14610|1|0.0|903|mmu:116914|solute carrier family 19 (thiamine transporter), member 2/3	gi|16905117|ref|NP_473428.1|	0	thiamine transporter 1 [Mus musculus]
mmu-miR-106b-5p	NM_054097	-25.4	0.840418	-42.7	319	GO:0043231,GO:0005515,GO:0016307,GO:0032559,GO:0046488,GO:0046474,GO:0006796,	K00920|1|0.0|650|mmu:117150|1-phosphatidylinositol-5-phosphate 4-kinase [EC:2.7.1.149]	gi|17298686|ref|NP_473438.1|	0	phosphatidylinositol 5-phosphate 4-kinase type-2 gamma [Mus musculus]
mmu-miR-106b-5p	NM_133206	-32	0.165669	-27.1	165	GO:0000323,GO:0044459,GO:0008021,GO:0046914,GO:0003824,	K10694|1|2e-134|481|mmu:170737|E3 ubiquitin-protein ligase ZNRF [EC:6.3.2.19]	gi|148679554|gb|EDL11501.1|	4.24E-152	zinc and ring finger 1, isoform CRA_a [Mus musculus]
mmu-miR-106b-5p	NM_153563	-28.1	0.412577	-25.7	164	GO:0043231,GO:0005488,	-	gi|392339015|ref|XP_001068288.2|	0	PREDICTED: protein FAM40A isoform 3 [Rattus norvegicus]

mmu-miR-106b-5p	NM_183034	-36	0.029543	-31.88	187	GO:0044424,GO:0043169,GO:0007165,	K15348|1|2e-11|73.6|bfo:BRAFLDRAFT_121069|pleckstrin homology domain-containing family M member 2	gi|33942120|ref|NP_898855.1|	0	pleckstrin homology domain-containing family M member 1 [Mus musculus]
mmu-miR-106b-5p	NR_033470	-28.2	0.348773	-25.68	169	GO:0044444,GO:0046914,GO:0004871,GO:0012502,GO:0023052,	K11985|1|1e-161|571|mmu:22036|TRAF-interacting protein	gi|31560535|ref|NP_035764.2|	6.89E-161	TRAF-interacting protein [Mus musculus]
mmu-miR-106b-5p	NR_033471	-28.2	0.346574	-25.68	169	GO:0044444,GO:0046914,GO:0004871,GO:0012502,GO:0023052,	K11985|1|6e-161|568|mmu:22036|TRAF-interacting protein	gi|31560535|ref|NP_035764.2|	4.43E-160	TRAF-interacting protein [Mus musculus]
mmu-miR-106b-5p	NR_045471	-28.8	0.236496	-25.86	173	-	-	gi|148679699|gb|EDL11646.1|	3.20E-87	mCG144621 [Mus musculus]

mmu-miR-106b-5p	NM_178267	-21.8	1	-42.13	315	GO:0043231,GO:0046914,GO:0003676,GO:0010467,	K09228|1|3e-32|143|bfo:BRAFLDRAFT_66688|KRAB domain-containing zinc finger protein	gi|332820436|ref|XP_517584.3|	0	PREDICTED: zinc finger protein 827-like [Pan troglodytes]
mmu-miR-106b-5p	NM_011375	-26.7	0.516843	-23.45	174	GO:0016021,GO:0031228,GO:0008373,GO:0001573,GO:0006464,	K03370|1|0.0|769|mmu:20454|lactosylceramide alpha-2,3-sialyltransferase (sialyltransferase 9) [EC:2.4.99.9]	gi|148666543|gb|EDK98959.1|	0	ST3 beta-galactoside alpha-2,3-sialyltransferase 5, isoform CRA_a [Mus musculus]
mmu-miR-106b-5p	NM_173437	-20.2	1	-22.62	173	-	-	gi|224922751|ref|NP_775613.2|	0	neuron navigator 1 [Mus musculus]

mmu-miR-106b-5p	NM_130867	-23.5	0.999758	-25.74	172	GO:0031224,GO:0030054,GO:0030425,GO:0003008,	K14319|1|5e-31|139|bfo:BRAFLDRAFT_126584|Ran GTPase-activating protein 1!K04910|2|3e-24|116|spu:578213|potassium voltage-gated channel Eag-related subfamily H member 7!K06755|4|2e-22|110|ptr:466839|roundabout, axon	gi|70608146|ref|NP_570937.2|	0	kin of IRRE-like protein 1 precursor [Mus musculus]

mmu-miR-106b-5p	NM_153420	-27.9	0.426444	-23.63	171	GO:0016791,GO:0006796,	K15116|1|1e-28|129|spu:583160|solute carrier family 25, member 33/36!K14395|3|2e-17|92.4|xla:100127261|lysophosphatidic acid phosphatase type 6 [EC:3.1.3.2]!K01078|4|4e-17|91.3|cbr:CBG06099|acid phosphatase [EC:3.1.3.2]	gi|23510309|ref|NP_700469.1|	0	acid phosphatase-like protein 2 precursor [Mus musculus]
mmu-miR-106b-5p	NR_037983	-26.8	0.542217	-20.09	170	-	K12019|1|1e-38|162|mmu:666731|tripartite motif-containing protein 43/48/49/64/77	gi|295390679|ref|NP_001171329.1|	7.14E-38	tripartite motif-containing protein 43C [Mus musculus]
mmu-miR-106b-5p	NR_073186	-26.6	0.528228	-21.96	169	-	-	-	-	-

mmu-miR-106b-5p	NM_178773	-24.7	0.952266	-20.29	168	GO:0034702,GO:0005253,GO:0006810,	K05169|1|4e-34|148|ptr:743996|single Ig IL-1R-related molecule!K13982|2|1e-26|123|dgr:Dgri_GH10918|probable ATP-dependent RNA helicase DDX4 [EC:3.6.4.13]	gi|148689550|gb|EDL21497.1|	0	transmembrane protein 16D (eight membrane-spanning domains), isoform CRA_a [Mus musculus]

mmu-miR-106b-5p	NR_033593	-23.8	0.979558	-21.32	167	GO:0043231,GO:0003676,GO:0005515,GO:0043169,	K15107|1|2e-13|79.3|hmg:100201695|solute carrier family 25 (mitochondrial glutamate transporter), member 18/22!K14303|2|8e-07|57.4|hmg:100204852|nuclear pore complex protein Nup160	gi|149234328|ref|XP_001480230.1|	0	PREDICTED: zinc finger BED domain-containing protein 4-like [Mus musculus]
mmu-miR-106b-5p	NR_045801	-25.2	0.651029	-20.6	166	-	-	gi|12857035|dbj|BAB30867.1|	6.42E-53	unnamed protein product [Mus musculus]
mmu-miR-106b-5p	NR_028280	-26.9	0.617902	-24.51	165	-	-	gi|255308901|ref|NP_848889.3|	3.00E-129	proline-rich protein 18 [Mus musculus]

mmu-miR-106b-5p	NM_212450	-28.6	0.461108	-23.15	164	GO:0016791,GO:0006796,	K15731|1|2e-34|149|dre:553744|carboxy-terminal domain RNA polymerase II polypeptide A small phosphatase [EC:3.1.3.16]	gi|47059059|ref|NP_997615.1|	0	CTD small phosphatase-like protein 2 [Mus musculus]
mmu-miR-106b-5p	NR_045001	-21.6	0.988427	-20.61	163	-	-	gi|148665703|gb|EDK98119.1|	2.04E-40	mCG142484 [Mus musculus]
mmu-miR-106b-5p	NM_183032	-25.3	0.453202	-20.95	162	GO:0016892,GO:0005488,	K01168|1|2e-07|57.4|mcc:704676|pancreatic ribonuclease [EC:3.1.27.5]	gi|118200345|ref|NP_898853.2|	7.39E-99	ribonuclease-like protein 9 precursor [Mus musculus]
mmu-miR-106b-5p	NR_040314	-23.9	0.975142	-22.27	161	-	-	gi|148675117|gb|EDL07064.1|	1.19E-72	mCG144576 [Mus musculus]

mmu-miR-106b-5p	NR_045795	-24	0.911559	-21.4	160	GO:0043231,GO:0031224,GO:0043169,GO:0016772,GO:0046469,GO:0006656,	-	gi|148677466|gb|EDL09413.1|	2.42E-26	mCG147326 [Mus musculus]
mmu-miR-106b-5p	NM_181416	-24.6	0.977641	-21.93	159	GO:0044464,GO:0008047,GO:0043087,GO:0023052,	K07526|1|3e-15|85.9|mmu:14270|SLIT-ROBO Rho GTPase activating protein	gi|37359732|dbj|BAC97844.1|	0	mKIAA0013 protein [Mus musculus]
mmu-miR-106b-5p	NR_040387	-27.1	0.408954	-20.2	158	-	-	-	-	-
mmu-miR-106b-5p	NR_073371	-25.1	0.523715	-20.26	157	-	-	-	-	-
mmu-miR-106b-5p	NM_180678	-22.4	0.9936	-20.7	156	GO:0016023,GO:0044429,GO:0005515,GO:0004812,GO:0032559,GO:0006887,GO:0006418,GO:0015960,	K01880|1|0.0|1319|mmu:353172|glycyl-tRNA synthetase [EC:6.1.1.14]	gi|148666303|gb|EDK98719.1|	0	glycyl-tRNA synthetase, isoform CRA_b [Mus musculus]
mmu-miR-106b-5p	NR_040411	-25.2	0.765328	-20.54	155	-	-	gi|148703130|gb|EDL35077.1|	1.87E-13	mCG1049722 [Mus musculus]

mmu-miR-106b-5p	NM_177823	-24.1	0.943243	-20.89	154	GO:0043231,GO:0044444,GO:0001816,GO:0050856,	-	gi|165905557|ref|NP_808491.2|	0	ubiquitin-associated and SH3 domain-containing protein A [Mus musculus]
mmu-miR-106b-5p	NM_138679	-22.7	1	-20.16	153	GO:0043296,GO:0031981,GO:0043232,GO:0003676,GO:0046914,GO:0016279,GO:0071103,GO:0006351,GO:0006325,GO:00071	K06101|1|0.0|5155|mmu:192195|histone-lysine N-methyltransferase ASH1L [EC:2.1.1.43]	gi|73622271|ref|NP_619620.3|	0	histone-lysine N-methyltransferase ASH1L [Mus musculus]
mmu-miR-106b-5p	NM_183275	-23.9	0.735437	-20.94	152	-	-	gi|134053922|ref|NP_899098.2|	0	transcription elongation factor, mitochondrial [Mus musculus]
mmu-miR-106b-5p	NM_178896	-22.1	0.999921	-20.07	151	-	K10624|1|9e-21|103|ame:724670|E3 ubiquitin-protein ligase RBBP6 [EC:6.3.2.19]	gi|395843823|ref|XP_003794672.1|	1.91E-166	PREDICTED: DCN1-like protein 4 [Otolemur garnettii]

mmu-miR-106b-5p	NM_033144	-20.1	1	-20.54	150	GO:0032156,GO:0032561,GO:0009987,	K04557|1|7e-65|250|xla:779425|peanut-like protein 1 (cell division control related protein 1)	gi|148701638|gb|EDL33585.1|	0	septin 8, isoform CRA_c [Mus musculus]

mmu-miR-106b-5p	NM_001033263	-21.1	1	-21.29	149	GO:0031981,GO:0044444,GO:0019900,GO:0032561,GO:0032559,GO:0008047,GO:0017111,GO:0005083,GO:0019887,GO:0046914,GO:0006357,GO:0001932,GO:0007010,GO:0032012,GO:0033674,GO:0009895,GO:0006935,GO:0043066,GO:0010740,GO:0043087,GO:0033598,GO:00161	K12491|1|0.0|1466|mmu:216439|Arf-GAP, GTPase, ANK repeat and PH domain-containing protein 1/2/3	gi|84370300|ref|NP_001028435.1|	0	arf-GAP with GTPase, ANK repeat and PH domain-containing protein 2 [Mus musculus]
mmu-miR-106b-5p	NR_028417	-21.8	0.982598	-21.12	148	-	-	gi|148705637|gb|EDL37584.1|	3.85E-39	mCG146113 [Mus musculus]
mmu-miR-106b-5p	NM_024244	-20.7	0.999999	-20.03	146	-	-	gi|148700024|gb|EDL31971.1|	0	RIKEN cDNA 1200015N20, isoform CRA_b [Mus musculus]

mmu-miR-106b-5p	NM_009416	NA	NA	-20.19	154	GO:0005865,GO:0008092,GO:0005198,GO:0033121,GO:0006936,	K10374|1|3e-95|350|mmu:22004|tropomyosin 2	gi|344271027|ref|XP_003407343.1|	1.78E-94	PREDICTED: tropomyosin beta chain-like isoform 2 [Loxodonta africana]
mmu-miR-106b-5p	NM_020515	NA	NA	-20.01	149	GO:0016020,GO:0004888,GO:0007166,	K04257|1|4e-163|574|mmu:57272|olfactory	gi|11464995|ref|NP_065261.1|	2.40E-162	olfactory receptor 140 [Mus musculus]
mmu-miR-106b-5p	NM_008455	NA	NA	-21.37	146	GO:0044424,GO:0016020,GO:0044421,GO:0004175,GO:0031638,GO:0002353,GO:00301	K01324|1|0.0|1344|mmu:16621|plasma kallikrein [EC:3.4.21.34]	gi|148703597|gb|EDL35544.1|	0	mCG119836 [Mus musculus]
mmu-miR-106b-5p	NM_011347	-22.8	0.99727	NA	NA	-	K06496|1|0.0|1614|mmu:20344|selectin, platele	gi|193566|gb|AAA37712.1|	0	granule membrane protein 140 [Mus musculus]
mmu-miR-106b-5p	NM_153581	-21.9	0.999815	NA	NA	GO:0031224,GO:0005261,GO:0006816,	-	gi|23957686|ref|NP_705809.1|	7.90E-151	neuronal membrane glycoprotein M6-a isoform 1 [Mus
mmu-miR-106b-5p	NM_001037221	-20.9	1	NA	NA	GO:0043005,GO:0044459,GO:0044424,GO:0045182,GO:0006412,	-	gi|82617646|ref|NP_001032298.1|	0	protein Smaug homolog 1 isoform 1 [Mus musculus]

mmu-miR-106b-5p	NM_001199105	-22.1	0.999993	NA	NA	GO:0016604,GO:0050896,GO:0012502,GO:0022402,	K15310|1|1e-118|280|rno:297822|tumor protein p53-inducible nuclear protein 1	gi|11230806|ref|NP_068697.1|	6.97E-124	tumor protein p53-inducible nuclear protein 1 isoform 1 [Mus musculus]
mmu-miR-106b-5p	NM_023526	-20.5	1	NA	NA	GO:0044424,GO:0017111,GO:0032561,GO:0007243,GO:0009207,	K07829|1|7e-12|74.7|mdo:100030293|Ras-related protein R-Ras!K07849|2|7e-12|74.7|ecb:100058975|Ras homolog enriched in brain like 1!K07827|3|9e-12|74.3|nve:NEMVE_v1g247341|GTPase KRas	gi|31541888|ref|NP_076015.2|	9.60E-94	NF-kappa-B inhibitor-interacting Ras-like protein 1 [Mus musculus]

mmu-miR-106b-5p	NM_001143849	-20.3	1	NA	NA	GO:0044425,GO:0044444,GO:0043169,GO:0019002,GO:0004114,GO:0042802,GO:0016208,GO:0023052,GO:0009169,GO:00460	K01120|1|0.0|1769|mmu:207728|3',5'-cyclic-nucleotide phosphodiesterase [EC:3.1.4.17]	gi|219803141|ref|NP_001137321.1|	0	cGMP-dependent 3',5'-cyclic phosphodiesterase isoform PDE2A2 precursor [Mus musculus]
mmu-miR-106b-5p	NM_013821	-22.6	0.970785	NA	NA	GO:0031224,GO:0019866,GO:0005740,GO:0033764,GO:0016863,GO:0010038,GO:0021543,GO:0051384,GO:0006826,GO:0006694,GO:0003006,	K00070|1|0.0|762|mmu:15497|3beta-hydroxy-delta5-steroid dehydrogenase / steroid delta-isomerase [EC:1.1.1.145 5.3.3.1]	gi|162461883|ref|NP_038849.2|	0	3 beta-hydroxysteroid dehydrogenase/Delta 5-->4-isomerase type 6 [Mus musculus]
mmu-miR-106b-5p	NM_146392	-23.3	0.71149	NA	NA	GO:0031224,GO:0004930,GO:0023052,GO:0007606,	K04257|1|0.0|644|mmu:258387|olfactory receptor	gi|33238902|ref|NP_666504.1|	0	olfactory receptor 720 [Mus musculus]

mmu-miR-106b-5p	NM_008079	-21.1	0.999998	NA	NA	GO:0000323,GO:0004553,GO:0043167,GO:0006681,	K01202|1|0.0|1363|mmu:14420|galactosylceramidase [EC:3.2.1.46]	gi|93102411|ref|NP_032105.2|	0	galactocerebrosidase precursor [Mus musculus]
mmu-miR-106b-5p	NM_033476	-20.5	1	NA	NA	GO:0043234,GO:0043231,GO:0003676,GO:0001071,GO:0006355,	K09275|1|0.0|958|mmu:21422|transcription factor CP2 and related proteins	gi|148672133|gb|EDL04080.1|	0	transcription factor CP2, isoform CRA_a [Mus musculus]
mmu-miR-106b-5p	NM_010111	-22.3	0.999873	NA	NA	GO:0016021,GO:0005102,GO:0051704,GO:0007166,GO:0009653,GO:0001944,GO:0048869,GO:0007154,	K05463|1|6e-174|612|mmu:13642|ephrin-B	gi|74208930|dbj|BAE21211.1|	0	unnamed protein product [Mus musculus]
mmu-miR-106b-5p	NM_031249	-21.3	0.999996	NA	NA	GO:0005622,GO:0003676,	K14407|1|0.0|714|mmu:83410|cleavage stimulation factor subunit 2	gi|148709763|gb|EDL41709.1|	0	cleavage stimulation factor, 3' pre-RNA subunit 2, tau [Mus musculus]

mmu-miR-106b-5p	NM_007462	-21.3	1	NA	NA	GO:0043296,GO:0030426,GO:0030424,GO:0043231,GO:0005815,GO:0005874,GO:0000775,GO:0001726,GO:0044297,GO:0019900,GO:0050839,GO:0019207,GO:0008017,GO:0019725,GO:0045841,GO:0006915,GO:0030278,GO:0002761,GO:0050680,GO:0032880,GO:0016477,GO:00424	K02085|1|0.0|4608|mmu:11789|adenomatosis polyposis coli protein	gi|110225370|ref|NP_031488.2|	0	adenomatous polyposis coli protein [Mus musculus]

mmu-miR-106b-5p	NM_021877	-22.7	0.999916	NA	NA	GO:0044451,GO:0043169,GO:0003712,GO:0003676,GO:0001071,GO:0019899,GO:0008134,GO:0051259,GO:0006355,GO:0043392,	K11449|1|2e-62|243|cfa:479221|jumonji domain-containing protein 1C [EC:1.14.11.-]!K15601|3|9e-53|210|isc:IscW_ISCW018588|lysine-specific demethylase 3 [EC:1.14.11.-]	gi|31544072|ref|NP_068677.2|	0	protein hairless [Mus musculus]
mmu-miR-106b-5p	NM_001081148	-23.4	0.946134	NA	NA	GO:0031090,GO:0008391,GO:0016712,GO:0005506,GO:0019369,GO:0010038,GO:0043434,GO:0042493,	K07412|1|0.0|884|mmu:243881|cytochrome P450, family 2, subfamily B [EC:1.14.14.1]	gi|124487255|ref|NP_001074617.1|	0	cytochrome P450, family 2, subfamily b, polypeptide 23 precursor [Mus musculus]
mmu-miR-106b-5p	NM_001270667	-24.5	0.781677	NA	NA	-	-	-	-	-

mmu-miR-106b-5p	NR_030780	-20.7	0.999987	NA	NA	-	K15503|1|2e-13|78.6|spu:584964|serine/threonine-protein phosphatase 6 regulatory ankyrin repeat subunit B	gi|269784664|ref|NP_598732.2|	0	ankyrin repeat domain-containing protein 10 isoform 1 [Mus musculus]
mmu-miR-106b-5p	NM_001251833	-25.2	0.991293	NA	NA	GO:0031981,GO:0046914,GO:0003676,GO:0001071,GO:0006351,	K09229|1|0.0|878|mcc:706188|KRAB and SCAN domains-containing zinc finger	gi|353249892|ref|NP_001238762.1|	0	zinc finger protein 192 isoform 2 [Mus musculus]
mmu-miR-106b-5p	NM_030153	-20.5	0.999998	NA	NA	GO:0016020,GO:0031981,GO:0005488,GO:0033002,	-	gi|172072615|ref|NP_084429.2|	0	N-alpha-acetyltransferase 35, NatC auxiliary subunit [Mus musculus]
mmu-miR-106b-5p	NM_177226	-20.1	1	NA	NA	GO:0043231,GO:0046914,GO:0003676,GO:0010467,	K09228|1|0.0|644|pon:100433614|KRAB domain-containing zinc finger	gi|50510451|dbj|BAD32211.1|	0	mKIAA0326 protein [Mus musculus]

mmu-miR-106b-5p	NM_001164275	-20.1	1	NA	NA	GO:0016021,GO:0046872,	K01314|1|6e-19|97.8|mdo:100022394|coagulation factor X [EC:3.4.21.6]	gi|256017178|ref|NP_081598.1|	1.14E-100	proline rich Gla (G-carboxyglutamic acid) 1 [Mus musculus]
mmu-miR-106b-5p	NM_021308	-21.4	1	NA	NA	GO:0043186,GO:0030529,GO:0003723,GO:0006412,GO:0006396,GO:0016458,GO:0007292,GO:0007127,GO:0019827,GO:0048232,GO:0034660,GO:0006306,	K02156|1|0.0|1855|mmu:57746|aubergine	gi|10946610|ref|NP_067283.1|	0	piwi-like protein 2 [Mus musculus]

mmu-miR-106b-5p	NM_001161423	-23.2	0.999864	NA	NA	GO:0043231,GO:0043234,GO:0044444,GO:0016772,GO:0060089,GO:0046983,GO:0043169,GO:0050851,GO:0051091,GO:0006796,GO:0002224,GO:0007249,GO:0012502,GO:0002260,GO:0033554,GO:0042991,GO:00	K07210|1|0.0|649|mmu:16151|inhibitor of nuclear factor kappa-B kinase subunit gamma	gi|209862941|ref|NP_001129539.1|	0	NF-kappa-B essential modulator isoform 3 [Mus musculus]

mmu-miR-106b-5p	NM_009547	-20	1	NA	NA	GO:0043231,GO:0003676,GO:0046914,GO:0006357,	K10500|1|2e-39|165|ssc:100620358|zinc finger and BTB domain-containing protein 17!K09228|4|2e-38|162|pon:100442659|KRAB domain-containing zinc finger protein	gi|348557370|ref|XP_003464492.1|	0	PREDICTED: LOW QUALITY PROTEIN: zinc finger protein 161 homolog [Cavia porcellus]
mmu-miR-106b-5p	NM_001034907	-20	1	NA	NA	GO:0004518,GO:0046914,	-	gi|110347404|ref|NP_001030079.2|	0	probable ribonuclease ZC3H12B [Mus musculus]
mmu-miR-106b-5p	NM_182698	-20.5	1	NA	NA	GO:0005902,GO:0015629,GO:0008092,GO:0017111,GO:0032559,GO:0042471,GO:0050954,GO:0007610,	K10361|1|0.0|4514|mmu:17910|myosin XV	gi|157041248|ref|NP_001096641.1|	0	unconventional myosin-XV isoform 3 [Mus musculus]

mmu-miR-106b-5p	NM_009186	-22.4	0.989275	NA	NA	-	K12897|1|6e-58|226|mdo:100026641|transformer-2 protein	gi|62087996|dbj|BAD92445.1|	9.14E-59	splicing factor, arginine/serine-rich 10 (transformer 2 homolog, Drosophila) variant [Homo sapiens]
mmu-miR-106b-5p	NM_001165955	-20.5	1	NA	NA	GO:0005488,	K11458|1|0.0|1202|mmu:241915|polyhomeotic-like protein 3	gi|260099711|ref|NP_001159426.1|	0	polyhomeotic-like protein 3 isoform 1 [Mus musculus]

mmu-miR-106b-5p	NM_008396	-22.8	0.999174	NA	NA	GO:0009986,GO:0033267,GO:0019867,GO:0043231,GO:0005887,GO:0044444,GO:0005924,GO:0004871,GO:0005102,GO:0046983,GO:0051704,GO:0008284,GO:0032092,GO:0010557,GO:0048871,GO:0010647,GO:0031349,GO:0006970,GO:0050974,GO:0002684,GO:0048730,GO:00301	K06481|1|0.0|2219|mmu:16398|integrin alpha 2	gi|41054731|ref|NP_032422.2|	0	integrin alpha-2 precursor [Mus musculus]
mmu-miR-106b-5p	NR_073508	-22.4	0.999999	NA	NA	-	-	-	-	-

mmu-miR-106b-5p	NM_133794	-21.6	0.999493	NA	NA	GO:0043234,GO:0044429,GO:0003676,GO:0019900,GO:0004812,GO:0032559,GO:0006418,GO:0043066,	K01886|1|0.0|1506|rno:290868|glutaminyl-tRNA synthetase [EC:6.1.1.18]	gi|269954677|ref|NP_598555.2|	0	glutaminyl-tRNA synthetase isoform 1 [Mus musculus]
mmu-miR-106b-5p	NM_025555	-21.7	0.941596	NA	NA	-	-	gi|12859593|dbj|BAB31704.1|	3.06E-81	unnamed protein product [Mus musculus]
mmu-miR-106b-5p	NM_023066	-20.2	1	NA	NA	GO:0031227,GO:0046872,GO:0016706,GO:0016701,GO:0005198,GO:0018126,GO:0003012,GO:0006874,GO:0035107,GO:00082	K00476|1|0.0|1231|mmu:65973|aspartate beta-hydroxylase [EC:1.14.11.16]	gi|125628659|ref|NP_075553.2|	0	aspartyl/asparaginyl beta-hydroxylase isoform 1 [Mus musculus]
mmu-miR-106b-5p	NM_001014288	-22.4	0.999999	NA	NA	GO:0016021,GO:0004725,GO:0005488,GO:0004871,GO:0007167,GO:0006470,	K06777|1|0.0|2517|rno:313278|protein tyrosine phosphatase, receptor type, D [EC:3.1.3.48]	gi|90403603|ref|NP_035341.2|	0	receptor-type tyrosine-protein phosphatase delta isoform B precursor [Mus musculus]

mmu-miR-106b-5p	NM_010019	-23	0.943939	NA	NA	GO:0044424,GO:0005515,GO:0004674,GO:0032559,GO:0035556,GO:0006464,GO:0012502,GO:0006796,	K08803|1|0.0|730|mmu:13143|death-associated protein kinase [EC:2.7.11.1]	gi|34328167|ref|NP_034149.2|		0	death-associated protein kinase 2 [Mus musculus]
mmu-miR-106b-5p	NM_010248	-22.5	0.99998	NA	NA	GO:0032991,GO:0044444,GO:0016020,GO:0030674,GO:0035091,GO:0048870,GO:0002573,GO:0007167,GO:0048017,GO:0033008,GO:0008283,	K08091|1|0.0|1317|mmu:14389|growth factor receptor bound protein 2-associated protein 2	gi|247270049|ref|NP_034378.2|		0	GRB2-associated-binding protein 2 isoform 1 [Mus musculus]
mmu-miR-106b-5p	NM_001164581	-20.8	0.999992	NA	NA	GO:0005488,	K09228|1|0.0|930|mmu:234413|KRAB domain-containing zinc finger protein	gi|257196138|ref|NP_001158053.1|		0	uncharacterized protein LOC234413 [Mus musculus]

mmu-miR-106b-5p	NM_001164433	-25.4	0.837771	NA	NA	GO:0043234,GO:0043232,GO:0046914,GO:0016491,GO:0019904,GO:0008152,GO:0006996,GO:0023052,	K06115|1|4e-14|81.6|tsp:Tsp_07859|spectrin beta	gi|256574763|ref|NP_001157905.1|	0	protein-methionine sulfoxide oxidase MICAL1 isoform 2 [Mus musculus]
mmu-miR-106b-5p	NM_029425	-25.1	0.918144	NA	NA	GO:0031224,GO:0003824,	K01080|1|1e-29|133|dre:557680|phosphatidate phosphatase [EC:3.1.3.4]	gi|148680413|gb|EDL12360.1|	0	RIKEN cDNA 4833424O15, isoform CRA_b [Mus musculus]
mmu-miR-106b-5p	NM_007506	-20	0.995466	NA	NA	GO:0031224,GO:0005753,GO:0033177,GO:0015078,GO:0005488,GO:0022900,GO:0006839,GO:0006754,GO:0015988,GO:00100	K02128|1|3e-55|215|mmu:11951|F-type H+-transporting ATPase subunit c [EC:3.6.3.14]	gi|31982497|ref|NP_031532.2|	1.73E-54	ATP synthase lipid-binding protein, mitochondrial [Mus musculus]

mmu-miR-106b-5p	NM_021458	-20	1	NA	NA	GO:0044456,GO:0043005,GO:0044297,GO:0031253,GO:0044424,GO:0031224,GO:0019904,GO:0004888,GO:0007411,GO:0014020,GO:0008544,GO:0030901,GO:0006357,GO:0016055,GO:0045137,GO:0021548,GO:0021543,GO:0042471,GO:0007187,GO:0001738,GO:0016477,GO:00217	K02329|1|0.0|1358|mmu:14365|frizzled 3	gi|148704084|gb|EDL36031.1|	0	frizzled homolog 3 (Drosophila), isoform CRA_a [Mus musculus]
mmu-miR-106b-5p	NM_146780	-20.9	0.992794	NA	NA	GO:0031224,GO:0004930,GO:0007606,	K04257|1|4e-168|591|mmu:258776|olfactory	gi|22129287|ref|NP_666991.1|	2.66E-167	olfactory receptor 715 [Mus musculus]

mmu-miR-106b-5p	NM_207205	-21.4	1	NA	NA	GO:0031224,	K06522|1|0.0|2327|mmu:78908|immunoglobulin superfamily, member 2/3	gi|37359966|dbj|BAC97961.1|	0	mKIAA0466 protein [Mus musculus]
mmu-miR-106b-5p	NM_001164733	-21.2	0.999712	NA	NA	GO:0031224,GO:0016776,GO:0019904,GO:0065003,	K06103|1|9e-115|415|ptr:100608149|calcium/calmodulin-dependent serine protein kinase [EC:2.7.11.1]	gi|257900522|ref|NP_001158205.1|	0	MAGUK p55 subfamily member 6 isoform a [Mus musculus]
mmu-miR-106b-5p	NM_181274	-20.5	1	NA	NA	-	K09228|1|0.0|1083|mmu:66869|KRAB domain-containing zinc finger protein	gi|148696799|gb|EDL28746.1|	0	mCG23322, isoform CRA_a [Mus musculus]

mmu-miR-107-3p	NM_001001144	-29.2	0.524255	-43.16	324	GO:0032991,GO:0030134,GO:0031224,GO:0031090,GO:0012505,GO:0005515,GO:0032934,GO:0016125,GO:0006633,GO:0043434,GO:0010894,GO:0010870,GO:00325	K10260|1|6e-17|91.3|pon:100173626|F-box and WD-40 domain protein 7	gi|148677071|gb|EDL09018.1|	0	SREBP cleavage activating protein, isoform CRA_a [Mus musculus]
mmu-miR-107-3p	NM_001001985	-26.4	0.966488	-26.08	161	GO:0005789,GO:0005740,GO:0008080,	-	gi|134288912|ref|NP_001001985.3|	4.43E-124	N-acetylaspartate synthetase [Mus musculus]
mmu-miR-107-3p	NM_001004062	-28.2	0.768385	-25.99	164	GO:0031981,GO:0016020,GO:0005488,GO:0003713,GO:0006357,GO:0051704,	K15309|1|0.0|1067|mmu:382056|CREB-regulated transcription coactivator	gi|37360010|dbj|BAC97983.1|	0	mKIAA0616 protein [Mus musculus]

mmu-miR-107-3p	NM_001004148	-25.3	0.941979	-50.76	322	GO:0044459,GO:0031224,GO:0015556,GO:0015291,GO:0015142,GO:0015744,GO:0006842,GO:0035725,	K14445|1|0.0|1066|mmu:237831|solute carrier family 13 (sodium-dependent dicarboxylate transporter), member 2/3/5	gi|51921291|ref|NP_001004148.1|	0	solute carrier family 13 member 5 [Mus musculus]
mmu-miR-107-3p	NM_001004761	-25.9	0.988489	-30.79	173	GO:0031224,GO:0004888,GO:0007166,	K08469|1|0.0|2289|mmu:241263|G protein-coupled receptor 158	gi|52350563|ref|NP_001004761.1|	0	probable G-protein coupled receptor 158 precursor [Mus musculus]
mmu-miR-107-3p	NM_001005420	-31.6	0.304559	-25.47	181	GO:0043231,GO:0005488,	-	gi|50510571|dbj|BAD32271.1|	0	mKIAA0649 protein [Mus musculus]
mmu-miR-107-3p	NM_001011721	-31.8	0.039552	-26.71	164	GO:0004871,GO:0023052,	K04257|1|3e-178|624|mmu:258218|olfactory	gi|255069766|ref|NP_001011721.2|	1.88E-177	olfactory receptor 102 [Mus musculus]

mmu-miR-107-3p	NM_001024478	-31.1	0.199043	-25.29	155	GO:0031224,GO:0046872,GO:0016337,	K04600|1|4e-34|147|tad:TRIADDRAFT_62266|cadherin EGF LAG seven-pass G-type receptor 1 (flamingo)!K06802|4|1e-31|139|dre:568370|cadherin 10, type 2, T2-cadherin!K04601|5|2e-31|139|nve:NEMVE_v1g84228|cadherin EGF LAG	gi|67846107|ref|NP_001019649.1|	0	cadherin-related family member 3 precursor [Mus musculus]
mmu-miR-107-3p	NM_001024619	-30.7	0.19494	-27.31	160	-	K08130|1|0.0|709|mmu:244152|tsukushi (leucine-rich repeat-containing protein 54)	gi|26330678|dbj|BAC29069.1|	0	unnamed protein product [Mus musculus]

mmu-miR-107-3p	NM_001024720	-25.2	0.999994	-42.86	315	GO:0044459,GO:0044444,GO:0005578,GO:0046872,GO:0006464,GO:0050953,	K12567|1|0.0|1253|rno:84015|titin [EC:2.7.11.1]	gi|154689979|ref|NP_001019891.2|	0	hemicentin 1 precursor [Mus musculus]
mmu-miR-107-3p	NM_001024837	-27.8	0.86128	-44.38	318	GO:0031981,GO:0043169,GO:0003723,GO:0004000,GO:0016553,GO:0006396,	K13194|1|0.0|1286|mmu:110532|double stranded RNA-specific editase B [EC:3.5.-.-]	gi|67625747|ref|NP_001020008.1|	0	double-stranded RNA-specific editase 1 isoform 2 [Mus musculus]
mmu-miR-107-3p	NM_001024918	-29.2	0.491532	-45.17	314	GO:0043231,GO:0003676,GO:0006357,GO:0021510,GO:0007420,GO:0009653,	K09174|1|0.0|1402|mmu:71137|regulatory factor X 4	gi|148689463|gb|EDL21410.1|	0	regulatory factor X, 4 (influences HLA class II expression), isoform CRA_c [Mus musculus]

mmu-miR-107-3p	NM_001024955	-27.3	0.883766	-25.2	151	GO:0043234,GO:0043231,GO:0044444,GO:0016020,GO:0035258,GO:0017046,GO:0019900,GO:0035014,GO:0019902,GO:0035004,GO:0070851,GO:0001952,GO:0006357,GO:0030098,GO:0001932,GO:0043434,GO:0007162,GO:0045661,GO:0019318,GO:0007169,GO:0030162,GO:00082	K02649|1|0.0|862|mmu:18708|phosphoinositide-3-kinase, regulatory subunit	gi|68299809|ref|NP_001020126.1|	0	phosphatidylinositol 3-kinase regulatory subunit alpha isoform 1 [Mus musculus]
mmu-miR-107-3p	NM_001025106	-27.3	0.822056	-45.24	307	GO:0016020,	-	gi|68342032|ref|NP_001020277.1|	0	transmembrane protein 201 isoform a [Mus musculus]

mmu-miR-107-3p	NM_001029936	-26.1	0.979684	-26.59	156	GO:0043231,	K11498|1|3e-20|102|mdo:100013897|centromeric protein E	gi|60360496|dbj|BAD90492.1|	0	mKIAA4061 protein [Mus musculus]
mmu-miR-107-3p	NM_001033145	-29.8	0.411239	-27.26	152	GO:0005798,GO:0030120,	-	gi|92058707|sp|Q3USZ8.1|DIA1_MOUSE	0	RecName: Full=Deleted in autism protein 1 homolog; Flags: Precursor
mmu-miR-107-3p	NM_001033331	-30.2	0.543389	-28.18	153	-	K10382|1|1e-08|64.3|spu:756550|dystonin	gi|120407058|ref|NP_001028503.2|	0	GAS2-like protein 3 [Mus musculus]

mmu-miR-107-3p	NM_001033369	-31.8	0.417366	-26.23	175	GO:0044424,GO:0031224,GO:0043235,GO:0070696,GO:0032559,GO:0043169,GO:0017002,GO:0032403,GO:0007179,GO:0006796,GO:0022602,GO:0050796,GO:0009855,GO:0021543,GO:0043434,GO:0006915,GO:0048869,GO:0006464,GO:0009746,GO:0022037,GO:0001832,GO:00108	K13568|1|0.0|774|mcc:697826|activin receptor type-1C [EC:2.7.11.30]	gi|122890143|emb|CAM13261.1|	0	activin A receptor type IC [Mus musculus]
mmu-miR-107-3p	NM_001033375	-34	0.139381	-26.66	169	GO:0043234,GO:0016192,	-	gi|126090572|ref|NP_001028547.2|	0	WASH complex subunit 7 [Mus musculus]

mmu-miR-107-3p	NM_001033481	-27	0.903459	-25.88	152	GO:0043231,GO:0016020,GO:0031641,GO:0006355,GO:0014003,	-	gi|242247266|ref|NP_001028653.1|	0	myelin gene regulatory factor [Mus musculus]
mmu-miR-107-3p	NM_001033776	-25.5	0.911259	-44.85	301	-	-	gi|76253890|ref|NP_001028948.1|	0	vertnin [Mus musculus]
mmu-miR-107-3p	NM_001034115	-25.8	0.992462	-46.55	315	GO:0016020,GO:0032947,GO:0035254,GO:0071855,	K15009|1|0.0|2565|mmu:243961|SH3 and multiple ankyrin repeat domains protein	gi|283549150|ref|NP_001029287.1|	0	SH3 and multiple ankyrin repeat domains protein 1 [Mus musculus]

mmu-miR-107-3p	NM_001037736	-30	0.49025	-30.82	168	GO:0044464,GO:0005088,GO:0019222,GO:0007266,	K05156|1|1e-24|117|gga:419067|tumor necrosis factor receptor superfamily member 19-like!K08878|2|2e-20|103|dre:560226|breakpoint cluster region protein [EC:2.7.11.1]!K04436|3|6e-20|101|ssc:100521718|mitogen-activated protein	gi|60360312|dbj|BAD90400.1|		0	mKIAA0294 protein [Mus musculus]

mmu-miR-107-3p	NM_001037938	-29.5	0.164226	-27.7	164	GO:0005635,GO:0042579,GO:0016620,GO:0016616,GO:0001523,	K11147|1|3e-147|522|mmu:28200|dehydrogenase/reductase SDR family member 4 [EC:1.1.-.-	gi|256220343|ref|NP_001033027.2|	2.07E-146	dehydrogenase/reductase SDR family member 4 isoform 1 [Mus musculus]
mmu-miR-107-3p	NM_001039167	-27.2	0.829655	-27.64	160	GO:0043231,GO:0044297,GO:0003676,GO:0006396,	K14946|1|6e-169|596|rno:287847|RNA binding protein fox-1	gi|85677497|ref|NP_001034256.1|	1.25E-170	RNA binding protein fox-1 homolog 3 isoform 1 [Mus musculus]
mmu-miR-107-3p	NM_001039168	-27.2	0.820229	-27.64	160	GO:0043231,GO:0044297,GO:0003676,GO:0006396,	K14946|1|4e-150|533|ecb:100050497|RNA binding protein fox-1	gi|148702730|gb|EDL34677.1|	8.41E-156	DNA segment, Chr 11, Brigham & Women's Genetics 0517 expressed, isoform CRA_d [Mus musculus]

mmu-miR-107-3p	NM_001042485	-29.9	0.32874	-25.61	170	GO:0031224,	K08443|1|7e-08|60.8|cfa:484897|egf-like module containing, mucin-like, hormone receptor-like 2	gi|148707669|gb|EDL39616.1|	0	RIKEN cDNA 1300007B12, isoform CRA_a [Mus musculus]
mmu-miR-107-3p	NM_001044308	-32.7	0.352452	-69.46	466	GO:0034704,GO:0005488,GO:0005245,GO:0006935,GO:0032412,GO:0023052,GO:0070838,	K04856|1|0.0|3385|mmu:239556|voltage-dependent calcium channel T type alpha-1I	gi|157057194|ref|NP_001037773.2|	0	calcium channel, voltage-dependent, alpha 1I subunit [Mus musculus]

mmu-miR-107-3p	NM_001045536	-30	0.744678	-48.69	333	GO:0000152,GO:0046914,GO:0007088,	K12233|1|7e-20|102|bfo:BRAFLDRAFT_276868|E3 ubiquitin-protein ligase HECTD3 [EC:6.3.2.19]!K11970|2|1e-19|101|xtr:100145662|p53-associated parkin-like cytoplasmic protein!K10613|3|1e-19|101|mcc:699545|cullin 7	gi|113865905|ref|NP_001039001.1|	0	zinc finger ZZ-type and EF-hand domain-containing protein 1 [Mus musculus]

mmu-miR-107-3p	NM_001077495	-27.3	0.91853	-25.2	151	GO:0005942,GO:0043231,GO:0016020,GO:0035091,GO:0035258,GO:0017046,GO:0035014,GO:0019900,GO:0019902,GO:0035004,GO:0070851,GO:0002027,GO:0001952,GO:0032868,GO:0006357,GO:0001932,GO:0030098,GO:0048017,GO:0007162,GO:0045661,GO:0010828,GO:00193	K02649|1|0.0|1258|mcc:698996|phosphoinositide-3-kinase, regulatory subunit	gi|117320524|ref|NP_001070963.1|	0	phosphatidylinositol 3-kinase regulatory subunit alpha isoform 2 [Mus musculus]
mmu-miR-107-3p	NM_001079876	-30.2	0.543547	-28.18	153	-	K10382|1|1e-08|64.3|spu:756550|dystonin	gi|120407058|ref|NP_001028503.2|	0	GAS2-like protein 3 [Mus musculus]

mmu-miR-107-3p	NM_001080127	-30.5	0.146389	-27.92	158	GO:0016604,GO:0044444,GO:0003676,GO:0000377,GO:0006405,GO:0000956,GO:0006397,GO:0006353,	K14325|1|5e-44|179|ssc:100737559|RNA-binding protein with serine-rich domain 1	gi|397469246|ref|XP_003806272.1|	3.47E-43	PREDICTED: RNA-binding protein with serine-rich domain 1-like [Pan paniscus]
mmu-miR-107-3p	NM_001081017	-30.9	0.54229	-29.22	164	GO:0044464,	-	gi|313661493|ref|NP_001074486.2|	0	protein unc-79 homolog [Mus musculus]
mmu-miR-107-3p	NM_001081083	-28.2	0.536791	-51.5	325	-	K02105|1|4e-07|58.2|tsp:Tsp_08140|catenin beta 1!K10056|2|3e-06|55.5|xla:398496|junction plakoglobin	gi|124487327|ref|NP_001074552.1|	0	armadillo repeat-containing protein 3 [Mus musculus]
mmu-miR-107-3p	NM_001081103	-29.6	0.51588	-27.18	154	GO:0031224,GO:0043231,GO:0031090,GO:0046872,GO:0016247,GO:0006875,	K16059|1|0.0|1236|mmu:116873|stromal interaction molecule	gi|219519777|gb|AAI45002.1|	0	Stim2 protein [Mus musculus]

mmu-miR-107-3p	NM_001081158	-26.1	0.96065	-42.3	311	-	K03255|1|0.0|2624|mmu:74148|protein TIF31	gi|124487201|ref|NP_001074627.1|	0	protein KIAA0664 [Mus musculus]
mmu-miR-107-3p	NM_001081214	-27.4	0.84585	-26.79	156	GO:0043231,GO:0003676,GO:0006351,	K07202|1|2e-44|182|mgp:100542168|peroxisome proliferative activated receptor, gamma, coactivator 1, alpha	gi|124487055|ref|NP_001074683.1|	0	peroxisome proliferator-activated receptor gamma coactivator-related protein 1 [Mus musculus]
mmu-miR-107-3p	NM_001081220	-25.8	0.996837	-53.6	295	GO:0031224,GO:0004888,	K08469|1|0.0|683|tgu:100229682|G protein-coupled receptor 158	gi|124487045|ref|NP_001074689.1|	0	probable G-protein coupled receptor 179 precursor [Mus musculus]

mmu-miR-107-3p	NM_001081336	-31.9	0.200889	-26.35	158	GO:0043231,GO:0016020,GO:0043169,GO:0016301,GO:0032559,GO:0001775,GO:0007186,GO:0006796,GO:0006461,GO:00075	K00901|1|0.0|2189|mmu:380921|diacylglycerol kinase [EC:2.7.1.107]	gi|124486741|ref|NP_001074805.1|	0	diacylglycerol kinase eta [Mus musculus]
mmu-miR-107-3p	NM_001081418	-31.1	0.351398	-48.07	330	GO:0005488,	K05626|1|6e-06|55.1|rno:29515|atrophin-1 (dentatorubral-pallidoluysian atrophy protein)	gi|124487237|ref|NP_001074887.1|	0	glioma tumor suppressor candidate region gene 1 protein [Mus musculus]
mmu-miR-107-3p	NM_001081437	-27.6	0.762292	-76.17	484	GO:0031012,GO:0046872,GO:0005198,GO:0010810,	K06825|1|1e-74|282|xtr:100490545|fibrillin 1	gi|124517693|ref|NP_001074906.1|	0	fibulin-2 isoform b precursor [Mus musculus]

mmu-miR-107-3p	NM_001083807	-32.3	0.146068	-27.19	172	GO:0031981,GO:0030674,	K15691|1|2e-11|72.4|mgp:100545594|E3 ubiquitin-protein ligase RFWD3 [EC:6.3.2.19]	gi|139947575|ref|NP_001077276.1|	0	RUN and SH3 domain-containing protein 1 isoform 2 [Mus musculus]
mmu-miR-107-3p	NM_001083955	-34.7	0.004013	-26.48	171	GO:0043234,GO:0015935,GO:0022892,GO:0005506,GO:0051931,GO:0015669,GO:0051259,GO:0000302,GO:0008219,GO:0043009,GO:0008217,GO:0048468,GO:00427	K13822|1|1e-77|289|mmu:110257|hemoglobin subunit alpha	gi|145301549|ref|NP_001077424.1|	6.52E-77	hemoglobin alpha, adult chain 2 [Mus musculus]
mmu-miR-107-3p	NM_001085499	-31.2	0.132491	-25.58	168	-	-	gi|148225760|ref|NP_001078968.1|	0	uncharacterized protein LOC209005 [Mus musculus]

mmu-miR-107-3p	NM_001097623	-33.9	0.109965	-26.04	164	GO:0043231,GO:0046872,GO:0001071,GO:0003677,GO:0005515,GO:0003713,GO:0065008,GO:0006355,GO:0032501,GO:00082	K11829|1|4e-10|68.9|xla:398247|REST corepressor 1	gi|148271107|ref|NP_001091092.1|	0	transcriptional-regulating factor 1 isoform 1 [Mus musculus]
mmu-miR-107-3p	NM_001100452	-30.9	0.440303	-28.06	175	-	-	gi|226958520|ref|NP_001093922.1|	0	uncharacterized protein KIAA0240 [Mus musculus]
mmu-miR-107-3p	NM_001103162	-27.8	0.718891	-43.16	324	GO:0032991,GO:0030134,GO:0031224,GO:0031090,GO:0012505,GO:0005515,GO:0032934,GO:0016125,GO:0006633,GO:0043434,GO:0010894,GO:0010870,GO:00325	K10260|1|6e-17|91.3|pon:100173626|F-box and WD-40 domain protein 7	gi|148677071|gb|EDL09018.1|	0	SREBP cleavage activating protein, isoform CRA_a [Mus musculus]

mmu-miR-107-3p	NM_001111030	-31.8	0.430549	-26.23	175	GO:0044424,GO:0031224,GO:0043235,GO:0070696,GO:0032559,GO:0043169,GO:0017002,GO:0032403,GO:0007179,GO:0006796,GO:0022602,GO:0050796,GO:0009855,GO:0021543,GO:0043434,GO:0006915,GO:0048869,GO:0006464,GO:0009746,GO:0022037,GO:0001832,GO:00108	K13568|1|0.0|951|rno:245921|activin receptor type-1C [EC:2.7.11.30]	gi|161333831|ref|NP_001104500.1|	0	activin receptor type-1C isoform 1 precursor [Mus musculus]

mmu-miR-107-3p	NM_001111272	-25.5	0.890588	-47	323	GO:0031981,GO:0015630,GO:0003723,GO:0051234,	K13194|1|2e-06|55.8|nve:NEMVE_v1g189235|double stranded RNA-specific editase B [EC:3.5.-.-]	gi|162287083|ref|NP_001104742.1|	0	double-stranded RNA-binding protein Staufen homolog 2 isoform 2 [Mus musculus]
mmu-miR-107-3p	NM_001112729	-29.1	0.597935	-26.13	162	-	-	gi|31542190|ref|NP_659176.2|	0	TELO2-interacting protein 2 isoform a [Mus musculus]
mmu-miR-107-3p	NM_001128145	-27.3	0.67368	-45.81	334	GO:0044464,GO:0002360,GO:0001816,	K06545|1|0.0|679|aml:100470523|CD163	gi|189458795|ref|NP_001121617.1|	0	scavenger receptor protein family member isoform 1 precursor [Mus musculus]
mmu-miR-107-3p	NM_001128146	-28.1	0.592685	-45.81	334	GO:0002360,GO:0001816,	K06545|1|0.0|688|cfa:477704|CD163 antigen	gi|189458797|ref|NP_001121618.1|	0	scavenger receptor protein family member isoform 2 precursor [Mus musculus]

mmu-miR-107-3p	NM_001130408	-25.1	0.849874	-25.01	153	GO:0016020,GO:0043231,GO:0030016,GO:0012505,GO:0032561,GO:0004871,GO:0017111,GO:0030003,GO:0048200,GO:0009207,GO:0048856,GO:0050688,GO:00355	K07937|1|1e-100|367|acs:100552859|ADP-ribosylation factor 1	gi|4502201|ref|NP_001649.1|	1.01E-99	ADP-ribosylation factor 1 [Homo sapiens]
mmu-miR-107-3p	NM_001136237	-31.1	0.124436	-46.82	322	GO:0044459,GO:0046915,GO:0072503,GO:0000041,GO:0009267,	K14711|1|0.0|756|mmu:72002|solute carrier family 39 (zinc transporter), member 5	gi|148692617|gb|EDL24564.1|	0	solute carrier family 39 (metal ion transporter), member 5, isoform CRA_b [Mus musculus]
mmu-miR-107-3p	NM_001145926	-26.6	0.645327	-25.2	165	GO:0016020,	-	gi|354467297|ref|XP_003496106.1|	3.02E-102	PREDICTED: uncharacterized protein C12orf53 homolog [Cricetulus griseus]
mmu-miR-107-3p	NM_001145927	-26.6	0.642287	-25.2	165	GO:0016020,	-	gi|354467297|ref|XP_003496106.1|	2.99E-102	PREDICTED: uncharacterized protein C12orf53 homolog [Cricetulus griseus]

mmu-miR-107-3p	NM_001159626	-33.4	0.021058	-27.6	164	GO:0044429,GO:0016790,GO:0046914,GO:0006749,GO:0048232,	K01069|1|3e-151|535|rno:24439|hydroxyacylglutathione hydrolase [EC:3.1.2.6]	gi|257051017|sp|Q99KB8.2|GLO2_MOUSE	4.22E-154	RecName: Full=Hydroxyacylglutathione hydrolase, mitochondrial; AltName: Full=Glyoxalase II; Short=Glx II; Flags: Precursor
mmu-miR-107-3p	NM_001162913	-27.5	0.630186	-45.51	313	GO:0031224,GO:0001871,GO:0004888,GO:0023052,GO:0002376,	K04005|1|3e-07|58.5|rno:54243|complement receptor related protein!K04012|2|3e-07|58.5|rno:289395|complement receptor type 2!K10955|3|1e-06|56.2|xtr:100493286|intestinal mucin-	gi|251823826|ref|NP_001156385.1|	0	sushi domain-containing protein 2 isoform 2 precursor [Mus musculus]

mmu-miR-107-3p	NM_001163487	-30.7	0.226389	-50.27	311	GO:0043234,GO:0044459,GO:0044444,GO:0032559,GO:0008443,GO:0043169,GO:0070061,GO:0019899,GO:0006007,GO:0006000,GO:0016310,GO:0050796,GO:0019725,GO:0051260,GO:0042593,	K00850|1|0.0|1537|mmu:18642|6-phosphofructokinase [EC:2.7.1.11]	gi|332206389|ref|XP_003252273.1|	0	PREDICTED: 6-phosphofructokinase, muscle type isoform 2 [Nomascus leucogenys]

mmu-miR-107-3p	NM_001163488	-29.7	0.317579	-50.27	311	GO:0043234,GO:0044459,GO:0044444,GO:0032559,GO:0008443,GO:0043169,GO:0070061,GO:0019899,GO:0006007,GO:0006000,GO:0016310,GO:0050796,GO:0019725,GO:0051260,GO:0042593,	K00850|1|0.0|1537|mmu:18642|6-phosphofructokinase [EC:2.7.1.11]	gi|254553344|ref|NP_067489.3|	0	6-phosphofructokinase, muscle type [Mus musculus]
mmu-miR-107-3p	NM_001163746	-30.8	0.330333	-26.02	157	GO:0044444,GO:0046872,GO:0016868,GO:0006045,GO:0048534,GO:0048232,GO:0006006,GO:0009790,GO:0006487,	K01836|1|0.0|996|mmu:109785|phosphoacetylglucosamine mutase [EC:5.4.2.3]	gi|255522941|ref|NP_001157218.1|	0	phosphoacetylglucosamine mutase isoform 2 [Mus musculus]
mmu-miR-107-3p	NM_001163752	-33	0.073067	-26.97	168	-	-	gi|255522949|ref|NP_001157224.1|	0	uncharacterized protein C19orf57 homolog isoform 2 [Mus musculus]

mmu-miR-107-3p	NM_001164572	-25.9	0.957635	-25.6	166	GO:0043231,GO:0004672,GO:0046872,GO:0032559,GO:0042981,GO:0006796,GO:0030097,GO:0006464,	K08802|1|0.0|1256|mmu:20623|SNF related kinase [EC:2.7.11.1]	gi|223462828|gb|AAI50743.1|	0	SNF related kinase [Mus musculus]
mmu-miR-107-3p	NM_001166506	-25.5	0.974422	-42.68	320	GO:0031224,GO:0043231,GO:0051234,	-	gi|148702667|gb|EDL34614.1|	0	SEC14-like 1 (S. cerevisiae), isoform CRA_c [Mus musculus]
mmu-miR-107-3p	NM_001166507	-25.5	0.967217	-42.68	320	GO:0031224,GO:0043231,GO:0051234,	-	gi|148702667|gb|EDL34614.1|	0	SEC14-like 1 (S. cerevisiae), isoform CRA_c [Mus musculus]
mmu-miR-107-3p	NM_001168539	-30.7	0.195274	-27.31	160	-	K08130|1|0.0|709|mmu:244152|tsukushi (leucine-rich repeat-containing protein 54)	gi|26330678|dbj|BAC29069.1|	0	unnamed protein product [Mus musculus]
mmu-miR-107-3p	NM_001195089	-26.4	0.770157	-45.9	317	GO:0031224,GO:0043231,	-	gi|304434590|ref|NP_001182017.1|	0	transmembrane channel-like protein 8 isoform A [Mus musculus]
mmu-miR-107-3p	NM_001195090	-26.4	0.769773	-45.9	317	GO:0031224,GO:0043231,	-	gi|304434590|ref|NP_001182017.1|	0	transmembrane channel-like protein 8 isoform A [Mus musculus]

mmu-miR-107-3p	NM_001199431	-29.9	0.504308	-26.62	159	GO:0000785,GO:0032991,GO:0043231,GO:0000775,GO:0046914,GO:0000166,GO:0003886,GO:0043565,GO:0043566,GO:0019899,GO:0006575,GO:0006355,GO:0008283,GO:0031062,GO:0032776,GO:0006790,GO:00	K00558|1|0.0|2940|mmu:13433|DNA (cytosine-5-)-methyltransferase [EC:2.1.1.37]	gi|148693194|gb|EDL25141.1|	0	DNA methyltransferase (cytosine-5) 1, isoform CRA_c [Mus musculus]
mmu-miR-107-3p	NM_001199988	-29.1	0.591988	-26.13	162	-	-	gi|316983174|ref|NP_001186917.1|	0	TELO2-interacting protein 2 isoform b [Mus musculus]

mmu-miR-107-3p	NM_001204411	-32.2	0.115723	-25.09	163	GO:0043231,GO:0042383,GO:0005887,GO:0005515,GO:0031420,GO:0032559,GO:0005242,GO:0015980,GO:0050796,GO:0048545,GO:0071805,GO:0019318,GO:0006873,GO:00	K05004|1|8e-174|611|rno:83535|potassium inwardly-rectifying channel subfamily J member 11	gi|26328501|dbj|BAC27989.1|	8.77E-174	unnamed protein product [Mus musculus]
mmu-miR-107-3p	NM_001253795	-29.7	0.167048	-53.26	329	GO:0043231,GO:0031332,GO:0044444,GO:0042802,GO:0003723,GO:0016246,GO:0033673,GO:0006412,GO:0006355,GO:0050687,GO:0035196,GO:0045069,	-	gi|359385734|ref|NP_001240724.1|	1.41E-135	RISC-loading complex subunit TARBP2 isoform 2 [Mus musculus]

mmu-miR-107-3p	NM_001254953	-32.5	0.041264	-47.21	327	-	K01395|1|1e-58|227|ptr:462745|meprin A, alpha [EC:3.4.24.18]!K15503|2|9e-13|75.5|tgu:100228130|serine/threonine-protein phosphatase 6 regulatory ankyrin repeat subunit B	gi|363807402|ref|NP_001241882.1|	7.51E-111	predicted gene 4368 [Mus musculus]
mmu-miR-107-3p	NM_001271563	-28.2	0.532205	-51.5	325	-	-	-	-	-
mmu-miR-107-3p	NM_001271564	-28.2	0.530665	-51.5	325	-	-	-	-	-
mmu-miR-107-3p	NM_001271565	-28.2	0.510788	-51.5	325	-	-	-	-	-

mmu-miR-107-3p	NM_007476	-25.1	0.818682	-25.01	153	GO:0016020,GO:0043231,GO:0030016,GO:0012505,GO:0032561,GO:0004871,GO:0017111,GO:0030003,GO:0048200,GO:0009207,GO:0048856,GO:0050688,GO:00355	K07937|1|1e-100|367|acs:100552859|ADP-ribosylation factor 1	gi|4502201|ref|NP_001649.1|	8.96E-100	ADP-ribosylation factor 1 [Homo sapiens]
mmu-miR-107-3p	NM_007478	-26.3	0.862258	-71.08	464	GO:0016020,GO:0043231,GO:0030016,GO:0012505,GO:0032561,GO:0004871,GO:0017111,GO:0030003,GO:0048200,GO:0009207,GO:0050688,GO:0035556,	K07938|1|2e-103|374|mcc:708716|ADP-ribosylation factor 3	gi|397510982|ref|XP_003825862.1|	3.51E-106	PREDICTED: ADP-ribosylation factor 3 isoform 2 [Pan paniscus]

mmu-miR-107-3p	NM_007789	-33.6	0.198007	-27.25	163	GO:0016020,GO:0046872,GO:0005539,GO:0050807,GO:0006935,	K06794|1|0.0|2318|mmu:13004|chondroitin sulfate proteoglycan 3 (neurocan)	gi|40789268|ref|NP_031815.2|	0	neurocan core protein precursor [Mus musculus]
mmu-miR-107-3p	NM_007930	-25.6	0.970726	-41.8	305	GO:0043232,GO:0034399,GO:0008092,GO:0048731,	K10462|1|0.0|1164|mmu:13803|kelch-like protein 25/37 (ectoderm-neural cortex protein)	gi|40254359|ref|NP_031956.3|	0	ectoderm-neural cortex protein 1 [Mus musculus]
mmu-miR-107-3p	NM_007992	-27.6	0.772889	-76.17	484	GO:0031012,GO:0046872,GO:0005198,GO:0010810,	K06825|1|1e-76|290|mgp:723984|fibrillin 1	gi|124517704|ref|NP_032018.2|	0	fibulin-2 isoform a precursor [Mus musculus]

mmu-miR-107-3p	NM_008218	-34.7	0.003794	-26.48	171	GO:0043234,GO:0015935,GO:0022892,GO:0005506,GO:0051931,GO:0015669,GO:0051259,GO:0000302,GO:0008219,GO:0043009,GO:0008217,GO:0048468,GO:00427	K13822|1|1e-77|289|mmu:110257|hemoglobin subunit alpha	gi|145301549|ref|NP_001077424.1|	5.97E-77	hemoglobin alpha, adult chain 2 [Mus musculus]
mmu-miR-107-3p	NM_008482	-34.6	0.109726	-28.64	166	GO:0043256,GO:0044444,GO:0005198,GO:0016477,GO:0030030,GO:0006935,GO:0009887,GO:0008284,GO:0000904,	K05636|1|0.0|3679|mmu:16777|laminin, beta 1	gi|148704971|gb|EDL36918.1|	0	laminin B1 subunit 1 [Mus musculus]

mmu-miR-107-3p	NM_008606	-32.4	0.086802	-26.73	162	GO:0031012,GO:0046914,GO:0004175,GO:0032963,GO:0019538,	K07993|1|0.0|993|mmu:17385|matrix metalloproteinase-11 (stromelysin 3) [EC:3.4.24.-]	gi|6678894|ref|NP_032632.1|	0	stromelysin-3 precursor [Mus musculus]
mmu-miR-107-3p	NM_008733	-33.3	0.16967	-26.76	171	-	-	gi|117606368|ref|NP_032759.3|	0	nebulin-related-anchoring protein isoform S [Mus musculus]
mmu-miR-107-3p	NM_008925	-31	0.13642	-43.97	307	GO:0044432,GO:0043234,GO:0016020,GO:0016772,GO:0003676,GO:0046872,GO:0019901,GO:0006952,GO:0035556,GO:0051259,GO:0006796,GO:0001822,GO:00064	K08288|1|0.0|817|mmu:19089|protein kinase C substrate 80K-H	gi|6679465|ref|NP_032951.1|	0	glucosidase 2 subunit beta precursor [Mus musculus]

mmu-miR-107-3p	NM_009072	-27.7	0.913337	-43.2	327	GO:0016020,GO:0005815,GO:0000922,GO:0004672,GO:0032559,GO:0043169,GO:0007010,GO:0007265,GO:0014020,GO:0010638,GO:0006935,GO:0006464,	K04514|1|0.0|2400|mmu:19878|Rho-associated, coiled-coil containing protein kinase [EC:2.7.11.1]	gi|134949013|ref|NP_033098.2|	0	rho-associated protein kinase 2 [Mus musculus]
mmu-miR-107-3p	NM_009319	-29.7	0.201061	-53.26	329	GO:0043231,GO:0031332,GO:0044444,GO:0042802,GO:0003723,GO:0016246,GO:0033673,GO:0006412,GO:0006355,GO:0050687,GO:0035196,GO:0045069,	-	gi|166295185|ref|NP_033345.2|	1.71E-175	RISC-loading complex subunit TARBP2 isoform 1 [Mus musculus]

mmu-miR-107-3p	NM_009390	-32.2	0.224107	-26.86	165	GO:0046914,GO:0005515,GO:0004175,GO:0048731,GO:0019538,GO:0048869,	K09608|1|0.0|2149|mmu:21892|tolloid-like protein 1 [EC:3.4.24.-]	gi|117414180|ref|NP_033416.2|	0	tolloid-like protein 1 precursor [Mus musculus]
mmu-miR-107-3p	NM_009579	-25.5	0.981718	-25.18	160	GO:0043227,GO:0042383,GO:0031224,GO:0046915,GO:0005246,GO:0015691,GO:0072503,GO:0007269,GO:0034766,GO:0043009,GO:0009636,GO:0070588,GO:0006875,GO:00	K14688|1|0.0|813|mmu:22782|solute carrier family 30 (zinc transporter), member 1	gi|6678017|ref|NP_033605.1|	0	zinc transporter 1 [Mus musculus]

mmu-miR-107-3p	NM_009594	-34.7	0.105959	-31.34	172	GO:0031981,GO:0016020,GO:0005635,GO:0043232,GO:0044444,GO:0046914,GO:0019901,GO:0004713,GO:0003676,GO:0019904,GO:0032559,GO:0007010,GO:0006351,GO:0007599,GO:0043085,GO:0045597,GO:0006469,GO:0000084,GO:0006935,GO:00	K06619|1|0.0|1993|mmu:11350|proto-oncogene tyrosine-protein kinase ABL1 [EC:2.7.10.2]	gi|162951865|ref|NP_033724.2|	0	tyrosine-protein kinase ABL1 isoform b [Mus musculus]

mmu-miR-107-3p	NM_009661	-31.3	0.205394	-25.37	167	GO:0044424,GO:0046914,GO:0016702,GO:0040008,GO:0016477,GO:0012502,GO:0051726,GO:0006691,GO:0030855,GO:0001655,GO:0008283,	K08022|1|0.0|1356|mmu:11688|arachidonate 15-lipoxygenase (second type) / 8-lipoxygenase (S-type) [EC:1.13.11.33	gi|148678536|gb|EDL10483.1|	0	arachidonate 8-lipoxygenase [Mus musculus]
mmu-miR-107-3p	NM_009671	-32.6	0.307196	-27.01	168	GO:0031090,GO:0043231,GO:0046914,GO:0016192,	K10380|1|3e-57|226|ame:409051|ankyrin	gi|85702366|ref|NP_033801.4|	0	ankyrin repeat and FYVE domain-containing protein 1 [Mus musculus]
mmu-miR-107-3p	NM_010045	-32.5	0.034205	-25.27	165	GO:0031224,GO:0019956,GO:0004871,GO:0007166,GO:0006952,	K06574|1|2e-173|608|mmu:13349|Duffy blood group glycoprotein	gi|130502090|ref|NP_034175.2|	1.28E-172	Duffy antigen/chemokine receptor [Mus musculus]

mmu-miR-107-3p	NM_010066	-29.9	0.504109	-26.62	159	GO:0000785,GO:0032991,GO:0043231,GO:0000775,GO:0046914,GO:0000166,GO:0003886,GO:0043565,GO:0043566,GO:0019899,GO:0006575,GO:0006355,GO:0008283,GO:0031062,GO:0032776,GO:0006790,GO:00	K00558|1|0.0|2932|mmu:13433|DNA (cytosine-5-)-methyltransferase [EC:2.1.1.37]	gi|148693194|gb|EDL25141.1|	0	DNA methyltransferase (cytosine-5) 1, isoform CRA_c [Mus musculus]
mmu-miR-107-3p	NM_010088	-30.7	0.055165	-28.22	164	GO:0005102,GO:0006950,GO:0048609,	K05439|1|8e-20|98.2|ecb:100034034|prolactin	gi|6753690|ref|NP_034218.1|	8.89E-133	prolactin family 8, subfamily a, member 2 precursor [Mus musculus]

mmu-miR-107-3p	NM_010476	-25.5	0.971383	-64.28	460	GO:0031090,GO:0043231,GO:0031224,GO:0005126,GO:0033764,GO:0008203,	K13373|1|0.0|670|mmu:15490|estradiol 17beta-dehydrogenase / 3-keto steroid reductase [EC:1.1.1.62 1.1.1.270]	gi|87162470|ref|NP_034606.3|	0	3-keto-steroid reductase [Mus musculus]
mmu-miR-107-3p	NM_010602	-32.2	0.138276	-25.09	163	GO:0043231,GO:0042383,GO:0005887,GO:0005515,GO:0031420,GO:0032559,GO:0005242,GO:0015980,GO:0050796,GO:0048545,GO:0071805,GO:0019318,GO:0006873,GO:00	K05004|1|0.0|741|rno:83535|potassium inwardly-rectifying channel subfamily J member 11	gi|6754426|ref|NP_034732.1|	0	ATP-sensitive inward rectifier potassium channel 11 isoform 1 [Mus musculus]

mmu-miR-107-3p	NM_010609	-32.5	0.033693	-26.54	155	GO:0016021,GO:0005261,GO:0034220,GO:0030001,	K04918|1|1e-145|516|mmu:16530|potassium channel subfamily K member	gi|51944955|ref|NP_034739.2|	6.53E-145	potassium channel subfamily K member 7 precursor [Mus musculus]
mmu-miR-107-3p	NM_010683	-32.8	0.27481	-49.24	340	GO:0043256,GO:0046625,GO:0005198,GO:0009888,GO:0030030,GO:0048870,GO:0007044,GO:0006935,GO:0008284,GO:0065003,GO:0030198,GO:0000904,	K05635|1|0.0|3070|mmu:226519|laminin, gamma 1	gi|148707495|gb|EDL39442.1|	0	laminin, gamma 1, isoform CRA_b [Mus musculus]

mmu-miR-107-3p	NM_001042699	-23.3	0.999954	-67.41	481	GO:0031965,GO:0034992,GO:0031224,GO:0008092,GO:0007010,	K10352|1|8e-07|58.2|xtr:448762|myosin heavy chain!K10366|2|1e-06|57.8|bta:537655|dystrophin!K10362|3|1e-06|57.4|bta:540437|myosin XVIII	gi|111607453|ref|NP_001036164.1|	0	nesprin-3 isoform alpha [Mus musculus]
mmu-miR-107-5p	NM_001001335	-27.2	0.876397	-26.07	168	GO:0044424,GO:0005319,GO:0008289,GO:0006869,	K08051|1|0.0|944|rno:500132|pleckstrin homology domain containing, family A (phosphoinositide binding specific) member 8	gi|148666292|gb|EDK98708.1|	0	pleckstrin homology domain containing, family A (phosphoinositide binding specific) member 8, isoform CRA_a [Mus musculus]

mmu-miR-107-5p	NM_001004468	-27.4	0.92346	-25.4	170	GO:0043232,GO:0051427,GO:0021846,GO:0021543,GO:0007051,	K14282|1|0.0|5333|mmu:57752|transforming acidic coiled-coil-containing protein 2	gi|52486843|ref|NP_001004468.1|	0	transforming acidic coiled-coil-containing protein 2 isoform c [Mus musculus]
mmu-miR-107-5p	NM_001039700	-27.3	0.86487	-42.82	319	GO:0031224,GO:0046872,GO:0015075,GO:0030246,GO:0006810,GO:0007186,	K04989|1|0.0|3511|mmu:244646|polycystin 1L3	gi|115583681|ref|NP_001034789.2|	0	polycystic kidney disease protein 1-like 3 isoform a precursor [Mus musculus]
mmu-miR-107-5p	NM_001076554	-30.3	0.515401	-45.56	335	GO:0032991,GO:0030864,GO:0005913,GO:0031674,GO:0043227,GO:0008092,GO:0046872,GO:0000149,GO:0046983,GO:00308	K06114|1|0.0|4771|mmu:20740|spectrin alpha	gi|115496850|ref|NP_001070022.1|	0	spectrin alpha chain, brain isoform 1 [Mus musculus]

mmu-miR-107-5p	NM_001081128	-25.3	0.958922	-43.06	330	GO:0044444,GO:0019842,GO:0008172,GO:0046914,GO:0016597,GO:0048731,GO:0000097,GO:0006730,GO:0008213,GO:00067	K00548|1|0.0|2512|mmu:238505|5-methyltetrahydrofolate--homocysteine methyltransferase [EC:2.1.1.13]	gi|124487479|ref|NP_001074597.1|	0	methionine synthase [Mus musculus]

mmu-miR-107-5p	NM_001081402	-28.7	0.287895	-25.12	172	-	K02208|1|4e-98|359|bmy:Bm1_33825|cyclin-dependent kinase 8/11 [EC:2.7.11.22 2.7.11.23]!K03130|2|2e-10|68.9|bfo:BRAFLDRAFT_65485|transcription initiation factor TFIID subunit 5!K12857|4|8e-10|66.6|dre:393292|Prp8 binding	gi|124487149|ref|NP_001074871.1|		0	WD repeat-containing protein 70 [Mus musculus]

mmu-miR-107-5p	NM_001082412	-30.6	0.210604	-29.97	170	GO:0019866,GO:0031224,GO:0043231,GO:0051234,	K15110|1|9e-10|67.0|rno:171151|solute carrier family 25 (mitochondrial 2-oxodicarboxylate transporter), member 21	gi|126723223|ref|NP_001075881.1|	0	mitochondrial carrier triple repeat protein 6 isoform 1 precursor [Mus musculus]
mmu-miR-107-5p	NM_001112701	-27.1	0.563329	-41.65	307	GO:0044424,GO:0016020,GO:0005083,GO:0005488,GO:0016192,GO:0019222,GO:0031175,GO:0007010,GO:0032011,GO:00071	K12495|1|3e-41|171|bfo:BRAFLDRAFT_106693|IQ motif and SEC7 domain-containing protein	gi|395751484|ref|XP_002829550.2|	0	PREDICTED: cytohesin-2 isoform 1 [Pongo abelii]

mmu-miR-107-5p	NM_001114179	-30.6	0.167169	-29.97	170	-	K15110|1|7e-10|67.0|rno:171151|solute carrier family 25 (mitochondrial 2-oxodicarboxylate transporter), member 21	gi|126723223|ref|NP_001075881.1|	0	mitochondrial carrier triple repeat protein 6 isoform 1 precursor [Mus musculus]
mmu-miR-107-5p	NM_001159635	-28.6	0.341568	-25.64	164	GO:0003676,	-	gi|227500539|ref|NP_001153107.1|	2.83E-86	probable RNA-binding protein 18 isoform 2 [Mus musculus]
mmu-miR-107-5p	NM_001163024	-25.6	0.995111	-25.36	170	GO:0044424,GO:0005083,GO:0006892,	-	gi|28972582|dbj|BAC65707.1|	0	mKIAA1040 protein [Mus musculus]
mmu-miR-107-5p	NM_001163025	-25.6	0.99507	-25.36	170	GO:0044424,GO:0005083,GO:0006810,	-	gi|148692491|gb|EDL24438.1|	0	MON2 homolog (yeast), isoform CRA_d [Mus musculus]
mmu-miR-107-5p	NM_001163505	-28.5	0.701523	-25.11	173	-	-	gi|254826716|ref|NP_001156977.1|	0	atlastin-3 isoform 1 [Mus musculus]

mmu-miR-107-5p	NM_001163750	-25	0.799256	-26.42	168	-	K10345|1|0.0|848|mmu:79043|SPRY domain-containing SOCS box protein 3	gi|254939631|ref|NP_001157222.1|	0	SPRY domain-containing SOCS box protein 3 isoform 1 [Mus musculus]
mmu-miR-107-5p	NM_001163751	-25	0.728444	-26.42	168	-	K10345|1|3e-175|615|mmu:79043|SPRY domain-containing SOCS box protein 3	gi|254939633|ref|NP_001157223.1|	0	SPRY domain-containing SOCS box protein 3 isoform 2 [Mus musculus]
mmu-miR-107-5p	NM_001164361	-27.2	0.879763	-26.07	168	GO:0044424,GO:0005319,GO:0008289,GO:0006869,	K08051|1|0.0|972|rno:500132|pleckstrin homology domain containing, family A (phosphoinositide binding specific) member 8	gi|256355025|ref|NP_001157833.1|	0	pleckstrin homology domain-containing family A member 8 isoform 1 [Mus musculus]

mmu-miR-107-5p	NM_001164724	-26	0.74401	-27.99	156	GO:0043231,GO:0044421,GO:0005102,GO:0032722,GO:0050709,GO:0006357,GO:0001818,GO:0002696,GO:0050714,GO:0002823,GO:0031349,GO:0002828,	K12967|1|2e-151|536|mmu:77125|interleukin 33	gi|257900486|ref|NP_598536.2|	1.70E-150	interleukin-33 precursor [Mus musculus]
mmu-miR-107-5p	NM_001165948	-25.4	0.99239	-30.01	167	-	K10500|1|4e-15|86.3|rno:313666|zinc finger and BTB domain-containing protein	gi|260099686|ref|NP_001159420.1|	0	pogo transposable element with ZNF domain isoform 2 [Mus musculus]

mmu-miR-107-5p	NM_001172472	-30.9	0.101152	-25.87	177	GO:0043005,GO:0030136,GO:0016020,GO:0001727,GO:0046872,GO:0032559,GO:0003676,GO:0019903,GO:0006066,GO:0007186,GO:0030149,GO:0034614,GO:0001932,GO:0007346,GO:0008284,GO:0032611,GO:0051091,GO:0045765,GO:0019932,GO:0006458,GO:0010243,GO:00069	K04718|1|0.0|689|mmu:20698|sphingosine kinase [EC:2.7.1.91]	gi|148702636|gb|EDL34583.1|	0	sphingosine kinase 1, isoform CRA_c [Mus musculus]

mmu-miR-107-5p	NM_001172473	-30.9	0.093531	-25.87	177	GO:0043005,GO:0030136,GO:0016020,GO:0001727,GO:0046872,GO:0032559,GO:0003676,GO:0019903,GO:0006066,GO:0007186,GO:0030149,GO:0034614,GO:0001932,GO:0007346,GO:0008284,GO:0032611,GO:0051091,GO:0045765,GO:0019932,GO:0006458,GO:0010243,GO:00069	K04718|1|0.0|689|mmu:20698|sphingosine kinase [EC:2.7.1.91]	gi|148702636|gb|EDL34583.1|	0	sphingosine kinase 1, isoform CRA_c [Mus musculus]

mmu-miR-107-5p	NM_001177667	-30.3	0.515271	-45.56	335	GO:0032991,GO:0005913,GO:0043227,GO:0031674,GO:0030864,GO:0005198,GO:0000149,GO:0046983,GO:0008092,GO:0046872,GO:0030835,GO:0006915,GO:00069	K06114|1|0.0|4729|rno:64159|spectrin alpha	gi|295054266|ref|NP_001171138.1|	0	spectrin alpha chain, brain isoform 2 [Mus musculus]
mmu-miR-107-5p	NM_001177668	-30.3	0.512653	-45.56	335	GO:0032991,GO:0030864,GO:0005913,GO:0031674,GO:0043227,GO:0008092,GO:0046872,GO:0000149,GO:0046983,GO:00308	K06114|1|0.0|4716|mmu:20740|spectrin alpha	gi|295054271|ref|NP_001171139.1|	0	spectrin alpha chain, brain isoform 3 [Mus musculus]
mmu-miR-107-5p	NM_001242419	-25.3	0.972072	-43.76	319	GO:0031224,	-	gi|37359864|dbj|BAC97910.1|	2.58E-172	mKIAA0247 protein [Mus musculus]

mmu-miR-107-5p	NM_001253860	-26.1	0.98101	-43.43	314	GO:0042383,GO:0031224,GO:0044291,GO:0005272,GO:0006814,	K04838|1|0.0|3639|mmu:20271|voltage-gated sodium channel type V alpha	gi|359718971|ref|NP_001240789.1|	0	sodium channel protein type 5 subunit alpha isoform 2 [Mus musculus]
mmu-miR-107-5p	NM_001271538	-25.3	0.988286	-41.23	327	-	-	-	-	-
mmu-miR-107-5p	NM_001271540	-25.3	0.987879	-41.23	327	-	-	-	-	-
mmu-miR-107-5p	NM_008909	-25.8	0.970211	-27.78	181	GO:0005911,GO:0019898,GO:0043232,GO:0043231,GO:0005488,GO:0005198,GO:00302	K10386|1|0.0|2920|mmu:19041|periplakin	gi|112421039|ref|NP_032935.2|	0	periplakin [Mus musculus]
mmu-miR-107-5p	NM_009137	-27	0.450557	-25.18	168	GO:0044421,GO:0005125,GO:0006952,GO:0002376,GO:0042221,	K05514|1|2e-33|144|mmu:20299|C-C motif chemokin	gi|5911365|gb|AAD55763.1|AF163476_1	1.24E-32	macrophage-derived chemokine [Mus musculus]

mmu-miR-107-5p	NM_009886	-26.6	0.984224	-29.68	152	GO:0016021,GO:0046872,GO:0005515,GO:0004888,GO:0014020,GO:0042471,GO:0016337,GO:0007186,GO:0008105,GO:0007610,GO:0001736,	K04600|1|0.0|5481|mmu:12614|cadherin EGF LAG seven-pass G-type receptor 1 (flamingo)	gi|115648153|ref|NP_034016.2|	0	cadherin EGF LAG seven-pass G-type receptor 1 precursor [Mus musculus]
mmu-miR-107-5p	NM_010586	-25	0.999695	-48.12	345	GO:0043005,GO:0030133,GO:0005789,GO:0031224,GO:0005262,GO:0005217,GO:0046873,GO:0005543,GO:0004872,GO:0015980,GO:0007599,GO:0006816,GO:0010863,GO:0007169,GO:0030073,GO:0006950,GO:00	K04959|1|0.0|5027|mmu:16439|inositol 1,4,5-triphosphate receptor type 2	gi|60592758|ref|NP_034716.1|	0	inositol 1,4,5-trisphosphate receptor type 2 isoform 2 [Mus musculus]

mmu-miR-107-5p	NM_011043	-25.9	0.923589	-25.44	160	GO:0016021,GO:0046872,GO:0016337,	K04602|1|1e-63|246|dre:100008568|cadherin EGF LAG seven-pass G-type receptor 3 (flamingo)	gi|28972738|dbj|BAC65785.1|	0	mKIAA1400 protein [Mus musculus]
mmu-miR-107-5p	NM_011323	-26.2	0.992685	-70.94	482	GO:0044297,GO:0031410,GO:0033267,GO:0005887,GO:0031420,GO:0005272,GO:0032559,GO:0048513,GO:0006935,GO:0034220,GO:0008366,GO:0006814,GO:00083	K04840|1|0.0|3398|mmu:20273|voltage-gated sodium channel type VIII alpha	gi|172072624|ref|NP_035453.2|	0	sodium channel protein type 8 subunit alpha isoform 2 [Mus musculus]

mmu-miR-107-5p	NM_012046	-29.6	0.157304	-25.29	163	GO:0043231,GO:0043232,GO:0003676,GO:0003824,GO:0032559,GO:0022602,GO:0006310,GO:0033365,GO:0048232,GO:0007281,GO:0007128,	K10878|1|0.0|685|mmu:26972|meiotic recombination protein SPO11	gi|6755624|ref|NP_036176.1|	0	meiotic recombination protein SPO11 isoform a [Mus musculus]
mmu-miR-107-5p	NM_013716	-32.2	0.090457	-26.22	171	GO:0005737,GO:0016020,GO:0003724,GO:0003676,GO:0004518,GO:0003678,GO:0000166,GO:0007264,	K12741|1|3e-08|61.6|dan:Dana_GF18582|heterogeneous nuclear ribonucleoprotein A1/A3!K01509|2|9e-08|60.1|dsi:Dsim_GD21374|adenosinetriphosphatase [EC:3.6.1.3]	gi|60359872|dbj|BAD90155.1|	0	mKIAA4115 protein [Mus musculus]

mmu-miR-107-5p	NM_015790	-32.4	0.085597	-27.37	166	GO:0016021,GO:0009986,GO:0005515,GO:0042102,GO:0023052,GO:0002204,GO:00421	K06710|1|3e-164|580|mmu:50723|inducible T-cell co-stimulator ligand	gi|7657220|ref|NP_056605.1|	1.90E-163	ICOS ligand precursor [Mus musculus]
mmu-miR-107-5p	NM_019570	-25.1	0.963982	-27.14	158	GO:0031981,GO:0046872,GO:0003677,GO:0034061,GO:0019985,GO:0009416,	K03515|1|0.0|2335|mmu:56210|DNA repair protein REV1 [EC:2.7.7.-]	gi|40254551|ref|NP_062516.2|	0	DNA repair protein REV1 [Mus musculus]
mmu-miR-107-5p	NM_019793	-30.1	0.130125	-27.19	164	GO:0031224,	K06537|1|4e-29|130|acs:100551648|CD151 antigen!K06497|3|4e-27|123|nve:NEMVE_v1g236446|CD63 antigen	gi|254281189|ref|NP_062767.3|	8.01E-130	tetraspanin-3 [Mus musculus]

mmu-miR-107-5p	NM_019923	-25	0.999711	-48.12	345	GO:0043005,GO:0030133,GO:0005789,GO:0031224,GO:0005262,GO:0005217,GO:0046873,GO:0005543,GO:0004872,GO:0015980,GO:0007599,GO:0006816,GO:0010863,GO:0007169,GO:0030073,GO:0006950,GO:00	K04959|1|0.0|5024|rno:81678|inositol 1,4,5-triphosphate receptor type 2	gi|60593032|ref|NP_064307.2|	0	inositol 1,4,5-trisphosphate receptor type 2 isoform 1 [Mus musculus]

mmu-miR-107-5p	NM_023214	-28	0.86622	-42.6	318	GO:0031981,GO:0016020,GO:0031410,GO:0012505,GO:0046914,GO:0001071,GO:0003677,GO:0046915,GO:0050856,GO:0006357,GO:0001816,GO:0000041,	K09428|1|6e-163|577|mmu:13709|E74-like factor 1/2/4!K14694|2|2e-157|559|cfa:479924|solute carrier family 30 (zinc transporter), member 7	gi|6679627|ref|NP_031946.1|	4.38E-162	ETS-related transcription factor Elf-1 [Mus musculus]
mmu-miR-107-5p	NM_023538	-25.8	0.724003	-26.9	170	GO:0005740,GO:0001727,GO:0032559,GO:0007186,GO:0016310,GO:0006672,	K09881|1|0.0|811|mmu:69923|acylglycerol kinase [EC:2.7.1.94]	gi|37537518|ref|NP_076027.1|	0	acylglycerol kinase, mitochondrial precursor [Mus musculus]
mmu-miR-107-5p	NM_026434	-28.6	0.353009	-25.64	164	GO:0003676,	K12842|1|2e-06|55.5|nvi:100118482|U2-associated protein SR140	gi|13385930|ref|NP_080710.1|	2.18E-105	probable RNA-binding protein 18 isoform 1 [Mus musculus]

mmu-miR-107-5p	NM_027141	-27.7	0.318125	-26.42	168	-	K10345|1|0.0|651|rno:302981|SPRY domain-containing SOCS box protein 3	gi|254939629|ref|NP_081417.2|		0	SPRY domain-containing SOCS box protein 3 isoform 3 [Mus musculus]
mmu-miR-107-5p	NM_027801	-34.2	0.05737	-28.9	178	-	K11423|1|1e-08|63.5|dgr:Dgri_GH16034|histone-lysine N-methyltransferase SETD2 [EC:2.1.1.43]!K11515|2|2e-07|59.3|oaa:100090319|inner centromere protein!K10352|3|3e-06|55.5|dwi:Dwil_GK20720|myosin heavy chain	gi|298358637|ref|NP_082077.1|		0	uncharacterized protein LOC212153 [Mus musculus]

mmu-miR-107-5p	NM_028021	-25.3	0.987405	-41.23	327	GO:0043005,GO:0032432,GO:0030427,GO:0042623,GO:0003774,GO:0003779,GO:0032559,GO:0006935,GO:0030029,	K10352|1|0.0|2806|mmu:71960|myosin heavy chain	gi|148690794|gb|EDL22741.1|	0	myosin, heavy polypeptide 14, isoform CRA_b [Mus musculus]
mmu-miR-107-5p	NM_028643	-28.3	0.356702	-46.1	324	GO:0043231,GO:0046872,	K15105|1|3e-09|64.7|nvi:100122016|solute carrier family 25 (mitochondrial aspartate/glutamate transporter), member 12/13	gi|27477043|ref|NP_082919.1|	0	EF-hand domain-containing family member A1 [Mus musculus]

mmu-miR-107-5p	NM_053087	-36.5	0.007217	-30.23	176	GO:0016021,GO:0004712,GO:0070851,GO:0007088,GO:0008284,GO:0043405,GO:0007176,GO:0048646,GO:0000165,	K08774|1|5e-09|63.5|gga:414743|transforming growth factor, alpha!K09784|2|1e-08|62.0|mcc:702502|epireguli	gi|16716373|ref|NP_444317.1|	7.60E-85	epigen precursor [Mus musculus]
mmu-miR-107-5p	NM_130863	-31	0.187952	-25.62	169	GO:0030425,GO:0044459,GO:0044444,GO:0004871,GO:0031753,GO:0031690,GO:0004674,GO:0032559,GO:0001541,GO:0010033,GO:0015031,GO:0002029,GO:0000302,GO:0010863,GO:0007169,GO:0006468,GO:0048522,GO:0003057,GO:00069	K00910|1|0.0|1313|pon:100461893|beta-adrenergic-receptor kinase [EC:2.7.11.15]	gi|13096806|gb|AAH03196.1|	0	Adrbk1 protein, partial [Mus musculus]

mmu-miR-107-5p	NM_133775	-26	0.741003	-27.99	156	GO:0043231,GO:0044421,GO:0005102,GO:0032722,GO:0050709,GO:0006357,GO:0001818,GO:0002696,GO:0050714,GO:0002823,GO:0031349,GO:0002828,	K12967|1|2e-151|536|mmu:77125|interleukin 33	gi|257900486|ref|NP_598536.2|	1.68E-150	interleukin-33 precursor [Mus musculus]
mmu-miR-107-5p	NM_134448	-27.6	0.994271	-64.77	478	GO:0043231,GO:0009925,GO:0005874,GO:0031410,GO:0005578,GO:0008017,GO:0046872,GO:0005102,GO:0022402,GO:0048667,GO:0031109,GO:0008088,GO:00071	K10382|1|0.0|8017|cfa:474948|dystonin	gi|111154076|ref|NP_604443.2|	0	dystonin isoform b [Mus musculus]

mmu-miR-107-5p	NM_139153	-25.9	0.83375	-42.99	319	GO:0044424,GO:0017111,GO:0005083,GO:0008047,GO:0046914,GO:0032561,GO:0043087,GO:0032012,	K12491|1|0.0|1548|mmu:213990|Arf-GAP, GTPase, ANK repeat and PH domain-containing protein 1/2/3	gi|124301219|ref|NP_631892.2|	0	arf-GAP with GTPase, ANK repeat and PH domain-containing protein 3 isoform a [Mus musculus]
mmu-miR-107-5p	NM_144847	-27.8	0.457818	-25.68	174	GO:0044424,GO:0016301,GO:0032559,GO:0030154,GO:0048731,GO:0006464,GO:00430	K08875|1|8e-173|608|rno:680451|nuclear receptor-binding protein	gi|298286836|sp|Q91V36.2|NRBP2_MOUSE	7.25E-178	RecName: Full=Nuclear receptor-binding protein 2
mmu-miR-107-5p	NM_146091	-28.5	0.696174	-25.11	173	-	-	gi|148701364|gb|EDL33311.1|	0	RIKEN cDNA 5730596K20 [Mus musculus]
mmu-miR-107-5p	NM_146129	-32.9	0.069513	-26.23	176	GO:0043231,	-	gi|344241581|gb|EGV97684.1|	0	Phosphorylated CTD-interacting factor 1 [Cricetulus griseus]
mmu-miR-107-5p	NM_153395	-25.6	0.995063	-25.36	170	GO:0044424,GO:0005083,GO:0006810,	-	gi|148692491|gb|EDL24438.1|	0	MON2 homolog (yeast), isoform CRA_d [Mus musculus]

mmu-miR-107-5p	NM_172449	-26.2	0.971235	-41.77	322	GO:0043231,GO:0004871,GO:0005102,GO:0023052,	K06102|1|3e-13|79.7|mgp:100544052|cingulin!K10352|5|3e-10|70.1|smm:Smp_085540.6|myosin heavy chain	gi|157909774|ref|NP_766037.2|	0	peripheral-type benzodiazepine receptor-associated protein 1 [Mus musculus]
mmu-miR-107-5p	NM_172683	-25.4	0.992499	-30.01	167	-	K10500|1|4e-15|86.3|rno:313666|zinc finger and BTB domain-containing protein	gi|260099684|ref|NP_766271.2|	0	pogo transposable element with ZNF domain isoform 1 [Mus musculus]
mmu-miR-107-5p	NM_172844	-25.4	0.881075	-40.6	326	GO:0005789,GO:0031224,GO:0016709,GO:0050662,GO:0000166,GO:0008152,	K00485|1|0.0|693|acs:100559458|dimethylaniline monooxygenase (N-oxide forming) [EC:1.14.13.8]	gi|27370272|ref|NP_766432.1|	0	flavin containing monooxygenase 9 [Mus musculus]

mmu-miR-107-5p	NM_172964	-26.5	0.89652	-42.02	317	GO:0044464,GO:0008047,GO:0043087,	K08878|1|5e-17|92.0|pon:100431481|breakpoint cluster region protein [EC:2.7.11.1]	gi|226342973|ref|NP_766552.3|	0	rho GTPase-activating protein 28 [Mus musculus]
mmu-miR-107-5p	NM_175244	-30	0.366446	-49.15	342	GO:0044444,GO:0000152,GO:0000149,GO:0019787,GO:0006511,GO:0007088,GO:0016567,	K12233|1|0.0|1666|mmu:76608|E3 ubiquitin-protein ligase HECTD3 [EC:6.3.2.19]	gi|148698628|gb|EDL30575.1|	0	HECT domain containing 3, isoform CRA_b [Mus musculus]
mmu-miR-107-5p	NM_178027	-31.2	0.350148	-47.41	321	GO:0012505,GO:0044444,GO:0046907,	-	gi|29825827|ref|NP_821170.1|	0	vacuolar protein sorting-associated protein 26B [Mus musculus]
mmu-miR-107-5p	NM_178682	-25.3	0.970936	-43.76	319	GO:0031224,	-	gi|30520071|ref|NP_848797.1|	9.37E-159	uncharacterized protein LOC217684 isoform 2 precursor [Mus musculus]
mmu-miR-107-5p	NM_181544	-27.3	0.85904	-42.82	319	GO:0031224,GO:0046872,GO:0015075,GO:0030246,GO:0006810,GO:0007186,	K04989|1|0.0|3348|mmu:244646|polycystin 1L3	gi|115583672|ref|NP_853522.2|	0	polycystic kidney disease protein 1-like 3 isoform b precursor [Mus musculus]

mmu-miR-107-5p	NM_181815	-27	0.813952	-44.58	310	-	K10352|1|3e-22|109|ecb:100062865|myosin heavy chain	gi|160333189|ref|NP_861536.3|	0	centrosomal protein of 128 kDa [Mus musculus]
mmu-miR-107-5p	NR_002928	-28.7	0.138609	-25.12	172	-	-	gi|38174374|gb|AAH61236.1|	8.19E-44	WD repeat domain 70 [Mus musculus]
mmu-miR-107-5p	NR_003633	-30.4	0.624706	-25.93	175	-	-	gi|148686746|gb|EDL18693.1|	9.05E-104	GTL2, imprinted maternally expressed untranslated mRNA [Mus musculus]
mmu-miR-107-5p	NR_027515	-28.6	0.345915	-25.64	164	GO:0003676,	-	gi|13385930|ref|NP_080710.1|	1.36E-89	probable RNA-binding protein 18 isoform 1 [Mus musculus]
mmu-miR-107-5p	NR_027651	-30.4	0.624342	-25.93	175	-	-	gi|148686746|gb|EDL18693.1|	5.00E-78	GTL2, imprinted maternally expressed untranslated mRNA [Mus musculus]
mmu-miR-107-5p	NR_028359	-28.3	0.855175	-42.47	320	GO:0031981,GO:0006351,	-	gi|28972794|dbj|BAC65813.1|	0	mKIAA1606 protein [Mus musculus]
mmu-miR-107-5p	NR_033554	-29.1	0.163678	-25.55	173	-	-	gi|148690367|gb|EDL22314.1|	1.58E-35	mCG1051026 [Mus musculus]

mmu-miR-107-5p	NR_040453	-25.4	0.948885	-42.73	304	-	K12797|1|2e-08|62.8|mdo:100018349|caspase recruitment domain-containing protein 6	gi|115270958|ref|NP_083276.2|	0	interferon-induced very large GTPase 1 [Mus musculus]
mmu-miR-107-5p	NR_040456	-25.4	0.949176	-42.73	304	-	K12797|1|2e-08|62.8|mdo:100018349|caspase recruitment domain-containing protein 6	gi|115270958|ref|NP_083276.2|	0	interferon-induced very large GTPase 1 [Mus musculus]
mmu-miR-107-5p	NR_045059	-27.4	0.477467	-41.72	328	-	-	gi|148688737|gb|EDL20684.1|	9.96E-22	mCG140306, isoform CRA_b [Mus musculus]
mmu-miR-107-5p	NR_045281	-25.7	0.81619	-25.75	174	-	-	gi|148672456|gb|EDL04403.1|	3.20E-25	mCG1027739 [Mus musculus]
mmu-miR-107-5p	NR_073205	-27.2	0.623185	-25.88	178	-	-	-	-	-

mmu-miR-107-5p	NM_178935	-22.1	0.999977	-61.55	485	-	K06106|1|2e-20|102|smm:Smp_164620|cortactin!K10352|3|6e-08|61.2|aga:AgaP_AGAP007523|myosin heavy chain	gi|30725808|ref|NP_849266.1|	0	gamma-taxilin [Mus musculus]
mmu-miR-107-5p	NM_201394	-24.9	0.99996	-67.86	484	-	K10388|1|0.0|3881|mmu:18810|plectin	gi|256418964|ref|NP_958788.2|	0	plectin isoform 1e [Mus musculus]
mmu-miR-107-5p	NM_001077499	-20.5	1	-70.94	482	GO:0044297,GO:0031410,GO:0033267,GO:0005887,GO:0031420,GO:0005272,GO:0032559,GO:0048513,GO:0006935,GO:0034220,GO:0008366,GO:0006814,GO:00083	K04840|1|0.0|3401|mmu:20273|voltage-gated sodium channel type VIII alpha	gi|117414174|ref|NP_001070967.1|	0	sodium channel protein type 8 subunit alpha isoform 1 [Mus musculus]

mmu-miR-107-5p	NM_013587	-20.4	1	-48.31	341	GO:0005788,GO:0016020,GO:0070325,GO:0004871,GO:0019899,GO:0005539,GO:0043393,GO:0010915,GO:0010469,	-	gi|63999380|ref|NP_038615.2|	0	alpha-2-macroglobulin receptor-associated protein precursor [Mus musculus]
mmu-miR-107-5p	NM_172903	-22	1	-42.34	339	GO:0012505,GO:0043231,GO:0031224,GO:0046914,GO:0015924,GO:0006487,GO:00193	K01231|1|0.0|2275|mmu:140481|alpha-mannosidase II [EC:3.2.1.114]	gi|326937500|ref|NP_766491.2|	0	alpha-mannosidase 2x [Mus musculus]
mmu-miR-107-5p	NM_007454	-21	1	-42.98	334	GO:0005764,GO:0030136,GO:0030119,GO:0012506,GO:0044431,GO:0005488,GO:0022892,GO:0006901,GO:0048193,GO:0050688,GO:0015031,	K12392|1|0.0|1671|mmu:11764|AP-1 complex subunit beta-1	gi|88853578|ref|NP_031480.2|	0	AP-1 complex subunit beta-1 isoform 2 [Mus musculus]
mmu-miR-107-5p	NM_029413	-20.2	1	-47.79	333	GO:0046914,GO:0032559,	-	gi|148691972|gb|EDL23919.1|	0	microrchidia 4, isoform CRA_b [Mus musculus]

mmu-miR-107-5p	NM_001081286	-20.3	1	-42.63	330	GO:0030054,GO:0031252,GO:0016021,GO:0042995,GO:0046872,GO:0016337,GO:0032502,GO:0007154,GO:0030036,	K06813|1|0.0|882|gga:423718|cadherin 23	gi|157951641|ref|NP_001074755.2|	0	FAT tumor suppressor homolog 1 precursor [Mus musculus]
mmu-miR-107-5p	NM_172437	-20.5	1	-42.91	328	GO:0003676,GO:0016866,GO:0009451,GO:0006399,	K06176|1|0.0|1377|mmu:78895|tRNA pseudouridine13 synthase [EC:5.4.99.27]	gi|27369583|ref|NP_766025.1|	0	pseudouridylate synthase 7 homolog-like protein [Mus musculus]
mmu-miR-107-5p	NM_009282	-24.8	0.993816	-42.6	325	GO:0044427,GO:0044459,GO:0031981,GO:0005488,GO:0007067,	K06671|1|0.0|2302|mmu:20842|cohesin complex subunit SA-1/2	gi|68533101|dbj|BAE06105.1|	0	STAG1 variant protein [Homo sapiens]

mmu-miR-107-5p	NM_028864	-21.2	0.999999	-42.59	324	GO:0043231,GO:0003676,GO:0016763,GO:0046914,GO:0019899,GO:0043462,GO:0001819,GO:0007249,GO:0043330,GO:00506	K15259|1|0.0|1534|mmu:78781|poly [ADP-ribose] polymerase 7/11/12/13 [EC:2.4.2.30]	gi|21746169|ref|NP_083140.1|	0	zinc finger CCCH-type antiviral protein 1 isoform 2 [Mus musculus]
mmu-miR-107-5p	NR_001461	-22.4	1	-48.46	322	-	K11849|1|6e-55|213|acs:100555596|ubiquitin carboxyl-terminal hydrolase 25/28 [EC:3.1.2.15]!K06478|4|4e-43|182|acs:100563672|protein tyrosine phosphatase, receptor type, C [EC:3.1.3.4	gi|209553980|gb|AAA39398.2|	0	ORF2 [Mus musculus domesticus]

mmu-miR-107-5p	NM_028901	-23.2	0.999996	-45.89	321	GO:0016459,GO:0030016,GO:0031981,GO:0008092,GO:0017111,GO:0032559,	K10362|1|0.0|4354|mmu:74376|myosin XVIII	gi|291327510|ref|NP_083177.2|	0	myosin XVIIIb [Mus musculus]
mmu-miR-107-5p	NM_198649	-23.6	0.998607	-42.57	320	GO:0044424,GO:0008092,GO:0046914,GO:0006996,GO:0006357,GO:0006935,	K07520|1|0.0|1335|mmu:319713|actin-binding LIM protein	gi|50510665|dbj|BAD32318.1|	0	mKIAA0843 protein [Mus musculus]
mmu-miR-107-5p	NM_172739	-20.9	1	-41.57	319	GO:0043231,GO:0005856,GO:0044444,GO:0004871,GO:0003712,GO:0003676,GO:0005099,GO:0006351,GO:0007166,GO:0014020,GO:0007420,GO:0042462,GO:0006935,GO:0043114,GO:0032319,	K05732|1|0.0|2774|mmu:232906|glucocorticoid receptor DNA-binding factor 1	gi|75677442|ref|NP_766327.3|	0	rho GTPase-activating protein 35 [Mus musculus]

mmu-miR-107-5p	NM_001081180	-23.6	0.998937	-45.26	318	GO:0043231,GO:0044444,GO:0031090,GO:0030414,GO:0008544,GO:0007155,GO:0010466,GO:0030162,	K06254|1|7e-11|71.2|tca:100141763|agrin	gi|124487159|ref|NP_001074649.1|	0	serine protease inhibitor Kazal-type 5 precursor [Mus musculus]
mmu-miR-107-5p	NM_027541	-21	0.999998	-41.82	316	GO:0016604,GO:0030529,GO:0005488,GO:0000377,GO:0050953,	K12843|1|0.0|1206|mmu:70767|U4/U6 small nuclear ribonucleoprotein PRP3	gi|12852223|dbj|BAB29324.1|	0	unnamed protein product [Mus musculus]
mmu-miR-107-5p	NM_133833	-24.9	0.999987	-44.3	315	GO:0043231,GO:0009925,GO:0005874,GO:0031410,GO:0005578,GO:0008017,GO:0046872,GO:0005102,GO:0022402,GO:0048667,GO:0031109,GO:0008088,GO:00071	K10382|1|0.0|1.000e+04|mmu:13518|dystonin	gi|111154082|ref|NP_598594.2|	0	dystonin isoform a [Mus musculus]

mmu-miR-107-5p	NM_028672	-20.3	0.999998	-41.55	314	-	-	gi|148675930|gb|EDL07877.1|	0	RIKEN cDNA 4930430E16, isoform CRA_a [Mus musculus]
mmu-miR-107-5p	NM_010683	-20.2	1	-43.68	308	GO:0043256,GO:0046625,GO:0005198,GO:0009888,GO:0030030,GO:0048870,GO:0007044,GO:0006935,GO:0008284,GO:0065003,GO:0030198,GO:0000904,	K05635|1|0.0|3070|mmu:226519|laminin, gamma 1	gi|148707495|gb|EDL39442.1|	0	laminin, gamma 1, isoform CRA_b [Mus musculus]
mmu-miR-107-5p	NM_011181	-20.7	0.999999	-41.65	307	GO:0044424,GO:0016020,GO:0005083,GO:0005488,GO:0016192,GO:0019222,GO:0031175,GO:0007010,GO:0032011,GO:00071	K12495|1|3e-41|171|bfo:BRAFLDRAFT_106693|IQ motif and SEC7 domain-containing protein	gi|332241233|ref|XP_003269786.1|	0	PREDICTED: cytohesin-2-like isoform 2 [Nomascus leucogenys]

mmu-miR-107-5p	NM_001199137	-21.1	1	-41.71	306	GO:0005856,GO:0044444,GO:0046872,GO:0003779,GO:0015631,GO:0022402,GO:0006612,GO:0007166,GO:0001704,	K10382|1|0.0|5254|mmu:13518|dystonin	gi|312433957|ref|NP_001186066.1|	0	microtubule-actin cross-linking factor 1 isoform 2 [Mus musculus]
mmu-miR-107-5p	NM_026472	-20	1	-40.17	305	GO:0000228,GO:0003676,GO:0016070,	K14838|1|2e-148|526|mmu:67949|nucleolar protein	gi|224809384|ref|NP_080748.3|	1.23E-147	MKI67 FHA domain-interacting nucleolar phosphoprotein [Mus musculus]

mmu-miR-107-5p	NM_177821	-21.9	1	-42.9	302	GO:0031981,GO:0043234,GO:0005815,GO:0035258,GO:0004468,GO:0046872,GO:0003712,GO:0008134,GO:0001071,GO:0003682,GO:0019901,GO:0000977,GO:0006357,GO:0001932,GO:0010557,GO:0051495,GO:0014706,GO:0007243,GO:0030162,GO:0033143,GO:0009653,GO:00319	K04498|1|0.0|2074|mmu:328572|E1A/CREB-binding protein [EC:2.3.1.48]	gi|94421034|ref|NP_808489.4|	0	histone acetyltransferase p300 [Mus musculus]
mmu-miR-107-5p	NM_021393	-21.2	0.999991	-46.74	301	GO:0031981,GO:0005488,GO:0006355,GO:0045935,GO:0006354,	K15180|1|0.0|1027|bta:512799|negative elongation factor B	gi|395741251|ref|XP_002820475.2|	0	PREDICTED: negative elongation factor B [Pongo abelii]

mmu-miR-107-5p	NM_001163945	-23.4	0.904848	-41.64	297	GO:0044444,GO:0030529,GO:0003676,GO:0005198,GO:0006412,GO:0019080,GO:00310	K02925|1|0.0|790|mmu:66211|large subunit ribosomal protein L3e	gi|255653009|ref|NP_001157417.1|	0	ribosomal protein L3-like isoform 1 [Mus musculus]
mmu-miR-10a-3p	NM_001110240	-28.2	0.81697	-27.62	178	GO:0031224,GO:0015081,GO:0015291,GO:0005515,GO:0005261,GO:0050953,GO:0007611,GO:0006875,GO:0048167,GO:00708	K13750|1|0.0|1358|mmu:76376|solute carrier family 24 (sodium/potassium/calcium exchanger), member	gi|159032066|ref|NP_001103710.1|	0	solute carrier family 24 (sodium/potassium/calcium exchanger), member 2 isoform 2 [Mus musculus]
mmu-miR-10a-3p	NM_172426	-28.2	0.819395	-27.62	178	GO:0031224,GO:0015081,GO:0015291,GO:0005515,GO:0005261,GO:0050953,GO:0007611,GO:0006875,GO:0048167,GO:00708	K13750|1|0.0|1191|rno:84550|solute carrier family 24 (sodium/potassium/calcium exchanger), member	gi|27369563|ref|NP_766014.1|	0	solute carrier family 24 (sodium/potassium/calcium exchanger), member 2 isoform 1 [Mus musculus]

mmu-miR-10a-3p	NM_009133	-27.2	0.204754	-24.23	179	GO:0042995,GO:0044444,GO:0008092,GO:0048731,GO:0030030,GO:0000226,GO:0033043,GO:0032319,GO:0016601,	K04381|1|1e-39|164|rno:29332|stathmin	gi|149033946|gb|EDL88729.1|	7.24E-80	stathmin-like 3, isoform CRA_b [Rattus norvegicus]
mmu-miR-10a-3p	NM_009549	-25.5	0.846714	-23	176	GO:0044459,GO:0043232,GO:0046914,	-	gi|157823829|ref|NP_033575.3|	0	zinc finger protein 185 isoform a [Mus musculus]
mmu-miR-10a-3p	NM_001039563	-25.2	0.25192	-21.17	174	GO:0006950,GO:0048232,	-	gi|49229829|gb|AAT57925.1|	7.02E-41	EP2C [Mus musculus]
mmu-miR-10a-3p	NR_029784	-24.3	0.026313	-24.04	171	-	-	-	-	-
mmu-miR-10a-3p	NR_045739	-28.7	0.259097	-23.72	170	GO:0044459,GO:0043005,GO:0044444,GO:0044430,GO:0044297,GO:0046914,	-	gi|148703605|gb|EDL35552.1|	3.52E-36	mCG146308 [Mus musculus]
mmu-miR-10a-3p	NM_144553	-24.2	0.938803	-21.64	167	GO:0043231,GO:0000922,GO:0016791,GO:0000278,GO:0006796,GO:0007154,	K15008|1|1e-17|93.2|tgu:100226961|discs, large-associated protein 1	gi|148688799|gb|EDL20746.1|	0	discs, large homolog 7 (Drosophila), isoform CRA_a [Mus musculus]

mmu-miR-10a-3p	NM_175456	-24.4	0.774511	-22.94	166	GO:0005856,GO:0030016,GO:0016020,GO:0008092,GO:0003712,GO:0043388,GO:0006357,GO:0006606,GO:0007266,	-	gi|30425194|ref|NP_780665.1|	0	actin-binding Rho-activating protein [Mus musculus]
mmu-miR-10a-3p	NM_148940	-27.5	0.308308	-23.09	165	GO:0070011,	K09615|1|2e-52|207|pon:100454004|tryptase gamma 1 [EC:3.4.21.-]!K01324|3|4e-50|200|pon:100172926|plasma kallikrein [EC:3.4.21.	gi|110625928|ref|NP_683742.2|	0	serine protease 44 precursor [Mus musculus]
mmu-miR-10a-3p	NR_029566	-22.3	0.036768	-21.28	163	-	-	-	-	-
mmu-miR-10a-3p	NM_145534	-23.3	0.998729	-23.06	162	-	K10478|1|0.0|966|aml:100474284|BTB/POZ domain-containing protein 3/6	gi|26006233|dbj|BAC41459.1|	0	mKIAA0952 protein [Mus musculus]

mmu-miR-10a-3p	NM_010889	-23.9	1	-23.39	161	GO:0031674,	K11498|1|2e-07|62.0|ptr:461413|centromeric protein E	gi|148222065|ref|NP_035019.1|	0	nebulin [Mus musculus]
mmu-miR-10a-3p	NM_001085509	-25.4	0.95082	-23.14	160	-	K12567|1|5e-73|278|dre:100001684|titin [EC:2.7.11.1]	gi|147906795|ref|NP_001078978.1|	0	myomesin-3 [Mus musculus]
mmu-miR-10a-3p	NM_009376	-21.7	0.999864	-20.46	158	-	K09667|1|1e-12|76.3|hmg:100201589|polypeptide N-acetylglucosaminyltransferase [EC:2.4.1.-]	gi|239915965|ref|NP_033402.2|	0	intraflagellar transport protein 88 homolog [Mus musculus]

mmu-miR-10a-3p	NM_177379	-21.3	1	-20.72	157	GO:0043231,GO:0030425,GO:0044459,GO:0044444,GO:0031090,GO:0012505,GO:0044430,GO:0008047,GO:0005543,GO:0007265,GO:0043087,GO:0009966,	K04450|1|1e-25|121|ptr:739083|activating transcription factor 2!K08878|2|1e-24|118|cin:100176232|breakpoint cluster region protein [EC:2.7.11.	gi|307344692|ref|NP_001182561.1|	0	rho GTPase-activating protein 32 isoform 1 [Mus musculus]
mmu-miR-10a-3p	NM_007938	-23.8	0.990318	-23.01	156	GO:0016021,GO:0004714,GO:0032559,GO:0006464,GO:0007167,	K05107|1|0.0|2100|mmu:13840|Eph receptor A6 [EC:2.7.10.1]	gi|145312274|ref|NP_031964.2|	0	ephrin type-A receptor 6 [Mus musculus]
mmu-miR-10a-3p	NR_027509	-22	0.999954	-22.09	155	GO:0000790,GO:0046914,GO:0018024,GO:0048869,GO:0034968,GO:0007126,GO:0006351,	K11419|1|0.0|732|mmu:64707|histone-lysine N-methyltransferase SUV39H [EC:2.1.1.43]	gi|31543790|ref|NP_073561.2|	0	histone-lysine N-methyltransferase SUV39H2 [Mus musculus]

mmu-miR-10a-3p	NM_174857	-20.5	1	-20.27	154	GO:0043231,GO:0005578,GO:0030247,GO:0018149,	K08606|1|3e-23|111|hmg:100199496|meprin A, beta [EC:3.4.24.	gi|33469047|ref|NP_777282.1|	0	MAM domain-containing protein 2 precursor [Mus musculus]
mmu-miR-10a-3p	NM_008943	-21.9	0.999654	-20.14	153	GO:0030427,GO:0005764,GO:0031982,GO:0005783,GO:0043234,GO:0019866,GO:0044459,GO:0016021,GO:0005815,GO:0045202,GO:0030425,GO:0031965,GO:0000775,GO:0031674,GO:0044297,GO:0070011,GO:0050839,GO:0005261,GO:0019904,GO:0007033,GO:0015711,GO:00484	K04505|1|0.0|754|mmu:19164|presenilin 1 [EC:3.4.23.-]	gi|6679493|ref|NP_032969.1|	0	presenilin-1 [Mus musculus]

mmu-miR-10a-3p	NM_133990	-22.1	0.999828	-21.92	152	GO:0005887,GO:0004896,GO:0030888,GO:0007166,GO:0002377,	K05076|1|0.0|859|mmu:16164|interleukin 13 receptor alpha-1	gi|40254373|ref|NP_598751.3|	0	interleukin-13 receptor subunit alpha-1 precursor [Mus musculus]
mmu-miR-10a-3p	NM_172440	NA	NA	-20.98	153	GO:0044424,GO:0031224,GO:0016192,	K08518|1|0.0|2258|rno:288080|syntaxin-binding protein 5	gi|167830458|ref|NP_766028.2|	0	syntaxin-binding protein 5-like isoform xb [Mus musculus]
mmu-miR-10a-3p	NM_033370	NA	NA	-20.35	151	GO:0030120,GO:0005798,GO:0005488,GO:0051704,GO:0048200,GO:0015031,	K12392|1|3e-15|85.1|smm:Smp_034870|AP-1 complex subunit beta-1!K11825|4|5e-14|81.3|bmy:Bm1_28810|AP-2 complex subunit beta-1!K12396|5|6e-14|80.9|gga:420079|AP-3 complex subunit delta-1	gi|15426055|ref|NP_203534.1|	0	coatomer subunit beta [Mus musculus]

mmu-miR-10a-3p	NM_017392	-20.7	1	NA	NA	GO:0031224,GO:0044424,GO:0046872,GO:0004888,GO:0016477,GO:0016337,GO:0060897,GO:0007186,GO:0006355,GO:0048812,GO:0048232,	K04601|1|0.0|5472|mmu:53883|cadherin EGF LAG seven-pass G-type receptor 2 (flamingo)	gi|114050895|ref|NP_059088.2|	0	cadherin EGF LAG seven-pass G-type receptor 2 isoform 1 precursor [Mus musculus]
mmu-miR-10a-3p	NM_021530	-22.1	1	NA	NA	GO:0031224,GO:0015301,GO:0015698,GO:0030001,	K13859|1|0.0|2114|mmu:59033|solute carrier family 4 (sodium bicarbonate cotransporter), member 8	gi|50510613|dbj|BAD32292.1|	0	mKIAA0739 protein [Mus musculus]
mmu-miR-10a-3p	NM_183138	-20.4	1	NA	NA	GO:0016701,GO:0043169,GO:0016706,GO:0008152,	K13097|1|0.0|651|tgu:100218736|methylcytosine dioxygenase [EC:1.14.11.-]	gi|148666664|gb|EDK99080.1|	0	mCG133587 [Mus musculus]

mmu-miR-10a-3p	NM_145221	-22.1	1	NA	NA	GO:0000118,GO:0005769,GO:0031090,GO:0012505,GO:0019901,GO:0006915,GO:0007169,GO:0051128,GO:0010827,	K08733|1|0.0|1288|mmu:72993|DCC-interacting protein 13 alpha	gi|21699066|ref|NP_660256.1|	0	DCC-interacting protein 13-alpha [Mus musculus]
mmu-miR-10a-3p	NM_175190	-20.3	0.990336	NA	NA	GO:0000790,GO:0005488,GO:0000725,	-	gi|123230365|emb|CAM15843.1|	1.37E-56	novel protein (2900010J23Rik) [Mus musculus]
mmu-miR-10a-3p	NM_015735	-26.3	0.78754	NA	NA	GO:0080008,GO:0031981,GO:0003677,GO:0007050,GO:0006289,GO:0006511,GO:0051704,GO:0016567,GO:0007166,	K10610|1|0.0|2225|mmu:13194|DNA damage-binding protein 1	gi|7657011|ref|NP_056550.1|	0	DNA damage-binding protein 1 [Mus musculus]

mmu-miR-10a-3p	NR_037690	-22.7	0.997833	NA	NA	GO:0004672,GO:0032559,GO:0006464,	K08798|1|9e-64|246|phu:Phum_PHUM509230|MAP/microtubule affinity-regulating kinase [EC:2.7.11.	gi|341942045|sp|Q8C0V7.2|SMKW_MOUSE	0	RecName: Full=Sperm motility kinase W
mmu-miR-10a-3p	NM_201390	-21.2	1	NA	NA	-	K10388|1|0.0|3881|mmu:18810|plectin	gi|256418964|ref|NP_958788.2|	0	plectin isoform 1e [Mus musculus]
mmu-miR-10a-3p	NM_011959	-21.1	0.999705	NA	NA	GO:0000808,GO:0031981,GO:0043565,GO:0005515,GO:0032559,GO:0007050,GO:0006259,GO:0051320,	K02607|1|0.0|882|mmu:26429|origin recognition complex subunit 5	gi|6754948|ref|NP_036089.1|	0	origin recognition complex subunit 5 [Mus musculus]
mmu-miR-10a-3p	NR_027651	-21.5	1	NA	NA	-	-	gi|148686746|gb|EDL18693.1|	5.00E-78	GTL2, imprinted maternally expressed untranslated mRNA [Mus musculus]
mmu-miR-10a-3p	NM_207209	-20.6	1	NA	NA	GO:0012507,GO:0005488,GO:0006901,GO:0048193,GO:0006487,	K14007|1|0.0|2049|mmu:99683|protein transport protein SEC24	gi|46402179|ref|NP_997092.1|	0	protein transport protein Sec24B [Mus musculus]

mmu-miR-10a-3p	NM_025814	-20.5	1	NA	NA	-	K13199|1|7e-146|520|hsa:26135|plasminogen activator inhibitor 1 RNA-binding protein	gi|395821934|ref|XP_003784284.1|	1.50E-151	PREDICTED: plasminogen activator inhibitor 1 RNA-binding protein isoform 3 [Otolemur garnettii]
mmu-miR-10a-3p	NM_001025086	-23	0.783187	NA	NA	GO:0003676,	K09330|1|8e-10|65.1|pon:100444086|paired mesoderm homeobox protein	gi|151358019|emb|CAM17358.2|	2.54E-152	novel protein similar to reproductive homeobox 7 Rhox7 [Mus musculus]
mmu-miR-10a-3p	NM_183022	-20.1	1	NA	NA	GO:0031224,GO:0005261,GO:0006814,	K04831|1|0.0|941|mmu:241118|amiloride-sensitive cation channel 4, pituitary	gi|344251702|gb|EGW07806.1|	0	Amiloride-sensitive cation channel 4 [Cricetulus griseus]

mmu-miR-10a-3p	NM_001081259	-23.4	0.995349	NA	NA	GO:0016021,GO:0004871,GO:0051184,GO:0040008,GO:0043009,GO:0051181,GO:0006875,GO:0030099,GO:0030326,	K08220|1|0.0|1021|mmu:226844|MFS transporter, FLVCR family, feline leukemia virus subgroup C receptor-related protein	gi|124486925|ref|NP_001074728.1|	0	feline leukemia virus subgroup C receptor-related protein 1 [Mus musculus]
mmu-miR-10a-3p	NM_172955	-21.3	0.999996	NA	NA	GO:0031012,GO:0071813,GO:0046872,GO:0005539,GO:0016477,GO:0007409,GO:0048513,GO:0006950,	K06793|1|0.0|724|mmu:13003|chondroitin sulfate proteoglycan 2 (versican)	gi|148668664|gb|EDL00983.1|	0	mCG116562, isoform CRA_c [Mus musculus]
mmu-miR-10a-3p	NM_001243011	-20.9	0.999802	NA	NA	-	K10485|1|3e-121|436|bta:513934|BTB/POZ domain-containing protein 13 (germ cell-less protein-like 1)	gi|339895754|ref|NP_001229940.1|	0	germ cell-less homolog 1 family member [Mus musculus]

mmu-miR-10a-3p	NM_001204915	-22.1	0.999995	NA	NA	-	K15601|1|1e-61|239|cfa:474695|lysine-specific demethylase 3 [EC:1.14.11.-]!K00939|2|4e-06|55.8|bmy:Bm1_13790|adenylate kinase [EC:2.7.4.3]	gi|326319958|ref|NP_001191844.1|	2.49E-144	receptor expression-enhancing protein 3 isoform 1 [Mus musculus]

mmu-miR-10a-3p	NM_009819	-20.2	1	NA	NA	GO:0070161,GO:0043005,GO:0005856,GO:0031252,GO:0005911,GO:0044444,GO:0050839,GO:0005198,GO:0050807,GO:0048667,GO:0009887,GO:0045597,GO:0032102,GO:0021932,GO:0048812,GO:00071	K05691|1|0.0|1724|rno:297357|catenin (cadherin-associated protein), alpha	gi|157817081|ref|NP_001100068.1|	0	catenin alpha-2 [Rattus norvegicus]
mmu-miR-10a-3p	NM_001113736	-20	0.999999	NA	NA	-	-	gi|166063976|ref|NP_001107207.1|	0	pramel family member [Mus musculus]
mmu-miR-10a-3p	NM_001080944	-23.7	0.997871	NA	NA	GO:0031224,GO:0042625,GO:0046872,GO:0005548,GO:0032559,GO:0009206,GO:0006869,	K01530|1|0.0|2255|mmu:241633|phospholipid-translocating ATPase [EC:3.6.3.1]	gi|157671921|ref|NP_001074413.2|	0	ATPase, class I, type 8B, member 4 [Mus musculus]
mmu-miR-10a-3p	NR_028516	-20.9	0.999787	NA	NA	-	-	gi|148691318|gb|EDL23265.1|	3.78E-59	mCG141134, isoform CRA_c [Mus musculus]

mmu-miR-10a-3p	NR_015515	-20.8	0.999639	NA	NA	-	-	gi|50510429|dbj|BAD32200.1|	2.13E-26	mKIAA0264 protein [Mus musculus]
mmu-miR-10a-3p	NM_001001492	-21.8	0.999831	NA	NA	-	K10421|1|3e-09|65.5|bta:534624|CAP-Gly domain-containing linker protein 1	gi|124517724|ref|NP_001001492.2|	0	Lebercilin-like protein [Mus musculus]
mmu-miR-10a-3p	NM_001037740	-21.1	1	NA	NA	-	-	gi|83627724|ref|NP_001032829.1|	0	protein FAM40B isoform b [Mus musculus]
mmu-miR-10a-3p	NM_023821	-22.1	1	NA	NA	-	K00907|1|1e-25|121|dan:Dana_GF13333|myosin-light-chain kinase [EC:2.7.11.18]!K14721|3|3e-23|114|phu:Phum_PHUM540970|DNA-directed RNA polymerase III subunit RPC5	gi|256773195|ref|NP_076310.2|	0	cardiomyopathy-associated protein 5 [Mus musculus]

mmu-miR-10a-3p	NM_146987	-20.3	0.996361	NA	NA	GO:0004888,GO:0023052,	K04257|1|5e-152|537|mmu:258989|olfactory	gi|22128963|ref|NP_667198.1|	3.46E-151	olfactory receptor 457 [Mus musculus]
mmu-miR-10a-3p	NM_133948	-20	1	NA	NA	GO:0031981,GO:0044444,GO:0003676,GO:0006310,GO:0019058,GO:0006351,	K00020|1|2e-08|62.4|aag:AaeL_AAEL006684|3-hydroxyisobutyrate dehydrogenase [EC:1.1.1.31]	gi|19527168|ref|NP_598709.1|	0	PC4 and SFRS1-interacting protein [Mus musculus]
mmu-miR-10a-3p	NM_011827	-20.6	0.880554	NA	NA	GO:0031224,GO:0005488,GO:0002682,	K07988|1|3e-35|147|mmu:23900|hematopoietic cell signal transducer	gi|93102419|ref|NP_035957.2|	2.04E-34	hematopoietic cell signal transducer precursor [Mus musculus]

mmu-miR-10a-3p	NM_001164597	-20.7	1	NA	NA	GO:0043231,GO:0046914,GO:0003676,	K15200|1|8e-33|144|bfo:BRAFLDRAFT_111367|general transcription factor 3C polypeptide 2!K00907|5|1e-13|80.9|bta:338037|myosin-light-chain kinase [EC:2.7.11.18]	gi|37360280|dbj|BAC98118.1|	0	mKIAA1196 protein [Mus musculus]
mmu-miR-10a-3p	NM_008609	-21.2	1	NA	NA	GO:0044421,GO:0016021,GO:0004175,GO:0046914,GO:0030234,GO:0019538,GO:0043627,GO:0043412,	K07995|1|0.0|1149|mmu:17388|matrix metalloproteinase-15 (membrane-inserted) [EC:3.4.24.-]	gi|6678900|ref|NP_032635.1|	0	matrix metalloproteinase-15 precursor [Mus musculus]

mmu-miR-10a-3p	NM_001190483	-20.5	1	NA	NA	GO:0016023,GO:0044431,GO:0044421,GO:0004175,GO:0005488,GO:0001501,GO:0035107,GO:0006518,GO:0001999,GO:0048513,GO:0001816,GO:0007154,GO:0007169,GO:0003002,GO:0019058,GO:0016486,GO:0048565,GO:0035295,	K08654|1|0.0|3801|rno:116548|proprotein convertase subtilisin/kexin type 5 [EC:3.4.21.-]	gi|299523019|ref|NP_001177412.1|	0	proprotein convertase subtilisin/kexin type 5 isoform 1 preproprotein [Mus musculus]
mmu-miR-10a-3p	NM_001033303	-21.1	0.999891	NA	NA	GO:0044444,GO:0043169,GO:0017022,GO:0016814,GO:0009112,GO:0009127,GO:00422	K01490|1|0.0|1473|mmu:229665|AMP deaminase [EC:3.5.4.6]	gi|114431240|ref|NP_001028475.2|	0	AMP deaminase 1 [Mus musculus]

mmu-miR-10a-3p	NM_009481	-23	1	NA	NA	GO:0044424,GO:0070011,GO:0016790,GO:0046332,GO:0007276,GO:0019941,GO:0000087,GO:0007178,GO:0070646,	K11840|1|0.0|4960|mmu:22284|ubiquitin carboxyl-terminal hydrolase 9/24 [EC:3.1.2.15]	gi|115511018|ref|NP_033507.2|	0	probable ubiquitin carboxyl-terminal hydrolase FAF-X [Mus musculus]

mmu-miR-10a-3p	NM_008234	-22.1	0.999338	NA	NA	GO:0043231,GO:0000775,GO:0017111,GO:0003676,GO:0032559,GO:0031055,GO:0006305,GO:0006342,GO:0002521,GO:0000087,GO:0043066,GO:0032943,	K14819|1|7e-135|482|nvi:100121251|dual specificity phosphatase 12 [EC:3.1.3.16 3.1.3.48]!K11654|2|1e-102|375|dvi:Dvir_GJ21345|SWI/SNF-related matrix-associated actin-dependent regulator of chromatin subfamily A member	gi|12232371|ref|NP_032260.2|	0	lymphocyte-specific helicase [Mus musculus]
mmu-miR-10a-3p	NM_001102613	-21.6	0.999166	NA	NA	-	-	gi|156616318|ref|NP_001096083.1|	0	pleckstrin homology-like domain family B member 3 [Mus musculus]

mmu-miR-10a-3p	NM_172717	-21.7	0.999885	NA	NA	GO:0016604,GO:0046914,GO:0019787,GO:0000075,GO:0019941,GO:0000087,GO:0016567,	K10644|1|0.0|1262|mmu:231600|E3 ubiquitin-protein ligase CHFR [EC:6.3.2.19]	gi|148688094|gb|EDL20041.1|	0	checkpoint with forkhead and ring finger domains, isoform CRA_d [Mus musculus]
mmu-miR-10a-3p	NM_153794	-22.1	1	NA	NA	GO:0031224,GO:0043231,	-	gi|24418876|ref|NP_722489.1|	6.38E-143	protein FAM210A [Mus musculus]
mmu-miR-10a-3p	NM_145354	-20.2	1	NA	NA	GO:0031981,GO:0016427,GO:0003723,GO:0006399,GO:0001510,	K15335|1|0.0|1535|mmu:28114|tRNA (cytosine34-C5)-methyltransferase [EC:2.1.1.203]	gi|148705068|gb|EDL37015.1|	0	NOL1/NOP2/Sun domain family 2, isoform CRA_a [Mus musculus]

mmu-miR-10a-3p	NM_010303	-20.4	1	NA	NA	GO:0031234,GO:0048770,GO:0005903,GO:0032561,GO:0017111,GO:0060089,GO:0031701,GO:0032403,GO:0050780,GO:0001525,GO:0007265,GO:0007599,GO:0043009,GO:0009207,GO:0051480,GO:0048869,GO:0007188,GO:0001775,GO:0010518,GO:00	K04639|1|0.0|756|mmu:14674|guanine nucleotide-binding protein subunit alpha-13	gi|148702406|gb|EDL34353.1|	0	guanine nucleotide binding protein, alpha 13, isoform CRA_a [Mus musculus]
mmu-miR-10a-3p	NM_173185	-20.8	1	NA	NA	GO:0044424,GO:0004672,GO:0032559,GO:0006464,GO:0007166,	K08958|1|0.0|898|mmu:214897|casein kinase 1, gamma [EC:2.7.11.1]	gi|148694162|gb|EDL26109.1|	0	casein kinase 1, gamma 1 [Mus musculus]

mmu-miR-10a-3p	NM_016684	-22.3	0.999908	NA	NA	GO:0005622,GO:0046872,GO:0003676,GO:0006351,	K09230|1|0.0|1009|mmu:22758|SCAN domain-containing zinc finger protein	gi|37359940|dbj|BAC97948.1|	0	mKIAA0426 protein [Mus musculus]
mmu-miR-10a-3p	NM_019810	-20.4	1	NA	NA	GO:0031224,GO:0030054,GO:0005903,GO:0005355,GO:0015291,GO:0015758,GO:0001822,GO:0030001,GO:0022600,	K14158|1|0.0|1134|mmu:20537|solute carrier family 5 (sodium/glucose cotransporter), member 1	gi|26329187|dbj|BAC28332.1|	0	unnamed protein product [Mus musculus]
mmu-miR-10a-3p	NM_028166	-23.9	0.966683	NA	NA	GO:0044464,	-	gi|148671042|gb|EDL02989.1|	6.66E-67	RIKEN cDNA 1600014C10, isoform CRA_a [Mus musculus]
mmu-miR-10a-3p	NM_001115075	-23.1	0.829941	NA	NA	GO:0042611,GO:0031224,GO:0002376,	K06751|1|3e-114|412|ptr:450151|major histocompatibility complex, class I	gi|169234920|ref|NP_001108547.1|	0	histocompatibility 2, M region locus 5 precursor [Mus musculus]

mmu-miR-10a-3p	NM_178259	-21.9	1	NA	NA	GO:0031224,GO:0017111,GO:0032559,GO:0051234,GO:0009207,	K05647|1|0.0|9723|mmu:268379|ATP binding cassette, subfamily A (ABC1), member 13	gi|116292744|ref|NP_839990.2|	0	ATP-binding cassette sub-family A member 13 [Mus musculus]
mmu-miR-10a-3p	NM_001177319	-20.1	1	NA	NA	GO:0044421,GO:0016020,GO:0004866,GO:0007599,GO:0010466,GO:0072376,	K03909|1|7e-140|499|mmu:21788|tissue factor pathway inhibitor	gi|148695336|gb|EDL27283.1|	2.43E-143	tissue factor pathway inhibitor, isoform CRA_c [Mus musculus]
mmu-miR-10a-3p	NM_011033	-21.1	0.999953	NA	NA	GO:0005681,GO:0044444,GO:0003729,GO:0005515,GO:0090079,GO:0006412,GO:0000377,GO:0031124,GO:0043488,GO:00002	K13126|1|0.0|1165|mmu:18459|polyadenylate-binding protein	gi|6754972|ref|NP_035163.1|	0	poly A binding protein, cytoplasmic 2 [Mus musculus]

mmu-miR-10a-3p	NM_029432	-24	0.992822	NA	NA	-	K01855|1|3e-31|139|smm:Smp_171830|tRNA pseudouridine38/39 synthase [EC:5.4.99.45]	gi|148696343|gb|EDL28290.1|	0	RIKEN cDNA 4930402H24 [Mus musculus]
mmu-miR-10a-3p	NM_008478	-21.3	1	NA	NA	GO:0031224,GO:0043231,GO:0009986,GO:0033267,GO:0048029,GO:0005102,GO:0019904,GO:0009967,GO:0048869,GO:0048731,GO:0016337,GO:0030030,GO:0006935,GO:0002376,GO:0007599,	K06550|1|0.0|2491|mmu:16728|L1 cell adhesion molecule	gi|112293279|ref|NP_032504.3|	0	neural cell adhesion molecule L1 precursor [Mus musculus]

mmu-miR-10a-3p	NM_013860	-21	1	NA	NA	GO:0043231,GO:0031332,GO:0046914,GO:0023052,GO:0032501,GO:0035194,GO:0022618,GO:0006351,	K05048|1|1e-76|289|ssc:100621687|solute carrier family 6 (neurotransmitter transporter, amino acid/orphan) member 15/16/17/18/20!K12792|2|8e-58|227|bta:615869|thyroid receptor-interacting protein 6	gi|224994267|ref|NP_038888.2|	0	LIM domain-containing protein 1 [Mus musculus]
mmu-miR-10a-3p	NM_198932	-22.1	1	NA	NA	GO:0043231,GO:0043234,GO:0003677,GO:0006357,GO:0031128,GO:0043584,	K09364|1|2e-140|503|mmu:18986|POU domain transcription factor, class 2	gi|114431258|ref|NP_945152.2|	1.16E-139	POU domain, class 2, transcription factor 1 isoform B [Mus musculus]
mmu-miR-10a-3p	NM_175564	-20.7	0.999997	NA	NA	GO:0016020,	-	gi|30425360|ref|NP_780773.1|	1.72E-143	transmembrane protein 169 [Mus musculus]

mmu-miR-10a-3p	NM_001082535	-20.6	0.999985	NA	NA	GO:0031224,GO:0004175,	K14212|1|0.0|1025|mmu:237958|signal peptide peptidase-like 2C [EC:3.4.23.-]	gi|126722669|ref|NP_001076004.1|	0	signal peptide peptidase-like 2C isoform b precursor [Mus musculus]
mmu-miR-10a-3p	NM_172468	-21	1	NA	NA	GO:0044424,GO:0005543,GO:0009987,GO:0006810,	K08545|1|2e-08|63.9|ssc:100155153|nuclear receptor subfamily 2 group E member 1	gi|33859789|ref|NP_766056.1|	0	sorting nexin-30 [Mus musculus]
mmu-miR-10a-3p	NM_153409	-24.4	0.99964	NA	NA	GO:0043231,GO:0003676,GO:0001071,GO:0006357,GO:0006915,	-	gi|148695060|gb|EDL27007.1|	0	RIKEN cDNA A330102K23 [Mus musculus]
mmu-miR-10a-3p	NM_173439	-21.5	0.999996	NA	NA	GO:0044459,GO:0044430,GO:0005488,GO:0048731,GO:0033554,GO:0016567,GO:00065	K10319|1|1e-146|521|mmu:268882|F-box protein 45	gi|344282457|ref|XP_003412990.1|	9.59E-146	PREDICTED: F-box/SPRY domain-containing protein 1-like [Loxodonta africana]

mmu-miR-10a-3p	NM_015801	-23.5	0.996121	NA	NA	GO:0031224,GO:0043231,GO:0031090,GO:0004091,GO:0048646,GO:0006650,	K14676|1|0.0|2524|mmu:50767|lysophospholipid hydrolase [EC:3.1.1.5]	gi|148689968|gb|EDL21915.1|	0	patatin-like phospholipase domain containing 6, isoform CRA_c [Mus musculus]
mmu-miR-10a-3p	NM_023464	-21.2	0.961712	NA	NA	GO:0015630,GO:0044444,GO:0043231,GO:0005515,GO:0022402,	-	gi|26382599|dbj|BAB22733.2|	5.41E-68	unnamed protein product [Mus musculus]

mmu-miR-10a-3p	NM_001080927	-20.3	1	NA	NA	GO:0031981,GO:0043234,GO:0005515,GO:0001071,GO:0003677,GO:0003824,GO:0001525,GO:0021536,GO:0007219,GO:0009952,GO:0006355,GO:0008283,GO:0001708,GO:0048732,GO:0009913,GO:0009855,GO:0006259,GO:0040034,GO:0000904,GO:0030098,GO:0006952,GO:00224	K06053|1|0.0|933|mmu:19664|recombining binding protein suppressor of hairless	gi|124286787|ref|NP_001074396.1|	0	recombining binding protein suppressor of hairless isoform 2 [Mus musculus]

mmu-miR-10a-3p	NM_181318	-22.6	0.996887	NA	NA	GO:0044464,GO:0005083,GO:0019222,GO:0009966,GO:0035556,	K07376|1|0.0|913|mcc:696308|protein kinase, cGMP-dependent [EC:2.7.11.12]!K06277|2|6e-21|103|bfo:BRAFLDRAFT_124752|Rap guanine nucleotide exchange factor (GEF) 1!K08018|3|6e-21|103|rno:310533|Rap guanine nucleotide	gi|31044455|ref|NP_851835.1|	0	ras-GEF domain-containing family member 1B isoform 2 [Mus musculus]	

mmu-miR-10a-3p	NM_010716	-20.4	1	NA	NA	GO:0031981,GO:0003676,GO:0003909,GO:0046914,GO:0032559,GO:0006266,GO:0006281,GO:0048232,GO:0002562,	K10776|1|0.0|1907|mmu:16882|DNA ligase 3 [EC:6.5.1.1]	gi|71061470|ref|NP_034846.2|	0	DNA ligase 3 [Mus musculus]
mmu-miR-10a-3p	NM_007740	-20	1	NA	NA	GO:0005593,GO:0005201,GO:0009987,GO:0009888,GO:0006935,GO:0048871,GO:00034	K08131|1|0.0|1243|mmu:12839|collagen, type IX, alpha	gi|226693380|ref|NP_031766.3|	0	collagen alpha-1(IX) chain precursor [Mus musculus]
mmu-miR-10a-3p	NM_001167995	-21.3	0.951186	NA	NA	GO:0043231,GO:0008168,GO:0006399,GO:0001510,GO:0010467,	K15331|1|2e-63|242|mmu:215201|tRNA (uracil-5-)-methyltransferase [EC:2.1.1.35]	gi|269784758|ref|NP_001161467.1|	4.97E-71	tRNA (uracil(54)-C(5))-methyltransferase homolog isoform b [Mus musculus]

mmu-miR-10a-3p	NM_008795	-20.8	0.999998	NA	NA	GO:0060089,GO:0004674,GO:0032559,GO:0006464,GO:0023052,	K15596|1|0.0|896|mmu:18557|cyclin-dependent kinase 18 [EC:2.7.11.22]	gi|6679233|ref|NP_032821.1|	0	cyclin-dependent kinase 18 [Mus musculus]
mmu-miR-10a-3p	NM_001252518	-20.4	0.999998	NA	NA	GO:0016604,GO:0034399,GO:0003676,GO:0005515,GO:0006396,GO:0006259,GO:0033554,GO:0006351,	K13214|1|0.0|663|mmu:53610|non-POU domain-containing octamer-binding protein	gi|74141990|dbj|BAE41058.1|	0	unnamed protein product [Mus musculus]
mmu-miR-10a-3p	NR_045961	-20.3	0.989838	NA	NA	-	-	gi|148704029|gb|EDL35976.1|	1.18E-20	mCG144936 [Mus musculus]
mmu-miR-10a-3p	NM_001171026	-20.7	1	NA	NA	GO:0000835,GO:0005515,GO:0043161,GO:0035437,GO:0033554,GO:0016567,	K10088|1|0.0|1054|mmu:216440|protein OS-9	gi|148692522|gb|EDL24469.1|	0	RIKEN cDNA 4632413K17, isoform CRA_b [Mus musculus]

mmu-miR-10a-3p	NM_178220	-22.9	0.999974	NA	NA	GO:0031410,GO:0005764,GO:0042995,GO:0031234,GO:0044427,GO:0012506,GO:0004468,GO:0008047,GO:0001664,GO:0043565,GO:0019899,GO:0001775,GO:0001541,GO:0002029,GO:0050954,GO:0016567,GO:0006198,GO:0007187,GO:0043433,GO:0007599,GO:0016573,GO:00481	K04439|1|0.0|665|mmu:109689|beta-arrestin	gi|30089688|ref|NP_835738.1|	0	beta-arrestin-1 isoform B [Mus musculus]
mmu-miR-10a-3p	NM_028389	-24.3	0.539978	NA	NA	-	-	gi|148685527|gb|EDL17474.1|	1.97E-109	mCG22386, isoform CRA_a [Mus musculus]
mmu-miR-10a-3p	NM_001159657	-20.1	1	NA	NA	-	-	gi|148676030|gb|EDL07977.1|	0	RIKEN cDNA 5430407P10, isoform CRA_a [Mus musculus]

mmu-miR-10a-3p	NM_023066	-20.4	1	NA	NA	GO:0031227,GO:0046872,GO:0016706,GO:0016701,GO:0005198,GO:0018126,GO:0003012,GO:0006874,GO:0035107,GO:00082	K00476|1|0.0|1231|mmu:65973|aspartate beta-hydroxylase [EC:1.14.11.16]	gi|125628659|ref|NP_075553.2|	0	aspartyl/asparaginyl beta-hydroxylase isoform 1 [Mus musculus]
mmu-miR-10a-3p	NR_046034	-20.6	0.99988	NA	NA	-	-	gi|148701551|gb|EDL33498.1|	2.19E-98	mCG1037741 [Mus musculus]
mmu-miR-10a-3p	NM_020272	-20.7	1	NA	NA	GO:0043234,GO:0044444,GO:0035004,GO:0032559,GO:0051648,GO:0046488,GO:0006915,GO:0001775,GO:0002526,GO:0006796,GO:0048017,GO:0007599,GO:0007166,GO:00	K00922|1|0.0|2222|mmu:30955|phosphatidylinositol-4,5-bisphosphate 3-kinase [EC:2.7.1.153]	gi|148704947|gb|EDL36894.1|	0	phosphoinositide-3-kinase, catalytic, gamma polypeptide, isoform CRA_b [Mus musculus]

mmu-miR-10a-3p	NM_001025093	-20	1	NA	NA	GO:0031981,GO:0046914,GO:0000982,GO:0003712,GO:0046983,GO:0043566,GO:0003007,GO:0007166,GO:0009888,GO:0032906,GO:0002224,GO:0030154,GO:00001	K04450|1|0.0|864|cfa:478806|activating transcription factor 2	gi|74004656|ref|XP_535970.2|	0	PREDICTED: cyclic AMP-dependent transcription factor ATF-2 isoform 1 [Canis lupus familiaris]
mmu-miR-10a-3p	NM_019826	-20	0.999999	NA	NA	GO:0005740,GO:0003995,GO:0050662,GO:0006551,GO:0051259,GO:0006635,GO:00090	K00253|1|0.0|811|mmu:56357|isovaleryl-CoA dehydrogenase [EC:1.3.8.4]	gi|9789985|ref|NP_062800.1|	0	isovaleryl-CoA dehydrogenase, mitochondrial precursor [Mus musculus]

mmu-miR-10a-3p	NM_015805	-20.9	1	NA	NA	GO:0031224,GO:0042625,GO:0004871,GO:0046872,GO:0005548,GO:0032559,GO:0009206,GO:0006811,GO:0023052,GO:0006869,GO:0009207,	K01530|1|0.0|2102|mmu:50771|phospholipid-translocating ATPase [EC:3.6.3.1]	gi|40807502|ref|NP_056620.2|	0	probable phospholipid-transporting ATPase IIB isoform 2 [Mus musculus]
mmu-miR-10a-5p	NM_001004062	-25	0.992365	-25.26	146	GO:0031981,GO:0016020,GO:0005488,GO:0003713,GO:0006357,GO:0051704,	K15309|1|0.0|1067|mmu:382056|CREB-regulated transcription coactivator	gi|37360010|dbj|BAC97983.1|	0	mKIAA0616 protein [Mus musculus]
mmu-miR-10a-5p	NM_001007567	-26.3	0.531907	-26.64	152	-	-	gi|148679408|gb|EDL11355.1|	4.19E-140	solute carrier family 7, member 6 opposite strand, isoform CRA_b [Mus musculus]
mmu-miR-10a-5p	NM_001008973	-29.6	0.278395	-26.52	170	GO:0031224,	-	gi|113374162|ref|NP_001008973.2|	0	transmembrane protein 232 [Mus musculus]

mmu-miR-10a-5p	NM_001048206	-26.9	0.933047	-25.25	154	GO:0043231,GO:0005887,GO:0004871,GO:0046914,GO:0019787,GO:0019900,GO:0002697,GO:0043433,GO:0019221,GO:0032479,GO:0006915,GO:0032446,GO:0002221,GO:00	K03174|1|0.0|1104|mmu:22031|TNF receptor-associated factor 3	gi|114842403|ref|NP_001041671.1|	0	TNF receptor-associated factor 3 isoform b [Mus musculus]
mmu-miR-10a-5p	NM_001080972	-30.9	0.042826	-30.32	177	GO:0016021,GO:0005549,GO:0004930,GO:0023052,GO:0010033,	K04614|1|3e-170|597|mmu:626299|vomeronasal1 receptor	gi|124430498|ref|NP_001074441.1|	1.97E-169	vomeronasal 1 receptor 194 [Mus musculus]
mmu-miR-10a-5p	NM_001081357	-27.9	0.677647	-25.07	167	GO:0004672,GO:0030695,GO:0032559,GO:0000165,GO:0006464,	K04406|1|0.0|1684|mmu:225028|mitogen-activated protein kinase kinase kinase kinase 3 [EC:2.7.11.1]	gi|124486875|ref|NP_001074826.1|	0	mitogen-activated protein kinase kinase kinase kinase 3 [Mus musculus]

mmu-miR-10a-5p	NM_001103177	-34	0.133967	-32.3	179	GO:0005856,GO:0008092,GO:0046914,GO:0006996,GO:0006357,GO:0050953,GO:0006935,GO:0009653,	K07520|1|0.0|1363|mmu:226251|actin-binding LIM protein	gi|157057174|ref|NP_001096647.1|	0	actin-binding LIM protein 1 isoform 2 [Mus musculus]
mmu-miR-10a-5p	NM_001103178	-34	0.126306	-32.3	179	GO:0005856,GO:0008092,GO:0046914,GO:0006996,GO:0006357,GO:0006935,	K07520|1|0.0|951|ptr:450753|actin-binding LIM protein	gi|157057145|ref|NP_848803.3|	0	actin-binding LIM protein 1 isoform 1 [Mus musculus]
mmu-miR-10a-5p	NM_001126324	-27.5	0.532797	-26.26	165	-	-	gi|187280849|ref|NP_001119796.1|	0	uncharacterized protein LOC279185 [Mus musculus]
mmu-miR-10a-5p	NM_001145820	-26.8	0.907672	-26.3	165	GO:0043231,GO:0043234,GO:0019866,GO:0052590,GO:0046872,GO:0016616,GO:0006629,GO:0006071,GO:0019674,GO:00060	K00111|1|0.0|1356|mmu:14571|glycerol-3-phosphate dehydrogenase [EC:1.1.5.3]	gi|224922803|ref|NP_034404.3|	0	glycerol-3-phosphate dehydrogenase, mitochondrial precursor [Mus musculus]

mmu-miR-10a-5p	NM_001159275	-32.3	0.040645	-27.92	172	GO:0031224,GO:0043231,GO:0019866,GO:0015179,GO:0019752,GO:0006839,GO:0019627,GO:0006865,	K15101|1|1e-139|497|mmu:83885|solute carrier family 25 (mitochondrial ornithine transporter) member 2/15	gi|226494439|ref|NP_001152747.1|	6.69E-139	mutant ornithine transporter 2 [Mus musculus]
mmu-miR-10a-5p	NM_001163464	-33	0.10939	-33.46	184	GO:0016791,GO:0043169,GO:0006796,	K06124|1|7e-153|542|rno:287644|phosphoethanolamine/phosphocholine phosphatase [EC:3.1.3.7	gi|157786864|ref|NP_001099303.1|	4.67E-152	phosphoethanolamine/phosphocholine phosphatase [Rattus norvegicus]
mmu-miR-10a-5p	NM_001168290	-30.4	0.322396	-28.46	161	GO:0043231,GO:0003676,GO:0006396,	K13166|1|0.0|2060|mmu:234373|splicing factor, arginine/serine-rich 14	gi|148696857|gb|EDL28804.1|	0	splicing factor, arginine/serine-rich 14, isoform CRA_b [Mus musculus]

mmu-miR-10a-5p	NM_001177995	-27.1	0.949201	-25.38	164	GO:0043231,GO:0043234,GO:0046914,GO:0005515,GO:0003712,GO:0003677,GO:0007423,GO:0002762,GO:0007179,GO:0006357,GO:0043467,GO:0045444,GO:0019827,GO:00	K04462|1|0.0|1054|cfa:488148|ecotropic virus integration site 1 protein	gi|295789156|ref|NP_001171466.1|	0	PR domain zinc finger protein 16 isoform 2 [Mus musculus]
mmu-miR-10a-5p	NM_001253890	-28.1	0.467937	-25.95	158	GO:0043234,GO:0044444,GO:0016208,GO:0019887,GO:0009653,GO:0015980,GO:0007599,GO:0033762,GO:0032147,GO:0010863,GO:0007610,GO:0007169,GO:0030073,GO:00	K04739|1|0.0|765|mmu:19085|cAMP-dependent protein kinase regulator	gi|254675178|ref|NP_032949.3|	0	cAMP-dependent protein kinase type I-beta regulatory subunit [Mus musculus]

mmu-miR-10a-5p	NM_001254951	-26.6	0.945248	-28.43	168	-	K09228|1|0.0|803|aml:100473713|KRAB domain-containing zinc finger	gi|363807379|ref|NP_001241880.1|	0	zinc finger protein 850 [Mus musculus]
mmu-miR-10a-5p	NM_007465	-26.3	0.808151	-26.96	140	GO:0032991,GO:0001739,GO:0005887,GO:0044444,GO:0046914,GO:0005515,GO:0019787,GO:0010033,GO:0048513,GO:0006511,GO:0051259,GO:0007249,GO:0016567,GO:0009719,GO:0006950,GO:00430	K16060|1|0.0|1189|mmu:11797|baculoviral IAP repeat-containing protein 2/3	gi|160333366|ref|NP_031491.2|	0	baculoviral IAP repeat-containing protein 2 [Mus musculus]
mmu-miR-10a-5p	NM_007737	-25.8	0.982306	-43.4	313	GO:0005583,GO:0005515,GO:0009887,GO:0008544,GO:0030198,	K06236|1|0.0|1592|mmu:12832|collagen, type I/II/III/V/XI, alpha	gi|86613790|ref|NP_031763.2|	0	collagen alpha-2(V) chain precursor [Mus musculus]

mmu-miR-10a-5p	NM_007739	-31.2	0.299104	-27.15	172	GO:0044420,GO:0005578,GO:0048592,GO:0010810,GO:0008283,	K07296|1|7e-26|121|acs:100568236|adiponectin	gi|148665761|gb|EDK98177.1|	4.63E-96	procollagen, type VIII, alpha 1, isoform CRA_b [Mus musculus]
mmu-miR-10a-5p	NM_007760	-31.5	0.233336	-28.27	173	GO:0019866,GO:0044439,GO:0016406,GO:0006091,GO:0006635,GO:0051234,	K00624|1|0.0|1263|mmu:12908|carnitine O-acetyltransferase [EC:2.3.1.7]	gi|85662408|ref|NP_031786.2|	0	carnitine O-acetyltransferase [Mus musculus]
mmu-miR-10a-5p	NM_008246	-30.1	0.248068	-27.1	169	GO:0031224,GO:0008493,GO:0015893,GO:0015850,GO:0006818,GO:0006811,	K14613|1|4e-07|58.2|der:Dere_GG23423|MFS transporter, PCFT/HCP family, solute carrier family 46 (folate transporter), member 1/3	gi|254540160|ref|NP_032272.2|	0	hippocampus abundant transcript 1 protein [Mus musculus]

mmu-miR-10a-5p	NM_009908	-27.9	0.35286	-25.52	152	GO:0043231,GO:0070567,GO:0008610,	K00983|1|0.0|810|mmu:12764|N-acylneuraminate cytidylyltransferase [EC:2.7.7.43]	gi|247269309|ref|NP_034038.2|	0	N-acylneuraminate cytidylyltransferase [Mus musculus]
mmu-miR-10a-5p	NM_010254	-25	0.832494	-25.83	172	GO:0031224,GO:0008188,GO:0003012,GO:0007267,GO:0006650,GO:0007610,GO:0051480,GO:0030030,GO:0007188,	K04231|1|0.0|647|mmu:14428|galanin receptor 2	gi|161353471|ref|NP_034384.3|	0	galanin receptor type 2 [Mus musculus]
mmu-miR-10a-5p	NM_010274	-26.8	0.903162	-26.3	165	GO:0043231,GO:0043234,GO:0019866,GO:0052590,GO:0046872,GO:0016616,GO:0006629,GO:0006071,GO:0019674,GO:00060	K00111|1|0.0|1356|mmu:14571|glycerol-3-phosphate dehydrogenase [EC:1.1.5.3]	gi|224922803|ref|NP_034404.3|	0	glycerol-3-phosphate dehydrogenase, mitochondrial precursor [Mus musculus]

mmu-miR-10a-5p	NM_010587	-28.5	0.931919	-25.79	155	GO:0044459,GO:0031252,GO:0016023,GO:0012505,GO:0008047,GO:0005088,GO:0046872,GO:0005515,GO:0006917,GO:0019222,GO:0007169,GO:0010740,GO:0006897,GO:0043066,GO:0007266,	K13738|1|8e-30|135|bfo:BRAFLDRAFT_90471|CD2-associated protein!K07976|4|2e-29|134|ame:409364|Rab family, other	gi|134288904|ref|NP_034717.2|	0	intersectin-1 isoform 1 [Mus musculus]
mmu-miR-10a-5p	NM_010605	-27.8	0.714569	-43.31	293	GO:0009986,GO:0042383,GO:0005887,GO:0005488,GO:0005242,GO:0007267,GO:0034220,GO:0030001,	K04999|1|0.0|805|mmu:16521|potassium inwardly-rectifying channel subfamily J member 5	gi|84370254|ref|NP_034735.3|	0	G protein-activated inward rectifier potassium channel 4 [Mus musculus]
mmu-miR-10a-5p	NM_010696	-27.5	0.651518	-26.23	174	-	K07361|1|0.0|904|mmu:16822|lymphocyte cytosolic protein 2	gi|31543115|ref|NP_034826.2|	0	lymphocyte cytosolic protein 2 [Mus musculus]

mmu-miR-10a-5p	NM_010768	-25.2	0.762743	-27.48	158	GO:0031981,GO:0004713,GO:0032559,GO:0006464,GO:0008283,GO:0009888,	K08888|1|0.0|975|mmu:17179|megakaryocyte-associated tyrosine kinase [EC:2.7.10.2]	gi|6754646|ref|NP_034898.1|	0	megakaryocyte-associated tyrosine-protein kinase [Mus musculus]
mmu-miR-10a-5p	NM_011073	-29.8	0.201313	-28.83	169	GO:0031224,GO:0005764,GO:0044440,GO:0031410,GO:0046872,GO:0015267,GO:0002347,GO:0051259,GO:0008219,GO:0048511,GO:0002252,GO:0010033,	K07818|1|0.0|1144|mmu:18646|perforin 1	gi|225735600|ref|NP_035203.3|	0	perforin-1 precursor [Mus musculus]

mmu-miR-10a-5p	NM_011076	-25.7	0.96194	-25.66	160	GO:0005911,GO:0031224,GO:0012505,GO:0032559,GO:0042626,GO:0048511,GO:0007589,GO:0010033,GO:0015893,GO:0010941,GO:0009207,GO:0033273,GO:00100	K05658|1|0.0|2296|mmu:18671|ATP-binding cassette, subfamily B (MDR/TAP), member 1	gi|225734206|pdb|3G60|A	0	Chain A, Structure Of P-Glycoprotein Reveals A Molecular Basis For Poly-Specific Drug Binding
mmu-miR-10a-5p	NM_011632	-26.9	0.934777	-25.25	154	GO:0043231,GO:0005887,GO:0044444,GO:0004871,GO:0046914,GO:0019787,GO:0019900,GO:0001818,GO:0002697,GO:0043433,GO:0019221,GO:0032479,GO:0006915,GO:0012502,GO:0032446,GO:0002221,GO:00	K03174|1|0.0|1139|rno:362788|TNF receptor-associated factor 3	gi|114842405|ref|NP_035762.2|	0	TNF receptor-associated factor 3 isoform a [Mus musculus]

mmu-miR-10a-5p	NM_011898	-27.6	0.752353	-25.29	165	GO:0042995,GO:0016020,GO:0005488,GO:0032501,GO:0043405,	K04704|1|2e-149|531|mmu:24066|sprouty	gi|5917720|gb|AAD56007.1|AF176906_1	1.05E-148	sprouty 4 [Mus musculus]
mmu-miR-10a-5p	NM_013820	-27.9	0.770476	-25.41	146	GO:0031966,GO:0004396,GO:0048029,GO:0032559,GO:0006007,GO:0006915,GO:0007589,GO:0016310,GO:0010827,	K00844|1|0.0|1834|mmu:15277|hexokinase [EC:2.7.1.1]	gi|7305143|ref|NP_038848.1|	0	hexokinase-2 [Mus musculus]
mmu-miR-10a-5p	NM_018801	-27.5	0.860487	-25.78	158	GO:0031224,GO:0000323,GO:0008021,GO:0044459,GO:0046872,GO:0007009,GO:0017156,GO:0030073,	K15290|1|2e-70|269|nvi:100120087|synaptotagmin-1	gi|9055364|ref|NP_061271.1|	0	synaptotagmin-7 alpha isoform [Mus musculus]

mmu-miR-10a-5p	NM_019403	-32	0.034954	-29.89	168	GO:0031224,GO:0031090,GO:0005740,GO:0046914,GO:0019787,GO:0000209,GO:0006515,	K10666|1|7e-85|314|rno:407784|E3 ubiquitin-protein ligase RNF5 [EC:6.3.2.19]	gi|9507059|ref|NP_062276.1|	4.61E-84	E3 ubiquitin-protein ligase RNF5 [Mus musculus]
mmu-miR-10a-5p	NM_019765	-28.9	0.552034	-27.74	162	GO:0043231,GO:0005874,GO:0000775,GO:0044444,GO:0008017,GO:0046914,GO:0042802,GO:0031112,GO:0007067,	K10421|1|0.0|2315|mmu:56430|CAP-Gly domain-containing linker protein 1	gi|23821025|ref|NP_062739.2|	0	CAP-Gly domain-containing linker protein 1 [Mus musculus]
mmu-miR-10a-5p	NM_021521	-28.6	0.810486	-25.16	149	GO:0043234,GO:0031981,GO:0004871,GO:0008134,GO:0003713,GO:0016055,GO:0006357,GO:0030518,GO:0006352,	K15162|1|0.0|3638|mmu:59024|mediator of RNA polymerase II transcription subunit 12	gi|123226658|emb|CAM24448.1|	0	mediator of RNA polymerase II transcription, subunit 12 homolog (yeast) [Mus musculus]

mmu-miR-10a-5p	NM_022889	-31.1	0.176979	-28.98	175	GO:0043234,GO:0031981,GO:0005694,GO:0030684,GO:0005488,GO:0000470,GO:0007067,GO:0006997,GO:0000460,	K14843|1|0.0|1055|mmu:64934|pescadillo	gi|12584984|ref|NP_075027.1|	0	pescadillo homolog [Mus musculus]
mmu-miR-10a-5p	NM_024412	-28.2	0.421437	-26.04	157	GO:0043231,GO:0044459,GO:0034702,GO:0005488,GO:0005254,GO:0010038,GO:0046903,GO:0009628,GO:0006821,GO:0001822,GO:0034220,GO:0043269,GO:00069	K05017|1|0.0|1256|mmu:12733|chloride channel Ka!K05018|3|0.0|1075|mmu:56365|chloride channel Kb	gi|226423866|ref|NP_077723.3|	0	chloride channel protein ClC-Ka [Mus musculus]

mmu-miR-10a-5p	NM_025316	-32.8	0.020834	-29.19	174	GO:0031224,GO:0005746,GO:0050136,GO:0051234,GO:0022904,	K03961|1|1e-106|386|mmu:66046|NADH dehydrogenase (ubiquinone) 1 beta subcomplex 5 [EC:1.6.5.3 1.6.99.3]	gi|27754144|ref|NP_079592.2|	8.22E-106	NADH dehydrogenase [ubiquinone] 1 beta subcomplex subunit 5, mitochondrial precursor [Mus musculus]
mmu-miR-10a-5p	NM_025659	-33	0.121312	-33.46	184	GO:0031252,GO:0044424,GO:0043169,GO:0016791,GO:0016477,GO:0006468,	K06124|1|7e-153|542|rno:287644|phosphoethanolamine/phosphocholine phosphatase [EC:3.1.3.7	gi|157786864|ref|NP_001099303.1|	5.15E-152	phosphoethanolamine/phosphocholine phosphatase [Rattus norvegicus]

mmu-miR-10a-5p	NM_026825	-27.2	0.839886	-46.23	311	GO:0044444,GO:0043231,GO:0007599,GO:0006082,	K11147|1|8e-17|91.3|oaa:100092951|dehydrogenase/reductase SDR family member 4 [EC:1.1.-.-]!K08727|3|1e-09|67.4|ptr:472336|nucleotide-binding oligomerization domain-containing protein 1	gi|154146185|ref|NP_081101.3|		0	leucine-rich repeat-containing protein 16A [Mus musculus]

mmu-miR-10a-5p	NM_027504	-27.1	0.951811	-25.38	164	GO:0043231,GO:0043234,GO:0046914,GO:0005515,GO:0003712,GO:0003677,GO:0007423,GO:0002762,GO:0007179,GO:0006357,GO:0043467,GO:0045444,GO:0019827,GO:00	K04462|1|0.0|1093|cfa:488148|ecotropic virus integration site 1 protein	gi|124107623|ref|NP_081780.3|	0	PR domain zinc finger protein 16 isoform 1 [Mus musculus]
mmu-miR-10a-5p	NM_027998	-29.3	0.218933	-28.14	158	GO:0043296,GO:0007043,	K06087|1|4e-139|495|mmu:71908|claudin	gi|21312576|ref|NP_082274.1|	2.94E-138	claudin-23 [Mus musculus]
mmu-miR-10a-5p	NM_029999	-27.2	0.665056	-30.14	156	GO:0031981,GO:0032501,GO:0006355,	-	gi|34328474|ref|NP_084275.3|	8.71E-56	protein LBH [Mus musculus]
mmu-miR-10a-5p	NM_030709	-29.2	0.256093	-27.34	161	GO:0031224,GO:0004175,GO:0004888,GO:0019538,GO:0023052,	K09636|1|0.0|898|mmu:80893|transmembrane protease, serine 5 (spinesin) [EC:3.4.21.-]	gi|148693772|gb|EDL25719.1|	0	transmembrane protease, serine 5 (spinesin), isoform CRA_a [Mus musculus]

mmu-miR-10a-5p	NM_030886	-26.9	0.976397	-25.23	162	GO:0043231,GO:0003676,GO:0051704,	K15503|1|7e-78|295|spu:584964|serine/threonine-protein phosphatase 6 regulatory ankyrin repeat subunit B	gi|148673379|gb|EDL05326.1|	0	ankyrin repeat domain 17, isoform CRA_c [Mus musculus]
mmu-miR-10a-5p	NM_080853	-28	0.671704	-26.18	167	GO:0031224,GO:0008021,GO:0044459,GO:0005768,GO:0015172,GO:0015291,GO:0015711,GO:0001505,GO:0030001,GO:0006865,	K12302|1|0.0|1189|mmu:140919|MFS transporter, ACS family, solute carrier family 17 (sodium-dependent inorganic phosphate cotransporter), member 6/7/8	gi|188219544|ref|NP_543129.3|	0	vesicular glutamate transporter 2 [Mus musculus]

mmu-miR-10a-5p	NM_133710	-26.4	0.899144	-45.7	302	GO:0043231,GO:0016791,GO:0043169,GO:0006796,	K15731|1|2e-158|561|mmu:69274|carboxy-terminal domain RNA polymerase II polypeptide A small phosphatase [EC:3.1.3.1	gi|148677300|gb|EDL09247.1|	1.62E-157	CTD (carboxy-terminal domain, RNA polymerase II, polypeptide A) small phosphatase-like [Mus musculus]
mmu-miR-10a-5p	NM_146152	-29.4	0.398993	-27.38	177	GO:0031074,GO:0043231,GO:0035258,GO:0022892,GO:0002146,	K15436|1|0.0|1522|tgu:100222227|transportin-3	gi|39104529|dbj|BAC98010.2|	0	mKIAA0724 protein [Mus musculus]
mmu-miR-10a-5p	NM_153572	-26.8	0.921429	-25.36	168	GO:0015630,GO:0042623,GO:0032559,GO:0000087,GO:0009207,	K07767|1|0.0|915|mmu:231912|microtubule-severing ATPase [EC:3.6.4.3]	gi|148673904|gb|EDL05851.1|	0	katanin p60 subunit A-like 1, isoform CRA_a [Mus musculus]

mmu-miR-10a-5p	NM_172578	-30.4	0.317211	-25.25	164	GO:0044427,GO:0005634,GO:0003676,GO:0034728,GO:0000087,	-	gi|148704698|gb|EDL36645.1|	0	expressed sequence C79407 [Mus musculus]
mmu-miR-10a-5p	NM_172739	-26.7	0.968887	-26.33	164	GO:0043231,GO:0005856,GO:0044444,GO:0004871,GO:0003712,GO:0003676,GO:0005099,GO:0006351,GO:0007166,GO:0014020,GO:0007420,GO:0042462,GO:0006935,GO:0043114,GO:0032319,	K05732|1|0.0|2774|mmu:232906|glucocorticoid receptor DNA-binding factor 1	gi|75677442|ref|NP_766327.3|	0	rho GTPase-activating protein 35 [Mus musculus]
mmu-miR-10a-5p	NM_172755	-30.4	0.307136	-28.46	161	GO:0043231,GO:0003676,GO:0006396,	K13166|1|0.0|2060|mmu:234373|splicing factor, arginine/serine-rich 14	gi|148696857|gb|EDL28804.1|	0	splicing factor, arginine/serine-rich 14, isoform CRA_b [Mus musculus]

mmu-miR-10a-5p	NM_173067	-27.5	0.86567	-25.78	158	GO:0031224,GO:0000323,GO:0008021,GO:0044459,GO:0046872,GO:0007009,GO:0017156,GO:0030073,	K15290|1|2e-68|262|nvi:100120087|synaptotagmin-1	gi|41281824|ref|NP_775090.1|	0	synaptotagmin-7 beta isoform [Mus musculus]
mmu-miR-10a-5p	NM_173068	-27.5	0.878733	-25.78	158	GO:0031224,GO:0000323,GO:0008021,GO:0044459,GO:0046872,GO:0017156,GO:00300	K15290|1|3e-68|262|nvi:100120087|synaptotagmin-1	gi|160948571|ref|NP_775091.2|	0	synaptotagmin-7 gamma isoform [Mus musculus]
mmu-miR-10a-5p	NM_175439	-27.2	0.641065	-27.93	168	GO:0044429,GO:0004812,GO:0032559,GO:0006418,	K01874|1|0.0|1211|mmu:212679|methionyl-tRNA synthetase [EC:6.1.1.10]	gi|26335385|dbj|BAC31393.1|	0	unnamed protein product [Mus musculus]

mmu-miR-10a-5p	NM_177066	-29.7	0.311391	-25.47	171	GO:0043231,GO:0043169,GO:0008092,GO:0004672,GO:0032559,GO:0006464,GO:0006796,	K15502|1|6e-41|170|aag:AaeL_AAEL014742|serine/threonine-protein phosphatase 6 regulatory ankyrin repeat subunit A	gi|260166668|ref|NP_796040.3|	0	serine/threonine-protein kinase TNNI3K [Mus musculus]

mmu-miR-10a-5p	NM_177410	-26.9	0.637853	-27.77	151	GO:0031966,GO:0031224,GO:0005635,GO:0051400,GO:0019903,GO:0042802,GO:0046983,GO:0003677,GO:0001894,GO:0014074,GO:0001934,GO:0035264,GO:0051384,GO:0002244,GO:0048754,GO:0016567,GO:0007409,GO:0001503,GO:0030183,GO:0003008,GO:0007006,GO:00330	K02161|1|3e-98|360|mmu:12043|apoptosis regulator BCL-2	gi|133893254|ref|NP_803129.2|	1.44E-101	apoptosis regulator Bcl-2 isoform 2 [Mus musculus]
mmu-miR-10a-5p	NM_177412	-28.4	0.711574	-25.05	162	GO:0031224,	-	gi|50510629|dbj|BAD32300.1|	0	mKIAA0779 protein [Mus musculus]

mmu-miR-10a-5p	NM_177790	-27.9	0.665132	-25.55	156	GO:0005488,	K06062|1|2e-15|86.3|bta:514420|histone acetyltransferase [EC:2.3.1.48]!K08421|2|1e-10|70.1|cfa:477591|G protein-coupled receptor 84	gi|267844809|ref|NP_808458.2|	1.13E-154	zinc finger protein 385C [Mus musculus]
mmu-miR-10a-5p	NM_177856	-27.1	0.413781	-26.14	163	GO:0031224,GO:0016787,GO:0005488,	-	gi|29244450|ref|NP_808524.1|	1.07E-167	uncharacterized protein C3orf33 homolog [Mus musculus]
mmu-miR-10a-5p	NM_178688	-34	0.142522	-32.3	179	GO:0005856,GO:0008092,GO:0046914,GO:0006996,GO:0006357,GO:0006935,	K07520|1|0.0|1427|cfa:477828|actin-binding LIM protein	gi|157057145|ref|NP_848803.3|	0	actin-binding LIM protein 1 isoform 1 [Mus musculus]
mmu-miR-10a-5p	NM_178694	-25.7	0.936113	-25.18	151	GO:0031461,GO:0005488,GO:0019787,GO:0031396,	K10350|1|0.0|1560|mmu:227693|Zyg-11 protein	gi|31542271|ref|NP_848809.2|	0	protein zer-1 homolog [Mus musculus]

mmu-miR-10a-5p	NM_178773	-25.3	0.961449	-25.14	164	GO:0034702,GO:0005253,GO:0006810,	K05169|1|4e-34|148|ptr:743996|single Ig IL-1R-related molecule! K13982|2|1e-26|123|dgr:Dgri_GH10918|probable ATP-dependent RNA helicase DDX4 [EC:3.6.4.13]	gi|148689550|gb|EDL21497.1|	0	transmembrane protein 16D (eight membrane-spanning domains), isoform CRA_a [Mus musculus]
mmu-miR-10a-5p	NM_206924	-30.1	0.083353	-26.71	173	GO:0016021,	-	gi|74226894|dbj|BAE27091.1|	2.73E-60	unnamed protein product [Mus musculus]
mmu-miR-10a-5p	NR_024328	-25.4	0.79971	-46.46	299	-	-	gi|148707601|gb|EDL39548.1|	7.88E-26	mCG148400 [Mus musculus]
mmu-miR-10a-5p	NR_033727	-30.6	0.027053	-29.13	175	GO:0043231,GO:0015935,GO:0003676,GO:0046914,GO:0005198,GO:0006412,GO:0019080,GO:0031016,GO:0007067,	K02978|1|2e-36|152|mdo:100020225|small subunit ribosomal protein S27e	gi|126307742|ref|XP_001372806.1|	9.90E-36	PREDICTED: 40S ribosomal protein S27-like [Monodelphis domestica]

mmu-miR-10a-5p	NR_040356	-29.7	0.099212	-29.02	167	-	-	gi|148694981|gb|EDL26928.1|	7.71E-23	mCG1040446 [Mus musculus]
mmu-miR-10a-5p	NR_040679	-27.8	0.226923	-26.03	165	-	-	gi|148688352|gb|EDL20299.1|	2.69E-15	mCG147648 [Mus musculus]
mmu-miR-10a-5p	NR_045820	-27.6	0.420968	-25.29	163	-	-	gi|148687710|gb|EDL19657.1|	3.76E-65	mCG1030587 [Mus musculus]
mmu-miR-10a-5p	NR_046275	-26	0.895221	-25.29	146	-	K09371|1|4e-06|55.1|bta:100337389|LIM homeobox transcription factor 1	gi|148676656|gb|EDL08603.1|	2.37E-74	hypothetical protein C130021I20 [Mus musculus]
mmu-miR-10a-5p	NR_033657	-21.4	1	-86.55	601	-	-	gi|148688037|gb|EDL19984.1|	6.79E-121	mCG128631, isoform CRA_c [Mus musculus]
mmu-miR-10a-5p	NM_027917	-22.8	0.999503	-44.57	323	GO:0016459,GO:0015630,GO:0003779,GO:0007015,GO:0032989,	-	gi|125628648|ref|NP_082193.2|	0	protein Shroom1 [Mus musculus]
mmu-miR-10a-5p	NM_001081064	-21.7	1	-44.69	322	GO:0030054,GO:0043231,GO:0044430,GO:0007165,	K06095|1|5e-31|139|gga:427360|multiple PDZ domain protein	gi|124486716|ref|NP_001074533.1|	0	PDZ domain-containing protein 2 [Mus musculus]

mmu-miR-10a-5p	NM_133904	-23.4	0.999997	-42.62	318	GO:0043231,GO:0044444,GO:0019842,GO:0043169,GO:0016879,GO:0016421,GO:0032559,GO:0009893,GO:0051260,GO:0003333,GO:0019217,GO:0006732,GO:0015980,GO:00	K11262|1|0.0|4772|mmu:100705|acetyl-CoA carboxylase / biotin carboxylase [EC:6.4.1.2 6.3.4.14]	gi|157042798|ref|NP_598665.2|	0	acetyl-Coenzyme A carboxylase beta precursor [Mus musculus]
mmu-miR-10a-5p	NM_011866	-22.2	1	-43.38	317	GO:0044444,GO:0043025,GO:0043169,GO:0019002,GO:0004114,GO:0016208,GO:0006195,GO:0001775,GO:0023052,GO:00075	K01120|1|0.0|1624|mmu:23984|3',5'-cyclic-nucleotide phosphodiesterase [EC:3.1.4.17]	gi|70909347|ref|NP_035996.2|	0	cAMP and cAMP-inhibited cGMP 3',5'-cyclic phosphodiesterase 10A [Mus musculus]
mmu-miR-10a-5p	NM_197990	-23	1	-41.69	313	-	-	gi|37574070|ref|NP_932107.1|	3.28E-62	uncharacterized protein C1orf21 homolog [Mus musculus]

mmu-miR-10a-5p	NM_145427	-22	0.998564	-41.88	311	GO:0016604,GO:0005488,GO:0070071,	K07556|1|2e-155|550|mmu:246782|ATP synthase mitochondrial F1 complex assembly factor 2	gi|238776839|ref|NP_663402.2|	1.35E-154	ATP synthase mitochondrial F1 complex assembly factor 2 [Mus musculus]
mmu-miR-10a-5p	NM_198625	-24.2	0.996999	-43.16	310	GO:0030674,GO:0008092,GO:0019904,GO:0030035,GO:0023052,	K05627|1|2e-10|70.1|xtr:100158528|BAI1-associated protein 2	gi|38348492|ref|NP_941027.1|	0	MTSS1-like protein [Mus musculus]

mmu-miR-10a-5p	NM_011941	-24	0.999818	-43.91	306	GO:0005488,GO:0000186,	K01047|1|4e-35|152|oaa:100089728|phospholipase A2 [EC:3.1.1.4]!K11856|2|6e-14|82.0|ptr:455080|ubiquitin carboxyl-terminal hydrolase 43 [EC:3.1.2.15]!K14555|3|7e-13|78.6|ptr:454343|U3 small nucleolar RNA-associated protein 13!K03362|5|8e-	gi|213385289|ref|NP_036071.3|	0	mitogen-activated protein kinase-binding protein 1 [Mus musculus]
mmu-miR-10a-5p	NM_007743	-20.7	1	-40.71	302	GO:0031012,GO:0005515,	K06236|1|0.0|793|mmu:12843|collagen, type I/II/III/V/XI, alpha	gi|74144541|dbj|BAE36107.1|	0	unnamed protein product [Mus musculus]
mmu-miR-10a-5p	NM_146091	-22.7	0.999999	-43.81	300	-	-	gi|148701364|gb|EDL33311.1|	0	RIKEN cDNA 5730596K20 [Mus musculus]

mmu-miR-10a-5p	NM_025276	-20.4	1	-41.65	299	GO:0005856,GO:0030054,GO:0005515,GO:0030216,GO:0006464,	K10383|1|0.0|3239|mmu:14027|envoplakin	gi|111185907|ref|NP_079552.3|	0	envoplakin [Mus musculus]
mmu-miR-10a-5p	NM_025989	-20.5	0.999998	-42.42	298	GO:0044424,GO:0031224,	K10307|1|8e-25|116|acs:100552900|F-box protein 30!K13912|2|3e-12|74.7|tgu:100226373|deleted in malignant brain tumors 1 protein	gi|27229028|ref|NP_080265.2|	0	pancreatic secretory granule membrane major glycoprotein GP2 precursor [Mus musculus]

mmu-miR-10a-5p	NM_007539	-21.8	0.994398	-46.52	297	GO:0043231,GO:0016021,GO:0042995,GO:0008528,GO:0010524,GO:0001932,GO:0007186,GO:0007600,GO:0016049,GO:0002237,GO:0002684,GO:0006952,GO:00082	K03915|1|1e-165|583|mmu:12061|bradykinin receptor B1	gi|4097022|gb|AAD00029.1|	4.53E-165	bradykinin B1 receptor [Mus musculus]
mmu-miR-10a-5p	NM_001126047	-22	0.999994	-44.4	295	GO:0031224,GO:0008021,GO:0044459,GO:0044430,GO:0005488,GO:0004871,GO:0048731,GO:0032872,GO:0030154,	K06521|1|0.0|1541|mmu:20353|semaphorin 4	gi|148682548|gb|EDL14495.1|	0	mCG17761, isoform CRA_b [Mus musculus]
mmu-miR-10a-5p	NM_178280	-22.3	1	-44.39	293	GO:0044424,GO:0005515,	K09228|1|5e-17|92.4|xtr:100216012|KRAB domain-containing zinc finger protein	gi|49257163|gb|AAH72631.1|	0	Sall3 protein, partial [Mus musculus]

mmu-miR-10a-5p	NM_028762	-21.4	1	-41.92	292	GO:0043232,GO:0031981,GO:0003676,GO:0009790,	K14787|1|0.0|1643|mmu:74111|multiple RNA-binding domain-containing protein 1	gi|30794154|ref|NP_083038.1|	0	probable RNA-binding protein 19 [Mus musculus]
mmu-miR-10a-5p	NM_001102607	-20	1	-42.09	284	GO:0031012,GO:0006935,GO:0009987,	K06238|1|0.0|4195|mmu:245026|collagen, type VI, alpha	gi|156616286|ref|NP_001096077.1|	0	collagen alpha-6(VI) chain isoform 1 precursor [Mus musculus]
mmu-miR-10a-5p	NM_053257	-20.1	0.999884	-30.23	177	-	K02910|1|1e-58|227|aml:100476308|large subunit ribosomal protein L31e	gi|148672373|gb|EDL04320.1|	2.54E-68	mCG49427 [Mus musculus]

mmu-miR-10a-5p	NM_009874	-20.8	1	-23.98	176	GO:0005667,GO:0043232,GO:0044444,GO:0003712,GO:0004674,GO:0032559,GO:0035258,GO:0042623,GO:0006357,GO:0006397,GO:0030518,GO:0006352,GO:0006796,GO:0051320,GO:0006353,GO:0051318,GO:0009207,GO:0006289,GO:0051726,GO:0006354,GO:0006464,GO:00459	K02202|1|0.0|702|mmu:12572|cyclin-dependent kinase 7 [EC:2.7.11.22 2.7.11.23]	gi|160333726|ref|NP_034004.2|	0	cyclin-dependent kinase 7 [Mus musculus]
mmu-miR-10a-5p	NM_178721	-27.1	0.964056	-23.53	173	GO:0043005,GO:0031224,GO:0043025,GO:0044424,GO:0005488,GO:0007417,GO:00452	K06782|1|0.0|757|rno:360687|immunoglobulin superfamily, member 4D	gi|225543375|ref|NP_848836.2|	0	cell adhesion molecule 2 isoform 1 precursor [Mus musculus]

mmu-miR-10a-5p	NM_172709	-23	0.998828	-29.97	172	GO:0044421,GO:0031224,GO:0042471,GO:0009888,GO:0009581,	-	gi|148705604|gb|EDL37551.1|		0	otopetrin 1, isoform CRA_b [Mus musculus]
mmu-miR-10a-5p	NM_029274	-24	0.999956	-25.86	171	GO:0043231,GO:0046914,GO:0001071,GO:0008168,GO:0034968,GO:0006351,GO:0006338,	K14959|1|0.0|3601|mmu:75410|histone-lysine N-methyltransferase MLL4 [EC:2.1.1.4	gi|115495457|ref|NP_083550.2|		0	histone-lysine N-methyltransferase MLL4 [Mus musculus]
mmu-miR-10a-5p	NM_178654	-23.2	0.99998	-22.85	170	GO:0043231,GO:0019899,GO:0004674,GO:0032559,GO:0006464,GO:0023052,	K06071|1|0.0|1677|mmu:109333|protein kinase N [EC:2.7.11.13]	gi|260099670|ref|NP_848769.2|		0	serine/threonine-protein kinase N2 [Mus musculus]
mmu-miR-10a-5p	NM_026574	-25.9	0.975612	-20.3	169	GO:0043231,GO:0003676,GO:0004386,GO:0032559,GO:0016568,GO:0009207,	K11665|1|0.0|2492|mmu:68142|DNA helicase INO80 [EC:3.6.4.12]	gi|62234443|ref|NP_080850.2|		0	DNA helicase INO80 [Mus musculus]

mmu-miR-10a-5p	NR_028297	-27.3	0.480177	-24.53	168	GO:0005634,GO:0005488,	K10892|1|0.0|479|mmu:72775|fanconi anemia group E protein	gi|255652993|ref|NP_001157291.1|	0	Fanconi anemia, complementation group E isoform 1 [Mus musculus]
mmu-miR-10a-5p	NR_046460	-24.2	0.918625	-22.07	167	-	-	-	-	-
mmu-miR-10a-5p	NM_148935	-28.2	0.498171	-23.23	166	GO:0043231,GO:0043234,GO:0005515,GO:0000981,GO:0043565,GO:0043566,GO:0000122,GO:0007275,GO:0045165,GO:0044057,GO:0048588,	K09407|1|0.0|993|mmu:116810|forkhead box protein N	gi|91598891|ref|NP_683737.2|	0	forkhead box protein N4 [Mus musculus]
mmu-miR-10b-3p	NM_001039720	-26.8	0.221489	-25.69	157	GO:0044425,GO:0009986,	K06846|1|6e-25|77.0|mmu:110454|lymphocyte antigen 6 complex	gi|148697528|gb|EDL29475.1|	3.37E-48	mCG22120 [Mus musculus]

mmu-miR-10b-3p	NM_001042659	-26.9	0.891237	-25.59	168	GO:0043231,GO:0031224,GO:0012505,GO:0019904,GO:0019900,GO:0004888,GO:0030217,GO:0048568,GO:0007417,GO:0006357,GO:0016055,GO:0043010,GO:0008283,GO:0045137,GO:0009791,GO:0021700,GO:0048667,GO:0001819,GO:0006915,GO:0000578,GO:0048646,GO:00022	K02375|1|0.0|1049|mmu:14367|frizzled 5/8	gi|47059488|ref|NP_073558.2|	0	frizzled-5 precursor [Mus musculus]

mmu-miR-10b-3p	NM_001083894	-29.5	0.338317	-28.73	166	GO:0044464,GO:0003824,GO:0006629,	K01066|1|7e-123|442|dre:445105|esterase / lipase [EC:3.1.1.-]!K13618|2|8e-74|280|mgp:100546329|phospholipase A1 member A [EC:3.1.1.-]	gi|148665200|gb|EDK97616.1|	0	lipase, member H, isoform CRA_d [Mus musculus]
mmu-miR-10b-3p	NM_001161329	-27.4	0.559738	-26.11	156	GO:0031981,	-	gi|238550126|ref|NP_001154801.1|	0	nucleolar protein 11 isoform 2 [Mus musculus]
mmu-miR-10b-3p	NM_001162538	-27.3	0.503782	-26.28	162	-	K11499|1|7e-18|93.6|hsa:1063|centromere protein F	gi|244793625|ref|NP_001156010.1|	0	outer dense fiber protein 2-like isoform 1 [Mus musculus]

mmu-miR-10b-3p	NM_007913	-26.4	0.714979	-25.26	162	GO:0043231,GO:0001071,GO:0046872,GO:0043565,GO:0043566,GO:0010332,GO:0007178,GO:0072126,GO:0048168,GO:0007610,GO:0019221,GO:0030178,GO:0043434,GO:0071398,GO:0009746,GO:0016925,GO:00	K09203|1|0.0|810|mmu:13653|early growth response protein 1	gi|1325982|gb|AAB00468.1|	0	zinc finger protein [Mus musculus]

mmu-miR-10b-3p	NM_008679		-27	0.903965	-27.98	156	GO:0031981,GO:0004871,GO:0004468,GO:0003676,GO:0003713,GO:0035258,GO:0008134,GO:0006357,GO:0040007,GO:0016570,GO:0048608,GO:0030520,GO:0006629,GO:00	K11256|1|0.0|2313|mmu:17979|nuclear receptor coactivator 3 [EC:2.3.1.48]	gi|118026946|ref|NP_032705.2|		0 nuclear receptor coactivator 3 [Mus musculus]
mmu-miR-10b-3p	NM_009988		-27.1	0.384452	-26.88	167	GO:0043296,GO:0070161,GO:0016021,GO:0030141,GO:0005488,GO:0004871,GO:0006996,GO:0002682,GO:0051704,GO:0034330,GO:0023052,GO:0008285,GO:0048747,GO:0048513,GO:0007599,	K06788|1|0.0|667|rno:89843|coxsackievirus and adenovirus receptor	gi|6857775|ref|NP_034118.1|		0 coxsackievirus and adenovirus receptor homolog isoform b precursor [Mus musculus]

mmu-miR-10b-3p	NM_013586	-26.5	0.79636	-25.67	152	GO:0044421,GO:0044464,GO:0046914,GO:0016641,GO:0004888,GO:0008152,GO:0023052,	K00280|1|0.0|1580|mmu:16950|lysyl oxidase-like protein 2/3/4 [EC:1.4.3.-]	gi|31543127|ref|NP_038614.2|	0	lysyl oxidase homolog 3 precursor [Mus musculus]
mmu-miR-10b-3p	NM_019402	-29.1	0.182313	-27.02	165	GO:0032991,GO:0031981,GO:0003729,GO:0019056,GO:0000377,GO:0003012,GO:0031124,GO:0006406,GO:0006353,	K14396|1|7e-94|345|mmu:54196|polyadenylate-binding protein 2	gi|9506945|ref|NP_062275.1|	4.74E-93	polyadenylate-binding protein 2 [Mus musculus]

mmu-miR-10b-3p	NM_022721	-26.9	0.894664	-25.59	168	GO:0043231,GO:0031224,GO:0012505,GO:0019904,GO:0019900,GO:0004888,GO:0030217,GO:0048568,GO:0007417,GO:0006357,GO:0016055,GO:0043010,GO:0008283,GO:0045137,GO:0009791,GO:0021700,GO:0048667,GO:0001819,GO:0006915,GO:0000578,GO:0048646,GO:00022	K02375|1|0.0|1049|mmu:14367|frizzled 5/8	gi|47059488|ref|NP_073558.2|	0	frizzled-5 precursor [Mus musculus]
mmu-miR-10b-3p	NM_025811	-28.5	0.481389	-27.08	165	GO:0044464,GO:0019725,	K12035|1|2e-06|56.2|phu:Phum_PHUM256370|tripartite motif-containing protein	gi|29789158|ref|NP_080087.1|	0	NHL repeat-containing protein 2 [Mus musculus]

mmu-miR-10b-3p	NM_030557	-29.2	0.404696	-27.88	169	GO:0043231,GO:0046914,GO:0003676,GO:0001071,GO:0006351,	K09228|1|2e-71|272|api:100159445|KRAB domain-containing zinc finger protein	gi|41056113|ref|NP_085034.2|	0	myoneurin [Mus musculus]
mmu-miR-10b-3p	NM_031185	-28.8	0.608366	-28.68	167	GO:0044424,GO:0016020,GO:0016772,GO:0032947,GO:0019899,GO:0006796,GO:0010737,GO:0006171,GO:0006886,	K09880|1|2e-09|66.6|aag:AaeL_AAEL000109|enolase-phosphatase E1 [EC:3.1.3.77]!K04573|3|1e-07|60.8|rno:24588|neurofilament medium polypeptide (neurofilament 3)!K00907|4|3e-07|59.7|dme:Dmel_CG18255|myosin-light-chain	gi|13626040|ref|NP_112462.1|	0	A-kinase anchor protein 12 [Mus musculus]

mmu-miR-10b-3p	NM_033617	-25.9	0.352936	-26.49	155	GO:0031224,GO:0043231,GO:0033176,GO:0031090,GO:0015077,GO:0007169,GO:0006875,GO:0015988,GO:0015031,	K03661|1|6e-104|377|mmu:114143|V-type H+-transporting ATPase 21kDa proteolipid subunit [EC:3.6.3.14]	gi|15991795|ref|NP_291095.1|	3.82E-103	V-type proton ATPase 21 kDa proteolipid subunit [Mus musculus]
mmu-miR-10b-3p	NM_133702	-27.4	0.567172	-26.11	156	GO:0031981,	-	gi|238550124|ref|NP_598463.2|	0	nucleolar protein 11 isoform 1 [Mus musculus]
mmu-miR-10b-3p	NM_153404	-29.5	0.179958	-28.73	166	GO:0044464,GO:0003824,GO:0006629,	K01066|1|2e-103|377|dre:445105|esterase / lipase [EC:3.1.1.-]!K13618|2|1e-58|228|mgp:100546329|phospholipase A1 member A [EC:3.1.1.-]	gi|148665198|gb|EDK97614.1|	0	lipase, member H, isoform CRA_b [Mus musculus]

mmu-miR-10b-3p	NM_178189	-27.9	0.40261	-25.77	160	GO:0000785,GO:0043231,GO:0003676,GO:0005515,GO:0034728,	K11251|1|2e-53|211|cfa:100856200|histone H2A	gi|380800771|gb|AFE72261.1|	1.59E-52	histone H2A type 1-B/E, partial [Macaca mulatta]
mmu-miR-10b-3p	NM_198103	-30.5	0.426899	-28.23	180	GO:0043234,GO:0043231,GO:0044444,GO:0030427,GO:0017016,GO:0005543,GO:00161	-	gi|37674218|ref|NP_932771.1|	0	exocyst complex component 8 [Mus musculus]
mmu-miR-10b-3p	NM_028004	-22.5	1	-41.43	307	-	K12567|1|0.0|4.870e+04|hsa:7273|titin [EC:2.7.11.1]	gi|77812699|ref|NP_082280.2|	0	titin isoform N2-B [Mus musculus]
mmu-miR-10b-3p	NM_021521	-23.4	0.999983	-22.02	174	GO:0043234,GO:0031981,GO:0004871,GO:0008134,GO:0003713,GO:0016055,GO:0006357,GO:0030518,GO:0006352,	K15162|1|0.0|3638|mmu:59024|mediator of RNA polymerase II transcription subunit 12	gi|123226658|emb|CAM24448.1|	0	mediator of RNA polymerase II transcription, subunit 12 homolog (yeast) [Mus musculus]

mmu-miR-10b-3p	NR_046182	-27.3	0.560488	-24.71	171	GO:0005488,	K05185|1|2e-12|75.9|rno:65191|gamma-aminobutyric acid receptor subunit epsilon	gi|256773195|ref|NP_076310.2|	0	cardiomyopathy-associated protein 5 [Mus musculus]
mmu-miR-10b-3p	NM_173066	-26.6	0.975783	-24.26	170	GO:0016604,GO:0043189,GO:0017111,GO:0003676,GO:0032559,GO:0016573,	K11320|1|0.0|3918|mmu:75560|E1A-binding protein p400 [EC:3.6.4.-]	gi|148688062|gb|EDL20009.1|	0	E1A binding protein p400, isoform CRA_c [Mus musculus]
mmu-miR-10b-3p	NM_016740	-25.2	0.22076	-20.62	169	GO:0031252,GO:0043231,GO:0042802,GO:0046872,GO:0048232,	K10384|1|3e-09|61.6|aml:100480898|filaggrin	gi|21886811|ref|NP_058020.1|	3.48E-50	protein S100-A11 [Mus musculus]
mmu-miR-10b-3p	NM_173861	-25	0.611859	-24.11	168	-	K00161|1|3e-07|57.0|mdo:100014220|pyruvate dehydrogenase E1 component subunit alpha [EC:1.2.4.1]	gi|344238891|gb|EGV94994.1|	2.31E-152	hypothetical protein I79_002834 [Cricetulus griseus]

mmu-miR-10b-3p	NM_028248	-25.8	0.912848	-24.22	167	GO:0031224,	K05117|1|2e-08|63.2|dre:100322133|c-mer proto-oncogene tyrosine kinase [EC:2.7.10.	gi|124487303|ref|NP_082524.2|	0	transmembrane protein 87B precursor [Mus musculus]
mmu-miR-10b-3p	NR_003188	-24.3	0.875897	-21.21	166	-	-	gi|148697167|gb|EDL29114.1|	1.64E-12	mCG147982 [Mus musculus]
mmu-miR-10b-3p	NM_178906	-25.4	0.968422	-23.76	165	-	-	gi|26343487|dbj|BAC35400.1|	4.87E-63	unnamed protein product [Mus musculus]
mmu-miR-10b-3p	NR_029450	-27	0.527791	-23.14	164	-	-	gi|26352149|dbj|BAC39711.1|	2.33E-49	unnamed protein product [Mus musculus]
mmu-miR-10b-3p	NM_173779	-23.7	0.878476	-20.45	163	-	-	gi|30424651|ref|NP_776140.1|	4.07E-162	ankyrin repeat domain-containing protein SOWAHD [Mus musculus]

mmu-miR-10b-3p	NM_177034	-22.4	0.999999	-20.72	162	GO:0032991,GO:0030136,GO:0016020,GO:0035091,GO:0019904,GO:0048731,GO:0043009,GO:0040007,GO:0023061,GO:0006887,GO:0010467,GO:0007610,GO:0065003,GO:0015031,GO:0010970,	K04531|1|0.0|1538|mmu:319924|amyloid beta (A4) precursor protein-binding, family A, member 1 (X11)	gi|187954913|gb|AAI41182.1|	0	Apba1 protein [Mus musculus]
mmu-miR-10b-3p	NR_027987	-24.7	0.893184	-22.3	161	-	-	gi|26338926|dbj|BAC33134.1|	1.77E-62	unnamed protein product [Mus musculus]
mmu-miR-10b-3p	NM_010435	-20.6	1	-20.43	160	GO:0016604,GO:0000785,GO:0005488,GO:0003712,GO:0001071,GO:0048598,GO:0006355,GO:0006325,	K11293|1|0.0|1791|mmu:15260|protein HIRA/HIR1	gi|52426778|ref|NP_034565.2|	0	protein HIRA [Mus musculus]

mmu-miR-10b-3p	NM_144824	-22.2	0.993778	-20.66	159	GO:0016604,GO:0030529,GO:0003676,GO:0006259,GO:0051347,	K03130|1|3e-14|80.9|cfa:100688981|transcription initiation factor TFIID subunit 5	gi|21450205|ref|NP_659073.1|	0	telomerase Cajal body protein 1 [Mus musculus]
mmu-miR-10b-3p	NR_036469	-22.8	0.997159	-25.56	158	-	-	-	-	-
mmu-miR-10b-3p	NR_030683	-22.9	0.841412	-21.97	157	-	-	gi|74193018|dbj|BAE20559.1|	1.35E-52	unnamed protein product [Mus musculus]
mmu-miR-10b-3p	NR_046275	-22.2	0.999926	-25.5	156	-	K09371|1|4e-06|55.1|bta:100337389|LIM homeobox transcription factor 1	gi|148676656|gb|EDL08603.1|	2.37E-74	hypothetical protein C130021I20 [Mus musculus]
mmu-miR-10b-3p	NM_147155	-23.3	0.977714	-20.39	155	GO:0044464,GO:0030695,GO:0019222,GO:0023052,	-	gi|148228285|ref|NP_671511.2|	0	T-cell activation GTPase-activating protein 1 [Mus musculus]
mmu-miR-10b-3p	NR_026565	-20.3	1	-20.15	154	GO:0000783,GO:0043566,GO:0006259,	-	gi|148678523|gb|EDL10470.1|	0	RIKEN cDNA 1500010J02, isoform CRA_b [Mus musculus]

mmu-miR-10b-3p	NM_177780	-20.6	1	-21.62	153	GO:0044424,GO:0032561,GO:0030695,GO:0019899,	K05727|1|0.0|3590|mmu:68813|dedicator of cytokinesis 3/4/5	gi|124358946|ref|NP_808448.2|	0	dedicator of cytokinesis protein 5 [Mus musculus]
mmu-miR-10b-3p	NR_045523	-22.6	0.995801	-22.02	152	GO:0031224,GO:0005764,GO:0031982,GO:0031090,GO:0046914,GO:0019787,GO:0016192,GO:0032446,	K10657|1|8e-136|485|mmu:224703|E3 ubiquitin-protein ligase MARCH2 [EC:6.3.2.19]	gi|31542053|ref|NP_663461.2|	5.68E-135	E3 ubiquitin-protein ligase MARCH2 isoform 1 [Mus musculus]
mmu-miR-10b-3p	NR_033503	-24.3	0.965727	-23.4	151	-	-	gi|149018180|gb|EDL76821.1|	5.08E-09	rCG62930 [Rattus norvegicus]
mmu-miR-10b-3p	NR_033582	-20.8	0.999985	-20.43	150	-	-	gi|148683718|gb|EDL15665.1|	9.37E-25	mCG1050993 [Mus musculus]
mmu-miR-10b-3p	NM_027830	-20.6	1	-20.74	149	GO:0016462,GO:0032559,GO:0006259,	K10738|1|0.0|2020|mmu:71567|minichromosome maintenance	gi|86439953|ref|NP_082106.2|	0	DNA replication licensing factor MCM9 [Mus musculus]

mmu-miR-10b-3p	NM_194054	-21.9	1	-21.09	148	GO:0032991,GO:0044297,GO:0042995,GO:0031227,GO:0005515,GO:0014046,GO:0048755,GO:0045596,GO:0032101,GO:0001837,GO:0007409,GO:0007029,GO:0010769,GO:0007169,GO:0051931,GO:0021545,GO:0014015,GO:0030308,GO:0021799,GO:0010001,GO:0010977,GO:00486	K00688|1|2e-20|103|ame:409267|starch phosphorylase [EC:2.4.1.1]!K09880|2|1e-09|67.4|aag:AaeL_AAEL000109|enolase-phosphatase E1 [EC:3.1.3.77]!K00907|4|2e-09|67.0|dmo:Dmoj_GI20420|myosin-light-chain kinase [EC:2.7.11.18]	gi|60360164|dbj|BAD90301.1|	0	mKIAA0886 protein [Mus musculus]

mmu-miR-10b-3p	NM_153424	-20.8	1	-21.91	147	GO:0005815,GO:0042995,GO:0005488,GO:0048856,GO:0007010,GO:0007155,GO:0023052,GO:0007610,	-	gi|124244073|ref|NP_700473.2|	0	nephrocystin-4 [Mus musculus]
mmu-miR-10b-3p	NR_033218	-20.5	1	-21.1	146	GO:0044451,GO:0003676,GO:0046914,GO:0007264,GO:0032501,GO:0006351,	K09228|1|7e-25|119|xla:397958|KRAB domain-containing zinc finger protein	gi|152060560|sp|Q3UH06.2|RREB1_MOUSE	0	RecName: Full=Ras-responsive element-binding protein 1; Short=RREB-1; AltName: Full=RAS-responsive zinc finger transcription factor RREB
mmu-miR-10b-3p	NR_030699	-21.3	1	-20.34	145	-	-	gi|124430547|ref|NP_001074446.1|	1.85E-43	round spermatid basic protein 1-like [Mus musculus]
mmu-miR-10b-3p	NM_080848	-21	0.999997	-21.81	142	GO:0035097,GO:0000123,GO:0042393,GO:0018024,GO:0048731,GO:0034968,GO:0006351,GO:0016573,	K14963|1|0.0|666|mcc:722123|COMPASS component SWD3	gi|6714707|emb|CAB66159.1|	0	hypothetical protein [Homo sapiens]
mmu-miR-10b-3p	NR_033623	-24.4	0.983342	-20.82	140	-	-	gi|28972824|dbj|BAC65828.1|	0	mKIAA1737 protein [Mus musculus]

mmu-miR-10b-3p	NM_011652	NA	NA	-41.43	307	-	K12567|1|0.0|4.370e+04|mmu:22138|titin [EC:2.7.11.	gi|77812697|ref|NP_035782.3|	0	titin isoform N2-A [Mus musculus]
mmu-miR-10b-3p	NM_027355	NA	NA	-22.13	159	GO:0043231,GO:0043234,GO:0046872,GO:0019787,GO:0032182,GO:0009314,GO:0006282,GO:0033522,	K12015|1|2e-06|56.6|dre:100137104|tripartite motif-containing protein 39	gi|148665366|gb|EDK97782.1|	0	ring finger protein 168, isoform CRA_b [Mus musculus]
mmu-miR-10b-3p	NM_199025	NA	NA	-24.91	153	GO:0043231,GO:0046914,GO:0003676,	K10505|1|0.0|886|rno:311910|zinc finger and BTB domain-containing protein	gi|50510995|dbj|BAD32483.1|	0	mKIAA1572 protein [Mus musculus]
mmu-miR-10b-3p	NM_009986	NA	NA	-24.36	149	GO:0031224,GO:0031981,GO:0000139,GO:0005488,GO:0006357,GO:0006891,GO:00485	K09313|1|0.0|2321|mmu:13047|homeobox protein cut-like	gi|110835729|ref|NP_034116.3|	0	protein CASP isoform a [Mus musculus]

mmu-miR-10b-3p	NM_016907	NA	NA	-23.08	146	GO:0005488,GO:0004866,GO:0043062,GO:0010466,GO:0048598,GO:0001701,GO:00015	K15619|1|0.0|960|mmu:20732|Kunitz-type protease inhibitor 1	gi|34328234|ref|NP_058603.2|	0	kunitz-type protease inhibitor 1 precursor [Mus musculus]
mmu-miR-10b-3p	NM_010347	NA	NA	-20.45	145	GO:0043231,GO:0005488,GO:0003712,GO:0048731,GO:0043393,GO:0030178,GO:0000122,GO:0034097,GO:0009653,	K04497|1|1e-43|177|dre:30206|groucho	gi|6754072|ref|NP_034477.1|	6.80E-83	amino-terminal enhancer of split [Mus musculus]
mmu-miR-10b-3p	NM_177715	NA	NA	-22.73	145	GO:0005887,GO:0005488,GO:0005267,GO:0034220,GO:0030001,	-	gi|123701966|ref|NP_808383.3|	3.56E-160	BTB/POZ domain-containing protein KCTD12 [Mus musculus]

mmu-miR-10b-3p	NM_008398	NA	NA	-20.27	144	GO:0005887,GO:0044424,GO:0004871,GO:0046983,GO:0048870,GO:0065008,GO:0031589,GO:0007166,GO:0009653,GO:00485	K06583|1|0.0|2160|mmu:16404|integrin alpha 7	gi|112293269|ref|NP_032424.2|	0	integrin alpha-7 [Mus musculus]
mmu-miR-10b-3p	NM_030075	NA	NA	-20.3	140	GO:0044424,	K10446|1|3e-19|98.2|cel:W02G9.2|kelch-like protein 8	gi|148689341|gb|EDL21288.1|	0	kelch domain containing 8B, isoform CRA_b [Mus musculus]

mmu-miR-10b-3p	NM_007553	-21.5	0.999889	NA	NA	GO:0032991,GO:0031982,GO:0044424,GO:0044421,GO:0070696,GO:0016616,GO:0017002,GO:0005126,GO:0008047,GO:0042802,GO:0046983,GO:0001935,GO:0030177,GO:0006915,GO:0030278,GO:0060415,GO:0007178,GO:0030282,GO:0043009,GO:0045687,GO:0006952,GO:00140	K04662|1|0.0|744|mmu:12156|bone morphogenetic protein 2/4	gi|71896669|ref|NP_031579.2|	0	bone morphogenetic protein 2 precursor [Mus musculus]

mmu-miR-10b-3p	NM_001163577	-28.4	0.480012	NA	NA	GO:0031513,GO:0044421,GO:0044459,GO:0005902,GO:0031988,GO:0016021,GO:0003407,GO:0001754,	K06532|1|0.0|1553|mmu:19126|prominin 1	gi|254675298|ref|NP_001157049.1|	0	prominin-1 isoform s2 precursor [Mus musculus]
mmu-miR-10b-3p	NM_009566	-26.9	0.848951	NA	NA	GO:0043231,GO:0043169,GO:0003676,GO:0006351,	K09228|1|0.0|993|mmu:22754|KRAB domain-containing zinc finger protein	gi|84370221|ref|NP_033592.2|	0	zinc finger protein 92 [Mus musculus]
mmu-miR-10b-3p	NR_046036	-21.5	0.939617	NA	NA	-	-	-	-	-
mmu-miR-10b-3p	NM_001163332	-23.3	0.999309	NA	NA	-	K06106|1|2e-10|70.1|api:100168801|cortactin!K10352|2|8e-10|67.8|dre:555454|myosin heavy chain	gi|37360382|dbj|BAC98169.1|	0	mKIAA1433 protein [Mus musculus]

mmu-miR-10b-3p	NM_181040	-20.1	1	NA	NA	GO:0043231,GO:0005488,GO:0065003,	K07555|1|2e-176|620|mmu:230649|ATP synthase mitochondrial F1 complex assembly factor 1	gi|344925843|ref|NP_851383.2|	4.24E-177	ATP synthase mitochondrial F1 complex assembly factor 1 [Mus musculus]
mmu-miR-10b-3p	NM_030197	-23.6	0.999439	NA	NA	GO:0000151,GO:0019899,GO:0019941,	K03869|1|4e-07|59.3|hmg:100202538|cullin 3	gi|58037533|ref|NP_084473.1|	1.38E-161	uncharacterized protein C10orf46 homolog isoform 1 [Mus musculus]
mmu-miR-10b-3p	NM_201639	-20.5	1	NA	NA	GO:0043234,GO:0045111,GO:0030016,GO:0070161,GO:0008092,GO:0032403,GO:0005198,GO:0007010,	K10376|1|0.0|2403|rno:308709|desmuslin	gi|153791809|ref|NP_964001.2|	0	synemin isoform H [Mus musculus]
mmu-miR-10b-3p	NR_028422	-22.4	0.99059	NA	NA	-	-	-	-	-

mmu-miR-10b-3p	NM_001163449	-21.5	1	NA	NA	GO:0031982,GO:0032589,GO:0012505,GO:0005783,GO:0016772,GO:0008092,GO:0019900,GO:0032947,GO:0033036,GO:0006810,GO:0010467,GO:0045597,GO:0043408,GO:0043009,GO:0007420,GO:0006935,GO:0009887,GO:0000187,	K04436|1|0.0|2410|mmu:30957|mitogen-activated protein kinase 8 interacting protein 3	gi|254540202|ref|NP_001156921.1|	0	C-Jun-amino-terminal kinase-interacting protein 3 isoform d [Mus musculus]

mmu-miR-10b-3p	NM_001166437	-20.5	0.999999	NA	NA	GO:0044464,GO:0005099,GO:0016769,GO:0032313,	K13136|1|1e-48|195|nvi:100114269|gem associated protein 8!K12813|2|3e-16|88.2|nvi:100117259|pre-mRNA-splicing factor ATP-dependent RNA helicase DHX16 [EC:3.6.4.13]!K11452|3|2e-09|65.9|tgu:100222038|chromobox protein 4	gi|261878622|ref|NP_001159909.1|	0	TBC1 domain family member 25 isoform 2 [Mus musculus]
mmu-miR-10b-3p	NM_133734	-22.4	0.997626	NA	NA	GO:0031461,GO:0005488,GO:0032446,	K11801|1|0.0|1018|mmu:28199|WD repeat-containing protein 23	gi|19526930|ref|NP_598495.1|	0	DDB1- and CUL4-associated factor 11 [Mus musculus]

mmu-miR-10b-3p	NM_001163503	-21.3	0.999885	NA	NA	GO:0031224,GO:0005886,GO:0015291,GO:0030001,GO:0008645,	K08175|1|0.0|692|rno:499462|MFS transporter, FHS family, Na+ dependent glucose transporter 1	gi|392338456|ref|XP_001062835.3|	0	PREDICTED: sodium-dependent glucose transporter 1 [Rattus norvegicus]
mmu-miR-10b-3p	NM_177461	-21.3	1	NA	NA	GO:0044424,GO:0046872,	K05699|1|4e-26|122|nve:NEMVE_v1g206855|actinin alpha	gi|168229165|ref|NP_803412.1|	0	MICAL-like protein 1 [Mus musculus]
mmu-miR-10b-3p	NM_001199060	-21	0.999999	NA	NA	GO:0043234,GO:0031981,GO:0030684,GO:0005488,GO:0000470,GO:0000460,GO:00071	K14863|1|0.0|814|mmu:57750|ribosome biogenesis protein YTM1	gi|10946614|ref|NP_067287.1|	0	ribosome biogenesis protein WDR12 [Mus musculus]

mmu-miR-10b-3p	NM_011394	-22.4	0.999779	NA	NA	GO:0016021,GO:0015081,GO:0004871,GO:0015114,GO:0015291,GO:0051704,GO:0006817,GO:0030001,GO:0023052,	K14640|1|0.0|1172|mmu:20516|solute carrier family 20 (sodium-dependent phosphate transporter)	gi|165377214|ref|NP_035524.2|	0	sodium-dependent phosphate transporter 2 precursor [Mus musculus]
mmu-miR-10b-3p	NM_001079905	-20.7	1	NA	NA	GO:0043231,GO:0000808,GO:0046914,GO:0003676,GO:0006259,	K09228|1|3e-105|384|pon:100459001|KRAB domain-containing zinc finger protein	gi|347658946|ref|NP_001073373.2|	0	replication initiator 1 isoform d [Mus musculus]
mmu-miR-10b-3p	NM_025337	-21.9	0.993887	NA	NA	GO:0044431,GO:0004033,GO:0044238,GO:0044237,	K15303|1|0.0|640|mmu:110198|aflatoxin B1 aldehyde reductase	gi|240120054|ref|NP_079613.3|	0	aflatoxin B1 aldehyde reductase member 2 [Mus musculus]
mmu-miR-10b-3p	NM_001166583	-22.8	0.998725	NA	NA	-	-	gi|148697178|gb|EDL29125.1|	1.13E-128	RIKEN cDNA 4632404H22, isoform CRA_b [Mus musculus]

mmu-miR-10b-3p	NM_009501	-23.5	0.761949	NA	NA	GO:0043231,GO:0001071,GO:0003677,GO:0003682,GO:0016477,GO:0007417,GO:0001654,GO:0006357,GO:0006935,GO:00074	K09318|1|3e-132|471|mmu:22326|homeobox protein ventral anterior	gi|6678557|ref|NP_033527.1|	2.01E-131	ventral anterior homeobox 1 [Mus musculus]
mmu-miR-10b-3p	NM_001039090	-21.6	1	NA	NA	GO:0016604,GO:0030141,GO:0005515,GO:0003712,GO:0003676,GO:0051291,GO:0000122,GO:0007179,GO:0014706,GO:0010033,GO:0031346,GO:0001824,GO:0022402,GO:0006915,GO:0045595,GO:0048232,GO:0051260,GO:0001776,	-	gi|84875520|ref|NP_001034179.1|	0	ski-like protein isoform b [Mus musculus]

mmu-miR-10b-3p	NM_026681	-21.2	1	NA	NA	-	K08408|1|0.0|2186|cfa:480418|G protein-coupled receptor 68!K10352|2|2e-25|120|cin:100176037|myosin heavy chain	gi|148686937|gb|EDL18884.1|	0	RIKEN cDNA 0610010D24, isoform CRA_b [Mus musculus]
mmu-miR-10b-3p	NM_001252520	-21.8	0.999995	NA	NA	GO:0044430,GO:0005905,GO:0030128,GO:0035091,GO:0030276,GO:0019904,GO:0048812,GO:0006901,GO:0006898,GO:0048534,GO:0045806,GO:0048667,GO:00081	-	gi|148674811|gb|EDL06758.1|	0	phosphatidylinositol binding clathrin assembly protein, isoform CRA_d [Mus musculus]

mmu-miR-10b-3p	NM_022327	-21.7	0.999501	NA	NA	GO:0016020,GO:0044444,GO:0032561,GO:0017111,GO:0007010,GO:0012501,GO:0009207,GO:0007169,GO:0007264,GO:0032880,GO:0001927,GO:0050793,	K07835|1|9e-107|388|mmu:64143|Ras-related protein Ral-B	gi|11612509|ref|NP_071722.1|	6.56E-106	ras-related protein Ral-B precursor [Mus musculus]
mmu-miR-10b-3p	NM_029974	-23.3	0.97606	NA	NA	GO:0016020,GO:0046872,	-	gi|116812883|ref|NP_084250.1|	0	DC-STAMP domain-containing protein 1 [Mus musculus]
mmu-miR-10b-3p	NM_177652	-21.7	1	NA	NA	GO:0031224,GO:0016528,GO:0005789,GO:0005218,GO:0004871,GO:0046872,GO:0023052,GO:0006875,GO:0006816,	K04963|1|0.0|9070|mmu:20192|ryanodine receptor 3	gi|125628627|ref|NP_808320.2|	0	ryanodine receptor 3 [Mus musculus]

mmu-miR-10b-3p	NM_008816	-25.6	0.852949	NA	NA	GO:0031224,GO:0030667,GO:0044421,GO:0044459,GO:0043292,GO:0005488,GO:0042506,GO:0007265,GO:0009611,GO:0007599,GO:0010033,GO:0050900,GO:0006887,GO:0006897,GO:0001775,	K06471|1|0.0|1300|mmu:18613|platelet/endothelial cell adhesion molecule	gi|74024907|ref|NP_032842.2|	0	platelet endothelial cell adhesion molecule isoform 1 precursor [Mus musculus]

mmu-miR-10b-3p	NM_001256082	-24.5	0.997962	NA	NA	GO:0031513,GO:0016461,GO:0005902,GO:0005764,GO:0048770,GO:0044459,GO:0008092,GO:0042802,GO:0032559,GO:0003774,GO:0042623,GO:0051648,GO:0030029,GO:0007033,GO:0050953,GO:0006906,GO:0009207,GO:0050954,GO:0060122,GO:0006897,GO:0042471,GO:00017	K10359|1|0.0|4131|mmu:17921|myosin VII	gi|367460066|ref|NP_001243011.1|	0	unconventional myosin-VIIa isoform 3 [Mus musculus]
mmu-miR-10b-3p	NM_001162928	-23	0.999696	NA	NA	-	K14316|1|0.0|1705|mmu:195236|nuclear pore complex protein Nup121	gi|242397452|ref|NP_001156400.1|	0	POM121-like protein 2 isoform 1 [Mus musculus]

mmu-miR-10b-3p	NM_178081	-24	0.979603	NA	NA	GO:0031224,GO:0004871,GO:0023052,GO:0006810,	-	gi|30017395|ref|NP_835182.1|	0	major facilitator superfamily domain-containing protein 6 isoform 2 [Mus musculus]
mmu-miR-10b-3p	NM_172695	-21.5	1	NA	NA	GO:0016004,GO:0006952,GO:0023052,GO:0019637,	K14018|1|0.0|1590|mmu:18786|phospholipase A-2-activating protein	gi|148698991|gb|EDL30938.1|	0	phospholipase A2, activating protein, isoform CRA_c [Mus musculus]
mmu-miR-10b-3p	NM_001164376	-23.2	0.997508	NA	NA	GO:0005856,GO:0031252,GO:0005913,GO:0003676,GO:0050839,GO:0006260,GO:0007155,	K05691|1|0.0|1588|mmu:216033|catenin (cadherin-associated protein),	gi|256985119|ref|NP_808280.2|	0	catenin alpha-3 isoform 1 [Mus musculus]

mmu-miR-10b-3p	NM_028817	-20.4	1	NA	NA	GO:0031224,GO:0031966,GO:0044439,GO:0016405,GO:0015645,GO:0032559,GO:0016408,GO:0007417,GO:0006631,GO:0035336,GO:0031667,GO:0006641,GO:00100	K01897|1|0.0|1416|mmu:74205|long-chain acyl-CoA synthetase [EC:6.2.1.3]	gi|75992920|ref|NP_083093.2|	0	long-chain-fatty-acid--CoA ligase 3 isoform a [Mus musculus]
mmu-miR-10b-3p	NM_001177617	-20.1	1	NA	NA	GO:0044424,GO:0009408,	K02360|1|3e-19|98.2|aag:AaeL_AAEL009189|a component of the cytoplasm (encore)	gi|294979163|ref|NP_001171088.1|	0	cAMP-regulated phosphoprotein 21 isoform 5 [Mus musculus]
mmu-miR-10b-3p	NM_001170788	-20	1	NA	NA	-	K11092|1|8e-06|53.5|bfo:BRAFLDRAFT_115963|U2 small nuclear ribonucleoprotein A'	gi|282847355|ref|NP_001164259.1|	0	leucine-rich repeat-containing protein 36 isoform 1 [Mus musculus]

mmu-miR-10b-3p	NM_172653	-21.7	1	NA	NA	GO:0031224,GO:0008324,GO:0000041,	K14716|1|0.0|1384|mmu:227059|solute carrier family 39 (zinc transporter), member 10	gi|40254228|ref|NP_766241.2|	0	zinc transporter ZIP10 precursor [Mus musculus]
mmu-miR-10b-3p	NM_172990	-21.7	0.999796	NA	NA	GO:0044424,GO:0016301,GO:0032559,GO:0009108,GO:0006796,	K09680|1|0.0|1475|mmu:269614|type II pantothenate kinase [EC:2.7.1.33]	gi|240255614|ref|NP_766578.2|	0	pantothenate kinase 4 [Mus musculus]
mmu-miR-10b-3p	NM_177708	-24.6	0.948395	NA	NA	GO:0009986,GO:0031225,GO:0004871,GO:0007409,GO:0007165,	K08129|1|1e-22|110|dre:568821|nyctalopin!K07523|2|8e-22|107|xtr:100188921|netrin-G1 ligand	gi|29244160|ref|NP_808376.1|	0	reticulon-4 receptor-like 1 precursor [Mus musculus]
mmu-miR-10b-3p	NM_001097979	-23.8	0.935186	NA	NA	GO:0000785,GO:0043231,GO:0003676,GO:0034728,	K11252|1|5e-48|193|mmu:665622|histone H2B	gi|160420310|ref|NP_001091448.2|	3.69E-47	H2b histone family, member A [Mus musculus]

mmu-miR-10b-3p	NM_170673	-25.6	0.79991	NA	NA	-	K06491|1|2e-10|68.9|cin:100183448|neural cell adhesion molecule!K15290|2|1e-07|60.1|cel:F31E8.2|synaptotagmin-1!K02677|3|1e-06|56.6|xtr:100496779|classical protein kinase C [EC:2.7.11.13]	gi|123778910|sp|Q1RLL3.1|CPNE9_MOUSE	0	RecName: Full=Copine-9; AltName: Full=Copine IX
mmu-miR-10b-3p	NM_011218	-20.8	1	NA	NA	GO:0016021,GO:0005488,GO:0004871,GO:0004725,GO:0022037,GO:0006470,GO:0021543,GO:0023052,	K06778|1|0.0|3687|mmu:19280|protein tyrosine phosphatase, receptor type, S [EC:3.1.3.48]	gi|158711690|ref|NP_035348.2|	0	receptor-type tyrosine-protein phosphatase S isoform 1 precursor [Mus musculus]
mmu-miR-10b-5p	NM_001008973	-29.6	0.272045	-26.58	170	GO:0031224,	-	gi|113374162|ref|NP_001008973.2|	0	transmembrane protein 232 [Mus musculus]

mmu-miR-10b-5p	NM_001024205	-27.7	0.937581	-25.22	173	GO:0005840,GO:0043231,GO:0003676,	-	gi|28972720|dbj|BAC65776.1|	0	mKIAA1321 protein [Mus musculus]
mmu-miR-10b-5p	NM_001039194	-25.7	0.875613	-26.05	164	GO:0031224,GO:0031966,GO:0003676,GO:0016649,GO:0050662,GO:0012502,GO:0006915,GO:0051276,GO:0008152,	K04727|1|2e-08|62.4|nve:NEMVE_v1g79630|programmed cell death 8 (apoptosis-inducing factor) [EC:1.-.-.-]	gi|30017355|ref|NP_835159.1|	0	apoptosis-inducing factor 2 isoform 2 [Mus musculus]
mmu-miR-10b-5p	NM_001080972	-29.1	0.095702	-29.11	169	GO:0016021,GO:0005549,GO:0004930,GO:0023052,GO:0010033,	K04614|1|3e-170|597|mmu:626299|vomeronasal1 receptor	gi|124430498|ref|NP_001074441.1|	1.97E-169	vomeronasal 1 receptor 194 [Mus musculus]
mmu-miR-10b-5p	NM_001103177	-31.3	0.338586	-29.67	171	GO:0005856,GO:0008092,GO:0046914,GO:0006996,GO:0006357,GO:0050953,GO:0006935,GO:0009653,	K07520|1|0.0|1363|mmu:226251|actin-binding LIM protein	gi|157057174|ref|NP_001096647.1|	0	actin-binding LIM protein 1 isoform 2 [Mus musculus]

mmu-miR-10b-5p	NM_001103178	-31.3	0.322905	-29.67	171	GO:0005856,GO:0008092,GO:0046914,GO:0006996,GO:0006357,GO:0006935,	K07520|1|0.0|951|ptr:450753|actin-binding LIM protein	gi|157057145|ref|NP_848803.3|	0	actin-binding LIM protein 1 isoform 1 [Mus musculus]
mmu-miR-10b-5p	NM_001145820	-25.5	0.980748	-25.08	157	GO:0043231,GO:0043234,GO:0019866,GO:0052590,GO:0046872,GO:0016616,GO:0006629,GO:0006071,GO:0019674,GO:00060	K00111|1|0.0|1356|mmu:14571|glycerol-3-phosphate dehydrogenase [EC:1.1.5.3]	gi|224922803|ref|NP_034404.3|	0	glycerol-3-phosphate dehydrogenase, mitochondrial precursor [Mus musculus]
mmu-miR-10b-5p	NM_001145977	-29.4	0.736005	-25.67	181	GO:0043005,GO:0031224,GO:0043025,GO:0044424,GO:0005488,GO:0007417,GO:00452	K06782|1|0.0|764|rno:360687|immunoglobulin superfamily, member 4D	gi|114052913|ref|NP_001040567.1|	0	cell adhesion molecule 2 precursor [Rattus norvegicus]
mmu-miR-10b-5p	NM_001177713	-26.2	0.915884	-25.15	162	-	K12664|1|0.0|989|mmu:232174|cytochrome P450, family 26, subfamily B	gi|148666702|gb|EDK99118.1|	0	cytochrome P450, family 26, subfamily b, polypeptide 1, isoform CRA_b [Mus musculus]

mmu-miR-10b-5p	NM_001252382	-25	0.86797	-25.08	156	GO:0043231,GO:0003676,GO:0001071,GO:0019221,GO:0006351,	K09446|1|0.0|933|mmu:27056|interferon regulatory factor 5	gi|356991180|ref|NP_001239311.1|	0	interferon regulatory factor 5 isoform 1 [Mus musculus]
mmu-miR-10b-5p	NM_001252572	-28.9	0.40821	-31.12	158	GO:0043232,GO:0031252,GO:0044444,GO:0005515,GO:0007417,GO:0006897,	K06106|1|0.0|937|mmu:13043|cortactin	gi|357588432|ref|NP_001239501.1|	0	src substrate cortactin isoform 2 [Mus musculus]
mmu-miR-10b-5p	NM_001254951	-26.5	0.948345	-27.35	164	-	K09228|1|0.0|803|aml:100473713|KRAB domain-containing zinc finger	gi|363807379|ref|NP_001241880.1|	0	zinc finger protein 850 [Mus musculus]
mmu-miR-10b-5p	NM_007803	-28.9	0.420298	-31.12	158	GO:0043232,GO:0031252,GO:0044444,GO:0005515,GO:0006897,	K06106|1|0.0|1030|mmu:13043|cortactin	gi|75677414|ref|NP_031829.2|	0	src substrate cortactin isoform 1 [Mus musculus]
mmu-miR-10b-5p	NM_008751	-27	0.475418	-27.58	168	GO:0005515,	-	gi|148681975|gb|EDL13922.1|	4.77E-170	neurexophilin 1, isoform CRA_a [Mus musculus]

mmu-miR-10b-5p	NM_010274	-25.5	0.979238	-25.08	157	GO:0043231,GO:0043234,GO:0019866,GO:0052590,GO:0046872,GO:0016616,GO:0006629,GO:0006071,GO:0019674,GO:00060	K00111|1|0.0|1356|mmu:14571|glycerol-3-phosphate dehydrogenase [EC:1.1.5.3]	gi|224922803|ref|NP_034404.3|	0	glycerol-3-phosphate dehydrogenase, mitochondrial precursor [Mus musculus]
mmu-miR-10b-5p	NM_010696	-31.7	0.159032	-30.61	182	-	K07361|1|0.0|904|mmu:16822|lymphocyte cytosolic protein 2	gi|31543115|ref|NP_034826.2|	0	lymphocyte cytosolic protein 2 [Mus musculus]
mmu-miR-10b-5p	NM_011076	-25.7	0.959417	-25.53	156	GO:0005911,GO:0031224,GO:0012505,GO:0032559,GO:0042626,GO:0048511,GO:0007589,GO:0010033,GO:0015893,GO:0010941,GO:0009207,GO:0033273,GO:00100	K05658|1|0.0|2296|mmu:18671|ATP-binding cassette, subfamily B (MDR/TAP), member 1	gi|225734206|pdb|3G60|A	0	Chain A, Structure Of P-Glycoprotein Reveals A Molecular Basis For Poly-Specific Drug Binding

mmu-miR-10b-5p	NM_012057	-25	0.861574	-25.08	156	GO:0043231,GO:0003676,GO:0001071,GO:0019221,GO:0006351,	K09446|1|0.0|933|mmu:27056|interferon regulatory factor 5	gi|356991180|ref|NP_001239311.1|	0	interferon regulatory factor 5 isoform 1 [Mus musculus]
mmu-miR-10b-5p	NM_019403	-27.4	0.259216	-25.1	160	GO:0031224,GO:0031090,GO:0005740,GO:0046914,GO:0019787,GO:0000209,GO:0006515,	K10666|1|7e-85|314|rno:407784|E3 ubiquitin-protein ligase RNF5 [EC:6.3.2.19]	gi|9507059|ref|NP_062276.1|	4.61E-84	E3 ubiquitin-protein ligase RNF5 [Mus musculus]
mmu-miR-10b-5p	NM_021882	-26.1	0.690575	-27.21	165	GO:0005771,GO:0031090,GO:0031224,GO:0048770,GO:0005488,GO:0006582,GO:00487	-	gi|31981217|ref|NP_068682.2|	0	melanocyte protein PMEL precursor [Mus musculus]
mmu-miR-10b-5p	NM_022889	-27.8	0.542276	-26.25	167	GO:0043234,GO:0031981,GO:0005694,GO:0030684,GO:0005488,GO:0000470,GO:0007067,GO:0006997,GO:0000460,	K14843|1|0.0|1055|mmu:64934|pescadillo	gi|12584984|ref|NP_075027.1|	0	pescadillo homolog [Mus musculus]

mmu-miR-10b-5p	NM_025659	-28.4	0.575233	-28.67	176	GO:0031252,GO:0044424,GO:0043169,GO:0016791,GO:0016477,GO:0006468,	K06124|1|7e-153|542|rno:287644|phosphoethanolamine/phosphocholine phosphatase [EC:3.1.3.7	gi|157786864|ref|NP_001099303.1|	5.15E-152	phosphoethanolamine/phosphocholine phosphatase [Rattus norvegicus]
mmu-miR-10b-5p	NM_028860	-25.7	0.973017	-25.81	165	GO:0031981,GO:0046914,GO:0004721,GO:0006464,GO:0006796,	K01104|1|0.0|2264|mmu:74302|protein-tyrosine phosphatase [EC:3.1.3.48]	gi|145207976|ref|NP_083136.2|	0	myotubularin-related protein 3 [Mus musculus]
mmu-miR-10b-5p	NM_030709	-29.2	0.250092	-25.77	157	GO:0031224,GO:0004175,GO:0004888,GO:0019538,GO:0023052,	K09636|1|0.0|898|mmu:80893|transmembrane protease, serine 5 (spinesin) [EC:3.4.21.-]	gi|148693772|gb|EDL25719.1|	0	transmembrane protease, serine 5 (spinesin), isoform CRA_a [Mus musculus]

mmu-miR-10b-5p	NM_146033	-27	0.601948	-25.24	154	GO:0042995,GO:0046914,	K15504|1|4e-12|74.3|dre:553206|serine/threonine-protein phosphatase 6 regulatory ankyrin repeat subunit C	gi|148704904|gb|EDL36851.1|	0	ankyrin repeat and MYND domain containing 2, isoform CRA_a [Mus musculus]
mmu-miR-10b-5p	NM_146152	-28.7	0.486562	-25.06	174	GO:0031074,GO:0043231,GO:0035258,GO:0022892,GO:0002146,	K15436|1|0.0|1522|tgu:100222227|transportin-3	gi|39104529|dbj|BAC98010.2|	0	mKIAA0724 protein [Mus musculus]
mmu-miR-10b-5p	NM_153779	-25.7	0.572152	-26.05	164	GO:0031224,GO:0031966,GO:0003676,GO:0016649,GO:0050662,GO:0012502,GO:0006915,GO:0051276,GO:0008152,	K04727|1|8e-09|62.4|nve:NEMVE_v1g79630|programmed cell death 8 (apoptosis-inducing factor) [EC:1.-.-.-]	gi|262050654|ref|NP_722474.2|	0	apoptosis-inducing factor 2 isoform 1 [Mus musculus]

mmu-miR-10b-5p	NM_172898	-27.9	0.553784	-26.55	166	GO:0031224,GO:0009987,	K14319|1|2e-28|129|bfo:BRAFLDRAFT_126584|Ran GTPase-activating protein 1!K05639|2|5e-27|124|cfa:476478|amyloid beta (A4) precursor-like protein 1!K06755|3|2e-25|119|mcc:716274|roundabout, axon guidance receptor 3	gi|148692073|gb|EDL24020.1|	0	kin of IRRE like 2 (Drosophila), isoform CRA_a [Mus musculus]
mmu-miR-10b-5p	NM_175439	-27.3	0.616582	-26.69	164	GO:0044429,GO:0004812,GO:0032559,GO:0006418,	K01874|1|0.0|1211|mmu:212679|methionyl-tRNA synthetase [EC:6.1.1.10]	gi|26335385|dbj|BAC31393.1|	0	unnamed protein product [Mus musculus]

mmu-miR-10b-5p	NM_175475	-26.2	0.917959	-25.15	162	-	K12664|1|0.0|989|mmu:232174|cytochrome P450, family 26, subfamily B	gi|148666702|gb|EDK99118.1|	0	cytochrome P450, family 26, subfamily b, polypeptide 1, isoform CRA_b [Mus musculus]
mmu-miR-10b-5p	NM_177066	-30.4	0.235989	-26.69	179	GO:0043231,GO:0043169,GO:0008092,GO:0004672,GO:0032559,GO:0006464,GO:0006796,	K15502|1|6e-41|170|aag:AaeL_AAEL014742|serine/threonine-protein phosphatase 6 regulatory ankyrin repeat subunit A	gi|260166668|ref|NP_796040.3|	0	serine/threonine-protein kinase TNNI3K [Mus musculus]
mmu-miR-10b-5p	NM_177856	-26.8	0.449445	-26.14	159	GO:0031224,GO:0016787,GO:0005488,	-	gi|29244450|ref|NP_808524.1|	1.07E-167	uncharacterized protein C3orf33 homolog [Mus musculus]

mmu-miR-10b-5p	NM_178058	-25.7	0.612839	-26.05	164	GO:0031224,GO:0031966,GO:0003676,GO:0016649,GO:0050662,GO:0012502,GO:0006915,GO:0051276,GO:0008152,	K04727|1|9e-09|62.4|nve:NEMVE_v1g79630|programmed cell death 8 (apoptosis-inducing factor) [EC:1.-.-.-]	gi|30017355|ref|NP_835159.1|	0	apoptosis-inducing factor 2 isoform 2 [Mus musculus]
mmu-miR-10b-5p	NM_178688	-31.3	0.355744	-29.67	171	GO:0005856,GO:0008092,GO:0046914,GO:0006996,GO:0006357,GO:0006935,	K07520|1|0.0|1427|cfa:477828|actin-binding LIM protein	gi|157057145|ref|NP_848803.3|	0	actin-binding LIM protein 1 isoform 1 [Mus musculus]
mmu-miR-10b-5p	NM_178721	-29.4	0.736925	-25.67	181	GO:0043005,GO:0031224,GO:0043025,GO:0044424,GO:0005488,GO:0007417,GO:00452	K06782|1|0.0|757|rno:360687|immunoglobulin superfamily, member 4D	gi|225543375|ref|NP_848836.2|	0	cell adhesion molecule 2 isoform 1 precursor [Mus musculus]
mmu-miR-10b-5p	NM_197990	-28.8	0.820723	-44.08	317	-	-	gi|37574070|ref|NP_932107.1|	3.28E-62	uncharacterized protein C1orf21 homolog [Mus musculus]

mmu-miR-10b-5p	NM_198625	-27.3	0.796665	-46.2	314	GO:0030674,GO:0008092,GO:0019904,GO:0030035,GO:0023052,	K05627|1|2e-10|70.1|xtr:100158528|BAI1-associated protein 2	gi|38348492|ref|NP_941027.1|	0	MTSS1-like protein [Mus musculus]
mmu-miR-10b-5p	NM_206924	-34.3	0.011603	-31.09	181	GO:0016021,	-	gi|74226894|dbj|BAE27091.1|	2.73E-60	unnamed protein product [Mus musculus]
mmu-miR-10b-5p	NR_024328	-28.8	0.294583	-47.13	303	-	-	gi|148707601|gb|EDL39548.1|	7.88E-26	mCG148400 [Mus musculus]
mmu-miR-10b-5p	NR_033727	-28.1	0.088501	-26.29	171	GO:0043231,GO:0015935,GO:0003676,GO:0046914,GO:0005198,GO:0006412,GO:0019080,GO:0031016,GO:0007067,	K02978|1|2e-36|152|mdo:100020225|small subunit ribosomal protein S27e	gi|126307742|ref|XP_001372806.1|	9.90E-36	PREDICTED: 40S ribosomal protein S27-like [Monodelphis domestica]
mmu-miR-10b-5p	NR_040356	-28.9	0.13729	-28.65	160	-	-	gi|148694981|gb|EDL26928.1|	7.71E-23	mCG1040446 [Mus musculus]
mmu-miR-10b-5p	NR_045820	-27.6	0.412927	-25.29	163	-	-	gi|148687710|gb|EDL19657.1|	3.76E-65	mCG1030587 [Mus musculus]
mmu-miR-10b-5p	NR_033657	-21.8	1	-64.81	445	-	-	gi|148688037|gb|EDL19984.1|	6.79E-121	mCG128631, isoform CRA_c [Mus musculus]

mmu-miR-10b-5p	NM_053257	-24.3	0.704885	-46.06	328	-	K02910|1|1e-58|227|aml:100476308|large subunit ribosomal protein L31e	gi|148672373|gb|EDL04320.1|	2.54E-68	mCG49427 [Mus musculus]
mmu-miR-10b-5p	NM_027917	-24.4	0.977372	-44.6	319	GO:0016459,GO:0015630,GO:0003779,GO:0007015,GO:0032989,	-	gi|125628648|ref|NP_082193.2|	0	protein Shroom1 [Mus musculus]
mmu-miR-10b-5p	NM_019483	-24.8	0.992906	-46.65	315	GO:0043234,GO:0031981,GO:0044444,GO:0005488,GO:0001071,GO:0005072,GO:0035295,GO:0045595,GO:0006355,GO:0007420,GO:0014070,GO:0003006,GO:0007178,GO:0006950,GO:0006464,	K04676|1|0.0|873|mmu:55994|mothers against decapentaplegic homolog 1/5/8	gi|84490384|ref|NP_062356.3|	0	mothers against decapentaplegic homolog 9 [Mus musculus]

mmu-miR-10b-5p	NM_001081064	-22.1	1	-43.85	310	GO:0030054,GO:0043231,GO:0044430,GO:0007165,	K06095|1|5e-31|139|gga:427360|multiple PDZ domain protein	gi|124486716|ref|NP_001074533.1|	0	PDZ domain-containing protein 2 [Mus musculus]
mmu-miR-10b-5p	NM_001081417	-23.3	0.999999	-42.37	307	GO:0043231,GO:0044427,GO:0017111,GO:0003676,GO:0032559,GO:0006351,GO:0090276,GO:0007507,GO:0001944,GO:0030326,GO:0043009,GO:0050954,GO:0001654,GO:0021675,GO:0042471,GO:0046545,GO:0030098,GO:0035264,GO:0003013,GO:0006325,GO	K14437|1|0.0|3897|mmu:320790|chromodomain-helicase-DNA-binding protein 7 [EC:3.6.4.12]	gi|124487249|ref|NP_001074886.1|	0	chromodomain-helicase-DNA-binding protein 7 [Mus musculus]
mmu-miR-10b-5p	NM_146091	-23.4	0.999957	-40.96	298	-	-	gi|148701364|gb|EDL33311.1|	0	RIKEN cDNA 5730596K20 [Mus musculus]

mmu-miR-10b-5p	NR_015555	-23.1	0.999564	-42.66	297	-	-	gi|148687353|gb|EDL19300.1|	5.04E-59	mCG18013, isoform CRA_a [Mus musculus]
mmu-miR-10b-5p	NM_025989	-20.5	0.999998	-42.42	296	GO:0044424,GO:0031224,	K10307|1|8e-25|116|acs:100552900|F-box protein 30!K13912|2|3e-12|74.7|tgu:100226373|deleted in malignant brain tumors 1 protein	gi|27229028|ref|NP_080265.2|	0	pancreatic secretory granule membrane major glycoprotein GP2 precursor [Mus musculus]
mmu-miR-10b-5p	NM_133710	-23.5	0.999408	-40.4	294	GO:0043231,GO:0016791,GO:0043169,GO:0006796,	K15731|1|2e-158|561|mmu:69274|carboxy-terminal domain RNA polymerase II polypeptide A small phosphatase [EC:3.1.3.1	gi|148677300|gb|EDL09247.1|	1.62E-157	CTD (carboxy-terminal domain, RNA polymerase II, polypeptide A) small phosphatase-like [Mus musculus]

mmu-miR-10b-5p	NM_001126047	-23.1	0.999413	-44.76	288	GO:0031224,GO:0008021,GO:0044459,GO:0044430,GO:0005488,GO:0004871,GO:0048731,GO:0032872,GO:0030154,	K06521|1|0.0|1541|mmu:20353|semaphorin 4	gi|148682548|gb|EDL14495.1|	0	mCG17761, isoform CRA_b [Mus musculus]
mmu-miR-10b-5p	NM_007539	-20.8	0.99969	-41.2	284	GO:0043231,GO:0016021,GO:0042995,GO:0008528,GO:0010524,GO:0001932,GO:0007186,GO:0007600,GO:0016049,GO:0002237,GO:0002684,GO:0006952,GO:00082	K03915|1|1e-165|583|mmu:12061|bradykinin receptor B1	gi|4097022|gb|AAD00029.1|	4.53E-165	bradykinin B1 receptor [Mus musculus]
mmu-miR-10b-5p	NM_028762	-21.8	0.999998	-41.67	283	GO:0043232,GO:0031981,GO:0003676,GO:0009790,	K14787|1|0.0|1643|mmu:74111|multiple RNA-binding domain-containing protein 1	gi|30794154|ref|NP_083038.1|	0	probable RNA-binding protein 19 [Mus musculus]

mmu-miR-10b-5p	NM_175111	-26.4	0.893207	-22.48	176	GO:0044424,	K00476|1|5e-14|81.6|dre:373126|aspartate beta-hydroxylase [EC:1.14.11.16]	gi|120587017|ref|NP_780320.2|	0	HSPB1-associated protein 1 [Mus musculus]
mmu-miR-10b-5p	NM_172473	-20.6	1	-22.45	175	GO:0043231,GO:0019787,GO:0016567,	K12166|1|0.0|1838|mmu:209462|E3 ubiquitin-protein ligase HACE1 [EC:6.3.2.19]	gi|283436216|ref|NP_766061.2|	0	E3 ubiquitin-protein ligase HACE1 [Mus musculus]
mmu-miR-10b-5p	NM_178660	-27	0.942886	-22.93	171	GO:0044424,GO:0003676,	K13208|1|2e-20|103|dre:431719|ELAV like protein 2/3/4	gi|148747313|ref|NP_848775.2|	0	RNA-binding motif, single-stranded-interacting protein 3 isoform 3 [Mus musculus]

mmu-miR-10b-5p	NM_007983	-27	0.815437	-23.89	170	GO:0043231,GO:0012505,GO:0031264,GO:0044444,GO:0019207,GO:0008134,GO:0019900,GO:0007155,GO:0042994,GO:0006461,GO:0009894,GO:0006511,GO:00069	K04649|1|3e-13|79.3|cel:C28G1.1|ubiquitin-conjugating enzyme (huntingtin interacting protein 2) [EC:6.3.2.19]	gi|40789280|ref|NP_032009.2|	0	FAS-associated factor 1 [Mus musculus]
mmu-miR-10b-5p	NM_177084	-25.3	0.9486	-22.76	169	GO:0044459,GO:0031224,GO:0005451,GO:0055067,GO:0006818,GO:0035725,	K13961|1|0.0|1434|mmu:110895|solute carrier family 9 (sodium/hydrogen exchanger), member 4	gi|45238583|ref|NP_796058.1|	0	sodium/hydrogen exchanger 4 precursor [Mus musculus]
mmu-miR-10b-5p	NR_028297	-27.3	0.471715	-24.53	168	GO:0005634,GO:0005488,	K10892|1|0.0|479|mmu:72775|fanconi anemia group E protein	gi|255652993|ref|NP_001157291.1|	0	Fanconi anemia, complementation group E isoform 1 [Mus musculus]

mmu-miR-10b-5p	NM_019966	-23.5	0.974782	-23.13	167	GO:0042579,GO:0016831,GO:0006084,GO:0019217,GO:0006635,	K01578|1|0.0|923|mmu:56690|malonyl-CoA decarboxylase [EC:4.1.1.9]	gi|56797739|ref|NP_064350.2|	0	malonyl-CoA decarboxylase, mitochondrial [Mus musculus]
mmu-miR-10b-5p	NM_148935	-28.2	0.489617	-23.29	166	GO:0043231,GO:0043234,GO:0005515,GO:0000981,GO:0043565,GO:0043566,GO:0000122,GO:0007275,GO:0045165,GO:0044057,GO:0048588,	K09407|1|0.0|993|mmu:116810|forkhead box protein N	gi|91598891|ref|NP_683737.2|	0	forkhead box protein N4 [Mus musculus]

mmu-miR-10b-5p	NM_011073	-25.8	0.724411	-24.31	165	GO:0031224,GO:0005764,GO:0044440,GO:0031410,GO:0046872,GO:0015267,GO:0002347,GO:0051259,GO:0008219,GO:0048511,GO:0002252,GO:0010033,	K07818|1|0.0|1144|mmu:18646|perforin 1	gi|225735600|ref|NP_035203.3|	0	perforin-1 precursor [Mus musculus]
mmu-miR-10b-5p	NM_178592	-25.1	0.810116	-20.6	164	GO:0031224,GO:0005488,GO:0003824,	-	gi|30519896|ref|NP_848707.1|	0	abhydrolase domain-containing protein 16A [Mus musculus]
mmu-miR-10b-5p	NR_045949	-25.2	0.92553	-24.22	163	GO:0005515,GO:0044267,	K09512|1|2e-30|135|mgp:100546860|DnaJ homolog subfamily B member	gi|148680015|gb|EDL11962.1|	5.56E-130	RIKEN cDNA 4930503B20, isoform CRA_b [Mus musculus]

mmu-miR-10b-5p	NM_199469	-24.8	0.986637	-20.81	162	GO:0043231,GO:0044444,GO:0016020,GO:0046914,GO:0005515,GO:0016044,GO:0043161,GO:0006996,	K14015|1|0.0|1139|mmu:217365|nuclear protein localization protein 4 homolog	gi|148702796|gb|EDL34743.1|	0	nuclear protein localization 4 homolog (S. cerevisiae), isoform CRA_a [Mus musculus]
mmu-miR-10b-5p	NR_003639	-29.5	0.160324	-24.32	161	GO:0043231,GO:0031090,GO:0019787,GO:0032559,GO:0012501,GO:0006511,GO:0006259,GO:0000209,GO:0001818,GO:00071	K06689|1|1e-75|284|rno:79435|ubiquitin-conjugating enzyme E2 D/E [EC:6.3.2.19]	gi|13591896|ref|NP_112263.1|	8.20E-75	ubiquitin-conjugating enzyme E2 D2B [Rattus norvegicus]
mmu-miR-10b-5p	NR_030699	-22.2	0.999999	-22.86	160	-	-	gi|124430547|ref|NP_001074446.1|	1.85E-43	round spermatid basic protein 1-like [Mus musculus]
mmu-miR-10b-5p	NR_044987	-22.2	0.716157	-22.05	159	-	-	-	-	-

mmu-miR-10b-5p	NR_027666	-26.5	0.541269	-22.87	158	GO:0044424,	K06472|1|5e-104|379|cfa:488658|low affinity immunoglobulin gamma Fc region receptor II-a!K06498|3|1e-37|158|aml:100471819|high affinity immunoglobulin gamma Fc	gi|18056677|gb|AAL58112.1|AF426462_1	2.77E-167	FREB [Mus musculus]
mmu-miR-10b-5p	NR_040679	-24.5	0.693696	-23.3	157	-	-	gi|148688352|gb|EDL20299.1|	2.69E-15	mCG147648 [Mus musculus]
mmu-miR-10b-5p	NR_073442	-22.7	0.993415	-22.19	156	-	-	-	-	-
mmu-miR-10b-5p	NM_198604	-24.9	0.940058	-23.41	155	GO:0032155,GO:0005819,GO:0005088,GO:0008047,GO:0007266,	K12330|1|4e-21|104|bta:513377|Rho guanine nucleotide exchange factor 1	gi|148667413|gb|EDK99829.1|	0	pleckstrin homology domain containing, family G (with RhoGef domain) member 6, isoform CRA_a [Mus musculus]

mmu-miR-10b-5p	NR_045716	-23.4	0.986804	-22.26	154	GO:0043231,GO:0005515,GO:0016307,GO:0032559,GO:0046488,GO:0046474,GO:0006796,	K00920|1|1e-21|106|rno:140607|1-phosphatidylinositol-5-phosphate 4-kinase [EC:2.7.1.149]	gi|335288176|ref|XP_003126376.2|	5.25E-21	PREDICTED: phosphatidylinositol-5-phosphate 4-kinase type-2 gamma-like [Sus scrofa]
mmu-miR-10b-5p	NR_040540	-23.3	0.823234	-20.6	153	-	-	gi|148680191|gb|EDL12138.1|	1.48E-37	mCG144628 [Mus musculus]

mmu-miR-10b-5p	NM_201601	-21.4	1	-21.4	152	GO:0031224,GO:0043231,GO:0045202,GO:0044444,GO:0044421,GO:0004714,GO:0017134,GO:0005539,GO:0032559,GO:0007224,GO:0021859,GO:0030177,GO:0010463,GO:0060688,GO:0043009,GO:0060602,GO:0055008,GO:0021761,GO:0001942,GO:0042471,GO:0006464,GO:00352	K05093|1|0.0|1374|cfa:415125|fibroblast growth factor receptor 2 [EC:2.7.10.1]	gi|116089355|ref|NP_963895.2|	0	fibroblast growth factor receptor 2 isoform IIIb [Mus musculus]
mmu-miR-10b-5p	NR_045175	-21.3	0.950982	-20.51	151	-	-	gi|148670353|gb|EDL02300.1|	6.36E-34	mCG1041367 [Mus musculus]
mmu-miR-10b-5p	NR_045892	-21	0.999956	-20.33	150	-	-	gi|74221512|dbj|BAE21481.1|	4.02E-50	unnamed protein product [Mus musculus]
mmu-miR-10b-5p	NR_027887	-25.2	0.91151	-22.37	149	-	-	gi|148674316|gb|EDL06263.1|	1.16E-57	mCG15731, isoform CRA_a [Mus musculus]

mmu-miR-10b-5p	NR_027641	-23.1	0.999792	-20.75	148	GO:0031090,GO:0043231,GO:0006810,	-	gi|50511189|dbj|BAD32580.1|	0	mKIAA1928 protein [Mus musculus]
mmu-miR-10b-5p	NR_045556	-20.3	1	-20.67	147	-	K06813|1|0.0|6239|mmu:22295|cadherin 23	gi|60417436|emb|CAI59610.1|	0	cadherin 23 [Mus musculus]
mmu-miR-10b-5p	NR_033586	-20.9	1	-20.65	146	-	-	gi|54887404|gb|AAH85163.1|	0	Fam120b protein [Mus musculus]
mmu-miR-10b-5p	NR_030675	-22.5	0.999894	-22.77	145	-	-	gi|74180681|dbj|BAE25568.1|	9.47E-49	unnamed protein product [Mus musculus]
mmu-miR-10b-5p	NR_015388	-21.7	1	-20.03	144	-	-	gi|148682006|gb|EDL13953.1|	2.99E-41	mCG19080, isoform CRA_a [Mus musculus]
mmu-miR-10b-5p	NR_045069	-22.9	0.999979	-21.57	143	-	-	gi|149026199|gb|EDL82442.1|	4.76E-07	rCG63049 [Rattus norvegicus]
mmu-miR-10b-5p	NM_198644	-24.1	0.996194	-23.68	142	GO:0043231,GO:0044444,GO:0046914,GO:0003676,GO:0006351,	K09228|1|5e-51|204|xtr:100216012|KRAB domain-containing zinc finger protein	gi|148697448|gb|EDL29395.1|	0	zinc finger protein 406, isoform CRA_b [Mus musculus]
mmu-miR-10b-5p	NR_033455	-20.2	1	-23.29	141	-	-	gi|74206333|dbj|BAE24905.1|	1.26E-23	unnamed protein product [Mus musculus]

mmu-miR-10b-5p	NR_001461	-20.5	1	-21.9	140	-	K11849|1|6e-55|213|acs:100555596|ubiquitin carboxyl-terminal hydrolase 25/28 [EC:3.1.2.15]!K06478|4|4e-43|182|acs:100563672|protein tyrosine phosphatase, receptor type, C [EC:3.1.3.4	gi|209553980|gb|AAA39398.2|	0	ORF2 [Mus musculus domesticus]
mmu-miR-10b-5p	NM_175683	NA	NA	-21.28	156	GO:0043231,GO:0004520,GO:0004527,GO:0009314,GO:0030098,GO:0006996,GO:00062	K10887|1|0.0|868|rno:259171|DNA cross-link repair 1C protein [EC:3.1.-.-]	gi|148675985|gb|EDL07932.1|	0	DNA cross-link repair 1C, PSO2 homolog (S. cerevisiae), isoform CRA_e [Mus musculus]
mmu-miR-10b-5p	NM_212485	NA	NA	-21.95	155	GO:0005882,	K07605|1|0.0|860|mmu:223915|type II keratin, basic	gi|47059013|ref|NP_997650.1|	0	keratin, type II cytoskeletal 73 [Mus musculus]

mmu-miR-10b-5p	NM_027998	NA	NA	-28.51	154	GO:0043296,GO:0007043,	K06087|1|4e-139|495|mmu:71908|claudin	gi|21312576|ref|NP_082274.1|	2.94E-138	claudin-23 [Mus musculus]
mmu-miR-10b-5p	NM_025689	NA	NA	-21.81	154	GO:0031224,GO:0043231,	-	gi|258679490|ref|NP_079965.2|	0	coiled-coil domain-containing protein 51 [Mus musculus]
mmu-miR-10b-5p	NM_013866	NA	NA	-25.03	153	GO:0005634,GO:0046914,	K08421|1|1e-63|245|cfa:477591|G protein-coupled receptor 84!K10137|2|3e-08|61.6|mgp:100547023|zinc finger, matrin type 3	gi|119360327|ref|NP_038894.2|	5.34E-172	zinc finger protein 385A [Mus musculus]
mmu-miR-10b-5p	NM_022563	NA	NA	-21.71	152	GO:0016021,GO:0044459,GO:0004871,GO:0004713,GO:0005515,GO:0032559,GO:0007167,GO:0008283,GO:0006464,	K05125|1|0.0|1665|mmu:18214|discoidin domain receptor family member 2 [EC:2.7.10.1]	gi|158508514|ref|NP_072075.2|	0	discoidin domain-containing receptor 2 precursor [Mus musculus]

mmu-miR-10b-5p	NM_183136	NA	NA	-21.06	151	GO:0004866,GO:0010466,	-	gi|114145596|ref|NP_898959.2|	1.56E-39	serine protease inhibitor Kazal-type 8 precursor [Mus musculus]
mmu-miR-10b-5p	NM_133227	NA	NA	-23.27	150	-	K14312|1|0.0|2612|mmu:170762|nuclear pore complex protein Nup155	gi|37360098|dbj|BAC98027.1|	0	mKIAA0791 protein [Mus musculus]
mmu-miR-10b-5p	NM_175751	NA	NA	-20.02	149	GO:0046872,	K10847|1|9e-52|207|spu:582941|DNA-repair protein complementing XP-A cells	gi|113199757|ref|NP_786927.2|	0	zinc finger protein 608 [Mus musculus]
mmu-miR-10b-5p	NM_029999	NA	NA	-27.31	148	GO:0031981,GO:0032501,GO:0006355,	-	gi|34328474|ref|NP_084275.3|	8.71E-56	protein LBH [Mus musculus]
mmu-miR-10b-5p	NM_023370	NA	NA	-20.67	147	-	K06813|1|0.0|6527|mmu:22295|cadherin 23	gi|124517680|ref|NP_075859.2|	0	cadherin-23 isoform 1 precursor [Mus musculus]
mmu-miR-1187	NM_001004455	-29.1	0.178862	-26.43	176	GO:0005930,GO:0043232,	-	gi|148705044|gb|EDL36991.1|	2.17E-82	cystin 1 [Mus musculus]

mmu-miR-1187	NM_001005419	-31.5	0.188451	-29.01	185	GO:0043231,GO:0016702,GO:0043169,GO:0008152,	K10712|1|2e-137|491|mmu:211488|cysteamine dioxygenase [EC:1.13.11.19]	gi|225703118|ref|NP_001005419.2|	1.16E-136	2-aminoethanethiol dioxygenase [Mus musculus]
mmu-miR-1187	NM_001007574	-33.4	0.141588	-30.46	187	-	K10480|1|0.0|1151|ptr:469383|BTB/POZ domain-containing protein	gi|341941146|sp|Q80TK0.5|K1107_MOUSE	0	RecName: Full=Uncharacterized protein KIAA1107

mmu-miR-1187	NM_001012477	-28.4	0.631144	-26.4	181	GO:0009986,GO:0016020,GO:0044421,GO:0060089,GO:0005125,GO:0014046,GO:0001935,GO:0007411,GO:0003006,GO:0032414,GO:0021537,GO:0007626,GO:0007276,GO:0002548,GO:0007166,GO:0001525,GO:0003013,GO:0042110,GO:0030832,GO:0006875,GO:0051707,GO:00098	K10031|1|1e-36|157|mmu:20315|C-X-C motif chemokine 12	gi|148667157|gb|EDK99573.1|	2.14E-50	chemokine (C-X-C motif) ligand 12, isoform CRA_c [Mus musculus]
mmu-miR-1187	NM_001013025	-39.7	0.008779	-29.59	177	GO:0044424,GO:0005126,GO:0030695,GO:0019222,GO:0007178,	-	gi|67906170|ref|NP_001013043.1|	0	transforming growth factor-beta receptor-associated protein 1 [Mus musculus]

mmu-miR-1187	NM_001013368	-29.7	0.306073	-52.28	355	GO:0043234,GO:0043231,GO:0042802,GO:0003676,GO:0001071,GO:0006351,	K09391|1|0.0|1683|mmu:108961|E2F transcription factor 7/8	gi|148691021|gb|EDL22968.1|		0	E2F transcription factor 8 [Mus musculus]
mmu-miR-1187	NM_001013441	-31.8	0.185449	-60.34	370	GO:0044444,GO:0016020,GO:0005515,GO:0030695,GO:0019222,GO:0007186,	-	gi|61806725|ref|NP_001013459.1|		0	synembryn-B isoform 1 [Mus musculus]
mmu-miR-1187	NM_001013753	-35.1	0.107249	-32.41	197	GO:0031224,GO:0046872,GO:0016337,	K04600|1|5e-65|252|tad:TRIADDRAFT_62266|cadherin EGF LAG seven-pass G-type receptor 1 (flamingo)	gi|260763908|ref|NP_001013775.2|		0	protocadherin 17 precursor [Mus musculus]

mmu-miR-1187	NM_001013811	-33.1	0.057782	-102.37	715	-	K10657|1|3e-08|62.0|mmu:224703|E3 ubiquitin-protein ligase MARCH2 [EC:6.3.2.19]	gi|62000668|ref|NP_001013833.1|	0	protein FAM169B [Mus musculus]
mmu-miR-1187	NM_001024699	-28.4	0.294403	-27.22	173	GO:0043169,	K10516|1|0.0|788|mmu:232879|zinc finger and BTB domain-containing protein 45	gi|85701534|ref|NP_001019870.1|	0	zinc finger and BTB domain-containing protein 45 [Mus musculus]
mmu-miR-1187	NM_001025156	-34.7	0.093118	-58.53	365	-	-	gi|148707840|gb|EDL39787.1|	0	coiled-coil domain containing 93, isoform CRA_b [Mus musculus]

mmu-miR-1187	NM_001025250	-25.3	0.903781	-25.62	177	GO:0031091,GO:0005578,GO:0016020,GO:0005539,GO:0005172,GO:0042802,GO:0046983,GO:0001775,GO:0021700,GO:0001935,GO:0006357,GO:0008284,GO:0010595,GO:0045765,GO:0043009,GO:0007169,GO:0001754,GO:0006887,GO:0009967,GO:0009791,GO:0007589,GO:00016	K05448|1|4e-152|540|mmu:22339|vascular endothelial growth factor A/B, PGF	gi|160358799|ref|NP_001020421.2|	2.90E-151	vascular endothelial growth factor A isoform 1 precursor [Mus musculus]

mmu-miR-1187	NM_001025257	-25.3	0.890277	-25.62	177	GO:0031091,GO:0005578,GO:0005539,GO:0005172,GO:0042802,GO:0046983,GO:0061138,GO:0001775,GO:0001935,GO:0048468,GO:0001894,GO:0006357,GO:0048609,GO:0008284,GO:0010595,GO:0048592,GO:0045765,GO:0007169,GO:0006887,GO:0009790,GO:0009967,GO:00016	K05448|1|3e-119|430|mmu:22339|vascular endothelial growth factor A/B, PGF	gi|160358807|ref|NP_001020428.2|	1.19E-122	vascular endothelial growth factor A isoform 3 precursor [Mus musculus]
mmu-miR-1187	NM_001025377	-36.3	0.007373	-30.92	188	GO:0044424,GO:0005100,GO:0032321,GO:0065008,	-	gi|148694910|gb|EDL26857.1|	8.17E-93	Rho GTPase activating protein 15, isoform CRA_e [Mus musculus]

mmu-miR-1187	NM_001025571	-36.1	0.031544	-224.55	1333	GO:0016020,GO:0030674,GO:0019904,GO:0051249,GO:0032502,GO:0007165,	K08273|1|0.0|719|mmu:27371|SH2 domain protein 2A	gi|70909334|ref|NP_001020742.1|	0	SH2 domain-containing protein 2A isoform 2 [Mus musculus]
mmu-miR-1187	NM_001033223	-36.6	0.03362	-62.61	382	GO:0030054,GO:0019904,GO:0016192,GO:0001505,GO:0065003,	K12075|1|1e-17|94.0|cel:C25F6.2|discs large protein	gi|332221001|ref|XP_003259645.1|	2.06E-72	PREDICTED: protein lin-7 homolog A-like [Nomascus leucogenys]
mmu-miR-1187	NM_001033234	-32.2	0.042302	-29.51	176	-	-	gi|85701742|ref|NP_001028406.1|	8.97E-91	uncharacterized protein LOC194854 [Mus musculus]
mmu-miR-1187	NM_001033317	-28.6	0.311769	-25.97	167	GO:0034703,GO:0016208,GO:0005267,GO:0005217,GO:0007606,GO:0030001,	K04951|1|0.0|1040|mmu:233649|cyclic nucleotide gated channel alpha 4	gi|84370304|ref|NP_001028489.1|	0	cyclic nucleotide-gated cation channel alpha-4 [Mus musculus]
mmu-miR-1187	NM_001033329	-35.9	0.036871	-84.43	539	GO:0044444,GO:0005088,GO:0006917,GO:0007267,GO:0019222,GO:0006811,GO:0009966,GO:0007172,GO:0007266,	K05769|1|0.0|664|tgu:100227332|Rho guanine nucleotide exchange factor 4	gi|84370312|ref|NP_001028501.1|	0	rho guanine nucleotide exchange factor 9 [Mus musculus]

mmu-miR-1187	NM_001033348	-34.8	0.121676	-31.69	188	GO:0043231,GO:0044444,GO:0005099,GO:0046983,GO:0032852,	K07207|1|2e-22|110|bfo:BRAFLDRAFT_64809|tuberous sclerosis 2	gi|158187505|ref|NP_001028520.2|	0	ral GTPase-activating protein subunit alpha-2 [Mus musculus]
mmu-miR-1187	NM_001033378	-29.8	0.237409	-26.89	185	-	-	gi|283837884|ref|NP_001028550.2|	0	uncharacterized protein LOC319493 [Mus musculus]
mmu-miR-1187	NM_001033382	-26.9	0.850901	-25.44	169	GO:0031224,GO:0043169,GO:0005261,GO:0050953,GO:0006810,	K04861|1|0.0|2175|mmu:319734|voltage-dependent calcium channel alpha-2/delta-4	gi|124517718|ref|NP_001028554.2|	0	voltage-dependent calcium channel subunit alpha-2/delta-4 [Mus musculus]
mmu-miR-1187	NM_001033399	-36.1	0.048315	-33.46	193	GO:0003824,GO:0008152,	K00078|1|3e-09|66.6|dpe:Dper_GL19398|dihydrodiol dehydrogenase / D-xylose 1-dehydrogenase (NADP) [EC:1.3.1.20	gi|85701893|ref|NP_001028571.1|	0	glucose-fructose oxidoreductase domain-containing protein 1 precursor [Mus musculus]

mmu-miR-1187	NM_001033493	-26.1	0.783722	-26.06	176	GO:0031224,GO:0004888,GO:0007186,	K08454|1|0.0|1231|mmu:435529|G protein-coupled receptor 111	gi|134949047|ref|NP_001028665.2|	0	G protein-coupled receptor 111 precursor [Mus musculus]
mmu-miR-1187	NM_001033536	-36.2	0.057832	-33.35	193	GO:0043231,GO:0003676,GO:0006351,	K08061|1|6e-62|241|mgp:100543120|regulatory factor X 5	gi|88703055|ref|NP_001028708.1|	0	regulatory factor X domain containing 2 [Mus musculus]
mmu-miR-1187	NM_001035532	-36.6	0.040819	-33.92	197	GO:0032991,GO:0005515,GO:0033036,GO:0007167,GO:0051259,GO:0030036,	-	gi|123231686|emb|CAM21286.1|	0	A kinase (PRKA) anchor protein 2 [Mus musculus]
mmu-miR-1187	NM_001035533	-36.6	0.040672	-33.92	197	GO:0032991,GO:0005515,GO:0033036,GO:0007167,GO:0051259,GO:0030036,	-	gi|123231688|emb|CAM21288.1|	0	Palm2-Akap2 fusion protein [Mus musculus]

mmu-miR-1187	NM_001037166	-35.1	0.010134	-32.73	193	GO:0015935,GO:0005198,GO:0006412,GO:0019080,GO:0031016,	K02951|1|3e-71|270|mmu:237433|small subunit ribosomal protein S12e	gi|81230474|ref|NP_001032243.1|	2.12E-70	ribosomal protein S12-like [Mus musculus]
mmu-miR-1187	NM_001037713	-34.3	0.030993	-30.85	177	GO:0044424,GO:0019221,	-	gi|161333845|ref|NP_001032802.2|	7.54E-162	XIAP-associated factor 1 [Mus musculus]
mmu-miR-1187	NM_001037745	-27.3	0.46851	-25.85	177	GO:0005622,GO:0046914,GO:0003676,GO:0006351,	K09228|1|0.0|1142|mmu:244556|KRAB domain-containing zinc finger protein	gi|83627693|ref|NP_001032834.1|	0	zinc finger protein 791 [Mus musculus]
mmu-miR-1187	NM_001037937	-33.4	0.061768	-56.46	368	GO:0044464,GO:0005488,GO:0009968,GO:0006915,GO:0008361,GO:0033673,	K12365|1|3e-28|128|xtr:100488039|phosphatidylinositol-3,4,5-trisphosphate-dependent Rac exchanger 1	gi|83816897|ref|NP_001033026.1|	0	DEP domain-containing mTOR-interacting protein isoform b [Mus musculus]

mmu-miR-1187	NM_001038637	-31.9	0.130725	-29.33	185	GO:0043231,GO:0031234,GO:0017111,GO:0005488,GO:0060089,GO:0001775,GO:0007267,GO:0033762,GO:0009207,GO:0015980,GO:0007599,GO:0007166,	K07826|1|3e-34|148|cfa:610817|guanine nucleotide-binding protein G(I)/G(S)/G(O) subunit gamma-2	gi|148669586|gb|EDL01533.1|	2.73E-35	mCG144516 [Mus musculus]
mmu-miR-1187	NM_001038701	-25.4	0.966001	-60.74	374	GO:0044459,GO:0033267,GO:0034702,GO:0005253,GO:0032403,GO:0016917,GO:0015276,GO:0007186,GO:0007270,GO:00156	K05181|1|0.0|939|mmu:14402|gamma-aminobutyric acid receptor subunit beta	gi|84662774|ref|NP_001033790.1|	0	gamma-aminobutyric acid receptor subunit beta-3 isoform b precursor [Mus musculus]
mmu-miR-1187	NM_001039200	-34	0.102616	-31.53	185	GO:0031224,GO:0005488,	-	gi|85861260|ref|NP_001034289.1|	0	ER membrane protein complex subunit 1 isoform 2 precursor [Mus musculus]

mmu-miR-1187	NM_001039354	-36.6	0.033926	-62.61	382	GO:0043296,GO:0044430,GO:0019904,GO:0016192,GO:0001505,GO:0065003,	K12075|1|1e-17|94.0|cel:C25F6.2|discs large protein	gi|4759306|ref|NP_004655.1|	1.25E-101	protein lin-7 homolog A [Homo sapiens]
mmu-miR-1187	NM_001039371	-34.5	0.090891	-28.89	185	GO:0031224,GO:0005215,GO:0006810,	K08211|1|0.0|932|mmu:242126|MFS transporter, OCT family, solute carrier family 22 (organic cation transporter), member 15	gi|260166627|ref|NP_001034460.2|	0	solute carrier family 22 member 15 [Mus musculus]
mmu-miR-1187	NM_001039483	-25.9	0.895895	-93.74	570	GO:0012505,GO:0031224,GO:0031090,GO:0043231,	K02920|1|6e-08|54.7|mcc:710317|large subunit ribosomal protein L36e	gi|332220032|ref|XP_003259161.1|	2.54E-91	PREDICTED: transmembrane and coiled-coil domain-containing protein 1-like, partial [Nomascus leucogenys]

mmu-miR-1187	NM_001040026	-29.7	0.34895	-26.33	169	GO:0043231,GO:0019866,GO:0046914,GO:0000041,GO:0019725,GO:0043623,	K07152|1|2e-138|494|mmu:52892|	gi|74228413|dbj|BAE24044.1|	1.71E-137	unnamed protein product [Mus musculus]
mmu-miR-1187	NM_001040112	-29.8	0.405501	-53.39	356	GO:0000139,GO:0046914,GO:0035091,GO:0005099,GO:0032879,GO:0065008,GO:0031346,GO:0032232,GO:0032012,GO:00323	K12490|1|0.0|2335|mmu:69710|Arf-GAP, Rho-GAP domain, ANK repeat and PH domain-containing protein	gi|187954747|gb|AAI41180.1|	0	ArfGAP with RhoGAP domain, ankyrin repeat and PH domain 1 [Mus musculus]
mmu-miR-1187	NM_001045533	-32	0.146308	-62.1	386	GO:0044431,GO:0031224,GO:0046914,GO:0019787,	K10659|1|0.0|649|mmu:381270|E3 ubiquitin-protein ligase MARCH4/9/11 [EC:6.3.2.19]	gi|28972736|dbj|BAC65784.1|	0	mKIAA1399 protein [Mus musculus]

mmu-miR-1187	NM_001077499	-26.7	0.976214	-30.42	193	GO:0044297,GO:0031410,GO:0033267,GO:0005887,GO:0031420,GO:0005272,GO:0032559,GO:0048513,GO:0006935,GO:0034220,GO:0008366,GO:0006814,GO:00083	K04840|1|0.0|3401|mmu:20273|voltage-gated sodium channel type VIII alpha	gi|117414174|ref|NP_001070967.1|	0	sodium channel protein type 8 subunit alpha isoform 1 [Mus musculus]

mmu-miR-1187	NM_001077695	-36.2	0.057883	-33.45	193	GO:0044456,GO:0031981,GO:0043005,GO:0044430,GO:0015630,GO:0044297,GO:0005783,GO:0060089,GO:0051427,GO:0010861,GO:0004468,GO:0019904,GO:0003713,GO:0006357,GO:0030518,GO:0006629,GO:0010469,GO:0022037,	K11255|1|0.0|2465|mmu:17978|nuclear receptor coactivator 2	gi|118026944|ref|NP_001071163.1|	0	nuclear receptor coactivator 2 isoform b [Mus musculus]

mmu-miR-1187	NM_001080126	-35.1	0.019386	-32.41	197	GO:0031981,GO:0005815,GO:0031966,GO:0042995,GO:0030686,GO:0031264,GO:0004175,GO:0005515,GO:0009612,GO:0002573,GO:0043627,GO:0034097,GO:0051259,GO:0007249,GO:0006917,GO:0010038,GO:0006950,GO:0006508,GO:0002237,GO:00	K04398|1|0.0|952|mmu:12370|caspase 8 [EC:3.4.22.61]	gi|33859520|ref|NP_033942.1|	0	caspase-8 [Mus musculus]
mmu-miR-1187	NM_001080742	-32	0.041564	-51.9	357	GO:0044431,GO:0016021,GO:0014706,GO:0006810,GO:0048513,GO:0048869,	K08514|1|6e-37|155|mmu:53620|vesicle-associated membrane protein 5	gi|13661881|gb|AAK38123.1|	9.06E-58	CAMP protein [Mus musculus]

mmu-miR-1187	NM_001081050	-36.7	0.050404	-33.92	197	GO:0043296,GO:0009987,	K04237|1|1e-162|576|mdo:100022889|partitioning defective protein 3	gi|163310767|ref|NP_001074519.2|	0	partitioning defective 3 homolog B [Mus musculus]
mmu-miR-1187	NM_001081101	-32.6	0.207436	-27.61	172	-	-	gi|124487376|ref|NP_001074570.1|	0	UV-stimulated scaffold protein A [Mus musculus]

mmu-miR-1187	NM_001081147	-28.2	0.581231	-58.17	366	GO:0005911,GO:0016021,GO:0042995,GO:0008528,GO:0014075,GO:0007166,GO:0051705,GO:0007611,GO:0051048,GO:0060746,GO:0007589,GO:0007320,GO:0032228,GO:0014073,GO:0043627,GO:0030900,GO:0022602,GO:0051480,GO:0045987,GO:0006950,GO:0007631,GO:00508	K04229|1|0.0|686|mmu:18430|oxytocin receptor	gi|124487243|ref|NP_001074616.1|	0	oxytocin receptor [Mus musculus]
mmu-miR-1187	NM_001081220	-29.4	0.658582	-25.24	177	GO:0031224,GO:0004888,	K08469|1|0.0|683|tgu:100229682|G protein-coupled receptor 158	gi|124487045|ref|NP_001074689.1|	0	probable G-protein coupled receptor 179 precursor [Mus musculus]

mmu-miR-1187	NM_001081267	-29.1	0.762104	-49.41	349	GO:0005515,GO:0017111,GO:0006355,GO:0051100,GO:0043486,GO:0045935,	K11657|1|0.0|2166|mmu:233532|remodeling and spacing factor 1	gi|124486905|ref|NP_001074736.1|	0	remodeling and spacing factor 1 [Mus musculus]
mmu-miR-1187	NM_001081477	-33.7	0.119761	-56.17	353	-	K11798|1|0.0|3097|mmu:382236|bromodomain and WD repeat domain containing protein 1/3	gi|125490358|ref|NP_001074946.1|	0	bromodomain and WD repeat-containing protein 3 [Mus musculus]
mmu-miR-1187	NM_001081557	-36.7	0.05068	-62.47	370	GO:0043231,GO:0005515,GO:0006351,	K13505|1|6e-29|107|ssc:100628225|vesicle-associated membrane protein	gi|125719159|ref|NP_001075026.1|	0	calmodulin-binding transcription activator 1 isoform 1 [Mus musculus]

mmu-miR-1187	NM_001081567	-25.2	0.99045	-54.28	360	GO:0031981,GO:0016020,GO:0044444,GO:0016909,GO:0004712,GO:0032559,GO:0007166,GO:0006355,GO:0002224,GO:0006468,GO:0000165,	K04440|1|0.0|921|mmu:26414|c-Jun N-terminal kinase [EC:2.7.11.24]	gi|148688303|gb|EDL20250.1|	0	mitogen activated protein kinase 10, isoform CRA_a [Mus musculus]
mmu-miR-1187	NM_001082960	-36.7	0.024211	-33.92	197	GO:0043231,GO:0009986,GO:0005887,GO:0004871,GO:0043394,GO:0046983,GO:0005539,GO:0016337,GO:0007599,GO:0042098,GO:0007166,GO:0030595,	K06461|1|0.0|2239|mmu:16409|integrin alpha M	gi|132626289|ref|NP_001076429.1|	0	integrin alpha-M isoform 1 precursor [Mus musculus]

mmu-miR-1187	NM_001082961	-26.1	0.601952	-28.86	180	GO:0030532,GO:0005515,GO:0009719,GO:0006396,	K11100|1|5e-62|239|ssc:100626432|small nuclear ribonucleoprotein N	gi|296215020|ref|XP_002753951.1|	1.20E-63	PREDICTED: small nuclear ribonucleoprotein-associated protein N-like [Callithrix jacchus]
mmu-miR-1187	NM_001082962	-26.1	0.610634	-28.86	180	GO:0030532,GO:0005515,GO:0009719,GO:0006396,	K11100|1|5e-62|239|ssc:100626432|small nuclear ribonucleoprotein N	gi|296215020|ref|XP_002753951.1|	1.23E-63	PREDICTED: small nuclear ribonucleoprotein-associated protein N-like [Callithrix jacchus]
mmu-miR-1187	NM_001083810	-35.4	0.037262	-26.69	173	GO:0043231,	-	gi|139948241|ref|NP_780390.3|	2.80E-180	proline-rich protein 5-like [Mus musculus]
mmu-miR-1187	NM_001085508	-29.9	0.359584	-27.17	181	-	-	gi|159574137|emb|CAP19218.1|	0	novel protein [Mus musculus]
mmu-miR-1187	NM_001099276	-32.2	0.257189	-30.88	184	GO:0043234,GO:0016023,GO:0016020,GO:0005543,GO:0016307,GO:0035004,GO:0032559,GO:0046488,GO:0007243,GO:00480	K00923|1|0.0|3145|mmu:240752|phosphatidylinositol-4-phosphate 3-kinase [EC:2.7.1.154]	gi|258645086|ref|NP_001092746.2|	0	phosphoinositide-3-kinase, class 2 beta polypeptide [Mus musculus]

mmu-miR-1187	NM_001099738	-36.2	0.020483	-59.64	366	GO:0005515,	K09504|1|1e-07|60.5|tad:TRIADDRAFT_64241|DnaJ homolog subfamily A member 3	gi|153267505|ref|NP_001093208.1|	0	dnaJ homolog subfamily C member 28 isoform 1 [Mus musculus]
mmu-miR-1187	NM_001100444	-30.2	0.17503	-31.83	193	-	-	gi|158303290|ref|NP_001093914.1|	2.96E-110	uncharacterized protein LOC546272 [Mus musculus]
mmu-miR-1187	NM_001101507	-25.5	0.562911	-29.55	173	GO:0031224,GO:0030246,	K06502|1|6e-20|99.4|pon:100451755|CD69 antigen!K10071|4|4e-18|93.6|aml:100474734|C-type lectin domain family 2,	gi|168229210|ref|NP_001094977.1|	4.38E-99	C-type lectin domain family 2 member L [Mus musculus]

mmu-miR-1187	NM_001103156	-34.7	0.135766	-35.83	197	GO:0005768,GO:0031228,GO:0005798,GO:0046914,GO:0050662,GO:0003824,GO:0006892,GO:0009719,GO:0006091,GO:0000041,GO:0006887,	K14738|1|0.0|935|mmu:74051|metalloreductase STEAP2 [EC:1.16.1.-]	gi|124249344|ref|NP_083010.2|	0	metalloreductase STEAP2 [Mus musculus]
mmu-miR-1187	NM_001103157	-34.7	0.14056	-35.83	197	GO:0005768,GO:0031228,GO:0005798,GO:0046914,GO:0050662,GO:0003824,GO:0006892,GO:0009719,GO:0006091,GO:0000041,GO:0006887,	K14738|1|0.0|935|mmu:74051|metalloreductase STEAP2 [EC:1.16.1.-]	gi|124249344|ref|NP_083010.2|	0	metalloreductase STEAP2 [Mus musculus]

mmu-miR-1187	NM_001105159	-29.4	0.188359	-26.54	182	GO:0043231,GO:0031090,GO:0033695,GO:0008289,GO:0070643,GO:0016712,GO:0005515,GO:0005506,GO:0008395,GO:0055114,GO:0010038,GO:0031667,GO:0006730,GO:0031960,GO:0006721,GO:0042493,GO:0008202,GO:0032502,GO:0017144,GO:00	K07424|1|0.0|1027|mmu:53973|cytochrome P450, family 3, subfamily A [EC:1.14.14.1]	gi|157364978|ref|NP_001098629.1|	0	cytochrome P450 3A41 [Mus musculus]

mmu-miR-1187	NM_001109757	-33.9	0.142798	-28.56	181	GO:0031224,GO:0005903,GO:0044459,GO:0005798,GO:0044297,GO:0005768,GO:0043682,GO:0032559,GO:0016531,GO:0005515,GO:0010467,GO:0021859,GO:0002294,GO:0035434,GO:0042415,GO:0030003,GO:0043009,GO:0043085,GO:0009072,GO:0085029,GO:0009207,GO:00435	K01533|1|0.0|2765|mmu:11977|Cu2+-exporting ATPase [EC:3.6.3.4]	gi|148682115|gb|EDL14062.1|	0	ATPase, Cu++ transporting, alpha polypeptide, isoform CRA_b [Mus musculus]
mmu-miR-1187	NM_001110253	-31.9	0.277553	-29.53	189	GO:0031224,GO:0046872,GO:0051234,	K12478|1|2e-24|117|dre:562943|early endosome antigen 1	gi|159110614|ref|NP_001103723.1|	0	FYVE and coiled-coil domain-containing protein 1 [Mus musculus]

mmu-miR-1187	NM_001110266	-25.3	0.848995	-25.62	177	GO:0031091,GO:0005578,GO:0016020,GO:0005539,GO:0005172,GO:0042802,GO:0046983,GO:0001775,GO:0021700,GO:0001935,GO:0006357,GO:0008284,GO:0010595,GO:0045765,GO:0043009,GO:0007169,GO:0001754,GO:0006887,GO:0009967,GO:0009791,GO:0007589,GO:00016	K05448|1|4e-48|194|mmu:22339|vascular endothelial growth factor A/B, PGF	gi|160358811|ref|NP_001103736.1|	3.99E-63	vascular endothelial growth factor A isoform 4 precursor [Mus musculus]

mmu-miR-1187	NM_001110267	-25.3	0.841552	-25.62	177	GO:0016023,GO:0005578,GO:0016020,GO:0005539,GO:0005172,GO:0042802,GO:0046983,GO:0021700,GO:0001935,GO:0006357,GO:0008284,GO:0010595,GO:0045765,GO:0043009,GO:0007169,GO:0001754,GO:0009967,GO:0009791,GO:0007589,GO:0001666,GO:0001934,GO:00328	K05448|1|5e-49|197|rno:83785|vascular endothelial growth factor A/B, PGF	gi|160358815|ref|NP_001103737.1|	3.50E-64	vascular endothelial growth factor A isoform 5 precursor [Mus musculus]

mmu-miR-1187	NM_001110268	-25.3	0.826847	-25.62	177	GO:0031091,GO:0005578,GO:0005539,GO:0005172,GO:0042802,GO:0046983,GO:0001775,GO:0021700,GO:0001935,GO:0006357,GO:0008284,GO:0010595,GO:0045765,GO:0043009,GO:0007169,GO:0001754,GO:0006887,GO:0009967,GO:0009791,GO:0007589,GO:0001666,GO:00019	K05448|1|3e-15|84.7|mmu:22339|vascular endothelial growth factor A/B, PGF	gi|160358817|ref|NP_001103738.1|	1.60E-34	vascular endothelial growth factor A isoform 6 precursor [Mus musculus]

mmu-miR-1187	NM_001110796	-35.1	0.190724	-32.41	197	GO:0016020,GO:0046872,GO:0005543,GO:0005515,GO:0006996,GO:0006903,GO:0006887,GO:0030072,GO:0019935,	K15297|1|1e-30|138|nvi:100121009|regulating synaptic membrane exocytosis protein 2!K15291|2|8e-29|132|acs:100568133|regulating synaptic membrane exocytosis protein 1	gi|160707978|ref|NP_001104266.1|	0	protein piccolo isoform 2 [Mus musculus]
mmu-miR-1187	NM_001110849	-35.4	0.035735	-26.69	173	GO:0043231,	-	gi|139948241|ref|NP_780390.3|	2.71E-180	proline-rich protein 5-like [Mus musculus]

mmu-miR-1187	NM_001111023	-25.4	0.985814	-59.76	373	GO:0031981,GO:0001071,GO:0046983,GO:0003676,GO:0046872,GO:0042802,GO:0032559,GO:0008134,GO:0002762,GO:0006357,GO:0033189,GO:0002763,GO:0048468,GO:0043009,GO:0007610,GO:0045765,GO:00	K08367|1|0.0|812|rno:50662|runt-related transcription factor 1	gi|161169031|ref|NP_001104493.1|	0	runt-related transcription factor 1 isoform 3 [Mus musculus]

mmu-miR-1187	NM_001113416	-29.7	0.231138	-27.02	167	GO:0001726,GO:0005924,GO:0043232,GO:0005515,GO:0071363,GO:0001840,GO:0010631,GO:0001837,GO:0009952,GO:0043393,GO:0003382,GO:0010468,GO:0007162,GO:0006929,GO:0030036,GO:0032880,GO:0001954,GO:0000578,GO:00017	K06107|1|6e-82|306|tgu:100218952|erythrocyte membrane protein band 4.1	gi|164663872|ref|NP_001106887.1|	0	band 4.1-like protein 5 isoform 2 [Mus musculus]

mmu-miR-1187	NM_001113559	-30.6	0.401494	-29.76	184	GO:0043231,GO:0000975,GO:0001071,GO:0043565,GO:0046983,GO:0007417,GO:0009888,GO:0006357,GO:0045664,GO:0043009,GO:0010001,	K09269|1|0.0|1071|mmu:20678|transcription factor SOX5/6/13 (SOX group D)	gi|341926306|ref|NP_001107031.2|	0	transcription factor SOX-5 isoform b [Mus musculus]
mmu-miR-1187	NM_001114328	-36.9	0.011803	-31.48	189	GO:0031224,GO:0009987,	K06672|1|6e-06|54.3|xla:445865|cohesin loading factor subunit SCC2	gi|166998947|ref|NP_001107800.1|	0	cell cycle progression protein 1 isoform 1 [Mus musculus]

mmu-miR-1187	NM_001122683	-32.6	0.081126	-60.78	384	GO:0019866,GO:0044429,GO:0016616,GO:0008289,GO:0007417,GO:0006629,GO:0009888,GO:0044248,GO:0044249,GO:0010038,GO:0043627,GO:0031667,GO:0043434,GO:0051384,GO:0048513,GO:00069	K00019|1|0.0|694|mmu:71911|3-hydroxybutyrate dehydrogenase [EC:1.1.1.30]	gi|170014720|ref|NP_780386.3|	0	D-beta-hydroxybutyrate dehydrogenase, mitochondrial precursor [Mus musculus]
mmu-miR-1187	NM_001122738	-33.6	0.086347	-28.86	184	-	K11649|1|2e-08|63.2|acs:100555956|SWI/SNF related-matrix-associated actin-dependent regulator of chromatin subfamily C	gi|170172570|ref|NP_001116210.1|	0	KAT8 regulatory NSL complex subunit 1-like protein isoform 1 [Mus musculus]

mmu-miR-1187	NM_001122895	-36.7	0.009439	-33.92	197	GO:0043231,GO:0003712,GO:0008366,GO:0001503,GO:2000026,GO:0006355,GO:00074	-	gi|171543864|ref|NP_001116367.1|	0	NGFI-A-binding protein 2 isoform 2 [Mus musculus]
mmu-miR-1187	NM_001128086	-31.6	0.282471	-29.35	183	GO:0030425,GO:0044459,GO:0016604,GO:0044430,	K10380|1|5e-30|135|tsp:Tsp_02713|ankyrin	gi|294345388|ref|NP_001121558.2|	0	ankyrin repeat and sterile alpha motif domain-containing protein 1B isoform 1 [Mus musculus]
mmu-miR-1187	NM_001131021	-34.7	0.053162	-32.54	189	-	K15048|1|4e-14|82.0|cel:F55C7.7|kalirin [EC:2.7.11.1]	gi|196162697|ref|NP_001124493.1|	0	uncharacterized protein KIAA1755 homolog isoform 2 [Mus musculus]
mmu-miR-1187	NM_001134457	-32.8	0.168599	-85.09	547	-	-	gi|197333803|ref|NP_001127929.1|	0	uncharacterized protein LOC385658 [Mus musculus]
mmu-miR-1187	NM_001134465	-29.9	0.33689	-45.07	330	-	-	gi|47847482|dbj|BAD21413.1|	0	mFLJ00229 protein [Mus musculus]

mmu-miR-1187	NM_001135151	-36.4	0.029328	-33.82	197	GO:0031252,GO:0043231,GO:0031224,GO:0005381,GO:0006829,GO:0006826,	K14720|1|0.0|984|mmu:213053|solute carrier family 39 (zinc transporter), member 14	gi|148703960|gb|EDL35907.1|	0	solute carrier family 39 (zinc transporter), member 14, isoform CRA_b [Mus musculus]
mmu-miR-1187	NM_001135152	-36.4	0.029066	-33.82	197	GO:0031252,GO:0043231,GO:0031224,GO:0005381,GO:0006829,GO:0006826,	K14720|1|0.0|984|mmu:213053|solute carrier family 39 (zinc transporter), member 14	gi|148703960|gb|EDL35907.1|	0	solute carrier family 39 (zinc transporter), member 14, isoform CRA_b [Mus musculus]
mmu-miR-1187	NM_001141922	-30.8	0.185986	-67.49	495	-	-	gi|213512434|ref|NP_001135394.1|	9.51E-152	protein BEAN1 isoform a [Mus musculus]
mmu-miR-1187	NM_001141924	-30.8	0.173206	-67.49	495	-	-	gi|213512434|ref|NP_001135394.1|	1.53E-119	protein BEAN1 isoform a [Mus musculus]
mmu-miR-1187	NM_001141925	-30.8	0.173325	-67.49	495	-	-	gi|213512434|ref|NP_001135394.1|	4.43E-127	protein BEAN1 isoform a [Mus musculus]

mmu-miR-1187	NM_001142963	-30.1	0.333562	-27.99	176	-	K09228|1|0.0|1006|rno:100125368|KRAB domain-containing zinc finger protein	gi|34785742|gb|AAH57364.1|	0	EG210583 protein [Mus musculus]
mmu-miR-1187	NM_001145875	-33.8	0.036564	-28.94	189	GO:0031224,	-	gi|30425128|ref|NP_780626.1|	2.58E-151	androgen-dependent TFPI-regulating protein isoform 1 [Mus musculus]
mmu-miR-1187	NM_001146007	-37	0.015694	-34.32	197	GO:0044424,GO:0042802,GO:0046914,GO:0051259,	K10648|1|0.0|1000|mmu:319236|tripartite motif-containing protein 5 [EC:6.3.2.19]!K11999|3|7e-131|469|bta:505265|tripartite motif-containing protein	gi|225579155|ref|NP_783608.3|	0	tripartite motif-containing 12C [Mus musculus]

mmu-miR-1187	NM_001159711	-36.7	0.000723	-33.92	197	GO:0043235,GO:0005576,GO:0016020,GO:0004872,GO:0001530,GO:0032490,GO:0007243,GO:0002224,GO:0007166,	K05400|1|5e-81|300|mmu:17087|lymphocyte antigen 96	gi|228008385|ref|NP_001153183.1|	3.08E-80	lymphocyte antigen 96 isoform B precursor [Mus musculus]
mmu-miR-1187	NM_001160400	-37.3	0.049142	-33.66	189	GO:0016020,GO:0046872,	K09434|1|0.0|1524|ssc:100516083|ETS domain-containing transcription factor ERF!K05637|5|4e-18|96.7|aml:100465830|laminin, alpha 1/2	gi|237874245|ref|NP_001153872.1|	0	multiple epidermal growth factor-like domains protein 8 precursor [Mus musculus]
mmu-miR-1187	NM_001161515	-36.6	0.008032	-33.92	197	GO:0044444,GO:0046914,GO:0016814,GO:0006213,GO:0009112,GO:0009117,	K01493|1|1e-99|365|mmu:320685|dCMP deaminase [EC:3.5.4.12]	gi|238859570|ref|NP_848903.2|	6.98E-99	deoxycytidylate deaminase [Mus musculus]

mmu-miR-1187	NM_001161516	-36.6	0.006767	-33.92	197	GO:0044444,GO:0046914,GO:0016814,GO:0006213,GO:0009112,GO:0009117,	K01493|1|9e-100|365|mmu:320685|dCMP deaminase [EC:3.5.4.1	gi|238859570|ref|NP_848903.2|	6.10E-99	deoxycytidylate deaminase [Mus musculus]
mmu-miR-1187	NM_001161807	-29.1	0.183491	-26.43	176	GO:0005930,GO:0043232,	-	gi|148705044|gb|EDL36991.1|	2.21E-82	cystin 1 [Mus musculus]
mmu-miR-1187	NM_001161822	-35.1	0.091112	-94.47	565	GO:0043231,GO:0016020,GO:0008047,GO:0060089,GO:0043087,GO:0007166,	K07524|1|9e-26|121|dre:445235|regulator of G-protein signalling 3	gi|240120077|ref|NP_001155294.1|	2.45E-111	regulator of G-protein signaling 17 isoform 1 [Mus musculus]
mmu-miR-1187	NM_001162938	-36.6	0.013595	-33.92	197	-	K10955|1|3e-07|58.5|tgu:100220116|intestinal mucin-2!K04989|3|1e-06|56.2|rno:100364048|polycystin 1L3!K13908|4|7e-06|53.9|cfa:483656|	gi|242397472|ref|NP_001156410.1|	0	pyrin domain-containing protein 3 [Mus musculus]

mmu-miR-1187	NM_001163145	-25.3	0.976843	-81.58	550	GO:0031224,	-	gi|148672505|gb|EDL04452.1|	4.93E-98	mCG141069 [Mus musculus]
mmu-miR-1187	NM_001163256	-28.4	0.404263	-55.71	362	GO:0043227,GO:0044459,GO:0043232,GO:0044444,GO:0046872,GO:0003676,GO:0065008,GO:0034330,	K06273|1|4e-57|224|mcc:706407|zyxin	gi|110625770|ref|NP_598515.3|	0	filamin-binding LIM protein 1 [Mus musculus]

mmu-miR-1187	NM_001164259	-27.6	0.428055	-27.5	181	GO:0031224,GO:0043231,GO:0031410,GO:0005539,GO:0019838,GO:0004714,GO:0016049,GO:0006796,GO:0008543,GO:0003170,GO:0008283,GO:0060411,GO:0060538,	K01217|1|1e-137|479|ecb:100146682|L-iduronidase [EC:3.2.1.76]!K05094|4|6e-37|156|xtr:100196923|fibroblast growth factor receptor 3 [EC:2.7.10.1]!K05095|5|1e-34|149|acs:100554153|fibroblast growth factor receptor 4	gi|256000817|ref|NP_001157731.1|	0	fibroblast growth factor receptor-like 1 isoform 2 precursor [Mus musculus]
mmu-miR-1187	NM_001081395	-20	1	-162.38	948	GO:0044424,GO:0043296,GO:0005515,	K06104|1|0.0|1605|mmu:75723|angiomotin like 1	gi|124487107|ref|NP_001074864.1|	0	angiomotin-like protein 1 [Mus musculus]

mmu-miR-1188-3p	NM_001001160	-26.8	0.998493	-36.07	166	GO:0005488,	K10316|1|0.0|1285|mmu:330369|F-box protein	gi|296482725|gb|DAA24840.1|	0	F-box protein 41-like [Bos taurus]
mmu-miR-1188-3p	NM_001001326	-28.1	0.944004	-28.08	154	GO:0005488,GO:0043410,	K12861|1|9e-165|582|gga:419884|pre-mRNA-splicing factor SPF27!K01289|2|8e-28|127|dme:Dmel_CG18659|carboxypeptidase [EC:3.4.16.	gi|47679093|ref|NP_001001326.1|	0	suppression of tumorigenicity 5 protein isoform 1 [Mus musculus]
mmu-miR-1188-3p	NM_001001983	-28.8	0.95958	-28.31	154	GO:0000139,GO:0016301,GO:0046488,GO:0048017,	K00888|1|0.0|3944|mmu:224020|phosphatidylinositol 4-kinase [EC:2.7.1.67]	gi|241982771|ref|NP_001001983.2|	0	phosphatidylinositol 4-kinase type 3 alpha [Mus musculus]
mmu-miR-1188-3p	NM_001001985	-27	0.997618	-31.83	162	GO:0005789,GO:0005740,GO:0008080,	-	gi|134288912|ref|NP_001001985.3|	4.43E-124	N-acetylaspartate synthetase [Mus musculus]
mmu-miR-1188-3p	NM_001003949	-39.2	0.023092	-35.49	173	GO:0031224,	-	gi|53759066|ref|NP_001003949.2|	0	membralin [Mus musculus]

mmu-miR-1188-3p	NM_001004152	-30.1	0.463045	-31.41	154	GO:0009690,GO:0002376,GO:0022414,	K06499|1|9e-116|418|rno:24926|carcinoembryonic antigen-related cell adhesion molecule	gi|148691137|gb|EDL23084.1|	0	mCG3735 [Mus musculus]
mmu-miR-1188-3p	NM_001004455	-29.1	0.574513	-29.5	150	GO:0005930,GO:0043232,	-	gi|148705044|gb|EDL36991.1|	2.17E-82	cystin 1 [Mus musculus]
mmu-miR-1188-3p	NM_001005868	-25	0.999995	-48.21	315	GO:0043231,GO:0009925,GO:0005578,GO:0005102,GO:0005198,GO:0008105,GO:0035088,GO:0040007,GO:0007010,GO:0007169,GO:0006886,	K12796|1|0.0|2752|mmu:59079|erbb2-interacting protein	gi|54607114|ref|NP_001005868.1|	0	protein LAP2 isoform 1 [Mus musculus]
mmu-miR-1188-3p	NM_001007571	-32	0.078671	-30.04	156	-	-	gi|56090602|ref|NP_001007572.1|	1.11E-34	leydig cell tumor 10 kDa protein homolog [Mus musculus]
mmu-miR-1188-3p	NM_001008423	-25.4	0.999586	-72.15	444	-	-	gi|119601406|gb|EAW81000.1|	2.12E-66	hCG1644165 [Homo sapiens]

mmu-miR-1188-3p	NM_001008791	-26.8	0.987819	-25.14	156	GO:0030425,GO:0044297,GO:0015629,GO:0005902,GO:0002141,GO:0031513,GO:0030427,GO:0005515,GO:0001894,GO:0050954,GO:0030030,GO:0050953,	K12076|1|1e-16|90.1|ssc:100157729|disks large protein 1!K06095|2|3e-16|89.0|tad:TRIADDRAFT_27973|multiple PDZ domain protein	gi|57012338|ref|NP_001008791.1|	0	whirlin isoform 2 [Mus musculus]

mmu-miR-1188-3p	NM_001008792	-26.8	0.987584	-25.14	156	GO:0030425,GO:0044297,GO:0015629,GO:0005902,GO:0002141,GO:0031513,GO:0030427,GO:0005515,GO:0001894,GO:0050954,GO:0030030,GO:0050953,	K13358|1|6e-17|91.3|xla:399076|Na(+)/H(+) exchange regulatory cofactor NHE-RF2!K12076|2|1e-16|90.1|ssc:100157729|disks large protein 1!K06095|4|3e-16|89.0|tad:TRIADDRAFT_27973|multiple PDZ domain	gi|57862816|ref|NP_001008792.1|	0	whirlin isoform 3 [Mus musculus]

mmu-miR-1188-3p	NM_001008793	-26.8	0.987412	-25.14	156	GO:0030425,GO:0044297,GO:0015629,GO:0005902,GO:0002141,GO:0031513,GO:0030427,GO:0005515,GO:0001894,GO:0050954,GO:0030030,GO:0050953,	K12076|1|1e-16|90.1|ssc:100157729|disks large protein 1!K06095|2|3e-16|89.0|tad:TRIADDRAFT_27973|multiple PDZ domain protein	gi|28972778|dbj|BAC65805.1|	0	mKIAA1526 protein [Mus musculus]
mmu-miR-1188-3p	NM_001009573	-29.5	0.805233	-26.54	157	GO:0000323,GO:0030133,GO:0005768,GO:0005488,GO:0002886,GO:0002323,GO:0017157,GO:0006897,GO:0002460,	K15621|1|0.0|1792|ecb:100059416|BAI1-associated protein 3	gi|62243588|ref|NP_001009573.2|	0	protein unc-13 homolog D [Mus musculus]
mmu-miR-1188-3p	NM_001009929	-37.8	0.082597	-36.45	166	GO:0046872,GO:0004672,GO:0032559,GO:0048858,GO:0030154,GO:0007163,GO:00064	K08796|1|0.0|1273|mmu:75770|BR serine/threonine kinase [EC:2.7.11.1]	gi|58036483|ref|NP_001009929.1|	0	serine/threonine-protein kinase BRSK2 isoform beta [Mus musculus]

mmu-miR-1188-3p	NM_001009930	-37.8	0.090759	-36.45	166	GO:0046872,GO:0004672,GO:0032559,GO:0030154,GO:0007163,GO:0006464,	K08796|1|0.0|1290|rno:293631|BR serine/threonine kinase [EC:2.7.11.1]	gi|58036485|ref|NP_001009930.1|	0	serine/threonine-protein kinase BRSK2 isoform gamma [Mus musculus]
mmu-miR-1188-3p	NM_001010941	-26.7	0.949539	-57.96	303	GO:0016021,GO:0045125,GO:0007166,GO:0006875,	K04315|1|3e-178|626|mmu:14738|G protein-coupled receptor 12	gi|6680061|ref|NP_032177.1|	2.06E-177	G-protein coupled receptor 12 [Mus musculus]
mmu-miR-1188-3p	NM_001013384	-26.5	0.935095	-27.06	158	GO:0031012,	K08764|1|1e-117|424|cfa:479564|sterol carrier protein 2 [EC:2.3.1.176]	gi|160419223|sp|Q6P3Y9.2|PONL1_MOUSE	0	RecName: Full=Podocan-like protein 1; Flags: Precursor
mmu-miR-1188-3p	NM_001017959	-32.8	0.184108	-30.78	162	GO:0005768,GO:0030667,GO:0030666,GO:0005764,GO:0031224,	K06528|1|0.0|738|mmu:16784|lysosomal-associated membrane protein 1/2	gi|63054837|ref|NP_001017959.1|	0	lysosome-associated membrane glycoprotein 2 isoform 1 precursor [Mus musculus]

mmu-miR-1188-3p	NM_001024136	-27	0.974471	-51.64	314	GO:0044424,GO:0042802,	K15503|1|9e-38|160|spu:584964|serine/threonine-protein phosphatase 6 regulatory ankyrin repeat subunit B	gi|67906181|ref|NP_001019307.1|	0	ankyrin repeat and SAM domain-containing protein 6 [Mus musculus]
mmu-miR-1188-3p	NM_001024139	-31.3	0.714729	-29.87	161	GO:0031012,GO:0046914,GO:0004175,GO:0019538,	K08629|1|0.0|1966|mmu:235130|a disintegrin and metalloproteinase with thrombospondin motifs 15 [EC:3.4.24.-]	gi|71725383|ref|NP_001019310.1|	0	A disintegrin and metalloproteinase with thrombospondin motifs 15 precursor [Mus musculus]

mmu-miR-1188-3p	NM_001024147	-26.8	0.804272	-25.7	153	GO:0031224,GO:0005343,GO:0015718,GO:0035725,	K14344|1|2e-66|246|mmu:231290|solute carrier family 10 (sodium/bile acid cotransporter), member 4	gi|85701520|ref|NP_001019318.1|	2.87E-68	uncharacterized protein LOC545758 [Mus musculus]
mmu-miR-1188-3p	NM_001024468	-31	0.83156	-25.23	159	GO:0043231,GO:0044444,GO:0005515,GO:0004084,GO:0008652,GO:0009063,	K00826|1|0.0|931|mmu:12035|branched-chain amino acid aminotransferase [EC:2.6.1.42]	gi|66792792|ref|NP_001019639.1|	0	branched-chain-amino-acid aminotransferase, cytosolic isoform 1 [Mus musculus]
mmu-miR-1188-3p	NM_001024703	-29.3	0.915304	-28.2	151	GO:0031224,GO:0046872,GO:0005543,GO:0019932,	K15293|1|3e-11|72.8|tad:TRIADDRAFT_22095|protein unc-13	gi|85701536|ref|NP_001019874.1|	0	multiple C2 and transmembrane domain-containing protein 2 [Mus musculus]
mmu-miR-1188-3p	NM_001024806	-26.7	0.990038	-27.67	154	GO:0043231,GO:0003676,GO:0006351,	K14832|1|0.0|1803|mmu:12607|ribosome biogenesis protein MAK21	gi|67188876|ref|NP_001019977.1|	0	CCAAT/enhancer-binding protein zeta [Mus musculus]

mmu-miR-1188-3p	NM_001025307	-28.6	0.834454	-29.12	150	GO:0031224,GO:0030054,GO:0043234,GO:0005766,GO:0042995,GO:0030141,GO:0030427,GO:0050543,GO:0005515,GO:0006979,GO:0030030,GO:0016044,GO:0006904,GO:00	K08486|1|5e-145|516|mmu:20908|syntaxin 1B/2/3	gi|70778802|ref|NP_001020478.1|	3.84E-144	syntaxin-3 isoform B [Mus musculus]

mmu-miR-1188-3p	NM_001025372	-25.9	0.999791	-26.91	152	GO:0043296,GO:0016021,GO:0042995,GO:0005783,GO:0004930,GO:0031267,GO:0042277,GO:0010676,GO:0045762,GO:0010941,GO:0045137,GO:0043627,GO:0019935,GO:0010863,GO:0007169,GO:0048869,GO:0048232,GO:0010522,GO:00069	K04587|1|0.0|903|mmu:11517|adenylate cyclase activating polypeptide 1 (pituitary) receptor type I	gi|70780370|ref|NP_001020543.1|	0	pituitary adenylate cyclase-activating polypeptide type I receptor isoform 2 precursor [Mus musculus]
mmu-miR-1188-3p	NM_001025599	-26.1	0.990593	-26.76	150	GO:0044424,GO:0003676,GO:0046914,	K12008|1|0.0|935|mmu:22670|tripartite motif-containing protein 26	gi|128485541|ref|NP_001020770.2|	0	tripartite motif-containing protein 26 isoform a [Mus musculus]
mmu-miR-1188-3p	NM_001029836	-29.4	0.794659	-28.97	170	-	K06824|1|0.0|1041|mmu:114249|nephronectin	gi|71067128|ref|NP_001025007.1|	0	nephronectin isoform b precursor [Mus musculus]

mmu-miR-1188-3p	NM_001029933	-26.3	0.957364	-29.78	156	GO:0005622,GO:0043169,GO:0003676,GO:0006351,	K09228|1|0.0|1221|mmu:232966|KRAB domain-containing zinc finger protein	gi|113930749|ref|NP_001025104.2|	0	zinc finger protein 114 [Mus musculus]
mmu-miR-1188-3p	NM_001029979	-31.3	0.508864	-27.59	162	-	K14946|1|3e-09|65.5|cbr:CBG20166|RNA binding protein fox-1	gi|148706226|gb|EDL38173.1|	0	scaffold attachment factor B2, isoform CRA_b [Mus musculus]
mmu-miR-1188-3p	NM_001030297	-38.5	0.019427	-30	154	GO:0016021,GO:0004175,GO:0019538,	K14359|1|0.0|851|mmu:435845|transmembrane protease, serine 11C [EC:3.4.21.	gi|71892422|ref|NP_001025468.1|	0	transmembrane protease, serine 11c [Mus musculus]
mmu-miR-1188-3p	NM_001033135	-30.2	0.544324	-27.09	157	GO:0016020,GO:0046872,	K15704|1|0.0|694|mmu:67702|E3 ubiquitin-protein ligase RNF149 [EC:6.3.2.19]	gi|224809379|ref|NP_001028307.2|	0	E3 ubiquitin-protein ligase RNF149 precursor [Mus musculus]

mmu-miR-1188-3p	NM_001033167	-28.1	0.98142	-26.62	151	GO:0031224,GO:0005215,GO:0006810,	K08215|1|0.0|1262|mmu:73102|MFS transporter, OCT family, solute carrier family 22 (organic anion transporter), member 23	gi|85701678|ref|NP_001028339.1|	0	solute carrier family 22 member 23 [Mus musculus]
mmu-miR-1188-3p	NM_001033205	-28.5	0.811377	-25.27	153	GO:0043231,GO:0046914,GO:0003676,GO:0010467,	K09228|1|4e-29|130|pon:100442553|KRAB domain-containing zinc finger protein	gi|85701718|ref|NP_001028377.1|	2.12E-122	zinc finger protein 575 [Mus musculus]
mmu-miR-1188-3p	NM_001033222	-32.6	0.609011	-31.6	155	GO:0043169,GO:0007165,	K10360|1|1e-06|58.2|mdo:100013745|myosin IX	gi|164698472|ref|NP_001028394.2|	0	PDZ domain containing 8 [Mus musculus]
mmu-miR-1188-3p	NM_001033225	-38.9	0.021494	-60.69	312	-	-	gi|113461982|ref|NP_001028397.2|	1.40E-130	proline-rich nuclear receptor coactivator 1 [Mus musculus]

mmu-miR-1188-3p	NM_001033273	-27.6	0.981165	-26.18	142	GO:0005488,	-	gi|85701776|ref|NP_001028445.1|	0	uncharacterized protein KIAA0930 homolog [Mus musculus]
mmu-miR-1188-3p	NM_001033274	-28.7	0.935491	-73.26	441	GO:0070775,GO:0046914,GO:0016573,	K11349|1|0.0|2110|mmu:223770|bromodomain-containing protein 1	gi|164698417|ref|NP_001028446.2|	0	bromodomain containing 1 [Mus musculus]
mmu-miR-1188-3p	NM_001033299	-25.5	0.999894	-33.46	165	GO:0031981,GO:0046872,GO:0043565,GO:0001071,GO:0007568,GO:0010467,	K09228|1|2e-24|116|dre:100148223|KRAB domain-containing zinc finger protein	gi|75677492|ref|NP_001028471.1|	0	zinc finger protein 217 [Mus musculus]
mmu-miR-1188-3p	NM_001033314	-28.5	0.707786	-29.1	152	-	-	gi|148691146|gb|EDL23093.1|	0	RIKEN cDNA C530028I08, isoform CRA_b [Mus musculus]
mmu-miR-1188-3p	NM_001033380	-30.6	0.838607	-30.95	156	GO:0031224,	-	gi|83921560|ref|NP_001028552.1|	0	inositol 1,4,5-trisphosphate receptor-interacting protein-like 2 precursor [Mus musculus]

mmu-miR-1188-3p	NM_001033383	-26.1	0.996179	-27.19	149	GO:0005488,	K09228|1|4e-62|241|ecb:100062322|KRAB domain-containing zinc finger protein	gi|75677466|ref|NP_001028555.1|	0	zinc finger protein 865 [Mus musculus]
mmu-miR-1188-3p	NM_001033415	-27	0.978644	-25.26	151	GO:0031224,GO:0031090,GO:0043231,GO:0032501,	-	gi|85986585|ref|NP_001028587.1|	7.41E-113	protein shisa-3 homolog precursor [Mus musculus]
mmu-miR-1188-3p	NM_001033445	-31.9	0.562822	-29.33	144	-	-	gi|134288888|ref|NP_001028617.2|	0	protein FAM59A [Mus musculus]
mmu-miR-1188-3p	NM_001033451	-31.8	0.48964	-29.42	162	GO:0005622,GO:0046872,GO:0005515,	K09228|1|1e-70|269|ptr:742564|KRAB domain-containing zinc finger protein	gi|110626129|ref|NP_001028623.1|	0	zinc finger protein 408 [Mus musculus]
mmu-miR-1188-3p	NM_001033534	-28.2	0.70624	-26.59	155	GO:0031224,GO:0005924,GO:0031252,GO:0005539,	K10077|1|0.0|744|mmu:244864|layilin	gi|226958426|ref|NP_001028706.1|	0	layilin precursor [Mus musculus]

mmu-miR-1188-3p	NM_001033542	-28.6	0.753303	-27.95	152	GO:0016020,GO:0015299,GO:0015893,GO:0006818,GO:0006812,	K03327|1|0.0|995|mmu:380701|multidrug resistance protein, MATE family	gi|85702045|ref|NP_001028714.1|	0	multidrug and toxin extrusion protein 2 [Mus musculus]
mmu-miR-1188-3p	NM_001033573	-30	0.549168	-26.94	163	GO:0031224,GO:0046914,GO:0016747,	K05766|1|2e-14|82.0|spu:575378|slingshot	gi|148669781|gb|EDL01728.1|	0	mCG18121, isoform CRA_c [Mus musculus]
mmu-miR-1188-3p	NM_001033630	-29.2	0.43093	-29.31	154	-	-	gi|148691298|gb|EDL23245.1|	1.95E-129	mCG141136, isoform CRA_b [Mus musculus]
mmu-miR-1188-3p	NM_001033805	-28.4	0.532268	-27.77	163	GO:0031224,	-	gi|85702141|ref|NP_001028977.1|	3.73E-109	transmembrane protein C14orf176 homolog [Mus musculus]
mmu-miR-1188-3p	NM_001033922	-25.4	0.970449	-25.26	150	GO:0023052,	K06742|1|8e-25|115|ecb:100066291|natural cytotoxicity triggering receptor 2	gi|148691659|gb|EDL23606.1|	1.22E-138	triggering receptor expressed on myeloid cells-like 4, isoform CRA_c [Mus musculus]
mmu-miR-1188-3p	NM_001034100	-29.1	0.187891	-25.64	162	GO:0000785,GO:0043231,GO:0003676,	K11251|1|4e-47|187|mmu:385328|histone H2A	gi|77539462|ref|NP_001029272.1|	2.12E-46	uncharacterized protein LOC385328 [Mus musculus]

mmu-miR-1188-3p	NM_001034904	-33.4	0.144933	-30.15	169	GO:0016740,GO:0032559,GO:0006464,	K08798|1|2e-66|254|xtr:100329119|MAP/microtubule affinity-regulating kinase [EC:2.7.11.	gi|85702264|ref|NP_001030076.1|	5.95E-178	serine/threonine kinase-like [Mus musculus]
mmu-miR-1188-3p	NM_001037136	-25.6	0.999997	-29.32	157	GO:0044424,GO:0005083,GO:0008047,GO:0046914,GO:0032561,GO:0006810,GO:0043087,GO:0032012,	K12491|1|0.0|1498|mmu:347722|Arf-GAP, GTPase, ANK repeat and PH domain-containing protein 1/2/3	gi|80978937|ref|NP_001032213.1|	0	arf-GAP with GTPase, ANK repeat and PH domain-containing protein 1 isoform 2 [Mus musculus]
mmu-miR-1188-3p	NM_001037717	-29.2	0.749438	-26.26	150	GO:0031224,GO:0030001,GO:0015837,	K14993|1|0.0|813|mmu:625098|solute carrier family 38 (sodium-coupled neutral amino acid transporter), member 6	gi|241896953|ref|NP_001032806.2|	0	solute carrier family 38, member 6 [Mus musculus]

mmu-miR-1188-3p	NM_001037736	-40.3	0.047091	-41.22	178	GO:0044464,GO:0005088,GO:0019222,GO:0007266,	K05156|1|1e-24|117|gga:419067|tumor necrosis factor receptor superfamily member 19-like!K08878|2|2e-20|103|dre:560226|breakpoint cluster region protein [EC:2.7.11.1]!K04436|3|6e-20|101|ssc:100521718|mitogen-activated protein	gi|60360312|dbj|BAD90400.1|	0	mKIAA0294 protein [Mus musculus]

mmu-miR-1188-3p	NM_001037764	-32.4	0.636604	-32.18	147	GO:0043231,GO:0046914,GO:0035264,GO:0048731,GO:0006355,	K09188|1|3e-13|79.7|dme:Dmel_CG3848|histone-lysine N-methyltransferase MLL3 [EC:2.1.1.43]	gi|83649745|ref|NP_001032853.1|	0	retinoic acid-induced protein 1 [Mus musculus]
mmu-miR-1188-3p	NM_001037865	-26.8	0.989659	-25.61	146	GO:0005578,GO:0030414,GO:0010466,	K06238|1|7e-43|177|cfa:100856491|collagen, type VI, alpha	gi|83745131|ref|NP_001032954.1|	0	collagen alpha-1(XXVIII) chain precursor [Mus musculus]
mmu-miR-1188-3p	NM_001038602	-32.3	0.365827	-27.5	162	GO:0031224,GO:0043296,GO:0050954,	K15183|1|9e-20|100|mgp:100549853|RNA polymerase II elongation factor ELL	gi|84370242|ref|NP_001033691.1|	0	MARVEL domain-containing protein 2 isoform 1 [Mus musculus]
mmu-miR-1188-3p	NM_001038624	-32.1	0.573231	-30.48	161	GO:0043231,GO:0016020,GO:0005102,GO:0051480,GO:0007268,	-	gi|84490396|ref|NP_001033713.1|	9.63E-154	protein RIC-3 isoform a precursor [Mus musculus]

mmu-miR-1188-3p	NM_001039059	-34.4	0.194016	-31.71	164	-	K10452|1|1e-120|435|mmu:236904|kelch-like protein	gi|23346619|ref|NP_694805.1|	6.72E-120	kelch-like 15 isoform c [Mus musculus]
mmu-miR-1188-3p	NM_001039060	-34.4	0.245646	-31.71	164	-	K10452|1|1e-120|436|ecb:100052043|kelch-like protein	gi|84794653|ref|NP_001034149.1|	3.68E-126	kelch-like 15 isoform b [Mus musculus]
mmu-miR-1188-3p	NM_001039061	-36.7	0.063172	-31.71	164	-	K10452|1|0.0|1148|rno:314111|kelch-like protein	gi|84794655|ref|NP_001034150.1|	0	kelch-like 15 isoform a [Mus musculus]
mmu-miR-1188-3p	NM_001039071	-25	0.999938	-25.26	148	GO:0031674,GO:0046914,GO:0019901,	K05760|1|4e-42|175|isc:IscW_ISCW009899|paxillin	gi|28144143|gb|AAO26188.1|	0	PDZ-LIM protein cypher3c [Mus musculus]

mmu-miR-1188-3p	NM_001039076	-25	0.979359	-25.26	148	GO:0042995,GO:0031674,GO:0046914,GO:0019901,	K05629|1|3e-07|57.4|pon:100443388|atrophin-1 interacting protein 1!K05631|3|7e-07|56.2|ecb:100049816|atrophin-1 interacting protein 3 (BAI1-associated protein 1)!K01104|4|1e-06|55.8|cel:C48D5.2|protein-tyrosine phosphatase [EC:3.1.3.48]!K08202	gi|148692936|gb|EDL24883.1|	7.92E-169	LIM domain binding 3, isoform CRA_b [Mus musculus]
mmu-miR-1188-3p	NM_001039106	-29.7	0.848048	-32.88	156	GO:0043169,GO:0003824,GO:0006629,	K13619|1|0.0|1454|mmu:114874|phospholipase DDHD1 [EC:3.1.1.-]	gi|148688758|gb|EDL20705.1|	0	DDHD domain containing 1, isoform CRA_b [Mus musculus]

mmu-miR-1188-3p	NM_001039158	-33.8	0.250321	-32.33	158	GO:0031090,GO:0043231,GO:0031224,GO:0019787,GO:0032559,GO:0006464,GO:0006950,	K04554|1|2e-131|471|mmu:140499|ubiquitin-conjugating enzyme E2 J2 [EC:6.3.2.19]	gi|148683112|gb|EDL15059.1|	1.48E-135	ubiquitin-conjugating enzyme E2, J2 homolog (yeast), isoform CRA_c [Mus musculus]
mmu-miR-1188-3p	NM_001039179	-32.3	0.610076	-28.7	155	GO:0031982,GO:0043232,GO:0043231,GO:0016020,GO:0005083,GO:0005488,GO:0019222,GO:0034453,GO:0072384,	K11498|1|4e-16|89.4|mdo:100013897|centromeric protein E!K10352|4|3e-14|83.2|aml:100473449|myosin heavy chain	gi|28972353|dbj|BAC65630.1|	0	mKIAA0699 protein [Mus musculus]

mmu-miR-1188-3p	NM_001039385	-25	0.996945	-36.96	151	GO:0044297,GO:0042995,GO:0030141,GO:0044421,GO:0005179,GO:0022602,GO:0007620,GO:0048167,GO:0043434,GO:0030154,GO:0030072,GO:0044237,GO:0006950,GO:00	-	gi|86476054|ref|NP_001034474.1|	8.75E-131	VGF nerve growth factor inducible precursor [Mus musculus]
mmu-miR-1188-3p	NM_001039493	-36.3	0.273707	-60.21	330	GO:0046914,GO:0007165,	-	gi|148667794|gb|EDL00211.1|	0	mCG123132, isoform CRA_b [Mus musculus]
mmu-miR-1188-3p	NM_001039520	-26.4	0.989879	-25.88	156	GO:0032991,GO:0015630,GO:0044459,GO:0030139,GO:0012505,GO:0044430,GO:0017111,GO:0019900,GO:0032561,GO:0009207,GO:0048193,GO:0006898,GO:00150	K01528|1|0.0|1499|mmu:13430|dynamin GTPase [EC:3.6.5.5]	gi|87299637|ref|NP_001034609.1|	0	dynamin-2 isoform 2 [Mus musculus]

mmu-miR-1188-3p	NM_001042488	-27.8	0.898585	-28.77	155	GO:0044297,GO:0043005,GO:0016020,GO:0005515,	K15008|1|9e-76|286|mdo:100012355|discs, large-associated protein 1	gi|74151726|dbj|BAE29656.1|	0	unnamed protein product [Mus musculus]
mmu-miR-1188-3p	NM_001042502	-27.3	0.891923	-29.61	148	GO:0043231,GO:0043234,GO:0005515,GO:0001071,GO:0003677,GO:0001525,GO:0007166,GO:0035051,GO:0021536,GO:0048845,GO:0006357,GO:0043010,GO:0008283,GO:0009855,GO:0030326,GO:0043009,GO:0055008,GO:0007519,GO:0060425,GO:00	K04686|1|2e-145|517|mmu:18741|paired-like homeodomain transcription factor 2	gi|109948275|ref|NP_001035967.1|	1.23E-144	pituitary homeobox 2 isoform c [Mus musculus]

mmu-miR-1188-3p	NM_001042503	-29.2	0.901838	-54.85	305	GO:0044424,GO:0046914,GO:0032501,	K12035|1|0.0|1472|mmu:636931|tripartite motif-containing protein	gi|109948300|ref|NP_001035968.1|	0	tripartite motif-containing protein 71 [Mus musculus]
mmu-miR-1188-3p	NM_001042528	-29.6	0.925922	-29.66	161	GO:0034704,GO:0044297,GO:0030425,GO:0033267,GO:0005515,GO:0005262,GO:0032559,GO:0033555,GO:0007269,GO:0006816,GO:0008015,GO:0044057,GO:0032412,GO:0048545,GO:0007610,GO:00423	K04849|1|0.0|4145|mmu:12287|voltage-dependent calcium channel N type alpha-1B	gi|110225368|ref|NP_001035993.1|	0	voltage-dependent N-type calcium channel subunit alpha-1B isoform 1 [Mus musculus]
mmu-miR-1188-3p	NM_001042556	-33.3	0.160286	-30.56	164	GO:0031981,GO:0005488,	K14847|1|4e-158|558|rno:294436|ribosome productio	gi|7670547|dbj|BAA95117.1|	4.16E-161	unnamed protein product [Mus musculus]

mmu-miR-1188-3p	NM_001042719	-29.7	0.857216	-32.88	156	-	K13619|1|0.0|1420|mmu:114874|phospholipase DDHD1 [EC:3.1.1.-]	gi|111955212|ref|NP_001036184.1|	0	phospholipase DDHD1 isoform 3 [Mus musculus]
mmu-miR-1188-3p	NM_001045520	-26	0.99383	-26.62	151	-	K11137|1|9e-67|256|api:100163744|telomere length regulation protein!K12471|3|6e-65|250|isc:IscW_ISCW008441|epsin	gi|154240730|ref|NP_001038985.2|	0	clathrin interactor 1 [Mus musculus]
mmu-miR-1188-3p	NM_001045530	-32.6	0.296647	-29.4	154	-	K06627|1|1e-09|66.2|hmg:100206919|cyclin A!K05868|4|2e-07|59.3|nvi:100122223|cyclin	gi|113865891|ref|NP_001038995.1|	0	cyclin-J-like protein [Mus musculus]

mmu-miR-1188-3p	NM_001045555	-26	0.952495	-31.23	156	-	K09448|1|3e-88|327|cfa:481758|TEA domain family member 1/3/4	gi|113930728|ref|NP_001039020.1|	0	tubby-related protein 2 isoform 2 [Mus musculus]
mmu-miR-1188-3p	NM_001048177	-27.8	0.97397	-43.66	296	GO:0034399,GO:0043232,GO:0044459,GO:0044444,GO:0005506,GO:0051427,GO:0035400,GO:0019900,GO:0032559,GO:0005126,GO:0019904,GO:0031958,GO:0042506,GO:0007162,GO:0032611,GO:0007169,GO:0035406,GO:0031349,GO:0008016,GO:0043523,GO:0051100,GO:00099	K04447|1|0.0|2272|mmu:16452|Janus kinase 2 [EC:2.7.10.2]	gi|114326478|ref|NP_001041642.1|	0	tyrosine-protein kinase JAK2 [Mus musculus]

mmu-miR-1188-3p	NM_001048206	-30.9	0.807589	-28.83	153	GO:0043231,GO:0005887,GO:0004871,GO:0046914,GO:0019787,GO:0019900,GO:0002697,GO:0043433,GO:0019221,GO:0032479,GO:0006915,GO:0032446,GO:0002221,GO:00	K03174|1|0.0|1104|mmu:22031|TNF receptor-associated factor 3	gi|114842403|ref|NP_001041671.1|	0	TNF receptor-associated factor 3 isoform b [Mus musculus]
mmu-miR-1188-3p	NM_001077363	-28.6	0.821425	-28.63	152	-	K13218|1|0.0|979|mmu:19205|polypyrimidine tract-binding protein 1	gi|116517301|ref|NP_001070831.1|	0	polypyrimidine tract-binding protein 1 isoform 1 [Mus musculus]
mmu-miR-1188-3p	NM_001081103	-28.3	0.952534	-29.88	148	GO:0031224,GO:0043231,GO:0031090,GO:0046872,GO:0016247,GO:0006875,	K16059|1|0.0|1236|mmu:116873|stromal interaction molecule	gi|219519777|gb|AAI45002.1|	0	Stim2 protein [Mus musculus]

mmu-miR-1188-3p	NM_001081117	-26.5	0.99995	-28.87	148	GO:0005634,GO:0005515,	K09228|1|7e-27|125|pon:100434122|KRAB domain-containing zinc finger protein	gi|169234624|ref|NP_001074586.2|	0	antigen KI-67 [Mus musculus]
mmu-miR-1188-3p	NM_001081125	-26	0.999835	-27.09	154	GO:0072372,GO:0043231,GO:0001071,GO:0043565,GO:0046914,GO:0030326,GO:0007224,GO:0001763,GO:0006357,GO:0021520,GO:0021521,GO:0048732,GO:0043009,GO:0021952,GO:0002062,GO:0014070,GO:0007418,GO:0033077,GO:0042476,GO:0048565,GO:0008544,GO:00215	K06230|1|0.0|2855|mmu:14633|zinc finger protein GLI	gi|124487481|ref|NP_001074594.1|	0	zinc finger protein GLI2 [Mus musculus]

mmu-miR-1188-3p	NM_001081139	-31	0.621879	-25.91	152	-	K15502|1|2e-26|122|mmu:105522|serine/threonine-protein phosphatase 6 regulatory ankyrin repeat subunit A	gi|124487271|ref|NP_001074608.1|	0	ankyrin repeat domain-containing protein 35 [Mus musculus]
mmu-miR-1188-3p	NM_001081150	-31.6	0.532264	-48.83	298	GO:0046914,GO:0042623,GO:0019538,	K12015|1|4e-09|65.1|xtr:100327256|tripartite motif-containing protein 39!K15697|4|5e-08|61.6|dre:414843|RING finger protein 114!K15684|5|8e-08|60.8|dre:562439|bifunctional apoptosis regulator	gi|124487241|ref|NP_001074619.1|	0	LON peptidase N-terminal domain and RING finger protein 1 [Mus musculus]

mmu-miR-1188-3p	NM_001081160	-30	0.91176	-32.12	150	GO:0031225,GO:0016477,GO:0021515,	K06255|1|5e-30|135|mmu:15530|heparan sulfate proteoglycan 2 (perlecan)	gi|124487209|ref|NP_001074629.1|	0	MAM domain-containing glycosylphosphatidylinositol anchor protein 1 precursor [Mus musculus]
mmu-miR-1188-3p	NM_001081168	-28.4	0.623678	-66.01	321	GO:0031981,GO:0043169,GO:0003676,GO:0010467,	-	gi|63102312|gb|AAH94930.1|	1.22E-94	Sap30l protein, partial [Mus musculus]
mmu-miR-1188-3p	NM_001081170	-30.3	0.808407	-25.79	154	GO:0044432,GO:0012501,GO:0051704,	-	gi|124487181|ref|NP_001074639.1|	0	phosphofurin acidic cluster sorting protein 2 [Mus musculus]
mmu-miR-1188-3p	NM_001081196	-37.3	0.1382	-33.65	161	GO:0043231,GO:0005488,	K12888|1|4e-126|454|ssc:100515571|heterogeneous nuclear ribonucleoprotein U	gi|124487099|ref|NP_001074665.1|	0	heterogeneous nuclear ribonucleoprotein U-like protein 2 [Mus musculus]
mmu-miR-1188-3p	NM_001081224	-27.2	0.892525	-30.67	147	-	-	gi|124487027|ref|NP_001074693.1|	1.67E-114	proline-rich protein 16 [Mus musculus]
mmu-miR-1188-3p	NM_001081226	-28.2	0.283107	-30.27	149	-	-	gi|124487011|ref|NP_001074695.1|	6.71E-76	uncharacterized protein LOC69549 [Mus musculus]

mmu-miR-1188-3p	NM_001081260	-32.6	0.539847	-55.2	309	GO:0044444,GO:0000784,GO:0008092,GO:0006278,GO:0000288,	-	gi|124486923|ref|NP_001074729.1|	0	182 kDa tankyrase-1-binding protein [Mus musculus]
mmu-miR-1188-3p	NM_001081290	-25.3	1	-25.2	147	-	-	gi|124486835|ref|NP_001074759.1|	0	protein PRRC2C [Mus musculus]
mmu-miR-1188-3p	NM_001081291	-29.1	0.900523	-27.48	153	GO:0044424,GO:0015631,GO:0007010,	K08408|1|2e-32|143|cfa:480418|G protein-coupled receptor 68!K06102|3|2e-07|59.7|aml:100476212|cingulin	gi|124486839|ref|NP_001074760.1|	0	coiled-coil domain-containing protein 88B precursor [Mus musculus]
mmu-miR-1188-3p	NM_001081295	-35.3	0.237228	-30.34	152	GO:0031252,GO:0005088,GO:0007266,	K13744|1|0.0|1661|mmu:622434|SH3 domain-containing guanine exchange factor	gi|124486829|ref|NP_001074764.1|	0	rho guanine nucleotide exchange factor 26 [Mus musculus]

mmu-miR-1188-3p	NM_001081382	-31.2	0.500881	-25.63	145	GO:0005622,GO:0046872,GO:0003676,	K09228|1|0.0|1533|mmu:72306|KRAB domain-containing zinc finger protein	gi|124487043|ref|NP_001074851.1|	0	zinc finger protein 777 [Mus musculus]
mmu-miR-1188-3p	NM_001040398	-20.7	1	-76.08	451	-	-	-	-	-
mmu-miR-1188-5p	NM_001004185	-32.6	0.128618	-32.39	159	GO:0044444,GO:0043229,	-	gi|60359958|dbj|BAD90198.1|	0	mKIAA1971 protein [Mus musculus]
mmu-miR-1188-5p	NM_001005342	-30.6	0.048597	-29.06	157	GO:0031981,	-	gi|52693937|ref|NP_001005342.1|	3.33E-73	protein yippee-like 4 [Mus musculus]
mmu-miR-1188-5p	NM_001005475	-25.1	0.993096	-26.38	163	GO:0044424,GO:0005083,GO:0032011,	K12495|1|0.0|1544|mmu:245666|IQ motif and SEC7 domain-containing protein	gi|350595687|ref|XP_003135163.3|	0	PREDICTED: IQ motif and SEC7 domain-containing protein 2 [Sus scrofa]
mmu-miR-1188-5p	NM_001007568	-26.5	0.860848	-71.96	446	GO:0005622,GO:0046872,	K09228|1|0.0|1314|mmu:71591|KRAB domain-containing zinc finger protein	gi|56090226|ref|NP_001007569.1|	0	zinc finger protein 251 [Mus musculus]

mmu-miR-1188-5p	NM_001008427	-25.8	0.907347	-42.46	298	-	K09228|1|0.0|1156|mmu:434179|KRAB domain-containing zinc finger protein	gi|148680477|gb|EDL12424.1|	0	mCG140430, isoform CRA_b [Mus musculus]
mmu-miR-1188-5p	NM_001008533	-28.9	0.706549	-29.25	161	GO:0033267,GO:0032589,GO:0044430,GO:0043231,GO:0044456,GO:0016021,GO:0044297,GO:0001882,GO:0004620,GO:0005102,GO:0004930,GO:0032403,GO:0046983,GO:0045986,GO:0048871,GO:0002790,GO:0032898,GO:0006470,GO:0035588,GO:0042321,GO:0050961,GO:00313	K04265|1|1e-140|503|mmu:11539|adenosine A1 receptor	gi|148707674|gb|EDL39621.1|	2.61E-144	mCG50903, isoform CRA_a [Mus musculus]

mmu-miR-1188-5p	NM_001009575	-32	0.148237	-29.95	158	-	K14284|1|1e-17|92.8|rno:680025|nuclear RNA export factor 1/2	gi|85662391|ref|NP_001009575.2|	0	armadillo repeat-containing X-linked protein 5 [Mus musculus]
mmu-miR-1188-5p	NM_001012638	-30.1	0.142138	-28.13	163	GO:0031981,GO:0000782,GO:0003676,GO:0019899,GO:0048731,GO:0035108,GO:0015031,GO:0034502,GO:0003002,GO:0016233,GO:0051347,GO:0007004,GO:00433	K11114|1|0.0|741|mmu:497652|adrenocortical dysplasia protein	gi|60593059|ref|NP_001012656.1|	0	adrenocortical dysplasia protein [Mus musculus]
mmu-miR-1188-5p	NM_001013376	-31.3	0.055253	-29	161	GO:0030681,GO:0031981,GO:0005488,GO:0004549,GO:0006399,	K14523|1|3e-136|485|mmu:227522|ribonucleases P/MRP protein subunit RPP38 [EC:3.1.26.5]	gi|247300905|ref|NP_001013394.2|	1.78E-135	ribonuclease P protein subunit p38 [Mus musculus]

mmu-miR-1188-5p	NM_001013749	-26.1	0.956456	-45.78	312	GO:0031224,	-	gi|85701444|ref|NP_001013771.1|	0	transmembrane protein 151B [Mus musculus]
mmu-miR-1188-5p	NM_001014288	-25.3	0.998229	-50.71	309	GO:0016021,GO:0004725,GO:0005488,GO:0004871,GO:0007167,GO:0006470,	K06777|1|0.0|2517|rno:313278|protein tyrosine phosphatase, receptor type, D [EC:3.1.3.48]	gi|90403603|ref|NP_035341.2|	0	receptor-type tyrosine-protein phosphatase delta isoform B precursor [Mus musculus]
mmu-miR-1188-5p	NM_001014836	-27.1	0.210419	-26.27	154	-	-	gi|148699471|gb|EDL31418.1|	1.44E-125	RIKEN cDNA 4930404N11, isoform CRA_a [Mus musculus]
mmu-miR-1188-5p	NM_001017959	-28.8	0.299448	-25.94	156	GO:0005768,GO:0030667,GO:0030666,GO:0005764,GO:0031224,	K06528|1|0.0|738|mmu:16784|lysosomal-associated membrane protein 1/2	gi|63054837|ref|NP_001017959.1|	0	lysosome-associated membrane glycoprotein 2 isoform 1 precursor [Mus musculus]
mmu-miR-1188-5p	NM_001024716	-28.3	0.491246	-44.91	295	GO:0043231,GO:0043232,GO:0019899,	K11498|1|1e-08|62.8|bta:281681|centromeric protein E	gi|88501745|ref|NP_001019887.1|	0	TRIO and F-actin-binding protein isoform 1 [Mus musculus]

mmu-miR-1188-5p	NM_001024931	-32.9	0.111791	-27.89	157	GO:0043231,GO:0044297,GO:0003676,GO:0006396,	K14946|1|3e-146|520|mmu:52897|RNA binding protein fox-1	gi|148702729|gb|EDL34676.1|	2.11E-147	DNA segment, Chr 11, Brigham & Women's Genetics 0517 expressed, isoform CRA_c [Mus musculus]
mmu-miR-1188-5p	NM_001024952	-30.1	0.756222	-28.31	162	GO:0044424,GO:0003676,GO:0046914,GO:0002698,	K15690|1|0.0|2014|mmu:381305|RING finger and CCCH-type zinc finger domain-containin	gi|50511255|dbj|BAD32613.1|	0	mKIAA2025 protein [Mus musculus]

mmu-miR-1188-5p	NM_001025084	-32.2	0.029471	-26.66	153	GO:0003676,	K09328|1|7e-09|61.6|bfo:BRAFLDRAFT_290641|homeobox protein Unc-4!K15607|2|8e-08|58.2|api:100573073|T-cell leukemia homeobox protein 3!K15606|4|4e-07|55.8|bta:539041|T-cell leukemia homeobox protein 2	gi|115311558|ref|NP_001020255.2|	4.24E-116	reproductive homeobox 1 [Mus musculus]
mmu-miR-1188-5p	NM_001025106	-30	0.467522	-47.84	316	GO:0016020,	-	gi|68342032|ref|NP_001020277.1|	0	transmembrane protein 201 isoform a [Mus musculus]

mmu-miR-1188-5p	NM_001025561	-33	0.059516	-28.85	159	GO:0043234,GO:0031981,GO:0003676,GO:0016866,GO:0003713,GO:0016836,GO:0006357,GO:0006399,GO:0001522,	K06173|1|0.0|919|mmu:56361|tRNA pseudouridine38-40 synthase [EC:5.4.99.12]	gi|148688057|gb|EDL20004.1|	0	pseudouridine synthase 1, isoform CRA_b [Mus musculus]
mmu-miR-1188-5p	NM_001025562	-33	0.04572	-28.85	159	GO:0043234,GO:0031981,GO:0003676,GO:0016866,GO:0003713,GO:0016836,GO:0006357,GO:0006399,GO:0001522,	K06173|1|0.0|805|rno:304567|tRNA pseudouridine38-40 synthase [EC:5.4.99.12]	gi|148688059|gb|EDL20006.1|	0	pseudouridine synthase 1, isoform CRA_d [Mus musculus]
mmu-miR-1188-5p	NM_001029850	-25.2	0.99872	-49.79	303	GO:0043296,GO:0043231,GO:0032559,	K05631|1|0.0|2546|mmu:14924|atrophin-1 interacting protein 3 (BAI1-associated protein 1)	gi|71533173|ref|NP_001025021.1|	0	membrane-associated guanylate kinase, WW and PDZ domain-containing protein 1 isoform c [Mus musculus]

mmu-miR-1188-5p	NM_001029876	-25.9	0.980833	-46.06	308	GO:0031981,	K14862|1|0.0|2786|mmu:382038|nucleolar pre-ribosomal-associated protein 2	gi|71274162|ref|NP_001025047.1|	0	unhealthy ribosome biogenesis protein 2 homolog [Mus musculus]
mmu-miR-1188-5p	NM_001029937	-30.6	0.340339	-26.46	162	GO:0031224,GO:0005488,	-	gi|71480138|ref|NP_001025108.1|	0	SEC14-like protein 3 [Mus musculus]
mmu-miR-1188-5p	NM_001030307	-28.5	0.495197	-28.33	159	GO:0016604,GO:0030529,GO:0003676,GO:0043021,GO:0003964,GO:0016866,GO:0016836,GO:0009451,GO:0006278,GO:00063	K11131|1|0.0|889|mmu:245474|H/ACA ribonucleoprotein complex subunit 4 [EC:5.4.99.-]	gi|9931991|emb|CAC04528.1|	0	DYSKERIN [Mus musculus]
mmu-miR-1188-5p	NM_001033139	-33.7	0.087299	-30.73	158	-	-	gi|148701354|gb|EDL33301.1|	0	mCG14919, isoform CRA_a [Mus musculus]

mmu-miR-1188-5p	NM_001033201	-27.7	0.662019	-27.67	153	GO:0031981,GO:0034518,GO:0044444,GO:0003723,GO:0006413,GO:0016458,GO:0000956,GO:0006353,GO:0031124,GO:0000398,GO:0006354,GO:0045935,GO:00064	K12882|1|0.0|1523|mmu:433702|nuclear cap-binding protein subunit 1	gi|144922627|ref|NP_001028373.2|	0	nuclear cap-binding protein subunit 1 [Mus musculus]
mmu-miR-1188-5p	NM_001033298	-30.8	0.173066	-28.13	167	GO:0005815,GO:0019900,GO:0000226,	-	gi|85701800|ref|NP_001028470.1|	0	centrosomal protein kizuna [Mus musculus]
mmu-miR-1188-5p	NM_001033318	-35	0.045809	-32.8	170	GO:0043231,GO:0043234,GO:0012505,GO:0005488,GO:0033365,GO:0031647,GO:0048193,GO:0015031,GO:0006464,	-	gi|148685307|gb|EDL17254.1|	0	component of oligomeric golgi complex 7 [Mus musculus]

mmu-miR-1188-5p	NM_001033347	-27.4	0.972449	-50.48	302	GO:0031224,	K15259|1|2e-80|303|cfa:482778|poly [ADP-ribose] polymerase 7/11/12/13 [EC:2.4.2.30]	gi|124358961|ref|NP_001028519.2|	0	uncharacterized protein LOC241589 [Mus musculus]
mmu-miR-1188-5p	NM_001033385	-28.7	0.805764	-28.37	154	-	-	gi|148806863|ref|NP_001028557.2|	0	protein broad-minded [Mus musculus]
mmu-miR-1188-5p	NM_001033475	-32.2	0.343524	-25.77	155	-	-	gi|84794627|ref|NP_001028647.1|	7.75E-154	protein TMED8 [Mus musculus]
mmu-miR-1188-5p	NM_001033488	-33.9	0.078884	-67.48	456	GO:0031224,GO:0051234,	K08824|1|2e-15|85.9|bfo:BRAFLDRAFT_90694|cyclin-dependent kinase-like [EC:2.7.11.22]	gi|74217769|dbj|BAE33602.1|	0	unnamed protein product [Mus musculus]
mmu-miR-1188-5p	NM_001033537	-30	0.26555	-25.9	155	GO:0005789,GO:0031224,GO:0016787,GO:0046488,GO:0006497,	-	gi|114326508|ref|NP_001028709.2|	1.26E-163	post-GPI attachment to proteins factor 3 precursor [Mus musculus]

mmu-miR-1188-5p	NM_001033539	-29.8	0.101198	-25.02	150	GO:0044424,	-	gi|84370324|ref|NP_001028711.1|	7.12E-54	protein BEX6 [Mus musculus]
mmu-miR-1188-5p	NM_001033540	-30.3	0.289811	-26.21	166	-	K09228|1|2e-104|227|ptr:455976|KRAB domain-containing zinc finger protein	gi|291412046|ref|XP_002722297.1|	8.44E-106	PREDICTED: zinc finger protein 582-like [Oryctolagus cuniculus]
mmu-miR-1188-5p	NM_001033782	-28.3	0.554521	-26.08	157	-	-	gi|85702095|ref|NP_001028954.1|	0	uncharacterized protein C2orf78 homolog [Mus musculus]
mmu-miR-1188-5p	NM_001034893	-25.3	0.94376	-62.18	439	-	K09228|1|0.0|1001|mmu:668620|KRAB domain-containing zinc finger protein	gi|85702250|ref|NP_001030065.1|	0	zinc finger protein 936 [Mus musculus]
mmu-miR-1188-5p	NM_001038699	-37.3	0.026683	-32.64	172	GO:0044444,GO:0016301,GO:0006520,GO:0006040,GO:0006796,	K15522|1|3e-116|421|mmu:63828|protein-fructosamine 3-kinase [EC:2.7.1.1	gi|84662727|ref|NP_001033788.1|	2.16E-115	fructosamine-3-kinase isoform b [Mus musculus]

mmu-miR-1188-5p	NM_001039092	-29	0.63411	-25.23	156	-	K12404|1|5e-21|105|pon:100172741|ADP-ribosylation factor-binding protein GGA!K04705|3|7e-20|101|nvi:100119432|signal transducing adaptor	gi|84875524|ref|NP_001034181.1|	0	TOM1-like protein 2 isoform b [Mus musculus]
mmu-miR-1188-5p	NM_001039143	-36.6	0.030408	-56.86	320	GO:0032991,GO:0031981,GO:0044444,GO:0032559,GO:0010608,GO:0043009,GO:0006461,GO:0019953,GO:0009653,	K04614|1|2e-121|438|mmu:627280|vomeronasal1 receptor	gi|148692429|gb|EDL24376.1|	0	mCG113308, isoform CRA_c [Mus musculus]

mmu-miR-1188-5p	NM_001039156	-28.3	0.855017	-66.21	445	GO:0043231,GO:0043232,GO:0019899,	K04989|1|4e-15|86.3|rno:100364048|polycystin 1L3!K13172|3|6e-13|79.0|aml:100474834|serine/arginine repetitive matrix protein 2	gi|88501749|ref|NP_001034245.1|	0	TRIO and F-actin-binding protein isoform 3 [Mus musculus]
mmu-miR-1188-5p	NM_001039167	-32.9	0.176313	-27.89	157	GO:0043231,GO:0044297,GO:0003676,GO:0006396,	K14946|1|6e-169|596|rno:287847|RNA binding protein fox-1	gi|85677497|ref|NP_001034256.1|	1.25E-170	RNA binding protein fox-1 homolog 3 isoform 1 [Mus musculus]
mmu-miR-1188-5p	NM_001039168	-32.9	0.170474	-27.89	157	GO:0043231,GO:0044297,GO:0003676,GO:0006396,	K14946|1|4e-150|533|ecb:100050497|RNA binding protein fox-1	gi|148702730|gb|EDL34677.1|	8.41E-156	DNA segment, Chr 11, Brigham & Women's Genetics 0517 expressed, isoform CRA_d [Mus musculus]
mmu-miR-1188-5p	NM_001039244	-30.9	0.138126	-31.07	159	GO:0031224,	-	gi|148686393|gb|EDL18340.1|	6.84E-164	mCG114452 [Mus musculus]

mmu-miR-1188-5p	NM_001039507	-29.5	0.360313	-28.61	154	GO:0043231,GO:0044459,GO:0044444,GO:0044421,GO:0019843,GO:0004091,GO:0006641,GO:0006352,GO:0016125,GO:0042221,GO:0046339,GO:0022414,GO:00064	K07188|1|0.0|1409|mmu:16890|hormone-sensitive lipase [EC:3.1.1.79]	gi|87239970|ref|NP_034849.2|	0	hormone-sensitive lipase isoform 1 [Mus musculus]
mmu-miR-1188-5p	NM_001039509	-28.1	0.574734	-25.69	150	GO:0043231,GO:0046914,GO:0016790,	K01069|1|2e-47|191|dan:Dana_GF10361|hydroxyacylglutathione hydrolase [EC:3.1.2.6]	gi|50510835|dbj|BAD32403.1|	0	mKIAA1184 protein [Mus musculus]

mmu-miR-1188-5p	NM_001039521	-31	0.274965	-26.12	152	GO:0031981,GO:0019899,GO:0006915,GO:0006355,GO:0022613,GO:0043009,GO:0006352,GO:0042592,GO:0006997,	K15216|1|0.0|1188|mmu:106298|RNA polymerase I-specific transcription initiation factor RRN3	gi|87299630|ref|NP_001034610.1|	0	RNA polymerase I-specific transcription initiation factor RRN3 [Mus musculus]
mmu-miR-1188-5p	NM_001039677	-30.7	0.281301	-26.59	153	GO:0031224,GO:0005764,GO:0005768,GO:0031410,GO:0031090,GO:0046915,GO:0006829,GO:0048518,	K14689|1|0.0|705|mmu:230810|solute carrier family 30 (zinc transporter), member 2	gi|89179312|ref|NP_001034766.1|	0	zinc transporter 2 [Mus musculus]
mmu-miR-1188-5p	NM_001040398	-28.9	0.838057	-25.03	159	-	-	-	-	-

mmu-miR-1188-5p	NM_001040426	-32.8	0.338176	-29.44	167	GO:0031012,GO:0004175,	K08630|1|7e-87|324|acs:100566040|a disintegrin and metalloproteinase with thrombospondin motifs 16 [EC:3.4.24.-]	gi|283135196|ref|NP_001035516.2|	0	thrombospondin type-1 domain-containing protein 4 isoform a precursor [Mus musculus]
mmu-miR-1188-5p	NM_001042487	-27.2	0.783332	-43.91	308	GO:0044297,GO:0043005,GO:0016020,GO:0005515,	K15008|1|1e-75|285|mdo:100012355|discs, large-associated protein 1	gi|109891940|ref|NP_001035952.1|	0	disks large-associated protein 4 isoform b [Mus musculus]
mmu-miR-1188-5p	NM_001042488	-29	0.438969	-43.91	308	GO:0044297,GO:0043005,GO:0016020,GO:0005515,	K15008|1|9e-76|286|mdo:100012355|discs, large-associated protein 1	gi|74151726|dbj|BAE29656.1|	0	unnamed protein product [Mus musculus]

mmu-miR-1188-5p	NM_001044740	-25.3	0.998264	-25.69	158	-	K13864|1|0.0|1207|mmu:11988|solute carrier family 7 (cationic amino acid transporter), member 2	gi|113680233|ref|NP_001038205.1|	0	low affinity cationic amino acid transporter 2 isoform 2 [Mus musculus]
mmu-miR-1188-5p	NM_001044747	-27.7	0.795226	-45.51	309	GO:0005488,	K09228|1|0.0|1212|mmu:24135|KRAB domain-containing zinc finger protein	gi|7305647|ref|NP_038872.1|	0	zinc finger protein 68 isoform a [Mus musculus]
mmu-miR-1188-5p	NM_001046637	-25	0.741032	-27.25	152	GO:0030529,GO:0031981,GO:0005488,GO:0000377,	K11091|1|3e-98|359|mmu:53607|U1 small nuclear ribonucleoprotein A	gi|12851426|dbj|BAB29037.1|	2.09E-97	unnamed protein product [Mus musculus]
mmu-miR-1188-5p	NM_001048250	-28.7	0.326848	-26.06	150	GO:0031224,	-	gi|148686509|gb|EDL18456.1|	1.12E-36	mCG51409, isoform CRA_a [Mus musculus]

mmu-miR-1188-5p	NM_001077707	-28.3	0.840898	-26.35	159	GO:0043231,GO:0000785,GO:0017111,GO:0003676,GO:0046914,GO:0032559,GO:0006259,GO:0033554,GO:0034728,	K15710|1|0.0|3300|mmu:268281|E3 ubiquitin-protein ligase SHPRH [EC:3.6.4.-6.3.2.19]	gi|118150662|ref|NP_001071175.1|	0	E3 ubiquitin-protein ligase SHPRH isoform a [Mus musculus]
mmu-miR-1188-5p	NM_001080387	-32.4	0.12164	-50.21	313	GO:0031981,GO:0003676,GO:0005515,GO:0000245,GO:0033119,GO:0046907,	K12900|1|2e-67|258|mdo:100010446|FUS-interacting serine-arginine-rich protein 1	gi|74141008|dbj|BAE22085.1|	3.97E-75	unnamed protein product [Mus musculus]
mmu-miR-1188-5p	NM_001080820	-29.2	0.386815	-27.79	161	GO:0044459,GO:0043232,	K05726|1|6e-57|223|spu:576962|breast cancer anti-estrogen resistance 1	gi|124249364|ref|NP_001074289.1|	0	cas scaffolding protein family member 4 isoform 2 [Mus musculus]
mmu-miR-1188-5p	NM_001081040	-27.2	0.649161	-28.68	165	GO:0016020,GO:0043231,	-	gi|124486652|ref|NP_001074509.1|	8.62E-107	coenzyme Q-binding protein COQ10 homolog A, mitochondrial [Mus musculus]

mmu-miR-1188-5p	NM_001081047	-31.5	0.153841	-30.21	154	GO:0030054,GO:0044444,GO:0005515,GO:0016772,GO:0007167,GO:0006796,GO:00072	-	gi|124486674|ref|NP_001074516.1|	0	connector enhancer of kinase suppressor of ras 1 [Mus musculus]
mmu-miR-1188-5p	NM_001081058	-26	0.98921	-42.29	294	GO:0016604,GO:0000307,GO:0004674,GO:0000166,GO:0006357,GO:0048534,GO:0007067,GO:0000398,GO:0008283,	K08819|1|0.0|1840|mmu:69562|cyclin-dependent kinase 12/13 [EC:2.7.11.22 2.7.11.23]	gi|124486698|ref|NP_001074527.1|	0	cyclin-dependent kinase 13 isoform 1 [Mus musculus]
mmu-miR-1188-5p	NM_001081064	-25.8	0.999702	-57.51	338	GO:0030054,GO:0043231,GO:0044430,GO:0007165,	K06095|1|5e-31|139|gga:427360|multiple PDZ domain protein	gi|124486716|ref|NP_001074533.1|	0	PDZ domain-containing protein 2 [Mus musculus]

mmu-miR-1188-5p	NM_001081078	-28.4	0.793744	-26.23	162	GO:0044459,GO:0016021,GO:0042995,GO:0004553,GO:0043167,GO:0048545,GO:0031667,GO:0006950,GO:0010039,GO:0007586,GO:0044238,GO:0034285,	K01229|1|0.0|3922|mmu:226413|lactase-phlorizin hydrolase [EC:3.2.1.108 3.2.1.62]	gi|124487297|ref|NP_001074547.1|	0	lactase-phlorizin hydrolase preproprotein [Mus musculus]
mmu-miR-1188-5p	NM_001081133	-26.1	0.968992	-26.1	155	GO:0015630,GO:0005543,GO:0003774,GO:0032559,GO:0007017,	K10392|1|0.0|2334|mmu:16558|kinesin family member 1/13/14	gi|124487287|ref|NP_001074602.1|	0	kinesin-like protein KIF16B [Mus musculus]

mmu-miR-1188-5p	NM_001081155	-28.8	0.508926	-28.56	155	GO:0043231,GO:0044444,GO:0017111,GO:0008047,GO:0042802,GO:0031267,GO:0043087,GO:0009966,	K04851|1|1e-75|286|xtr:780391|voltage-dependent calcium channel L type alpha-1D!K08013|2|4e-54|214|acs:100553026|signal-induced proliferation-associated gene 1	gi|124487211|ref|NP_001074624.1|	0	rap1 GTPase-activating protein 1 isoform 1 [Mus musculus]
mmu-miR-1188-5p	NM_001081178	-28.9	0.827714	-67.94	454	GO:0031224,GO:0007186,	K08458|1|0.0|2580|mmu:224792|G protein-coupled receptor 116	gi|124487171|ref|NP_001074647.1|	0	probable G-protein coupled receptor 116 precursor [Mus musculus]
mmu-miR-1188-5p	NM_001081193	-31.2	0.331223	-26.66	163	GO:0031224,GO:0005637,GO:0005488,GO:0030509,GO:0007179,GO:0032924,	K13091|1|9e-23|110|tad:TRIADDRAFT_37071|RNA-binding protein	gi|145864461|ref|NP_001074662.2|	0	inner nuclear membrane protein Man1 [Mus musculus]

mmu-miR-1188-5p	NM_001081225	-25.8	0.991567	-27.12	148	-	K06521|1|4e-12|75.9|acs:100564148|semaphorin 4	gi|148709994|gb|EDL41940.1|	0	mCG127475, isoform CRA_b [Mus musculus]	
mmu-miR-1188-5p	NM_001081230	-27.4	0.904592	-29.84	160	GO:0044424,	K10434|1|0.0|949|mmu:213582|microtubule-associated protein 9	gi|124486997|ref|NP_001074699.1|	0	microtubule-associated protein 9 [Mus musculus]	
mmu-miR-1188-5p	NM_001081260	-26.9	0.931077	-53.22	330	GO:0044444,GO:0000784,GO:0008092,GO:0006278,GO:0000288,	-	gi|124486923|ref|NP_001074729.1|	0	182 kDa tankyrase-1-binding protein [Mus musculus]	

mmu-miR-1188-5p	NM_001081280	-33.6	0.120993	-57.23	313	GO:0044424,GO:0032559,GO:0007243,GO:0043433,GO:0046649,	K12800|1|2e-82|308|dre:561595|NACHT, LRR and PYD domains-containing protein 3!K10165|5|5e-49|197|mmu:257632|nucleotide-binding oligomerization domain-containing protein 2	gi|124486873|ref|NP_001074749.1|	0	protein NLRC3 [Mus musculus]
mmu-miR-1188-5p	NM_001081301	-29.4	0.115905	-49.49	316	GO:0031224,GO:0004871,GO:0007166,	K04257|1|3e-134|478|mmu:546896|olfactory	gi|240255445|ref|NP_001074770.2|	1.95E-133	olfactory receptor 455, pseudogene 1 [Mus musculus]
mmu-miR-1188-5p	NM_001081343	-25.2	0.95393	-40.94	299	-	-	gi|124486791|ref|NP_001074812.1|	0	uncharacterized protein C9orf72 homolog [Mus musculus]

mmu-miR-1188-5p	NM_001081347	-29.6	0.499072	-27.1	163	GO:0044444,GO:0016020,GO:0032561,GO:0035556,GO:0009966,	K07868|1|0.0|1371|mmu:69288|Rho-related BTB domain-containing protein 1/2	gi|124486809|ref|NP_001074816.1|	0	rho-related BTB domain-containing protein 1 isoform 1 [Mus musculus]
mmu-miR-1188-5p	NM_001081374	-33.8	0.080046	-28.27	155	GO:0044424,GO:0031012,GO:0004175,GO:0019538,	K09630|1|0.0|1756|mmu:77613|protease, serine, 36 [EC:3.4.21.-]	gi|239052282|ref|NP_001074843.2|	0	polyserase-2 precursor [Mus musculus]
mmu-miR-1188-5p	NM_001083319	-27.2	0.802676	-25.33	155	GO:0043231,GO:0003712,GO:0003676,GO:0001071,GO:0048646,GO:0006355,GO:00190	K09275|1|0.0|1047|mmu:22221|transcription factor CP2 and related proteins	gi|134032032|ref|NP_001076788.1|	0	upstream-binding protein 1 isoform a [Mus musculus]
mmu-miR-1188-5p	NM_001085513	-29.4	0.190154	-25.84	160	-	-	gi|148222952|ref|NP_001078982.1|	2.34E-153	uncharacterized protein LOC277743 [Mus musculus]

mmu-miR-1188-5p	NM_001099323	-27.6	0.629351	-26.6	156	GO:0043231,GO:0043169,GO:0010467,	K09229|1|0.0|1522|mmu:100041581|KRAB and SCAN domains-containing zinc finger protein	gi|150010617|ref|NP_001092793.1|	0	zinc finger protein 483 [Mus musculus]
mmu-miR-1188-5p	NM_001099328	-27.9	0.944917	-45.2	313	-	K13181|1|3e-114|415|mgp:100549747|ATP-dependent RNA helicase DDX27 [EC:3.6.4.13]!K09239|2|1e-30|137|xtr:100489803|human immunodeficiency virus type I enhancer-binding protein	gi|150010581|ref|NP_001092798.1|	0	zinc finger protein 831 [Mus musculus]

mmu-miR-1188-5p	NM_001099624	-25.1	0.99754	-42.31	294	GO:0016021,GO:0005088,GO:0060089,GO:0046872,GO:0008289,GO:0005099,GO:0019935,GO:0007243,GO:0009966,	K08018|1|0.0|2651|mmu:76089|Rap guanine nucleotide exchange factor (GEF) 2	gi|44890797|gb|AAH67056.1|	0	Rapgef2 protein [Mus musculus]
mmu-miR-1188-5p	NM_001099636	-34.6	0.084076	-31.38	169	-	-	gi|153791731|ref|NP_001093106.1|	0	PNMA-like protein 2 [Mus musculus]
mmu-miR-1188-5p	NM_001100460	-33.7	0.096964	-28.56	167	GO:0043231,GO:0003676,GO:0046914,GO:0000122,	K10490|1|3e-50|201|mdo:100012474|zinc finger and BTB domain-containing protein 3	gi|257743328|ref|NP_001093930.1|	0	zinc finger and BTB domain-containing protein 42 [Mus musculus]
mmu-miR-1188-5p	NM_001101433	-27.3	0.82813	-25.6	157	GO:0046914,	-	gi|160333288|ref|NP_001094903.1|	4.62E-130	zinc finger CCHC domain-containing protein 24 [Mus musculus]

mmu-miR-1188-5p	NM_001103171	-25.3	0.999851	-29.42	163	GO:0005902,GO:0015629,GO:0008092,GO:0017111,GO:0032559,GO:0042471,GO:0050954,GO:0007610,	K10361|1|0.0|6171|mmu:17910|myosin XV	gi|157041248|ref|NP_001096641.1|	0	unconventional myosin-XV isoform 3 [Mus musculus]
mmu-miR-1188-5p	NM_001103182	-29.7	0.359871	-26.52	154	GO:0031981,GO:0006259,	-	gi|332252019|ref|XP_003275151.1|	0	PREDICTED: protein lin-9 homolog isoform 1 [Nomascus leucogenys]
mmu-miR-1188-5p	NM_001104634	-32.4	0.112829	-26.03	153	GO:0031224,GO:0004888,GO:0007166,GO:0010033,	K04613|1|0.0|1615|mmu:667180|vomeronasal 2 receptor	gi|157277994|ref|NP_001098104.1|	0	vomeronasal receptor Vmn2r20 precursor [Mus musculus]
mmu-miR-1188-5p	NM_001104635	-32.4	0.112829	-26.03	153	GO:0031224,GO:0004888,GO:0007166,GO:0010033,	K04613|1|0.0|1604|mmu:667180|vomeronasal 2 receptor	gi|157277996|ref|NP_001098105.1|	0	vomeronasal receptor Vmn2r21 precursor [Mus musculus]
mmu-miR-1188-5p	NM_001104637	-30.8	0.209423	-26.37	154	GO:0031224,GO:0004888,GO:0007166,GO:0010033,	K04613|1|0.0|1619|mmu:546913|vomeronasal 2 receptor	gi|157277998|ref|NP_001098107.1|	0	vomeronasal receptor Vmn2r22 precursor [Mus musculus]

mmu-miR-1188-5p	NM_001109873	-31.4	0.458206	-49.91	312	GO:0031981,GO:0012505,GO:0046914,GO:0001071,GO:0002573,GO:0006351,	K10053|1|0.0|798|ptr:472816|runt-related transcription factor 1; translocated to, 1	gi|160013478|sp|O54972.2|MTG16_MOUSE	0	RecName: Full=Protein CBFA2T3; AltName: Full=Eight twenty one protein 2; AltName: Full=MTG8-related protein 2; AltName: Full=Protein ETO-2
mmu-miR-1188-5p	NM_001110208	-26.5	0.834594	-47.35	308	GO:0031981,GO:0031252,GO:0030141,GO:0016020,GO:0004672,GO:0032559,GO:0019901,GO:0010466,GO:0032868,GO:0006915,GO:0006357,GO:0002028,GO:0010828,GO:0007169,GO:0008380,GO:0005979,GO:0031328,GO:0016477,GO:0001934,GO:0043408,GO:0010746,GO:00509	K04456|1|0.0|986|mmu:11652|RAC serine/threonine-protein kinase [EC:2.7.11.1]	gi|6680674|ref|NP_031460.1|	0	RAC-beta serine/threonine-protein kinase [Mus musculus]

mmu-miR-1188-5p	NM_001110275	-30.9	0.404013	-29.78	167	GO:0044459,GO:0031252,GO:0016023,GO:0012505,GO:0008047,GO:0005088,GO:0046872,GO:0005515,GO:0016192,GO:0006917,GO:0019222,GO:0010740,GO:0043066,GO:00	K07976|1|6e-30|134|ame:409364|Rab family, other!K13738|3|1e-29|134|bfo:BRAFLDRAFT_90471|CD2-associated protein	gi|148671857|gb|EDL03804.1|	0	intersectin 1 (SH3 domain protein 1A), isoform CRA_c [Mus musculus]

mmu-miR-1188-5p	NM_001110796	-28.6	0.970286	-46.35	304	GO:0016020,GO:0046872,GO:0005543,GO:0005515,GO:0006996,GO:0006903,GO:0006887,GO:0030072,GO:0019935,	K15297|1|1e-30|138|nvi:100121009|regulating synaptic membrane exocytosis protein 2!K15291|2|8e-29|132|acs:100568133|regulating synaptic membrane exocytosis protein 1	gi|160707978|ref|NP_001104266.1|	0	protein piccolo isoform 2 [Mus musculus]
mmu-miR-1188-5p	NM_001111324	-29.5	0.5323	-25.13	157	GO:0043231,GO:0031252,GO:0005924,GO:0044444,GO:0005819,GO:0005488,GO:0007015,GO:0000087,GO:0007166,	K05726|1|1e-159|565|xtr:447979|breast cancer anti-estrogen resistance 1	gi|148709029|gb|EDL40975.1|	0	neural precursor cell expressed, developmentally down-regulated gene 9, isoform CRA_c [Mus musculus]

mmu-miR-1188-5p	NM_001112699	-29.2	0.443024	-26.34	155	GO:0016020,GO:0044424,GO:0005083,GO:0005488,GO:0019222,GO:0007155,GO:0006810,GO:0032011,	K12495|1|2e-39|165|dpo:Dpse_GA23806|IQ motif and SEC7 domain-containing protein	gi|60360016|dbj|BAD90227.1|	0	mKIAA4240 protein [Mus musculus]
mmu-miR-1188-5p	NM_001112700	-29.2	0.436625	-26.34	155	GO:0016020,GO:0044424,GO:0005083,GO:0005488,GO:0019222,GO:0007155,GO:0006810,GO:0032011,	K12495|1|2e-39|165|bfo:BRAFLDRAFT_106693|IQ motif and SEC7 domain-containing protein	gi|162951837|ref|NP_001106170.1|	0	cytohesin-1 isoform 3 [Mus musculus]
mmu-miR-1188-5p	NM_001113412	-29.4	0.258901	-26.01	150	GO:0016772,GO:0044238,GO:0006796,GO:0019725,	K00864|1|2e-08|62.0|tgu:100232763|glycerol kinase [EC:2.7.1.30]	gi|167555144|ref|NP_001106883.1|	0	FGGY carbohydrate kinase domain-containing protein isoform a [Mus musculus]
mmu-miR-1188-5p	NM_001113471	-31.4	0.116468	-27.83	158	GO:0044444,GO:0005488,GO:0006641,	K08768|1|0.0|932|mmu:103968|perilipin	gi|148675107|gb|EDL07054.1|	0	perilipin, isoform CRA_e [Mus musculus]

mmu-miR-1188-5p	NM_001122639	-32.3	0.063132	-27.91	167	GO:0012505,GO:0043231,GO:0031224,GO:0030246,GO:0008376,	K00710|1|6e-141|501|mdo:100028262|polypeptide N-acetylgalactosaminyltransferase [EC:2.4.1.41]	gi|169790935|ref|NP_938048.2|	3.42E-145	polypeptide N-acetylgalactosaminyltransferase 9 isoform A [Mus musculus]
mmu-miR-1188-5p	NM_001122949	-31.8	0.166467	-27.78	162	GO:0016021,GO:0004888,GO:0007599,GO:0001775,GO:0007166,	K05082|1|0.0|1288|mmu:17480|thrombopoietin receptor	gi|171846242|ref|NP_001116421.1|	0	thrombopoietin receptor isoform 1 precursor [Mus musculus]

mmu-miR-1188-5p	NM_001081364	-22.5	1	-78.71	456	GO:0043231,GO:0044459,GO:0043232,GO:0012506,GO:0008047,GO:0005488,GO:0043087,GO:0023052,	K04450|1|2e-29|133|ptr:739083|activating transcription factor 2!K08878|2|7e-29|131|cin:100176232|breakpoint cluster region protein [EC:2.7.11.1]!K07526|3|4e-27|125|nve:NEMVE_v1g88221|SLIT-ROBO Rho GTPase activating	gi|203097667|ref|NP_001074833.3|	0	rho GTPase-activating protein 21 isoform 2 [Mus musculus]

mmu-miR-1190	NM_001001326	-29.4	0.546385	-27.5	172	GO:0005488,GO:0043410,	K12861|1|9e-165|582|gga:419884|pre-mRNA-splicing factor SPF27!K01289|2|8e-28|127|dme:Dmel_CG18659|carboxypeptidase [EC:3.4.16.	gi|47679093|ref|NP_001001326.1|	0	suppression of tumorigenicity 5 protein isoform 1 [Mus musculus]
mmu-miR-1190	NM_001001495	-33.2	0.139333	-33.87	166	-	-	gi|110665744|ref|NP_001001495.2|	1.78E-98	RIKEN cDNA 9030611K07 [Mus musculus]
mmu-miR-1190	NM_001001984	-25.5	0.997342	-51.32	317	GO:0031981,GO:0003676,GO:0046914,GO:0016706,GO:0016701,GO:0070076,GO:0006351,	K10276|1|0.0|2158|mmu:225876|F-box and leucine-rich repeat protein 10/11 [EC:1.14.11.27]	gi|100818178|ref|NP_001001984.2|	0	lysine-specific demethylase 2A [Mus musculus]

mmu-miR-1190	NM_001004062	-36.2	0.071634	-33.71	167	GO:0031981,GO:0016020,GO:0005488,GO:0003713,GO:0006357,GO:0051704,	K15309|1|0.0|1067|mmu:382056|CREB-regulated transcription coactivator	gi|37360010|dbj|BAC97983.1|	0	mKIAA0616 protein [Mus musculus]
mmu-miR-1190	NM_001004139	-26.6	0.96281	-75.82	469	-	K09228|1|0.0|2482|mmu:70227|KRAB domain-containing zinc finger protein	gi|160333308|ref|NP_001004139.2|	0	zinc finger protein 208 [Mus musculus]
mmu-miR-1190	NM_001004142	-28.6	0.707057	-25.79	158	GO:0043231,GO:0005515,GO:0032559,GO:0006915,GO:0006952,GO:0012502,	K12798|1|0.0|2246|mmu:195046|NACHT, LRR and PYD domains-containing protein 1	gi|241666421|ref|NP_001004142.2|	0	NLR family, pyrin domain containing 1A [Mus musculus]

mmu-miR-1190	NM_001004156	-31.9	0.244268	-28.87	179	GO:0044444,GO:0016020,GO:0005488,GO:0005088,GO:0060089,GO:0016477,GO:0006917,GO:0019222,GO:0007249,GO:0007266,	K12331|1|2e-25|119|bta:511220|Rho guanine nucleotide exchange factor 11!K12330|2|2e-25|119|aml:100477081|Rho guanine nucleotide exchange factor 1	gi|81910721|sp|Q66T02.1|PKHG5_MOUSE	0	RecName: Full=Pleckstrin homology domain-containing family G member 5; Short=PH domain-containing family G member 5; AltName: Full=Synectin-binding RhoA exchange factor; Short=SYX
mmu-miR-1190	NM_001005475	-29.9	0.550463	-25.08	160	GO:0044424,GO:0005083,GO:0032011,	K12495|1|0.0|1544|mmu:245666|IQ motif and SEC7 domain-containing protein	gi|350595687|ref|XP_003135163.3|	0	PREDICTED: IQ motif and SEC7 domain-containing protein 2 [Sus scrofa]
mmu-miR-1190	NM_001007460	-30.9	0.096701	-26.77	157	GO:0031224,GO:0046914,GO:0016747,	K05766|1|9e-12|72.0|spu:575378|slingshot	gi|74184789|dbj|BAE27992.1|	0	unnamed protein product [Mus musculus]

mmu-miR-1190	NM_001008232	-35.4	0.094813	-33.18	166	GO:0044424,GO:0005083,GO:0046914,GO:0043087,GO:0032012,	K12488|1|0.0|1744|mmu:230837|Arf-GAP with SH3 domain, ANK repeat and PH domain-containing protein	gi|56711264|ref|NP_001008233.1|	0	arf-GAP with SH3 domain, ANK repeat and PH domain-containing protein 3 [Mus musculus]
mmu-miR-1190	NM_001008498	-31.4	0.121167	-27.42	160	GO:0044424,	K15437|1|9e-41|169|hmg:100206473|aminoacyl tRNA synthase complex-interacting multifunctional protein 1!K10415|2|2e-24|115|dre:767729|dynein intermediate chain, cytosolic	gi|139948827|ref|NP_001008498.2|	0	WD repeat-containing protein 34 [Mus musculus]

mmu-miR-1190	NM_001009547	-29.7	0.313426	-26.05	166	GO:0031224,GO:0004175,GO:0046914,GO:0005102,GO:0019538,GO:0048232,GO:0048869,	K08613|1|0.0|1281|mmu:13525|disintegrin and metalloproteinase domain-containing protein 26 [EC:3.4.24.-]!K08612|2|0.0|723|ssc:100153750|disintegrin and metalloproteinase domain-containing protein 25 [EC:3.4.24.-]!K08609|3|0.0|716|	gi|57222280|ref|NP_001009547.1|	0	a disintegrin and metalloprotease domain 26b precursor [Mus musculus]
mmu-miR-1190	NM_001011741	-28.1	0.262417	-27.1	161	GO:0016020,GO:0004888,GO:0007166,	K04257|1|3e-177|621|mmu:257888|olfactory	gi|58801276|ref|NP_001011741.1|	1.84E-176	olfactory receptor 1386 [Mus musculus]
mmu-miR-1190	NM_001013256	-31.4	0.294383	-32.59	159	GO:0000783,GO:0043566,GO:0006259,	-	gi|219689064|ref|NP_001013274.2|	0	CST complex subunit CTC1 isoform a [Mus musculus]

mmu-miR-1190	NM_001013367	-35.1	0.086303	-30.47	171	GO:0044444,GO:0004690,GO:0032559,GO:0043169,GO:0010893,GO:0046317,GO:0007169,GO:0022402,GO:0019318,GO:0006464,GO:0006950,GO:0019217,GO:00001	K07198|1|0.0|1031|mmu:105787|5'-AMP-activated protein kinase, catalytic alpha subunit [EC:2.7.11.11]	gi|94681061|ref|NP_001013385.3|	0	5'-AMP-activated protein kinase catalytic subunit alpha-1 [Mus musculus]
mmu-miR-1190	NM_001013814	-38	0.004513	-34.63	180	GO:0043231,GO:0016769,GO:0008168,GO:0006730,GO:0006544,	K00605|1|0.0|804|mmu:434437|aminomethyltransferase [EC:2.1.2.10]	gi|62000670|ref|NP_001013836.1|	0	aminomethyltransferase, mitochondrial precursor [Mus musculus]
mmu-miR-1190	NM_001017419	-33.8	0.032674	-30.54	162	-	-	gi|31414725|gb|AAP50907.1|	1.53E-168	spatial-delta [Mus musculus]
mmu-miR-1190	NM_001017433	-33.8	0.035967	-30.54	162	GO:0044444,GO:0005488,GO:0003006,GO:0048232,GO:0048869,	-	gi|31414727|gb|AAP50908.1|	0	spatial-epsilon [Mus musculus]

mmu-miR-1190	NM_001024148	-25.5	0.906778	-26.98	156	GO:0031224,GO:0008194,GO:0007606,GO:0042221,	K00699|1|0.0|1059|mmu:552899|glucuronosyltransferase [EC:2.4.1.17]	gi|66571303|ref|NP_001019319.1|	0	UDP-glucuronosyltransferase 2A2 precursor [Mus musculus]
mmu-miR-1190	NM_001024468	-30.7	0.58384	-50.43	309	GO:0043231,GO:0044444,GO:0005515,GO:0004084,GO:0008652,GO:0009063,	K00826|1|0.0|931|mmu:12035|branched-chain amino acid aminotransferase [EC:2.6.1.42]	gi|66792792|ref|NP_001019639.1|	0	branched-chain-amino-acid aminotransferase, cytosolic isoform 1 [Mus musculus]
mmu-miR-1190	NM_001024539	-29.9	0.452511	-45.66	308	GO:0044444,GO:0007169,GO:0007264,	K06279|1|0.0|1078|mmu:216148|src homology 2 domain-containing transforming protein C	gi|169790806|ref|NP_001019710.1|	0	SHC-transforming protein 2 [Mus musculus]
mmu-miR-1190	NM_001024720	-25.4	0.999996	-46.47	308	GO:0044459,GO:0044444,GO:0005578,GO:0046872,GO:0006464,GO:0050953,	K12567|1|0.0|1253|rno:84015|titin [EC:2.7.11.1]	gi|154689979|ref|NP_001019891.2|	0	hemicentin 1 precursor [Mus musculus]

mmu-miR-1190	NM_001025245	-31.4	0.33419	-26.94	165	GO:0043231,GO:0044297,GO:0043209,GO:0033267,GO:0016020,GO:0019899,GO:0005198,GO:0008366,GO:0007267,GO:0031345,GO:0002376,	-	gi|69885018|ref|NP_001020416.1|	4.24E-108	Golli-Mbp isoform 2 [Mus musculus]
mmu-miR-1190	NM_001025381	-33.2	0.19573	-30.44	178	GO:0016021,GO:0001637,GO:0001608,GO:0035588,	K08404|1|0.0|650|mmu:574402|G protein-coupled receptor 17	gi|71725394|ref|NP_001020552.1|	0	uracil nucleotide/cysteinyl leukotriene receptor [Mus musculus]
mmu-miR-1190	NM_001025428	-27	0.959611	-25.36	157	GO:0044444,GO:0051019,GO:0008092,GO:0032091,GO:0045597,GO:0051259,GO:0016197,GO:0010740,GO:0042692,GO:00001	K04436|1|0.0|1051|mdo:100010499|mitogen-activated protein kinase 8 interacting protein 3	gi|50510507|dbj|BAD32239.1|	0	mKIAA0516 protein [Mus musculus]

mmu-miR-1190	NM_001025429	-27	0.959563	-25.36	157	GO:0044444,GO:0051019,GO:0008092,GO:0032091,GO:0045597,GO:0051259,GO:0016197,GO:0010740,GO:0042692,GO:00001	K04436|1|0.0|1059|rno:302983|mitogen-activated protein kinase 8 interacting protein 3	gi|50510507|dbj|BAD32239.1|	0	mKIAA0516 protein [Mus musculus]
mmu-miR-1190	NM_001025430	-27	0.959367	-25.36	157	GO:0044444,GO:0051019,GO:0008092,GO:0032091,GO:0045597,GO:0051259,GO:0016197,GO:0010740,GO:0042692,GO:00001	K04436|1|0.0|1046|rno:302983|mitogen-activated protein kinase 8 interacting protein 3	gi|50510507|dbj|BAD32239.1|	0	mKIAA0516 protein [Mus musculus]
mmu-miR-1190	NM_001030014	-27.2	0.564963	-48.47	323	GO:0043231,GO:0004312,GO:0006631,	K00645|1|0.0|725|mmu:223722|[acyl-carrier-protein] S-malonyltransferase [EC:2.3.1.39]	gi|71725343|ref|NP_001025185.1|	0	malonyl-CoA-acyl carrier protein transacylase, mitochondrial [Mus musculus]

mmu-miR-1190	NM_001033042	-29.4	0.390558	-50.79	304	-	-	gi|40675542|gb|AAH64819.1|	0	AI595366 protein, partial [Mus musculus]
mmu-miR-1190	NM_001033194	-30.9	0.255174	-26.61	163	-	K15201|1|0.0|1608|mmu:98488|general transcription factor 3C polypeptide 3 (transcription factor C subunit 4)	gi|148667591|gb|EDL00008.1|	0	mCG113059 [Mus musculus]
mmu-miR-1190	NM_001033207	-26	0.991061	-47.7	311	GO:0043231,GO:0044444,GO:0000977,GO:0001961,GO:0001960,GO:0010557,GO:0001818,GO:0043433,GO:0002252,GO:0042325,GO:0006952,	K08060|1|2e-39|166|tgu:100223542|class II, major histocompatibility complex, transactivator	gi|254692966|ref|NP_001028379.2|	0	protein NLRC5 [Mus musculus]
mmu-miR-1190	NM_001033220	-28	0.333751	-25.77	164	-	-	gi|268838809|ref|NP_001028392.2|	0	UPF0764 protein C16orf89 homolog precursor [Mus musculus]

mmu-miR-1190	NM_001033273	-25	0.995036	-44.49	301	GO:0005488,	-	gi|85701776|ref|NP_001028445.1|	0	uncharacterized protein KIAA0930 homolog [Mus musculus]
mmu-miR-1190	NM_001033275	-32.4	0.316347	-30.68	170	GO:0031224,GO:0008194,GO:0006493,	K13676|1|0.0|731|mmu:223827|UDP-xylose:glucoside alpha-1,3-xylosyltransferase [EC:2.4.2.-]	gi|152125897|sp|Q3UHH8.2|GXLT1_MOUSE	0	RecName: Full=Glucoside xylosyltransferase 1; AltName: Full=Glycosyltransferase 8 domain-containing protein 3
mmu-miR-1190	NM_001033276	-25.3	0.999999	-68.05	458	GO:0043231,GO:0034708,GO:0016740,GO:0043565,GO:0008283,GO:0048545,GO:0030520,GO:0007292,GO:0016568,	K09187|1|0.0|4273|mmu:381022|histone-lysine N-methyltransferase MLL2 [EC:2.1.1.43]	gi|359718904|ref|NP_001028448.3|	0	histone-lysine N-methyltransferase MLL2 [Mus musculus]
mmu-miR-1190	NM_001033341	-26.2	0.992315	-55.53	313	GO:0043231,GO:0046914,GO:0003676,	K09228|1|8e-72|274|bfo:BRAFLDRAFT_66688|KRAB domain-containing zinc finger protein	gi|256223449|ref|NP_001028513.2|	0	zinc finger protein 407 [Mus musculus]

mmu-miR-1190	NM_001033420	-28.3	0.853318	-25.58	164	GO:0044444,GO:0008047,GO:0030695,GO:0019899,GO:0032561,GO:0019904,GO:0012501,GO:0035556,GO:0006909,GO:0048870,GO:0006935,GO:0007599,GO:00071	K13708|1|0.0|3465|mmu:330662|dedicator of cytokinesis 1	gi|88853584|ref|NP_001028592.1|	0	dedicator of cytokinesis protein 1 [Mus musculus]
mmu-miR-1190	NM_001033431	-25.7	0.970554	-41.41	303	GO:0044424,GO:0016505,GO:0032559,GO:0032661,GO:0032731,GO:0001933,GO:0007249,GO:0006915,GO:0008592,GO:0031349,GO:0050707,GO:0042036,GO:00326	K12800|1|0.0|815|oaa:100087792|NACHT, LRR and PYD domains-containing protein 3	gi|257468329|ref|NP_001028603.1|	0	NACHT, LRR and PYD domains-containing protein 12 [Mus musculus]

mmu-miR-1190	NM_001033451	-25.3	0.973472	-26.14	160	GO:0005622,GO:0046872,GO:0005515,	K09228|1|1e-70|269|ptr:742564|KRAB domain-containing zinc finger protein	gi|110626129|ref|NP_001028623.1|	0	zinc finger protein 408 [Mus musculus]
mmu-miR-1190	NM_001033473	-32.6	0.044955	-29.48	172	-	K05626|1|4e-06|53.1|ssc:100153709|atrophin-1 (dentatorubral-pallidoluysian atrophy protein)	gi|85701995|ref|NP_001028645.1|	7.09E-164	outer dense fiber protein 3-like protein 2 [Mus musculus]
mmu-miR-1190	NM_001033484	-28.7	0.750003	-52.93	312	GO:0044464,GO:0005083,GO:0005515,GO:0009966,	K05767|1|0.0|2908|mmu:404710|IQ motif containing GTPase activating protein	gi|118344446|ref|NP_001028656.1|	0	IQ motif containing GTPase activating protein 3 [Mus musculus]

mmu-miR-1190	NM_001033813	-28.2	0.586457	-50.86	316	GO:0005622,GO:0046872,GO:0003676,	K09228|1|0.0|1004|mmu:619310|KRAB domain-containing zinc finger protein	gi|85702145|ref|NP_001028985.1|	0	zinc finger protein 564 [Mus musculus]
mmu-miR-1190	NM_001035122	-30	0.401827	-28.47	162	GO:0016021,GO:0043231,	K10352|1|2e-09|66.2|isc:IscW_ISCW001340|myosin heavy chain!K14972|2|5e-06|54.7|aga:AgaP_AGAP004734|PAX-interacting protein 1	gi|78190500|ref|NP_001030294.1|	0	Golgi membrane protein 1 [Mus musculus]
mmu-miR-1190	NM_001037725	-28.9	0.713888	-25.65	153	-	-	gi|83582792|ref|NP_001032814.1|	0	protein FAM117B isoform a [Mus musculus]
mmu-miR-1190	NM_001037740	-35.6	0.082712	-34.27	173	-	-	gi|83627724|ref|NP_001032829.1|	0	protein FAM40B isoform b [Mus musculus]

mmu-miR-1190	NM_001038602	-30.7	0.278724	-33.08	153	GO:0031224,GO:0043296,GO:0050954,	K15183|1|9e-20|100|mgp:100549853|RNA polymerase II elongation factor ELL	gi|84370242|ref|NP_001033691.1|	0	MARVEL domain-containing protein 2 isoform 1 [Mus musculus]
mmu-miR-1190	NM_001038846	-30.6	0.243957	-26.07	169	-	-	gi|84781793|ref|NP_001033935.1|	0	capZ-interacting protein isoform b [Mus musculus]

mmu-miR-1190	NM_001039201	-33.5	0.071341	-26.36	157	GO:0031224,GO:0031090,GO:0003824,	K01101|1|8e-50|199|dme:Dmel_CG17294|4-nitrophenyl phosphatase [EC:3.1.3.41]!K11725|2|2e-44|181|xtr:100498369|phospholysine phosphohistidine inorganic pyrophosphate phosphatase [EC:3.6.1.13.1.3.-]	gi|34849757|gb|AAH58177.1|	1.11E-124	Haloacid dehalogenase-like hydrolase domain containing 2 [Mus musculus]
mmu-miR-1190	NM_001039586	-34.3	0.100791	-30.04	161	GO:0043231,GO:0016301,GO:0032559,GO:0006464,GO:0006796,	K15788|1|0.0|917|mmu:235582|glycerate kinase [EC:2.7.1.31]	gi|88759352|ref|NP_001034675.1|	0	glycerate kinase [Mus musculus]

mmu-miR-1190	NM_001039934	-34.5	0.130822	-31.44	167	GO:0032991,GO:0015630,GO:0030425,GO:0031981,GO:0044297,GO:0008092,GO:0002162,GO:0007019,GO:0006468,GO:0048667,GO:0031175,	K10430|1|0.0|769|mmu:17756|microtubule-associated protein 2	gi|90186270|ref|NP_001035023.1|	0	microtubule-associated protein 2 isoform 1 [Mus musculus]
mmu-miR-1190	NM_001040683	-30.8	0.286179	-27.99	161	GO:0043234,GO:0043231,GO:0006355,	K15157|1|8e-145|515|mmu:94112|mediator of RNA polymerase II transcription subunit	gi|117938842|gb|AAH03221.1|	5.34E-144	Med15 protein [Mus musculus]
mmu-miR-1190	NM_001042577	-32.6	0.088046	-32.45	157	GO:0043231,GO:0046914,GO:0001071,GO:0003677,GO:0006351,GO:0008406,	K09373|1|0.0|702|ptr:469628|LIM homeobox protein 2/9	gi|110611159|ref|NP_001036042.1|	0	LIM/homeobox protein Lhx9 isoform c [Mus musculus]

mmu-miR-1190	NM_001042655	-25.3	0.924633	-50.19	317	GO:0044464,GO:0005099,GO:0032313,	K13136|1|4e-98|360|nvi:100114269|gem associated protein 8!K11452|2|2e-13|78.6|tgu:100222038|chromobox protein 4	gi|74208127|dbj|BAE29165.1|	0	unnamed protein product [Mus musculus]
mmu-miR-1190	NM_001042726	-29.8	0.442005	-26.22	155	GO:0044459,GO:0043005,GO:0016021,GO:0046872,GO:0007613,GO:0016337,GO:0007267,GO:0009952,GO:0002009,	K04600|1|3e-56|221|der:Dere_GG20142|cadherin EGF LAG seven-pass G-type receptor 1 (flamingo)	gi|112181187|ref|NP_001036191.1|	0	protocadherin-8 isoform 2 precursor [Mus musculus]

mmu-miR-1190	NM_001042760	-30.1	0.171363	-25.75	164	GO:0016021,GO:0044459,GO:0012505,GO:0044424,GO:0015238,GO:0008509,GO:0019899,GO:0015291,GO:0015893,GO:0003008,GO:0006812,	K08214|1|2e-168|592|mmu:18400|MFS transporter, OCT family, solute carrier family 22 (organic cation transporter), member 18	gi|112363086|ref|NP_001036225.1|	1.43E-167	solute carrier family 22 member 18 [Mus musculus]
mmu-miR-1190	NM_001043322	-26.6	0.99694	-47.11	315	GO:0070161,GO:0043231,GO:0005856,GO:0008092,GO:0003676,GO:0019904,GO:0043170,GO:0051125,GO:0035108,	K10367|1|0.0|1675|mmu:14260|formin 1	gi|112807205|ref|NP_034360.2|	0	formin-1 isoform 1 [Mus musculus]
mmu-miR-1190	NM_001044308	-27	0.986561	-44.93	319	GO:0034704,GO:0005488,GO:0005245,GO:0006935,GO:0032412,GO:0023052,GO:0070838,	K04856|1|0.0|3385|mmu:239556|voltage-dependent calcium channel T type alpha-1I	gi|157057194|ref|NP_001037773.2|	0	calcium channel, voltage-dependent, alpha 1I subunit [Mus musculus]

mmu-miR-1190	NM_001045513	-28.4	0.908886	-45.73	313	GO:0042995,GO:0016020,GO:0043231,GO:0009987,	-	gi|341604762|ref|NP_001038978.3|	0	Ras association (RalGDS/AF-6) and pleckstrin homology domains 1 [Mus musculus]
mmu-miR-1190	NM_001045514	-25.8	0.984858	-54.85	316	GO:0003676,GO:0006351,	-	gi|113865877|ref|NP_001038979.1|	0	AT-hook-containing transcription factor [Mus musculus]
mmu-miR-1190	NM_001045959	-30.9	0.400883	-25.57	161	GO:0044424,GO:0030695,GO:0004672,GO:0032559,GO:0043383,GO:0000165,GO:0019222,GO:0006796,GO:0006464,	K04413|1|0.0|2156|mmu:50932|misshapen/NIK-related kinase [EC:2.7.11.1]	gi|114052522|ref|NP_001039424.1|	0	misshapen-like kinase 1 isoform 3 [Mus musculus]
mmu-miR-1190	NM_001045964	-30.9	0.402243	-25.57	161	GO:0044424,GO:0030695,GO:0004672,GO:0032559,GO:0043383,GO:0000165,GO:0019222,GO:0006796,GO:0006464,	K04413|1|0.0|2117|rno:303259|misshapen/NIK-related kinase [EC:2.7.11.1]	gi|114052104|ref|NP_001039429.1|	0	misshapen-like kinase 1 isoform 4 [Mus musculus]

mmu-miR-1190	NM_001048148	-27.4	0.568426	-45.3	298	GO:0043231,GO:0046914,GO:0001071,GO:0003677,GO:0007176,GO:0006355,	K13103|1|7e-10|66.6|tsp:Tsp_06173|tuftelin-interacting protein 11	gi|148675464|gb|EDL07411.1|	0	zinc finger, CCCH-type with G patch domain, isoform CRA_a [Mus musculus]
mmu-miR-1190	NM_001077499	-28.2	0.954867	-45.32	309	GO:0044297,GO:0031410,GO:0033267,GO:0005887,GO:0031420,GO:0005272,GO:0032559,GO:0048513,GO:0006935,GO:0034220,GO:0008366,GO:0006814,GO:00083	K04840|1|0.0|3401|mmu:20273|voltage-gated sodium channel type VIII alpha	gi|117414174|ref|NP_001070967.1|	0	sodium channel protein type 8 subunit alpha isoform 1 [Mus musculus]
mmu-miR-1190	NM_001080818	-36.6	0.046713	-32.7	171	GO:0031981,GO:0015630,GO:0005488,GO:0004721,GO:0006470,	K06639|1|0.0|1090|mmu:229776|cell division cycle 14 [EC:3.1.3.48]	gi|124249117|ref|NP_001074287.1|	0	dual specificity protein phosphatase CDC14A isoform 1 [Mus musculus]
mmu-miR-1190	NM_001080940	-31.7	0.041262	-28.12	154	-	-	gi|148693271|gb|EDL25218.1|	1.13E-92	mCG14039 [Mus musculus]

mmu-miR-1190	NM_001080968	-29.2	0.587368	-26.4	159	-	K10352|1|2e-17|92.8|aag:AaeL_AAEL001411|myosin heavy chain	gi|124378030|ref|NP_001074437.1|	0	Golgin subfamily A member 2 isoform b [Mus musculus]
mmu-miR-1190	NM_001080969	-30.5	0.076586	-25.14	161	GO:0043231,GO:0008192,GO:0051260,GO:0008033,	K10761|1|9e-176|616|mmu:66628|tRNA(His) guanylyltransferase [EC:2.7.7.7	gi|124377988|ref|NP_001074438.1|	5.79E-175	probable tRNA(His) guanylyltransferase [Mus musculus]
mmu-miR-1190	NM_001081014	-29	0.706931	-26.9	160	-	K12861|1|5e-09|65.5|gga:419884|pre-mRNA-splicing factor SPF27	gi|124486610|ref|NP_001074483.1|	0	DENN domain-containing protein 4C isoform 2 [Mus musculus]

mmu-miR-1190	NM_001081035	-28.3	0.88508	-25.92	161	-	K05699|1|1e-10|71.6|nve:NEMVE_v1g206855|actinin alpha!K06115|2|3e-09|66.6|phu:Phum_PHUM488290|spectrin beta!K04437|3|3e-09|66.6|nvi:100123445|filamin	gi|124486648|ref|NP_001074504.1|	0	neuron navigator 3 [Mus musculus]
mmu-miR-1190	NM_001081037	-27.2	0.962652	-26.08	159	GO:0044444,GO:0005099,GO:0017016,GO:0048870,GO:0006935,GO:0032319,	K07526|1|0.0|2040|mmu:117600|SLIT-ROBO Rho GTPase activating protein	gi|124486650|ref|NP_001074506.1|	0	SLIT-ROBO Rho GTPase-activating protein 1 isoform 1 [Mus musculus]
mmu-miR-1190	NM_001081039	-36.1	0.107642	-31.83	171	-	K05727|1|6e-16|89.0|tgu:100231732|dedicator of cytokinesis 3/4/5	gi|124486664|ref|NP_001074508.1|	0	dedicator of cytokinesis protein 9 isoform 1 [Mus musculus]

mmu-miR-1190	NM_001081041	-28.2	0.541199	-25.81	159	GO:0031224,GO:0043231,GO:0006810,GO:0010876,	-	gi|148701257|gb|EDL33204.1|	0	mCG11742, isoform CRA_b [Mus musculus]
mmu-miR-1190	NM_001081055	-35	0.098291	-79.06	485	GO:0043231,GO:0031224,GO:0046873,GO:0035725,	-	gi|124486684|ref|NP_001074524.1|	0	trafficking protein particle complex subunit 10 [Mus musculus]
mmu-miR-1190	NM_001081063	-28.7	0.183192	-28.03	167	GO:0031224,GO:0004175,GO:0019538,	K09638|1|4e-45|182|dre:798480|transmembrane protease, serine 7 [EC:3.4.21.-]!K09640|2|5e-45|182|mgp:100551130|transmembrane protease, serine 9 [EC:3.4.21.-]	gi|124486704|ref|NP_001074532.1|	1.37E-180	serine protease 55 precursor [Mus musculus]

mmu-miR-1190	NM_001081092	-25.2	0.987538	-28.65	164	GO:0035097,GO:0005667,GO:0003712,GO:0003676,GO:0001071,GO:0022602,GO:0051704,GO:0006355,GO:0006352,GO:00063	K03129|1|0.0|1068|mmu:228980|transcription initiation factor TFIID subunit 4	gi|123288532|emb|CAM18347.1|	0	TAF4A RNA polymerase II, TATA box binding protein (TBP)-associated factor [Mus musculus]
mmu-miR-1190	NM_001081097	-28.7	0.875989	-25.41	160	GO:0016021,GO:0030425,GO:0043025,GO:0033267,GO:0044459,GO:0005102,GO:0005231,GO:0004970,GO:0004930,GO:0007193,GO:0006811,GO:0006873,GO:00508	K05203|1|0.0|1802|mmu:14807|glutamate receptor, ionotropic kainate 3	gi|124487364|ref|NP_001074566.1|	0	glutamate receptor, ionotropic kainate 3 precursor [Mus musculus]

mmu-miR-1190	NM_001081115	-30.6	0.137033	-28.6	165	-	K10102|1|8e-24|112|xtr:779735|F-box protein 27!K10099|3|8e-19|95.9|ssc:100511640|F-box protein 2!K10100|4|1e-17|92.0|ptr:469799|F-box protein 6	gi|124487435|ref|NP_001074584.1|	8.44E-157	non-specific cytotoxic cell receptor protein 1 homolog [Mus musculus]
mmu-miR-1190	NM_001081155	-35	0.058662	-35.38	170	GO:0043231,GO:0044444,GO:0017111,GO:0008047,GO:0042802,GO:0031267,GO:0043087,GO:0009966,	K04851|1|1e-75|286|xtr:780391|voltage-dependent calcium channel L type alpha-1D!K08013|2|4e-54|214|acs:100553026|signal-induced proliferation-associated gene 1	gi|124487211|ref|NP_001074624.1|	0	rap1 GTPase-activating protein 1 isoform 1 [Mus musculus]

mmu-miR-1190	NM_001081164	-29.4	0.739816	-26.21	164	-	K13718|1|0.0|2034|mmu:73945|OTU domain-containing protein 4	gi|124487191|ref|NP_001074633.1|	0	OTU domain-containing protein 4 isoform 2 [Mus musculus]
mmu-miR-1190	NM_001081166	-28.7	0.602032	-54.83	314	GO:0046914,	K11643|1|2e-10|69.3|smm:Smp_079650|chromodomain-helicase-DNA-binding protein 4 [EC:3.6.4.12]	gi|264681520|ref|NP_001074635.2|	0	PHD finger protein 21B [Mus musculus]
mmu-miR-1190	NM_001081167	-32.3	0.116101	-27.72	163	GO:0043231,GO:0031224,GO:0008375,GO:0016266,	K00739|1|0.0|780|mmu:272411|acetylgalactosaminyl-0-glycosyl-glycoprotein beta-1,3-N-acetylglucosaminyltransferase [EC:2.4.1.147]	gi|124487189|ref|NP_001074636.1|	0	UDP-GlcNAc:betaGal beta-1,3-N-acetylglucosaminyltransferase 6 [Mus musculus]

mmu-miR-1190	NM_001081182	-27.9	0.83903	-26.67	152	GO:0031224,GO:0042625,GO:0046872,GO:0005548,GO:0032559,GO:0009206,GO:0006811,GO:0006869,GO:0009207,	K01530|1|0.0|2229|mmu:54667|phospholipid-translocating ATPase [EC:3.6.3.1]!K14802|5|0.0|2135|pon:100460078|phospholipid-transporting ATPase [EC:3.6.3.1]	gi|124487147|ref|NP_001074651.1|		0	probable phospholipid-transporting ATPase ID [Mus musculus]
mmu-miR-1190	NM_001081220	-26.9	0.985939	-25.54	151	GO:0031224,GO:0004888,	K08469|1|0.0|683|tgu:100229682|G protein-coupled receptor 158	gi|124487045|ref|NP_001074689.1|		0	probable G-protein coupled receptor 179 precursor [Mus musculus]
mmu-miR-1190	NM_001081249	-31.5	0.633577	-28.11	158	GO:0044421,GO:0005539,GO:0016477,GO:0048513,	K06793|1|0.0|5341|rno:114122|chondroitin sulfate proteoglycan 2	gi|148668663|gb|EDL00982.1|		0	mCG116562, isoform CRA_b [Mus musculus]

mmu-miR-1190	NM_001081251	-29.2	0.797628	-26.45	156	GO:0043231,GO:0005694,GO:0003676,GO:0016568,GO:0008283,GO:0000087,	K11757|1|0.0|2834|mmu:66923|protein polybromo-1	gi|124486951|ref|NP_001074720.1|	0	protein polybromo-1 [Mus musculus]
mmu-miR-1190	NM_001081257	-30.1	0.245999	-27.93	161	GO:0044464,GO:0004553,GO:0043167,GO:0044238,	K07965|1|0.0|1087|mmu:545291|heparanase 2 [EC:3.2.1.-]	gi|124486931|ref|NP_001074726.1|	0	heparanase-2 precursor [Mus musculus]
mmu-miR-1190	NM_001081260	-27.8	0.865061	-43.5	306	GO:0044444,GO:0000784,GO:0008092,GO:0006278,GO:0000288,	-	gi|124486923|ref|NP_001074729.1|	0	182 kDa tankyrase-1-binding protein [Mus musculus]
mmu-miR-1190	NM_001081290	-26.7	0.993649	-49.84	311	-	-	gi|124486835|ref|NP_001074759.1|	0	protein PRRC2C [Mus musculus]
mmu-miR-1190	NM_001081291	-25.2	0.992113	-52.18	301	GO:0044424,GO:0015631,GO:0007010,	K08408|1|2e-32|143|cfa:480418|G protein-coupled receptor 68!K06102|3|2e-07|59.7|aml:100476212|cingulin	gi|124486839|ref|NP_001074760.1|	0	coiled-coil domain-containing protein 88B precursor [Mus musculus]

mmu-miR-1190	NM_001081293	-30.7	0.608498	-26.52	160	GO:0005488,	K15559|1|9e-21|105|nvi:100114420|regulator of Ty1 transposition protein 103	gi|124486841|ref|NP_001074762.1|	0	regulation of nuclear pre-mRNA domain-containing protein 2 [Mus musculus]
mmu-miR-1190	NM_001081298	-26.3	0.976199	-41.24	300	GO:0031224,GO:0019904,GO:0030246,GO:0004888,GO:0007417,GO:0007186,	K04593|1|0.0|2921|mmu:99633|latrophilin 2	gi|124486821|ref|NP_001074767.1|	0	latrophilin-2 precursor [Mus musculus]
mmu-miR-1190	NM_001081360	-29.7	0.721254	-47.81	306	GO:0015630,GO:0005488,	-	gi|124486889|ref|NP_001074829.1|	0	calmodulin-regulated spectrin-associated protein 2 [Mus musculus]
mmu-miR-1190	NM_001081401	-30.5	0.599276	-29.04	163	GO:0031012,GO:0004175,GO:0046914,GO:0005539,GO:0007166,GO:0032963,GO:0006508,GO:0030198,	K08619|1|0.0|2476|mmu:330119|a disintegrin and metalloproteinase with thrombospondin motifs 3 [EC:3.4.24.-]	gi|124487141|ref|NP_001074870.1|	0	a disintegrin-like and metalloprotease with thrombospondin type 1 motif, 3 isoform 2 precursor [Mus musculus]

mmu-miR-1190	NM_001081414	-25.5	0.998743	-25.93	163	GO:0044430,GO:0016021,GO:0030425,GO:0031685,GO:0019904,GO:0008066,GO:0006351,GO:0007200,GO:0007611,GO:0048017,GO:0007204,GO:0040011,GO:0006464,GO:0045744,GO:0048169,GO:00001	K04604|1|0.0|2158|mmu:108071|metabotropic glutamate receptor 1/5	gi|126116570|ref|NP_001074883.1|	0	metabotropic glutamate receptor 5 isoform a precursor [Mus musculus]

mmu-miR-1190	NM_001081417	-29.6	0.793457	-44.29	308	GO:0043231,GO:0044427,GO:0017111,GO:0003676,GO:0032559,GO:0006351,GO:0090276,GO:0007507,GO:0001944,GO:0030326,GO:0043009,GO:0050954,GO:0001654,GO:0021675,GO:0042471,GO:0046545,GO:0030098,GO:0035264,GO:0003013,GO:0006325,GO	K14437|1|0.0|3897|mmu:320790|chromodomain-helicase-DNA-binding protein 7 [EC:3.6.4.12]	gi|124487249|ref|NP_001074886.1|	0	chromodomain-helicase-DNA-binding protein 7 [Mus musculus]
mmu-miR-1190	NM_001081432	-31.3	0.466435	-27.78	170	GO:0031224,GO:0004871,GO:0004721,GO:0042471,GO:0006470,GO:0045595,GO:00230	K06252|1|6e-123|444|bfo:BRAFLDRAFT_124749|tenascin	gi|124487427|ref|NP_001074901.1|	0	phosphotidylinositol phosphatase PTPRQ precursor [Mus musculus]

mmu-miR-1190	NM_001081454	-26	0.951203	-44.51	308	GO:0030658,GO:0031224,GO:0005795,GO:0005768,GO:0005798,GO:0044421,GO:0004175,GO:0043121,GO:0043169,GO:0004866,GO:0019899,GO:0007179,GO:0006518,GO:0031330,GO:0032900,GO:0007169,GO:0019058,GO:0031331,GO:0016486,GO:0018200,GO:0016477,GO:00469	K01349|1|0.0|1458|mmu:18550|furin [EC:3.4.21.75]	gi|6754996|ref|NP_035176.1|	0	furin preproprotein [Mus musculus]

mmu-miR-1190	NM_001081977	-27.5	0.868501	-47.82	324	GO:0031224,GO:0043231,GO:0046914,GO:0003824,	K11975|1|1e-167|592|ptr:459008|E3 ubiquitin-protein ligase RNF144 [EC:6.3.2.19]	gi|50510393|dbj|BAD32182.1|	0	mKIAA0161 protein [Mus musculus]
mmu-miR-1190	NM_001082476	-26.3	0.951312	-26.17	161	GO:0005856,GO:0043231,GO:0044444,GO:0046914,GO:0050662,GO:0003824,GO:0000166,GO:0009987,GO:0008152,	K00327|1|5e-71|271|ssc:100170114|NADPH-ferrihemoprotein reductase [EC:1.6.2.4]	gi|126723293|ref|NP_001075945.1|	0	NADPH-dependent diflavin oxidoreductase 1 isoform 1 [Mus musculus]
mmu-miR-1190	NM_001083318	-30.9	0.412733	-28.5	163	GO:0043231,GO:0043234,GO:0001071,GO:0003677,GO:0006357,	K09433|1|0.0|1000|mmu:27049|ETS translocation variant 3	gi|134031941|ref|NP_001076787.1|	0	ETS translocation variant 3 [Mus musculus]

mmu-miR-1190	NM_001081391	-22.8	1	-65.66	464	GO:0031224,	K04011|1|7e-104|381|hsa:1378|complement component (3b/4b) receptor 1	gi|161086986|ref|NP_001074860.2|	0	CUB and sushi domain-containing protein 3 [Mus musculus]
mmu-miR-1191a	NM_001033276	-26.3	0.997867	-25.86	166	GO:0043231,GO:0034708,GO:0016740,GO:0043565,GO:0008283,GO:0048545,GO:0030520,GO:0007292,GO:0016568,	K09187|1|0.0|4273|mmu:381022|histone-lysine N-methyltransferase MLL2 [EC:2.1.1.43]	gi|359718904|ref|NP_001028448.3|	0	histone-lysine N-methyltransferase MLL2 [Mus musculus]
mmu-miR-1191a	NM_001038637	-28.9	0.355021	-25.86	158	GO:0043231,GO:0031234,GO:0017111,GO:0005488,GO:0060089,GO:0001775,GO:0007267,GO:0033762,GO:0009207,GO:0015980,GO:0007599,GO:0007166,	K07826|1|3e-34|148|cfa:610817|guanine nucleotide-binding protein G(I)/G(S)/G(O) subunit gamma-2	gi|148669586|gb|EDL01533.1|	2.73E-35	mCG144516 [Mus musculus]

mmu-miR-1191a	NM_001039181	-27.7	0.757353	-28.03	162	GO:0032991,GO:0031224,GO:0001653,GO:0042802,GO:0048731,GO:0031280,GO:0048017,GO:0007589,GO:0003014,GO:0008015,GO:0007188,GO:0033687,GO:00706	K12325|1|0.0|993|mmu:18162|atrial natriuretic peptide clearance receptor	gi|148671333|gb|EDL03280.1|	0	natriuretic peptide receptor 3, isoform CRA_c [Mus musculus]

mmu-miR-1191a	NM_001110140	-29.3	0.374201	-26.44	161	GO:0032991,GO:0016021,GO:0005789,GO:0001664,GO:0019829,GO:0046872,GO:0032559,GO:0009888,GO:0007599,GO:0008016,GO:0006816,GO:0006936,GO:0009207,GO:0007165,GO:0001775,GO:0009206,GO:0009725,GO:0006875,	K05853|1|0.0|2035|mmu:11938|Ca2+ transporting ATPase, sarcoplasmic/endoplasmic reticulum [EC:3.6.3.8]	gi|60360584|dbj|BAD90532.1|	0	mKIAA4195 protein [Mus musculus]
mmu-miR-1191a	NM_001164561	-31	0.052375	-26.14	187	GO:0043234,GO:0031981,GO:0044444,GO:0003676,GO:0004527,GO:0000291,GO:00104	K07573|1|1e-84|314|mmu:66583|exosome complex component CSL4	gi|148709922|gb|EDL41868.1|	8.66E-90	exosome component 1, isoform CRA_c [Mus musculus]

mmu-miR-1191a	NM_001252498	-30.1	0.298874	-29.09	166	GO:0016020,GO:0031267,GO:0019222,	K08020|1|0.0|730|mmu:192786|Rap guanine nucleotide exchange factor (GEF) 6	gi|357394799|ref|NP_001239427.1|	0	Rap guanine nucleotide exchange factor (GEF) 6 isoform 5 [Mus musculus]
mmu-miR-1191a	NM_008728	-27.7	0.757497	-28.03	162	GO:0032991,GO:0031224,GO:0001653,GO:0042802,GO:0048731,GO:0031280,GO:0048017,GO:0007589,GO:0003014,GO:0008015,GO:0007188,GO:0033687,GO:00706	K12325|1|0.0|985|mmu:18162|atrial natriuretic peptide clearance receptor	gi|148671333|gb|EDL03280.1|	0	natriuretic peptide receptor 3, isoform CRA_c [Mus musculus]
mmu-miR-1191a	NM_009135	-30.8	0.340272	-26.96	174	GO:0005887,GO:0005272,GO:0030001,	K04839|1|0.0|3229|mmu:20272|voltage-gated sodium channel type VII alpha	gi|157058300|ref|NP_033161.2|	0	sodium channel, voltage-gated, type VII, alpha polypeptide [Mus musculus]

mmu-miR-1191a	NM_009722	-29.3	0.359028	-26.44	161	GO:0032991,GO:0016021,GO:0005789,GO:0001664,GO:0019829,GO:0046872,GO:0032559,GO:0009888,GO:0007599,GO:0008016,GO:0006816,GO:0006936,GO:0009207,GO:0007165,GO:0001775,GO:0009206,GO:0009725,GO:0006875,	K05853|1|0.0|1929|mmu:11938|Ca2+ transporting ATPase, sarcoplasmic/endoplasmic reticulum [EC:3.6.3.8]	gi|6806903|ref|NP_033852.1|	0	sarcoplasmic/endoplasmic reticulum calcium ATPase 2 isoform b [Mus musculus]
mmu-miR-1191a	NM_024237	-28.2	0.369024	-26.72	175	GO:0031012,GO:0005539,GO:0046872,GO:0009987,	K08023|1|6e-24|114|mdo:100018129|latent transforming growth factor beta binding protein	gi|148696273|gb|EDL28220.1|	0	RIKEN cDNA 1600015H20, isoform CRA_a [Mus musculus]

mmu-miR-1191a	NM_025644	-31	0.058374	-26.14	187	GO:0043234,GO:0031981,GO:0044444,GO:0003676,GO:0004527,GO:0000291,GO:0042254,GO:0010467,	K07573|1|4e-107|389|rno:679140|exosome complex component CSL4	gi|22267446|ref|NP_079920.1|	2.27E-107	exosome complex component CSL4 isoform 1 [Mus musculus]
mmu-miR-1191a	NM_026166	-32.1	0.088138	-30.63	187	GO:0031090,GO:0043231,GO:0031224,GO:0005488,GO:0012502,GO:0010212,	K10352|1|2e-09|65.9|hmg:100205834|myosin heavy chain!K12478|3|3e-07|58.9|aml:100478615|early endosome antigen 1	gi|121583481|ref|NP_080442.2|	0	inhibitor of nuclear factor kappa-B kinase-interacting protein isoform 1 [Mus musculus]

mmu-miR-1191a	NM_029784	-26.9	0.555981	-26.49	163	-	K10352|1|2e-10|69.3|hmg:100205834|myosin heavy chain!K06674|3|3e-09|65.1|tca:662857|structural maintenance of chromosome 2!K12478|5|2e-08|62.4|xtr:100216061|early endosome antigen 1	gi|58037475|ref|NP_084060.1|	0	protein FAM81A [Mus musculus]

mmu-miR-1191a	NM_033321	-30.8	0.100141	-26.72	167	GO:0016021,GO:0032561,GO:0046914,GO:0005231,GO:0032559,GO:0016502,GO:0048731,GO:0048589,GO:0001508,GO:0035587,GO:0022603,GO:0051259,GO:0009967,GO:00	K05219|1|0.0|884|mmu:94045|purinergic receptor P2X, ligand-gated ion channel 5	gi|238624118|ref|NP_201578.2|	0	purinergic receptor P2X5 [Mus musculus]
mmu-miR-1191a	NM_175210	-29.7	0.525324	-26.12	166	GO:0031224,GO:0017111,GO:0032559,GO:0010876,GO:0051234,GO:0009987,	K05646|1|0.0|5073|mmu:74591|ATP-binding cassette, subfamily A (ABC1), member 12	gi|225703056|ref|NP_780419.2|	0	ATP-binding cassette, sub-family A (ABC1), member 12 [Mus musculus]
mmu-miR-1191a	NM_178918	-29.4	0.326477	-26.12	164	GO:0031981,GO:0042254,	K14549|1|0.0|1016|mmu:105372|U3 small nucleolar RNA-associated protein 15	gi|30725776|ref|NP_849249.1|	0	U3 small nucleolar RNA-associated protein 15 homolog [Mus musculus]

mmu-miR-1191a	NR_040311	-32	0.108708	-28.31	157	-	-	gi|148675117|gb|EDL07064.1|	1.27E-72	mCG144576 [Mus musculus]
mmu-miR-1191a	NR_040312	-32	0.104683	-28.31	157	-	-	gi|148675117|gb|EDL07064.1|	1.23E-72	mCG144576 [Mus musculus]
mmu-miR-1191a	NR_040313	-32	0.102189	-28.31	157	-	-	gi|148675117|gb|EDL07064.1|	1.21E-72	mCG144576 [Mus musculus]
mmu-miR-1191a	NR_040314	-32	0.100581	-28.31	157	-	-	gi|148675117|gb|EDL07064.1|	1.19E-72	mCG144576 [Mus musculus]
mmu-miR-1191a	NM_024180	-22.8	0.974931	-21.83	179	GO:0031090,GO:0043231,GO:0031224,GO:0046519,	K12667|1|4e-35|150|smm:Smp_144800|oligosaccharyltransferase complex subunit delta (ribophorin II)	gi|395744435|ref|XP_003778106.1|	2.78E-76	PREDICTED: ORM1-like protein 2 isoform 2 [Pongo abelii]

mmu-miR-1191a	NM_133857	-29.7	0.289237	-22.54	176	-	K14206|1|3e-31|138|dpe:Dper_GL26826|solute carrier family 15 (oligopeptide transporter), member 1!K15176|2|6e-06|54.7|dgr:Dgri_GH15212|RNA polymerase-associated protein	gi|33469031|ref|NP_598618.1|	0	inactive ubiquitin carboxyl-terminal hydrolase 53 [Mus musculus]
mmu-miR-1191a	NM_007908	-21.5	1	-23.91	175	GO:0044444,GO:0003723,GO:0046872,GO:0005515,GO:0004683,GO:0032559,GO:0006412,GO:0006796,GO:0007169,GO:00064	K08292|1|0.0|1437|mmu:13631|elongation factor 2 kinase [EC:2.7.11.20]	gi|148685284|gb|EDL17231.1|	0	eukaryotic elongation factor-2 kinase, isoform CRA_d [Mus musculus]

mmu-miR-1191a	NM_134250	-21.3	0.999925	-30.06	174	GO:0044464,GO:0004871,GO:0023052,	K15148|1|1e-61|239|cfa:479318|mediator of RNA polymerase II transcription subunit 7	gi|45385110|gb|AAS59531.1|	2.32E-121	T-cell immunoglobulin mucin [Mus musculus]
mmu-miR-1191a	NM_194339	-28.7	0.434157	-23.34	171	GO:0031981,GO:0032561,GO:0042254,	K14569|1|0.0|2115|mmu:213895|ribosome biogenesis protein BMS1	gi|37359828|dbj|BAC97892.1|	0	mKIAA0187 protein [Mus musculus]
mmu-miR-1191a	NM_199152	-25.8	0.999757	-24.82	170	-	K12567|1|0.0|1170|mmu:22138|titin [EC:2.7.11.1]	gi|345842337|ref|NP_954603.3|	0	obscurin isoform 2 [Mus musculus]
mmu-miR-1191a	NM_207666	-28.1	0.197673	-21.43	169	GO:0031224,GO:0046872,GO:0045595,	K06051|1|1e-66|254|ssc:100620481|delta	gi|102468234|ref|NP_997549.2|	0	protein delta homolog 2 [Mus musculus]
mmu-miR-1191a	NM_175088	-29.2	0.299227	-23.48	168	GO:0031981,GO:0008134,GO:0016055,GO:0006355,GO:0006606,GO:0045935,GO:00001	-	gi|74192518|dbj|BAE43047.1|	7.84E-162	unnamed protein product [Mus musculus]

mmu-miR-1191a	NR_037610	-25.3	0.962295	-21.15	167	GO:0005818,GO:0030427,GO:0031981,GO:0072372,GO:0030424,GO:0005875,GO:0005635,GO:0005815,GO:0000775,GO:0044297,GO:0016407,GO:0005539,GO:0042802,GO:0015631,GO:0019899,GO:0045502,GO:0007346,GO:0051301,GO:0003006,GO:0021952,GO:0040001,GO:00215	K01062|1|0.0|850|oaa:100078186|1-alkyl-2-acetylglycerophosphocholine esterase [EC:3.1.1.47]	gi|7305363|ref|NP_038653.1|	0	platelet-activating factor acetylhydrolase IB subunit alpha [Mus musculus]
mmu-miR-1191a	NM_174847	-25.8	0.948724	-20.37	166	GO:0043231,GO:0044444,	-	gi|60360478|dbj|BAD90483.1|	0	mKIAA4045 protein [Mus musculus]
mmu-miR-1191a	NR_045455	-20.8	1	-25.12	165	-	-	gi|148691961|gb|EDL23908.1|	8.64E-23	mCG147816 [Mus musculus]

mmu-miR-1191a	NM_146339	-26.3	0.624076	-21.58	164	GO:0031224,GO:0004930,GO:0007166,GO:0007606,	K04257|1|2e-171|603|mmu:258336|olfactory	gi|22129623|ref|NP_666451.1|	1.55E-170	olfactory receptor 77 [Mus musculus]
mmu-miR-1191a	NR_040569	-23.1	0.946774	-20.34	163	-	-	gi|148665881|gb|EDK98297.1|	3.92E-21	mCG1038666 [Mus musculus]
mmu-miR-1191a	NR_033261	-24.7	0.814033	-23.84	162	-	-	gi|148686210|gb|EDL18157.1|	3.94E-180	mCG145277 [Mus musculus]
mmu-miR-1191a	NR_073452	-22	1	-20.19	161	-	-	-	-	-
mmu-miR-1191a	NM_194446	-23.2	0.91483	-21.46	160	GO:0043231,GO:0004674,GO:0032559,GO:0000080,GO:0006464,GO:0008283,	K02449|1|0.0|678|pon:100435086|cyclin-dependent kinase 10 [EC:2.7.11.	gi|37595744|ref|NP_919428.1|	0	cyclin-dependent kinase 10 isoform 1 [Mus musculus]
mmu-miR-1191a	NM_172938	-21.6	0.999998	-20.94	159	GO:0043231,GO:0003676,	K11466|1|0.0|773|mmu:268297|sex comb on midleg-like protein 4	gi|226437663|ref|NP_766526.3|	0	sex comb on midleg-like protein 4 [Mus musculus]
mmu-miR-1191a	NR_040744	-23.4	0.753063	-20.49	158	-	-	gi|148693419|gb|EDL25366.1|	5.97E-20	mCG64965 [Mus musculus]

mmu-miR-1191a	NM_178749	-26.6	0.708096	-22.99	157	GO:0043169,GO:0004672,GO:0032559,GO:0006464,	K08793|1|0.0|759|mmu:269019|serine/threonine kinase 32 [EC:2.7.11.1]	gi|30520199|ref|NP_848864.1|	0	serine/threonine-protein kinase 32A [Mus musculus]
mmu-miR-1191a	NM_177710	-25.2	0.993447	-22.24	156	GO:0044459,GO:0043232,GO:0008092,GO:0004721,GO:0006470,GO:0030032,GO:0030832,GO:0010769,	K05766|1|0.0|2725|mmu:237860|slingshot	gi|37674238|ref|NP_808378.2|	0	protein phosphatase Slingshot homolog 2 [Mus musculus]
mmu-miR-1191a	NM_172308	-25.3	0.884118	-21.45	155	GO:0043231,GO:0042802,GO:0016879,GO:0032559,GO:0032787,GO:0009256,	K13402|1|0.0|1768|mmu:270685|monofunctional C1-tetrahydrofolate synthase, mitochondrial [EC:6.3.4.3]	gi|148671648|gb|EDL03595.1|	0	methylenetetrahydrofolate dehydrogenase (NADP+ dependent) 1-like, isoform CRA_a [Mus musculus]
mmu-miR-1191a	NR_029441	-25.3	0.803738	-22.87	154	-	-	-	-	-
mmu-miR-1191a	NR_045984	-24.7	0.580054	-21.96	153	-	-	gi|148666212|gb|EDK98628.1|	4.37E-57	mCG1036764 [Mus musculus]

mmu-miR-1191a	NM_031394	-24	0.977948	-24.18	152	GO:0019898,GO:0030133,GO:0048770,GO:0005083,GO:0046914,GO:0035091,GO:0005515,GO:0051047,GO:0019222,GO:0006887,GO:0015031,	K15290|1|3e-16|88.6|spu:373478|synaptotagmin-1	gi|91718886|ref|NP_113571.2|	0	synaptotagmin-like protein 2 isoform 3 [Mus musculus]
mmu-miR-1191a	NM_023816	-24.2	0.984094	-21.37	151	-	K10645|1|3e-16|89.4|aga:AgaP_AGAP011932|E3 ubiquitin-protein ligase mind-bomb [EC:6.3.2.19]!K12460|2|8e-15|84.3|tca:100141654|ankyrin repeat-rich membrane spanning protein	gi|125628652|ref|NP_076305.2|	0	ankyrin repeat domain 36 [Mus musculus]

mmu-miR-1191a	NM_001081430	-24.6	0.971605	-20.27	150	GO:0008080,	K00670|1|3e-141|504|mmu:70646|peptide alpha-N-acetyltransferase [EC:2.3.1.8	gi|124487477|ref|NP_001074899.1|	1.79E-140	N-alpha-acetyltransferase 30 [Mus musculus]
mmu-miR-1191a	NM_175343	-22.9	0.999844	-22.41	149	GO:0019866,GO:0043231,GO:0016614,GO:0050662,GO:0019695,	K00108|1|0.0|1245|mmu:218865|choline dehydrogenase [EC:1.1.99.1]	gi|148692803|gb|EDL24750.1|	0	choline dehydrogenase [Mus musculus]
mmu-miR-1191a	NM_007808	-23.7	0.972804	-21.95	148	GO:0005743,GO:0044425,GO:0009055,GO:0005506,GO:0000737,GO:0072593,GO:0022904,GO:0051234,GO:0006919,GO:00069	K08738|1|2e-56|222|rno:25309|cytochrome c	gi|395857982|ref|XP_003801357.1|	2.98E-56	PREDICTED: uncharacterized protein LOC100960229 [Otolemur garnettii]

mmu-miR-1191a	NM_177270	-24.1	0.979302	-22.65	147	GO:0043231,GO:0004674,GO:0032559,GO:0006464,	K08824|1|0.0|1111|mmu:53886|cyclin-dependent kinase-like [EC:2.7.11.22]	gi|33859825|ref|NP_796244.2|	0	cyclin-dependent kinase-like 2 isoform 2 [Mus musculus]
mmu-miR-1191a	NM_134080	-23.4	0.999972	-23.7	146	GO:0005856,GO:0043231,GO:0031224,GO:0030016,GO:0008092,GO:0007010,GO:0048513,GO:0023052,GO:0048869,	K04437|1|0.0|5205|mmu:286940|filamin	gi|145966915|ref|NP_598841.1|	0	filamin-B [Mus musculus]
mmu-miR-1191a	NM_025719	-20.1	0.999918	-20.45	145	-	-	gi|254553396|ref|NP_079995.2|	1.09E-97	NFKB activating protein-like [Mus musculus]

mmu-miR-1191a	NM_172739	-20.7	1	NA	NA	GO:0043231,GO:0005856,GO:0044444,GO:0004871,GO:0003712,GO:0003676,GO:0005099,GO:0006351,GO:0007166,GO:0014020,GO:0007420,GO:0042462,GO:0006935,GO:0043114,GO:0032319,	K05732|1|0.0|2774|mmu:232906|glucocorticoid receptor DNA-binding factor 1	gi|75677442|ref|NP_766327.3|	0	rho GTPase-activating protein 35 [Mus musculus]
mmu-miR-1191a	NM_029772	-23.6	0.793819	NA	NA	GO:0031224,GO:0042995,GO:0030141,GO:0005488,GO:0005261,GO:0048869,GO:0048870,GO:0048232,GO:0006816,GO:00342	K04849|1|3e-06|53.9|aml:100466608|voltage-dependent calcium channel N type alpha-1B	gi|148709282|gb|EDL41228.1|	0	cation channel, sperm associated 3, isoform CRA_b [Mus musculus]

mmu-miR-1191a	NM_176928	-20	1	NA	NA	GO:0043231,GO:0005488,GO:0006355,	K11798|1|1e-53|212|ssc:100625737|bromodomain and WD repeat domain containing protein 1/3	gi|157057184|ref|NP_795902.1|	6.41E-53	bromodomain and WD repeat-containing protein 1 isoform C [Mus musculus]
mmu-miR-1191a	NM_010840	-21.9	0.999999	NA	NA	GO:0044444,GO:0016646,GO:0000166,GO:0050662,GO:0000097,GO:0001101,GO:0033273,GO:0034097,GO:0006760,	K00297|1|0.0|1272|mmu:17769|methylenetetrahydrofolate reductase (NADPH) [EC:1.5.1.20]	gi|187466137|emb|CAQ51916.1|	0	5,10-methylenetetrahydrofolate reductase [Mus musculus]
mmu-miR-1191a	NM_033526	-25.8	0.790506	NA	NA	GO:0044444,GO:0031090,GO:0043231,GO:0005515,	K04523|1|0.0|810|mmu:94232|ubiquilin	gi|15805016|ref|NP_277068.1|	0	ubiquilin-4 [Mus musculus]
mmu-miR-1191a	NM_012018	-20.7	1	NA	NA	GO:0005815,GO:0005488,GO:0022402,	K12478|1|5e-25|119|ptr:467212|early endosome antigen 1	gi|189458793|ref|NP_036148.2|	0	centriolin isoform 1 [Mus musculus]

mmu-miR-1191a	NM_008442	-20.4	1	NA	NA	GO:0000323,GO:0005819,GO:0003774,GO:0032559,GO:0048869,GO:0048731,GO:0007017,GO:0007067,GO:0007599,	K10393|1|0.0|1271|rno:84391|kinesin family member 2/24	gi|149059280|gb|EDM10287.1|	0	rCG44775, isoform CRA_a [Rattus norvegicus]
mmu-miR-1191a	NM_177073	-25.4	0.78823	NA	NA	GO:0031224,GO:0004871,GO:0008219,GO:0023052,	K05156|1|0.0|739|mmu:320100|tumor necrosis factor receptor superfamily member	gi|31982219|ref|NP_796047.2|	0	tumor necrosis factor receptor superfamily member 19L precursor [Mus musculus]
mmu-miR-1191a	NM_027941	-21.4	0.998881	NA	NA	-	K08727|1|2e-15|85.1|mdo:100032532|nucleotide-binding oligomerization domain-containing protein 1	gi|110625843|ref|NP_082217.1|	0	leucine-rich repeat-containing protein 34 [Mus musculus]
mmu-miR-1191a	NM_001081017	-20.1	1	NA	NA	GO:0044464,	-	gi|313661493|ref|NP_001074486.2|	0	protein unc-79 homolog [Mus musculus]

mmu-miR-1191a	NM_001005847	-20.7	0.998288	NA	NA	GO:0000323,GO:0005856,GO:0004067,GO:0005515,GO:0006530,GO:0006464,	K01444|1|0.0|644|mmu:11593|N4-(beta-N-acetylglucosaminyl)-L-asparaginase [EC:3.5.1.26]	gi|54292135|ref|NP_001005847.1|	0	N(4)-(beta-N-acetylglucosaminyl)-L-asparaginase isoform 1 precursor [Mus musculus]
mmu-miR-1191a	NM_001013385	-20.4	1	NA	NA	GO:0031982,GO:0042734,GO:0016021,GO:0030425,GO:0033267,GO:0001640,GO:0007193,GO:0007611,GO:0008283,GO:0043523,GO:0030030,GO:0010740,GO:0050804,GO:0001505,GO:0007270,GO:0046903,GO:00	K04607|1|0.0|1623|mmu:268934|metabotropic glutamate receptor 4	gi|62945392|ref|NP_001013403.1|	0	metabotropic glutamate receptor 4 precursor [Mus musculus]
mmu-miR-1191a	NM_029859	-20.7	0.99975	NA	NA	-	-	gi|254675160|ref|NP_084135.1|	0	uncharacterized coiled-coil domain-containing protein KIAA1984 [Mus musculus]

mmu-miR-1191a	NM_008379	-24.1	0.995961	NA	NA	GO:0031981,GO:0044444,GO:0005635,GO:0022892,GO:0005048,GO:0046914,GO:0005515,GO:0006915,GO:0051704,GO:0022415,GO:0006606,GO:0000737,	K14293|1|0.0|1672|mmu:16211|importin subunit beta-1	gi|88014720|ref|NP_032405.3|		0	importin subunit beta-1 [Mus musculus]
mmu-miR-1191a	NM_172439	-21.3	0.999992	NA	NA	GO:0030427,GO:0016020,GO:0044424,GO:0031252,GO:0030425,GO:0046030,GO:0034594,GO:0010975,GO:0001933,GO:00311	K01106|1|0.0|1418|mmu:170835|inositol-1,4,5-trisphosphate 5-phosphatase [EC:3.1.3.56]	gi|262231799|ref|NP_766027.2|		0	phosphatidylinositol 4,5-bisphosphate 5-phosphatase A [Mus musculus]
mmu-miR-1191a	NM_001166427	-23.7	0.934306	NA	NA	GO:0005681,GO:0031981,GO:0003723,GO:0008134,GO:0010468,GO:0000377,	K12898|1|0.0|796|rno:64200|heterogeneous nuclear ribonucleoprotein F/H	gi|26345420|dbj|BAC36361.1|		0	unnamed protein product [Mus musculus]

mmu-miR-1191a	NR_028592	-20.8	1	NA	NA	GO:0030532,GO:0016604,GO:0008144,GO:0016859,GO:0044267,GO:0000377,GO:0065003,	K09567|1|1e-101|372|rno:366461|peptidyl-prolyl isomerase H (cyclophilin H) [EC:5.2.1.8]	gi|148698503|gb|EDL30450.1|	8.93E-101	mCG4207, isoform CRA_b [Mus musculus]
mmu-miR-1191a	NM_001085409	-23.6	0.979054	NA	NA	GO:0031224,GO:0005770,GO:0031090,GO:0016723,GO:0046914,GO:0050662,GO:0012502,GO:0000041,GO:0006875,GO:0008152,GO:0015031,	K10142|1|0.0|888|mmu:68428|metalloreductase STEAP3 [EC:1.16.1.-]	gi|28372380|gb|AAO38239.1|	0	Tsap6 [Mus musculus]
mmu-miR-1191a	NM_001168569	-20.1	1	NA	NA	GO:0043231,GO:0044459,GO:0044444,GO:0046872,GO:0005496,GO:0016879,GO:0032559,GO:0006220,GO:0009064,	K01937|1|0.0|1083|mmu:55936|CTP synthase [EC:6.3.4.2]	gi|9055198|ref|NP_061207.1|	0	CTP synthase 2 isoform a [Mus musculus]

mmu-miR-1191a	NM_144812	-22.6	1	NA	NA	GO:0030529,GO:0003676,GO:0006412,GO:0016458,	K13098|1|4e-07|60.8|acs:100556650|RNA-binding protein FUS	gi|67782332|ref|NP_659061.2|	0	trinucleotide repeat-containing gene 6B protein isoform 1 [Mus musculus]
mmu-miR-1191a	NM_145617	-20.2	1	NA	NA	GO:0043231,GO:0044444,GO:0005083,GO:0016879,GO:0051234,GO:0043412,	K10594|1|0.0|8726|mmu:235439|E3 ubiquitin-protein ligase HERC1 [EC:6.3.2.19]	gi|257196144|ref|NP_663592.3|	0	hect domain and RCC1-like domain 1 [Mus musculus]
mmu-miR-1191a	NM_028247	-24.3	0.875569	NA	NA	GO:0044459,GO:0031224,GO:0005275,GO:0006865,GO:0006807,	K08187|1|4e-150|532|mmu:72472|MFS transporter, MCP family, solute carrier family 16 (monocarboxylic acid transporters), member 10	gi|148672996|gb|EDL04943.1|	0	solute carrier family 16 (monocarboxylic acid transporters), member 10, isoform CRA_b [Mus musculus]

mmu-miR-1191a	NM_001130693	-23.8	0.980901	NA	NA	GO:0031224,GO:0032561,GO:0009975,GO:0016301,GO:0032559,GO:0007606,GO:0007165,GO:0006464,	K12321|1|0.0|2109|mmu:14918|guanylate cyclase 2D/E/F [EC:4.6.1.2]	gi|194688157|ref|NP_001124165.1|	0	olfactory guanylyl cyclase GC-D [Mus musculus]
mmu-miR-1191a	NM_001040112	-21.1	1	NA	NA	GO:0000139,GO:0046914,GO:0035091,GO:0005099,GO:0032879,GO:0065008,GO:0031346,GO:0032232,GO:0032012,GO:00323	K12490|1|0.0|2335|mmu:69710|Arf-GAP, Rho-GAP domain, ANK repeat and PH domain-containing protein	gi|187954747|gb|AAI41180.1|	0	ArfGAP with RhoGAP domain, ankyrin repeat and PH domain 1 [Mus musculus]

mmu-miR-1191a	NM_031199	-25.3	0.912344	NA	NA	GO:0043231,GO:0016021,GO:0044421,GO:0060089,GO:0004712,GO:0070851,GO:0009611,GO:0042221,GO:0006874,GO:0043523,GO:0007088,GO:0008284,GO:0007176,GO:0048646,GO:0043066,GO:00001	K08774|1|1e-70|269|mmu:21802|transforming growth factor, alpha	gi|224420|prf||1103301A	9.45E-80	transforming growth factor alpha
mmu-miR-1191a	NM_026861	-20.2	0.999997	NA	NA	GO:0031224,	-	gi|90568036|ref|NP_081137.2|	7.88E-170	ubiquitin-associated domain-containing protein 2 precursor [Mus
mmu-miR-1191a	NM_172769	-20.6	0.999984	NA	NA	GO:0031224,GO:0043231,GO:0031090,GO:0016717,GO:0046996,GO:0046914,GO:0006631,GO:0006695,	K00227|1|1e-174|613|mmu:235293|lathosterol oxidase [EC:1.14.21.6]	gi|27777693|ref|NP_766357.1|	9.41E-174	lathosterol oxidase [Mus musculus]

mmu-miR-1191a	NM_011404	-22.1	0.999753	NA	NA	GO:0031224,GO:0044459,GO:0044444,GO:0015175,GO:0015173,GO:0003823,GO:0019752,GO:0015801,GO:0048869,GO:0048731,GO:0002376,GO:0015803,GO:0006807,GO:00	K13780|1|0.0|864|mmu:20539|solute carrier family 7 (L-type amino acid transporter), member 5	gi|31982764|ref|NP_035534.2|	0	large neutral amino acids transporter small subunit 1 [Mus musculus]
mmu-miR-1191a	NM_148673	-23.7	0.987005	NA	NA	GO:0005681,GO:0016604,GO:0003729,GO:0046914,GO:0003824,GO:0006376,	K12819|1|0.0|1021|mmu:193116|pre-mRNA-processing factor SLU7	gi|148701913|gb|EDL33860.1|	0	DNA segment, Chr 11, ERATO Doi 730, expressed, isoform CRA_b [Mus musculus]

mmu-miR-1191a	NM_011580	-20.4	1	NA	NA	GO:0009986,GO:0005615,GO:0031091,GO:0005102,GO:0005543,GO:0046872,GO:0017134,GO:0005539,GO:0032403,GO:0071813,GO:0043498,GO:0001948,GO:0005518,GO:0007179,GO:0006954,GO:0010466,GO:0042108,GO:0001935,GO:0010595,GO:0045765,GO:0030194,GO:00072	K04659|1|0.0|2311|mmu:21825|thrombospondin	gi|47059073|ref|NP_035710.2|	0	thrombospondin-1 precursor [Mus musculus]

mmu-miR-1191a	NM_023727	-22.1	0.999838	NA	NA	GO:0048856,GO:0050953,	K08282|1|6e-06|54.7|mdo:100012101|non-specific serine/threonine protein kinase [EC:2.7.11.1]	gi|148681027|gb|EDL12974.1|	7.74E-171	RIKEN cDNA 3322402L07, isoform CRA_b [Mus musculus]
mmu-miR-1191a	NM_178696	-20.7	1	NA	NA	GO:0019866,GO:0031224,GO:0043231,GO:0006810,	K15121|1|0.0|652|rno:365841|solute carrier family 25, member 44	gi|148683335|gb|EDL15282.1|	0	RIKEN cDNA B430110G05, isoform CRA_b [Mus musculus]

mmu-miR-1191a	NM_010700	-21.2	1	NA	NA	GO:0016021,GO:0000323,GO:0009986,GO:0034358,GO:0030665,GO:0044459,GO:0010008,GO:0030228,GO:0030169,GO:0046872,GO:0016125,GO:0034381,GO:0043627,GO:0042157,GO:0023052,GO:0043434,GO:0030301,GO:0006897,GO:0051704,GO:0032502,GO	K12473|1|0.0|1764|mmu:16835|low-density lipoprotein receptor	gi|113195700|ref|NP_034830.2|	0	low-density lipoprotein receptor isoform 1 precursor [Mus musculus]
mmu-miR-1191a	NR_015489	-20.4	0.999481	NA	NA	-	-	gi|26386178|dbj|BAC25590.1|	2.51E-53	unnamed protein product [Mus musculus]

mmu-miR-1191a	NM_007977	-20.3	1	NA	NA	GO:0031091,GO:0016020,GO:0044421,GO:0004175,GO:0046914,GO:0001775,GO:0002526,GO:0006887,GO:0072376,GO:00075	K03899|1|0.0|3939|mmu:14069|coagulation factor VIII	gi|238624180|ref|NP_032003.2|	0	coagulation factor VIII isoform 1 precursor [Mus musculus]
mmu-miR-1191a	NM_172875	-21.9	0.994642	NA	NA	GO:0043231,GO:0044444,GO:0008047,GO:0005488,GO:0016831,GO:0006595,GO:0006520,GO:0048232,	K01583|1|0.0|780|rno:366473|arginine decarboxylase [EC:4.1.1.19]	gi|62079257|ref|NP_001014283.1|	0	uncharacterized protein LOC366473 [Rattus norvegicus]
mmu-miR-1191a	NM_001081548	-20.2	1	NA	NA	GO:0031224,GO:0046914,GO:0019787,GO:0043009,GO:0007631,GO:0019941,GO:0007606,GO:0032446,GO:0007635,	K11978|1|0.0|3536|mmu:68795|E3 ubiquitin-protein ligase UBR3 [EC:6.3.2.19]	gi|160948616|ref|NP_001075017.2|	0	E3 ubiquitin-protein ligase UBR3 isoform 1 [Mus musculus]

mmu-miR-1191a	NM_026376	-22.5	0.999995	NA	NA	GO:0031224,GO:0005488,GO:0004888,GO:0001525,GO:0006935,GO:0023052,GO:00606	K06822|1|0.0|3678|mmu:67784|plexin D	gi|51243714|gb|AAT99561.1|	0	plexin D1 [Mus musculus]
mmu-miR-1191a	NM_029686	-24.5	0.996469	NA	NA	GO:0044464,GO:0046872,GO:0030246,GO:0005515,GO:0007186,	K04988|1|0.0|4756|mmu:76645|polycystin 1L2	gi|294862233|ref|NP_083962.4|	0	polycystic kidney disease protein 1-like 2 precursor [Mus musculus]
mmu-miR-1191a	NM_010048	-21	1	NA	NA	GO:0031224,GO:0004871,GO:0030246,GO:0009653,GO:0042221,GO:0009987,GO:0023052,	K03068|1|6e-09|64.7|bta:534450|low density lipoprotein receptor-related protein 5/6	gi|83816901|ref|NP_034178.2|	0	integral membrane protein DGCR2/IDD isoform 1 precursor [Mus musculus]

mmu-miR-1191a	NM_001039669	-25.1	0.832441	NA	NA	-	K00294|1|1e-107|392|gga:419467|1-pyrroline-5-carboxylate dehydrogenase [EC:1.5.1.12]!K07610|4|7e-12|73.9|acs:100560815|desmi	gi|89179308|ref|NP_001034758.1|	0	intermediate filament family orphan 1 isoform a [Mus musculus]
mmu-miR-1191a	NM_133972	-23.4	0.950818	NA	NA	GO:0005488,	K08844|1|1e-09|66.2|bfo:BRAFLDRAFT_119973|leucine-rich repeat kinase 2 [EC:2.7.11.1]	gi|148696855|gb|EDL28802.1|	0	mCG23104, isoform CRA_b [Mus musculus]

mmu-miR-1191a	NM_001253904	-24.8	0.996744	NA	NA	GO:0043234,GO:0044451,GO:0003712,GO:0019899,GO:0005102,GO:0006629,GO:0000122,	K06065|1|0.0|3803|mmu:20602|nuclear receptor co-repressor 2	gi|341942242|sp|Q9WU42.3|NCOR2_MOUSE	0	RecName: Full=Nuclear receptor corepressor 2; Short=N-CoR2; AltName: Full=Silencing mediator of retinoic acid and thyroid hormone receptor; Short=SMRT; Short=SMRTe; AltName: Full=T3 receptor-associating factor; Short=TRAC; AltName: Full=Thyroid-, retinoic-acid-receptor-associated corepressor
mmu-miR-1191a	NM_001145947	-24.1	0.981145	NA	NA	GO:0044431,GO:0043005,GO:0012506,GO:0016021,GO:0004175,GO:0005488,GO:0008242,GO:0009100,GO:0033619,	K04521|1|0.0|920|mmu:23821|beta-site APP-cleaving enzyme 1 (memapsin 2) [EC:3.4.23.46]	gi|225543122|ref|NP_001139419.1|	0	beta-secretase 1 isoform 2 precursor [Mus musculus]

mmu-miR-1191a	NM_001033443	-22.7	0.995539	NA	NA	GO:0004674,GO:0032559,GO:0006464,	K08824|1|0.0|667|mmu:381113|cyclin-dependent kinase-like [EC:2.7.11.22]	gi|84370314|ref|NP_001028615.1|	0	cyclin-dependent kinase-like 4 [Mus musculus]
mmu-miR-1191a	NM_013830	-21.2	1	NA	NA	GO:0005681,GO:0031981,GO:0043232,GO:0005488,GO:0016301,GO:0000377,	K08827|1|0.0|1256|mmu:19134|serine/threonine-protein kinase PRP4 [EC:2.7.11.1]	gi|148708948|gb|EDL40894.1|	0	PRP4 pre-mRNA processing factor 4 homolog B (yeast), isoform CRA_a [Mus musculus]
mmu-miR-1191a	NM_001199044	-29.8	0.446293	NA	NA	-	-	gi|312261233|ref|NP_001185973.1|	0	protein PRRC2A isoform 2 [Mus musculus]
mmu-miR-1191a	NM_028129	-24.2	0.847933	NA	NA	-	K14570|1|0.0|883|rno:309036|RNA exonuclease 1 [EC:3.1.-.-]	gi|148685239|gb|EDL17186.1|	0	RIKEN cDNA 2610020H08, isoform CRA_a [Mus musculus]

mmu-miR-1191a	NM_001195498	-23.1	0.99787	NA	NA	GO:0043232,GO:0004672,GO:0032559,GO:0007165,	K08805|1|0.0|1220|mmu:70762|doublecortin and CaM kinase-like 1 [EC:2.7.11.1]	gi|306922352|ref|NP_001182427.1|	0	serine/threonine-protein kinase DCLK2 isoform 4 [Mus musculus]
mmu-miR-1191b-3p	NM_001004155	-31.1	0.110077	-26.92	167	-	-	gi|21618991|gb|AAH32212.1|	0	9930012K11Rik protein, partial [Mus musculus]
mmu-miR-1191b-3p	NM_001007584	-34.3	0.021539	-31.02	185	-	-	gi|56090519|ref|NP_001007585.1|	6.58E-57	uncharacterized protein LOC432589 [Mus musculus]

mmu-miR-1191b-3p	NM_001025380	-26.2	0.720103	-25.81	159	GO:0031224,GO:0004175,GO:0046914,GO:0005102,GO:0019538,GO:0048232,	K08612|1|0.0|1431|mmu:23793|disintegrin and metalloproteinase domain-containing protein 25 [EC:3.4.24.-]!K08609|3|0.0|853|bta:782699|disintegrin and metalloproteinase domain-containing protein 20 [EC:3.4.24.-]!K08611|4|0.0|813|mmu:135	gi|119392068|ref|NP_001020551.3|	0	testase 9 [Mus musculus]

mmu-miR-1191b-3p	NM_001033528	-28.8	0.631665	-26.19	156	GO:0031981,GO:0016790,GO:0070011,GO:0019941,	K11855|1|0.0|1919|mmu:72344|ubiquitin carboxyl-terminal hydrolase 36/42 [EC:3.1.2.15]	gi|148702709|gb|EDL34656.1|	0	mCG13907, isoform CRA_c [Mus musculus]
mmu-miR-1191b-3p	NM_001037877	-32.9	0.083117	-29.14	172	GO:0043231,GO:0003676,GO:0001071,GO:0006357,	-	gi|148679789|gb|EDL11736.1|	0	transcription factor 25 (basic helix-loop-helix), isoform CRA_b [Mus musculus]
mmu-miR-1191b-3p	NM_001037878	-32.9	0.076239	-29.14	172	GO:0043231,GO:0003676,GO:0001071,GO:0006357,GO:0048513,	-	gi|148679790|gb|EDL11737.1|	0	transcription factor 25 (basic helix-loop-helix), isoform CRA_c [Mus musculus]

mmu-miR-1191b-3p	NM_001081008	-29.7	0.63031	-26.13	179	GO:0005667,GO:0035097,GO:0042393,GO:0003712,GO:0004468,GO:0004672,GO:0032559,GO:0043565,GO:0008134,GO:0070897,GO:0016570,GO:0051318,GO:0060260,GO:0006468,GO:0006354,GO:0031331,GO:0051704,GO:0033554,GO:00000	K03125|1|0.0|3168|mmu:270627|transcription initiation factor TFIID subunit 1	gi|124486596|ref|NP_001074477.1|	0	transcription initiation factor TFIID subunit 1 [Mus musculus]
mmu-miR-1191b-3p	NM_001081300	-29.3	0.570662	-25.51	160	-	K09236|1|0.0|1962|mmu:110796|teashirt	gi|60360598|dbj|BAD90534.1|	0	mKIAA4206 protein [Mus musculus]

mmu-miR-1191b-3p	NM_001081306	-33.5	0.200104	-25.47	167	GO:0016021,GO:0044420,GO:0005488,GO:0004871,GO:0004725,GO:0048667,GO:0006796,GO:0023052,GO:0006464,	K08114|1|0.0|4377|mmu:19283|protein tyrosine phosphatase, receptor-type, Z polypeptide [EC:3.1.3.48]	gi|124486807|ref|NP_001074775.1|		0	receptor-type tyrosine-protein phosphatase zeta precursor [Mus musculus]
mmu-miR-1191b-3p	NM_001081366	-27.1	0.83409	-25.31	153	GO:0046914,	-	gi|148665188|gb|EDK97604.1|		0	mCG141781, isoform CRA_b [Mus musculus]
mmu-miR-1191b-3p	NM_001081378	-33.2	0.204478	-27.13	168	GO:0031224,GO:0044444,GO:0000165,GO:0007169,	K12460|1|0.0|3213|mmu:77480|ankyrin repeat-rich membrane spanning protein	gi|124487039|ref|NP_001074847.1|		0	kinase D-interacting substrate of 220 kDa [Mus musculus]
mmu-miR-1191b-3p	NM_001081379	-27.3	0.93562	-43.7	321	-	K10683|1|4e-31|139|acs:100563125|BRCA1-associated RING domain protein 1 [EC:6.3.2.1	gi|323423025|ref|NP_001074848.2|		0	ankyrin repeat domain 11 [Mus musculus]

mmu-miR-1191b-3p	NM_001112735	-31.1	0.109876	-26.92	167	-	-	gi|21618991|gb|AAH32212.1|	0	9930012K11Rik protein, partial [Mus musculus]
mmu-miR-1191b-3p	NM_001126316	-28	0.26589	-25.08	166	GO:0031224,GO:0016787,	K14351|1|2e-158|559|rno:366498|arylacetamide deacetylase-like 3/4 [EC:3.1.1.-]	gi|187283023|ref|NP_001119788.1|	0	uncharacterized protein LOC329993 [Mus musculus]
mmu-miR-1191b-3p	NM_001128599	-31.2	0.121975	-26.11	159	GO:0031224,GO:0004930,GO:0016918,GO:0007623,GO:0009583,GO:0006464,GO:0050953,GO:0007166,	K04255|1|0.0|742|mmu:30044|r-opsin	gi|190610044|ref|NP_001122071.1|	0	melanopsin isoform 2 [Mus musculus]
mmu-miR-1191b-3p	NM_001145979	-30.7	0.200767	-25.39	160	GO:0032561,GO:0017111,GO:0009207,	K14416|1|2e-18|95.5|nvi:100116941|elongation factor 1 alpha-like protein	gi|148691529|gb|EDL23476.1|	0	GTP binding protein 2, isoform CRA_b [Mus musculus]

mmu-miR-1191b-3p	NM_001163671	-27.1	0.812557	-25.52	154	GO:0031981,GO:0044444,GO:0016505,GO:0032559,GO:0019901,GO:0016909,GO:0070555,GO:0001932,GO:0010557,GO:0010243,GO:0016567,GO:0010742,GO:0031328,GO:0010952,GO:0000904,GO:0009411,GO:0032305,GO:0043408,GO:0009605,GO:0001516,GO:0001819,GO:00073	K04440|1|0.0|798|mmu:26420|c-Jun N-terminal kinase [EC:2.7.11.24]	gi|254750733|ref|NP_001157143.1|	0	mitogen-activated protein kinase 9 isoform beta2 [Mus musculus]

mmu-miR-1191b-3p	NM_001163672	-27.1	0.812881	-25.52	154	GO:0031981,GO:0044444,GO:0016505,GO:0032559,GO:0019901,GO:0016909,GO:0070555,GO:0010557,GO:0010243,GO:0016567,GO:0010742,GO:0031328,GO:0010952,GO:0000904,GO:0009411,GO:0032305,GO:0043408,GO:0009605,GO:0001516,GO:0001819,GO:0007399,GO:00066	K04440|1|0.0|768|rno:50658|c-Jun N-terminal kinase [EC:2.7.11.24]	gi|8394233|ref|NP_059018.1|	0	mitogen-activated protein kinase 9 isoform 1 [Rattus norvegicus]

mmu-miR-1191b-3p	NM_001170978	-33.3	0.11637	-27.13	162	GO:0043234,GO:0044429,GO:0008483,GO:0042802,GO:0019899,GO:0048037,GO:0007618,GO:0010038,GO:0042133,GO:0008217,GO:0009063,GO:0014073,GO:0006950,GO:00	K13524|1|0.0|899|mmu:268860|4-aminobutyrate aminotransferase / (S)-3-amino-2-methylpropionate transaminase [EC:2.6.1.19 2.6.1.22]	gi|148664876|gb|EDK97292.1|	0	4-aminobutyrate aminotransferase, isoform CRA_c [Mus musculus]
mmu-miR-1191b-3p	NM_001253752	-28.2	0.699689	-25.07	162	GO:0043231,GO:0046914,	K02981|1|6e-39|149|ptr:456848|small subunit ribosomal protein S2e	gi|140972030|ref|NP_659091.3|	0	zinc finger MYM-type protein 5 [Mus musculus]
mmu-miR-1191b-3p	NM_001253753	-28.2	0.704358	-25.07	162	GO:0043231,GO:0046914,	K02981|1|6e-39|149|ptr:456848|small subunit ribosomal protein S2e	gi|140972030|ref|NP_659091.3|	0	zinc finger MYM-type protein 5 [Mus musculus]

mmu-miR-1191b-3p	NM_001253863	-31.5	0.205796	-26.31	163	GO:0043231,GO:0043234,GO:0001071,GO:0043565,GO:0006355,GO:0032501,	K15603|1|0.0|905|ssc:100512080|transcription factor 4/12	gi|359718979|ref|NP_001240792.1|	0	transcription factor 12 isoform 3 [Mus musculus]
mmu-miR-1191b-3p	NM_001253864	-31.5	0.202011	-26.31	163	GO:0043234,GO:0043231,GO:0003676,GO:0032501,GO:0006355,	K15603|1|0.0|862|cfa:100684199|transcription factor 4/12	gi|359718981|ref|NP_001240793.1|	0	transcription factor 12 isoform 4 [Mus musculus]
mmu-miR-1191b-3p	NM_001253865	-31.5	0.214915	-26.31	163	GO:0043231,GO:0043234,GO:0001071,GO:0043565,GO:0006355,GO:0032501,	K15603|1|0.0|832|mmu:21406|transcription factor 4/12	gi|359718981|ref|NP_001240793.1|	0	transcription factor 12 isoform 4 [Mus musculus]
mmu-miR-1191b-3p	NM_007834	-27.8	0.465671	-25.14	157	GO:0012505,GO:0043231,GO:0046907,	-	gi|6681145|ref|NP_031860.1|	1.03E-157	Down syndrome critical region protein 3 homolog [Mus musculus]
mmu-miR-1191b-3p	NM_007882	-31.1	0.309157	-26.64	171	GO:0005911,GO:0031224,GO:0044424,GO:0046872,GO:0005515,GO:0043009,GO:0016337,GO:0031647,	K07602|1|0.0|1769|mmu:13507|desmocollin 3	gi|133778947|ref|NP_031908.3|	0	desmocollin-3 precursor [Mus musculus]

mmu-miR-1191b-3p	NM_008254	-29.8	0.124753	-27.33	154	GO:0019866,GO:0044429,GO:0043169,GO:0016833,GO:0005504,GO:0033993,GO:0044249,GO:0006996,GO:0031667,GO:0006732,GO:0048513,GO:0006950,GO:00434	K01640|1|0.0|638|mmu:15356|hydroxymethylglutaryl-CoA lyase [EC:4.1.3.4]	gi|171543858|ref|NP_032280.2|	0	hydroxymethylglutaryl-CoA lyase, mitochondrial precursor [Mus musculus]
mmu-miR-1191b-3p	NM_009182	-26.9	0.909748	-25.87	163	GO:0031228,GO:0008373,GO:0006687,GO:0006487,	K06613|1|0.0|752|pon:100448743|alpha-N-acetyl-neuraminate alpha-2,8-sialyltransferase (sialyltransferase 8C)	gi|390474021|ref|XP_003734712.1|	0	PREDICTED: LOW QUALITY PROTEIN: sia-alpha-2,3-Gal-beta-1,4-GlcNAc-R:alpha 2,8-sialyltransferase [Callithrix jacchus]

mmu-miR-1191b-3p	NM_009307	-32	0.141036	-29.71	180	GO:0031224,GO:0008021,GO:0044459,GO:0030139,GO:0030667,GO:0046872,GO:0005543,GO:0006887,GO:0001505,	K15290|1|1e-132|475|tgu:751605|synaptotagmin-1	gi|31543797|ref|NP_033333.2|	0	synaptotagmin-2 [Mus musculus]

mmu-miR-1191b-3p	NM_010009	-28.6	0.359951	-25.91	158	GO:0031966,GO:0005506,GO:0016709,GO:0030278,GO:0055065,GO:0003006,GO:0007589,GO:0044237,GO:0006694,GO:0008283,GO:0006775,GO:0048545,GO:0030858,GO:0010979,GO:0007050,GO:0034097,GO:0043434,GO:0009967,GO:0033273,GO:0010038,GO:0070838,GO:00327	K07438|1|0.0|940|mmu:13115|cytochrome P450, family 27, subfamily B (25-hydroxyvitamin D3 1alpha-hydroxylase) [EC:1.14.13.13]	gi|2443371|dbj|BAA22434.1|	0	25-hydroxyvitamin D3 1alpha-hydroxylase [Mus musculus]
mmu-miR-1191b-3p	NM_010124	-31	0.099613	-27.42	171	GO:0008135,GO:0031369,GO:0007169,GO:0019935,GO:0006446,	K07205|1|2e-29|131|rno:116636|eukaryotic translation initiation factor 4E	gi|6753742|ref|NP_034254.1|	6.44E-58	eukaryotic translation initiation factor 4E-binding protein 2 [Mus musculus]

mmu-miR-1191b-3p	NM_011456	-26.7	0.54499	-25.01	152	GO:0043231,GO:0044444,GO:0044421,GO:0019899,GO:0004869,GO:0002444,GO:0043627,GO:0002437,GO:0001909,GO:0006508,GO:0010466,GO:0070229,	K13963|1|0.0|772|mmu:20710|serpin B	gi|15826846|ref|NP_035586.1|	0	serine (or cysteine) proteinase inhibitor, clade B, member 9e [Mus musculus]
mmu-miR-1191b-3p	NM_011544	-31.5	0.240479	-26.31	163	GO:0043231,GO:0043234,GO:0001071,GO:0043565,GO:0006355,GO:0002376,GO:00485	K15603|1|0.0|1282|mmu:21406|transcription factor 4/12	gi|166295206|ref|NP_035674.2|	0	transcription factor 12 isoform 1 [Mus musculus]
mmu-miR-1191b-3p	NM_011843	-30.8	0.243464	-27.2	171	GO:0031224,	K00923|1|6e-16|87.8|mdo:100017523|phosphatidylinositol-4-phosphate 3-kinase [EC:2.7.1.1	gi|33859650|ref|NP_035973.1|	0	extended synaptotagmin-1 [Mus musculus]

mmu-miR-1191b-3p	NM_011866	-31.2	0.409924	-29.33	167	GO:0044444,GO:0043025,GO:0043169,GO:0019002,GO:0004114,GO:0016208,GO:0006195,GO:0001775,GO:0023052,GO:00075	K01120|1|0.0|1624|mmu:23984|3',5'-cyclic-nucleotide phosphodiesterase [EC:3.1.4.17]	gi|70909347|ref|NP_035996.2|	0	cAMP and cAMP-inhibited cGMP 3',5'-cyclic phosphodiesterase 10A [Mus musculus]
mmu-miR-1191b-3p	NM_013848	-29.5	0.455612	-28.81	172	GO:0031982,GO:0031224,	K06712|1|0.0|1079|mmu:27028|butyrophilin	gi|148698520|gb|EDL30467.1|	0	erythroblast membrane-associated protein, isoform CRA_a [Mus musculus]
mmu-miR-1191b-3p	NM_013887	-31.2	0.115355	-26.11	159	GO:0031224,GO:0004930,GO:0016918,GO:0007623,GO:0009583,GO:0006464,GO:0050953,GO:0007166,	K04255|1|0.0|717|mmu:30044|r-opsin	gi|148692938|gb|EDL24885.1|	0	opsin 4 (melanopsin) [Mus musculus]
mmu-miR-1191b-3p	NM_016806	-30.4	0.309592	-27.1	167	GO:0005681,GO:0031981,GO:0003697,GO:0000377,GO:0050657,	K13158|1|1e-106|389|ptr:463299|heterogeneous nuclear ribonucleoprotein A2/B1	gi|148666221|gb|EDK98637.1|	1.26E-113	mCG119114, isoform CRA_a [Mus musculus]

mmu-miR-1191b-3p	NM_016961	-27.1	0.812881	-25.52	154	GO:0031981,GO:0044444,GO:0016505,GO:0032559,GO:0019901,GO:0016909,GO:0070555,GO:0001932,GO:0010557,GO:0010243,GO:0016567,GO:0010742,GO:0031328,GO:0010952,GO:0000904,GO:0009411,GO:0032305,GO:0043408,GO:0009605,GO:0001516,GO:0001819,GO:00073	K04440|1|0.0|767|mmu:26420|c-Jun N-terminal kinase [EC:2.7.11.24]	gi|254750733|ref|NP_001157143.1|	0	mitogen-activated protein kinase 9 isoform beta2 [Mus musculus]
mmu-miR-1191b-3p	NM_017467	-29.9	0.545369	-26.13	179	GO:0044464,GO:0046914,GO:0003676,GO:0006351,	K09228|1|0.0|1679|mmu:54201|KRAB domain-containing zinc finger protein	gi|255708390|ref|NP_059495.3|	0	zinc finger protein 316 [Mus musculus]

mmu-miR-1191b-3p	NM_018763	-33.1	0.179229	-28.91	170	GO:0000139,GO:0031224,GO:0008146,GO:0006952,GO:0032501,GO:0006041,	K04745|1|0.0|807|mmu:54371|carbohydrate 6-sulfotransferase 2 [EC:2.8.2.-]	gi|148688988|gb|EDL20935.1|	0	carbohydrate sulfotransferase 2 [Mus musculus]
mmu-miR-1191b-3p	NM_019412	-30.2	0.410805	-25.61	161	GO:0043231,GO:0016020,GO:0005488,GO:0007272,	K12567|1|3e-33|145|aml:100476638|titin [EC:2.7.11.1]	gi|170172589|ref|NP_932165.2|	0	periaxin isoform L [Mus musculus]
mmu-miR-1191b-3p	NM_019517	-29.3	0.412617	-25.74	171	GO:0031224,GO:0043231,GO:0004175,GO:0010561,GO:0033619,	K07747|1|0.0|944|mmu:56175|beta-site APP-cleaving enzyme 2 (memapsin 1) [EC:3.4.23.45]	gi|244798416|ref|NP_062390.3|	0	beta-secretase 2 precursor [Mus musculus]
mmu-miR-1191b-3p	NM_019581	-30.7	0.203293	-25.39	160	GO:0032561,GO:0017111,GO:0009207,	K14416|1|2e-18|95.5|nvi:100116941|elongation factor 1 alpha-like protein	gi|225543388|ref|NP_062527.2|	0	GTP-binding protein 2 isoform 1 [Mus musculus]

mmu-miR-1191b-3p	NM_019649	-26.3	0.874698	-26.18	162	GO:0009986,GO:0016021,GO:0033077,	-	gi|103472025|ref|NP_062623.2|	0	cleft lip and palate transmembrane protein 1 homolog [Mus musculus]
mmu-miR-1191b-3p	NM_019986	-31.1	0.148527	-27.93	179	GO:0044444,GO:0005488,GO:0007599,GO:0009987,	K13199|1|1e-114|414|bta:514807|plasminogen activator inhibitor 1 RNA-binding protein	gi|160333783|ref|NP_064370.2|	7.71E-143	intracellular hyaluronan-binding protein 4 [Mus musculus]
mmu-miR-1191b-3p	NM_025448	-31	0.05791	-27.81	163	GO:0031090,GO:0043231,GO:0031224,GO:0042277,GO:0006612,	K13250|1|7e-93|341|rno:295235|translocon-associated protein subunit beta	gi|148683321|gb|EDL15268.1|	2.29E-108	signal sequence receptor, beta, isoform CRA_c [Mus musculus]

mmu-miR-1191b-3p	NM_025768	-32.2	0.038748	-26.43	157	GO:0044464,GO:0005099,GO:0032313,	K11837|1|2e-14|81.3|hsa:9098|ubiquitin carboxyl-terminal hydrolase 6/32 [EC:3.1.2.15]!K13136|2|4e-08|60.1|nvi:100114269|gem associated protein 8	gi|85861226|ref|NP_080044.2|	0	growth hormone-regulated TBC protein 1 [Mus musculus]
mmu-miR-1191b-3p	NM_025804	-32.9	0.074752	-29.14	172	GO:0043231,GO:0003676,GO:0001071,GO:0006357,	-	gi|148679789|gb|EDL11736.1|	0	transcription factor 25 (basic helix-loop-helix), isoform CRA_b [Mus musculus]
mmu-miR-1191b-3p	NM_026248	-32.7	0.049571	-29.8	172	-	K15502|1|2e-24|114|mcc:100428048|serine/threonine-protein phosphatase 6 regulatory ankyrin repeat subunit A	gi|258645154|ref|NP_080524.2|	0	uncharacterized protein LOC67575 [Mus musculus]

mmu-miR-1191b-3p	NM_027994	-27.9	0.871861	-42.67	314	GO:0043231,GO:0043234,GO:0008134,GO:0048869,GO:0045898,GO:0044092,GO:00324	-	gi|37360114|dbj|BAC98035.1|	0	mKIAA0829 protein [Mus musculus]
mmu-miR-1191b-3p	NM_029457	-28.8	0.549914	-25.71	168	GO:0016604,GO:0005635,GO:0031410,GO:0005515,GO:0019783,GO:0006351,GO:0007166,GO:0050658,GO:0043393,GO:0001932,GO:0009798,GO:0070646,	K03345|1|0.0|1085|mmu:75826|sentrin-specific protease 2 (axin associating molecule) [EC:3.4.22.-]	gi|13386400|ref|NP_083733.1|	0	sentrin-specific protease 2 [Mus musculus]
mmu-miR-1191b-3p	NM_029748	-29.9	0.05263	-26.13	179	GO:0031224,	-	gi|22267454|ref|NP_084024.1|	1.42E-66	transmembrane protein 234 precursor [Mus musculus]
mmu-miR-1191b-3p	NM_030198	-30.9	0.148787	-25.18	175	GO:0043231,GO:0006259,	K10734|1|2e-121|437|mmu:78833|GINS complex subunit 3	gi|21313504|ref|NP_084474.1|	1.31E-120	DNA replication complex GINS protein PSF3 [Mus musculus]

mmu-miR-1191b-3p	NM_030743	-33	0.081899	-27.19	164	GO:0044464,GO:0046914,GO:0048232,GO:0048869,	K15697|1|2e-125|451|mmu:81018|RING finger protein 114	gi|27229275|ref|NP_109668.2|	1.12E-124	RING finger protein 114 [Mus musculus]
mmu-miR-1191b-3p	NM_032540	-33.7	0.049105	-28.14	169	GO:0031224,GO:0004175,GO:0043169,GO:0019538,GO:0050880,	K06577|1|0.0|1381|mmu:23925|Kell blood group glycoprotein [EC:3.4.24.-]	gi|160333081|ref|NP_115929.3|	0	kell blood group glycoprotein homolog [Mus musculus]
mmu-miR-1191b-3p	NM_080445	-29.9	0.294561	-26.13	179	GO:0000139,GO:0031985,GO:0031224,GO:0035250,GO:0006464,GO:0006023,	K00734|1|0.0|647|mmu:117592|galactosylxylosylprotein 3-beta-galactosyltransferase [EC:2.4.1.134]	gi|17978260|ref|NP_536693.1|	0	beta-1,3-galactosyltransferase 6 [Mus musculus]
mmu-miR-1191b-3p	NM_133187	-29.6	0.574459	-25.44	163	GO:0012505,GO:0031224,GO:0043231,	-	gi|134032028|ref|NP_573450.2|	0	protein FAM198B [Mus musculus]

mmu-miR-1191b-3p	NM_133981	-38.7	0.006643	-33.57	182	GO:0031224,GO:0005789,GO:0000030,GO:0006497,GO:0006487,	K03846|1|0.0|1073|mmu:102580|alpha-1,2-mannosyltransferase [EC:2.4.1.259	gi|19527202|ref|NP_598742.1|	0	alpha-1,2-mannosyltransferase ALG9 [Mus musculus]
mmu-miR-1191b-3p	NM_134099	-35	0.043201	-30.64	175	GO:0031461,GO:0042802,GO:0019787,GO:0043161,GO:0006259,GO:0016567,	K10291|1|0.0|790|rno:310363|F-box protein 4	gi|157818251|ref|NP_001101142.1|	0	F-box only protein 4 [Rattus norvegicus]
mmu-miR-1191b-3p	NM_144842	-28.2	0.703763	-25.07	162	GO:0043231,GO:0046914,	K02981|1|6e-39|149|ptr:456848|small subunit ribosomal protein S2e	gi|140972030|ref|NP_659091.3|	0	zinc finger MYM-type protein 5 [Mus musculus]
mmu-miR-1191b-3p	NM_145823	-30.8	0.396916	-26.23	160	GO:0043231,GO:0005548,GO:0005488,GO:0010876,GO:0051234,GO:0023052,GO:0006650,	K01106|1|3e-45|186|gga:771549|inositol-1,4,5-trisphosphate 5-phosphatase [EC:3.1.3.56]	gi|351710336|gb|EHB13255.1|	1.40E-174	Cytoplasmic phosphatidylinositol transfer protein 1 [Heterocephalus glaber]

mmu-miR-1191b-3p	NM_172124	-28.5	0.583774	-25.07	153	GO:0012505,GO:0043231,GO:0031224,GO:0015020,GO:0043169,GO:0005975,	K10157|1|0.0|663|mmu:280645|galactosylgalactosylxylosylprotein 3-beta-glucuronosyltransferase 2 [EC:2.4.1.135]	gi|60360092|dbj|BAD90265.1|	0	mKIAA1963 protein [Mus musculus]
mmu-miR-1191b-3p	NM_172285	-30.6	0.305769	-27	172	GO:0016020,GO:0004629,GO:0060089,GO:0005488,GO:0007599,GO:0010467,GO:0012501,GO:0050851,GO:0032957,GO:0051480,GO:0010038,GO:0002237,GO:0006644,GO:00	K05859|1|0.0|2536|mmu:234779|phosphatidylinositol phospholipase C, gamma-2 [EC:3.1.4.11]	gi|148679636|gb|EDL11583.1|	0	phospholipase C, gamma 2 [Mus musculus]
mmu-miR-1191b-3p	NM_172719	-32.2	0.326627	-27.78	172	GO:0030529,GO:0003723,GO:0006412,	-	gi|112807186|ref|NP_766307.2|	0	GCN1 general control of amino-acid synthesis 1-like 1 [Mus musculus]
mmu-miR-1191b-3p	NM_172868	-30.6	0.509081	-27.53	183	GO:0016020,GO:0065008,	-	gi|27777697|ref|NP_766456.1|	0	paralemmin-2 [Mus musculus]

mmu-miR-1191b-3p	NM_172874	-30.2	0.261482	-25.91	155	GO:0031012,GO:0044424,GO:0005515,GO:0016477,GO:0008283,	K08764|1|0.0|904|cfa:479564|sterol carrier protein 2 [EC:2.3.1.176]	gi|32526861|ref|NP_766462.2|	0	podocan precursor [Mus musculus]
mmu-miR-1191b-3p	NM_172961	-33.3	0.121263	-27.13	162	GO:0043234,GO:0044429,GO:0008483,GO:0042802,GO:0019899,GO:0048037,GO:0007618,GO:0010038,GO:0042133,GO:0008217,GO:0009063,GO:0014073,GO:0006950,GO:00	K13524|1|0.0|1001|rno:81632|4-aminobutyrate aminotransferase / (S)-3-amino-2-methylpropionate transaminase [EC:2.6.1.19 2.6.1.22]	gi|37202121|ref|NP_766549.2|	0	4-aminobutyrate aminotransferase, mitochondrial isoform 1 precursor [Mus musculus]
mmu-miR-1191b-3p	NM_173437	-25.9	0.998422	-44.7	319	-	-	gi|224922751|ref|NP_775613.2|	0	neuron navigator 1 [Mus musculus]
mmu-miR-1191b-3p	NM_175692	-30	0.474113	-51.03	350	-	-	gi|26348733|dbj|BAC38006.1|	2.84E-55	unnamed protein product [Mus musculus]

mmu-miR-1191b-3p	NM_177647	-31.7	0.139205	-27.93	179	GO:0005102,	K13190|1|2e-46|188|gga:770664|RNA-binding protein	gi|148675989|gb|EDL07936.1|	8.66E-117	arginine-rich, mutated in early stage tumors-like 1 [Mus musculus]
mmu-miR-1191b-3p	NM_183197	-26.7	0.544781	-25.01	152	GO:0043231,GO:0044444,GO:0044421,GO:0019899,GO:0004869,GO:0002444,GO:0043627,GO:0002437,GO:0001909,GO:0006508,GO:0010466,GO:0070229,	K13963|1|0.0|741|mmu:544923|serpin B	gi|377835960|ref|XP_619089.3|	0	PREDICTED: LOW QUALITY PROTEIN: uncharacterized protein LOC544923, partial [Mus musculus]
mmu-miR-1191b-3p	NM_198048	-30.2	0.372293	-25.61	161	GO:0043231,GO:0016020,GO:0005488,GO:0007272,	K12567|1|3e-33|145|aml:100476638|titin [EC:2.7.11.1]	gi|170172589|ref|NP_932165.2|	0	periaxin isoform L [Mus musculus]

mmu-miR-1191b-3p	NM_207692	-27.1	0.812557	-25.52	154	GO:0031981,GO:0044444,GO:0016505,GO:0032559,GO:0019901,GO:0016909,GO:0070555,GO:0001932,GO:0010557,GO:0010243,GO:0016567,GO:0010742,GO:0031328,GO:0010952,GO:0000904,GO:0009411,GO:0032305,GO:0043408,GO:0009605,GO:0001516,GO:0001819,GO:00073	K04440|1|0.0|799|rno:50658|c-Jun N-terminal kinase [EC:2.7.11.24]	gi|8394233|ref|NP_059018.1|	0	mitogen-activated protein kinase 9 isoform 1 [Rattus norvegicus]

mmu-miR-1191b-3p	NR_027488	-28.8	0.555414	-25.71	168	-	K03345|1|0.0|1020|mmu:75826|sentrin-specific protease 2 (axin associating molecule) [EC:3.4.22.-]	gi|13386400|ref|NP_083733.1|	0	sentrin-specific protease 2 [Mus musculus]
mmu-miR-1191b-3p	NR_036594	-28.4	0.167554	-25.39	170	-	-	gi|148705208|gb|EDL37155.1|	1.28E-33	mCG1050052 [Mus musculus]
mmu-miR-1191b-3p	NR_037865	-27.3	0.937318	-43.7	321	-	K10683|1|1e-29|134|acs:100563125|BRCA1-associated RING domain protein 1 [EC:6.3.2.1	gi|219521157|gb|AAI72141.1|	0	Ankrd11 protein [Mus musculus]
mmu-miR-1191b-3p	NR_045601	-38.7	0.006389	-33.57	182	GO:0031224,GO:0005789,GO:0000030,GO:0006497,GO:0006487,	K03846|1|0.0|800|mmu:102580|alpha-1,2-mannosyltransferase [EC:2.4.1.259	gi|148693819|gb|EDL25766.1|	0	asparagine-linked glycosylation 9 homolog (yeast, alpha 1,2 mannosyltransferase) [Mus musculus]

mmu-miR-1191b-3p	NR_001461	-22.6	1	-63.78	477	-	K11849|1|6e-55|213|acs:100555596|ubiquitin carboxyl-terminal hydrolase 25/28 [EC:3.1.2.15]!K06478|4|4e-43|182|acs:100563672|protein tyrosine phosphatase, receptor type, C [EC:3.1.3.4	gi|209553980|gb|AAA39398.2|	0	ORF2 [Mus musculus domesticus]
mmu-miR-1191b-3p	NM_001081064	-21.4	1	-42.63	308	GO:0030054,GO:0043231,GO:0044430,GO:0007165,	K06095|1|5e-31|139|gga:427360|multiple PDZ domain protein	gi|124486716|ref|NP_001074533.1|	0	PDZ domain-containing protein 2 [Mus musculus]
mmu-miR-1191b-3p	NR_028425	-23.9	0.958773	-41.21	305	-	-	-	-	-
mmu-miR-1191b-3p	NM_001163528	-20.9	1	-27.72	180	GO:0031224,	-	gi|148696226|gb|EDL28173.1|	0	mCG51762 [Mus musculus]

mmu-miR-1191b-3p	NM_009884	-24.3	0.995246	-26.13	179	GO:0031981,GO:0001071,GO:0046983,GO:0042802,GO:0043566,GO:0006355,GO:0034728,GO:0043392,GO:0006282,GO:0030218,GO:0030098,GO:0043388,GO:0042108,GO:0016568,GO:0001909,	K10049|1|7e-57|224|mmu:12611|CCAAT/enhancer binding protein (C/EBP), gamma	gi|55928|emb|CAA45745.1|	5.44E-84	CCAAT /enhancer binding protein gamma [Rattus norvegicus]
mmu-miR-1191b-3p	NM_181278	-26.3	0.90316	-22.2	176	-	-	gi|148709277|gb|EDL41223.1|	5.91E-123	RIKEN cDNA B230219D22 [Mus musculus]
mmu-miR-1191b-3p	NM_178667	-22.6	1	-25.1	174	GO:0043231,GO:0043234,GO:0012505,GO:0000989,GO:0003676,GO:0001071,GO:0005515,GO:0006351,	K09392|1|0.0|649|aml:100464401|transcription factor Dp-2	gi|8039811|sp|Q64163.2|TFDP2_MOUSE	0	RecName: Full=Transcription factor Dp-2; AltName: Full=Dp-3; AltName: Full=E2F dimerization partner 2
mmu-miR-1191b-3p	NM_025419	-26.4	0.802352	-22.78	173	-	-	gi|31541808|ref|NP_079695.2|	1.74E-81	uncharacterized protein KIAA1143 homolog [Mus musculus]

mmu-miR-1191b-3p	NM_001081056	-26.5	0.932517	-22.53	172	GO:0005635,GO:0031981,GO:0003723,GO:0022892,GO:0006405,GO:0015031,	K14288|1|0.0|1865|mmu:73192|exportin-T	gi|124486686|ref|NP_001074525.1|	0	exportin-T [Mus musculus]
mmu-miR-1191b-3p	NM_172450	-20.4	1	-26.98	171	-	-	gi|148690646|gb|EDL22593.1|	0	RIKEN cDNA 4930539E08, isoform CRA_a [Mus musculus]
mmu-miR-1191b-3p	NR_040556	-26.5	0.551841	-21.06	170	-	-	gi|45478140|gb|AAS66241.1|	1.29E-13	LRRGT00150 [Rattus norvegicus]
mmu-miR-1191b-3p	NM_001144855	-27.7	0.813944	-22.65	169	-	K10352|1|2e-14|83.6|ecb:100054482|myosin heavy chain	gi|221554491|ref|NP_001138327.1|	0	liprin-alpha-4 [Mus musculus]
mmu-miR-1191b-3p	NM_029211	-26.6	0.463007	-22	168	GO:0031224,GO:0046914,	K15698|1|2e-174|612|rno:308871|RING finger protein 121	gi|70778932|ref|NP_083487.2|	1.65E-173	RING finger protein 121 [Mus musculus]
mmu-miR-1191b-3p	NR_045075	-26	0.292039	-20.59	167	-	-	gi|148700458|gb|EDL32405.1|	5.97E-43	mCG146041 [Mus musculus]
mmu-miR-1191b-3p	NR_028286	-25.8	0.362927	-22.75	166	-	-	gi|148696403|gb|EDL28350.1|	5.45E-25	mCG67504, isoform CRA_a [Mus musculus]

mmu-miR-1191b-3p	NM_021530	-21.6	1	-24.2	165	GO:0031224,GO:0015301,GO:0015698,GO:0030001,	K13859|1|0.0|2114|mmu:59033|solute carrier family 4 (sodium bicarbonate cotransporter), member 8	gi|50510613|dbj|BAD32292.1|	0	mKIAA0739 protein [Mus musculus]
mmu-miR-1191b-3p	NM_139063	-21.9	0.998154	-23.76	164	GO:0031082,GO:0005488,GO:0042472,GO:0006810,	-	gi|148708994|gb|EDL40940.1|	4.40E-115	mCG144029 [Mus musculus]
mmu-miR-1191b-3p	NR_045471	-25.5	0.814443	-21.14	163	-	-	gi|148679699|gb|EDL11646.1|	3.20E-87	mCG144621 [Mus musculus]
mmu-miR-1191b-3p	NM_183319	-25.8	0.835689	-22.1	162	GO:0031224,	K08008|1|2e-94|348|mgp:100538688|NADPH oxidase	gi|59858577|ref|NP_899142.2|	0	XK-related protein 2 [Mus musculus]
mmu-miR-1191b-3p	NR_040524	-26.8	0.805236	-22.43	161	-	-	gi|148676412|gb|EDL08359.1|	1.29E-86	mCG145106 [Mus musculus]

mmu-miR-1191b-3p	NM_173441	-28.2	0.878541	-23.77	160	GO:0003676,GO:0010467,	K15177|1|7e-13|79.0|mdo:100031351|RNA polymerase-associated protein LEO1	gi|148664636|gb|EDK97052.1|	0	IWS1 homolog (S. cerevisiae), isoform CRA_b [Mus musculus]
mmu-miR-1191b-3p	NR_045793	-23.1	0.9995	-21.87	159	GO:0031981,GO:0003676,GO:0009790,	K14787|1|6e-24|114|mmu:74111|multiple RNA-binding domain-containing protein 1	gi|148687831|gb|EDL19778.1|	2.63E-108	mCG145980 [Mus musculus]
mmu-miR-1191b-5p	NM_001001798	-29.5	0.621875	-28.23	167	GO:0031224,GO:0042625,GO:0046872,GO:0005548,GO:0032559,GO:0009206,GO:0006811,GO:0006869,GO:0009207,	K01530|1|0.0|2070|mmu:320940|phospholipid-translocating ATPase [EC:3.6.3.1]	gi|49355804|ref|NP_001001798.1|	0	probable phospholipid-transporting ATPase 11C isoform b [Mus musculus]

mmu-miR-1191b-5p	NM_001004143	-32.5	0.193733	-29.56	163	GO:0070461,GO:0016790,GO:0046914,GO:0004402,GO:0003713,GO:0019783,GO:0019941,GO:0016567,GO:0016570,GO:0006351,GO:0007275,GO:0070646,GO:00073	K11366|1|0.0|1113|mmu:216825|ubiquitin carboxyl-terminal hydrolase 22/27/51 [EC:3.1.2.15]	gi|60360058|dbj|BAD90248.1|	0	mKIAA1063 protein [Mus musculus]
mmu-miR-1191b-5p	NM_001005510	-31.2	0.812768	-27.83	173	GO:0030175,GO:0042175,GO:0031965,GO:0031981,GO:0031674,GO:0005924,GO:0034992,GO:0030027,GO:0005783,GO:0008092,GO:0016477,GO:0007097,GO:00070	K06115|1|8e-50|202|xtr:100486199|spectrin beta!K10388|5|2e-48|198|dre:567639|plectin	gi|145699091|ref|NP_001005510.2|	0	nesprin-2 [Mus musculus]

mmu-miR-1191b-5p	NM_001011780	-26.6	0.600499	-26.1	151	-	K04257|1|1e-173|610|mmu:634104|olfactory	gi|74210629|dbj|BAE23666.1|	0	unnamed protein product [Mus musculus]
mmu-miR-1191b-5p	NM_001013026	-28.7	0.640368	-26.18	149	GO:0043231,GO:0043234,GO:0030529,GO:0003676,GO:0046914,GO:0042623,GO:0032559,GO:0006396,GO:0009207,GO:00063	K15173|1|0.0|2063|mmu:74044|transcription termination factor 2 [EC:3.6.4.-]	gi|254692909|ref|NP_001013044.2|	0	transcription termination factor 2 [Mus musculus]
mmu-miR-1191b-5p	NM_001013365	-30.3	0.179706	-27.56	151	GO:0005576,GO:0005887,GO:0005126,GO:0001934,GO:0046883,GO:0007260,GO:0006357,GO:0012501,GO:0008283,GO:0007610,GO:0010740,GO:0006950,GO:0007399,GO:00	K05418|1|1e-97|358|mmu:18413|oncostatin M	gi|148708522|gb|EDL40469.1|	7.61E-97	oncostatin M, isoform CRA_b [Mus musculus]

mmu-miR-1191b-5p	NM_001024848	-29.3	0.307697	-25.74	171	-	K14480|1|5e-134|479|mmu:278679|apolipoprotein	gi|256355165|ref|NP_001020019.2|	3.58E-133	apolipoprotein L 7b [Mus musculus]
mmu-miR-1191b-5p	NM_001025561	-30.5	0.162172	-25.2	152	GO:0043234,GO:0031981,GO:0003676,GO:0016866,GO:0003713,GO:0016836,GO:0006357,GO:0006399,GO:0001522,	K06173|1|0.0|919|mmu:56361|tRNA pseudouridine38-40 synthase [EC:5.4.99.12]	gi|148688057|gb|EDL20004.1|	0	pseudouridine synthase 1, isoform CRA_b [Mus musculus]
mmu-miR-1191b-5p	NM_001029872	-29.8	0.433918	-29.39	162	GO:0005887,GO:0004871,GO:0046983,GO:0031589,GO:0007166,GO:0042098,	K06594|1|0.0|2360|mmu:381924|integrin alpha D	gi|294997269|ref|NP_001025043.3|	0	integrin alpha-D precursor [Mus musculus]
mmu-miR-1191b-5p	NM_001033274	-29.5	0.567383	-26.48	155	GO:0070775,GO:0046914,GO:0016573,	K11349|1|0.0|2110|mmu:223770|bromodomain-containing protein 1	gi|164698417|ref|NP_001028446.2|	0	bromodomain containing 1 [Mus musculus]

mmu-miR-1191b-5p	NM_001037863	-29.5	0.628183	-28.23	167	GO:0031224,GO:0042625,GO:0046872,GO:0005548,GO:0032559,GO:0009206,GO:0006811,GO:0006869,GO:0009207,	K01530|1|0.0|2042|mmu:320940|phospholipid-translocating ATPase [EC:3.6.3.1]	gi|83745137|ref|NP_001032952.1|	0	probable phospholipid-transporting ATPase 11C isoform a [Mus musculus]

mmu-miR-1191b-5p	NM_001038839	-29.2	0.362938	-25.85	152	GO:0009986,GO:0033267,GO:0031229,GO:0030054,GO:0016021,GO:0045202,GO:0044297,GO:0005231,GO:0032559,GO:0016502,GO:0005515,GO:0046914,GO:0030295,GO:0006461,GO:0050707,GO:0006906,GO:0001894,GO:0006915,GO:0030278,GO:0035587,GO:0009628,GO:00022	K05220|1|0.0|910|mmu:18439|purinergic receptor P2X, ligand-gated ion channel 7	gi|84781760|ref|NP_001033934.1|	0	P2X purinoceptor 7 isoform b [Mus musculus]

mmu-miR-1191b-5p	NM_001038845	-29.2	0.232778	-25.85	152	GO:0009986,GO:0033267,GO:0031229,GO:0030054,GO:0016021,GO:0045202,GO:0044297,GO:0005231,GO:0032559,GO:0016502,GO:0005515,GO:0046914,GO:0030295,GO:0006461,GO:0050707,GO:0006906,GO:0001894,GO:0006915,GO:0030278,GO:0035587,GO:0009628,GO:00022	K05220|1|0.0|908|mmu:18439|purinergic receptor P2X, ligand-gated ion channel 7	gi|84781760|ref|NP_001033934.1|	0	P2X purinoceptor 7 isoform b [Mus musculus]

mmu-miR-1191b-5p	NM_001038887	-29.2	0.102135	-25.85	152	GO:0009986,GO:0033267,GO:0031229,GO:0030054,GO:0016021,GO:0045202,GO:0044297,GO:0005231,GO:0032559,GO:0016502,GO:0005515,GO:0046914,GO:0030295,GO:0006461,GO:0050707,GO:0006906,GO:0001894,GO:0006915,GO:0030278,GO:0035587,GO:0009628,GO:00022	K05220|1|8e-66|250|mmu:18439|purinergic receptor P2X, ligand-gated ion channel 7	gi|84781764|ref|NP_001033976.1|	2.39E-78	P2X purinoceptor 7 isoform d [Mus musculus]

mmu-miR-1191b-5p	NM_001039388	-26.9	0.882488	-26.99	161	-	K14863|1|3e-18|95.9|mmu:57750|ribosome biogenesis protein YTM1!K01062|2|4e-18|95.5|cin:100180225|1-alkyl-2-acetylglycerophosphocholine esterase [EC:3.1.1.47]	gi|37360172|dbj|BAC98064.1|	0	mKIAA0982 protein [Mus musculus]
mmu-miR-1191b-5p	NM_001042652	-30.2	0.284858	-26.05	175	GO:0031981,GO:0005819,GO:0003676,GO:0015631,GO:0000070,GO:0033205,GO:00070	K11565|1|2e-22|108|mmu:70645|opa-interacting protein 5	gi|111118996|ref|NP_001036117.1|	0	nucleolar and spindle-associated protein 1 isoform b [Mus musculus]

mmu-miR-1191b-5p	NM_001077595	-32.1	0.332286	-29.34	162	GO:0070161,GO:0015630,GO:0005911,GO:0008092,GO:0065008,GO:0014020,GO:0033059,GO:0008105,GO:0007010,GO:0032989,GO:0002064,	-	gi|117606395|ref|NP_056571.2|	0	protein Shroom3 isoform 1 [Mus musculus]
mmu-miR-1191b-5p	NM_001077596	-32.1	0.334307	-29.34	162	GO:0070161,GO:0015630,GO:0005911,GO:0008092,GO:0065008,GO:0014020,GO:0033059,GO:0008105,GO:0007010,GO:0032989,GO:0002064,	-	gi|117606395|ref|NP_056571.2|	0	protein Shroom3 isoform 1 [Mus musculus]

mmu-miR-1191b-5p	NM_001081363	-30	0.764403	-27.42	161	GO:0034399,GO:0000777,GO:0005819,GO:0012505,GO:0044444,GO:0042802,GO:0006810,GO:0007275,GO:0051303,GO:0010564,GO:0051319,GO:0048856,GO:0007067,GO:0007346,GO:0000086,	K11499|1|0.0|5131|mmu:108000|centromere protein F	gi|189458891|ref|NP_001074832.2|	0	centromere protein F [Mus musculus]
mmu-miR-1191b-5p	NM_001083967	-27.2	0.940397	-44.36	312	-	K15603|1|0.0|1151|mmu:21413|transcription factor 4/12	gi|148677648|gb|EDL09595.1|	0	transcription factor 4, isoform CRA_c [Mus musculus]

mmu-miR-1191b-5p	NM_001102455	-27.6	0.722889	-27.62	162	GO:0031224,GO:0043231,GO:0003676,GO:0004866,GO:0005515,GO:0007618,GO:0043062,GO:0050905,GO:0007420,GO:0030003,GO:0007166,GO:0010469,GO:00076	K08117|1|0.0|1219|mmu:11804|amyloid beta (A4) precursor-like protein 2	gi|156255192|ref|NP_001095925.1|	0	amyloid-like protein 2 isoform a precursor [Mus musculus]
mmu-miR-1191b-5p	NM_001102456	-27.6	0.709679	-27.62	162	GO:0031224,GO:0043231,GO:0003676,GO:0004866,GO:0005515,GO:0007618,GO:0043062,GO:0050905,GO:0007420,GO:0030003,GO:0007166,GO:0010469,GO:00076	K08117|1|0.0|1004|aml:100470064|amyloid beta (A4) precursor-like protein 2	gi|148693392|gb|EDL25339.1|	0	amyloid beta (A4) precursor-like protein 2, isoform CRA_a [Mus musculus]

mmu-miR-1191b-5p	NM_001103181	-35.2	0.149818	-30.94	178	GO:0044424,GO:0005488,GO:0001071,GO:0006351,GO:0031667,GO:0007165,	K09448|1|2e-11|74.3|cfa:481758|TEA domain family member 1/3/4	gi|16905077|ref|NP_473381.1|		0	tubby-related protein 4 isoform a [Mus musculus]
mmu-miR-1191b-5p	NM_001110017	-31.8	0.316156	-29.85	166	GO:0044424,GO:0046914,GO:0003676,GO:0043130,GO:0019787,GO:0016567,	K10642|1|0.0|2317|mmu:224170|E3 ubiquitin-protein ligase DZIP3 [EC:6.3.2.19]	gi|158517947|ref|NP_001103487.1|		0	E3 ubiquitin-protein ligase DZIP3 isoform 1 [Mus musculus]
mmu-miR-1191b-5p	NM_001110222	-29.2	0.803	-25.64	174	GO:0015630,GO:0043005,GO:0044444,GO:0015631,GO:0016477,GO:0009628,GO:0006935,GO:0048588,GO:0007165,GO:00219	K08805|1|2e-144|516|ssc:100623824|doublecortin and CaM kinase-like 1 [EC:2.7.11.1]	gi|397502923|ref|XP_003822087.1|		0	PREDICTED: neuronal migration protein doublecortin isoform 3 [Pan paniscus]

mmu-miR-1191b-5p	NM_001110223	-29.2	0.800484	-25.64	174	GO:0015630,GO:0043005,GO:0044444,GO:0015631,GO:0016477,GO:0009628,GO:0006935,GO:0048588,GO:0007165,GO:00219	K08805|1|2e-144|516|ssc:100623824|doublecortin and CaM kinase-like 1 [EC:2.7.11.1]	gi|397502923|ref|XP_003822087.1|	0	PREDICTED: neuronal migration protein doublecortin isoform 3 [Pan paniscus]
mmu-miR-1191b-5p	NM_001110224	-29.2	0.800386	-25.64	174	GO:0015630,GO:0043005,GO:0044444,GO:0015631,GO:0016477,GO:0009628,GO:0006935,GO:0048588,GO:0007165,GO:00219	K08805|1|5e-144|514|ssc:100623824|doublecortin and CaM kinase-like 1 [EC:2.7.11.1]	gi|397502923|ref|XP_003822087.1|	0	PREDICTED: neuronal migration protein doublecortin isoform 3 [Pan paniscus]
mmu-miR-1191b-5p	NM_001134802	-29.3	0.307697	-25.74	171	-	K14480|1|1e-135|484|mmu:666348|apolipoprotein	gi|198278477|ref|NP_001128274.1|	8.51E-135	apolipoprotein L 7e [Mus musculus]
mmu-miR-1191b-5p	NM_001145198	-30.6	0.091545	-27.3	171	-	-	gi|223633950|ref|NP_001138670.1|	4.13E-28	overexpressed in colon carcinoma 1 protein homolog [Mus musculus]

mmu-miR-1191b-5p	NM_001159662	-30.1	0.5609	-26.39	165	GO:0043231,GO:0016020,GO:0019902,GO:0060491,GO:0023052,	K12329|1|6e-43|178|acs:100566099|protein phosphatase 1 regulatory subunit 12B	gi|23346511|ref|NP_694729.1|	0	protein phosphatase 1 regulatory inhibitor subunit 16B [Mus musculus]
mmu-miR-1191b-5p	NM_001163695	-28.2	0.478671	-26.82	157	-	-	gi|19920334|ref|NP_083181.1|	5.74E-124	uncharacterized protein C21orf62 homolog precursor [Mus musculus]
mmu-miR-1191b-5p	NM_001167939	-27.7	0.832712	-25.57	154	GO:0044427,GO:0043234,GO:0031981,GO:0005515,GO:0007064,	K11266|1|0.0|1234|mmu:74549|MAternally affected uncoordination	gi|269308215|ref|NP_001161411.1|	0	MAU2 chromatid cohesion factor homolog isoform 1 [Mus musculus]
mmu-miR-1191b-5p	NM_001168371	-28.3	0.712425	-27.89	155	GO:0043231,GO:0031224,GO:0044444,GO:0046914,GO:0016790,GO:0070011,GO:0006511,GO:0007049,	K11847|1|0.0|2582|mmu:71472|ubiquitin carboxyl-terminal hydrolase 19 [EC:3.1.2.15]!K06243|2|0.0|2430|ssc:100187735|laminin, beta 2	gi|49022846|dbj|BAC65678.4|	0	mKIAA0891 protein [Mus musculus]

mmu-miR-1191b-5p	NM_001168372	-28.3	0.712201	-27.89	155	GO:0043231,GO:0031224,GO:0044444,GO:0046914,GO:0016790,GO:0070011,GO:0006511,GO:0007049,	K11847|1|0.0|2575|mmu:71472|ubiquitin carboxyl-terminal hydrolase 19 [EC:3.1.2.15]!K06243|2|0.0|2424|ssc:100187735|laminin, beta 2	gi|49022846|dbj|BAC65678.4|	0	mKIAA0891 protein [Mus musculus]
mmu-miR-1191b-5p	NM_001168373	-28.3	0.711978	-27.89	155	GO:0043231,GO:0031224,GO:0044444,GO:0046914,GO:0016790,GO:0070011,GO:0006511,GO:0007049,	K11847|1|0.0|2573|mmu:71472|ubiquitin carboxyl-terminal hydrolase 19 [EC:3.1.2.15]!K06243|2|0.0|2421|ssc:100187735|laminin, beta 2	gi|49022846|dbj|BAC65678.4|	0	mKIAA0891 protein [Mus musculus]

mmu-miR-1191b-5p	NM_001168624	-26.7	0.959821	-25.17	158	-	K08534|1|7e-13|78.6|ecb:100060134|RAR-related orphan receptor gamma	gi|58037552|ref|NP_941016.2|	0	C2 calcium-dependent domain-containing protein 4C [Mus musculus]
mmu-miR-1191b-5p	NM_001170960	-29.8	0.326916	-28.18	172	-	-	gi|283837781|ref|NP_001164431.1|	4.52E-91	DTW domain-containing protein 2 isoform 2 [Mus musculus]
mmu-miR-1191b-5p	NM_001174107	-26.5	0.994174	-44.72	316	GO:0004712,GO:0042802,GO:0004709,GO:0032559,GO:0000187,	K04417|1|0.0|1793|mmu:338372|mitogen-activated protein kinase kinase kinase 9 [EC:2.7.11.25]	gi|291575174|ref|NP_001167578.1|	0	mitogen-activated protein kinase kinase kinase 9 isoform 1 [Mus musculus]
mmu-miR-1191b-5p	NM_001177798	-29.3	0.325938	-25.21	163	GO:0070461,GO:0003712,GO:0003676,GO:0004468,GO:0005506,GO:0015669,GO:0006355,GO:0016573,	K03131|1|0.0|1174|mmu:225895|transcription initiation factor TFIID subunit 6	gi|148701423|gb|EDL33370.1|	0	TAF6-like RNA polymerase II, p300/CBP-associated factor (PCAF)-associated factor, isoform CRA_b [Mus musculus]

mmu-miR-1191b-5p	NM_001198587	-31.7	0.4352	-27.58	168	GO:0031224,GO:0043169,GO:0001505,GO:0007399,	K07377|1|0.0|3005|rno:116508|neurexin	gi|355390244|ref|NP_001185516.2|	0	neurexin III isoform 1 precursor [Mus musculus]
mmu-miR-1191b-5p	NM_001198933	-31.4	0.215502	-27.58	171	GO:0043231,GO:0044444,GO:0046914,GO:0044470,GO:0032559,GO:0006629,GO:0043648,GO:0051259,GO:0006739,GO:0009719,GO:0010033,	K00029|1|0.0|1048|mmu:17436|malate dehydrogenase (oxaloacetate-decarboxylating)(NADP+) [EC:1.1.1.40]	gi|312147392|ref|NP_001185862.1|	0	NADP-dependent malic enzyme isoform 2 [Mus musculus]

mmu-miR-1191b-5p	NM_001204152	-27.5	0.557653	-44.41	332	-	K01227|1|1e-33|145|acs:100563201|mannosyl-glycoprotein endo-beta-N-acetylglucosaminidase [EC:3.2.1.96]!K07296|2|1e-13|76.3|bta:282865|adiponectin	gi|148540036|ref|NP_082607.3|	3.49E-59	complement C1q tumor necrosis factor-related protein 6 isoform 1 precursor [Mus musculus]
mmu-miR-1191b-5p	NM_001204153	-27.5	0.547171	-44.41	332	-	K01227|1|1e-33|145|acs:100563201|mannosyl-glycoprotein endo-beta-N-acetylglucosaminidase [EC:3.2.1.96]!K07296|2|3e-12|74.7|bta:282865|adiponectin	gi|148540036|ref|NP_082607.3|	2.20E-58	complement C1q tumor necrosis factor-related protein 6 isoform 1 precursor [Mus musculus]

mmu-miR-1191b-5p	NM_001252074	-31.7	0.296938	-27.58	168	GO:0031224,GO:0043169,GO:0001505,GO:0007399,	K07377|1|0.0|862|rno:116508|neurexin	gi|148687013|gb|EDL18960.1|	0	mCG6347, isoform CRA_a [Mus musculus]
mmu-miR-1191b-5p	NM_001252382	-31.3	0.146646	-28.41	163	GO:0043231,GO:0003676,GO:0001071,GO:0019221,GO:0006351,	K09446|1|0.0|933|mmu:27056|interferon regulatory factor 5	gi|356991180|ref|NP_001239311.1|	0	interferon regulatory factor 5 isoform 1 [Mus musculus]
mmu-miR-1191b-5p	NM_001254760	-29.5	0.066167	-25.61	170	-	-	gi|344255205|gb|EGW11309.1|	4.24E-07	hypothetical protein I79_014698 [Cricetulus griseus]

mmu-miR-1191b-5p	NM_007646	-26.9	0.753846	-42.78	317	GO:0043231,GO:0031224,GO:0016799,GO:0016829,GO:0033189,GO:0048167,GO:0050851,GO:0043627,GO:0050796,GO:0019229,GO:0030888,GO:0051480,GO:0034097,GO:0006917,GO:0032845,GO:0016049,GO:0022414,GO:0006979,	K01242|1|3e-160|566|mmu:12494|NAD+ nucleosidase [EC:3.2.2.5]	gi|31542362|ref|NP_031672.2|	2.39E-159	ADP-ribosyl cyclase 1 [Mus musculus]
mmu-miR-1191b-5p	NM_007730	-28.5	0.928201	-26.17	167	GO:0005593,GO:0005201,GO:0048731,GO:0030198,	K08132|1|0.0|5479|mmu:12816|collagen, type XII, alpha	gi|111074529|ref|NP_031756.2|	0	collagen alpha-1(XII) chain precursor [Mus musculus]
mmu-miR-1191b-5p	NM_008494	-29.5	0.295505	-26.38	161	GO:0031228,GO:0008375,GO:0022602,GO:0007126,GO:0009653,GO:0009952,	K05948|1|0.0|677|mmu:16848|fringe [EC:2.4.1.222]	gi|6678680|ref|NP_032520.1|	0	beta-1,3-N-acetylglucosaminyltransferase lunatic fringe precursor [Mus musculus]

mmu-miR-1191b-5p	NM_008615		-31.4	0.21032	-27.58	171	GO:0043231,GO:0044444,GO:0046914,GO:0004470,GO:0032559,GO:0006629,GO:0043648,GO:0051259,GO:0006739,GO:0009719,GO:0010033,	K00029|1|0.0|1064|rno:24552|malate dehydrogenase (oxaloacetate-decarboxylating)(NADP+) [EC:1.1.1.40]	gi|162139827|ref|NP_032641.2|	0	NADP-dependent malic enzyme isoform 1 [Mus musculus]
mmu-miR-1191b-5p	NM_008925		-28.9	0.324418	-26.56	159	GO:0044432,GO:0043234,GO:0016020,GO:0016772,GO:0003676,GO:0046872,GO:0019901,GO:0006952,GO:0035556,GO:0051259,GO:0006796,GO:0001822,GO:00064	K08288|1|0.0|817|mmu:19089|protein kinase C substrate 80K-H	gi|6679465|ref|NP_032951.1|	0	glucosidase 2 subunit beta precursor [Mus musculus]

mmu-miR-1191b-5p	NM_009230	-31.7	0.275905	-28.72	173	GO:0031224,GO:0043231,GO:0031090,GO:0032934,GO:0004772,GO:0005504,GO:0055092,GO:0090077,GO:0034434,GO:0030301,GO:0016125,GO:0019915,GO:0034377,GO:00	K00637|1|0.0|1045|mmu:20652|sterol O-acyltransferase [EC:2.3.1.26]	gi|84619697|ref|NP_033256.2|	0	sterol O-acyltransferase 1 [Mus musculus]
mmu-miR-1191b-5p	NM_009331	-31.1	0.157084	-27.68	169	GO:0043231,GO:0001071,GO:0043565,GO:0002681,GO:0006915,GO:0048562,GO:0007399,GO:0070670,GO:0006351,GO:0008283,GO:0007166,GO:0035112,	K02620|1|8e-143|508|rno:363595|transcription factor 7	gi|148701673|gb|EDL33620.1|	1.72E-143	transcription factor 7, T-cell specific, isoform CRA_a [Mus musculus]

mmu-miR-1191b-5p	NM_009675	-26.4	0.91769	-44.24	316	GO:0031224,GO:0044421,GO:0046914,GO:0042802,GO:0016641,GO:0048037,GO:0009987,GO:0002526,GO:0006807,GO:0002684,GO:0010827,GO:0008015,GO:0032502,GO:00	K00276|1|0.0|1481|mmu:11754|primary-amine oxidase [EC:1.4.3.21]	gi|6753066|ref|NP_033805.1|	0	membrane primary amine oxidase [Mus musculus]
mmu-miR-1191b-5p	NM_009691	-27.6	0.705953	-27.62	162	GO:0031224,GO:0043231,GO:0003676,GO:0004866,GO:0005515,GO:0007618,GO:0043062,GO:0050905,GO:0007420,GO:0030003,GO:0007166,GO:0010469,GO:00076	K08117|1|0.0|968|aml:100470064|amyloid beta (A4) precursor-like protein 2	gi|148693392|gb|EDL25339.1|	0	amyloid beta (A4) precursor-like protein 2, isoform CRA_a [Mus musculus]

mmu-miR-1191b-5p	NM_009920	-30.2	0.11175	-26.84	158	GO:0008328,GO:0031090,GO:0043231,GO:0051234,GO:0051641,GO:0007165,GO:0006873,GO:0007270,	-	gi|6753470|ref|NP_034050.1|	1.70E-77	protein cornichon homolog 2 [Mus musculus]
mmu-miR-1191b-5p	NM_010025	-29.2	0.799897	-25.64	174	GO:0015630,GO:0043005,GO:0044444,GO:0015631,GO:0016477,GO:0009628,GO:0006935,GO:0048588,GO:0007165,GO:00219	K08805|1|5e-142|508|ecb:100062396|doublecortin and CaM kinase-like 1 [EC:2.7.11.1]	gi|344286316|ref|XP_003414905.1|	0	PREDICTED: neuronal migration protein doublecortin-like [Loxodonta africana]
mmu-miR-1191b-5p	NM_010331	-31.4	0.124886	-45.1	315	GO:0030176,GO:0008092,GO:0035091,GO:0016787,GO:0035437,GO:0006497,GO:00650	K05289|1|0.0|1026|mmu:14731|glycosylphosphatidylinositol transamidase	gi|6754046|ref|NP_034461.1|	0	glycosylphosphatidylinositol anchor attachment 1 protein [Mus musculus]

mmu-miR-1191b-5p	NM_010775	-30.3	0.072682	-28.37	164	GO:0044421,GO:0048029,GO:0046872,GO:0042802,GO:0001871,GO:0019899,GO:0006956,GO:0045807,GO:0042742,GO:0044126,GO:0051852,	K03991|1|4e-91|335|mmu:17194|mannose-binding lectin	gi|6754654|ref|NP_034905.1|	2.51E-90	mannose-binding protein A precursor [Mus musculus]

mmu-miR-1191b-5p	NM_011027	-29.2	0.595972	-25.85	152	GO:0033267,GO:0031229,GO:0045202,GO:0030054,GO:0009986,GO:0044297,GO:0016021,GO:0042802,GO:0030295,GO:0046914,GO:0005231,GO:0016502,GO:0032559,GO:0033619,GO:0007600,GO:0001894,GO:0030163,GO:0009887,GO:0051181,GO:0042742,GO:0009628,GO:00019	K05220|1|0.0|1008|rno:29665|purinergic receptor P2X, ligand-gated ion channel 7	gi|84781766|ref|NP_035157.2|	0	P2X purinoceptor 7 isoform a [Mus musculus]

mmu-miR-1191b-5p	NM_011403	-28.9	0.613122	-25.63	165	GO:0005856,GO:0016021,GO:0044459,GO:0031674,GO:0008092,GO:0015103,GO:0042802,GO:0015301,GO:0015698,GO:00508	K06573|1|0.0|1541|mmu:20533|solute carrier family 4 (anion exchanger), member 1	gi|6755560|ref|NP_035533.1|	0	band 3 anion transport protein [Mus musculus]
mmu-miR-1191b-5p	NM_011740	-26.3	0.896582	-46.34	310	GO:0032991,GO:0044430,GO:0048770,GO:0005515,GO:0042221,GO:0007599,GO:0006605,GO:0016070,GO:0002441,GO:0001775,GO:0043066,	K06630|1|1e-129|465|aml:100484483|tyrosine 3-monooxygenase/tryptophan 5-monooxygenase activation protein	gi|301756356|ref|XP_002914041.1|	9.76E-129	PREDICTED: 14-3-3 protein zeta/delta-like [Ailuropoda melanoleuca]
mmu-miR-1191b-5p	NM_011763	-28.1	0.674242	-26.27	169	GO:0043231,GO:0046914,GO:0003676,GO:0006351,	K09228|1|0.0|981|mmu:22750|KRAB domain-containing zinc finger protein	gi|27261814|ref|NP_035893.1|	0	zinc finger protein 9 [Mus musculus]

mmu-miR-1191b-5p	NM_013474	-27.6	0.114	-25.27	168	GO:0034364,GO:0034385,GO:0044432,GO:0004857,GO:0070325,GO:0005543,GO:0032934,GO:0008047,GO:0042802,GO:0071813,GO:0046983,GO:0015248,GO:0006954,GO:0051704,GO:0042108,GO:0010608,GO:0046470,GO:0032787,GO:0015914,GO:0055092,GO:0016125,GO:00108	K08758|1|8e-51|199|mmu:11807|apolipoprotein A-II	gi|157951676|ref|NP_038502.2|	4.92E-50	apolipoprotein A-II precursor [Mus musculus]

mmu-miR-1191b-5p	NM_013622	-26.1	0.925401	-48.62	322	GO:0016021,GO:0044424,GO:0032589,GO:0033267,GO:0043226,GO:0004985,GO:0070848,GO:0007626,GO:0051931,GO:0002376,GO:0007189,GO:0032413,GO:00066	K04213|1|3e-162|573|mmu:18386|delta-type opioid receptor	gi|124244064|ref|NP_038650.3|	2.08E-161	delta-type opioid receptor [Mus musculus]
mmu-miR-1191b-5p	NM_013776	-28.5	0.187023	-46.45	306	-	-	gi|7305561|ref|NP_038804.1|	1.18E-49	protein TCL1B5 [Mus musculus]
mmu-miR-1191b-5p	NM_015756	-32.1	0.347675	-29.34	162	GO:0070161,GO:0015630,GO:0005911,GO:0008092,GO:0065008,GO:0014020,GO:0033059,GO:0008105,GO:0007010,GO:0032989,GO:0002064,	K05868|1|1e-14|84.7|mgp:100543246|cyclin B	gi|117606395|ref|NP_056571.2|	0	protein Shroom3 isoform 1 [Mus musculus]

mmu-miR-1191b-5p	NM_015794	-31.3	0.729797	-28.48	167	-	K10283|1|0.0|1050|mmu:50758|F-box and leucine-rich repeat protein	gi|255683359|ref|NP_056609.1|	0	F-box/LRR-repeat protein 17 [Mus musculus]
mmu-miR-1191b-5p	NM_016863	-27	0.325363	-25.11	156	GO:0005789,GO:0034703,GO:0005515,GO:0000166,GO:0005527,GO:0016859,GO:0005218,GO:0002027,GO:0032512,GO:0006457,GO:0018193,GO:0007417,GO:0010880,GO:0033189,GO:0032412,GO:0006936,GO:0000302,GO:0042110,GO:0009790,GO:0030323,GO:0030072,GO:00487	K09568|1|7e-62|238|ptr:741549|FK506-binding protein 1 [EC:5.2.1.8]	gi|123173728|ref|NP_058559.3|	1.22E-56	peptidyl-prolyl cis-trans isomerase FKBP1B [Mus musculus]

mmu-miR-1191b-5p	NM_016891	-38	0.008926	-34.96	180	GO:0043231,GO:0044427,GO:0008287,GO:0005856,GO:0044444,GO:0004721,GO:0046983,GO:0019888,GO:0042221,GO:0007155,GO:0016055,GO:0042503,GO:0006396,GO:0065003,GO:0022402,GO:0006915,GO:0012502,GO:0046519,GO:0016049,GO:0006470,GO:0006260,GO:00001	K03456|1|0.0|1149|ecb:100062627|protein phosphatase 2 (formerly 2A), regulatory subunit A	gi|338709914|ref|XP_001496310.2|	0	PREDICTED: serine/threonine-protein phosphatase 2A 65 kDa regulatory subunit A alpha isoform-like isoform 1 [Equus caballus]

mmu-miR-1191b-5p	NM_016977	-28.6	0.468849	-25.07	171	GO:0031224,GO:0004977,GO:0019899,GO:0032846,GO:0015980,GO:0007610,GO:0043434,GO:0006171,GO:0030072,GO:0002021,GO:0007187,	K04202|1|1e-178|627|mmu:17202|melanocortin 4 receptor	gi|85861190|ref|NP_058673.2|	8.01E-178	melanocortin receptor 4 [Mus musculus]
mmu-miR-1191b-5p	NM_017391	-32.9	0.381317	-29.13	176	GO:0031224,GO:0015291,GO:0005365,GO:0019751,GO:0015791,GO:0007399,GO:0035725,GO:0051239,	K14383|1|0.0|1367|mmu:53881|solute carrier family 5 (sodium/myo-inositol cotransporter), member 3	gi|148536859|ref|NP_059087.2|	0	sodium/myo-inositol cotransporter [Mus musculus]
mmu-miR-1191b-5p	NM_017480	-30.2	0.323158	-30.2	165	GO:0009986,GO:0016021,GO:0005488,GO:0007155,GO:0031294,	K06713|1|3e-107|390|mmu:54167|inducible T-cell co-	gi|224809335|ref|NP_059508.2|	1.99E-106	inducible T-cell costimulator precursor [Mus musculus]

mmu-miR-1191b-5p	NM_018878	-32.5	0.22131	-29.22	168	GO:0034399,GO:0034708,GO:0005488,GO:0002699,GO:0009888,GO:0042770,GO:0031062,GO:0006259,GO:0060260,GO:0016567,GO:0030154,GO:0009314,GO:0000414,GO:0016477,GO:0016573,	K14972|1|0.0|937|mmu:55982|PAX-interacting protein 1	gi|42734451|ref|NP_061366.2|	0	PAX-interacting protein 1 [Mus musculus]
mmu-miR-1191b-5p	NM_019913	-29.8	0.13839	-26.09	156	GO:0031981,GO:0016671,GO:0005515,GO:0015036,GO:0009746,GO:0009719,GO:0031667,GO:0009611,GO:0006091,GO:0019725,GO:0018904,GO:0051234,	K03671|1|2e-91|336|mmu:56551|thioredoxin 1	gi|148697704|gb|EDL29651.1|	1.51E-100	thioredoxin 2, isoform CRA_a [Mus musculus]

mmu-miR-1191b-5p	NM_020035	-26	0.942281	-67.77	463	GO:0031090,GO:0043231,GO:0031224,GO:0005488,GO:0016772,GO:0006506,	K05288|1|0.0|1740|mmu:56703|phosphatidylinositol glycan, class O	gi|60360026|dbj|BAD90232.1|	0	mFLJ00350 protein [Mus musculus]
mmu-miR-1191b-5p	NM_020608	-27.6	0.920743	-48.35	322	-	-	gi|169234772|ref|NP_065633.2|	0	protein cramped-like [Mus musculus]
mmu-miR-1191b-5p	NM_021459	-32.7	0.095612	-28.19	180	GO:0043231,GO:0046914,GO:0001071,GO:0003677,GO:0007411,GO:0021536,GO:0045596,GO:0031016,GO:0055007,GO:0006357,GO:0001667,GO:0001708,GO:0050796,GO:0009887,GO:0021522,	K09370|1|0.0|725|ssc:100511417|insulin gene enhancer protein ISL	gi|94400881|ref|NP_059035.3|	0	insulin gene enhancer protein ISL-1 [Rattus norvegicus]

mmu-miR-1191b-5p	NM_021464	-32.8	0.251112	-30.46	170	GO:0031224,GO:0004871,GO:0004721,GO:0050839,GO:0042805,GO:0016337,GO:0007167,GO:0006470,	K13297|1|0.0|2795|mmu:19281|protein tyrosine phosphatase, receptor type, T [EC:3.1.3.48]	gi|148674357|gb|EDL06304.1|	0	protein tyrosine phosphatase, receptor type, T [Mus musculus]
mmu-miR-1191b-5p	NM_021509	-27.7	0.644529	-26.19	167	GO:0031090,GO:0043231,GO:0031224,GO:0046914,GO:0016715,GO:0006576,	K15266|1|2e-104|381|bfo:BRAFLDRAFT_125270|dimethyladenosine transferase 1, mitochondrial!K00503|3|3e-83|311|xtr:100492247|dopamine beta-monooxygenase [EC:1.14.17.1]	gi|31981183|ref|NP_067484.2|	0	DBH-like monooxygenase protein 1 precursor [Mus musculus]

mmu-miR-1191b-5p	NM_021704	-34.3	0.033406	-30.8	178	GO:0009986,GO:0016020,GO:0044421,GO:0060089,GO:0005125,GO:0014046,GO:0001935,GO:0009628,GO:0007411,GO:0003006,GO:0032414,GO:0021537,GO:0007626,GO:0007276,GO:0002548,GO:0007166,GO:0001525,GO:0003013,GO:0006950,GO:0042110,GO:0030832,GO:00068	K10031|1|4e-38|160|mmu:20315|C-X-C motif chemokine 12	gi|148667157|gb|EDK99573.1|	9.55E-52	chemokine (C-X-C motif) ligand 12, isoform CRA_c [Mus musculus]
mmu-miR-1191b-5p	NM_023047	-31.8	0.281767	-27.54	165	-	K07529|1|0.0|1143|mmu:65254|dihydropyrimidinase-like 5	gi|148705344|gb|EDL37291.1|	0	dihydropyrimidinase-like 5, isoform CRA_c [Mus musculus]

mmu-miR-1191b-5p	NM_023217	-29.9	0.4924	-25.98	156	GO:0044444,GO:0070011,GO:0019538,	K01304|1|8e-120|433|mmu:66522|pyroglutamyl-peptidase [EC:3.4.19.3]	gi|12963583|ref|NP_075706.1|	5.38E-119	pyroglutamyl-peptidase 1 [Mus musculus]
mmu-miR-1191b-5p	NM_023526	-26.1	0.94679	-25.68	160	GO:0044424,GO:0017111,GO:0032561,GO:0007243,GO:0009207,	K07829|1|7e-12|74.7|mdo:100030293|Ras-related protein R-Ras!K07849|2|7e-12|74.7|ecb:100058975|Ras homolog enriched in brain like 1!K07827|3|9e-12|74.3|nve:NEMVE_v1g247341|GTPase KRas	gi|31541888|ref|NP_076015.2|	9.60E-94	NF-kappa-B inhibitor-interacting Ras-like protein 1 [Mus musculus]

mmu-miR-1191b-5p	NM_023537	-27.6	0.687609	-25.19	167	GO:0030136,GO:0016020,GO:0017111,GO:0032561,GO:0035556,GO:0006887,GO:0006479,GO:0009207,	K06108|1|8e-123|442|mmu:69908|Ras-related protein Rab-3B	gi|148698775|gb|EDL30722.1|	2.01E-122	RAB3B, member RAS oncogene family, isoform CRA_b [Mus musculus]
mmu-miR-1191b-5p	NM_025413	-29.4	0.108395	-26.2	170	-	-	gi|344255205|gb|EGW11309.1|	8.21E-09	hypothetical protein I79_014698 [Cricetulus griseus]
mmu-miR-1191b-5p	NM_025903	-27.8	0.458738	-25.51	159	-	-	gi|13385396|ref|NP_080179.1|	1.52E-180	interferon-related developmental regulator 2 [Mus musculus]
mmu-miR-1191b-5p	NM_025954	-29.4	0.12623	-26.24	161	GO:0016791,GO:0044238,GO:0006796,	K01091|1|4e-175|614|mmu:67078|phosphoglycolate phosphatase [EC:3.1.3.1	gi|40254507|ref|NP_080230.2|	2.58E-174	phosphoglycolate phosphatase [Mus musculus]
mmu-miR-1191b-5p	NM_026005	-27.5	0.532032	-25.95	164	-	-	gi|165905593|ref|NP_080281.3|	3.91E-105	protein C8orf37 homolog [Mus musculus]
mmu-miR-1191b-5p	NM_026325	-29.3	0.101476	-25.21	163	GO:0044464,	-	gi|257467568|ref|NP_080601.2|	4.69E-85	transmembrane protein 179B precursor [Mus musculus]

mmu-miR-1191b-5p	NM_026394	-29.4	0.084833	-26.2	170	-	-	gi|12844209|dbj|BAB26276.1|	1.75E-08	unnamed protein product [Mus musculus]
mmu-miR-1191b-5p	NM_026554	-29	0.316667	-26.86	161	GO:0031981,GO:0034518,GO:0044444,GO:0016758,GO:0000339,GO:0006413,GO:0016458,GO:0006497,GO:0000956,GO:0032241,GO:0006353,GO:0000398,GO:0006354,GO:0045935,GO:0006405,	K12883|1|2e-76|288|rno:689116|nuclear cap-binding protein subunit 2	gi|13386056|ref|NP_080830.1|	1.06E-75	nuclear cap-binding protein subunit 2 [Mus musculus]
mmu-miR-1191b-5p	NM_026579	-32.3	0.164087	-26.09	171	-	-	gi|44662811|ref|NP_080855.1|	1.46E-78	uncharacterized protein C12orf45 homolog [Mus musculus]
mmu-miR-1191b-5p	NM_026751	-25.3	0.881949	-43.93	324	GO:0031224,	-	gi|223278372|ref|NP_081027.1|	3.04E-167	myeloid-associated differentiation marker-like protein 2 isoform 1 [Mus musculus]
mmu-miR-1191b-5p	NM_026822	-29.4	0.066652	-26.2	170	-	-	gi|351710180|gb|EHB13099.1|	4.40E-06	Polymerase delta-interacting protein 2 [Heterocephalus glaber]

mmu-miR-1191b-5p	NM_026854	-29.8	0.356551	-28.18	172	-	-	gi|110625694|ref|NP_081130.1|	1.54E-148	DTW domain-containing protein 2 isoform 1 [Mus musculus]
mmu-miR-1191b-5p	NM_026921	-28.8	0.317848	-25.11	169	GO:0043231,GO:0051540,GO:0043169,GO:0003008,GO:0031163,	K13628|1|1e-68|261|cfa:607596|iron-sulfur cluster assembly protein	gi|332260003|ref|XP_003279075.1|	2.74E-70	PREDICTED: iron-sulfur cluster assembly 1 homolog, mitochondrial-like [Nomascus leucogenys]
mmu-miR-1191b-5p	NM_027081	-30.3	0.427185	-27.55	160	-	-	gi|49355814|ref|NP_081357.2|	0	protein FAM116B [Mus musculus]
mmu-miR-1191b-5p	NM_027137	-29.4	0.071266	-26.2	170	-	-	gi|344255205|gb|EGW11309.1|	7.95E-09	hypothetical protein I79_014698 [Cricetulus griseus]
mmu-miR-1191b-5p	NM_027341	-31.8	0.285636	-29.85	166	GO:0044424,GO:0046914,GO:0003676,GO:0043130,GO:0019787,GO:0016567,	K10642|1|0.0|1479|bta:537349|E3 ubiquitin-protein ligase DZIP3 [EC:6.3.2.19]	gi|124249054|ref|NP_081617.1|	0	E3 ubiquitin-protein ligase DZIP3 isoform 2 [Mus musculus]
mmu-miR-1191b-5p	NM_008065	-23.8	0.999644	-84.12	586	GO:0031981,GO:0003712,GO:0001071,GO:0043565,GO:0046983,GO:0043009,	K09441|1|0.0|874|mmu:14390|GA-binding protein transcription factor, alpha	gi|34328119|ref|NP_032091.2|	0	GA-binding protein alpha chain [Mus musculus]

mmu-miR-1192	NM_001013371	-25.1	0.911791	-40.85	376	GO:0043231,GO:0046914,GO:0005515,GO:0019787,GO:0006513,GO:0006325,GO:00335	K06058|1|0.0|1474|mmu:209200|deltex	gi|133930784|ref|NP_001013389.2|	0	E3 ubiquitin-protein ligase DTX3L [Mus musculus]
mmu-miR-1192	NM_001013616	-28.1	0.366225	-44.23	371	GO:0044424,GO:0046914,GO:0051259,	K11999|1|0.0|947|mmu:94088|tripartite motif-containing protein 6/22/34	gi|61966933|ref|NP_001013637.1|	0	tripartite motif-containing protein 6 [Mus musculus]
mmu-miR-1192	NM_001024705	-25.5	0.350938	-41.63	366	-	K13911|1|2e-13|77.8|mcc:695131|basic salivary proline-rich protein 1/2	gi|344258094|gb|EGW14198.1|	1.45E-34	hypothetical protein I79_023565 [Cricetulus griseus]
mmu-miR-1192	NM_001025309	-28.9	0.330168	-25.77	188	GO:0012505,GO:0044459,GO:0031090,GO:0043231,GO:0044430,GO:0046914,GO:0003824,	K10634|1|0.0|1348|mmu:224938|E3 ubiquitin-protein ligase Praja2 [EC:6.3.2.19]	gi|28972221|dbj|BAC65564.1|	0	mKIAA0438 protein [Mus musculus]

mmu-miR-1192	NM_001037925	-28.6	0.121881	-25.26	187	-	-	gi|85702324|ref|NP_001033014.1|	2.47E-96	2-cell-stage, variable group, member 3-like [Mus musculus]
mmu-miR-1192	NM_001162537	-29.8	0.280406	-26.8	191	GO:0005488,	-	gi|242117990|ref|NP_001156009.1|	0	uncharacterized protein LOC212448 [Mus musculus]
mmu-miR-1192	NM_001163734	-25.5	0.828738	-41.63	371	GO:0044444,GO:0030684,GO:0031981,GO:0005488,GO:0042254,	K14849|1|0.0|1087|mmu:72462|ribosomal RNA-processing protein 1	gi|37359820|dbj|BAC97888.1|	0	mKIAA0179 protein [Mus musculus]
mmu-miR-1192	NM_001205331	-31.1	0.098596	-48.5	379	GO:0015630,GO:0005488,GO:0007019,	K10431|1|4e-49|197|mmu:17758|microtubule-associated protein 4	gi|148677083|gb|EDL09030.1|	1.37E-52	microtubule-associated protein 4, isoform CRA_c [Mus musculus]

mmu-miR-1192	NM_001252684	-25.7	0.873121	-42.95	366	GO:0043232,GO:0043231,GO:0032559,GO:0006259,GO:0033554,	K06674|1|7e-14|81.6|phu:Phum_PHUM129190|structural maintenance of chromosome 2!K06669|4|6e-13|78.6|cin:100185546|structural maintenance of chromosome 3 (chondroitin sulfate proteoglycan 6)	gi|358248335|ref|NP_001239613.1|	0	structural maintenance of chromosomes protein 5 isoform 1 [Mus musculus]

mmu-miR-1192	NM_001252685	-25.7	0.863392	-42.95	366	GO:0043232,GO:0043231,GO:0032559,GO:0006259,GO:0033554,	K06674|1|1e-14|84.0|cfa:481637|structural maintenance of chromosome 2!K10352|4|2e-13|80.1|tsp:Tsp_08431|myosin heavy chain	gi|148709653|gb|EDL41599.1|	0	mCG5312, isoform CRA_b [Mus musculus]
mmu-miR-1192	NM_001271843	-26.1	0.533396	-44.27	371	-	-	-	-	-
mmu-miR-1192	NM_007548	-29.8	0.256332	-26.8	191	GO:0043231,GO:0046914,GO:0001071,GO:0003677,GO:0019899,GO:0000122,GO:0002832,GO:0003006,GO:0001890,GO:0030888,GO:0045577,GO:0001701,	K09228|1|1e-40|170|bfo:BRAFLDRAFT_71028|KRAB domain-containing zinc finger protein	gi|125859109|gb|AAI29802.1|	0	Prdm1 protein [Mus musculus]

mmu-miR-1192	NM_007983	-26.9	0.605025	-45.54	366	GO:0043231,GO:0012505,GO:0031264,GO:0044444,GO:0019207,GO:0008134,GO:0019900,GO:0007155,GO:0042994,GO:0006461,GO:0009894,GO:0006511,GO:00069	K04649|1|3e-13|79.3|cel:C28G1.1|ubiquitin-conjugating enzyme (huntingtin interacting protein 2) [EC:6.3.2.19]	gi|40789280|ref|NP_032009.2|	0	FAS-associated factor 1 [Mus musculus]
mmu-miR-1192	NM_008123	-29.4	0.390923	-25.19	188	GO:0005921,GO:0031224,GO:0005488,GO:0022829,GO:0043010,GO:0051259,GO:00071	K07617|1|0.0|775|mmu:14616|gap junction protein, alpha 8	gi|6680009|ref|NP_032149.1|	0	gap junction alpha-8 protein [Mus musculus]

mmu-miR-1192	NM_008962	-31.8	0.063899	-28.97	196	GO:0031224,GO:0005488,GO:0004955,GO:0046128,GO:0006874,GO:0071379,GO:0051240,GO:0007275,GO:0007610,GO:0007166,GO:0042311,	K04332|1|2e-175|617|mmu:19214|prostaglandin D2 receptor	gi|225543126|ref|NP_032988.3|	1.27E-174	prostaglandin D2 receptor [Mus musculus]
mmu-miR-1192	NM_020621	-26.4	0.686342	-42.04	367	GO:0016021,GO:0044459,GO:0015065,GO:0032559,GO:0006821,GO:0035588,GO:0051480,	K04271|1|0.0|720|mmu:57385|pyrimidinergic receptor P2Y, G protein-coupled, 4	gi|10181172|ref|NP_065646.1|	0	P2Y purinoceptor 4 [Mus musculus]
mmu-miR-1192	NM_024182	-25.5	0.761228	-43.3	371	GO:0004672,GO:0032559,GO:0006464,	K08872|1|0.0|985|mmu:66878|RIO kinase 3 [EC:2.7.11.1]	gi|110625737|ref|NP_077144.2|	0	serine/threonine-protein kinase RIO3 [Mus musculus]

mmu-miR-1192	NM_026361	-30.5	0.174724	-27.03	196	GO:0005911,GO:0005488,GO:0009987,	K05690|1|2e-122|442|gga:428852|catenin (cadherin-associated protein),	gi|51491821|ref|NP_080637.1|	0	plakophilin-4 isoform 1 [Mus musculus]
mmu-miR-1192	NM_027594	-25.5	0.90634	-42.94	366	GO:0005815,GO:0043005,GO:0043025,GO:0016881,GO:0048731,GO:0043412,GO:0048869,	K00273|1|1e-91|339|ame:551507|D-amino-acid oxidase [EC:1.4.3.3]!K06047|2|7e-19|98.2|xtr:100135000|tubulin---tyrosine ligase	gi|226442787|ref|NP_081870.1|	0	tubulin polyglutamylase TTLL7 [Mus musculus]
mmu-miR-1192	NM_028244	-25.5	0.848627	-41.63	371	GO:0044444,GO:0030684,GO:0031981,GO:0005488,GO:0042254,	K14849|1|0.0|1087|mmu:72462|ribosomal RNA-processing protein 1	gi|254911009|ref|NP_082520.2|	0	ribosomal RNA processing protein 1 homolog B isoform 1 [Mus musculus]

mmu-miR-1192	NM_029952	-29.5	0.209339	-25.77	188	GO:0044464,GO:0046914,GO:0006351,	K09228|1|3e-157|556|rno:100360287|KRAB domain-containing zinc finger	gi|40254149|ref|NP_084228.2|	0	zinc finger protein 883A [Mus musculus]
mmu-miR-1192	NM_053079	-25.5	0.702302	-41.63	371	GO:0016021,GO:0015295,GO:0015322,GO:0016247,GO:0015833,GO:0032501,GO:0032891,	K14206|1|0.0|1367|mmu:56643|solute carrier family 15 (oligopeptide transporter), member 1	gi|119392092|ref|NP_444309.2|	0	solute carrier family 15 member 1 [Mus musculus]
mmu-miR-1192	NM_134010	-30.5	0.109816	-26.8	191	GO:0005643,GO:0000776,GO:0044444,GO:0005488,GO:0005215,GO:0044238,GO:0015758,GO:0006405,GO:0007067,	K14301|1|0.0|1794|mmu:103468|nuclear pore complex protein Nup107	gi|29789351|ref|NP_598771.1|	0	nuclear pore complex protein Nup107 [Mus musculus]

mmu-miR-1192	NM_144551	-30.5	0.156504	-27.03	196	GO:0031981,GO:0043232,GO:0016301,GO:0055103,GO:0032559,GO:0019887,GO:0019899,GO:0045596,GO:0033673,GO:0031398,GO:0043392,GO:0009966,GO:0031331,GO:00	K08814|1|0.0|700|mmu:217410|tribbles [EC:2.7.11.-]	gi|31542100|ref|NP_653134.2|	0	tribbles homolog 2 [Mus musculus]
mmu-miR-1192	NM_144859	-28.9	0.318113	-25.77	188	GO:0044459,GO:0031090,GO:0043231,GO:0044430,GO:0046914,GO:0003824,	K10634|1|0.0|1139|mmu:224938|E3 ubiquitin-protein ligase Praja2 [EC:6.3.2.19]	gi|21450079|ref|NP_659108.1|	0	E3 ubiquitin-protein ligase Praja-2 isoform b [Mus musculus]
mmu-miR-1192	NM_153523	-28.6	0.050153	-25.26	187	-	-	gi|23943805|ref|NP_705743.1|	2.16E-96	2-cell-stage, variable group, member 3 [Mus musculus]

mmu-miR-1192	NM_153808	-25.7	0.871265	-42.95	366	GO:0043232,GO:0043231,GO:0032559,GO:0006259,GO:0033554,	K06674|1|1e-14|84.3|cfa:481637|structural maintenance of chromosome 2!K10352|4|2e-13|80.1|tsp:Tsp_08431|myosin heavy chain!K06669|5|6e-13|78.6|cin:100185546|structural maintenance of chromosome 3 (chondroitin sulfate proteoglycan 6)	gi|148709653|gb|EDL41599.1|	0	mCG5312, isoform CRA_b [Mus musculus]
mmu-miR-1192	NM_172405	-25.5	0.613463	-64.92	554	GO:0043231,GO:0043234,GO:0043130,GO:0009314,GO:0006282,GO:0000077,GO:0006325,	K11862|1|4e-47|190|ptr:450809|ubiquitin thioesterase ZRANB1 [EC:3.1.2.-]	gi|148688324|gb|EDL20271.1|	0	coiled-coil domain containing 98, isoform CRA_e [Mus musculus]

mmu-miR-1192	NM_172827	-25.7	0.844036	-40.32	376	GO:0016021,GO:0044297,GO:0030141,GO:0046914,GO:0008528,GO:0008238,GO:0009719,GO:0003014,GO:0043171,GO:0048168,GO:0019538,GO:0009057,GO:0022414,GO:00	K01257|1|0.0|1961|mmu:240028|cystinyl aminopeptidase [EC:3.4.11.3]	gi|27370240|ref|NP_766415.1|	0	leucyl-cystinyl aminopeptidase [Mus musculus]
mmu-miR-1192	NM_175464	-30.5	0.169467	-27.03	196	GO:0044459,GO:0009987,	K05690|1|2e-122|442|gga:428852|catenin (cadherin-associated protein),	gi|147899467|ref|NP_780673.2|	0	plakophilin-4 isoform 2 [Mus musculus]
mmu-miR-1192	NR_015524	-31.4	0.105052	-28.7	196	-	-	gi|148689640|gb|EDL21587.1|	3.87E-78	mCG11209 [Mus musculus]
mmu-miR-1192	NR_027958	-24.9	0.830915	-64.14	550	-	-	gi|123255305|emb|CAM26932.1|	1.55E-125	novel protein [Mus musculus]

mmu-miR-1192	NR_027323	-23.3	0.979835	-49.48	379	GO:0031224,	K13023|1|2e-31|139|pon:100174713|carboxypeptidase N regulatory subunit	gi|149036465|gb|EDL91083.1|	0	rCG56516 [Rattus norvegicus]
mmu-miR-1192	NM_029955	-24.3	0.991006	-46.69	375	-	K10352|1|2e-07|60.8|bta:530050|myosin heavy chain	gi|148707840|gb|EDL39787.1|	0	coiled-coil domain containing 93, isoform CRA_b [Mus musculus]
mmu-miR-1192	NR_045745	-24.3	0.923955	-41.63	371	-	-	gi|148694212|gb|EDL26159.1|	2.86E-104	mCG144749 [Mus musculus]

mmu-miR-1192	NM_010700	-23.9	0.974666	-44.91	370	GO:0016021,GO:0000323,GO:0009986,GO:0034358,GO:0030665,GO:0044459,GO:0010008,GO:0030228,GO:0030169,GO:0046872,GO:0016125,GO:0034381,GO:0043627,GO:0042157,GO:0023052,GO:0043434,GO:0030301,GO:0006897,GO:0051704,GO:0032502,GO	K12473|1|0.0|1764|mmu:16835|low-density lipoprotein receptor	gi|113195700|ref|NP_034830.2|	0	low-density lipoprotein receptor isoform 1 precursor [Mus musculus]
mmu-miR-1192	NR_015508	-23.3	0.982774	-40.32	368	-	-	gi|67975423|ref|XP_668908.1|	3.69E-19	hypothetical protein [Plasmodium berghei strain ANKA]
mmu-miR-1192	NM_174851	-23.3	0.9863	-44.42	367	GO:0005887,GO:0005488,GO:0004871,GO:0050794,GO:0050688,GO:0023052,	K05140|1|0.0|895|mmu:242700|interleukin 28 receptor alpha	gi|225543249|ref|NP_777276.3|	0	interleukin-28 receptor subunit alpha precursor [Mus musculus]

mmu-miR-1192	NR_026853	-24.3	0.795162	-42.21	366	-	-	gi|148678048|gb|EDL09995.1|	5.98E-86	mCG145926 [Mus musculus]
mmu-miR-1192	NM_011382	-24.3	0.975524	-40.37	356	GO:0043231,GO:0001071,GO:0003677,GO:0048704,GO:0042471,GO:0006357,GO:0022008,GO:0048534,GO:0014706,GO:0043066,GO:0014812,	K15615|1|0.0|1239|mmu:20474|homeobox protein SIX4	gi|33859632|ref|NP_035512.1|	0	homeobox protein SIX4 [Mus musculus]
mmu-miR-1192	NM_027450	-24.7	0.667243	-41.94	355	GO:0012505,GO:0043231,	-	gi|47059151|ref|NP_081726.1|	1.11E-85	Golgi-associated plant pathogenesis-related protein 1 [Mus musculus]
mmu-miR-1192	NR_045381	-28.2	0.139827	-24.76	188	-	-	gi|148664647|gb|EDK97063.1|	7.40E-42	mCG145773 [Mus musculus]
mmu-miR-1192	NM_178906	-27.3	0.629483	-23.32	187	-	-	gi|26343487|dbj|BAC35400.1|	4.87E-63	unnamed protein product [Mus musculus]
mmu-miR-1192	NM_172666	-25.8	0.917352	-22.34	186	GO:0044439,GO:0016020,GO:0016765,GO:0050662,GO:0050896,GO:0045017,	K00803|1|0.0|1278|mmu:228061|alkyldihydroxyacetonephosphate synthase [EC:2.5.1.26]	gi|295444834|ref|NP_766254.2|	0	alkyldihydroxyacetonephosphate synthase, peroxisomal [Mus musculus]

mmu-miR-1192	NM_029466	-20.4	1	-24.02	184	GO:0044464,GO:0032561,GO:0035556,	K07950|1|2e-100|368|mmu:75869|ADP-ribosylation factor-like 5B	gi|27229225|ref|NP_083742.2|	1.17E-99	ADP-ribosylation factor-like protein 5B [Mus musculus]
mmu-miR-1192	NR_045878	-25.5	0.329997	-21.47	183	-	-	gi|148665300|gb|EDK97716.1|	3.01E-35	mCG126624, isoform CRA_a [Mus musculus]
mmu-miR-1192	NR_038051	-26.1	0.481537	-23.09	182	-	-	gi|148677656|gb|EDL09603.1|	1.00E-77	mCG145150 [Mus musculus]
mmu-miR-1192	NM_023336	-22.3	0.99985	-23.81	181	GO:0005488,	K11721|1|0.0|870|mmu:67382|bromodomain-containing protein 3	gi|148676419|gb|EDL08366.1|	0	bromodomain containing 3, isoform CRA_b [Mus musculus]
mmu-miR-1192	NR_045859	-23.3	0.632009	-20.16	180	-	-	-	-	-
mmu-miR-1192	NR_045292	-22.6	0.927602	-20.5	179	-	-	gi|148700021|gb|EDL31968.1|	1.12E-22	mCG145721, isoform CRA_a [Mus musculus]
mmu-miR-1192	NR_033140	-23.3	0.898512	-20.16	178	-	-	gi|148670335|gb|EDL02282.1|	3.69E-47	mCG1041362 [Mus musculus]
mmu-miR-1192	NM_025461	-24.8	0.644913	-20.6	177	-	-	gi|344235739|gb|EGV91842.1|	1.20E-54	Cytochrome c oxidase assembly protein COX16-like, mitochondrial [Cricetulus griseus]
mmu-miR-1192	NR_045088	-24.2	0.738101	-20.14	176	-	-	gi|148709058|gb|EDL41004.1|	5.81E-60	mCG145082 [Mus musculus]

mmu-miR-1192	NR_028381	-21.6	0.999946	-22.23	175	-	-	gi|148682148|gb|EDL14095.1|	3.42E-45	mCG15773, isoform CRA_a [Mus musculus]
mmu-miR-1192	NM_139141	-25.6	0.958938	-22.49	174	GO:0031981,GO:0046914,GO:0003676,GO:0001071,GO:0006351,	K09229|1|0.0|1192|mmu:93681|KRAB and SCAN domains-containing zinc finger	gi|46852149|ref|NP_631880.2|	0	zinc finger protein 192 isoform 1 [Mus musculus]
mmu-miR-1192	NR_033228	-23.3	0.998474	-20.38	173	GO:0043231,GO:0044444,GO:0016859,GO:0005515,GO:0044409,GO:0006310,GO:0044267,GO:0001775,GO:0006260,GO:0002376,GO:0030154,GO:0050792,GO:00075	K03767|1|3e-82|188|mmu:268373|peptidyl-prolyl cis-trans isomerase A (cyclophilin A) [EC:5.2.1.8]	gi|392353052|ref|XP_002728136.2|	7.57E-87	PREDICTED: peptidyl-prolyl cis-trans isomerase A-like [Rattus norvegicus]
mmu-miR-1192	NM_025438	-22.9	0.948789	-24.68	171	-	-	gi|354472542|ref|XP_003498497.1|	2.18E-77	PREDICTED: arginine/serine-rich coiled-coil protein 2-like [Cricetulus griseus]

mmu-miR-1192	NM_175201	-23.3	0.994686	-21.56	170	GO:0042995,GO:0046914,	K13201|1|1e-45|187|bfo:BRAFLDRAFT_124262|nucleolysin TIA-1/TIAR!K15692|3|1e-10|70.5|xtr:493377|E3 ubiquitin-protein ligase RNF13 [EC:6.3.2.19]	gi|332228409|ref|XP_003263382.1|	0	PREDICTED: RING finger protein 38 isoform 3 [Nomascus leucogenys]
mmu-miR-1192	NR_038023	-22.9	0.979406	-20.15	169	-	-	gi|68062078|ref|XP_673041.1|	1.11E-15	hypothetical protein [Plasmodium berghei strain ANKA]
mmu-miR-1192	NM_001037809	-20.2	1	-20.77	167	GO:0005911,GO:0031224,GO:0044424,GO:0046872,GO:0016337,GO:0045216,GO:00509	K06796|1|0.0|1501|mmu:12560|cadherin 3, type 1, P-cadherin	gi|83715978|ref|NP_001032898.1|	0	cadherin-3 isoform a precursor [Mus musculus]
mmu-miR-1192	NM_145536	-23.2	0.91303	-21.04	166	-	-	gi|32171369|sp|Q9NUG4.3|CT160_HUMAN	0	RecName: Full=Uncharacterized protein C20orf160

mmu-miR-1192	NM_025410	-21.8	0.788294	-20.99	162	GO:0005488,	K15902|1|3e-81|301|mmu:66192|polarized growth chromatin-associated controller 1	gi|13384798|ref|NP_079686.1|	2.21E-80	L antigen family member 3 [Mus musculus]
mmu-miR-1192	NM_001166138	-22.3	0.997937	-20.58	154	-	K06059|1|2e-13|79.7|spu:585715|disintegrin and metalloproteinase domain-containing protein 17 [EC:3.4.24.86]!K15043|2|7e-06|54.3|cin:100182844|importin subunit	gi|260763999|ref|NP_001159610.1|	0	armadillo repeat-containing protein 8 isoform 2 [Mus musculus]
mmu-miR-1192	NM_201358	NA	NA	-20.77	167	-	-	gi|148708964|gb|EDL40910.1|	1.28E-48	cDNA sequence BC034664 [Mus musculus]

mmu-miR-1192	NM_028150	NA	NA	-20.87	166	GO:0070461,GO:0003712,GO:0004468,GO:0072595,GO:0016573,GO:0010467,	K11316|1|0.0|717|mmu:72195|STAGA complex 65 subunit gamma	gi|58037285|ref|NP_082426.1|	0	STAGA complex 65 subunit gamma [Mus musculus]
mmu-miR-1192	NM_146825	NA	NA	-22.21	156	GO:0031224,GO:0004930,GO:0007166,GO:0007606,	K04257|1|2e-174|612|mmu:258822|olfactory	gi|257467472|ref|NP_667036.2|	1.07E-173	olfactory receptor 39 [Mus musculus]
mmu-miR-1192	NM_175164	NA	NA	-20.42	148	GO:0043232,GO:0005924,GO:0044444,GO:0005099,GO:0030674,GO:0019904,GO:0048731,GO:0035556,GO:0030035,GO:0007010,GO:0009966,	K13736|1|0.0|770|mdo:100018415|Rho GTPase-activating protein 10	gi|37360016|dbj|BAC97986.1|	0	mKIAA0621 protein [Mus musculus]
mmu-miR-1192	NM_001100444	-20	0.999999	NA	NA	-	-	gi|158303290|ref|NP_001093914.1|	2.96E-110	uncharacterized protein LOC546272 [Mus musculus]

mmu-miR-1192	NM_181585	-21.4	0.999995	NA	NA	GO:0043234,GO:0044444,GO:0005488,GO:0035004,GO:0035014,GO:0019222,GO:0007169,GO:0016310,GO:0007599,GO:00312	K02649|1|0.0|912|mmu:18710|phosphoinositide-3-kinase, regulatory subunit	gi|31712016|ref|NP_853616.1|	0	phosphatidylinositol 3-kinase regulatory subunit gamma [Mus musculus]
mmu-miR-1192	NM_001164662	-21.1	1	NA	NA	GO:0044459,GO:0031252,GO:0044444,GO:0034518,GO:0003779,GO:0017048,GO:0048731,GO:0048588,GO:0030031,	K05749|1|0.0|2375|mmu:20430|cytoplasmic FMR1 interacting protein	gi|148689929|gb|EDL21876.1|	0	cytoplasmic FMR1 interacting protein 1, isoform CRA_a [Mus musculus]
mmu-miR-1192	NM_001080388	-20.7	1	NA	NA	-	K08798|1|0.0|1406|mmu:13728|MAP/microtubule affinity-regulating kinase [EC:2.7.11.	gi|26337255|dbj|BAC32312.1|	0	unnamed protein product [Mus musculus]

mmu-miR-1192	NM_177602	-23	0.963788	NA	NA	-	K12816|1|1e-38|162|aga:AgaP_AGAP008925|pre-mRNA-processing factor 17	gi|226958523|ref|NP_808270.2|	0	WD repeat-containing protein 25 [Mus musculus]
mmu-miR-1192	NR_002322	-24.2	0.899743	NA	NA	-	-	gi|397481736|ref|XP_003812095.1|	8.41E-41	PREDICTED: uncharacterized protein LOC100983160 [Pan paniscus]
mmu-miR-1192	NM_025391	-20.5	0.999995	NA	NA	GO:0031981,GO:0003676,GO:0042254,	K07565|1|3e-99|363|rno:192180|60S ribosome subunit biogenesis protein NIP7	gi|257153336|ref|NP_079667.2|	1.27E-99	60S ribosome subunit biogenesis protein NIP7 homolog isoform 1 [Mus musculus]

mmu-miR-1192	NM_001195538	-21	1	NA	NA	GO:0016021,GO:0004672,GO:0004871,GO:0032559,GO:0016477,GO:0016192,GO:0051707,GO:0006796,GO:0007420,GO:0048588,GO:0007165,GO:0021955,GO:00064	K08805|1|0.0|1274|aml:100484701|doublecortin and CaM kinase-like 1 [EC:2.7.11.1]	gi|26006151|dbj|BAC41418.1|	0	mKIAA0369 protein [Mus musculus]
mmu-miR-1192	NM_175318	-20.3	1	NA	NA	-	K15193|1|0.0|868|mmu:101685|protein SPT2	gi|26326843|dbj|BAC27165.1|	0	unnamed protein product [Mus musculus]
mmu-miR-1192	NM_175937	-22.2	0.999985	NA	NA	GO:0044424,GO:0003727,GO:0006412,	K02602|1|2e-54|216|spu:581876|oo18 RNA-binding protein	gi|293651586|ref|NP_787951.2|	0	cytoplasmic polyadenylation element-binding protein 2 isoform 1 [Mus musculus]

mmu-miR-1192	NM_025832	-23.7	0.969486	NA	NA	GO:0043231,GO:0043234,GO:0043021,GO:0006473,GO:0006355,	K00670|1|0.0|1665|mmu:66897|peptide alpha-N-acetyltransferase [EC:2.3.1.88]	gi|21313242|ref|NP_080108.1|	0	N-alpha-acetyltransferase 16, NatA auxiliary subunit [Mus musculus]
mmu-miR-1192	NM_146386	-21.9	0.999944	NA	NA	GO:0043231,GO:0003712,GO:0042802,GO:0019899,GO:0001558,GO:0007507,GO:0008283,GO:0048742,GO:0006939,GO:00165	-	gi|154240726|ref|NP_666498.2|	0	myocardin isoform B [Mus musculus]
mmu-miR-1192	NM_009688	-20.1	1	NA	NA	GO:0043231,GO:0044444,GO:0046914,GO:0019899,GO:0003824,GO:0004869,GO:0033554,GO:0010466,GO:0043066,	K04725|1|0.0|1017|mmu:11798|E3 ubiquitin-protein ligase XIAP	gi|157951674|ref|NP_033818.2|	0	E3 ubiquitin-protein ligase XIAP [Mus musculus]

mmu-miR-1192	NM_001009940	-22	0.873944	NA	NA	GO:0044421,GO:0005102,GO:0002376,GO:0032635,GO:0012502,GO:0044237,GO:00230	K05444|1|3e-97|355|mmu:329244|interleukin 19/20/24	gi|57977321|ref|NP_001009940.1|	2.26E-96	interleukin-19 precursor [Mus musculus]
mmu-miR-1192	NM_001037987	-21.2	0.999999	NA	NA	GO:0046872,GO:0005102,GO:0032501,GO:0010810,	K03902|1|2e-71|272|ecb:100058503|coagulation factor V (labile factor)	gi|83921564|ref|NP_001033076.1|	0	EGF-like repeat and discoidin I-like domain-containing protein 3 isoform a precursor [Mus musculus]
mmu-miR-1192	NM_001100446	-20	0.999998	NA	NA	-	-	gi|154689748|ref|NP_001093916.1|	1.62E-108	uncharacterized protein LOC100043216 [Mus musculus]
mmu-miR-1192	NR_027891	-21.5	0.999235	NA	NA	-	-	gi|148664508|gb|EDK96924.1|	6.17E-32	mCG145686 [Mus musculus]
mmu-miR-1192	NM_133927	-21.3	0.998528	NA	NA	-	-	gi|19527142|ref|NP_598688.1|	0	integrin-alpha FG-GAP repeat-containing protein 2 [Mus musculus]
mmu-miR-1192	NM_001093776	-21	1	NA	NA	GO:0043231,GO:0046914,GO:0003676,GO:0001071,GO:0006351,GO:0032501,GO:0048869,	K11307|1|7e-08|62.0|acs:100564728|histone acetyltransferase MYST2 [EC:2.3.1.4	gi|148271075|ref|NP_032692.2|	0	myelin transcription factor 1-like protein isoform 2 [Mus musculus]

mmu-miR-1192	NM_001103156	-23.1	0.999973	NA	NA	GO:0005768,GO:0031228,GO:0005798,GO:0046914,GO:0050662,GO:0003824,GO:0006892,GO:0009719,GO:0006091,GO:0000041,GO:0006887,	K14738|1|0.0|935|mmu:74051|metalloreductase STEAP2 [EC:1.16.1.-]	gi|124249344|ref|NP_083010.2|	0	metalloreductase STEAP2 [Mus musculus]
mmu-miR-1192	NM_001077407	-20.3	1	NA	NA	GO:0031224,GO:0043005,GO:0005488,GO:0004714,GO:0048869,GO:0001667,GO:0006935,GO:0007169,GO:0048513,	K06819|1|0.0|1773|cfa:488487|neuropilin 2	gi|116686144|ref|NP_001070875.1|	0	neuropilin-2 isoform 6 precursor [Mus musculus]
mmu-miR-1192	NR_003617	-21	0.999974	NA	NA	GO:0044464,GO:0005488,GO:0006952,GO:0009116,	-	gi|12839801|dbj|BAB24674.1|	5.30E-54	unnamed protein product [Mus musculus]

mmu-miR-1192	NM_001162410	-21	0.999981	NA	NA	GO:0031981,GO:0043234,GO:0005887,GO:0044444,GO:0046983,GO:0004674,GO:0032559,GO:0007265,GO:0048017,GO:0007589,GO:0002573,GO:0050673,GO:0042476,GO:0050851,GO:0051091,GO:0002224,GO:0007169,GO:0002009,GO:0006468,GO:0007249,GO:0042692,GO:00022	K04467|1|0.0|1430|mmu:12675|inhibitor of nuclear factor kappa-B kinase subunit alpha [EC:2.7.11.10]	gi|242332490|ref|NP_001155882.1|	0	inhibitor of nuclear factor kappa-B kinase subunit alpha isoform 2 [Mus musculus]
mmu-miR-1192	NM_028672	-23.6	0.836423	NA	NA	-	-	gi|148675930|gb|EDL07877.1|	0	RIKEN cDNA 4930430E16, isoform CRA_a [Mus musculus]

mmu-miR-1192	NM_198022	-20.4	1	NA	NA	GO:0003676,GO:0016458,	K06237|1|2e-08|64.3|dan:Dana_GF14161|collagen, type IV, alpha	gi|124378035|ref|NP_932139.2|	0	trinucleotide repeat-containing gene 6C protein [Mus musculus]
mmu-miR-1192	NM_026047	-22.2	0.997957	NA	NA	GO:0046914,	-	gi|88759344|ref|NP_080323.3|	0	RING finger protein 219 [Mus musculus]
mmu-miR-1192	NM_201518	-20.1	1	NA	NA	GO:0016021,GO:0031012,GO:0004871,GO:0005515,GO:0009987,	K13023|1|7e-29|131|hsa:1370|carboxypeptidase N regulatory subunit!K07523|2|2e-28|130|xtr:100188921|netrin-	gi|41462393|ref|NP_958926.1|	0	fibronectin leucine rich transmembrane protein 2 [Mus musculus]

mmu-miR-1192	NM_175750	-21	1	NA	NA	GO:0031224,GO:0005488,GO:0004888,GO:0021561,GO:0021564,GO:0021785,GO:0021563,GO:0050920,GO:0030516,GO:0007422,GO:0021537,GO:0021559,GO:00071	K06820|1|0.0|3821|mmu:243743|plexin A	gi|171543899|ref|NP_786926.2|		0	plexin-A4 precursor [Mus musculus]
mmu-miR-1192	NM_028758	-21.9	0.999935	NA	NA	GO:0030135,GO:0044431,GO:0031090,GO:0030119,GO:0031267,GO:0015031,	K12404|1|0.0|1041|mmu:74105|ADP-ribosylation factor-binding protein GGA	gi|37360224|dbj|BAC98090.1|		0	mKIAA1080 protein [Mus musculus]
mmu-miR-1192	NM_177612	-21.1	0.999977	NA	NA	GO:0005856,GO:0031252,GO:0005913,GO:0003676,GO:0050839,GO:0006260,GO:0007155,	K05691|1|0.0|1588|mmu:216033|catenin (cadherin-associated protein),	gi|256985119|ref|NP_808280.2|		0	catenin alpha-3 isoform 1 [Mus musculus]

mmu-miR-1192	NM_001142948	-21.7	0.999999	NA	NA	GO:0043231,GO:0003676,GO:0006351,GO:0032501,	K09228|1|8e-14|81.6|ssc:100737868|KRAB domain-containing zinc finger protein	gi|219273495|ref|NP_001136420.1|	0	pogo transposable element with KRAB domain isoform 2 [Mus musculus]
mmu-miR-1192	NM_172824	-21.5	0.999952	NA	NA	-	K04257|1|2e-21|105|mdo:100017656|olfactory receptor	gi|169646270|ref|NP_766412.2|	0	coiled-coil domain-containing protein 14 [Mus musculus]
mmu-miR-1192	NM_019974	-20.4	0.996746	NA	NA	GO:0004175,GO:0019538,	K09620|1|2e-146|519|mmu:56538|kallikrein 11 [EC:3.4.21.-]	gi|293651560|ref|NP_001170844.1|	1.19E-145	kallikrein-11 isoform 1 precursor [Mus musculus]
mmu-miR-1193-3p	NM_001004180	-29.6	0.199159	-26.62	164	-	-	gi|51921355|ref|NP_001004180.1|	0	protein FAM222A [Mus musculus]

mmu-miR-1193-3p	NM_001033215	-29.8	0.186204	-25.28	174	GO:0043231,GO:0001071,GO:0043565,	K09188|1|4e-12|74.7|dwi:Dwil_GK16377|histone-lysine N-methyltransferase MLL3 [EC:2.1.1.43]	gi|85701614|ref|NP_001028387.1|	0	uncharacterized protein LOC105590 [Mus musculus]
mmu-miR-1193-3p	NM_001033713	-28.7	0.302051	-26.96	163	GO:0031981,GO:0000785,GO:0019900,GO:0001071,GO:0003677,GO:0012501,GO:0048731,GO:0002224,GO:0006355,GO:00455	K09260|1|0.0|776|mmu:17258|MADS-box transcription enhancer factor 2A	gi|148675249|gb|EDL07196.1|	0	myocyte enhancer factor 2A, isoform CRA_d [Mus musculus]
mmu-miR-1193-3p	NM_001081061	-28.3	0.78581	-26.02	169	-	K15198|1|0.0|4487|mmu:544971|transcription factor TFIIIB component B''	gi|145587077|ref|NP_001074530.1|	0	transcription factor TFIIIB component B'' homolog [Mus musculus]

mmu-miR-1193-3p	NM_001081191	-30.6	0.463356	-25.3	183	-	K15420|1|2e-99|366|tgu:100219108|echinoderm microtubule-associated protein-like 4	gi|124487111|ref|NP_001074660.1|	0	echinoderm microtubule-associated protein-like 5 [Mus musculus]
mmu-miR-1193-3p	NM_001099779	-27.2	0.473111	-25.66	158	GO:0044444,GO:0031420,GO:0016301,GO:0032559,GO:0006007,GO:0010038,GO:0009746,GO:0006090,GO:0006796,GO:0031667,GO:0009206,GO:0032868,GO:0006950,GO:00	K12406|1|0.0|1055|mmu:18770|pyruvate kinase isozymes R/L [EC:2.7.1.40]	gi|74146312|dbj|BAE28927.1|	0	unnamed protein product [Mus musculus]

mmu-miR-1193-3p	NM_001104531	-29.4	0.1156	-26.99	156	GO:0031090,GO:0043231,GO:0005506,GO:0016712,GO:0010033,GO:0008152,	K07414|1|0.0|907|mmu:13101|cytochrome P450, family 2, subfamily D [EC:1.14.14.1]	gi|157154304|ref|NP_001098001.1|	0	cytochrome P450 2D11 [Mus musculus]
mmu-miR-1193-3p	NM_001109750	-30.6	0.207465	-27.5	170	GO:0031224,GO:0004871,GO:0030246,GO:0009653,GO:0042221,GO:0009987,GO:0023052,	K03068|1|6e-09|64.7|bta:534450|low density lipoprotein receptor-related protein 5/6	gi|157909810|ref|NP_001103220.1|	0	integral membrane protein DGCR2/IDD isoform 2 precursor [Mus musculus]
mmu-miR-1193-3p	NM_001114339	-28.2	0.422957	-48.79	311	GO:0031981,GO:0044444,GO:0016301,GO:0032559,GO:0006575,GO:0009108,GO:0006796,GO:0006766,	K09680|1|0.0|961|mmu:75735|type II pantothenate kinase [EC:2.7.1.33]	gi|167234376|ref|NP_001107811.1|	0	pantothenate kinase 1 isoform 1 [Mus musculus]

mmu-miR-1193-3p	NM_001146005	-26.8	0.635958	-51.26	332	GO:0031224,GO:0004942,GO:0007166,GO:0042221,	K04171|1|9e-179|628|mmu:319430|G protein-coupled receptor 77	gi|225579148|ref|NP_001139477.1|	5.96E-178	C5a anaphylatoxin chemotactic receptor C5L2 isoform 2 [Mus musculus]
mmu-miR-1193-3p	NM_001146023	-31.4	0.034251	-27.49	164	-	K15434|1|7e-29|129|aml:100482760|protein	gi|225637492|ref|NP_001139495.1|	3.45E-145	uncharacterized protein LOC73833 [Mus musculus]
mmu-miR-1193-3p	NM_001159521	-26.6	0.87304	-41.67	315	GO:0031224,GO:0008047,GO:0005488,GO:0004871,GO:0031346,GO:0032501,GO:00099	K06821|1|0.0|3692|mmu:140570|plexin B	gi|226958474|ref|NP_620088.2|	0	plexin-B2 precursor [Mus musculus]
mmu-miR-1193-3p	NM_001159631	-25.4	0.823674	-44.17	328	GO:0043231,GO:0008092,GO:0004672,GO:0019900,GO:0046872,GO:0060089,GO:0032559,GO:0012501,GO:0006468,GO:0007088,GO:0007249,	K08857|1|0.0|645|mmu:59126|NIMA (never in mitosis gene a)-related kinase [EC:2.7.11.1]	gi|354499098|ref|XP_003511648.1|	0	PREDICTED: serine/threonine-protein kinase Nek6-like [Cricetulus griseus]

mmu-miR-1193-3p	NM_001162926	-27.6	0.691684	-26.19	161	GO:0044424,GO:0016020,GO:0005488,	-	gi|242397444|ref|NP_001156398.1|	4.84E-156	uncharacterized protein LOC399603 [Mus musculus]
mmu-miR-1193-3p	NM_001164789	-28	0.47351	-25.29	162	GO:0043231,GO:0003676,GO:0045595,GO:0001837,GO:0001825,GO:0021953,GO:0006351,GO:0002293,GO:0001704,	K10173|1|0.0|1124|mmu:13813|eomesodermin protein	gi|258645096|ref|NP_001158261.1|	0	eomesodermin homolog isoform 2 [Mus musculus]
mmu-miR-1193-3p	NM_001178012	-30	0.180255	-28.04	174	GO:0005740,GO:0031224,GO:0015075,GO:0015142,GO:0000041,	K03351|1|2e-72|275|smm:Smp_136960|anaphase-promoting complex subunit 4	gi|295821210|ref|NP_001171483.1|	9.32E-153	sideroflexin-3 isoform 2 [Mus musculus]
mmu-miR-1193-3p	NM_001200041	-27.9	0.432215	-26.49	161	GO:0000166,	K08858|1|6e-40|167|mgp:100539383|SH3-binding domain kinase 1 [EC:2.7.11.1]	gi|160184875|sp|P0C5K0.1|SG110_MOUSE	4.82E-134	RecName: Full=Putative uncharacterized serine/threonine-protein kinase SgK110; AltName: Full=Sugen kinase 110

mmu-miR-1193-3p	NM_001252453	-28	0.635826	-26.25	163	GO:0016021,GO:0005488,GO:0004871,GO:0004725,GO:0022037,GO:0006470,GO:0021543,GO:0023052,	K06778|1|0.0|2672|mgp:100551029|protein tyrosine phosphatase, receptor type, S [EC:3.1.3.48]!K06777|3|0.0|2253|hsa:5789|protein tyrosine phosphatase, receptor type, D [EC:3.1.3.48]	gi|356995860|ref|NP_001239382.1|	0	receptor-type tyrosine-protein phosphatase S isoform 2 precursor [Mus musculus]

mmu-miR-1193-3p	NM_001252455	-28	0.635034	-26.25	163	GO:0016021,GO:0005488,GO:0004871,GO:0004725,GO:0022037,GO:0006470,GO:0021543,GO:0023052,	K06778|1|0.0|2657|mgp:100551029|protein tyrosine phosphatase, receptor type, S [EC:3.1.3.48]!K06777|3|0.0|2252|rno:313278|protein tyrosine phosphatase, receptor type, D [EC:3.1.3.48]	gi|356995864|ref|NP_001239384.1|		0	receptor-type tyrosine-protein phosphatase S isoform 3 precursor [Mus musculus]

mmu-miR-1193-3p	NM_001252456	-28	0.633312	-26.25	163	GO:0016021,GO:0005488,GO:0004871,GO:0004725,GO:0022037,GO:0006470,GO:0021543,GO:0023052,	K06778|1|0.0|2672|mgp:100551029|protein tyrosine phosphatase, receptor type, S [EC:3.1.3.48]!K06777|3|0.0|2253|hsa:5789|protein tyrosine phosphatase, receptor type, D [EC:3.1.3.48]	gi|356995860|ref|NP_001239382.1|	0	receptor-type tyrosine-protein phosphatase S isoform 2 precursor [Mus musculus]

mmu-miR-1193-3p	NM_001253883	-28.7	0.253247	-27.07	158	GO:0016020,GO:0043231,GO:0042995,GO:0044444,GO:0016301,GO:0031420,GO:0032559,GO:0042246,GO:0014854,GO:0006007,GO:0006090,GO:0006796,GO:0043434,GO:0009628,GO:0009887,GO:0008219,GO:0009206,GO:0031667,	K00873|1|0.0|1026|hsa:5315|pyruvate kinase [EC:2.7.1.40]	gi|359807367|ref|NP_001240812.1|	0	pyruvate kinase isozymes M1/M2 isoform 2 [Mus musculus]
mmu-miR-1193-3p	NM_007387	-28.1	0.548025	-25.07	162	GO:0031224,GO:0005764,GO:0031982,GO:0045309,GO:0016791,GO:0012501,GO:0048731,GO:0007267,GO:0006796,GO:0042221,GO:0007033,	K14410|1|0.0|848|mmu:11432|acid phosphatase [EC:3.1.3.2]	gi|29150253|ref|NP_031413.1|	0	lysosomal acid phosphatase precursor [Mus musculus]

mmu-miR-1193-3p	NM_008062	-29.5	0.200877	-26.92	166	GO:0005815,GO:0043231,GO:0044459,GO:0000166,GO:0016616,GO:0042802,GO:0048029,GO:0048469,GO:0006098,GO:0002034,GO:0010033,GO:0019302,GO:0008203,GO:0001816,GO:0006790,GO:0050789,GO:0035264,GO:0010731,GO:0006979,GO:00	K00036|1|0.0|1029|mmu:14381|glucose-6-phosphate 1-dehydrogenase [EC:1.1.1.49]	gi|6996917|ref|NP_032088.1|	0	glucose-6-phosphate 1-dehydrogenase X [Mus musculus]

mmu-miR-1193-3p	NM_008305	-26.4	0.988793	-25.58	161	GO:0005604,GO:0016020,GO:0005515,GO:0007417,GO:0043062,GO:0001503,GO:0044238,GO:0051216,GO:0048646,GO:0044260,GO:0048562,GO:0030154,GO:0014706,GO:00	K06255|1|0.0|8774|mmu:15530|heparan sulfate proteoglycan 2 (perlecan)	gi|183979966|ref|NP_032331.2|	0	basement membrane-specific heparan sulfate proteoglycan core protein precursor [Mus musculus]
mmu-miR-1193-3p	NM_010048	-30.6	0.20794	-27.5	170	GO:0031224,GO:0004871,GO:0030246,GO:0009653,GO:0042221,GO:0009987,GO:0023052,	K03068|1|6e-09|64.7|bta:534450|low density lipoprotein receptor-related protein 5/6	gi|83816901|ref|NP_034178.2|	0	integral membrane protein DGCR2/IDD isoform 1 precursor [Mus musculus]

mmu-miR-1193-3p	NM_010136	-28	0.4792	-25.29	162	GO:0043231,GO:0003676,GO:0045595,GO:0001837,GO:0001825,GO:0021953,GO:0006351,GO:0002293,GO:0001704,	K10173|1|0.0|1113|mmu:13813|eomesodermin protein	gi|148677303|gb|EDL09250.1|	0	eomesodermin homolog (Xenopus laevis), isoform CRA_a [Mus musculus]
mmu-miR-1193-3p	NM_010378	-29.5	0.066351	-27.57	163	GO:0031224,GO:0000323,GO:0042611,GO:0009986,GO:0003823,GO:0002478,GO:00455	K06752|1|4e-137|488|mmu:14960|major histocompatibility complex, class II	gi|31981716|ref|NP_034508.2|	2.96E-136	histocompatibility 2, class II antigen A, alpha precursor [Mus musculus]

mmu-miR-1193-3p	NM_010568	-25.4	0.991149	-45.27	315	GO:0031090,GO:0043231,GO:0005887,GO:0044444,GO:0004714,GO:0005102,GO:0017046,GO:0032561,GO:0032559,GO:0019902,GO:0019904,GO:0019901,GO:0005520,GO:0043169,GO:0031406,GO:0031016,GO:0032409,GO:0032868,GO:2000026,GO:0010828,GO:0007169,GO:00321	K04527|1|0.0|2726|mmu:16337|insulin receptor [EC:2.7.10.1]	gi|157057178|ref|NP_034698.2|		0	insulin receptor precursor [Mus musculus]

mmu-miR-1193-3p	NM_010740	-34.7	0.069869	-32.3	166	GO:0031224,GO:0031410,GO:0046872,GO:0004871,GO:0030246,GO:0001846,GO:0002274,GO:0007155,GO:0023052,GO:00068	K06702|1|0.0|1217|mmu:17064|CDW93 antigen	gi|6754578|ref|NP_034870.1|	0	complement component C1q receptor precursor [Mus musculus]
mmu-miR-1193-3p	NM_010755	-31.2	0.060352	-25.2	150	GO:0031981,GO:0003677,GO:0050896,GO:0045595,GO:0043009,GO:0007599,GO:0006351,GO:0022414,	K09037|1|2e-53|211|ptr:743669|transcription factor MAFF/G/K	gi|332859773|ref|XP_003317281.1|	4.52E-51	PREDICTED: transcription factor MafF isoform 4 [Pan troglodytes]

mmu-miR-1193-3p	NM_011099	-28.7	0.253247	-27.07	158	GO:0043231,GO:0042995,GO:0044444,GO:0016020,GO:0031420,GO:0016301,GO:0032559,GO:0006007,GO:0008219,GO:0006090,GO:0006796,GO:0009206,GO:0009887,GO:00	K00873|1|0.0|1048|xla:100037030|pyruvate kinase [EC:2.7.1.40]	gi|31981562|ref|NP_035229.2|		0	pyruvate kinase isozymes M1/M2 isoform 1 [Mus musculus]
mmu-miR-1193-3p	NM_011218	-28	0.706794	-26.25	163	GO:0016021,GO:0005488,GO:0004871,GO:0004725,GO:0022037,GO:0006470,GO:0021543,GO:0023052,	K06778|1|0.0|3687|mmu:19280|protein tyrosine phosphatase, receptor type, S [EC:3.1.3.48]	gi|158711690|ref|NP_035348.2|		0	receptor-type tyrosine-protein phosphatase S isoform 1 precursor [Mus musculus]

mmu-miR-1193-3p	NM_011599	-30.1	0.267807	-25.72	166	GO:0043231,GO:0043234,GO:0003712,GO:0005515,GO:0016055,GO:0006357,GO:0032501,GO:0009653,	K04497|1|0.0|1011|dre:30206|groucho	gi|148699116|gb|EDL31063.1|	0	mCG2299, isoform CRA_b [Mus musculus]
mmu-miR-1193-3p	NM_011706	-30.3	0.151658	-27.95	165	GO:0048770,GO:0016021,GO:0005488,GO:0005261,GO:0050877,GO:0070838,GO:0009628,GO:0006950,	K04971|1|0.0|1397|mmu:22368|transient receptor potential cation channel subfamily V member 2	gi|148678407|gb|EDL10354.1|	0	transient receptor potential cation channel, subfamily V, member 2, isoform CRA_a [Mus musculus]

mmu-miR-1193-3p	NM_013631	-27.2	0.490767	-25.66	158	GO:0044444,GO:0016301,GO:0031420,GO:0032559,GO:0015980,GO:0031016,GO:0032868,GO:0009893,GO:0006007,GO:0006090,GO:0006796,GO:0010038,GO:0009607,GO:0009746,GO:0006950,GO:0009206,GO:00	K12406|1|0.0|1042|mmu:18770|pyruvate kinase isozymes R/L [EC:2.7.1.40]	gi|153792131|ref|NP_038659.2|	0	pyruvate kinase isozymes R/L isoform 1 [Mus musculus]
mmu-miR-1193-3p	NM_013883	-29.8	0.232965	-25.48	166	GO:0043231,GO:0044427,GO:0003676,GO:0001071,GO:0016568,GO:0003002,GO:0006355,GO:0048232,	K11461|1|0.0|1224|mmu:29871|polycomb protein SCMH1	gi|148698480|gb|EDL30427.1|	0	sex comb on midleg homolog 1, isoform CRA_b [Mus musculus]

mmu-miR-1193-3p	NM_019468	-29.5	0.114514	-26.92	166	GO:0005815,GO:0043231,GO:0044459,GO:0000166,GO:0016616,GO:0042802,GO:0048029,GO:0048469,GO:0006098,GO:0002034,GO:0010033,GO:0019302,GO:0008203,GO:0001816,GO:0006790,GO:0050789,GO:0035264,GO:0010731,GO:0006979,GO:00	K00036|1|0.0|1026|mmu:14380|glucose-6-phosphate 1-dehydrogenase [EC:1.1.1.49]	gi|227330582|ref|NP_062341.2|	0	glucose-6-phosphate 1-dehydrogenase 2 [Mus musculus]
mmu-miR-1193-3p	NM_021427	-27.9	0.26153	-46.42	308	-	-	gi|120587013|ref|NP_067402.2|	0	protein FAM181B [Mus musculus]

mmu-miR-1193-3p	NM_021606	-25.4	0.822986	-44.17	328	GO:0043231,GO:0008092,GO:0004672,GO:0019900,GO:0046872,GO:0060089,GO:0032559,GO:0012501,GO:0006468,GO:0007088,GO:0007249,	K08857|1|0.0|645|mmu:59126|NIMA (never in mitosis gene a)-related kinase [EC:2.7.11.1]	gi|354499098|ref|XP_003511648.1|	0	PREDICTED: serine/threonine-protein kinase Nek6-like [Cricetulus griseus]
mmu-miR-1193-3p	NM_025686	-26.7	0.310928	-25.68	158	GO:0031981,GO:0046914,GO:0006351,	K15197|1|0.0|779|mmu:66653|transcription factor IIIB 50 kDa subunit	gi|28076877|ref|NP_079962.1|	0	transcription factor IIIB 50 kDa subunit [Mus musculus]
mmu-miR-1193-3p	NM_027279	-27.1	0.283388	-46.69	320	GO:0008168,GO:0008213,	-	gi|31980620|ref|NP_081555.2|	0	histidine protein methyltransferase 1 homolog [Mus musculus]
mmu-miR-1193-3p	NM_027513	-29.3	0.476282	-26.33	175	GO:0005635,GO:0005488,GO:0044238,GO:0050658,GO:0006606,GO:0015758,	K14310|1|0.0|3811|mmu:70699|nuclear pore complex protein Nup205	gi|226437676|ref|NP_081789.1|	0	nucleoporin 205 [Mus musculus]

mmu-miR-1193-3p	NM_027551	-26.4	0.651676	-25.16	158	-	K10466|1|0.0|1196|mmu:70788|kelch-like protein	gi|58037217|ref|NP_081827.1|	0	kelch-like protein 30 [Mus musculus]
mmu-miR-1193-3p	NM_028785	-28.1	0.736214	-28.08	164	GO:0044444,GO:0032561,GO:0030695,GO:0019899,GO:0007599,	K05727|1|2e-13|80.5|nvi:100118634|dedicator of cytokinesis 3/4/5!K13708|2|4e-13|79.7|tca:661070|dedicator of cytokinesis 1	gi|62241030|ref|NP_083061.2|	0	dedicator of cytokinesis protein 8 [Mus musculus]
mmu-miR-1193-3p	NM_030143	-29.1	0.243304	-25.41	163	GO:0044424,GO:0007165,	K08270|1|3e-21|105|xtr:394782|DNA-damage-inducible transcript 4	gi|344258652|gb|EGW14756.1|	8.94E-112	DNA-damage-inducible transcript 4-like protein [Cricetulus griseus]

mmu-miR-1193-3p	NM_030715	-28.9	0.248966	-26.97	166	GO:0031981,GO:0043169,GO:0003677,GO:0034061,GO:0009411,GO:0006281,	K03509|1|0.0|1201|mmu:80905|DNA polymerase eta subunit [EC:2.7.7.7]	gi|17105328|ref|NP_109640.1|	0	DNA polymerase eta [Mus musculus]
mmu-miR-1193-3p	NM_053197	-30	0.187095	-28.04	174	GO:0005740,GO:0031224,GO:0015075,GO:0015142,GO:0000041,	K03351|1|3e-94|347|smm:Smp_136960|anaphase-promoting complex subunit 4	gi|16716499|ref|NP_444427.1|	1.69E-173	sideroflexin-3 isoform 1 [Mus musculus]
mmu-miR-1193-3p	NM_053253	-25.3	0.624388	-45.36	317	GO:0044424,GO:0046914,	-	gi|31982595|ref|NP_444483.2|	0	zinc finger MYND domain-containing protein 10 [Mus musculus]
mmu-miR-1193-3p	NM_138749	-26.6	0.875507	-41.67	315	GO:0031224,GO:0008047,GO:0005488,GO:0004871,GO:0031346,GO:0032501,GO:00099	K06821|1|0.0|3692|mmu:140570|plexin B	gi|226958474|ref|NP_620088.2|	0	plexin-B2 precursor [Mus musculus]

mmu-miR-1193-3p	NM_139001	-25.9	0.966123	-45.86	303	GO:0016021,GO:0044459,GO:0030027,GO:0019900,GO:0060089,GO:0016477,GO:0048646,GO:0000165,GO:0007167,GO:0042551,GO:0001934,	K08115|1|0.0|4433|mmu:121021|chondroitin sulfate proteoglycan 4	gi|146231960|ref|NP_620570.2|	0	chondroitin sulfate proteoglycan 4 precursor [Mus musculus]
mmu-miR-1193-3p	NM_145473	-29.6	0.182245	-26.13	159	GO:0043231,GO:0003676,GO:0006351,GO:0008334,GO:0006396,	-	gi|6114756|emb|CAA62001.2|	8.90E-112	PIPPin protein [Rattus norvegicus]
mmu-miR-1193-3p	NM_145475	-30.6	0.231979	-29.23	169	GO:0043231,GO:0001727,GO:0046872,GO:0032559,GO:0007186,GO:0046519,	K04715|1|0.0|1016|mmu:223753|ceramide kinase [EC:2.7.1.138]	gi|62899061|ref|NP_663450.3|	0	ceramide kinase [Mus musculus]

mmu-miR-1193-3p	NM_146243	-29.6	0.273225	-27.52	170	GO:0015629,GO:0008092,GO:0032559,GO:0009987,	K10354|1|2e-90|335|cin:100184134|actin, alpha skeletal muscle!K05692|2|2e-90|335|aml:100466132|actin beta/gamma 1!K12314|3|3e-90|334|bfo:BRAFLDRAFT_115565|actin, alpha cardiac muscle	gi|5031571|ref|NP_005713.1|	0	actin-related protein 2 isoform b [Homo sapiens]
mmu-miR-1193-3p	NM_153159	-28.3	0.339242	-26.4	162	GO:0016020,GO:0031981,GO:0043169,GO:0004518,GO:0048646,GO:0032501,GO:0012501,GO:0048869,	-	gi|148698376|gb|EDL30323.1|	0	zinc finger CCCH type containing 12A [Mus musculus]

mmu-miR-1193-3p	NM_172864	-32	0.084813	-29.42	178	-	K11143|1|6e-27|124|nve:NEMVE_v1g186877|dynein intermediate chain 2, axonemal	gi|197927139|ref|NP_766452.2|	0	WD repeat domain 63 [Mus musculus]
mmu-miR-1193-3p	NM_177613	-29.6	0.080799	-25.26	162	GO:0031981,GO:0016787,GO:0032559,GO:0019787,GO:0000209,GO:0010033,GO:0009968,GO:0006259,GO:0006511,GO:0022402,GO:0043065,	K02207|1|1e-115|417|rno:299602|ubiquitin-conjugating enzyme E2 R [EC:6.3.2.19]	gi|397502346|ref|XP_003821822.1|	8.37E-115	PREDICTED: ubiquitin-conjugating enzyme E2 R1 [Pan paniscus]

mmu-miR-1193-3p	NM_178608	-29.4	0.303838	-26.62	160	GO:0005740,GO:0031224,GO:0001664,GO:0034613,	K15601|1|2e-65|252|cfa:474695|lysine-specific demethylase 3 [EC:1.14.11.-]!K00939|2|2e-06|56.6|bmy:Bm1_13790|adenylate kinase [EC:2.7.4.3]	gi|30519931|ref|NP_848723.1|	5.27E-82	receptor expression-enhancing protein 1 [Mus musculus]
mmu-miR-1193-3p	NM_181470	-25.4	0.607375	-40.16	304	-	K14798|1|0.0|854|mmu:353258|protein LTV1	gi|31324539|ref|NP_852135.1|	0	protein LTV1 homolog [Mus musculus]
mmu-miR-1193-3p	NR_015462	-25.1	0.870081	-48.25	310	-	-	gi|148695789|gb|EDL27736.1|	2.72E-63	mCG1040702 [Mus musculus]
mmu-miR-1193-3p	NR_015614	-27.5	0.397577	-25.01	162	-	-	gi|148674821|gb|EDL06768.1|	4.25E-18	mCG1028297, isoform CRA_c [Mus musculus]

mmu-miR-1193-3p	NR_033573	-29.2	0.109643	-27.17	158	GO:0044424,GO:0030695,GO:0004672,GO:0032559,GO:0000187,	K08833|1|1e-20|101|mmu:399510|mitogen-activated protein kinase kinase kinase kinase 5 [EC:2.7.11.1]	gi|148704651|gb|EDL36598.1|	5.34E-25	mCG1041621, isoform CRA_b [Mus musculus]
mmu-miR-1193-3p	NR_040741	-28.1	0.06964	-27.64	169	-	-	gi|149028701|gb|EDL84042.1|	7.49E-07	rCG58877 [Rattus norvegicus]
mmu-miR-1193-3p	NM_030238	-22.3	1	-64.78	460	GO:0030286,GO:0043231,GO:0044444,GO:0003774,GO:0016887,GO:0032559,GO:0000278,GO:0022402,GO:0007017,GO:0051234,GO:0022618,	K10413|1|0.0|9088|mmu:13424|dynein heavy chain 1, cytosolic	gi|134288917|ref|NP_084514.2|	0	cytoplasmic dynein 1 heavy chain 1 [Mus musculus]

mmu-miR-1193-3p	NM_176912	-20.6	1	-51.26	332	GO:0031224,GO:0004942,GO:0007166,GO:0042221,	K04171|1|1e-173|611|mmu:319430|G protein-coupled receptor 77	gi|225579146|ref|NP_795886.2|	1.62E-178	C5a anaphylatoxin chemotactic receptor C5L2 isoform 1 [Mus musculus]
mmu-miR-1193-3p	NM_008417	-24.5	0.99969	-46.35	324	-	K04875|1|0.0|884|rno:25468|potassium voltage-gated channel Shaker-related subfamily A member 2	gi|25742772|ref|NP_037102.1|	0	potassium voltage-gated channel subfamily A member 2 [Rattus norvegicus]
mmu-miR-1193-3p	NM_012060	-22.8	0.8788	-43.31	319	GO:0043231,GO:0016021,GO:0044444,GO:0031090,GO:0012505,GO:0005515,GO:0006915,GO:0002376,GO:0015031,	K14009|1|4e-95|348|mmu:27061|B-cell receptor-associated protein 31	gi|148697937|gb|EDL29884.1|	1.70E-114	B-cell receptor-associated protein 31, isoform CRA_a [Mus musculus]

mmu-miR-1193-3p	NM_172590	-24.6	0.908877	-50.07	318	-	K10260|1|1e-10|69.7|tad:TRIADDRAFT_30121|F-box and WD-40 domain protein 7	gi|123702001|ref|NP_766178.2|	0	WD repeat-containing protein 41 [Mus musculus]
mmu-miR-1193-3p	NM_013803	-22.9	0.9993	-41.88	313	GO:0016021,GO:0033267,GO:0004629,GO:0005488,GO:0004888,GO:0007166,GO:0043462,GO:0010959,GO:0008283,GO:0007610,GO:0070838,GO:0050880,GO:0032502,GO:0009593,GO:0006875,GO:00001	K04612|1|0.0|1922|mmu:12374|calcium-sensing receptor	gi|148762954|ref|NP_038831.2|	0	extracellular calcium-sensing receptor precursor [Mus musculus]

mmu-miR-1193-3p	NM_145142	-24.9	0.882405	-44.21	312	GO:0012505,GO:0043231,GO:0031224,GO:0008146,GO:0007613,GO:0005975,GO:00099	K09674|1|0.0|782|mmu:98388|carbohydrate 3-sulfotransferase 10 [EC:2.8.2.-]	gi|148682602|gb|EDL14549.1|	0	carbohydrate sulfotransferase 10, isoform CRA_b [Mus musculus]
mmu-miR-1193-3p	NM_011031	-24.5	0.843742	-45.44	310	GO:0044432,GO:0019842,GO:0046914,GO:0016701,GO:0019798,GO:0019471,	K00472|1|0.0|1038|rno:360526|prolyl 4-hydroxylase [EC:1.14.11.2]	gi|148701600|gb|EDL33547.1|	0	procollagen-proline, 2-oxoglutarate 4-dioxygenase (proline 4-hydroxylase), alpha II polypeptide, isoform CRA_e [Mus musculus]

mmu-miR-1193-3p	NM_001081171	-23.5	0.999993	-42.35	309	GO:0043256,GO:0004871,GO:0005102,GO:0007166,GO:0072659,GO:0008544,GO:0007155,GO:0001667,GO:0008283,GO:0042476,GO:0061138,GO:2000026,GO:0007044,GO:0006996,GO:0000904,GO:0030323,GO:0048754,GO:0030855,GO:00109	K06240|1|0.0|7470|mmu:16776|laminin, alpha 3/5	gi|124487155|ref|NP_001074640.1|	0	laminin subunit alpha-5 precursor [Mus musculus]
mmu-miR-1193-3p	NM_144811	-24.4	0.91547	-42.23	306	GO:0000785,GO:0043231,GO:0042393,GO:0003723,GO:0006325,GO:0010467,	K11454|1|3e-70|267|mmu:52609|chromobox protein 7	gi|21450179|ref|NP_659060.1|	2.22E-69	chromobox protein homolog 7 [Mus musculus]

mmu-miR-1193-3p	NR_037992	-23.4	0.996447	-40.67	303	-	K11323|1|2e-09|66.6|nvi:100116682|histone arginine demethylase JMJD6 [EC:1.14.1	gi|30520031|ref|NP_848774.1|	0	jmjC domain-containing protein 4 isoform 1 [Mus musculus]
mmu-miR-1193-3p	NR_027375	-27.5	0.689851	-23.67	177	-	-	gi|34785681|gb|AAH57158.1|	0	Ythdf3 protein [Mus musculus]
mmu-miR-1193-3p	NM_009147	-29.1	0.361539	-24.82	174	GO:0012507,GO:0005789,GO:0046914,GO:0006901,GO:0048193,GO:0015031,GO:00064	K14006|1|0.0|1560|rno:58817|protein transport protein SEC23	gi|67906177|ref|NP_033173.2|	0	protein transport protein Sec23A [Mus musculus]
mmu-miR-1193-3p	NM_013836	-28.2	0.695221	-23.88	173	GO:0043231,GO:0003712,GO:0046914,GO:0001071,GO:0043565,	K09188|1|1e-12|77.8|dya:Dyak_GE16999|histone-lysine N-methyltransferase MLL3 [EC:2.1.1.43]	gi|166706889|ref|NP_038864.3|	0	transcription factor 20 isoform b [Mus musculus]

mmu-miR-1193-3p	NM_023868	-29.5	0.788223	-24.54	171	GO:0005789,GO:0031674,GO:0034703,GO:0004871,GO:0051018,GO:0046872,GO:0005218,GO:0006941,GO:0010880,GO:0010038,GO:0006950,GO:0014074,GO:0009593,GO:00	K04962|1|0.0|9380|mmu:20191|ryanodine receptor 2	gi|124430578|ref|NP_076357.2|	0	ryanodine receptor 2 [Mus musculus]
mmu-miR-1193-3p	NM_027912	-27.8	0.335845	-22.83	170	GO:0042579,GO:0004175,GO:0019538,	-	gi|254675122|ref|NP_082188.1|	0	peroxisomal leader peptide-processing protease [Mus musculus]
mmu-miR-1193-3p	NM_201370	-24.5	0.905253	-20.18	169	GO:0005815,GO:0031981,GO:0046872,GO:0004713,GO:0032559,GO:0000087,GO:00064	K06632|1|0.0|1101|mmu:381759|wee1-like protein kinase [EC:2.7.11.1]	gi|148681638|gb|EDL13585.1|	0	cDNA sequence BC052883, isoform CRA_b [Mus musculus]
mmu-miR-1193-3p	NR_040388	-26	0.219981	-22.52	168	-	-	gi|149271743|ref|XP_001476904.1|	1.85E-122	PREDICTED: spindlin-2A-like isoform 2 [Mus musculus]

mmu-miR-1193-3p	NM_207221	-25.9	0.970032	-21.54	167	GO:0031981,GO:0043169,GO:0032452,GO:0016701,GO:0008134,GO:0070076,GO:0007599,GO:0006351,	K11449|1|0.0|4581|mmu:108829|jumonji domain-containing protein 1C [EC:1.14.11.-]	gi|226531205|ref|NP_997104.2|	0	probable JmjC domain-containing histone demethylation protein 2C isoform 1 [Mus musculus]
mmu-miR-1193-3p	NR_045793	-26.4	0.75454	-24.31	166	GO:0031981,GO:0003676,GO:0009790,	K14787|1|6e-24|114|mmu:74111|multiple RNA-binding domain-containing protein 1	gi|148687831|gb|EDL19778.1|	2.63E-108	mCG145980 [Mus musculus]
mmu-miR-1193-3p	NM_178256	-26.2	0.942287	-21.57	165	GO:0044424,GO:0005488,GO:0065003,GO:0007169,	K12477|1|2e-10|70.9|cfa:608795|EH domain-containing protein 4	gi|84490401|ref|NP_839987.2|	0	ralBP1-associated Eps domain-containing protein 2 [Mus musculus]

mmu-miR-1193-3p	NM_183152	-25.3	0.693216	-20.73	164	GO:0043169,GO:0004672,GO:0032559,GO:0006464,GO:0023052,	K08861|1|6e-122|439|xtr:100125189|polo-like kinase 2 [EC:2.7.11.21]	gi|65508357|ref|NP_898975.2|	0	inactive serine/threonine-protein kinase PLK5 [Mus musculus]
mmu-miR-1193-3p	NR_027784	-25.9	0.425819	-22.79	163	-	-	gi|148670098|gb|EDL02045.1|	1.08E-54	mCG142215, isoform CRA_a [Mus musculus]
mmu-miR-1193-3p	NR_040354	-25.7	0.868218	-21.89	162	GO:0044421,GO:0016023,GO:0004175,GO:0005515,GO:0048731,GO:0016486,	K01360|1|3e-28|129|mmu:18549|proprotein convertase subtilisin/kexin type 2 [EC:3.4.21.94]	gi|74179798|dbj|BAE36477.1|	5.09E-33	unnamed protein product [Mus musculus]
mmu-miR-1193-3p	NR_028417	-29.3	0.105834	-24.5	161	-	-	gi|148705637|gb|EDL37584.1|	3.85E-39	mCG146113 [Mus musculus]
mmu-miR-1193-3p	NR_040343	-26.1	0.682884	-23.57	160	-	-	-	-	-
mmu-miR-1193-3p	NR_030708	-25.4	0.920374	-22.96	159	GO:0044424,GO:0031224,GO:0046872,GO:0016337,	K06803|1|8e-97|193|mmu:12552|cadherin 11, type 2, OB-cadherin	gi|74145351|dbj|BAE36133.1|	1.08E-128	unnamed protein product [Mus musculus]

mmu-miR-1193-3p	NR_073191	-22.5	0.954728	-20.6	158	-	-	-	-	-
mmu-miR-1193-3p	NR_073427	-24.2	0.724409	-20.22	157	-	-	-	-	-
mmu-miR-1193-3p	NR_037590	-23.2	0.982951	-20.36	156	GO:0031224,GO:0043231,GO:0012506,GO:0060089,GO:0005515,GO:0030177,GO:0009798,GO:0007249,GO:0001704,	-	gi|148679892|gb|EDL11839.1|	0	G protein-coupled receptor 177, isoform CRA_a [Mus musculus]
mmu-miR-1193-3p	NR_033148	-21.3	0.999999	-21.05	155	GO:0016604,GO:0005488,GO:0048731,GO:0006355,	K10130|1|3e-24|115|ptr:450935|leucine-rich repeats and death domain-containing protein	gi|22122701|ref|NP_666276.1|	0	leucine-rich repeat and calponin homology domain-containing protein 4 isoform 1 [Mus musculus]
mmu-miR-1193-3p	NM_199152	-23.3	1	-21.08	154	-	K12567|1|0.0|1170|mmu:22138|titin [EC:2.7.11.1]	gi|345842337|ref|NP_954603.3|	0	obscurin isoform 2 [Mus musculus]

mmu-miR-1193-3p	NR_045394	-23.8	0.451198	-20.35	153	GO:0016021,GO:0004714,GO:0032559,GO:0006464,GO:0007167,	K05102|1|2e-23|82.4|ptr:463808|Eph receptor A1 [EC:2.7.10.1]	gi|239782133|pdb|3HIL|A	3.95E-24	Chain A, Sam Domain Of Human Ephrin Type-A Receptor 1 (Epha1)
mmu-miR-1193-3p	NR_046179	-24.9	0.883681	-23.96	152	-	-	gi|148666314|gb|EDK98730.1|	3.20E-45	mCG144490 [Mus musculus]
mmu-miR-1193-3p	NR_033615	-24.6	0.991279	-21.21	151	GO:0044451,GO:0003676,GO:0046872,GO:0007264,GO:0032501,GO:0006351,	K09228|1|4e-15|85.9|xla:779424|KRAB domain-containing zinc finger protein	gi|152060560|sp|Q3UH06.2|RREB1_MOUSE	1.60E-167	RecName: Full=Ras-responsive element-binding protein 1; Short=RREB-1; AltName: Full=RAS-responsive zinc finger transcription factor RREB
mmu-miR-1193-3p	NR_040563	-24.7	0.413816	-20.85	150	-	-	gi|74205457|dbj|BAE21039.1|	9.47E-54	unnamed protein product [Mus musculus]
mmu-miR-1193-3p	NR_045413	-24.1	0.383798	-20.59	149	-	-	-	-	-
mmu-miR-1193-3p	NR_045693	-21.1	1	-21.11	148	-	K08835|1|0.0|904|rno:316064|oxidative-stress responsive protein 1 [EC:2.7.11.1]	gi|148677282|gb|EDL09229.1|	0	mCG20975, isoform CRA_c [Mus musculus]

mmu-miR-1193-5p	NM_001033205	-25.2	0.863396	-43	322	GO:0043231,GO:0046914,GO:0003676,GO:0010467,	K09228|1|4e-29|130|pon:100442553|KRAB domain-containing zinc finger protein	gi|85701718|ref|NP_001028377.1|	2.12E-122	zinc finger protein 575 [Mus musculus]
mmu-miR-1193-5p	NM_001081050	-32.7	0.247836	-30.79	161	GO:0043296,GO:0009987,	K04237|1|1e-162|576|mdo:100022889|partitioning defective protein 3	gi|163310767|ref|NP_001074519.2|	0	partitioning defective 3 homolog B [Mus musculus]
mmu-miR-1193-5p	NM_001081672	-27.5	0.461632	-45.26	315	-	-	gi|62649906|ref|XP_233583.3|	2.65E-142	PREDICTED: protein FAM43B-like [Rattus norvegicus]
mmu-miR-1193-5p	NM_001115132	-31.9	0.127893	-28.48	168	GO:0043232,GO:0043231,GO:0030217,GO:0051276,	K11490|1|0.0|1125|mmu:52683|condensin-2 complex subunit H2	gi|148672401|gb|EDL04348.1|	0	DNA segment, Chr 15, ERATO Doi 785, expressed, isoform CRA_b [Mus musculus]
mmu-miR-1193-5p	NM_001122993	-28	0.690376	-27.32	158	GO:0012505,GO:0043231,GO:0031224,GO:0035250,GO:0006464,	K03877|1|8e-174|612|mmu:93961|beta-1,3-galactosyltransferase 5 [EC:2.4.1.-	gi|15011870|ref|NP_149161.1|	5.40E-173	beta-1,3-galactosyltransferase 5 [Mus musculus]

mmu-miR-1193-5p	NM_001163662	-32.5	0.075245	-28.61	161	GO:0005856,GO:0044444,GO:0044421,GO:0043231,GO:0046872,GO:0003676,	K12385|1|1e-66|255|isc:IscW_ISCW020300|Niemann-Pick C1 protein	gi|148690913|gb|EDL22860.1|	0	nucleobindin 1, isoform CRA_a [Mus musculus]
mmu-miR-1193-5p	NM_001163793	-25.9	0.970204	-43.84	303	-	K13172|1|5e-09|65.9|oaa:100075123|serine/arginine repetitive matrix protein 2!K03626|2|5e-08|62.4|mmu:17938|nascent polypeptide-associated complex subunit alpha!K14000|3|9e-08|61.6|dre:553448|ribosome-binding protein 1!K04574|4|1e-	gi|256985117|ref|NP_001157265.1|	0	uncharacterized protein KIAA1211 [Mus musculus]

mmu-miR-1193-5p	NM_001167994	-26.2	0.793064	-25.45	165	GO:0043231,GO:0008757,GO:0006399,GO:0001510,GO:0010467,	K15331|1|0.0|1015|mmu:215201|tRNA (uracil-5-)-methyltransferase [EC:2.1.1.35]	gi|269784754|ref|NP_766128.2|	0	tRNA (uracil(54)-C(5))-methyltransferase homolog isoform a [Mus musculus]
mmu-miR-1193-5p	NM_001177544	-29.6	0.02765	-25.57	159	GO:0000785,GO:0043231,GO:0003676,GO:0005515,GO:0034728,	K11254|1|2e-56|218|rno:291152|histone H4!K11251|2|2e-54|211|cfa:100856200|histone H2A	gi|380800771|gb|AFE72261.1|	1.20E-53	histone H2A type 1-B/E, partial [Macaca mulatta]
mmu-miR-1193-5p	NM_001253680	-29.1	0.238072	-26.22	164	GO:0016021,GO:0044459,GO:0005275,GO:0019752,GO:0002376,GO:0006807,GO:0007599,GO:0065003,GO:0006865,	K13867|1|0.0|934|mmu:20540|solute carrier family 7 (L-type amino acid transporter), member 7	gi|110625647|ref|NP_035535.2|	0	Y+L amino acid transporter 1 [Mus musculus]
mmu-miR-1193-5p	NM_001271601	-31.9	0.128025	-28.48	168	-	-	-	-	-

mmu-miR-1193-5p	NM_007566	-28.9	0.896199	-26.59	161	-	K10586|1|0.0|8523|mmu:12211|baculoviral IAP repeat-containing protein 6 (apollon) [EC:6.3.2.19]	gi|380876899|sp|O88738.2|BIRC6_MOUSE	0	RecName: Full=Baculoviral IAP repeat-containing protein 6; AltName: Full=BIR repeat-containing ubiquitin-conjugating enzyme; Short=BRUCE; AltName: Full=Ubiquitin-conjugating BIR domain enzyme apollon; Short=APOLLON
mmu-miR-1193-5p	NM_007634	-26.5	0.722941	-26.4	164	GO:0043231,GO:0031461,GO:0005813,GO:0005488,GO:0043161,GO:0010639,GO:0032446,GO:0000087,GO:0048513,	K10289|1|0.0|1432|mmu:12449|F-box protein 1 (cyclin F)	gi|148540012|ref|NP_031660.3|	0	cyclin-F [Mus musculus]

mmu-miR-1193-5p	NM_008575	-28	0.534193	-25.59	164	GO:0043231,GO:0046914,GO:0005515,GO:0000320,GO:0007050,GO:0006357,GO:0006915,GO:0031647,GO:0009895,GO:0065003,GO:0008283,	K10127|1|0.0|942|mmu:17248|protein Mdm4	gi|315432 45|ref|NP_032601.2|	0	protein Mdm4 [Mus musculus]
mmu-miR-1193-5p	NM_008749	-32.5	0.074814	-28.61	161	GO:0005856,GO:0044444,GO:0044421,GO:0043231,GO:0046872,GO:0003676,	K12385|1|1e-66|255|isc:IscW_ISCW020300|Niemann-Pick C1 protein	gi|148690913|gb|EDL22860.1|	0	nucleobindin 1, isoform CRA_a [Mus musculus]
mmu-miR-1193-5p	NM_009462	-27.2	0.629638	-48.06	310	GO:0005856,GO:0005768,GO:0016790,GO:0005515,GO:0019783,GO:0006259,GO:0019941,GO:0042770,GO:0070646,	K11841|1|0.0|1522|mmu:22224|ubiquitin carboxyl-terminal hydrolase 10 [EC:3.1.2.15]	gi|148679672|gb|EDL11619.1|	0	ubiquitin specific peptidase 10, isoform CRA_a [Mus musculus]

mmu-miR-1193-5p	NM_009747	-29	0.18863	-26	168	GO:0043231,GO:0016021,GO:0004629,GO:0031701,GO:0046983,GO:0008528,GO:0019899,GO:0015909,GO:0008283,GO:0006936,GO:0002437,GO:0042310,GO:0007167,GO:0051480,GO:0001933,GO:0006970,GO:0001666,GO:0006915,GO:0007600,GO:00	K03916|1|0.0|731|mmu:12062|bradykinin receptor B2	gi|148686811|gb|EDL18758.1|	0	bradykinin receptor, beta 2, isoform CRA_a [Mus musculus]

mmu-miR-1193-5p	NM_009909	-28.6	0.39824	-25.77	165	GO:0005764,GO:0016021,GO:0004950,GO:0019958,GO:0004896,GO:0012501,GO:0006952,GO:0007186,GO:0002274,GO:0008283,GO:0030595,	K05050|1|0.0|685|mmu:12765|interleukin 8 receptor beta	gi|6753456|ref|NP_034039.1|	0	C-X-C chemokine receptor type 2 [Mus musculus]
mmu-miR-1193-5p	NM_009932	-29.5	0.538884	-26.75	158	GO:0005604,GO:0005488,GO:0001525,	K06237|1|0.0|2370|mmu:12827|collagen, type IV, alpha	gi|226437587|ref|NP_034062.3|	0	collagen alpha-2(IV) chain preproprotein [Mus musculus]
mmu-miR-1193-5p	NM_010437	-29.4	0.703613	-26.34	164	GO:0043231,GO:0043234,GO:0003676,GO:0046914,GO:0001071,GO:0023052,GO:0006351,	K09239|1|0.0|4360|mmu:15273|human immunodeficiency virus type I enhancer-binding protein	gi|85861241|ref|NP_034567.2|	0	transcription factor HIVEP2 [Mus musculus]

mmu-miR-1193-5p	NM_011197	-26.4	0.915195	-44.91	306	GO:0031224,GO:0043231,GO:0031090,GO:0005488,GO:0004871,GO:0006412,GO:0006996,GO:0023052,	K06729|1|0.0|1670|mmu:19221|prostaglandin F2 receptor negative regulator	gi|148675683|gb|EDL07630.1|	0	prostaglandin F2 receptor negative regulator [Mus musculus]
mmu-miR-1193-5p	NM_019676	-29.2	0.283847	-26.53	152	GO:0044425,GO:0005740,GO:0004629,GO:0005515,GO:0060089,GO:0035091,GO:0046872,GO:0032959,GO:0007166,GO:0048568,GO:0007204,GO:0008283,GO:0006629,GO:0010038,GO:0006950,GO:0048646,GO:0034694,GO:0014061,	K05857|1|0.0|1464|mmu:18799|phosphatidylinositol phospholipase C, delta [EC:3.1.4.11]	gi|9790167|ref|NP_062650.1|	0	1-phosphatidylinositol 4,5-bisphosphate phosphodiesterase delta-1 [Mus musculus]

mmu-miR-1193-5p	NM_022417	-33.3	0.037531	-30.16	174	GO:0031224,GO:0005764,GO:0005515,GO:0032559,GO:0043068,GO:0010975,	-	gi|297265084|ref|XP_001112946.2|	2.13E-121	PREDICTED: integral membrane protein 2C isoform 5 [Macaca mulatta]
mmu-miR-1193-5p	NM_022999	-28.5	0.174216	-26.33	150	GO:0016021,GO:0046872,	K01320|1|4e-11|70.1|ecb:100067072|coagulation factor VII [EC:3.4.21.21]!K01321|2|5e-11|69.7|xla:100049721|coagulation factor IX (Christmas factor) [EC:3.4.21.22]!K01314|5|2e-09|64.7|aml:100471482|coagulation factor X [EC:3.4.21.6]	gi|148690844|gb|EDL22791.1|	8.78E-112	proline-rich Gla (G-carboxyglutamic acid) polypeptide 2, isoform CRA_b [Mus musculus]

mmu-miR-1193-5p	NM_023596	-29.2	0.508084	-26.73	156	GO:0005770,GO:0005764,GO:0031224,GO:0015932,GO:0006139,GO:0015931,	K15014|1|0.0|800|mmu:71279|solute carrier family 29 (equilibrative nucleoside transporter), member 1/2/3	gi|12963743|ref|NP_076085.1|	0	equilibrative nucleoside transporter 3 [Mus musculus]
mmu-miR-1193-5p	NM_025864	-31.5	0.192384	-27.24	155	GO:0031224,	-	gi|13385348|ref|NP_080140.1|	9.80E-162	transmembrane protein 206 [Mus musculus]
mmu-miR-1193-5p	NM_026267	-27.7	0.436647	-26.22	163	GO:0012505,GO:0030118,GO:0030136,GO:0005488,GO:0016192,	-	gi|27229051|ref|NP_080543.2|	7.32E-132	adaptin ear-binding coat-associated protein 1 [Mus musculus]
mmu-miR-1193-5p	NM_027394	-28.4	0.263044	-25.19	157	GO:0044424,GO:0003824,	K12584|1|8e-13|76.3|tca:658490|scavenger mRNA-decapping enzyme DcpS [EC:3.-.-.-]	gi|110625767|ref|NP_081670.1|	0	E3 ubiquitin-protein ligase E3D [Mus musculus]

mmu-miR-1193-5p	NM_027460	-29.4	0.181727	-28.16	152	GO:0019866,GO:0031224,GO:0043231,GO:0051234,	K15116|1|5e-178|625|mmu:70556|solute carrier family 25, member 33/36	gi|117647275|ref|NP_081736.2|	3.27E-177	solute carrier family 25 member 33 [Mus musculus]
mmu-miR-1193-5p	NM_027985	-27.9	0.206856	-25.01	156	GO:0031981,GO:0000152,GO:0042575,GO:0015630,GO:0016779,GO:0019901,GO:0001934,GO:0000725,GO:0019985,GO:0042770,GO:0045841,GO:0032435,GO:0009416,GO:00	K13728|1|4e-114|412|mmu:71890|mitotic spindle assembly checkpoint protein MAD2B	gi|126090494|ref|NP_082261.2|	2.48E-113	mitotic spindle assembly checkpoint protein MAD2B [Mus musculus]
mmu-miR-1193-5p	NM_028801	-28	0.952116	-63.47	454	GO:0005488,	K13908|1|0.0|8364|mmu:74180|mucin-5B	gi|147905740|ref|NP_083077.2|	0	mucin 5, subtype B, tracheobronchial precursor [Mus musculus]

mmu-miR-1193-5p	NM_028973	-28.4	0.632973	-26.21	161	GO:0031224,GO:0009987,	K13023|1|0.0|847|mcc:707574|carboxypeptidase N regulatory subunit!K06260|3|2e-81|305|gga:100857508|platelet glycoprot	gi|58037377|ref|NP_083249.1|	0	leucine-rich repeat-containing protein 15 precursor [Mus musculus]
mmu-miR-1193-5p	NM_029554	-29.4	0.180954	-25.45	156	-	-	gi|148705758|gb|EDL37705.1|	0	RIKEN cDNA 0610040J01, isoform CRA_c [Mus musculus]
mmu-miR-1193-5p	NM_029951	-28	0.517461	-25.22	161	GO:0005488,	-	gi|26336262|dbj|BAC31816.1|	1.04E-102	unnamed protein product [Mus musculus]
mmu-miR-1193-5p	NM_031198	-29.2	0.185516	-25.35	155	GO:0043231,GO:0003676,GO:0006351,GO:0009408,	K15591|1|9e-166|584|mmu:21426|transcription factor EC	gi|13654264|ref|NP_112475.1|	6.00E-165	transcription factor EC [Mus musculus]

mmu-miR-1193-5p	NM_031874	-29.3	0.388335	-26.66	156	GO:0030141,GO:0016020,GO:0017111,GO:0019899,GO:0032561,GO:0035556,GO:0006887,GO:0006479,GO:0009207,	K07884|1|7e-108|393|mmu:19340|Ras-related protein Rab-3D	gi|15042957|ref|NP_114080.2|	4.81E-107	ras-related protein Rab-3D [Mus musculus]
mmu-miR-1193-5p	NM_033149	-28	0.66401	-27.32	158	GO:0012505,GO:0043231,GO:0031224,GO:0035250,GO:0006464,	K03877|1|7e-174|612|mmu:93961|beta-1,3-galactosyltransferase 5 [EC:2.4.1.-	gi|15011870|ref|NP_149161.1|	4.99E-173	beta-1,3-galactosyltransferase 5 [Mus musculus]
mmu-miR-1193-5p	NM_144853	-26.3	0.590279	-25.64	155	-	-	gi|74190182|dbj|BAE37209.1|	1.62E-68	unnamed protein product [Mus musculus]
mmu-miR-1193-5p	NM_145430	-28.5	0.499783	-26.07	164	-	-	gi|38649280|gb|AAH63099.1|	0	CDNA sequence BC017647 [Mus musculus]
mmu-miR-1193-5p	NM_146164	-25.5	0.859855	-46.01	307	GO:0016604,GO:0005488,GO:0048731,GO:0006355,	K10130|1|2e-24|115|ptr:450935|leucine-rich repeats and death domain-containing protein	gi|22122701|ref|NP_666276.1|	0	leucine-rich repeat and calponin homology domain-containing protein 4 isoform 1 [Mus musculus]

mmu-miR-1193-5p	NM_153560	-27.7	0.652826	-26.1	163	-	-	gi|23956346|ref|NP_705788.1|	2.33E-171	protein FAM102A [Mus musculus]
mmu-miR-1193-5p	NM_172540	-26.2	0.785402	-25.45	165	GO:0043231,GO:0008757,GO:0006399,GO:0001510,GO:0010467,	K15331|1|0.0|1015|mmu:215201|tRNA (uracil-5-)-methyltransferase [EC:2.1.1.35]	gi|269784754|ref|NP_766128.2|	0	tRNA (uracil(54)-C(5))-methyltransferase homolog isoform a [Mus musculus]
mmu-miR-1193-5p	NM_172784	-28.6	0.441417	-28.65	156	GO:0031224,GO:0004871,GO:0023052,	K15619|1|3e-30|135|tgu:100226787|Kunitz-type protease inhibitor 1	gi|31542055|ref|NP_766372.1|	0	low-density lipoprotein receptor-related protein 11 precursor [Mus musculus]
mmu-miR-1193-5p	NM_173370	-26.4	0.818752	-26.8	154	GO:0031224,GO:0043231,GO:0031090,GO:0017169,GO:0070567,GO:0006656,GO:0050953,GO:0023052,GO:0006651,	K00981|1|0.0|859|mmu:74596|phosphatidate cytidylyltransferase [EC:2.7.7.41]	gi|33186923|ref|NP_775546.2|	0	phosphatidate cytidylyltransferase 1 [Mus musculus]
mmu-miR-1193-5p	NM_173414	-29.4	0.380101	-25.97	157	-	-	gi|238637328|ref|NP_775590.2|	0	lanC-like protein 3 [Mus musculus]

mmu-miR-1193-5p	NM_173733	-29.1	0.260844	-29.51	162	GO:0005740,GO:0005506,GO:0050662,GO:0016670,GO:0031667,GO:0008152,	K00387|1|0.0|1048|mmu:211389|sulfite oxidase [EC:1.8.3.1]	gi|74024924|ref|NP_776094.2|	0	sulfite oxidase, mitochondrial precursor [Mus musculus]
mmu-miR-1193-5p	NM_174857	-29.4	0.329346	-25.05	154	GO:0043231,GO:0005578,GO:0030247,GO:0018149,	K08606|1|3e-23|111|hmg:100199496|meprin A, beta [EC:3.4.24.	gi|33469047|ref|NP_777282.1|	0	MAM domain-containing protein 2 precursor [Mus musculus]
mmu-miR-1193-5p	NM_177775	-29.7	0.386921	-27.07	169	GO:0031224,	K15290|1|4e-12|75.5|isc:IscW_ISCW011463|synaptotagmin-1	gi|60360572|dbj|BAD90311.1|	0	mKIAA4186 protein [Mus musculus]
mmu-miR-1193-5p	NM_178185	-29.6	0.017601	-25.57	159	GO:0000785,GO:0043231,GO:0003676,GO:0005515,GO:0034728,	K11251|1|1e-54|211|cfa:100856200|histone H2A	gi|380800771|gb|AFE72261.1|	8.77E-54	histone H2A type 1-B/E, partial [Macaca mulatta]
mmu-miR-1193-5p	NM_178186	-29.6	0.028795	-25.57	159	GO:0000785,GO:0043231,GO:0003676,GO:0005515,GO:0034728,	K11251|1|2e-54|211|cfa:100856200|histone H2A	gi|380800771|gb|AFE72261.1|	1.30E-53	histone H2A type 1-B/E, partial [Macaca mulatta]

mmu-miR-1193-5p	NM_178189	-29.6	0.229196	-25.57	159	GO:0000785,GO:0043231,GO:0003676,GO:0005515,GO:0034728,	K11251|1|2e-53|211|cfa:100856200|histone H2A	gi|380800771|gb|AFE72261.1|	1.59E-52	histone H2A type 1-B/E, partial [Macaca mulatta]
mmu-miR-1193-5p	NM_199305	-28.6	0.245701	-25.48	158	GO:0031224,	-	gi|148698217|gb|EDL30164.1|	0	transmembrane protein 39b, isoform CRA_b [Mus musculus]
mmu-miR-1193-5p	NR_040593	-30.8	0.04127	-27.84	166	-	-	gi|148703345|gb|EDL35292.1|	3.50E-61	mCG145531 [Mus musculus]
mmu-miR-1193-5p	NR_073376	-31.9	0.127144	-28.48	168	-	-	-	-	-
mmu-miR-1193-5p	NR_073378	-36	0.011799	-32.71	174	-	-	-	-	-
mmu-miR-1193-5p	NM_001013749	-24.6	0.987891	-43.61	315	GO:0031224,	-	gi|85701444|ref|NP_001013771.1|	0	transmembrane protein 151B [Mus musculus]
mmu-miR-1193-5p	NR_033148	-24.1	0.993881	-46.01	307	GO:0016604,GO:0005488,GO:0048731,GO:0006355,	K10130|1|3e-24|115|ptr:450935|leucine-rich repeats and death domain-containing protein	gi|22122701|ref|NP_666276.1|	0	leucine-rich repeat and calponin homology domain-containing protein 4 isoform 1 [Mus musculus]

mmu-miR-1193-5p	NM_028993	-23.3	0.999735	-42.93	306	GO:0044427,GO:0043234,GO:0031981,GO:0005515,GO:0007064,	K11266|1|0.0|1174|pon:100454877|MAternally affected uncoordination	gi|148696826|gb|EDL28773.1|	0	RIKEN cDNA 9130404D08 [Mus musculus]
mmu-miR-1193-5p	NR_045571	-23.2	0.922352	-43.82	304	GO:0043232,	-	gi|13385766|ref|NP_080533.1|	0	UBX domain-containing protein 11 [Mus musculus]

mmu-miR-1193-5p	NM_001081116	-22.7	1	-42.03	303	GO:0044444,GO:0005088,GO:0006917,GO:0007010,GO:0009966,GO:0007169,GO:0007264,	K05156|1|0.0|791|gga:419067|tumor necrosis factor receptor superfamily member 19-like!K01634|2|2e-54|217|mgp:100545432|sphinganine-1-phosphate aldolase [EC:4.1.2.27]!K04436|3|3e-29|133|xtr:100170186|mitogen-activated protein	gi|124487439|ref|NP_001074585.1|	0	rho guanine nucleotide exchange factor 17 [Mus musculus]

mmu-miR-1193-5p	NM_199027	-20.2	1	-48.42	301	GO:0044464,GO:0046914,	K09228|1|9e-38|160|bfo:BRAFLDRAFT_66688|KRAB domain-containing zinc finger protein	gi|124517714|ref|NP_950192.2|	0	zinc finger protein 335 [Mus musculus]
mmu-miR-1193-5p	NM_201394	-22.6	1	-41.55	297	-	K10388|1|0.0|3881|mmu:18810|plectin	gi|256418964|ref|NP_958788.2|	0	plectin isoform 1e [Mus musculus]
mmu-miR-1193-5p	NM_001081229	-20.7	1	-40.42	290	-	-	gi|148703386|gb|EDL35333.1|	3.09E-136	mCG8104 [Mus musculus]

mmu-miR-1193-5p	NM_008809	-20.1	1	-30.36	172	GO:0016021,GO:0019838,GO:0005017,GO:0004930,GO:0070851,GO:0032559,GO:0019900,GO:0009611,GO:0006023,GO:0007178,GO:0010557,GO:0033189,GO:0008283,GO:0043627,GO:0060537,GO:0000302,GO:0043009,GO:0007169,GO:0001932,GO:0006935,GO:0009887,GO:00352	K05089|1|0.0|2057|mmu:18596|platelet-derived growth factor receptor beta [EC:2.7.10.1]	gi|148677841|gb|EDL09788.1|		0	platelet derived growth factor receptor, beta polypeptide, isoform CRA_b [Mus musculus]
mmu-miR-1193-5p	NM_027429	-26.5	0.835037	-21.1	169	GO:0044427,GO:0043231,GO:0044444,GO:0007067,	K11504|1|0.0|663|mmu:70454|centromere protein L	gi|229576902|ref|NP_081705.2|		0	centromere protein L isoform 2 [Mus musculus]

mmu-miR-1193-5p	NM_011105	-20.7	1	-27.11	168	GO:0031224,GO:0015075,GO:0046872,GO:0006811,GO:0009987,GO:0007338,	K04988|1|2e-101|372|bfo:BRAFLDRAFT_92411|polycystin 1L2	gi|115583675|ref|NP_035235.2|	0	polycystic kidney disease and receptor for egg jelly-related protein precursor [Mus musculus]
mmu-miR-1193-5p	NM_007377	-20.2	1	-27.43	167	GO:0043231,GO:0031224,GO:0004672,GO:0032559,GO:0006468,GO:0012501,	K08899|1|1e-128|462|ssc:100623034|lemur tyrosine kinase 3 [EC:2.7.10.1]	gi|39104555|dbj|BAC41437.2|	0	mKIAA0641 protein [Mus musculus]
mmu-miR-1193-5p	NM_133224	-25.3	0.932978	-23.3	166	GO:0031224,GO:0043169,GO:0042625,GO:0032559,GO:0009206,GO:0006811,GO:0009207,	K14950|1|0.0|2165|mmu:170759|cation-transporting ATPase 13A1 [EC:3.6.3.-]	gi|283135194|ref|NP_573487.2|	0	probable cation-transporting ATPase 13A1 [Mus musculus]
mmu-miR-1193-5p	NR_046061	-26.6	0.416705	-21.83	165	-	-	gi|148709290|gb|EDL41236.1|	2.68E-51	mCG1044050 [Mus musculus]
mmu-miR-1193-5p	NR_045780	-27.1	0.107655	-24.02	164	-	-	-	-	-
mmu-miR-1193-5p	NM_133721	-20.2	1	-24.15	163	GO:0005887,GO:0004871,GO:0006935,GO:0007166,	K06585|1|0.0|1915|rno:685004|integrin alpha 9	gi|165377045|ref|NP_598482.2|	0	integrin alpha 9 isoform a precursor [Mus musculus]

mmu-miR-1193-5p	NM_146951	-24.9	0.500868	-20.17	162	GO:0004888,GO:0023052,	K04257|1|7e-154|543|mmu:258953|olfactory	gi|22129019|ref|NP_667162.1|	4.80E-153	olfactory receptor 340 [Mus musculus]
mmu-miR-1193-5p	NR_037617	-22.3	0.999976	-23.75	161	GO:0005488,GO:0043410,	K12861|1|9e-165|582|gga:419884|pre-mRNA-splicing factor SPF27!K01289|2|8e-28|127|dme:Dmel_CG18659|carboxypeptidase [EC:3.4.16.	gi|47679093|ref|NP_001001326.1|	0	suppression of tumorigenicity 5 protein isoform 1 [Mus musculus]
mmu-miR-1193-5p	NR_036665	-28	0.36462	-24.44	160	GO:0044424,GO:0005515,GO:0022602,GO:0001667,GO:0009887,GO:0007169,GO:0060537,GO:0008202,GO:0008406,	K04514|1|4e-27|124|tca:663533|Rho-associated, coiled-coil containing protein kinase [EC:2.7.11.1]	gi|164663886|ref|NP_001106892.1|	5.17E-177	schwannomin-interacting protein 1 isoform c [Mus musculus]

mmu-miR-1193-5p	NM_177265	-26.3	0.767752	-22.95	159	GO:0031224,GO:0050896,	K06566|1|4e-08|61.6|xla:734801|interferon induced transmembrane protein	gi|148686194|gb|EDL18141.1|	4.81E-121	RIKEN cDNA 6330512M04 [Mus musculus]
mmu-miR-1193-5p	NM_201243	-26.9	0.839779	-21.91	158	GO:0044421,GO:0016021,GO:0044424,GO:0046872,GO:0048731,GO:0016337,	K04600|1|4e-61|238|dvi:Dvir_GJ22222|cadherin EGF LAG seven-pass G-type receptor 1 (flamingo)	gi|51092279|ref|NP_957695.1|	0	protocadherin alpha-9 precursor [Mus musculus]
mmu-miR-1193-5p	NM_178786	-26	0.87144	-23.03	157	GO:0044464,	K06712|1|2e-45|185|mcc:700608|butyrophilin	gi|240255629|ref|NP_848901.2|	0	selection and upkeep of intraepithelial T-cells protein 4 [Mus musculus]
mmu-miR-1193-5p	NM_198414	-26.6	0.591691	-23.24	156	GO:0031224,GO:0004871,	-	gi|116089314|ref|NP_940806.2|	0	progestin and adipoQ receptor family member 9 [Mus musculus]

mmu-miR-1193-5p	NR_028260	-23.6	0.997163	-20.52	155	GO:0043231,GO:0031224,GO:0004175,GO:0046914,GO:0016462,GO:0032559,GO:0009057,GO:0019538,	K08956|1|0.0|968|mmu:114896|AFG3 family protein [EC:3.4.24.-]	gi|66792806|ref|NP_473411.2|	0	AFG3-like protein 1 [Mus musculus]
mmu-miR-1193-5p	NM_203319	-24.5	0.982962	-20.65	154	GO:0032559,GO:0042623,	K14780|1|0.0|1988|mmu:208144|ATP-dependent RNA helicase DHX37/DHR1 [EC:3.6.4.13]	gi|42600571|ref|NP_976064.1|	0	probable ATP-dependent RNA helicase DHX37 [Mus musculus]
mmu-miR-1193-5p	NM_201245	-21.1	1	-20.39	153	GO:0043232,	-	gi|41152239|ref|NP_957697.1|	0	myosin phosphatase Rho-interacting protein isoform 1 [Mus musculus]

mmu-miR-1193-5p	NR_045902	-22.8	0.969454	-21.6	152	GO:0044421,GO:0051259,	K01227|1|8e-27|122|acs:100563201|mannosyl-glycoprotein endo-beta-N-acetylglucosaminidase [EC:3.2.1.96]	gi|148702723|gb|EDL34670.1|	8.37E-57	mCG146303 [Mus musculus]
mmu-miR-1193-5p	NM_177643	-24.1	0.997072	-22.22	151	GO:0043231,GO:0046914,GO:0001071,GO:0043565,GO:0006357,	K09228|1|4e-28|128|bfo:BRAFLDRAFT_71028|KRAB domain-containing zinc finger protein	gi|29244048|ref|NP_808311.1|	0	zinc finger protein 281 [Mus musculus]
mmu-miR-1193-5p	NR_030670	-21.2	0.999999	-20.29	150	GO:0005840,GO:0043231,GO:0005488,	-	gi|23956152|ref|NP_080154.1|	5.33E-46	28S ribosomal protein S18b, mitochondrial [Mus musculus]
mmu-miR-1193-5p	NR_046060	-25.2	0.7726	-23.51	149	-	-	gi|148705533|gb|EDL37480.1|	8.45E-57	mCG2552, isoform CRA_b [Mus musculus]
mmu-miR-1193-5p	NR_073406	-20.5	1	-21.23	148	-	-	-	-	-

mmu-miR-1193-5p	NM_183168	-22.6	0.988135	-20.77	147	GO:0016021,GO:0044459,GO:0015065,GO:0000166,GO:0007186,GO:0006821,GO:0014909,	K04272|1|0.0|635|mmu:233571|pyrimidinergic receptor P2Y, G protein-coupled, 6	gi|34147203|ref|NP_898991.1|	2.49E-180	P2Y purinoceptor 6 [Mus musculus]
mmu-miR-1193-5p	NR_040369	-24.5	0.790215	-20.41	146	-	-	-	-	-
mmu-miR-1193-5p	NM_172438	-22.5	0.995326	-21.74	145	GO:0005856,GO:0031981,GO:0000346,GO:0003676,GO:0006396,GO:0035162,GO:0002762,GO:0006406,	K13174|1|0.0|1342|mmu:107829|THO complex subunit 5	gi|27369587|ref|NP_766026.1|	0	THO complex subunit 5 homolog [Mus musculus]
mmu-miR-1193-5p	NM_145950	-24.3	0.942164	-20.54	144	GO:0022403,GO:0003006,	-	gi|227496929|ref|NP_666062.3|	0	oxidative stress induced growth inhibitor family member 2 [Mus musculus]

mmu-miR-1193-5p	NM_198612	-21.2	0.999658	-22.73	143	GO:0031224,GO:0008194,GO:0006493,	K13676|1|0.0|786|mmu:232313|UDP-xylose:glucoside alpha-1,3-xylosyltransferase [EC:2.4.2.-]	gi|38348470|ref|NP_941014.1|	0	glucoside xylosyltransferase 2 precursor [Mus musculus]
mmu-miR-1193-5p	NM_080795	-21.8	0.999998	-20.97	142	GO:0046914,GO:0019904,GO:0051259,	K10692|1|0.0|1338|mmu:140887|ligand of Numb protein X	gi|165932387|ref|NP_542985.4|	0	ligand of Numb protein X 2 [Mus musculus]
mmu-miR-1193-5p	NM_009849	-21.4	0.999545	-22.71	141	GO:0031224,GO:0005604,GO:0016462,GO:0032559,GO:0001775,GO:0009137,GO:00071	K01509|1|0.0|920|mmu:12496|adenosinetriphosphatase [EC:3.6.1.3]	gi|161484610|ref|NP_033979.2|	0	ectonucleoside triphosphate diphosphohydrolase 2 precursor [Mus musculus]
mmu-miR-1193-5p	NM_029937	-20.3	1	-20.14	140	GO:0031224,	K14314|1|0.0|3706|mmu:77595|nuclear pore complex protein Nup210	gi|254675162|ref|NP_084213.1|	0	nuclear pore membrane glycoprotein 210-like precursor [Mus musculus]

mmu-miR-1193-5p	NM_011652	NA	NA	-21.55	165	-	K12567|1|0.0|4.370e+04|mmu:22138|titin [EC:2.7.11.	gi|77812697|ref|NP_035782.3|	0	titin isoform N2-A [Mus musculus]
mmu-miR-1193-5p	NM_009067	NA	NA	-20.48	144	GO:0016020,GO:0044444,GO:0005100,GO:0017048,GO:0042623,GO:0000278,GO:0042221,GO:0008285,GO:0000279,GO:0016192,GO:0032321,	K08773|1|0.0|947|mmu:19765|RalA-binding protein 1	gi|148747257|ref|NP_033093.2|	0	ralA-binding protein 1 [Mus musculus]
mmu-miR-1193-5p	NM_152822	NA	NA	-21.08	143	GO:0035097,GO:0005488,	K02331|1|6e-60|233|xla:734350|DNA polymerase phi subunit [EC:2.7.7.7]	gi|71979675|ref|NP_690035.2|	0	ribosomal biogenesis protein LAS1L [Mus musculus]

mmu-miR-1193-5p	NM_008275	NA	NA	-20.56	141	GO:0043231,GO:0001071,GO:0003677,GO:0060688,GO:0003002,GO:0006357,GO:0046546,GO:0060442,GO:0008283,GO:0030326,GO:0003401,	K09298|1|6e-150|532|mmu:15433|homeobox protein HoxA/C/D13	gi|120586994|ref|NP_032301.2|	4.04E-149	homeobox protein Hox-D13 [Mus musculus]
mmu-miR-1193-5p	NM_030153	-23.1	0.991927	NA	NA	GO:0016020,GO:0031981,GO:0005488,GO:0033002,	-	gi|172072615|ref|NP_084429.2|	0	N-alpha-acetyltransferase 35, NatC auxiliary subunit [Mus musculus]
mmu-miR-1193-5p	NM_172569	-23.5	0.996004	NA	NA	GO:0043231,GO:0046914,	K13056|1|2e-06|56.2|xtr:549576|CCCH zinc finger protein	gi|148702600|gb|EDL34547.1|	0	zinc finger CCCH type containing 5, isoform CRA_b [Mus musculus]
mmu-miR-1194	NM_001170912	-28.7	0.743223	-26.03	176	GO:0044427,GO:0031981,GO:0016234,GO:0046914,GO:0042802,GO:0006355,	K12032|1|0.0|2504|mmu:330627|tripartite motif-containing protein	gi|284004881|ref|NP_001164383.1|	0	tripartite motif-containing protein 66 isoform 1 [Mus musculus]

mmu-miR-1194	NM_001170913	-28.7	0.745464	-26.03	176	GO:0044427,GO:0031981,GO:0016234,GO:0046914,GO:0042802,GO:0006355,	K12032|1|0.0|2318|mmu:330627|tripartite motif-containing protein	gi|284004881|ref|NP_001164383.1|	0	tripartite motif-containing protein 66 isoform 1 [Mus musculus]
mmu-miR-1194	NM_019506	-28.3	0.396979	-26.45	154	GO:0044421,GO:0005102,GO:0001934,GO:0009888,GO:0007178,GO:0006357,GO:0045597,GO:0001935,GO:0001503,GO:0010596,GO:0045765,GO:0016049,GO:0006260,GO:00	K05503|1|0.0|842|mmu:12165|bone morphogenetic protein 9/10	gi|83582807|ref|NP_062379.3|	0	growth/differentiation factor 2 precursor [Mus musculus]
mmu-miR-1194	NM_181853	-28.7	0.740958	-26.03	176	GO:0044427,GO:0031981,GO:0016234,GO:0046914,GO:0042802,GO:0006355,	K12032|1|0.0|2440|mmu:330627|tripartite motif-containing protein	gi|284004881|ref|NP_001164383.1|	0	tripartite motif-containing protein 66 isoform 1 [Mus musculus]
mmu-miR-1194	NR_045746	-25.4	0.291248	-21.86	179	GO:0043231,GO:0043232,GO:0019899,	-	gi|74137735|dbj|BAE24054.1|	3.36E-40	unnamed protein product [Mus musculus]

mmu-miR-1194	NM_010583	-30.4	0.263087	-24.33	172	GO:0044444,GO:0016020,GO:0043169,GO:0004713,GO:0032559,GO:0006952,GO:0046632,GO:0050851,GO:0001816,GO:00064	K07363|1|0.0|1284|mmu:16428|IL2-inducible T-cell kinase [EC:2.7.10.2]	gi|148701875|gb|EDL33822.1|	0	IL2-inducible T-cell kinase, isoform CRA_b [Mus musculus]
mmu-miR-1194	NR_033537	-22.5	0.975123	-20.72	171	-	-	gi|74147959|dbj|BAE22327.1|	7.48E-58	unnamed protein product [Mus musculus]
mmu-miR-1194	NM_178908	-27.1	0.381254	-22.4	170	GO:0031224,	-	gi|26351867|dbj|BAC39570.1|	0	unnamed protein product [Mus musculus]
mmu-miR-1194	NM_001170847	-23.5	0.999778	-20.84	169	GO:0043231,GO:0046914,GO:0003676,	K13213|1|5e-21|105|acs:100567568|matrin	gi|283046745|ref|NP_001164318.1|	0	probable RNA-binding protein 20 [Mus musculus]
mmu-miR-1194	NM_011771	-28.1	0.541092	-23.88	168	GO:0043231,GO:0046914,GO:0003676,GO:0001071,GO:0046983,GO:0009888,GO:00063	K09220|1|0.0|1055|mmu:22780|IKAROS family zinc finger protein	gi|113374152|ref|NP_035901.1|	0	zinc finger protein Aiolos [Mus musculus]

mmu-miR-1194	NM_009702	-25.1	0.964326	-22.07	167	GO:0005681,GO:0003676,GO:0000377,	K12874|1|0.0|2767|mmu:11834|intron-binding protein aquarius	gi|28972277|dbj|BAC65592.1|	0	mKIAA0560 protein [Mus musculus]
mmu-miR-1194	NM_134249	-22.7	0.997706	-20.71	166	GO:0044421,GO:0030139,GO:0016020,GO:0004871,GO:0006909,GO:0009636,GO:0031667,GO:0023052,	K15148|1|6e-21|103|mdo:100029969|mediator of RNA polymerase II transcription subunit 7	gi|238624134|ref|NP_599010.4|	2.36E-138	T-cell immunoglobulin and mucin domain-containing protein 2 precursor [Mus musculus]
mmu-miR-1194	NM_009828	-24.6	0.913951	-21	165	GO:0031981,GO:0045120,GO:0019900,GO:0031572,GO:0006355,GO:0043627,GO:0043434,GO:0008284,GO:0007264,GO:0009887,GO:0000087,	K06627|1|0.0|845|mmu:12428|cyclin A	gi|161353444|ref|NP_033958.2|	0	cyclin-A2 [Mus musculus]

mmu-miR-1194	NM_017467	-20.6	1	-24.67	164	GO:0044464,GO:0046914,GO:0003676,GO:0006351,	K09228|1|0.0|1679|mmu:54201|KRAB domain-containing zinc finger protein	gi|255708390|ref|NP_059495.3|	0	zinc finger protein 316 [Mus musculus]
mmu-miR-1194	NR_045865	-24.9	0.553273	-21.16	163	-	-	gi|74203638|dbj|BAE23075.1|	2.99E-97	unnamed protein product [Mus musculus]
mmu-miR-1194	NR_045643	-23.3	0.999693	-22.24	162	-	-	gi|74206245|dbj|BAE24883.1|	8.43E-55	unnamed protein product [Mus musculus]
mmu-miR-1194	NM_175688	-24.8	0.889392	-20.83	161	-	K08429|1|1e-31|139|mgp:100548093|G protein-coupled receptor 141	gi|254692882|ref|NP_783619.2|	4.94E-127	G protein-coupled receptor-like [Mus musculus]
mmu-miR-1194	NM_178643	-27	0.395571	-23.93	160	GO:0043234,GO:0031981,GO:0003676,GO:0006259,	K10898|1|2e-120|433|mmu:101831|fanconi anemia-associate	gi|30519993|ref|NP_848758.1|	1.32E-119	Fanconi anemia-associated protein of 24 kDa [Mus musculus]
mmu-miR-1194	NR_033508	-27.1	0.213293	-23.46	159	-	-	gi|148706290|gb|EDL38237.1|	9.33E-52	mCG145591 [Mus musculus]
mmu-miR-1194	NR_033261	-24.9	0.816664	-20.45	158	-	-	gi|148686210|gb|EDL18157.1|	3.94E-180	mCG145277 [Mus musculus]

mmu-miR-1194	NM_173002	-26.1	0.842508	-21.9	156	GO:0043231,GO:0046914,GO:0019904,GO:0001071,GO:0006351,	K09228|1|1e-40|170|mgp:100547559|KRAB domain-containing zinc finger protein	gi|148666869|gb|EDK99285.1|	0	ZXD family zinc finger C, isoform CRA_b [Mus musculus]
mmu-miR-1194	NM_153398	-24.8	0.895937	-23.96	155	GO:0043231,GO:0046914,GO:0003676,GO:0001071,GO:0006351,	K10503|1|0.0|1415|mmu:268294|zinc finger and BTB domain-containing protein 24	gi|31542230|ref|NP_700447.2|	0	zinc finger and BTB domain-containing protein 24 [Mus musculus]

mmu-miR-1194	NM_027667	-20	1	-20.32	154	GO:0043231,GO:0044444,GO:0008047,GO:0035556,GO:0043087,GO:0009966,	K10360|1|4e-12|75.5|ecb:100069995|myosin IX!K12490|5|1e-11|73.9|oaa:100090045|Arf-GAP, Rho-GAP domain, ANK repeat and PH domain-containing protein	gi|148709914|gb|EDL41860.1|	0	Rho GTPase activating protein 19, isoform CRA_a [Mus musculus]
mmu-miR-1194	NR_030701	-27.4	0.730747	-21.46	153	-	-	gi|332825664|ref|XP_003311673.1|	1.30E-06	PREDICTED: hypothetical protein LOC100611452 [Pan troglodytes]
mmu-miR-1194	NM_173779	-20.6	0.999828	-20.14	152	-	-	gi|30424651|ref|NP_776140.1|	4.07E-162	ankyrin repeat domain-containing protein SOWAHD [Mus musculus]
mmu-miR-1194	NM_203490	-26.9	0.628555	-22.56	151	GO:0031224,GO:0004888,GO:0007166,	K08392|1|8e-174|612|mmu:211578|Mas-related G protein-coupled receptor member D	gi|45237187|ref|NP_987075.1|	5.25E-173	mas-related G-protein coupled receptor member D [Mus musculus]

mmu-miR-1194	NM_198101	-24.7	0.960638	-20.16	149	GO:0044444,GO:0005099,GO:0043169,GO:0032319,	K04450|1|1e-29|133|ptr:739083|activating transcription factor 2!K02649|2|2e-27|126|bfo:BRAFLDRAFT_91712|phosphoinositide-3-kinase, regulatory subunit!K10360|3|1e-26|123|dre:560956|	gi|148696806|gb|EDL28753.1|	0	Gem-interacting protein, isoform CRA_a [Mus musculus]
mmu-miR-1194	NM_178017	-25.8	0.865186	-22.54	147	GO:0031010,GO:0003676,GO:0007041,GO:0016055,	K11298|1|0.0|873|mmu:70823|high mobility group protein 2-like 1	gi|148678874|gb|EDL10821.1|	0	high mobility group box 2-like 1, isoform CRA_a [Mus musculus]

mmu-miR-1194	NM_028481	-22.8	0.999793	-20.97	146	-	K11498|1|4e-45|185|mcc:711186|centromeric protein E!K10421|5|8e-45|184|dan:Dana_GF14794|CAP-Gly domain-containing linker protein 1	gi|58037313|ref|NP_082757.1|	0	coiled-coil domain-containing protein 18 [Mus musculus]
mmu-miR-1194	NM_172606	-21.4	1	NA	NA	GO:0031227,GO:0046914,GO:0019899,GO:0019787,GO:0000209,	K10661|1|0.0|1566|mmu:223455|E3 ubiquitin-protein ligase MARCH6 [EC:6.3.2.19]	gi|71043942|ref|NP_766194.2|	0	E3 ubiquitin-protein ligase MARCH6 [Mus musculus]
mmu-miR-1194	NM_001164575	-22	0.99998	NA	NA	GO:0043231,GO:0043169,GO:0001071,GO:0006351,	K09228|1|0.0|1564|mmu:67911|KRAB domain-containing zinc finger protein	gi|257153346|ref|NP_080726.3|	0	GLI-Kruppel family member HKR1 isoform a [Mus musculus]

mmu-miR-1194	NM_029001	-22.1	0.999997	NA	NA	GO:0031224,GO:0043231,GO:0031090,GO:0004312,GO:0005488,GO:0000038,GO:0035336,GO:0006641,GO:0019368,	K10250|1|4e-170|600|mmu:74559|elongation of very long chain fatty acids protein 7	gi|148540000|ref|NP_083277.3|	2.87E-169	elongation of very long chain fatty acids protein 7 [Mus musculus]
mmu-miR-1194	NM_007602	-20.8	1	NA	NA	GO:0044464,GO:0004197,GO:0030154,GO:0019538,GO:0022602,GO:0023052,	K08574|1|0.0|1299|mmu:12337|calpain-5 [EC:3.4.22.-]	gi|6680846|ref|NP_031628.1|	0	calpain-5 [Mus musculus]
mmu-miR-1194	NM_009149	-26.7	0.739278	NA	NA	GO:0031224,GO:0031985,GO:0012505,GO:0044421,GO:0017134,GO:0030246,GO:0002062,GO:0007179,GO:0009653,GO:00150	K06816|1|0.0|2144|mmu:20340|golgi apparatus protein 1	gi|6677905|ref|NP_033175.1|	0	Golgi apparatus protein 1 precursor [Mus musculus]

mmu-miR-1194	NM_144851	-22.5	0.999996	NA	NA	GO:0043231,GO:0019783,GO:0070646,GO:0006917,GO:0032501,	K08592|1|0.0|1150|mmu:223870|sentrin-specific protease 1 [EC:3.4.22.-]	gi|148672263|gb|EDL04210.1|	0	SUMO1/sentrin specific peptidase 1, isoform CRA_b [Mus musculus]
mmu-miR-1194	NM_010580	-22.4	0.9994	NA	NA	GO:0005924,GO:0005887,GO:0004871,GO:0005102,GO:0031589,GO:0007166,GO:00030	K06588|1|0.0|1523|mmu:16419|integrin beta 5	gi|225007617|ref|NP_034710.2|	0	integrin beta-5 isoform 2 precursor [Mus musculus]
mmu-miR-1194	NM_080444	-20.3	0.999999	NA	NA	GO:0007165,	K10332|1|0.0|917|mmu:117590|ankyrin repeat and SOCS box protein	gi|17978258|ref|NP_536692.1|	0	ankyrin repeat and SOCS box protein 10 [Mus musculus]
mmu-miR-1194	NM_201608	-21.7	0.994678	NA	NA	GO:0031224,	K06751|1|0.0|645|mmu:110696|major histocompatibility complex, class I	gi|148691346|gb|EDL23293.1|	0	mCG145379 [Mus musculus]

mmu-miR-1194	NM_175491	-20.8	1	NA	NA	-	K03165|1|3e-08|62.0|ecb:100051666|DNA topoisomerase III [EC:5.99.1.2]	gi|146198710|ref|NP_001078909.1|	0	Smith-Magenis syndrome chromosomal region candidate gene 8 protein homolog isoform 1 [Mus musculus]
mmu-miR-1194	NM_029181	-20.3	0.991606	NA	NA	-	-	gi|256000801|ref|NP_083457.1|	1.28E-69	Slx-like 1 [Mus musculus]
mmu-miR-1194	NM_177378	-20.3	1	NA	NA	GO:0031224,GO:0046914,	K15701|1|9e-94|347|mdo:100032093|E3 ubiquitin-protein ligase RNF130 [EC:6.3.2.19]	gi|90991708|ref|NP_796352.2|	0	RING finger protein 150 precursor [Mus musculus]
mmu-miR-1194	NM_001161535	-27.1	0.701309	NA	NA	GO:0031224,GO:0005488,GO:0048731,GO:0030307,	K06838|1|1e-21|106|cfa:486818|slit 1	gi|238859603|ref|NP_001155007.1|	0	immunoglobulin superfamily containing leucine-rich repeat protein 2 isoform a [Mus musculus]
mmu-miR-1194	NM_145425	-21.1	0.999964	NA	NA	GO:0044459,GO:0005930,GO:0044444,GO:0048858,GO:0008104,GO:0008360,GO:00070	-	gi|31981772|ref|NP_663400.2|	0	WD repeat-containing and planar cell polarity effector protein fritz homolog [Mus musculus]

mmu-miR-1194	NR_038081	-23.2	0.991948	NA	NA	GO:0005488,	K11219|1|0.0|780|cfa:476714|non-receptor tyrosine kinase [EC:2.7.10.2]!K13219|2|2e-11|72.8|ame:726178|splicing factor, proline- and glutamine-rich!K13214|3|2e-10|69.3|xla:380427|non-POU domain-containing octamer-binding protein	gi|12836469|dbj|BAB23670.1|	0	unnamed protein product [Mus musculus]
mmu-miR-1194	NM_027759	-23.5	0.906994	NA	NA	GO:0005488,	-	gi|21313244|ref|NP_082035.1|	0	fibrous sheath-interacting protein 1 [Mus musculus]

mmu-miR-1194	NM_015733	-21.5	0.999997	NA	NA	GO:0043231,GO:0044444,GO:0005488,GO:0004175,GO:0012502,GO:0043065,GO:0010038,GO:0043627,GO:0009416,GO:0033554,GO:0019538,GO:0002237,GO:00325	K04399|1|0.0|894|mmu:12371|caspase 9 [EC:3.4.22.62]	gi|31560479|ref|NP_056548.2|	0	caspase-9 [Mus musculus]
mmu-miR-1194	NM_133797	-20.3	1	NA	NA	-	-	gi|50510837|dbj|BAD32404.1|	0	mKIAA1191 protein [Mus musculus]
mmu-miR-1194	NM_153402	-21.4	1	NA	NA	GO:0044444,GO:0030529,GO:0008135,GO:0006412,GO:0006401,GO:0035195,	K11593|1|0.0|1737|mmu:214150|eukaryotic translation initiation	gi|240120065|ref|NP_700451.2|	0	protein argonaute-3 [Mus musculus]

mmu-miR-1194	NM_001043354	-22.1	1	NA	NA	GO:0031981,GO:0046914,GO:0004930,GO:0004879,GO:0001071,GO:0003677,GO:0007166,GO:0048511,GO:0007417,GO:0009755,GO:0050953,GO:0001754,	K08533|1|0.0|873|mmu:225998|RAR-related orphan receptor beta	gi|349096|gb|AAA42095.1|	0	transcription factor, partial [Rattus norvegicus]
mmu-miR-1194	NM_008856	-21.1	0.999998	NA	NA	GO:0044444,GO:0016020,GO:0043169,GO:0005515,GO:0004674,GO:0032559,GO:0007599,GO:0001775,GO:0006464,GO:0007165,GO:0006796,	K06068|1|0.0|1415|mmu:18755|novel protein kinase C [EC:2.7.11.13]	gi|148704556|gb|EDL36503.1|	0	protein kinase C, eta [Mus musculus]
mmu-miR-1194	NR_015499	-21.1	0.999997	NA	NA	-	-	gi|148673237|gb|EDL05184.1|	6.91E-24	mCG1028558, isoform CRA_a [Mus musculus]

mmu-miR-1194	NM_008420	-25.3	0.998014	NA	NA	GO:0032589,GO:0043025,GO:0005887,GO:0005249,GO:0005515,GO:0006461,GO:0030073,GO:0015980,GO:0034220,GO:0030001,	K04885|1|0.0|1605|mmu:16500|potassium voltage-gated channel Shab-related subfamily B member 1	gi|31560819|ref|NP_032446.2|	0	potassium voltage-gated channel subfamily B member 1 [Mus musculus]
mmu-miR-1194	NM_146001	-21	1	NA	NA	GO:0030117,GO:0031224,GO:0043232,GO:0030136,GO:0005198,GO:0005543,GO:0008092,GO:0006351,GO:0006901,GO:0006915,GO:0012502,GO:0048869,GO:00458	K04559|1|0.0|1919|mmu:215114|huntingtin interacting protein 1	gi|225007582|ref|NP_666113.2|	0	huntingtin-interacting protein 1 [Mus musculus]

mmu-miR-1194	NM_001146005	-21.7	0.999972	NA	NA	GO:0031224,GO:0004942,GO:0007166,GO:0042221,	K04171|1|9e-179|628|mmu:319430|G protein-coupled receptor 77	gi|225579148|ref|NP_001139477.1|	5.96E-178	C5a anaphylatoxin chemotactic receptor C5L2 isoform 2 [Mus musculus]
mmu-miR-1194	NM_021402	-22	0.999914	NA	NA	GO:0031090,GO:0043231,GO:0031224,GO:0019787,GO:0032559,GO:0006464,GO:0006950,	K04554|1|3e-138|493|mmu:140499|ubiquitin-conjugating enzyme E2 J2 [EC:6.3.2.19]	gi|148683112|gb|EDL15059.1|	4.22E-150	ubiquitin-conjugating enzyme E2, J2 homolog (yeast), isoform CRA_c [Mus musculus]
mmu-miR-1194	NM_001166497	-20.9	1	NA	NA	GO:0046872,	K09228|1|0.0|881|rno:499056|KRAB domain-containing zinc finger protein	gi|262073018|ref|NP_001159969.1|	0	zinc finger protein 51-like [Mus musculus]

mmu-miR-1194	NR_045545	-20.2	1	NA	NA	-	K08883|1|1e-16|90.9|bfo:BRAFLDRAFT_69357|tripartite motif-containing protein 33 [EC:6.3.2.19]!K08881|3|8e-16|87.8|mmu:21848|tripartite motif-containing protein 24	gi|357576270|ref|NP_001239513.1|	0	protein kinase C-binding protein 1 isoform 2 [Mus musculus]
mmu-miR-1194	NM_001177567	-22.3	0.999999	NA	NA	-	K03900|1|1e-179|632|xtr:100492314|von Willebrand factor	gi|294774587|ref|NP_001171038.1|	0	otogelin-like precursor [Mus musculus]

mmu-miR-1194	NM_019626	-23	0.984766	NA	NA	GO:0044459,GO:0042802,GO:0048731,GO:0007267,	K03986|1|8e-08|60.1|ecb:100058097|complement C1q subcomponent subunit A!K03988|2|5e-07|57.4|ecb:100058056|complement C1q subcomponent subunit C!K03987|3|1e-06|56.2|hsa:713|complement C1q subcomponent subunit	gi|291410219|ref|XP_002721388.1|	1.04E-98	PREDICTED: cerebellin 1-like [Oryctolagus cuniculus]
mmu-miR-1194	NM_011316	-22.7	0.98569	NA	NA	GO:0005576,GO:0002526,	-	gi|6755398|ref|NP_035446.1|	1.10E-72	serum amyloid A-4 protein precursor [Mus musculus]

mmu-miR-1194	NM_173874	-22.2	0.999998	NA	NA	GO:0031224,GO:0030133,GO:0044459,GO:0005770,GO:0005769,GO:0012505,GO:0042802,GO:0015291,GO:0046983,GO:0019904,GO:0005254,GO:0032559,GO:0051452,GO:0015698,GO:0034220,GO:00432	K05012|1|0.0|1567|mdo:100021050|chloride channel 3	gi|189458826|ref|NP_776299.1|	0	H(+)/Cl(-) exchange transporter 3 isoform e [Mus musculus]
mmu-miR-1194	NM_133911	-20	1	NA	NA	GO:0009986,GO:0031224,GO:0004888,GO:0007186,	K08462|1|0.0|2421|mmu:70693|G protein-coupled receptor 125	gi|30851397|gb|AAH52391.1|	0	Gpr125 protein, partial [Mus musculus]
mmu-miR-1194	NM_212445	-20.1	1	NA	NA	GO:0044432,	K13667|1|3e-33|145|cin:100184131|protein glucosyltransferase [EC:2.4.1.-]	gi|47078285|ref|NP_997610.1|	0	KDEL (Lys-Asp-Glu-Leu) containing 2 protein precursor [Mus musculus]

mmu-miR-1194	NM_001163608	-20	1	NA	NA	GO:0043296,GO:0043005,GO:0031224,GO:0044297,GO:0044424,GO:0048646,	-	gi|148684158|gb|EDL16105.1|	0	plexin domain containing 1, isoform CRA_a [Mus musculus]
mmu-miR-1194	NM_001113199	-23.2	0.999955	NA	NA	GO:0043231,GO:0003676,GO:0003712,GO:0008134,GO:0006351,GO:0006810,	K03626|1|0.0|3384|mmu:17938|nascent polypeptide-associated complex subunit alpha	gi|163965357|ref|NP_001106670.1|	0	nascent polypeptide-associated complex subunit alpha isoform a [Mus musculus]
mmu-miR-1194	NM_134115	-24.5	0.948667	NA	NA	GO:0035097,GO:0004672,GO:0046872,GO:0019901,GO:0032559,GO:0035556,GO:00434	K08790|1|0.0|949|mmu:106504|serine/threonine kinase 38 [EC:2.7.11.1]	gi|148690654|gb|EDL22601.1|	0	serine/threonine kinase 38, isoform CRA_a [Mus musculus]

mmu-miR-1194	NM_017405	-21.3	0.999783	NA	NA	GO:0034358,GO:0043231,GO:0016020,GO:0071813,GO:0008289,GO:0004888,GO:0006629,GO:0048513,GO:0015031,	-	gi|1236083|gb|AAA92719.1|	0	Lisch7, partial [Mus musculus]
mmu-miR-1194	NM_018771	-22.6	0.970493	NA	NA	GO:0030425,GO:0012505,GO:0030136,GO:0008047,GO:0008092,GO:0019904,GO:0042802,GO:0043087,GO:0023061,GO:0007179,GO:0006886,GO:0031330,GO:0010608,GO:0050804,GO:0016477,	-	gi|9055336|ref|NP_061241.1|	9.45E-162	PDZ domain-containing protein GIPC1 [Mus musculus]
mmu-miR-1194	NR_045070	-21.2	0.984957	NA	NA	GO:0044421,GO:0044424,GO:0008235,GO:0009888,GO:0044237,	-	gi|148676914|gb|EDL08861.1|	4.50E-51	mCG58383 [Mus musculus]

mmu-miR-1194	NM_001003672	-20.2	1	NA	NA	GO:0016021,GO:0046872,GO:0004871,GO:0048731,GO:0016337,	K04600|1|2e-71|272|tad:TRIADDRAFT_62266|cadherin EGF LAG seven-pass G-type receptor 1 (flamingo)	gi|51092283|ref|NP_001003672.1|	0	protocadherin alpha-C2 precursor [Mus musculus]
mmu-miR-1194	NM_172831	-22.6	0.986956	NA	NA	-	-	gi|26352630|dbj|BAC39945.1|	0	unnamed protein product [Mus musculus]
mmu-miR-1194	NM_015794	-20.2	1	NA	NA	-	K10283|1|0.0|1050|mmu:50758|F-box and leucine-rich repeat protein	gi|255683359|ref|NP_056609.1|	0	F-box/LRR-repeat protein 17 [Mus musculus]
mmu-miR-1194	NM_001161665	-21	1	NA	NA	GO:0015630,GO:0003774,GO:0032559,GO:0007017,	K10404|1|0.0|3446|mmu:269152|kinesin family member 26	gi|239582737|ref|NP_001155137.1|	0	kinesin-like protein KIF26B [Mus musculus]

mmu-miR-1194	NM_029633	-23.9	0.998036	NA	NA	GO:0044431,GO:0000776,GO:0005876,GO:0008017,GO:0031127,GO:0048870,GO:0007067,GO:0006935,GO:0007019,	-	gi|126506304|ref|NP_083909.2|	0	CLIP-associating protein 2 isoform a [Mus musculus]
mmu-miR-1194	NM_001080931	-20.6	1	NA	NA	GO:0043234,GO:0031981,GO:0004871,GO:0008134,GO:0003713,GO:0006357,GO:0030518,GO:0006352,	K15164|1|0.0|3992|mmu:327987|mediator of RNA polymerase II transcription subunit	gi|124286862|ref|NP_001074400.1|	0	mediator of RNA polymerase II transcription subunit 13 [Mus musculus]
mmu-miR-1194	NM_145962	-25.4	0.985426	NA	NA	GO:0031981,GO:0016301,GO:0032559,GO:0009108,GO:0006796,	K09680|1|0.0|701|ptr:462250|type II pantothenate kinase [EC:2.7.1.33]	gi|74200242|dbj|BAE22924.1|	0	unnamed protein product [Mus musculus]

mmu-miR-1194	NM_027060	-23.9	0.999362	NA	NA	GO:0009987,	K10481|1|0.0|1196|mmu:224671|BTB/POZ domain-containing protein 9	gi|27819604|ref|NP_766206.1|	0	BTB/POZ domain-containing protein 9 [Mus musculus]
mmu-miR-1194	NM_001130000	-20.4	1	NA	NA	GO:0005813,GO:0032991,GO:0042995,GO:0019900,GO:0007098,GO:0033036,	K12478|1|1e-30|137|mgp:100545401|early endosome antigen 1	gi|148674210|gb|EDL06157.1|	0	mCG5221 [Mus musculus]
mmu-miR-1194	NM_183216	-20.3	1	NA	NA	GO:0031227,GO:0046914,GO:0016215,GO:0010872,GO:0042742,GO:0045444,GO:0006631,GO:0008366,GO:0044126,	K00507|1|0.0|741|mmu:329065|stearoyl-CoA desaturase (delta-9 desaturase) [EC:1.14.19.1]	gi|38261958|ref|NP_899039.2|	0	stearoyl-CoA desaturase 4 [Mus musculus]

mmu-miR-1194	NM_010649	-21.1	0.985119	NA	NA	GO:0016020,GO:0004871,GO:0023052,	K10069|1|2e-14|80.1|mmu:243653|C-type lectin domain family 1, member A!K10075|4|4e-11|69.3|ssc:100144592|killer cell lectin-like receptor subfamily F, member 1	gi|6754462|ref|NP_034779.1|	1.71E-153	killer cell lectin-like receptor 4 isoform 1 [Mus musculus]
mmu-miR-1194	NM_146717	-24.4	0.582832	NA	NA	GO:0004888,GO:0023052,	K04257|1|8e-164|576|mmu:258712|olfactory	gi|238018106|ref|NP_666928.2|	5.66E-163	olfactory receptor 433 [Mus musculus]
mmu-miR-1194	NM_008339	-21.9	0.974927	NA	NA	GO:0019814,GO:0016021,GO:0031981,GO:0009986,GO:0005488,GO:0050851,	K06507|1|5e-113|408|mmu:15985|CD79B antigen	gi|123242277|emb|CAM23794.1|	7.27E-121	CD79B antigen [Mus musculus]

mmu-miR-1194	NM_144887	-21.1	1	NA	NA	-	K05766|1|1e-58|230|spu:575378|slingshot	gi|50511087|dbj|BAD32529.1|	0	mKIAA1748 protein [Mus musculus]
mmu-miR-1194	NR_003617	-21.7	0.999967	NA	NA	GO:0044464,GO:0005488,GO:0006952,GO:0009116,	-	gi|12839801|dbj|BAB24674.1|	5.30E-54	unnamed protein product [Mus musculus]
mmu-miR-1194	NM_011373	-23.9	0.993533	NA	NA	GO:0031228,GO:0008373,GO:0006643,GO:0006464,	K03374|1|3e-159|563|rno:407764|N-acetylgalactosaminide alpha-2,6-sialyltransferase (sialyltransferase 7D) [EC:2.4.99.7]	gi|21759447|sp|Q9R2B6.1|SIA7D_MOUSE	1.54E-169	RecName: Full=Alpha-N-acetyl-neuraminyl-2,3-beta-galactosyl-1,3-N-acetyl-galactosaminide alpha-2,6-sialyltransferase; AltName: Full=NeuAc-alpha-2,3-Gal-beta-1,3-GalNAc-alpha-2,6-sialyltransferase; AltName: Full=ST6GalNAc IV; Short=ST6GalNAcIV; AltName: Full=Sialyltransferase 7D; Short=SIAT7-D

mmu-miR-1194	NM_001037759	-22.8	0.999928	NA	NA	GO:0005768,GO:0031410,GO:0004672,GO:0005543,GO:0032559,GO:0050896,GO:0043066,GO:0006464,	K13304|1|0.0|1018|mmu:170755|serum/glucocorticoid-regulated kinase 3 [EC:2.7.11.1]	gi|18959280|ref|NP_573483.1|	0	serine/threonine-protein kinase Sgk3 [Mus musculus]
mmu-miR-1194	NM_001159967	-22.2	1	NA	NA	GO:0016020,GO:0044424,GO:0005488,GO:0030695,GO:0019222,GO:0035556,	K04349|1|2e-31|140|rno:192213|Ras-specific guanine nucleotide-releasing factor 1	gi|148707424|gb|EDL39371.1|	0	Ral GEF with PH domain and SH3 binding motif 2, isoform CRA_b [Mus musculus]
mmu-miR-1194	NM_007945	-20.8	1	NA	NA	GO:0008328,GO:0044430,GO:0030674,GO:0007169,GO:0007626,GO:0030036,GO:00305	K07365|1|1e-07|60.5|api:100161543|NCK adaptor protein	gi|74196170|dbj|BAE32995.1|	0	unnamed protein product [Mus musculus]

mmu-miR-1194	NM_001243064	-21.3	0.999954	NA	NA	GO:0032991,GO:0012505,GO:0042995,GO:0001669,GO:0016021,GO:0005924,GO:0000149,GO:0008047,GO:0019899,GO:0033483,GO:0045185,GO:0001935,GO:0030177,GO:0006639,GO:0042506,GO:0019229,GO:0043405,GO:0048732,GO:0014706,GO:0055092,GO:0042391,GO:00075	K06278|1|9e-96|352|mmu:12389|caveolin 1	gi|6680854|ref|NP_031642.1|	6.13E-95	caveolin-1 isoform 1 [Mus musculus]
mmu-miR-1194	NM_177306	-21.9	0.999846	NA	NA	GO:0043231,GO:0043565,GO:0031016,GO:0030154,GO:0030073,GO:0006351,GO:00335	K09174|1|5e-91|337|oaa:100077122|regulatory factor X 4	gi|226874821|ref|NP_001152861.1|	0	DNA-binding protein RFX6 isoform 1 [Mus musculus]

mmu-miR-1194	NM_025675	-20.3	1	NA	NA	-	-	gi|13385136|ref|NP_079951.1|	1.30E-132	ATP-binding domain-containing protein 4 [Mus musculus]
mmu-miR-1194	NM_133788	-22.3	0.999983	NA	NA	GO:0031224,GO:0043231,GO:0031090,GO:0003713,GO:0003880,GO:0006575,GO:0043009,GO:0040007,GO:0006790,GO:0006479,GO:0006605,GO:0048513,GO:0051056,GO:00	K00587|1|3e-144|514|mmu:57295|protein-S-isoprenylcysteine O-methyltransferase [EC:2.1.1.100]	gi|51317397|ref|NP_598549.1|	2.44E-143	protein-S-isoprenylcysteine O-methyltransferase [Mus musculus]
mmu-miR-1194	NM_144515	-20.2	1	NA	NA	GO:0005622,GO:0046914,GO:0006351,	K09228|1|0.0|1573|mmu:22710|KRAB domain-containing zinc finger protein	gi|122937377|ref|NP_653098.2|	0	zinc finger protein 52 [Mus musculus]

mmu-miR-1194	NM_007378	-28.2	0.735818	NA	NA	GO:0016021,GO:0017111,GO:0005548,GO:0015405,GO:0032559,GO:0050953,GO:0007602,GO:0009207,GO:0015914,GO:00439	K05644|1|0.0|4566|mmu:11304|ATP-binding cassette, subfamily A (ABC1), member 4	gi|6671495|ref|NP_031404.1|	0	retinal-specific ATP-binding cassette transporter [Mus musculus]
mmu-miR-1194	NM_026172	-20.4	1	NA	NA	GO:0031981,GO:0044429,GO:0016020,GO:0016628,GO:0050661,GO:0051260,GO:0009062,	K13236|1|0.0|671|mmu:67460|2,4-dienoyl-CoA reductase, mitochondrial [EC:1.3.1.34]	gi|13385680|ref|NP_080448.1|	0	2,4-dienoyl-CoA reductase, mitochondrial precursor [Mus musculus]

mmu-miR-1194	NM_145506	-20.2	1	NA	NA	GO:0001726,GO:0005924,GO:0043232,GO:0005515,GO:0071363,GO:0001840,GO:0010631,GO:0001837,GO:0009952,GO:0043393,GO:0003382,GO:0010468,GO:0007162,GO:0006929,GO:0030036,GO:0032880,GO:0001954,GO:0000578,GO:00017	K06107|1|1e-81|306|tgu:100218952|erythrocyte membrane protein band 4.1	gi|148707867|gb|EDL39814.1|	0	erythrocyte protein band 4.1-like 5, isoform CRA_b [Mus musculus]

mmu-miR-1194	NM_011374	-21.3	1	NA	NA	GO:0031228,GO:0008373,GO:0006687,GO:0009408,GO:0008283,GO:0006464,	K03371|1|0.0|739|mmu:20449|alpha-N-acetyl-neuraminate alpha-2,8-sialyltransferase (sialyltransferase 8A) [EC:2.4.99.	gi|70980543|ref|NP_035504.2|	0	alpha-N-acetylneuraminide alpha-2,8-sialyltransferase [Mus musculus]
mmu-miR-1194	NM_001005423	-20.8	0.999993	NA	NA	GO:0044459,GO:0050931,GO:0022405,	-	gi|53749212|ref|NP_001005423.1|	3.99E-96	melanoregulin [Mus musculus]
mmu-miR-1194	NM_008442	-20.9	1	NA	NA	GO:0000323,GO:0005819,GO:0003774,GO:0032559,GO:0048869,GO:0048731,GO:0007017,GO:0007067,GO:0007599,	K10393|1|0.0|1271|rno:84391|kinesin family member 2/24	gi|149059280|gb|EDM10287.1|	0	rCG44775, isoform CRA_a [Rattus norvegicus]

mmu-miR-1194	NM_020032	-22.6	0.993221	NA	NA	GO:0043231,GO:0043169,GO:0003676,GO:0034061,GO:0002566,GO:0006281,	K03512|1|0.0|1087|mmu:56626|DNA polymerase lambda subunit [EC:2.7.7.74.2.99.-]	gi|9910500|ref|NP_064416.1|	0	DNA polymerase lambda [Mus musculus]
mmu-miR-1195	NM_001001454	-45.1	0.001835	-41.79	190	GO:0030175,GO:0043005,GO:0005789,GO:0034702,GO:0005253,GO:0046872,GO:0022890,GO:0030035,GO:0000087,GO:0007155,GO:0015698,GO:0006826,	-	gi|148699268|gb|EDL31215.1|	0	tweety homolog 1 (Drosophila), isoform CRA_c [Mus musculus]
mmu-miR-1195	NM_001004150	-48.1	0.000197	-45.31	197	GO:0031228,GO:0035250,GO:0006687,GO:0016044,	K01988|1|0.0|690|mmu:239559|lactosylceramide 4-alpha-galactosyltransferase [EC:2.4.1.228]	gi|51921295|ref|NP_001004150.1|	0	lactosylceramide 4-alpha-galactosyltransferase [Mus musculus]

mmu-miR-1195	NM_001012324	-41.1	0.008116	-38.82	189	GO:0005578,GO:0005518,GO:0005539,GO:0005102,GO:0043062,GO:0010810,	K08119|1|0.0|1226|mmu:407800|extracellular matrix protein 2	gi|148709136|gb|EDL41082.1|	0	extracellular matrix protein 2, female organ and adipocyte specific, isoform CRA_b [Mus musculus]
mmu-miR-1195	NM_001013750	-31.1	0.280157	-25.42	157	-	-	gi|85719316|ref|NP_001013772.1|	0	uncharacterized protein LOC210962 [Mus musculus]
mmu-miR-1195	NM_001025103	-28.1	0.462408	-38.82	189	-	-	gi|29124550|gb|AAH48916.1|	8.71E-142	Efcab4a protein, partial [Mus musculus]
mmu-miR-1195	NM_001025313	-48.1	0.000319	-45.31	197	GO:0042824,GO:0046977,GO:0015440,GO:0003823,GO:0042287,GO:0002396,GO:0042590,GO:0048193,GO:0050822,GO:00069	K08058|1|0.0|749|mmu:21356|TAP binding protein (tapasin)	gi|148678278|gb|EDL10225.1|	0	TAP binding protein, isoform CRA_a [Mus musculus]
mmu-miR-1195	NM_001025589	-48.1	0.000519	-45.31	197	GO:0043231,GO:0000989,GO:0003676,GO:0001071,GO:0019899,GO:0006357,GO:0007264,	K08062|1|2e-137|491|mmu:19727|regulatory factor X-associated ankyrin-	gi|148696839|gb|EDL28786.1|	1.34E-151	regulatory factor X-associated ankyrin-containing protein, isoform CRA_b [Mus musculus]

mmu-miR-1195	NM_001029912	-25.6	0.994747	-26.49	163	GO:0046914,	-	gi|28972770|dbj|BAC65801.1|	0	mKIAA1511 protein [Mus musculus]
mmu-miR-1195	NM_001033128	-27.6	0.921679	-45.31	197	GO:0031253,GO:0043234,GO:0044424,GO:0005488,GO:0042384,GO:0001894,GO:00439	K01277|1|0.0|978|aml:100484390|dipeptidyl-peptidase III [EC:3.4.14.4]	gi|148701131|gb|EDL33078.1|	0	mCG8379 [Mus musculus]
mmu-miR-1195	NM_001033178	-28.3	0.472613	-26.17	146	GO:0031224,GO:0005488,GO:0051704,	-	gi|75677514|ref|NP_001028350.1|	0	transmembrane protein 181 [Mus musculus]
mmu-miR-1195	NM_001033339	-48.1	0.000454	-45.31	197	GO:0031012,GO:0031224,GO:0004175,GO:0008047,GO:0046914,GO:0019538,GO:0006952,	K08003|1|0.0|1073|mmu:240047|matrix metalloproteinase-25 (membrane-inserted) [EC:3.4.24.-]	gi|75677470|ref|NP_001028511.1|	0	matrix metalloproteinase-25 precursor [Mus musculus]
mmu-miR-1195	NM_001033778	-36.5	0.018378	-34.46	175	-	-	gi|76253875|ref|NP_001028950.1|	8.70E-68	uncharacterized protein LOC433481 [Mus musculus]

mmu-miR-1195	NM_001033966	-31.1	0.118159	-29.87	181	GO:0019866,GO:0005740,GO:0019205,GO:0016776,GO:0032559,GO:0006144,GO:0009117,GO:0006796,	K00939|1|8e-134|476|mmu:11637|adenylate kinase [EC:2.7.4.3]	gi|77020262|ref|NP_001029138.1|	5.52E-133	adenylate kinase 2, mitochondrial isoform a [Mus musculus]
mmu-miR-1195	NM_001033979	-25.5	0.868202	-45.31	197	-	-	gi|109475699|ref|XP_578500.2|	8.58E-74	PREDICTED: low-density lipoprotein receptor class A domain-containing protein 2-like [Rattus norvegicus]

mmu-miR-1195	NM_001035226	-48.1	0.00084	-45.31	197	GO:0032991,GO:0042175,GO:0016604,GO:0000775,GO:0044444,GO:0005515,GO:0003676,GO:0022892,GO:0042221,GO:0050658,GO:0006357,GO:0006605,GO:0009894,GO:0033157,GO:0007067,GO:0022415,GO:0051704,GO:0033365,GO:00466	K14290|1|0.0|2142|mmu:103573|exportin-1	gi|238537882|pdb|3GJX|A	0	Chain A, Crystal Structure Of The Nuclear Export Complex Crm1-Snurportin1-Rangtp
mmu-miR-1195	NM_001037166	-48.1	0.000111	-45.31	197	GO:0015935,GO:0005198,GO:0006412,GO:0019080,GO:0031016,	K02951|1|3e-71|270|mmu:237433|small subunit ribosomal protein S12e	gi|81230474|ref|NP_001032243.1|	2.12E-70	ribosomal protein S12-like [Mus musculus]

mmu-miR-1195	NM_001038637	-25.8	0.969381	-45.31	197	GO:0043231,GO:0031234,GO:0017111,GO:0005488,GO:0060089,GO:0001775,GO:0007267,GO:0033762,GO:0009207,GO:0015980,GO:0007599,GO:0007166,	K07826|1|3e-34|148|cfa:610817|guanine nucleotide-binding protein G(I)/G(S)/G(O) subunit gamma-2	gi|148669586|gb|EDL01533.1|	2.73E-35	mCG144516 [Mus musculus]
mmu-miR-1195	NM_001039200	-30.8	0.563712	-45.31	197	GO:0031224,GO:0005488,	-	gi|85861260|ref|NP_001034289.1|	0	ER membrane protein complex subunit 1 isoform 2 precursor [Mus musculus]
mmu-miR-1195	NM_001040026	-25.3	0.992126	-45.31	197	GO:0043231,GO:0019866,GO:0046914,GO:0000041,GO:0019725,GO:0043623,	K07152|1|2e-138|494|mmu:52892|	gi|74228413|dbj|BAE24044.1|	1.71E-137	unnamed protein product [Mus musculus]
mmu-miR-1195	NM_001045482	-48.1	0.000447	-45.31	197	GO:0043231,GO:0046914,GO:0006351,	K09228|1|0.0|1530|mmu:57782|KRAB domain-containing zinc finger protein	gi|148687122|gb|EDL19069.1|	0	RB-associated KRAB repressor, isoform CRA_a [Mus musculus]

mmu-miR-1195	NM_001076791	-25.4	0.984908	-43.46	197	GO:0043169,	K09228|1|0.0|962|mmu:408067|KRAB domain-containing zinc finger protein	gi|115647917|ref|NP_001070259.1|	0	regulator of sex-limitation candidate 16 [Mus musculus]
mmu-miR-1195	NM_001081021	-48.1	0.001008	-45.31	197	GO:0005622,GO:0046872,GO:0003676,	K09228|1|0.0|1735|mmu:338354|KRAB domain-containing zinc finger protein	gi|124486618|ref|NP_001074490.1|	0	zinc finger protein 780B [Mus musculus]
mmu-miR-1195	NM_001081022	-33.5	0.257864	-30.2	155	-	-	gi|124486628|ref|NP_001074491.1|	0	uncharacterized protein KIAA0556 [Mus musculus]
mmu-miR-1195	NM_001081087	-48.1	0.000276	-45.31	197	GO:0005815,GO:0031252,GO:0016020,GO:0031981,GO:0042995,GO:0005488,GO:0031271,GO:0006936,	K10473|1|0.0|1185|mmu:228003|kelch repeat and BTB domain-containing protein 5/10	gi|124487329|ref|NP_001074556.1|	0	kelch repeat and BTB domain-containing protein 10 [Mus musculus]

mmu-miR-1195	NM_001081242	-48.1	0.003199	-45.31	197	GO:0005856,GO:0005924,GO:0031252,GO:0008092,GO:0005102,GO:0005198,GO:0034329,GO:0007010,	K06271|1|0.0|4323|mmu:70549|talin	gi|163310736|ref|NP_001074711.2|	0	talin-2 [Mus musculus]
mmu-miR-1195	NM_001081387	-34.2	0.102183	-45.31	197	GO:0043231,GO:0046914,GO:0043565,GO:0005515,GO:0006351,GO:0006306,GO:0031060,	K09228|1|7e-63|243|mmu:630579|KRAB domain-containing zinc finger protein	gi|124487069|ref|NP_001074856.1|	0	transcriptional repressor CTCFL [Mus musculus]
mmu-miR-1195	NM_001085371	-30.9	0.348967	-26.59	166	GO:0043231,GO:0030154,GO:0008283,GO:0048513,	K08809|1|0.0|1429|rno:363256|striated muscle-specific serine/threonine protein kinase [EC:2.7.11.1]	gi|145699119|ref|NP_001078840.1|	0	striated muscle-specific serine/threonine-protein kinase isoform 3 [Mus musculus]
mmu-miR-1195	NM_001085373	-45.7	0.004199	-43.15	193	-	-	gi|145699114|ref|NP_001078842.1|	0	mutated in colorectal cancers isoform 1 [Mus musculus]

mmu-miR-1195	NM_001101471	-47.8	0.001517	-45.31	197	GO:0044463,GO:0044459,GO:0044444,GO:0019899,GO:0051049,GO:0010737,GO:0015980,GO:0030073,GO:0048522,GO:0006171,GO:0032501,	K04573|1|2e-16|90.5|xla:397995|neurofilament medium polypeptide (neurofilament 3)!K00907|2|4e-16|89.4|dvi:Dvir_GJ20094|myosin-light-chain kinase [EC:2.7.11.18]	gi|158187517|ref|NP_001094941.1|	0	A-kinase anchor protein 5 [Mus musculus]
mmu-miR-1195	NM_001109765	-45.1	0.001516	-41.79	190	GO:0030175,GO:0043005,GO:0005789,GO:0034702,GO:0005253,GO:0046872,GO:0030035,GO:0000087,GO:0007155,GO:00156	-	gi|148699270|gb|EDL31217.1|	0	tweety homolog 1 (Drosophila), isoform CRA_e [Mus musculus]

mmu-miR-1195	NM_001113401	-32.6	0.101148	-29.93	156	GO:0016604,GO:0005488,GO:0012502,GO:0016049,GO:0050680,GO:0006355,	K15186|1|4e-116|419|mmu:106389|ELL-associated factor	gi|164663793|ref|NP_001106872.1|	2.75E-115	ELL-associated factor 2 isoform b [Mus musculus]
mmu-miR-1195	NM_001113553	-48.1	0.000385	-45.31	197	GO:0031090,GO:0044444,GO:0016301,GO:0046983,GO:0042802,GO:0032559,GO:0051091,GO:0002224,GO:0043433,GO:0019221,GO:0006468,GO:0007249,GO:0006915,GO:00	K04731|1|0.0|1164|mmu:108960|interleukin-1 receptor-associated kinase 2 [EC:2.7.11.1]	gi|148667078|gb|EDK99494.1|	0	mCG132967, isoform CRA_b [Mus musculus]
mmu-miR-1195	NM_001114879	-48.1	0.001549	-45.31	197	-	-	gi|168823452|ref|NP_001108351.1|	0	protein FAM208A isoform 1 [Mus musculus]

mmu-miR-1195	NM_001115130	-48.1	0.002061	-45.31	197	GO:0043231,GO:0046914,GO:0003676,	K10515|1|0.0|876|mmu:235132|zinc finger and BTB domain-containing protein 44	gi|169646141|ref|NP_001108602.1|	0	zinc finger and BTB domain-containing protein 44 isoform a [Mus musculus]
mmu-miR-1195	NM_001130150	-26.4	0.92522	-28.64	161	GO:0016020,GO:0044444,GO:0005088,GO:0008047,GO:0005488,GO:0043087,GO:0007266,GO:0031346,GO:0010769,GO:0007169,GO:0031345,	K12330|1|0.0|1680|mmu:16801|Rho guanine nucleotide exchange factor 1	gi|148692356|gb|EDL24303.1|	0	Rho guanine nucleotide exchange factor (GEF) 1, isoform CRA_b [Mus musculus]

mmu-miR-1195	NM_001130151	-26.4	0.924749	-28.64	161	GO:0016020,GO:0044444,GO:0005088,GO:0008047,GO:0005488,GO:0043087,GO:0007266,GO:0031346,GO:0010769,GO:0007169,GO:0031345,	K12330|1|0.0|1659|mmu:16801|Rho guanine nucleotide exchange factor 1	gi|148692356|gb|EDL24303.1|	0	Rho guanine nucleotide exchange factor (GEF) 1, isoform CRA_b [Mus musculus]
mmu-miR-1195	NM_001130152	-26.4	0.915854	-28.64	161	GO:0016020,GO:0044444,GO:0005088,GO:0008047,GO:0005488,GO:0043087,GO:0007266,GO:0031346,GO:0010769,GO:0007169,GO:0031345,	K12330|1|0.0|1545|rno:60323|Rho guanine nucleotide exchange factor 1	gi|148692357|gb|EDL24304.1|	0	Rho guanine nucleotide exchange factor (GEF) 1, isoform CRA_c [Mus musculus]

mmu-miR-1195	NM_001130153	-26.4	0.915317	-28.64	161	GO:0016020,GO:0044444,GO:0005088,GO:0008047,GO:0005488,GO:0043087,GO:0007266,GO:0031346,GO:0010769,GO:0007169,GO:0031345,	K12330|1|0.0|1545|rno:60323|Rho guanine nucleotide exchange factor 1	gi|148692358|gb|EDL24305.1|	0	Rho guanine nucleotide exchange factor (GEF) 1, isoform CRA_d [Mus musculus]
mmu-miR-1195	NM_001134767	-32.6	0.225766	-31.85	171	GO:0043231,	K11498|1|7e-10|67.8|ecb:100068227|centromeric protein E!K11499|3|2e-09|66.2|spu:592172|centromere protein F!K10421|5|6e-09|64.7|phu:Phum_PHUM291650|CAP-Gly domain-containing linker protein 1	gi|198041652|ref|NP_001128239.1|	0	coiled-coil domain-containing protein 62 [Mus musculus]

mmu-miR-1195	NM_001142968	-27.3	0.754108	-43.46	197	GO:0046872,GO:0022402,	K10382|1|3e-09|65.1|ptr:462793|dystonin	gi|148697583|gb|EDL29530.1|	0	cDNA sequence BC024139, isoform CRA_c [Mus musculus]
mmu-miR-1195	NM_001146005	-48.1	0.000466	-71.22	352	GO:0031224,GO:0004942,GO:0007166,GO:0042221,	K04171|1|9e-179|628|mmu:319430|G protein-coupled receptor 77	gi|225579148|ref|NP_001139477.1|	5.96E-178	C5a anaphylatoxin chemotactic receptor C5L2 isoform 2 [Mus musculus]
mmu-miR-1195	NM_001159324	-48.1	0.000522	-45.31	197	GO:0005764,GO:0030246,GO:0004558,GO:0003011,GO:0008016,GO:0005984,GO:0007033,GO:0051651,GO:0005977,GO:0007610,GO:0055008,GO:0019725,GO:0019318,GO:00	K12316|1|0.0|1883|mmu:14387|lysosomal alpha-glucosidase [EC:3.2.1.20]	gi|226693367|ref|NP_032090.3|	0	lysosomal alpha-glucosidase precursor [Mus musculus]

mmu-miR-1195	NM_001159609	-27.3	0.631823	-32.28	166	-	K10130|1|5e-16|87.0|xtr:100488788|leucine-rich repeats and death domain-containing protein!K01768|3|3e-15|84.3|dpo:Dpse_GA10197|adenylate cyclase [EC:4.6.1.1]	gi|148696062|gb|EDL28009.1|	4.57E-150	leucine rich repeat containing 57, isoform CRA_d [Mus musculus]

mmu-miR-1195	NM_001159610	-27.3	0.642133	-32.28	166	-	K10130|1|5e-16|87.0|xtr:100488788|leucine-rich repeats and death domain-containing protein!K01768|3|3e-15|84.3|dpo:Dpse_GA10197|adenylate cyclase [EC:4.6.1.1]	gi|148696062|gb|EDL28009.1|	1.13E-151	leucine rich repeat containing 57, isoform CRA_d [Mus musculus]

mmu-miR-1195	NM_001159612	-27.3	0.645302	-32.28	166	-	K10130|1|5e-16|87.0|xtr:100488788|leucine-rich repeats and death domain-containing protein!K01768|3|3e-15|84.3|dpo:Dpse_GA10197|adenylate cyclase [EC:4.6.1.1]	gi|12849514|dbj|BAB28373.1|	4.32E-151	unnamed protein product [Mus musculus]
mmu-miR-1195	NM_001159646	-34.6	0.057033	-31.61	181	GO:0031981,GO:0044444,GO:0043169,GO:0019103,GO:0030545,GO:0047429,GO:0005102,GO:0006259,GO:0006213,GO:0009177,GO:0009213,GO:0009112,GO:00104	K01520|1|3e-77|290|mmu:110074|dUTP pyrophosphatase [EC:3.6.1.23]	gi|21281687|ref|NP_076084.2|	2.40E-76	deoxyuridine triphosphatase isoform 2 [Mus musculus]

mmu-miR-1195	NM_001159850	-25.8	0.961882	-25.07	154	GO:0005887,GO:0005249,GO:0042391,GO:0034220,GO:0007166,GO:0030001,	K04913|1|0.0|817|mmu:16526|potassium channel subfamily K member	gi|229577199|ref|NP_001153322.1|	0	potassium channel subfamily K member 2 isoform 1 [Mus musculus]
mmu-miR-1195	NM_001160386	-25	0.999996	-26.35	141	GO:0030286,GO:0005930,GO:0003774,GO:0032559,GO:0048870,GO:0007017,	K10408|1|0.0|3943|tgu:100230839|dynein heavy chain, axonemal	gi|237874196|ref|NP_001153858.1|	0	dynein, axonemal, heavy chain 7B [Mus musculus]
mmu-miR-1195	NM_001161329	-43	0.003038	-40.67	189	GO:0031981,	-	gi|238550126|ref|NP_001154801.1|	0	nucleolar protein 11 isoform 2 [Mus musculus]
mmu-miR-1195	NM_001161413	-48.1	0.000332	-45.31	197	GO:0031224,GO:0044459,GO:0048770,GO:0015081,GO:0015171,GO:0043167,GO:0015801,GO:0015820,GO:0044238,GO:0040007,GO:0002376,GO:0006807,GO:0007599,GO:00	K06519|1|0.0|1010|mmu:17254|solute carrier family 3, member 2	gi|238637277|ref|NP_001154885.1|	0	4F2 cell-surface antigen heavy chain isoform a [Mus musculus]

mmu-miR-1195	NM_001162489	-48.1	0.000896	-45.31	197	-	-	gi|28972856|dbj|BAC65844.1|	0	mKIAA1833 protein [Mus musculus]
mmu-miR-1195	NM_001162932	-34.5	0.108407	-45.31	197	GO:0043231,GO:0003676,GO:0006259,	K15365|1|3e-66|254|mmu:223970|RecQ-mediated genome instability protein 2	gi|242397460|ref|NP_001156404.1|	1.97E-65	recQ-mediated genome instability protein 2 [Mus musculus]
mmu-miR-1195	NM_001162965	-48.1	0.00056	-45.31	197	GO:0043231,GO:0005099,GO:0032313,	K10657|1|7e-07|57.8|mmu:224703|E3 ubiquitin-protein ligase MARCH2 [EC:6.3.2.19]!K13136|2|1e-06|57.0|nvi:100114269|gem associated protein 8	gi|244790047|ref|NP_001156437.1|	0	small G protein signaling modulator 1 isoform b [Mus musculus]

mmu-miR-1195	NM_001162970	-27.5	0.897464	-26.16	150	-	K01886|1|3e-08|62.8|tgu:100226583|glutaminyl-tRNA synthetase [EC:6.1.1.1	gi|244790065|ref|NP_001156442.1|	0	absent in melanoma 1-like protein [Mus musculus]
mmu-miR-1195	NM_001163430	-28.3	0.430461	-38.27	187	GO:0000784,GO:0051287,GO:0016763,GO:0046914,GO:0017136,GO:0016458,GO:0035601,GO:0006351,	K11416|1|1e-171|603|mmu:50721|mono-ADP-ribosyltransferase sirtuin 6 [EC:2.4.2.31]	gi|31712018|ref|NP_853617.1|	0	NAD-dependent protein deacetylase sirtuin-6 isoform 1 [Mus musculus]
mmu-miR-1195	NM_001163564	-35.2	0.063795	-91.1	624	GO:0044452,GO:0030529,GO:0016866,GO:0003723,GO:0042254,GO:0001522,	K14763|1|0.0|655|mmu:234344|H/ACA ribonucleoprotein complex non-core subunit NAF1	gi|254675189|ref|NP_001157036.1|	0	H/ACA ribonucleoprotein complex non-core subunit NAF1 [Mus musculus]

mmu-miR-1195	NM_001163684	-30.8	0.222512	-45.31	197	GO:0044444,GO:0043231,GO:0005488,GO:0006807,GO:0032769,	K13125|1|1e-157|557|mmu:66394|nitric oxide synthase-interacting protein	gi|254826728|ref|NP_001157156.1|	9.38E-157	nitric oxide synthase-interacting protein isoform 2 [Mus musculus]
mmu-miR-1195	NM_001163734	-48.1	0.000729	-45.31	197	GO:0044444,GO:0030684,GO:0031981,GO:0005488,GO:0042254,	K14849|1|0.0|1087|mmu:72462|ribosomal RNA-processing protein 1	gi|37359820|dbj|BAC97888.1|	0	mKIAA0179 protein [Mus musculus]
mmu-miR-1195	NM_001163815	-30.3	0.483395	-27.24	159	-	K05730|1|0.0|1644|mmu:22324|vav oncogene	gi|255308867|ref|NP_001157287.1|	0	proto-oncogene vav isoform 2 [Mus musculus]
mmu-miR-1195	NM_001163816	-30.3	0.479501	-27.24	159	-	K05730|1|0.0|1536|mmu:22324|vav oncogene	gi|255308869|ref|NP_001157288.1|	0	proto-oncogene vav isoform 3 [Mus musculus]

mmu-miR-1195	NM_001164229	-47.4	0.000397	-45.25	197	GO:0043231,GO:0043234,GO:0001071,GO:0006996,GO:0001101,GO:0043627,GO:0009628,GO:0006355,GO:0009887,GO:00022	K11831|1|0.0|656|gga:416677|nuclear respiratory factor 1	gi|255982551|ref|NP_001157701.1|	0	nuclear respiratory factor 1 isoform d [Mus musculus]
mmu-miR-1195	NM_001164230	-47.4	0.000377	-45.25	197	GO:0043231,GO:0043234,GO:0001071,GO:0006996,GO:0001101,GO:0043627,GO:0009628,GO:0006355,GO:0009887,GO:00022	K11831|1|0.0|647|gga:416677|nuclear respiratory factor 1	gi|255982553|ref|NP_001157702.1|	0	nuclear respiratory factor 1 isoform e [Mus musculus]
mmu-miR-1195	NM_001164366	-31.4	0.360611	-26.19	157	GO:0031224,GO:0043169,GO:0042625,GO:0032559,GO:0009206,GO:0006811,GO:0009207,	K13526|1|0.0|2075|mmu:74772|cation-transporting ATPase 13A2 [EC:3.6.3.-	gi|256985108|ref|NP_001157838.1|	0	probable cation-transporting ATPase 13A2 isoform 2 [Mus musculus]

mmu-miR-1195	NM_001164472	-48.1	0.000349	-45.31	197	GO:0031981,GO:0003676,GO:0042254,	K07565|1|8e-72|273|mmu:66164|60S ribosome subunit biogenesis protein NIP7	gi|257153334|ref|NP_001157944.1|	5.27E-71	60S ribosome subunit biogenesis protein NIP7 homolog isoform 2 [Mus musculus]
mmu-miR-1195	NM_001164729	-29.7	0.155668	-26.8	163	GO:0031966,GO:0006810,	-	gi|13384732|ref|NP_079641.1|	3.71E-24	mitochondrial import receptor subunit TOM6 homolog [Mus musculus]
mmu-miR-1195	NM_001164791	-48.1	0.001362	-45.31	197	GO:0043231,GO:0046872,GO:0004702,GO:0032559,GO:0035556,GO:0006464,GO:0006996,GO:0006796,	K04424|1|0.0|796|mmu:65964|sterile alpha motif and leucine zipper containing kinase AZK [EC:2.7.11.25]	gi|258645102|ref|NP_001158263.1|	0	mitogen-activated protein kinase kinase kinase MLT isoform 3 [Mus musculus]
mmu-miR-1195	NM_001166584	-32.4	0.514956	-26.15	168	GO:0043231,GO:0043234,GO:0003676,GO:0001071,GO:0035556,GO:0006357,GO:00485	K09448|1|0.0|805|mmu:21676|TEA domain family member 1/3/4	gi|395816134|ref|XP_003781566.1|	0	PREDICTED: transcriptional enhancer factor TEF-1 [Otolemur garnettii]

mmu-miR-1195	NM_001166585	-32.4	0.498426	-26.15	168	GO:0043231,GO:0043234,GO:0003676,GO:0001071,GO:0035556,GO:0006357,GO:0048513,GO:0008283,	K09448|1|0.0|801|mdo:100030213|TEA domain family member 1/3/4	gi|344280874|ref|XP_003412207.1|	0	PREDICTED: transcriptional enhancer factor TEF-1 [Loxodonta africana]
mmu-miR-1195	NM_001166645	-41.6	0.012348	-38.32	189	GO:0016020,GO:0005488,GO:0009408,GO:0032496,	K09228|1|2e-172|608|ptr:468733|KRAB domain-containing zinc finger protein	gi|262263349|ref|NP_001160117.1|	0	zinc finger protein 882 [Mus musculus]
mmu-miR-1195	NM_001168600	-45.6	0.002155	-88.77	394	GO:0031981,GO:0032561,GO:0022613,	K14538|1|0.0|1020|mmu:237107|nuclear GTP-binding protein	gi|148675551|gb|EDL07498.1|	0	guanine nucleotide binding protein-like 3 (nucleolar)-like [Mus musculus]

mmu-miR-1195	NM_001170552	-39.2	0.033054	-61.37	339	GO:0030054,GO:0032991,GO:0043232,GO:0005488,GO:0030695,GO:0001525,GO:0035556,GO:0043393,GO:0019725,GO:0030010,GO:0001935,GO:0010594,GO:0043066,	K04984|1|3e-11|72.8|gga:420180|transient receptor potential cation channel subfamily A member 1!K08848|2|3e-11|72.8|gga:418529|receptor-interacting serine/threonine-protein kinase 4 [EC:2.7.11.1]!K06694|4|6e-11|72.0|mgp:10054	gi|148682673|gb|EDL14620.1|	0	KRIT1, ankyrin repeat containing, isoform CRA_c [Mus musculus]

mmu-miR-1195	NM_001170954	-48.1	0.000204	-45.31	197	GO:0031228,GO:0035250,GO:0006687,GO:0016044,	K01988|1|0.0|690|mmu:239559|lactosylceramide 4-alpha-galactosyltransferase [EC:2.4.1.228]	gi|51921295|ref|NP_001004150.1|	0	lactosylceramide 4-alpha-galactosyltransferase [Mus musculus]
mmu-miR-1195	NM_001170960	-30.5	0.33946	-25.28	159	-	-	gi|283837781|ref|NP_001164431.1|	4.52E-91	DTW domain-containing protein 2 isoform 2 [Mus musculus]
mmu-miR-1195	NM_001177313	-48.1	0.000089	-45.31	197	GO:0000808,GO:0031981,GO:0043565,GO:0016462,GO:0032559,GO:0006259,	K02606|1|2e-72|273|mmu:26428|origin recognition complex subunit 4	gi|148694925|gb|EDL26872.1|	5.69E-72	origin recognition complex, subunit 4-like (S. cerevisiae), isoform CRA_b [Mus musculus]
mmu-miR-1195	NM_001177673	-25.8	0.94146	-42.37	193	GO:0016021,GO:0004888,GO:0007166,GO:0006952,	K08408|1|0.0|729|mmu:238377|G protein-coupled receptor 68	gi|148686940|gb|EDL18887.1|	0	G protein-coupled receptor 68, isoform CRA_a [Mus musculus]

mmu-miR-1195	NM_001177674	-25.8	0.950264	-42.37	193	GO:0016021,GO:0004888,GO:0007166,GO:0006952,	K08408|1|0.0|729|mmu:238377|G protein-coupled receptor 68	gi|148686940|gb|EDL18887.1|	0	G protein-coupled receptor 68, isoform CRA_a [Mus musculus]
mmu-miR-1195	NM_001201569	-33.7	0.215086	-27.38	162	GO:0031224,GO:0042625,GO:0004871,GO:0046872,GO:0005548,GO:0008324,GO:0032559,GO:0009206,GO:0006811,GO:0023052,GO:0006869,GO:0009207,	K01530|1|0.0|2131|mmu:50771|phospholipid-translocating ATPase [EC:3.6.3.1]	gi|320089590|ref|NP_001188498.1|	0	probable phospholipid-transporting ATPase IIB isoform 1 [Mus musculus]
mmu-miR-1195	NM_001243761	-26.3	0.978101	-29.24	153	-	K08060|1|0.0|2005|mmu:12265|class II, major histocompatibility complex, transactivator	gi|344217728|ref|NP_001230690.1|	0	MHC class II transactivator isoform 3 [Mus musculus]

mmu-miR-1195	NM_001252449	-45.4	0.000754	-42.37	193	GO:0032991,GO:0043231,GO:0044444,GO:0004672,GO:0032559,GO:0019899,GO:0006605,GO:0007169,GO:0051259,GO:0022402,GO:0045860,GO:0019217,	K08271|1|0.0|764|mmu:72149|protein kinase LYK5	gi|356995842|ref|NP_001239378.1|	0	STE20-related kinase adapter protein alpha isoform 3 [Mus musculus]
mmu-miR-1195	NM_001253702	-31.2	0.476624	-26.24	159	GO:0031224,	-	gi|358679327|ref|NP_001240631.1|	0	suppressor of tumorigenicity 7 protein-like isoform 2 [Mus musculus]
mmu-miR-1195	NM_001253703	-31.2	0.468189	-26.24	159	GO:0031224,	-	gi|358679329|ref|NP_001240632.1|	0	suppressor of tumorigenicity 7 protein-like isoform 3 [Mus musculus]
mmu-miR-1195	NM_001271797	-48.1	0.000164	-45.31	197	-	-	-	-	-
mmu-miR-1195	NM_007463	-27.8	0.98339	-26.59	166	GO:0043231,GO:0004672,GO:0032559,GO:0030154,GO:0006464,GO:0008283,GO:0048513,	K08809|1|0.0|5477|rno:363256|striated muscle-specific serine/threonine protein kinase [EC:2.7.11.1]	gi|148667988|gb|EDL00405.1|	0	SPEG complex locus, isoform CRA_b [Mus musculus]

mmu-miR-1195	NM_007625	-26.1	0.985163	-27.46	171	GO:0031981,GO:0043232,GO:0003723,GO:0003712,GO:0042393,GO:0006357,GO:0006325,GO:0043066,	K11452|1|0.0|900|mmu:12418|chromobox protein 4	gi|161353508|ref|NP_031651.2|	0	E3 SUMO-protein ligase CBX4 [Mus musculus]
mmu-miR-1195	NM_007866	-25.3	0.925818	-45.31	197	GO:0031224,GO:0005102,GO:0007498,GO:0045686,GO:0043009,GO:0008593,GO:0010720,GO:0045165,GO:0009952,	K06051|1|0.0|1092|mmu:13389|delta	gi|116292184|ref|NP_031892.2|	0	delta-like protein 3 precursor [Mus musculus]

mmu-miR-1195	NM_008064	-48.1	0.000544	-45.31	197	GO:0005764,GO:0030246,GO:0004558,GO:0003011,GO:0008016,GO:0005984,GO:0007033,GO:0051651,GO:0005977,GO:0007610,GO:0055008,GO:0019725,GO:0019318,GO:00	K12316|1|0.0|1883|mmu:14387|lysosomal alpha-glucosidase [EC:3.2.1.20]	gi|226693367|ref|NP_032090.3|	0	lysosomal alpha-glucosidase precursor [Mus musculus]
mmu-miR-1195	NM_008108	-48.1	0.000197	-45.31	197	GO:0044421,GO:0005102,GO:0043009,GO:0048646,GO:0000578,GO:0009888,GO:0001702,GO:0048568,	K05495|1|0.0|706|mmu:14562|growth differentiation factor 1/3	gi|75677587|ref|NP_032134.2|	0	growth/differentiation factor 3 precursor [Mus musculus]

mmu-miR-1195	NM_008252	-28.1	0.642196	-45.31	197	GO:0031981,GO:0032991,GO:0005694,GO:0044444,GO:0044421,GO:0005102,GO:0001071,GO:0043566,GO:0042393,GO:0043565,GO:0001935,GO:0006915,GO:0006284,GO:0048017,GO:0008406,GO:0032069,GO:0002562,GO:0048232,GO:0006266,GO:0000737,GO:0006997,GO:00069	K11295|1|3e-97|357|mmu:97165|high mobility group protein B2	gi|6680229|ref|NP_032278.1|	1.95E-96	high mobility group protein B2 [Mus musculus]

mmu-miR-1195	NM_008488	-26.4	0.915137	-28.64	161	GO:0016020,GO:0044444,GO:0005088,GO:0008047,GO:0005488,GO:0043087,GO:0007266,GO:0031346,GO:0010769,GO:0007169,GO:0031345,	K12330|1|0.0|1549|rno:60323|Rho guanine nucleotide exchange factor 1	gi|148692358|gb|EDL24305.1|		0	Rho guanine nucleotide exchange factor (GEF) 1, isoform CRA_d [Mus musculus]
mmu-miR-1195	NM_008515	-26.6	0.864257	-29.34	159	GO:0043231,GO:0003676,GO:0006351,	K10352|1|2e-13|79.0|api:100167379|myosin heavy chain	gi|6678722|ref|NP_032541.1|		0	leucine-rich repeat flightless-interacting protein 1 isoform 2 [Mus musculus]
mmu-miR-1195	NM_008577	-48.1	0.000283	-45.31	197	GO:0044459,GO:0031224,GO:0048770,GO:0015171,GO:0043167,GO:0015801,GO:0015820,GO:0044238,	K06519|1|0.0|937|mmu:17254|solute carrier family 3, member 2	gi|238637277|ref|NP_001154885.1|		0	4F2 cell-surface antigen heavy chain isoform a [Mus musculus]

mmu-miR-1195	NM_008796	-26.7	0.733874	-36.16	185	GO:0044444,GO:0005548,GO:0005543,GO:0006869,GO:0016125,	K08283|1|5e-11|70.5|bfo:BRAFLDRAFT_118505|Goodpasture-antigen-binding protein kinase [EC:2.7.11.9]	gi|117320552|ref|NP_032822.2|	3.78E-123	phosphatidylcholine transfer protein [Mus musculus]
mmu-miR-1195	NM_008996	-25.8	0.924336	-45.31	197	GO:0043231,GO:0044297,GO:0016020,GO:0017111,GO:0032561,GO:0007265,GO:0009207,GO:0048193,GO:0015031,	K07874|1|2e-116|421|pon:100447267|Ras-related protein Rab-1A	gi|119620329|gb|EAW99923.1|	1.42E-115	RAB1A, member RAS oncogene family, isoform CRA_f [Homo sapiens]
mmu-miR-1195	NM_009054	-32.1	0.285519	-45.31	197	GO:0016604,GO:0016021,GO:0005635,GO:0003676,GO:0046914,GO:0004713,GO:0006357,GO:0051259,GO:0010629,GO:00482	K12009|1|0.0|988|ptr:739369|tripartite motif-containing protein 27	gi|47122750|gb|AAH69924.1|	0	Tripartite motif-containing 27 [Mus musculus]

mmu-miR-1195	NM_009230	-30.4	0.525145	-45.31	197	GO:0031224,GO:0043231,GO:0031090,GO:0032934,GO:0004772,GO:0005504,GO:0055092,GO:0090077,GO:0034434,GO:0030301,GO:0016125,GO:0019915,GO:0034377,GO:00	K00637|1|0.0|1045|mmu:20652|sterol O-acyltransferase [EC:2.3.1.26]	gi|84619697|ref|NP_033256.2|	0	sterol O-acyltransferase 1 [Mus musculus]
mmu-miR-1195	NM_009318	-48.1	0.000318	-45.31	197	GO:0042824,GO:0046977,GO:0015440,GO:0003823,GO:0042287,GO:0002396,GO:0042590,GO:0048193,GO:0050822,GO:00069	K08058|1|0.0|744|mmu:21356|TAP binding protein (tapasin)	gi|148678278|gb|EDL10225.1|	0	TAP binding protein, isoform CRA_a [Mus musculus]

mmu-miR-1195	NM_009346	-32.4	0.512836	-26.15	168	GO:0043231,GO:0043234,GO:0003676,GO:0001071,GO:0035556,GO:0006357,GO:0048513,GO:0008283,	K09448|1|0.0|814|mdo:100030213|TEA domain family member 1/3/4	gi|395816134|ref|XP_003781566.1|	0	PREDICTED: transcriptional enhancer factor TEF-1 [Otolemur garnettii]
mmu-miR-1195	NM_009623	-27.3	0.925077	-46.1	318	GO:0031224,GO:0004016,GO:0032559,GO:0043169,GO:0015980,GO:0007267,GO:0007613,GO:0009124,GO:0033762,GO:0032147,GO:0010863,GO:0007169,GO:0007188,GO:0006164,GO:0042044,	K08048|1|0.0|2338|mmu:11514|adenylate cyclase 8 [EC:4.6.1.1]	gi|124430532|ref|NP_033753.2|	0	adenylate cyclase type 8 [Mus musculus]

mmu-miR-1195	NM_010049	-29	0.756058	-28.29	166	GO:0044444,GO:0016646,GO:0000166,GO:0006575,GO:0006544,GO:0009117,GO:0006766,GO:0009396,GO:0014070,GO:00000	K00287|1|4e-104|381|mmu:13361|dihydrofolate reductase [EC:1.5.1.3]	gi|7106289|ref|NP_034179.1|	2.66E-103	dihydrofolate reductase [Mus musculus]
mmu-miR-1195	NM_010102	-48.1	0.000249	-45.31	197	GO:0016021,GO:0005488,GO:0004930,GO:0010863,GO:0007188,GO:0002376,GO:0051480,	K04293|1|8e-161|560|rno:314649|sphingosine 1-phosphate receptor 4	gi|157823827|ref|NP_001101545.1|	5.28E-160	sphingosine 1-phosphate receptor 4 [Rattus norvegicus]

mmu-miR-1195	NM_009874	-23.5	1	-67.75	351	GO:0005667,GO:0043232,GO:0044444,GO:0003712,GO:0004674,GO:0032559,GO:0035258,GO:0042623,GO:0006357,GO:0006397,GO:0030518,GO:0006352,GO:0006796,GO:0051320,GO:0006353,GO:0051318,GO:0009207,GO:0006289,GO:0051726,GO:0006354,GO:0006464,GO:00459	K02202|1|0.0|702|mmu:12572|cyclin-dependent kinase 7 [EC:2.7.11.22 2.7.11.23]	gi|160333726|ref|NP_034004.2|	0	cyclin-dependent kinase 7 [Mus musculus]

mmu-miR-1197-3p	NM_001009949	-30	0.311166	-27.06	167	GO:0019866,GO:0031224,GO:0043231,GO:0051234,	K15110|1|4e-16|88.6|isc:IscW_ISCW017282|solute carrier family 25 (mitochondrial 2-oxodicarboxylate transporter), member 21	gi|74220685|dbj|BAE33610.1|	0	unnamed protein product [Mus musculus]
mmu-miR-1197-3p	NM_001024145	-26.7	0.6538	-48.75	315	GO:0005764,GO:0001726,GO:0043169,GO:0004623,GO:0006644,GO:0006693,GO:0046677,GO:0014070,GO:0015909,	K01047|1|0.0|1488|rno:691907|phospholipase A2 [EC:3.1.1.4]	gi|67972423|ref|NP_001019316.1|	0	cytosolic phospholipase A2 zeta [Mus musculus]
mmu-miR-1197-3p	NM_001024703	-28.2	0.652703	-25.39	160	GO:0031224,GO:0046872,GO:0005543,GO:0019932,	K15293|1|3e-11|72.8|tad:TRIADDRAFT_22095|protein unc-13	gi|85701536|ref|NP_001019874.1|	0	multiple C2 and transmembrane domain-containing protein 2 [Mus musculus]

mmu-miR-1197-3p	NM_001033276	-31.4	0.618842	-27.73	160	GO:0043231,GO:0034708,GO:0016740,GO:0043565,GO:0008283,GO:0048545,GO:0030520,GO:0007292,GO:0016568,	K09187|1|0.0|4273|mmu:381022|histone-lysine N-methyltransferase MLL2 [EC:2.1.1.43]	gi|359718904|ref|NP_001028448.3|	0	histone-lysine N-methyltransferase MLL2 [Mus musculus]
mmu-miR-1197-3p	NM_001033473	-31.2	0.04175	-26.86	163	-	K05626|1|4e-06|53.1|ssc:100153709|atrophin-1 (dentatorubral-pallidoluysian atrophy protein)	gi|85701995|ref|NP_001028645.1|	7.09E-164	outer dense fiber protein 3-like protein 2 [Mus musculus]
mmu-miR-1197-3p	NM_001033794	-29.7	0.108231	-25.65	171	-	K10267|1|0.0|693|mmu:434440|F-box and WD-40 domain protein 12/13/14/15/16/17/19	gi|85702119|ref|NP_001028966.1|	0	F-box and WD-40 domain protein 18 [Mus musculus]

mmu-miR-1197-3p	NM_001033851	-26.1	0.212767	-45.07	305	-	K04601|1|6e-07|54.7|tgu:100232614|cadherin EGF LAG seven-pass G-type receptor 2 (flamingo)	gi|76563928|ref|NP_001029023.1|	2.41E-54	copine-8 isoform 2 [Mus musculus]
mmu-miR-1197-3p	NM_001039153	-28.2	0.288173	-26.04	169	GO:0044421,GO:0007224,GO:0030900,GO:0043009,GO:0001838,GO:0021522,GO:00219	-	gi|109809735|ref|NP_001034242.2|	0	tectonic-1 precursor [Mus musculus]
mmu-miR-1197-3p	NM_001081022	-29.2	0.525195	-25.23	163	-	-	gi|124486628|ref|NP_001074491.1|	0	uncharacterized protein KIAA0556 [Mus musculus]
mmu-miR-1197-3p	NM_001081158	-27.8	0.686904	-47.61	311	-	K03255|1|0.0|2624|mmu:74148|protein TIF31	gi|124487201|ref|NP_001074627.1|	0	protein KIAA0664 [Mus musculus]

mmu-miR-1197-3p	NM_001081282	-28.9	0.537923	-25.1	157	GO:0031981,GO:0004860,GO:0019900,GO:0006469,GO:0051283,	K10523|1|6e-13|78.6|api:100162530|speckle-type POZ protein!K10595|4|2e-12|77.0|nve:NEMVE_v1g170394|E3 ubiquitin-protein ligase HERC2 [EC:6.3.2.19]	gi|124486857|ref|NP_001074751.1|	0	inhibitor of Bruton tyrosine kinase [Mus musculus]
mmu-miR-1197-3p	NM_001081390	-27.8	0.530123	-25.06	167	GO:0005924,GO:0031252,GO:0043231,GO:0030016,GO:0015629,GO:0051393,GO:00069	K12567|1|5e-55|217|bta:540561|titin [EC:2.7.11.1]	gi|124487061|ref|NP_001074859.1|	0	palladin [Mus musculus]

mmu-miR-1197-3p	NM_001083616	-29.6	0.604789	-27.56	157	GO:0034704,GO:0044459,GO:0005245,GO:0015980,GO:0007611,GO:0032412,GO:0050954,GO:0070838,GO:0030073,GO:0006935,GO:0007187,	K04851|1|0.0|3781|mmu:12289|voltage-dependent calcium channel L type alpha-1D	gi|134288875|ref|NP_001077085.1|	0	voltage-dependent L-type calcium channel subunit alpha-1D isoform 2 [Mus musculus]
mmu-miR-1197-3p	NM_001101433	-27.9	0.592701	-25.91	161	GO:0046914,	-	gi|160333288|ref|NP_001094903.1|	4.62E-130	zinc finger CCHC domain-containing protein 24 [Mus musculus]
mmu-miR-1197-3p	NM_001109975	-26.5	0.812918	-42.96	293	GO:0043296,GO:0030425,GO:0043025,GO:0032432,GO:0008092,GO:0032231,	-	gi|74182827|dbj|BAE34731.1|	0	unnamed protein product [Mus musculus]

mmu-miR-1197-3p	NM_001110192	-28.3	0.576388	-45.22	314	GO:0016020,GO:0015629,GO:0044444,GO:0046030,GO:0016312,GO:0019904,GO:0034594,GO:0002683,GO:0007599,GO:0030853,GO:0019724,GO:0050851,GO:0010259,GO:0006796,GO:0045639,GO:0030888,GO:0006915,GO:0032845,GO:0045577,GO:00	K03084|1|0.0|2163|mmu:16331|phosphatidylinositol-3,4,5-trisphosphate 5-phosphatase 1 [EC:3.1.3.86]	gi|158854001|ref|NP_001103662.1|	0	phosphatidylinositol 3,4,5-trisphosphate 5-phosphatase 1 isoform 2 [Mus musculus]

mmu-miR-1197-3p	NM_001110193	-28.3	0.562404	-45.22	314	GO:0016020,GO:0015629,GO:0044444,GO:0046030,GO:0016312,GO:0019904,GO:0002683,GO:0030853,GO:0019724,GO:0010259,GO:0006796,GO:0045639,GO:0030888,GO:0006915,GO:0007165,GO:0032845,GO:0045577,GO:0042036,	K03084|1|0.0|2045|mmu:16331|phosphatidylinositol-3,4,5-trisphosphate 5-phosphatase 1 [EC:3.1.3.86]	gi|148708208|gb|EDL40155.1|	0	inositol polyphosphate-5-phosphatase D, isoform CRA_g [Mus musculus]
mmu-miR-1197-3p	NM_001113379	-27.9	0.540648	-45.57	303	GO:0016021,GO:0042130,GO:0010467,	K05401|1|3e-30|135|dme:Dmel_CG5195|toll-like receptor 3	gi|164607126|ref|NP_001106850.1|	0	leucine rich repeat containing 32 precursor [Mus musculus]

mmu-miR-1197-3p	NM_001136484	-29.6	0.369515	-25.85	173	GO:0031224,GO:0043231,GO:0012505,GO:0044421,GO:0008375,GO:0009887,GO:0043434,GO:0006486,	K00727|1|0.0|872|mmu:14537|beta-1,3-galactosyl-O-glycosyl-glycoprotein beta-1,6-N-acetylglucosaminyltransferase [EC:2.4.1.1	gi|27734104|ref|NP_775618.1|	0	beta-1,3-galactosyl-O-glycosyl-glycoprotein beta-1,6-N-acetylglucosaminyltransferase [Mus musculus]
mmu-miR-1197-3p	NM_001145957	-30.1	0.277442	-25.65	169	-	K10798|1|3e-54|215|bfo:BRAFLDRAFT_73983|poly [ADP-ribose] polymerase [EC:2.4.2.30]	gi|148693525|gb|EDL25472.1|	0	loss of heterozygosity, 11, chromosomal region 2, gene A homolog (human), isoform CRA_b [Mus musculus]

mmu-miR-1197-3p	NM_001146307	-30.5	0.134531	-25.51	165	GO:0043231,GO:0044459,GO:0034702,GO:0005488,GO:0005254,GO:0010038,GO:0046903,GO:0009628,GO:0006821,GO:0001822,GO:0034220,GO:0043269,GO:00069	K05017|1|0.0|1256|mmu:12733|chloride channel Ka!K05018|3|0.0|1075|mmu:56365|chloride channel Kb	gi|226423866|ref|NP_077723.3|	0	chloride channel protein ClC-Ka [Mus musculus]
mmu-miR-1197-3p	NM_001160214	-33.9	0.071287	-28.72	170	GO:0015630,GO:0043231,GO:0005488,	-	gi|50511247|dbj|BAD32609.1|	0	mKIAA2010 protein [Mus musculus]
mmu-miR-1197-3p	NM_001161515	-28.7	0.24048	-25.6	159	GO:0044444,GO:0046914,GO:0016814,GO:0006213,GO:0009112,GO:0009117,	K01493|1|1e-99|365|mmu:320685|dCMP deaminase [EC:3.5.4.12]	gi|238859570|ref|NP_848903.2|	6.98E-99	deoxycytidylate deaminase [Mus musculus]
mmu-miR-1197-3p	NM_001161516	-28.7	0.213367	-25.6	159	GO:0044444,GO:0046914,GO:0016814,GO:0006213,GO:0009112,GO:0009117,	K01493|1|9e-100|365|mmu:320685|dCMP deaminase [EC:3.5.4.1	gi|238859570|ref|NP_848903.2|	6.10E-99	deoxycytidylate deaminase [Mus musculus]

mmu-miR-1197-3p	NM_001162999	-27.1	0.867268	-25.27	157	GO:0044424,GO:0005488,GO:0006468,	K08020|1|5e-13|79.0|acs:100553829|Rap guanine nucleotide exchange factor (GEF) 6	gi|244790458|ref|NP_001156471.1|	0	folliculin-interacting protein 2 [Mus musculus]
mmu-miR-1197-3p	NM_001164805	-26.8	0.965772	-43.91	316	GO:0031224,	K08624|1|5e-42|176|spu:586332|a disintegrin and metalloproteinase with thrombospondin motifs 9 [EC:3.4.24.-]	gi|50510729|dbj|BAD32350.1|	0	mKIAA0960 protein [Mus musculus]
mmu-miR-1197-3p	NM_001167697	-30.1	0.107557	-27.69	161	GO:0043231,GO:0016021,GO:0004888,GO:0007166,	K04316|1|0.0|805|mmu:14760|G protein-coupled receptor 19	gi|266457701|ref|NP_001161166.1|	0	probable G-protein coupled receptor 19 isoform a [Mus musculus]

mmu-miR-1197-3p	NM_001167994	-31.1	0.153234	-28.07	178	GO:0043231,GO:0008757,GO:0006399,GO:0001510,GO:0010467,	K15331|1|0.0|1015|mmu:215201|tRNA (uracil-5-)-methyltransferase [EC:2.1.1.35]	gi|269784754|ref|NP_766128.2|	0	tRNA (uracil(54)-C(5))-methyltransferase homolog isoform a [Mus musculus]
mmu-miR-1197-3p	NM_001168498	-29.5	0.129552	-25.88	167	-	-	gi|148698035|gb|EDL29982.1|	4.87E-49	RIKEN cDNA A330049M08, isoform CRA_b [Mus musculus]
mmu-miR-1197-3p	NM_001168500	-29.5	0.149131	-25.88	167	-	-	gi|148698035|gb|EDL29982.1|	5.64E-49	RIKEN cDNA A330049M08, isoform CRA_b [Mus musculus]
mmu-miR-1197-3p	NM_001172160	-27.9	0.606528	-25.58	174	GO:0016021,GO:0031012,GO:0004871,GO:0005515,GO:0009987,	K07523|1|3e-30|135|xtr:100188921|netrin-G1 ligand!K06839|2|1e-27|126|dpe:Dper_GL16263|slit 2	gi|37360402|dbj|BAC98179.1|	0	mKIAA1469 protein [Mus musculus]

mmu-miR-1197-3p	NM_001177525	-30.5	0.069638	-26.25	167	-	K09228|1|0.0|553|mmu:668620|KRAB domain-containing zinc finger protein	gi|44890292|gb|AAH66874.1|	0	LOC100045488 protein [Mus musculus]
mmu-miR-1197-3p	NM_001190852	-27.9	0.651148	-27.85	166	GO:0044459,GO:0031674,GO:0044430,GO:0008092,GO:0032947,GO:0046914,GO:0019901,GO:0048814,GO:0044087,GO:00485	K05760|1|3e-40|169|nve:NEMVE_v1g104924|paxillin	gi|300069024|ref|NP_001177781.1|	0	PDZ and LIM domain protein 5 isoform ENH1e [Mus musculus]
mmu-miR-1197-3p	NM_001190854	-27.9	0.620864	-27.85	166	GO:0044459,GO:0031674,GO:0044430,GO:0008092,GO:0046914,GO:0019901,GO:0048814,GO:0044087,	K05760|1|3e-40|169|nve:NEMVE_v1g104924|paxillin	gi|300069036|ref|NP_001177783.1|	0	PDZ and LIM domain protein 5 isoform ENH1b [Mus musculus]

mmu-miR-1197-3p	NM_001190855	-27.9	0.631282	-27.85	166	GO:0044459,GO:0031674,GO:0044430,GO:0008092,GO:0046914,GO:0019901,GO:0048814,GO:0044087,	K05760|1|3e-40|169|nve:NEMVE_v1g104924|paxillin	gi|300069038|ref|NP_001177784.1|	0	PDZ and LIM domain protein 5 isoform ENH1c [Mus musculus]
mmu-miR-1197-3p	NM_001190856	-27.9	0.642274	-27.85	166	GO:0044459,GO:0031674,GO:0044430,GO:0008092,GO:0032947,GO:0046914,GO:0019901,GO:0048814,GO:0044087,GO:00485	K05760|1|3e-40|169|nve:NEMVE_v1g104924|paxillin	gi|300069041|ref|NP_001177785.1|	0	PDZ and LIM domain protein 5 isoform ENH1d [Mus musculus]
mmu-miR-1197-3p	NM_001198866	-25.1	0.944015	-26.2	157	GO:0030286,GO:0000775,GO:0044444,GO:0005819,GO:0003774,GO:0015631,GO:0048731,GO:0000087,	K04648|1|0.0|2055|mmu:13191|dynactin 1	gi|118601017|ref|NP_031861.2|	0	dynactin subunit 1 isoform 1 [Mus musculus]

mmu-miR-1197-3p	NM_001198867	-25.1	0.94126	-26.2	157	GO:0030286,GO:0005819,GO:0044444,GO:0015631,GO:0003774,GO:0009987,	K04648|1|0.0|2041|mmu:13191|dynactin 1	gi|311893360|ref|NP_001185796.1|	0	dynactin subunit 1 isoform 3 [Mus musculus]
mmu-miR-1197-3p	NM_001243306	-29.5	0.165243	-25.88	167	-	-	gi|148698035|gb|EDL29982.1|	6.30E-49	RIKEN cDNA A330049M08, isoform CRA_b [Mus musculus]
mmu-miR-1197-3p	NM_001271019	-25.4	0.764851	-26.54	158	-	-	-	-	-
mmu-miR-1197-3p	NM_001271472	-31.2	0.346103	-27.34	161	-	-	-	-	-
mmu-miR-1197-3p	NM_001271473	-31.2	0.333692	-27.34	161	-	-	-	-	-
mmu-miR-1197-3p	NM_007405	-29.6	0.459339	-25.83	161	-	K08046|1|0.0|2223|mmu:11512|adenylate cyclase 6 [EC:4.6.1.1]	gi|86604721|ref|NP_031431.2|	0	adenylate cyclase type 6 [Mus musculus]
mmu-miR-1197-3p	NM_007835	-25.1	0.950384	-26.2	157	GO:0030286,GO:0000775,GO:0044444,GO:0005819,GO:0003774,GO:0015631,GO:0048731,GO:0000087,	K04648|1|0.0|2055|mmu:13191|dynactin 1	gi|118601017|ref|NP_031861.2|	0	dynactin subunit 1 isoform 1 [Mus musculus]

mmu-miR-1197-3p	NM_008216	-30.8	0.223587	-27.32	157	GO:0016021,GO:0008194,	K00752|1|0.0|1075|mmu:15117|hyaluronan synthase [EC:2.4.1.212]	gi|160358868|ref|NP_032242.3|	0	hyaluronan synthase 2 [Mus musculus]
mmu-miR-1197-3p	NM_008224	-25.6	0.98474	-43.84	307	GO:0035097,GO:0000123,GO:0003712,GO:0001071,GO:0005515,GO:0006461,GO:0031647,GO:0006351,GO:0019042,GO:00073	K14966|1|0.0|2933|mmu:15161|host cell factor	gi|34328130|ref|NP_032250.2|	0	host cell factor 1 [Mus musculus]

mmu-miR-1197-3p	NM_008352	-26.3	0.53113	-48.84	315	GO:0043234,GO:0044424,GO:0044421,GO:0019972,GO:0004888,GO:0005126,GO:0042802,GO:0046983,GO:0050691,GO:0042108,GO:0042506,GO:0002294,GO:0042508,GO:0032609,GO:0001912,GO:0010661,GO:0048870,GO:0031349,GO:0001562,GO:0022402,GO:0034341,GO:00024	K05425|1|0.0|678|mmu:16160|interleukin 12B	gi|6680397|ref|NP_032378.1|	0	interleukin-12 subunit beta precursor [Mus musculus]

mmu-miR-1197-3p	NM_008525	-26	0.444299	-25.51	149	GO:0044444,GO:0044421,GO:0046914,GO:0005515,GO:0016836,GO:0009314,GO:0033993,GO:0001101,GO:0051259,GO:0033273,GO:0031960,GO:0002237,GO:0006779,GO:0010288,GO:0006950,	K01698|1|0.0|637|mmu:17025|porphobilinogen synthase [EC:4.2.1.24]	gi|34328485|ref|NP_032551.3|	0	delta-aminolevulinic acid dehydratase [Mus musculus]

mmu-miR-1197-3p	NM_008591	-28.2	0.701584	-26.46	156	GO:0043005,GO:0016323,GO:0044430,GO:0016021,GO:0045202,GO:0044297,GO:0044421,GO:0004714,GO:0019900,GO:0032559,GO:0046983,GO:0046685,GO:0070741,GO:0008406,GO:0021782,GO:0007169,GO:0014706,GO:0001678,GO:0031346,GO:0032870,GO:0001933,GO:00164	K05099|1|0.0|2642|mmu:17295|proto-oncogene tyrosine-protein kinase Met [EC:2.7.10.1]	gi|146198696|ref|NP_032617.2|	0	hepatocyte growth factor receptor precursor [Mus musculus]

mmu-miR-1197-3p	NM_009109	-25.5	0.999348	-65.63	458	GO:0016021,GO:0033017,GO:0030017,GO:0005218,GO:0004871,GO:0046872,GO:0005515,GO:0003012,GO:0051283,GO:0014074,GO:0006816,GO:0006950,	K04961|1|0.0|8764|mmu:20190|ryanodine receptor 1	gi|145046267|ref|NP_033135.2|	0	ryanodine receptor 1 [Mus musculus]
mmu-miR-1197-3p	NM_009611	-31.3	0.052289	-26.75	154	GO:0043073,GO:0043232,GO:0005198,	K05692|1|2e-85|317|bmy:Bm1_21705|actin beta/gam	gi|6752956|ref|NP_033741.1|	0	actin-like protein 7A [Mus musculus]
mmu-miR-1197-3p	NM_010143	-27.1	0.699259	-25.15	174	GO:0016021,GO:0005003,GO:0032559,GO:0007411,GO:0007167,GO:0021955,GO:00064	K05112|1|0.0|1905|mmu:13845|Eph receptor B3 [EC:2.7.10.1]	gi|148665184|gb|EDK97600.1|	0	Eph receptor B3, isoform CRA_a [Mus musculus]

mmu-miR-1197-3p	NM_010265	-29.6	0.367996	-25.85	173	GO:0031224,GO:0043231,GO:0012505,GO:0044421,GO:0008375,GO:0009887,GO:0043434,GO:0006486,	K00727|1|0.0|872|mmu:14537|beta-1,3-galactosyl-O-glycosyl-glycoprotein beta-1,6-N-acetylglucosaminyltransferase [EC:2.4.1.1	gi|27734104|ref|NP_775618.1|	0	beta-1,3-galactosyl-O-glycosyl-glycoprotein beta-1,6-N-acetylglucosaminyltransferase [Mus musculus]
mmu-miR-1197-3p	NM_010466	-29.2	0.224796	-25.14	171	GO:0043231,GO:0001071,GO:0003677,GO:0003002,GO:0035115,GO:0006357,GO:0031016,GO:0009887,GO:0030154,	K09308|1|6e-128|459|bta:100295249|homeobox protein HoxB/C/D8	gi|359323087|ref|XP_849475.2|	3.04E-130	PREDICTED: uncharacterized protein LOC607625 [Canis lupus familiaris]

mmu-miR-1197-3p	NM_010566	-28.3	0.576616	-45.22	314	GO:0016020,GO:0015629,GO:0044444,GO:0046030,GO:0016312,GO:0019904,GO:0034594,GO:0002683,GO:0007599,GO:0030853,GO:0019724,GO:0050851,GO:0010259,GO:0006796,GO:0045639,GO:0030888,GO:0006915,GO:0032845,GO:0045577,GO:00	K03084|1|0.0|2163|mmu:16331|phosphatidylinositol-3,4,5-trisphosphate 5-phosphatase 1 [EC:3.1.3.86]	gi|158853999|ref|NP_034696.2|	0	phosphatidylinositol 3,4,5-trisphosphate 5-phosphatase 1 isoform 1 [Mus musculus]

mmu-miR-1197-3p	NM_010577	-28.4	0.517903	-25.1	167	GO:0044424,GO:0009986,GO:0001726,GO:0005924,GO:0043234,GO:0044421,GO:0005887,GO:0004871,GO:0005172,GO:0001934,GO:0007166,GO:0007599,GO:0007611,GO:0016337,GO:0010810,GO:0034329,GO:0044319,GO:0009967,GO:0006935,GO:0002376,GO:0051704,GO:00486	K06484|1|0.0|1961|mmu:16402|integrin alpha 5	gi|148671975|gb|EDL03922.1|	0	integrin alpha 5 (fibronectin receptor alpha), isoform CRA_b [Mus musculus]
mmu-miR-1197-3p	NM_010593	-25.3	0.954413	-25.48	156	-	K10056|1|0.0|1363|mmu:16480|junction plakoglobin	gi|28395018|ref|NP_034723.1|	0	junction plakoglobin [Mus musculus]

mmu-miR-1197-3p	NM_010635	-32.9	0.025509	-29.77	169	GO:0043231,GO:0046914,GO:0001071,GO:0043565,GO:0016568,GO:0043009,GO:0030099,GO:0048469,	K09204|1|0.0|665|mmu:16596|krueppel-like factor 1/2/4	gi|225543580|ref|NP_034765.2|	0	Krueppel-like factor 1 [Mus musculus]
mmu-miR-1197-3p	NM_010865	-28.5	0.259771	-25.33	155	GO:0042995,GO:0005783,GO:0044421,GO:0005488,GO:0032502,	K04593|1|2e-49|198|bfo:BRAFLDRAFT_86900|latrophilin 2!K04594|3|7e-48|192|rno:170641|latrophilin 3	gi|190610029|ref|NP_034995.3|	0	myocilin precursor [Mus musculus]
mmu-miR-1197-3p	NM_011116	-27.2	0.445724	-26.97	161	GO:0031090,GO:0043231,GO:0031224,GO:0005488,GO:0004620,GO:0006629,	-	gi|7242181|ref|NP_035246.1|	0	phospholipase D3 [Mus musculus]

mmu-miR-1197-3p	NM_011510	-31.1	0.232464	-26.4	166	GO:0008021,GO:0005887,GO:0017111,GO:0000149,GO:0046873,GO:0005261,GO:0015405,GO:0032559,GO:0004888,GO:0015980,GO:0050796,GO:0009207,GO:0043434,GO:0010038,GO:0009628,GO:0034220,GO:0006813,GO:0002237,	K05032|1|0.0|2826|mmu:20927|ATP-binding cassette subfamily C (CFTR/MRP) member 8	gi|62241017|ref|NP_035640.2|	0	ATP-binding cassette, sub-family C (CFTR/MRP), member 8 [Mus musculus]
mmu-miR-1197-3p	NM_011520	-31.2	0.224935	-26.78	161	GO:0043005,GO:0032991,GO:0031224,GO:0009987,	K06257|1|7e-81|303|mmu:20970|syndecan	gi|37359970|dbj|BAC97963.1|	5.02E-80	mKIAA0468 protein [Mus musculus]
mmu-miR-1197-3p	NM_011784	-30.8	0.186003	-27.77	158	GO:0031982,GO:0043231,GO:0016021,GO:0005488,GO:0004888,GO:0032501,GO:00071	K04174|1|0.0|670|mmu:23796|apelin receptor	gi|6753014|ref|NP_035914.1|	0	apelin receptor [Mus musculus]

mmu-miR-1197-3p	NM_011948	-27.5	0.725815	-25.42	162	GO:0044444,GO:0043169,GO:0032559,GO:0004702,GO:0043406,GO:0000165,GO:0009953,	K04428|1|0.0|2982|mmu:26407|mitogen-activated protein kinase kinase kinase 4 [EC:2.7.11.25]	gi|93102421|ref|NP_036078.2|	0	mitogen-activated protein kinase kinase kinase 4 [Mus musculus]
mmu-miR-1197-3p	NM_013598	-29.8	0.398127	-26.15	171	GO:0031224,GO:0043232,GO:0044421,GO:0005126,GO:0001934,GO:0006275,GO:0046578,GO:0001667,GO:0002573,GO:0070665,GO:0008406,GO:0022602,GO:0045634,GO:0043405,GO:0033024,	K05461|1|7e-136|486|mmu:17311|KIT ligand	gi|7305267|ref|NP_038626.1|	4.63E-135	kit ligand precursor [Mus musculus]

mmu-miR-1197-3p	NM_013684	-29.7	0.137379	-26.37	170	GO:0005667,GO:0045120,GO:0001071,GO:0043565,GO:0008134,GO:0006355,GO:0006352,GO:0048232,GO:0006354,GO:00063	K03120|1|2e-126|453|mmu:21374|transcription initiation factor TFIID TATA-box-binding protein	gi|148688513|gb|EDL20460.1|	2.48E-129	TATA box binding protein, isoform CRA_b [Mus musculus]
mmu-miR-1197-3p	NM_013763	-32.3	0.12959	-28.18	164	-	K13917|1|1e-69|266|ame:414001|mRNA-capping enzyme [EC:2.7.7.50 3.1.3.33]! K03130|2|1e-11|73.9|xla:100036804|transcription initiation factor TFIID subunit 5	gi|148687432|gb|EDL19379.1|	0	transducin (beta)-like 2, isoform CRA_a [Mus musculus]

mmu-miR-1197-3p	NM_013834	-27.9	0.593918	-25.33	170	GO:0016020,GO:0044444,GO:0031012,GO:0001664,GO:0004888,GO:0019904,GO:0061135,GO:0004175,GO:0005539,GO:0010952,GO:0034695,GO:0000737,GO:0033144,GO:0043065,GO:0002762,GO:0006357,GO:0060442,GO:0050796,GO:0010165,GO:0070555,GO:0014020,GO:00713	K02166|1|2e-155|551|mmu:20377|secreted frizzled-related protein 1	gi|65301472|ref|NP_038862.2|	1.58E-154	secreted frizzled-related protein 1 precursor [Mus musculus]
mmu-miR-1197-3p	NM_015794	-27.1	0.986546	-42.9	317	-	K10283|1|0.0|1050|mmu:50758|F-box and leucine-rich repeat protein	gi|255683359|ref|NP_056609.1|	0	F-box/LRR-repeat protein 17 [Mus musculus]

mmu-miR-1197-3p	NM_016769	-29.9	0.363809	-25.81	169	-	K04500|1|0.0|852|ssc:397260|mothers against decapentaplegic homolog 2/3	gi|5174513|ref|NP_005893.1|	0	mothers against decapentaplegic homolog 3 isoform 1 [Homo sapiens]
mmu-miR-1197-3p	NM_018729	-28.5	0.445868	-25.32	158	GO:0009986,GO:0044425,GO:0005488,GO:0004871,GO:0065007,GO:0023052,	K06582|1|0.0|820|mmu:18106|CD244 natural killer cell receptor 2B4	gi|122114646|ref|NP_061199.2|	0	natural killer cell receptor 2B4 precursor [Mus musculus]
mmu-miR-1197-3p	NM_018789	-29.8	0.240816	-26.06	157	GO:0031981,GO:0043234,GO:0044444,GO:0000981,GO:0043565,GO:0043566,GO:0019900,GO:0031572,GO:0001525,GO:0000122,GO:0048017,GO:0051148,GO:0008283,GO:0048513,GO:0051318,GO:00071	K12358|1|0.0|938|mmu:54601|forkhead box protein O4	gi|9055158|ref|NP_061259.1|	0	forkhead box protein O4 [Mus musculus]

mmu-miR-1197-3p	NM_018887	-28.3	0.400477	-25.09	151	GO:0043231,GO:0031090,GO:0016709,GO:0005506,GO:0008395,GO:0006706,GO:0032501,GO:0006694,GO:0008203,GO:0044237,	K07439|1|0.0|940|mmu:56050|cytochrome P450, family 39, subfamily A (24-hydroxycholesterol 7alpha-hydroxylase) [EC:1.14.13.99]	gi|9256529|ref|NP_061375.1|	0	24-hydroxycholesterol 7-alpha-hydroxylase [Mus musculus]
mmu-miR-1197-3p	NM_019808	-27.9	0.646077	-27.85	166	GO:0044459,GO:0031674,GO:0044430,GO:0008092,GO:0032947,GO:0046914,GO:0019901,GO:0048814,GO:0044087,GO:00485	K05760|1|3e-40|169|nve:NEMVE_v1g104924|paxillin	gi|148680117|gb|EDL12064.1|	0	PDZ and LIM domain 5, isoform CRA_b [Mus musculus]
mmu-miR-1197-3p	NM_021480	-27.2	0.384789	-46.71	319	GO:0043231,GO:0048037,GO:0016616,GO:0008152,	K15789|1|0.0|757|mmu:58865|threonine 3-dehydrogenase [EC:1.1.1.103]	gi|254281204|ref|NP_067455.5|	0	L-threonine 3-dehydrogenase, mitochondrial precursor [Mus musculus]

mmu-miR-1197-3p	NM_022723	-31.2	0.342777	-27.34	161	GO:0009986,GO:0044421,GO:0019898,GO:0046872,GO:0046983,GO:0007599,GO:0006952,GO:0030855,GO:0007507,GO:00512	K06825|1|3e-57|226|mdo:100025769|fibrillin 1	gi|103472029|ref|NP_073560.2|	0	signal peptide, CUB and EGF-like domain-containing protein 1 precursor [Mus musculus]
mmu-miR-1197-3p	NM_023126	-28.7	0.230176	-67.38	453	GO:0043005,GO:0016020,GO:0044430,GO:0044297,GO:0072372,GO:0031410,GO:0005768,GO:0032561,GO:0019900,GO:0032401,GO:0051049,GO:0048168,GO:0006906,GO:0006887,GO:0008104,GO:0010927,GO:00	K07901|1|2e-114|413|rno:117103|Ras-related protein Rab-8A	gi|38372905|ref|NP_075615.2|	1.63E-113	ras-related protein Rab-8A [Mus musculus]

mmu-miR-1197-3p	NM_023655	-30.4	0.168943	-25.84	158	GO:0044424,GO:0046914,GO:0001071,GO:0006351,	K12010|1|0.0|1100|mmu:72169|tripartite motif-containing protein 29	gi|160333881|ref|NP_076144.2|	0	tripartite motif-containing protein 29 [Mus musculus]
mmu-miR-1197-3p	NM_024412	-30.5	0.134795	-25.51	165	GO:0043231,GO:0044459,GO:0034702,GO:0005488,GO:0005254,GO:0010038,GO:0046903,GO:0009628,GO:0006821,GO:0001822,GO:0034220,GO:0043269,GO:00069	K05017|1|0.0|1256|mmu:12733|chloride channel Ka!K05018|3|0.0|1075|mmu:56365|chloride channel Kb	gi|226423866|ref|NP_077723.3|	0	chloride channel protein ClC-Ka [Mus musculus]
mmu-miR-1197-3p	NM_025611	-26.7	0.84871	-42.85	299	GO:0000152,GO:0019899,GO:0006915,GO:0007049,GO:0019941,GO:0030856,	K10613|1|0.0|2989|mmu:66515|cullin 7	gi|57013279|ref|NP_079887.3|	0	cullin-7 [Mus musculus]
mmu-miR-1197-3p	NM_025705	-25.4	0.83752	-56.08	319	GO:0031224,GO:0009987,	K14616|1|4e-11|71.6|dre:100144772|cubili	gi|165377069|ref|NP_079981.2|	0	discoidin, CUB and LCCL domain-containing protein 1 precursor [Mus musculus]

mmu-miR-1197-3p	NM_025815	-26.1	0.770338	-45.07	305	-	K06491|1|8e-12|73.9|cin:100183448|neural cell adhesion molecule!K15290|2|2e-09|65.9|cel:F31E8.2|synaptotagmin-1!K02677|4|2e-07|59.7|xtr:100496779|classical protein kinase C [EC:2.7.11.13]	gi|21630253|ref|NP_080091.1|	0	copine-8 isoform 1 [Mus musculus]
mmu-miR-1197-3p	NM_025962	-31.5	0.067268	-46.53	328	GO:0043231,GO:0019842,	K14618|1|2e-149|530|mmu:67096|methylmalonic aciduria homocystinuria type C	gi|156105691|ref|NP_080238.2|	1.50E-148	methylmalonic aciduria and homocystinuria type C protein homolog [Mus musculus]

mmu-miR-1197-3p	NM_026658	-29.7	0.183504	-25.97	167	GO:0043231,GO:0050662,GO:0002098,	K03495|1|0.0|1208|mmu:68291|tRNA uridine 5-carboxymethylaminomethyl modification enzyme	gi|171460946|ref|NP_080934.2|	0	protein MTO1 homolog, mitochondrial precursor [Mus musculus]
mmu-miR-1197-3p	NM_026932	-30.2	0.192276	-26.65	173	GO:0031981,GO:0005488,GO:0042254,	K14823|1|7e-166|585|mmu:69072|rRNA-processing protein EBP2	gi|148698540|gb|EDL30487.1|	9.86E-174	EBNA1 binding protein 2, isoform CRA_a [Mus musculus]
mmu-miR-1197-3p	NM_027913	-29.9	0.259864	-27.07	166	-	K06702|1|2e-30|135|acs:100554163|CDW93 antigen	gi|226442830|ref|NP_082189.1|	0	von Willebrand factor C and EGF domain-containing protein precursor [Mus musculus]
mmu-miR-1197-3p	NM_027917	-27.8	0.500775	-25.34	150	GO:0016459,GO:0015630,GO:0003779,GO:0007015,GO:0032989,	-	gi|125628648|ref|NP_082193.2|	0	protein Shroom1 [Mus musculus]

mmu-miR-1197-3p	NM_028061	-26.3	0.809365	-44.56	305	GO:0031224,GO:0043169,GO:0051234,GO:0006091,	K11318|1|5e-80|300|aml:100466161|ataxin-7!K08371|4|3e-38|162|mdo:100022046|cytochrome b-561 domain containin	gi|37805394|gb|AAH60107.1|	1.26E-97	Cyb561d1 protein, partial [Mus musculus]
mmu-miR-1197-3p	NM_028715	-30.6	0.176166	-26.87	163	-	K12804|1|2e-18|95.9|rno:300732|proline-serine-threonine phosphatase interacting protein 1	gi|255683303|ref|NP_082991.3|	0	FCH domain only protein 1 [Mus musculus]

mmu-miR-1197-3p	NM_028973	-29	0.499567	-27.95	162	GO:0031224,GO:0009987,	K13023|1|0.0|847|mcc:707574|carboxypeptidase N regulatory subunit!K06260|3|2e-81|305|gga:100857508|platelet glycoprot	gi|58037377|ref|NP_083249.1|	0	leucine-rich repeat-containing protein 15 precursor [Mus musculus]
mmu-miR-1197-3p	NM_028981	-29.6	0.589703	-27.56	157	GO:0034704,GO:0044459,GO:0005245,GO:0015980,GO:0007611,GO:0032412,GO:0050954,GO:0070838,GO:0030073,GO:0006935,GO:0007187,	K04851|1|0.0|3754|mmu:12289|voltage-dependent calcium channel L type alpha-1D	gi|134288900|ref|NP_083257.2|	0	voltage-dependent L-type calcium channel subunit alpha-1D isoform 1 [Mus musculus]

mmu-miR-1197-3p	NM_029348	-26.6	0.93329	-25.35	152	GO:0005488,	K10491|1|0.0|1250|mmu:75580|zinc finger and BTB domain-containing protein 4	gi|50510977|dbj|BAD32474.1|	0	mKIAA1538 protein [Mus musculus]
mmu-miR-1197-3p	NM_029550	-29.2	0.152172	-25.91	162	GO:0005815,GO:0043231,GO:0016410,GO:0015631,	K15517|1|2e-116|420|aml:100476924|glycine N-phenylacetyltransferase [EC:2.3.1.192]!K00628|2|5e-115|415|cfa:483463|glycine N-acyltransferase [EC:2.3.1.13]	gi|19482166|ref|NP_083826.1|	1.12E-173	glycine N-acyltransferase-like protein Keg1 [Mus musculus]
mmu-miR-1197-3p	NM_029835	-29.6	0.522147	-26.19	164	GO:0043231,GO:0005488,GO:0009314,GO:0006413,GO:0033314,	-	gi|78354983|ref|NP_084111.1|	0	treslin [Mus musculus]

mmu-miR-1197-3p	NM_029983	-28	0.373226	-25.57	165	GO:0031410,GO:0031090,GO:0005768,GO:0030674,GO:0002449,GO:0006357,GO:0009968,GO:0050864,GO:0002429,	K05854|1|3e-33|144|gga:421127|tyrosine-protein kinase Lyn [EC:2.7.10.2]!K05856|2|3e-33|144|mdo:100025680|lymphocyte-specific protein tyrosine kinase [EC:2.7.10.2]!K08890|4|1e-32|142|tgu:100223292|B lymphoid tyrosine kinase [EC:2.7.10.	gi|124249228|ref|NP_084259.1|	4.27E-114	src-like-adapter 2 [Mus musculus]

mmu-miR-1197-3p	NM_031178	-25	0.916382	-43.44	310	GO:0000323,GO:0031090,GO:0031224,GO:0045335,GO:0044459,GO:0004872,GO:0005126,GO:0003723,GO:0043565,GO:0042108,GO:0006914,GO:0032481,GO:0051091,GO:0007169,GO:0043406,GO:0043433,GO:0002252,GO:0001774,GO:0010669,GO:0002224,GO:0007249,GO:00521	K10161|1|0.0|1941|mmu:81897|toll-like receptor 9	gi|157057166|ref|NP_112455.2|	0	toll-like receptor 9 precursor [Mus musculus]

mmu-miR-1197-3p	NM_031181	-27.5	0.336092	-25.62	162	GO:0016021,GO:0004872,GO:0007165,	K06748|1|0.0|718|rno:292846|sialic acid binding Ig-like lectin 8!K06740|3|1e-129|464|mcc:719831|sialic acid binding Ig-like lectin 9!K06739|5|4e-127|456|hsa:27036|sialic acid binding Ig-like	gi|148690724|gb|EDL22671.1|	0	sialic acid binding Ig-like lectin E [Mus musculus]
mmu-miR-1197-3p	NM_031408	-28.7	0.552854	-41.47	308	GO:0005488,GO:0007169,	-	gi|154091042|ref|NP_113596.2|	0	PERQ amino acid-rich with GYF domain-containing protein 1 [Mus musculus]
mmu-miR-1197-3p	NM_054042	-33.1	0.050147	-28.58	158	GO:0044424,GO:0031224,GO:0031012,GO:0046872,GO:0030246,	K06706|1|0.0|1397|mmu:70445|CD248 antigen, endosialin	gi|16923944|ref|NP_473383.1|	0	endosialin precursor [Mus musculus]

mmu-miR-1197-3p	NM_054070	-32.3	0.125657	-28.76	170	GO:0043231,GO:0031224,GO:0004175,GO:0046914,GO:0016462,GO:0032559,GO:0009057,GO:0019538,	K08956|1|0.0|1448|mmu:114896|AFG3 family protein [EC:3.4.24.-]	gi|66792806|ref|NP_473411.2|	0	AFG3-like protein 1 [Mus musculus]
mmu-miR-1197-3p	NM_009275	-23.6	0.981204	-123.35	972	GO:0031224,GO:0032991,GO:0005768,GO:0016023,GO:0031090,GO:0012505,GO:0016323,GO:0004871,GO:0019899,GO:0032561,GO:0005506,GO:0000041,GO:0023052,GO:00	K12272|1|2e-135|484|mmu:20818|signal recognition particle receptor subunit beta	gi|6678137|ref|NP_033301.1|	1.56E-134	signal recognition particle receptor subunit beta [Mus musculus]

mmu-miR-1197-5p	NM_001005605	-26.6	0.930394	-42.32	319	GO:0031519,GO:0003712,GO:0003676,GO:0046914,GO:0006351,GO:0006325,	K09232|1|3e-13|79.7|cin:778878|zinc finger protein GLIS1/3!K06230|3|6e-13|78.6|aml:100478391|zinc finger protein	gi|190358164|sp|Q9Z248.2|AEBP2_MOUSE	5.62E-161	RecName: Full=Zinc finger protein AEBP2; AltName: Full=Adipocyte enhancer-binding protein 2; Short=AE-binding protein 2
mmu-miR-1197-5p	NM_001010825	-27.9	0.55566	-25.27	160	GO:0031224,GO:0070566,GO:0032319,	-	gi|169646312|ref|NP_001010825.2|	0	adenosine monophosphate-protein transferase FICD [Mus
mmu-miR-1197-5p	NM_001033764	-29.9	0.385374	-26.9	174	-	-	gi|237757316|ref|NP_001028936.2|	0	uncharacterized protein LOC210940 [Mus musculus]
mmu-miR-1197-5p	NM_001079690	-30.1	0.384357	-26.78	157	GO:0044459,GO:0031224,GO:0015377,GO:0005488,GO:0015698,GO:0042592,GO:0003008,GO:0048513,GO:0030001,	K14425|1|0.0|2013|mmu:20495|solute carrier family 12 (sodium/potassium/chloride transporter), member	gi|119226233|ref|NP_001073158.1|	0	solute carrier family 12 member 1 isoform F [Mus musculus]

mmu-miR-1197-5p	NM_001081247	-32.1	0.192899	-27.05	174	GO:0044451,GO:0003676,GO:0046914,GO:0034062,GO:0001882,GO:0006952,GO:0051707,GO:0032481,GO:0006353,GO:00063	K03018|1|0.0|2734|mmu:218832|DNA-directed RNA polymerase III subunit RPC1 [EC:2.7.7.6]	gi|124486963|ref|NP_001074716.1|	0	DNA-directed RNA polymerase III subunit RPC1 [Mus musculus]
mmu-miR-1197-5p	NM_001136076	-29.5	0.242199	-25.79	150	GO:0044432,GO:0019842,GO:0046914,GO:0016701,GO:0019798,GO:0019471,	K00472|1|0.0|1050|mmu:18452|prolyl 4-hydroxylase [EC:1.14.11.2]	gi|148701600|gb|EDL33547.1|	0	procollagen-proline, 2-oxoglutarate 4-dioxygenase (proline 4-hydroxylase), alpha II polypeptide, isoform CRA_e [Mus musculus]

mmu-miR-1197-5p	NM_001159299	-27	0.689684	-27.63	154	GO:0016020,GO:0044424,GO:0046872,GO:0005539,GO:0004866,GO:0010466,GO:0030203,	K10798|1|4e-12|74.7|nve:NEMVE_v1g88877|poly [ADP-ribose] polymerase [EC:2.4.2.30]!K05316|2|2e-09|66.2|cqu:CpipJ_CPIJ005979|voltage-dependent calcium channel alpha-2/delta, invertebrate	gi|148692824|gb|EDL24771.1|	0	inter alpha-trypsin inhibitor, heavy chain 4, isoform CRA_d [Mus musculus]

mmu-miR-1197-5p	NM_001159328	-27	0.660192	-25.33	159	GO:0005771,GO:0016023,GO:0005769,GO:0016772,GO:0046914,GO:0005515,GO:0016192,GO:0007041,GO:0006796,GO:0009894,GO:0008283,GO:0015031,GO:0007173,GO:00	K12182|1|0.0|1348|mmu:15239|hepatocyte growth factor-regulated tyrosine kinase substrate	gi|226693388|ref|NP_001152800.1|	0	hepatocyte growth factor-regulated tyrosine kinase substrate isoform 1 [Mus musculus]
mmu-miR-1197-5p	NM_001162477	-28.4	0.715218	-26.12	155	GO:0032991,GO:0044444,GO:0016020,GO:0030674,GO:0035091,GO:0048870,GO:0002573,GO:0007167,GO:0048017,GO:0033008,GO:0008283,	K08091|1|0.0|1321|mmu:14389|growth factor receptor bound protein 2-associated protein 2	gi|247270076|ref|NP_001155949.1|	0	GRB2-associated-binding protein 2 isoform 2 [Mus musculus]

mmu-miR-1197-5p	NM_001163155	-25.3	0.990752	-41.66	303	GO:0005604,GO:0045202,GO:0048741,	K06237|1|8e-162|573|mmu:12830|collagen, type IV, alpha	gi|123122921|emb|CAM20992.1|	5.89E-161	procollagen type IV alpha 5 [Mus musculus]
mmu-miR-1197-5p	NM_001163518	-31.2	0.132011	-25.94	165	-	-	gi|242332551|ref|NP_666171.2|	0	coiled-coil domain containing 121-like isoform 1 [Mus musculus]

mmu-miR-1197-5p	NM_001163634	-32.5	0.120962	-25.34	174	GO:0044424,GO:0016020,GO:0031012,GO:0060089,GO:0005198,GO:0001664,GO:0033483,GO:0048858,GO:0042692,GO:0072044,GO:0060433,GO:0043009,GO:0043062,GO:0001656,GO:0016337,GO:0035567,GO:0030900,GO:0007163,GO:0031960,GO:0042476,GO:0012502,GO:00455	K00572|1|0.0|664|bta:525135|wingless-type MMTV integration site family, member 7	gi|254692926|ref|NP_001157106.1|	0	protein Wnt-7b isoform 2 [Mus musculus]

mmu-miR-1197-5p	NM_001164627	-26.1	0.547279	-25.56	161	GO:0044444,GO:0008047,GO:0035556,GO:0043087,GO:0009966,	K10360|1|1e-17|92.0|nvi:100121553|myosin IX!K07526|2|3e-16|87.4|spu:594484|SLIT-ROBO Rho GTPase activating protein!K01514|5|1e-15|85.5|mcc:708898|exopolyphosphatase [EC:3.6.1.11]	gi|92092529|gb|AAH10306.2|	0	Arhgap8 protein [Mus musculus]

mmu-miR-1197-5p	NM_001164628	-26.1	0.54512	-25.56	161	-	K10360|1|1e-17|92.0|nvi:100121553|myosin IX!K07526|2|3e-16|87.4|spu:594484|SLIT-ROBO Rho GTPase activating protein!K01514|5|1e-15|85.5|mcc:708898|exopolyphosphatase [EC:3.6.1.11]	gi|92092529|gb|AAH10306.2|	0	Arhgap8 protein [Mus musculus]
mmu-miR-1197-5p	NM_001166067	-28.7	0.614627	-47.46	323	GO:0044459,GO:0031224,GO:0015081,GO:0015301,GO:0015698,GO:0055067,GO:0030001,	K13857|1|0.0|1859|mmu:232156|solute carrier family 4 (sodium bicarbonate cotransporter), member 5	gi|260593708|ref|NP_001159539.1|	0	solute carrier family 4, sodium bicarbonate cotransporter, member 5 [Mus musculus]

mmu-miR-1197-5p	NM_001168321	-26.2	0.955076	-43.1	317	GO:0043231,GO:0043234,GO:0003712,GO:0043565,GO:0019787,GO:0019899,GO:0000122,GO:0030279,GO:0007389,GO:0070168,GO:0009887,GO:0032446,GO:0000414,GO:00	K10683|1|3e-16|89.7|ecb:100053962|BRCA1-associated RING domain protein 1 [EC:6.3.2.19]	gi|37360460|dbj|BAC98208.1|	0	mKIAA1575 protein [Mus musculus]
mmu-miR-1197-5p	NM_001199632	-25.1	0.892378	-26.91	144	-	K10279|1|0.0|1555|mmu:320118|F-box and leucine-rich repeat protein	gi|313851009|ref|NP_001186561.1|	0	F-box/LRR-repeat protein 13 isoform a [Mus musculus]

mmu-miR-1197-5p	NM_001205334	-26.1	0.55263	-25.56	161	GO:0044444,GO:0008047,GO:0035556,GO:0043087,GO:0009966,	K10360|1|1e-17|92.0|nvi:100121553|myosin IX!K07526|2|3e-16|87.4|spu:594484|SLIT-ROBO Rho GTPase activating protein!K01514|5|1e-15|85.5|mcc:708898|exopolyphosphatase [EC:3.6.1.11]	gi|92092529|gb|AAH10306.2|		0	Arhgap8 protein [Mus musculus]
mmu-miR-1197-5p	NM_001252580	-29.3	0.454152	-27.02	163	GO:0019898,GO:0010008,GO:0016790,GO:0004175,GO:0019904,GO:0019783,GO:0006996,GO:0007264,GO:0019941,GO:00165	K11839|1|0.0|1909|mmu:84092|ubiquitin carboxyl-terminal hydrolase 8 [EC:3.1.2.15]	gi|148696217|gb|EDL28164.1|		0	ubiquitin specific peptidase 8, isoform CRA_b [Mus musculus]

mmu-miR-1197-5p	NM_007736	-25.3	0.989714	-41.66	303	GO:0005604,GO:0045202,GO:0048741,	K06237|1|8e-162|573|mmu:12830|collagen, type IV, alpha	gi|123122921|emb|CAM20992.1|	5.72E-161	procollagen type IV alpha 5 [Mus musculus]
mmu-miR-1197-5p	NM_008062	-31.7	0.119642	-26.95	178	GO:0005815,GO:0043231,GO:0044459,GO:0000166,GO:0016616,GO:0042802,GO:0048029,GO:0048469,GO:0006098,GO:0002034,GO:0010033,GO:0019302,GO:0008203,GO:0001816,GO:0006790,GO:0050789,GO:0035264,GO:0010731,GO:0006979,GO:00	K00036|1|0.0|1029|mmu:14381|glucose-6-phosphate 1-dehydrogenase [EC:1.1.1.49]	gi|6996917|ref|NP_032088.1|	0	glucose-6-phosphate 1-dehydrogenase X [Mus musculus]

mmu-miR-1197-5p	NM_008244	-27	0.659794	-25.33	159	GO:0005771,GO:0016023,GO:0005769,GO:0016772,GO:0046914,GO:0005515,GO:0016192,GO:0007041,GO:0006796,GO:0009894,GO:0008283,GO:0015031,GO:0007173,GO:00	K12182|1|0.0|1342|mmu:15239|hepatocyte growth factor-regulated tyrosine kinase substrate	gi|226874952|ref|NP_032270.3|	0	hepatocyte growth factor-regulated tyrosine kinase substrate isoform 2 [Mus musculus]
mmu-miR-1197-5p	NM_008369	-29.7	0.127488	-25.73	162	GO:0016020,GO:0004888,GO:0007166,	K04737|1|0.0|763|mmu:16188|interleukin 3 receptor alpha	gi|148688684|gb|EDL20631.1|	0	interleukin 3 receptor, alpha chain, isoform CRA_b [Mus musculus]

mmu-miR-1197-5p	NM_008656	-29.2	0.247357	-26.08	150	GO:0031981,GO:0000981,GO:0046983,GO:0007160,GO:0048729,GO:0006357,GO:0045597,GO:0048562,GO:0014706,GO:0009952,GO:0016337,GO:0001654,GO:0042692,GO:00	K09064|1|2e-48|194|xtr:394569|myogenic factor	gi|6678982|ref|NP_032682.1|	9.89E-130	myogenic factor 5 [Mus musculus]

mmu-miR-1197-5p	NM_008898	-29.4	0.271686	-25.43	167	GO:0043231,GO:0031090,GO:0046914,GO:0005515,GO:0000166,GO:0016675,GO:0050662,GO:0016653,GO:0010893,GO:0002062,GO:0032768,GO:0008203,GO:0009698,GO:0006475,GO:0006730,GO:0006631,GO:0006520,GO:0006915,GO:0090345,GO:0008589,GO:0044270,GO:00328	K00327|1|0.0|1309|mmu:18984|NADPH-ferrihemoprotein reductase [EC:1.6.2.4]	gi|6679421|ref|NP_032924.1|	0	NADPH--cytochrome P450 reductase [Mus musculus]

mmu-miR-1197-5p	NM_008907	-31.6	0.021538	-27.11	173	GO:0043231,GO:0044444,GO:0016859,GO:0005515,GO:0044409,GO:0006310,GO:0044267,GO:0001775,GO:0006260,GO:0002376,GO:0030154,GO:0050792,GO:00075	K03767|1|2e-93|342|mmu:268373|peptidyl-prolyl cis-trans isomerase A (cyclophilin A) [EC:5.2.1.8]	gi|392353052|ref|XP_002728136.2|	1.56E-96	PREDICTED: peptidyl-prolyl cis-trans isomerase A-like [Rattus norvegicus]
mmu-miR-1197-5p	NM_010043	-30.6	0.148152	-28.51	159	GO:0005913,GO:0005882,GO:0031674,GO:0005488,GO:0005198,GO:0048513,	K07610|1|0.0|708|mmu:13346|desmin	gi|148667985|gb|EDL00402.1|	0	desmin [Mus musculus]
mmu-miR-1197-5p	NM_010061	-30.2	0.094717	-25.27	175	GO:0012505,GO:0043231,GO:0008092,GO:0016888,GO:0006259,GO:0012501,	K11994|1|8e-142|504|mmu:13419|deoxyribonuclease-1 [EC:3.1.21.1]	gi|148664803|gb|EDK97219.1|	3.08E-141	deoxyribonuclease I, isoform CRA_a [Mus musculus]

mmu-miR-1197-5p	NM_010248	-28.4	0.715392	-26.12	155	GO:0032991,GO:0044444,GO:0016020,GO:0030674,GO:0035091,GO:0048870,GO:0002573,GO:0007167,GO:0048017,GO:0033008,GO:0008283,	K08091|1|0.0|1317|mmu:14389|growth factor receptor bound protein 2-associated protein 2	gi|247270049|ref|NP_034378.2|	0	GRB2-associated-binding protein 2 isoform 1 [Mus musculus]
mmu-miR-1197-5p	NM_011031	-29.5	0.242863	-25.79	150	GO:0044432,GO:0019842,GO:0046914,GO:0016701,GO:0019798,GO:0019471,	K00472|1|0.0|1038|rno:360526|prolyl 4-hydroxylase [EC:1.14.11.2]	gi|148701600|gb|EDL33547.1|	0	procollagen-proline, 2-oxoglutarate 4-dioxygenase (proline 4-hydroxylase), alpha II polypeptide, isoform CRA_e [Mus musculus]
mmu-miR-1197-5p	NM_011215	-26.6	0.872305	-26.72	159	GO:0016021,GO:0033267,GO:0016023,GO:0005488,GO:0004871,GO:0004725,GO:0006470,GO:0043087,GO:0023052,	K07817|1|0.0|1833|mmu:19276|protein tyrosine phosphatase, receptor type, N [EC:3.1.3.48]	gi|226052731|ref|NP_035345.2|	0	receptor-type tyrosine-protein phosphatase N2 precursor [Mus musculus]

mmu-miR-1197-5p	NM_013675	-26.8	0.979279	-25.06	161	GO:0032991,GO:0030864,GO:0009898,GO:0005198,GO:0003779,GO:0030835,GO:0006778,GO:0006935,	K06115|1|0.0|4436|mmu:20741|spectrin beta	gi|84490394|ref|NP_038703.3|	0	spectrin beta chain, erythrocyte [Mus musculus]
mmu-miR-1197-5p	NM_013788	-26.5	0.701659	-26.57	153	GO:0007166,	K03069|1|2e-62|232|mmu:14296|frequently rearranged in advanced T-cell lymphom	gi|7305067|ref|NP_038816.1|	4.25E-96	paternally expressed 12 [Mus musculus]
mmu-miR-1197-5p	NM_013854	-27.7	0.588349	-28.65	164	GO:0031981,GO:0005840,GO:0012505,GO:0090079,GO:0017111,GO:0043021,GO:0032559,GO:0003723,GO:0006412,GO:0022613,GO:0006952,	K06184|1|0.0|1157|mmu:224742|ATP-binding cassette, subfamily F, member 1	gi|39930335|ref|NP_038882.1|	0	ATP-binding cassette sub-family F member 1 [Mus musculus]

mmu-miR-1197-5p	NM_018746	-27	0.690043	-27.63	154	GO:0016020,GO:0044424,GO:0046872,GO:0005539,GO:0004866,GO:0010466,GO:0030203,	K10798|1|4e-12|74.7|nve:NEMVE_v1g88877|poly [ADP-ribose] polymerase [EC:2.4.2.30]!K05316|2|2e-09|66.2|cqu:CpipJ_CPIJ005979|voltage-dependent calcium channel alpha-2/delta, invertebrate	gi|226531047|ref|NP_061216.2|	0	inter alpha-trypsin inhibitor, heavy chain 4 isoform 1 precursor [Mus musculus]
mmu-miR-1197-5p	NM_019729	-29.3	0.451379	-27.02	163	GO:0019898,GO:0010008,GO:0016790,GO:0004175,GO:0019904,GO:0019783,GO:0006996,GO:0007264,GO:0019941,GO:00165	K11839|1|0.0|1917|mmu:84092|ubiquitin carboxyl-terminal hydrolase 8 [EC:3.1.2.15]	gi|28972047|dbj|BAC65477.1|	0	mKIAA0055 protein [Mus musculus]

mmu-miR-1197-5p	NM_021464	-28.7	0.696865	-45.02	312	GO:0031224,GO:0004871,GO:0004721,GO:0050839,GO:0042805,GO:0016337,GO:0007167,GO:0006470,	K13297|1|0.0|2795|mmu:19281|protein tyrosine phosphatase, receptor type, T [EC:3.1.3.48]	gi|148674357|gb|EDL06304.1|	0	protein tyrosine phosphatase, receptor type, T [Mus musculus]
mmu-miR-1197-5p	NM_027335	-27.4	0.585421	-26.26	159	-	-	gi|254675232|ref|NP_081611.1|	7.93E-173	uncharacterized protein LOC70153 [Mus musculus]
mmu-miR-1197-5p	NM_027452	-31.8	0.133429	-51.38	320	GO:0044459,GO:0031224,GO:0005488,	K07523|1|1e-21|106|dre:553785|netrin-G1 ligand	gi|28972684|dbj|BAC65758.1|	0	mKIAA1246 protein [Mus musculus]

mmu-miR-1197-5p	NM_028455	-26.1	0.550498	-25.56	161	-	K10360|1|1e-17|92.0|nvi:100121553|myosin IX!K07526|2|3e-16|87.4|spu:594484|SLIT-ROBO Rho GTPase activating protein!K01514|5|1e-15|85.5|mcc:708898|exopolyphosphatase [EC:3.6.1.11]	gi|92092529|gb|AAH10306.2|	0	Arhgap8 protein [Mus musculus]

mmu-miR-1197-5p	NM_028712	-28.8	0.524121	-26.2	167	GO:0010008,GO:0032561,GO:0005515,GO:0017111,GO:0051234,GO:0001934,GO:0007265,GO:0045859,GO:0043408,GO:0010769,GO:0009207,GO:0030036,GO:0008104,GO:00	K07838|1|1e-101|336|ptr:739324|Ras-related protein Rap-2B	gi|13386338|ref|NP_082988.1|	7.64E-90	ras-related protein Rap-2b precursor [Mus musculus]
mmu-miR-1197-5p	NM_133780	-28.5	0.777387	-28.56	154	GO:0031981,GO:0000776,GO:0005635,GO:0032559,GO:0004812,GO:0050658,GO:0045841,GO:0006418,GO:0006605,GO:00157	K09291|1|0.0|3638|mmu:108989|nucleoprotein TPR	gi|270309140|ref|NP_598541.3|	0	nuclear pore complex-associated protein Tpr [Mus musculus]
mmu-miR-1197-5p	NM_146059	-31.2	0.124767	-25.94	165	-	-	gi|242332551|ref|NP_666171.2|	0	coiled-coil domain containing 121-like isoform 1 [Mus musculus]

mmu-miR-1197-5p	NM_146380	-33.5	0.013771	-27.85	171	GO:0004871,GO:0023052,	K04257|1|0.0|664|mmu:258378|olfactory receptor	gi|22129545|ref|NP_666492.1|	0	olfactory receptor 593 [Mus musculus]
mmu-miR-1197-5p	NM_146477	-31.9	0.032042	-26.41	174	GO:0004871,GO:0023052,	K04257|1|3e-152|538|mmu:258469|olfactory	gi|255069782|ref|NP_666688.2|	1.83E-151	olfactory receptor 90 [Mus musculus]
mmu-miR-1197-5p	NM_146909	-33.7	0.011319	-29.97	176	GO:0004888,GO:0023052,	K04257|1|2e-176|618|mmu:18351|olfactory	gi|49170066|ref|NP_667120.1|	1.14E-175	olfactory receptor 51 [Mus musculus]
mmu-miR-1197-5p	NM_172205	-25.1	0.855985	-61.85	485	GO:0044424,	K12841|1|2e-16|88.6|nvi:100116536|calcium homeostasis endoplasmic reticulum protein!K03903|3|1e-12|76.3|cfa:475473|fibrinogen alpha	gi|144953885|ref|NP_757342.2|	0	suprabasin isoform 1 precursor [Mus musculus]

mmu-miR-1197-5p	NM_172525	-31	0.311068	-46.92	313	GO:0044444,GO:0005099,GO:0043169,GO:0007265,GO:0043087,GO:0009966,	K02649|1|1e-25|120|bfo:BRAFLDRAFT_91712|phosphoinositide-3-kinase, regulatory subunit!K04450|2|8e-25|117|ptr:739083|activating transcription factor 2!K10360|3|1e-24|117|tca:656579|	gi|33563303|ref|NP_766113.1|	0	rho GTPase-activating protein 29 [Mus musculus]
mmu-miR-1197-5p	NM_173751	-26.6	0.640253	-25.24	165	GO:0031224,GO:0046872,GO:0003824,GO:0019842,	K11259|1|0.0|1150|mmu:216136|acetolactate synthase-like protein [EC:2.2.1.-]	gi|148699779|gb|EDL31726.1|	0	ilvB (bacterial acetolactate synthase)-like, isoform CRA_d [Mus musculus]

mmu-miR-1197-5p	NM_176829	-28.6	0.461638	-29.76	158	GO:0044424,GO:0019787,GO:0031396,	K10349|1|2e-94|348|dre:567119|Fem-1 homolog b	gi|56238583|emb|CAI26025.1|	0	novel protein similar to Mus musculus feminization 1 homolog a (C. elegans)
mmu-miR-1197-5p	NM_177076	-25.1	0.883082	-26.91	144	-	K10279|1|0.0|1471|mmu:320118|F-box and leucine-rich repeat protein	gi|161333858|ref|NP_796050.2|	0	F-box/LRR-repeat protein 13 isoform b [Mus musculus]
mmu-miR-1197-5p	NM_177304	-31.2	0.172935	-27.82	164	GO:0031224,GO:0008081,GO:0042439,GO:0006629,	K08743|1|0.0|905|mmu:320981|ectonucleotide pyrophosphatase/phosphodiesterase family member 6 [EC:3.1.4.-]	gi|28893409|ref|NP_796278.1|	0	ectonucleotide pyrophosphatase/phosphodiesterase family member 6 precursor [Mus musculus]
mmu-miR-1197-5p	NM_178675	-30.6	0.345507	-26.47	161	GO:0031224,GO:0051234,	K15287|1|0.0|765|mmu:215085|solute carrier family 35, member F1/2	gi|40254332|ref|NP_848790.2|	0	solute carrier family 35 member F1 [Mus musculus]

mmu-miR-1197-5p	NM_178803	-26.6	0.922918	-42.32	319	GO:0031519,GO:0003712,GO:0003676,GO:0046914,GO:0006351,GO:0006325,	K09232|1|3e-13|79.7|cin:778878|zinc finger protein GLIS1/3	gi|190358164|sp|Q9Z248.2|AEBP2_MOUSE	3.48E-152	RecName: Full=Zinc finger protein AEBP2; AltName: Full=Adipocyte enhancer-binding protein 2; Short=AE-binding protein 2
mmu-miR-1197-5p	NM_183354	-30.1	0.384357	-26.78	157	GO:0044459,GO:0031224,GO:0015377,GO:0005488,GO:0015698,GO:0042592,GO:0003008,GO:0048513,GO:0030001,	K14425|1|0.0|2021|mmu:20495|solute carrier family 12 (sodium/potassium/chloride transporter), member	gi|119226231|ref|NP_899197.2|	0	solute carrier family 12 member 1 isoform A [Mus musculus]
mmu-miR-1197-5p	NM_198306	-35.8	0.02438	-30.05	175	GO:0012505,GO:0043231,GO:0031224,GO:0030246,GO:0008376,	K00710|1|0.0|1121|hsa:50614|polypeptide N-acetylgalactosaminyltransferase [EC:2.4.1.41]	gi|169790935|ref|NP_938048.2|	0	polypeptide N-acetylgalactosaminyltransferase 9 isoform A [Mus musculus]
mmu-miR-1197-5p	NM_207631	-30.7	0.049389	-26.8	168	GO:0004871,GO:0023052,	K04257|1|8e-167|586|mmu:236785|olfactory	gi|52421341|ref|NP_997514.1|	5.13E-166	olfactory receptor 1321 [Mus musculus]

mmu-miR-1197-5p	NR_027805	-31.4	0.103757	-27.9	173	-	-	gi|148709434|gb|EDL41380.1|	3.66E-63	RIKEN cDNA A430093F15, isoform CRA_b [Mus musculus]
mmu-miR-1197-5p	NM_029078	-20.9	1	-72.53	496	GO:0043234,GO:0031981,GO:0000377,GO:0006397,GO:0006353,	K14400|1|0.0|2521|mmu:74737|pre-mRNA cleavage complex 2 protein Pcf11	gi|50510655|dbj|BAD32313.1|	0	mKIAA0824 protein [Mus musculus]
mmu-miR-1197-5p	NM_175046	-21	1	-43.1	317	GO:0043231,GO:0043234,GO:0003712,GO:0043565,GO:0019787,GO:0019899,GO:0000122,GO:0030279,GO:0007389,GO:0070168,GO:0009887,GO:0032446,GO:0000414,GO:00	K10683|1|3e-16|89.7|ecb:100053962|BRCA1-associated RING domain protein 1 [EC:6.3.2.19]	gi|37360460|dbj|BAC98208.1|	0	mKIAA1575 protein [Mus musculus]
mmu-miR-1197-5p	NM_001166549	-23.6	0.996979	-45.29	316	GO:0043231,GO:0044444,GO:0008135,GO:0006810,GO:0006412,	-	gi|262118204|ref|NP_001160020.1|	0	eukaryotic translation initiation factor 4E transporter isoform b [Mus musculus]

mmu-miR-1197-5p	NM_023743	-23.6	0.996891	-42.7	313	GO:0043231,GO:0044444,GO:0008135,GO:0006810,GO:0006412,	-	gi|262118200|ref|NP_076232.2|	0	eukaryotic translation initiation factor 4E transporter isoform a [Mus musculus]
mmu-miR-1197-5p	NM_016769	-24.6	0.993473	-48.1	311	-	K04500|1|0.0|852|ssc:397260|mothers against decapentaplegic homolog 2/3	gi|5174513|ref|NP_005893.1|	0	mothers against decapentaplegic homolog 3 isoform 1 [Homo sapiens]
mmu-miR-1197-5p	NM_199221	-20.6	1	-30.06	187	GO:0016020,GO:0004871,GO:0023052,	K06719|1|2e-29|132|bta:524761|CD300 antigen	gi|80978941|ref|NP_954691.2|	1.00E-146	CMRF35-like molecule 7 [Mus musculus]
mmu-miR-1197-5p	NM_027151	-29.4	0.209487	-24.56	178	-	K10424|1|0.0|669|rno:299850|dynactin	gi|28076935|ref|NP_081427.1|	0	dynactin subunit 2 isoform 3 [Mus musculus]

mmu-miR-1197-5p	NM_008740	-20	1	-29.62	176	GO:0044430,GO:0044431,GO:0030425,GO:0004175,GO:0000149,GO:0005083,GO:0032559,GO:0043169,GO:0042623,GO:0030001,GO:0016044,GO:0006887,GO:0009207,GO:0001505,GO:0019538,	K06027|1|0.0|1375|mmu:18195|vesicle-fusing ATPase [EC:3.6.4.6]	gi|49518|emb|CAA33678.1|	0	N-ethylmaleimide sensitive fusion protein [Cricetulus longicaudatus]
mmu-miR-1197-5p	NM_009508	-20.8	1	-30.69	175	GO:0016021,GO:0008021,GO:0030665,GO:0043679,GO:0030427,GO:0015175,GO:0005280,GO:0015804,GO:0006818,GO:0006811,GO:0001505,GO:0032502,	K15015|1|0.0|1009|mmu:22348|solute carrier family 32 (vesicular inhibitory amino acid transporter)	gi|125490380|ref|NP_033534.2|	0	vesicular inhibitory amino acid transporter [Mus musculus]

mmu-miR-1197-5p	NM_029044	-28.8	0.255856	-23.25	171	GO:0044424,	K11293|1|4e-11|70.5|cel:K10D2.1|protein HIRA/HIR1	gi|21312844|ref|NP_083320.1|	0	leucine-rich repeat-containing protein 48 [Mus musculus]
mmu-miR-1197-5p	NM_178362	-24.7	0.997009	-21.65	170	GO:0031981,GO:0005815,GO:0032432,GO:0005913,GO:0005924,GO:0005887,GO:0005102,GO:0030674,GO:0008092,GO:0019900,GO:0003012,GO:0032868,GO:0008610,GO:0051017,GO:0007044,GO:0007169,GO:0005979,GO:0031589,GO:0048522,GO:00	K06086|1|0.0|1485|rno:686098|sorbin and SH3 domain containing 1	gi|148709890|gb|EDL41836.1|	0	sorbin and SH3 domain containing 1, isoform CRA_d [Mus musculus]

mmu-miR-1197-5p	NM_019652	-25.9	0.507275	-20.94	169	GO:0031981,GO:0015103,GO:0043169,GO:0019829,GO:0032559,GO:0010035,GO:0015698,GO:0030003,	K01551|1|2e-179|629|ssc:100520362|arsenite-transporting ATPase [EC:3.6.3.16]	gi|344282711|ref|XP_003413116.1|	1.38E-178	PREDICTED: ATPase Asna1-like [Loxodonta africana]
mmu-miR-1197-5p	NM_023122	-27.9	0.523029	-23.57	168	GO:0031224,GO:0048731,GO:0048869,	-	gi|148708789|gb|EDL40736.1|	5.55E-161	glycoprotein m6b, isoform CRA_d [Mus musculus]
mmu-miR-1197-5p	NM_147046	-29.4	0.087957	-24.87	167	GO:0031224,GO:0004930,GO:0007166,	K04257|1|4e-161|567|mmu:259048|olfactory	gi|268607577|ref|NP_667257.2|	2.40E-160	olfactory receptor 600 [Mus musculus]
mmu-miR-1197-5p	NM_181418	-29	0.434979	-22.92	166	GO:0019904,	-	gi|148696967|gb|EDL28914.1|	0	Usher syndrome 1C binding protein 1 [Mus musculus]
mmu-miR-1197-5p	NR_033540	-21.5	0.999995	-22.41	165	-	-	gi|74181893|dbj|BAE32647.1|	9.38E-37	unnamed protein product [Mus musculus]
mmu-miR-1197-5p	NM_201611	-26	0.529345	-20.69	164	GO:0031224,	K06751|1|0.0|647|mmu:399549|major histocompatibility complex, class I	gi|42475548|ref|NP_963905.1|	0	histocompatibility 2, M region locus 10.6 precursor [Mus musculus]
mmu-miR-1197-5p	NR_045156	-28.1	0.560637	-23.53	163	-	-	gi|74196599|dbj|BAE34410.1|	3.04E-20	unnamed protein product [Mus musculus]

mmu-miR-1197-5p	NM_194348	-20.6	1	-22.02	162	GO:0005488,	-	gi|37359928|dbj|BAC97942.1|	0	mKIAA0404 protein [Mus musculus]
mmu-miR-1197-5p	NM_183294	-29.6	0.167407	-21.88	161	GO:0043231,GO:0004674,GO:0032559,GO:0006464,GO:0048513,	K08824|1|0.0|733|mmu:71091|cyclin-dependent kinase-like [EC:2.7.11.22]	gi|34304105|ref|NP_899117.1|	0	cyclin-dependent kinase-like 1 [Mus musculus]
mmu-miR-1197-5p	NR_045844	-24.6	0.669015	-20.01	160	-	-	gi|148705031|gb|EDL36978.1|	1.59E-48	mCG1051104 [Mus musculus]
mmu-miR-1197-5p	NM_198300	-25.7	0.972836	-21.25	159	GO:0003676,	K02602|1|1e-54|217|oaa:100078527|oo18 RNA-binding protein	gi|26006231|dbj|BAC41458.1|	0	mKIAA0940 protein [Mus musculus]
mmu-miR-1197-5p	NR_027993	-22.2	1	-20.67	158	-	-	gi|392333127|ref|XP_003752802.1|	0	PREDICTED: uncharacterized LOC100912366 [Rattus norvegicus]
mmu-miR-1197-5p	NM_177909	-20	1	-21.01	157	GO:0005770,GO:0031224,GO:0005451,GO:0055067,GO:0006818,GO:0030001,	K14725|1|0.0|1120|mmu:331004|solute carrier family 9 (sodium/hydrogen exchanger), member 9	gi|148688985|gb|EDL20932.1|	0	solute carrier family 9 (sodium/hydrogen exchanger), isoform 9, isoform CRA_c [Mus musculus]

mmu-miR-1197-5p	NR_040441	-22.1	0.999555	-20.32	156	GO:0016020,	K15284|1|2e-27|125|mmu:320541|solute carrier family 35, member E2	gi|28893193|ref|NP_796160.1|	1.50E-26	solute carrier family 35 member E2 [Mus musculus]
mmu-miR-1197-5p	NR_040336	-23.1	0.969244	-20.58	155	-	-	gi|148695944|gb|EDL27891.1|	1.04E-33	mCG144765 [Mus musculus]
mmu-miR-1197-5p	NM_207239	-21.4	1	-23.87	154	GO:0005667,GO:0003676,GO:0006383,	K15199|1|0.0|3712|mmu:233863|general transcription factor 3C polypeptide 1	gi|46402235|ref|NP_997122.1|	0	general transcription factor 3C polypeptide 1 [Mus musculus]
mmu-miR-1197-5p	NR_033305	-27.1	0.749963	-20.72	153	-	-	gi|148675524|gb|EDL07471.1|	1.02E-56	mCG147212 [Mus musculus]
mmu-miR-1197-5p	NM_207557	-20.2	0.999434	-20.24	152	GO:0016020,GO:0004872,GO:0023052,	K04257|1|5e-144|510|mmu:404318|olfactory	gi|284005566|ref|NP_997440.2|	3.50E-143	olfactory receptor 681 [Mus musculus]
mmu-miR-1197-5p	NR_045519	-21	0.999998	-20.82	151	GO:0043231,GO:0006810,	K15304|1|0.0|747|mmu:71810|Ran-binding protein 3	gi|148706239|gb|EDL38186.1|	0	RAN binding protein 3, isoform CRA_c [Mus musculus]

mmu-miR-1197-5p	NM_153071	-24.6	0.948674	-22.52	150	GO:0016021,GO:0004888,GO:0007166,GO:0019932,GO:0001101,	K04622|1|0.0|1827|mmu:210198|G protein-coupled receptor family C group 6 member A	gi|23346475|ref|NP_694711.1|	0	G-protein coupled receptor family C group 6 member A precursor [Mus musculus]
mmu-miR-1197-5p	NM_178647	-20.8	1	-20.19	149	GO:0043231,GO:0043566,GO:0010467,	-	gi|30520001|ref|NP_848762.1|	7.22E-91	CGG triplet repeat-binding protein 1 [Mus musculus]
mmu-miR-1197-5p	NR_073122	-24.7	0.951024	-20.26	148	-	-	-	-	-
mmu-miR-1197-5p	NM_182990	-23.7	0.985802	-20.7	147	GO:0031981,GO:0043232,GO:0003676,GO:0006259,GO:0033554,GO:0045935,GO:00063	K09272|1|0.0|1112|mmu:20833|structure-specific recognition protein 1	gi|123229649|emb|CAM22342.1|	0	structure specific recognition protein 1 [Mus musculus]
mmu-miR-1197-5p	NR_033631	-21.1	0.999991	-21.97	146	-	-	gi|148665299|gb|EDK97715.1|	2.60E-76	mCG1050907 [Mus musculus]

mmu-miR-1197-5p	NM_022983	-21.5	0.999956	-20.56	145	GO:0016021,GO:0043227,GO:0005515,GO:0008289,GO:0045125,GO:0007267,GO:0043270,GO:0007204,GO:0030031,GO:0010740,GO:0007186,GO:0000165,	K04294|1|0.0|674|mmu:65086|lysophosphatidic acid receptor 3	gi|12667796|ref|NP_075359.1|	0	lysophosphatidic acid receptor 3 [Mus musculus]
mmu-miR-1197-5p	NM_182994	-21.6	1	-20.39	144	GO:0044464,GO:0032561,GO:0035556,	K07949|1|2e-103|378|ptr:736445|ADP-ribosylation factor-like 5A	gi|33695144|ref|NP_892039.1|	5.09E-99	ADP-ribosylation factor-like protein 5A [Mus musculus]
mmu-miR-1197-5p	NR_040524	-21.8	0.999998	-21.34	143	-	-	gi|148676412|gb|EDL08359.1|	1.29E-86	mCG145106 [Mus musculus]

mmu-miR-1197-5p	NM_207237	-22.8	0.999948	-20.1	141	GO:0031228,GO:0046872,GO:0015924,GO:0006029,GO:0019318,GO:0006487,	K01230|1|0.0|1176|mmu:230815|mannosyl-oligosaccharide alpha-1,2-mannosidase [EC:3.2.1.113]	gi|46402229|ref|NP_997120.1|	0	mannosidase, alpha, class 1C, member 1 [Mus musculus]
mmu-miR-1197-5p	NM_053179	-24.9	0.823358	NA	NA	GO:0044444,GO:0016765,GO:0070567,GO:0005975,GO:0008610,	K05304|1|0.0|729|mmu:94181|sialic acid synthase [EC:2.5.1.56 2.5.1.57]	gi|16716467|ref|NP_444409.1|	0	sialic acid synthase [Mus musculus]

mmu-miR-1197-5p	NM_001037723	-25.6	0.979584	NA	NA	GO:0031224,GO:0032559,GO:0043169,GO:0016829,GO:0009975,GO:0015980,GO:0007267,GO:0045471,GO:0045762,GO:0033762,GO:0032147,GO:0010863,GO:0007169,GO:0007188,GO:0048609,GO:00420	K08047|1|0.0|1958|mmu:11513|adenylate cyclase 7 [EC:4.6.1.1]	gi|83582788|ref|NP_001032813.1|	0	adenylate cyclase type 7 [Mus musculus]
mmu-miR-1197-5p	NM_001025947	-20.4	1	NA	NA	GO:0031966,GO:0042579,GO:0044431,GO:0017111,GO:0019899,GO:0032561,GO:0006915,GO:0009207,GO:0032501,GO:00002	K01528|1|0.0|1307|mmu:74006|dynamin GTPase [EC:3.6.5.5]	gi|148665002|gb|EDK97418.1|	0	dynamin 1-like, isoform CRA_e [Mus musculus]

mmu-miR-1198-3p	NM_001003670	-28.4	0.254952	-26	165	GO:0005882,GO:0005488,GO:0005198,GO:0030216,GO:0048729,GO:0045104,GO:0008283,GO:0007166,	K07605|1|0.0|837|mmu:406223|type II keratin, basic	gi|51092301|ref|NP_001003670.1|	0	type II keratin Kb14 [Mus musculus]
mmu-miR-1198-3p	NM_001024919	-33.5	0.022403	-27.66	164	GO:0000776,GO:0015630,GO:0000087,	-	gi|149052217|gb|EDM04034.1|	3.73E-63	hypothetical LOC287173 [Rattus norvegicus]
mmu-miR-1198-3p	NM_001033355	-28.6	0.73428	-26.01	170	GO:0060026,GO:0014022,	K09228|1|0.0|1403|mmu:243905|KRAB domain-containing zinc finger protein	gi|268607554|ref|NP_001028527.2|	0	zinc finger protein 568 isoform 1 [Mus musculus]
mmu-miR-1198-3p	NM_001033481	-31.7	0.254521	-25.38	163	GO:0043231,GO:0016020,GO:0031641,GO:0006355,GO:0014003,	-	gi|242247266|ref|NP_001028653.1|	0	myelin gene regulatory factor [Mus musculus]

mmu-miR-1198-3p	NM_001037098	-31.5	0.328098	-26.97	167	GO:0044451,GO:0008283,GO:0051259,	K10486|1|0.0|1112|mmu:67991|BTB/POZ domain-containing protein 14	gi|80861477|ref|NP_001032175.1|	0	nucleus accumbens-associated protein 2 isoform 2 [Mus musculus]
mmu-miR-1198-3p	NM_001039386	-29.5	0.301072	-26.51	172	GO:0043231,GO:0016020,	-	gi|86476082|ref|NP_001034475.1|	0	nasal embryonic luteinizing hormone-releasing hormone factor isoform A [Mus musculus]
mmu-miR-1198-3p	NM_001039387	-29.5	0.300487	-26.51	172	GO:0043231,GO:0016020,	-	gi|86476079|ref|NP_001034476.1|	0	nasal embryonic luteinizing hormone-releasing hormone factor isoform B [Mus musculus]
mmu-miR-1198-3p	NM_001039530	-35.4	0.0846	-33.59	180	GO:0016020,GO:0044424,GO:0016757,	K15261|1|0.0|3471|mmu:547253|poly [ADP-ribose] polymerase 10/14/15 [EC:2.4.2.30]	gi|166197700|ref|NP_001034619.2|	0	poly [ADP-ribose] polymerase 14 [Mus musculus]

mmu-miR-1198-3p	NM_001081011	-32.6	0.260792	-28.26	165	GO:0019866,GO:0044444,GO:0005488,GO:0035556,GO:0043087,GO:0006935,GO:00099	K07526|1|0.0|2057|mmu:14270|SLIT-ROBO Rho GTPase activating protein	gi|3413874|dbj|BAA32301.1|	0	KIAA0456 protein [Homo sapiens]
mmu-miR-1198-3p	NM_001099288	-33.9	0.077336	-25.19	165	GO:0044444,GO:0008047,GO:0035556,GO:0009966,	K13709|1|3e-20|102|dre:795752|Rho GTPase-activating protein 5!K05732|2|3e-20|102|xtr:100498460|glucocorticoid receptor DNA-binding factor 1!K07526|5|6e-19|97.8|tsp:Tsp_03919|SLIT-ROBO Rho GTPase activating protein	gi|151108451|ref|NP_001092758.1|	0	rho GTPase-activating protein 44 isoform 1 [Mus musculus]

mmu-miR-1198-3p	NM_001159572	-28.8	0.562004	-52.25	330	GO:0031224,GO:0004871,GO:0023052,	-	gi|47847392|dbj|BAD21368.1|	2.14E-168	mFLJ00041 protein [Mus musculus]
mmu-miR-1198-3p	NM_001163144	-33.4	0.140985	-28.9	165	GO:0016023,GO:0044431,GO:0031224,GO:0044421,GO:0004175,GO:0005488,GO:0001501,GO:0035107,GO:0006518,GO:0001999,GO:0048513,GO:0001816,GO:0007154,GO:0007169,GO:0003002,GO:0019058,GO:0016486,GO:0048565,GO:00352	K08654|1|0.0|1915|mmu:18552|proprotein convertase subtilisin/kexin type 5 [EC:3.4.21.-]	gi|253314509|ref|NP_001156616.1|	0	proprotein convertase subtilisin/kexin type 5 isoform 2 preproprotein [Mus musculus]
mmu-miR-1198-3p	NM_001167872	-28.6	0.734606	-26.01	170	GO:0060026,GO:0014022,	K09228|1|0.0|1403|mmu:243905|KRAB domain-containing zinc finger protein	gi|268607554|ref|NP_001028527.2|	0	zinc finger protein 568 isoform 1 [Mus musculus]

mmu-miR-1198-3p	NM_001167873	-28.6	0.734466	-26.01	170	GO:0060026,GO:0014022,	K09228|1|0.0|1397|mmu:243905|KRAB domain-containing zinc finger protein	gi|268607558|ref|NP_001161345.1|	0	zinc finger protein 568 isoform 2 [Mus musculus]
mmu-miR-1198-3p	NM_001177374	-28.8	0.728782	-25.26	172	GO:0043234,GO:0031981,GO:0016020,GO:0046914,GO:0019787,GO:0019941,GO:0007127,GO:0048232,	K10626|1|0.0|3410|mmu:224826|E3 ubiquitin-protein ligase UBR2 [EC:6.3.2.19]	gi|293651567|ref|NP_001170845.1|	0	E3 ubiquitin-protein ligase UBR2 isoform 2 [Mus musculus]
mmu-miR-1198-3p	NM_001177654	-29.5	0.299607	-26.51	172	GO:0043231,GO:0016020,	-	gi|294997247|ref|NP_001171125.1|	0	nasal embryonic luteinizing hormone-releasing hormone factor isoform D [Mus musculus]
mmu-miR-1198-3p	NM_001177655	-29.5	0.291638	-26.51	172	GO:0043231,GO:0016020,	-	gi|148676249|gb|EDL08196.1|	0	nasal embryonic LHRH factor, isoform CRA_d [Mus musculus]

mmu-miR-1198-3p	NM_001190483	-33.4	0.16392	-28.9	165	GO:0016023,GO:0044431,GO:0044421,GO:0004175,GO:0005488,GO:0001501,GO:0035107,GO:0006518,GO:0001999,GO:0048513,GO:0001816,GO:0007154,GO:0007169,GO:0003002,GO:0019058,GO:0016486,GO:0048565,GO:0035295,	K08654|1|0.0|3801|rno:116548|proprotein convertase subtilisin/kexin type 5 [EC:3.4.21.-]	gi|299523019|ref|NP_001177412.1|	0	proprotein convertase subtilisin/kexin type 5 isoform 1 preproprotein [Mus musculus]
mmu-miR-1198-3p	NM_001252472	-33.8	0.067986	-30.9	180	GO:0031224,GO:0004871,GO:0023052,	K06511|1|7e-62|240|mmu:12523|CD84 antigen	gi|357197147|ref|NP_001239401.1|	2.33E-68	SLAM family member 5 isoform 2 precursor [Mus musculus]
mmu-miR-1198-3p	NM_001267872	-33.8	0.261294	-27.16	163	-	-	-	-	-

mmu-miR-1198-3p	NM_007566	-28.9	0.908733	-26.35	153	-	K10586|1|0.0|8523|mmu:12211|baculoviral IAP repeat-containing protein 6 (apollon) [EC:6.3.2.19]	gi|380876899|sp|O88738.2|BIRC6_MOUSE	0	RecName: Full=Baculoviral IAP repeat-containing protein 6; AltName: Full=BIR repeat-containing ubiquitin-conjugating enzyme; Short=BRUCE; AltName: Full=Ubiquitin-conjugating BIR domain enzyme apollon; Short=APOLLON
mmu-miR-1198-3p	NM_009074	-30.5	0.327234	-26.38	166	GO:0031224,GO:0032432,GO:0004871,GO:0004713,GO:0032559,GO:0051707,GO:0007167,GO:0032501,GO:0006464,	K05100|1|0.0|2660|mmu:19882|macrophage-stimulating 1 receptor [EC:2.7.10.1]	gi|254826769|ref|NP_033100.2|	0	macrophage-stimulating protein receptor precursor [Mus musculus]
mmu-miR-1198-3p	NM_009567	-26.4	0.803859	-26.04	160	GO:0043231,GO:0046872,GO:0003676,GO:0006351,	K09228|1|0.0|1398|mmu:22755|KRAB domain-containing zinc finger protein	gi|6677629|ref|NP_033593.1|	0	zinc finger protein 235 [Mus musculus]

mmu-miR-1198-3p	NM_009891	-30.4	0.204935	-25.97	167	GO:0043005,GO:0043231,GO:0044297,GO:0044444,GO:0016413,GO:0043169,GO:0060024,GO:0007611,GO:0031175,GO:0007528,GO:0042136,GO:0008344,	K00623|1|0.0|1243|mmu:12647|choline O-acetyltransferase [EC:2.3.1.6]	gi|45476575|ref|NP_034021.1|	0	choline O-acetyltransferase [Mus musculus]
mmu-miR-1198-3p	NM_016854	-33.6	0.052766	-29.66	176	GO:0044444,GO:0005515,GO:0004721,GO:0005980,GO:0006886,GO:0005978,	K07189|1|2e-179|630|mmu:53412|protein phosphatase 1, regulatory (inhibitor)	gi|33468957|ref|NP_058550.1|	1.56E-178	protein phosphatase 1 regulatory subunit 3C [Mus musculus]

mmu-miR-1198-3p	NM_016972	-34.1	0.0717	-28.85	168	GO:0016021,GO:0044459,GO:0044424,GO:0015171,GO:0008324,GO:0003823,GO:0019752,GO:0055080,GO:0002376,GO:0042221,GO:0006807,GO:0007599,GO:0006812,GO:00	K13781|1|0.0|948|mmu:50934|solute carrier family 7 (L-type amino acid transporter), member 8	gi|8394325|ref|NP_058668.1|	0	large neutral amino acids transporter small subunit 2 [Mus musculus]
mmu-miR-1198-3p	NM_019780	-30.4	0.056328	-27.94	153	GO:0031090,GO:0043231,GO:0043169,GO:0016791,GO:0006810,GO:0006796,	K07095|1|1e-102|373|ssc:100737886|	gi|71042152|pdb|1Z2W|A	4.94E-103	Chain A, Crystal Structure Of Mouse Vps29 Complexed With Mn2+
mmu-miR-1198-3p	NM_020276	-29.5	0.294305	-26.51	172	GO:0043231,GO:0016020,	-	gi|10190662|ref|NP_064672.2|	0	nasal embryonic luteinizing hormone-releasing hormone factor isoform C [Mus musculus]

mmu-miR-1198-3p	NM_026495	-31.5	0.306198	-26.97	167	GO:0044451,GO:0008283,GO:0051259,	K10486|1|0.0|1112|mmu:67991|BTB/POZ domain-containing protein 14	gi|80861477|ref|NP_001032175.1|	0	nucleus accumbens-associated protein 2 isoform 2 [Mus musculus]
mmu-miR-1198-3p	NM_026613	-30	0.174158	-25.19	163	-	-	gi|205361118|ref|NP_080889.3|	8.57E-89	coiled-coil domain-containing protein 34 [Mus musculus]
mmu-miR-1198-3p	NM_026700	-28.7	0.094984	-25.39	176	-	-	gi|15022807|ref|NP_080976.1|	1.09E-83	protein dopey-2 isoform 2 [Mus musculus]
mmu-miR-1198-3p	NM_027293	-28.7	0.726489	-25.39	176	GO:0012505,GO:0006892,GO:0032501,GO:0006996,	-	gi|62243808|ref|NP_081569.1|	0	protein dopey-2 isoform 1 [Mus musculus]
mmu-miR-1198-3p	NM_028637	-36.5	0.006256	-31.71	181	-	-	gi|198278569|ref|NP_082913.1|	1.93E-97	uncharacterized protein C20orf196 homolog [Mus musculus]
mmu-miR-1198-3p	NM_028673	-33.8	0.26221	-27.16	163	-	-	gi|255982598|ref|NP_082949.1|	0	DBF4-type zinc finger-containing protein 2 homolog [Mus musculus]
mmu-miR-1198-3p	NM_028732	-28.8	0.562234	-52.25	330	GO:0031224,GO:0004871,GO:0023052,	-	gi|47847392|dbj|BAD21368.1|	5.27E-167	mFLJ00041 protein [Mus musculus]

mmu-miR-1198-3p	NM_029888	-30.7	0.448577	-26.25	165	-	K09228|1|8e-66|254|ptr:455992|KRAB domain-containing zinc finger protein	gi|148667920|gb|EDL00337.1|	0	mCG133876, isoform CRA_d [Mus musculus]
mmu-miR-1198-3p	NM_030058	-29.4	0.060107	-26.5	168	GO:0004866,GO:0010466,	K03909|1|1e-08|60.8|bta:508763|tissue factor pathway inhibitor	gi|148674462|gb|EDL06409.1|	3.61E-80	serine protease inhibitor, Kunitz type 4 [Mus musculus]
mmu-miR-1198-3p	NM_030206	-32.3	0.076004	-26.99	175	GO:0044297,GO:0042995,GO:0044424,GO:0016209,GO:0005506,GO:0022892,GO:0006950,GO:0015669,	K00669|1|1e-17|92.4|ssc:100518330|arylalkylamine N-acetyltransferase [EC:2.3.1.87]!K13826|2|1e-13|79.7|gga:100858080|hemoglobin subunit	gi|148702641|gb|EDL34588.1|	2.70E-127	cytoglobin [Mus musculus]

mmu-miR-1198-3p	NM_054085	-30.4	0.43436	-27.65	167	GO:0043231,GO:0004672,GO:0032559,GO:0006464,GO:0048513,	K08868|1|0.0|2993|mmu:116904|alpha-kinase	gi|117938324|ref|NP_473426.2|	0	alpha-protein kinase 3 [Mus musculus]
mmu-miR-1198-3p	NM_133643	-27.3	0.916251	-68.75	472	GO:0044424,GO:0004871,GO:0032813,GO:0042476,GO:0008544,GO:0048732,GO:0007243,GO:0048869,	-	gi|127139913|ref|NP_598398.3|	4.26E-116	ectodysplasin-A receptor-associated adapter protein isoform 1 [Mus musculus]
mmu-miR-1198-3p	NM_145403	-25.8	0.740649	-42.48	323	-	K09635|1|0.0|852|mmu:214523|transmembrane protease, serine 4	gi|21703806|ref|NP_663378.1|	0	transmembrane protease serine 4 [Mus musculus]
mmu-miR-1198-3p	NM_146078	-28.8	0.728782	-25.26	172	GO:0043234,GO:0031981,GO:0016020,GO:0046914,GO:0019787,GO:0019941,GO:0007127,GO:0048232,	K10626|1|0.0|3386|mmu:224826|E3 ubiquitin-protein ligase UBR2 [EC:6.3.2.19]	gi|58615693|ref|NP_666190.2|	0	E3 ubiquitin-protein ligase UBR2 isoform 1 [Mus musculus]

mmu-miR-1198-3p	NM_173398	-35.2	0.020366	-30.91	188	GO:0031224,GO:0001608,GO:0035588,	K08441|1|3e-173|609|mmu:229323|G protein-coupled receptor 171	gi|27734176|ref|NP_775574.1|	2.29E-172	probable G-protein coupled receptor 171 [Mus musculus]
mmu-miR-1198-3p	NM_175003	-33.9	0.078527	-25.19	165	GO:0005622,GO:0050794,	K13709|1|3e-20|102|dre:795752|Rho GTPase-activating protein 5!K05732|2|3e-20|102|xtr:100498460|glucocorticoid receptor DNA-binding factor 1!K07526|5|6e-19|97.8|tsp:Tsp_03919|SLIT-ROBO Rho GTPase activating protein	gi|34328406|ref|NP_778168.2|	0	rho GTPase-activating protein 44 isoform 2 [Mus musculus]

mmu-miR-1198-3p	NM_175207	-34.4	0.034988	-29.09	170	-	-	gi|148686700|gb|EDL18647.1|	3.64E-177	ankyrin repeat domain 9, isoform CRA_a [Mus musculus]
mmu-miR-1198-3p	NM_183031	-33.7	0.056596	-29.86	178	GO:0016021,GO:0001608,GO:0035588,GO:0002312,	K04305|1|0.0|670|mmu:321019|Epstein-Barr virus induced gene 2	gi|88853593|ref|NP_898852.2|	0	G-protein coupled receptor 183 [Mus musculus]
mmu-miR-1198-3p	NM_199011	-26.3	0.890827	-26.2	160	GO:0016020,GO:0016604,GO:0043232,GO:0044444,GO:0016301,GO:0032559,GO:0043169,GO:0019899,GO:0008134,GO:0007243,GO:0007599,GO:0010033,GO:0006796,GO:0007186,GO:0001775,	K00901|1|0.0|1824|mmu:110524|diacylglycerol kinase [EC:2.7.1.107]	gi|39841023|ref|NP_950176.1|	0	diacylglycerol kinase theta [Mus musculus]
mmu-miR-1198-3p	NR_029460	-27.9	0.655058	-26.07	162	GO:0031225,GO:0048667,	K07522|1|2e-13|79.3|ecb:100060362|netrin G1	gi|148676466|gb|EDL08413.1|	1.30E-76	mCG145104 [Mus musculus]

mmu-miR-1198-3p	NR_045455	-27.9	0.577398	-25.85	162	-	-	gi|148691961|gb|EDL23908.1|	8.64E-23	mCG147816 [Mus musculus]
mmu-miR-1198-3p	NM_181047	-24.7	0.789625	-47.32	323	-	-	gi|148698035|gb|EDL29982.1|	4.86E-49	RIKEN cDNA A330049M08, isoform CRA_b [Mus musculus]
mmu-miR-1198-3p	NM_009378	-21.8	0.999994	-46.62	322	GO:0016021,GO:0044421,GO:0046872,GO:0004872,GO:0030194,GO:0010212,GO:0023052,GO:0002376,GO:0002237,GO:0030162,GO:0007275,GO:0022414,GO:00105	K03907|1|0.0|1117|mmu:21824|thrombomodulin	gi|6678339|ref|NP_033404.1|	0	thrombomodulin precursor [Mus musculus]

mmu-miR-1198-3p	NM_176940	-23.4	0.999986	-43.21	301	GO:0032559,	K11887|1|6e-24|115|ame:409686|proteasomal ATPase-associated factor 1!K11127|2|4e-16|89.4|spu:584519|telomerase protein component 1!K14555|4|3e-10|70.1|ame:724596|U3 small nucleolar RNA-associated protein 13!K14855|5|3e-	gi|295391943|ref|NP_795914.3|	0	NACHT and WD repeat domain-containing protein 1 [Mus musculus]
mmu-miR-1198-3p	NM_001134457	-21.6	1	-25.08	174	-	-	gi|197333803|ref|NP_001127929.1|	0	uncharacterized protein LOC385658 [Mus musculus]

mmu-miR-1198-3p	NM_144731	-26.3	0.877657	-21.07	172	GO:0012505,GO:0043231,GO:0031224,GO:0030246,GO:0008376,GO:0006486,	K00710|1|0.0|1226|rno:29750|polypeptide N-acetylgalactosaminyltransferase [EC:2.4.1.41]	gi|269784707|ref|NP_653332.3|	0	N-acetylgalactosaminyltransferase 7 isoform 1 [Mus musculus]
mmu-miR-1198-3p	NM_175318	-27	0.846306	-21.38	170	-	K15193|1|0.0|868|mmu:101685|protein SPT2	gi|26326843|dbj|BAC27165.1|	0	unnamed protein product [Mus musculus]
mmu-miR-1198-3p	NM_145558	-20.4	0.999998	-25.92	169	GO:0019866,GO:0005759,GO:0016616,GO:0000166,GO:0005504,GO:0016836,GO:0016408,GO:0009062,	K07509|1|0.0|934|mmu:231086|acetyl-CoA acyltransferase [EC:2.3.1.16]	gi|148705321|gb|EDL37268.1|	0	mCG11629, isoform CRA_d [Mus musculus]
mmu-miR-1198-3p	NR_045950	-26.6	0.746507	-24.72	168	-	-	-	-	-
mmu-miR-1198-3p	NR_001592	-25.6	0.818893	-20.96	167	-	-	gi|51132|emb|CAA41173.1|	5.90E-74	unnamed protein product [Mus musculus]

mmu-miR-1198-3p	NM_172539	-26.2	0.695593	-23.76	166	GO:0046914,GO:0004175,GO:0019538,	K08778|1|0.0|809|mmu:215095|astacin-like metalloendopeptidase [EC:3.4.-.-]	gi|76364089|sp|Q6HA09.3|ASTL_MOUSE	0	RecName: Full=Astacin-like metalloendopeptidase; AltName: Full=Oocyte astacin; AltName: Full=Ovastacin; AltName: Full=Sperm acrosomal SLLP1-binding protein; Flags: Precursor
mmu-miR-1198-3p	NM_201607	-29	0.385936	-23.44	165	GO:0044444,GO:0043169,GO:0004114,GO:0050796,GO:0006195,	K01120|1|0.0|1211|mmu:110385|3',5'-cyclic-nucleotide phosphodiesterase [EC:3.1.4.17]	gi|148696916|gb|EDL28863.1|	0	phosphodiesterase 4C, cAMP specific, isoform CRA_a [Mus musculus]

mmu-miR-1198-3p	NR_001461	-23.3	1	-20.64	164	-	K11849|1|6e-55|213|acs:100555596|ubiquitin carboxyl-terminal hydrolase 25/28 [EC:3.1.2.15]!K06478|4|4e-43|182|acs:100563672|protein tyrosine phosphatase, receptor type, C [EC:3.1.3.4	gi|209553980|gb|AAA39398.2|	0	ORF2 [Mus musculus domesticus]

mmu-miR-1198-3p	NM_181412	-25.5	0.971631	-21.28	163	GO:0043231,GO:0003676,GO:0005515,GO:0043169,	K15107|1|4e-17|92.4|hmg:100201695|solute carrier family 25 (mitochondrial glutamate transporter), member 18/22!K14303|2|3e-12|76.3|hmg:100204852|nuclear pore complex protein Nup160	gi|31088890|ref|NP_852077.1|	0	zinc finger BED domain-containing protein 4 [Mus musculus]
mmu-miR-1198-3p	NR_033228	-29.3	0.588176	-20.53	162	GO:0043231,GO:0044444,GO:0016859,GO:0005515,GO:0044409,GO:0006310,GO:0044267,GO:0001775,GO:0006260,GO:0002376,GO:0030154,GO:0050792,GO:00075	K03767|1|3e-82|188|mmu:268373|peptidyl-prolyl cis-trans isomerase A (cyclophilin A) [EC:5.2.1.8]	gi|392353052|ref|XP_002728136.2|	7.57E-87	PREDICTED: peptidyl-prolyl cis-trans isomerase A-like [Rattus norvegicus]

mmu-miR-1198-3p	NR_037996	-26.4	0.481229	-21.78	161	GO:0005694,GO:0005488,GO:0048523,GO:0007165,GO:0006351,	K09283|1|7e-12|72.8|mmu:15364|high mobility group AT-hook protein 2	gi|12851702|dbj|BAB29137.1|	7.57E-17	unnamed protein product [Mus musculus]
mmu-miR-1198-3p	NR_073368	-25.3	0.387944	-20.44	160	-	-	-	-	-
mmu-miR-1198-3p	NM_198616	-23.6	0.998533	-21.86	159	GO:0005815,GO:0043231,GO:0005488,GO:0045596,GO:0016049,GO:0006355,	K04502|1|4e-18|95.1|mmu:14283|fos-like antigen 1	gi|395852409|ref|XP_003798731.1|	5.08E-52	PREDICTED: coiled-coil domain-containing protein 85B [Otolemur garnettii]
mmu-miR-1198-3p	NM_199065	-23.2	0.999451	-21.78	158	GO:0031224,GO:0048667,	K06839|1|3e-27|125|dme:Dmel_CG8355|slit 2	gi|50511177|dbj|BAD32574.1|	0	mKIAA1910 protein [Mus musculus]
mmu-miR-1198-3p	NR_040658	-20.4	0.999902	-25.46	157	-	-	gi|148689457|gb|EDL21404.1|	9.75E-58	mCG144705 [Mus musculus]
mmu-miR-1198-3p	NR_045276	-25.7	0.675888	-22.62	156	-	-	gi|148708474|gb|EDL40421.1|	1.44E-28	RIKEN cDNA 8430429K09 [Mus musculus]
mmu-miR-1198-3p	NR_073364	-24.2	0.98965	-20.18	155	-	-	-	-	-

mmu-miR-1198-3p	NM_201600	-25.3	0.990253	-21.67	154	GO:0030425,GO:0044297,GO:0015629,GO:0044444,GO:0008092,GO:0017111,GO:0005083,GO:0035254,GO:0032559,GO:0006810,GO:0019222,GO:0008104,	K10357|1|0.0|3460|mmu:17919|myosin V	gi|46399202|ref|NP_963894.1|	0	unconventional myosin-Vb [Mus musculus]
mmu-miR-1198-3p	NM_173422	-23.5	0.999129	-20.86	153	GO:0044444,GO:0043231,GO:0048029,GO:0006950,GO:0016337,	K10065|1|1e-119|432|mmu:239447|collectin sub-family member 10	gi|226053018|ref|NP_775598.2|	8.61E-119	collectin-10 precursor [Mus musculus]
mmu-miR-1198-3p	NM_178911	-25.2	0.782654	-20.05	152	GO:0031224,GO:0005488,GO:0004620,GO:0006629,	-	gi|30725764|ref|NP_849242.1|	0	phospholipase D4 [Mus musculus]
mmu-miR-1198-3p	NR_045988	-27.9	0.636032	-24.54	151	-	-	gi|148681499|gb|EDL13446.1|	3.50E-47	mCG144644 [Mus musculus]

mmu-miR-1198-3p	NM_177767	-24.4	0.996006	-22.13	150	GO:0046914,GO:0016705,GO:0019842,GO:0016701,GO:0008152,	-	gi|147901538|ref|NP_808435.3|	0	2-oxoglutarate and iron-dependent oxygenase domain-containing protein 1 isoform 1 [Mus musculus]
mmu-miR-1198-3p	NM_183276	-21.6	1	-20.85	149	-	K07295|1|4e-26|122|dpe:Dper_GL19043|nuclear receptor subfamily 2 group A!K11887|2|3e-09|67.0|ame:409686|proteasomal ATPase-associated factor 1!K10342|5|7e-08|62.4|mcc:710401|WD repeat and SOCS box-containing protein 2	gi|254911027|ref|NP_899099.2|	0	neurobeachin-like protein 2 [Mus musculus]

mmu-miR-1198-3p	NM_021416	-23.3	0.9991	-21.1	148	-	K10352|1|6e-15|84.7|tca:663025|myosin heavy chain	gi|188219653|ref|NP_067391.3|	0	protein FAM184B [Mus musculus]
mmu-miR-1198-3p	NM_207277	-22.9	0.997241	-20.15	147	-	-	gi|46402311|ref|NP_997160.1|	1.88E-142	myb/SANT-like DNA-binding domain-containing protein 1 [Mus musculus]
mmu-miR-1198-3p	NM_177371	-26.8	0.894133	-23.1	146	GO:0016021,GO:0044421,GO:0032813,GO:0006915,GO:0050707,GO:0043170,GO:0002376,GO:0007249,	K05478|1|1e-153|545|mmu:326623|tumor necrosis factor ligand superfamily member	gi|238776819|ref|NP_796345.3|	8.99E-153	tumor necrosis factor ligand superfamily member 15 [Mus musculus]
mmu-miR-1198-3p	NR_040443	-20.2	1	-20.42	145	-	-	gi|148697976|gb|EDL29923.1|	1.61E-27	mCG1049130 [Mus musculus]
mmu-miR-1198-3p	NR_045728	-26.5	0.216092	-20.92	144	GO:0044464,GO:0005088,GO:0019222,GO:0007266,	-	gi|354491243|ref|XP_003507765.1|	1.42E-29	PREDICTED: rho guanine nucleotide exchange factor 40 [Cricetulus griseus]

mmu-miR-1198-3p	NM_011276	-20.7	1	-22.72	143	GO:0043231,GO:0043234,GO:0003712,GO:0046914,GO:0019787,GO:0006357,GO:0032446,GO:0019941,GO:0043392,GO:0009048,	K15692|1|4e-14|82.8|xtr:493377|E3 ubiquitin-protein ligase RNF13 [EC:6.3.2.19]!K15706|5|2e-13|80.9|dre:100333827|E3 ubiquitin-protein ligase RNF167 [EC:6.3.2.19]	gi|47078289|ref|NP_035406.3|		0	E3 ubiquitin-protein ligase RLIM [Mus musculus]
mmu-miR-1198-3p	NM_011374	-21.8	1	-20.3	142	GO:0031228,GO:0008373,GO:0006687,GO:0009408,GO:0008283,GO:0006464,	K03371|1|0.0|739|mmu:20449|alpha-N-acetyl-neuraminate alpha-2,8-sialyltransferase (sialyltransferase 8A) [EC:2.4.99.	gi|70980543|ref|NP_035504.2|		0	alpha-N-acetylneuraminide alpha-2,8-sialyltransferase [Mus musculus]

mmu-miR-1198-3p	NR_038006	NA	NA	-23.38	157	GO:0006811,	K14697|1|7e-53|210|cfa:488599|solute carrier family 30 (zinc transporter), member 10	gi|148681119|gb|EDL13066.1|	1.19E-61	RIKEN cDNA 9630028B13 [Mus musculus]
mmu-miR-1198-3p	NM_026150	NA	NA	-24.26	150	-	-	gi|13385654|ref|NP_080426.1|	1.19E-90	uncharacterized protein C5orf52 homolog [Mus musculus]
mmu-miR-1198-3p	NM_028390	NA	NA	-23.3	148	GO:0043231,GO:0015629,GO:0044444,GO:0008092,GO:0031106,GO:0000087,GO:00073	K03243|1|1e-06|57.8|tca:662115|translation initiation factor 5B	gi|148693327|gb|EDL25274.1|	0	anillin, actin binding protein (scraps homolog, Drosophila), isoform CRA_b [Mus musculus]
mmu-miR-1198-3p	NM_028763	NA	NA	-22.02	141	GO:0043229,GO:0006325,	K11453|1|4e-173|610|mmu:494448|chromobox protein 6	gi|61657984|ref|NP_083039.2|	2.77E-172	chromobox protein homolog 6 [Mus musculus]

mmu-miR-1198-3p	NM_001037755	-23.6	0.962671	NA	NA	GO:0031981,GO:0044459,GO:0031252,GO:0042995,GO:0030674,GO:0019904,GO:0007265,GO:0030035,GO:0048667,GO:00071	K05627|1|0.0|952|hsa:10458|BAI1-associated protein 2	gi|262231776|ref|NP_001032844.2|	0	brain-specific angiogenesis inhibitor 1-associated protein 2 isoform a [Mus musculus]
mmu-miR-1198-3p	NM_001164640	-21.6	0.999838	NA	NA	-	K14480|1|8e-114|412|mmu:75761|apolipoprotein	gi|257467561|ref|NP_083695.2|	5.53E-113	apolipoprotein L 7a [Mus musculus]
mmu-miR-1198-3p	NM_025660	-21.6	0.997425	NA	NA	-	-	gi|13385124|ref|NP_079936.1|	1.02E-154	RIB43A-like with coiled-coils protein 1 [Mus musculus]

mmu-miR-1198-3p	NM_001146053	-21	0.998658	NA	NA	GO:0043231,GO:0012505,GO:0000166,GO:0003712,GO:0016903,GO:0004672,GO:0045897,GO:0006913,GO:0022603,GO:0006468,GO:0012502,GO:0048869,GO:0051704,GO:0048646,GO:0043066,GO:0032501,GO:00	-	gi|225690589|ref|NP_058561.2|	1.72E-133	oxidoreductase HTATIP2 isoform a precursor [Mus musculus]
mmu-miR-1198-3p	NM_010709	-22	0.998911	NA	NA	GO:0044424,GO:0008135,GO:0004871,GO:0006412,GO:0015031,	K15027|1|0.0|1030|mmu:16865|translation initiation factor 2D	gi|148707770|gb|EDL39717.1|	0	ligatin, isoform CRA_c [Mus musculus]

mmu-miR-1198-3p	NM_008138	-24.3	0.919216	NA	NA	GO:0005815,GO:0043231,GO:0031234,GO:0032561,GO:0005102,GO:0017111,GO:0060089,GO:0032403,GO:0035588,GO:0031280,GO:0007599,GO:0008283,GO:0009207,GO:0007188,GO:0050804,GO:0032413,GO:0001775,GO:0031667,GO:00001	K04630|1|0.0|714|mmu:14678|guanine nucleotide-binding protein G(i) subunit alpha	gi|332216026|ref|XP_003257143.1|	0	PREDICTED: guanine nucleotide-binding protein G(i) subunit alpha-2 isoform 1 [Nomascus leucogenys]
mmu-miR-1198-3p	NM_015807	-21.7	0.952921	NA	NA	GO:0043231,GO:0044444,GO:0043169,GO:0008252,GO:0000166,GO:0006796,GO:0006213,GO:0006244,GO:0009112,GO:00061	K01081|1|3e-115|414|mmu:50773|5'-nucleotidase [EC:3.1.3.5]	gi|7657031|ref|NP_056622.1|	2.05E-114	5'(3')-deoxyribonucleotidase, cytosolic type [Mus musculus]

mmu-miR-1198-3p	NM_177175	-20.2	1	NA	NA	GO:0031224,	-	gi|260274390|ref|NP_796149.2|	2.23E-118	transmembrane protein 215 [Mus musculus]
mmu-miR-1198-3p	NM_172607	-23.5	0.956016	NA	NA	GO:0043231,GO:0044444,GO:0016763,GO:0019357,GO:0019359,GO:0006950,GO:0009820,	K00763|1|0.0|986|mmu:223646|nicotinate phosphoribosyltransferase [EC:2.4.2.11]	gi|148697546|gb|EDL29493.1|	0	nicotinate phosphoribosyltransferase domain containing 1, isoform CRA_a [Mus musculus]
mmu-miR-1198-3p	NM_001159529	-21.8	0.9853	NA	NA	GO:0005743,GO:0022900,	K02270|1|5e-73|275|mmu:20463|cytochrome c oxidase subunit subunit	gi|148706604|gb|EDL38551.1|	1.37E-90	cytochrome c oxidase subunit VIIa polypeptide 2-like, isoform CRA_d [Mus musculus]

mmu-miR-1198-3p	NM_031199	-20.8	1	NA	NA	GO:0043231,GO:0016021,GO:0044421,GO:0060089,GO:0004712,GO:0070851,GO:0009611,GO:0042221,GO:0006874,GO:0043523,GO:0007088,GO:0008284,GO:0007176,GO:0048646,GO:0043066,GO:00001	K08774|1|1e-70|269|mmu:21802|transforming growth factor, alpha	gi|224420|prf||1103301A	9.45E-80	transforming growth factor alpha

mmu-miR-1198-3p	NM_023816	-20.8	1	NA	NA	-	K10645|1|3e-16|89.4|aga:AgaP_AGAP011932|E3 ubiquitin-protein ligase mind-bomb [EC:6.3.2.19]!K12460|2|8e-15|84.3|tca:100141654|ankyrin repeat-rich membrane spanning protein	gi|125628652|ref|NP_076305.2|	0	ankyrin repeat domain 36 [Mus musculus]
mmu-miR-1198-3p	NM_016709	-24.9	0.68324	NA	NA	GO:0044429,GO:0003729,GO:0016836,GO:0000956,GO:0009063,	K05607|1|4e-154|545|mmu:11992|methylglutaconyl-CoA hydratase [EC:4.2.1.18]	gi|116268115|ref|NP_057918.2|	2.80E-153	methylglutaconyl-CoA hydratase, mitochondrial precursor [Mus musculus]

mmu-miR-1198-5p	NM_001001883	-30.1	0.723836	-26.36	166	GO:0044424,GO:0019787,GO:0016567,	K12168|1|0.0|2968|mmu:329152|E3 ubiquitin-protein ligase HECW2 [EC:6.3.2.19]	gi|49457861|ref|NP_001001883.1|	0	E3 ubiquitin-protein ligase HECW2 isoform 1 [Mus musculus]
mmu-miR-1198-5p	NM_001011527	-29.2	0.114174	-27.77	168	GO:0031224,GO:0004930,GO:0007166,GO:0007606,	K04257|1|4e-168|590|mmu:259051|olfactory	gi|58372132|ref|NP_001011527.1|	0	olfactory receptor 503 [Mus musculus]
mmu-miR-1198-5p	NM_001013761	-28	0.675344	-25.11	163	GO:0043231,GO:0005488,GO:0006259,	-	gi|71274117|ref|NP_001013783.1|	0	geminin coiled-coil domain-containing protein 1 [Mus musculus]
mmu-miR-1198-5p	NM_001024468	-26.7	0.96851	-28.37	155	GO:0043231,GO:0044444,GO:0005515,GO:0004084,GO:0008652,GO:0009063,	K00826|1|0.0|931|mmu:12035|branched-chain amino acid aminotransferase [EC:2.6.1.42]	gi|66792792|ref|NP_001019639.1|	0	branched-chain-amino-acid aminotransferase, cytosolic isoform 1 [Mus musculus]

mmu-miR-1198-5p	NM_001045520	-30.3	0.298226	-26.34	164	-	K11137|1|9e-67|256|api:100163744|telomere length regulation protein!K12471|3|6e-65|250|isc:IscW_ISCW008441|epsin	gi|154240730|ref|NP_001038985.2|	0	clathrin interactor 1 [Mus musculus]
mmu-miR-1198-5p	NM_001080817	-28.7	0.500052	-25.09	167	GO:0043231,GO:0046914,GO:0003676,	K09228|1|2e-36|155|dre:796272|KRAB domain-containing zinc finger protein	gi|124249099|ref|NP_001074286.1|	0	PR domain zinc finger protein 10 precursor [Mus musculus]
mmu-miR-1198-5p	NM_001081213	-33	0.179402	-32.92	159	GO:0031090,GO:0043231,GO:0031224,GO:0043169,GO:0070011,GO:0019538,GO:00226	-	gi|148709742|gb|EDL41688.1|	0	mCG124990, isoform CRA_b [Mus musculus]

mmu-miR-1198-5p	NM_001083618	-32.7	0.10293	-28.95	164	GO:0005815,GO:0042995,GO:0016881,GO:0032559,GO:0043412,	K00273|1|2e-39|165|ame:551507|D-amino-acid oxidase [EC:1.4.3.3]!K05755|2|4e-25|117|tgu:100190205|actin related protein 2/3 complex, subunit 4!K06047|4|8e-18|93.6|bfo:BRAFLDRAFT_65827|tubulin---tyrosine ligase [EC:6.3.2.2	gi|148674057|gb|EDL06004.1|	0	tubulin tyrosine ligase like family 9, isoform CRA_a [Mus musculus]
mmu-miR-1198-5p	NM_001083917	-25.5	0.958831	-40.6	318	GO:0031224,GO:0005272,GO:0006814,GO:0048731,GO:0007600,GO:0006935,GO:0034220,	K04847|1|4e-125|450|ptr:466810|voltage-gated sodium channel type III beta	gi|50510815|dbj|BAD32393.1|	5.35E-152	mKIAA1158 protein [Mus musculus]

mmu-miR-1198-5p	NM_001103366	-26.9	0.663818	-25.14	168	GO:0031224,GO:0004888,GO:0007166,	K04613|1|0.0|1292|mmu:22300|vomeronasal 2 receptor	gi|157074221|ref|NP_001096836.1|	0	vomeronasal receptor Vmn2r87 precursor [Mus musculus]
mmu-miR-1198-5p	NM_001109751	-29.4	0.364003	-27.78	167	GO:0031224,GO:0043005,GO:0005488,GO:0045596,GO:0007417,GO:0016337,GO:0050804,GO:0006935,GO:0007409,	K06762|1|0.0|1363|mmu:269784|contactin 4	gi|157909805|ref|NP_766592.2|	0	contactin-4 isoform 2 precursor [Mus musculus]
mmu-miR-1198-5p	NM_001110212	-32.9	0.196468	-28.75	165	GO:0050905,GO:0019538,GO:0001894,GO:0021515,GO:0050879,GO:0040007,GO:0007626,GO:0007169,GO:0051320,	-	gi|148708223|gb|EDL40170.1|	0	trinucleotide repeat containing 15, isoform CRA_g [Mus musculus]

mmu-miR-1198-5p	NM_001110826	-27.7	0.843159	-27.5	157	GO:0031981,GO:0031332,GO:0044444,GO:0003676,GO:0042623,GO:0003724,GO:0032559,GO:0009207,GO:0000291,GO:00325	K12614|1|0.0|929|mmu:13209|ATP-dependent RNA helicase DDX6/DHH1 [EC:3.6.4.13]	gi|148693657|gb|EDL25604.1|	0	DEAD (Asp-Glu-Ala-Asp) box polypeptide 6, isoform CRA_a [Mus musculus]
mmu-miR-1198-5p	NM_001111107	-28.6	0.651322	-26.76	165	GO:0043231,GO:0046914,GO:0003676,GO:0006351,	K09228|1|8e-98|360|mmu:626316|KRAB domain-containing zinc finger protein	gi|27369830|ref|NP_766174.1|	0	zinc finger protein 322 [Mus musculus]
mmu-miR-1198-5p	NM_001115009	-32.2	0.17622	-30.41	175	GO:0030130,GO:0046872,GO:0016192,GO:0015031,	-	gi|169234726|ref|NP_001108481.1|	0	synergin gamma isoform 1 [Mus musculus]
mmu-miR-1198-5p	NM_001127376	-30.2	0.119442	-27.21	167	GO:0005102,GO:0019838,GO:0016049,GO:0023052,GO:0007154,	K06827|1|5e-67|255|dre:321449|connective tissue growth factor	gi|188497673|ref|NP_001120848.1|	0	WNT1-inducible-signaling pathway protein 3 precursor [Mus musculus]

mmu-miR-1198-5p	NM_001136077	-27.3	0.630042	-28.3	158	GO:0016021,GO:0043169,GO:0001871,GO:0008081,GO:0004091,GO:0016462,GO:0004527,GO:0004888,GO:0016477,GO:0046470,GO:0023052,GO:0002376,GO:00422	K01122|1|0.0|1777|mmu:18606|ectonucleotide pyrophosphatase/phosphodiesterase family member 2 [EC:3.1.4.39]	gi|209862963|ref|NP_001129549.1|	0	ectonucleotide pyrophosphatase/phosphodiesterase family member 2 isoform 1 [Mus musculus]
mmu-miR-1198-5p	NM_001145968	-33.4	0.023154	-29.88	188	-	-	gi|225543231|ref|NP_081376.2|	2.03E-152	RWD domain-containing protein 2A [Mus musculus]
mmu-miR-1198-5p	NM_001145969	-27.6	0.295492	-29.88	188	-	-	gi|225543231|ref|NP_081376.2|	2.12E-152	RWD domain-containing protein 2A [Mus musculus]
mmu-miR-1198-5p	NM_001159289	-28	0.372636	-26.97	169	GO:0043231,GO:0003712,GO:0046914,GO:0030098,GO:0032446,GO:0006351,	-	gi|226530152|ref|NP_001152761.1|	6.00E-144	B-cell lymphoma/leukemia 11A isoform 2 [Mus musculus]

mmu-miR-1198-5p	NM_001159290	-28	0.304541	-26.97	169	GO:0043231,GO:0003712,GO:0046914,GO:0030098,GO:0032446,GO:0006351,	-	gi|226530152|ref|NP_001152761.1|	1.81E-132	B-cell lymphoma/leukemia 11A isoform 2 [Mus musculus]
mmu-miR-1198-5p	NM_001160268	-29.2	0.694848	-25.15	158	-	K03426|1|2e-07|60.5|ssc:100512459|NAD+ diphosphatase [EC:3.6.1.22]!K06082|2|8e-06|55.1|tgu:100221501|FERM, RhoGEF and pleckstrin domain protein 2	gi|50510733|dbj|BAD32352.1|	0	mKIAA0969 protein [Mus musculus]
mmu-miR-1198-5p	NM_001163319	-31	0.437393	-28.71	167	GO:0000931,GO:0005819,GO:0015631,GO:0000226,GO:0007049,	K01769|1|8e-36|154|tca:657050|guanylate cyclase, other [EC:4.6.1.2]	gi|254039634|ref|NP_001156791.1|	0	tubulin, gamma complex associated protein 6 [Mus musculus]

mmu-miR-1198-5p	NM_001164659	-27.4	0.966629	-28.96	171	GO:0003676,GO:0003678,GO:0032559,GO:0006259,	-	gi|257467634|ref|NP_001158131.1|	0	TPR and ankyrin repeat-containing protein 1 [Mus musculus]
mmu-miR-1198-5p	NM_001167705	-28.2	0.354341	-25.52	167	GO:0044464,GO:0004866,GO:0010466,GO:0009725,GO:0006952,GO:0006508,	K04525|1|6e-141|501|mmu:20716|serpin peptidase inhibitor, clade A, member 3	gi|267844860|ref|NP_001161177.1|	0	serine protease inhibitor A3A precursor [Mus musculus]
mmu-miR-1198-5p	NM_001170975	-35	0.016249	-29.79	180	-	-	gi|339717165|ref|NP_001164446.2|	7.41E-68	dysbindin domain-containing protein 1 isoform 2 [Mus musculus]
mmu-miR-1198-5p	NM_001174086	-26.1	0.941242	-27.81	152	GO:0005887,GO:0032589,GO:0048168,	-	gi|291463305|ref|NP_001167557.1|	0	protein shisa-9 isoform 2 precursor [Mus musculus]

mmu-miR-1198-5p	NM_001177995	-28.2	0.872448	-25.27	158	GO:0043231,GO:0043234,GO:0046914,GO:0005515,GO:0003712,GO:0003677,GO:0007423,GO:0002762,GO:0007179,GO:0006357,GO:0043467,GO:0045444,GO:0019827,GO:00	K04462|1|0.0|1054|cfa:488148|ecotropic virus integration site 1 protein	gi|295789156|ref|NP_001171466.1|	0	PR domain zinc finger protein 16 isoform 2 [Mus musculus]

mmu-miR-1198-5p	NM_001190448	-32.8	0.061924	-29.04	174	GO:0043005,GO:0044297,GO:0030136,GO:0031406,GO:0005515,GO:0016831,GO:0048037,GO:0019752,GO:0043279,GO:0009058,GO:0070848,GO:0048511,GO:0006586,GO:0001505,GO:0017085,GO:0042493,GO:0007275,GO:0042417,GO:00158	K01593|1|0.0|953|mmu:13195|aromatic-L-amino-acid decarboxylase [EC:4.1.1.28]	gi|148708700|gb|EDL40647.1|	0	dopa decarboxylase [Mus musculus]
mmu-miR-1198-5p	NM_001242349	-27.3	0.812846	-25.12	155	GO:0034702,GO:0005254,GO:0015698,GO:0048513,	K05169|1|4e-26|122|ptr:743996|single Ig IL-1R-related molecule	gi|148686318|gb|EDL18265.1|	0	mCG126938, isoform CRA_h [Mus musculus]

mmu-miR-1198-5p	NM_001242934	-28	0.809653	-26.97	169	GO:0043231,GO:0003712,GO:0046914,GO:0030098,GO:0032446,GO:0006351,	K09228|1|5e-17|92.0|api:100159445|KRAB domain-containing zinc finger protein	gi|339409205|ref|NP_001229863.1|	0	B-cell lymphoma/leukemia 11A isoform 4 [Mus musculus]
mmu-miR-1198-5p	NM_001252572	-32.1	0.139425	-29.59	170	GO:0043232,GO:0031252,GO:0044444,GO:0005515,GO:0007417,GO:0006897,	K06106|1|0.0|937|mmu:13043|cortactin	gi|357588432|ref|NP_001239501.1|	0	src substrate cortactin isoform 2 [Mus musculus]
mmu-miR-1198-5p	NM_007532	-26.7	0.968736	-28.37	155	GO:0043231,GO:0044444,GO:0005515,GO:0004084,GO:0022402,GO:0008652,GO:0009063,	K00826|1|0.0|792|mmu:12035|branched-chain amino acid aminotransferase [EC:2.6.1.42]	gi|74185103|dbj|BAE39154.1|	0	unnamed protein product [Mus musculus]
mmu-miR-1198-5p	NM_007803	-32.1	0.145006	-29.59	170	GO:0043232,GO:0031252,GO:0044444,GO:0005515,GO:0006897,	K06106|1|0.0|1030|mmu:13043|cortactin	gi|75677414|ref|NP_031829.2|	0	src substrate cortactin isoform 1 [Mus musculus]

mmu-miR-1198-5p	NM_007841	-27.7	0.840588	-27.5	157	GO:0031981,GO:0031332,GO:0044444,GO:0003676,GO:0042623,GO:0003724,GO:0032559,GO:0009207,GO:0000291,GO:00325	K12614|1|0.0|929|mmu:13209|ATP-dependent RNA helicase DDX6/DHH1 [EC:3.6.4.13]	gi|148693657|gb|EDL25604.1|	0	DEAD (Asp-Glu-Ala-Asp) box polypeptide 6, isoform CRA_a [Mus musculus]
mmu-miR-1198-5p	NM_008013	-31.2	0.239624	-27.52	177	GO:0005615,GO:0044464,GO:0008047,GO:0005515,GO:0016787,GO:0022414,GO:0051345,GO:0023052,	K10104|1|1e-55|219|nve:NEMVE_v1g90597|ficolin!K05466|2|4e-50|201|mdo:100028850|angi	gi|31542814|ref|NP_032039.2|	0	fibroleukin precursor [Mus musculus]
mmu-miR-1198-5p	NM_008564	-32.5	0.130974	-27.47	171	GO:0000808,GO:0031981,GO:0043169,GO:0043565,GO:0005515,GO:0032559,GO:0007050,GO:0006261,GO:0051320,GO:0006351,GO:0034728,	K02540|1|0.0|1631|mmu:17216|minichromosome maintenance protein 2	gi|74226965|dbj|BAE27124.1|	0	unnamed protein product [Mus musculus]

mmu-miR-1198-5p	NM_009305	-30.7	0.187053	-28.61	173	GO:0031224,GO:0032991,GO:0044463,GO:0044459,GO:0030672,GO:0032934,GO:0046872,GO:0019904,GO:0019905,GO:0016192,GO:0016044,GO:0048168,GO:00160	-	gi|15928723|gb|AAH14823.1|	4.49E-124	Synaptophysin [Mus musculus]
mmu-miR-1198-5p	NM_010696	-27.4	0.690196	-26.06	167	-	K07361|1|0.0|904|mmu:16822|lymphocyte cytosolic protein 2	gi|31543115|ref|NP_034826.2|	0	lymphocyte cytosolic protein 2 [Mus musculus]
mmu-miR-1198-5p	NM_011041	-30.4	0.218124	-47.28	334	GO:0043231,GO:0043234,GO:0003676,GO:0001071,GO:0009888,GO:0006357,GO:0070848,GO:0032501,GO:0009653,	K09382|1|0.0|699|aml:100466359|paired box protein 1/9	gi|148704759|gb|EDL36706.1|	0	paired box gene 9, isoform CRA_a [Mus musculus]

mmu-miR-1198-5p	NM_011395	-25.8	0.908275	-28.25	162	GO:0016021,GO:0005488,GO:0008504,GO:0008324,GO:0051937,GO:0001504,GO:0031667,GO:0015695,	K08200|1|0.0|1074|mmu:20519|MFS transporter, OCT family, solute carrier family 22 (organic cation transporter), member 3	gi|6755538|ref|NP_035525.1|	0	solute carrier family 22 member 3 [Mus musculus]
mmu-miR-1198-5p	NM_011460	-29.1	0.287727	-27.3	159	GO:0043231,GO:0044444,GO:0044421,GO:0019899,GO:0004869,GO:0002444,GO:0043627,GO:0002437,GO:0001909,GO:0006508,GO:0010466,GO:0070229,	K13963|1|0.0|747|mmu:20726|serpin B	gi|148700399|gb|EDL32346.1|	0	mCG15549 [Mus musculus]

mmu-miR-1198-5p	NM_011588	-29.7	0.351985	-26.71	172	GO:0000790,GO:0046914,GO:0016301,GO:0003712,GO:0001071,GO:0019904,GO:0003677,GO:0006357,GO:0006468,GO:00009	K08882|1|0.0|1340|mmu:21849|tripartite motif-containing protein 28	gi|148706135|gb|EDL38082.1|	0	tripartite motif protein 28 [Mus musculus]
mmu-miR-1198-5p	NM_013774	-29.3	0.117873	-25.6	166	-	-	gi|148877859|gb|AAI45789.1|	3.22E-68	Tcl1b4 protein [Mus musculus]
mmu-miR-1198-5p	NM_013776	-29.3	0.120364	-25.6	166	-	-	gi|7305561|ref|NP_038804.1|	1.18E-49	protein TCL1B5 [Mus musculus]
mmu-miR-1198-5p	NM_015745	-29.1	0.603493	-25.52	157	GO:0005578,GO:0005501,GO:0070011,GO:0051234,GO:0019538,GO:0050953,GO:00017	-	gi|21729751|ref|NP_056560.1|	0	retinol-binding protein 3 precursor [Mus musculus]

mmu-miR-1198-5p	NM_016672	-32.8	0.059891	-29.04	174	GO:0043005,GO:0044297,GO:0030136,GO:0031406,GO:0005515,GO:0016831,GO:0048037,GO:0019752,GO:0043279,GO:0009058,GO:0070848,GO:0048511,GO:0006586,GO:0001505,GO:0017085,GO:0042493,GO:0007275,GO:0042417,GO:00158	K01593|1|0.0|953|mmu:13195|aromatic-L-amino-acid decarboxylase [EC:4.1.1.28]	gi|148708700|gb|EDL40647.1|	0	dopa decarboxylase [Mus musculus]
mmu-miR-1198-5p	NM_016707	-28	0.606843	-26.97	169	GO:0043231,GO:0003712,GO:0046914,GO:0030098,GO:0032446,GO:0006351,	K09228|1|2e-11|72.8|ecb:100056421|KRAB domain-containing zinc finger protein	gi|7710022|ref|NP_057916.1|	0	B-cell lymphoma/leukemia 11A isoform 1 [Mus musculus]

mmu-miR-1198-5p	NM_019648	-26.9	0.880814	-45.1	319	GO:0044424,GO:0043169,GO:0008238,GO:0008237,GO:0018193,GO:0051604,	K01265|1|0.0|851|mmu:56307|methionyl aminopeptidase [EC:3.4.11.	gi|9789873|ref|NP_062622.1|	0	methionine aminopeptidase 2 [Mus musculus]
mmu-miR-1198-5p	NM_019831	-29.4	0.570076	-27.55	174	GO:0043231,GO:0046914,GO:0003676,GO:0032501,	-	gi|9790027|ref|NP_062805.1|	0	zinc finger MYM-type protein 3 isoform 1 [Mus musculus]

mmu-miR-1198-5p	NM_021458	-25	0.999942	-42.25	315	GO:0044456,GO:0043005,GO:0044297,GO:0031253,GO:0044424,GO:0031224,GO:0019904,GO:0004888,GO:0007411,GO:0014020,GO:0008544,GO:0030901,GO:0006357,GO:0016055,GO:0045137,GO:0021548,GO:0021543,GO:0042471,GO:0007187,GO:0001738,GO:0016477,GO:00217	K02329|1|0.0|1358|mmu:14365|frizzled 3	gi|148704084|gb|EDL36031.1|	0	frizzled homolog 3 (Drosophila), isoform CRA_a [Mus musculus]
mmu-miR-1198-5p	NM_026009	-29	0.442791	-25.27	159	GO:0043231,GO:0031224,GO:0046872,GO:0006984,GO:0055065,GO:0007275,	-	gi|148702331|gb|EDL34278.1|	0	mCG3218, isoform CRA_b [Mus musculus]

mmu-miR-1198-5p	NM_026099	-26.3	0.207169	-26.46	167	-	-	gi|50979268|ref|NP_080375.2|	9.54E-80	uncharacterized protein C5orf48 homolog [Mus musculus]
mmu-miR-1198-5p	NM_026157	-25.3	0.894859	-41.77	314	GO:0043231,GO:0070566,GO:0003676,GO:0032559,GO:0000956,GO:0006396,	K13291|1|5e-20|100|mdo:100018817|terminal uridylyltransferase [EC:2.7.7.52]	gi|148691104|gb|EDL23051.1|	0	PAP associated domain containing 1, isoform CRA_b [Mus musculus]
mmu-miR-1198-5p	NM_026168	-29.5	0.440113	-53.03	343	-	-	gi|21312962|ref|NP_080444.1|	0	endoplasmic reticulum-Golgi intermediate compartment protein 2 isoform 1 [Mus musculus]
mmu-miR-1198-5p	NM_026283	-29.4	0.661843	-26.51	162	GO:0031224,GO:0006684,	K04714|1|9e-88|327|bfo:BRAFLDRAFT_104157|shingomyelin synthase [EC:2.7.8.27]	gi|21312864|ref|NP_080559.1|	0	sphingomyelin synthase-related protein 1 [Mus musculus]
mmu-miR-1198-5p	NM_026655	-31.1	0.140463	-28	168	GO:0005515,	-	gi|21311825|ref|NP_080931.1|	0	uncharacterized protein C10orf88 homolog [Mus musculus]

mmu-miR-1198-5p	NM_026824	-28.3	0.3498	-26.13	171	GO:0016627,GO:0050662,GO:0006399,	K05542|1|0.0|961|mmu:68730|tRNA-dihydrouridine synthase 1 [EC:1.3.1.88]	gi|31980834|ref|NP_081100.2|	0	tRNA-dihydrouridine(16/17) synthase [NAD(P)(+)]-like [Mus musculus]
mmu-miR-1198-5p	NM_026872	-28.3	0.654232	-25.59	163	-	-	gi|28076915|ref|NP_081148.1|	0	ubiquitin-associated protein 2 [Mus musculus]
mmu-miR-1198-5p	NM_027100	-33.4	0.026904	-29.88	188	-	-	gi|225543231|ref|NP_081376.2|	2.34E-152	RWD domain-containing protein 2A [Mus musculus]
mmu-miR-1198-5p	NM_027504	-28.2	0.877159	-25.27	158	GO:0043231,GO:0043234,GO:0046914,GO:0005515,GO:0003712,GO:0003677,GO:0007423,GO:0002762,GO:0007179,GO:0006357,GO:0043467,GO:0045444,GO:0019827,GO:00	K04462|1|0.0|1093|cfa:488148|ecotropic virus integration site 1 protein	gi|124107623|ref|NP_081780.3|	0	PR domain zinc finger protein 16 isoform 1 [Mus musculus]
mmu-miR-1198-5p	NM_028157	-29.3	0.497658	-25	159	-	-	gi|140970996|ref|NP_082433.2|	0	uncharacterized protein LOC72219 [Mus musculus]

mmu-miR-1198-5p	NM_028245	-28.4	0.491868	-27.36	170	GO:0043231,GO:0046914,GO:0003676,GO:0001071,GO:0006351,	K10500|1|6e-38|160|cfa:607083|zinc finger and BTB domain-containing protein 17	gi|148686383|gb|EDL18330.1|	0	zinc finger protein 131, isoform CRA_a [Mus musculus]
mmu-miR-1198-5p	NM_028264	-28.4	0.396968	-25.94	158	GO:0005764,GO:0031224,GO:0005770,GO:0003824,	K13084|1|9e-153|541|mmu:72519|phosphatidylinositol-4,5-bisphosphate 4-phosphatase [EC:3.1.3.78]	gi|21312268|ref|NP_082540.1|	6.00E-152	transmembrane protein 55A [Mus musculus]
mmu-miR-1198-5p	NM_028277	-26.1	0.942745	-27.81	152	GO:0005887,GO:0032589,GO:0048168,	-	gi|291463307|ref|NP_082553.2|	0	protein shisa-9 isoform 1 precursor [Mus musculus]
mmu-miR-1198-5p	NM_028901	-26.9	0.964934	-46.84	313	GO:0016459,GO:0030016,GO:0031981,GO:0008092,GO:0017111,GO:0032559,	K10362|1|0.0|4354|mmu:74376|myosin XVIII	gi|291327510|ref|NP_083177.2|	0	myosin XVIIIb [Mus musculus]

mmu-miR-1198-5p	NM_029330	-29.4	0.415535	-47.62	328	GO:0044444,GO:0032561,GO:0070568,GO:0006005,	K00976|1|0.0|1157|mmu:75540|fucose-1-phosphate guanylyltransferase [EC:2.7.7.30]	gi|117676389|ref|NP_083606.2|	0	fucose-1-phosphate guanylyltransferase [Mus musculus]
mmu-miR-1198-5p	NM_029337	-30.9	0.601702	-28.88	171	GO:0016604,GO:0043189,GO:0017111,GO:0003676,GO:0032559,GO:0016573,	K11320|1|0.0|4099|mmu:75560|E1A-binding protein p400 [EC:3.6.4.-]	gi|153945880|ref|NP_083613.2|	0	E1A-binding protein p400 isoform 1 [Mus musculus]
mmu-miR-1198-5p	NM_029489	-25.7	0.898275	-28.64	169	-	K11457|1|1e-14|83.2|dre:171465|polyhomeotic-like protein 2	gi|254039607|ref|NP_083765.2|	0	sterile alpha motif domain-containing protein 7 [Mus musculus]
mmu-miR-1198-5p	NM_029494	-33.2	0.051857	-29.98	166	GO:0044431,GO:0016020,GO:0017111,GO:0032561,GO:0006810,GO:0035556,GO:00092	K07917|1|2e-112|407|ecb:100051006|Ras-related protein Rab-30	gi|21361418|ref|NP_055303.2|	1.17E-111	ras-related protein Rab-30 [Homo sapiens]

mmu-miR-1198-5p	NM_029992		-31	0.196124	-27.68	173	GO:0044448,GO:0005882,GO:0043231,GO:0016020,GO:0005488,GO:0016049,GO:0012501,	K10352|1|3e-35|151|cin:100176037|myosin heavy chain!K11423|2|5e-07|57.8|dgr:Dgri_GH16034|histone-lysine N-methyltransferase SETD2 [EC:2.1.1.43]	gi|148222456|ref|NP_084268.2|	3.10E-164	trichoplein keratin filament-binding protein [Mus musculus]
mmu-miR-1198-5p	NM_080466		-35	0.124167	-30.53	170	GO:0031224,GO:0005515,GO:0015269,GO:0034220,GO:0030001,	K04944|1|0.0|1152|mmu:140493|potassium intermediate/small conductance calcium-activated channel subfamily N member 3	gi|158854044|ref|NP_536714.2|	0	small conductance calcium-activated potassium channel protein 3 [Mus musculus]

mmu-miR-1198-5p	NM_133227	-32.2	0.280741	-26.8	168	-	K14312|1|0.0|2612|mmu:170762|nuclear pore complex protein Nup155	gi|37360098|dbj|BAC98027.1|	0	mKIAA0791 protein [Mus musculus]
mmu-miR-1198-5p	NM_133678	-29.3	0.16777	-26.09	168	GO:0005815,GO:0051297,GO:0007051,GO:0000087,	-	gi|119508435|ref|NP_598439.3|	0	SAC3 domain-containing protein 1 [Mus musculus]
mmu-miR-1198-5p	NM_145379	-33.8	0.04008	-29.6	178	GO:0031224,GO:0004888,GO:0007166,	K08394|1|0.0|671|mmu:211577|Mas-related G protein-coupled receptor member F	gi|148686343|gb|EDL18290.1|	0	MAS-related GPR, member F, isoform CRA_a [Mus musculus]
mmu-miR-1198-5p	NM_146112	-35.1	0.087684	-32.01	170	GO:0005488,GO:0050905,GO:0019538,GO:0001894,GO:0021515,GO:0050879,GO:0040007,GO:0007626,GO:0007169,GO:00513	-	gi|148708223|gb|EDL40170.1|	0	trinucleotide repeat containing 15, isoform CRA_g [Mus musculus]

mmu-miR-1198-5p	NM_146182	-32.7	0.054951	-27.92	170	GO:0005875,GO:0015631,GO:0003774,GO:0030572,GO:0010970,	K10407|1|0.0|712|mmu:232943|kinesin light chain	gi|148691194|gb|EDL23141.1|		0	kinesin light chain 3, isoform CRA_a [Mus musculus]
mmu-miR-1198-5p	NM_172442	-29.2	0.62258	-26.64	176	GO:0044424,GO:0046914,GO:0007166,	K06058|1|0.0|1165|mmu:207521|delte	gi|188219611|ref|NP_766030.3|		0	E3 ubiquitin-protein ligase DTX4 [Mus musculus]
mmu-miR-1198-5p	NM_172541	-29.7	0.129928	-27.6	171	GO:0031224,	K14165|1|4e-161|568|mcc:715846|dual specificity phosphatase [EC:3.1.3.16 3.1.3.48]!K14574|2|6e-73|275|spu:588956|ribosome maturation protein SDO1	gi|29789387|ref|NP_766129.1|		0	transmembrane protein 120A [Mus musculus]

mmu-miR-1198-5p	NM_172586	-28.6	0.64567	-26.76	165	GO:0043231,GO:0046914,GO:0003676,GO:0006351,	K09228|1|8e-98|360|mmu:626316|KRAB domain-containing zinc finger protein	gi|27369830|ref|NP_766174.1|	0	zinc finger protein 322 [Mus musculus]
mmu-miR-1198-5p	NM_172770	-31.2	0.230806	-26.35	162	-	K09553|1|2e-15|85.9|dmo:Dmoj_GI17037|stress-induced-phosphoprotein 1	gi|27370132|ref|NP_766358.1|	0	tetratricopeptide repeat protein 12 [Mus musculus]
mmu-miR-1198-5p	NM_172868	-33.1	0.260147	-29.39	171	GO:0016020,GO:0065008,	-	gi|27777697|ref|NP_766456.1|	0	paralemmin-2 [Mus musculus]
mmu-miR-1198-5p	NM_172916	-36.2	0.169649	-43.1	318	-	-	gi|46361984|ref|NP_766504.3|	0	hydrocephalus-inducing protein [Mus musculus]
mmu-miR-1198-5p	NM_173004	-29.4	0.354544	-27.78	167	GO:0031224,GO:0043005,GO:0005488,GO:0045596,GO:0007417,GO:0016337,GO:0050804,GO:0006935,GO:0007409,	K06762|1|0.0|1363|mmu:269784|contactin 4	gi|157909805|ref|NP_766592.2|	0	contactin-4 isoform 2 precursor [Mus musculus]

mmu-miR-1198-5p	NM_173066	-30.9	0.598215	-28.88	171	GO:0016604,GO:0043189,GO:0017111,GO:0003676,GO:0032559,GO:0016573,	K11320|1|0.0|3918|mmu:75560|E1A-binding protein p400 [EC:3.6.4.-]	gi|148688062|gb|EDL20009.1|	0	E1A binding protein p400, isoform CRA_c [Mus musculus]
mmu-miR-1198-5p	NM_176922	-31.2	0.309301	-50.35	326	GO:0005887,GO:0004871,GO:0005515,GO:0016477,GO:0031589,GO:0007166,GO:00485	K06587|1|0.0|2383|mmu:319480|integrin alpha 11	gi|225703035|ref|NP_795896.4|	0	integrin alpha-11 precursor [Mus musculus]
mmu-miR-1198-5p	NM_177005	-25.9	0.629553	-26.3	170	GO:0016740,GO:0008152,	-	gi|147901175|ref|NP_795979.2|	0	glycosyltransferase 1 domain-containing protein 1 precursor [Mus musculus]
mmu-miR-1198-5p	NM_177068	-27.8	0.599958	-27.51	163	-	K04592|1|6e-49|197|ssc:100514215|latrophilin 1	gi|224922773|ref|NP_796042.3|	0	olfactomedin-like protein 2B precursor [Mus musculus]
mmu-miR-1198-5p	NM_177140	-31.5	0.054632	-26.42	163	GO:0031224,	-	gi|354489678|ref|XP_003506988.1|	1.55E-89	PREDICTED: UPF0694 transmembrane protein C14orf109 homolog [Cricetulus griseus]
mmu-miR-1198-5p	NM_177890	-29	0.418022	-25.37	163	-	-	gi|116089290|ref|NP_808558.2|	0	uncharacterized protein LOC330513 [Mus musculus]

mmu-miR-1198-5p	NM_178227	-25.5	0.961335	-40.6	318	GO:0031224,GO:0005272,GO:0006814,GO:0048731,GO:0007600,GO:0006935,GO:0034220,	K04847|1|4e-125|450|ptr:466810|voltage-gated sodium channel type III beta	gi|50510815|dbj|BAD32393.1|	5.45E-152	mKIAA1158 protein [Mus musculus]
mmu-miR-1198-5p	NM_178642	-27.3	0.813436	-25.12	155	GO:0034702,GO:0005254,GO:0015698,GO:0048513,	K05169|1|4e-26|122|ptr:743996|single Ig IL-1R-related molecule	gi|148686318|gb|EDL18265.1|	0	mCG126938, isoform CRA_h [Mus musculus]
mmu-miR-1198-5p	NM_178656	-31.6	0.16337	-25.93	167	GO:0031224,	-	gi|30520017|ref|NP_848771.1|	3.23E-63	phosphoinositide-interacting protein [Mus musculus]
mmu-miR-1198-5p	NM_178801	-28	0.49419	-26.04	163	GO:0031224,	K15281|1|5e-09|64.3|bta:617113|solute carrier family 35	gi|148747427|ref|NP_848916.2|	2.06E-164	transmembrane protein 241 [Mus musculus]

mmu-miR-1198-5p	NM_181324	-27.7	0.841721	-27.5	157	GO:0031981,GO:0031332,GO:0044444,GO:0003676,GO:0042623,GO:0003724,GO:0032559,GO:0009207,GO:0000291,GO:00325	K12614|1|0.0|929|mmu:13209|ATP-dependent RNA helicase DDX6/DHH1 [EC:3.6.4.13]	gi|148693657|gb|EDL25604.1|	0	DEAD (Asp-Glu-Ala-Asp) box polypeptide 6, isoform CRA_a [Mus musculus]
mmu-miR-1198-5p	NM_181402	-26.1	0.893238	-27.53	166	GO:0016763,	K15259|1|0.0|641|mmu:101187|poly [ADP-ribose] polymerase 7/11/12/13 [EC:2.4.2.30]	gi|148667444|gb|EDK99860.1|	0	poly (ADP-ribose) polymerase family, member 11, isoform CRA_b [Mus musculus]

mmu-miR-1198-5p	NM_182930	-29.2	0.709681	-25.15	158	-	K03426|1|3e-09|66.6|ssc:100512459|NAD+ diphosphatase [EC:3.6.1.22]!K06082|2|2e-08|63.5|tgu:100221501|FERM, RhoGEF and pleckstrin domain protein 2	gi|148707707|gb|EDL39654.1|	0	pleckstrin homology domain containing, family A member 6, isoform CRA_a [Mus musculus]

mmu-miR-1198-5p	NM_183355	-26.4	0.969269	-46.44	301	GO:0031981,GO:0043234,GO:0001071,GO:0046983,GO:0003677,GO:0001501,GO:0030097,GO:0045596,GO:0008202,GO:0006357,GO:0003006,GO:0008283,GO:0035108,GO:0001503,GO:0043392,GO:0003002,GO:0048754,GO:0009887,GO:00352	K09355|1|0.0|738|ecb:100058426|pre-B-cell leukemia transcription factor 1	gi|149755772|ref|XP_001493290.1|	0	PREDICTED: pre-B-cell leukemia transcription factor 1 isoform 1 [Equus caballus]
mmu-miR-1198-5p	NM_198022	-28	0.899404	-26.64	170	GO:0003676,GO:0016458,	K06237|1|2e-08|64.3|dan:Dana_GF14161|collagen, type IV, alpha	gi|124378035|ref|NP_932139.2|	0	trinucleotide repeat-containing gene 6C protein [Mus musculus]
mmu-miR-1198-5p	NM_198680	-29.4	0.196279	-25.24	156	GO:0005488,GO:0004866,GO:0006508,	K13963|1|0.0|711|mmu:383548|serpin B	gi|38371730|ref|NP_941373.1|	0	serine (or cysteine) proteinase inhibitor, clade b, member 3b [Mus musculus]

mmu-miR-1198-5p	NM_181397	-23.9	0.986601	-51.5	332	GO:0016020,	-	gi|74142212|dbj|BAE31871.1|	0	unnamed protein product [Mus musculus]
mmu-miR-1199-3p	NM_001001160	-27	0.966106	-81.07	442	GO:0005488,	K10316|1|0.0|1285|mmu:330369|F-box protein	gi|296482725|gb|DAA24840.1|	0	F-box protein 41-like [Bos taurus]
mmu-miR-1199-3p	NM_001001565	-26.8	0.854195	-48.84	303	GO:0000139,GO:0031224,GO:0015020,GO:0043169,GO:0008376,	K00747|1|0.0|1271|rno:316533|chondroitin polymerizing factor [EC:2.4.1.175 2.4.1.226]	gi|148667995|gb|EDL00412.1|	0	DNA segment, Chr 1, Brigham & Women's Genetics 1363 expressed, isoform CRA_b [Mus musculus]
mmu-miR-1199-3p	NM_001001566	-26.8	0.841675	-48.84	303	GO:0000139,GO:0031224,GO:0015020,GO:0043169,GO:0008376,	K00747|1|0.0|1436|rno:316533|chondroitin polymerizing factor [EC:2.4.1.175 2.4.1.226]	gi|48526514|ref|NP_001001566.1|	0	chondroitin sulfate synthase 2 isoform a [Mus musculus]

mmu-miR-1199-3p	NM_001001602	-26.6	0.979069	-27.51	150	GO:0009898,GO:0044424,GO:0008047,GO:0060090,GO:0005083,GO:0042802,GO:0019901,GO:0019903,GO:0032813,GO:0006915,GO:0035411,GO:0016049,GO:0032318,GO:0034260,GO:0043433,GO:0001837,GO:0006469,GO:0070849,GO:0030178,GO:0007173,GO:0000122,GO:00082	K04352|1|4e-46|188|cin:100184835|Ras GTPase-activating protein 1	gi|166295210|ref|NP_001001602.2|	0	disabled homolog 2-interacting protein isoform 3 [Mus musculus]
mmu-miR-1199-3p	NM_001001807	-29.1	0.160028	-29.18	152	GO:0004871,GO:0023052,	K04257|1|1e-165|582|mmu:258502|olfactory	gi|49227367|ref|NP_001001807.1|	9.22E-165	olfactory receptor 279 [Mus musculus]
mmu-miR-1199-3p	NM_001001885	-27.4	0.843394	-30.07	167	GO:0031224,	-	gi|49457865|ref|NP_001001885.1|	0	transmembrane protein 151A [Mus musculus]

mmu-miR-1199-3p	NM_001001984	-25	0.999597	-26.18	157	GO:0031981,GO:0003676,GO:0046914,GO:0016706,GO:0016701,GO:0070076,GO:0006351,	K10276|1|0.0|2158|mmu:225876|F-box and leucine-rich repeat protein 10/11 [EC:1.14.11.27]	gi|100818178|ref|NP_001001984.2|	0	lysine-specific demethylase 2A [Mus musculus]
mmu-miR-1199-3p	NM_001001985	-33.3	0.267232	-107.18	620	GO:0005789,GO:0005740,GO:0008080,	-	gi|134288912|ref|NP_001001985.3|	4.43E-124	N-acetylaspartate synthetase [Mus musculus]
mmu-miR-1199-3p	NM_001001999	-28.1	0.510436	-54.95	300	GO:0016021,GO:0005488,GO:0001775,GO:0072376,GO:0007599,	K06262|1|1e-66|255|mmu:14724|platelet glycoprotein Ib beta chain	gi|517494|gb|AAA20398.1|	2.03E-66	glycoprotein Ib beta [Homo sapiens]
mmu-miR-1199-3p	NM_001002267	-30.2	0.212713	-89.73	467	GO:0031224,GO:0005488,	-	gi|116805336|ref|NP_001002267.2|	1.57E-109	transmembrane protein 158 precursor [Mus musculus]

mmu-miR-1199-3p	NM_001002929	-29.5	0.342025	-25.45	157	GO:0005643,GO:0000776,GO:0044444,GO:0005819,GO:0005488,GO:0044238,GO:0050658,GO:0015758,GO:0007067,GO:0030595,GO:0030031,	K14304|1|0.0|1279|mmu:445007|nuclear pore complex protein Nup85	gi|108773813|ref|NP_001002929.3|	0	nuclear pore complex protein Nup85 [Mus musculus]
mmu-miR-1199-3p	NM_001003668	-28.7	0.379801	-26.18	147	GO:0005882,GO:0005488,GO:0009888,	K07605|1|0.0|961|mmu:406219|type II keratin, basic	gi|269914157|ref|NP_001003668.2|	0	keratin, type II cuticular Hb3 [Mus musculus]
mmu-miR-1199-3p	NM_001003719	-34.8	0.195034	-58.95	300	GO:0043231,GO:0044444,GO:0005099,GO:0046983,GO:0032852,GO:0006351,	K07207|1|7e-19|98.6|bfo:BRAFLDRAFT_64809|tuberous sclerosis 2	gi|51230692|ref|NP_001003719.1|	0	ral GTPase-activating protein subunit alpha-1 isoform 3 [Mus musculus]

mmu-miR-1199-3p	NM_001003824	-29.1	0.505182	-31.79	158	GO:0032991,GO:0005887,GO:0005515,GO:0005267,GO:0048731,GO:0007267,GO:0006935,GO:0034220,GO:0030001,	K04927|1|0.0|1436|mmu:16536|potassium voltage-gated channel KQT-like subfamily member 2	gi|148675436|gb|EDL07383.1|	0	potassium voltage-gated channel, subfamily Q, member 2, isoform CRA_j [Mus musculus]
mmu-miR-1199-3p	NM_001003825	-29.1	0.503327	-31.79	158	GO:0032991,GO:0005887,GO:0005515,GO:0005267,GO:0048731,GO:0007267,GO:0006935,GO:0034220,GO:0030001,	K04927|1|0.0|1355|mmu:16536|potassium voltage-gated channel KQT-like subfamily member 2	gi|51474502|ref|NP_001003825.1|	0	potassium voltage-gated channel subfamily KQT member 2 isoform 3 [Mus musculus]
mmu-miR-1199-3p	NM_001003909	-29.8	0.693427	-60.82	316	GO:0046914,	K11967|1|0.0|2089|mmu:70797|ankyrin repeat and IBR domain-containing protein 1	gi|37360350|dbj|BAC98153.1|	0	mKIAA1386 protein [Mus musculus]

mmu-miR-1199-3p	NM_001003918	-34.1	0.170718	-30.29	153	GO:0016604,GO:0016790,GO:0004175,GO:0042802,GO:0019899,GO:0019783,GO:0012502,GO:0010608,GO:0051704,GO:0006355,GO:0019941,GO:0032501,GO:00706	K11838|1|0.0|2205|mmu:252870|ubiquitin carboxyl-terminal hydrolase 7 [EC:3.1.2.15]	gi|154146209|ref|NP_001003918.2|	0	ubiquitin carboxyl-terminal hydrolase 7 [Mus musculus]
mmu-miR-1199-3p	NM_001003950	-38.1	0.015613	-31.61	154	GO:0005815,GO:0043231,GO:0030695,GO:0019899,GO:0006892,GO:0019222,GO:0010927,GO:0034613,	-	gi|51556219|ref|NP_001003950.1|	0	rab-3A-interacting protein [Mus musculus]

mmu-miR-1199-3p	NM_001004144	-28.2	0.710523	-26.81	151	GO:0005924,GO:0044424,GO:0016772,GO:0005083,GO:0008047,GO:0004871,GO:0046914,GO:0042802,GO:0051020,GO:0043087,GO:0007186,GO:0006796,GO:00320	K05737|1|0.0|1467|mmu:216963|G protein-coupled receptor kinase interactor 1	gi|51921285|ref|NP_001004144.1|	0	ARF GTPase-activating protein GIT1 [Mus musculus]
mmu-miR-1199-3p	NM_001004147	-25.4	0.798481	-54.31	296	GO:0016741,GO:0042254,GO:0006399,	K14563|1|1e-127|457|mmu:237730|rRNA 2'-O-methyltransferase fibrillarin [EC:2.1.1.-]	gi|51921297|ref|NP_001004147.1|	6.93E-127	rRNA/tRNA 2'-O-methyltransferase fibrillarin-like protein 1 [Mus musculus]
mmu-miR-1199-3p	NM_001004361	-27.8	0.797283	-32.05	159	GO:0015630,	-	gi|218156330|ref|NP_001004361.2|	8.23E-150	tubulin polyglutamylase complex subunit 2 isoform a [Mus musculus]

mmu-miR-1199-3p	NM_001004365	-34.7	0.034317	-29.27	164	GO:0043232,GO:0008092,GO:0032559,GO:0008064,	K05692|1|9e-67|255|aml:100466132|actin beta/gamma 1	gi|52345394|ref|NP_001004365.1|	0	actin-related protein 3B [Mus musculus]
mmu-miR-1199-3p	NM_001004367	-27.9	0.876731	-25.45	147	GO:0031410,GO:0046914,GO:0019904,GO:0003676,GO:0016055,GO:0000578,	K03344|1|5e-96|354|pon:100437255|Dvl-binding protein IDAX	gi|365812519|ref|NP_001004367.2|	6.86E-142	CXXC-type zinc finger protein 4 [Mus musculus]
mmu-miR-1199-3p	NM_001004455	-31	0.158814	-56.29	294	GO:0005930,GO:0043232,	-	gi|148705044|gb|EDL36991.1|	2.17E-82	cystin 1 [Mus musculus]
mmu-miR-1199-3p	NM_001005331	-34.1	0.171779	-29.48	151	GO:0043234,GO:0044444,GO:0008135,GO:0031369,GO:0006413,	K03260|1|0.0|2575|mmu:208643|translation initiation factor 4G	gi|56699434|ref|NP_001005331.1|	0	eukaryotic translation initiation factor 4 gamma 1 isoform b [Mus musculus]
mmu-miR-1199-3p	NM_001005343	-31.1	0.251794	-145.07	763	GO:0003676,GO:0009967,GO:0035115,	K09199|1|0.0|653|mmu:381373|transcription factor Sp9	gi|52693933|ref|NP_001005343.1|	0	transcription factor Sp9 [Mus musculus]

mmu-miR-1199-3p	NM_001005419	-25.4	0.988657	-80.7	450	GO:0043231,GO:0016702,GO:0043169,GO:0008152,	K10712|1|2e-137|491|mmu:211488|cysteamine dioxygenase [EC:1.13.11.19]	gi|225703118|ref|NP_001005419.2|	1.16E-136	2-aminoethanethiol dioxygenase [Mus musculus]
mmu-miR-1199-3p	NM_001005423	-30	0.32724	-25.03	146	GO:0044459,GO:0050931,GO:0022405,	-	gi|53749212|ref|NP_001005423.1|	3.99E-96	melanoregulin [Mus musculus]
mmu-miR-1199-3p	NM_001005424	-26.5	0.953088	-52.95	298	-	-	gi|148762971|ref|NP_001005424.2|	0	uncharacterized protein C9orf172 homolog [Mus musculus]
mmu-miR-1199-3p	NM_001005477	-34.7	0.050952	-120.41	621	GO:0043231,GO:0001071,GO:0003677,GO:0016477,GO:0006412,GO:0006355,GO:0001558,GO:0045165,GO:0050767,	K09360|1|1e-179|630|mmu:104382|BarH-like	gi|56699436|ref|NP_001005477.1|	1.01E-178	barH-like 2 homeobox protein [Mus musculus]

mmu-miR-1199-3p	NM_001005509	-38.4	0.011175	-34.88	168	GO:0043234,GO:0044424,GO:0043021,GO:0008135,GO:0006412,GO:0042254,GO:0006984,GO:0006464,	K15026|1|0.0|1101|mmu:229317|translation initiation factor 2A	gi|26347343|dbj|BAC37320.1|	0	unnamed protein product [Mus musculus]
mmu-miR-1199-3p	NM_001005511	-31.6	0.352237	-77.06	448	GO:0031224,GO:0004672,GO:0032559,GO:0006464,	K08899|1|0.0|1253|mmu:381983|lemur tyrosine kinase 3 [EC:2.7.10.1]	gi|392344238|ref|XP_003748906.1|	0	PREDICTED: LOW QUALITY PROTEIN: serine/threonine-protein kinase LMTK3-like [Rattus norvegicus]
mmu-miR-1199-3p	NM_001005863	-27.4	0.945326	-25.69	145	GO:0016020,GO:0043231,GO:0009987,	K10352|1|2e-14|84.0|tca:663025|myosin heavy chain!K11423|5|8e-14|81.6|dgr:Dgri_GH16034|histone-lysine N-methyltransferase SETD2 [EC:2.1.1.43]	gi|54312129|ref|NP_001005863.1|	0	microtubule-associated tumor suppressor 1 homolog isoform 1 [Mus musculus]

mmu-miR-1199-3p	NM_001006668	-29.1	0.500717	-31.79	158	GO:0032991,GO:0005887,GO:0005515,GO:0005267,GO:0048731,GO:0007267,GO:0006935,GO:0034220,GO:0030001,	K04927|1|0.0|1405|mmu:16536|potassium voltage-gated channel KQT-like subfamily member 2	gi|54873644|ref|NP_001006669.1|	0	potassium voltage-gated channel subfamily KQT member 2 isoform 4 [Mus musculus]
mmu-miR-1199-3p	NM_001006669	-29.1	0.491648	-31.79	158	GO:0005887,GO:0032991,GO:0005267,GO:0005515,GO:0034220,GO:0030001,	K04927|1|0.0|1340|mmu:16536|potassium voltage-gated channel KQT-like subfamily member 2	gi|54873646|ref|NP_001006670.1|	0	potassium voltage-gated channel subfamily KQT member 2 isoform 5 [Mus musculus]
mmu-miR-1199-3p	NM_001006674	-29.1	0.431223	-31.79	158	GO:0005887,GO:0032991,GO:0005267,GO:0005515,GO:0034220,GO:0030001,	K04927|1|0.0|1058|mmu:16536|potassium voltage-gated channel KQT-like subfamily member 2	gi|148675435|gb|EDL07382.1|	0	potassium voltage-gated channel, subfamily Q, member 2, isoform CRA_i [Mus musculus]

mmu-miR-1199-3p	NM_001007465	-31.9	0.236872	-28.14	148	GO:0043227,GO:0031410,GO:0046914,GO:0003824,GO:0012501,GO:0015031,	K10871|1|5e-70|267|mcc:718665|RAD51-like protein 3!K10641|3|3e-10|68.6|tad:TRIADDRAFT_53391|E3 ubiquitin-protein ligase LRSAM1 [EC:6.3.2.19]!K16060|4|4e-09|65.1|spu:581540|baculoviral IAP repeat-containing protein 2/3!K10637|5|5e-	gi|148683726|gb|EDL15673.1|	0	ring finger and FYVE like domain containing protein, isoform CRA_c [Mus musculus]
mmu-miR-1199-3p	NM_001007577	-36.9	0.043491	-25.33	154	GO:0043231,GO:0003676,GO:0006355,	K03145|1|3e-27|125|dgr:Dgri_GH10818|transcription elongation factor S-II	gi|145699116|ref|NP_001007578.2|	0	transcription elongation factor A N-terminal and central domain-containing protein [Mus musculus]

mmu-miR-1199-3p	NM_001007589	-33.3	0.0612	-61.3	325	GO:0043231,GO:0043234,GO:0006357,GO:0001819,GO:0002237,GO:0007275,GO:00082	-	gi|86604728|ref|NP_001007590.2|	2.67E-69	akirin-2 [Mus musculus]
mmu-miR-1199-3p	NM_001008422	-27.2	0.876911	-31.34	163	GO:0043231,GO:0005515,GO:0006351,GO:0006396,	-	gi|148690840|gb|EDL22787.1|	0	expressed sequence AI480556 [Mus musculus]
mmu-miR-1199-3p	NM_001008542	-28.3	0.809319	-87.46	440	GO:0043231,GO:0003712,GO:0003676,GO:0006357,GO:0042992,GO:0008283,	K09114|1|6e-129|463|mmu:17859|MAX dimerization protein	gi|114520596|ref|NP_001008542.2|	3.95E-128	max-interacting protein 1 isoform b [Mus musculus]
mmu-miR-1199-3p	NM_001009573	-25.6	0.976203	-25.42	146	GO:0000323,GO:0030133,GO:0005768,GO:0005488,GO:0002886,GO:0002323,GO:0017157,GO:0006897,GO:0002460,	K15621|1|0.0|1792|ecb:100059416|BAI1-associated protein 3	gi|62243588|ref|NP_001009573.2|	0	protein unc-13 homolog D [Mus musculus]

mmu-miR-1199-3p	NM_001009929	-27.5	0.841109	-29.89	154	GO:0046872,GO:0004672,GO:0032559,GO:0048858,GO:0030154,GO:0007163,GO:00064	K08796|1|0.0|1273|mmu:75770|BR serine/threonine kinase [EC:2.7.11.1]	gi|58036483|ref|NP_001009929.1|	0	serine/threonine-protein kinase BRSK2 isoform beta [Mus musculus]
mmu-miR-1199-3p	NM_001009930	-27.5	0.863609	-55.12	304	GO:0046872,GO:0004672,GO:0032559,GO:0030154,GO:0007163,GO:0006464,	K08796|1|0.0|1290|rno:293631|BR serine/threonine kinase [EC:2.7.11.1]	gi|58036485|ref|NP_001009930.1|	0	serine/threonine-protein kinase BRSK2 isoform gamma [Mus musculus]
mmu-miR-1199-3p	NM_001011518	-36.2	0.00994	-31.16	153	GO:0016020,GO:0004872,GO:0023052,	K04257|1|1e-80|300|mdo:100618888|olfactory receptor	gi|58372122|ref|NP_001011518.1|	0	olfactory receptor 94 [Mus musculus]
mmu-miR-1199-3p	NM_001012396	-34.6	0.013393	-32.34	161	GO:0031224,GO:0043231,GO:0031090,GO:0019212,GO:0004721,GO:0006631,GO:0023052,GO:0032501,GO:0006464,	K10703|1|5e-110|234|bta:615191|3-hydroxyacyl-CoA dehydratase [EC:4.2.1.-]	gi|81869460|sp|Q9QY80.1|HACD1_MOUSE	1.60E-110	RecName: Full=3-hydroxyacyl-CoA dehydratase 1; Short=HACD1; AltName: Full=Protein-tyrosine phosphatase-like member A

mmu-miR-1199-3p	NM_001012765	-33.9	0.170414	-31.41	158	GO:0043231,GO:0031224,GO:0005886,GO:0004016,GO:0032559,GO:0043169,GO:0035588,GO:0015980,GO:0007267,GO:0007193,GO:0033762,GO:0032147,GO:0010863,GO:0007610,GO:0007169,GO:0007189,GO:0050905,GO:0042044,	K08045|1|0.0|2018|mmu:224129|adenylate cyclase 5 [EC:4.6.1.1]	gi|148747309|ref|NP_001012783.3|	0	adenylate cyclase type 5 [Mus musculus]
mmu-miR-1199-3p	NM_001013012	-30.8	0.186033	-59.04	297	GO:0043231,GO:0046914,GO:0003676,GO:0010467,	K09228|1|5e-60|233|bta:785799|KRAB domain-containing zinc finger protein	gi|67846115|ref|NP_001013030.1|	2.46E-180	zinc finger protein 787 [Mus musculus]
mmu-miR-1199-3p	NM_001013028	-26.1	0.946845	-57.26	305	GO:0016020,	-	gi|61557491|ref|NP_001013046.1|	5.46E-39	UPF0444 transmembrane protein C12orf23 homolog [Mus musculus]

mmu-miR-1199-3p	NM_001013360	-29.3	0.634591	-36.67	171	GO:0016020,GO:0001847,GO:0023052,	K11453|1|2e-153|544|ecb:100055293|chromobox protein 6	gi|113374168|ref|NP_001013378.2|	0	neuronal pentraxin with chromo domain isoform 1 [Mus musculus]
mmu-miR-1199-3p	NM_001013362	-29.3	0.656906	-36.67	171	GO:0016020,GO:0001847,GO:0023052,	K11453|1|2e-153|545|ecb:100055293|chromobox protein 6	gi|113374171|ref|NP_001013380.2|	0	neuronal pentraxin with chromo domain isoform 2 [Mus musculus]
mmu-miR-1199-3p	NM_001013375	-31.6	0.195009	-25.36	151	GO:0031981,GO:0042254,	K14553|1|0.0|965|mmu:217109|U3 small nucleolar RNA-associated protein	gi|61656180|ref|NP_001013393.1|	0	U3 small nucleolar RNA-associated protein 18 homolog [Mus musculus]
mmu-miR-1199-3p	NM_001013389	-30.4	0.232709	-31.18	148	GO:0019866,GO:0031224,GO:0043231,GO:0006810,	K16075|1|0.0|867|mmu:380836|magnesium transporter	gi|61656188|ref|NP_001013407.1|	0	magnesium transporter MRS2 homolog, mitochondrial [Mus musculus]
mmu-miR-1199-3p	NM_001013741	-39.9	0.012072	-36.77	165	GO:0030425,GO:0032589,GO:0031090,GO:0043231,GO:0031224,GO:0043025,	-	gi|169234955|ref|NP_001013763.1|	0	dendrin [Mus musculus]

mmu-miR-1199-3p	NM_001013749	-26.8	0.939209	-58.83	305	GO:0031224,	-	gi|85701444|ref|NP_001013771.1|	0	transmembrane protein 151B [Mus musculus]
mmu-miR-1199-3p	NM_001013758	-35.9	0.052326	-59.52	322	GO:0031224,	K07523|1|2e-32|142|xtr:100188921|netrin-G1 ligand	gi|148699538|gb|EDL31485.1|	0	mCG55384 [Mus musculus]
mmu-miR-1199-3p	NM_001013784	-25.1	0.98984	-53.58	297	-	-	gi|62000658|ref|NP_001013806.1|	3.36E-95	uncharacterized protein LOC432582 [Mus musculus]
mmu-miR-1199-3p	NM_001013826	-34	0.024216	-28.09	152	GO:0044424,GO:0004721,GO:0006470,	K14165|1|1e-123|443|mmu:435391|dual specificity phosphatase [EC:3.1.3.16 3.1.3.48]	gi|85701508|ref|NP_001013848.1|	6.61E-123	dual specificity phosphatase DUPD1 [Mus musculus]
mmu-miR-1199-3p	NM_001013829	-31.4	0.117266	-57.48	301	-	K07365|1|7e-12|72.8|hmg:100201881|NCK adaptor protein	gi|123857940|emb|CAM16326.1|	1.39E-154	Src homology 2 domain containing F [Mus musculus]
mmu-miR-1199-3p	NM_001014836	-31.3	0.040824	-30.48	148	-	-	gi|148699471|gb|EDL31418.1|	1.44E-125	RIKEN cDNA 4930404N11, isoform CRA_a [Mus musculus]
mmu-miR-1199-3p	NM_001014900	-35.9	0.016849	-37	160	GO:0044464,GO:0046914,	-	gi|225543354|ref|NP_001014900.2|	0	zinc finger, MYND domain containing 12 [Mus musculus]

mmu-miR-1199-3p	NM_001017429	-28.7	0.068279	-27.67	146	GO:0005740,GO:0005507,GO:0030234,GO:0007417,GO:0044237,GO:0048513,GO:0000041,	K02260|1|5e-19|93.2|ptr:100609675|cytochrome c oxidase assembly protein subunit	gi|395844800|ref|XP_003795139.1|	3.44E-18	PREDICTED: cytochrome c oxidase copper chaperone-like [Otolemur garnettii]
mmu-miR-1199-3p	NM_001017968	-25.7	0.929056	-27.11	148	GO:0031224,GO:0046914,GO:0008104,	K05766|1|1e-27|126|spu:575378|slingshot	gi|114326532|ref|NP_001017968.2|	3.98E-172	palmitoyltransferase ZDHHC18 [Mus musculus]
mmu-miR-1199-3p	NM_001017985	-34.2	0.23541	-32.38	162	-	-	gi|125347500|ref|NP_001017985.2|	0	C2 domain-containing protein 3 [Mus musculus]
mmu-miR-1199-3p	NM_001024135	-30.4	0.475457	-56.38	298	GO:0005488,	K10474|1|0.0|1268|mmu:211255|kelch repeat and BTB domain-containing protein 6/7	gi|254540156|ref|NP_001019306.2|	0	kelch repeat and BTB (POZ) domain containing 7 [Mus musculus]
mmu-miR-1199-3p	NM_001024142	-30.7	0.446	-26.03	147	GO:0043234,GO:0019787,GO:0032446,	K10296|1|0.0|1867|mmu:269529|F-box protein	gi|32451765|gb|AAH54731.1|	0	Fbxo10 protein, partial [Mus musculus]

mmu-miR-1199-3p	NM_001024539	-29.7	0.523751	-52.17	299	GO:0044444,GO:0007169,GO:0007264,	K06279|1|0.0|1078|mmu:216148|src homology 2 domain-containing transforming protein C	gi|169790806|ref|NP_001019710.1|	0	SHC-transforming protein 2 [Mus musculus]
mmu-miR-1199-3p	NM_001024624	-28.7	0.591791	-29.24	151	GO:0031981,GO:0030426,GO:0030425,GO:0001726,GO:0004674,GO:0032559,GO:0017048,GO:0030307,GO:0031346,GO:0010975,GO:0006468,GO:0032321,GO:00164	K08824|1|0.0|1816|mmu:382253|cyclin-dependent kinase-like [EC:2.7.11.22]	gi|124244037|ref|NP_001019795.1|	0	cyclin-dependent kinase-like 5 [Mus musculus]

mmu-miR-1199-3p	NM_001024645	-26.5	0.950728	-25.43	154	-	K11147|1|1e-40|170|oaa:100092951|dehydrogenase/reductase SDR family member 4 [EC:1.1.-.-	gi|66912205|ref|NP_001019816.1|	0	leucine-rich repeat-containing protein 16B [Mus musculus]
mmu-miR-1199-3p	NM_001024707	-35.6	0.068909	-64.67	322	GO:0012505,GO:0031224,GO:0004871,GO:0006897,	K04550|1|3e-31|138|dvi:Dvir_GJ21697|low density lipoprotein-related protein 1 (alpha-2-macroglobulin receptor)	gi|148539563|ref|NP_001019878.1|	0	low density lipoprotein receptor-related protein 3 precursor [Mus musculus]
mmu-miR-1199-3p	NM_001024720	-29.7	0.949623	-25.54	151	GO:0044459,GO:0044444,GO:0005578,GO:0046872,GO:0006464,GO:0050953,	K12567|1|0.0|1253|rno:84015|titin [EC:2.7.11.1]	gi|154689979|ref|NP_001019891.2|	0	hemicentin 1 precursor [Mus musculus]
mmu-miR-1199-3p	NM_001024926	-31	0.221359	-28.94	152	GO:0005506,GO:0048731,	-	gi|167736380|ref|NP_001020097.2|	9.85E-145	neuferricin precursor [Mus musculus]

mmu-miR-1199-3p	NM_001024927	-26.2	0.990437	-62.95	306	GO:0031224,GO:0005548,GO:0046872,GO:0005102,GO:0050953,GO:0006650,	K13619|1|6e-09|65.5|ame:724115|phospholipase DDHD1 [EC:3.1.1.-]	gi|67972419|ref|NP_001020098.1|	0	membrane-associated phosphatidylinositol transfer protein 3 isoform 1 [Mus musculus]
mmu-miR-1199-3p	NM_001024952	-36.5	0.153144	-31.43	162	GO:0044424,GO:0003676,GO:0046914,GO:0002698,	K15690|1|0.0|2014|mmu:381305|RING finger and CCCH-type zinc finger domain-containin	gi|50511255|dbj|BAD32613.1|	0	mKIAA2025 protein [Mus musculus]

mmu-miR-1199-3p	NM_001025074	-25.9	0.977605	-30.01	158	GO:0044456,GO:0030427,GO:0044430,GO:0044297,GO:0016021,GO:0030425,GO:0033267,GO:0044444,GO:0043121,GO:0004713,GO:0032559,GO:0004872,GO:0023061,GO:0045762,GO:0010975,GO:0007610,GO:0007169,GO:0006468,GO:0042462,GO:00	K04360|1|0.0|1655|mmu:18212|neurotrophic tyrosine kinase receptor type 2 [EC:2.7.10.1]	gi|68215970|ref|NP_001020245.1|	0	BDNF/NT-3 growth factors receptor isoform a precursor [Mus musculus]
mmu-miR-1199-3p	NM_001025263	-32.5	0.120735	-30.91	161	GO:0043231,GO:0044444,GO:0042802,GO:0046872,GO:0046983,GO:0030098,	-	gi|148673248|gb|EDL05195.1|	5.62E-81	mCG10134, isoform CRA_f [Mus musculus]

mmu-miR-1199-3p	NM_001025264	-32.5	0.119816	-30.91	161	GO:0043231,GO:0044444,GO:0042802,GO:0046872,GO:0046983,GO:0030098,	-	gi|148673247|gb|EDL05194.1|	2.59E-78	mCG10134, isoform CRA_e [Mus musculus]
mmu-miR-1199-3p	NM_001025395	-28.9	0.628511	-29.19	150	GO:0044430,GO:0019866,GO:0044459,GO:0044444,GO:0004713,GO:0005506,GO:0035258,GO:0032559,GO:0019904,GO:0019901,GO:0030674,GO:0006006,GO:0010467,GO:0030520,GO:0003018,GO:0030177,GO:0050707,GO:0001894,GO:0032868,GO:0006915,GO:0009628,GO:00074	K05704|1|0.0|1095|mmu:20779|proto-oncogene tyrosine-protein kinase Src [EC:2.7.10.2]	gi|70794809|ref|NP_001020566.1|	0	neuronal proto-oncogene tyrosine-protein kinase Src isoform 2 [Mus musculus]

mmu-miR-1199-3p	NM_001025438	-34.8	0.102794	-37.92	157	GO:0031981,GO:0030139,GO:0042383,GO:0043234,GO:0045202,GO:0005515,GO:0004674,GO:0032559,GO:0006941,GO:0007267,GO:0008283,GO:0019221,GO:0006468,GO:0070838,GO:0006950,GO:0016049,GO:0000086,GO:0006875,	K04515|1|0.0|1026|mmu:108058|calcium/calmodulin-dependent protein kinase (CaM kinase) II [EC:2.7.11.17]	gi|26667180|ref|NP_001212.2|	0	calcium/calmodulin-dependent protein kinase type II subunit delta isoform 3 [Homo sapiens]

mmu-miR-1199-3p	NM_001025439	-34.8	0.103147	-37.92	157	GO:0043234,GO:0031981,GO:0045202,GO:0030139,GO:0042383,GO:0004674,GO:0005515,GO:0032559,GO:0006941,GO:0006468,GO:0000086,GO:0006875,GO:0070838,GO:0008283,GO:0006950,	K04515|1|0.0|1045|mcc:702504|calcium/calmodulin-dependent protein kinase (CaM kinase) II [EC:2.7.11.17]	gi|60360170|dbj|BAD90304.1|	0	mKIAA4163 protein [Mus musculus]
mmu-miR-1199-3p	NM_001025570	-30.3	0.453701	-25.18	145	GO:0043231,GO:0003712,GO:0001071,GO:0003677,GO:0048704,GO:0048514,GO:0009888,GO:0042471,GO:0006357,GO:0008589,GO:0035108,GO:0008284,	K09329|1|2e-102|375|mmu:18933|paired mesoderm homeobox protein	gi|70909354|ref|NP_001020741.1|	1.11E-101	paired mesoderm homeobox protein 1 isoform c [Mus musculus]

mmu-miR-1199-3p	NM_001025577	-30.1	0.438783	-26.73	153	GO:0043231,GO:0044427,GO:0042802,GO:0001071,GO:0046983,GO:0043566,GO:0043010,GO:0006357,GO:0002062,	K09035|1|2e-59|232|mmu:17132|transcription factor Maf	gi|67773400|gb|AAY81957.1|	1.33E-58	c-Maf long form [Mus musculus]
mmu-miR-1199-3p	NM_001025597	-25.3	0.994914	-52.37	293	GO:0043231,GO:0000775,GO:0043234,GO:0046914,GO:0001071,GO:0046983,GO:0043565,GO:0009888,GO:0000122,GO:0048537,GO:0007420,GO:0030854,GO:0035264,GO:0046638,GO:0006325,	K09220|1|0.0|1043|mmu:22778|IKAROS family zinc finger protein	gi|60359882|dbj|BAD90160.1|	0	mKIAA4227 protein [Mus musculus]
mmu-miR-1199-3p	NM_001025615	-28.7	0.846191	-25.91	154	GO:0044424,GO:0005488,	-	gi|148665283|gb|EDK97699.1|	1.20E-135	coiled-coil domain containing 50, isoform CRA_a [Mus musculus]

mmu-miR-1199-3p	NM_001029850	-25.7	0.998267	-72.16	444	GO:0043296,GO:0043231,GO:0032559,	K05631|1|0.0|2546|mmu:14924|atrophin-1 interacting protein 3 (BAI1-associated protein 1)	gi|71533173|ref|NP_001025021.1|	0	membrane-associated guanylate kinase, WW and PDZ domain-containing protein 1 isoform c [Mus musculus]
mmu-miR-1199-3p	NM_001029890	-34.4	0.163874	-56.07	311	GO:0030529,GO:0043231,GO:0046914,GO:0003676,	K15686|1|0.0|778|mmu:72640|RNA-binding protein MEX3	gi|308193357|ref|NP_001025061.2|	0	RNA-binding protein MEX3A [Mus musculus]
mmu-miR-1199-3p	NM_001030292	-36.1	0.00861	-67.32	305	GO:0031224,GO:0005261,GO:0030001,	K04923|1|0.0|648|mmu:241769|potassium channel subfamily K member	gi|71892416|ref|NP_001025463.1|	0	potassium channel subfamily K member 15 [Mus musculus]
mmu-miR-1199-3p	NM_001030296	-34.8	0.023281	-26.63	148	-	-	gi|71892426|ref|NP_001025467.1|	6.29E-60	proline-rich protein 7 [Mus musculus]

mmu-miR-1199-3p	NM_001033124	-28.7	0.508497	-57.25	298	GO:0043231,GO:0016021,GO:0019838,GO:0004713,GO:0004674,GO:0032559,GO:0004872,GO:0070848,GO:0048017,GO:0045762,GO:0010863,GO:0007169,GO:0007264,GO:0006468,GO:0030098,GO:0043066,GO:0000165,GO:0030182,	K03176|1|0.0|1477|mmu:18211|neurotrophic tyrosine kinase receptor type 1 [EC:2.7.10.1]	gi|169234626|ref|NP_001028296.1|		0	high affinity nerve growth factor receptor precursor [Mus musculus]
mmu-miR-1199-3p	NM_001033135	-28.9	0.456071	-25.57	153	GO:0016020,GO:0046872,	K15704|1|0.0|694|mmu:67702|E3 ubiquitin-protein ligase RNF149 [EC:6.3.2.19]	gi|224809379|ref|NP_001028307.2|		0	E3 ubiquitin-protein ligase RNF149 precursor [Mus musculus]

mmu-miR-1199-3p	NM_001033136	-27.3	0.661596	-25.49	149	GO:0005740,GO:0031224,GO:0005819,GO:0005488,GO:0012501,GO:0048869,	-	gi|33585791|gb|AAH55754.1|	0	Family with sequence similarity 82, member A2 [Mus musculus]
mmu-miR-1199-3p	NM_001033155	-27.5	0.553591	-115.3	590	GO:0031224,GO:0005515,GO:0044267,	K09520|1|0.0|689|mmu:70604|DnaJ homolog subfamily B member 14	gi|226371690|ref|NP_001028327.1|	0	dnaJ homolog subfamily B member 14 [Mus musculus]
mmu-miR-1199-3p	NM_001033157	-31.6	0.183585	-29.95	156	-	K09228|1|3e-142|474|mmu:233987|KRAB domain-containing zinc finger protein	gi|85701598|ref|NP_001028329.1|	0	zinc finger protein-like isoform 2 [Mus musculus]

mmu-miR-1199-3p	NM_001033161	-27.2	0.484016	-49.04	291	GO:0043234,GO:0043232,GO:0044425,GO:0044444,GO:0060089,GO:0032403,GO:0019899,GO:0019904,GO:0006917,GO:0051259,GO:0019221,GO:0045684,GO:00072	K03171|1|2e-153|543|mmu:71609|tumor necrosis factor receptor type 1-associated DEATH domain protein	gi|75677522|ref|NP_001028333.1|	1.11E-152	tumor necrosis factor receptor type 1-associated DEATH domain protein [Mus musculus]
mmu-miR-1199-3p	NM_001033166	-25.1	0.529981	-29.92	152	GO:0005488,	-	gi|76781489|ref|NP_001028338.1|	1.68E-49	selenoprotein H [Mus musculus]
mmu-miR-1199-3p	NM_001033167	-26.6	0.977116	-27.51	147	GO:0031224,GO:0005215,GO:0006810,	K08215|1|0.0|1262|mmu:73102|MFS transporter, OCT family, solute carrier family 22 (organic anion transporter), member 23	gi|85701678|ref|NP_001028339.1|	0	solute carrier family 22 member 23 [Mus musculus]

mmu-miR-1199-3p	NM_001033173	-28.3	0.95219	-30.67	155	GO:0008233,	K11852|1|0.0|1986|mmu:76179|ubiquitin carboxyl-terminal hydrolase 31 [EC:3.1.2.15]	gi|226443061|ref|NP_001028345.1|	0	ubiquitin specific protease 31 [Mus musculus]
mmu-miR-1199-3p	NM_001033174	-26	0.973668	-28.62	152	GO:0012505,GO:0043231,GO:0032934,GO:0051234,GO:0010876,	K08283|1|2e-20|102|tad:TRIADDRAFT_22163|Goodpasture-antigen-binding protein kinase [EC:2.7.11.9]	gi|205829307|sp|Q3B7Z2.2|OSBP1_MOUSE	0	RecName: Full=Oxysterol-binding protein 1

mmu-miR-1199-3p	NM_001033201	-26.3	0.899424	-34.21	149	GO:0031981,GO:0034518,GO:0044444,GO:0003723,GO:0006413,GO:0016458,GO:0000956,GO:0006353,GO:0031124,GO:0000398,GO:0006354,GO:0045935,GO:00064	K12882|1|0.0|1523|mmu:433702|nuclear cap-binding protein subunit 1	gi|144922627|ref|NP_001028373.2|	0	nuclear cap-binding protein subunit 1 [Mus musculus]
mmu-miR-1199-3p	NM_001033212	-28.2	0.268624	-36.98	181	GO:0031224,	K10128|1|8e-24|111|bta:614739|reprimo, TP53-dependent G2 arrest mediator candidate	gi|85701722|ref|NP_001028384.1|	3.10E-61	reprimo-like protein [Mus musculus]
mmu-miR-1199-3p	NM_001033145	-20.8	1	-82.4	449	GO:0005798,GO:0030120,	-	gi|92058707|sp|Q3USZ8.1|DIA1_MOUSE	0	RecName: Full=Deleted in autism protein 1 homolog; Flags: Precursor
mmu-miR-1199-5p	NM_001001160	-31	0.476351	-27.52	160	GO:0005488,	K10316|1|0.0|1285|mmu:330369|F-box protein	gi|296482725|gb|DAA24840.1|	0	F-box protein 41-like [Bos taurus]

mmu-miR-1199-5p	NM_001001333	-29.8	0.268106	-26.23	152	GO:0043231,GO:0015929,GO:0043167,GO:0044238,	K14459|1|0.0|967|mmu:238023|hexosaminidase [EC:3.2.1.52]	gi|225703121|ref|NP_001001333.2|	0	hexosaminidase D isoform 2 [Mus musculus]
mmu-miR-1199-5p	NM_001001492	-29.8	0.383025	-30.41	159	-	K10421|1|3e-09|65.5|bta:534624|CAP-Gly domain-containing linker protein 1	gi|124517724|ref|NP_001001492.2|	0	Lebercilin-like protein [Mus musculus]
mmu-miR-1199-5p	NM_001001495	-27.4	0.78438	-25.81	147	-	-	gi|110665744|ref|NP_001001495.2|	1.78E-98	RIKEN cDNA 9030611K07 [Mus musculus]

mmu-miR-1199-5p	NM_001001602	-32.3	0.318765	-31.53	155	GO:0009898,GO:0044424,GO:0008047,GO:0060090,GO:0005083,GO:0042802,GO:0019901,GO:0019903,GO:0032813,GO:0006915,GO:0035411,GO:0016049,GO:0032318,GO:0034260,GO:0043433,GO:0001837,GO:0006469,GO:0070849,GO:0030178,GO:0007173,GO:0000122,GO:00082	K04352|1|4e-46|188|cin:100184835|Ras GTPase-activating protein 1	gi|166295210|ref|NP_001001602.2|	0	disabled homolog 2-interacting protein isoform 3 [Mus musculus]
mmu-miR-1199-5p	NM_001003815	-29.8	0.62739	-27.32	153	GO:0043232,GO:0044444,GO:0044425,GO:0008092,GO:0007267,GO:0030036,	K06107|1|0.0|1568|mmu:13821|erythrocyte membrane protein band 4.1	gi|54873604|ref|NP_001003815.1|	0	band 4.1-like protein 1 isoform b [Mus musculus]

mmu-miR-1199-5p	NM_001003817	-31.4	0.341002	-28.77	165	GO:0031224,GO:0043234,GO:0016323,GO:0043231,GO:0042995,GO:0043209,GO:0044444,GO:0004714,GO:0005102,GO:0032559,GO:0019902,GO:0003676,GO:0046983,GO:0031072,GO:0006915,GO:0043405,GO:0008284,GO:0048017,GO:0048732,GO:0033080,GO:0007411,GO:00226	K05083|1|0.0|2392|mmu:13866|receptor tyrosine-protein kinase erbB-2 [EC:2.7.10.1]	gi|37360638|dbj|BAC98297.1|	0	mKIAA3023 protein [Mus musculus]

mmu-miR-1199-5p	NM_001003912	-30.5	0.488425	-26.89	160	GO:0016020,GO:0043231,GO:0044444,GO:0005102,GO:0005088,GO:0060089,GO:0008047,GO:0007010,GO:0007266,GO:0007163,GO:0006355,GO:0006936,GO:0007169,GO:0006917,GO:0009966,GO:0006935,GO:00	K12331|1|0.0|2758|mmu:213498|Rho guanine nucleotide exchange factor 11	gi|51491850|ref|NP_001003912.1|	0	rho guanine nucleotide exchange factor 11 [Mus musculus]
mmu-miR-1199-5p	NM_001008425	-29	0.239592	-25.85	149	GO:0000346,GO:0003676,GO:0006406,GO:0006396,	K13175|1|0.0|642|mmu:386612|THO complex subunit 6	gi|56605680|ref|NP_001008425.1|	0	THO complex subunit 6 homolog [Mus musculus]
mmu-miR-1199-5p	NM_001009546	-26.1	0.788325	-27.74	155	GO:0044459,GO:0031224,GO:0008238,GO:0043169,GO:0019538,	K01301|1|0.0|1360|mmu:381204|glutamate carboxypeptidase II [EC:3.4.17.21]	gi|57222282|ref|NP_001009546.1|	0	N-acetylated-alpha-linked acidic dipeptidase-like protein [Mus musculus]

mmu-miR-1199-5p	NM_001012518	-27.7	0.83782	-46.64	302	GO:0043231,GO:0043232,GO:0046914,GO:0018024,GO:0005515,GO:0034968,GO:0006357,GO:0006305,GO:0018022,GO:00072	K11420|1|0.0|2434|mmu:77683|euchromatic histone-lysine N-methyltransferase [EC:2.1.1.43]	gi|157951633|ref|NP_001012536.2|	0	histone-lysine N-methyltransferase EHMT1 isoform 1 [Mus musculus]
mmu-miR-1199-5p	NM_001017407	-26.1	0.440569	-26.75	155	GO:0044444,GO:0005488,GO:0003006,GO:0048232,GO:0048869,	-	gi|148700204|gb|EDL32151.1|	7.42E-90	RIKEN cDNA 1700021K02, isoform CRA_a [Mus musculus]
mmu-miR-1199-5p	NM_001017409	-26.1	0.468422	-26.75	155	GO:0044444,GO:0005488,GO:0003006,GO:0048232,GO:0048869,	-	gi|63079697|ref|NP_001017409.1|	7.47E-91	protein SPATIAL isoform 6 [Mus musculus]
mmu-miR-1199-5p	NM_001017419	-26.1	0.620483	-26.75	155	-	-	gi|31414725|gb|AAP50907.1|	1.53E-168	spatial-delta [Mus musculus]
mmu-miR-1199-5p	NM_001017433	-26.1	0.648447	-26.75	155	GO:0044444,GO:0005488,GO:0003006,GO:0048232,GO:0048869,	-	gi|31414727|gb|AAP50908.1|	0	spatial-epsilon [Mus musculus]

mmu-miR-1199-5p	NM_001017441	-26.1	0.474466	-26.75	155	GO:0044444,GO:0005488,GO:0003006,GO:0048232,GO:0048869,	-	gi|31414725|gb|AAP50907.1|	1.83E-100	spatial-delta [Mus musculus]
mmu-miR-1199-5p	NM_001024624	-27.1	0.775603	-26.52	156	GO:0031981,GO:0030426,GO:0030425,GO:0001726,GO:0004674,GO:0032559,GO:0017048,GO:0030307,GO:0031346,GO:0010975,GO:0006468,GO:0032321,GO:00164	K08824|1|0.0|1816|mmu:382253|cyclin-dependent kinase-like [EC:2.7.11.22]	gi|124244037|ref|NP_001019795.1|	0	cyclin-dependent kinase-like 5 [Mus musculus]
mmu-miR-1199-5p	NM_001024703	-34.6	0.13167	-32.5	160	GO:0031224,GO:0046872,GO:0005543,GO:0019932,	K15293|1|3e-11|72.8|tad:TRIADDRAFT_22095|protein unc-13	gi|85701536|ref|NP_001019874.1|	0	multiple C2 and transmembrane domain-containing protein 2 [Mus musculus]

mmu-miR-1199-5p	NM_001025192	-28	0.826395	-26.81	156	GO:0043296,GO:0070161,GO:0016021,GO:0030141,GO:0005488,GO:0004871,GO:0006996,GO:0002682,GO:0051704,GO:0034330,GO:0023052,GO:0008285,GO:0048747,GO:0048513,GO:0007599,	K06788|1|0.0|711|mmu:13052|coxsackievirus and adenovirus receptor	gi|68510034|ref|NP_001020363.1|	0	coxsackievirus and adenovirus receptor homolog isoform a precursor [Mus musculus]
mmu-miR-1199-5p	NM_001025310	-26.5	0.756123	-25.58	151	GO:0031228,GO:0008373,GO:0001573,GO:0006464,	K03376|1|2e-177|623|mmu:50935|N-acetylgalactosaminide alpha-2,6-sialyltransferase (sialyltransferase 7F) [EC:2.4.99.	gi|148676612|gb|EDL08559.1|	0	ST6 (alpha-N-acetyl-neuraminyl-2,3-beta-galactosyl-1,3)-N-acetylgalactosaminide alpha-2,6-sialyltransferase 6, isoform CRA_c [Mus musculus]

mmu-miR-1199-5p	NM_001025311	-26.5	0.738593	-25.58	151	GO:0031228,GO:0008373,GO:0001573,GO:0006464,	K03376|1|4e-175|615|mmu:50935|N-acetylgalactosaminide alpha-2,6-sialyltransferase (sialyltransferase 7F) [EC:2.4.99.	gi|70778764|ref|NP_001020482.1|	0	alpha-N-acetylgalactosaminide alpha-2,6-sialyltransferase 6 isoform 3 [Mus musculus]
mmu-miR-1199-5p	NM_001025432	-25.4	0.997997	-41.84	306	-	K04498|1|0.0|3832|mmu:12914|E1A/CREB-binding protein [EC:2.3.1.48]	gi|70995311|ref|NP_001020603.1|	0	CREB-binding protein [Mus musculus]
mmu-miR-1199-5p	NM_001029993	-29.1	0.54279	-49.2	308	GO:0005488,	K13092|1|1e-180|634|mmu:76014|nuclear protein NHN1	gi|71979671|ref|NP_001025164.1|	9.34E-180	zinc finger CCCH domain-containing protein 18 isoform a [Mus musculus]
mmu-miR-1199-5p	NM_001029994	-29.1	0.535683	-49.2	308	GO:0005488,	K13092|1|2e-162|574|mmu:76014|nuclear protein NHN1	gi|71725355|ref|NP_001025165.1|	1.28E-165	zinc finger CCCH domain-containing protein 18 isoform b [Mus musculus]

mmu-miR-1199-5p	NM_001030290	-26.4	0.574067	-25.06	150	GO:0016021,GO:0044424,GO:0008106,GO:0033764,GO:0006694,GO:0050953,GO:00015	K11150|1|6e-156|551|mmu:235033|retinol dehydrogenase 8 [EC:1.1.1.-	gi|71892412|ref|NP_001025461.1|	4.31E-155	retinol dehydrogenase 8 [Mus musculus]
mmu-miR-1199-5p	NM_001033312	-29	0.447196	-28	154	-	K10284|1|0.0|1343|rno:500484|F-box and leucine-rich repeat protein	gi|392352338|ref|XP_001053150.3|	0	PREDICTED: F-box/LRR-repeat protein 18-like [Rattus norvegicus]
mmu-miR-1199-5p	NM_001033316	-28.3	0.345288	-26.06	162	GO:0016021,GO:0005488,GO:0004888,GO:0007166,	K04326|1|2e-160|566|mmu:233080|free fatty acid receptor 3	gi|85701812|ref|NP_001028488.1|	1.50E-159	free fatty acid receptor 3 [Mus musculus]
mmu-miR-1199-5p	NM_001033348	-26.7	0.990604	-25.12	157	GO:0043231,GO:0044444,GO:0005099,GO:0046983,GO:0032852,	K07207|1|2e-22|110|bfo:BRAFLDRAFT_64809|tuberous sclerosis 2	gi|158187505|ref|NP_001028520.2|	0	ral GTPase-activating protein subunit alpha-2 [Mus musculus]

mmu-miR-1199-5p	NM_001033364	-26.2	0.933567	-68.82	450	GO:0044459,GO:0031224,GO:0005488,GO:0016049,GO:0007155,	K06813|1|5e-82|307|hsa:100653137|cadherin 23	gi|282165827|ref|NP_001028536.2|	0	protocadherin 24 precursor [Mus musculus]
mmu-miR-1199-5p	NM_001033416	-25.7	0.924451	-42.9	297	GO:0000139,GO:0031224,GO:0030554,GO:0050694,GO:0005975,GO:0006029,GO:00071	K09677|1|0.0|904|mmu:330217|galactose-3-O-sulfotransferase 4 [EC:2.8.2.-]	gi|84370027|ref|NP_001028588.1|	0	galactose-3-O-sulfotransferase 4 [Mus musculus]
mmu-miR-1199-5p	NM_001033420	-25.7	0.994013	-47.91	305	GO:0044444,GO:0008047,GO:0030695,GO:0019899,GO:0032561,GO:0019904,GO:0012501,GO:0035556,GO:0006909,GO:0048870,GO:0006935,GO:0007599,GO:00071	K13708|1|0.0|3465|mmu:330662|dedicator of cytokinesis 1	gi|88853584|ref|NP_001028592.1|	0	dedicator of cytokinesis protein 1 [Mus musculus]

mmu-miR-1199-5p	NM_001033455	-31.1	0.157687	-26.63	157	-	K08286|1|2e-06|55.1|oaa:100076537|protein-serine/threonine kinase [EC:2.7.11.-]	gi|85701967|ref|NP_001028627.1|	0	coiled-coil domain-containing protein 27 [Mus musculus]
mmu-miR-1199-5p	NM_001033466	-30.1	0.353154	-25.49	156	GO:0043231,GO:0046914,GO:0003676,	K10489|1|0.0|999|mmu:381990|zinc finger and BTB domain-containing protein 2	gi|226423893|ref|NP_001028638.2|	0	zinc finger and BTB domain containing 2 [Mus musculus]

mmu-miR-1199-5p	NM_001033548	-26.7	0.691765	-29.88	158	-	K04574|1|2e-07|58.5|oaa:100087063|neurofilament heavy polypeptide!K11423|2|3e-07|58.2|dre:100535983|histone-lysine N-methyltransferase SETD2 [EC:2.1.1.43]!K14007|3|3e-06|54.7|dre:100000526|protein transport protein SEC24!K07515|5|4e	gi|85702051|ref|NP_001028720.1|	0	dynein assembly factor 3, axonemal [Mus musculus]
mmu-miR-1199-5p	NM_001033573	-28.3	0.467804	-27.01	157	GO:0031224,GO:0046914,GO:0016747,	K05766|1|2e-14|82.0|spu:575378|slingshot	gi|148669781|gb|EDL01728.1|	0	mCG18121, isoform CRA_c [Mus musculus]

mmu-miR-1199-5p	NM_001033813	-27	0.758037	-27.72	149	GO:0005622,GO:0046872,GO:0003676,	K09228|1|0.0|1004|mmu:619310|KRAB domain-containing zinc finger protein	gi|85702145|ref|NP_001028985.1|	0	zinc finger protein 564 [Mus musculus]
mmu-miR-1199-5p	NM_001034895	-25.2	0.964995	-25.01	161	-	K13203|1|8e-174|612|oaa:100077137|zinc finger RNA-binding protein	gi|163954931|ref|NP_001030067.2|	0	zinc finger RNA-binding protein 2 [Mus musculus]
mmu-miR-1199-5p	NM_001038230	-26	0.912632	-27.44	164	GO:0031981,GO:0000152,GO:0044444,GO:0046914,GO:0019787,GO:0043161,GO:0045841,GO:0007050,GO:0051439,	K03358|1|9e-53|209|cfa:483370|anaphase-promoting complex subunit 11	gi|13384770|ref|NP_079665.1|	9.41E-51	anaphase-promoting complex subunit 11 [Mus musculus]

mmu-miR-1199-5p	NM_001038637	-28.4	0.632046	-27.55	158	GO:0043231,GO:0031234,GO:0017111,GO:0005488,GO:0060089,GO:0001775,GO:0007267,GO:0033762,GO:0009207,GO:0015980,GO:0007599,GO:0007166,	K07826|1|3e-34|148|cfa:610817|guanine nucleotide-binding protein G(I)/G(S)/G(O) subunit gamma-2	gi|148669586|gb|EDL01533.1|	2.73E-35	mCG144516 [Mus musculus]
mmu-miR-1199-5p	NM_001038695	-27.6	0.834673	-27.5	148	GO:0043231,GO:0046914,GO:0016701,GO:0035258,GO:0048869,GO:0030518,GO:0070076,GO:0048232,GO:0006351,GO:00442	K15601|1|0.0|2693|mmu:104263|lysine-specific demethylase 3 [EC:1.14.11.-]	gi|37360072|dbj|BAC98014.1|	0	mKIAA0742 protein [Mus musculus]

mmu-miR-1199-5p	NM_001039149	-27.7	0.529759	-26.09	152	GO:0016021,GO:0004871,GO:0005102,GO:0019900,GO:0002420,GO:0023052,GO:0002696,GO:0001912,GO:0002714,GO:00325	K06567|1|4e-108|393|mmu:225825|CD226 antigen	gi|85540459|ref|NP_001034238.1|	2.47E-107	CD226 antigen isoform b [Mus musculus]
mmu-miR-1199-5p	NM_001039162	-25.8	0.975338	-52.15	306	GO:0043005,GO:0005874,GO:0030141,GO:0008017,GO:0007019,	K10422|1|0.0|1769|mmu:269713|CAP-Gly domain-containing linker protein 2	gi|148687480|gb|EDL19427.1|	0	cytoplasmic linker 2, isoform CRA_b [Mus musculus]
mmu-miR-1199-5p	NM_001039175	-26.6	0.578118	-26.02	156	GO:0031227,GO:0004312,GO:0005488,GO:0000038,GO:0035336,GO:0006641,GO:0019368,GO:0006665,	K10247|1|3e-120|432|mmu:54325|elongation of very long chain fatty acids protein 1	gi|148698556|gb|EDL30503.1|	6.62E-120	elongation of very long chain fatty acids (FEN1/Elo2, SUR4/Elo3, yeast)-like 1, isoform CRA_a [Mus musculus]

mmu-miR-1199-5p	NM_001039176	-26.6	0.626018	-26.02	156	GO:0031227,GO:0004312,GO:0005488,GO:0000038,GO:0035336,GO:0006641,GO:0019368,GO:0006665,	K10247|1|7e-160|564|aml:100476157|elongation of very long chain fatty acids protein 1	gi|9507145|ref|NP_062295.1|	1.34E-164	elongation of very long chain fatty acids protein 1 isoform 1 [Mus musculus]
mmu-miR-1199-5p	NM_001039351	-26.8	0.849324	-43.65	298	GO:0005654,GO:0005515,GO:0001071,GO:0030515,GO:0005048,GO:0006355,GO:0008283,GO:0016074,GO:0006950,GO:00066	-	gi|86198327|ref|NP_001034440.1|	1.32E-98	nucleolar and coiled-body phosphoprotein 1 isoform B [Mus musculus]
mmu-miR-1199-5p	NM_001039352	-26.8	0.849778	-43.65	298	GO:0005654,GO:0005515,GO:0001071,GO:0030515,GO:0005048,GO:0006355,GO:0008283,GO:0016074,GO:0006950,GO:00066	-	gi|86198327|ref|NP_001034440.1|	1.32E-98	nucleolar and coiled-body phosphoprotein 1 isoform B [Mus musculus]

mmu-miR-1199-5p	NM_001039353	-26.8	0.849551	-43.65	298	GO:0005654,GO:0005515,GO:0001071,GO:0030515,GO:0005048,GO:0006355,GO:0008283,GO:0016074,GO:0006950,GO:00066	-	gi|86198327|ref|NP_001034440.1|	1.32E-98	nucleolar and coiled-body phosphoprotein 1 isoform B [Mus musculus]
mmu-miR-1199-5p	NM_001039472	-25.5	0.999077	-55.44	310	GO:0015630,GO:0003774,GO:0032559,GO:0007017,	K10395|1|0.0|2754|mmu:16565|kinesin family member 4/7/21/27	gi|86990454|ref|NP_001034561.1|	0	kinesin-like protein KIF21B [Mus musculus]
mmu-miR-1199-5p	NM_001039514	-28.2	0.293958	-26.4	154	GO:0044444,GO:0016765,GO:0051260,GO:0046203,GO:0018205,	K00809|1|0.0|716|mmu:330817|deoxyhypusine synthase [EC:2.5.1.46]	gi|87252720|ref|NP_001034603.1|	0	deoxyhypusine synthase [Mus musculus]

mmu-miR-1199-5p	NM_001042528	-27.2	0.947344	-54.75	307	GO:0034704,GO:0044297,GO:0030425,GO:0033267,GO:0005515,GO:0005262,GO:0032559,GO:0033555,GO:0007269,GO:0006816,GO:0008015,GO:0044057,GO:0032412,GO:0048545,GO:0007610,GO:00423	K04849|1|0.0|4145|mmu:12287|voltage-dependent calcium channel N type alpha-1B	gi|110225368|ref|NP_001035993.1|	0	voltage-dependent N-type calcium channel subunit alpha-1B isoform 1 [Mus musculus]
mmu-miR-1199-5p	NM_001042615	-26.5	0.725967	-32.98	162	GO:0004175,GO:0019838,GO:0019538,GO:0016049,	K08785|1|0.0|672|mmu:78558|HtrA serine peptidase 3 [EC:3.4.21.-]	gi|110815867|ref|NP_001036080.1|	0	serine protease HTRA3 isoform b precursor [Mus musculus]
mmu-miR-1199-5p	NM_001076790	-29.7	0.183138	-28.98	156	GO:0005815,GO:0032991,GO:0019899,GO:0004866,GO:0010466,GO:0006508,	K13963|1|6e-141|501|mmu:97848|serpin B	gi|115647930|ref|NP_001070258.1|	0	serine (or cysteine) peptidase inhibitor, clade B, member 6d [Mus musculus]

mmu-miR-1199-5p	NM_001077403	-27.2	0.942321	-44.53	307	GO:0031224,GO:0043005,GO:0005488,GO:0004714,GO:0048869,GO:0001667,GO:0006935,GO:0007169,GO:0048513,	K06819|1|0.0|1873|mmu:18187|neuropilin 2	gi|116686132|ref|NP_001070871.1|	0	neuropilin-2 isoform 1 precursor [Mus musculus]
mmu-miR-1199-5p	NM_001077404	-27.2	0.941997	-44.53	307	GO:0031224,GO:0043005,GO:0005488,GO:0004714,GO:0048869,GO:0001667,GO:0006935,GO:0007169,GO:0048513,	K06819|1|0.0|1856|mmu:18187|neuropilin 2	gi|116686140|ref|NP_001070872.1|	0	neuropilin-2 isoform 2 precursor [Mus musculus]
mmu-miR-1199-5p	NM_001077405	-27.2	0.940878	-44.53	307	GO:0031224,GO:0043005,GO:0005488,GO:0004714,GO:0048869,GO:0001667,GO:0006935,GO:0007169,GO:0048513,	K06819|1|0.0|1854|mmu:18187|neuropilin 2	gi|116686142|ref|NP_001070873.1|	0	neuropilin-2 isoform 4 precursor [Mus musculus]

mmu-miR-1199-5p	NM_001077499	-30	0.793713	-71.01	462	GO:0044297,GO:0031410,GO:0033267,GO:0005887,GO:0031420,GO:0005272,GO:0032559,GO:0048513,GO:0006935,GO:0034220,GO:0008366,GO:0006814,GO:00083	K04840|1|0.0|3401|mmu:20273|voltage-gated sodium channel type VIII alpha	gi|117414174|ref|NP_001070967.1|	0	sodium channel protein type 8 subunit alpha isoform 1 [Mus musculus]
mmu-miR-1199-5p	NM_001077632	-31.2	0.089558	-28.32	155	GO:0043231,GO:0005515,GO:0001071,GO:0003677,GO:0007166,GO:0045596,GO:0048664,GO:0021529,GO:0006357,GO:0003312,GO:0003310,GO:0001708,GO:0045687,GO:0003309,GO:0021782,	K08029|1|1e-123|444|mmu:18088|homeobox protein Nkx-2.2	gi|117647245|ref|NP_001071100.1|	6.78E-123	homeobox protein Nkx-2.2 isoform 2 [Mus musculus]

mmu-miR-1199-5p	NM_001078167	-26.6	0.951831	-28.13	155	GO:0005681,GO:0005654,GO:0003676,GO:0019904,GO:0006941,GO:0043009,GO:0000245,GO:0006405,GO:0006353,	K12890|1|2e-79|298|mmu:110809|splicing factor, arginine/serine-rich 1/9	gi|118582271|ref|NP_001071635.1|	1.72E-78	serine/arginine-rich splicing factor 1 isoform 2 [Mus musculus]
mmu-miR-1199-5p	NM_001079883	-37.3	0.069484	-36.44	168	GO:0043231,GO:0000982,GO:0000977,GO:0046872,GO:0042127,GO:0008544,GO:0006357,GO:0021756,GO:0042476,GO:0045058,GO:0009791,GO:0048667,GO:0002681,GO:0006629,GO:0006915,GO:00020	K09228|1|1e-18|97.8|api:100159445|KRAB domain-containing zinc finger protein	gi|120587007|ref|NP_001073352.1|	0	B-cell lymphoma/leukemia 11B isoform a [Mus musculus]

mmu-miR-1199-5p	NM_001079931	-27.3	0.660528	-26.02	143	GO:0031224,GO:0004175,GO:0046914,GO:0005102,GO:0019538,GO:0048232,GO:0048869,	K08613|1|0.0|1210|mmu:13525|disintegrin and metalloproteinase domain-containing protein 26 [EC:3.4.24.-]!K08612|2|0.0|772|ssc:100153750|disintegrin and metalloproteinase domain-containing protein 25 [EC:3.4.24.-]!K08609|3|0.0|770|	gi|121247243|ref|NP_001073400.1|	0	a disintegrin and metallopeptidase domain 34-like [Mus musculus]
mmu-miR-1199-5p	NM_001080979	-30.2	0.314512	-47.94	305	GO:0043234,GO:0003676,GO:0001071,GO:0035556,GO:0006357,GO:0045165,GO:00018	K09448|1|0.0|714|mmu:21679|TEA domain family member 1/3/4	gi|148667455|gb|EDK99871.1|	0	TEA domain family member 4, isoform CRA_b [Mus musculus]

mmu-miR-1199-5p	NM_001081030	-32.5	0.289373	-29.71	156	GO:0043231,GO:0031224,GO:0004721,GO:0019212,GO:0006464,GO:0016049,GO:0048232,GO:0006796,	K01104|1|2e-39|166|mdo:100030849|protein-tyrosine phosphatase [EC:3.1.3.48]	gi|37360634|dbj|BAC98295.1|	0	mKIAA3020 protein [Mus musculus]
mmu-miR-1199-5p	NM_001081177	-26.7	0.946991	-26.04	165	GO:0015630,GO:0003774,GO:0032559,GO:0019900,GO:0046649,GO:0007017,GO:0006886,GO:0023052,	K10392|1|0.0|3371|mmu:16554|kinesin family member 1/13/14	gi|124487163|ref|NP_001074646.1|	0	kinesin-like protein KIF13B [Mus musculus]

mmu-miR-1199-5p	NM_001081195	-29.6	0.624277	-25.18	153	GO:0043229,GO:0043234,GO:0001071,GO:0006355,	K07874|1|9e-24|114|ame:409456|Ras-related protein Rab-1A!K11653|3|3e-09|66.2|hmg:100209641|AT-rich interactive domain-containing protein 1!K11765|5|1e-08|64.3|tca:664298|AT-rich interactive domain-containin	gi|124487105|ref|NP_001074664.1|	0	AT-rich interactive domain-containing protein 4A [Mus musculus]

mmu-miR-1199-5p	NM_001081219	-33.6	0.103431	-34.25	160	GO:0044297,GO:0042995,GO:0005884,GO:0016323,GO:0030427,GO:0008092,GO:0017111,GO:0032559,GO:0051640,GO:0050954,GO:0008104,GO:0030031,	K10356|1|0.0|2015|mmu:432516|myosin I	gi|124487037|ref|NP_001074688.1|	0	unconventional myosin-Ia [Mus musculus]
mmu-miR-1199-5p	NM_001081270	-28.4	0.84065	-27.2	150	GO:0031224,GO:0042802,GO:0007417,GO:0008038,GO:0003002,GO:0016337,GO:0048667,GO:0045165,GO:0048562,	K06768|1|0.0|3999|mmu:114873|Down syndrome cell adhesion molecule like 1	gi|124486901|ref|NP_001074739.1|	0	Down syndrome cell adhesion molecule-like protein 1 homolog [Mus musculus]
mmu-miR-1199-5p	NM_001081289	-28.1	0.493551	-25.93	151	-	-	gi|148675342|gb|EDL07289.1|	0	mCG54925 [Mus musculus]
mmu-miR-1199-5p	NM_001081290	-25.6	0.999438	-27.3	168	-	-	gi|124486835|ref|NP_001074759.1|	0	protein PRRC2C [Mus musculus]

mmu-miR-1199-5p	NM_001081291	-26.1	0.964246	-51.72	313	GO:0044424,GO:0015631,GO:0007010,	K08408|1|2e-32|143|cfa:480418|G protein-coupled receptor 68!K06102|3|2e-07|59.7|aml:100476212|cingulin	gi|124486839|ref|NP_001074760.1|	0	coiled-coil domain-containing protein 88B precursor [Mus musculus]
mmu-miR-1199-5p	NM_001081421	-33.1	0.149029	-32.77	159	GO:0031224,GO:0043231,GO:0030246,GO:0008376,	K00710|1|0.0|1102|mmu:108760|polypeptide N-acetylgalactosaminyltransferase [EC:2.4.1.41]	gi|50510795|dbj|BAD32383.1|	0	mKIAA1130 protein [Mus musculus]

mmu-miR-1199-5p	NM_001082960	-28.2	0.748016	-25.18	151	GO:0043231,GO:0009986,GO:0005887,GO:0004871,GO:0043394,GO:0046983,GO:0005539,GO:0016337,GO:0007599,GO:0042098,GO:0007166,GO:0030595,	K06461|1|0.0|2239|mmu:16409|integrin alpha M	gi|132626289|ref|NP_001076429.1|		0	integrin alpha-M isoform 1 precursor [Mus musculus]
mmu-miR-1199-5p	NM_001083328	-30.8	0.126363	-30.13	160	-	K03979|1|0.0|699|mmu:52856|GTP-binding protein	gi|134031950|ref|NP_001076798.1|		0	GTP binding protein 5 [Mus musculus]
mmu-miR-1199-5p	NM_001083329	-30.8	0.117084	-30.13	160	-	K03979|1|0.0|699|mmu:52856|GTP-binding protein	gi|134031950|ref|NP_001076798.1|		0	GTP binding protein 5 [Mus musculus]
mmu-miR-1199-5p	NM_001085518	-26.8	0.856258	-50.51	313	GO:0016021,GO:0005488,GO:0005337,GO:0015858,GO:0001894,GO:0035725,GO:00346	K11536|1|0.0|1142|mmu:269346|pyrimidine nucleoside transport protein	gi|147901770|ref|NP_001078987.1|		0	solute carrier family 28 (sodium-coupled nucleoside transporter), member 2-like [Mus musculus]

mmu-miR-1199-5p	NM_001097617	-25.9	0.888767	-44.87	288	GO:0031012,GO:0046872,GO:0004175,GO:0030154,GO:0019538,GO:0006952,GO:0010033,GO:0002455,	K01331|1|0.0|1386|mmu:50908|complement component 1, s subcomponent [EC:3.4.21.42]	gi|148277054|ref|NP_659187.2|		0	complement C1s-A subcomponent precursor [Mus musculus]
mmu-miR-1199-5p	NM_001098170	-27.8	0.872405	-34.23	171	GO:0016021,GO:0046872,GO:0016337,	K04602|1|2e-63|246|dre:100008568|cadherin EGF LAG seven-pass G-type receptor 3 (flamingo)	gi|28972738|dbj|BAC65785.1|		0	mKIAA1400 protein [Mus musculus]
mmu-miR-1199-5p	NM_001098171	-27.8	0.933857	-34.23	171	GO:0016021,GO:0046872,GO:0016337,	K04602|1|2e-63|246|dre:100008568|cadherin EGF LAG seven-pass G-type receptor 3 (flamingo)	gi|28972738|dbj|BAC65785.1|		0	mKIAA1400 protein [Mus musculus]

mmu-miR-1199-5p	NM_001098172	-27.8	0.769407	-34.23	171	GO:0016021,GO:0046872,GO:0016337,	K04602|1|1e-63|246|dre:100008568|cadherin EGF LAG seven-pass G-type receptor 3 (flamingo)	gi|28972738|dbj|BAC65785.1|	0	mKIAA1400 protein [Mus musculus]
mmu-miR-1199-5p	NM_001099297	-33.9	0.152701	-31.34	162	GO:0031224,GO:0044459,GO:0000166,GO:0016209,GO:0050664,GO:0050662,GO:0005506,GO:0009058,GO:0006801,GO:0042743,GO:0007275,GO:0007166,GO:0006950,GO:00	K13411|1|0.0|3045|mmu:99439|dual oxidase [EC:1.6.3.1 1.11.1.-]	gi|150010671|ref|NP_001092767.1|	0	dual oxidase 1 precursor [Mus musculus]

mmu-miR-1199-5p	NM_001101502	-33.1	0.118182	-28.81	156	GO:0043231,GO:0005488,GO:0009653,GO:0033598,GO:0007179,GO:0009966,GO:0030855,GO:0007049,	-	gi|160333268|ref|NP_001094972.1|	0	zinc finger protein 703 isoform 1 [Mus musculus]
mmu-miR-1199-5p	NM_001101620	-28	0.278797	-27	153	GO:0016020,GO:0051707,	K10074|1|1e-14|81.6|pon:100457129|C-type lectin domain family 7, member A!K10069|4|3e-13|77.0|bta:536944|C-type lectin domain family 1, member	gi|197333718|ref|NP_001095090.1|	2.41E-158	killer cell lectin-like receptor 8 isoform 1 [Mus musculus]
mmu-miR-1199-5p	NM_001101656	-27.3	0.257453	-46.57	295	GO:0016020,GO:0005488,GO:0004871,GO:0023052,GO:0002573,	K06719|1|2e-28|126|mcc:703483|CD300 antigen	gi|155969725|ref|NP_001095126.1|	3.51E-96	uncharacterized protein LOC100043123 precursor [Mus musculus]

mmu-miR-1199-5p	NM_001101657	-27.3	0.257453	-46.57	295	GO:0016020,GO:0005488,GO:0004871,GO:0023052,GO:0002573,	K06719|1|2e-28|126|mcc:703483|CD300 antigen	gi|155969725|ref|NP_001095126.1|	3.51E-96	uncharacterized protein LOC100043123 precursor [Mus musculus]
mmu-miR-1199-5p	NM_001102563	-29.8	0.310064	-27.76	152	-	-	gi|156523248|ref|NP_001096033.1|	1.04E-144	proline-rich transmembrane protein 2 [Mus musculus]
mmu-miR-1199-5p	NM_001102579	-34.1	0.060953	-30.78	164	-	K04613|1|0.0|1030|rno:293069|vomeronasal 2 receptor	gi|156938269|ref|NP_001096049.1|	0	vomeronasal receptor Vmn2r67 precursor [Mus musculus]
mmu-miR-1199-5p	NM_001104529	-27.8	0.749453	-25.15	152	GO:0016021,GO:0004888,GO:0007166,	K04276|1|2e-164|580|mmu:64095|G protein-coupled receptor 35	gi|157267234|ref|NP_071715.3|	1.69E-163	G-protein coupled receptor 35 [Mus musculus]
mmu-miR-1199-5p	NM_001105056	-25.8	0.870975	-25.56	150	-	K04613|1|0.0|1487|mmu:637873|vomeronasal 2 receptor	gi|157311586|ref|NP_001098526.1|	0	vomeronasal receptor Vmn2r59 precursor [Mus musculus]
mmu-miR-1199-5p	NM_001105057	-25.8	0.86729	-25.56	150	-	K04613|1|0.0|1432|mmu:637873|vomeronasal 2 receptor	gi|157311589|ref|NP_001098527.1|	0	vomeronasal 2, receptor 60 precursor [Mus musculus]

mmu-miR-1199-5p	NM_001105181	-34.1	0.060953	-30.78	164	-		K04613|1|0.0|1038|rno:293069|vomeronasal 2 receptor	gi|157384946|ref|NP_001098651.1|	0	vomeronasal receptor Vmn2r68 precursor [Mus musculus]
mmu-miR-1199-5p	NM_001105182	-30.1	0.283873	-25.48	160	-		K04613|1|0.0|1018|rno:293069|vomeronasal 2 receptor	gi|157384948|ref|NP_001098652.1|	0	vomeronasal 2, receptor 69 precursor [Mus musculus]
mmu-miR-1199-5p	NM_001105183	-33.5	0.077963	-30.03	160	-		K04613|1|0.0|974|rno:293069|vomeronasal 2 receptor	gi|157384950|ref|NP_001098653.1|	0	vomeronasal 2, receptor 70 precursor [Mus musculus]
mmu-miR-1199-5p	NM_001105184	-29.8	0.316844	-25.83	156	-		K04613|1|0.0|1008|rno:293069|vomeronasal 2 receptor	gi|157384952|ref|NP_001098654.1|	0	vomeronasal receptor Vmn2r71 precursor [Mus musculus]
mmu-miR-1199-5p	NM_001105186	-34.1	0.060953	-30.78	164	-		K04613|1|0.0|1045|rno:293069|vomeronasal 2 receptor	gi|157384958|ref|NP_001098656.1|	0	vomeronasal 2, receptor 73 precursor [Mus musculus]
mmu-miR-1199-5p	NM_001105187	-34.1	0.061135	-30.78	164	-		K04613|1|0.0|978|rno:293069|vomeronasal 2 receptor	gi|157384961|ref|NP_001098657.1|	0	vomeronasal receptor Vmn2r74 precursor [Mus musculus]

mmu-miR-1199-5p	NM_001105188	-34.1	0.061226	-30.78	164	-	K04613|1|0.0|1106|rno:293069|vomeronasal 2 receptor	gi|157384963|ref|NP_001098658.1|	0	vomeronasal receptor Vmn2r77 precursor [Mus musculus]
mmu-miR-1199-5p	NM_001105189	-34.4	0.054135	-30.78	164	-	K04613|1|0.0|1087|rno:293069|vomeronasal 2 receptor	gi|157384965|ref|NP_001098659.1|	0	vomeronasal 2, receptor 78 precursor [Mus musculus]
mmu-miR-1199-5p	NM_001105190	-34.4	0.053972	-30.78	164	-	K04613|1|0.0|1066|rno:293069|vomeronasal 2 receptor	gi|157384967|ref|NP_001098660.1|	0	vomeronasal receptor Vmn2r79 precursor [Mus musculus]
mmu-miR-1199-5p	NM_001109686	-27.7	0.828858	-46.64	302	GO:0043231,GO:0043232,GO:0046914,GO:0018024,GO:0005515,GO:0034968,GO:0006357,GO:0006305,GO:0018022,GO:00072	K11420|1|0.0|2303|mmu:77683|euchromatic histone-lysine N-methyltransferase [EC:2.1.1.43]	gi|157838004|ref|NP_001103156.1|	0	histone-lysine N-methyltransferase EHMT1 isoform 3 [Mus musculus]

mmu-miR-1199-5p	NM_001109687	-27.7	0.829727	-46.64	302	GO:0043231,GO:0043232,GO:0046914,GO:0018024,GO:0005515,GO:0034968,GO:0006357,GO:0006305,GO:0018022,GO:00072	K11420|1|0.0|2305|mmu:77683|euchromatic histone-lysine N-methyltransferase [EC:2.1.1.43]	gi|157838009|ref|NP_001103157.1|	0	histone-lysine N-methyltransferase EHMT1 isoform 4 [Mus musculus]
mmu-miR-1199-5p	NM_001109690	-25.8	0.990226	-30.25	154	GO:0031981,GO:0003676,GO:0046914,GO:0006357,GO:0007599,GO:0006325,GO:0007631,	K11643|1|4e-15|85.9|isc:IscW_ISCW005933|chromodomain-helicase-DNA-binding protein 4 [EC:3.6.4.12]	gi|157841176|ref|NP_001103160.1|	0	PHD finger protein 21A isoform 2 [Mus musculus]
mmu-miR-1199-5p	NM_001081117	-20.3	1	-84.82	589	GO:0005634,GO:0005515,	K09228|1|7e-27|125|pon:100434122|KRAB domain-containing zinc finger protein	gi|169234624|ref|NP_001074586.2|	0	antigen KI-67 [Mus musculus]

mmu-miR-122-3p	NM_027279	-29.5	0.061455	-26.15	165	GO:0008168,GO:0008213,	-	gi|31980620|ref|NP_081555.2|	0	histidine protein methyltransferase 1 homolog [Mus musculus]
mmu-miR-122-3p	NM_027760	-28.8	0.106553	-25.35	164	GO:0023052,	K09856|1|0.0|686|mmu:71323|Ras association domain-containing protein 8	gi|23503231|ref|NP_082036.1|	0	ras association domain-containing protein 8 [Mus musculus]
mmu-miR-122-3p	NM_001081971	-24.7	0.922875	-41.19	308	-	K15502|1|4e-12|75.5|bta:529062|serine/threonine-protein phosphatase 6 regulatory ankyrin repeat subunit A	gi|126517480|ref|NP_001075440.1|	4.62E-171	ankyrin repeat domain-containing protein 63 [Mus musculus]
mmu-miR-122-3p	NM_021362	-28.6	0.647774	-23.96	174	GO:0044424,GO:0044421,GO:0046914,GO:0004175,GO:0048869,GO:0022414,	K07762|1|0.0|3240|mmu:18491|pappalysin-1 [EC:3.4.24.79]	gi|20148857|gb|AAM12687.1|AF439513_1	0	pregnancy-associated plasma protein-A, partial [Mus musculus]

mmu-miR-122-3p	NM_027928	-28.2	0.132422	-23.18	173	GO:0012505,GO:0043231,GO:0031224,GO:0034481,GO:0006024,	K07779|1|0.0|644|mmu:71797|chondroitin 4-sulfotransferase 13 [EC:2.8.2.5]	gi|254692861|ref|NP_082204.1|	0	carbohydrate sulfotransferase 13 precursor [Mus musculus]
mmu-miR-122-3p	NM_011682	-25.4	0.987985	-21.37	170	GO:0031981,GO:0044459,GO:0043232,GO:0008092,GO:0046914,GO:0003012,GO:0048513,GO:0001952,	K10366|1|0.0|1570|gga:396236|dystrophin	gi|110431378|ref|NP_035812.3|	0	utrophin [Mus musculus]
mmu-miR-122-3p	NM_198599	-26.9	0.462992	-22.71	167	GO:0043232,GO:0016023,GO:0044431,GO:0015631,GO:0006500,	K10432|1|5e-09|64.7|aml:100477292|microtubule-associated protein 6	gi|74201189|dbj|BAE37442.1|	1.63E-108	unnamed protein product [Mus musculus]
mmu-miR-122-3p	NM_153581	-24.9	0.799962	-21.5	166	GO:0031224,GO:0005261,GO:0006816,	-	gi|23957686|ref|NP_705809.1|	7.90E-151	neuronal membrane glycoprotein M6-a isoform 1 [Mus
mmu-miR-122-3p	NR_045167	-23.5	0.527691	-20.06	165	-	-	-	-	-

mmu-miR-122-3p	NR_003544	-27	0.473562	-23.02	164	GO:0031224,GO:0005743,GO:0000166,GO:0008746,GO:0006818,GO:0009060,	K00323|1|0.0|958|mmu:18115|NAD(P) transhydrogenase [EC:1.6.1.2]	gi|26328961|dbj|BAC28219.1|	0	unnamed protein product [Mus musculus]
mmu-miR-122-3p	NM_153805	-26.6	0.468329	-23.65	163	GO:0043231,GO:0044444,GO:0004674,GO:0032559,GO:0006464,GO:0023052,	K06071|1|0.0|1569|mmu:263803|protein kinase N [EC:2.7.11.13]	gi|24418929|ref|NP_722500.1|	0	serine/threonine-protein kinase N3 [Mus musculus]

mmu-miR-122-3p	NM_172723	-25.1	0.642986	-21.53	162	GO:0043231,GO:0016020,GO:0005083,GO:0004871,GO:0046914,GO:0043178,GO:0035091,GO:0019932,GO:0033124,GO:0032012,	K12491|1|4e-23|110|api:100169536|Arf-GAP, GTPase, ANK repeat and PH domain-containing protein 1/2/3!K13051|2|3e-22|107|spu:582857|beta-aspartyl-peptidase (threonine type) [EC:3.4.19.5]!K12489|3|3e-21|104|tad:TRIADDRAFT_57442|Arf-	gi|148368976|ref|NP_766311.2|	0	centaurin, alpha 1 [Mus musculus]

mmu-miR-122-3p	NM_172485	-21.6	0.999997	-20.23	161	GO:0044464,	K08624|1|1e-40|170|mmu:101401|a disintegrin and metalloproteinase with thrombospondin motifs 9 [EC:3.4.24.-]	gi|40254219|ref|NP_766073.2|	0	thrombospondin type-1 domain-containing protein 7B precursor [Mus musculus]
mmu-miR-122-3p	NM_007925	-24.4	0.899141	-20.39	160	GO:0043231,GO:0005576,GO:0005488,GO:0005201,GO:0051017,GO:0008064,GO:0014706,GO:0030198,	K14211|1|6e-14|81.3|rno:25043|elastin	gi|345801223|ref|XP_859408.2|	2.22E-32	PREDICTED: uncharacterized protein LOC607775 [Canis lupus familiaris]
mmu-miR-122-3p	NM_027974	-24.2	0.762279	-20.6	159	GO:0043231,GO:0044297,GO:0042995,GO:0046872,GO:0005515,GO:0006915,GO:00068	-	gi|148682438|gb|EDL14385.1|	0	mCG7901 [Mus musculus]

mmu-miR-122-3p	NM_009656	-23.6	0.853247	-22.27	157	GO:0044429,GO:0004030,GO:0005515,GO:0044238,GO:0048545,GO:0070542,GO:0042133,GO:0006067,GO:0014070,GO:0048513,GO:0002237,GO:0006950,	K00128|1|0.0|957|mmu:11669|aldehyde dehydrogenase (NAD+) [EC:1.2.1.3]	gi|6753036|ref|NP_033786.1|	0	aldehyde dehydrogenase, mitochondrial precursor [Mus musculus]
mmu-miR-122-3p	NM_009916	-24.9	0.733859	-20.04	156	GO:0016021,GO:0044297,GO:0009986,GO:0004950,GO:0016477,GO:0006952,GO:0050921,GO:0002376,GO:0007166,GO:00514	K04179|1|0.0|676|mmu:12773|C-C chemokine receptor type 4	gi|162287123|ref|NP_034046.2|	0	C-C chemokine receptor type 4 [Mus musculus]
mmu-miR-122-3p	NR_027987	-24.9	0.706272	-20.67	154	-	-	gi|26338926|dbj|BAC33134.1|	1.77E-62	unnamed protein product [Mus musculus]

mmu-miR-122-3p	NM_175185	-24.8	0.797663	-20.24	151	GO:0043231,GO:0003824,GO:0005488,GO:0008152,	K00044|1|1e-63|245|xla:495218|estradiol 17beta-dehydrogenase [EC:1.1.1.62]!K00100|4|2e-56|222|spu:593779|[EC:1.1.1.-	gi|30424792|ref|NP_780394.1|	0	inactive hydroxysteroid dehydrogenase-like protein 1 [Mus musculus]
mmu-miR-122-3p	NM_028365	-20.2	1	-20.59	145	-	-	gi|241897001|ref|NP_082641.1|	0	zinc finger protein 839 isoform 1 [Mus musculus]
mmu-miR-122-3p	NM_178612	-21	0.995822	NA	NA	GO:0005515,GO:0008104,	K00552|1|1e-42|175|mgp:100540940|glycine N-methyltransferase [EC:2.1.1.20]	gi|148687259|gb|EDL19206.1|	1.45E-126	RIKEN cDNA 2610019P18 [Mus musculus]

mmu-miR-122-3p	NM_029981	-20.2	0.999999	NA	NA	GO:0031012,GO:0004175,GO:0046914,GO:0007179,	K08630|1|2e-54|215|spu:574964|a disintegrin and metalloproteinase with thrombospondin motifs 16 [EC:3.4.24.-]!K08632|5|7e-53|210|mdo:100014389|a disintegrin and metalloproteinase with thrombospondin motifs 18 [EC:3.4.24.	gi|148676410|gb|EDL08357.1|	0	ADAMTS-like 2, isoform CRA_b [Mus musculus]
mmu-miR-122-3p	NM_181548	-23.8	0.533214	NA	NA	GO:0016020,GO:0017111,GO:0032561,GO:0035556,GO:0009207,	K07827|1|7e-24|112|xtr:100485959|GTPase KRas	gi|31559801|ref|NP_853526.1|	1.87E-106	GTPase ERas precursor [Mus musculus]

mmu-miR-122-3p	NM_007742	-22.5	0.998776	NA	NA	-	K06236|1|1e-151|539|mmu:12842|collagen, type I/II/III/V/XI, alpha	gi|148684009|gb|EDL15956.1|	6.73E-151	procollagen, type I, alpha 1, isoform CRA_a [Mus musculus]
mmu-miR-122-3p	NM_011419	-22	0.999929	NA	NA	GO:0043231,GO:0046914,GO:0003676,GO:0016701,GO:0006325,GO:0008152,GO:00482	K11446|1|0.0|2942|mmu:20592|histone demethylase JARID1 [EC:1.14.1	gi|33859626|ref|NP_035549.1|	0	lysine-specific demethylase 5D [Mus musculus]
mmu-miR-122-3p	NM_001083807	-20.5	0.999997	NA	NA	GO:0031981,GO:0030674,	K15691|1|2e-11|72.4|mgp:100545594|E3 ubiquitin-protein ligase RFWD3 [EC:6.3.2.19]	gi|139947575|ref|NP_001077276.1|	0	RUN and SH3 domain-containing protein 1 isoform 2 [Mus musculus]
mmu-miR-122-3p	NM_025473	-21.9	0.969246	NA	NA	-	K09666|1|2e-19|97.8|bmy:Bm1_54625|beta-1,2-N-acetylglucosaminyltransferase [EC:2.4.1.-]	gi|148697866|gb|EDL29813.1|	7.51E-160	RIKEN cDNA 1810037C20, isoform CRA_a [Mus musculus]

mmu-miR-122-3p	NM_013486	-22.4	0.814967	NA	NA	GO:0009986,GO:0044421,GO:0044459,GO:0031225,GO:0032991,GO:0042802,GO:0043498,GO:0005102,GO:0019900,GO:0046649,GO:0007155,	K06449|1|7e-160|563|mmu:12481|CD2 antigen	gi|7304949|ref|NP_038514.1|	4.64E-159	T-cell surface antigen CD2 precursor [Mus musculus]
mmu-miR-122-3p	NR_040445	-20.9	0.999578	NA	NA	-	-	gi|148703185|gb|EDL35132.1|	8.22E-46	mCG6463 [Mus musculus]
mmu-miR-122-3p	NM_172775	-20.2	1	NA	NA	GO:0043235,GO:0016021,GO:0008047,GO:0005102,GO:0004888,GO:0065008,GO:0048870,GO:0031346,GO:0006935,GO:0033043,GO:0032501,GO:0009966,GO:00071	K06821|1|0.0|4042|mmu:235611|plexin B	gi|148689398|gb|EDL21345.1|	0	plexin B1, isoform CRA_b [Mus musculus]

mmu-miR-122-3p	NM_001029856	-20.2	1	NA	NA	GO:0043231,GO:0016462,GO:0032559,	K10754|1|1e-14|84.7|cel:C54G10.2|replication factor C subunit	gi|71143102|ref|NP_001025027.1|		0	ATPase family AAA domain-containing protein 5 [Mus musculus]
mmu-miR-122-3p	NM_175331	-20.7	1	NA	NA	GO:0044444,GO:0043231,GO:0003824,GO:0043169,	K11757|1|0.0|655|ssc:100525781|protein polybromo-1!K01081|3|7e-49|197|phu:Phum_PHUM227090|5'-nucleotidase [EC:3.1.3.5]	gi|111494223|ref|NP_780540.2|		0	5'-nucleotidase domain-containing protein 3 [Mus musculus]
mmu-miR-122-3p	NM_031881	-20.1	1	NA	NA	GO:0044424,GO:0005488,GO:0008200,GO:0019787,GO:0010035,GO:0051704,GO:0003008,GO:0002028,GO:0009894,GO:0006875,GO:0016567,GO:0030100,	K13305|1|0.0|1738|mmu:83814|E3 ubiquitin-protein ligase NEDD4-like [EC:6.3.2.19]	gi|167466245|ref|NP_001107858.1|		0	E3 ubiquitin-protein ligase NEDD4-like isoform 1 [Mus musculus]

mmu-miR-122-3p	NM_023543	-20.7	0.999902	NA	NA	GO:0005100,GO:0043169,GO:0032321,	K04450|1|1e-127|457|ptr:739083|activating transcription factor 2!K02649|2|5e-37|157|bfo:BRAFLDRAFT_91712|phosphoinositide-3-kinase, regulatory subunit!K10360|3|2e-33|145|tca:656579|myosin IX!K13736|4|5e-32|140|dre:692277|Rho	gi|254692938|ref|NP_076032.2|	0	beta-chimaerin isoform 1 [Mus musculus]

mmu-miR-122-3p	NM_010432	-20.7	1	NA	NA	GO:0005815,GO:0016604,GO:0003712,GO:0003676,GO:0004672,GO:0032559,GO:0003002,GO:0006796,GO:0051099,GO:0006351,GO:0008630,GO:0008283,GO:0007166,GO:00	K08826|1|0.0|2093|mmu:15257|homeodomain interacting protein kinase [EC:2.7.11.1]	gi|162770931|emb|CAP58492.1|	0	homeodomain interacting protein kinase 1 [Mus musculus]
mmu-miR-122-3p	NM_175481	-20.2	1	NA	NA	GO:0043231,GO:0033267,GO:0043025,GO:0008328,GO:0004970,GO:0005231,GO:0006810,GO:0048545,GO:0007267,GO:00071	K05204|1|0.0|1868|mmu:110637|glutamate receptor, ionotropic kainate 4	gi|304766513|ref|NP_780690.2|	0	glutamate receptor, ionotropic kainate 4 precursor [Mus musculus]
mmu-miR-122-3p	NM_146107	-21.4	0.994691	NA	NA	GO:0005875,GO:0032559,	K05692|1|2e-120|433|bmy:Bm1_21705|actin beta/gam	gi|22122615|ref|NP_666219.1|	0	beta-centractin [Mus musculus]

mmu-miR-122-3p	NM_008780	-21.1	0.999498	NA	NA	GO:0043231,GO:0043234,GO:0003676,GO:0001071,GO:0006357,GO:0048732,GO:0046632,GO:0009653,GO:0009952,GO:00357	K09382|1|0.0|763|mmu:18503|paired box protein 1/9	gi|197276660|ref|NP_032806.2|	0	paired box protein Pax-1 [Mus musculus]
mmu-miR-122-3p	NM_001162919	-21.2	0.99986	NA	NA	GO:0044464,	-	gi|148700707|gb|EDL32654.1|	0	RIKEN cDNA 4921509E05, isoform CRA_a [Mus musculus]
mmu-miR-122-3p	NM_025658	-25.8	0.335313	NA	NA	GO:0031224,GO:0004871,GO:0023052,	K06466|1|4e-09|63.5|oaa:100073769|membrane-spanning 4-domains, subfamily A, member 1	gi|148709484|gb|EDL41430.1|	3.10E-124	mCG12900 [Mus musculus]
mmu-miR-122-3p	NM_021893	-21.6	0.999687	NA	NA	GO:0031224,GO:0009986,GO:0005488,GO:0004871,GO:0042098,GO:0007165,GO:00312	K06745|1|1e-166|588|mmu:60533|programmed cell death 1 ligand 1	gi|11230798|ref|NP_068693.1|	7.35E-166	programmed cell death 1 ligand 1 precursor [Mus musculus]

mmu-miR-122-3p	NM_001164606	-22.5	0.996629	NA	NA	-	-	gi|257196215|ref|NP_084055.2|	0	coiled-coil domain-containing protein 116 [Mus musculus]
mmu-miR-122-3p	NM_027444	-22.1	0.999998	NA	NA	GO:0043231,GO:0003676,GO:0010467,	K09270|1|2e-09|67.4|bfo:BRAFLDRAFT_125030|transcription factor SOX7/8/9/10/18 (SOX group E/F)!K09267|3|5e-09|66.2|cbr:CBG16696|transcription factor SOX1/2/3/14/21 (SOX group B)!K04495|5|2e-08|63.9|acs:100558619|transcription	gi|148665707|gb|EDK98123.1|	0	bobby sox homolog (Drosophila), isoform CRA_b [Mus musculus]

mmu-miR-122-3p	NM_177378	-21.9	1	NA	NA	GO:0031224,GO:0046914,	K15701|1|9e-94|347|mdo:100032093|E3 ubiquitin-protein ligase RNF130 [EC:6.3.2.19]	gi|90991708|ref|NP_796352.2|	0	RING finger protein 150 precursor [Mus musculus]
mmu-miR-122-3p	NM_001111320	-21.1	0.998718	NA	NA	GO:0043231,GO:0044444,GO:0046872,GO:0042802,GO:0004448,GO:0000166,GO:0006629,GO:0009725,GO:0043648,GO:0006790,GO:0044262,GO:0009060,GO:0008406,GO:00	K00031|1|0.0|798|mmu:15926|isocitrate dehydrogenase [EC:1.1.1.42]	gi|26352311|dbj|BAC39792.1|	0	unnamed protein product [Mus musculus]

mmu-miR-122-3p	NM_029081	-21.6	0.999917	NA	NA	-	K06130|1|2e-08|63.2|spu:584566|lysophospholipase II [EC:3.1.1.5]!K15290|2|2e-06|56.6|tgu:751605|synaptotagmin-1	gi|148678709|gb|EDL10656.1|	0	RIKEN cDNA 5730419I09, isoform CRA_b [Mus musculus]
mmu-miR-122-3p	NR_038097	-20.5	0.999981	NA	NA	GO:0031234,GO:0048770,GO:0005903,GO:0032561,GO:0017111,GO:0060089,GO:0031701,GO:0032403,GO:0050780,GO:0001525,GO:0007265,GO:0007599,GO:0043009,GO:0009207,GO:0051480,GO:0048869,GO:0007188,GO:0001775,GO:0010518,GO:00	K04639|1|7e-20|100|ssc:100522824|guanine nucleotide-binding protein subunit alpha-13	gi|122889959|emb|CAM14527.1|	3.41E-54	novel protein [Mus musculus]

mmu-miR-122-3p	NM_019488	-27.5	0.237449	NA	NA	GO:0016021,GO:0030136,GO:0012506,GO:0048029,GO:0005353,GO:0005355,GO:0019318,GO:0008645,GO:0007169,GO:0007127,GO:0006950,	K08145|1|0.0|885|mmu:56017|MFS transporter, SP family, solute carrier family 2 (facilitated glucose transporter), member 8	gi|9506735|ref|NP_062361.1|	0	solute carrier family 2, facilitated glucose transporter member 8 [Mus musculus]
mmu-miR-122-3p	NM_172661	-24.3	0.994077	NA	NA	-	-	gi|148676580|gb|EDL08527.1|	0	RIKEN cDNA 5830434P21, isoform CRA_b [Mus musculus]
mmu-miR-122-3p	NM_001170401	-21.2	0.999945	NA	NA	GO:0031224,	-	gi|30520191|ref|NP_848860.1|	5.62E-97	transmembrane protein 229B [Mus musculus]
mmu-miR-122-3p	NR_040444	-20.9	0.997904	NA	NA	-	-	gi|148698905|gb|EDL30852.1|	2.11E-38	mCG146032 [Mus musculus]
mmu-miR-122-3p	NM_024291	-24	0.979943	NA	NA	GO:0031674,GO:0016787,	K14786|1|1e-24|117|smm:Smp_133510|protein KRI1	gi|148689102|gb|EDL21049.1|	0	kyphoscoliosis peptidase, isoform CRA_a [Mus musculus]

mmu-miR-122-3p	NM_178608	-21	0.999984	NA	NA	GO:0005740,GO:0031224,GO:0001664,GO:0034613,	K15601|1|2e-65|252|cfa:474695|lysine-specific demethylase 3 [EC:1.14.11.-]!K00939|2|2e-06|56.6|bmy:Bm1_13790|adenylate kinase [EC:2.7.4.3]	gi|30519931|ref|NP_848723.1|	5.27E-82	receptor expression-enhancing protein 1 [Mus musculus]
mmu-miR-122-3p	NM_027533	-21.5	0.999937	NA	NA	GO:0031224,GO:0005198,GO:0030154,	K06460|1|1e-44|183|xla:443887|CD9 antigen	gi|70608157|ref|NP_081809.2|	2.48E-104	tetraspanin-2 isoform 1 [Mus musculus]

mmu-miR-122-3p	NM_010927	-21.5	0.999892	NA	NA	GO:0042579,GO:0005856,GO:0000166,GO:0016597,GO:0050662,GO:0042802,GO:0005506,GO:0016709,GO:0019887,GO:0032496,GO:0009611,GO:0048771,GO:0033280,GO:0050673,GO:0008015,GO:0044057,GO:0016310,GO:0043627,GO:0042742,GO:0022602,GO:0044271,GO:00170	K13241|1|0.0|2334|mmu:18126|nitric-oxide synthase, inducible [EC:1.14.13.39]	gi|6754872|ref|NP_035057.1|		0	nitric oxide synthase, inducible [Mus musculus]
mmu-miR-122-3p	NM_001029872	-20.8	0.999996	NA	NA	GO:0005887,GO:0004871,GO:0046983,GO:0031589,GO:0007166,GO:0042098,	K06594|1|0.0|2360|mmu:381924|integrin alpha D	gi|294997269|ref|NP_001025043.3|		0	integrin alpha-D precursor [Mus musculus]

mmu-miR-122-3p	NM_146103	-21.8	0.997029	NA	NA	GO:0031224,	K00547|1|1e-25|119|smm:Smp_147610|homocysteine S-methyltransferase [EC:2.1.1.10]	gi|22122617|ref|NP_666215.1|	0	transmembrane protein 185B [Mus musculus]
mmu-miR-122-3p	NM_007943	-20.6	1	NA	NA	GO:0005768,GO:0030128,GO:0004871,GO:0046872,GO:0019904,GO:0006901,GO:0016197,GO:0007173,	K12472|1|0.0|1265|ecb:100062532|epidermal growth factor receptor substrate 15	gi|6679671|ref|NP_031969.1|	0	epidermal growth factor receptor substrate 15 isoform A [Mus musculus]
mmu-miR-122-3p	NM_013675	-20	1	NA	NA	GO:0032991,GO:0030864,GO:0009898,GO:0005198,GO:0003779,GO:0030835,GO:0006778,GO:0006935,	K06115|1|0.0|4436|mmu:20741|spectrin beta	gi|84490394|ref|NP_038703.3|	0	spectrin beta chain, erythrocyte [Mus musculus]

mmu-miR-122-3p	NM_145394	-21.4	0.998451	NA	NA	GO:0031224,GO:0005275,GO:0015697,	K15282|1|0.0|1304|mmu:213603|solute carrier family 44 (choline transporter-like protein), member 3	gi|225579159|ref|NP_663369.2|	0	choline transporter-like protein 3 [Mus musculus]
mmu-miR-122-3p	NM_001271768	-21.9	0.999983	NA	NA	-	-	-	-	-
mmu-miR-122-3p	NM_008196	-20.6	0.942037	NA	NA	GO:0004175,GO:0019538,	K08663|1|8e-155|546|mmu:14945|granzyme K [EC:3.4.21.-]	gi|6680143|ref|NP_032222.1|	5.53E-154	granzyme K precursor [Mus musculus]
mmu-miR-122-3p	NM_001005525	-22.7	0.945723	NA	NA	-	-	gi|354472542|ref|XP_003498497.1|	2.06E-77	PREDICTED: arginine/serine-rich coiled-coil protein 2-like [Cricetulus griseus]

mmu-miR-122-3p	NM_001112723	-20.8	0.999999	NA	NA	GO:0005856,GO:0005088,GO:0016811,GO:0005515,GO:0019222,GO:0007266,	K05156|1|2e-23|112|gga:419067|tumor necrosis factor receptor superfamily member 19-like!K04436|2|2e-22|109|xtr:100170186|mitogen-activated protein kinase 8 interacting protein	gi|37360480|dbj|BAC98218.1|	0	mKIAA1626 protein [Mus musculus]
mmu-miR-122-3p	NM_026763	-21.6	1	NA	NA	-	K06238|1|0.0|4267|mmu:68553|collagen, type VI, alpha	gi|242247116|ref|NP_081039.2|	0	collagen alpha-4(VI) chain precursor [Mus musculus]
mmu-miR-122-3p	NM_030074	-21.8	0.999885	NA	NA	GO:0031981,GO:0046914,GO:0003676,GO:0006351,	K09228|1|4e-16|88.6|cfa:482782|KRAB domain-containing zinc finger protein	gi|27229259|ref|NP_084350.1|	0	zinc finger protein 687 [Mus musculus]

mmu-miR-122-3p	NM_001163631	-20.7	1	NA	NA	GO:0034399,GO:0016604,GO:0000790,GO:0001071,GO:0043566,GO:0009605,GO:0006357,GO:0035710,GO:0006915,GO:0051704,GO:0046632,GO:0006479,GO:0006325,GO:00	-	gi|254826759|ref|NP_001157102.1|	0	DNA-binding protein SATB1 [Mus musculus]
mmu-miR-122-3p	NM_178922	-24.1	0.986454	NA	NA	GO:0031981,GO:0005924,GO:0003676,GO:0046914,GO:0005515,GO:0006355,	K09228|1|2e-33|146|mmu:54678|KRAB domain-containing zinc finger protein	gi|37360196|dbj|BAC98076.1|	0	mKIAA1020 protein [Mus musculus]
mmu-miR-122-3p	NM_001172097	-22.2	0.999862	NA	NA	-	K03869|1|5e-07|58.9|hmg:100202538|cullin 3	gi|285402250|ref|NP_001165568.1|	2.36E-118	uncharacterized protein C10orf46 homolog isoform 3 [Mus musculus]
mmu-miR-122-3p	NR_024202	-21.5	0.075533	NA	NA	-	-	-	-	-

mmu-miR-122-3p	NM_001111049	-21.6	0.983393	NA	NA	GO:0044444,GO:0016021,GO:0005488,GO:0007044,	K06537|1|2e-123|443|mmu:12476|CD151 antigen	gi|161353452|ref|NP_033972.2|	1.28E-122	CD151 antigen [Mus musculus]
mmu-miR-122-3p	NM_008017	-22.8	0.998564	NA	NA	GO:0000793,GO:0043231,GO:0046983,GO:0032559,GO:0000070,GO:0044419,	K06674|1|0.0|2066|mmu:14211|structural maintenance of chromosome 2	gi|62990166|ref|NP_032043.3|	0	structural maintenance of chromosomes protein 2 [Mus musculus]
mmu-miR-122-3p	NM_001039555	-21.7	0.979182	NA	NA	GO:0031090,GO:0043231,GO:0005506,GO:0016712,GO:0008152,	K07413|1|0.0|961|mmu:13099|cytochrome P450, family 2, subfamily C [EC:1.14.14.1]	gi|254281186|ref|NP_034134.2|	0	cytochrome P450 2C40 [Mus musculus]

mmu-miR-122-3p	NM_177798	-24	0.982936	NA	NA	GO:0043231,GO:0016021,GO:0008047,GO:0030674,GO:0070851,GO:0048699,GO:0042127,GO:0007369,GO:0003156,GO:0010863,GO:0007169,GO:0060442,GO:0007420,GO:0000578,GO:0001743,GO:0003231,GO:00	K12461|1|0.0|970|mmu:327826|fibroblast growth factor receptor substrate 2	gi|29244340|ref|NP_808466.1|	0	fibroblast growth factor receptor substrate 2 [Mus musculus]
mmu-miR-122-3p	NR_037995	-23	0.62442	NA	NA	-	-	gi|148670730|gb|EDL02677.1|	3.29E-77	mCG1027832 [Mus musculus]
mmu-miR-122-3p	NM_029035	-20.3	0.999997	NA	NA	GO:0044424,GO:0004871,GO:0007165,	K10343|1|1e-161|568|cfa:479598|SPRY domain-containing SOCS box protein 1/4	gi|62088698|dbj|BAD92796.1|	1.52E-165	SPRY domain-containing SOCS box protein SSB-1 variant [Homo sapiens]

mmu-miR-122-3p	NM_054053	-21.5	1	NA	NA	GO:0031224,GO:0002141,GO:0044424,GO:0008092,GO:0046872,GO:0004888,GO:0045596,GO:0048731,GO:0050954,GO:0030030,GO:0007186,GO:0050953,GO:0007155,GO:0043954,	K08455|1|5e-17|94.0|mcc:693375|G protein-coupled receptor 112!K05849|3|8e-17|93.2|api:100167616|solute carrier family 8 (sodium/calcium exchanger)	gi|153791474|ref|NP_473394.3|	0	G-protein coupled receptor 98 precursor [Mus musculus]
mmu-miR-122-3p	NM_001013749	-23.3	0.991806	NA	NA	GO:0031224,	-	gi|85701444|ref|NP_001013771.1|	0	transmembrane protein 151B [Mus musculus]
mmu-miR-122-3p	NM_013924	-23.1	0.992274	NA	NA	GO:0043234,GO:0031981,GO:0003676,GO:0021515,GO:0006351,	K14785|1|4e-121|437|mmu:30946|ESF2/ABP1 family	gi|2065210|emb|CAA73251.1|	2.78E-160	Pro-Pol-dUTPase polyprotein [Mus musculus]
mmu-miR-122-3p	NM_001162375	-21.5	0.999989	NA	NA	GO:0044464,	-	gi|240849401|ref|NP_001155847.1|	0	protein FAM73A isoform 2 [Mus musculus]

mmu-miR-122-3p	NR_046286	-22	0.995542	NA	NA	GO:0044425,	K06751|1|0.0|460|mmu:15024|major histocompatibility complex, class I	gi|199664|gb|AAA39695.1|	0	MHC H2-TL-T10-129 [Mus musculus]
mmu-miR-122-3p	NM_011028	-20.3	0.999957	NA	NA	GO:0016021,GO:0030425,GO:0044297,GO:0044459,GO:0044430,GO:0016502,GO:0005231,GO:0005515,GO:0032559,GO:0035587,GO:0051259,GO:0003012,GO:00068	K05221|1|0.0|744|rno:25041|purinergic receptor P2X-like 1, orphan receptor	gi|227116251|ref|NP_035158.2|	0	P2X purinoceptor 6 isoform 1 [Mus musculus]
mmu-miR-122-3p	NM_026437	-21.3	0.998037	NA	NA	-	-	gi|268370235|ref|NP_080713.2|	0	protein FAM45A isoform 1 [Mus musculus]

mmu-miR-122-3p	NM_015787	-24.4	0.674695	NA	NA	GO:0043231,GO:0005694,GO:0003676,GO:0034728,	K11275|1|1e-31|138|mmu:50709|histone H1/5!K11252|5|5e-31|136|mcc:696872|histone H2B	gi|13430890|ref|NP_056602.1|	9.63E-31	histone H1.4 [Mus musculus]
mmu-miR-122-3p	NM_001252208	-22.5	0.995408	NA	NA	GO:0005887,GO:0005488,GO:0005249,GO:0034220,GO:0007267,GO:0030001,	K05009|1|0.0|827|mmu:16517|potassium inwardly-rectifying channel subfamily J member	gi|130505929|ref|NP_034734.3|	0	inward rectifier potassium channel 16 [Mus musculus]
mmu-miR-122-3p	NM_011099	-22.5	0.972708	NA	NA	GO:0043231,GO:0042995,GO:0044444,GO:0016020,GO:0031420,GO:0016301,GO:0032559,GO:0006007,GO:0008219,GO:0006090,GO:0006796,GO:0009206,GO:0009887,GO:00	K00873|1|0.0|1048|xla:100037030|pyruvate kinase [EC:2.7.1.40]	gi|31981562|ref|NP_035229.2|	0	pyruvate kinase isozymes M1/M2 isoform 1 [Mus musculus]

mmu-miR-122-3p	NM_001081057	-22.5	0.999967	NA	NA	GO:0005488,	-	gi|37359890|dbj|BAC97923.1|	0	mKIAA0297 protein [Mus musculus]
mmu-miR-122-3p	NM_008844	-20.4	1	NA	NA	GO:0044444,GO:0005886,GO:0016307,GO:0000166,GO:0046488,GO:0048667,GO:0006796,GO:0006935,	K00889|1|0.0|949|rno:314641|1-phosphatidylinositol-4-phosphate 5-kinase [EC:2.7.1.68]	gi|226442755|ref|NP_032870.2|	0	phosphatidylinositol 4-phosphate 5-kinase type-1 gamma isoform 1 [Mus musculus]

mmu-miR-122-3p	NM_009993	-23	0.886974	NA	NA	GO:0043231,GO:0031090,GO:0005515,GO:0033695,GO:0005506,GO:0016709,GO:0016712,GO:0018894,GO:0015980,GO:0008202,GO:0010467,GO:0042743,GO:0043627,GO:0009698,GO:0017144,GO:0006730,GO:0033013,GO:0006721,GO:0006805,GO:0030323,GO:0007090,GO:00424	K07409|1|0.0|1046|mmu:13077|cytochrome P450, family 1, subfamily A, polypeptide 2 [EC:1.14.14.1]	gi|6753556|ref|NP_034123.1|	0	cytochrome P450 1A2 [Mus musculus]
mmu-miR-122-3p	NM_001126338	-23.7	0.799153	NA	NA	GO:0031224,GO:0046914,GO:0030003,GO:0051259,	-	gi|148696387|gb|EDL28334.1|	4.30E-103	mCG15178, isoform CRA_a [Mus musculus]

mmu-miR-122-3p	NM_020259	-20.1	1	NA	NA	GO:0016021,GO:0044424,GO:0046914,GO:0030323,GO:0003002,GO:0008589,GO:0008543,GO:0009653,GO:0048699,	K06231|1|0.0|1437|mmu:15245|hedgehog interacting protein	gi|160358774|ref|NP_064655.4|	0	hedgehog-interacting protein precursor [Mus musculus]
mmu-miR-122-3p	NM_145557	-28.3	0.207767	NA	NA	GO:0016020,	-	gi|225543202|ref|NP_663532.2|	2.13E-97	uncharacterized protein LOC230996 precursor [Mus musculus]
mmu-miR-122-3p	NM_010587	-20.7	1	NA	NA	GO:0044459,GO:0031252,GO:0016023,GO:0012505,GO:0008047,GO:0005088,GO:0046872,GO:0005515,GO:0006917,GO:0019222,GO:0007169,GO:0010740,GO:0006897,GO:0043066,GO:0007266,	K13738|1|8e-30|135|bfo:BRAFLDRAFT_90471|CD2-associated protein!K07976|4|2e-29|134|ame:409364|Rab family, other	gi|134288904|ref|NP_034717.2|	0	intersectin-1 isoform 1 [Mus musculus]

mmu-miR-122-3p	NM_001163630	-20.7	1	NA	NA	GO:0034399,GO:0016604,GO:0000790,GO:0001071,GO:0043566,GO:0009605,GO:0006357,GO:0035710,GO:0006915,GO:0051704,GO:0046632,GO:0006479,GO:0006325,GO:00	-	gi|254826759|ref|NP_001157102.1|	0	DNA-binding protein SATB1 [Mus musculus]
mmu-miR-122-3p	NM_144518	-25	0.649797	NA	NA	-	-	gi|148664969|gb|EDK97385.1|	2.03E-110	RIKEN cDNA 2900011O08, isoform CRA_a [Mus musculus]
mmu-miR-122-3p	NM_027179	-21.2	0.956409	NA	NA	GO:0031224,GO:0043231,GO:0031090,GO:0003995,GO:0016628,GO:0006631,GO:0035336,GO:0006641,GO:0008202,	K10258|1|2e-175|615|mmu:106529|enoyl reductase [EC:1.3.1.-]	gi|148678962|gb|EDL10909.1|	0	mCG11048, isoform CRA_b [Mus musculus]

mmu-miR-122-3p	NM_177747	-20.5	1	NA	NA	GO:0043231,GO:0046914,GO:0003677,GO:0006355,	K09228|1|2e-64|249|bfo:BRAFLDRAFT_71028|KRAB domain-containing zinc finger protein	gi|125347348|ref|NP_808415.2|	0	zinc finger protein 711 [Mus musculus]
mmu-miR-122-3p	NM_027400	-20.7	0.999994	NA	NA	GO:0031224,GO:0030016,GO:0005793,GO:0030134,GO:0031090,GO:0012505,GO:0048029,GO:0046872,GO:0005515,GO:0007599,GO:0048193,GO:0006487,	K10080|1|0.0|934|mmu:70361|lectin, mannose-binding 1	gi|21312570|ref|NP_081676.1|	0	protein ERGIC-53 precursor [Mus musculus]
mmu-miR-122-3p	NM_010870	-21	1	NA	NA	GO:0043169,GO:0006915,GO:0006950,	K12807|1|0.0|2749|mmu:17951|baculoviral IAP repeat-containing protein 1	gi|124107602|ref|NP_035000.2|	0	baculoviral IAP repeat-containing protein 1e [Mus musculus]

mmu-miR-122-3p	NM_010483	-20.2	0.999893	NA	NA	GO:0031224,GO:0043176,GO:0008227,GO:0007186,	K04161|1|5e-171|602|mmu:15564|5-hydroxytryptamine receptor 5	gi|162135957|ref|NP_034613.2|	3.26E-170	5-hydroxytryptamine receptor 5B [Mus musculus]
mmu-miR-1224-3p	NM_001001184	-32.7	0.151509	-31.85	158	GO:0003899,GO:0010467,GO:0006259,	K11513|1|8e-13|77.4|mmu:71876|centromere protein U	gi|47564100|ref|NP_001001184.1|	0	coiled-coil domain-containing protein 111 [Mus musculus]

mmu-miR-1224-3p	NM_001001602	-34.1	0.157647	-32.6	169	GO:0009898,GO:0044424,GO:0008047,GO:0060090,GO:0005083,GO:0042802,GO:0019901,GO:0019903,GO:0032813,GO:0006915,GO:0035411,GO:0016049,GO:0032318,GO:0034260,GO:0043433,GO:0001837,GO:0006469,GO:0070849,GO:0030178,GO:0007173,GO:0000122,GO:00082	K04352|1|4e-46|188|cin:100184835|Ras GTPase-activating protein 1	gi|166295210|ref|NP_001001602.2|	0	disabled homolog 2-interacting protein isoform 3 [Mus musculus]

mmu-miR-1224-3p	NM_001001881	-28.6	0.696029	-25.37	160	-	K07514|1|7e-100|367|ssc:448846|enoyl-CoA hydratase / 3-hydroxyacyl-CoA dehydrogenase / 3,2-trans-enoyl-CoA isomerase [EC:4.2.1.17 1.1.1.35 5.3.3.8]	gi|49457853|ref|NP_001001881.1|	1.27E-134	UPF0524 protein C3orf70 homolog [Mus musculus]
mmu-miR-1224-3p	NM_001002011	-28.8	0.483484	-25.7	158	GO:0034399,GO:0044444,GO:0012505,GO:0005882,GO:0005488,GO:0006915,GO:0006606,GO:0007569,GO:0055013,GO:00069	K12641|1|0.0|876|pon:100432939|lamin A/C	gi|224901|prf||1203377A	0	lamin A
mmu-miR-1224-3p	NM_001002775	-30	0.367937	-25.67	169	-	-	gi|377834441|ref|XP_003689482.1|	0	PREDICTED: uncharacterized protein LOC442809 [Mus musculus]

mmu-miR-1224-3p	NM_001003955	-30.6	0.475061	-25.15	153	GO:0010008,GO:0031966,GO:0015631,GO:0006810,	K12484|1|0.0|1948|mmu:52055|Rab11 family-interacting protein 1/2/5	gi|37360124|dbj|BAC98040.1|	0	mKIAA0857 protein [Mus musculus]
mmu-miR-1224-3p	NM_001004150	-29.2	0.298055	-50.89	309	GO:0031228,GO:0035250,GO:0006687,GO:0016044,	K01988|1|0.0|690|mmu:239559|lactosylceramide 4-alpha-galactosyltransferase [EC:2.4.1.228]	gi|51921295|ref|NP_001004150.1|	0	lactosylceramide 4-alpha-galactosyltransferase [Mus musculus]
mmu-miR-1224-3p	NM_001004176	-30.2	0.549652	-29.15	166	GO:0005654,GO:0003712,GO:0006357,GO:0007166,	K06061|1|0.0|1785|mmu:433586|mastermind	gi|205277330|ref|NP_001004176.2|	0	mastermind-like 3 [Mus musculus]
mmu-miR-1224-3p	NM_001004365	-34.7	0.02474	-30.6	168	GO:0043232,GO:0008092,GO:0032559,GO:0008064,	K05692|1|9e-67|255|aml:100466132|actin beta/gamma 1	gi|52345394|ref|NP_001004365.1|	0	actin-related protein 3B [Mus musculus]

mmu-miR-1224-3p	NM_001004436	-34.8	0.083822	-55.66	314	GO:0044427,GO:0000794,GO:0005488,GO:0051704,GO:0006260,GO:0035561,GO:0000087,GO:0007063,GO:0034502,GO:00422	-	gi|37359874|dbj|BAC97915.1|	0	mKIAA0261 protein [Mus musculus]
mmu-miR-1224-3p	NM_001005424	-27.6	0.802422	-29.99	174	-	-	gi|148762971|ref|NP_001005424.2|	0	uncharacterized protein C9orf172 homolog [Mus musculus]
mmu-miR-1224-3p	NM_001007572	-28	0.527861	-25.12	154	GO:0016021,GO:0044459,GO:0005488,GO:0004871,GO:0005261,GO:0051259,GO:0023052,GO:0006875,GO:0006816,	K04974|1|0.0|1360|mmu:194352|transient receptor potential cation channel subfamily V member 5	gi|56090239|ref|NP_001007573.1|	0	transient receptor potential cation channel subfamily V member 5 [Mus musculus]
mmu-miR-1224-3p	NM_001008230	-26.4	0.747238	-32.31	167	GO:0031224,GO:0001664,GO:0034613,	-	gi|148665259|gb|EDK97675.1|	8.51E-136	receptor transporter protein 2, isoform CRA_b [Mus musculus]

mmu-miR-1224-3p	NM_001008419	-29.6	0.522183	-30.13	159	GO:0046914,GO:0003824,GO:0051536,GO:0050662,GO:0000166,GO:0008152,	K00157|1|0.0|2536|mmu:213043|aldehyde oxidase [EC:1.2.3.1]	gi|56606023|ref|NP_001008419.1|	0	aldehyde oxidase 3-like 1 [Mus musculus]
mmu-miR-1224-3p	NM_001008420	-30.5	0.459747	-27.95	163	GO:0031224,GO:0046872,GO:0045216,GO:0016337,	K06804|1|0.0|1503|mmu:215654|cadherin 12, type 2, N-cadherin	gi|56606025|ref|NP_001008420.1|	0	cadherin-12 precursor [Mus musculus]
mmu-miR-1224-3p	NM_001008498	-38.2	0.005759	-34.27	170	GO:0044424,	K15437|1|9e-41|169|hmg:100206473|aminoacyl tRNA synthase complex-interacting multifunctional protein 1!K10415|2|2e-24|115|dre:767729|dynein intermediate chain, cytosolic	gi|139948827|ref|NP_001008498.2|	0	WD repeat-containing protein 34 [Mus musculus]

mmu-miR-1224-3p	NM_001009573	-25.6	0.95492	-49.44	305	GO:0000323,GO:0030133,GO:0005768,GO:0005488,GO:0002886,GO:0002323,GO:0017157,GO:0006897,GO:0002460,	K15621|1|0.0|1792|ecb:100059416|BAI1-associated protein 3	gi|62243588|ref|NP_001009573.2|	0	protein unc-13 homolog D [Mus musculus]
mmu-miR-1224-3p	NM_001010834	-30.9	0.297998	-28.24	157	GO:0031224,GO:0005343,GO:0015718,GO:0035725,	K14343|1|0.0|760|mmu:241877|solute carrier family 10 (sodium/bile acid cotransporter), member 3/5	gi|58082065|ref|NP_001010834.1|	0	sodium/bile acid cotransporter 5 precursor [Mus musculus]
mmu-miR-1224-3p	NM_001010836	-27.7	0.64511	-28.59	154	GO:0043231,GO:0003712,GO:0005515,GO:0012501,GO:0006941,GO:0043010,GO:0042303,GO:0040007,GO:0000122,GO:0003208,GO:0048738,	K06270|1|1e-12|76.6|dya:Dyak_GE22289|protein phosphatase 1 regulatory subunit 12A	gi|58082069|ref|NP_001010836.1|	0	relA-associated inhibitor [Mus musculus]

mmu-miR-1224-3p	NM_001011874	-28.4	0.597658	-25.95	151	GO:0031224,	-	gi|59858539|ref|NP_001011874.1|	0	XK-related protein 4 [Mus musculus]
mmu-miR-1224-3p	NM_001012335	-34.7	0.008628	-30.65	158	GO:0005539,GO:0005102,GO:0048522,GO:0048870,GO:0031960,GO:0006950,GO:0035270,GO:0023052,GO:0048869,	K06828|1|4e-54|211|mmu:17242|midkine	gi|6754668|ref|NP_034914.1|	2.94E-53	midkine precursor [Mus musculus]
mmu-miR-1224-3p	NM_001012363	-34.8	0.058499	-26.12	148	GO:0012505,GO:0016021,GO:0015149,GO:0008645,GO:0006082,	K08146|1|0.0|1002|mmu:117591|MFS transporter, SP family, solute carrier family 2 (facilitated glucose transporter), member 9	gi|156231031|ref|NP_001012363.2|	0	solute carrier family 2, member 9 isoform 2 [Mus musculus]

mmu-miR-1224-3p	NM_001012623	-28.2	0.673271	-26.64	147	GO:0044459,GO:0005083,GO:0046914,GO:0019904,GO:0019222,GO:0048168,GO:0065003,GO:0031340,GO:0015031,	K15291|1|0.0|2320|mmu:116837|regulating synaptic membrane exocytosis protein 1	gi|60592782|ref|NP_001012641.1|	0	regulating synaptic membrane exocytosis protein 1 isoform 3 [Mus musculus]
mmu-miR-1224-3p	NM_001012624	-28.2	0.666778	-26.64	147	GO:0044459,GO:0005083,GO:0046914,GO:0019904,GO:0019222,GO:0048168,GO:0065003,GO:0031340,GO:0015031,	K15291|1|0.0|2291|mmu:116837|regulating synaptic membrane exocytosis protein 1	gi|60592776|ref|NP_001012642.1|	0	regulating synaptic membrane exocytosis protein 1 isoform 4 [Mus musculus]
mmu-miR-1224-3p	NM_001012625	-28.2	0.652481	-26.64	147	GO:0044459,GO:0046872,GO:0019904,GO:0019222,GO:0048168,GO:0065003,GO:0031340,GO:0015031,	K15291|1|0.0|2061|mmu:116837|regulating synaptic membrane exocytosis protein 1	gi|60592786|ref|NP_001012643.1|	0	regulating synaptic membrane exocytosis protein 1 isoform 5 [Mus musculus]

mmu-miR-1224-3p	NM_001012765	-33.1	0.176502	-31.83	165	GO:0043231,GO:0031224,GO:0005886,GO:0004016,GO:0032559,GO:0043169,GO:0035588,GO:0015980,GO:0007267,GO:0007193,GO:0033762,GO:0032147,GO:0010863,GO:0007610,GO:0007169,GO:0007189,GO:0050905,GO:0042044,	K08045|1|0.0|2018|mmu:224129|adenylate cyclase 5 [EC:4.6.1.1]	gi|148747309|ref|NP_001012783.3|	0	adenylate cyclase type 5 [Mus musculus]
mmu-miR-1224-3p	NM_001013028	-31.9	0.2018	-27.53	155	GO:0016020,	-	gi|61557491|ref|NP_001013046.1|	5.46E-39	UPF0444 transmembrane protein C12orf23 homolog [Mus musculus]
mmu-miR-1224-3p	NM_001013360	-32.8	0.178272	-30.63	165	GO:0016020,GO:0001847,GO:0023052,	K11453|1|2e-153|544|ecb:100055293|chromobox protein 6	gi|113374168|ref|NP_001013378.2|	0	neuronal pentraxin with chromo domain isoform 1 [Mus musculus]

mmu-miR-1224-3p	NM_001013362	-32.8	0.1899	-30.63	165	GO:0016020,GO:0001847,GO:0023052,	K11453|1|2e-153|545|ecb:100055293|chromobox protein 6	gi|113374171|ref|NP_001013380.2|	0	neuronal pentraxin with chromo domain isoform 2 [Mus musculus]
mmu-miR-1224-3p	NM_001013371	-26.1	0.960413	-25.28	147	GO:0043231,GO:0046914,GO:0005515,GO:0019787,GO:0006513,GO:0006325,GO:00335	K06058|1|0.0|1474|mmu:209200|deltex	gi|133930784|ref|NP_001013389.2|	0	E3 ubiquitin-protein ligase DTX3L [Mus musculus]
mmu-miR-1224-3p	NM_001014288	-33.3	0.247604	-27.77	168	GO:0016021,GO:0004725,GO:0005488,GO:0004871,GO:0007167,GO:0006470,	K06777|1|0.0|2517|rno:313278|protein tyrosine phosphatase, receptor type, D [EC:3.1.3.48]	gi|90403603|ref|NP_035341.2|	0	receptor-type tyrosine-protein phosphatase delta isoform B precursor [Mus musculus]
mmu-miR-1224-3p	NM_001014761	-29.5	0.519655	-26.79	159	GO:0005887,GO:0005272,GO:0006814,GO:0048731,GO:0007267,GO:0017085,GO:0034220,	K04846|1|2e-105|385|mmu:72821|voltage-gated sodium channel type II beta	gi|148693689|gb|EDL25636.1|	1.37E-105	sodium channel, voltage-gated, type II, beta [Mus musculus]

mmu-miR-1224-3p	NM_001014995	-29.8	0.345855	-26.16	157	GO:0031224,GO:0019904,	-	gi|397492400|ref|XP_003817110.1|	0	PREDICTED: protein FAM189B [Pan paniscus]
mmu-miR-1224-3p	NM_001015039	-31.7	0.228653	-61.9	321	GO:0005769,GO:0046914,GO:0035091,GO:0007176,	K01104|1|4e-15|85.1|tca:657269|protein-tyrosine phosphatase [EC:3.1.3.48]!K12182|2|7e-15|84.3|api:100165322|hepatocyte growth factor-regulated tyrosine kinase substrate!K12482|5|4e-14|82.0|tgu:100229989|RUN and FYVE domain-containin	gi|37360492|dbj|BAC98224.1|	0	mKIAA1643 protein [Mus musculus]
mmu-miR-1224-3p	NM_001018087	-26.5	0.512772	-27.29	150	GO:0005488,GO:0008283,	-	gi|66348058|ref|NP_001018097.1|	1.50E-56	protein LDOC1 [Mus musculus]

mmu-miR-1224-3p	NM_001024616	-30	0.332515	-49.74	312	GO:0043231,GO:0032561,GO:0035556,	-	gi|112181302|ref|NP_001019787.2|	0	rab-like protein 1 [Mus musculus]
mmu-miR-1224-3p	NM_001024618	-33.4	0.351185	-56.58	320	GO:0044459,GO:0046914,GO:0008092,GO:0007010,	K00907|1|5e-11|73.2|dwi:Dwil_GK22102|myosin-light-chain kinase [EC:2.7.11.18]!K09377|5|1e-06|58.5|xtr:448578|cysteine and glycine-rich protein	gi|66841385|ref|NP_001019789.1|	0	xin actin-binding repeat-containing protein 2 isoform 1 [Mus musculus]
mmu-miR-1224-3p	NM_001024699	-39.4	0.004545	-37.67	161	GO:0043169,	K10516|1|0.0|788|mmu:232879|zinc finger and BTB domain-containing protein 45	gi|85701534|ref|NP_001019870.1|	0	zinc finger and BTB domain-containing protein 45 [Mus musculus]
mmu-miR-1224-3p	NM_001024702	-29.8	0.062756	-28.44	159	-	K07296|1|1e-17|90.5|gga:404536|adiponectin	gi|71725391|ref|NP_001019873.1|	4.20E-99	complement C1q-like protein 4 precursor [Mus musculus]

mmu-miR-1224-3p	NM_001024856	-29.6	0.810905	-26.14	157	GO:0004672,GO:0032559,GO:0006468,	K08815|1|0.0|2242|mmu:140810|tau tubulin kinase [EC:2.7.11.26]	gi|67906805|ref|NP_542966.2|	0	tau-tubulin kinase 2 isoform 1 [Mus musculus]
mmu-miR-1224-3p	NM_001024857	-29.6	0.809027	-26.14	157	GO:0004672,GO:0032559,GO:0006468,	K08815|1|0.0|2242|mmu:140810|tau tubulin kinase [EC:2.7.11.26]	gi|67906805|ref|NP_542966.2|	0	tau-tubulin kinase 2 isoform 1 [Mus musculus]
mmu-miR-1224-3p	NM_001025156	-28.1	0.864951	-26.09	157	-	-	gi|148707840|gb|EDL39787.1|	0	coiled-coil domain containing 93, isoform CRA_b [Mus musculus]
mmu-miR-1224-3p	NM_001025245	-31.7	0.275075	-29.21	172	GO:0043231,GO:0044297,GO:0043209,GO:0033267,GO:0016020,GO:0019899,GO:0005198,GO:0008366,GO:0007267,GO:0031345,GO:0002376,	-	gi|69885018|ref|NP_001020416.1|	4.24E-108	Golli-Mbp isoform 2 [Mus musculus]

mmu-miR-1224-3p	NM_001025313	-30.9	0.209647	-27.05	156	GO:0042824,GO:0046977,GO:0015440,GO:0003823,GO:0042287,GO:0002396,GO:0042590,GO:0048193,GO:0050822,GO:00069	K08058|1|0.0|749|mmu:21356|TAP binding protein (tapasin)	gi|148678278|gb|EDL10225.1|	0	TAP binding protein, isoform CRA_a [Mus musculus]
mmu-miR-1224-3p	NM_001025571	-28	0.750887	-46.14	319	GO:0016020,GO:0030674,GO:0019904,GO:0051249,GO:0032502,GO:0007165,	K08273|1|0.0|719|mmu:27371|SH2 domain protein 2A	gi|70909334|ref|NP_001020742.1|	0	SH2 domain-containing protein 2A isoform 2 [Mus musculus]
mmu-miR-1224-3p	NM_001025586	-30.7	0.083607	-29.8	164	GO:0031981,GO:0004871,GO:0023052,	-	gi|70980537|ref|NP_001020757.1|	5.15E-68	nuclear receptor 2C2-associated protein isoform 1 [Mus musculus]
mmu-miR-1224-3p	NM_001025587	-30.7	0.08342	-29.8	164	GO:0031981,GO:0004871,GO:0023052,	-	gi|148696836|gb|EDL28783.1|	6.29E-67	RIKEN cDNA 2310073E15, isoform CRA_b [Mus musculus]
mmu-miR-1224-3p	NM_001025594	-25.8	0.970525	-25.22	150	GO:0031981,GO:0046914,GO:0003676,GO:0006351,	K10514|1|0.0|932|mmu:71834|zinc finger and BTB domain-containing protein	gi|28972211|dbj|BAC65559.1|	0	mKIAA0414 protein [Mus musculus]

mmu-miR-1224-3p	NM_001029890	-33.6	0.168992	-36.91	175	GO:0030529,GO:0043231,GO:0046914,GO:0003676,	K15686|1|0.0|778|mmu:72640|RNA-binding protein MEX3	gi|308193357|ref|NP_001025061.2|	0	RNA-binding protein MEX3A [Mus musculus]
mmu-miR-1224-3p	NM_001029983	-32.9	0.137856	-29.8	156	GO:0031224,GO:0044432,GO:0046872,GO:0015924,GO:0043161,GO:0006487,	K01230|1|0.0|1273|rno:499751|mannosyl-oligosaccharide alpha-1,2-mannosidase [EC:3.2.1.113]	gi|71534295|ref|NP_001025154.1|	0	endoplasmic reticulum mannosyl-oligosaccharide 1,2-alpha-mannosidase [Mus musculus]
mmu-miR-1224-3p	NM_001033042	-30.4	0.251637	-29.07	154	-	-	gi|40675542|gb|AAH64819.1|	0	AI595366 protein, partial [Mus musculus]
mmu-miR-1224-3p	NM_001033170	-29.4	0.374631	-51.54	299	-	-	gi|167000146|ref|NP_001028342.2|	0	protein FAM83E [Mus musculus]
mmu-miR-1224-3p	NM_001033212	-29.1	0.146769	-25.03	161	GO:0031224,	K10128|1|8e-24|111|bta:614739|reprimo, TP53-dependent G2 arrest mediator candidate	gi|85701722|ref|NP_001028384.1|	3.10E-61	reprimo-like protein [Mus musculus]

mmu-miR-1224-3p	NM_001033235	-32.6	0.082757	-29.78	162	GO:0044464,GO:0046914,	K12016|1|7e-142|505|mmu:195359|tripartite motif-containing protein	gi|84794619|ref|NP_001028407.1|	4.58E-141	tripartite motif-containing protein 40 isoform 1 [Mus musculus]
mmu-miR-1224-3p	NM_001033238	-28.1	0.828005	-25.35	151	GO:0031981,GO:0044425,GO:0046914,GO:0060089,GO:0003824,GO:0019900,GO:0050856,GO:0002666,GO:0010033,GO:0042130,GO:0009628,GO:0009719,GO:0009896,GO:00	K04707|1|0.0|1688|mmu:208650|E3 ubiquitin-protein ligase CBL [EC:6.3.2.19]	gi|118344448|ref|NP_001028410.1|	0	E3 ubiquitin-protein ligase CBL-B [Mus musculus]
mmu-miR-1224-3p	NM_001033272	-30	0.629947	-27.58	157	-	K05769|1|0.0|720|mdo:100025913|Rho guanine nucleotide exchange factor 4	gi|242247225|ref|NP_001028444.1|	0	spermatogenesis-associated protein 13 [Mus musculus]

mmu-miR-1224-3p	NM_001033306	-28.9	0.422139	-26.22	146	GO:0016020,GO:0030674,GO:0048646,GO:0032501,GO:0007165,GO:0012501,GO:0048869,GO:0042113,	K07365|1|6e-10|67.4|hmg:100201881|NCK adaptor protein!K06619|2|7e-08|60.5|bmy:Bm1_30590|proto-oncogene tyrosine-protein kinase ABL1 [EC:2.7.10.2]!K08887|3|2e-07|59.3|oaa:100081824|proto-oncogene tyrosine-protein kinase	gi|37747535|gb|AAH58986.1|	0	Shb protein, partial [Mus musculus]

mmu-miR-1224-3p	NM_001033338	-28.5	0.752823	-27.27	161	GO:0043231,	K07210|1|9e-06|54.7|ssc:100127355|inhibitor of nuclear factor kappa-B kinase subunit gamma	gi|191252808|ref|NP_001028510.2|	0	RIMS-binding protein 3 [Mus musculus]
mmu-miR-1224-3p	NM_001033383	-29.9	0.415459	-30.38	156	GO:0005488,	K09228|1|4e-62|241|ecb:100062322|KRAB domain-containing zinc finger protein	gi|75677466|ref|NP_001028555.1|	0	zinc finger protein 865 [Mus musculus]
mmu-miR-1224-3p	NM_001033433	-28.9	0.30663	-26.05	163	GO:0044464,GO:0005488,GO:0006915,GO:0023052,GO:0010033,	-	gi|145966766|ref|NP_001028605.2|	0	transmembrane protein 102 [Mus musculus]
mmu-miR-1224-3p	NM_001033500	-33	0.189402	-25.14	167	-	-	gi|148226059|ref|NP_001028672.2|	0	WD repeat domain 72 [Mus musculus]
mmu-miR-1224-3p	NM_001033539	-32.4	0.030052	-25.88	158	GO:0044424,	-	gi|84370324|ref|NP_001028711.1|	7.12E-54	protein BEX6 [Mus musculus]

mmu-miR-1224-3p	NM_001033776	-26	0.869141	-29.45	156	-	-	gi|76253890|ref|NP_001028948.1|	0	vertnin [Mus musculus]
mmu-miR-1224-3p	NM_001034859	-29.3	0.379868	-27.76	153	-	K14139|1|1e-30|136|cfa:481473|immunity-related GTPase family M protein [EC:3.6.5.-]!K14140|2|2e-26|122|rno:303163|interferon gamma induced GTPase	gi|294712534|ref|NP_001030031.2|	0	interferon-inducible GTPase-like [Mus musculus]
mmu-miR-1224-3p	NM_001034860	-37.8	0.006845	-32.19	159	-	K10523|1|3e-76|286|acs:100564527|speckle-type POZ	gi|85702195|ref|NP_001030032.1|	6.35E-138	TD and POZ domain containing-like isoform 2 [Mus musculus]

mmu-miR-1224-3p	NM_001034869	-28.3	0.355274	-25.36	153	-	K09381|1|3e-06|54.3|dya:Dyak_GE12435|paired box protein 3/7!K09371|5|7e-06|53.1|hsa:4010|LIM homeobox transcription factor 1	gi|85702209|ref|NP_001030041.1|	4.71E-149	uncharacterized protein LOC245263 [Mus musculus]
mmu-miR-1224-3p	NM_001034872	-30	0.233163	-27.56	161	GO:0016831,GO:0006595,	K01581|1|3e-171|602|mcc:705256|ornithine decarboxylase [EC:4.1.1.1	gi|85702213|ref|NP_001030044.1|	0	ornithine decarboxylase-like [Mus musculus]
mmu-miR-1224-3p	NM_001037294	-28.5	0.819238	-55.26	306	GO:0016740,	K08868|1|0.0|4208|mmu:225638|alpha-kinase	gi|254675181|ref|NP_001032371.1|	0	alpha-protein kinase 2 [Mus musculus]

mmu-miR-1224-3p	NM_001037665	-26.9	0.573836	-26.86	152	GO:0043231,GO:0046914,GO:0003676,GO:0006351,	K09228|1|0.0|848|mmu:22640|KRAB domain-containing zinc finger protein	gi|74150287|dbj|BAE32201.1|	0	unnamed protein product [Mus musculus]
mmu-miR-1224-3p	NM_001037711	-30.1	0.472293	-26.97	148	GO:0043296,GO:0015629,GO:0008092,	K06102|1|0.0|1888|mmu:70737|cingulin	gi|50510881|dbj|BAD32426.1|	0	mKIAA1319 protein [Mus musculus]
mmu-miR-1224-3p	NM_001037764	-33.6	0.208433	-29.27	171	GO:0043231,GO:0046914,GO:0035264,GO:0048731,GO:0006355,	K09188|1|3e-13|79.7|dme:Dmel_CG3848|histone-lysine N-methyltransferase MLL3 [EC:2.1.1.43]	gi|83649745|ref|NP_001032853.1|	0	retinoic acid-induced protein 1 [Mus musculus]

mmu-miR-1224-3p	NM_001038607	-26.5	0.9733	-48.41	307	GO:0005887,GO:0004673,GO:0005249,GO:0060089,GO:0005515,GO:0006796,GO:0000768,GO:0007165,GO:0006351,GO:0034220,GO:0030001,	K04904|1|0.0|1820|mmu:16510|potassium voltage-gated channel Eag-related subfamily H member 1	gi|84370250|ref|NP_001033696.1|	0	potassium voltage-gated channel subfamily H member 1 isoform 2 [Mus musculus]

mmu-miR-1224-3p	NM_001038642	-26.6	0.915168	-44.44	294	GO:0043231,GO:0043234,GO:0005515,GO:0001071,GO:0003677,GO:0021536,GO:0009605,GO:0022617,GO:0034614,GO:0008283,GO:0043627,GO:0022602,GO:0034405,GO:0045639,GO:0034097,GO:0051726,GO:0012502,GO:0030575,GO:0045765,GO:0016477,GO	K02678|1|0.0|735|mmu:23871|c-ets proto-oncogene protein	gi|84579950|ref|NP_001033731.1|	0	protein C-ets-1 isoform 2 [Mus musculus]
mmu-miR-1224-3p	NM_001038658	-31.3	0.303374	-58.43	316	GO:0044459,GO:0031224,GO:0012501,GO:0007399,	K06890|1|3e-66|254|bta:404134|	gi|37360156|dbj|BAC98056.1|	5.95E-157	mKIAA0950 protein [Mus musculus]

mmu-miR-1224-3p	NM_001038699	-27	0.820266	-25.37	151	GO:0044444,GO:0016301,GO:0006520,GO:0006040,GO:0006796,	K15522|1|3e-116|421|mmu:63828|protein-fructosamine 3-kinase [EC:2.7.1.1	gi|84662727|ref|NP_001033788.1|	2.16E-115	fructosamine-3-kinase isoform b [Mus musculus]
mmu-miR-1224-3p	NM_001039179	-26.1	0.978989	-72.78	462	GO:0031982,GO:0043232,GO:0043231,GO:0016020,GO:0005083,GO:0005488,GO:0019222,GO:0034453,GO:0072384,	K11498|1|4e-16|89.4|mdo:100013897|centromeric protein E!K10352|4|3e-14|83.2|aml:100473449|myosin heavy chain	gi|28972353|dbj|BAC65630.1|	0	mKIAA0699 protein [Mus musculus]

mmu-miR-1224-3p	NM_001039484	-26.4	0.949134	-27.82	155	GO:0016021,GO:0043234,GO:0042995,GO:0044459,GO:0005515,GO:0032559,GO:0005242,GO:0055065,GO:0014003,GO:0050953,GO:0048168,GO:0015813,GO:0051931,GO:0071805,GO:0021545,GO:0009416,GO:0031960,GO:0051260,GO:0042391,GO:0006952,GO:0051933,GO:00083	K05003|1|0.0|807|ptr:457431|potassium inwardly-rectifying channel subfamily J member 10	gi|71534081|gb|AAH99932.1|	0	Kcnj10 protein, partial [Mus musculus]

mmu-miR-1224-3p	NM_001039537	-34.3	0.081522	-32.32	156	GO:0044421,GO:0005126,GO:0033138,GO:0042508,GO:0048609,GO:0003006,GO:0045687,GO:0060706,GO:0046887,GO:0043409,GO:0048771,GO:0072182,GO:0010975,GO:0010740,GO:0008283,GO:0003337,GO:0001525,GO:0002763,GO:0007167,GO:0030323,GO:0042503,GO:00109	K05419|1|4e-94|347|rno:60584|leukemia inhibitory factor	gi|6678694|ref|NP_032527.1|	1.57E-102	leukemia inhibitory factor isoform a precursor [Mus musculus]
mmu-miR-1224-3p	NM_001040131	-30.2	0.606934	-28.38	158	GO:0043234,GO:0044424,GO:0008135,GO:0006413,GO:0022402,	K03260|1|0.0|1445|mmu:13690|translation initiation factor 4G	gi|148685061|gb|EDL17008.1|	0	eukaryotic translation initiation factor 4, gamma 2, isoform CRA_d [Mus musculus]

mmu-miR-1224-3p	NM_001040426	-26.3	0.990978	-25.97	150	GO:0031012,GO:0004175,	K08630|1|7e-87|324|acs:100566040|a disintegrin and metalloproteinase with thrombospondin motifs 16 [EC:3.4.24.-]	gi|283135196|ref|NP_001035516.2|	0	thrombospondin type-1 domain-containing protein 4 isoform a precursor [Mus musculus]
mmu-miR-1224-3p	NM_001042541	-28.4	0.608496	-45.75	309	GO:0005743,GO:0031224,GO:0045202,GO:0016772,GO:0051018,GO:0003676,GO:0019903,GO:0015631,GO:0019900,GO:0042992,GO:0032868,GO:0003300,GO:0010627,GO:00	-	gi|110347477|ref|NP_001036006.1|	0	A-kinase anchor protein 1, mitochondrial [Mus musculus]

mmu-miR-1224-3p	NM_001045530	-30.4	0.244783	-27.87	153	-	K06627|1|1e-09|66.2|hmg:100206919|cyclin A!K05868|4|2e-07|59.3|nvi:100122223|cyclin	gi|113865891|ref|NP_001038995.1|	0	cyclin-J-like protein [Mus musculus]
mmu-miR-1224-3p	NM_001048054	-27.2	0.815738	-71.54	457	GO:0043231,GO:0031410,GO:0016772,GO:0005488,GO:0008138,GO:0000165,GO:0006796,GO:0045208,GO:0010741,GO:00064	K04459|1|0.0|678|mmu:70686|dual specificity phosphatase [EC:3.1.3.16 3.1.3.48]	gi|148678577|gb|EDL10524.1|	0	dual specificity phosphatase 16, isoform CRA_c [Mus musculus]
mmu-miR-1224-3p	NM_001048061	-31.2	0.172116	-26.28	147	GO:0032991,GO:0043231,GO:0003723,GO:0001071,GO:0043565,GO:0000904,	K13044|1|2e-110|400|rno:83498|heterogeneous nuclear ribonucleoprotein A/B/D	gi|146260280|ref|NP_001041526.1|	1.45E-109	heterogeneous nuclear ribonucleoprotein A/B isoform 1 [Mus musculus]

mmu-miR-1224-3p	NM_001048139	-30	0.419545	-27.51	151	GO:0030136,GO:0005167,GO:0023061,GO:0033555,GO:0045597,GO:0007405,GO:0031175,GO:0007610,GO:0051899,GO:0014070,GO:0008037,GO:0033273,GO:0016202,GO:0006935,GO:0006954,GO:0035295,GO:0009719,GO:0048169,GO:0043066,GO:00	K04355|1|2e-134|481|mmu:12064|brain-derived neurotrophic factor	gi|114326463|ref|NP_031566.4|	7.47E-138	brain-derived neurotrophic factor isoform 1 precursor [Mus musculus]

mmu-miR-1224-3p	NM_001048141	-30	0.406394	-27.51	151	GO:0030136,GO:0005167,GO:0023061,GO:0033555,GO:0045597,GO:0007405,GO:0031175,GO:0007610,GO:0051899,GO:0014070,GO:0008037,GO:0033273,GO:0016202,GO:0006935,GO:0006954,GO:0035295,GO:0009719,GO:0048169,GO:0043066,GO:00	K04355|1|2e-134|481|mmu:12064|brain-derived neurotrophic factor	gi|114326463|ref|NP_031566.4|	7.15E-138	brain-derived neurotrophic factor isoform 1 precursor [Mus musculus]

mmu-miR-1224-3p	NM_001048142	-30	0.40706	-27.51	151	GO:0030136,GO:0005167,GO:0023061,GO:0033555,GO:0045597,GO:0007405,GO:0031175,GO:0007610,GO:0051899,GO:0014070,GO:0008037,GO:0033273,GO:0016202,GO:0006935,GO:0006954,GO:0035295,GO:0009719,GO:0048169,GO:0043066,GO:00	K04355|1|3e-145|517|pon:100447350|brain-derived neurotrophic factor	gi|397174764|ref|NP_001257559.1|	7.86E-161	brain-derived neurotrophic factor isoform 1 [Rattus norvegicus]
mmu-miR-1224-3p	NM_001048148	-28.2	0.413122	-52.5	309	GO:0043231,GO:0046914,GO:0001071,GO:0003677,GO:0007176,GO:0006355,	K13103|1|7e-10|66.6|tsp:Tsp_06173|tuftelin-interacting protein 11	gi|148675464|gb|EDL07411.1|	0	zinc finger, CCCH-type with G patch domain, isoform CRA_a [Mus musculus]

mmu-miR-1224-3p	NM_001048208	-29.8	0.625068	-26.33	156	GO:0043234,GO:0031981,GO:0004871,GO:0008134,GO:0003713,GO:0006357,GO:0030518,GO:0006352,	K15156|1|0.0|2606|mmu:26896|mediator of RNA polymerase II transcription subunit 14	gi|115270977|ref|NP_001041673.1|	0	mediator of RNA polymerase II transcription subunit 14 isoform a [Mus musculus]
mmu-miR-1224-3p	NM_001048227	-30.9	0.273131	-27.4	168	GO:0033673,	-	gi|148674448|gb|EDL06395.1|	3.67E-61	dysbindin (dystrobrevin binding protein 1) domain containing 2, isoform CRA_e [Mus musculus]
mmu-miR-1224-3p	NM_001048228	-30.9	0.274888	-27.4	168	GO:0033673,	-	gi|148674447|gb|EDL06394.1|	1.32E-58	dysbindin (dystrobrevin binding protein 1) domain containing 2, isoform CRA_d [Mus musculus]
mmu-miR-1224-3p	NM_001048229	-30.9	0.275108	-27.4	168	GO:0005488,GO:0033673,	-	gi|148674447|gb|EDL06394.1|	1.05E-47	dysbindin (dystrobrevin binding protein 1) domain containing 2, isoform CRA_d [Mus musculus]
mmu-miR-1224-3p	NM_001077410	-29.1	0.495427	-29.14	165	GO:0043231,GO:0032561,	K05305|1|8e-13|77.4|dre:100150934|fucokinase [EC:2.7.1.52]	gi|47059011|ref|NP_997651.1|	0	GTPase IMAP family member 8 [Mus musculus]

mmu-miR-1224-3p	NM_001077508	-26.4	0.673776	-45.82	300	GO:0009986,GO:0016021,GO:0005488,GO:0004871,GO:0008283,GO:0012502,	K05146|1|1e-124|447|mmu:21942|tumor necrosis factor receptor superfamily member	gi|117422435|ref|NP_001070976.1|	1.01E-123	tumor necrosis factor receptor superfamily member 9 isoform 2 precursor [Mus musculus]
mmu-miR-1224-3p	NM_001079686	-25.1	0.999843	-32.12	165	GO:0031224,GO:0031965,GO:0030016,GO:0031981,GO:0034992,GO:0008092,GO:0007010,GO:0043578,GO:0030154,GO:00516	K10382|1|5e-60|235|gga:421884|dystonin	gi|119120865|ref|NP_001073154.1|	0	nesprin-1 isoform 3 [Mus musculus]
mmu-miR-1224-3p	NM_001079932	-28.3	0.464749	-28.82	160	GO:0012506,GO:0005886,GO:0046914,GO:0005543,GO:0016192,GO:0051259,GO:0007009,GO:0003008,GO:0048513,	K12036|1|0.0|931|mmu:434246|tripartite motif-containing protein 72	gi|121247302|ref|NP_001073401.1|	0	tripartite motif-containing protein 72 [Mus musculus]

mmu-miR-1224-3p	NM_001080557	-25.9	0.891053	-58.37	330	GO:0031966,GO:0016021,GO:0008021,GO:0044459,GO:0030139,GO:0012506,GO:0005488,GO:0006810,GO:0001505,	K08510|1|4e-36|154|mmu:22317|vesicle-associated membrane protein 1	gi|74186948|dbj|BAE20518.1|	1.22E-68	unnamed protein product [Mus musculus]
mmu-miR-1224-3p	NM_001080808	-27.7	0.442944	-25.23	151	GO:0005815,GO:0005083,GO:0008092,GO:0048731,GO:0046907,GO:0030030,	K15369|1|2e-06|55.1|cqu:CpipJ_CPIJ001929|trafficking kinesin-binding protein 1	gi|124248514|ref|NP_001074277.1|	0	bicaudal D-related protein 1 [Mus musculus]
mmu-miR-1224-3p	NM_001080930	-27.3	0.93987	-25.77	151	GO:0043231,GO:0043005,	-	gi|124286823|ref|NP_001074399.1|	0	ataxin-1-like [Mus musculus]
mmu-miR-1224-3p	NM_001080977	-33.5	0.168484	-28.18	157	-	-	gi|124430547|ref|NP_001074446.1|	0	round spermatid basic protein 1-like [Mus musculus]
mmu-miR-1224-3p	NM_001029877	-24.6	0.999825	-75.35	458	GO:0043231,GO:0003676,	K14944|1|9e-135|483|ssc:100512786|RNA-binding protein Nova	gi|119577801|gb|EAW57397.1|	2.93E-136	neuro-oncological ventral antigen 2 [Homo sapiens]

mmu-miR-1224-5p	NM_001001309	-30.2	0.54675	-29.56	153	GO:0032589,GO:0043025,GO:0044430,GO:0005887,GO:0004871,GO:0007417,GO:0043062,GO:0048869,GO:0042471,GO:0007611,GO:0007179,GO:0001822,GO:00315	K06584|1|0.0|2086|mmu:241226|integrin alpha 8	gi|47717127|ref|NP_001001309.1|	0	integrin alpha-8 precursor [Mus musculus]
mmu-miR-1224-5p	NM_001001327	-34.6	0.013386	-55.07	324	GO:0031224,GO:0006775,	K05357|1|3e-10|66.6|aml:100476494|vitamin-K-epoxide reductase (warfarin-sensitive) [EC:1.1.4.1]	gi|47717109|ref|NP_001001327.1|	1.33E-71	vitamin K epoxide reductase complex subunit 1-like protein 1 isoform 2 [Mus musculus]

mmu-miR-1224-5p	NM_001001489	-27	0.685209	-25.76	153	GO:0044459,GO:0031224,GO:0015291,GO:0030001,GO:0008645,	K08175|1|6e-58|226|rno:337920|MFS transporter, FHS family, Na+ dependent glucose transporter 1	gi|18255724|gb|AAH21785.1|	5.64E-76	BC021785 protein, partial [Mus musculus]

mmu-miR-1224-5p	NM_001001602	-27.3	0.931569	-48.43	306	GO:0009898,GO:0044424,GO:0008047,GO:0060090,GO:0005083,GO:0042802,GO:0019901,GO:0019903,GO:0032813,GO:0006915,GO:0035411,GO:0016049,GO:0032318,GO:0034260,GO:0043433,GO:0001837,GO:0006469,GO:0070849,GO:0030178,GO:0007173,GO:0000122,GO:00082	K04352|1|4e-46|188|cin:100184835|Ras GTPase-activating protein 1	gi|166295210|ref|NP_001001602.2|	0	disabled homolog 2-interacting protein isoform 3 [Mus musculus]
mmu-miR-1224-5p	NM_001001807	-26.5	0.392425	-30.5	161	GO:0004871,GO:0023052,	K04257|1|1e-165|582|mmu:258502|olfactory	gi|49227367|ref|NP_001001807.1|	9.22E-165	olfactory receptor 279 [Mus musculus]
mmu-miR-1224-5p	NM_001001985	-33.1	0.253856	-27.29	153	GO:0005789,GO:0005740,GO:0008080,	-	gi|134288912|ref|NP_001001985.3|	4.43E-124	N-acetylaspartate synthetase [Mus musculus]

mmu-miR-1224-5p	NM_001001986	-35.4	0.096819	-27.75	159	-	-	gi|50925384|gb|AAH79552.1|	0	8430427H17Rik protein, partial [Mus musculus]
mmu-miR-1224-5p	NM_001002012	-28.5	0.486395	-26.11	160	GO:0043073,GO:0000794,GO:0005515,GO:0032559,GO:0008289,GO:0007127,GO:0070193,GO:0031660,GO:0007281,GO:0048232,GO:0006950,	K03283|1|0.0|1139|mmu:15512|heat shock 70kDa protein 1/8	gi|31560686|ref|NP_032327.2|	0	heat shock-related 70 kDa protein 2 [Mus musculus]
mmu-miR-1224-5p	NM_001002775	-31	0.291291	-49.58	303	-	-	gi|377834441|ref|XP_003689482.1|	0	PREDICTED: uncharacterized protein LOC442809 [Mus musculus]

mmu-miR-1224-5p	NM_001003912	-31.7	0.34863	-54.46	317	GO:0016020,GO:0043231,GO:0044444,GO:0005102,GO:0005088,GO:0060089,GO:0008047,GO:0007010,GO:0007266,GO:0007163,GO:0006355,GO:0006936,GO:0007169,GO:0006917,GO:0009966,GO:0006935,GO:00	K12331|1|0.0|2758|mmu:213498|Rho guanine nucleotide exchange factor 11	gi|51491850|ref|NP_001003912.1|	0	rho guanine nucleotide exchange factor 11 [Mus musculus]
mmu-miR-1224-5p	NM_001003917	-33.2	0.140252	-29.98	164	GO:0031224,GO:0005774,GO:0031982,GO:0005770,GO:0006810,GO:0044248,GO:00070	-	gi|86990446|ref|NP_001003917.2|	0	autophagy-related protein 9A [Mus musculus]

mmu-miR-1224-5p	NM_001003960	-30.3	0.427797	-56.01	318	GO:0000790,GO:0046914,GO:0003712,GO:0003886,GO:0043565,GO:0042221,GO:0006575,GO:0045597,GO:0031062,GO:0006342,GO:0032776,GO:0008104,GO:0006790,GO:0009314,GO:0031061,	K00558|1|0.0|1595|mmu:13436|DNA (cytosine-5-)-methyltransferase [EC:2.1.1.37]	gi|172088101|ref|NP_001003960.2|	0	DNA (cytosine-5)-methyltransferase 3B isoform 2 [Mus musculus]

mmu-miR-1224-5p	NM_001003961	-30.3	0.432513	-56.01	318	GO:0000790,GO:0046914,GO:0003712,GO:0003886,GO:0043565,GO:0042221,GO:0006575,GO:0045597,GO:0031062,GO:0006342,GO:0032776,GO:0008104,GO:0006790,GO:0009314,GO:0031061,	K00558|1|0.0|1572|mmu:13436|DNA (cytosine-5-)-methyltransferase [EC:2.1.1.37]	gi|172088099|ref|NP_001003961.2|	0	DNA (cytosine-5)-methyltransferase 3B isoform 1 [Mus musculus]

mmu-miR-1224-5p	NM_001003963	-30.3	0.417497	-56.01	318	GO:0000790,GO:0046914,GO:0003712,GO:0003886,GO:0043565,GO:0042221,GO:0006575,GO:0045597,GO:0031062,GO:0006342,GO:0032776,GO:0008104,GO:0006790,GO:0009314,GO:0031061,	K00558|1|0.0|1350|mmu:13436|DNA (cytosine-5-)-methyltransferase [EC:2.1.1.37]	gi|172088105|ref|NP_001003963.2|		0	DNA (cytosine-5)-methyltransferase 3B isoform 4 [Mus musculus]
mmu-miR-1224-5p	NM_001004149	-30.7	0.255564	-29.56	154	GO:0043231,GO:0046914,GO:0003676,	K09228|1|1e-70|269|mcc:711981|KRAB domain-containing zinc finger protein	gi|51921301|ref|NP_001004149.1|		0	zinc finger protein 366 [Mus musculus]

mmu-miR-1224-5p	NM_001004150	-30.1	0.238688	-46.24	309	GO:0031228,GO:0035250,GO:0006687,GO:0016044,	K01988|1|0.0|690|mmu:239559|lactosylceramide 4-alpha-galactosyltransferase [EC:2.4.1.228]	gi|51921295|ref|NP_001004150.1|	0	lactosylceramide 4-alpha-galactosyltransferase [Mus musculus]
mmu-miR-1224-5p	NM_001004185	-33.3	0.107143	-30.45	170	GO:0044444,GO:0043229,	-	gi|60359958|dbj|BAD90198.1|	0	mKIAA1971 protein [Mus musculus]
mmu-miR-1224-5p	NM_001004194	-31.8	0.209428	-28.48	152	GO:0032559,GO:0006950,	K12800|1|2e-124|448|aml:100468792|NACHT, LRR and PYD domains-containing protein 3	gi|256542271|ref|NP_001004194.2|	0	NACHT, LRR and PYD domains-containing protein 4E [Mus musculus]

mmu-miR-1224-5p	NM_001005868	-29.4	0.706791	-33.51	162	GO:0043231,GO:0009925,GO:0005578,GO:0005102,GO:0005198,GO:0008105,GO:0035088,GO:0040007,GO:0007010,GO:0007169,GO:0006886,	K12796|1|0.0|2752|mmu:59079|erbb2-interacting protein	gi|54607114|ref|NP_001005868.1|	0	protein LAP2 isoform 1 [Mus musculus]
mmu-miR-1224-5p	NM_001007582	-30.8	0.026194	-26.21	154	-	K05230|1|6e-29|126|mmu:382000|defensin, alpha	gi|57222284|ref|NP_001007583.1|	3.59E-28	uncharacterized protein LOC382000 precursor [Mus musculus]
mmu-miR-1224-5p	NM_001008794	-25.4	0.92091	-25.9	160	GO:0030425,GO:0044297,GO:0015629,GO:0005902,GO:0002141,GO:0031513,GO:0030427,GO:0005515,GO:0050954,GO:0030030,	K06092|1|5e-06|54.3|rno:140581|InaD-like protein!K05631|2|9e-06|53.5|bta:783484|atrophin-1 interacting protein 3 (BAI1-associated protein 1)	gi|57012338|ref|NP_001008791.1|	0	whirlin isoform 2 [Mus musculus]

mmu-miR-1224-5p	NM_001008795	-25.4	0.918469	-25.9	160	GO:0030425,GO:0044297,GO:0015629,GO:0005902,GO:0002141,GO:0031513,GO:0030427,GO:0005515,GO:0050954,GO:0030030,	K06092|1|5e-06|54.3|rno:140581|InaD-like protein!K05631|2|8e-06|53.5|bta:783484|atrophin-1 interacting protein 3 (BAI1-associated protein 1)	gi|57526175|ref|NP_001008797.1|	0	whirlin isoform 8 [Mus musculus]
mmu-miR-1224-5p	NM_001008796	-29.2	0.37424	-25.9	160	GO:0030425,GO:0044297,GO:0015629,GO:0005902,GO:0002141,GO:0031513,GO:0030427,GO:0005515,GO:0050954,GO:0030030,	K06092|1|4e-06|54.3|rno:140581|InaD-like protein!K05631|2|8e-06|53.5|bta:783484|atrophin-1 interacting protein 3 (BAI1-associated protein 1)	gi|57862816|ref|NP_001008792.1|	0	whirlin isoform 3 [Mus musculus]

mmu-miR-1224-5p	NM_001011524	-30.2	0.089849	-46.34	305	GO:0004888,GO:0023052,	K04257|1|9e-168|589|mmu:258227|olfactory	gi|58372130|ref|NP_001011524.1|	6.03E-167	olfactory receptor 1418 [Mus musculus]
mmu-miR-1224-5p	NM_001011531	-33.9	0.027112	-29.42	150	GO:0004888,GO:0023052,	K04257|1|4e-168|591|mmu:259148|olfactory	gi|289547739|ref|NP_001011531.2|	2.49E-167	olfactory receptor 329 [Mus musculus]
mmu-miR-1224-5p	NM_001011784	-30.4	0.082653	-27.91	154	GO:0004888,GO:0023052,	K04257|1|0.0|636|mmu:257950|olfactory receptor	gi|282847340|ref|NP_001011784.2|	0	olfactory receptor 1039 [Mus musculus]
mmu-miR-1224-5p	NM_001011797	-29.6	0.11472	-28.06	161	GO:0031224,GO:0004930,GO:0023052,GO:0007606,	K04257|1|3e-163|574|mmu:257985|olfactory	gi|58801360|ref|NP_001011797.1|	1.97E-162	olfactory receptor 782 [Mus musculus]
mmu-miR-1224-5p	NM_001011810	-28.1	0.217867	-25.16	160	GO:0016020,GO:0004888,GO:0007166,	K04257|1|3e-146|518|mmu:258489|olfactory	gi|289666805|ref|NP_001011810.2|	3.88E-174	olfactory receptor 485 [Mus musculus]
mmu-miR-1224-5p	NM_001011821	-29.6	0.11472	-28.06	161	GO:0031224,GO:0004930,GO:0023052,GO:0007606,	K04257|1|2e-156|552|mmu:257985|olfactory	gi|58801406|ref|NP_001011821.1|	1.50E-162	olfactory receptor 804 [Mus musculus]

mmu-miR-1224-5p	NM_001011861	-33.9	0.017093	-27.24	153	GO:0004888,GO:0023052,	K04257|1|3e-144|511|mmu:258261|olfactory	gi|374532830|ref|NP_001011861.2|	4.64E-151	olfactory receptor 331 [Mus musculus]
mmu-miR-1224-5p	NM_001011865	-29.6	0.111896	-25.44	150	GO:0031224,GO:0004930,GO:0023052,	K04257|1|1e-173|608|mmu:258189|olfactory	gi|111185926|ref|NP_001011865.2|	9.10E-173	olfactory receptor 624 [Mus musculus]
mmu-miR-1224-5p	NM_001012269	-33.6	0.019118	-29.74	166	GO:0031224,GO:0004930,GO:0023052,GO:0007606,	K04257|1|7e-152|536|mmu:258424|olfactory	gi|282396065|ref|NP_001012269.2|	1.19E-159	olfactory receptor 1513 [Mus musculus]

mmu-miR-1224-5p	NM_001012477	-26	0.981215	-25.81	150	GO:0009986,GO:0016020,GO:0044421,GO:0060089,GO:0005125,GO:0014046,GO:0001935,GO:0007411,GO:0003006,GO:0032414,GO:0021537,GO:0007626,GO:0007276,GO:0002548,GO:0007166,GO:0001525,GO:0003013,GO:0042110,GO:0030832,GO:0006875,GO:0051707,GO:00098	K10031|1|1e-36|157|mmu:20315|C-X-C motif chemokine 12	gi|148667157|gb|EDK99573.1|	2.14E-50	chemokine (C-X-C motif) ligand 12, isoform CRA_c [Mus musculus]
mmu-miR-1224-5p	NM_001013022	-30.9	0.077334	-27.45	158	-	-	gi|61316404|ref|NP_001013040.1|	2.68E-137	outer dense fiber protein 3B [Mus musculus]
mmu-miR-1224-5p	NM_001013256	-34.7	0.0897	-32.31	170	GO:0000783,GO:0043566,GO:0006259,	-	gi|219689064|ref|NP_001013274.2|	0	CST complex subunit CTC1 isoform a [Mus musculus]

mmu-miR-1224-5p	NM_001013385	-29.3	0.435355	-27.14	167	GO:0031982,GO:0042734,GO:0016021,GO:0030425,GO:0033267,GO:0001640,GO:0007193,GO:0007611,GO:0008283,GO:0043523,GO:0030030,GO:0010740,GO:0050804,GO:0001505,GO:0007270,GO:0046903,GO:00	K04607|1|0.0|1623|mmu:268934|metabotropic glutamate receptor 4	gi|62945392|ref|NP_001013403.1|	0	metabotropic glutamate receptor 4 precursor [Mus musculus]
mmu-miR-1224-5p	NM_001013759	-25	0.946994	-37.27	170	GO:0043232,GO:0022402,	K10382|1|3e-12|74.7|spu:756550|dystonin	gi|62000636|ref|NP_001013781.1|	0	GAS2-like protein 2 [Mus musculus]
mmu-miR-1224-5p	NM_001014761	-25.4	0.984427	-55.24	324	GO:0005887,GO:0005272,GO:0006814,GO:0048731,GO:0007267,GO:0017085,GO:0034220,	K04846|1|2e-105|385|mmu:72821|voltage-gated sodium channel type II beta	gi|148693689|gb|EDL25636.1|	1.37E-105	sodium channel, voltage-gated, type II, beta [Mus musculus]

mmu-miR-1224-5p	NM_001014995	-29.1	0.471303	-26.35	149	GO:0031224,GO:0019904,	-	gi|397492400|ref|XP_003817110.1|	0	PREDICTED: protein FAM189B [Pan paniscus]
mmu-miR-1224-5p	NM_001024205	-33.5	0.335582	-30	162	GO:0005840,GO:0043231,GO:0003676,	-	gi|28972720|dbj|BAC65776.1|	0	mKIAA1321 protein [Mus musculus]
mmu-miR-1224-5p	NM_001024604	-33.8	0.160147	-29.46	159	GO:0031981,GO:0005488,	K15502|1|0.0|2039|mmu:105522|serine/threonine-protein phosphatase 6 regulatory ankyrin repeat subunit A	gi|148692871|gb|EDL24818.1|	0	ankyrin repeat domain 28, isoform CRA_a [Mus musculus]
mmu-miR-1224-5p	NM_001024927	-26.2	0.985519	-52.42	307	GO:0031224,GO:0005548,GO:0046872,GO:0005102,GO:0050953,GO:0006650,	K13619|1|6e-09|65.5|ame:724115|phospholipase DDHD1 [EC:3.1.1.-]	gi|67972419|ref|NP_001020098.1|	0	membrane-associated phosphatidylinositol transfer protein 3 isoform 1 [Mus musculus]
mmu-miR-1224-5p	NM_001024931	-31	0.247667	-29.72	160	GO:0043231,GO:0044297,GO:0003676,GO:0006396,	K14946|1|3e-146|520|mmu:52897|RNA binding protein fox-1	gi|148702729|gb|EDL34676.1|	2.11E-147	DNA segment, Chr 11, Brigham & Women's Genetics 0517 expressed, isoform CRA_c [Mus musculus]

mmu-miR-1224-5p	NM_001025574	-32.3	0.100242	-57.61	319	-	-	gi|70909314|ref|NP_001020745.1|	0	BPI fold containing family B, member 9B precursor [Mus musculus]
mmu-miR-1224-5p	NM_001025615	-29.3	0.737064	-27.54	151	GO:0044424,GO:0005488,	-	gi|148665283|gb|EDK97699.1|	1.20E-135	coiled-coil domain containing 50, isoform CRA_a [Mus musculus]
mmu-miR-1224-5p	NM_001029838	-28.4	0.622042	-27.34	159	GO:0005856,GO:0043231,GO:0003677,GO:0001071,GO:0003779,GO:0006355,	K15613|1|3e-59|231|nvi:100117144|homeobox protein Meis 1	gi|71067110|ref|NP_683752.2|	0	homeobox protein PKNOX2 [Mus musculus]
mmu-miR-1224-5p	NM_001029877	-25.3	0.99923	-50.51	302	GO:0043231,GO:0003676,	K14944|1|9e-135|483|ssc:100512786|RNA-binding protein Nova	gi|119577801|gb|EAW57397.1|	2.93E-136	neuro-oncological ventral antigen 2 [Homo sapiens]
mmu-miR-1224-5p	NM_001029895	-30.3	0.482758	-27.35	161	GO:0043231,GO:0016747,GO:0016755,GO:0006464,	K00685|1|0.0|946|mmu:11907|arginine-tRNA-protein transferase [EC:2.3.2.8]	gi|71274127|ref|NP_001025066.1|	0	arginyl-tRNA--protein transferase 1 isoform 3 [Mus musculus]

mmu-miR-1224-5p	NM_001029985	-25.1	0.993194	-51.7	305	-	K03900|1|1e-58|229|mmu:22371|von Willebrand factor	gi|327180710|ref|NP_001025156.3|	0	kielin/chordin-like protein precursor [Mus musculus]
mmu-miR-1224-5p	NM_001029990	-25.6	0.758479	-26.06	165	GO:0043231,GO:0030529,GO:0046914,GO:0016741,GO:0006730,GO:0010467,	-	gi|148710332|gb|EDL42278.1|	0	methyltransferase 11 domain containing 1, isoform CRA_b [Mus musculus]
mmu-miR-1224-5p	NM_001031772	-29.9	0.565501	-28.59	154	GO:0043231,GO:0003676,GO:0046914,GO:0035196,GO:0010586,GO:0006351,	K09276|1|2e-10|70.1|spu:580680|Y-box-binding protein 1!K09277|2|2e-10|70.1|xtr:100170415|Y-box-binding	gi|78042603|ref|NP_001026942.1|	7.05E-160	protein lin-28 homolog B [Mus musculus]

mmu-miR-1224-5p	NM_001033150	-30.3	0.41959	-28.99	157	GO:0044424,GO:0008092,GO:0006996,	K15348|1|0.0|1864|mmu:69582|pleckstrin homology domain-containing family M member 2	gi|226958488|ref|NP_001028322.1|	0	pleckstrin homology domain-containing family M member 2 [Mus musculus]
mmu-miR-1224-5p	NM_001033158	-28.9	0.413961	-26.12	154	GO:0044464,GO:0032561,GO:0017111,GO:0035556,GO:0009207,	K07854|1|1e-148|527|mmu:70784|Ras-like, family 12	gi|251823865|ref|NP_001028330.2|	9.69E-148	ras-like protein family member 12 isoform 1 [Mus musculus]
mmu-miR-1224-5p	NM_001033173	-29.4	0.839599	-44.27	293	GO:0008233,	K11852|1|0.0|1986|mmu:76179|ubiquitin carboxyl-terminal hydrolase 31 [EC:3.1.2.15]	gi|226443061|ref|NP_001028345.1|	0	ubiquitin specific protease 31 [Mus musculus]
mmu-miR-1224-5p	NM_001033185	-27.1	0.47206	-26.1	154	-	-	gi|148707222|gb|EDL39169.1|	7.28E-179	mCG1047291 [Mus musculus]
mmu-miR-1224-5p	NM_001033222	-25.5	0.996991	-48.89	296	GO:0043169,GO:0007165,	K10360|1|1e-06|58.2|mdo:100013745|myosin IX	gi|164698472|ref|NP_001028394.2|	0	PDZ domain containing 8 [Mus musculus]

mmu-miR-1224-5p	NM_001033246	-32.4	0.126825	-27.95	157	GO:0043231,	K12478|1|1e-17|93.2|acs:100552393|early endosome antigen 1	gi|85701744|ref|NP_001028418.1|	0	coiled-coil domain-containing protein 110 [Mus musculus]
mmu-miR-1224-5p	NM_001033306	-27	0.741538	-31.68	165	GO:0016020,GO:0030674,GO:0048646,GO:0032501,GO:0007165,GO:0012501,GO:0048869,GO:0042113,	K07365|1|6e-10|67.4|hmg:100201881|NCK adaptor protein!K06619|2|7e-08|60.5|bmy:Bm1_30590|proto-oncogene tyrosine-protein kinase ABL1 [EC:2.7.10.2]!K08887|3|2e-07|59.3|oaa:100081824|proto-oncogene tyrosine-protein kinase	gi|37747535|gb|AAH58986.1|	0	Shb protein, partial [Mus musculus]

mmu-miR-1224-5p	NM_001033320	-25.1	0.933286	-29.45	167	-	-	gi|85701822|ref|NP_001028492.1|	0	leucine-rich repeat-containing protein 16C [Mus musculus]
mmu-miR-1224-5p	NM_001033337	-26.9	0.852818	-30.85	171	-	K05476|1|3e-06|55.8|mmu:24099|tumor necrosis factor ligand superfamily member	gi|116283802|gb|AAH30849.1|	0	Ttc38 protein [Mus musculus]
mmu-miR-1224-5p	NM_001033348	-27.4	0.974293	-95.98	622	GO:0043231,GO:0044444,GO:0005099,GO:0046983,GO:0032852,	K07207|1|2e-22|110|bfo:BRAFLDRAFT_64809|tuberous sclerosis 2	gi|158187505|ref|NP_001028520.2|	0	ral GTPase-activating protein subunit alpha-2 [Mus musculus]
mmu-miR-1224-5p	NM_001033349	-27.7	0.461282	-45.53	314	-	-	gi|85701850|ref|NP_001028521.1|	3.52E-109	protein ANKUB1 [Mus musculus]

mmu-miR-1224-5p	NM_001033369	-25.9	0.997208	-27.52	152	GO:0044424,GO:0031224,GO:0043235,GO:0070696,GO:0032559,GO:0043169,GO:0017002,GO:0032403,GO:0007179,GO:0006796,GO:0022602,GO:0050796,GO:0009855,GO:0021543,GO:0043434,GO:0006915,GO:0048869,GO:0006464,GO:0009746,GO:0022037,GO:0001832,GO:00108	K13568|1|0.0|774|mcc:697826|activin receptor type-1C [EC:2.7.11.30]	gi|122890143|emb|CAM13261.1|	0	activin A receptor type IC [Mus musculus]
mmu-miR-1224-5p	NM_001033383	-31.4	0.281236	-28.78	155	GO:0005488,	K09228|1|4e-62|241|ecb:100062322|KRAB domain-containing zinc finger protein	gi|75677466|ref|NP_001028555.1|	0	zinc finger protein 865 [Mus musculus]

mmu-miR-1224-5p	NM_001033385	-31.3	0.478847	-49.34	305	-	-	gi|148806863|ref|NP_001028557.2|	0	protein broad-minded [Mus musculus]
mmu-miR-1224-5p	NM_001033393	-25.6	0.978946	-47.59	304	GO:0031224,	-	gi|148702521|gb|EDL34468.1|	0	mCG6601, isoform CRA_c [Mus musculus]
mmu-miR-1224-5p	NM_001033400	-30.5	0.250854	-25.83	154	-	-	gi|85701903|ref|NP_001028572.1|	0	protein FAM22 [Mus musculus]
mmu-miR-1224-5p	NM_001033405	-33.8	0.136768	-29.58	158	GO:0016020,GO:0004871,GO:0005488,GO:0023052,GO:0046649,	K14364|1|4e-173|610|mmu:328833|trem-like transcript 2 protein	gi|133908636|ref|NP_001028577.2|	2.96E-172	trem-like transcript 2 protein precursor [Mus musculus]
mmu-miR-1224-5p	NM_001033427	-25.5	0.904058	-25.93	154	GO:0004553,GO:0000270,GO:0009056,	-	gi|85701937|ref|NP_001028599.1|	2.32E-123	lysozyme g-like protein 2 precursor [Mus musculus]
mmu-miR-1224-5p	NM_001033436	-35.6	0.034097	-55.61	317	-	K11318|1|0.0|1227|mmu:380753|ataxin-7	gi|295317311|ref|NP_001028608.3|	0	ataxin-7-like protein 1 isoform 1 [Mus musculus]
mmu-miR-1224-5p	NM_001033447	-35.6	0.028717	-32.91	161	-	-	gi|353523843|ref|NP_001028619.2|	0	uncharacterized protein LOC381196 [Mus musculus]

mmu-miR-1224-5p	NM_001033538	-36.3	0.041917	-33.52	169	GO:0044459,GO:0043232,	K05726|1|2e-45|185|spu:576962|breast cancer anti-estrogen resistance 1	gi|75677627|ref|NP_001028710.1|	0	cas scaffolding protein family member 4 isoform 1 [Mus musculus]
mmu-miR-1224-5p	NM_001034030	-33.8	0.113505	-29.32	175	GO:0044431,GO:0004672,GO:0046914,GO:0046983,GO:0032559,GO:0006796,GO:0048232,GO:0007281,GO:0006464,	K05744|1|0.0|784|mmu:16886|LIM domain kinase 2 [EC:2.7.11.1]	gi|148708465|gb|EDL40412.1|	0	LIM motif-containing protein kinase 2, isoform CRA_b [Mus musculus]
mmu-miR-1224-5p	NM_001034097	-25.7	0.84747	-31.13	161	-	K05475|1|2e-105|384|mmu:69583|tumor necrosis factor ligand superfamily member	gi|77539772|ref|NP_001029269.1|	0	tumor necrosis factor (ligand) superfamily, member 12-member 13 isoform a [Mus musculus]
mmu-miR-1224-5p	NM_001034867	-27.9	0.845811	-51.05	320	GO:0003824,	-	gi|298493323|ref|NP_001030039.2|	0	peptidase M20 domain-containing protein 2 [Mus musculus]

mmu-miR-1224-5p	NM_001034875	-28.7	0.391714	-25.48	166	GO:0044424,GO:0005488,	K05399|1|2e-16|88.2|hsa:3929|lipopolysaccharide-binding protein	gi|223468638|ref|NP_001030047.2|	0	BPI fold-containing family B member 4 precursor [Mus musculus]
mmu-miR-1224-5p	NM_001034909	-27.4	0.476531	-26.33	153	-	K06751|1|2e-30|134|mmu:547349|major histocompatibility complex, class I	gi|294661840|ref|NP_001030081.2|	2.75E-52	H2-GS14-2 antigen [Mus musculus]
mmu-miR-1224-5p	NM_001037493	-29.2	0.353441	-52.03	305	GO:0031224,GO:0015075,GO:0005488,GO:0006811,	K15122|1|0.0|838|mmu:71699|solute carrier family 41	gi|82734225|ref|NP_001032570.1|	0	solute carrier family 41 member 3 isoform 2 [Mus musculus]
mmu-miR-1224-5p	NM_001037764	-29.7	0.691533	-30	154	GO:0043231,GO:0046914,GO:0035264,GO:0048731,GO:0006355,	K09188|1|3e-13|79.7|dme:Dmel_CG3848|histone-lysine N-methyltransferase MLL3 [EC:2.1.1.43]	gi|83649745|ref|NP_001032853.1|	0	retinoic acid-induced protein 1 [Mus musculus]

mmu-miR-1224-5p	NM_001037859	-26.8	0.871268	-54.45	313	-	K05090|1|0.0|1896|mmu:12978|macrophage colony-stimulating factor 1 receptor [EC:2.7.10.1]	gi|44890287|gb|AAH66863.1|	0	Colony stimulating factor 1 receptor [Mus musculus]
mmu-miR-1224-5p	NM_001037905	-26.6	0.891042	-25.62	158	GO:0043231,GO:0031410,GO:0012505,GO:0005515,GO:0006355,GO:0000902,GO:0043009,GO:0060071,GO:0030178,GO:0001932,GO:0006897,GO:0031331,GO:0016049,GO:00	K12475|1|0.0|853|mmu:13132|disabled homolog 2	gi|74227265|dbj|BAE38389.1|	0	unnamed protein product [Mus musculus]
mmu-miR-1224-5p	NM_001037914	-32.6	0.103064	-29.33	162	-	K10749|1|0.0|657|mmu:622408|geminin	gi|85702306|ref|NP_001033003.1|	0	multicilin [Mus musculus]

mmu-miR-1224-5p	NM_001037922	-30.8	0.210994	-25.19	148	GO:0031981,GO:0005488,GO:0019887,GO:0051726,GO:0019222,GO:0051320,GO:0016310,	K02219|1|6e-40|166|mdo:100021385|cyclin-dependent kinase regulatory subunit CKS1	gi|148679055|gb|EDL11002.1|	6.80E-53	mCG132387 [Mus musculus]
mmu-miR-1224-5p	NM_001038613	-25.1	0.944749	-27.16	153	GO:0044421,GO:0044432,GO:0005488,GO:0006461,GO:0048731,	K04593|1|3e-55|217|mmu:99633|latrophilin 2	gi|84370353|ref|NP_001033702.1|	0	noelin isoform c precursor [Mus musculus]
mmu-miR-1224-5p	NM_001038999	-26.8	0.981605	-49.79	305	-	K14802|1|0.0|2304|mmu:11980|phospholipid-transporting ATPase [EC:3.6.3.1]	gi|60360628|dbj|BAD90541.1|	0	mKIAA4233 protein [Mus musculus]

mmu-miR-1224-5p	NM_001039080	-25.1	0.995511	-26.82	165	GO:0043231,GO:0003676,GO:0010467,	K13208|1|2e-22|110|bfo:BRAFLDRAFT_92208|ELAV like protein 2/3/4!K13088|2|2e-22|110|ssc:100337681|ELAV-like protein 1	gi|84872203|ref|NP_062685.2|	0	RNA-binding motif, single-stranded-interacting protein 2 isoform a [Mus musculus]
mmu-miR-1224-5p	NM_001039114	-29.9	0.316865	-26.08	156	GO:0043231,GO:0044444,GO:0015645,GO:0016289,GO:0032559,GO:0048869,GO:0009062,GO:0048232,	K15013|1|0.0|1331|mmu:328845|long-chain-fatty-acid--CoA ligase ACSBG [EC:6.2.1.3]	gi|84993736|ref|NP_001034203.1|	0	long-chain-fatty-acid--CoA ligase ACSBG2 [Mus musculus]
mmu-miR-1224-5p	NM_001039121	-30.9	0.01856	-27.51	151	GO:0006952,	-	gi|84993767|ref|NP_001034210.1|	4.27E-29	beta-defensin 43 precursor [Mus musculus]
mmu-miR-1224-5p	NM_001039126	-27.2	0.926554	-29.43	156	GO:0007165,GO:0046546,	K10323|1|0.0|658|mmu:65247|ankyrin repeat and SOCS box protein 1	gi|85060520|ref|NP_075533.3|	0	ankyrin repeat and SOCS box protein 1 isoform b [Mus musculus]

mmu-miR-1224-5p	NM_001039167	-31	0.362681	-29.72	160	GO:0043231,GO:0044297,GO:0003676,GO:0006396,	K14946|1|6e-169|596|rno:287847|RNA binding protein fox-1	gi|85677497|ref|NP_001034256.1|	1.25E-170	RNA binding protein fox-1 homolog 3 isoform 1 [Mus musculus]
mmu-miR-1224-5p	NM_001039168	-31	0.35284	-29.72	160	GO:0043231,GO:0044297,GO:0003676,GO:0006396,	K14946|1|4e-150|533|ecb:100050497|RNA binding protein fox-1	gi|148702730|gb|EDL34677.1|	8.41E-156	DNA segment, Chr 11, Brigham & Women's Genetics 0517 expressed, isoform CRA_d [Mus musculus]
mmu-miR-1224-5p	NM_001039485	-29.8	0.808574	-52.16	308	GO:0031224,GO:0015075,GO:0006811,	-	gi|328887946|ref|NP_001034574.4|	0	piezo-type mechanosensitive ion channel component 2 [Mus musculus]

mmu-miR-1224-5p	NM_001039510	-26.9	0.917722	-26.71	161	GO:0033267,GO:0032589,GO:0044430,GO:0043231,GO:0044456,GO:0016021,GO:0044297,GO:0001882,GO:0004620,GO:0005102,GO:0004930,GO:0032403,GO:0046983,GO:0045986,GO:0048871,GO:0002790,GO:0032898,GO:0006470,GO:0035588,GO:0042321,GO:0050961,GO:00313	K04265|1|8e-141|503|mmu:11539|adenosine A1 receptor	gi|148707674|gb|EDL39621.1|	2.23E-144	mCG50903, isoform CRA_a [Mus musculus]

mmu-miR-1224-5p	NM_001039652	-32.9	0.104989	-30.42	163	GO:0016021,GO:0032589,GO:0030425,GO:0005924,GO:0043025,GO:0004985,GO:0009611,GO:0007193,GO:0014073,GO:0014072,GO:0007610,GO:0051931,GO:0051899,GO:0002376,GO:0007189,GO:0002237,GO:00	K04215|1|0.0|879|mmu:18390|mu-type opioid receptor	gi|89001114|ref|NP_001034741.1|	0	mu-type opioid receptor [Mus musculus]
mmu-miR-1224-5p	NM_001039676	-25.9	0.835622	-46.59	313	GO:0016021,GO:0031410,GO:0046915,GO:0006829,	K14709|1|4e-145|516|mmu:214922|solute carrier family 39 (zinc transporter), member 1/2/3	gi|225543187|ref|NP_001034765.2|	2.78E-144	solute carrier family 39 (zinc transporter), member 2 [Mus musculus]
mmu-miR-1224-5p	NM_001040398	-28.1	0.927731	-64.98	450	-	-	-	-	-

mmu-miR-1224-5p	NM_001040691	-32.9	0.071517	-32.24	158	GO:0043231,GO:0005488,GO:0019104,GO:0050263,GO:0006281,GO:0006915,GO:0002566,GO:0051704,GO:0002562,	K03648|1|3e-175|615|mmu:22256|uracil-DNA glycosylase [EC:3.2.2.27]	gi|101943608|ref|NP_001035781.1|	2.06E-174	uracil-DNA glycosylase isoform a [Mus musculus]
mmu-miR-1224-5p	NM_001042411	-27.9	0.617435	-45.36	304	GO:0031012,GO:0043231,GO:0016020,GO:0046914,GO:0019842,GO:0019798,GO:0016701,GO:0008152,GO:0040007,	K08134|1|0.0|1121|rno:114200|leucine proline-enriched proteoglycan (leprecan)	gi|109150433|ref|NP_062757.2|	0	prolyl 3-hydroxylase 1 isoform 2 precursor [Mus musculus]
mmu-miR-1224-5p	NM_001042499	-30.4	0.204385	-27.09	154	GO:0032561,GO:0035556,	K07933|1|8e-126|451|mmu:67657|Rab-like	gi|109948290|ref|NP_001035964.1|	5.43E-125	rab-like protein 3 [Mus musculus]

mmu-miR-1224-5p	NM_001042630	-29.2	0.130405	-30.45	157	GO:0019840,GO:0051234,GO:0001523,	K01830|1|1e-10|67.8|xla:397884|prostaglandin-H2 D-isomerase [EC:5.3.99.2]	gi|110835737|ref|NP_031973.2|	2.70E-97	epididymal-specific lipocalin-5 isoform a precursor [Mus musculus]
mmu-miR-1224-5p	NM_001013749	-20.2	1	-88.14	483	GO:0031224,	-	gi|85701444|ref|NP_001013771.1|	0	transmembrane protein 151B [Mus musculus]
mmu-miR-122-5p	NM_001025438	-29	0.464269	-48.07	326	GO:0031981,GO:0030139,GO:0042383,GO:0043234,GO:0045202,GO:0005515,GO:0004674,GO:0032559,GO:0006941,GO:0007267,GO:0008283,GO:0019221,GO:0006468,GO:0070838,GO:0006950,GO:0016049,GO:0000086,GO:0006875,	K04515|1|0.0|1026|mmu:108058|calcium/calmodulin-dependent protein kinase (CaM kinase) II [EC:2.7.11.17]	gi|26667180|ref|NP_001212.2|	0	calcium/calmodulin-dependent protein kinase type II subunit delta isoform 3 [Homo sapiens]

mmu-miR-122-5p	NM_001025439	-29	0.465349	-48.07	326	GO:0043234,GO:0031981,GO:0045202,GO:0030139,GO:0042383,GO:0004674,GO:0005515,GO:0032559,GO:0006941,GO:0006468,GO:0000086,GO:0006875,GO:0070838,GO:0008283,GO:0006950,	K04515|1|0.0|1045|mcc:702504|calcium/calmodulin-dependent protein kinase (CaM kinase) II [EC:2.7.11.17]	gi|60360170|dbj|BAD90304.1|	0	mKIAA4163 protein [Mus musculus]
mmu-miR-122-5p	NM_001033348	-29.4	0.697243	-49.3	315	GO:0043231,GO:0044444,GO:0005099,GO:0046983,GO:0032852,	K07207|1|2e-22|110|bfo:BRAFLDRAFT_64809|tuberous sclerosis 2	gi|158187505|ref|NP_001028520.2|	0	ral GTPase-activating protein subunit alpha-2 [Mus musculus]
mmu-miR-122-5p	NM_001033355	-27.6	0.844615	-25.33	168	GO:0060026,GO:0014022,	K09228|1|0.0|1403|mmu:243905|KRAB domain-containing zinc finger protein protein	gi|268607554|ref|NP_001028527.2|	0	zinc finger protein 568 isoform 1 [Mus musculus]

mmu-miR-122-5p	NM_001034858	-29.2	0.481258	-45.39	304	-	-	gi|167900462|ref|NP_001030030.2|	0	armadillo repeat-containing protein 2 [Mus musculus]
mmu-miR-122-5p	NM_001035532	-25.6	0.981417	-45.83	308	GO:0032991,GO:0005515,GO:0033036,GO:0007167,GO:0051259,GO:0030036,	-	gi|123231686|emb|CAM21286.1|	0	A kinase (PRKA) anchor protein 2 [Mus musculus]
mmu-miR-122-5p	NM_001035533	-25.6	0.981227	-45.83	308	GO:0032991,GO:0005515,GO:0033036,GO:0007167,GO:0051259,GO:0030036,	-	gi|123231688|emb|CAM21288.1|	0	Palm2-Akap2 fusion protein [Mus musculus]
mmu-miR-122-5p	NM_001081148	-26	0.671205	-25.17	168	GO:0031090,GO:0008391,GO:0016712,GO:0005506,GO:0019369,GO:0010038,GO:0043434,GO:0042493,	K07412|1|0.0|884|mmu:243881|cytochrome P450, family 2, subfamily B [EC:1.14.14.1]	gi|124487255|ref|NP_001074617.1|	0	cytochrome P450, family 2, subfamily b, polypeptide 23 precursor [Mus musculus]

mmu-miR-122-5p	NM_001109684	-28	0.572015	-26.53	161	GO:0005911,GO:0005856,GO:0043231,GO:0030216,	K04407|1|6e-09|64.7|xtr:100491719|mitogen-activated protein kinase kinase kinase kinase 4 [EC:2.7.11.1]!K10352|3|2e-07|59.7|dmo:Dmoj_GI19126|myosin heavy chain!K10421|5|9e-07|57.4|acs:100566771|CAP-Gly domain-containing linker	gi|50510765|dbj|BAD32368.1|	0	mKIAA1026 protein [Mus musculus]

mmu-miR-122-5p	NM_001109685	-28	0.573798	-26.53	161	GO:0005911,GO:0005856,GO:0043231,GO:0030216,	K04407|1|6e-09|64.7|xtr:100491719|mitogen-activated protein kinase kinase kinase kinase 4 [EC:2.7.11.1]!K10352|3|2e-07|59.7|dmo:Dmoj_GI19126|myosin heavy chain!K10421|5|9e-07|57.4|acs:100566771|CAP-Gly domain-containing linker	gi|157837992|ref|NP_001103155.1|	0	kazrin isoform 3 [Mus musculus]
mmu-miR-122-5p	NM_001115077	-26.9	0.366411	-27.24	160	-	-	gi|169234924|ref|NP_001108549.1|	0	uncharacterized protein LOC627009 [Mus musculus]

mmu-miR-122-5p	NM_001159404	-26.2	0.87677	-30.19	174	GO:0015629,GO:0019900,GO:0007417,GO:0065003,GO:0016192,GO:0030036,GO:00300	K06094|1|0.0|1917|mmu:16897|lethal giant larvae	gi|226874867|ref|NP_001152877.1|	0	lethal(2) giant larvae protein homolog 1 isoform 1 [Mus musculus]
mmu-miR-122-5p	NM_001159405	-26.2	0.8718	-30.19	174	GO:0015629,GO:0019900,GO:0007417,GO:0065003,GO:0016192,GO:0030036,GO:00300	K06094|1|0.0|1917|mmu:16897|lethal giant larvae	gi|226874867|ref|NP_001152877.1|	0	lethal(2) giant larvae protein homolog 1 isoform 1 [Mus musculus]
mmu-miR-122-5p	NM_001163531	-25.9	0.798511	-44.69	315	GO:0031224,	-	gi|148688148|gb|EDL20095.1|	0	RIKEN cDNA 3010001K23, isoform CRA_a [Mus musculus]
mmu-miR-122-5p	NM_001163532	-25.9	0.798843	-44.69	315	GO:0031224,	-	gi|148688149|gb|EDL20096.1|	0	RIKEN cDNA 3010001K23, isoform CRA_b [Mus musculus]
mmu-miR-122-5p	NM_001167757	-25.6	0.49033	-25.13	155	GO:0008406,	K15502|1|2e-22|107|rno:306264|serine/threonine-protein phosphatase 6 regulatory ankyrin repeat subunit A	gi|268370076|ref|NP_001161229.1|	1.69E-151	ankyrin repeat domain-containing protein 7 isoform 1 [Mus musculus]

mmu-miR-122-5p	NM_001167872	-27.6	0.844877	-25.33	168	GO:0060026,GO:0014022,	K09228|1|0.0|1403|mmu:243905|KRAB domain-containing zinc finger protein	gi|268607554|ref|NP_001028527.2|	0	zinc finger protein 568 isoform 1 [Mus musculus]
mmu-miR-122-5p	NM_001167873	-27.6	0.844765	-25.33	168	GO:0060026,GO:0014022,	K09228|1|0.0|1397|mmu:243905|KRAB domain-containing zinc finger protein	gi|268607558|ref|NP_001161345.1|	0	zinc finger protein 568 isoform 2 [Mus musculus]
mmu-miR-122-5p	NM_001177396	-26.6	0.85838	-65.09	457	-	K11322|1|8e-13|77.8|api:100158774|enhancer of polycomb-like protein!K12474|2|9e-09|64.3|mgp:100546547|low density lipoprotein receptor adapter protein 1	gi|294345392|ref|NP_001170867.1|	0	ankyrin repeat and sterile alpha motif domain-containing protein 1B isoform 3 [Mus musculus]

mmu-miR-122-5p	NM_001177961	-29.8	0.374427	-25.29	169	GO:0031224,GO:0048731,GO:0048869,	-	gi|148708789|gb|EDL40736.1|	7.01E-174	glycoprotein m6b, isoform CRA_d [Mus musculus]
mmu-miR-122-5p	NM_001271433	-26.4	0.410989	-42.73	300	-	-	-	-	-
mmu-miR-122-5p	NM_001271434	-26.4	0.411551	-42.73	300	-	-	-	-	-
mmu-miR-122-5p	NM_007513	-25.2	0.992544	-27.55	163	GO:0031224,GO:0004871,GO:0015174,GO:0015802,GO:0009966,	K13863|1|0.0|1129|mmu:11987|solute carrier family 7 (cationic amino acid transporter), member 1	gi|148673899|gb|EDL05846.1|	0	solute carrier family 7 (cationic amino acid transporter, y+ system), member 1 [Mus musculus]
mmu-miR-122-5p	NM_008480	-26.5	0.975328	-43.82	295	-	K05637|1|0.0|6084|mmu:16772|laminin, alpha 1/2	gi|117168301|ref|NP_032506.2|	0	laminin subunit alpha-1 precursor [Mus musculus]
mmu-miR-122-5p	NM_008502	-26.2	0.876434	-30.19	174	GO:0015629,GO:0019900,GO:0007417,GO:0065003,GO:0016192,GO:0030036,GO:00300	K06094|1|0.0|1909|mmu:16897|lethal giant larvae	gi|226874867|ref|NP_001152877.1|	0	lethal(2) giant larvae protein homolog 1 isoform 1 [Mus musculus]

mmu-miR-122-5p	NM_008706	-29.2	0.159095	-27.34	170	GO:0044444,GO:0016655,GO:0016653,GO:0048037,GO:0043065,GO:0044092,GO:0006520,GO:0042221,GO:0044271,GO:0007268,GO:0006950,	K00355|1|6e-149|528|mmu:18104|NAD(P)H dehydrogenase (quinone) [EC:1.6.5.2]	gi|94400773|ref|NP_032732.3|	4.29E-148	NAD(P)H dehydrogenase [quinone] 1 [Mus musculus]
mmu-miR-122-5p	NM_009649	-25.6	0.979643	-45.83	308	GO:0032991,GO:0005515,GO:0033036,GO:0007167,GO:0051259,GO:0030036,	-	gi|123231688|emb|CAM21288.1|	0	Palm2-Akap2 fusion protein [Mus musculus]
mmu-miR-122-5p	NM_010487	-30.7	0.305253	-28.16	152	GO:0003723,GO:0048731,GO:0048869,	K13208|1|0.0|638|cfa:484940|ELAV like protein 2/3/4	gi|27229298|ref|NP_758827.1|	2.54E-180	ELAV-like protein 3 [Rattus norvegicus]

mmu-miR-122-5p	NM_011129	-30.2	0.111568	-28.69	173	GO:0043231,GO:0032156,GO:0017111,GO:0032561,GO:0007417,GO:0006915,GO:0009207,GO:0007005,GO:0007281,	K04557|1|5e-159|561|acs:100563096|peanut-like protein 1 (cell division control related protein 1)	gi|6755120|ref|NP_035259.1|	0	septin-4 [Mus musculus]
mmu-miR-122-5p	NM_011546	-28.8	0.604959	-26.45	161	-	K09299|1|0.0|1840|mmu:21417|zinc finger homeobox protein 1/2	gi|6756073|ref|NP_035676.1|	0	zinc finger E-box-binding homeobox 1 [Mus musculus]
mmu-miR-122-5p	NM_011674	-27.9	0.562614	-41.59	321	GO:0031224,GO:0035251,GO:0035250,GO:0006687,GO:0008366,	K04628|1|0.0|1102|mmu:22239|2-hydroxyacylsphingosine 1-beta-galactosyltransferase [EC:2.4.1.45]	gi|31543926|ref|NP_035804.2|	0	2-hydroxyacylsphingosine 1-beta-galactosyltransferase precursor [Mus musculus]

mmu-miR-122-5p	NM_018823	-25.6	0.999027	-43.73	310	-	K04446|1|0.0|2588|mmu:54446|nuclear factor of activated T-cells, cytoplasmic, calcineurin-dependent	gi|163937840|ref|NP_061293.2|	0	nuclear factor of activated T-cells 5 isoform b [Mus musculus]
mmu-miR-122-5p	NM_018881	-25	0.930038	-25.02	154	GO:0031224,GO:0033643,GO:0005789,GO:0016709,GO:0000166,GO:0050662,GO:0055114,GO:0019748,GO:0046496,	K00485|1|0.0|1042|mmu:55990|dimethylaniline monooxygenase (N-oxide forming) [EC:1.14.13.8]	gi|31542819|ref|NP_061369.2|	0	dimethylaniline monooxygenase [N-oxide-forming] 2 [Mus musculus]
mmu-miR-122-5p	NM_019697	-31.9	0.179486	-27.75	173	-	K04892|1|0.0|1140|mmu:16508|potassium voltage-gated channel Shal-related subfamily D member 2	gi|26006243|dbj|BAC41464.1|	0	mKIAA1044 protein [Mus musculus]

mmu-miR-122-5p	NM_019931	-31.5	0.079395	-26.69	166	GO:0005887,GO:0044297,GO:0042995,GO:0043169,GO:0015271,GO:0034220,GO:0030001,	K04893|1|0.0|1155|pon:100431693|potassium voltage-gated channel Shal-related subfamily D member 3	gi|9910302|ref|NP_064315.1|	0	potassium voltage-gated channel subfamily D member 3 isoform 2 precursor [Mus musculus]
mmu-miR-122-5p	NM_021432	-30.8	0.103345	-28.23	164	GO:0005488,	K11283|1|2e-56|221|mmu:58243|nucleosome assembly protein 1-like 5	gi|148666333|gb|EDK98749.1|	1.22E-78	mCG123507 [Mus musculus]
mmu-miR-122-5p	NM_023044	-29.6	0.215236	-26.59	155	GO:0005764,GO:0016020,GO:0015295,GO:0015322,GO:0009057,GO:0015833,GO:0006818,GO:0006811,	K14638|1|0.0|864|mmu:65221|solute carrier family 15 (peptide/histidine transporter), member 3/4	gi|225007542|ref|NP_075531.2|	0	solute carrier family 15 member 3 [Mus musculus]

mmu-miR-122-5p	NM_023395	-31.1	0.047052	-26.13	167	GO:0044421,GO:0004866,GO:0010466,GO:0043627,GO:0008285,GO:0016049,	K01537|1|4e-19|96.3|aml:100474574|Ca2+-transporting ATPase [EC:3.6.3.8]	gi|148679665|gb|EDL11612.1|	1.56E-125	WAP four-disulfide core domain 1, isoform CRA_c [Mus musculus]
mmu-miR-122-5p	NM_023813	-29	0.456803	-48.07	326	GO:0031981,GO:0030139,GO:0042383,GO:0043234,GO:0045202,GO:0005515,GO:0004674,GO:0032559,GO:0006941,GO:0007267,GO:0008283,GO:0019221,GO:0006468,GO:0070838,GO:0006950,GO:0016049,GO:0000086,GO:0006875,	K04515|1|0.0|976|ecb:100072618|calcium/calmodulin-dependent protein kinase (CaM kinase) II [EC:2.7.11.17]	gi|60360170|dbj|BAD90304.1|	0	mKIAA4163 protein [Mus musculus]
mmu-miR-122-5p	NM_025455	-32.3	0.016621	-50.65	337	-	-	gi|70887806|ref|NP_079731.2|	7.57E-91	coiled-coil domain-containing protein 28B [Mus musculus]

mmu-miR-122-5p	NM_025517	-28.3	0.234926	-26.02	167	GO:0031981,GO:0016874,GO:0009975,GO:0003676,GO:0032559,GO:0010467,	K01974|1|0.0|722|mmu:66368|RNA 3'-terminal phosphate cyclase [EC:6.5.1.4]	gi|254587958|ref|NP_079793.2|	0	RNA 3'-terminal phosphate cyclase [Mus musculus]
mmu-miR-122-5p	NM_026177	-29.3	0.507632	-47.7	310	-	-	gi|50511061|dbj|BAD32516.1|	3.38E-178	mKIAA1704 protein [Mus musculus]
mmu-miR-122-5p	NM_026376	-29	0.638776	-48.16	302	GO:0031224,GO:0005488,GO:0004888,GO:0001525,GO:0006935,GO:0023052,GO:00606	K06822|1|0.0|3678|mmu:67784|plexin D	gi|51243714|gb|AAT99561.1|	0	plexin D1 [Mus musculus]
mmu-miR-122-5p	NM_026450	-33.2	0.09861	-29.96	163	GO:0043231,GO:0043169,GO:0001071,GO:0006351,	K09228|1|0.0|1564|mmu:67911|KRAB domain-containing zinc finger protein	gi|257153346|ref|NP_080726.3|	0	GLI-Kruppel family member HKR1 isoform a [Mus musculus]
mmu-miR-122-5p	NM_027042	-30.7	0.036946	-27.91	167	-	-	gi|254587906|ref|NP_081318.1|	2.67E-22	late cornified envelope-like proline-rich protein 1 [Mus musculus]

mmu-miR-122-5p	NM_027770	-31.6	0.307961	-29.61	164	GO:0005576,	K06236|1|6e-74|271|acs:100556853|collagen, type I/II/III/V/XI, alpha	gi|116326001|ref|NP_082046.2|	0	collagen alpha-1(XXIV) chain precursor [Mus musculus]
mmu-miR-122-5p	NM_029005	-28.8	0.20885	-26.86	166	GO:0016301,GO:0032559,GO:0006464,	K08849|1|0.0|915|mmu:74568|mixed lineage kinase domain-like [EC:2.7.11.1]	gi|110626037|ref|NP_083281.1|	0	mixed lineage kinase domain-like protein [Mus musculus]
mmu-miR-122-5p	NM_029202	-25.6	0.489298	-25.13	155	GO:0008406,	K15502|1|8e-23|108|rno:306264|serine/threonine-protein phosphatase 6 regulatory ankyrin repeat subunit A	gi|268370074|ref|NP_083478.2|	6.40E-151	ankyrin repeat domain-containing protein 7 isoform 2 [Mus musculus]

mmu-miR-122-5p	NM_029401	-26.4	0.814202	-25.93	172	-	K10798|1|2e-33|145|bfo:BRAFLDRAFT_73983|poly [ADP-ribose] polymerase [EC:2.4.2.30]	gi|198278486|ref|NP_083677.1|	0	von Willebrand factor A domain-containing protein 5B1 precursor [Mus musculus]
mmu-miR-122-5p	NM_080436	-25.1	0.947603	-42.42	313	GO:0044432,GO:0016021,GO:0016616,GO:0042802,GO:0001523,GO:0035238,	K11154|1|0.0|639|mmu:107605|retinol dehydrogenase 16 [EC:1.1.1.-]	gi|165377241|ref|NP_536684.2|	0	retinol dehydrogenase 1 (all trans) precursor [Mus musculus]
mmu-miR-122-5p	NM_133957	-25.6	0.999051	-43.73	310	-	K04446|1|0.0|2473|mmu:54446|nuclear factor of activated T-cells, cytoplasmic, calcineurin-dependent	gi|163937840|ref|NP_061293.2|	0	nuclear factor of activated T-cells 5 isoform b [Mus musculus]
mmu-miR-122-5p	NM_138741	-27.1	0.627025	-25.31	162	GO:0044459,GO:0044444,GO:0005543,GO:0006357,	K09291|1|8e-06|53.9|tca:655393|nucleoprotein TPR	gi|20270267|ref|NP_620080.1|	0	serum deprivation-response protein [Mus musculus]

mmu-miR-122-5p	NM_145706	-25.5	0.53929	-25.04	151	GO:0005643,GO:0000776,GO:0044444,GO:0005488,GO:0044238,GO:0050658,GO:0015758,GO:0007067,	K14305|1|0.0|793|mmu:69912|nuclear pore complex protein Nup43	gi|148671597|gb|EDL03544.1|	0	nucleoporin 43, isoform CRA_a [Mus musculus]
mmu-miR-122-5p	NM_146542	-28.3	0.127283	-25.83	168	GO:0031224,GO:0004930,GO:0007166,GO:0007606,	K04257|1|8e-180|629|mmu:218066|olfactory	gi|153945888|ref|NP_666753.2|	5.12E-179	olfactory receptor 11 [Mus musculus]
mmu-miR-122-5p	NM_153535	-26.5	0.895848	-25.02	161	-	-	gi|23956296|ref|NP_705763.1|	0	protein FAM149A [Mus musculus]

mmu-miR-122-5p	NM_170779	-28.8	0.401903	-27.06	158	GO:0043231,GO:0001726,GO:0044444,GO:0016020,GO:0005488,GO:0003712,GO:0048870,GO:0010740,GO:0006351,	K05631|1|1e-17|93.6|mcc:698092|atrophin-1 interacting protein 3 (BAI1-associated protein 1)!K05629|2|4e-16|88.2|nve:NEMVE_v1g20232|atrophin-1 interacting protein 1!K06112|3|5e-16|87.8|mdo:100033161|membrane-associated guanylate	gi|82524278|ref|NP_740749.1|	0	protein KIBRA [Mus musculus]

mmu-miR-122-5p	NM_172443	-25.6	0.982844	-45	308	GO:0044464,GO:0005099,GO:0032318,	K11452|1|7e-117|424|tgu:100222038|chromobox protein 4!K13136|2|6e-46|188|nvi:100114269|gem associated protein 8!K11837|3|1e-06|58.2|hsa:9098|ubiquitin carboxyl-terminal hydrolase 6/32 [EC:3.1.2.15]	gi|148702739|gb|EDL34686.1|	0	TBC1 domain family, member 16, isoform CRA_b [Mus musculus]
mmu-miR-122-5p	NM_172581	-28.9	0.393018	-25.22	161	-	-	gi|148670839|gb|EDL02786.1|	0	RIKEN cDNA 9830169C18, isoform CRA_b [Mus musculus]
mmu-miR-122-5p	NM_172743	-28.2	0.585124	-25.15	166	-	K06102|1|1e-06|57.0|mgp:100544052|cingulin	gi|170785877|gb|ACB38004.1|	0	heart adaptor protein 1b [Mus musculus]

mmu-miR-122-5p	NM_172841	-26.1	0.980711	-44.91	320	GO:0031224,GO:0051234,	K14356|1|0.0|1574|mmu:240726|solute carrier organic anion transporter family, member 5A	gi|124378011|ref|NP_766429.2|	0	solute carrier organic anion transporter family, member 5A1 [Mus musculus]
mmu-miR-122-5p	NM_172943	-29.3	0.544133	-28.74	160	GO:0031224,GO:0043169,GO:0016701,GO:0008152,	K10767|1|0.0|727|mmu:268420|alkylated DNA repair protein alkB homolog 5 [EC:1.14.11.-]	gi|31044423|ref|NP_766531.2|	0	probable alpha-ketoglutarate-dependent dioxygenase ABH5 [Mus musculus]

mmu-miR-122-5p	NM_173385	-29.1	0.439243	-27.17	156	GO:0031012,GO:0016791,GO:0016462,GO:0009968,	K03544|1|0.0|1694|bta:100138870|ATP-dependent Clp protease ATP-binding subunit ClpX!K13908|3|2e-07|59.7|aml:100484941|mucin-5B!K06841|5|7e-07|57.8|api:100165671|semap	gi|27734196|ref|NP_775561.1|	0	cartilage intermediate layer protein 1 precursor [Mus musculus]

mmu-miR-122-5p	NM_173396	-28.6	0.370242	-25.51	164	GO:0043231,GO:0001071,GO:0003677,GO:0048856,GO:0045597,GO:0006351,	K11649|1|6e-20|100|tgu:100229325|SWI/SNF related-matrix-associated actin-dependent regulator of chromatin subfamily C!K15613|2|3e-13|78.2|spu:757216|homeobox protein Meis 1	gi|27734180|ref|NP_775572.1|	8.76E-132	homeobox protein TGIF2 [Mus musculus]
mmu-miR-122-5p	NM_173417	-30.5	0.192354	-25.95	166	GO:0043231,GO:0005887,GO:0005249,GO:0016247,GO:0030073,GO:0015980,GO:0034220,GO:0030001,	K04933|1|0.0|913|mmu:238076|potassium voltage-gated channel delayed-rectifier subfamily S member 3	gi|27734146|ref|NP_775593.1|	0	potassium voltage-gated channel subfamily S member 3 isoform 1 [Mus musculus]

mmu-miR-122-5p	NM_175163	-28.3	0.505775	-28.69	153	GO:0043231,GO:0046914,GO:0003676,GO:0006351,	K09228|1|0.0|999|mmu:71131|KRAB domain-containing zinc finger protein	gi|169808405|ref|NP_780372.2|	0	zinc finger protein 689 [Mus musculus]
[truncated: 307,434 more chars]
